# Supplementary material for: Computational modeling and experimental validation of the EPI-X4/CXCR4 complex allows rational design of small peptide antagonists
Source: Commun Biol. 2021 Sep 22;4:1113. doi: 10.1038/s42003-021-02638-5 (PMC8458281; doi:10.1038/s42003-021-02638-5)
Supplement: Supplementary file 2 — Supplemental Information [file 42003_2021_2638_MOESM2_ESM.pdf]

Supplementary Material to:

Computational Modeling and Experimental Validation of the EPI-X4/CXCR4 Complex Allows Rational Design of Small Peptide Antagonists

Pandian Sokkar[1,2,≠], Mirja Harms[3,≠], Christina Stürzel[3], Andrea Gilg[3], Gönül Kizilsavas[4], Martina Raasholm[5], Nico Preising[6], Manfred Wagner[4], Frank Kirchhoff[3], Ludger Ständker[6], Gilbert Weidinger[5], Benjamin Mayer[7], Jan Münch[3,6,*] and Elsa Sanchez-Garcia[1,*]

[1] Computational Biochemistry, Center of Medical Biotechnology, University of Duisburg-Essen, Essen, Germany

[2] Faculty of Allied Health Science, Chettinad Hospital and Research Institute, Chettinad Academy of Research and Education, Kelambakkam, Tamil Nadu, India

[3] Institute of Molecular Virology, Ulm University Medical Center, Ulm, Germany

[4] Max Planck Institute for Polymer Research, Mainz, Germany

[5] Institute of Biochemistry and Molecular Biology, Ulm University, Ulm, 89081, Germany

[6] Core Facility Functional Peptidomics, Ulm University Medical Center, Ulm, 89081, Germany

[7] Institute for Epidemiology and Medical Biochemistry, Ulm University, 89081, Ulm, Germany

[≠] These authors contributed equally

* Corresponding authors' email:

elsa.sanchez-garcia@uni-due.de; Jan.Muench@uni-ulm.de

SUPPLEMENTARY FIGURES

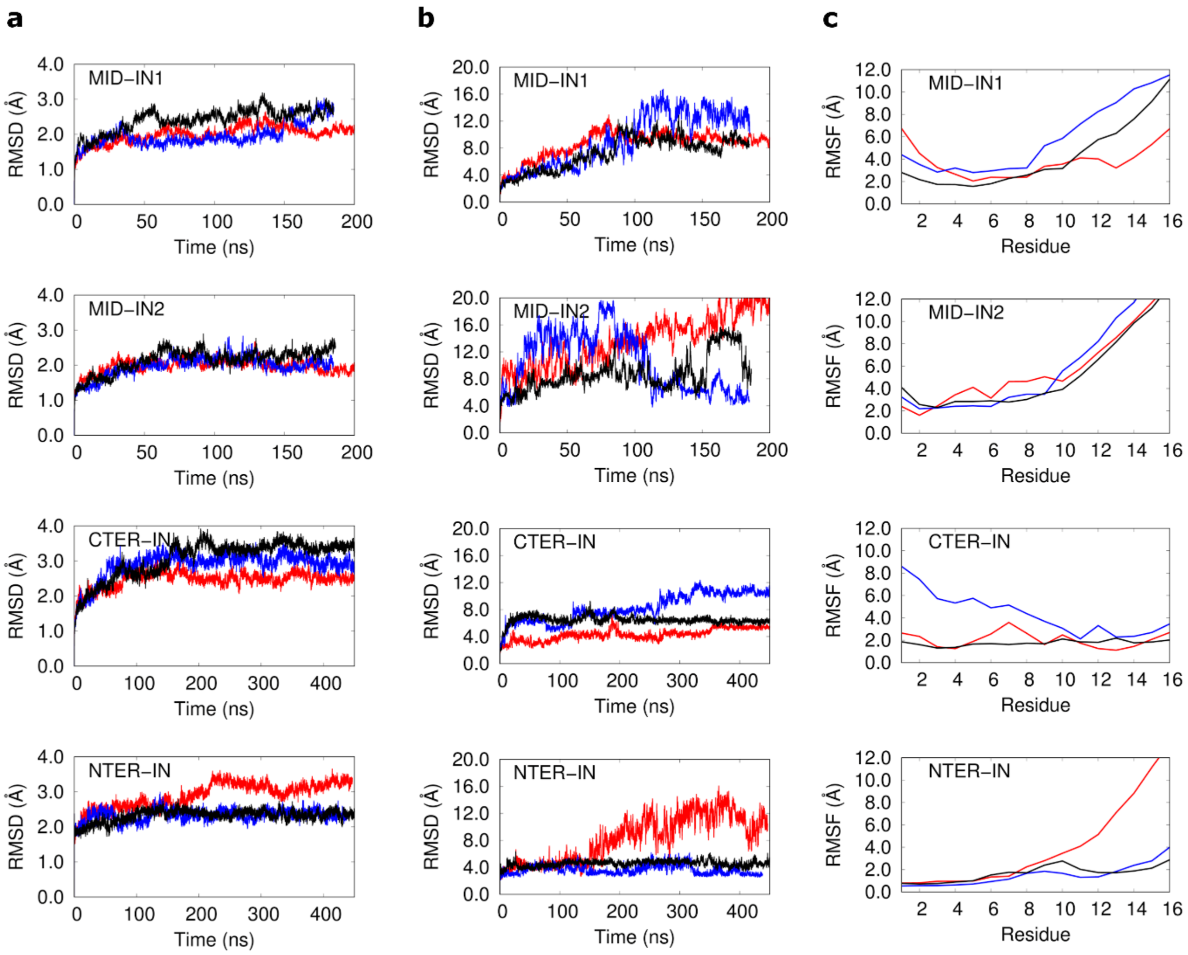

Figure S1. Root mean squared deviation (RMSD) and root mean squared fluctuation (RMSF) from the MD simulations of CXCR4/EPI-X4 complexes. a) RMSD of the backbone atoms of CXCR4 during the three replicas of the simulations (red, blue, and black lines). b) RMSD of the backbone atoms of EPI-X4 and c) RMSF of EPI-X4 residues averaged over the simulation time. The analysis was carried out after aligning the backbone atoms of CXCR4 to the initial structure. The N- and C-terminal loops of CXCR4 were omitted in the RMSD analysis. The analysis of the MDs indicated that the N-terminal and C-terminal loops of CXCR4 are highly disordered, as shown by the root mean squared deviation (RMSD) values. The rest of the protein displayed very low RMSD values (<4 Å), signaling few structural fluctuations (a). On the other hand, the RMSDs of EPI-X4 show large variations, especially in case of the MID-IN and MID-IN2 binding modes (b). The CTER-IN and NTER-IN conformations exhibited a somewhat stable behavior (b), suggesting that these are favored. The root mean squared fluctuation (RMSF) of EPI-X4 evidenced large variations in the C-terminal region in all trajectories corresponding to the MID-IN1 and MID-IN2 binding modes

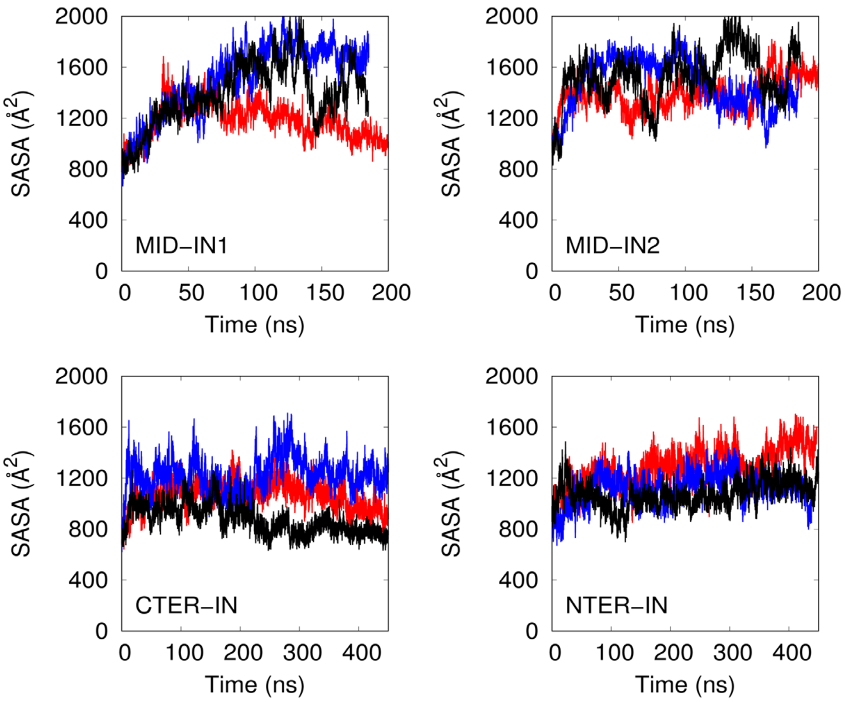

Figure S2. SASA of peptides from the MD simulations of MID-IN1, MID-IN2, CTER-IN and NTER-IN modes.

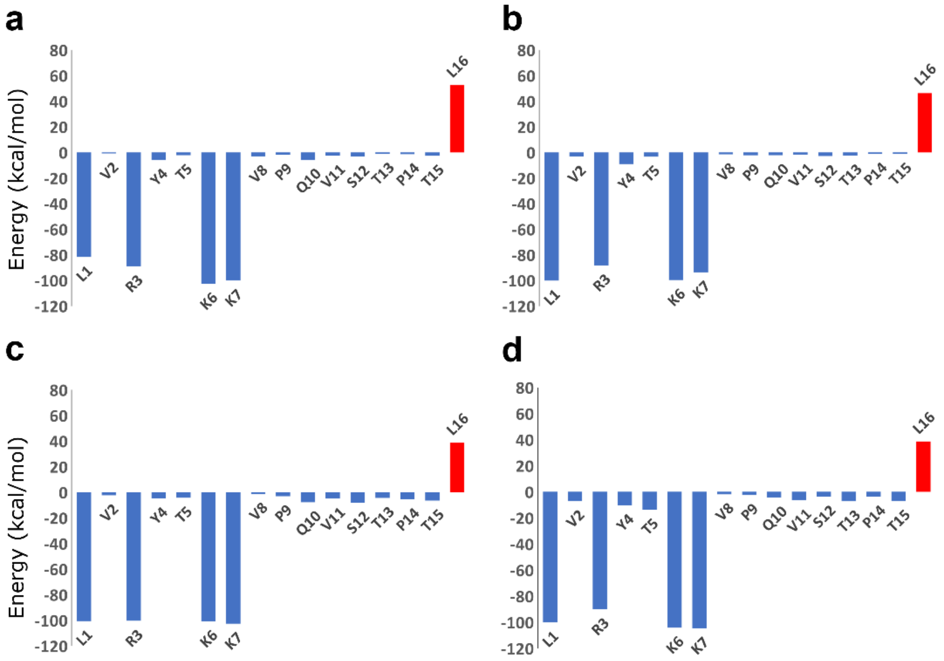

Figure S3. Contributions to the interaction energy by individual residues of EPI-X4 from the simulations of a) MID-IN, b) MID-IN2, c) CTER-IN and d) NTER-IN modes.

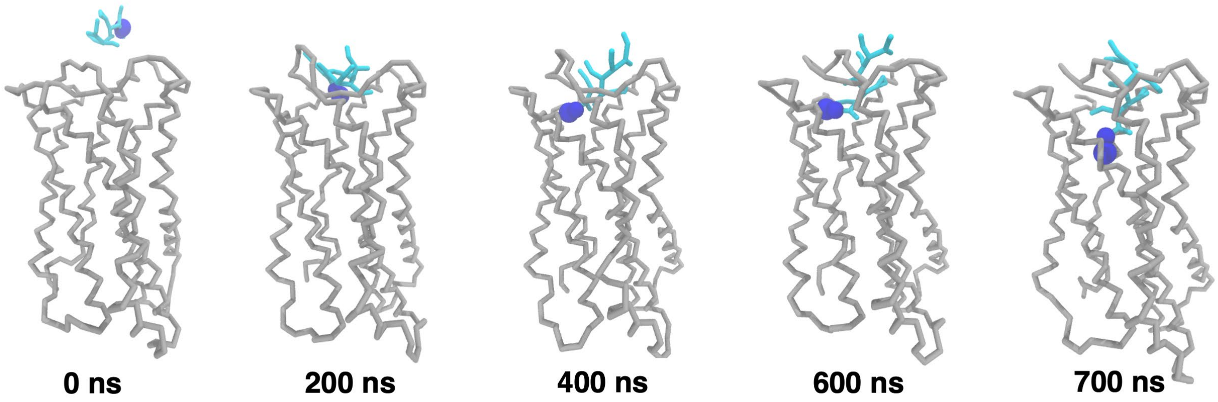

Figure S4. Snapshots of a CG MD simulation with pulling force (k_f=0.2 kJ/mol/Å^2). CXCR4 is shown as grey sticks and the peptide is shown as cyan sticks. The L1 residue is indicated with blue spheres to highlight the N-terminal region of EPI-X4.

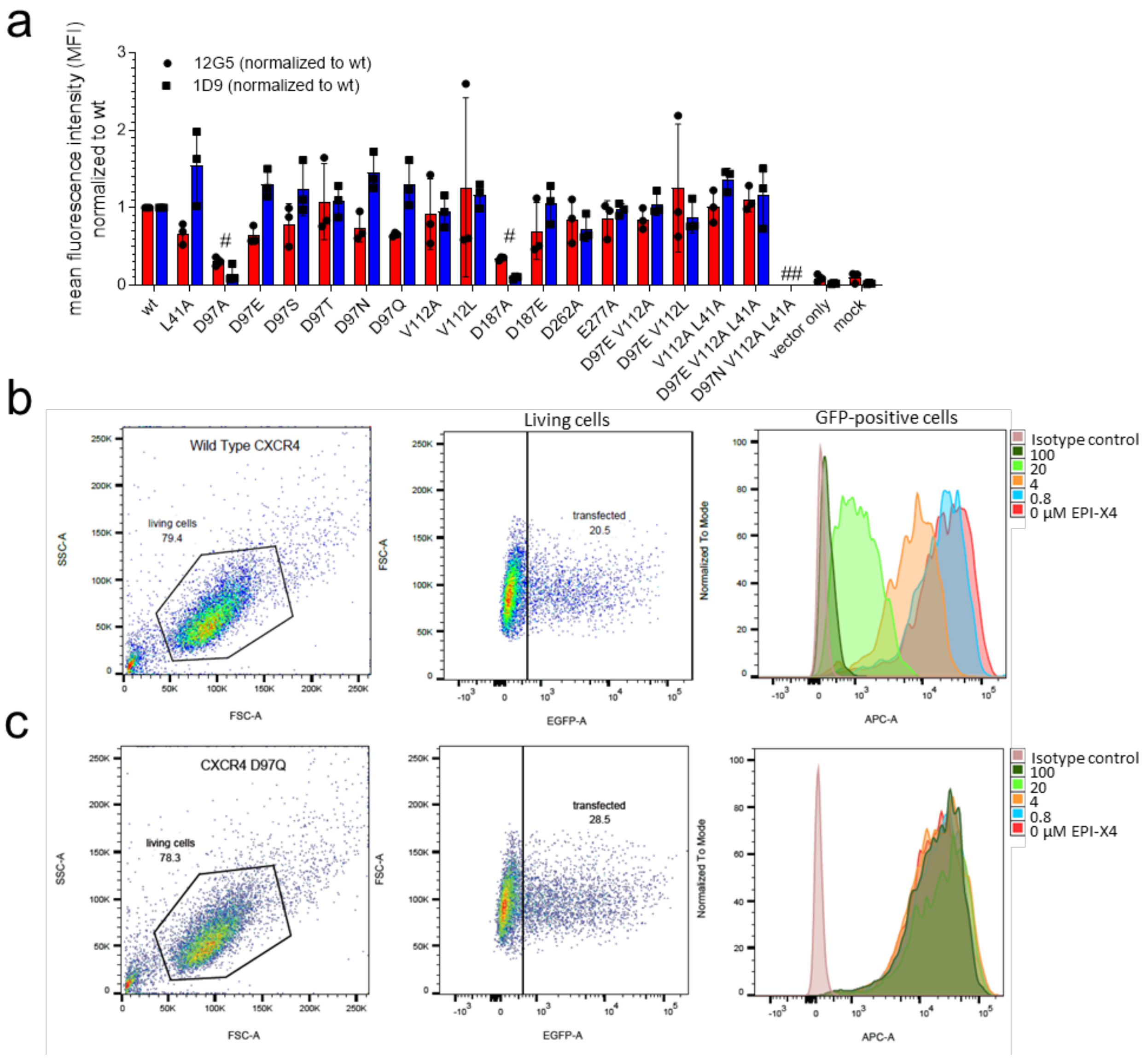

Figure S5. a) CXCR4 expression levels of CXCR4 mutants in 293T cells. Point mutations were introduced into the sequence of CXCR4 in an IRES-GFP expression vector. GPF positive cells were then stained by either the CXCR4 antibody 12G5 (ECL2) or 1D9 (N-terminus) and analyzed by flow cytometry (with the exception of mock control, where also GFP-negative cells were analyzed). Results were normalized to CXCR4 wt expression levels transfected in 293T cells. Shown are data derived from 3 individual experiments ± SEM. # cells were excluded from further binding experiments due to low expression levels, ## no GFP positive cells could be detected and cells were also excluded from further binding experiments. b,c) Gating strategy and exemplary plots for 12G5 competition. HEK293T cells were transfected with wild type CXCR4 (b) or CXCR4 with the mutation D97Q (c) in an IRES-GFP vector. Cells were harvested the next day and 12G5 competition assay performed in the presence of serially diluted EPI-X4. For analysis, APC-signal (bound 12G5-APC antibody) for GFP-positive cells was determined.

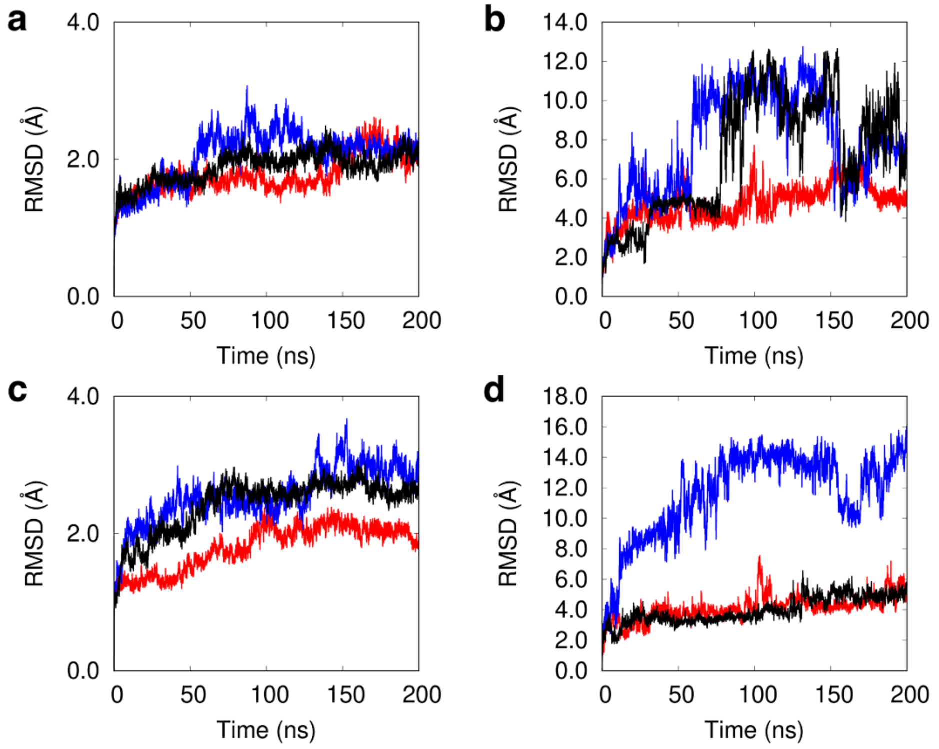

Figure S6. RMSD profiles for the CXCR4/WSC02 and CXCR4/JM#21 complexes during the three replicas of the MD simulations. a) and b) Backbone RMSD of CXCR4 and WSC02, respectively, in the CXCR4/WSC02 complex. c) and d) Backbone RMSD of CXCR4 and JM#21, respectively, in the CXCR4/JM#21 complex.

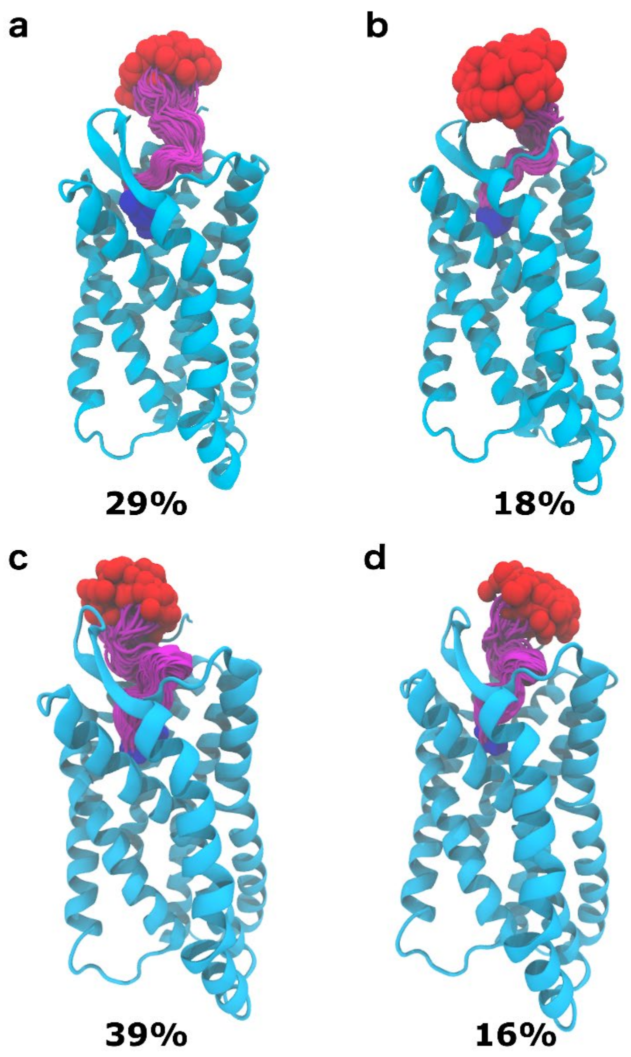

Figure S7. Clustering analysis from the MD simulations of CXCR4/WSC02 (a and b) and CXCR4/JM#21 (c and d) complexes. Color scheme is same as that of Figure 1. Two of the top-ranking clusters are shown for each complex. Clustering analysis was performed with the RMSD cutoff of 3 Å, using the VMD plug-in.

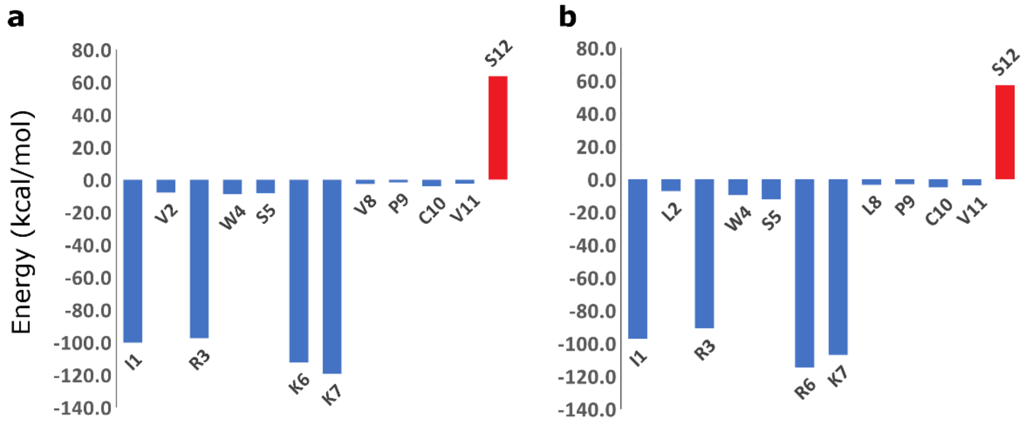

Figure S8. Contribution to the interaction energy by individual amino acids of a) WSC02 and b) JM21.

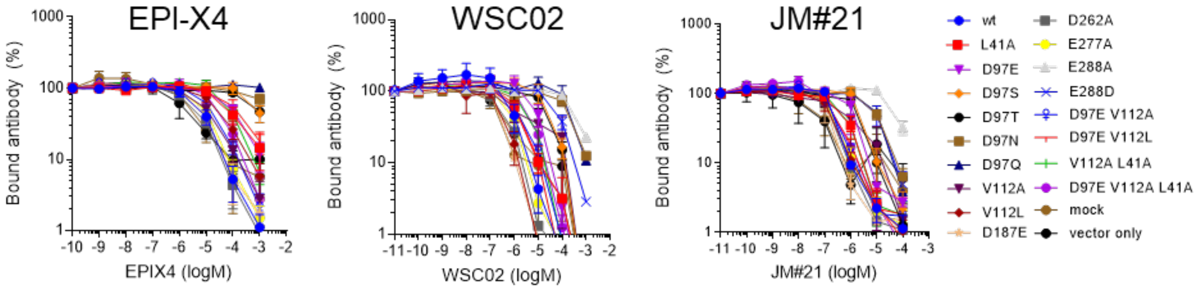

Figure S9. EPI-X4, WSC02 and JM#21 interaction to point-mutated CXCR4 by antibody competition. Amino acid substitutions were introduced in the sequence of CXCR4 by site-directed mutagenesis, cloned into an IRES-GFP expression vector and transfected into 293T cells. Afterwards, cells were incubated with serially diluted EPI-X4, WSC02, or JM#21 in presence of a constant concentration of CXCR4 specific antibody (clone 12G5). After 2 hours, bound antibody was analyzed by flow cytometry. Shown are data derived from at least 3 individual experiments ± SEM. (see also Figure 6)

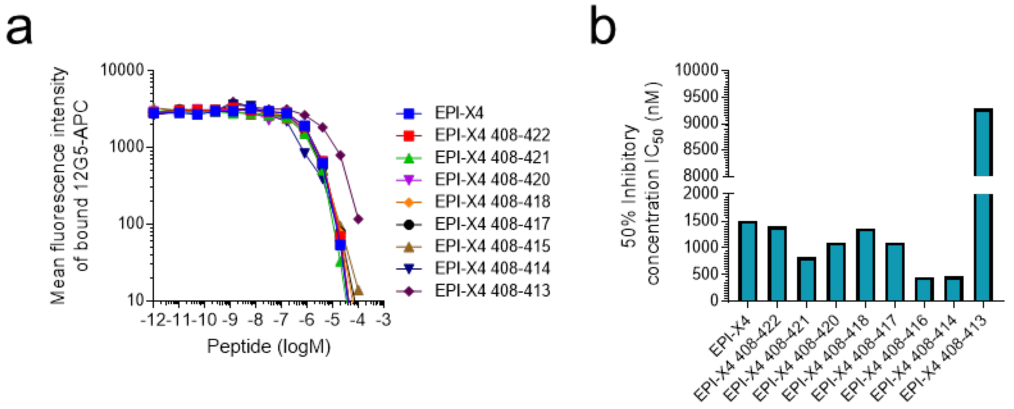

Figure S10. C-terminally truncated EPI-X4 competes with an CXCR4 specific antibody. EPI-X4 analogues were designed that are serially truncated at the C-terminus. a) Peptides were serially diluted and added to SupT1 cells together with a constant concentration of a CXCR4 antibody that binds close to the binding pocket of the receptor. After 2 hours, unbound antibody was removed and antibody binding determined by flow cytometry. b) IC_{50} values were determined by non-linear regression. Shown are data derived from one single assay.

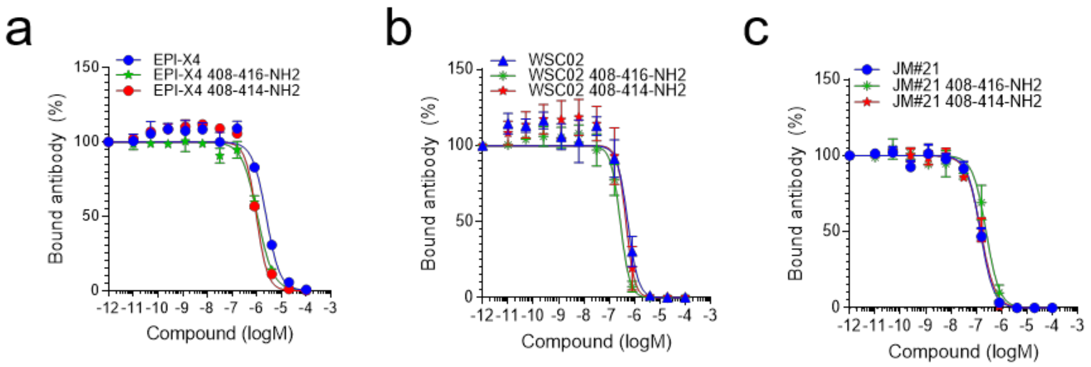

Figure S11. Rationally designed C-terminally truncated EPI-X4 peptides compete with 12G5 antibody binding to CXCR4. EPI-X4 (a), WSC02 (b), or JM#21 (c) and truncated versions thereof were serially diluted and added to SupT1 cells together with a constant concentration of CXCR4 antibody (clone 12G5). After 2 hours, the unbound antibody was removed, and the remaining antibody analyzed in flow cytometry. Shown are data derived from 3 or 4 individual experiments ± SEM.

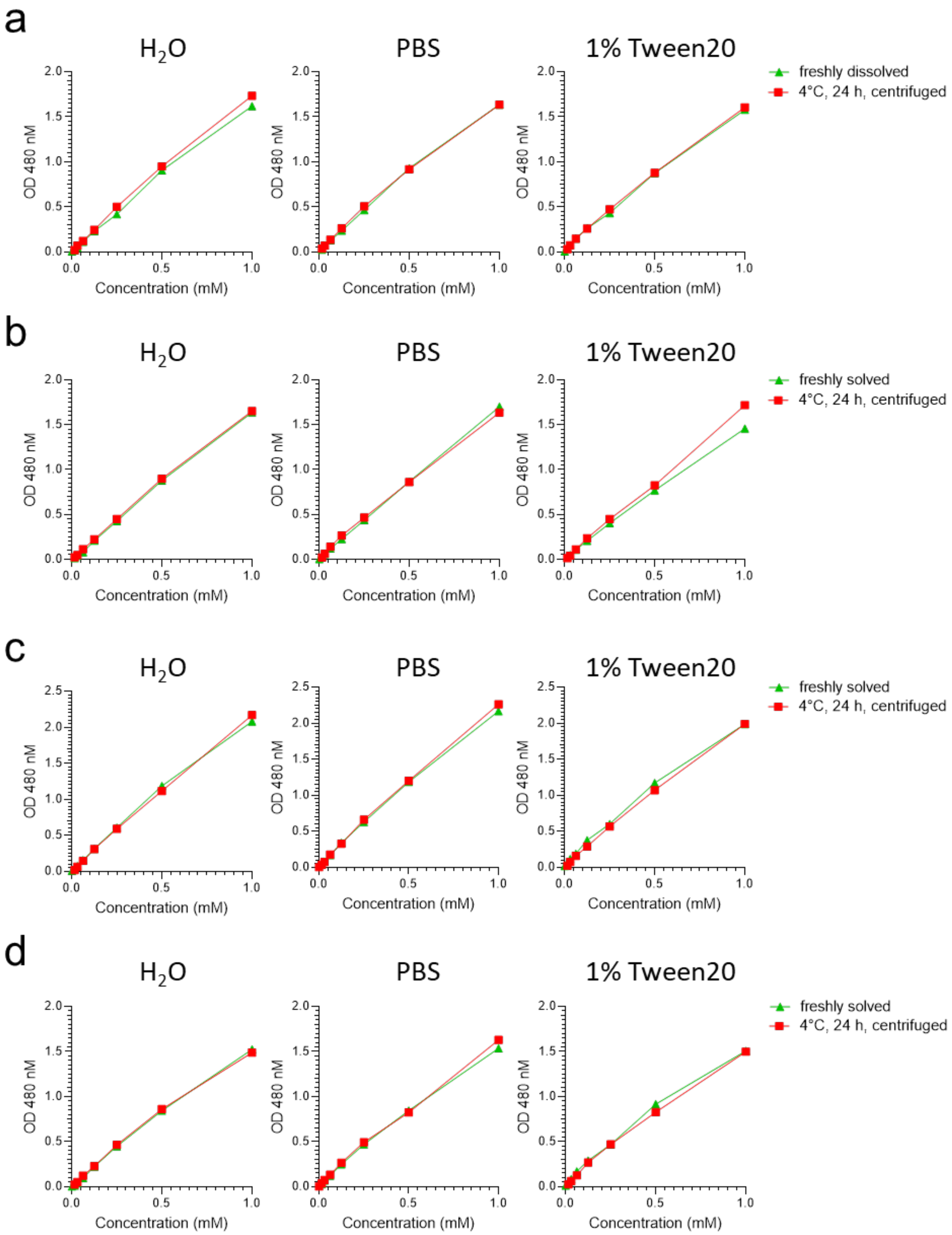

Figure S12: Solubility of selected peptides in H_2O, PBS and Tween20. EPI-X4 (a), WSC02 (b), JM#21 (c) or JM#21 408-414-NH_2 (d) were serially diluted in either H_2O, PBS or Tween20 in PBS (1 %) and incubated at 4°C for 24 hours. Afterwards each mixture was centrifuged at 20,000 x g for 5 min. The supernatant was then analyzed for peptide concentration using BCA assay. For comparison the peptide was freshly dissolved shortly before the assay. Shown is one representative experiment.

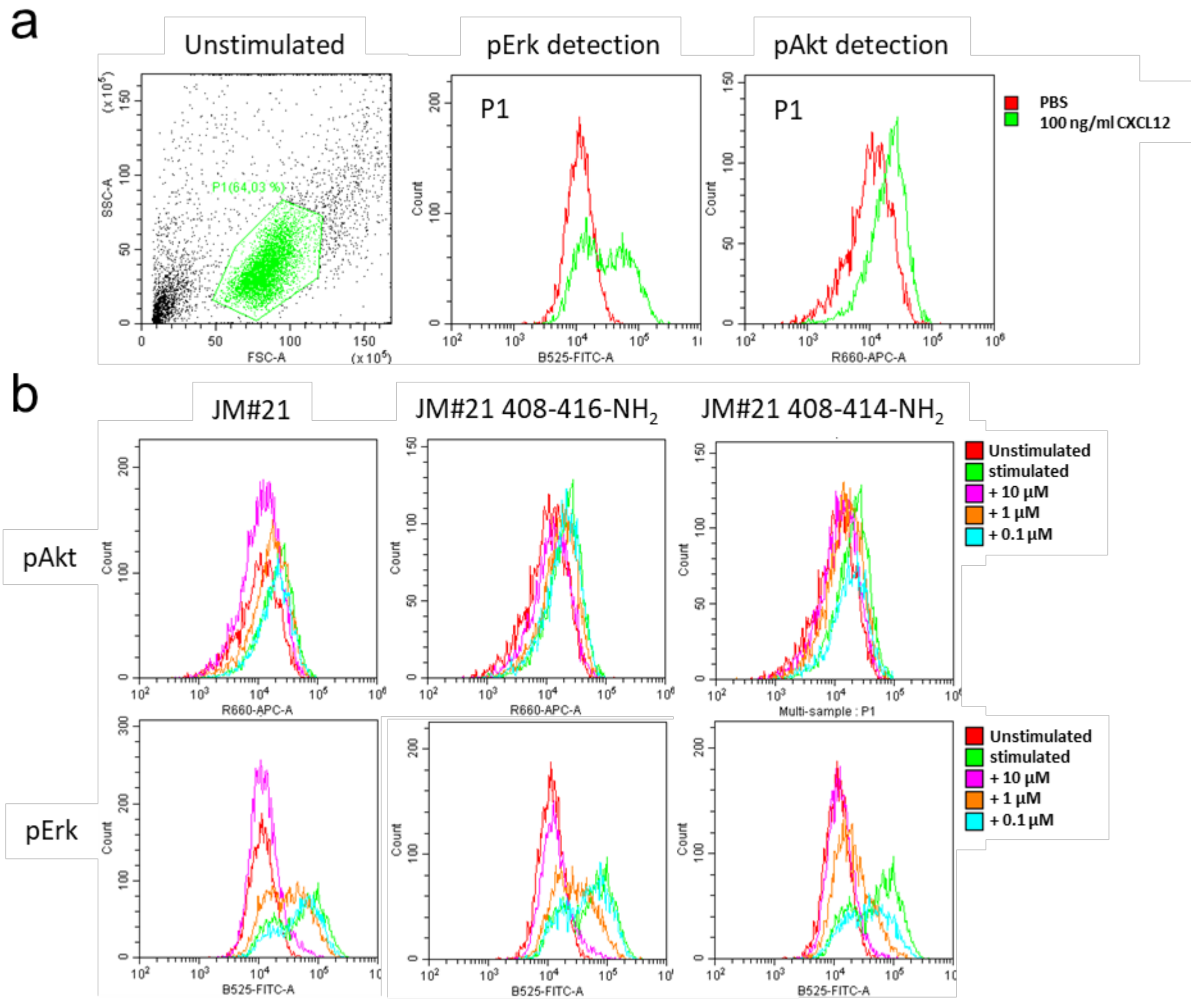

Figure S13. Exemplary presentation of FACS histograms for CXCL12-induced phospho-Akt and phospho-Erk inhibition. SupT1 cells were stimulated with 100 ng/ml CXCL12 in the presence of peptides for 2 min. Afterwards reaction was stopped by adding 2% PFA and shifting the cells to 4 °C. Cells were then permeabilized and subsequently stained with antibodies against pAkt and pErk for analysis in flow cytometry. a) Gating strategy. b) Dose dependent inhibition of pAkt and pErk signal by JM#21 and truncated derivatives.

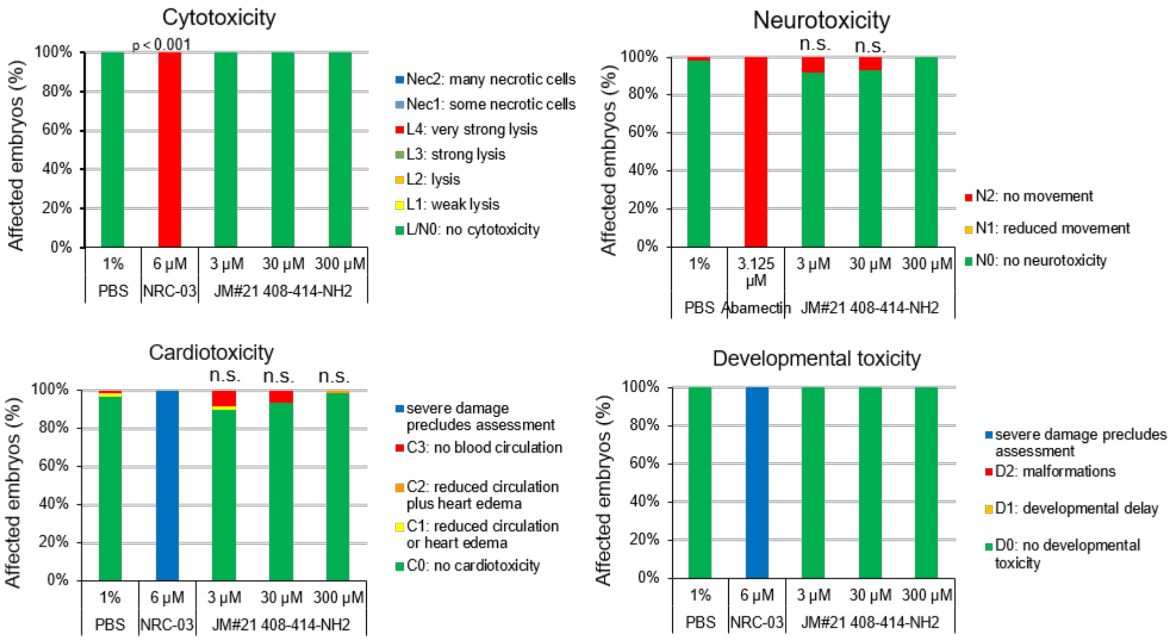

Figure S14. JM#21 408-414-NH2 is not toxic to zebrafish embryos. Zebrafish embryos were scored for mortality or altered phenotypes at 48 hpf after exposure for 24 hrs to the peptide or the negative control (PBS) at the indicated concentrations. As positive control the antimicrobial peptide NRC-03 was used. Altered phenotypes include necrosis and non-lethal lysis (cytotoxicity), heart edema, reduced or absent circulation (cardiotoxicity), delayed development or malformations (developmental toxicity) and reduced or absent touch escape response (neurotoxicity). Note that none of the observed defects occurred significantly more often in the peptide treated groups than in negative controls (Chi-Square test). n= 60 embryos each group.

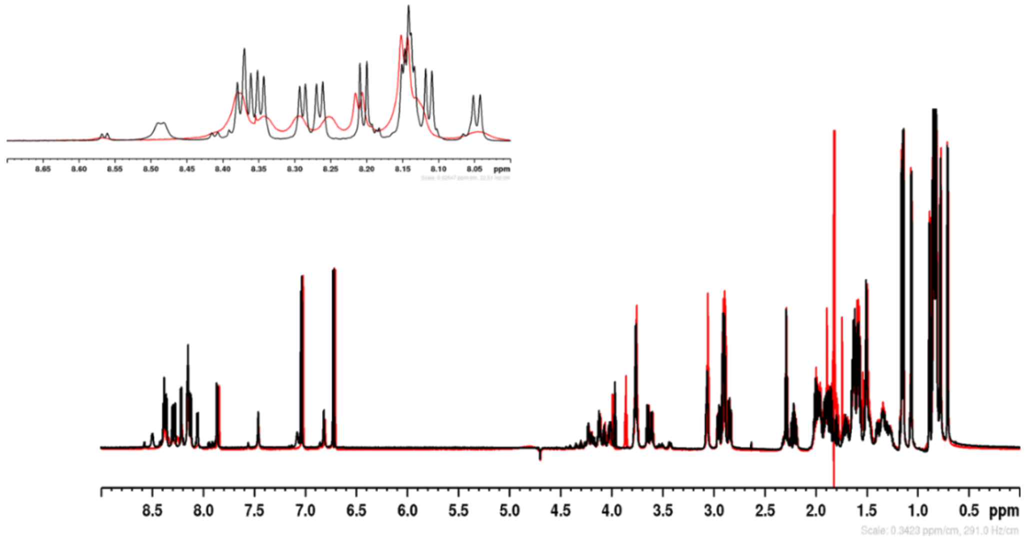

Figure S15. ^{1}H NMR spectrum of EPI-X4 in NaP-buffer red: with 50 mM NaOAc and black: without NaOAc.

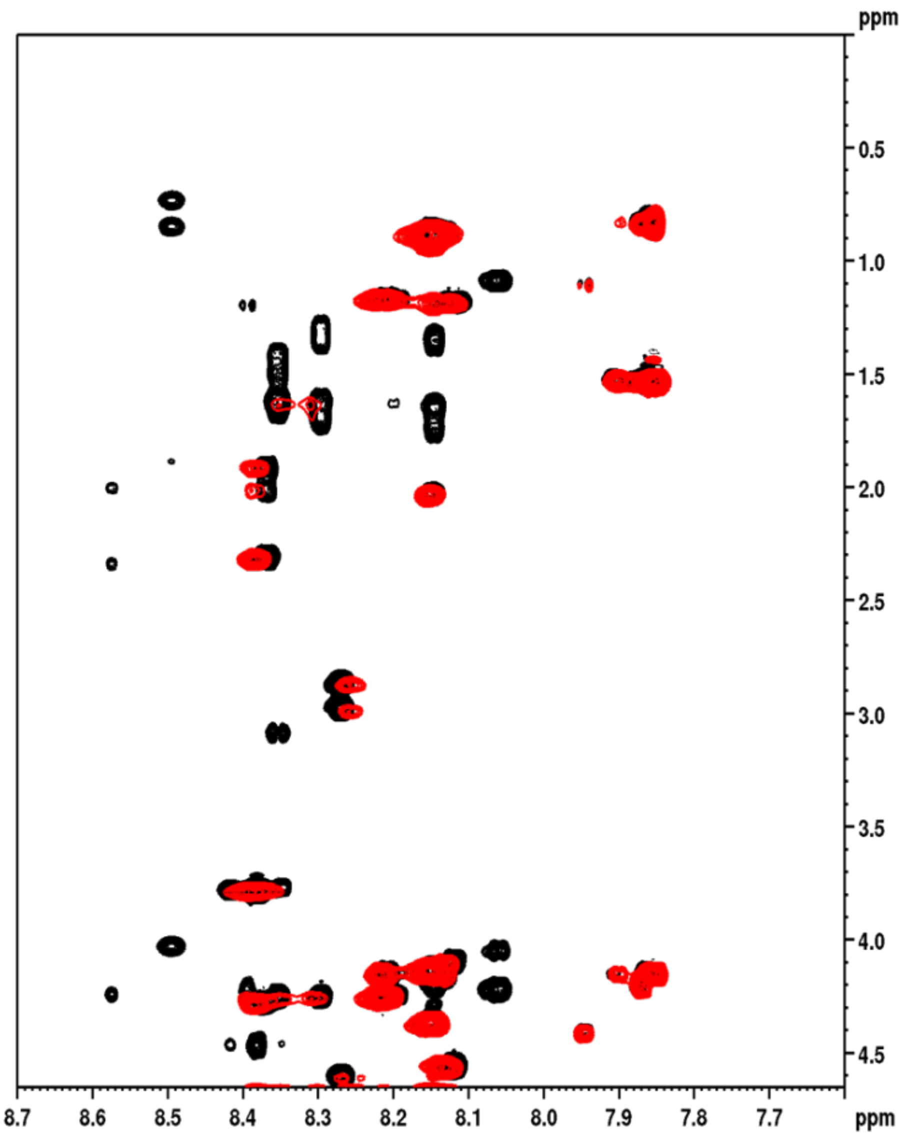

Figure S16. ^{1}H-^{1}H TOCSY NMR spectrum of EPI-X4 in NaP-buffer red: with 50 mM NaOAc and black: without NaOAc

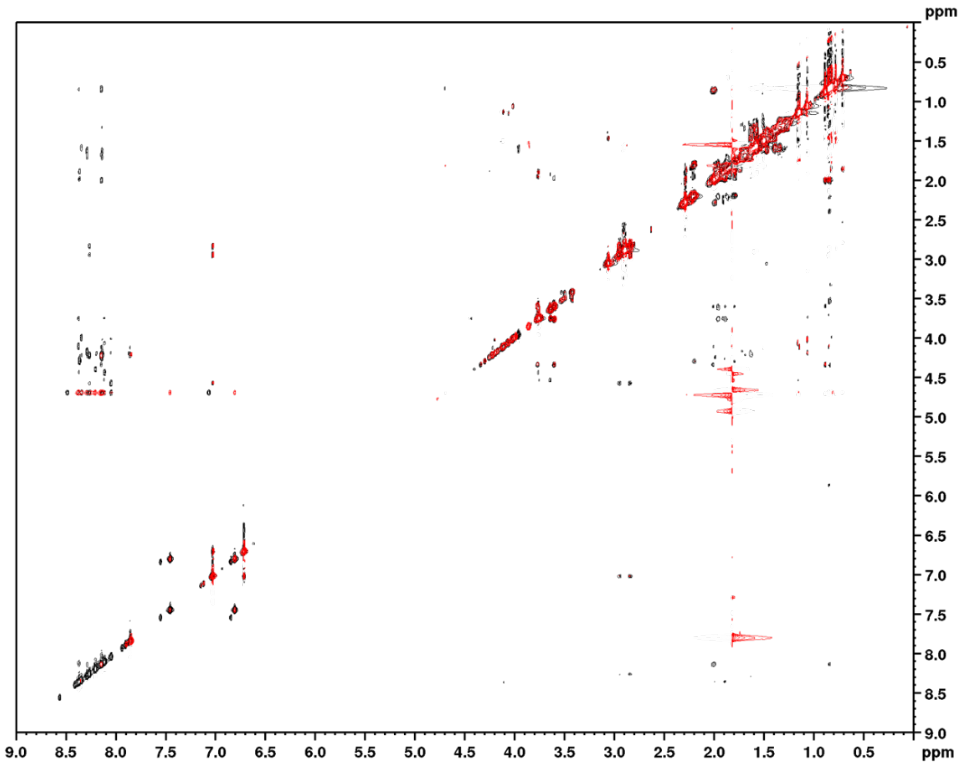

Figure S17. ^{1}H-^{1}H NOESY NMR spectrum of EPI-X4 in NaP-buffer red: with 50 mM NaOAc and black: without NaOAc.

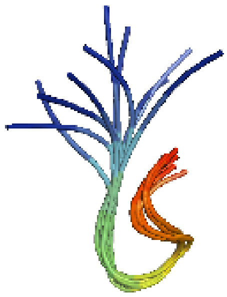

Figure S18. EPI-X4 blue to red: N-terminus to C-terminus with 50 mM NaOAc. The first two amino acids of the N-terminus of EPI-X4 are free and flexible, while the C-terminus is engaged in hydrogen bonds between Thr5 and Gln10 sidechains as well as a hydrogen bond between the backbone carbonyl of Thr5 and the amide hydrogen of Val11, which can be also validated by existing NOE signals in the corresponding NMR spectrum. Additionally, the calculated structure states propose that the sidechain of Ser12 and the carboxylic terminus of Leu16 can be engaged in hydrogen bonds with each other. In those states where the N-terminus comes close to the C-terminus, the sidechain guanidino group of Arg3 can build a hydrogen bond with the carboxylic end of the peptide chain. However, for the contacts between Ser12 and Leu16, as well as between Arg3 and Leu16, no NOE signals were detected. Thus, although those contacts can in theory be established, they are not as populated in the conformational range as to be detected via NMR.

SUPPLEMENTARY TABLES

Table S1. SASA and protein-peptide interaction interface area of EPI-X4 in the different binding modes[a]

Binding mode	SASA ($Å^2$)	Protein-peptide Interface area ($Å^2$)
MID-IN1	1329	810
MID-IN2	1470	836
CTER-IN	1053 (1072)	1061 (991)
NTER-IN	1159 (1098)	1103 (1137)

[a] We also show in parenthesis data from 600 ns simulations of CTER-IN and NTER-IN for comparison using the same simulation time in all four cases.

Table S2. Occupancy of H-bonds between CXCR4 and *EPI-X4* during the MD simulations

**Donor**	**Acceptor**	**Occupancy (%)**
**MID-IN1**		
K7-Side	D187-Side	33.8
R3-Side	D193-Side	20.7
L1-Main	E26-Side	20.5
K6-Side	D262-Side	19.5
K6-Side	E277-Side	17.6
S12-Side	D181-Side	11.7
**MID-IN2**		
K6-Side	D187-Side	31.4
L1-Main	E32-Side	28.2
K7-Side	D62-Side	25.5
Y4-Side	D97-Side	21.1
L1-Main	E31-Side	14.6
Y4-Main	R30-Main	12.9
K7-Side	E288-Side	10.0

**CTER-IN**		
S12-Side	D262-Side	35.1
R3-Side	D97-Side	19.8
T13-Main	D262-Side	19.6
K7-Side	D187-Side	14.0
L1-Main	D181-Side	13.8
T15-Side	E288-Side	13.5
R3-Side	D187-Side	12.9
R188-Side	*L16-Side*	11.4
**NTER-IN**		
T5-Side	D187-Side	69.5
T5-Main	D187-Side	49.9
R3-Side	E31-Side	40.8
R3-Main	D97-Side	37.1
L1-Main	D97-Side	33.8
K7-Side	D262-Side	33.4
R30-Main	*V11-Main*	26.4
K6-Side	R188-Main	24.3
C28-Main	*T13-Main*	17.3
K6-Side	D187-Side	17.1
K271-Side	*L16-Side*	16.2
R3-Side	D181-Side	12.9
R30-Side	*T13-Main*	12.1
R30-Side	*T13-Side*	11.7
T15-Side	E277-Side	10.0

H-Bond criteria: distance cutoff = 3.0 Å and angle cutoff = 20°

Residues of EPI-X4 are highlighted in red italics.

Table S3. Interaction energies EPI-X4 in different binding modes

**Binding mode**	**vdW energy (kcal/mol)**	**Electrostatic energy (kcal/mol)**	**Total interaction energy (kcal/mol)**
MID-IN1	-47.3	-654.8	-702.1 ± 7.5
MID-IN2	-49.4	-682.1	-731.5 ± 6.3
CTER-IN	-71.4	-765.7	-837.1 ± 4.5
NTER-IN	-71.8	-784.2	-856.0 ± 3.2

The interaction energies were calculated every 0.5 ns from the simulation trajectories under vacuum conditions (i.e., the contributions to the energy by water, membrane and ions were neglected) at the force field level. The errors were estimated by bootstrap analysis using 500 steps.

Table S4. H-bonds formed between protein and peptide in CXCR4/WSC02 and CXCR4/JM#21 complexes

**Donor**	**Acceptor**	**Occupancy (%)**
**WSC02**		
K7-Side	E277-Side	40.8
K6-Side	D262-Side	33.9
S5-Main	D187-Side	32.0
S5-Side	D187-Side	23.9
R3-Side	D182-Side	20.7
I1-Main	E288-Side	17.2
V2-Main	D97-Side	13.2
R3-Main	D97-Side	12.0
R3-Side	E2-Side	11.5
R3-Side	D97-Side	10.2
I1-Main	D97-Side	8.1
**JM#21**		
R6-Side	D262-Side	81.0
S5-Side	D187-Side	57.6
S5-Main	D187-Side	52.1
R3-Side	D97-Side	38.2
I1-Main	E288-Side	32.4
K7-Side	E277-Side	20.4
R3-Side	E31-Side	18.4
S12-Side	D182-Side	13.0
R6-Side	D193-Side	12.4

Table S5. IC_{50} values for WSC02 and JM#21 determined in an 12G5-competition assay for CXCR4 point mutants

**CXCR4 mutation**	**WSC02**		**JM#21**	
	IC_{50} ± SEM (μM)	Fold change	IC_{50} ± SEM (μM)	Fold change
**wt**	**1.13 ± 0.58**		**0.66 ± 0.11**	
**L41A**	1.71 ± 0.79	1.5	0.72 ± 0.22	1.1
**D97E**	9.54 ± 3.71	8	1.91 ± 0.57	3
**D97S**	20.37 ± 7.8	18	5.39 ± 2.20	8
**D97T**	35.39 ± 26.70	31	3.83 ± 1.91	6
**D97N**	527.05 ± 376.15	466	11.05 ± 2.72	17
**D97Q**	> 100	-	8.05 ± 2.11	12
**V112A**	0.67 ± 0.26	0.6	3.45 ± 3.10	5
**V112L**	0.42 ± 0.26	0.4	0.41 ± 0.26	0.6
**D187E**	0.49 ± 0.18	0.4	0.31 ± 0.24	0.5
**D262A**	0.52 ± 0.25	0.4	0.23 ± 0.06	0.3
**E277A**	0.77 ± 0.23	0.7	0.28 ± 0.07	0.4
**E288A**	> 100	-	86.36 ± 6.73	131
**E288D**	83.16 ± 10.21	74	14.17 ± 5.03	21
**D97E+V112A**	1.69 ± 0.36	1.5	0.47 ± 0.17	0.7
**D97E+V112L**	2.00 ± 0.71	1.8	0.37 ± 0.17	0.6
**V112A+L41A**	4.70 ± 2.63	4	0.63 ± 0.21	1
**D97E+V112A+L41A**	5.77 ± 1.79	5	0.40 ± 0.17	0.6

Table S6. Criteria used to assess toxicity classes and severity in zebrafish embryos

**Cytotoxicity / Acute toxicity**	
L1:	few lysed cells floating in medium; embryos look like wild-type
L2:	lysed cells in medium; embryos show some visible tissue damage
L3:	embryos show strong tissue damage
L4:	embryos are completely disintegrated
Nec1:	individual necrotic cells (dark areas in brightfield)
Nec2:	many necrotic cells (dark areas in brightfield)
**Developmental toxicity**	
D1:	developmental delay (slow development)
D2:	developmental defects (malformations)
**Cardiotoxicity**	
C1:	reduced circulation or heart edema
C2:	reduced circulation plus heart edema
C3:	no circulation plus heart edema
**Neurotoxicity**	
N0:	normal movement in response to touch
N1:	reduced movement in response to touch
N2:	no movement in response to touch
**Overall toxicity (combination of above phenotypes)**	
wt:	wild type (no visible phenotype AND normal movement)
T1:	embryos that show a phenotype
T2:	severe damage so that other phenotypes cannot be assessed (L3, L4, Nec2)

Table S7. Primers used for site-directed mutagenesis of CXCR4

**Primer name**	**Primer sequence 5' -> 3'**
NheI_for	CGGCTAGCATGGAGGGGATCAGTATATACACTTCAG
HindIII_elongation_rev	CGAAGCTTTTATTTATCGTATAAAAAAAAGTCTTTTACATCTGTGTTAGCTGGAGTGAAAACTTGAAGACTC
41_Ala_for	TAAAATCTTCGCGCCCACCATCTAC
41_Ala_rev	TTGAAATTAGCATTTTCTTCAC
97_Ser/Glu_rev	AAGGGAAGCGTGATGACAAAGAGG
97_Glu_for	CTGGGCAGTTGAAGCCGTGGCAA
97_Ser_for	CTGGGCAGTTAGTGCCGTGGCAA
97_Thr_for	CTGGGCAGTTACTGCCGTGGCAA
97_Thr_rev	AAGGGAAGCGTGATGACAAAG
97_Asn_for	CTGGGCAGTTAATGCCGTGGCAA
97_Asn/Gln_rev	AAGGGAAGCGTGATGACAAAGAG
97_Gln_for	CTGGGCAGTTCAGGCCGTGGCAA
112_Ala_for	ATGCAAGGCAGCCCATGTCATCT
112_Ala_rev	AGGAAGTTCCCAAAGTACC
112_Leu_for	ATGCAAGGCACTCCATGTCATCTAC
112_Leu_rev	AGGAAGTTCCCAAAGTAC
187_Glu_for	ATATATCTGTGAACGCTTCTACC
187_rev	CTGTCATCTGCCTCACTG
262_Ala_for	GATCAGCATCGCCTCCTTCATCC
262_Ala_rev	CCAATGTAGTAAGGCAGC
277_Ala_for	GTGTGAGTTTGCGAACACTGTGC
277_Ala_rev	CCTTGCTTGATGATTTCC
frameshift_for	TAATGAGGGGATCAGTATATACACTTC
frameshift_rev	CATGCTAGCCAGCTTGGG
stop_for	ACTGAGAAGCTAGACGGACAAG
stop_rev	TTCTTCTGGTAACCCATG

SUPPLEMENTARY DISCUSSION

Analysis of the NMR spectra

Although the linewidths of the NMR spectra were broadened in acidic environment with respect to the neutral medium, no changes in the chemical shift region representing the amide backbone protons were found (Figure S15). This indicates a faster proton exchange event at the peptide backbone compared to EPI-X4 without NaOAc. The remaining protons of the peptide seem to be unaffected. The only affected signal shifted from 3.963 ppm to 3.860 ppm and does not belong to any of the spin systems of EPI-X4 as there is no TOCSY (Total Correlation Spectroscopy) cross-peak at all at this chemical shift. The prominent signal at around 1.8 ppm evidences the presence of acetic acid in the sample. The broader linewidths at the amide backbone region result in the loss of signals at the corresponding region in the TOCSY spectrum (Figure S16). Otherwise, the loss of cross-peaks in the NOESY (Nuclear Overhauser Effect Spectroscopy) spectrum indicates a loss of dipolar spatial couplings between protons of EPI-X4 (Figure S17). Hence, EPI-X4 gains in flexibility and mobility when it is exposed to ions (Figure S18).

Coarse-Grained (CG) pulling simulations

Our results indicated that when the force constant is very small (k_f=0.1 kJ/mol/Å^2 and k_f=0.2 kJ/mol/Å^2), 6/20 trajectories resulted in the formation of NTER-IN complexes (Figure S4). For the rest of the trajectories, the peptide could not enter the binding pocket. In other words, there is a certain amount of energetic barrier to overcome the steric and/or electrostatic repulsions involved in the binding process. When the force constant was large enough (k_f=0.5 kJ/mol/Å^2), 14/20 trajectories resulted in the NTER-IN binding mode and 1/20 in CTER-IN. Thus, these pulling simulations indicate that NTER-IN is favored over the other modes.

SUPPLEMENTARY METHODS

NMR experiments

To experimentally investigate the conformational properties of EPI-X4 on a medium enriched with negative charges, like the binding pocket of CXCR4, we performed NMR studies of EPI-X4 in the presence of NaOAc. Due to the large flexibility and degrees of freedom associated with the 16-amino acid sequence of EPI-X4, structural information in solution is not directly transferable to predict its binding mode to CXCR4. Further, the anionic binding pocket of CXCR4 also influences the conformational properties of EPI-X4. Indeed, our NMR studies of EPI-X4 in a solution with 50 mM NaOAc indicate that EPI-X4 gains in flexibility in such acidic environment. This flexibility supports the need for molecular dynamics studies of the interactions of EPI-X4 with CXCR4.

For the NMR experiments with NaOAc, 5 mg of EPI-X4 were dissolved in 450 ml of 10 mM NaP-buffer (NaH_2PO_4/Na_2HPO_4) and 50 ml D_2O. For the experiments with acetate ions sodium acetate (C2H3NaO2) was added with a final concentration of 50 mM. If necessary, the pH was adjusted with HCl or NaOH to pH 7. All experiments reported here were recorded on an 850 MHz AVANCE III Bruker system equipped with a 5 mm quadruple resonance QXI 1H/13C/15N/31P probe with a z-gradient. Experiments were carried out at 298 K. Nuclear Overhauser Effect Spectroscopy (NOESY) spectra acquiring 2D homonuclear correlation via dipolar coupling with water suppression using watergate W5 pulse sequence with gradients[1,2] were recorded for a mixing time of 100, 200 and 300 ms, using 2 x 16k x 256 data matrices, corresponding to acquisition times of ~480 and 8 ms in the t1 and t2 dimensions, respectively. Through-bond connectivity was obtained from a Total Correlation Spectroscopy (TOCSY) spectrum recorded with the MLEV-17 mixing scheme[3] with water suppression using 3-9-19 pulse sequence with gradients[4,5], using a 13 µs 90° pulse and a 80 ms mixing period.

NMRFAM-Sparky was used for signal assignment and NOE signal volume determination.[6] For NOE signal integration a gaussian fit was used with allowing peak motion and adjusting linewidths and baseline fitting. For the 3D structure calculation of EPI-X4 the software package ARIA (Ambiguous Restraints for Iterative Assignment) was used.[7]

Coarse-Grained (CG) pulling simulations

The CXCR4 receptor complexed with the EPI-X4 derivative (EPI-X4D: 408-416) was simulated at the coarse-grained (CG) level in a POPC lipid-water environment. The CHARMM-GUI server was used to generate the initial configurations.[8] We employed the Martini 2.2 CG force field[9] implemented for GROMACS program (version 2016.3)[10]. To reduce the system size, the N- terminal loop (corresponding to residues 1-32) and the C-terminal loop (residues 304-319) were removed, and the termini were set as neutral in the CG model. The truncated CXCR4 along with the EPI-X4 derivative (EPI-X4D) were embedded in a POPC bilayer (consisting of 254 lipids) and solvated with an uncharged water model.[9] The total charge of the system was neutralized by the addition of nine chloride ions. In total, the system contained ~10000 CG particles. Constraints were applied to keep the regular secondary structures intact. NPT simulations were performed using velocity rescale thermostat at 310 K and a Berendsen barostat at 1 atm.[11,12] The relative dielectric constant, ε_r=15 was used to account for the screening effect of uncharged water. The short-range LJ interactions were cutoff at 11 Å and shift scheme was used to smoothen the potential. The electrostatic interactions were computed with the reaction-field approach[13], using a cutoff of 11 Å. A time step of 20 fs was used for the integration of position and velocities.

Three equilibration MD simulations were performed with position restraints on the secondary structures using force constants of 10, 5 and 1 kcal/mol/Å^2, respectively. The production MD was performed with position restraints operating only on the transmembrane helices of CXCR4

with a force constant of 0.1 kcal/mol/Å^2. This was necessary to restrict the excessive displacement of the helices.

To simulate the self-assembly of EPI-X4D in the CXCR4 pocket, the peptide was pulled from the solution phase to the binding pockets by constant-force MD simulations at the CG level. For this purpose, we defined a reference point located between the major and minor binding pockets (obtained as the center of geometry of four binding pocket residues D187, E288, D262 and D97). The coordinates of this point served as the absolute reference for the pulling simulations. The peptides were kept in the solution phase and at a distance of ~30 Å from the reference point. A 1 μs MD simulation was performed applying the position restraints on the central residue of the peptide (k_f=10 kcal/mol/Å^2) as well as on the TM-helices (k_f=0.1 kcal/mol/Å^2). From this simulation, 20 snapshots (with the interval of 50 ns) were taken for the pulling simulations.

Different force constants ranging from 0.025 to 2.5 kcal/mol/Å^2 were tested for constant force simulations. We applied biasing forces to the peptide on three different points (i.e., residues 1, 5 and 9) towards the binding pocket. The pulling forces were equal in magnitude (same force constant) on all the three sites and were operating simultaneously. This ensures that the pulling is not biased to a particular site on the peptide. The aim of these simulations was to determine if there is a preferred pattern or binding mode. Therefore, we used very small force constants to allow plenty of conformation sampling that reduces the bias associated with initial configurations. Using twenty different initial structures, we performed twenty simulations, each for 1 μs and under three force constants setups: 0.1, 0.2 and 0.5 kJ/mol/Å^2.

SUPPLEMENTARY REFERENCES

1. Jeener, J., Meier, B. H., Bachmann, P. & Ernst, R. R. Investigation of exchange processes by two-dimensional NMR spectroscopy. *J. Chem. Phys.* **71**, 4546–4553 (1979).

2. Liu, M. *et al.* Improved WATERGATE pulse sequences for solvent suppression in NMR spectroscopy. *J. Magn. Reson.* **132**, 125–129 (1998).

3. Bax, A. D. & Davis, D. G. MLEV-17-based two-dimensional homonuclear magnetization transfer spectroscopy. *J. Magn. Reson.* **65**, 355–360 (1985).

4. Piotto, M., Saudek, V. & Sklenâ\vr, V. Gradient-tailored excitation for single-quantum NMR spectroscopy of aqueous solutions. *J. Biomol. NMR* **2**, 661–665 (1992).

5. Sklenar, V., Piotto, M., Leppik, R. & Saudek, V. Gradient-tailored water suppression for 1H-15N HSQC experiments optimized to retain full sensitivity. *J. Magn. Reson. Ser. A* **102**, 241–245 (1993).

6. Lee, W., Tonelli, M. & Markley, J. L. NMRFAM-SPARKY: enhanced software for biomolecular NMR spectroscopy. *Bioinformatics* **31**, 1325–1327 (2015).

7. Rieping, W. *et al.* ARIA2: Automated NOE assignment and data integration in NMR structure calculation. *Bioinformatics* **23**, 381–382 (2007).

8. Jo, S., Kim, T., Iyer, V. G. & Im, W. CHARMM-GUI: a web-based graphical user interface for CHARMM. *J. Comput. Chem.* **29**, 1859–1865 (2008).

9. de Jong, D. H. *et al.* Improved parameters for the martini coarse-grained protein force field. *J. Chem. Theory Comput.* **9**, 687–697 (2013).

10. Van Der Spoel, D. *et al.* GROMACS: Fast, flexible, and free. *J. Comput. Chem.* **26**, 1701–1718 (2005).

11. Bussi, G., Donadio, D. & Parrinello, M. Canonical sampling through velocity rescaling. *J. Chem. Phys.* **126**, 14101 (2007).

12. Berendsen, H. J. C., Postma, J. P. M. van, van Gunsteren, W. F., DiNola, A. & Haak, J. R. Molecular dynamics with coupling to an external bath. *J. Chem. Phys.* **81**, 3684–3690 (1984).

13. Tironi, I. G., Sperb, R., Smith, P. E. & van Gunsteren, W. F. A generalized reaction field method for molecular dynamics simulations. *J. Chem. Phys.* **102**, 5451–5459 (1995).
