# Supplementary material for: Computational modeling and experimental validation of the EPI-X4/CXCR4 complex allows rational design of small peptide antagonists
Source: Commun Biol. 2021 Sep 22;4:1113. doi: 10.1038/s42003-021-02638-5 (PMC8458281; doi:10.1038/s42003-021-02638-5)
Supplement: Supplementary file 3 — Description of Supplementary Files [file 42003_2021_2638_MOESM3_ESM.pdf]

Description of Additional Supplementary Files

File name: Supplementary Data 1.

Description: Atomic coordinates of the binding modes of the EPI-X4 - CXCR4 complex.

File name: Supplementary Data 2.

Description: Raw data derived from flow cytometry analysis for figure 4, figure 6, and figure 7.
