# Supplementary material for: Computational modeling and experimental validation of the EPI-X4/CXCR4 complex allows rational design of small peptide antagonists
Source: Commun Biol. 2021 Sep 22;4:1113. doi: 10.1038/s42003-021-02638-5 (PMC8458281; doi:10.1038/s42003-021-02638-5)
Supplement: Supplementary file 4 — Supplementary Data 1 [file 42003_2021_2638_MOESM4_ESM.pdf]

1. A representative structure from the highly populated cluster of NTER-IN:

```
CRYST1   90.674   92.205  140.373  90.00  90.00  90.00 P 1           1
ATOM      1  N   MET P   1      -1.349  24.087  20.278  0.00  0.00      PROA
ATOM      2  HT1 MET P   1      -2.146  24.120  20.944  0.00  0.00      PROA
ATOM      3  HT2 MET P   1      -1.701  23.660  19.397  0.00  0.00      PROA
ATOM      4  HT3 MET P   1      -1.064  25.070  20.092  0.00  0.00      PROA
ATOM      5  CA  MET P   1      -0.347  23.202  20.941  0.00  0.00      PROA
ATOM      6  HA  MET P   1      -0.745  22.218  21.142  0.00  0.00      PROA
ATOM      7  CB  MET P   1       0.903  22.992  19.990  0.00  0.00      PROA
ATOM      8  HB1 MET P   1       0.484  22.930  18.963  0.00  0.00      PROA
ATOM      9  HB2 MET P   1       1.440  22.026  20.104  0.00  0.00      PROA
ATOM     10  CG  MET P   1       2.027  24.097  19.890  0.00  0.00      PROA
ATOM     11  HG1 MET P   1       2.615  24.292  20.812  0.00  0.00      PROA
ATOM     12  HG2 MET P   1       1.469  25.048  19.750  0.00  0.00      PROA
ATOM     13  SD  MET P   1       2.985  23.939  18.344  0.00  0.00      PROA
ATOM     14  CE  MET P   1       4.416  25.021  18.733  0.00  0.00      PROA
ATOM     15  HE1 MET P   1       5.111  24.432  19.369  0.00  0.00      PROA
ATOM     16  HE2 MET P   1       4.125  25.909  19.333  0.00  0.00      PROA
ATOM     17  HE3 MET P   1       5.012  25.257  17.826  0.00  0.00      PROA
ATOM     18  C   MET P   1       0.250  23.765  22.238  0.00  0.00      PROA
ATOM     19  O   MET P   1       0.124  24.942  22.540  0.00  0.00      PROA
ATOM     20  N   GLU P   2       0.802  22.854  23.004  0.00  0.00      PROA
ATOM     21  HN  GLU P   2       0.586  21.896  22.834  0.00  0.00      PROA
ATOM     22  CA  GLU P   2       1.542  23.090  24.232  0.00  0.00      PROA
ATOM     23  HA  GLU P   2       0.880  23.565  24.940  0.00  0.00      PROA
ATOM     24  CB  GLU P   2       1.796  21.669  24.893  0.00  0.00      PROA
ATOM     25  HB1 GLU P   2       2.454  21.917  25.754  0.00  0.00      PROA
ATOM     26  HB2 GLU P   2       2.289  20.987  24.168  0.00  0.00      PROA
ATOM     27  CG  GLU P   2       0.467  21.071  25.366  0.00  0.00      PROA
ATOM     28  HG1 GLU P   2      -0.104  20.655  24.509  0.00  0.00      PROA
ATOM     29  HG2 GLU P   2      -0.199  21.744  25.947  0.00  0.00      PROA
ATOM     30  CD  GLU P   2       0.716  19.864  26.232  0.00  0.00      PROA
ATOM     31  OE1 GLU P   2       1.017  18.797  25.649  0.00  0.00      PROA
ATOM     32  OE2 GLU P   2       0.669  19.940  27.480  0.00  0.00      PROA
ATOM     33  C   GLU P   2       2.777  24.020  24.278  0.00  0.00      PROA
ATOM     34  O   GLU P   2       3.581  23.968  23.366  0.00  0.00      PROA
ATOM     35  N   GLY P   3       2.869  24.922  25.197  0.00  0.00      PROA
ATOM     36  HN  GLY P   3       2.246  24.893  25.975  0.00  0.00      PROA
ATOM     37  CA  GLY P   3       4.009  25.833  25.250  0.00  0.00      PROA
ATOM     38  HA1 GLY P   3       3.968  26.392  26.173  0.00  0.00      PROA
ATOM     39  HA2 GLY P   3       3.998  26.459  24.370  0.00  0.00      PROA
ATOM     40  C   GLY P   3       5.382  25.369  25.492  0.00  0.00      PROA
ATOM     41  O   GLY P   3       5.570  24.175  25.732  0.00  0.00      PROA
ATOM     42  N   ILE P   4       6.314  26.364  25.596  0.00  0.00      PROA
ATOM     43  HN  ILE P   4       6.021  27.312  25.697  0.00  0.00      PROA
ATOM     44  CA  ILE P   4       7.710  26.159  25.853  0.00  0.00      PROA
ATOM     45  HA  ILE P   4       8.006  25.501  25.049  0.00  0.00      PROA
ATOM     46  CB  ILE P   4       8.490  27.468  25.709  0.00  0.00      PROA
ATOM     47  HB  ILE P   4       7.977  28.165  26.406  0.00  0.00      PROA
ATOM     48  CG2 ILE P   4       9.926  27.381  26.248  0.00  0.00      PROA
ATOM     49 HG21 ILE P   4      10.625  26.638  25.807  0.00  0.00      PROA
ATOM     50 HG22 ILE P   4       9.849  27.018  27.295  0.00  0.00      PROA
ATOM     51 HG23 ILE P   4      10.357  28.402  26.171  0.00  0.00      PROA
ATOM     52  CG1 ILE P   4       8.297  27.993  24.267  0.00  0.00      PROA
ATOM     53 HG11 ILE P   4       7.269  27.704  23.959  0.00  0.00      PROA
ATOM     54 HG12 ILE P   4       8.937  27.430  23.555  0.00  0.00      PROA
ATOM     55  CD  ILE P   4       8.556  29.501  24.003  0.00  0.00      PROA
ATOM     56  HD1 ILE P   4       7.839  30.145  24.557  0.00  0.00      PROA
ATOM     57  HD2 ILE P   4       8.450  29.598  22.902  0.00  0.00      PROA
```

```
ATOM 58 HD3 ILE P 4 9.578 29.792 24.326 0.00 0.00 PROA
ATOM 59 C ILE P 4 7.949 25.454 27.156 0.00 0.00 PROA
ATOM 60 O ILE P 4 7.397 25.885 28.123 0.00 0.00 PROA
ATOM 61 N SER P 5 8.699 24.382 27.207 0.00 0.00 PROA
ATOM 62 HN SER P 5 9.058 23.945 26.386 0.00 0.00 PROA
ATOM 63 CA SER P 5 8.920 23.647 28.477 0.00 0.00 PROA
ATOM 64 HA SER P 5 8.338 24.054 29.290 0.00 0.00 PROA
ATOM 65 CB SER P 5 8.665 22.120 28.363 0.00 0.00 PROA
ATOM 66 HB1 SER P 5 9.449 21.566 27.804 0.00 0.00 PROA
ATOM 67 HB2 SER P 5 7.578 21.977 28.183 0.00 0.00 PROA
ATOM 68 OG SER P 5 8.997 21.639 29.661 0.00 0.00 PROA
ATOM 69 HG1 SER P 5 8.149 21.370 30.023 0.00 0.00 PROA
ATOM 70 C SER P 5 10.331 23.893 28.840 0.00 0.00 PROA
ATOM 71 O SER P 5 11.295 23.624 28.101 0.00 0.00 PROA
ATOM 72 N ILE P 6 10.654 24.392 30.060 0.00 0.00 PROA
ATOM 73 HN ILE P 6 9.919 24.759 30.625 0.00 0.00 PROA
ATOM 74 CA ILE P 6 12.033 24.613 30.505 0.00 0.00 PROA
ATOM 75 HA ILE P 6 12.701 24.709 29.661 0.00 0.00 PROA
ATOM 76 CB ILE P 6 12.216 25.935 31.309 0.00 0.00 PROA
ATOM 77 HB ILE P 6 11.628 26.668 30.717 0.00 0.00 PROA
ATOM 78 CG2 ILE P 6 11.628 25.904 32.776 0.00 0.00 PROA
ATOM 79 HG21 ILE P 6 12.356 25.251 33.302 0.00 0.00 PROA
ATOM 80 HG22 ILE P 6 10.626 25.456 32.952 0.00 0.00 PROA
ATOM 81 HG23 ILE P 6 11.678 26.948 33.154 0.00 0.00 PROA
ATOM 82 CG1 ILE P 6 13.647 26.542 31.289 0.00 0.00 PROA
ATOM 83 HG11 ILE P 6 14.278 25.952 31.987 0.00 0.00 PROA
ATOM 84 HG12 ILE P 6 13.594 27.506 31.838 0.00 0.00 PROA
ATOM 85 CD ILE P 6 14.311 26.800 29.930 0.00 0.00 PROA
ATOM 86 HD1 ILE P 6 14.427 25.816 29.426 0.00 0.00 PROA
ATOM 87 HD2 ILE P 6 15.237 27.412 29.925 0.00 0.00 PROA
ATOM 88 HD3 ILE P 6 13.688 27.527 29.366 0.00 0.00 PROA
ATOM 89 C ILE P 6 12.557 23.447 31.255 0.00 0.00 PROA
ATOM 90 O ILE P 6 13.707 23.359 31.609 0.00 0.00 PROA
ATOM 91 N TYR P 7 11.623 22.492 31.538 0.00 0.00 PROA
ATOM 92 HN TYR P 7 10.691 22.717 31.266 0.00 0.00 PROA
ATOM 93 CA TYR P 7 11.811 21.317 32.365 0.00 0.00 PROA
ATOM 94 HA TYR P 7 12.605 21.458 33.083 0.00 0.00 PROA
ATOM 95 CB TYR P 7 10.431 20.986 33.082 0.00 0.00 PROA
ATOM 96 HB1 TYR P 7 10.460 20.065 33.702 0.00 0.00 PROA
ATOM 97 HB2 TYR P 7 9.708 21.004 32.238 0.00 0.00 PROA
ATOM 98 CG TYR P 7 9.929 22.011 34.057 0.00 0.00 PROA
ATOM 99 CD1 TYR P 7 8.522 22.326 34.051 0.00 0.00 PROA
ATOM 100 HD1 TYR P 7 7.832 21.950 33.311 0.00 0.00 PROA
ATOM 101 CE1 TYR P 7 8.036 23.391 34.868 0.00 0.00 PROA
ATOM 102 HE1 TYR P 7 6.991 23.650 34.953 0.00 0.00 PROA
ATOM 103 CZ TYR P 7 8.949 23.911 35.788 0.00 0.00 PROA
ATOM 104 OH TYR P 7 8.479 24.875 36.683 0.00 0.00 PROA
ATOM 105 HH TYR P 7 9.210 25.050 37.280 0.00 0.00 PROA
ATOM 106 CD2 TYR P 7 10.724 22.529 35.054 0.00 0.00 PROA
ATOM 107 HD2 TYR P 7 11.705 22.092 35.170 0.00 0.00 PROA
ATOM 108 CE2 TYR P 7 10.230 23.445 35.944 0.00 0.00 PROA
ATOM 109 HE2 TYR P 7 10.802 23.834 36.774 0.00 0.00 PROA
ATOM 110 C TYR P 7 12.273 20.069 31.589 0.00 0.00 PROA
ATOM 111 O TYR P 7 13.198 19.299 31.959 0.00 0.00 PROA
ATOM 112 N THR P 8 11.550 19.670 30.523 0.00 0.00 PROA
ATOM 113 HN THR P 8 10.813 20.269 30.221 0.00 0.00 PROA
ATOM 114 CA THR P 8 11.724 18.450 29.781 0.00 0.00 PROA
ATOM 115 HA THR P 8 11.560 17.699 30.540 0.00 0.00 PROA
ATOM 116 CB THR P 8 10.607 18.396 28.753 0.00 0.00 PROA
ATOM 117 HB THR P 8 10.648 19.310 28.122 0.00 0.00 PROA
ATOM 118 OG1 THR P 8 9.342 18.374 29.395 0.00 0.00 PROA
```

ATOM	119	HG1	THR	P	8	9.054	19.269	29.590	0.00	0.00	PROA
ATOM	120	CG2	THR	P	8	10.710	17.147	27.894	0.00	0.00	PROA
ATOM	121	HG21	THR	P	8	11.503	17.216	27.119	0.00	0.00	PROA
ATOM	122	HG22	THR	P	8	9.734	16.940	27.404	0.00	0.00	PROA
ATOM	123	HG23	THR	P	8	11.037	16.254	28.469	0.00	0.00	PROA
ATOM	124	C	THR	P	8	13.059	18.285	29.124	0.00	0.00	PROA
ATOM	125	O	THR	P	8	13.579	19.190	28.475	0.00	0.00	PROA
ATOM	126	N	SER	P	9	13.676	17.028	29.176	0.00	0.00	PROA
ATOM	127	HN	SER	P	9	13.188	16.350	29.720	0.00	0.00	PROA
ATOM	128	CA	SER	P	9	14.907	16.642	28.537	0.00	0.00	PROA
ATOM	129	HA	SER	P	9	15.352	17.494	28.046	0.00	0.00	PROA
ATOM	130	CB	SER	P	9	15.903	16.050	29.464	0.00	0.00	PROA
ATOM	131	HB1	SER	P	9	16.201	16.689	30.322	0.00	0.00	PROA
ATOM	132	HB2	SER	P	9	16.785	15.648	28.921	0.00	0.00	PROA
ATOM	133	OG	SER	P	9	15.488	14.825	30.084	0.00	0.00	PROA
ATOM	134	HG1	SER	P	9	14.844	15.115	30.735	0.00	0.00	PROA
ATOM	135	C	SER	P	9	14.534	15.706	27.348	0.00	0.00	PROA
ATOM	136	O	SER	P	9	13.707	14.850	27.321	0.00	0.00	PROA
ATOM	137	N	ASP	P	10	15.277	15.946	26.165	0.00	0.00	PROA
ATOM	138	HN	ASP	P	10	16.016	16.615	26.169	0.00	0.00	PROA
ATOM	139	CA	ASP	P	10	14.974	15.379	24.879	0.00	0.00	PROA
ATOM	140	HA	ASP	P	10	14.145	15.927	24.456	0.00	0.00	PROA
ATOM	141	CB	ASP	P	10	16.147	15.711	24.010	0.00	0.00	PROA
ATOM	142	HB1	ASP	P	10	16.162	14.961	23.191	0.00	0.00	PROA
ATOM	143	HB2	ASP	P	10	17.083	15.404	24.524	0.00	0.00	PROA
ATOM	144	CG	ASP	P	10	16.194	17.147	23.541	0.00	0.00	PROA
ATOM	145	OD1	ASP	P	10	15.165	17.885	23.460	0.00	0.00	PROA
ATOM	146	OD2	ASP	P	10	17.241	17.577	22.983	0.00	0.00	PROA
ATOM	147	C	ASP	P	10	14.652	13.897	24.699	0.00	0.00	PROA
ATOM	148	O	ASP	P	10	13.561	13.492	24.174	0.00	0.00	PROA
ATOM	149	N	ASN	P	11	15.610	13.037	25.071	0.00	0.00	PROA
ATOM	150	HN	ASN	P	11	16.514	13.375	25.320	0.00	0.00	PROA
ATOM	151	CA	ASN	P	11	15.393	11.580	25.100	0.00	0.00	PROA
ATOM	152	HA	ASN	P	11	14.764	11.325	24.260	0.00	0.00	PROA
ATOM	153	CB	ASN	P	11	16.726	10.727	25.035	0.00	0.00	PROA
ATOM	154	HB1	ASN	P	11	16.644	9.667	24.715	0.00	0.00	PROA
ATOM	155	HB2	ASN	P	11	17.220	10.734	26.031	0.00	0.00	PROA
ATOM	156	CG	ASN	P	11	17.774	11.313	24.132	0.00	0.00	PROA
ATOM	157	OD1	ASN	P	11	18.388	12.274	24.629	0.00	0.00	PROA
ATOM	158	ND2	ASN	P	11	17.941	10.743	22.938	0.00	0.00	PROA
ATOM	159	HD21	ASN	P	11	17.267	10.110	22.558	0.00	0.00	PROA
ATOM	160	HD22	ASN	P	11	18.706	11.094	22.399	0.00	0.00	PROA
ATOM	161	C	ASN	P	11	14.671	10.956	26.333	0.00	0.00	PROA
ATOM	162	O	ASN	P	11	14.648	9.700	26.425	0.00	0.00	PROA
ATOM	163	N	TYR	P	12	14.046	11.737	27.220	0.00	0.00	PROA
ATOM	164	HN	TYR	P	12	14.207	12.721	27.200	0.00	0.00	PROA
ATOM	165	CA	TYR	P	12	13.505	11.265	28.427	0.00	0.00	PROA
ATOM	166	HA	TYR	P	12	13.465	10.185	28.437	0.00	0.00	PROA
ATOM	167	CB	TYR	P	12	14.462	11.717	29.592	0.00	0.00	PROA
ATOM	168	HB1	TYR	P	12	14.092	11.284	30.545	0.00	0.00	PROA
ATOM	169	HB2	TYR	P	12	14.446	12.827	29.653	0.00	0.00	PROA
ATOM	170	CG	TYR	P	12	15.923	11.261	29.471	0.00	0.00	PROA
ATOM	171	CD1	TYR	P	12	16.863	12.096	28.849	0.00	0.00	PROA
ATOM	172	HD1	TYR	P	12	16.541	12.968	28.300	0.00	0.00	PROA
ATOM	173	CE1	TYR	P	12	18.150	11.670	28.671	0.00	0.00	PROA
ATOM	174	HE1	TYR	P	12	18.864	12.343	28.221	0.00	0.00	PROA
ATOM	175	CZ	TYR	P	12	18.581	10.460	29.323	0.00	0.00	PROA
ATOM	176	OH	TYR	P	12	19.960	10.168	29.461	0.00	0.00	PROA
ATOM	177	HH	TYR	P	12	19.980	9.333	29.934	0.00	0.00	PROA
ATOM	178	CD2	TYR	P	12	16.295	10.001	29.984	0.00	0.00	PROA
ATOM	179	HD2	TYR	P	12	15.514	9.325	30.301	0.00	0.00	PROA

```
ATOM    180  CE2 TYR P  12      17.658   9.583  29.847  0.00  0.00      PROA
ATOM    181  HE2 TYR P  12      18.060   8.729  30.371  0.00  0.00      PROA
ATOM    182  C   TYR P  12      12.070  11.669  28.622  0.00  0.00      PROA
ATOM    183  O   TYR P  12      11.570  12.665  28.125  0.00  0.00      PROA
ATOM    184  N   THR P  13      11.361  10.922  29.412  0.00  0.00      PROA
ATOM    185  HN  THR P  13      11.784  10.102  29.788  0.00  0.00      PROA
ATOM    186  CA  THR P  13       9.994  11.241  29.769  0.00  0.00      PROA
ATOM    187  HA  THR P  13       9.426  11.172  28.853  0.00  0.00      PROA
ATOM    188  CB  THR P  13       9.337  10.200  30.659  0.00  0.00      PROA
ATOM    189  HB  THR P  13       9.265   9.242  30.100  0.00  0.00      PROA
ATOM    190  OG1 THR P  13       8.066  10.593  31.178  0.00  0.00      PROA
ATOM    191  HG1 THR P  13       8.283  11.058  31.990  0.00  0.00      PROA
ATOM    192  CG2 THR P  13      10.212   9.910  31.916  0.00  0.00      PROA
ATOM    193 HG21 THR P  13       9.906   8.871  32.163  0.00  0.00      PROA
ATOM    194 HG22 THR P  13       9.945  10.665  32.685  0.00  0.00      PROA
ATOM    195 HG23 THR P  13      11.297  10.007  31.693  0.00  0.00      PROA
ATOM    196  C   THR P  13       9.774  12.656  30.271  0.00  0.00      PROA
ATOM    197  O   THR P  13      10.550  13.248  30.977  0.00  0.00      PROA
ATOM    198  N   GLU P  14       8.727  13.238  29.745  0.00  0.00      PROA
ATOM    199  HN  GLU P  14       8.046  12.697  29.257  0.00  0.00      PROA
ATOM    200  CA  GLU P  14       8.374  14.640  29.793  0.00  0.00      PROA
ATOM    201  HA  GLU P  14       9.307  15.087  29.484  0.00  0.00      PROA
ATOM    202  CB  GLU P  14       7.271  15.068  28.746  0.00  0.00      PROA
ATOM    203  HB1 GLU P  14       7.181  16.171  28.840  0.00  0.00      PROA
ATOM    204  HB2 GLU P  14       6.270  14.631  28.947  0.00  0.00      PROA
ATOM    205  CG  GLU P  14       7.662  14.876  27.269  0.00  0.00      PROA
ATOM    206  HG1 GLU P  14       8.478  14.122  27.243  0.00  0.00      PROA
ATOM    207  HG2 GLU P  14       7.991  15.857  26.866  0.00  0.00      PROA
ATOM    208  CD  GLU P  14       6.510  14.339  26.467  0.00  0.00      PROA
ATOM    209  OE1 GLU P  14       6.261  14.888  25.329  0.00  0.00      PROA
ATOM    210  OE2 GLU P  14       5.989  13.266  26.821  0.00  0.00      PROA
ATOM    211  C   GLU P  14       8.169  15.248  31.245  0.00  0.00      PROA
ATOM    212  O   GLU P  14       7.657  14.526  32.085  0.00  0.00      PROA
ATOM    213  N   GLU P  15       8.707  16.503  31.550  0.00  0.00      PROA
ATOM    214  HN  GLU P  15       9.184  17.022  30.845  0.00  0.00      PROA
ATOM    215  CA  GLU P  15       8.462  17.066  32.891  0.00  0.00      PROA
ATOM    216  HA  GLU P  15       7.865  16.430  33.529  0.00  0.00      PROA
ATOM    217  CB  GLU P  15       9.646  17.394  33.687  0.00  0.00      PROA
ATOM    218  HB1 GLU P  15       9.423  18.046  34.558  0.00  0.00      PROA
ATOM    219  HB2 GLU P  15      10.306  18.006  33.036  0.00  0.00      PROA
ATOM    220  CG  GLU P  15      10.363  16.069  34.177  0.00  0.00      PROA
ATOM    221  HG1 GLU P  15      10.980  15.717  33.323  0.00  0.00      PROA
ATOM    222  HG2 GLU P  15       9.827  15.205  34.625  0.00  0.00      PROA
ATOM    223  CD  GLU P  15      11.341  16.476  35.245  0.00  0.00      PROA
ATOM    224  OE1 GLU P  15      11.043  16.938  36.388  0.00  0.00      PROA
ATOM    225  OE2 GLU P  15      12.564  16.340  34.939  0.00  0.00      PROA
ATOM    226  C   GLU P  15       7.598  18.371  32.840  0.00  0.00      PROA
ATOM    227  O   GLU P  15       7.706  19.250  31.941  0.00  0.00      PROA
ATOM    228  N   MET P  16       6.716  18.487  33.857  0.00  0.00      PROA
ATOM    229  HN  MET P  16       6.654  17.763  34.540  0.00  0.00      PROA
ATOM    230  CA  MET P  16       5.678  19.518  33.967  0.00  0.00      PROA
ATOM    231  HA  MET P  16       6.247  20.416  33.779  0.00  0.00      PROA
ATOM    232  CB  MET P  16       4.507  19.453  32.930  0.00  0.00      PROA
ATOM    233  HB1 MET P  16       5.050  19.425  31.961  0.00  0.00      PROA
ATOM    234  HB2 MET P  16       3.939  20.404  32.851  0.00  0.00      PROA
ATOM    235  CG  MET P  16       3.748  18.188  32.978  0.00  0.00      PROA
ATOM    236  HG1 MET P  16       2.792  18.322  33.528  0.00  0.00      PROA
ATOM    237  HG2 MET P  16       4.251  17.426  33.611  0.00  0.00      PROA
ATOM    238  SD  MET P  16       3.385  17.449  31.352  0.00  0.00      PROA
ATOM    239  CE  MET P  16       5.110  16.908  31.019  0.00  0.00      PROA
ATOM    240  HE1 MET P  16       5.500  16.242  31.818  0.00  0.00      PROA
```

```
ATOM    241  HE2 MET P  16       5.862  17.680  30.751  0.00  0.00      PROA
ATOM    242  HE3 MET P  16       4.992  16.269  30.119  0.00  0.00      PROA
ATOM    243  C   MET P  16       5.098  19.557  35.368  0.00  0.00      PROA
ATOM    244  O   MET P  16       5.202  18.631  36.196  0.00  0.00      PROA
ATOM    245  N   GLY P  17       4.632  20.816  35.736  0.00  0.00      PROA
ATOM    246  HN  GLY P  17       4.616  21.524  35.035  0.00  0.00      PROA
ATOM    247  CA  GLY P  17       4.026  21.026  37.051  0.00  0.00      PROA
ATOM    248  HA1 GLY P  17       4.148  22.095  37.148  0.00  0.00      PROA
ATOM    249  HA2 GLY P  17       4.628  20.473  37.757  0.00  0.00      PROA
ATOM    250  C   GLY P  17       2.623  20.535  37.206  0.00  0.00      PROA
ATOM    251  O   GLY P  17       2.065  20.088  36.192  0.00  0.00      PROA
ATOM    252  N   SER P  18       2.114  20.742  38.434  0.00  0.00      PROA
ATOM    253  HN  SER P  18       2.730  21.135  39.112  0.00  0.00      PROA
ATOM    254  CA  SER P  18       0.742  20.472  38.874  0.00  0.00      PROA
ATOM    255  HA  SER P  18       0.521  19.484  38.497  0.00  0.00      PROA
ATOM    256  CB  SER P  18       0.707  20.485  40.441  0.00  0.00      PROA
ATOM    257  HB1 SER P  18       0.538  21.557  40.677  0.00  0.00      PROA
ATOM    258  HB2 SER P  18       1.675  20.245  40.930  0.00  0.00      PROA
ATOM    259  OG  SER P  18      -0.379  19.733  40.907  0.00  0.00      PROA
ATOM    260  HG1 SER P  18      -0.079  18.823  40.863  0.00  0.00      PROA
ATOM    261  C   SER P  18      -0.288  21.343  38.226  0.00  0.00      PROA
ATOM    262  O   SER P  18      -1.449  21.023  38.403  0.00  0.00      PROA
ATOM    263  N   GLY P  19       0.023  22.427  37.486  0.00  0.00      PROA
ATOM    264  HN  GLY P  19       0.979  22.697  37.410  0.00  0.00      PROA
ATOM    265  CA  GLY P  19      -1.000  23.260  36.800  0.00  0.00      PROA
ATOM    266  HA1 GLY P  19      -0.551  24.194  36.498  0.00  0.00      PROA
ATOM    267  HA2 GLY P  19      -1.810  23.552  37.452  0.00  0.00      PROA
ATOM    268  C   GLY P  19      -1.605  22.599  35.531  0.00  0.00      PROA
ATOM    269  O   GLY P  19      -1.338  21.422  35.233  0.00  0.00      PROA
ATOM    270  N   ASP P  20      -2.439  23.439  34.841  0.00  0.00      PROA
ATOM    271  HN  ASP P  20      -2.663  24.369  35.121  0.00  0.00      PROA
ATOM    272  CA  ASP P  20      -3.252  23.043  33.727  0.00  0.00      PROA
ATOM    273  HA  ASP P  20      -4.014  23.806  33.670  0.00  0.00      PROA
ATOM    274  CB  ASP P  20      -2.419  23.172  32.396  0.00  0.00      PROA
ATOM    275  HB1 ASP P  20      -2.984  22.851  31.495  0.00  0.00      PROA
ATOM    276  HB2 ASP P  20      -1.570  22.466  32.520  0.00  0.00      PROA
ATOM    277  CG  ASP P  20      -1.980  24.616  32.097  0.00  0.00      PROA
ATOM    278  OD1 ASP P  20      -0.811  24.824  31.639  0.00  0.00      PROA
ATOM    279  OD2 ASP P  20      -2.821  25.570  32.180  0.00  0.00      PROA
ATOM    280  C   ASP P  20      -4.020  21.652  33.763  0.00  0.00      PROA
ATOM    281  O   ASP P  20      -4.141  21.024  34.821  0.00  0.00      PROA
ATOM    282  N   TYR P  21      -4.517  21.236  32.675  0.00  0.00      PROA
ATOM    283  HN  TYR P  21      -4.435  21.860  31.901  0.00  0.00      PROA
ATOM    284  CA  TYR P  21      -5.351  20.078  32.552  0.00  0.00      PROA
ATOM    285  HA  TYR P  21      -5.513  19.603  33.509  0.00  0.00      PROA
ATOM    286  CB  TYR P  21      -6.856  20.465  32.215  0.00  0.00      PROA
ATOM    287  HB1 TYR P  21      -6.817  21.332  31.522  0.00  0.00      PROA
ATOM    288  HB2 TYR P  21      -7.211  20.900  33.174  0.00  0.00      PROA
ATOM    289  CG  TYR P  21      -7.821  19.338  31.739  0.00  0.00      PROA
ATOM    290  CD1 TYR P  21      -7.849  18.095  32.438  0.00  0.00      PROA
ATOM    291  HD1 TYR P  21      -7.184  18.014  33.285  0.00  0.00      PROA
ATOM    292  CE1 TYR P  21      -8.765  17.084  32.052  0.00  0.00      PROA
ATOM    293  HE1 TYR P  21      -8.795  16.130  32.558  0.00  0.00      PROA
ATOM    294  CZ  TYR P  21      -9.610  17.353  30.954  0.00  0.00      PROA
ATOM    295  OH  TYR P  21     -10.559  16.368  30.556  0.00  0.00      PROA
ATOM    296  HH  TYR P  21     -10.458  15.528  31.011  0.00  0.00      PROA
ATOM    297  CD2 TYR P  21      -8.684  19.550  30.627  0.00  0.00      PROA
ATOM    298  HD2 TYR P  21      -8.447  20.427  30.045  0.00  0.00      PROA
ATOM    299  CE2 TYR P  21      -9.577  18.536  30.289  0.00  0.00      PROA
ATOM    300  HE2 TYR P  21     -10.177  18.908  29.472  0.00  0.00      PROA
ATOM    301  C   TYR P  21      -4.832  18.989  31.643  0.00  0.00      PROA
```

ATOM	302	O	TYR	P	21	-4.965	18.948	30.451	0.00	0.00	PROA
ATOM	303	N	ASP	P	22	-4.281	17.855	32.176	0.00	0.00	PROA
ATOM	304	HN	ASP	P	22	-4.074	17.761	33.147	0.00	0.00	PROA
ATOM	305	CA	ASP	P	22	-3.838	16.765	31.286	0.00	0.00	PROA
ATOM	306	HA	ASP	P	22	-4.651	16.616	30.590	0.00	0.00	PROA
ATOM	307	CB	ASP	P	22	-2.338	16.871	30.710	0.00	0.00	PROA
ATOM	308	HB1	ASP	P	22	-1.675	16.345	31.430	0.00	0.00	PROA
ATOM	309	HB2	ASP	P	22	-1.958	17.910	30.609	0.00	0.00	PROA
ATOM	310	CG	ASP	P	22	-2.081	16.228	29.312	0.00	0.00	PROA
ATOM	311	OD1	ASP	P	22	-1.018	16.609	28.782	0.00	0.00	PROA
ATOM	312	OD2	ASP	P	22	-2.942	15.557	28.751	0.00	0.00	PROA
ATOM	313	C	ASP	P	22	-4.022	15.534	32.050	0.00	0.00	PROA
ATOM	314	O	ASP	P	22	-4.269	15.566	33.287	0.00	0.00	PROA
ATOM	315	N	SER	P	23	-3.963	14.363	31.410	0.00	0.00	PROA
ATOM	316	HN	SER	P	23	-3.855	14.368	30.419	0.00	0.00	PROA
ATOM	317	CA	SER	P	23	-4.057	13.023	31.962	0.00	0.00	PROA
ATOM	318	HA	SER	P	23	-3.973	12.988	33.038	0.00	0.00	PROA
ATOM	319	CB	SER	P	23	-5.202	12.250	31.446	0.00	0.00	PROA
ATOM	320	HB1	SER	P	23	-5.134	12.040	30.357	0.00	0.00	PROA
ATOM	321	HB2	SER	P	23	-6.159	12.809	31.527	0.00	0.00	PROA
ATOM	322	OG	SER	P	23	-5.388	11.034	32.120	0.00	0.00	PROA
ATOM	323	HG1	SER	P	23	-5.568	11.197	33.049	0.00	0.00	PROA
ATOM	324	C	SER	P	23	-2.791	12.347	31.523	0.00	0.00	PROA
ATOM	325	O	SER	P	23	-2.346	12.515	30.359	0.00	0.00	PROA
ATOM	326	N	MET	P	24	-2.147	11.598	32.431	0.00	0.00	PROA
ATOM	327	HN	MET	P	24	-2.611	11.406	33.292	0.00	0.00	PROA
ATOM	328	CA	MET	P	24	-0.969	10.857	32.114	0.00	0.00	PROA
ATOM	329	HA	MET	P	24	-0.414	11.402	31.365	0.00	0.00	PROA
ATOM	330	CB	MET	P	24	-0.001	10.773	33.342	0.00	0.00	PROA
ATOM	331	HB1	MET	P	24	0.905	10.181	33.087	0.00	0.00	PROA
ATOM	332	HB2	MET	P	24	-0.560	10.440	34.242	0.00	0.00	PROA
ATOM	333	CG	MET	P	24	0.607	12.132	33.812	0.00	0.00	PROA
ATOM	334	HG1	MET	P	24	1.365	11.750	34.529	0.00	0.00	PROA
ATOM	335	HG2	MET	P	24	-0.104	12.735	34.418	0.00	0.00	PROA
ATOM	336	SD	MET	P	24	1.418	13.215	32.586	0.00	0.00	PROA
ATOM	337	CE	MET	P	24	0.721	14.684	33.300	0.00	0.00	PROA
ATOM	338	HE1	MET	P	24	1.078	14.740	34.351	0.00	0.00	PROA
ATOM	339	HE2	MET	P	24	-0.388	14.622	33.284	0.00	0.00	PROA
ATOM	340	HE3	MET	P	24	1.055	15.593	32.755	0.00	0.00	PROA
ATOM	341	C	MET	P	24	-1.234	9.554	31.472	0.00	0.00	PROA
ATOM	342	O	MET	P	24	-1.994	8.750	32.082	0.00	0.00	PROA
ATOM	343	N	LYS	P	25	-0.550	9.182	30.385	0.00	0.00	PROA
ATOM	344	HN	LYS	P	25	0.143	9.802	30.025	0.00	0.00	PROA
ATOM	345	CA	LYS	P	25	-0.762	8.022	29.464	0.00	0.00	PROA
ATOM	346	HA	LYS	P	25	-1.670	7.525	29.770	0.00	0.00	PROA
ATOM	347	CB	LYS	P	25	-0.773	8.329	27.931	0.00	0.00	PROA
ATOM	348	HB1	LYS	P	25	-0.955	7.383	27.378	0.00	0.00	PROA
ATOM	349	HB2	LYS	P	25	0.201	8.770	27.629	0.00	0.00	PROA
ATOM	350	CG	LYS	P	25	-1.783	9.404	27.483	0.00	0.00	PROA
ATOM	351	HG1	LYS	P	25	-1.379	10.413	27.715	0.00	0.00	PROA
ATOM	352	HG2	LYS	P	25	-2.701	9.390	28.110	0.00	0.00	PROA
ATOM	353	CD	LYS	P	25	-2.016	9.178	25.958	0.00	0.00	PROA
ATOM	354	HD1	LYS	P	25	-2.414	8.153	25.798	0.00	0.00	PROA
ATOM	355	HD2	LYS	P	25	-1.064	9.249	25.390	0.00	0.00	PROA
ATOM	356	CE	LYS	P	25	-2.958	10.270	25.459	0.00	0.00	PROA
ATOM	357	HE1	LYS	P	25	-2.624	11.276	25.792	0.00	0.00	PROA
ATOM	358	HE2	LYS	P	25	-3.906	10.170	26.029	0.00	0.00	PROA
ATOM	359	NZ	LYS	P	25	-3.218	10.218	24.084	0.00	0.00	PROA
ATOM	360	HZ1	LYS	P	25	-3.011	9.299	23.643	0.00	0.00	PROA
ATOM	361	HZ2	LYS	P	25	-2.519	10.840	23.631	0.00	0.00	PROA
ATOM	362	HZ3	LYS	P	25	-4.176	10.525	23.818	0.00	0.00	PROA

ATOM	363	C	LYS	P	25	0.277	6.983	29.796	0.00	0.00	PROA
ATOM	364	O	LYS	P	25	0.178	5.898	29.254	0.00	0.00	PROA
ATOM	365	N	GLU	P	26	1.177	7.255	30.782	0.00	0.00	PROA
ATOM	366	HN	GLU	P	26	1.315	8.233	30.917	0.00	0.00	PROA
ATOM	367	CA	GLU	P	26	2.071	6.315	31.458	0.00	0.00	PROA
ATOM	368	HA	GLU	P	26	2.945	6.249	30.827	0.00	0.00	PROA
ATOM	369	CB	GLU	P	26	2.785	6.881	32.753	0.00	0.00	PROA
ATOM	370	HB1	GLU	P	26	2.007	7.338	33.401	0.00	0.00	PROA
ATOM	371	HB2	GLU	P	26	3.377	7.741	32.375	0.00	0.00	PROA
ATOM	372	CG	GLU	P	26	3.448	5.873	33.762	0.00	0.00	PROA
ATOM	373	HG1	GLU	P	26	4.130	5.166	33.243	0.00	0.00	PROA
ATOM	374	HG2	GLU	P	26	2.630	5.232	34.155	0.00	0.00	PROA
ATOM	375	CD	GLU	P	26	4.092	6.518	34.964	0.00	0.00	PROA
ATOM	376	OE1	GLU	P	26	3.438	6.584	36.043	0.00	0.00	PROA
ATOM	377	OE2	GLU	P	26	5.260	6.967	34.941	0.00	0.00	PROA
ATOM	378	C	GLU	P	26	1.638	4.900	31.664	0.00	0.00	PROA
ATOM	379	O	GLU	P	26	2.206	3.914	31.157	0.00	0.00	PROA
ATOM	380	N	PRO	P	27	0.627	4.690	32.517	0.00	0.00	PROA
ATOM	381	CD	PRO	P	27	-0.244	5.694	33.114	0.00	0.00	PROA
ATOM	382	HD1	PRO	P	27	-0.691	6.342	32.329	0.00	0.00	PROA
ATOM	383	HD2	PRO	P	27	0.381	6.245	33.849	0.00	0.00	PROA
ATOM	384	CA	PRO	P	27	0.058	3.368	32.625	0.00	0.00	PROA
ATOM	385	HA	PRO	P	27	0.845	2.641	32.756	0.00	0.00	PROA
ATOM	386	CB	PRO	P	27	-0.729	3.471	33.989	0.00	0.00	PROA
ATOM	387	HB1	PRO	P	27	0.065	3.327	34.752	0.00	0.00	PROA
ATOM	388	HB2	PRO	P	27	-1.539	2.734	34.175	0.00	0.00	PROA
ATOM	389	CG	PRO	P	27	-1.280	4.911	33.915	0.00	0.00	PROA
ATOM	390	HG1	PRO	P	27	-2.149	4.840	33.227	0.00	0.00	PROA
ATOM	391	HG2	PRO	P	27	-1.564	5.403	34.870	0.00	0.00	PROA
ATOM	392	C	PRO	P	27	-0.916	2.852	31.531	0.00	0.00	PROA
ATOM	393	O	PRO	P	27	-1.770	1.991	31.864	0.00	0.00	PROA
ATOM	394	N	CYS	P	28	-0.922	3.295	30.260	0.00	0.00	PROA
ATOM	395	HN	CYS	P	28	-0.254	3.906	29.842	0.00	0.00	PROA
ATOM	396	CA	CYS	P	28	-1.961	2.801	29.299	0.00	0.00	PROA
ATOM	397	HA	CYS	P	28	-2.799	2.403	29.852	0.00	0.00	PROA
ATOM	398	CB	CYS	P	28	-2.471	4.033	28.475	0.00	0.00	PROA
ATOM	399	HB1	CYS	P	28	-3.149	3.622	27.697	0.00	0.00	PROA
ATOM	400	HB2	CYS	P	28	-1.558	4.406	27.963	0.00	0.00	PROA
ATOM	401	SG	CYS	P	28	-3.481	5.204	29.488	0.00	0.00	PROA
ATOM	402	C	CYS	P	28	-1.441	1.738	28.317	0.00	0.00	PROA
ATOM	403	O	CYS	P	28	-0.529	1.955	27.504	0.00	0.00	PROA
ATOM	404	N	PHE	P	29	-2.095	0.522	28.344	0.00	0.00	PROA
ATOM	405	HN	PHE	P	29	-2.765	0.494	29.082	0.00	0.00	PROA
ATOM	406	CA	PHE	P	29	-1.578	-0.640	27.723	0.00	0.00	PROA
ATOM	407	HA	PHE	P	29	-0.870	-0.428	26.935	0.00	0.00	PROA
ATOM	408	CB	PHE	P	29	-0.853	-1.654	28.597	0.00	0.00	PROA
ATOM	409	HB1	PHE	P	29	-0.570	-2.600	28.087	0.00	0.00	PROA
ATOM	410	HB2	PHE	P	29	-1.530	-1.912	29.439	0.00	0.00	PROA
ATOM	411	CG	PHE	P	29	0.385	-1.093	29.276	0.00	0.00	PROA
ATOM	412	CD1	PHE	P	29	0.184	-0.572	30.575	0.00	0.00	PROA
ATOM	413	HD1	PHE	P	29	-0.791	-0.528	31.038	0.00	0.00	PROA
ATOM	414	CE1	PHE	P	29	1.293	-0.100	31.321	0.00	0.00	PROA
ATOM	415	HE1	PHE	P	29	1.049	0.281	32.302	0.00	0.00	PROA
ATOM	416	CZ	PHE	P	29	2.554	-0.137	30.787	0.00	0.00	PROA
ATOM	417	HZ	PHE	P	29	3.364	0.275	31.371	0.00	0.00	PROA
ATOM	418	CD2	PHE	P	29	1.667	-1.053	28.722	0.00	0.00	PROA
ATOM	419	HD2	PHE	P	29	1.853	-1.238	27.674	0.00	0.00	PROA
ATOM	420	CE2	PHE	P	29	2.804	-0.708	29.456	0.00	0.00	PROA
ATOM	421	HE2	PHE	P	29	3.729	-0.621	28.905	0.00	0.00	PROA
ATOM	422	C	PHE	P	29	-2.789	-1.345	27.101	0.00	0.00	PROA
ATOM	423	O	PHE	P	29	-3.876	-1.265	27.693	0.00	0.00	PROA

ATOM	424	N	ARG	P	30	-2.699	-1.999	25.903	0.00	0.00	PROA
ATOM	425	HN	ARG	P	30	-1.895	-1.981	25.314	0.00	0.00	PROA
ATOM	426	CA	ARG	P	30	-3.776	-2.733	25.281	0.00	0.00	PROA
ATOM	427	HA	ARG	P	30	-4.630	-2.073	25.322	0.00	0.00	PROA
ATOM	428	CB	ARG	P	30	-3.499	-3.159	23.782	0.00	0.00	PROA
ATOM	429	HB1	ARG	P	30	-3.679	-2.180	23.288	0.00	0.00	PROA
ATOM	430	HB2	ARG	P	30	-4.248	-3.892	23.413	0.00	0.00	PROA
ATOM	431	CG	ARG	P	30	-2.137	-3.784	23.467	0.00	0.00	PROA
ATOM	432	HG1	ARG	P	30	-2.165	-4.878	23.657	0.00	0.00	PROA
ATOM	433	HG2	ARG	P	30	-1.307	-3.510	24.154	0.00	0.00	PROA
ATOM	434	CD	ARG	P	30	-1.744	-3.612	21.968	0.00	0.00	PROA
ATOM	435	HD1	ARG	P	30	-2.360	-4.201	21.255	0.00	0.00	PROA
ATOM	436	HD2	ARG	P	30	-0.718	-4.028	21.881	0.00	0.00	PROA
ATOM	437	NE	ARG	P	30	-1.883	-2.177	21.513	0.00	0.00	PROA
ATOM	438	HE	ARG	P	30	-1.703	-1.450	22.177	0.00	0.00	PROA
ATOM	439	CZ	ARG	P	30	-1.724	-1.750	20.270	0.00	0.00	PROA
ATOM	440	NH1	ARG	P	30	-1.196	-2.507	19.386	0.00	0.00	PROA
ATOM	441	HH11	ARG	P	30	-0.709	-3.353	19.606	0.00	0.00	PROA
ATOM	442	HH12	ARG	P	30	-1.061	-2.114	18.476	0.00	0.00	PROA
ATOM	443	NH2	ARG	P	30	-2.137	-0.517	19.898	0.00	0.00	PROA
ATOM	444	HH21	ARG	P	30	-2.061	-0.209	18.950	0.00	0.00	PROA
ATOM	445	HH22	ARG	P	30	-2.760	-0.035	20.514	0.00	0.00	PROA
ATOM	446	C	ARG	P	30	-4.288	-4.014	25.958	0.00	0.00	PROA
ATOM	447	O	ARG	P	30	-3.507	-4.822	26.384	0.00	0.00	PROA
ATOM	448	N	GLU	P	31	-5.634	-4.235	25.960	0.00	0.00	PROA
ATOM	449	HN	GLU	P	31	-6.254	-3.756	25.344	0.00	0.00	PROA
ATOM	450	CA	GLU	P	31	-6.334	-5.311	26.526	0.00	0.00	PROA
ATOM	451	HA	GLU	P	31	-5.634	-6.044	26.899	0.00	0.00	PROA
ATOM	452	CB	GLU	P	31	-7.074	-4.730	27.732	0.00	0.00	PROA
ATOM	453	HB1	GLU	P	31	-7.834	-5.468	28.066	0.00	0.00	PROA
ATOM	454	HB2	GLU	P	31	-7.706	-3.871	27.419	0.00	0.00	PROA
ATOM	455	CG	GLU	P	31	-6.195	-4.282	28.933	0.00	0.00	PROA
ATOM	456	HG1	GLU	P	31	-5.358	-3.631	28.599	0.00	0.00	PROA
ATOM	457	HG2	GLU	P	31	-5.733	-5.189	29.378	0.00	0.00	PROA
ATOM	458	CD	GLU	P	31	-7.036	-3.453	29.853	0.00	0.00	PROA
ATOM	459	OE1	GLU	P	31	-6.925	-3.706	31.065	0.00	0.00	PROA
ATOM	460	OE2	GLU	P	31	-7.959	-2.657	29.463	0.00	0.00	PROA
ATOM	461	C	GLU	P	31	-7.199	-5.962	25.492	0.00	0.00	PROA
ATOM	462	O	GLU	P	31	-7.735	-5.369	24.544	0.00	0.00	PROA
ATOM	463	N	GLU	P	32	-7.424	-7.263	25.695	0.00	0.00	PROA
ATOM	464	HN	GLU	P	32	-7.206	-7.632	26.595	0.00	0.00	PROA
ATOM	465	CA	GLU	P	32	-8.062	-8.160	24.671	0.00	0.00	PROA
ATOM	466	HA	GLU	P	32	-7.519	-8.039	23.745	0.00	0.00	PROA
ATOM	467	CB	GLU	P	32	-7.891	-9.702	25.007	0.00	0.00	PROA
ATOM	468	HB1	GLU	P	32	-8.496	-10.349	24.337	0.00	0.00	PROA
ATOM	469	HB2	GLU	P	32	-8.232	-9.875	26.050	0.00	0.00	PROA
ATOM	470	CG	GLU	P	32	-6.451	-10.151	24.785	0.00	0.00	PROA
ATOM	471	HG1	GLU	P	32	-5.907	-9.759	25.671	0.00	0.00	PROA
ATOM	472	HG2	GLU	P	32	-5.944	-9.704	23.904	0.00	0.00	PROA
ATOM	473	CD	GLU	P	32	-6.242	-11.668	24.780	0.00	0.00	PROA
ATOM	474	OE1	GLU	P	32	-6.600	-12.309	23.744	0.00	0.00	PROA
ATOM	475	OE2	GLU	P	32	-5.889	-12.247	25.815	0.00	0.00	PROA
ATOM	476	C	GLU	P	32	-9.469	-7.838	24.355	0.00	0.00	PROA
ATOM	477	O	GLU	P	32	-10.273	-7.628	25.217	0.00	0.00	PROA
ATOM	478	N	ASN	P	33	-9.744	-7.810	23.063	0.00	0.00	PROA
ATOM	479	HN	ASN	P	33	-9.062	-8.091	22.393	0.00	0.00	PROA
ATOM	480	CA	ASN	P	33	-11.087	-7.718	22.575	0.00	0.00	PROA
ATOM	481	HA	ASN	P	33	-11.751	-7.302	23.319	0.00	0.00	PROA
ATOM	482	CB	ASN	P	33	-11.244	-7.041	21.192	0.00	0.00	PROA
ATOM	483	HB1	ASN	P	33	-12.191	-7.317	20.682	0.00	0.00	PROA
ATOM	484	HB2	ASN	P	33	-10.382	-7.335	20.556	0.00	0.00	PROA

ATOM	485	CG	ASN	P	33	-11.072	-5.493	21.298	0.00	0.00	PROA
ATOM	486	OD1	ASN	P	33	-10.283	-4.871	20.667	0.00	0.00	PROA
ATOM	487	ND2	ASN	P	33	-11.981	-4.938	22.121	0.00	0.00	PROA
ATOM	488	HD21	ASN	P	33	-12.081	-3.944	22.166	0.00	0.00	PROA
ATOM	489	HD22	ASN	P	33	-12.702	-5.444	22.595	0.00	0.00	PROA
ATOM	490	C	ASN	P	33	-11.549	-9.207	22.219	0.00	0.00	PROA
ATOM	491	O	ASN	P	33	-11.520	-9.719	21.129	0.00	0.00	PROA
ATOM	492	N	ALA	P	34	-12.210	-9.896	23.218	0.00	0.00	PROA
ATOM	493	HN	ALA	P	34	-12.208	-9.396	24.080	0.00	0.00	PROA
ATOM	494	CA	ALA	P	34	-12.578	-11.311	23.041	0.00	0.00	PROA
ATOM	495	HA	ALA	P	34	-11.877	-11.694	22.315	0.00	0.00	PROA
ATOM	496	CB	ALA	P	34	-12.382	-12.169	24.352	0.00	0.00	PROA
ATOM	497	HB1	ALA	P	34	-11.335	-12.129	24.722	0.00	0.00	PROA
ATOM	498	HB2	ALA	P	34	-12.587	-13.216	24.043	0.00	0.00	PROA
ATOM	499	HB3	ALA	P	34	-13.037	-11.831	25.183	0.00	0.00	PROA
ATOM	500	C	ALA	P	34	-13.952	-11.428	22.366	0.00	0.00	PROA
ATOM	501	O	ALA	P	34	-14.377	-12.507	21.886	0.00	0.00	PROA
ATOM	502	N	ASN	P	35	-14.661	-10.246	22.391	0.00	0.00	PROA
ATOM	503	HN	ASN	P	35	-14.144	-9.457	22.713	0.00	0.00	PROA
ATOM	504	CA	ASN	P	35	-16.016	-10.182	21.939	0.00	0.00	PROA
ATOM	505	HA	ASN	P	35	-16.414	-11.181	21.840	0.00	0.00	PROA
ATOM	506	CB	ASN	P	35	-16.961	-9.436	23.024	0.00	0.00	PROA
ATOM	507	HB1	ASN	P	35	-17.929	-9.273	22.504	0.00	0.00	PROA
ATOM	508	HB2	ASN	P	35	-16.474	-8.510	23.398	0.00	0.00	PROA
ATOM	509	CG	ASN	P	35	-17.295	-10.239	24.290	0.00	0.00	PROA
ATOM	510	OD1	ASN	P	35	-16.842	-11.436	24.324	0.00	0.00	PROA
ATOM	511	ND2	ASN	P	35	-17.990	-9.632	25.274	0.00	0.00	PROA
ATOM	512	HD21	ASN	P	35	-18.292	-10.171	26.059	0.00	0.00	PROA
ATOM	513	HD22	ASN	P	35	-18.110	-8.645	25.161	0.00	0.00	PROA
ATOM	514	C	ASN	P	35	-15.985	-9.466	20.587	0.00	0.00	PROA
ATOM	515	O	ASN	P	35	-15.341	-8.434	20.391	0.00	0.00	PROA
ATOM	516	N	PHE	P	36	-16.594	-10.002	19.521	0.00	0.00	PROA
ATOM	517	HN	PHE	P	36	-17.092	-10.857	19.641	0.00	0.00	PROA
ATOM	518	CA	PHE	P	36	-16.622	-9.421	18.165	0.00	0.00	PROA
ATOM	519	HA	PHE	P	36	-17.368	-9.986	17.627	0.00	0.00	PROA
ATOM	520	CB	PHE	P	36	-17.294	-7.961	18.118	0.00	0.00	PROA
ATOM	521	HB1	PHE	P	36	-16.719	-7.236	18.733	0.00	0.00	PROA
ATOM	522	HB2	PHE	P	36	-18.297	-8.153	18.554	0.00	0.00	PROA
ATOM	523	CG	PHE	P	36	-17.432	-7.299	16.746	0.00	0.00	PROA
ATOM	524	CD1	PHE	P	36	-16.601	-6.361	16.153	0.00	0.00	PROA
ATOM	525	HD1	PHE	P	36	-15.869	-5.880	16.785	0.00	0.00	PROA
ATOM	526	CE1	PHE	P	36	-16.665	-6.005	14.838	0.00	0.00	PROA
ATOM	527	HE1	PHE	P	36	-16.053	-5.224	14.411	0.00	0.00	PROA
ATOM	528	CZ	PHE	P	36	-17.664	-6.670	14.054	0.00	0.00	PROA
ATOM	529	HZ	PHE	P	36	-17.767	-6.381	13.018	0.00	0.00	PROA
ATOM	530	CD2	PHE	P	36	-18.334	-7.928	15.983	0.00	0.00	PROA
ATOM	531	HD2	PHE	P	36	-18.928	-8.738	16.379	0.00	0.00	PROA
ATOM	532	CE2	PHE	P	36	-18.544	-7.567	14.675	0.00	0.00	PROA
ATOM	533	HE2	PHE	P	36	-19.231	-8.076	14.016	0.00	0.00	PROA
ATOM	534	C	PHE	P	36	-15.329	-9.625	17.329	0.00	0.00	PROA
ATOM	535	O	PHE	P	36	-15.362	-10.277	16.294	0.00	0.00	PROA
ATOM	536	N	ASN	P	37	-14.188	-9.056	17.769	0.00	0.00	PROA
ATOM	537	HN	ASN	P	37	-14.231	-8.580	18.644	0.00	0.00	PROA
ATOM	538	CA	ASN	P	37	-12.938	-9.032	17.002	0.00	0.00	PROA
ATOM	539	HA	ASN	P	37	-13.161	-8.587	16.043	0.00	0.00	PROA
ATOM	540	CB	ASN	P	37	-11.848	-8.083	17.597	0.00	0.00	PROA
ATOM	541	HB1	ASN	P	37	-10.880	-8.172	17.059	0.00	0.00	PROA
ATOM	542	HB2	ASN	P	37	-11.607	-8.346	18.649	0.00	0.00	PROA
ATOM	543	CG	ASN	P	37	-12.233	-6.577	17.587	0.00	0.00	PROA
ATOM	544	OD1	ASN	P	37	-13.382	-6.212	17.472	0.00	0.00	PROA
ATOM	545	ND2	ASN	P	37	-11.245	-5.689	17.640	0.00	0.00	PROA

ATOM	546	HD21	ASN	P	37	-11.476	-4.719	17.708	0.00	0.00	PROA
ATOM	547	HD22	ASN	P	37	-10.423	-6.067	18.066	0.00	0.00	PROA
ATOM	548	C	ASN	P	37	-12.357	-10.426	16.681	0.00	0.00	PROA
ATOM	549	O	ASN	P	37	-11.850	-10.682	15.606	0.00	0.00	PROA
ATOM	550	N	LYS	P	38	-12.381	-11.385	17.586	0.00	0.00	PROA
ATOM	551	HN	LYS	P	38	-12.773	-11.210	18.486	0.00	0.00	PROA
ATOM	552	CA	LYS	P	38	-11.814	-12.740	17.404	0.00	0.00	PROA
ATOM	553	HA	LYS	P	38	-10.784	-12.477	17.211	0.00	0.00	PROA
ATOM	554	CB	LYS	P	38	-11.845	-13.712	18.625	0.00	0.00	PROA
ATOM	555	HB1	LYS	P	38	-11.326	-14.656	18.354	0.00	0.00	PROA
ATOM	556	HB2	LYS	P	38	-12.935	-13.763	18.832	0.00	0.00	PROA
ATOM	557	CG	LYS	P	38	-11.301	-13.262	19.997	0.00	0.00	PROA
ATOM	558	HG1	LYS	P	38	-11.463	-13.954	20.852	0.00	0.00	PROA
ATOM	559	HG2	LYS	P	38	-11.731	-12.240	20.054	0.00	0.00	PROA
ATOM	560	CD	LYS	P	38	-9.751	-13.221	19.953	0.00	0.00	PROA
ATOM	561	HD1	LYS	P	38	-9.418	-12.429	19.249	0.00	0.00	PROA
ATOM	562	HD2	LYS	P	38	-9.366	-14.183	19.551	0.00	0.00	PROA
ATOM	563	CE	LYS	P	38	-9.002	-13.031	21.290	0.00	0.00	PROA
ATOM	564	HE1	LYS	P	38	-9.230	-13.928	21.905	0.00	0.00	PROA
ATOM	565	HE2	LYS	P	38	-9.364	-12.129	21.827	0.00	0.00	PROA
ATOM	566	NZ	LYS	P	38	-7.574	-12.899	21.181	0.00	0.00	PROA
ATOM	567	HZ1	LYS	P	38	-7.216	-13.514	20.422	0.00	0.00	PROA
ATOM	568	HZ2	LYS	P	38	-7.046	-13.006	22.070	0.00	0.00	PROA
ATOM	569	HZ3	LYS	P	38	-7.291	-11.947	20.873	0.00	0.00	PROA
ATOM	570	C	LYS	P	38	-12.346	-13.558	16.224	0.00	0.00	PROA
ATOM	571	O	LYS	P	38	-11.666	-14.268	15.581	0.00	0.00	PROA
ATOM	572	N	ILE	P	39	-13.636	-13.519	15.956	0.00	0.00	PROA
ATOM	573	HN	ILE	P	39	-14.152	-13.044	16.665	0.00	0.00	PROA
ATOM	574	CA	ILE	P	39	-14.220	-14.171	14.859	0.00	0.00	PROA
ATOM	575	HA	ILE	P	39	-13.722	-15.094	14.600	0.00	0.00	PROA
ATOM	576	CB	ILE	P	39	-15.721	-14.421	15.140	0.00	0.00	PROA
ATOM	577	HB	ILE	P	39	-16.133	-13.424	15.404	0.00	0.00	PROA
ATOM	578	CG2	ILE	P	39	-16.460	-14.869	13.849	0.00	0.00	PROA
ATOM	579	HG21	ILE	P	39	-17.515	-15.192	13.974	0.00	0.00	PROA
ATOM	580	HG22	ILE	P	39	-15.883	-15.705	13.399	0.00	0.00	PROA
ATOM	581	HG23	ILE	P	39	-16.582	-13.950	13.237	0.00	0.00	PROA
ATOM	582	CG1	ILE	P	39	-15.934	-15.420	16.270	0.00	0.00	PROA
ATOM	583	HG11	ILE	P	39	-15.211	-15.213	17.089	0.00	0.00	PROA
ATOM	584	HG12	ILE	P	39	-15.623	-16.448	15.986	0.00	0.00	PROA
ATOM	585	CD	ILE	P	39	-17.335	-15.446	16.837	0.00	0.00	PROA
ATOM	586	HD1	ILE	P	39	-17.726	-14.490	17.248	0.00	0.00	PROA
ATOM	587	HD2	ILE	P	39	-17.480	-16.279	17.558	0.00	0.00	PROA
ATOM	588	HD3	ILE	P	39	-18.017	-15.774	16.024	0.00	0.00	PROA
ATOM	589	C	ILE	P	39	-14.134	-13.223	13.621	0.00	0.00	PROA
ATOM	590	O	ILE	P	39	-13.820	-13.661	12.521	0.00	0.00	PROA
ATOM	591	N	PHE	P	40	-14.431	-11.865	13.804	0.00	0.00	PROA
ATOM	592	HN	PHE	P	40	-14.525	-11.483	14.721	0.00	0.00	PROA
ATOM	593	CA	PHE	P	40	-14.595	-10.935	12.743	0.00	0.00	PROA
ATOM	594	HA	PHE	P	40	-15.259	-11.478	12.086	0.00	0.00	PROA
ATOM	595	CB	PHE	P	40	-15.248	-9.516	13.174	0.00	0.00	PROA
ATOM	596	HB1	PHE	P	40	-14.526	-9.170	13.945	0.00	0.00	PROA
ATOM	597	HB2	PHE	P	40	-16.222	-9.693	13.679	0.00	0.00	PROA
ATOM	598	CG	PHE	P	40	-15.409	-8.415	12.133	0.00	0.00	PROA
ATOM	599	CD1	PHE	P	40	-14.799	-7.123	12.381	0.00	0.00	PROA
ATOM	600	HD1	PHE	P	40	-14.262	-6.955	13.302	0.00	0.00	PROA
ATOM	601	CE1	PHE	P	40	-14.851	-6.102	11.400	0.00	0.00	PROA
ATOM	602	HE1	PHE	P	40	-14.418	-5.130	11.584	0.00	0.00	PROA
ATOM	603	CZ	PHE	P	40	-15.481	-6.362	10.181	0.00	0.00	PROA
ATOM	604	HZ	PHE	P	40	-15.374	-5.651	9.376	0.00	0.00	PROA
ATOM	605	CD2	PHE	P	40	-16.141	-8.595	10.972	0.00	0.00	PROA
ATOM	606	HD2	PHE	P	40	-16.602	-9.545	10.744	0.00	0.00	PROA

ATOM	607	CE2	PHE	P	40	-16.167	-7.606	10.016	0.00	0.00	PROA
ATOM	608	HE2	PHE	P	40	-16.610	-7.745	9.041	0.00	0.00	PROA
ATOM	609	C	PHE	P	40	-13.429	-10.755	11.780	0.00	0.00	PROA
ATOM	610	O	PHE	P	40	-13.642	-10.715	10.550	0.00	0.00	PROA
ATOM	611	N	LEU	P	41	-12.267	-10.489	12.355	0.00	0.00	PROA
ATOM	612	HN	LEU	P	41	-12.239	-10.390	13.346	0.00	0.00	PROA
ATOM	613	CA	LEU	P	41	-11.047	-10.176	11.720	0.00	0.00	PROA
ATOM	614	HA	LEU	P	41	-11.304	-9.565	10.868	0.00	0.00	PROA
ATOM	615	CB	LEU	P	41	-10.060	-9.363	12.550	0.00	0.00	PROA
ATOM	616	HB1	LEU	P	41	-9.144	-9.308	11.925	0.00	0.00	PROA
ATOM	617	HB2	LEU	P	41	-9.832	-9.899	13.496	0.00	0.00	PROA
ATOM	618	CG	LEU	P	41	-10.509	-7.964	12.970	0.00	0.00	PROA
ATOM	619	HG	LEU	P	41	-11.467	-8.187	13.486	0.00	0.00	PROA
ATOM	620	CD1	LEU	P	41	-9.615	-7.264	13.947	0.00	0.00	PROA
ATOM	621	HD11	LEU	P	41	-9.339	-7.968	14.761	0.00	0.00	PROA
ATOM	622	HD12	LEU	P	41	-10.225	-6.531	14.517	0.00	0.00	PROA
ATOM	623	HD13	LEU	P	41	-8.768	-6.708	13.491	0.00	0.00	PROA
ATOM	624	CD2	LEU	P	41	-10.850	-6.985	11.874	0.00	0.00	PROA
ATOM	625	HD21	LEU	P	41	-11.117	-5.976	12.254	0.00	0.00	PROA
ATOM	626	HD22	LEU	P	41	-11.665	-7.271	11.174	0.00	0.00	PROA
ATOM	627	HD23	LEU	P	41	-9.960	-6.826	11.229	0.00	0.00	PROA
ATOM	628	C	LEU	P	41	-10.360	-11.420	10.998	0.00	0.00	PROA
ATOM	629	O	LEU	P	41	-10.085	-11.281	9.801	0.00	0.00	PROA
ATOM	630	N	PRO	P	42	-10.150	-12.566	11.437	0.00	0.00	PROA
ATOM	631	CD	PRO	P	42	-10.136	-12.945	12.926	0.00	0.00	PROA
ATOM	632	HD1	PRO	P	42	-11.201	-13.081	13.212	0.00	0.00	PROA
ATOM	633	HD2	PRO	P	42	-9.707	-12.124	13.538	0.00	0.00	PROA
ATOM	634	CA	PRO	P	42	-9.867	-13.859	10.717	0.00	0.00	PROA
ATOM	635	HA	PRO	P	42	-8.866	-13.718	10.338	0.00	0.00	PROA
ATOM	636	CB	PRO	P	42	-9.927	-14.930	11.778	0.00	0.00	PROA
ATOM	637	HB1	PRO	P	42	-9.305	-15.786	11.440	0.00	0.00	PROA
ATOM	638	HB2	PRO	P	42	-10.965	-15.286	11.950	0.00	0.00	PROA
ATOM	639	CG	PRO	P	42	-9.462	-14.213	13.014	0.00	0.00	PROA
ATOM	640	HG1	PRO	P	42	-9.796	-14.855	13.857	0.00	0.00	PROA
ATOM	641	HG2	PRO	P	42	-8.357	-14.122	12.950	0.00	0.00	PROA
ATOM	642	C	PRO	P	42	-10.778	-14.131	9.593	0.00	0.00	PROA
ATOM	643	O	PRO	P	42	-10.444	-14.554	8.529	0.00	0.00	PROA
ATOM	644	N	THR	P	43	-12.152	-13.835	9.807	0.00	0.00	PROA
ATOM	645	HN	THR	P	43	-12.307	-13.561	10.753	0.00	0.00	PROA
ATOM	646	CA	THR	P	43	-13.202	-13.940	8.772	0.00	0.00	PROA
ATOM	647	HA	THR	P	43	-13.042	-14.893	8.289	0.00	0.00	PROA
ATOM	648	CB	THR	P	43	-14.655	-13.888	9.242	0.00	0.00	PROA
ATOM	649	HB	THR	P	43	-14.922	-12.895	9.664	0.00	0.00	PROA
ATOM	650	OG1	THR	P	43	-14.864	-14.934	10.160	0.00	0.00	PROA
ATOM	651	HG1	THR	P	43	-14.463	-14.668	10.991	0.00	0.00	PROA
ATOM	652	CG2	THR	P	43	-15.657	-14.113	8.084	0.00	0.00	PROA
ATOM	653	HG21	THR	P	43	-15.683	-13.254	7.379	0.00	0.00	PROA
ATOM	654	HG22	THR	P	43	-16.669	-14.262	8.518	0.00	0.00	PROA
ATOM	655	HG23	THR	P	43	-15.377	-14.975	7.441	0.00	0.00	PROA
ATOM	656	C	THR	P	43	-12.999	-12.898	7.584	0.00	0.00	PROA
ATOM	657	O	THR	P	43	-12.988	-13.331	6.425	0.00	0.00	PROA
ATOM	658	N	ILE	P	44	-12.689	-11.595	7.931	0.00	0.00	PROA
ATOM	659	HN	ILE	P	44	-12.663	-11.335	8.893	0.00	0.00	PROA
ATOM	660	CA	ILE	P	44	-12.311	-10.619	6.874	0.00	0.00	PROA
ATOM	661	HA	ILE	P	44	-13.144	-10.675	6.190	0.00	0.00	PROA
ATOM	662	CB	ILE	P	44	-12.144	-9.196	7.457	0.00	0.00	PROA
ATOM	663	HB	ILE	P	44	-11.593	-9.366	8.407	0.00	0.00	PROA
ATOM	664	CG2	ILE	P	44	-11.428	-8.141	6.635	0.00	0.00	PROA
ATOM	665	HG21	ILE	P	44	-11.802	-8.099	5.589	0.00	0.00	PROA
ATOM	666	HG22	ILE	P	44	-10.349	-8.395	6.703	0.00	0.00	PROA
ATOM	667	HG23	ILE	P	44	-11.503	-7.149	7.129	0.00	0.00	PROA

```
ATOM    668  CG1 ILE P  44     -13.431  -8.493   7.823  0.00  0.00      PROA
ATOM    669 HG11 ILE P  44     -13.236  -7.620   8.481  0.00  0.00      PROA
ATOM    670 HG12 ILE P  44     -14.006  -9.186   8.473  0.00  0.00      PROA
ATOM    671  CD  ILE P  44     -14.304  -8.116   6.530  0.00  0.00      PROA
ATOM    672  HD1 ILE P  44     -15.231  -7.631   6.904  0.00  0.00      PROA
ATOM    673  HD2 ILE P  44     -14.490  -9.065   5.982  0.00  0.00      PROA
ATOM    674  HD3 ILE P  44     -13.665  -7.509   5.854  0.00  0.00      PROA
ATOM    675  C   ILE P  44     -11.017 -11.068   6.170  0.00  0.00      PROA
ATOM    676  O   ILE P  44     -10.985 -11.142   4.947  0.00  0.00      PROA
ATOM    677  N   TYR P  45      -9.937 -11.488   6.949  0.00  0.00      PROA
ATOM    678  HN  TYR P  45      -9.942 -11.359   7.938  0.00  0.00      PROA
ATOM    679  CA  TYR P  45      -8.688 -11.980   6.370  0.00  0.00      PROA
ATOM    680  HA  TYR P  45      -8.247 -11.266   5.690  0.00  0.00      PROA
ATOM    681  CB  TYR P  45      -7.591 -12.424   7.431  0.00  0.00      PROA
ATOM    682  HB1 TYR P  45      -6.682 -12.941   7.057  0.00  0.00      PROA
ATOM    683  HB2 TYR P  45      -8.038 -13.131   8.162  0.00  0.00      PROA
ATOM    684  CG  TYR P  45      -7.036 -11.179   8.122  0.00  0.00      PROA
ATOM    685  CD1 TYR P  45      -6.369 -11.384   9.318  0.00  0.00      PROA
ATOM    686  HD1 TYR P  45      -6.310 -12.416   9.631  0.00  0.00      PROA
ATOM    687  CE1 TYR P  45      -5.702 -10.315   9.964  0.00  0.00      PROA
ATOM    688  HE1 TYR P  45      -5.079 -10.412  10.841  0.00  0.00      PROA
ATOM    689  CZ  TYR P  45      -5.662  -9.082   9.405  0.00  0.00      PROA
ATOM    690  OH  TYR P  45      -4.888  -8.113  10.049  0.00  0.00      PROA
ATOM    691  HH  TYR P  45      -5.069  -7.264   9.640  0.00  0.00      PROA
ATOM    692  CD2 TYR P  45      -7.164  -9.869   7.636  0.00  0.00      PROA
ATOM    693  HD2 TYR P  45      -7.610  -9.677   6.671  0.00  0.00      PROA
ATOM    694  CE2 TYR P  45      -6.369  -8.806   8.190  0.00  0.00      PROA
ATOM    695  HE2 TYR P  45      -6.388  -7.856   7.677  0.00  0.00      PROA
ATOM    696  C   TYR P  45      -8.883 -13.127   5.416  0.00  0.00      PROA
ATOM    697  O   TYR P  45      -8.353 -13.151   4.360  0.00  0.00      PROA
ATOM    698  N   SER P  46      -9.778 -14.153   5.781  0.00  0.00      PROA
ATOM    699  HN  SER P  46     -10.124 -14.148   6.716  0.00  0.00      PROA
ATOM    700  CA  SER P  46     -10.119 -15.337   4.989  0.00  0.00      PROA
ATOM    701  HA  SER P  46      -9.228 -15.944   4.937  0.00  0.00      PROA
ATOM    702  CB  SER P  46     -11.102 -16.223   5.734  0.00  0.00      PROA
ATOM    703  HB1 SER P  46     -11.373 -17.128   5.149  0.00  0.00      PROA
ATOM    704  HB2 SER P  46     -12.073 -15.711   5.901  0.00  0.00      PROA
ATOM    705  OG  SER P  46     -10.517 -16.703   6.983  0.00  0.00      PROA
ATOM    706  HG1 SER P  46     -10.357 -15.941   7.544  0.00  0.00      PROA
ATOM    707  C   SER P  46     -10.707 -15.008   3.665  0.00  0.00      PROA
ATOM    708  O   SER P  46     -10.338 -15.514   2.601  0.00  0.00      PROA
ATOM    709  N   ILE P  47     -11.711 -14.055   3.557  0.00  0.00      PROA
ATOM    710  HN  ILE P  47     -11.947 -13.742   4.473  0.00  0.00      PROA
ATOM    711  CA  ILE P  47     -12.450 -13.546   2.358  0.00  0.00      PROA
ATOM    712  HA  ILE P  47     -12.821 -14.388   1.792  0.00  0.00      PROA
ATOM    713  CB  ILE P  47     -13.643 -12.581   2.740  0.00  0.00      PROA
ATOM    714  HB  ILE P  47     -13.379 -11.695   3.357  0.00  0.00      PROA
ATOM    715  CG2 ILE P  47     -14.299 -12.094   1.446  0.00  0.00      PROA
ATOM    716 HG21 ILE P  47     -15.176 -11.482   1.745  0.00  0.00      PROA
ATOM    717 HG22 ILE P  47     -14.644 -12.981   0.873  0.00  0.00      PROA
ATOM    718 HG23 ILE P  47     -13.616 -11.551   0.757  0.00  0.00      PROA
ATOM    719  CG1 ILE P  47     -14.637 -13.396   3.541  0.00  0.00      PROA
ATOM    720 HG11 ILE P  47     -14.162 -14.071   4.286  0.00  0.00      PROA
ATOM    721 HG12 ILE P  47     -15.119 -13.972   2.723  0.00  0.00      PROA
ATOM    722  CD  ILE P  47     -15.608 -12.544   4.358  0.00  0.00      PROA
ATOM    723  HD1 ILE P  47     -16.210 -13.174   5.048  0.00  0.00      PROA
ATOM    724  HD2 ILE P  47     -16.303 -12.066   3.636  0.00  0.00      PROA
ATOM    725  HD3 ILE P  47     -15.042 -11.747   4.886  0.00  0.00      PROA
ATOM    726  C   ILE P  47     -11.477 -12.910   1.413  0.00  0.00      PROA
ATOM    727  O   ILE P  47     -11.412 -13.279   0.225  0.00  0.00      PROA
ATOM    728  N   ILE P  48     -10.585 -11.983   1.898  0.00  0.00      PROA
```

ATOM	729	HN	ILE	P	48	-10.813	-11.764	2.843	0.00	0.00	PROA
ATOM	730	CA	ILE	P	48	-9.440	-11.338	1.210	0.00	0.00	PROA
ATOM	731	HA	ILE	P	48	-9.804	-10.858	0.314	0.00	0.00	PROA
ATOM	732	CB	ILE	P	48	-8.821	-10.258	2.074	0.00	0.00	PROA
ATOM	733	HB	ILE	P	48	-8.420	-10.675	3.023	0.00	0.00	PROA
ATOM	734	CG2	ILE	P	48	-7.562	-9.596	1.379	0.00	0.00	PROA
ATOM	735	HG21	ILE	P	48	-6.876	-10.443	1.162	0.00	0.00	PROA
ATOM	736	HG22	ILE	P	48	-7.114	-8.866	2.087	0.00	0.00	PROA
ATOM	737	HG23	ILE	P	48	-7.867	-9.100	0.433	0.00	0.00	PROA
ATOM	738	CG1	ILE	P	48	-9.871	-9.223	2.634	0.00	0.00	PROA
ATOM	739	HG11	ILE	P	48	-10.786	-9.727	3.012	0.00	0.00	PROA
ATOM	740	HG12	ILE	P	48	-10.121	-8.546	1.789	0.00	0.00	PROA
ATOM	741	CD	ILE	P	48	-9.253	-8.287	3.727	0.00	0.00	PROA
ATOM	742	HD1	ILE	P	48	-9.129	-8.983	4.585	0.00	0.00	PROA
ATOM	743	HD2	ILE	P	48	-9.955	-7.429	3.790	0.00	0.00	PROA
ATOM	744	HD3	ILE	P	48	-8.269	-7.848	3.457	0.00	0.00	PROA
ATOM	745	C	ILE	P	48	-8.333	-12.344	0.710	0.00	0.00	PROA
ATOM	746	O	ILE	P	48	-7.859	-12.340	-0.403	0.00	0.00	PROA
ATOM	747	N	PHE	P	49	-7.943	-13.220	1.678	0.00	0.00	PROA
ATOM	748	HN	PHE	P	49	-8.301	-13.207	2.608	0.00	0.00	PROA
ATOM	749	CA	PHE	P	49	-6.978	-14.308	1.436	0.00	0.00	PROA
ATOM	750	HA	PHE	P	49	-6.088	-13.786	1.116	0.00	0.00	PROA
ATOM	751	CB	PHE	P	49	-6.543	-15.002	2.736	0.00	0.00	PROA
ATOM	752	HB1	PHE	P	49	-7.470	-15.376	3.221	0.00	0.00	PROA
ATOM	753	HB2	PHE	P	49	-6.076	-14.258	3.416	0.00	0.00	PROA
ATOM	754	CG	PHE	P	49	-5.689	-16.241	2.526	0.00	0.00	PROA
ATOM	755	CD1	PHE	P	49	-4.467	-16.032	1.923	0.00	0.00	PROA
ATOM	756	HD1	PHE	P	49	-4.096	-15.022	1.821	0.00	0.00	PROA
ATOM	757	CE1	PHE	P	49	-3.705	-17.087	1.557	0.00	0.00	PROA
ATOM	758	HE1	PHE	P	49	-2.776	-16.884	1.044	0.00	0.00	PROA
ATOM	759	CZ	PHE	P	49	-4.190	-18.401	1.761	0.00	0.00	PROA
ATOM	760	HZ	PHE	P	49	-3.720	-19.250	1.287	0.00	0.00	PROA
ATOM	761	CD2	PHE	P	49	-6.142	-17.540	2.811	0.00	0.00	PROA
ATOM	762	HD2	PHE	P	49	-7.065	-17.687	3.351	0.00	0.00	PROA
ATOM	763	CE2	PHE	P	49	-5.362	-18.675	2.369	0.00	0.00	PROA
ATOM	764	HE2	PHE	P	49	-5.735	-19.687	2.427	0.00	0.00	PROA
ATOM	765	C	PHE	P	49	-7.445	-15.240	0.268	0.00	0.00	PROA
ATOM	766	O	PHE	P	49	-6.626	-15.596	-0.634	0.00	0.00	PROA
ATOM	767	N	LEU	P	50	-8.673	-15.704	0.324	0.00	0.00	PROA
ATOM	768	HN	LEU	P	50	-9.254	-15.494	1.107	0.00	0.00	PROA
ATOM	769	CA	LEU	P	50	-9.293	-16.564	-0.587	0.00	0.00	PROA
ATOM	770	HA	LEU	P	50	-8.541	-17.316	-0.777	0.00	0.00	PROA
ATOM	771	CB	LEU	P	50	-10.490	-17.199	0.082	0.00	0.00	PROA
ATOM	772	HB1	LEU	P	50	-11.110	-16.460	0.633	0.00	0.00	PROA
ATOM	773	HB2	LEU	P	50	-9.927	-17.757	0.861	0.00	0.00	PROA
ATOM	774	CG	LEU	P	50	-11.434	-18.218	-0.593	0.00	0.00	PROA
ATOM	775	HG	LEU	P	50	-11.953	-17.604	-1.360	0.00	0.00	PROA
ATOM	776	CD1	LEU	P	50	-10.737	-19.435	-1.316	0.00	0.00	PROA
ATOM	777	HD11	LEU	P	50	-10.014	-20.013	-0.701	0.00	0.00	PROA
ATOM	778	HD12	LEU	P	50	-10.110	-18.986	-2.116	0.00	0.00	PROA
ATOM	779	HD13	LEU	P	50	-11.531	-20.065	-1.771	0.00	0.00	PROA
ATOM	780	CD2	LEU	P	50	-12.378	-18.753	0.528	0.00	0.00	PROA
ATOM	781	HD21	LEU	P	50	-13.124	-19.515	0.217	0.00	0.00	PROA
ATOM	782	HD22	LEU	P	50	-12.968	-17.859	0.822	0.00	0.00	PROA
ATOM	783	HD23	LEU	P	50	-11.767	-19.194	1.345	0.00	0.00	PROA
ATOM	784	C	LEU	P	50	-9.546	-15.890	-1.943	0.00	0.00	PROA
ATOM	785	O	LEU	P	50	-9.220	-16.439	-2.996	0.00	0.00	PROA
ATOM	786	N	THR	P	51	-9.982	-14.598	-1.881	0.00	0.00	PROA
ATOM	787	HN	THR	P	51	-10.245	-14.303	-0.966	0.00	0.00	PROA
ATOM	788	CA	THR	P	51	-10.141	-13.738	-3.090	0.00	0.00	PROA
ATOM	789	HA	THR	P	51	-10.821	-14.253	-3.751	0.00	0.00	PROA

```
ATOM    790  CB  THR P  51     -10.752 -12.417  -2.636  0.00  0.00      PROA
ATOM    791  HB  THR P  51     -10.213 -12.168  -1.697  0.00  0.00      PROA
ATOM    792  OG1 THR P  51     -12.151 -12.559  -2.385  0.00  0.00      PROA
ATOM    793  HG1 THR P  51     -12.240 -12.634  -1.432  0.00  0.00      PROA
ATOM    794  CG2 THR P  51     -10.599 -11.318  -3.655  0.00  0.00      PROA
ATOM    795 HG21 THR P  51      -9.549 -10.957  -3.712  0.00  0.00      PROA
ATOM    796 HG22 THR P  51     -11.333 -10.487  -3.596  0.00  0.00      PROA
ATOM    797 HG23 THR P  51     -10.997 -11.706  -4.616  0.00  0.00      PROA
ATOM    798  C   THR P  51      -8.802 -13.529  -3.763  0.00  0.00      PROA
ATOM    799  O   THR P  51      -8.764 -13.724  -4.958  0.00  0.00      PROA
ATOM    800  N   GLY P  52      -7.691 -13.284  -3.056  0.00  0.00      PROA
ATOM    801  HN  GLY P  52      -7.684 -13.024  -2.093  0.00  0.00      PROA
ATOM    802  CA  GLY P  52      -6.392 -13.221  -3.680  0.00  0.00      PROA
ATOM    803  HA1 GLY P  52      -5.706 -12.881  -2.919  0.00  0.00      PROA
ATOM    804  HA2 GLY P  52      -6.451 -12.595  -4.558  0.00  0.00      PROA
ATOM    805  C   GLY P  52      -5.794 -14.530  -4.194  0.00  0.00      PROA
ATOM    806  O   GLY P  52      -5.305 -14.558  -5.286  0.00  0.00      PROA
ATOM    807  N   ILE P  53      -5.932 -15.564  -3.424  0.00  0.00      PROA
ATOM    808  HN  ILE P  53      -6.276 -15.401  -2.502  0.00  0.00      PROA
ATOM    809  CA  ILE P  53      -5.559 -16.926  -3.850  0.00  0.00      PROA
ATOM    810  HA  ILE P  53      -4.566 -16.945  -4.273  0.00  0.00      PROA
ATOM    811  CB  ILE P  53      -5.559 -18.004  -2.759  0.00  0.00      PROA
ATOM    812  HB  ILE P  53      -6.473 -17.746  -2.182  0.00  0.00      PROA
ATOM    813  CG2 ILE P  53      -5.865 -19.478  -3.135  0.00  0.00      PROA
ATOM    814 HG21 ILE P  53      -5.721 -20.081  -2.213  0.00  0.00      PROA
ATOM    815 HG22 ILE P  53      -5.159 -19.906  -3.878  0.00  0.00      PROA
ATOM    816 HG23 ILE P  53      -6.859 -19.682  -3.586  0.00  0.00      PROA
ATOM    817  CG1 ILE P  53      -4.356 -17.836  -1.782  0.00  0.00      PROA
ATOM    818 HG11 ILE P  53      -4.677 -18.543  -0.988  0.00  0.00      PROA
ATOM    819 HG12 ILE P  53      -4.339 -16.806  -1.365  0.00  0.00      PROA
ATOM    820  CD  ILE P  53      -2.972 -18.165  -2.377  0.00  0.00      PROA
ATOM    821  HD1 ILE P  53      -2.370 -18.776  -1.670  0.00  0.00      PROA
ATOM    822  HD2 ILE P  53      -2.487 -17.181  -2.553  0.00  0.00      PROA
ATOM    823  HD3 ILE P  53      -3.069 -18.861  -3.238  0.00  0.00      PROA
ATOM    824  C   ILE P  53      -6.311 -17.364  -5.052  0.00  0.00      PROA
ATOM    825  O   ILE P  53      -5.703 -17.764  -6.060  0.00  0.00      PROA
ATOM    826  N   VAL P  54      -7.615 -17.160  -5.167  0.00  0.00      PROA
ATOM    827  HN  VAL P  54      -8.148 -16.867  -4.377  0.00  0.00      PROA
ATOM    828  CA  VAL P  54      -8.365 -17.518  -6.357  0.00  0.00      PROA
ATOM    829  HA  VAL P  54      -7.842 -18.369  -6.767  0.00  0.00      PROA
ATOM    830  CB  VAL P  54      -9.837 -17.751  -6.135  0.00  0.00      PROA
ATOM    831  HB  VAL P  54     -10.232 -17.098  -5.328  0.00  0.00      PROA
ATOM    832  CG1 VAL P  54     -10.575 -17.614  -7.511  0.00  0.00      PROA
ATOM    833 HG11 VAL P  54     -10.701 -16.601  -7.950  0.00  0.00      PROA
ATOM    834 HG12 VAL P  54     -11.586 -17.983  -7.238  0.00  0.00      PROA
ATOM    835 HG13 VAL P  54     -10.099 -18.298  -8.246  0.00  0.00      PROA
ATOM    836  CG2 VAL P  54      -9.948 -19.190  -5.630  0.00  0.00      PROA
ATOM    837 HG21 VAL P  54     -10.778 -19.332  -4.905  0.00  0.00      PROA
ATOM    838 HG22 VAL P  54      -9.018 -19.426  -5.070  0.00  0.00      PROA
ATOM    839 HG23 VAL P  54      -9.996 -19.976  -6.415  0.00  0.00      PROA
ATOM    840  C   VAL P  54      -8.105 -16.530  -7.507  0.00  0.00      PROA
ATOM    841  O   VAL P  54      -8.035 -17.023  -8.633  0.00  0.00      PROA
ATOM    842  N   GLY P  55      -7.869 -15.220  -7.258  0.00  0.00      PROA
ATOM    843  HN  GLY P  55      -8.053 -15.011  -6.300  0.00  0.00      PROA
ATOM    844  CA  GLY P  55      -7.566 -14.144  -8.185  0.00  0.00      PROA
ATOM    845  HA1 GLY P  55      -7.659 -13.179  -7.708  0.00  0.00      PROA
ATOM    846  HA2 GLY P  55      -8.202 -14.387  -9.023  0.00  0.00      PROA
ATOM    847  C   GLY P  55      -6.202 -14.225  -8.865  0.00  0.00      PROA
ATOM    848  O   GLY P  55      -6.119 -14.196 -10.086  0.00  0.00      PROA
ATOM    849  N   ASN P  56      -5.172 -14.536  -8.055  0.00  0.00      PROA
ATOM    850  HN  ASN P  56      -5.332 -14.449  -7.075  0.00  0.00      PROA
```

ATOM	851	CA	ASN	P	56	-3.835	-14.720	-8.558	0.00	0.00	PROA
ATOM	852	HA	ASN	P	56	-3.587	-13.901	-9.217	0.00	0.00	PROA
ATOM	853	CB	ASN	P	56	-2.729	-14.720	-7.406	0.00	0.00	PROA
ATOM	854	HB1	ASN	P	56	-1.699	-15.087	-7.598	0.00	0.00	PROA
ATOM	855	HB2	ASN	P	56	-3.038	-15.508	-6.686	0.00	0.00	PROA
ATOM	856	CG	ASN	P	56	-2.790	-13.395	-6.656	0.00	0.00	PROA
ATOM	857	OD1	ASN	P	56	-3.112	-12.377	-7.316	0.00	0.00	PROA
ATOM	858	ND2	ASN	P	56	-2.377	-13.359	-5.387	0.00	0.00	PROA
ATOM	859	HD21	ASN	P	56	-2.748	-12.595	-4.859	0.00	0.00	PROA
ATOM	860	HD22	ASN	P	56	-2.224	-14.239	-4.937	0.00	0.00	PROA
ATOM	861	C	ASN	P	56	-3.629	-15.863	-9.484	0.00	0.00	PROA
ATOM	862	O	ASN	P	56	-2.943	-15.640	-10.498	0.00	0.00	PROA
ATOM	863	N	GLY	P	57	-4.254	-17.051	-9.345	0.00	0.00	PROA
ATOM	864	HN	GLY	P	57	-4.589	-17.275	-8.433	0.00	0.00	PROA
ATOM	865	CA	GLY	P	57	-4.097	-18.163	-10.367	0.00	0.00	PROA
ATOM	866	HA1	GLY	P	57	-4.622	-19.054	-10.055	0.00	0.00	PROA
ATOM	867	HA2	GLY	P	57	-3.053	-18.364	-10.557	0.00	0.00	PROA
ATOM	868	C	GLY	P	57	-4.777	-17.901	-11.670	0.00	0.00	PROA
ATOM	869	O	GLY	P	57	-4.344	-18.392	-12.686	0.00	0.00	PROA
ATOM	870	N	LEU	P	58	-5.833	-17.077	-11.613	0.00	0.00	PROA
ATOM	871	HN	LEU	P	58	-6.153	-16.792	-10.713	0.00	0.00	PROA
ATOM	872	CA	LEU	P	58	-6.539	-16.634	-12.786	0.00	0.00	PROA
ATOM	873	HA	LEU	P	58	-6.552	-17.417	-13.529	0.00	0.00	PROA
ATOM	874	CB	LEU	P	58	-8.033	-16.302	-12.457	0.00	0.00	PROA
ATOM	875	HB1	LEU	P	58	-8.633	-15.867	-13.285	0.00	0.00	PROA
ATOM	876	HB2	LEU	P	58	-8.107	-15.482	-11.711	0.00	0.00	PROA
ATOM	877	CG	LEU	P	58	-8.945	-17.443	-12.003	0.00	0.00	PROA
ATOM	878	HG	LEU	P	58	-8.480	-17.804	-11.060	0.00	0.00	PROA
ATOM	879	CD1	LEU	P	58	-10.334	-16.834	-11.648	0.00	0.00	PROA
ATOM	880	HD11	LEU	P	58	-10.246	-16.042	-10.874	0.00	0.00	PROA
ATOM	881	HD12	LEU	P	58	-11.009	-17.653	-11.321	0.00	0.00	PROA
ATOM	882	HD13	LEU	P	58	-10.830	-16.404	-12.544	0.00	0.00	PROA
ATOM	883	CD2	LEU	P	58	-9.052	-18.636	-12.924	0.00	0.00	PROA
ATOM	884	HD21	LEU	P	58	-9.782	-19.425	-12.642	0.00	0.00	PROA
ATOM	885	HD22	LEU	P	58	-8.118	-19.234	-12.990	0.00	0.00	PROA
ATOM	886	HD23	LEU	P	58	-9.264	-18.270	-13.951	0.00	0.00	PROA
ATOM	887	C	LEU	P	58	-5.961	-15.493	-13.562	0.00	0.00	PROA
ATOM	888	O	LEU	P	58	-6.049	-15.479	-14.818	0.00	0.00	PROA
ATOM	889	N	VAL	P	59	-5.218	-14.570	-12.870	0.00	0.00	PROA
ATOM	890	HN	VAL	P	59	-5.271	-14.536	-11.875	0.00	0.00	PROA
ATOM	891	CA	VAL	P	59	-4.249	-13.665	-13.518	0.00	0.00	PROA
ATOM	892	HA	VAL	P	59	-4.715	-13.147	-14.343	0.00	0.00	PROA
ATOM	893	CB	VAL	P	59	-3.756	-12.539	-12.561	0.00	0.00	PROA
ATOM	894	HB	VAL	P	59	-3.370	-13.045	-11.651	0.00	0.00	PROA
ATOM	895	CG1	VAL	P	59	-2.656	-11.564	-13.170	0.00	0.00	PROA
ATOM	896	HG11	VAL	P	59	-3.083	-11.057	-14.062	0.00	0.00	PROA
ATOM	897	HG12	VAL	P	59	-1.722	-11.902	-13.668	0.00	0.00	PROA
ATOM	898	HG13	VAL	P	59	-2.549	-10.702	-12.478	0.00	0.00	PROA
ATOM	899	CG2	VAL	P	59	-4.930	-11.635	-12.165	0.00	0.00	PROA
ATOM	900	HG21	VAL	P	59	-4.606	-10.928	-11.372	0.00	0.00	PROA
ATOM	901	HG22	VAL	P	59	-5.773	-12.255	-11.790	0.00	0.00	PROA
ATOM	902	HG23	VAL	P	59	-5.204	-11.021	-13.049	0.00	0.00	PROA
ATOM	903	C	VAL	P	59	-3.085	-14.241	-14.214	0.00	0.00	PROA
ATOM	904	O	VAL	P	59	-2.852	-13.943	-15.367	0.00	0.00	PROA
ATOM	905	N	ILE	P	60	-2.292	-15.170	-13.591	0.00	0.00	PROA
ATOM	906	HN	ILE	P	60	-2.342	-15.357	-12.613	0.00	0.00	PROA
ATOM	907	CA	ILE	P	60	-1.139	-15.807	-14.120	0.00	0.00	PROA
ATOM	908	HA	ILE	P	60	-0.438	-15.059	-14.461	0.00	0.00	PROA
ATOM	909	CB	ILE	P	60	-0.309	-16.537	-13.102	0.00	0.00	PROA
ATOM	910	HB	ILE	P	60	-1.067	-17.130	-12.546	0.00	0.00	PROA
ATOM	911	CG2	ILE	P	60	0.693	-17.493	-13.826	0.00	0.00	PROA

```
ATOM    912 HG21 ILE P  60       1.253 -16.902 -14.582  0.00  0.00      PROA
ATOM    913 HG22 ILE P  60       0.251 -18.377 -14.333  0.00  0.00      PROA
ATOM    914 HG23 ILE P  60       1.411 -17.882 -13.073  0.00  0.00      PROA
ATOM    915  CG1 ILE P  60       0.459 -15.585 -12.154  0.00  0.00      PROA
ATOM    916 HG11 ILE P  60       0.007 -14.576 -12.045  0.00  0.00      PROA
ATOM    917 HG12 ILE P  60       1.519 -15.448 -12.455  0.00  0.00      PROA
ATOM    918  CD  ILE P  60       0.585 -16.260 -10.741  0.00  0.00      PROA
ATOM    919  HD1 ILE P  60       1.112 -15.562 -10.055  0.00  0.00      PROA
ATOM    920  HD2 ILE P  60       1.141 -17.205 -10.922  0.00  0.00      PROA
ATOM    921  HD3 ILE P  60      -0.461 -16.467 -10.430  0.00  0.00      PROA
ATOM    922  C   ILE P  60      -1.694 -16.602 -15.354  0.00  0.00      PROA
ATOM    923  O   ILE P  60      -1.271 -16.486 -16.540  0.00  0.00      PROA
ATOM    924  N   LEU P  61      -2.802 -17.334 -15.158  0.00  0.00      PROA
ATOM    925  HN  LEU P  61      -3.207 -17.272 -14.249  0.00  0.00      PROA
ATOM    926  CA  LEU P  61      -3.320 -18.227 -16.171  0.00  0.00      PROA
ATOM    927  HA  LEU P  61      -2.689 -19.083 -16.361  0.00  0.00      PROA
ATOM    928  CB  LEU P  61      -4.578 -18.942 -15.680  0.00  0.00      PROA
ATOM    929  HB1 LEU P  61      -5.332 -18.238 -15.269  0.00  0.00      PROA
ATOM    930  HB2 LEU P  61      -4.173 -19.633 -14.910  0.00  0.00      PROA
ATOM    931  CG  LEU P  61      -5.337 -19.776 -16.711  0.00  0.00      PROA
ATOM    932  HG  LEU P  61      -5.695 -19.145 -17.552  0.00  0.00      PROA
ATOM    933  CD1 LEU P  61      -4.570 -20.918 -17.315  0.00  0.00      PROA
ATOM    934 HD11 LEU P  61      -4.035 -21.530 -16.557  0.00  0.00      PROA
ATOM    935 HD12 LEU P  61      -3.817 -20.436 -17.975  0.00  0.00      PROA
ATOM    936 HD13 LEU P  61      -5.264 -21.557 -17.902  0.00  0.00      PROA
ATOM    937  CD2 LEU P  61      -6.538 -20.356 -16.037  0.00  0.00      PROA
ATOM    938 HD21 LEU P  61      -7.335 -19.583 -15.980  0.00  0.00      PROA
ATOM    939 HD22 LEU P  61      -6.353 -20.747 -15.014  0.00  0.00      PROA
ATOM    940 HD23 LEU P  61      -6.933 -21.178 -16.671  0.00  0.00      PROA
ATOM    941  C   LEU P  61      -3.645 -17.505 -17.454  0.00  0.00      PROA
ATOM    942  O   LEU P  61      -3.120 -17.833 -18.491  0.00  0.00      PROA
ATOM    943  N   VAL P  62      -4.543 -16.506 -17.387  0.00  0.00      PROA
ATOM    944  HN  VAL P  62      -4.858 -16.129 -16.520  0.00  0.00      PROA
ATOM    945  CA  VAL P  62      -4.974 -15.823 -18.572  0.00  0.00      PROA
ATOM    946  HA  VAL P  62      -5.296 -16.580 -19.271  0.00  0.00      PROA
ATOM    947  CB  VAL P  62      -6.215 -14.866 -18.288  0.00  0.00      PROA
ATOM    948  HB  VAL P  62      -5.966 -14.282 -17.376  0.00  0.00      PROA
ATOM    949  CG1 VAL P  62      -6.337 -13.947 -19.490  0.00  0.00      PROA
ATOM    950 HG11 VAL P  62      -7.320 -13.430 -19.457  0.00  0.00      PROA
ATOM    951 HG12 VAL P  62      -6.345 -14.587 -20.398  0.00  0.00      PROA
ATOM    952 HG13 VAL P  62      -5.539 -13.195 -19.668  0.00  0.00      PROA
ATOM    953  CG2 VAL P  62      -7.503 -15.757 -18.100  0.00  0.00      PROA
ATOM    954 HG21 VAL P  62      -7.382 -16.281 -17.128  0.00  0.00      PROA
ATOM    955 HG22 VAL P  62      -7.645 -16.568 -18.846  0.00  0.00      PROA
ATOM    956 HG23 VAL P  62      -8.328 -15.028 -17.947  0.00  0.00      PROA
ATOM    957  C   VAL P  62      -3.831 -15.162 -19.318  0.00  0.00      PROA
ATOM    958  O   VAL P  62      -3.706 -15.256 -20.540  0.00  0.00      PROA
ATOM    959  N   MET P  63      -2.876 -14.515 -18.556  0.00  0.00      PROA
ATOM    960  HN  MET P  63      -2.815 -14.619 -17.566  0.00  0.00      PROA
ATOM    961  CA  MET P  63      -1.926 -13.557 -19.101  0.00  0.00      PROA
ATOM    962  HA  MET P  63      -2.426 -13.031 -19.901  0.00  0.00      PROA
ATOM    963  CB  MET P  63      -1.327 -12.540 -18.057  0.00  0.00      PROA
ATOM    964  HB1 MET P  63      -0.485 -11.958 -18.489  0.00  0.00      PROA
ATOM    965  HB2 MET P  63      -0.947 -13.210 -17.257  0.00  0.00      PROA
ATOM    966  CG  MET P  63      -2.432 -11.662 -17.463  0.00  0.00      PROA
ATOM    967  HG1 MET P  63      -1.987 -10.954 -16.732  0.00  0.00      PROA
ATOM    968  HG2 MET P  63      -3.138 -12.380 -16.992  0.00  0.00      PROA
ATOM    969  SD  MET P  63      -3.287 -10.642 -18.718  0.00  0.00      PROA
ATOM    970  CE  MET P  63      -4.763 -10.657 -17.600  0.00  0.00      PROA
ATOM    971  HE1 MET P  63      -4.446 -10.267 -16.609  0.00  0.00      PROA
ATOM    972  HE2 MET P  63      -5.184 -11.674 -17.447  0.00  0.00      PROA
```

```
ATOM    973  HE3 MET P  63      -5.542 -10.034 -18.090  0.00  0.00      PROA
ATOM    974  C   MET P  63      -0.675 -14.335 -19.715  0.00  0.00      PROA
ATOM    975  O   MET P  63      -0.060 -13.872 -20.688  0.00  0.00      PROA
ATOM    976  N   GLY P  64      -0.481 -15.600 -19.249  0.00  0.00      PROA
ATOM    977  HN  GLY P  64      -0.908 -15.867 -18.388  0.00  0.00      PROA
ATOM    978  CA  GLY P  64       0.649 -16.462 -19.621  0.00  0.00      PROA
ATOM    979  HA1 GLY P  64       1.053 -17.044 -18.806  0.00  0.00      PROA
ATOM    980  HA2 GLY P  64       1.477 -15.874 -19.987  0.00  0.00      PROA
ATOM    981  C   GLY P  64       0.262 -17.548 -20.546  0.00  0.00      PROA
ATOM    982  O   GLY P  64       1.092 -17.889 -21.397  0.00  0.00      PROA
ATOM    983  N   TYR P  65      -0.972 -18.091 -20.465  0.00  0.00      PROA
ATOM    984  HN  TYR P  65      -1.545 -17.841 -19.688  0.00  0.00      PROA
ATOM    985  CA  TYR P  65      -1.391 -19.269 -21.215  0.00  0.00      PROA
ATOM    986  HA  TYR P  65      -0.482 -19.705 -21.601  0.00  0.00      PROA
ATOM    987  CB  TYR P  65      -2.228 -20.351 -20.385  0.00  0.00      PROA
ATOM    988  HB1 TYR P  65      -2.693 -21.058 -21.105  0.00  0.00      PROA
ATOM    989  HB2 TYR P  65      -3.055 -19.827 -19.861  0.00  0.00      PROA
ATOM    990  CG  TYR P  65      -1.450 -21.150 -19.391  0.00  0.00      PROA
ATOM    991  CD1 TYR P  65      -1.421 -22.539 -19.559  0.00  0.00      PROA
ATOM    992  HD1 TYR P  65      -2.008 -23.022 -20.327  0.00  0.00      PROA
ATOM    993  CE1 TYR P  65      -0.696 -23.317 -18.620  0.00  0.00      PROA
ATOM    994  HE1 TYR P  65      -0.610 -24.391 -18.690  0.00  0.00      PROA
ATOM    995  CZ  TYR P  65      -0.136 -22.717 -17.463  0.00  0.00      PROA
ATOM    996  OH  TYR P  65       0.613 -23.519 -16.570  0.00  0.00      PROA
ATOM    997  HH  TYR P  65       0.790 -23.068 -15.741  0.00  0.00      PROA
ATOM    998  CD2 TYR P  65      -0.845 -20.503 -18.294  0.00  0.00      PROA
ATOM    999  HD2 TYR P  65      -0.896 -19.431 -18.173  0.00  0.00      PROA
ATOM   1000  CE2 TYR P  65      -0.114 -21.336 -17.398  0.00  0.00      PROA
ATOM   1001  HE2 TYR P  65       0.363 -20.850 -16.560  0.00  0.00      PROA
ATOM   1002  C   TYR P  65      -2.226 -18.837 -22.367  0.00  0.00      PROA
ATOM   1003  O   TYR P  65      -1.935 -19.031 -23.564  0.00  0.00      PROA
ATOM   1004  N   GLN P  66      -3.399 -18.193 -22.070  0.00  0.00      PROA
ATOM   1005  HN  GLN P  66      -3.408 -17.901 -21.117  0.00  0.00      PROA
ATOM   1006  CA  GLN P  66      -4.457 -17.886 -22.913  0.00  0.00      PROA
ATOM   1007  HA  GLN P  66      -4.564 -18.755 -23.546  0.00  0.00      PROA
ATOM   1008  CB  GLN P  66      -5.694 -17.638 -21.963  0.00  0.00      PROA
ATOM   1009  HB1 GLN P  66      -6.571 -17.552 -22.640  0.00  0.00      PROA
ATOM   1010  HB2 GLN P  66      -5.554 -16.653 -21.469  0.00  0.00      PROA
ATOM   1011  CG  GLN P  66      -5.896 -18.696 -20.953  0.00  0.00      PROA
ATOM   1012  HG1 GLN P  66      -6.849 -18.435 -20.446  0.00  0.00      PROA
ATOM   1013  HG2 GLN P  66      -5.192 -18.689 -20.093  0.00  0.00      PROA
ATOM   1014  CD  GLN P  66      -6.055 -20.126 -21.473  0.00  0.00      PROA
ATOM   1015  OE1 GLN P  66      -5.424 -21.027 -20.888  0.00  0.00      PROA
ATOM   1016  NE2 GLN P  66      -7.036 -20.275 -22.379  0.00  0.00      PROA
ATOM   1017 HE21 GLN P  66      -7.202 -21.200 -22.721  0.00  0.00      PROA
ATOM   1018 HE22 GLN P  66      -7.464 -19.442 -22.732  0.00  0.00      PROA
ATOM   1019  C   GLN P  66      -4.190 -16.699 -23.873  0.00  0.00      PROA
ATOM   1020  O   GLN P  66      -4.706 -16.617 -24.977  0.00  0.00      PROA
ATOM   1021  N   LYS P  67      -3.211 -15.867 -23.558  0.00  0.00      PROA
ATOM   1022  HN  LYS P  67      -2.855 -15.943 -22.630  0.00  0.00      PROA
ATOM   1023  CA  LYS P  67      -2.631 -14.984 -24.491  0.00  0.00      PROA
ATOM   1024  HA  LYS P  67      -3.236 -15.002 -25.386  0.00  0.00      PROA
ATOM   1025  CB  LYS P  67      -2.444 -13.530 -23.876  0.00  0.00      PROA
ATOM   1026  HB1 LYS P  67      -2.226 -12.891 -24.758  0.00  0.00      PROA
ATOM   1027  HB2 LYS P  67      -1.620 -13.334 -23.157  0.00  0.00      PROA
ATOM   1028  CG  LYS P  67      -3.770 -12.963 -23.214  0.00  0.00      PROA
ATOM   1029  HG1 LYS P  67      -4.009 -13.537 -22.293  0.00  0.00      PROA
ATOM   1030  HG2 LYS P  67      -4.654 -13.115 -23.870  0.00  0.00      PROA
ATOM   1031  CD  LYS P  67      -3.649 -11.523 -22.656  0.00  0.00      PROA
ATOM   1032  HD1 LYS P  67      -3.418 -10.798 -23.465  0.00  0.00      PROA
ATOM   1033  HD2 LYS P  67      -2.785 -11.489 -21.959  0.00  0.00      PROA
```

```
ATOM   1034  CE  LYS P  67      -4.901 -11.010 -21.949  0.00  0.00      PROA
ATOM   1035  HE1 LYS P  67      -4.958 -11.577 -20.996  0.00  0.00      PROA
ATOM   1036  HE2 LYS P  67      -5.798 -11.032 -22.604  0.00  0.00      PROA
ATOM   1037  NZ  LYS P  67      -4.724  -9.537 -21.607  0.00  0.00      PROA
ATOM   1038  HZ1 LYS P  67      -5.230  -9.260 -20.741  0.00  0.00      PROA
ATOM   1039  HZ2 LYS P  67      -5.033  -8.971 -22.423  0.00  0.00      PROA
ATOM   1040  HZ3 LYS P  67      -3.716  -9.378 -21.406  0.00  0.00      PROA
ATOM   1041  C   LYS P  67      -1.308 -15.449 -25.057  0.00  0.00      PROA
ATOM   1042  O   LYS P  67      -0.230 -15.495 -24.421  0.00  0.00      PROA
ATOM   1043  N   LYS P  68      -1.308 -15.883 -26.330  0.00  0.00      PROA
ATOM   1044  HN  LYS P  68      -2.050 -15.858 -26.995  0.00  0.00      PROA
ATOM   1045  CA  LYS P  68      -0.087 -16.458 -26.922  0.00  0.00      PROA
ATOM   1046  HA  LYS P  68       0.214 -17.102 -26.110  0.00  0.00      PROA
ATOM   1047  CB  LYS P  68      -0.472 -17.263 -28.264  0.00  0.00      PROA
ATOM   1048  HB1 LYS P  68       0.426 -17.878 -28.485  0.00  0.00      PROA
ATOM   1049  HB2 LYS P  68      -0.581 -16.504 -29.068  0.00  0.00      PROA
ATOM   1050  CG  LYS P  68      -1.782 -18.028 -28.091  0.00  0.00      PROA
ATOM   1051  HG1 LYS P  68      -2.027 -18.474 -29.079  0.00  0.00      PROA
ATOM   1052  HG2 LYS P  68      -2.628 -17.400 -27.739  0.00  0.00      PROA
ATOM   1053  CD  LYS P  68      -1.683 -19.184 -27.088  0.00  0.00      PROA
ATOM   1054  HD1 LYS P  68      -1.233 -18.756 -26.167  0.00  0.00      PROA
ATOM   1055  HD2 LYS P  68      -1.027 -19.927 -27.591  0.00  0.00      PROA
ATOM   1056  CE  LYS P  68      -3.104 -19.688 -26.702  0.00  0.00      PROA
ATOM   1057  HE1 LYS P  68      -3.577 -20.243 -27.541  0.00  0.00      PROA
ATOM   1058  HE2 LYS P  68      -3.781 -18.864 -26.391  0.00  0.00      PROA
ATOM   1059  NZ  LYS P  68      -3.047 -20.607 -25.596  0.00  0.00      PROA
ATOM   1060  HZ1 LYS P  68      -2.525 -20.320 -24.744  0.00  0.00      PROA
ATOM   1061  HZ2 LYS P  68      -2.442 -21.401 -25.887  0.00  0.00      PROA
ATOM   1062  HZ3 LYS P  68      -3.988 -20.947 -25.312  0.00  0.00      PROA
ATOM   1063  C   LYS P  68       1.027 -15.396 -27.249  0.00  0.00      PROA
ATOM   1064  O   LYS P  68       2.184 -15.725 -27.302  0.00  0.00      PROA
ATOM   1065  N   LEU P  69       0.665 -14.125 -27.432  0.00  0.00      PROA
ATOM   1066  HN  LEU P  69      -0.299 -13.890 -27.329  0.00  0.00      PROA
ATOM   1067  CA  LEU P  69       1.659 -13.089 -27.451  0.00  0.00      PROA
ATOM   1068  HA  LEU P  69       2.678 -13.401 -27.276  0.00  0.00      PROA
ATOM   1069  CB  LEU P  69       1.678 -12.161 -28.725  0.00  0.00      PROA
ATOM   1070  HB1 LEU P  69       2.117 -11.154 -28.563  0.00  0.00      PROA
ATOM   1071  HB2 LEU P  69       0.663 -12.015 -29.151  0.00  0.00      PROA
ATOM   1072  CG  LEU P  69       2.687 -12.731 -29.833  0.00  0.00      PROA
ATOM   1073  HG  LEU P  69       2.460 -13.810 -29.963  0.00  0.00      PROA
ATOM   1074  CD1 LEU P  69       2.554 -11.978 -31.199  0.00  0.00      PROA
ATOM   1075 HD11 LEU P  69       3.031 -10.981 -31.088  0.00  0.00      PROA
ATOM   1076 HD12 LEU P  69       1.454 -11.976 -31.357  0.00  0.00      PROA
ATOM   1077 HD13 LEU P  69       3.095 -12.630 -31.918  0.00  0.00      PROA
ATOM   1078  CD2 LEU P  69       4.150 -12.569 -29.341  0.00  0.00      PROA
ATOM   1079 HD21 LEU P  69       4.225 -13.095 -28.365  0.00  0.00      PROA
ATOM   1080 HD22 LEU P  69       4.301 -11.514 -29.025  0.00  0.00      PROA
ATOM   1081 HD23 LEU P  69       4.937 -12.904 -30.050  0.00  0.00      PROA
ATOM   1082  C   LEU P  69       1.303 -12.237 -26.242  0.00  0.00      PROA
ATOM   1083  O   LEU P  69       0.127 -12.053 -25.916  0.00  0.00      PROA
ATOM   1084  N   ARG P  70       2.310 -11.812 -25.470  0.00  0.00      PROA
ATOM   1085  HN  ARG P  70       3.233 -12.072 -25.740  0.00  0.00      PROA
ATOM   1086  CA  ARG P  70       2.045 -10.907 -24.312  0.00  0.00      PROA
ATOM   1087  HA  ARG P  70       1.038 -10.957 -23.924  0.00  0.00      PROA
ATOM   1088  CB  ARG P  70       2.831 -11.290 -23.039  0.00  0.00      PROA
ATOM   1089  HB1 ARG P  70       2.831 -10.408 -22.363  0.00  0.00      PROA
ATOM   1090  HB2 ARG P  70       3.868 -11.543 -23.348  0.00  0.00      PROA
ATOM   1091  CG  ARG P  70       2.300 -12.544 -22.203  0.00  0.00      PROA
ATOM   1092  HG1 ARG P  70       1.273 -12.277 -21.873  0.00  0.00      PROA
ATOM   1093  HG2 ARG P  70       3.010 -12.678 -21.360  0.00  0.00      PROA
ATOM   1094  CD  ARG P  70       2.019 -13.881 -22.990  0.00  0.00      PROA
```

ATOM 1095 HD1 ARG P 70 1.246 -13.806 -23.785 0.00 0.00 PROA
ATOM 1096 HD2 ARG P 70 1.698 -14.562 -22.173 0.00 0.00 PROA
ATOM 1097 NE ARG P 70 3.333 -14.298 -23.594 0.00 0.00 PROA
ATOM 1098 HE ARG P 70 4.119 -13.685 -23.517 0.00 0.00 PROA
ATOM 1099 CZ ARG P 70 3.543 -15.418 -24.191 0.00 0.00 PROA
ATOM 1100 NH1 ARG P 70 2.576 -16.298 -24.405 0.00 0.00 PROA
ATOM 1101 HH11 ARG P 70 1.648 -16.027 -24.148 0.00 0.00 PROA
ATOM 1102 HH12 ARG P 70 2.708 -17.032 -25.072 0.00 0.00 PROA
ATOM 1103 NH2 ARG P 70 4.784 -15.584 -24.641 0.00 0.00 PROA
ATOM 1104 HH21 ARG P 70 5.354 -14.865 -24.242 0.00 0.00 PROA
ATOM 1105 HH22 ARG P 70 4.881 -15.777 -25.617 0.00 0.00 PROA
ATOM 1106 C ARG P 70 2.154 -9.483 -24.679 0.00 0.00 PROA
ATOM 1107 O ARG P 70 3.150 -9.035 -25.239 0.00 0.00 PROA
ATOM 1108 N SER P 71 1.088 -8.718 -24.301 0.00 0.00 PROA
ATOM 1109 HN SER P 71 0.375 -9.132 -23.740 0.00 0.00 PROA
ATOM 1110 CA SER P 71 1.259 -7.260 -24.409 0.00 0.00 PROA
ATOM 1111 HA SER P 71 1.720 -7.005 -25.352 0.00 0.00 PROA
ATOM 1112 CB SER P 71 -0.066 -6.456 -24.380 0.00 0.00 PROA
ATOM 1113 HB1 SER P 71 -0.753 -6.854 -25.157 0.00 0.00 PROA
ATOM 1114 HB2 SER P 71 0.163 -5.393 -24.607 0.00 0.00 PROA
ATOM 1115 OG SER P 71 -0.797 -6.484 -23.114 0.00 0.00 PROA
ATOM 1116 HG1 SER P 71 -0.889 -7.393 -22.819 0.00 0.00 PROA
ATOM 1117 C SER P 71 2.294 -6.745 -23.413 0.00 0.00 PROA
ATOM 1118 O SER P 71 2.691 -7.390 -22.470 0.00 0.00 PROA
ATOM 1119 N MET P 72 2.882 -5.545 -23.673 0.00 0.00 PROA
ATOM 1120 HN MET P 72 2.612 -5.067 -24.506 0.00 0.00 PROA
ATOM 1121 CA MET P 72 3.667 -4.830 -22.692 0.00 0.00 PROA
ATOM 1122 HA MET P 72 4.593 -5.367 -22.546 0.00 0.00 PROA
ATOM 1123 CB MET P 72 3.969 -3.474 -23.228 0.00 0.00 PROA
ATOM 1124 HB1 MET P 72 4.579 -2.896 -22.502 0.00 0.00 PROA
ATOM 1125 HB2 MET P 72 3.030 -2.949 -23.506 0.00 0.00 PROA
ATOM 1126 CG MET P 72 4.653 -3.535 -24.597 0.00 0.00 PROA
ATOM 1127 HG1 MET P 72 4.042 -3.963 -25.421 0.00 0.00 PROA
ATOM 1128 HG2 MET P 72 5.530 -4.204 -24.460 0.00 0.00 PROA
ATOM 1129 SD MET P 72 5.488 -1.873 -24.919 0.00 0.00 PROA
ATOM 1130 CE MET P 72 5.088 -1.916 -26.715 0.00 0.00 PROA
ATOM 1131 HE1 MET P 72 3.995 -1.872 -26.909 0.00 0.00 PROA
ATOM 1132 HE2 MET P 72 5.490 -2.816 -27.229 0.00 0.00 PROA
ATOM 1133 HE3 MET P 72 5.634 -1.085 -27.210 0.00 0.00 PROA
ATOM 1134 C MET P 72 2.947 -4.668 -21.371 0.00 0.00 PROA
ATOM 1135 O MET P 72 3.478 -4.750 -20.306 0.00 0.00 PROA
ATOM 1136 N THR P 73 1.597 -4.314 -21.401 0.00 0.00 PROA
ATOM 1137 HN THR P 73 1.195 -4.143 -22.297 0.00 0.00 PROA
ATOM 1138 CA THR P 73 0.686 -4.287 -20.274 0.00 0.00 PROA
ATOM 1139 HA THR P 73 1.164 -3.663 -19.534 0.00 0.00 PROA
ATOM 1140 CB THR P 73 -0.719 -3.667 -20.618 0.00 0.00 PROA
ATOM 1141 HB THR P 73 -1.348 -4.408 -21.158 0.00 0.00 PROA
ATOM 1142 OG1 THR P 73 -0.495 -2.570 -21.538 0.00 0.00 PROA
ATOM 1143 HG1 THR P 73 -1.311 -2.351 -21.994 0.00 0.00 PROA
ATOM 1144 CG2 THR P 73 -1.329 -3.176 -19.298 0.00 0.00 PROA
ATOM 1145 HG21 THR P 73 -1.668 -3.962 -18.589 0.00 0.00 PROA
ATOM 1146 HG22 THR P 73 -2.221 -2.540 -19.480 0.00 0.00 PROA
ATOM 1147 HG23 THR P 73 -0.570 -2.571 -18.758 0.00 0.00 PROA
ATOM 1148 C THR P 73 0.586 -5.557 -19.540 0.00 0.00 PROA
ATOM 1149 O THR P 73 0.644 -5.680 -18.318 0.00 0.00 PROA
ATOM 1150 N ASP P 74 0.538 -6.645 -20.350 0.00 0.00 PROA
ATOM 1151 HN ASP P 74 0.475 -6.553 -21.341 0.00 0.00 PROA
ATOM 1152 CA ASP P 74 0.418 -7.948 -19.802 0.00 0.00 PROA
ATOM 1153 HA ASP P 74 -0.434 -7.924 -19.138 0.00 0.00 PROA
ATOM 1154 CB ASP P 74 0.148 -9.194 -20.863 0.00 0.00 PROA
ATOM 1155 HB1 ASP P 74 -0.036 -10.128 -20.291 0.00 0.00 PROA

ATOM	1156	HB2	ASP	P	74	1.007	-9.369	-21.546	0.00	0.00	PROA
ATOM	1157	CG	ASP	P	74	-1.186	-8.969	-21.632	0.00	0.00	PROA
ATOM	1158	OD1	ASP	P	74	-1.240	-9.194	-22.891	0.00	0.00	PROA
ATOM	1159	OD2	ASP	P	74	-2.155	-8.539	-20.996	0.00	0.00	PROA
ATOM	1160	C	ASP	P	74	1.589	-8.368	-18.927	0.00	0.00	PROA
ATOM	1161	O	ASP	P	74	1.510	-8.937	-17.814	0.00	0.00	PROA
ATOM	1162	N	LYS	P	75	2.813	-7.972	-19.333	0.00	0.00	PROA
ATOM	1163	HN	LYS	P	75	2.895	-7.605	-20.257	0.00	0.00	PROA
ATOM	1164	CA	LYS	P	75	4.082	-7.996	-18.567	0.00	0.00	PROA
ATOM	1165	HA	LYS	P	75	4.099	-8.989	-18.142	0.00	0.00	PROA
ATOM	1166	CB	LYS	P	75	5.234	-7.606	-19.540	0.00	0.00	PROA
ATOM	1167	HB1	LYS	P	75	6.159	-7.349	-18.981	0.00	0.00	PROA
ATOM	1168	HB2	LYS	P	75	4.973	-6.632	-20.007	0.00	0.00	PROA
ATOM	1169	CG	LYS	P	75	5.519	-8.576	-20.689	0.00	0.00	PROA
ATOM	1170	HG1	LYS	P	75	6.190	-8.004	-21.366	0.00	0.00	PROA
ATOM	1171	HG2	LYS	P	75	4.585	-8.723	-21.273	0.00	0.00	PROA
ATOM	1172	CD	LYS	P	75	6.160	-9.950	-20.315	0.00	0.00	PROA
ATOM	1173	HD1	LYS	P	75	6.127	-10.449	-21.307	0.00	0.00	PROA
ATOM	1174	HD2	LYS	P	75	5.466	-10.585	-19.723	0.00	0.00	PROA
ATOM	1175	CE	LYS	P	75	7.505	-9.942	-19.610	0.00	0.00	PROA
ATOM	1176	HE1	LYS	P	75	7.536	-9.472	-18.604	0.00	0.00	PROA
ATOM	1177	HE2	LYS	P	75	8.144	-9.329	-20.280	0.00	0.00	PROA
ATOM	1178	NZ	LYS	P	75	7.964	-11.329	-19.446	0.00	0.00	PROA
ATOM	1179	HZ1	LYS	P	75	8.920	-11.422	-19.048	0.00	0.00	PROA
ATOM	1180	HZ2	LYS	P	75	7.975	-11.754	-20.395	0.00	0.00	PROA
ATOM	1181	HZ3	LYS	P	75	7.313	-11.955	-18.931	0.00	0.00	PROA
ATOM	1182	C	LYS	P	75	4.043	-7.128	-17.363	0.00	0.00	PROA
ATOM	1183	O	LYS	P	75	4.448	-7.611	-16.296	0.00	0.00	PROA
ATOM	1184	N	TYR	P	76	3.547	-5.873	-17.396	0.00	0.00	PROA
ATOM	1185	HN	TYR	P	76	3.440	-5.408	-18.271	0.00	0.00	PROA
ATOM	1186	CA	TYR	P	76	3.422	-4.965	-16.183	0.00	0.00	PROA
ATOM	1187	HA	TYR	P	76	4.423	-4.771	-15.827	0.00	0.00	PROA
ATOM	1188	CB	TYR	P	76	2.795	-3.566	-16.536	0.00	0.00	PROA
ATOM	1189	HB1	TYR	P	76	2.963	-2.882	-15.677	0.00	0.00	PROA
ATOM	1190	HB2	TYR	P	76	1.735	-3.794	-16.780	0.00	0.00	PROA
ATOM	1191	CG	TYR	P	76	3.493	-2.636	-17.597	0.00	0.00	PROA
ATOM	1192	CD1	TYR	P	76	4.759	-2.777	-18.141	0.00	0.00	PROA
ATOM	1193	HD1	TYR	P	76	5.399	-3.429	-17.565	0.00	0.00	PROA
ATOM	1194	CE1	TYR	P	76	5.189	-2.074	-19.310	0.00	0.00	PROA
ATOM	1195	HE1	TYR	P	76	6.176	-2.200	-19.730	0.00	0.00	PROA
ATOM	1196	CZ	TYR	P	76	4.267	-1.224	-19.878	0.00	0.00	PROA
ATOM	1197	OH	TYR	P	76	4.589	-0.511	-21.039	0.00	0.00	PROA
ATOM	1198	HH	TYR	P	76	3.857	0.041	-21.325	0.00	0.00	PROA
ATOM	1199	CD2	TYR	P	76	2.613	-1.699	-18.113	0.00	0.00	PROA
ATOM	1200	HD2	TYR	P	76	1.697	-1.607	-17.547	0.00	0.00	PROA
ATOM	1201	CE2	TYR	P	76	2.952	-1.017	-19.261	0.00	0.00	PROA
ATOM	1202	HE2	TYR	P	76	2.120	-0.437	-19.629	0.00	0.00	PROA
ATOM	1203	C	TYR	P	76	2.503	-5.674	-15.159	0.00	0.00	PROA
ATOM	1204	O	TYR	P	76	2.702	-5.650	-13.928	0.00	0.00	PROA
ATOM	1205	N	ARG	P	77	1.337	-6.297	-15.518	0.00	0.00	PROA
ATOM	1206	HN	ARG	P	77	1.072	-6.211	-16.476	0.00	0.00	PROA
ATOM	1207	CA	ARG	P	77	0.571	-7.147	-14.619	0.00	0.00	PROA
ATOM	1208	HA	ARG	P	77	0.259	-6.568	-13.763	0.00	0.00	PROA
ATOM	1209	CB	ARG	P	77	-0.885	-7.462	-15.258	0.00	0.00	PROA
ATOM	1210	HB1	ARG	P	77	-0.573	-7.929	-16.216	0.00	0.00	PROA
ATOM	1211	HB2	ARG	P	77	-1.329	-6.463	-15.452	0.00	0.00	PROA
ATOM	1212	CG	ARG	P	77	-1.816	-8.327	-14.480	0.00	0.00	PROA
ATOM	1213	HG1	ARG	P	77	-1.368	-9.292	-14.163	0.00	0.00	PROA
ATOM	1214	HG2	ARG	P	77	-2.670	-8.489	-15.173	0.00	0.00	PROA
ATOM	1215	CD	ARG	P	77	-2.384	-7.703	-13.210	0.00	0.00	PROA
ATOM	1216	HD1	ARG	P	77	-3.319	-8.188	-12.856	0.00	0.00	PROA

```
ATOM   1217  HD2 ARG P  77      -2.631  -6.636 -13.395  0.00  0.00      PROA
ATOM   1218  NE  ARG P  77      -1.417  -7.948 -12.063  0.00  0.00      PROA
ATOM   1219  HE  ARG P  77      -0.685  -8.565 -12.350  0.00  0.00      PROA
ATOM   1220  CZ  ARG P  77      -1.400  -7.497 -10.825  0.00  0.00      PROA
ATOM   1221  NH1 ARG P  77      -2.281  -6.667 -10.345  0.00  0.00      PROA
ATOM   1222 HH11 ARG P  77      -2.916  -6.356 -11.052  0.00  0.00      PROA
ATOM   1223 HH12 ARG P  77      -2.020  -6.205  -9.497  0.00  0.00      PROA
ATOM   1224  NH2 ARG P  77      -0.424  -7.986 -10.128  0.00  0.00      PROA
ATOM   1225 HH21 ARG P  77       0.011  -8.849 -10.386  0.00  0.00      PROA
ATOM   1226 HH22 ARG P  77      -0.535  -7.829  -9.146  0.00  0.00      PROA
ATOM   1227  C   ARG P  77       1.250  -8.432 -14.040  0.00  0.00      PROA
ATOM   1228  O   ARG P  77       1.125  -8.655 -12.863  0.00  0.00      PROA
ATOM   1229  N   LEU P  78       1.906  -9.259 -14.866  0.00  0.00      PROA
ATOM   1230  HN  LEU P  78       1.835  -8.941 -15.808  0.00  0.00      PROA
ATOM   1231  CA  LEU P  78       2.670 -10.457 -14.505  0.00  0.00      PROA
ATOM   1232  HA  LEU P  78       2.042 -11.096 -13.903  0.00  0.00      PROA
ATOM   1233  CB  LEU P  78       3.184 -11.224 -15.787  0.00  0.00      PROA
ATOM   1234  HB1 LEU P  78       3.966 -11.963 -15.512  0.00  0.00      PROA
ATOM   1235  HB2 LEU P  78       3.765 -10.536 -16.438  0.00  0.00      PROA
ATOM   1236  CG  LEU P  78       2.075 -12.036 -16.579  0.00  0.00      PROA
ATOM   1237  HG  LEU P  78       1.163 -11.460 -16.844  0.00  0.00      PROA
ATOM   1238  CD1 LEU P  78       2.758 -12.582 -17.860  0.00  0.00      PROA
ATOM   1239 HD11 LEU P  78       3.657 -13.227 -17.760  0.00  0.00      PROA
ATOM   1240 HD12 LEU P  78       2.884 -11.750 -18.585  0.00  0.00      PROA
ATOM   1241 HD13 LEU P  78       2.021 -13.258 -18.343  0.00  0.00      PROA
ATOM   1242  CD2 LEU P  78       1.549 -13.184 -15.675  0.00  0.00      PROA
ATOM   1243 HD21 LEU P  78       0.759 -13.761 -16.203  0.00  0.00      PROA
ATOM   1244 HD22 LEU P  78       1.105 -12.684 -14.789  0.00  0.00      PROA
ATOM   1245 HD23 LEU P  78       2.410 -13.760 -15.273  0.00  0.00      PROA
ATOM   1246  C   LEU P  78       3.863 -10.219 -13.526  0.00  0.00      PROA
ATOM   1247  O   LEU P  78       4.177 -10.983 -12.638  0.00  0.00      PROA
ATOM   1248  N   HSE P  79       4.497  -9.022 -13.655  0.00  0.00      PROA
ATOM   1249  HN  HSE P  79       4.309  -8.406 -14.416  0.00  0.00      PROA
ATOM   1250  CA  HSE P  79       5.561  -8.656 -12.756  0.00  0.00      PROA
ATOM   1251  HA  HSE P  79       6.297  -9.437 -12.639  0.00  0.00      PROA
ATOM   1252  CB  HSE P  79       6.255  -7.327 -13.245  0.00  0.00      PROA
ATOM   1253  HB1 HSE P  79       7.016  -6.973 -12.517  0.00  0.00      PROA
ATOM   1254  HB2 HSE P  79       5.537  -6.487 -13.364  0.00  0.00      PROA
ATOM   1255  ND1 HSE P  79       7.920  -8.467 -14.673  0.00  0.00      PROA
ATOM   1256  CG  HSE P  79       7.005  -7.431 -14.557  0.00  0.00      PROA
ATOM   1257  CE1 HSE P  79       8.442  -8.301 -15.900  0.00  0.00      PROA
ATOM   1258  HE1 HSE P  79       9.066  -8.989 -16.471  0.00  0.00      PROA
ATOM   1259  NE2 HSE P  79       7.879  -7.250 -16.541  0.00  0.00      PROA
ATOM   1260  HE2 HSE P  79       8.006  -6.819 -17.434  0.00  0.00      PROA
ATOM   1261  CD2 HSE P  79       6.964  -6.690 -15.724  0.00  0.00      PROA
ATOM   1262  HD2 HSE P  79       6.391  -5.779 -15.848  0.00  0.00      PROA
ATOM   1263  C   HSE P  79       5.155  -8.420 -11.310  0.00  0.00      PROA
ATOM   1264  O   HSE P  79       5.804  -8.871 -10.339  0.00  0.00      PROA
ATOM   1265  N   LEU P  80       4.036  -7.650 -11.123  0.00  0.00      PROA
ATOM   1266  HN  LEU P  80       3.612  -7.192 -11.900  0.00  0.00      PROA
ATOM   1267  CA  LEU P  80       3.516  -7.369  -9.870  0.00  0.00      PROA
ATOM   1268  HA  LEU P  80       4.423  -7.262  -9.293  0.00  0.00      PROA
ATOM   1269  CB  LEU P  80       2.626  -6.125  -9.853  0.00  0.00      PROA
ATOM   1270  HB1 LEU P  80       1.694  -6.330 -10.422  0.00  0.00      PROA
ATOM   1271  HB2 LEU P  80       3.025  -5.279 -10.452  0.00  0.00      PROA
ATOM   1272  CG  LEU P  80       2.189  -5.521  -8.464  0.00  0.00      PROA
ATOM   1273  HG  LEU P  80       1.760  -6.432  -7.995  0.00  0.00      PROA
ATOM   1274  CD1 LEU P  80       3.370  -4.834  -7.747  0.00  0.00      PROA
ATOM   1275 HD11 LEU P  80       4.027  -5.625  -7.327  0.00  0.00      PROA
ATOM   1276 HD12 LEU P  80       3.017  -4.249  -6.870  0.00  0.00      PROA
ATOM   1277 HD13 LEU P  80       3.818  -4.188  -8.532  0.00  0.00      PROA
```

ATOM 1278 CD2 LEU P 80 0.987 -4.531 -8.645 0.00 0.00 PROA
ATOM 1279 HD21 LEU P 80 0.365 -5.015 -9.428 0.00 0.00 PROA
ATOM 1280 HD22 LEU P 80 1.137 -3.499 -9.027 0.00 0.00 PROA
ATOM 1281 HD23 LEU P 80 0.451 -4.315 -7.696 0.00 0.00 PROA
ATOM 1282 C LEU P 80 2.677 -8.545 -9.351 0.00 0.00 PROA
ATOM 1283 O LEU P 80 2.444 -8.606 -8.188 0.00 0.00 PROA
ATOM 1284 N SER P 81 2.260 -9.504 -10.199 0.00 0.00 PROA
ATOM 1285 HN SER P 81 2.459 -9.351 -11.164 0.00 0.00 PROA
ATOM 1286 CA SER P 81 1.692 -10.744 -9.735 0.00 0.00 PROA
ATOM 1287 HA SER P 81 0.977 -10.532 -8.955 0.00 0.00 PROA
ATOM 1288 CB SER P 81 0.885 -11.532 -10.809 0.00 0.00 PROA
ATOM 1289 HB1 SER P 81 0.459 -12.477 -10.410 0.00 0.00 PROA
ATOM 1290 HB2 SER P 81 1.544 -11.776 -11.669 0.00 0.00 PROA
ATOM 1291 OG SER P 81 -0.279 -10.667 -11.176 0.00 0.00 PROA
ATOM 1292 HG1 SER P 81 -1.025 -10.922 -10.628 0.00 0.00 PROA
ATOM 1293 C SER P 81 2.681 -11.694 -9.047 0.00 0.00 PROA
ATOM 1294 O SER P 81 2.170 -12.479 -8.254 0.00 0.00 PROA
ATOM 1295 N VAL P 82 4.011 -11.658 -9.170 0.00 0.00 PROA
ATOM 1296 HN VAL P 82 4.324 -11.244 -10.021 0.00 0.00 PROA
ATOM 1297 CA VAL P 82 4.931 -12.200 -8.171 0.00 0.00 PROA
ATOM 1298 HA VAL P 82 4.713 -13.244 -8.000 0.00 0.00 PROA
ATOM 1299 CB VAL P 82 6.436 -12.156 -8.651 0.00 0.00 PROA
ATOM 1300 HB VAL P 82 6.594 -11.081 -8.885 0.00 0.00 PROA
ATOM 1301 CG1 VAL P 82 7.435 -12.698 -7.713 0.00 0.00 PROA
ATOM 1302 HG11 VAL P 82 7.713 -12.032 -6.868 0.00 0.00 PROA
ATOM 1303 HG12 VAL P 82 8.307 -13.149 -8.233 0.00 0.00 PROA
ATOM 1304 HG13 VAL P 82 6.869 -13.536 -7.252 0.00 0.00 PROA
ATOM 1305 CG2 VAL P 82 6.554 -12.871 -10.092 0.00 0.00 PROA
ATOM 1306 HG21 VAL P 82 7.620 -13.076 -10.330 0.00 0.00 PROA
ATOM 1307 HG22 VAL P 82 6.101 -12.372 -10.975 0.00 0.00 PROA
ATOM 1308 HG23 VAL P 82 6.054 -13.857 -9.988 0.00 0.00 PROA
ATOM 1309 C VAL P 82 4.781 -11.601 -6.848 0.00 0.00 PROA
ATOM 1310 O VAL P 82 4.628 -12.303 -5.832 0.00 0.00 PROA
ATOM 1311 N ALA P 83 4.770 -10.309 -6.780 0.00 0.00 PROA
ATOM 1312 HN ALA P 83 4.699 -9.784 -7.625 0.00 0.00 PROA
ATOM 1313 CA ALA P 83 4.634 -9.529 -5.551 0.00 0.00 PROA
ATOM 1314 HA ALA P 83 5.474 -9.937 -5.009 0.00 0.00 PROA
ATOM 1315 CB ALA P 83 5.027 -8.026 -5.852 0.00 0.00 PROA
ATOM 1316 HB1 ALA P 83 4.798 -7.570 -4.865 0.00 0.00 PROA
ATOM 1317 HB2 ALA P 83 4.422 -7.620 -6.691 0.00 0.00 PROA
ATOM 1318 HB3 ALA P 83 6.104 -7.973 -6.117 0.00 0.00 PROA
ATOM 1319 C ALA P 83 3.321 -9.698 -4.668 0.00 0.00 PROA
ATOM 1320 O ALA P 83 3.339 -9.875 -3.452 0.00 0.00 PROA
ATOM 1321 N ASP P 84 2.169 -9.760 -5.356 0.00 0.00 PROA
ATOM 1322 HN ASP P 84 2.258 -9.525 -6.321 0.00 0.00 PROA
ATOM 1323 CA ASP P 84 0.858 -10.112 -4.831 0.00 0.00 PROA
ATOM 1324 HA ASP P 84 0.544 -9.505 -3.995 0.00 0.00 PROA
ATOM 1325 CB ASP P 84 -0.222 -9.778 -5.925 0.00 0.00 PROA
ATOM 1326 HB1 ASP P 84 -1.224 -9.958 -5.480 0.00 0.00 PROA
ATOM 1327 HB2 ASP P 84 -0.164 -10.360 -6.870 0.00 0.00 PROA
ATOM 1328 CG ASP P 84 -0.373 -8.273 -6.282 0.00 0.00 PROA
ATOM 1329 OD1 ASP P 84 0.038 -7.386 -5.459 0.00 0.00 PROA
ATOM 1330 OD2 ASP P 84 -1.113 -7.998 -7.256 0.00 0.00 PROA
ATOM 1331 C ASP P 84 0.761 -11.525 -4.346 0.00 0.00 PROA
ATOM 1332 O ASP P 84 0.236 -11.840 -3.332 0.00 0.00 PROA
ATOM 1333 N LEU P 85 1.384 -12.471 -5.036 0.00 0.00 PROA
ATOM 1334 HN LEU P 85 1.796 -12.284 -5.924 0.00 0.00 PROA
ATOM 1335 CA LEU P 85 1.529 -13.793 -4.554 0.00 0.00 PROA
ATOM 1336 HA LEU P 85 0.586 -14.154 -4.170 0.00 0.00 PROA
ATOM 1337 CB LEU P 85 1.971 -14.623 -5.825 0.00 0.00 PROA
ATOM 1338 HB1 LEU P 85 3.007 -14.312 -6.078 0.00 0.00 PROA

ATOM 1339 HB2 LEU P 85 1.347 -14.385 -6.713 0.00 0.00 PROA
ATOM 1340 CG LEU P 85 2.085 -16.127 -5.701 0.00 0.00 PROA
ATOM 1341 HG LEU P 85 3.006 -16.494 -5.199 0.00 0.00 PROA
ATOM 1342 CD1 LEU P 85 0.827 -16.647 -4.907 0.00 0.00 PROA
ATOM 1343 HD11 LEU P 85 0.772 -17.738 -5.111 0.00 0.00 PROA
ATOM 1344 HD12 LEU P 85 -0.087 -16.173 -5.324 0.00 0.00 PROA
ATOM 1345 HD13 LEU P 85 1.044 -16.428 -3.840 0.00 0.00 PROA
ATOM 1346 CD2 LEU P 85 1.873 -16.813 -7.032 0.00 0.00 PROA
ATOM 1347 HD21 LEU P 85 0.810 -16.620 -7.291 0.00 0.00 PROA
ATOM 1348 HD22 LEU P 85 1.985 -17.906 -6.865 0.00 0.00 PROA
ATOM 1349 HD23 LEU P 85 2.579 -16.360 -7.761 0.00 0.00 PROA
ATOM 1350 C LEU P 85 2.398 -14.062 -3.279 0.00 0.00 PROA
ATOM 1351 O LEU P 85 2.014 -14.733 -2.346 0.00 0.00 PROA
ATOM 1352 N LEU P 86 3.625 -13.417 -3.196 0.00 0.00 PROA
ATOM 1353 HN LEU P 86 3.928 -12.828 -3.941 0.00 0.00 PROA
ATOM 1354 CA LEU P 86 4.408 -13.274 -1.937 0.00 0.00 PROA
ATOM 1355 HA LEU P 86 4.701 -14.241 -1.554 0.00 0.00 PROA
ATOM 1356 CB LEU P 86 5.831 -12.688 -2.080 0.00 0.00 PROA
ATOM 1357 HB1 LEU P 86 6.267 -12.740 -1.059 0.00 0.00 PROA
ATOM 1358 HB2 LEU P 86 5.739 -11.665 -2.503 0.00 0.00 PROA
ATOM 1359 CG LEU P 86 6.838 -13.468 -2.946 0.00 0.00 PROA
ATOM 1360 HG LEU P 86 6.619 -13.247 -4.013 0.00 0.00 PROA
ATOM 1361 CD1 LEU P 86 8.186 -12.866 -2.710 0.00 0.00 PROA
ATOM 1362 HD11 LEU P 86 9.008 -13.145 -3.404 0.00 0.00 PROA
ATOM 1363 HD12 LEU P 86 8.434 -12.869 -1.627 0.00 0.00 PROA
ATOM 1364 HD13 LEU P 86 8.158 -11.760 -2.811 0.00 0.00 PROA
ATOM 1365 CD2 LEU P 86 6.876 -14.955 -2.640 0.00 0.00 PROA
ATOM 1366 HD21 LEU P 86 7.012 -15.225 -1.571 0.00 0.00 PROA
ATOM 1367 HD22 LEU P 86 7.658 -15.422 -3.276 0.00 0.00 PROA
ATOM 1368 HD23 LEU P 86 5.976 -15.405 -3.112 0.00 0.00 PROA
ATOM 1369 C LEU P 86 3.578 -12.711 -0.834 0.00 0.00 PROA
ATOM 1370 O LEU P 86 3.592 -13.268 0.232 0.00 0.00 PROA
ATOM 1371 N PHE P 87 2.783 -11.656 -1.133 0.00 0.00 PROA
ATOM 1372 HN PHE P 87 3.031 -11.188 -1.978 0.00 0.00 PROA
ATOM 1373 CA PHE P 87 1.896 -10.927 -0.168 0.00 0.00 PROA
ATOM 1374 HA PHE P 87 2.632 -10.614 0.558 0.00 0.00 PROA
ATOM 1375 CB PHE P 87 1.165 -9.675 -0.806 0.00 0.00 PROA
ATOM 1376 HB1 PHE P 87 0.589 -9.965 -1.710 0.00 0.00 PROA
ATOM 1377 HB2 PHE P 87 2.072 -9.129 -1.142 0.00 0.00 PROA
ATOM 1378 CG PHE P 87 0.349 -8.896 0.137 0.00 0.00 PROA
ATOM 1379 CD1 PHE P 87 0.864 -8.230 1.312 0.00 0.00 PROA
ATOM 1380 HD1 PHE P 87 1.881 -8.433 1.614 0.00 0.00 PROA
ATOM 1381 CE1 PHE P 87 0.041 -7.706 2.275 0.00 0.00 PROA
ATOM 1382 HE1 PHE P 87 0.453 -7.200 3.136 0.00 0.00 PROA
ATOM 1383 CZ PHE P 87 -1.392 -7.725 2.030 0.00 0.00 PROA
ATOM 1384 HZ PHE P 87 -1.981 -7.526 2.913 0.00 0.00 PROA
ATOM 1385 CD2 PHE P 87 -1.033 -8.868 -0.047 0.00 0.00 PROA
ATOM 1386 HD2 PHE P 87 -1.483 -9.203 -0.971 0.00 0.00 PROA
ATOM 1387 CE2 PHE P 87 -1.926 -8.209 0.830 0.00 0.00 PROA
ATOM 1388 HE2 PHE P 87 -2.993 -8.169 0.667 0.00 0.00 PROA
ATOM 1389 C PHE P 87 0.866 -11.732 0.617 0.00 0.00 PROA
ATOM 1390 O PHE P 87 0.853 -11.772 1.821 0.00 0.00 PROA
ATOM 1391 N VAL P 88 -0.020 -12.395 -0.083 0.00 0.00 PROA
ATOM 1392 HN VAL P 88 0.040 -12.339 -1.077 0.00 0.00 PROA
ATOM 1393 CA VAL P 88 -1.179 -13.027 0.545 0.00 0.00 PROA
ATOM 1394 HA VAL P 88 -1.747 -12.302 1.109 0.00 0.00 PROA
ATOM 1395 CB VAL P 88 -2.134 -13.545 -0.481 0.00 0.00 PROA
ATOM 1396 HB VAL P 88 -2.942 -14.171 -0.045 0.00 0.00 PROA
ATOM 1397 CG1 VAL P 88 -2.757 -12.362 -1.240 0.00 0.00 PROA
ATOM 1398 HG11 VAL P 88 -3.426 -12.737 -2.043 0.00 0.00 PROA
ATOM 1399 HG12 VAL P 88 -2.024 -11.628 -1.639 0.00 0.00 PROA

```
ATOM   1400 HG13 VAL P  88      -3.400 -11.709  -0.611  0.00  0.00      PROA
ATOM   1401  CG2 VAL P  88      -1.399 -14.512  -1.444  0.00  0.00      PROA
ATOM   1402 HG21 VAL P  88      -0.550 -13.994  -1.938  0.00  0.00      PROA
ATOM   1403 HG22 VAL P  88      -1.955 -14.950  -2.301  0.00  0.00      PROA
ATOM   1404 HG23 VAL P  88      -1.000 -15.384  -0.883  0.00  0.00      PROA
ATOM   1405  C   VAL P  88      -0.921 -14.176   1.578  0.00  0.00      PROA
ATOM   1406  O   VAL P  88      -1.807 -14.575   2.261  0.00  0.00      PROA
ATOM   1407  N   ILE P  89       0.362 -14.677   1.648  0.00  0.00      PROA
ATOM   1408  HN  ILE P  89       1.061 -14.357   1.013  0.00  0.00      PROA
ATOM   1409  CA  ILE P  89       0.788 -15.660   2.619  0.00  0.00      PROA
ATOM   1410  HA  ILE P  89      -0.010 -16.363   2.812  0.00  0.00      PROA
ATOM   1411  CB  ILE P  89       2.086 -16.394   2.168  0.00  0.00      PROA
ATOM   1412  HB  ILE P  89       2.930 -15.680   2.057  0.00  0.00      PROA
ATOM   1413  CG2 ILE P  89       2.357 -17.641   2.990  0.00  0.00      PROA
ATOM   1414 HG21 ILE P  89       1.442 -18.267   3.067  0.00  0.00      PROA
ATOM   1415 HG22 ILE P  89       2.704 -17.436   4.025  0.00  0.00      PROA
ATOM   1416 HG23 ILE P  89       3.157 -18.306   2.600  0.00  0.00      PROA
ATOM   1417  CG1 ILE P  89       1.965 -16.834   0.689  0.00  0.00      PROA
ATOM   1418 HG11 ILE P  89       2.880 -17.393   0.400  0.00  0.00      PROA
ATOM   1419 HG12 ILE P  89       1.857 -16.036  -0.076  0.00  0.00      PROA
ATOM   1420  CD  ILE P  89       0.899 -17.987   0.526  0.00  0.00      PROA
ATOM   1421  HD1 ILE P  89      -0.078 -17.614   0.901  0.00  0.00      PROA
ATOM   1422  HD2 ILE P  89       1.234 -18.931   1.008  0.00  0.00      PROA
ATOM   1423  HD3 ILE P  89       0.699 -18.152  -0.555  0.00  0.00      PROA
ATOM   1424  C   ILE P  89       1.110 -15.006   3.955  0.00  0.00      PROA
ATOM   1425  O   ILE P  89       1.214 -15.665   4.989  0.00  0.00      PROA
ATOM   1426  N   THR P  90       1.122 -13.613   3.994  0.00  0.00      PROA
ATOM   1427  HN  THR P  90       1.012 -13.105   3.144  0.00  0.00      PROA
ATOM   1428  CA  THR P  90       1.137 -12.899   5.302  0.00  0.00      PROA
ATOM   1429  HA  THR P  90       1.907 -13.274   5.959  0.00  0.00      PROA
ATOM   1430  CB  THR P  90       1.405 -11.374   5.154  0.00  0.00      PROA
ATOM   1431  HB  THR P  90       1.480 -10.863   6.138  0.00  0.00      PROA
ATOM   1432  OG1 THR P  90       0.464 -10.795   4.324  0.00  0.00      PROA
ATOM   1433  HG1 THR P  90       0.562 -11.057   3.406  0.00  0.00      PROA
ATOM   1434  CG2 THR P  90       2.752 -11.171   4.355  0.00  0.00      PROA
ATOM   1435 HG21 THR P  90       3.051 -10.134   4.616  0.00  0.00      PROA
ATOM   1436 HG22 THR P  90       2.724 -11.311   3.254  0.00  0.00      PROA
ATOM   1437 HG23 THR P  90       3.554 -11.819   4.769  0.00  0.00      PROA
ATOM   1438  C   THR P  90      -0.114 -12.967   6.170  0.00  0.00      PROA
ATOM   1439  O   THR P  90      -0.133 -13.096   7.371  0.00  0.00      PROA
ATOM   1440  N   LEU P  91      -1.312 -12.831   5.580  0.00  0.00      PROA
ATOM   1441  HN  LEU P  91      -1.396 -12.595   4.615  0.00  0.00      PROA
ATOM   1442  CA  LEU P  91      -2.648 -12.827   6.259  0.00  0.00      PROA
ATOM   1443  HA  LEU P  91      -2.571 -12.068   7.023  0.00  0.00      PROA
ATOM   1444  CB  LEU P  91      -3.774 -12.436   5.275  0.00  0.00      PROA
ATOM   1445  HB1 LEU P  91      -4.716 -12.608   5.839  0.00  0.00      PROA
ATOM   1446  HB2 LEU P  91      -3.844 -13.063   4.361  0.00  0.00      PROA
ATOM   1447  CG  LEU P  91      -3.863 -10.969   4.846  0.00  0.00      PROA
ATOM   1448  HG  LEU P  91      -3.962 -10.412   5.802  0.00  0.00      PROA
ATOM   1449  CD1 LEU P  91      -2.616 -10.432   4.056  0.00  0.00      PROA
ATOM   1450 HD11 LEU P  91      -2.793  -9.397   3.695  0.00  0.00      PROA
ATOM   1451 HD12 LEU P  91      -2.458 -11.106   3.187  0.00  0.00      PROA
ATOM   1452 HD13 LEU P  91      -1.861 -10.456   4.871  0.00  0.00      PROA
ATOM   1453  CD2 LEU P  91      -5.113 -10.731   4.051  0.00  0.00      PROA
ATOM   1454 HD21 LEU P  91      -5.181  -9.657   3.777  0.00  0.00      PROA
ATOM   1455 HD22 LEU P  91      -5.997 -11.082   4.624  0.00  0.00      PROA
ATOM   1456 HD23 LEU P  91      -5.050 -11.291   3.093  0.00  0.00      PROA
ATOM   1457  C   LEU P  91      -3.023 -14.080   6.977  0.00  0.00      PROA
ATOM   1458  O   LEU P  91      -3.528 -13.950   8.121  0.00  0.00      PROA
ATOM   1459  N   PRO P  92      -2.675 -15.298   6.534  0.00  0.00      PROA
ATOM   1460  CD  PRO P  92      -2.485 -15.706   5.163  0.00  0.00      PROA
```

ATOM	1461	HD1	PRO	P	92	-1.488	-15.311	4.876	0.00	0.00	PROA
ATOM	1462	HD2	PRO	P	92	-3.276	-15.385	4.452	0.00	0.00	PROA
ATOM	1463	CA	PRO	P	92	-2.520	-16.426	7.419	0.00	0.00	PROA
ATOM	1464	HA	PRO	P	92	-3.539	-16.663	7.689	0.00	0.00	PROA
ATOM	1465	CB	PRO	P	92	-1.840	-17.464	6.577	0.00	0.00	PROA
ATOM	1466	HB1	PRO	P	92	-2.024	-18.477	6.995	0.00	0.00	PROA
ATOM	1467	HB2	PRO	P	92	-0.748	-17.259	6.545	0.00	0.00	PROA
ATOM	1468	CG	PRO	P	92	-2.457	-17.290	5.212	0.00	0.00	PROA
ATOM	1469	HG1	PRO	P	92	-1.878	-17.685	4.350	0.00	0.00	PROA
ATOM	1470	HG2	PRO	P	92	-3.516	-17.583	5.044	0.00	0.00	PROA
ATOM	1471	C	PRO	P	92	-1.996	-16.257	8.865	0.00	0.00	PROA
ATOM	1472	O	PRO	P	92	-2.711	-16.583	9.824	0.00	0.00	PROA
ATOM	1473	N	PHE	P	93	-0.794	-15.688	8.945	0.00	0.00	PROA
ATOM	1474	HN	PHE	P	93	-0.403	-15.477	8.052	0.00	0.00	PROA
ATOM	1475	CA	PHE	P	93	-0.033	-15.400	10.134	0.00	0.00	PROA
ATOM	1476	HA	PHE	P	93	-0.137	-16.165	10.890	0.00	0.00	PROA
ATOM	1477	CB	PHE	P	93	1.414	-15.267	9.769	0.00	0.00	PROA
ATOM	1478	HB1	PHE	P	93	2.005	-15.062	10.687	0.00	0.00	PROA
ATOM	1479	HB2	PHE	P	93	1.525	-14.357	9.141	0.00	0.00	PROA
ATOM	1480	CG	PHE	P	93	2.213	-16.317	9.030	0.00	0.00	PROA
ATOM	1481	CD1	PHE	P	93	1.891	-17.658	9.163	0.00	0.00	PROA
ATOM	1482	HD1	PHE	P	93	1.149	-18.019	9.860	0.00	0.00	PROA
ATOM	1483	CE1	PHE	P	93	2.581	-18.609	8.496	0.00	0.00	PROA
ATOM	1484	HE1	PHE	P	93	2.476	-19.676	8.628	0.00	0.00	PROA
ATOM	1485	CZ	PHE	P	93	3.665	-18.221	7.696	0.00	0.00	PROA
ATOM	1486	HZ	PHE	P	93	4.217	-18.982	7.166	0.00	0.00	PROA
ATOM	1487	CD2	PHE	P	93	3.241	-15.951	8.205	0.00	0.00	PROA
ATOM	1488	HD2	PHE	P	93	3.436	-14.913	7.976	0.00	0.00	PROA
ATOM	1489	CE2	PHE	P	93	4.060	-16.894	7.535	0.00	0.00	PROA
ATOM	1490	HE2	PHE	P	93	4.961	-16.676	6.981	0.00	0.00	PROA
ATOM	1491	C	PHE	P	93	-0.698	-14.285	10.897	0.00	0.00	PROA
ATOM	1492	O	PHE	P	93	-0.772	-14.260	12.137	0.00	0.00	PROA
ATOM	1493	N	TRP	P	94	-1.217	-13.274	10.164	0.00	0.00	PROA
ATOM	1494	HN	TRP	P	94	-1.135	-13.121	9.183	0.00	0.00	PROA
ATOM	1495	CA	TRP	P	94	-1.852	-12.149	10.891	0.00	0.00	PROA
ATOM	1496	HA	TRP	P	94	-1.254	-11.921	11.761	0.00	0.00	PROA
ATOM	1497	CB	TRP	P	94	-1.977	-10.939	9.961	0.00	0.00	PROA
ATOM	1498	HB1	TRP	P	94	-2.593	-10.171	10.475	0.00	0.00	PROA
ATOM	1499	HB2	TRP	P	94	-2.372	-11.197	8.955	0.00	0.00	PROA
ATOM	1500	CG	TRP	P	94	-0.652	-10.288	9.779	0.00	0.00	PROA
ATOM	1501	CD1	TRP	P	94	0.180	-9.869	10.808	0.00	0.00	PROA
ATOM	1502	HD1	TRP	P	94	-0.063	-9.946	11.857	0.00	0.00	PROA
ATOM	1503	NE1	TRP	P	94	1.152	-9.021	10.341	0.00	0.00	PROA
ATOM	1504	HE1	TRP	P	94	1.861	-8.581	10.846	0.00	0.00	PROA
ATOM	1505	CE2	TRP	P	94	0.967	-8.837	9.023	0.00	0.00	PROA
ATOM	1506	CD2	TRP	P	94	-0.191	-9.613	8.609	0.00	0.00	PROA
ATOM	1507	CE3	TRP	P	94	-0.678	-9.391	7.334	0.00	0.00	PROA
ATOM	1508	HE3	TRP	P	94	-1.492	-9.967	6.919	0.00	0.00	PROA
ATOM	1509	CZ3	TRP	P	94	-0.032	-8.456	6.480	0.00	0.00	PROA
ATOM	1510	HZ3	TRP	P	94	-0.483	-8.347	5.505	0.00	0.00	PROA
ATOM	1511	CZ2	TRP	P	94	1.580	-7.953	8.146	0.00	0.00	PROA
ATOM	1512	HZ2	TRP	P	94	2.443	-7.421	8.517	0.00	0.00	PROA
ATOM	1513	CH2	TRP	P	94	1.016	-7.732	6.895	0.00	0.00	PROA
ATOM	1514	HH2	TRP	P	94	1.412	-6.869	6.380	0.00	0.00	PROA
ATOM	1515	C	TRP	P	94	-3.235	-12.618	11.513	0.00	0.00	PROA
ATOM	1516	O	TRP	P	94	-3.665	-12.223	12.621	0.00	0.00	PROA
ATOM	1517	N	ALA	P	95	-3.982	-13.558	10.827	0.00	0.00	PROA
ATOM	1518	HN	ALA	P	95	-3.746	-13.742	9.876	0.00	0.00	PROA
ATOM	1519	CA	ALA	P	95	-5.110	-14.391	11.451	0.00	0.00	PROA
ATOM	1520	HA	ALA	P	95	-5.809	-13.658	11.827	0.00	0.00	PROA
ATOM	1521	CB	ALA	P	95	-5.692	-15.310	10.352	0.00	0.00	PROA

```
ATOM   1522  HB1 ALA P  95      -5.904 -14.866   9.356  0.00  0.00      PROA
ATOM   1523  HB2 ALA P  95      -6.599 -15.846  10.704  0.00  0.00      PROA
ATOM   1524  HB3 ALA P  95      -4.933 -16.042  10.003  0.00  0.00      PROA
ATOM   1525  C   ALA P  95      -4.805 -15.270  12.630  0.00  0.00      PROA
ATOM   1526  O   ALA P  95      -5.545 -15.361  13.607  0.00  0.00      PROA
ATOM   1527  N   VAL P  96      -3.668 -15.957  12.675  0.00  0.00      PROA
ATOM   1528  HN  VAL P  96      -2.960 -15.917  11.974  0.00  0.00      PROA
ATOM   1529  CA  VAL P  96      -3.348 -16.845  13.786  0.00  0.00      PROA
ATOM   1530  HA  VAL P  96      -4.246 -17.362  14.092  0.00  0.00      PROA
ATOM   1531  CB  VAL P  96      -2.285 -17.885  13.435  0.00  0.00      PROA
ATOM   1532  HB  VAL P  96      -1.386 -17.423  12.976  0.00  0.00      PROA
ATOM   1533  CG1 VAL P  96      -1.970 -18.877  14.580  0.00  0.00      PROA
ATOM   1534 HG11 VAL P  96      -1.312 -19.623  14.086  0.00  0.00      PROA
ATOM   1535 HG12 VAL P  96      -2.873 -19.414  14.943  0.00  0.00      PROA
ATOM   1536 HG13 VAL P  96      -1.375 -18.485  15.432  0.00  0.00      PROA
ATOM   1537  CG2 VAL P  96      -2.960 -18.860  12.417  0.00  0.00      PROA
ATOM   1538 HG21 VAL P  96      -2.368 -19.790  12.278  0.00  0.00      PROA
ATOM   1539 HG22 VAL P  96      -2.998 -18.365  11.422  0.00  0.00      PROA
ATOM   1540 HG23 VAL P  96      -3.986 -19.148  12.731  0.00  0.00      PROA
ATOM   1541  C   VAL P  96      -3.032 -16.069  15.016  0.00  0.00      PROA
ATOM   1542  O   VAL P  96      -3.284 -16.477  16.130  0.00  0.00      PROA
ATOM   1543  N   ASP P  97      -2.511 -14.834  14.849  0.00  0.00      PROA
ATOM   1544  HN  ASP P  97      -2.209 -14.472  13.971  0.00  0.00      PROA
ATOM   1545  CA  ASP P  97      -2.286 -13.867  15.922  0.00  0.00      PROA
ATOM   1546  HA  ASP P  97      -1.645 -14.404  16.606  0.00  0.00      PROA
ATOM   1547  CB  ASP P  97      -1.364 -12.682  15.363  0.00  0.00      PROA
ATOM   1548  HB1 ASP P  97      -1.797 -12.250  14.436  0.00  0.00      PROA
ATOM   1549  HB2 ASP P  97      -0.464 -13.240  15.028  0.00  0.00      PROA
ATOM   1550  CG  ASP P  97      -1.062 -11.562  16.349  0.00  0.00      PROA
ATOM   1551  OD1 ASP P  97      -0.014 -11.624  17.091  0.00  0.00      PROA
ATOM   1552  OD2 ASP P  97      -1.949 -10.745  16.461  0.00  0.00      PROA
ATOM   1553  C   ASP P  97      -3.530 -13.436  16.725  0.00  0.00      PROA
ATOM   1554  O   ASP P  97      -3.594 -13.457  17.953  0.00  0.00      PROA
ATOM   1555  N   ALA P  98      -4.669 -13.100  16.014  0.00  0.00      PROA
ATOM   1556  HN  ALA P  98      -4.661 -13.197  15.022  0.00  0.00      PROA
ATOM   1557  CA  ALA P  98      -5.879 -12.685  16.660  0.00  0.00      PROA
ATOM   1558  HA  ALA P  98      -5.544 -11.925  17.349  0.00  0.00      PROA
ATOM   1559  CB  ALA P  98      -6.949 -12.160  15.729  0.00  0.00      PROA
ATOM   1560  HB1 ALA P  98      -7.927 -11.905  16.189  0.00  0.00      PROA
ATOM   1561  HB2 ALA P  98      -7.141 -12.938  14.960  0.00  0.00      PROA
ATOM   1562  HB3 ALA P  98      -6.585 -11.343  15.070  0.00  0.00      PROA
ATOM   1563  C   ALA P  98      -6.526 -13.697  17.578  0.00  0.00      PROA
ATOM   1564  O   ALA P  98      -6.874 -13.391  18.733  0.00  0.00      PROA
ATOM   1565  N   VAL P  99      -6.547 -14.991  17.041  0.00  0.00      PROA
ATOM   1566  HN  VAL P  99      -6.105 -15.152  16.162  0.00  0.00      PROA
ATOM   1567  CA  VAL P  99      -7.233 -16.062  17.695  0.00  0.00      PROA
ATOM   1568  HA  VAL P  99      -8.001 -15.689  18.356  0.00  0.00      PROA
ATOM   1569  CB  VAL P  99      -7.767 -17.087  16.657  0.00  0.00      PROA
ATOM   1570  HB  VAL P  99      -8.162 -17.954  17.229  0.00  0.00      PROA
ATOM   1571  CG1 VAL P  99      -8.881 -16.428  15.775  0.00  0.00      PROA
ATOM   1572 HG11 VAL P  99      -8.483 -15.731  15.007  0.00  0.00      PROA
ATOM   1573 HG12 VAL P  99      -9.672 -15.956  16.396  0.00  0.00      PROA
ATOM   1574 HG13 VAL P  99      -9.334 -17.232  15.156  0.00  0.00      PROA
ATOM   1575  CG2 VAL P  99      -6.691 -17.692  15.752  0.00  0.00      PROA
ATOM   1576 HG21 VAL P  99      -6.491 -17.112  14.825  0.00  0.00      PROA
ATOM   1577 HG22 VAL P  99      -6.853 -18.742  15.424  0.00  0.00      PROA
ATOM   1578 HG23 VAL P  99      -5.662 -17.631  16.166  0.00  0.00      PROA
ATOM   1579  C   VAL P  99      -6.360 -16.940  18.564  0.00  0.00      PROA
ATOM   1580  O   VAL P  99      -6.850 -17.759  19.317  0.00  0.00      PROA
ATOM   1581  N   ALA P 100      -5.011 -16.752  18.468  0.00  0.00      PROA
ATOM   1582  HN  ALA P 100      -4.676 -16.074  17.818  0.00  0.00      PROA
```

ATOM	1583	CA	ALA	P	100	-4.055	-17.486	19.229	0.00	0.00	PROA
ATOM	1584	HA	ALA	P	100	-4.469	-17.861	20.154	0.00	0.00	PROA
ATOM	1585	CB	ALA	P	100	-3.913	-18.932	18.536	0.00	0.00	PROA
ATOM	1586	HB1	ALA	P	100	-4.952	-19.311	18.425	0.00	0.00	PROA
ATOM	1587	HB2	ALA	P	100	-3.453	-19.666	19.231	0.00	0.00	PROA
ATOM	1588	HB3	ALA	P	100	-3.321	-18.836	17.600	0.00	0.00	PROA
ATOM	1589	C	ALA	P	100	-2.859	-16.712	19.613	0.00	0.00	PROA
ATOM	1590	O	ALA	P	100	-2.870	-15.930	20.625	0.00	0.00	PROA
ATOM	1591	N	ASN	P	101	-1.748	-16.893	18.882	0.00	0.00	PROA
ATOM	1592	HN	ASN	P	101	-1.778	-17.562	18.143	0.00	0.00	PROA
ATOM	1593	CA	ASN	P	101	-0.504	-16.209	19.201	0.00	0.00	PROA
ATOM	1594	HA	ASN	P	101	-0.742	-15.201	19.505	0.00	0.00	PROA
ATOM	1595	CB	ASN	P	101	0.230	-17.010	20.347	0.00	0.00	PROA
ATOM	1596	HB1	ASN	P	101	0.535	-17.971	19.880	0.00	0.00	PROA
ATOM	1597	HB2	ASN	P	101	-0.565	-17.340	21.050	0.00	0.00	PROA
ATOM	1598	CG	ASN	P	101	1.349	-16.347	21.191	0.00	0.00	PROA
ATOM	1599	OD1	ASN	P	101	1.536	-15.146	20.896	0.00	0.00	PROA
ATOM	1600	ND2	ASN	P	101	2.082	-17.105	22.050	0.00	0.00	PROA
ATOM	1601	HD21	ASN	P	101	2.815	-16.731	22.618	0.00	0.00	PROA
ATOM	1602	HD22	ASN	P	101	1.844	-18.057	22.242	0.00	0.00	PROA
ATOM	1603	C	ASN	P	101	0.449	-16.132	17.983	0.00	0.00	PROA
ATOM	1604	O	ASN	P	101	0.252	-16.816	16.987	0.00	0.00	PROA
ATOM	1605	N	TRP	P	102	1.527	-15.356	18.024	0.00	0.00	PROA
ATOM	1606	HN	TRP	P	102	1.895	-14.941	18.852	0.00	0.00	PROA
ATOM	1607	CA	TRP	P	102	2.435	-15.382	16.919	0.00	0.00	PROA
ATOM	1608	HA	TRP	P	102	1.889	-15.483	15.992	0.00	0.00	PROA
ATOM	1609	CB	TRP	P	102	3.069	-13.954	16.763	0.00	0.00	PROA
ATOM	1610	HB1	TRP	P	102	3.545	-13.759	17.748	0.00	0.00	PROA
ATOM	1611	HB2	TRP	P	102	2.286	-13.177	16.633	0.00	0.00	PROA
ATOM	1612	CG	TRP	P	102	4.141	-13.776	15.739	0.00	0.00	PROA
ATOM	1613	CD1	TRP	P	102	5.508	-14.045	15.675	0.00	0.00	PROA
ATOM	1614	HD1	TRP	P	102	5.970	-14.117	16.648	0.00	0.00	PROA
ATOM	1615	NE1	TRP	P	102	5.985	-13.778	14.403	0.00	0.00	PROA
ATOM	1616	HE1	TRP	P	102	6.820	-14.130	14.042	0.00	0.00	PROA
ATOM	1617	CE2	TRP	P	102	4.929	-13.470	13.577	0.00	0.00	PROA
ATOM	1618	CD2	TRP	P	102	3.731	-13.497	14.336	0.00	0.00	PROA
ATOM	1619	CE3	TRP	P	102	2.516	-13.144	13.791	0.00	0.00	PROA
ATOM	1620	HE3	TRP	P	102	1.568	-13.088	14.306	0.00	0.00	PROA
ATOM	1621	CZ3	TRP	P	102	2.479	-12.868	12.442	0.00	0.00	PROA
ATOM	1622	HZ3	TRP	P	102	1.521	-12.694	11.975	0.00	0.00	PROA
ATOM	1623	CZ2	TRP	P	102	4.879	-13.180	12.229	0.00	0.00	PROA
ATOM	1624	HZ2	TRP	P	102	5.758	-13.340	11.623	0.00	0.00	PROA
ATOM	1625	CH2	TRP	P	102	3.611	-12.954	11.610	0.00	0.00	PROA
ATOM	1626	HH2	TRP	P	102	3.518	-12.927	10.535	0.00	0.00	PROA
ATOM	1627	C	TRP	P	102	3.482	-16.509	16.991	0.00	0.00	PROA
ATOM	1628	O	TRP	P	102	4.415	-16.614	17.779	0.00	0.00	PROA
ATOM	1629	N	TYR	P	103	3.283	-17.445	16.048	0.00	0.00	PROA
ATOM	1630	HN	TYR	P	103	2.601	-17.440	15.321	0.00	0.00	PROA
ATOM	1631	CA	TYR	P	103	4.049	-18.693	16.166	0.00	0.00	PROA
ATOM	1632	HA	TYR	P	103	4.588	-18.760	17.099	0.00	0.00	PROA
ATOM	1633	CB	TYR	P	103	3.269	-19.965	16.035	0.00	0.00	PROA
ATOM	1634	HB1	TYR	P	103	3.937	-20.808	16.316	0.00	0.00	PROA
ATOM	1635	HB2	TYR	P	103	2.984	-20.331	15.026	0.00	0.00	PROA
ATOM	1636	CG	TYR	P	103	2.009	-20.106	16.960	0.00	0.00	PROA
ATOM	1637	CD1	TYR	P	103	2.176	-20.318	18.323	0.00	0.00	PROA
ATOM	1638	HD1	TYR	P	103	3.205	-20.348	18.649	0.00	0.00	PROA
ATOM	1639	CE1	TYR	P	103	1.028	-20.564	19.137	0.00	0.00	PROA
ATOM	1640	HE1	TYR	P	103	1.244	-20.702	20.186	0.00	0.00	PROA
ATOM	1641	CZ	TYR	P	103	-0.224	-20.721	18.580	0.00	0.00	PROA
ATOM	1642	OH	TYR	P	103	-1.356	-21.078	19.384	0.00	0.00	PROA
ATOM	1643	HH	TYR	P	103	-1.181	-21.130	20.327	0.00	0.00	PROA

ATOM	1644	CD2	TYR	P	103	0.764	-20.204	16.388	0.00	0.00	PROA
ATOM	1645	HD2	TYR	P	103	0.745	-20.105	15.313	0.00	0.00	PROA
ATOM	1646	CE2	TYR	P	103	-0.363	-20.479	17.179	0.00	0.00	PROA
ATOM	1647	HE2	TYR	P	103	-1.328	-20.588	16.707	0.00	0.00	PROA
ATOM	1648	C	TYR	P	103	5.110	-18.706	15.111	0.00	0.00	PROA
ATOM	1649	O	TYR	P	103	5.688	-19.752	14.851	0.00	0.00	PROA
ATOM	1650	N	PHE	P	104	5.467	-17.612	14.449	0.00	0.00	PROA
ATOM	1651	HN	PHE	P	104	5.120	-16.742	14.791	0.00	0.00	PROA
ATOM	1652	CA	PHE	P	104	6.303	-17.667	13.263	0.00	0.00	PROA
ATOM	1653	HA	PHE	P	104	6.547	-18.655	12.903	0.00	0.00	PROA
ATOM	1654	CB	PHE	P	104	5.468	-17.011	12.137	0.00	0.00	PROA
ATOM	1655	HB1	PHE	P	104	6.035	-17.187	11.198	0.00	0.00	PROA
ATOM	1656	HB2	PHE	P	104	5.211	-15.930	12.157	0.00	0.00	PROA
ATOM	1657	CG	PHE	P	104	4.108	-17.683	12.164	0.00	0.00	PROA
ATOM	1658	CD1	PHE	P	104	3.953	-19.085	11.835	0.00	0.00	PROA
ATOM	1659	HD1	PHE	P	104	4.852	-19.635	11.597	0.00	0.00	PROA
ATOM	1660	CE1	PHE	P	104	2.684	-19.706	11.881	0.00	0.00	PROA
ATOM	1661	HE1	PHE	P	104	2.713	-20.767	11.684	0.00	0.00	PROA
ATOM	1662	CZ	PHE	P	104	1.559	-19.010	12.294	0.00	0.00	PROA
ATOM	1663	HZ	PHE	P	104	0.600	-19.506	12.257	0.00	0.00	PROA
ATOM	1664	CD2	PHE	P	104	2.930	-17.007	12.599	0.00	0.00	PROA
ATOM	1665	HD2	PHE	P	104	2.930	-15.991	12.967	0.00	0.00	PROA
ATOM	1666	CE2	PHE	P	104	1.722	-17.715	12.622	0.00	0.00	PROA
ATOM	1667	HE2	PHE	P	104	0.821	-17.240	12.983	0.00	0.00	PROA
ATOM	1668	C	PHE	P	104	7.678	-17.002	13.383	0.00	0.00	PROA
ATOM	1669	O	PHE	P	104	8.313	-16.790	12.397	0.00	0.00	PROA
ATOM	1670	N	GLY	P	105	8.156	-16.738	14.581	0.00	0.00	PROA
ATOM	1671	HN	GLY	P	105	7.594	-16.920	15.384	0.00	0.00	PROA
ATOM	1672	CA	GLY	P	105	9.501	-16.268	14.996	0.00	0.00	PROA
ATOM	1673	HA1	GLY	P	105	10.296	-16.966	14.777	0.00	0.00	PROA
ATOM	1674	HA2	GLY	P	105	9.370	-16.073	16.050	0.00	0.00	PROA
ATOM	1675	C	GLY	P	105	9.958	-14.959	14.468	0.00	0.00	PROA
ATOM	1676	O	GLY	P	105	9.189	-14.306	13.729	0.00	0.00	PROA
ATOM	1677	N	ASN	P	106	11.138	-14.505	14.879	0.00	0.00	PROA
ATOM	1678	HN	ASN	P	106	11.667	-15.167	15.405	0.00	0.00	PROA
ATOM	1679	CA	ASN	P	106	11.705	-13.256	14.397	0.00	0.00	PROA
ATOM	1680	HA	ASN	P	106	10.971	-12.517	14.683	0.00	0.00	PROA
ATOM	1681	CB	ASN	P	106	12.974	-12.969	15.127	0.00	0.00	PROA
ATOM	1682	HB1	ASN	P	106	13.560	-12.096	14.767	0.00	0.00	PROA
ATOM	1683	HB2	ASN	P	106	13.652	-13.837	14.983	0.00	0.00	PROA
ATOM	1684	CG	ASN	P	106	12.716	-12.781	16.619	0.00	0.00	PROA
ATOM	1685	OD1	ASN	P	106	12.365	-11.604	16.905	0.00	0.00	PROA
ATOM	1686	ND2	ASN	P	106	12.979	-13.696	17.545	0.00	0.00	PROA
ATOM	1687	HD21	ASN	P	106	12.832	-13.428	18.497	0.00	0.00	PROA
ATOM	1688	HD22	ASN	P	106	12.770	-14.663	17.400	0.00	0.00	PROA
ATOM	1689	C	ASN	P	106	11.872	-13.199	12.903	0.00	0.00	PROA
ATOM	1690	O	ASN	P	106	11.639	-12.207	12.280	0.00	0.00	PROA
ATOM	1691	N	PHE	P	107	12.327	-14.247	12.237	0.00	0.00	PROA
ATOM	1692	HN	PHE	P	107	12.701	-15.052	12.691	0.00	0.00	PROA
ATOM	1693	CA	PHE	P	107	12.616	-14.396	10.846	0.00	0.00	PROA
ATOM	1694	HA	PHE	P	107	13.238	-13.558	10.568	0.00	0.00	PROA
ATOM	1695	CB	PHE	P	107	13.369	-15.726	10.476	0.00	0.00	PROA
ATOM	1696	HB1	PHE	P	107	12.673	-16.544	10.760	0.00	0.00	PROA
ATOM	1697	HB2	PHE	P	107	14.333	-15.898	11.001	0.00	0.00	PROA
ATOM	1698	CG	PHE	P	107	13.675	-15.810	8.999	0.00	0.00	PROA
ATOM	1699	CD1	PHE	P	107	14.255	-14.769	8.326	0.00	0.00	PROA
ATOM	1700	HD1	PHE	P	107	14.501	-13.836	8.812	0.00	0.00	PROA
ATOM	1701	CE1	PHE	P	107	14.691	-14.901	6.973	0.00	0.00	PROA
ATOM	1702	HE1	PHE	P	107	15.175	-14.121	6.404	0.00	0.00	PROA
ATOM	1703	CZ	PHE	P	107	14.599	-16.174	6.298	0.00	0.00	PROA
ATOM	1704	HZ	PHE	P	107	14.963	-16.369	5.301	0.00	0.00	PROA

ATOM	1705	CD2	PHE	P	107	13.576	-17.036	8.405	0.00	0.00	PROA
ATOM	1706	HD2	PHE	P	107	13.357	-17.915	8.994	0.00	0.00	PROA
ATOM	1707	CE2	PHE	P	107	14.051	-17.223	7.066	0.00	0.00	PROA
ATOM	1708	HE2	PHE	P	107	14.004	-18.271	6.808	0.00	0.00	PROA
ATOM	1709	C	PHE	P	107	11.394	-14.205	9.933	0.00	0.00	PROA
ATOM	1710	O	PHE	P	107	11.499	-13.631	8.852	0.00	0.00	PROA
ATOM	1711	N	LEU	P	108	10.146	-14.716	10.346	0.00	0.00	PROA
ATOM	1712	HN	LEU	P	108	10.062	-15.236	11.193	0.00	0.00	PROA
ATOM	1713	CA	LEU	P	108	8.994	-14.539	9.466	0.00	0.00	PROA
ATOM	1714	HA	LEU	P	108	9.368	-14.318	8.477	0.00	0.00	PROA
ATOM	1715	CB	LEU	P	108	8.139	-15.888	9.283	0.00	0.00	PROA
ATOM	1716	HB1	LEU	P	108	7.422	-15.757	8.444	0.00	0.00	PROA
ATOM	1717	HB2	LEU	P	108	7.543	-16.136	10.187	0.00	0.00	PROA
ATOM	1718	CG	LEU	P	108	8.995	-17.107	8.879	0.00	0.00	PROA
ATOM	1719	HG	LEU	P	108	9.823	-17.240	9.608	0.00	0.00	PROA
ATOM	1720	CD1	LEU	P	108	8.151	-18.391	8.918	0.00	0.00	PROA
ATOM	1721	HD11	LEU	P	108	8.617	-19.357	8.627	0.00	0.00	PROA
ATOM	1722	HD12	LEU	P	108	7.293	-18.147	8.256	0.00	0.00	PROA
ATOM	1723	HD13	LEU	P	108	7.745	-18.485	9.948	0.00	0.00	PROA
ATOM	1724	CD2	LEU	P	108	9.535	-16.952	7.439	0.00	0.00	PROA
ATOM	1725	HD21	LEU	P	108	10.231	-16.090	7.352	0.00	0.00	PROA
ATOM	1726	HD22	LEU	P	108	8.746	-16.981	6.658	0.00	0.00	PROA
ATOM	1727	HD23	LEU	P	108	10.177	-17.855	7.349	0.00	0.00	PROA
ATOM	1728	C	LEU	P	108	8.137	-13.309	9.906	0.00	0.00	PROA
ATOM	1729	O	LEU	P	108	7.450	-12.746	9.099	0.00	0.00	PROA
ATOM	1730	N	CYS	P	109	8.506	-12.807	11.133	0.00	0.00	PROA
ATOM	1731	HN	CYS	P	109	9.127	-13.365	11.679	0.00	0.00	PROA
ATOM	1732	CA	CYS	P	109	8.157	-11.436	11.550	0.00	0.00	PROA
ATOM	1733	HA	CYS	P	109	7.077	-11.461	11.549	0.00	0.00	PROA
ATOM	1734	CB	CYS	P	109	8.651	-11.157	12.970	0.00	0.00	PROA
ATOM	1735	HB1	CYS	P	109	9.751	-11.167	12.820	0.00	0.00	PROA
ATOM	1736	HB2	CYS	P	109	8.321	-11.969	13.653	0.00	0.00	PROA
ATOM	1737	SG	CYS	P	109	8.086	-9.655	13.758	0.00	0.00	PROA
ATOM	1738	C	CYS	P	109	8.658	-10.349	10.612	0.00	0.00	PROA
ATOM	1739	O	CYS	P	109	8.019	-9.372	10.346	0.00	0.00	PROA
ATOM	1740	N	LYS	P	110	9.955	-10.458	10.263	0.00	0.00	PROA
ATOM	1741	HN	LYS	P	110	10.396	-11.292	10.585	0.00	0.00	PROA
ATOM	1742	CA	LYS	P	110	10.474	-9.651	9.143	0.00	0.00	PROA
ATOM	1743	HA	LYS	P	110	10.436	-8.588	9.329	0.00	0.00	PROA
ATOM	1744	CB	LYS	P	110	12.002	-9.861	8.918	0.00	0.00	PROA
ATOM	1745	HB1	LYS	P	110	12.400	-9.272	8.064	0.00	0.00	PROA
ATOM	1746	HB2	LYS	P	110	12.209	-10.943	8.776	0.00	0.00	PROA
ATOM	1747	CG	LYS	P	110	12.769	-9.502	10.258	0.00	0.00	PROA
ATOM	1748	HG1	LYS	P	110	12.115	-9.825	11.096	0.00	0.00	PROA
ATOM	1749	HG2	LYS	P	110	12.807	-8.395	10.343	0.00	0.00	PROA
ATOM	1750	CD	LYS	P	110	14.105	-10.231	10.533	0.00	0.00	PROA
ATOM	1751	HD1	LYS	P	110	14.803	-9.963	9.711	0.00	0.00	PROA
ATOM	1752	HD2	LYS	P	110	13.939	-11.321	10.401	0.00	0.00	PROA
ATOM	1753	CE	LYS	P	110	14.649	-10.095	11.926	0.00	0.00	PROA
ATOM	1754	HE1	LYS	P	110	15.428	-10.780	12.324	0.00	0.00	PROA
ATOM	1755	HE2	LYS	P	110	13.769	-10.409	12.528	0.00	0.00	PROA
ATOM	1756	NZ	LYS	P	110	14.996	-8.684	12.274	0.00	0.00	PROA
ATOM	1757	HZ1	LYS	P	110	15.887	-8.544	12.792	0.00	0.00	PROA
ATOM	1758	HZ2	LYS	P	110	14.281	-8.295	12.922	0.00	0.00	PROA
ATOM	1759	HZ3	LYS	P	110	15.068	-8.034	11.465	0.00	0.00	PROA
ATOM	1760	C	LYS	P	110	9.871	-9.863	7.805	0.00	0.00	PROA
ATOM	1761	O	LYS	P	110	9.580	-8.900	7.087	0.00	0.00	PROA
ATOM	1762	N	ALA	P	111	9.581	-11.123	7.437	0.00	0.00	PROA
ATOM	1763	HN	ALA	P	111	9.862	-11.944	7.927	0.00	0.00	PROA
ATOM	1764	CA	ALA	P	111	9.018	-11.436	6.136	0.00	0.00	PROA
ATOM	1765	HA	ALA	P	111	9.662	-10.999	5.387	0.00	0.00	PROA

ATOM	1766	CB	ALA	P	111	8.809	-12.927	5.876	0.00	0.00	PROA
ATOM	1767	HB1	ALA	P	111	8.561	-13.095	4.806	0.00	0.00	PROA
ATOM	1768	HB2	ALA	P	111	8.030	-13.290	6.579	0.00	0.00	PROA
ATOM	1769	HB3	ALA	P	111	9.787	-13.430	6.032	0.00	0.00	PROA
ATOM	1770	C	ALA	P	111	7.707	-10.735	5.896	0.00	0.00	PROA
ATOM	1771	O	ALA	P	111	7.602	-10.126	4.848	0.00	0.00	PROA
ATOM	1772	N	VAL	P	112	6.760	-10.758	6.900	0.00	0.00	PROA
ATOM	1773	HN	VAL	P	112	6.984	-11.243	7.742	0.00	0.00	PROA
ATOM	1774	CA	VAL	P	112	5.455	-10.172	6.600	0.00	0.00	PROA
ATOM	1775	HA	VAL	P	112	5.164	-10.626	5.664	0.00	0.00	PROA
ATOM	1776	CB	VAL	P	112	4.457	-10.487	7.724	0.00	0.00	PROA
ATOM	1777	HB	VAL	P	112	3.551	-9.854	7.610	0.00	0.00	PROA
ATOM	1778	CG1	VAL	P	112	4.189	-11.952	7.744	0.00	0.00	PROA
ATOM	1779	HG11	VAL	P	112	3.471	-12.304	8.516	0.00	0.00	PROA
ATOM	1780	HG12	VAL	P	112	5.054	-12.600	8.001	0.00	0.00	PROA
ATOM	1781	HG13	VAL	P	112	3.842	-12.299	6.747	0.00	0.00	PROA
ATOM	1782	CG2	VAL	P	112	4.963	-10.075	9.162	0.00	0.00	PROA
ATOM	1783	HG21	VAL	P	112	5.167	-8.993	9.017	0.00	0.00	PROA
ATOM	1784	HG22	VAL	P	112	5.876	-10.675	9.368	0.00	0.00	PROA
ATOM	1785	HG23	VAL	P	112	4.223	-10.055	9.990	0.00	0.00	PROA
ATOM	1786	C	VAL	P	112	5.553	-8.596	6.344	0.00	0.00	PROA
ATOM	1787	O	VAL	P	112	4.991	-8.090	5.378	0.00	0.00	PROA
ATOM	1788	N	HSD	P	113	6.315	-7.835	7.154	0.00	0.00	PROA
ATOM	1789	HN	HSD	P	113	6.862	-8.218	7.895	0.00	0.00	PROA
ATOM	1790	CA	HSD	P	113	6.442	-6.383	6.981	0.00	0.00	PROA
ATOM	1791	HA	HSD	P	113	5.443	-6.007	6.820	0.00	0.00	PROA
ATOM	1792	CB	HSD	P	113	7.114	-5.707	8.128	0.00	0.00	PROA
ATOM	1793	HB1	HSD	P	113	7.234	-4.629	7.890	0.00	0.00	PROA
ATOM	1794	HB2	HSD	P	113	8.145	-6.118	8.174	0.00	0.00	PROA
ATOM	1795	ND1	HSD	P	113	6.748	-6.823	10.339	0.00	0.00	PROA
ATOM	1796	HD1	HSD	P	113	7.486	-7.489	10.238	0.00	0.00	PROA
ATOM	1797	CG	HSD	P	113	6.344	-5.900	9.439	0.00	0.00	PROA
ATOM	1798	CE1	HSD	P	113	6.001	-6.573	11.448	0.00	0.00	PROA
ATOM	1799	HE1	HSD	P	113	6.238	-7.099	12.373	0.00	0.00	PROA
ATOM	1800	NE2	HSD	P	113	5.255	-5.476	11.307	0.00	0.00	PROA
ATOM	1801	CD2	HSD	P	113	5.409	-5.108	10.021	0.00	0.00	PROA
ATOM	1802	HD2	HSD	P	113	4.910	-4.278	9.536	0.00	0.00	PROA
ATOM	1803	C	HSD	P	113	7.187	-6.107	5.620	0.00	0.00	PROA
ATOM	1804	O	HSD	P	113	6.845	-5.169	4.915	0.00	0.00	PROA
ATOM	1805	N	VAL	P	114	8.308	-6.873	5.332	0.00	0.00	PROA
ATOM	1806	HN	VAL	P	114	8.733	-7.560	5.916	0.00	0.00	PROA
ATOM	1807	CA	VAL	P	114	9.064	-6.669	4.124	0.00	0.00	PROA
ATOM	1808	HA	VAL	P	114	9.500	-5.682	4.176	0.00	0.00	PROA
ATOM	1809	CB	VAL	P	114	10.183	-7.715	4.127	0.00	0.00	PROA
ATOM	1810	HB	VAL	P	114	9.809	-8.732	4.372	0.00	0.00	PROA
ATOM	1811	CG1	VAL	P	114	10.902	-7.840	2.748	0.00	0.00	PROA
ATOM	1812	HG11	VAL	P	114	11.646	-8.662	2.807	0.00	0.00	PROA
ATOM	1813	HG12	VAL	P	114	11.379	-6.901	2.394	0.00	0.00	PROA
ATOM	1814	HG13	VAL	P	114	10.243	-8.150	1.909	0.00	0.00	PROA
ATOM	1815	CG2	VAL	P	114	11.203	-7.348	5.191	0.00	0.00	PROA
ATOM	1816	HG21	VAL	P	114	10.823	-7.050	6.192	0.00	0.00	PROA
ATOM	1817	HG22	VAL	P	114	11.837	-6.472	4.935	0.00	0.00	PROA
ATOM	1818	HG23	VAL	P	114	11.839	-8.258	5.231	0.00	0.00	PROA
ATOM	1819	C	VAL	P	114	8.302	-6.805	2.885	0.00	0.00	PROA
ATOM	1820	O	VAL	P	114	8.344	-5.926	2.019	0.00	0.00	PROA
ATOM	1821	N	ILE	P	115	7.507	-7.828	2.653	0.00	0.00	PROA
ATOM	1822	HN	ILE	P	115	7.484	-8.609	3.271	0.00	0.00	PROA
ATOM	1823	CA	ILE	P	115	6.651	-7.831	1.435	0.00	0.00	PROA
ATOM	1824	HA	ILE	P	115	7.346	-7.599	0.642	0.00	0.00	PROA
ATOM	1825	CB	ILE	P	115	6.169	-9.255	1.113	0.00	0.00	PROA
ATOM	1826	HB	ILE	P	115	6.936	-10.055	1.197	0.00	0.00	PROA

```
ATOM   1827  CG2 ILE P 115       5.164  -9.829   2.129  0.00  0.00      PROA
ATOM   1828 HG21 ILE P 115       4.735 -10.832   1.919  0.00  0.00      PROA
ATOM   1829 HG22 ILE P 115       4.304  -9.146   2.298  0.00  0.00      PROA
ATOM   1830 HG23 ILE P 115       5.665  -9.895   3.118  0.00  0.00      PROA
ATOM   1831  CG1 ILE P 115       5.591  -9.311  -0.310  0.00  0.00      PROA
ATOM   1832 HG11 ILE P 115       4.629  -8.772  -0.174  0.00  0.00      PROA
ATOM   1833 HG12 ILE P 115       5.325 -10.364  -0.546  0.00  0.00      PROA
ATOM   1834  CD  ILE P 115       6.387  -8.814  -1.525  0.00  0.00      PROA
ATOM   1835  HD1 ILE P 115       5.834  -9.021  -2.466  0.00  0.00      PROA
ATOM   1836  HD2 ILE P 115       7.405  -9.240  -1.652  0.00  0.00      PROA
ATOM   1837  HD3 ILE P 115       6.534  -7.714  -1.468  0.00  0.00      PROA
ATOM   1838  C   ILE P 115       5.571  -6.787   1.335  0.00  0.00      PROA
ATOM   1839  O   ILE P 115       5.429  -6.148   0.300  0.00  0.00      PROA
ATOM   1840  N   TYR P 116       4.729  -6.521   2.352  0.00  0.00      PROA
ATOM   1841  HN  TYR P 116       4.708  -7.090   3.170  0.00  0.00      PROA
ATOM   1842  CA  TYR P 116       3.685  -5.455   2.404  0.00  0.00      PROA
ATOM   1843  HA  TYR P 116       2.962  -5.783   1.672  0.00  0.00      PROA
ATOM   1844  CB  TYR P 116       2.881  -5.456   3.783  0.00  0.00      PROA
ATOM   1845  HB1 TYR P 116       3.675  -5.378   4.556  0.00  0.00      PROA
ATOM   1846  HB2 TYR P 116       2.383  -6.444   3.881  0.00  0.00      PROA
ATOM   1847  CG  TYR P 116       1.850  -4.388   3.889  0.00  0.00      PROA
ATOM   1848  CD1 TYR P 116       0.923  -4.217   2.856  0.00  0.00      PROA
ATOM   1849  HD1 TYR P 116       0.976  -4.809   1.955  0.00  0.00      PROA
ATOM   1850  CE1 TYR P 116      -0.156  -3.350   2.993  0.00  0.00      PROA
ATOM   1851  HE1 TYR P 116      -0.856  -3.234   2.178  0.00  0.00      PROA
ATOM   1852  CZ  TYR P 116      -0.238  -2.601   4.179  0.00  0.00      PROA
ATOM   1853  OH  TYR P 116      -1.226  -1.692   4.365  0.00  0.00      PROA
ATOM   1854  HH  TYR P 116      -0.989  -1.276   5.197  0.00  0.00      PROA
ATOM   1855  CD2 TYR P 116       1.667  -3.661   5.053  0.00  0.00      PROA
ATOM   1856  HD2 TYR P 116       2.383  -3.804   5.849  0.00  0.00      PROA
ATOM   1857  CE2 TYR P 116       0.619  -2.848   5.274  0.00  0.00      PROA
ATOM   1858  HE2 TYR P 116       0.604  -2.290   6.199  0.00  0.00      PROA
ATOM   1859  C   TYR P 116       4.189  -4.087   2.078  0.00  0.00      PROA
ATOM   1860  O   TYR P 116       3.687  -3.304   1.217  0.00  0.00      PROA
ATOM   1861  N   THR P 117       5.367  -3.773   2.706  0.00  0.00      PROA
ATOM   1862  HN  THR P 117       5.734  -4.349   3.432  0.00  0.00      PROA
ATOM   1863  CA  THR P 117       6.161  -2.606   2.159  0.00  0.00      PROA
ATOM   1864  HA  THR P 117       5.529  -1.734   2.248  0.00  0.00      PROA
ATOM   1865  CB  THR P 117       7.401  -2.369   3.059  0.00  0.00      PROA
ATOM   1866  HB  THR P 117       7.781  -3.386   3.293  0.00  0.00      PROA
ATOM   1867  OG1 THR P 117       7.042  -1.640   4.243  0.00  0.00      PROA
ATOM   1868  HG1 THR P 117       7.589  -2.046   4.919  0.00  0.00      PROA
ATOM   1869  CG2 THR P 117       8.596  -1.577   2.491  0.00  0.00      PROA
ATOM   1870 HG21 THR P 117       8.185  -0.584   2.207  0.00  0.00      PROA
ATOM   1871 HG22 THR P 117       9.050  -2.030   1.584  0.00  0.00      PROA
ATOM   1872 HG23 THR P 117       9.391  -1.325   3.225  0.00  0.00      PROA
ATOM   1873  C   THR P 117       6.616  -2.584   0.710  0.00  0.00      PROA
ATOM   1874  O   THR P 117       6.529  -1.608  -0.009  0.00  0.00      PROA
ATOM   1875  N   VAL P 118       7.143  -3.728   0.184  0.00  0.00      PROA
ATOM   1876  HN  VAL P 118       7.239  -4.493   0.815  0.00  0.00      PROA
ATOM   1877  CA  VAL P 118       7.558  -3.847  -1.226  0.00  0.00      PROA
ATOM   1878  HA  VAL P 118       8.316  -3.146  -1.541  0.00  0.00      PROA
ATOM   1879  CB  VAL P 118       8.279  -5.161  -1.540  0.00  0.00      PROA
ATOM   1880  HB  VAL P 118       7.599  -5.982  -1.226  0.00  0.00      PROA
ATOM   1881  CG1 VAL P 118       8.473  -5.418  -3.104  0.00  0.00      PROA
ATOM   1882 HG11 VAL P 118       8.945  -6.416  -3.222  0.00  0.00      PROA
ATOM   1883 HG12 VAL P 118       9.108  -4.634  -3.570  0.00  0.00      PROA
ATOM   1884 HG13 VAL P 118       7.516  -5.303  -3.656  0.00  0.00      PROA
ATOM   1885  CG2 VAL P 118       9.664  -5.054  -0.941  0.00  0.00      PROA
ATOM   1886 HG21 VAL P 118      10.172  -6.026  -1.118  0.00  0.00      PROA
ATOM   1887 HG22 VAL P 118       9.655  -4.818   0.145  0.00  0.00      PROA
```

ATOM	1888	HG23	VAL	P	118	10.249	-4.292	-1.500	0.00	0.00	PROA
ATOM	1889	C	VAL	P	118	6.333	-3.704	-2.139	0.00	0.00	PROA
ATOM	1890	O	VAL	P	118	6.396	-3.019	-3.153	0.00	0.00	PROA
ATOM	1891	N	ASN	P	119	5.193	-4.285	-1.713	0.00	0.00	PROA
ATOM	1892	HN	ASN	P	119	5.209	-4.845	-0.888	0.00	0.00	PROA
ATOM	1893	CA	ASN	P	119	3.972	-4.337	-2.454	0.00	0.00	PROA
ATOM	1894	HA	ASN	P	119	4.257	-4.650	-3.448	0.00	0.00	PROA
ATOM	1895	CB	ASN	P	119	3.241	-5.552	-1.865	0.00	0.00	PROA
ATOM	1896	HB1	ASN	P	119	3.083	-5.218	-0.817	0.00	0.00	PROA
ATOM	1897	HB2	ASN	P	119	3.898	-6.427	-1.674	0.00	0.00	PROA
ATOM	1898	CG	ASN	P	119	1.906	-5.864	-2.593	0.00	0.00	PROA
ATOM	1899	OD1	ASN	P	119	0.787	-5.526	-2.263	0.00	0.00	PROA
ATOM	1900	ND2	ASN	P	119	2.130	-6.669	-3.688	0.00	0.00	PROA
ATOM	1901	HD21	ASN	P	119	1.341	-7.088	-4.137	0.00	0.00	PROA
ATOM	1902	HD22	ASN	P	119	3.067	-6.837	-3.994	0.00	0.00	PROA
ATOM	1903	C	ASN	P	119	3.257	-3.009	-2.520	0.00	0.00	PROA
ATOM	1904	O	ASN	P	119	2.720	-2.628	-3.534	0.00	0.00	PROA
ATOM	1905	N	LEU	P	120	3.296	-2.171	-1.475	0.00	0.00	PROA
ATOM	1906	HN	LEU	P	120	3.676	-2.551	-0.635	0.00	0.00	PROA
ATOM	1907	CA	LEU	P	120	2.701	-0.852	-1.418	0.00	0.00	PROA
ATOM	1908	HA	LEU	P	120	1.732	-1.017	-1.866	0.00	0.00	PROA
ATOM	1909	CB	LEU	P	120	2.730	-0.237	0.094	0.00	0.00	PROA
ATOM	1910	HB1	LEU	P	120	3.761	-0.146	0.498	0.00	0.00	PROA
ATOM	1911	HB2	LEU	P	120	2.347	-1.037	0.763	0.00	0.00	PROA
ATOM	1912	CG	LEU	P	120	2.014	1.138	0.299	0.00	0.00	PROA
ATOM	1913	HG	LEU	P	120	2.507	1.953	-0.274	0.00	0.00	PROA
ATOM	1914	CD1	LEU	P	120	0.600	1.037	-0.135	0.00	0.00	PROA
ATOM	1915	HD11	LEU	P	120	0.497	0.915	-1.235	0.00	0.00	PROA
ATOM	1916	HD12	LEU	P	120	0.075	2.008	-0.015	0.00	0.00	PROA
ATOM	1917	HD13	LEU	P	120	0.117	0.152	0.333	0.00	0.00	PROA
ATOM	1918	CD2	LEU	P	120	1.766	1.539	1.771	0.00	0.00	PROA
ATOM	1919	HD21	LEU	P	120	2.786	1.542	2.211	0.00	0.00	PROA
ATOM	1920	HD22	LEU	P	120	1.208	0.738	2.301	0.00	0.00	PROA
ATOM	1921	HD23	LEU	P	120	1.246	2.520	1.754	0.00	0.00	PROA
ATOM	1922	C	LEU	P	120	3.363	0.150	-2.407	0.00	0.00	PROA
ATOM	1923	O	LEU	P	120	2.696	0.774	-3.168	0.00	0.00	PROA
ATOM	1924	N	TYR	P	121	4.698	0.136	-2.451	0.00	0.00	PROA
ATOM	1925	HN	TYR	P	121	5.238	-0.282	-1.724	0.00	0.00	PROA
ATOM	1926	CA	TYR	P	121	5.266	1.162	-3.289	0.00	0.00	PROA
ATOM	1927	HA	TYR	P	121	4.665	2.058	-3.333	0.00	0.00	PROA
ATOM	1928	CB	TYR	P	121	6.670	1.476	-2.720	0.00	0.00	PROA
ATOM	1929	HB1	TYR	P	121	7.168	2.282	-3.300	0.00	0.00	PROA
ATOM	1930	HB2	TYR	P	121	7.256	0.535	-2.793	0.00	0.00	PROA
ATOM	1931	CG	TYR	P	121	6.625	2.060	-1.292	0.00	0.00	PROA
ATOM	1932	CD1	TYR	P	121	5.641	3.091	-1.010	0.00	0.00	PROA
ATOM	1933	HD1	TYR	P	121	5.261	3.693	-1.822	0.00	0.00	PROA
ATOM	1934	CE1	TYR	P	121	5.380	3.450	0.343	0.00	0.00	PROA
ATOM	1935	HE1	TYR	P	121	4.689	4.242	0.589	0.00	0.00	PROA
ATOM	1936	CZ	TYR	P	121	5.918	2.656	1.342	0.00	0.00	PROA
ATOM	1937	OH	TYR	P	121	5.627	2.884	2.699	0.00	0.00	PROA
ATOM	1938	HH	TYR	P	121	6.058	2.156	3.153	0.00	0.00	PROA
ATOM	1939	CD2	TYR	P	121	7.249	1.376	-0.229	0.00	0.00	PROA
ATOM	1940	HD2	TYR	P	121	7.879	0.565	-0.563	0.00	0.00	PROA
ATOM	1941	CE2	TYR	P	121	6.862	1.648	1.078	0.00	0.00	PROA
ATOM	1942	HE2	TYR	P	121	7.263	1.093	1.913	0.00	0.00	PROA
ATOM	1943	C	TYR	P	121	5.411	0.565	-4.676	0.00	0.00	PROA
ATOM	1944	O	TYR	P	121	5.270	1.385	-5.627	0.00	0.00	PROA
ATOM	1945	N	SER	P	122	5.664	-0.674	-4.939	0.00	0.00	PROA
ATOM	1946	HN	SER	P	122	5.974	-1.324	-4.248	0.00	0.00	PROA
ATOM	1947	CA	SER	P	122	5.601	-1.231	-6.295	0.00	0.00	PROA
ATOM	1948	HA	SER	P	122	6.162	-0.549	-6.917	0.00	0.00	PROA

ATOM 1949 CB SER P 122 6.201 -2.606 -6.380 0.00 0.00 PROA
ATOM 1950 HB1 SER P 122 7.279 -2.550 -6.116 0.00 0.00 PROA
ATOM 1951 HB2 SER P 122 6.123 -2.918 -7.444 0.00 0.00 PROA
ATOM 1952 OG SER P 122 5.444 -3.557 -5.610 0.00 0.00 PROA
ATOM 1953 HG1 SER P 122 5.470 -3.309 -4.683 0.00 0.00 PROA
ATOM 1954 C SER P 122 4.233 -1.197 -6.999 0.00 0.00 PROA
ATOM 1955 O SER P 122 4.184 -0.935 -8.159 0.00 0.00 PROA
ATOM 1956 N SER P 123 3.084 -1.310 -6.236 0.00 0.00 PROA
ATOM 1957 HN SER P 123 3.120 -1.617 -5.288 0.00 0.00 PROA
ATOM 1958 CA SER P 123 1.744 -1.273 -6.904 0.00 0.00 PROA
ATOM 1959 HA SER P 123 1.715 -1.843 -7.820 0.00 0.00 PROA
ATOM 1960 CB SER P 123 0.760 -1.728 -5.857 0.00 0.00 PROA
ATOM 1961 HB1 SER P 123 0.818 -1.215 -4.873 0.00 0.00 PROA
ATOM 1962 HB2 SER P 123 0.979 -2.772 -5.549 0.00 0.00 PROA
ATOM 1963 OG SER P 123 -0.585 -1.580 -6.093 0.00 0.00 PROA
ATOM 1964 HG1 SER P 123 -0.969 -1.981 -5.310 0.00 0.00 PROA
ATOM 1965 C SER P 123 1.478 0.140 -7.249 0.00 0.00 PROA
ATOM 1966 O SER P 123 0.882 0.409 -8.297 0.00 0.00 PROA
ATOM 1967 N VAL P 124 1.752 1.125 -6.429 0.00 0.00 PROA
ATOM 1968 HN VAL P 124 1.905 0.812 -5.495 0.00 0.00 PROA
ATOM 1969 CA VAL P 124 1.584 2.508 -6.794 0.00 0.00 PROA
ATOM 1970 HA VAL P 124 0.598 2.631 -7.216 0.00 0.00 PROA
ATOM 1971 CB VAL P 124 1.619 3.358 -5.513 0.00 0.00 PROA
ATOM 1972 HB VAL P 124 2.500 3.109 -4.885 0.00 0.00 PROA
ATOM 1973 CG1 VAL P 124 1.566 4.881 -5.793 0.00 0.00 PROA
ATOM 1974 HG11 VAL P 124 0.900 4.998 -6.675 0.00 0.00 PROA
ATOM 1975 HG12 VAL P 124 2.569 5.273 -6.065 0.00 0.00 PROA
ATOM 1976 HG13 VAL P 124 1.206 5.471 -4.923 0.00 0.00 PROA
ATOM 1977 CG2 VAL P 124 0.412 2.976 -4.639 0.00 0.00 PROA
ATOM 1978 HG21 VAL P 124 0.389 1.908 -4.336 0.00 0.00 PROA
ATOM 1979 HG22 VAL P 124 -0.526 3.156 -5.207 0.00 0.00 PROA
ATOM 1980 HG23 VAL P 124 0.442 3.610 -3.727 0.00 0.00 PROA
ATOM 1981 C VAL P 124 2.548 3.007 -7.855 0.00 0.00 PROA
ATOM 1982 O VAL P 124 2.174 3.742 -8.752 0.00 0.00 PROA
ATOM 1983 N TRP P 125 3.862 2.570 -7.789 0.00 0.00 PROA
ATOM 1984 HN TRP P 125 4.223 2.007 -7.049 0.00 0.00 PROA
ATOM 1985 CA TRP P 125 4.696 2.747 -8.884 0.00 0.00 PROA
ATOM 1986 HA TRP P 125 4.803 3.813 -9.016 0.00 0.00 PROA
ATOM 1987 CB TRP P 125 6.106 2.255 -8.584 0.00 0.00 PROA
ATOM 1988 HB1 TRP P 125 6.533 2.058 -9.590 0.00 0.00 PROA
ATOM 1989 HB2 TRP P 125 6.093 1.331 -7.967 0.00 0.00 PROA
ATOM 1990 CG TRP P 125 7.064 3.226 -7.948 0.00 0.00 PROA
ATOM 1991 CD1 TRP P 125 7.690 3.031 -6.725 0.00 0.00 PROA
ATOM 1992 HD1 TRP P 125 7.611 2.117 -6.155 0.00 0.00 PROA
ATOM 1993 NE1 TRP P 125 8.231 4.231 -6.258 0.00 0.00 PROA
ATOM 1994 HE1 TRP P 125 8.412 4.540 -5.350 0.00 0.00 PROA
ATOM 1995 CE2 TRP P 125 7.964 5.194 -7.228 0.00 0.00 PROA
ATOM 1996 CD2 TRP P 125 7.243 4.580 -8.253 0.00 0.00 PROA
ATOM 1997 CE3 TRP P 125 6.978 5.395 -9.425 0.00 0.00 PROA
ATOM 1998 HE3 TRP P 125 6.524 5.073 -10.351 0.00 0.00 PROA
ATOM 1999 CZ3 TRP P 125 7.272 6.743 -9.371 0.00 0.00 PROA
ATOM 2000 HZ3 TRP P 125 7.155 7.274 -10.304 0.00 0.00 PROA
ATOM 2001 CZ2 TRP P 125 8.259 6.488 -7.178 0.00 0.00 PROA
ATOM 2002 HZ2 TRP P 125 8.666 7.014 -6.327 0.00 0.00 PROA
ATOM 2003 CH2 TRP P 125 7.906 7.288 -8.270 0.00 0.00 PROA
ATOM 2004 HH2 TRP P 125 8.111 8.346 -8.194 0.00 0.00 PROA
ATOM 2005 C TRP P 125 4.165 2.131 -10.168 0.00 0.00 PROA
ATOM 2006 O TRP P 125 4.349 2.698 -11.252 0.00 0.00 PROA
ATOM 2007 N ILE P 126 3.447 1.020 -10.255 0.00 0.00 PROA
ATOM 2008 HN ILE P 126 3.302 0.446 -9.453 0.00 0.00 PROA
ATOM 2009 CA ILE P 126 2.823 0.512 -11.457 0.00 0.00 PROA

ATOM	2010	HA	ILE	P	126	3.661	0.331	-12.114	0.00	0.00	PROA
ATOM	2011	CB	ILE	P	126	2.177	-0.865	-11.114	0.00	0.00	PROA
ATOM	2012	HB	ILE	P	126	1.739	-0.858	-10.093	0.00	0.00	PROA
ATOM	2013	CG2	ILE	P	126	1.048	-1.221	-12.063	0.00	0.00	PROA
ATOM	2014	HG21	ILE	P	126	1.467	-1.123	-13.088	0.00	0.00	PROA
ATOM	2015	HG22	ILE	P	126	0.147	-0.582	-11.943	0.00	0.00	PROA
ATOM	2016	HG23	ILE	P	126	0.652	-2.248	-11.914	0.00	0.00	PROA
ATOM	2017	CG1	ILE	P	126	3.191	-1.980	-11.152	0.00	0.00	PROA
ATOM	2018	HG11	ILE	P	126	2.664	-2.933	-10.933	0.00	0.00	PROA
ATOM	2019	HG12	ILE	P	126	3.962	-1.832	-10.366	0.00	0.00	PROA
ATOM	2020	CD	ILE	P	126	4.015	-2.246	-12.377	0.00	0.00	PROA
ATOM	2021	HD1	ILE	P	126	4.508	-3.240	-12.327	0.00	0.00	PROA
ATOM	2022	HD2	ILE	P	126	4.869	-1.545	-12.489	0.00	0.00	PROA
ATOM	2023	HD3	ILE	P	126	3.444	-2.272	-13.331	0.00	0.00	PROA
ATOM	2024	C	ILE	P	126	1.834	1.470	-12.095	0.00	0.00	PROA
ATOM	2025	O	ILE	P	126	1.794	1.695	-13.267	0.00	0.00	PROA
ATOM	2026	N	LEU	P	127	1.078	2.232	-11.242	0.00	0.00	PROA
ATOM	2027	HN	LEU	P	127	1.166	2.165	-10.252	0.00	0.00	PROA
ATOM	2028	CA	LEU	P	127	0.040	3.234	-11.772	0.00	0.00	PROA
ATOM	2029	HA	LEU	P	127	-0.545	2.829	-12.584	0.00	0.00	PROA
ATOM	2030	CB	LEU	P	127	-0.695	3.876	-10.542	0.00	0.00	PROA
ATOM	2031	HB1	LEU	P	127	-1.384	4.675	-10.892	0.00	0.00	PROA
ATOM	2032	HB2	LEU	P	127	0.166	4.273	-9.963	0.00	0.00	PROA
ATOM	2033	CG	LEU	P	127	-1.510	2.874	-9.668	0.00	0.00	PROA
ATOM	2034	HG	LEU	P	127	-0.853	2.317	-8.966	0.00	0.00	PROA
ATOM	2035	CD1	LEU	P	127	-2.301	3.679	-8.690	0.00	0.00	PROA
ATOM	2036	HD11	LEU	P	127	-1.586	4.240	-8.051	0.00	0.00	PROA
ATOM	2037	HD12	LEU	P	127	-2.821	3.056	-7.931	0.00	0.00	PROA
ATOM	2038	HD13	LEU	P	127	-3.041	4.385	-9.123	0.00	0.00	PROA
ATOM	2039	CD2	LEU	P	127	-2.400	1.841	-10.440	0.00	0.00	PROA
ATOM	2040	HD21	LEU	P	127	-3.039	2.313	-11.216	0.00	0.00	PROA
ATOM	2041	HD22	LEU	P	127	-3.017	1.420	-9.617	0.00	0.00	PROA
ATOM	2042	HD23	LEU	P	127	-1.824	1.060	-10.982	0.00	0.00	PROA
ATOM	2043	C	LEU	P	127	0.734	4.361	-12.472	0.00	0.00	PROA
ATOM	2044	O	LEU	P	127	0.277	4.934	-13.466	0.00	0.00	PROA
ATOM	2045	N	ALA	P	128	1.923	4.692	-11.921	0.00	0.00	PROA
ATOM	2046	HN	ALA	P	128	2.120	4.233	-11.059	0.00	0.00	PROA
ATOM	2047	CA	ALA	P	128	2.803	5.628	-12.412	0.00	0.00	PROA
ATOM	2048	HA	ALA	P	128	2.223	6.524	-12.577	0.00	0.00	PROA
ATOM	2049	CB	ALA	P	128	3.834	6.018	-11.316	0.00	0.00	PROA
ATOM	2050	HB1	ALA	P	128	3.231	6.203	-10.401	0.00	0.00	PROA
ATOM	2051	HB2	ALA	P	128	4.366	6.977	-11.496	0.00	0.00	PROA
ATOM	2052	HB3	ALA	P	128	4.663	5.280	-11.270	0.00	0.00	PROA
ATOM	2053	C	ALA	P	128	3.481	5.284	-13.767	0.00	0.00	PROA
ATOM	2054	O	ALA	P	128	3.454	6.111	-14.703	0.00	0.00	PROA
ATOM	2055	N	PHE	P	129	3.963	4.102	-13.966	0.00	0.00	PROA
ATOM	2056	HN	PHE	P	129	3.865	3.467	-13.203	0.00	0.00	PROA
ATOM	2057	CA	PHE	P	129	4.245	3.539	-15.269	0.00	0.00	PROA
ATOM	2058	HA	PHE	P	129	4.825	4.208	-15.887	0.00	0.00	PROA
ATOM	2059	CB	PHE	P	129	4.879	2.096	-15.172	0.00	0.00	PROA
ATOM	2060	HB1	PHE	P	129	4.733	1.454	-16.067	0.00	0.00	PROA
ATOM	2061	HB2	PHE	P	129	4.606	1.459	-14.304	0.00	0.00	PROA
ATOM	2062	CG	PHE	P	129	6.336	2.378	-15.014	0.00	0.00	PROA
ATOM	2063	CD1	PHE	P	129	7.202	2.393	-16.089	0.00	0.00	PROA
ATOM	2064	HD1	PHE	P	129	6.962	2.088	-17.097	0.00	0.00	PROA
ATOM	2065	CE1	PHE	P	129	8.554	2.776	-15.946	0.00	0.00	PROA
ATOM	2066	HE1	PHE	P	129	9.209	2.712	-16.802	0.00	0.00	PROA
ATOM	2067	CZ	PHE	P	129	9.072	3.192	-14.725	0.00	0.00	PROA
ATOM	2068	HZ	PHE	P	129	10.061	3.612	-14.612	0.00	0.00	PROA
ATOM	2069	CD2	PHE	P	129	6.904	2.790	-13.771	0.00	0.00	PROA
ATOM	2070	HD2	PHE	P	129	6.246	2.868	-12.918	0.00	0.00	PROA

ATOM	2071	CE2	PHE	P	129	8.161	3.406	-13.650	0.00	0.00	PROA
ATOM	2072	HE2	PHE	P	129	8.436	3.863	-12.711	0.00	0.00	PROA
ATOM	2073	C	PHE	P	129	3.100	3.431	-16.292	0.00	0.00	PROA
ATOM	2074	O	PHE	P	129	3.324	3.661	-17.478	0.00	0.00	PROA
ATOM	2075	N	ILE	P	130	1.903	3.030	-15.842	0.00	0.00	PROA
ATOM	2076	HN	ILE	P	130	1.763	2.790	-14.884	0.00	0.00	PROA
ATOM	2077	CA	ILE	P	130	0.799	2.989	-16.861	0.00	0.00	PROA
ATOM	2078	HA	ILE	P	130	0.993	2.249	-17.623	0.00	0.00	PROA
ATOM	2079	CB	ILE	P	130	-0.589	2.440	-16.370	0.00	0.00	PROA
ATOM	2080	HB	ILE	P	130	-0.912	2.934	-15.429	0.00	0.00	PROA
ATOM	2081	CG2	ILE	P	130	-1.762	2.805	-17.348	0.00	0.00	PROA
ATOM	2082	HG21	ILE	P	130	-2.776	2.360	-17.262	0.00	0.00	PROA
ATOM	2083	HG22	ILE	P	130	-1.505	2.478	-18.379	0.00	0.00	PROA
ATOM	2084	HG23	ILE	P	130	-1.822	3.912	-17.418	0.00	0.00	PROA
ATOM	2085	CG1	ILE	P	130	-0.607	0.937	-16.189	0.00	0.00	PROA
ATOM	2086	HG11	ILE	P	130	0.362	0.723	-15.690	0.00	0.00	PROA
ATOM	2087	HG12	ILE	P	130	-0.577	0.434	-17.179	0.00	0.00	PROA
ATOM	2088	CD	ILE	P	130	-1.778	0.487	-15.378	0.00	0.00	PROA
ATOM	2089	HD1	ILE	P	130	-1.626	-0.596	-15.179	0.00	0.00	PROA
ATOM	2090	HD2	ILE	P	130	-2.804	0.736	-15.725	0.00	0.00	PROA
ATOM	2091	HD3	ILE	P	130	-1.704	0.967	-14.379	0.00	0.00	PROA
ATOM	2092	C	ILE	P	130	0.548	4.370	-17.463	0.00	0.00	PROA
ATOM	2093	O	ILE	P	130	0.329	4.459	-18.668	0.00	0.00	PROA
ATOM	2094	N	SER	P	131	0.536	5.417	-16.570	0.00	0.00	PROA
ATOM	2095	HN	SER	P	131	0.466	5.229	-15.593	0.00	0.00	PROA
ATOM	2096	CA	SER	P	131	0.383	6.883	-16.921	0.00	0.00	PROA
ATOM	2097	HA	SER	P	131	-0.516	7.043	-17.498	0.00	0.00	PROA
ATOM	2098	CB	SER	P	131	0.316	7.746	-15.620	0.00	0.00	PROA
ATOM	2099	HB1	SER	P	131	0.303	8.819	-15.906	0.00	0.00	PROA
ATOM	2100	HB2	SER	P	131	1.275	7.557	-15.093	0.00	0.00	PROA
ATOM	2101	OG	SER	P	131	-0.802	7.405	-14.818	0.00	0.00	PROA
ATOM	2102	HG1	SER	P	131	-0.533	6.730	-14.189	0.00	0.00	PROA
ATOM	2103	C	SER	P	131	1.406	7.391	-17.883	0.00	0.00	PROA
ATOM	2104	O	SER	P	131	1.102	8.246	-18.739	0.00	0.00	PROA
ATOM	2105	N	LEU	P	132	2.714	6.990	-17.684	0.00	0.00	PROA
ATOM	2106	HN	LEU	P	132	2.840	6.474	-16.840	0.00	0.00	PROA
ATOM	2107	CA	LEU	P	132	3.796	7.112	-18.673	0.00	0.00	PROA
ATOM	2108	HA	LEU	P	132	3.866	8.165	-18.903	0.00	0.00	PROA
ATOM	2109	CB	LEU	P	132	5.156	6.595	-18.154	0.00	0.00	PROA
ATOM	2110	HB1	LEU	P	132	5.898	6.370	-18.950	0.00	0.00	PROA
ATOM	2111	HB2	LEU	P	132	4.972	5.693	-17.532	0.00	0.00	PROA
ATOM	2112	CG	LEU	P	132	5.960	7.558	-17.303	0.00	0.00	PROA
ATOM	2113	HG	LEU	P	132	5.253	7.803	-16.481	0.00	0.00	PROA
ATOM	2114	CD1	LEU	P	132	7.177	6.875	-16.663	0.00	0.00	PROA
ATOM	2115	HD11	LEU	P	132	7.752	7.572	-16.017	0.00	0.00	PROA
ATOM	2116	HD12	LEU	P	132	7.866	6.409	-17.400	0.00	0.00	PROA
ATOM	2117	HD13	LEU	P	132	6.767	6.093	-15.989	0.00	0.00	PROA
ATOM	2118	CD2	LEU	P	132	6.381	8.813	-18.063	0.00	0.00	PROA
ATOM	2119	HD21	LEU	P	132	5.498	9.486	-18.072	0.00	0.00	PROA
ATOM	2120	HD22	LEU	P	132	6.794	8.541	-19.058	0.00	0.00	PROA
ATOM	2121	HD23	LEU	P	132	7.185	9.263	-17.442	0.00	0.00	PROA
ATOM	2122	C	LEU	P	132	3.489	6.494	-19.995	0.00	0.00	PROA
ATOM	2123	O	LEU	P	132	3.572	7.076	-21.090	0.00	0.00	PROA
ATOM	2124	N	ASP	P	133	3.114	5.183	-19.958	0.00	0.00	PROA
ATOM	2125	HN	ASP	P	133	3.061	4.752	-19.060	0.00	0.00	PROA
ATOM	2126	CA	ASP	P	133	2.754	4.362	-21.055	0.00	0.00	PROA
ATOM	2127	HA	ASP	P	133	3.548	4.316	-21.786	0.00	0.00	PROA
ATOM	2128	CB	ASP	P	133	2.580	2.859	-20.510	0.00	0.00	PROA
ATOM	2129	HB1	ASP	P	133	1.948	2.832	-19.596	0.00	0.00	PROA
ATOM	2130	HB2	ASP	P	133	3.644	2.588	-20.338	0.00	0.00	PROA
ATOM	2131	CG	ASP	P	133	1.984	2.001	-21.568	0.00	0.00	PROA

```
ATOM   2132  OD1 ASP P 133       0.790   2.112 -21.916  0.00  0.00      PROA
ATOM   2133  OD2 ASP P 133       2.629   1.052 -21.985  0.00  0.00      PROA
ATOM   2134  C   ASP P 133       1.578   4.939 -21.864  0.00  0.00      PROA
ATOM   2135  O   ASP P 133       1.677   5.017 -23.054  0.00  0.00      PROA
ATOM   2136  N   ARG P 134       0.522   5.351 -21.207  0.00  0.00      PROA
ATOM   2137  HN  ARG P 134       0.334   5.037 -20.279  0.00  0.00      PROA
ATOM   2138  CA  ARG P 134      -0.494   6.097 -21.814  0.00  0.00      PROA
ATOM   2139  HA  ARG P 134      -0.862   5.417 -22.569  0.00  0.00      PROA
ATOM   2140  CB  ARG P 134      -1.659   6.129 -20.807  0.00  0.00      PROA
ATOM   2141  HB1 ARG P 134      -2.509   6.665 -21.281  0.00  0.00      PROA
ATOM   2142  HB2 ARG P 134      -1.311   6.817 -20.008  0.00  0.00      PROA
ATOM   2143  CG  ARG P 134      -2.217   4.801 -20.456  0.00  0.00      PROA
ATOM   2144  HG1 ARG P 134      -3.080   4.917 -19.766  0.00  0.00      PROA
ATOM   2145  HG2 ARG P 134      -1.460   4.172 -19.941  0.00  0.00      PROA
ATOM   2146  CD  ARG P 134      -2.766   3.948 -21.658  0.00  0.00      PROA
ATOM   2147  HD1 ARG P 134      -3.169   4.673 -22.398  0.00  0.00      PROA
ATOM   2148  HD2 ARG P 134      -3.534   3.219 -21.322  0.00  0.00      PROA
ATOM   2149  NE  ARG P 134      -1.671   3.266 -22.356  0.00  0.00      PROA
ATOM   2150  HE  ARG P 134      -0.804   2.961 -21.961  0.00  0.00      PROA
ATOM   2151  CZ  ARG P 134      -1.769   2.665 -23.534  0.00  0.00      PROA
ATOM   2152  NH1 ARG P 134      -2.717   2.944 -24.338  0.00  0.00      PROA
ATOM   2153 HH11 ARG P 134      -3.536   3.299 -23.887  0.00  0.00      PROA
ATOM   2154 HH12 ARG P 134      -2.907   2.215 -24.995  0.00  0.00      PROA
ATOM   2155  NH2 ARG P 134      -0.772   1.920 -23.973  0.00  0.00      PROA
ATOM   2156 HH21 ARG P 134      -0.039   1.939 -23.293  0.00  0.00      PROA
ATOM   2157 HH22 ARG P 134      -0.692   1.598 -24.917  0.00  0.00      PROA
ATOM   2158  C   ARG P 134      -0.034   7.493 -22.279  0.00  0.00      PROA
ATOM   2159  O   ARG P 134      -0.430   7.908 -23.384  0.00  0.00      PROA
ATOM   2160  N   TYR P 135       0.822   8.329 -21.610  0.00  0.00      PROA
ATOM   2161  HN  TYR P 135       1.164   8.067 -20.711  0.00  0.00      PROA
ATOM   2162  CA  TYR P 135       1.406   9.474 -22.304  0.00  0.00      PROA
ATOM   2163  HA  TYR P 135       0.612  10.190 -22.458  0.00  0.00      PROA
ATOM   2164  CB  TYR P 135       2.451  10.125 -21.291  0.00  0.00      PROA
ATOM   2165  HB1 TYR P 135       2.690   9.365 -20.517  0.00  0.00      PROA
ATOM   2166  HB2 TYR P 135       1.969  10.996 -20.798  0.00  0.00      PROA
ATOM   2167  CG  TYR P 135       3.805  10.648 -21.872  0.00  0.00      PROA
ATOM   2168  CD1 TYR P 135       4.984  10.145 -21.408  0.00  0.00      PROA
ATOM   2169  HD1 TYR P 135       5.065   9.330 -20.704  0.00  0.00      PROA
ATOM   2170  CE1 TYR P 135       6.170  10.707 -21.837  0.00  0.00      PROA
ATOM   2171  HE1 TYR P 135       7.135  10.370 -21.488  0.00  0.00      PROA
ATOM   2172  CZ  TYR P 135       6.196  11.885 -22.584  0.00  0.00      PROA
ATOM   2173  OH  TYR P 135       7.419  12.533 -22.814  0.00  0.00      PROA
ATOM   2174  HH  TYR P 135       8.158  12.288 -22.252  0.00  0.00      PROA
ATOM   2175  CD2 TYR P 135       3.776  11.782 -22.626  0.00  0.00      PROA
ATOM   2176  HD2 TYR P 135       2.867  12.324 -22.839  0.00  0.00      PROA
ATOM   2177  CE2 TYR P 135       4.932  12.414 -23.099  0.00  0.00      PROA
ATOM   2178  HE2 TYR P 135       4.916  13.220 -23.816  0.00  0.00      PROA
ATOM   2179  C   TYR P 135       1.982   9.300 -23.629  0.00  0.00      PROA
ATOM   2180  O   TYR P 135       1.635   9.877 -24.659  0.00  0.00      PROA
ATOM   2181  N   LEU P 136       2.849   8.222 -23.684  0.00  0.00      PROA
ATOM   2182  HN  LEU P 136       2.973   7.827 -22.777  0.00  0.00      PROA
ATOM   2183  CA  LEU P 136       3.571   7.815 -24.823  0.00  0.00      PROA
ATOM   2184  HA  LEU P 136       4.012   8.693 -25.271  0.00  0.00      PROA
ATOM   2185  CB  LEU P 136       4.630   6.764 -24.591  0.00  0.00      PROA
ATOM   2186  HB1 LEU P 136       5.024   6.478 -25.590  0.00  0.00      PROA
ATOM   2187  HB2 LEU P 136       4.068   5.963 -24.065  0.00  0.00      PROA
ATOM   2188  CG  LEU P 136       5.969   7.159 -23.877  0.00  0.00      PROA
ATOM   2189  HG  LEU P 136       5.644   7.610 -22.915  0.00  0.00      PROA
ATOM   2190  CD1 LEU P 136       6.795   5.876 -23.655  0.00  0.00      PROA
ATOM   2191 HD11 LEU P 136       7.691   6.180 -23.073  0.00  0.00      PROA
ATOM   2192 HD12 LEU P 136       6.930   5.330 -24.614  0.00  0.00      PROA
```

ATOM	2193	HD13	LEU	P	136	6.263	5.161	-22.992	0.00	0.00	PROA
ATOM	2194	CD2	LEU	P	136	6.684	8.195	-24.842	0.00	0.00	PROA
ATOM	2195	HD21	LEU	P	136	6.070	9.119	-24.776	0.00	0.00	PROA
ATOM	2196	HD22	LEU	P	136	6.703	7.763	-25.866	0.00	0.00	PROA
ATOM	2197	HD23	LEU	P	136	7.749	8.301	-24.544	0.00	0.00	PROA
ATOM	2198	C	LEU	P	136	2.604	7.404	-25.939	0.00	0.00	PROA
ATOM	2199	O	LEU	P	136	2.704	7.782	-27.092	0.00	0.00	PROA
ATOM	2200	N	ALA	P	137	1.604	6.652	-25.526	0.00	0.00	PROA
ATOM	2201	HN	ALA	P	137	1.551	6.279	-24.603	0.00	0.00	PROA
ATOM	2202	CA	ALA	P	137	0.657	6.025	-26.449	0.00	0.00	PROA
ATOM	2203	HA	ALA	P	137	1.266	5.532	-27.193	0.00	0.00	PROA
ATOM	2204	CB	ALA	P	137	-0.293	5.061	-25.576	0.00	0.00	PROA
ATOM	2205	HB1	ALA	P	137	-0.731	5.613	-24.717	0.00	0.00	PROA
ATOM	2206	HB2	ALA	P	137	0.288	4.223	-25.135	0.00	0.00	PROA
ATOM	2207	HB3	ALA	P	137	-0.995	4.602	-26.305	0.00	0.00	PROA
ATOM	2208	C	ALA	P	137	-0.278	7.015	-27.084	0.00	0.00	PROA
ATOM	2209	O	ALA	P	137	-0.784	6.778	-28.200	0.00	0.00	PROA
ATOM	2210	N	ILE	P	138	-0.625	8.080	-26.377	0.00	0.00	PROA
ATOM	2211	HN	ILE	P	138	-0.117	8.252	-25.536	0.00	0.00	PROA
ATOM	2212	CA	ILE	P	138	-1.714	8.905	-26.803	0.00	0.00	PROA
ATOM	2213	HA	ILE	P	138	-2.394	8.353	-27.434	0.00	0.00	PROA
ATOM	2214	CB	ILE	P	138	-2.505	9.402	-25.544	0.00	0.00	PROA
ATOM	2215	HB	ILE	P	138	-1.864	9.630	-24.665	0.00	0.00	PROA
ATOM	2216	CG2	ILE	P	138	-3.543	10.528	-25.851	0.00	0.00	PROA
ATOM	2217	HG21	ILE	P	138	-4.035	10.830	-24.902	0.00	0.00	PROA
ATOM	2218	HG22	ILE	P	138	-4.325	10.073	-26.496	0.00	0.00	PROA
ATOM	2219	HG23	ILE	P	138	-3.142	11.438	-26.346	0.00	0.00	PROA
ATOM	2220	CG1	ILE	P	138	-3.262	8.186	-25.022	0.00	0.00	PROA
ATOM	2221	HG11	ILE	P	138	-2.510	7.377	-24.904	0.00	0.00	PROA
ATOM	2222	HG12	ILE	P	138	-3.967	7.794	-25.786	0.00	0.00	PROA
ATOM	2223	CD	ILE	P	138	-4.073	8.368	-23.744	0.00	0.00	PROA
ATOM	2224	HD1	ILE	P	138	-4.053	7.414	-23.174	0.00	0.00	PROA
ATOM	2225	HD2	ILE	P	138	-5.124	8.677	-23.931	0.00	0.00	PROA
ATOM	2226	HD3	ILE	P	138	-3.605	9.046	-22.999	0.00	0.00	PROA
ATOM	2227	C	ILE	P	138	-1.149	10.057	-27.539	0.00	0.00	PROA
ATOM	2228	O	ILE	P	138	-1.671	10.463	-28.567	0.00	0.00	PROA
ATOM	2229	N	VAL	P	139	0.013	10.652	-27.146	0.00	0.00	PROA
ATOM	2230	HN	VAL	P	139	0.512	10.348	-26.338	0.00	0.00	PROA
ATOM	2231	CA	VAL	P	139	0.551	11.848	-27.877	0.00	0.00	PROA
ATOM	2232	HA	VAL	P	139	-0.270	12.286	-28.425	0.00	0.00	PROA
ATOM	2233	CB	VAL	P	139	1.235	12.715	-26.865	0.00	0.00	PROA
ATOM	2234	HB	VAL	P	139	2.065	12.160	-26.377	0.00	0.00	PROA
ATOM	2235	CG1	VAL	P	139	1.889	13.974	-27.480	0.00	0.00	PROA
ATOM	2236	HG11	VAL	P	139	2.618	13.649	-28.252	0.00	0.00	PROA
ATOM	2237	HG12	VAL	P	139	2.520	14.384	-26.662	0.00	0.00	PROA
ATOM	2238	HG13	VAL	P	139	1.109	14.666	-27.864	0.00	0.00	PROA
ATOM	2239	CG2	VAL	P	139	0.208	13.110	-25.767	0.00	0.00	PROA
ATOM	2240	HG21	VAL	P	139	-0.028	12.186	-25.197	0.00	0.00	PROA
ATOM	2241	HG22	VAL	P	139	-0.694	13.413	-26.340	0.00	0.00	PROA
ATOM	2242	HG23	VAL	P	139	0.576	13.895	-25.073	0.00	0.00	PROA
ATOM	2243	C	VAL	P	139	1.514	11.482	-28.966	0.00	0.00	PROA
ATOM	2244	O	VAL	P	139	1.522	12.168	-30.028	0.00	0.00	PROA
ATOM	2245	N	HSD	P	140	2.380	10.444	-28.726	0.00	0.00	PROA
ATOM	2246	HN	HSD	P	140	2.276	10.035	-27.823	0.00	0.00	PROA
ATOM	2247	CA	HSD	P	140	3.634	10.326	-29.404	0.00	0.00	PROA
ATOM	2248	HA	HSD	P	140	3.688	11.047	-30.206	0.00	0.00	PROA
ATOM	2249	CB	HSD	P	140	4.865	10.433	-28.391	0.00	0.00	PROA
ATOM	2250	HB1	HSD	P	140	5.785	10.495	-29.010	0.00	0.00	PROA
ATOM	2251	HB2	HSD	P	140	4.955	9.580	-27.685	0.00	0.00	PROA
ATOM	2252	ND1	HSD	P	140	5.390	12.916	-28.350	0.00	0.00	PROA
ATOM	2253	HD1	HSD	P	140	5.537	13.060	-29.329	0.00	0.00	PROA

ATOM	2254	CG	HSD	P	140	4.961	11.730	-27.696	0.00	0.00	PROA
ATOM	2255	CE1	HSD	P	140	5.396	13.837	-27.349	0.00	0.00	PROA
ATOM	2256	HE1	HSD	P	140	5.326	14.907	-27.543	0.00	0.00	PROA
ATOM	2257	NE2	HSD	P	140	5.099	13.309	-26.158	0.00	0.00	PROA
ATOM	2258	CD2	HSD	P	140	4.850	11.994	-26.332	0.00	0.00	PROA
ATOM	2259	HD2	HSD	P	140	4.558	11.378	-25.490	0.00	0.00	PROA
ATOM	2260	C	HSD	P	140	3.705	9.066	-30.174	0.00	0.00	PROA
ATOM	2261	O	HSD	P	140	2.931	8.122	-29.961	0.00	0.00	PROA
ATOM	2262	N	ALA	P	141	4.697	8.925	-31.154	0.00	0.00	PROA
ATOM	2263	HN	ALA	P	141	5.198	9.775	-31.297	0.00	0.00	PROA
ATOM	2264	CA	ALA	P	141	5.260	7.674	-31.710	0.00	0.00	PROA
ATOM	2265	HA	ALA	P	141	6.123	7.858	-32.332	0.00	0.00	PROA
ATOM	2266	CB	ALA	P	141	5.710	6.714	-30.618	0.00	0.00	PROA
ATOM	2267	HB1	ALA	P	141	6.226	5.865	-31.115	0.00	0.00	PROA
ATOM	2268	HB2	ALA	P	141	4.999	6.355	-29.844	0.00	0.00	PROA
ATOM	2269	HB3	ALA	P	141	6.455	7.276	-30.015	0.00	0.00	PROA
ATOM	2270	C	ALA	P	141	4.247	6.999	-32.701	0.00	0.00	PROA
ATOM	2271	O	ALA	P	141	4.245	5.767	-32.786	0.00	0.00	PROA
ATOM	2272	N	THR	P	142	3.430	7.836	-33.466	0.00	0.00	PROA
ATOM	2273	HN	THR	P	142	3.408	8.815	-33.280	0.00	0.00	PROA
ATOM	2274	CA	THR	P	142	2.581	7.423	-34.560	0.00	0.00	PROA
ATOM	2275	HA	THR	P	142	1.783	6.844	-34.119	0.00	0.00	PROA
ATOM	2276	CB	THR	P	142	2.103	8.643	-35.351	0.00	0.00	PROA
ATOM	2277	HB	THR	P	142	2.896	9.421	-35.376	0.00	0.00	PROA
ATOM	2278	OG1	THR	P	142	0.957	9.266	-34.784	0.00	0.00	PROA
ATOM	2279	HG1	THR	P	142	1.346	9.474	-33.931	0.00	0.00	PROA
ATOM	2280	CG2	THR	P	142	1.611	8.235	-36.776	0.00	0.00	PROA
ATOM	2281	HG21	THR	P	142	2.427	7.934	-37.467	0.00	0.00	PROA
ATOM	2282	HG22	THR	P	142	1.103	9.109	-37.238	0.00	0.00	PROA
ATOM	2283	HG23	THR	P	142	0.873	7.405	-36.734	0.00	0.00	PROA
ATOM	2284	C	THR	P	142	3.218	6.303	-35.379	0.00	0.00	PROA
ATOM	2285	O	THR	P	142	4.365	6.443	-35.844	0.00	0.00	PROA
ATOM	2286	N	ASN	P	143	2.541	5.163	-35.508	0.00	0.00	PROA
ATOM	2287	HN	ASN	P	143	1.605	5.076	-35.174	0.00	0.00	PROA
ATOM	2288	CA	ASN	P	143	3.002	3.956	-36.271	0.00	0.00	PROA
ATOM	2289	HA	ASN	P	143	2.221	3.223	-36.134	0.00	0.00	PROA
ATOM	2290	CB	ASN	P	143	3.258	4.201	-37.764	0.00	0.00	PROA
ATOM	2291	HB1	ASN	P	143	3.465	3.212	-38.224	0.00	0.00	PROA
ATOM	2292	HB2	ASN	P	143	4.125	4.890	-37.861	0.00	0.00	PROA
ATOM	2293	CG	ASN	P	143	2.046	4.732	-38.393	0.00	0.00	PROA
ATOM	2294	OD1	ASN	P	143	2.167	5.705	-39.158	0.00	0.00	PROA
ATOM	2295	ND2	ASN	P	143	0.863	4.181	-38.116	0.00	0.00	PROA
ATOM	2296	HD21	ASN	P	143	0.032	4.621	-38.455	0.00	0.00	PROA
ATOM	2297	HD22	ASN	P	143	0.821	3.392	-37.503	0.00	0.00	PROA
ATOM	2298	C	ASN	P	143	4.266	3.279	-35.747	0.00	0.00	PROA
ATOM	2299	O	ASN	P	143	4.964	2.564	-36.423	0.00	0.00	PROA
ATOM	2300	N	SER	P	144	4.643	3.490	-34.425	0.00	0.00	PROA
ATOM	2301	HN	SER	P	144	4.213	4.255	-33.954	0.00	0.00	PROA
ATOM	2302	CA	SER	P	144	5.837	2.828	-33.838	0.00	0.00	PROA
ATOM	2303	HA	SER	P	144	5.981	1.853	-34.281	0.00	0.00	PROA
ATOM	2304	CB	SER	P	144	7.105	3.834	-33.967	0.00	0.00	PROA
ATOM	2305	HB1	SER	P	144	6.766	4.744	-33.427	0.00	0.00	PROA
ATOM	2306	HB2	SER	P	144	7.278	4.157	-35.016	0.00	0.00	PROA
ATOM	2307	OG	SER	P	144	8.293	3.335	-33.363	0.00	0.00	PROA
ATOM	2308	HG1	SER	P	144	9.040	3.793	-33.755	0.00	0.00	PROA
ATOM	2309	C	SER	P	144	5.655	2.465	-32.355	0.00	0.00	PROA
ATOM	2310	O	SER	P	144	4.984	3.166	-31.628	0.00	0.00	PROA
ATOM	2311	N	GLN	P	145	6.209	1.282	-32.016	0.00	0.00	PROA
ATOM	2312	HN	GLN	P	145	6.710	0.766	-32.706	0.00	0.00	PROA
ATOM	2313	CA	GLN	P	145	6.262	0.724	-30.675	0.00	0.00	PROA
ATOM	2314	HA	GLN	P	145	5.508	1.125	-30.014	0.00	0.00	PROA

ATOM 2315 CB GLN P 145 5.880 -0.792 -30.676 0.00 0.00 PROA
ATOM 2316 HB1 GLN P 145 4.887 -0.970 -31.141 0.00 0.00 PROA
ATOM 2317 HB2 GLN P 145 5.827 -1.142 -29.622 0.00 0.00 PROA
ATOM 2318 CG GLN P 145 6.931 -1.667 -31.474 0.00 0.00 PROA
ATOM 2319 HG1 GLN P 145 7.929 -1.253 -31.219 0.00 0.00 PROA
ATOM 2320 HG2 GLN P 145 6.845 -1.550 -32.576 0.00 0.00 PROA
ATOM 2321 CD GLN P 145 6.924 -3.186 -31.154 0.00 0.00 PROA
ATOM 2322 OE1 GLN P 145 5.925 -3.835 -30.866 0.00 0.00 PROA
ATOM 2323 NE2 GLN P 145 8.127 -3.831 -31.361 0.00 0.00 PROA
ATOM 2324 HE21 GLN P 145 7.936 -4.799 -31.523 0.00 0.00 PROA
ATOM 2325 HE22 GLN P 145 9.010 -3.382 -31.491 0.00 0.00 PROA
ATOM 2326 C GLN P 145 7.589 1.041 -29.938 0.00 0.00 PROA
ATOM 2327 O GLN P 145 7.746 0.639 -28.807 0.00 0.00 PROA
ATOM 2328 N ARG P 146 8.634 1.657 -30.540 0.00 0.00 PROA
ATOM 2329 HN ARG P 146 8.601 2.156 -31.403 0.00 0.00 PROA
ATOM 2330 CA ARG P 146 9.953 1.869 -29.944 0.00 0.00 PROA
ATOM 2331 HA ARG P 146 10.328 0.883 -29.715 0.00 0.00 PROA
ATOM 2332 CB ARG P 146 10.964 2.395 -31.016 0.00 0.00 PROA
ATOM 2333 HB1 ARG P 146 10.605 3.302 -31.548 0.00 0.00 PROA
ATOM 2334 HB2 ARG P 146 11.038 1.604 -31.792 0.00 0.00 PROA
ATOM 2335 CG ARG P 146 12.349 2.721 -30.432 0.00 0.00 PROA
ATOM 2336 HG1 ARG P 146 12.350 3.609 -29.764 0.00 0.00 PROA
ATOM 2337 HG2 ARG P 146 12.993 2.873 -31.324 0.00 0.00 PROA
ATOM 2338 CD ARG P 146 13.065 1.537 -29.724 0.00 0.00 PROA
ATOM 2339 HD1 ARG P 146 13.021 0.641 -30.378 0.00 0.00 PROA
ATOM 2340 HD2 ARG P 146 12.674 1.310 -28.709 0.00 0.00 PROA
ATOM 2341 NE ARG P 146 14.420 2.092 -29.502 0.00 0.00 PROA
ATOM 2342 HE ARG P 146 14.726 2.888 -30.023 0.00 0.00 PROA
ATOM 2343 CZ ARG P 146 15.265 1.751 -28.485 0.00 0.00 PROA
ATOM 2344 NH1 ARG P 146 15.271 0.641 -27.875 0.00 0.00 PROA
ATOM 2345 HH11 ARG P 146 14.585 -0.070 -28.027 0.00 0.00 PROA
ATOM 2346 HH12 ARG P 146 16.068 0.460 -27.299 0.00 0.00 PROA
ATOM 2347 NH2 ARG P 146 16.295 2.532 -28.367 0.00 0.00 PROA
ATOM 2348 HH21 ARG P 146 16.543 3.193 -29.075 0.00 0.00 PROA
ATOM 2349 HH22 ARG P 146 17.060 2.048 -27.940 0.00 0.00 PROA
ATOM 2350 C ARG P 146 9.934 2.616 -28.621 0.00 0.00 PROA
ATOM 2351 O ARG P 146 10.618 2.045 -27.726 0.00 0.00 PROA
ATOM 2352 N PRO P 147 9.287 3.762 -28.330 0.00 0.00 PROA
ATOM 2353 CD PRO P 147 8.894 4.778 -29.327 0.00 0.00 PROA
ATOM 2354 HD1 PRO P 147 7.882 4.501 -29.693 0.00 0.00 PROA
ATOM 2355 HD2 PRO P 147 9.578 4.789 -30.201 0.00 0.00 PROA
ATOM 2356 CA PRO P 147 9.369 4.385 -26.974 0.00 0.00 PROA
ATOM 2357 HA PRO P 147 10.417 4.485 -26.735 0.00 0.00 PROA
ATOM 2358 CB PRO P 147 8.651 5.754 -27.129 0.00 0.00 PROA
ATOM 2359 HB1 PRO P 147 8.911 6.502 -26.349 0.00 0.00 PROA
ATOM 2360 HB2 PRO P 147 7.557 5.563 -27.131 0.00 0.00 PROA
ATOM 2361 CG PRO P 147 8.984 6.100 -28.561 0.00 0.00 PROA
ATOM 2362 HG1 PRO P 147 8.224 6.805 -28.963 0.00 0.00 PROA
ATOM 2363 HG2 PRO P 147 9.988 6.525 -28.775 0.00 0.00 PROA
ATOM 2364 C PRO P 147 8.681 3.547 -25.935 0.00 0.00 PROA
ATOM 2365 O PRO P 147 9.035 3.698 -24.797 0.00 0.00 PROA
ATOM 2366 N ARG P 148 7.535 2.882 -26.290 0.00 0.00 PROA
ATOM 2367 HN ARG P 148 7.146 2.846 -27.207 0.00 0.00 PROA
ATOM 2368 CA ARG P 148 6.936 1.936 -25.345 0.00 0.00 PROA
ATOM 2369 HA ARG P 148 6.846 2.404 -24.376 0.00 0.00 PROA
ATOM 2370 CB ARG P 148 5.547 1.544 -25.782 0.00 0.00 PROA
ATOM 2371 HB1 ARG P 148 5.241 0.656 -25.188 0.00 0.00 PROA
ATOM 2372 HB2 ARG P 148 5.553 1.196 -26.837 0.00 0.00 PROA
ATOM 2373 CG ARG P 148 4.536 2.645 -25.504 0.00 0.00 PROA
ATOM 2374 HG1 ARG P 148 4.761 3.553 -26.104 0.00 0.00 PROA
ATOM 2375 HG2 ARG P 148 4.528 2.979 -24.444 0.00 0.00 PROA

```
ATOM   2376  CD  ARG P 148       3.042   2.255 -25.881  0.00  0.00      PROA
ATOM   2377  HD1 ARG P 148       3.026   1.699 -26.842  0.00  0.00      PROA
ATOM   2378  HD2 ARG P 148       2.398   3.160 -25.914  0.00  0.00      PROA
ATOM   2379  NE  ARG P 148       2.463   1.352 -24.885  0.00  0.00      PROA
ATOM   2380  HE  ARG P 148       2.704   1.440 -23.919  0.00  0.00      PROA
ATOM   2381  CZ  ARG P 148       1.782   0.218 -25.099  0.00  0.00      PROA
ATOM   2382  NH1 ARG P 148       1.302  -0.101 -26.265  0.00  0.00      PROA
ATOM   2383 HH11 ARG P 148       1.104   0.689 -26.846  0.00  0.00      PROA
ATOM   2384 HH12 ARG P 148       0.745  -0.928 -26.201  0.00  0.00      PROA
ATOM   2385  NH2 ARG P 148       1.552  -0.462 -24.027  0.00  0.00      PROA
ATOM   2386 HH21 ARG P 148       1.758   0.000 -23.165  0.00  0.00      PROA
ATOM   2387 HH22 ARG P 148       1.133  -1.370 -24.012  0.00  0.00      PROA
ATOM   2388  C   ARG P 148       7.767   0.736 -25.019  0.00  0.00      PROA
ATOM   2389  O   ARG P 148       7.758   0.258 -23.890  0.00  0.00      PROA
ATOM   2390  N   LYS P 149       8.414   0.080 -26.059  0.00  0.00      PROA
ATOM   2391  HN  LYS P 149       8.129   0.257 -26.998  0.00  0.00      PROA
ATOM   2392  CA  LYS P 149       9.267  -1.124 -25.997  0.00  0.00      PROA
ATOM   2393  HA  LYS P 149       8.635  -1.902 -25.595  0.00  0.00      PROA
ATOM   2394  CB  LYS P 149       9.646  -1.638 -27.398  0.00  0.00      PROA
ATOM   2395  HB1 LYS P 149      10.641  -2.131 -27.445  0.00  0.00      PROA
ATOM   2396  HB2 LYS P 149       9.763  -0.738 -28.039  0.00  0.00      PROA
ATOM   2397  CG  LYS P 149       8.595  -2.475 -28.065  0.00  0.00      PROA
ATOM   2398  HG1 LYS P 149       8.754  -2.642 -29.152  0.00  0.00      PROA
ATOM   2399  HG2 LYS P 149       7.606  -1.998 -27.897  0.00  0.00      PROA
ATOM   2400  CD  LYS P 149       8.282  -3.864 -27.497  0.00  0.00      PROA
ATOM   2401  HD1 LYS P 149       7.411  -4.244 -28.073  0.00  0.00      PROA
ATOM   2402  HD2 LYS P 149       7.824  -4.016 -26.497  0.00  0.00      PROA
ATOM   2403  CE  LYS P 149       9.425  -4.817 -27.637  0.00  0.00      PROA
ATOM   2404  HE1 LYS P 149      10.252  -4.475 -26.979  0.00  0.00      PROA
ATOM   2405  HE2 LYS P 149       9.769  -4.896 -28.690  0.00  0.00      PROA
ATOM   2406  NZ  LYS P 149       9.106  -6.255 -27.263  0.00  0.00      PROA
ATOM   2407  HZ1 LYS P 149       8.499  -6.761 -27.938  0.00  0.00      PROA
ATOM   2408  HZ2 LYS P 149       8.643  -6.206 -26.333  0.00  0.00      PROA
ATOM   2409  HZ3 LYS P 149      10.017  -6.731 -27.105  0.00  0.00      PROA
ATOM   2410  C   LYS P 149      10.460  -0.931 -25.098  0.00  0.00      PROA
ATOM   2411  O   LYS P 149      10.697  -1.751 -24.193  0.00  0.00      PROA
ATOM   2412  N   LEU P 150      11.190   0.223 -25.236  0.00  0.00      PROA
ATOM   2413  HN  LEU P 150      10.865   0.827 -25.961  0.00  0.00      PROA
ATOM   2414  CA  LEU P 150      12.297   0.688 -24.358  0.00  0.00      PROA
ATOM   2415  HA  LEU P 150      13.030  -0.101 -24.441  0.00  0.00      PROA
ATOM   2416  CB  LEU P 150      13.031   1.995 -24.901  0.00  0.00      PROA
ATOM   2417  HB1 LEU P 150      12.347   2.863 -24.792  0.00  0.00      PROA
ATOM   2418  HB2 LEU P 150      13.349   1.775 -25.943  0.00  0.00      PROA
ATOM   2419  CG  LEU P 150      14.336   2.350 -24.167  0.00  0.00      PROA
ATOM   2420  HG  LEU P 150      14.055   2.767 -23.177  0.00  0.00      PROA
ATOM   2421  CD1 LEU P 150      15.317   1.222 -24.180  0.00  0.00      PROA
ATOM   2422 HD11 LEU P 150      16.327   1.520 -23.825  0.00  0.00      PROA
ATOM   2423 HD12 LEU P 150      15.416   0.701 -25.156  0.00  0.00      PROA
ATOM   2424 HD13 LEU P 150      14.878   0.486 -23.472  0.00  0.00      PROA
ATOM   2425  CD2 LEU P 150      15.018   3.554 -24.897  0.00  0.00      PROA
ATOM   2426 HD21 LEU P 150      15.952   3.842 -24.367  0.00  0.00      PROA
ATOM   2427 HD22 LEU P 150      14.417   4.458 -25.136  0.00  0.00      PROA
ATOM   2428 HD23 LEU P 150      15.314   3.024 -25.827  0.00  0.00      PROA
ATOM   2429  C   LEU P 150      11.928   0.965 -22.952  0.00  0.00      PROA
ATOM   2430  O   LEU P 150      12.570   0.593 -22.014  0.00  0.00      PROA
ATOM   2431  N   LEU P 151      10.727   1.502 -22.720  0.00  0.00      PROA
ATOM   2432  HN  LEU P 151      10.092   1.639 -23.476  0.00  0.00      PROA
ATOM   2433  CA  LEU P 151      10.076   1.550 -21.413  0.00  0.00      PROA
ATOM   2434  HA  LEU P 151      10.619   2.197 -20.740  0.00  0.00      PROA
ATOM   2435  CB  LEU P 151       8.752   2.295 -21.455  0.00  0.00      PROA
ATOM   2436  HB1 LEU P 151       8.071   1.726 -22.123  0.00  0.00      PROA
```

ATOM	2437	HB2	LEU	P	151	8.841	3.313	-21.891	0.00	0.00	PROA
ATOM	2438	CG	LEU	P	151	7.960	2.537	-20.154	0.00	0.00	PROA
ATOM	2439	HG	LEU	P	151	8.023	1.538	-19.672	0.00	0.00	PROA
ATOM	2440	CD1	LEU	P	151	8.582	3.685	-19.322	0.00	0.00	PROA
ATOM	2441	HD11	LEU	P	151	8.031	3.940	-18.391	0.00	0.00	PROA
ATOM	2442	HD12	LEU	P	151	8.651	4.671	-19.828	0.00	0.00	PROA
ATOM	2443	HD13	LEU	P	151	9.618	3.457	-18.992	0.00	0.00	PROA
ATOM	2444	CD2	LEU	P	151	6.572	2.880	-20.448	0.00	0.00	PROA
ATOM	2445	HD21	LEU	P	151	6.125	2.185	-21.191	0.00	0.00	PROA
ATOM	2446	HD22	LEU	P	151	6.503	3.940	-20.774	0.00	0.00	PROA
ATOM	2447	HD23	LEU	P	151	6.040	2.777	-19.479	0.00	0.00	PROA
ATOM	2448	C	LEU	P	151	9.913	0.135	-20.811	0.00	0.00	PROA
ATOM	2449	O	LEU	P	151	10.500	-0.232	-19.769	0.00	0.00	PROA
ATOM	2450	N	ALA	P	152	9.100	-0.723	-21.461	0.00	0.00	PROA
ATOM	2451	HN	ALA	P	152	8.743	-0.420	-22.341	0.00	0.00	PROA
ATOM	2452	CA	ALA	P	152	8.807	-2.099	-21.132	0.00	0.00	PROA
ATOM	2453	HA	ALA	P	152	8.246	-2.127	-20.210	0.00	0.00	PROA
ATOM	2454	CB	ALA	P	152	8.025	-2.723	-22.324	0.00	0.00	PROA
ATOM	2455	HB1	ALA	P	152	7.248	-2.004	-22.662	0.00	0.00	PROA
ATOM	2456	HB2	ALA	P	152	7.533	-3.657	-21.976	0.00	0.00	PROA
ATOM	2457	HB3	ALA	P	152	8.659	-2.926	-23.213	0.00	0.00	PROA
ATOM	2458	C	ALA	P	152	9.982	-3.004	-20.683	0.00	0.00	PROA
ATOM	2459	O	ALA	P	152	9.948	-3.649	-19.636	0.00	0.00	PROA
ATOM	2460	N	GLU	P	153	11.035	-3.129	-21.526	0.00	0.00	PROA
ATOM	2461	HN	GLU	P	153	10.949	-2.762	-22.449	0.00	0.00	PROA
ATOM	2462	CA	GLU	P	153	12.226	-3.914	-21.284	0.00	0.00	PROA
ATOM	2463	HA	GLU	P	153	11.930	-4.821	-20.778	0.00	0.00	PROA
ATOM	2464	CB	GLU	P	153	12.619	-4.494	-22.662	0.00	0.00	PROA
ATOM	2465	HB1	GLU	P	153	13.525	-5.128	-22.558	0.00	0.00	PROA
ATOM	2466	HB2	GLU	P	153	12.950	-3.591	-23.218	0.00	0.00	PROA
ATOM	2467	CG	GLU	P	153	11.595	-5.454	-23.336	0.00	0.00	PROA
ATOM	2468	HG1	GLU	P	153	10.581	-5.001	-23.360	0.00	0.00	PROA
ATOM	2469	HG2	GLU	P	153	11.506	-6.350	-22.685	0.00	0.00	PROA
ATOM	2470	CD	GLU	P	153	12.007	-5.829	-24.773	0.00	0.00	PROA
ATOM	2471	OE1	GLU	P	153	11.340	-6.668	-25.445	0.00	0.00	PROA
ATOM	2472	OE2	GLU	P	153	13.051	-5.314	-25.281	0.00	0.00	PROA
ATOM	2473	C	GLU	P	153	13.406	-3.327	-20.576	0.00	0.00	PROA
ATOM	2474	O	GLU	P	153	14.265	-4.091	-20.194	0.00	0.00	PROA
ATOM	2475	N	LYS	P	154	13.392	-2.043	-20.345	0.00	0.00	PROA
ATOM	2476	HN	LYS	P	154	12.601	-1.497	-20.611	0.00	0.00	PROA
ATOM	2477	CA	LYS	P	154	14.462	-1.377	-19.593	0.00	0.00	PROA
ATOM	2478	HA	LYS	P	154	15.009	-2.148	-19.071	0.00	0.00	PROA
ATOM	2479	CB	LYS	P	154	15.273	-0.531	-20.567	0.00	0.00	PROA
ATOM	2480	HB1	LYS	P	154	16.081	0.017	-20.036	0.00	0.00	PROA
ATOM	2481	HB2	LYS	P	154	14.669	0.167	-21.184	0.00	0.00	PROA
ATOM	2482	CG	LYS	P	154	15.967	-1.386	-21.649	0.00	0.00	PROA
ATOM	2483	HG1	LYS	P	154	16.319	-0.576	-22.324	0.00	0.00	PROA
ATOM	2484	HG2	LYS	P	154	15.149	-1.838	-22.249	0.00	0.00	PROA
ATOM	2485	CD	LYS	P	154	17.044	-2.282	-21.238	0.00	0.00	PROA
ATOM	2486	HD1	LYS	P	154	16.689	-2.956	-20.429	0.00	0.00	PROA
ATOM	2487	HD2	LYS	P	154	17.923	-1.753	-20.811	0.00	0.00	PROA
ATOM	2488	CE	LYS	P	154	17.450	-3.327	-22.378	0.00	0.00	PROA
ATOM	2489	HE1	LYS	P	154	16.649	-4.088	-22.492	0.00	0.00	PROA
ATOM	2490	HE2	LYS	P	154	18.352	-3.883	-22.044	0.00	0.00	PROA
ATOM	2491	NZ	LYS	P	154	17.800	-2.686	-23.622	0.00	0.00	PROA
ATOM	2492	HZ1	LYS	P	154	18.415	-1.877	-23.405	0.00	0.00	PROA
ATOM	2493	HZ2	LYS	P	154	16.959	-2.379	-24.152	0.00	0.00	PROA
ATOM	2494	HZ3	LYS	P	154	18.305	-3.335	-24.259	0.00	0.00	PROA
ATOM	2495	C	LYS	P	154	13.955	-0.471	-18.496	0.00	0.00	PROA
ATOM	2496	O	LYS	P	154	14.263	-0.697	-17.348	0.00	0.00	PROA
ATOM	2497	N	VAL	P	155	13.214	0.636	-18.811	0.00	0.00	PROA

ATOM	2498	HN	VAL	P	155	12.903	0.788	-19.746	0.00	0.00	PROA
ATOM	2499	CA	VAL	P	155	13.052	1.622	-17.695	0.00	0.00	PROA
ATOM	2500	HA	VAL	P	155	14.062	1.928	-17.465	0.00	0.00	PROA
ATOM	2501	CB	VAL	P	155	12.342	2.815	-18.286	0.00	0.00	PROA
ATOM	2502	HB	VAL	P	155	11.342	2.464	-18.618	0.00	0.00	PROA
ATOM	2503	CG1	VAL	P	155	12.187	3.833	-17.113	0.00	0.00	PROA
ATOM	2504	HG11	VAL	P	155	11.263	3.517	-16.581	0.00	0.00	PROA
ATOM	2505	HG12	VAL	P	155	11.942	4.864	-17.445	0.00	0.00	PROA
ATOM	2506	HG13	VAL	P	155	13.064	3.910	-16.436	0.00	0.00	PROA
ATOM	2507	CG2	VAL	P	155	13.166	3.334	-19.519	0.00	0.00	PROA
ATOM	2508	HG21	VAL	P	155	12.926	4.405	-19.692	0.00	0.00	PROA
ATOM	2509	HG22	VAL	P	155	13.023	2.632	-20.368	0.00	0.00	PROA
ATOM	2510	HG23	VAL	P	155	14.263	3.440	-19.383	0.00	0.00	PROA
ATOM	2511	C	VAL	P	155	12.297	1.123	-16.458	0.00	0.00	PROA
ATOM	2512	O	VAL	P	155	12.648	1.415	-15.324	0.00	0.00	PROA
ATOM	2513	N	VAL	P	156	11.289	0.294	-16.709	0.00	0.00	PROA
ATOM	2514	HN	VAL	P	156	10.973	0.132	-17.640	0.00	0.00	PROA
ATOM	2515	CA	VAL	P	156	10.434	-0.242	-15.621	0.00	0.00	PROA
ATOM	2516	HA	VAL	P	156	10.116	0.701	-15.201	0.00	0.00	PROA
ATOM	2517	CB	VAL	P	156	9.262	-0.943	-16.274	0.00	0.00	PROA
ATOM	2518	HB	VAL	P	156	9.009	-0.261	-17.114	0.00	0.00	PROA
ATOM	2519	CG1	VAL	P	156	9.670	-2.321	-16.876	0.00	0.00	PROA
ATOM	2520	HG11	VAL	P	156	10.004	-2.930	-16.009	0.00	0.00	PROA
ATOM	2521	HG12	VAL	P	156	10.481	-2.183	-17.623	0.00	0.00	PROA
ATOM	2522	HG13	VAL	P	156	8.803	-2.808	-17.371	0.00	0.00	PROA
ATOM	2523	CG2	VAL	P	156	8.131	-0.945	-15.251	0.00	0.00	PROA
ATOM	2524	HG21	VAL	P	156	7.216	-1.312	-15.764	0.00	0.00	PROA
ATOM	2525	HG22	VAL	P	156	7.791	0.026	-14.832	0.00	0.00	PROA
ATOM	2526	HG23	VAL	P	156	8.298	-1.735	-14.488	0.00	0.00	PROA
ATOM	2527	C	VAL	P	156	11.074	-1.009	-14.518	0.00	0.00	PROA
ATOM	2528	O	VAL	P	156	10.660	-0.897	-13.408	0.00	0.00	PROA
ATOM	2529	N	TYR	P	157	12.140	-1.773	-14.734	0.00	0.00	PROA
ATOM	2530	HN	TYR	P	157	12.496	-1.803	-15.665	0.00	0.00	PROA
ATOM	2531	CA	TYR	P	157	12.850	-2.533	-13.757	0.00	0.00	PROA
ATOM	2532	HA	TYR	P	157	12.161	-3.240	-13.319	0.00	0.00	PROA
ATOM	2533	CB	TYR	P	157	13.977	-3.355	-14.492	0.00	0.00	PROA
ATOM	2534	HB1	TYR	P	157	14.682	-3.894	-13.823	0.00	0.00	PROA
ATOM	2535	HB2	TYR	P	157	14.550	-2.687	-15.170	0.00	0.00	PROA
ATOM	2536	CG	TYR	P	157	13.288	-4.442	-15.340	0.00	0.00	PROA
ATOM	2537	CD1	TYR	P	157	13.110	-5.766	-14.773	0.00	0.00	PROA
ATOM	2538	HD1	TYR	P	157	13.380	-5.738	-13.727	0.00	0.00	PROA
ATOM	2539	CE1	TYR	P	157	12.490	-6.764	-15.545	0.00	0.00	PROA
ATOM	2540	HE1	TYR	P	157	12.163	-7.751	-15.250	0.00	0.00	PROA
ATOM	2541	CZ	TYR	P	157	12.308	-6.575	-16.877	0.00	0.00	PROA
ATOM	2542	OH	TYR	P	157	11.598	-7.577	-17.618	0.00	0.00	PROA
ATOM	2543	HH	TYR	P	157	11.368	-8.325	-17.062	0.00	0.00	PROA
ATOM	2544	CD2	TYR	P	157	13.080	-4.320	-16.755	0.00	0.00	PROA
ATOM	2545	HD2	TYR	P	157	13.208	-3.321	-17.144	0.00	0.00	PROA
ATOM	2546	CE2	TYR	P	157	12.608	-5.377	-17.569	0.00	0.00	PROA
ATOM	2547	HE2	TYR	P	157	12.427	-5.289	-18.630	0.00	0.00	PROA
ATOM	2548	C	TYR	P	157	13.488	-1.754	-12.679	0.00	0.00	PROA
ATOM	2549	O	TYR	P	157	13.346	-2.029	-11.449	0.00	0.00	PROA
ATOM	2550	N	VAL	P	158	14.209	-0.684	-13.003	0.00	0.00	PROA
ATOM	2551	HN	VAL	P	158	14.351	-0.479	-13.968	0.00	0.00	PROA
ATOM	2552	CA	VAL	P	158	14.835	0.235	-12.137	0.00	0.00	PROA
ATOM	2553	HA	VAL	P	158	15.397	-0.269	-11.365	0.00	0.00	PROA
ATOM	2554	CB	VAL	P	158	15.950	1.025	-12.845	0.00	0.00	PROA
ATOM	2555	HB	VAL	P	158	16.431	1.712	-12.117	0.00	0.00	PROA
ATOM	2556	CG1	VAL	P	158	17.050	0.008	-13.296	0.00	0.00	PROA
ATOM	2557	HG11	VAL	P	158	17.918	0.530	-13.753	0.00	0.00	PROA
ATOM	2558	HG12	VAL	P	158	16.754	-0.621	-14.163	0.00	0.00	PROA

ATOM	2559	HG13	VAL	P	158	17.443	-0.594	-12.450	0.00	0.00	PROA
ATOM	2560	CG2	VAL	P	158	15.516	1.877	-14.076	0.00	0.00	PROA
ATOM	2561	HG21	VAL	P	158	14.604	2.477	-13.867	0.00	0.00	PROA
ATOM	2562	HG22	VAL	P	158	15.138	1.182	-14.856	0.00	0.00	PROA
ATOM	2563	HG23	VAL	P	158	16.330	2.516	-14.480	0.00	0.00	PROA
ATOM	2564	C	VAL	P	158	13.786	1.159	-11.421	0.00	0.00	PROA
ATOM	2565	O	VAL	P	158	13.945	1.496	-10.298	0.00	0.00	PROA
ATOM	2566	N	GLY	P	159	12.707	1.609	-12.134	0.00	0.00	PROA
ATOM	2567	HN	GLY	P	159	12.549	1.139	-12.999	0.00	0.00	PROA
ATOM	2568	CA	GLY	P	159	11.696	2.469	-11.706	0.00	0.00	PROA
ATOM	2569	HA1	GLY	P	159	11.236	2.775	-12.635	0.00	0.00	PROA
ATOM	2570	HA2	GLY	P	159	12.311	3.155	-11.143	0.00	0.00	PROA
ATOM	2571	C	GLY	P	159	10.622	1.977	-10.622	0.00	0.00	PROA
ATOM	2572	O	GLY	P	159	10.038	2.706	-9.871	0.00	0.00	PROA
ATOM	2573	N	VAL	P	160	10.271	0.709	-10.713	0.00	0.00	PROA
ATOM	2574	HN	VAL	P	160	10.736	0.145	-11.392	0.00	0.00	PROA
ATOM	2575	CA	VAL	P	160	9.194	0.127	-9.930	0.00	0.00	PROA
ATOM	2576	HA	VAL	P	160	8.508	0.909	-9.640	0.00	0.00	PROA
ATOM	2577	CB	VAL	P	160	8.354	-0.857	-10.704	0.00	0.00	PROA
ATOM	2578	HB	VAL	P	160	9.064	-1.631	-11.069	0.00	0.00	PROA
ATOM	2579	CG1	VAL	P	160	7.180	-1.501	-9.859	0.00	0.00	PROA
ATOM	2580	HG11	VAL	P	160	7.006	-0.862	-8.968	0.00	0.00	PROA
ATOM	2581	HG12	VAL	P	160	7.534	-2.483	-9.478	0.00	0.00	PROA
ATOM	2582	HG13	VAL	P	160	6.265	-1.597	-10.481	0.00	0.00	PROA
ATOM	2583	CG2	VAL	P	160	7.712	-0.038	-11.797	0.00	0.00	PROA
ATOM	2584	HG21	VAL	P	160	6.959	0.673	-11.394	0.00	0.00	PROA
ATOM	2585	HG22	VAL	P	160	7.184	-0.701	-12.515	0.00	0.00	PROA
ATOM	2586	HG23	VAL	P	160	8.458	0.550	-12.374	0.00	0.00	PROA
ATOM	2587	C	VAL	P	160	9.732	-0.522	-8.676	0.00	0.00	PROA
ATOM	2588	O	VAL	P	160	9.163	-0.447	-7.579	0.00	0.00	PROA
ATOM	2589	N	TRP	P	161	10.915	-1.217	-8.789	0.00	0.00	PROA
ATOM	2590	HN	TRP	P	161	11.262	-1.213	-9.724	0.00	0.00	PROA
ATOM	2591	CA	TRP	P	161	11.257	-2.129	-7.703	0.00	0.00	PROA
ATOM	2592	HA	TRP	P	161	10.380	-2.259	-7.087	0.00	0.00	PROA
ATOM	2593	CB	TRP	P	161	11.792	-3.447	-8.369	0.00	0.00	PROA
ATOM	2594	HB1	TRP	P	161	12.324	-4.157	-7.699	0.00	0.00	PROA
ATOM	2595	HB2	TRP	P	161	12.563	-3.066	-9.072	0.00	0.00	PROA
ATOM	2596	CG	TRP	P	161	10.751	-4.251	-9.088	0.00	0.00	PROA
ATOM	2597	CD1	TRP	P	161	10.820	-4.596	-10.429	0.00	0.00	PROA
ATOM	2598	HD1	TRP	P	161	11.504	-4.156	-11.141	0.00	0.00	PROA
ATOM	2599	NE1	TRP	P	161	9.699	-5.304	-10.741	0.00	0.00	PROA
ATOM	2600	HE1	TRP	P	161	9.741	-5.901	-11.512	0.00	0.00	PROA
ATOM	2601	CE2	TRP	P	161	8.930	-5.451	-9.651	0.00	0.00	PROA
ATOM	2602	CD2	TRP	P	161	9.556	-4.802	-8.553	0.00	0.00	PROA
ATOM	2603	CE3	TRP	P	161	8.879	-4.796	-7.360	0.00	0.00	PROA
ATOM	2604	HE3	TRP	P	161	9.263	-4.252	-6.509	0.00	0.00	PROA
ATOM	2605	CZ3	TRP	P	161	7.702	-5.570	-7.174	0.00	0.00	PROA
ATOM	2606	HZ3	TRP	P	161	7.207	-5.527	-6.215	0.00	0.00	PROA
ATOM	2607	CZ2	TRP	P	161	7.706	-6.115	-9.548	0.00	0.00	PROA
ATOM	2608	HZ2	TRP	P	161	7.339	-6.611	-10.434	0.00	0.00	PROA
ATOM	2609	CH2	TRP	P	161	7.127	-6.199	-8.276	0.00	0.00	PROA
ATOM	2610	HH2	TRP	P	161	6.194	-6.737	-8.193	0.00	0.00	PROA
ATOM	2611	C	TRP	P	161	12.285	-1.550	-6.734	0.00	0.00	PROA
ATOM	2612	O	TRP	P	161	12.155	-1.786	-5.530	0.00	0.00	PROA
ATOM	2613	N	ILE	P	162	13.251	-0.750	-7.264	0.00	0.00	PROA
ATOM	2614	HN	ILE	P	162	13.335	-0.519	-8.230	0.00	0.00	PROA
ATOM	2615	CA	ILE	P	162	14.330	-0.218	-6.445	0.00	0.00	PROA
ATOM	2616	HA	ILE	P	162	14.772	-1.036	-5.896	0.00	0.00	PROA
ATOM	2617	CB	ILE	P	162	15.671	0.172	-7.246	0.00	0.00	PROA
ATOM	2618	HB	ILE	P	162	15.282	0.979	-7.903	0.00	0.00	PROA
ATOM	2619	CG2	ILE	P	162	16.834	0.785	-6.249	0.00	0.00	PROA

ATOM 2620 HG21 ILE P 162 17.710 0.833 -6.930 0.00 0.00 PROA
ATOM 2621 HG22 ILE P 162 16.972 0.066 -5.414 0.00 0.00 PROA
ATOM 2622 HG23 ILE P 162 16.472 1.766 -5.874 0.00 0.00 PROA
ATOM 2623 CG1 ILE P 162 16.130 -1.000 -8.157 0.00 0.00 PROA
ATOM 2624 HG11 ILE P 162 15.337 -1.339 -8.858 0.00 0.00 PROA
ATOM 2625 HG12 ILE P 162 16.259 -1.816 -7.414 0.00 0.00 PROA
ATOM 2626 CD ILE P 162 17.452 -0.894 -8.886 0.00 0.00 PROA
ATOM 2627 HD1 ILE P 162 17.525 -1.817 -9.501 0.00 0.00 PROA
ATOM 2628 HD2 ILE P 162 18.324 -0.801 -8.205 0.00 0.00 PROA
ATOM 2629 HD3 ILE P 162 17.545 0.043 -9.476 0.00 0.00 PROA
ATOM 2630 C ILE P 162 13.860 0.725 -5.286 0.00 0.00 PROA
ATOM 2631 O ILE P 162 14.260 0.527 -4.171 0.00 0.00 PROA
ATOM 2632 N PRO P 163 12.905 1.605 -5.644 0.00 0.00 PROA
ATOM 2633 CD PRO P 163 12.622 2.208 -6.963 0.00 0.00 PROA
ATOM 2634 HD1 PRO P 163 11.887 1.506 -7.413 0.00 0.00 PROA
ATOM 2635 HD2 PRO P 163 13.493 2.255 -7.651 0.00 0.00 PROA
ATOM 2636 CA PRO P 163 12.290 2.385 -4.595 0.00 0.00 PROA
ATOM 2637 HA PRO P 163 13.121 2.896 -4.132 0.00 0.00 PROA
ATOM 2638 CB PRO P 163 11.293 3.292 -5.382 0.00 0.00 PROA
ATOM 2639 HB1 PRO P 163 11.001 4.207 -4.825 0.00 0.00 PROA
ATOM 2640 HB2 PRO P 163 10.359 2.702 -5.497 0.00 0.00 PROA
ATOM 2641 CG PRO P 163 12.051 3.583 -6.707 0.00 0.00 PROA
ATOM 2642 HG1 PRO P 163 11.406 3.770 -7.592 0.00 0.00 PROA
ATOM 2643 HG2 PRO P 163 12.908 4.278 -6.583 0.00 0.00 PROA
ATOM 2644 C PRO P 163 11.647 1.643 -3.511 0.00 0.00 PROA
ATOM 2645 O PRO P 163 11.767 2.098 -2.393 0.00 0.00 PROA
ATOM 2646 N ALA P 164 10.952 0.597 -3.749 0.00 0.00 PROA
ATOM 2647 HN ALA P 164 10.874 0.206 -4.663 0.00 0.00 PROA
ATOM 2648 CA ALA P 164 10.368 -0.314 -2.788 0.00 0.00 PROA
ATOM 2649 HA ALA P 164 9.680 0.210 -2.141 0.00 0.00 PROA
ATOM 2650 CB ALA P 164 9.390 -1.197 -3.613 0.00 0.00 PROA
ATOM 2651 HB1 ALA P 164 8.628 -0.655 -4.213 0.00 0.00 PROA
ATOM 2652 HB2 ALA P 164 8.907 -1.834 -2.841 0.00 0.00 PROA
ATOM 2653 HB3 ALA P 164 10.041 -1.819 -4.264 0.00 0.00 PROA
ATOM 2654 C ALA P 164 11.394 -1.032 -1.917 0.00 0.00 PROA
ATOM 2655 O ALA P 164 11.182 -1.288 -0.698 0.00 0.00 PROA
ATOM 2656 N LEU P 165 12.474 -1.486 -2.551 0.00 0.00 PROA
ATOM 2657 HN LEU P 165 12.573 -1.332 -3.531 0.00 0.00 PROA
ATOM 2658 CA LEU P 165 13.506 -2.233 -1.898 0.00 0.00 PROA
ATOM 2659 HA LEU P 165 13.085 -3.015 -1.283 0.00 0.00 PROA
ATOM 2660 CB LEU P 165 14.461 -2.951 -2.818 0.00 0.00 PROA
ATOM 2661 HB1 LEU P 165 15.240 -3.494 -2.242 0.00 0.00 PROA
ATOM 2662 HB2 LEU P 165 14.871 -2.130 -3.443 0.00 0.00 PROA
ATOM 2663 CG LEU P 165 13.785 -3.940 -3.733 0.00 0.00 PROA
ATOM 2664 HG LEU P 165 12.731 -3.651 -3.932 0.00 0.00 PROA
ATOM 2665 CD1 LEU P 165 14.576 -4.092 -5.022 0.00 0.00 PROA
ATOM 2666 HD11 LEU P 165 13.925 -4.061 -5.922 0.00 0.00 PROA
ATOM 2667 HD12 LEU P 165 15.293 -4.940 -5.013 0.00 0.00 PROA
ATOM 2668 HD13 LEU P 165 15.210 -3.190 -5.163 0.00 0.00 PROA
ATOM 2669 CD2 LEU P 165 13.776 -5.357 -3.075 0.00 0.00 PROA
ATOM 2670 HD21 LEU P 165 13.309 -5.397 -2.068 0.00 0.00 PROA
ATOM 2671 HD22 LEU P 165 14.836 -5.689 -3.094 0.00 0.00 PROA
ATOM 2672 HD23 LEU P 165 13.260 -6.131 -3.683 0.00 0.00 PROA
ATOM 2673 C LEU P 165 14.273 -1.347 -0.984 0.00 0.00 PROA
ATOM 2674 O LEU P 165 14.873 -1.772 -0.009 0.00 0.00 PROA
ATOM 2675 N LEU P 166 14.551 -0.054 -1.383 0.00 0.00 PROA
ATOM 2676 HN LEU P 166 14.257 0.374 -2.234 0.00 0.00 PROA
ATOM 2677 CA LEU P 166 15.292 0.940 -0.652 0.00 0.00 PROA
ATOM 2678 HA LEU P 166 16.238 0.520 -0.344 0.00 0.00 PROA
ATOM 2679 CB LEU P 166 15.473 2.263 -1.524 0.00 0.00 PROA
ATOM 2680 HB1 LEU P 166 15.704 3.179 -0.939 0.00 0.00 PROA

ATOM	2681	HB2	LEU	P	166	14.454	2.435	-1.933	0.00	0.00	PROA
ATOM	2682	CG	LEU	P	166	16.620	2.236	-2.545	0.00	0.00	PROA
ATOM	2683	HG	LEU	P	166	16.542	1.346	-3.206	0.00	0.00	PROA
ATOM	2684	CD1	LEU	P	166	16.442	3.432	-3.391	0.00	0.00	PROA
ATOM	2685	HD11	LEU	P	166	15.577	3.421	-4.088	0.00	0.00	PROA
ATOM	2686	HD12	LEU	P	166	17.393	3.516	-3.958	0.00	0.00	PROA
ATOM	2687	HD13	LEU	P	166	16.231	4.384	-2.859	0.00	0.00	PROA
ATOM	2688	CD2	LEU	P	166	18.067	2.138	-1.907	0.00	0.00	PROA
ATOM	2689	HD21	LEU	P	166	18.946	2.046	-2.581	0.00	0.00	PROA
ATOM	2690	HD22	LEU	P	166	17.873	1.183	-1.373	0.00	0.00	PROA
ATOM	2691	HD23	LEU	P	166	18.128	2.912	-1.113	0.00	0.00	PROA
ATOM	2692	C	LEU	P	166	14.595	1.325	0.595	0.00	0.00	PROA
ATOM	2693	O	LEU	P	166	15.220	1.833	1.495	0.00	0.00	PROA
ATOM	2694	N	LEU	P	167	13.283	1.042	0.739	0.00	0.00	PROA
ATOM	2695	HN	LEU	P	167	12.762	0.719	-0.047	0.00	0.00	PROA
ATOM	2696	CA	LEU	P	167	12.546	1.259	1.976	0.00	0.00	PROA
ATOM	2697	HA	LEU	P	167	13.073	1.939	2.629	0.00	0.00	PROA
ATOM	2698	CB	LEU	P	167	11.260	1.953	1.543	0.00	0.00	PROA
ATOM	2699	HB1	LEU	P	167	10.520	2.179	2.340	0.00	0.00	PROA
ATOM	2700	HB2	LEU	P	167	10.686	1.282	0.868	0.00	0.00	PROA
ATOM	2701	CG	LEU	P	167	11.469	3.262	0.801	0.00	0.00	PROA
ATOM	2702	HG	LEU	P	167	12.062	3.127	-0.129	0.00	0.00	PROA
ATOM	2703	CD1	LEU	P	167	10.090	3.882	0.462	0.00	0.00	PROA
ATOM	2704	HD11	LEU	P	167	10.261	4.696	-0.274	0.00	0.00	PROA
ATOM	2705	HD12	LEU	P	167	9.812	4.222	1.482	0.00	0.00	PROA
ATOM	2706	HD13	LEU	P	167	9.346	3.177	0.033	0.00	0.00	PROA
ATOM	2707	CD2	LEU	P	167	12.249	4.282	1.644	0.00	0.00	PROA
ATOM	2708	HD21	LEU	P	167	11.881	4.357	2.690	0.00	0.00	PROA
ATOM	2709	HD22	LEU	P	167	12.220	5.253	1.104	0.00	0.00	PROA
ATOM	2710	HD23	LEU	P	167	13.312	3.960	1.652	0.00	0.00	PROA
ATOM	2711	C	LEU	P	167	12.249	0.024	2.827	0.00	0.00	PROA
ATOM	2712	O	LEU	P	167	11.740	0.115	3.944	0.00	0.00	PROA
ATOM	2713	N	THR	P	168	12.636	-1.194	2.257	0.00	0.00	PROA
ATOM	2714	HN	THR	P	168	13.006	-1.227	1.332	0.00	0.00	PROA
ATOM	2715	CA	THR	P	168	12.361	-2.434	2.956	0.00	0.00	PROA
ATOM	2716	HA	THR	P	168	11.548	-2.165	3.614	0.00	0.00	PROA
ATOM	2717	CB	THR	P	168	11.882	-3.597	2.078	0.00	0.00	PROA
ATOM	2718	HB	THR	P	168	11.298	-3.139	1.251	0.00	0.00	PROA
ATOM	2719	OG1	THR	P	168	11.134	-4.409	2.925	0.00	0.00	PROA
ATOM	2720	HG1	THR	P	168	10.352	-4.654	2.426	0.00	0.00	PROA
ATOM	2721	CG2	THR	P	168	13.000	-4.520	1.540	0.00	0.00	PROA
ATOM	2722	HG21	THR	P	168	12.549	-5.184	0.773	0.00	0.00	PROA
ATOM	2723	HG22	THR	P	168	13.418	-5.235	2.280	0.00	0.00	PROA
ATOM	2724	HG23	THR	P	168	13.788	-3.928	1.028	0.00	0.00	PROA
ATOM	2725	C	THR	P	168	13.598	-2.854	3.825	0.00	0.00	PROA
ATOM	2726	O	THR	P	168	13.562	-3.687	4.676	0.00	0.00	PROA
ATOM	2727	N	ILE	P	169	14.728	-2.152	3.701	0.00	0.00	PROA
ATOM	2728	HN	ILE	P	169	14.543	-1.448	3.019	0.00	0.00	PROA
ATOM	2729	CA	ILE	P	169	15.983	-2.290	4.448	0.00	0.00	PROA
ATOM	2730	HA	ILE	P	169	16.133	-3.355	4.345	0.00	0.00	PROA
ATOM	2731	CB	ILE	P	169	17.207	-1.627	3.856	0.00	0.00	PROA
ATOM	2732	HB	ILE	P	169	16.971	-0.542	3.862	0.00	0.00	PROA
ATOM	2733	CG2	ILE	P	169	18.437	-2.111	4.700	0.00	0.00	PROA
ATOM	2734	HG21	ILE	P	169	18.388	-3.221	4.698	0.00	0.00	PROA
ATOM	2735	HG22	ILE	P	169	18.528	-1.779	5.757	0.00	0.00	PROA
ATOM	2736	HG23	ILE	P	169	19.345	-1.817	4.131	0.00	0.00	PROA
ATOM	2737	CG1	ILE	P	169	17.337	-2.120	2.374	0.00	0.00	PROA
ATOM	2738	HG11	ILE	P	169	16.421	-2.641	2.021	0.00	0.00	PROA
ATOM	2739	HG12	ILE	P	169	18.199	-2.815	2.284	0.00	0.00	PROA
ATOM	2740	CD	ILE	P	169	17.693	-0.994	1.374	0.00	0.00	PROA
ATOM	2741	HD1	ILE	P	169	18.518	-0.311	1.670	0.00	0.00	PROA

```
ATOM   2742  HD2 ILE P 169      16.812  -0.321   1.307  0.00  0.00      PROA
ATOM   2743  HD3 ILE P 169      17.906  -1.447   0.382  0.00  0.00      PROA
ATOM   2744  C   ILE P 169      15.819  -2.121   5.955  0.00  0.00      PROA
ATOM   2745  O   ILE P 169      16.308  -2.963   6.669  0.00  0.00      PROA
ATOM   2746  N   PRO P 170      15.104  -1.079   6.472  0.00  0.00      PROA
ATOM   2747  CD  PRO P 170      14.980   0.227   5.790  0.00  0.00      PROA
ATOM   2748  HD1 PRO P 170      14.703   0.139   4.718  0.00  0.00      PROA
ATOM   2749  HD2 PRO P 170      15.999   0.670   5.803  0.00  0.00      PROA
ATOM   2750  CA  PRO P 170      14.778  -0.974   7.848  0.00  0.00      PROA
ATOM   2751  HA  PRO P 170      15.643  -1.278   8.419  0.00  0.00      PROA
ATOM   2752  CB  PRO P 170      14.287   0.492   8.051  0.00  0.00      PROA
ATOM   2753  HB1 PRO P 170      15.102   1.005   8.604  0.00  0.00      PROA
ATOM   2754  HB2 PRO P 170      13.406   0.591   8.721  0.00  0.00      PROA
ATOM   2755  CG  PRO P 170      14.123   1.075   6.650  0.00  0.00      PROA
ATOM   2756  HG1 PRO P 170      13.039   1.043   6.407  0.00  0.00      PROA
ATOM   2757  HG2 PRO P 170      14.544   2.102   6.703  0.00  0.00      PROA
ATOM   2758  C   PRO P 170      13.676  -1.984   8.239  0.00  0.00      PROA
ATOM   2759  O   PRO P 170      13.717  -2.414   9.384  0.00  0.00      PROA
ATOM   2760  N   ASP P 171      12.726  -2.314   7.332  0.00  0.00      PROA
ATOM   2761  HN  ASP P 171      12.683  -1.828   6.462  0.00  0.00      PROA
ATOM   2762  CA  ASP P 171      11.619  -3.209   7.764  0.00  0.00      PROA
ATOM   2763  HA  ASP P 171      11.268  -2.913   8.742  0.00  0.00      PROA
ATOM   2764  CB  ASP P 171      10.467  -3.401   6.678  0.00  0.00      PROA
ATOM   2765  HB1 ASP P 171      10.029  -4.420   6.750  0.00  0.00      PROA
ATOM   2766  HB2 ASP P 171      10.823  -3.287   5.632  0.00  0.00      PROA
ATOM   2767  CG  ASP P 171       9.368  -2.355   6.891  0.00  0.00      PROA
ATOM   2768  OD1 ASP P 171       9.576  -1.244   7.509  0.00  0.00      PROA
ATOM   2769  OD2 ASP P 171       8.267  -2.646   6.324  0.00  0.00      PROA
ATOM   2770  C   ASP P 171      12.213  -4.569   8.117  0.00  0.00      PROA
ATOM   2771  O   ASP P 171      11.800  -5.233   9.064  0.00  0.00      PROA
ATOM   2772  N   PHE P 172      13.140  -5.050   7.313  0.00  0.00      PROA
ATOM   2773  HN  PHE P 172      13.379  -4.542   6.488  0.00  0.00      PROA
ATOM   2774  CA  PHE P 172      13.895  -6.195   7.725  0.00  0.00      PROA
ATOM   2775  HA  PHE P 172      13.270  -7.066   7.858  0.00  0.00      PROA
ATOM   2776  CB  PHE P 172      14.834  -6.675   6.540  0.00  0.00      PROA
ATOM   2777  HB1 PHE P 172      15.368  -5.773   6.173  0.00  0.00      PROA
ATOM   2778  HB2 PHE P 172      14.281  -7.035   5.646  0.00  0.00      PROA
ATOM   2779  CG  PHE P 172      15.807  -7.678   6.903  0.00  0.00      PROA
ATOM   2780  CD1 PHE P 172      17.177  -7.518   6.995  0.00  0.00      PROA
ATOM   2781  HD1 PHE P 172      17.637  -6.541   7.020  0.00  0.00      PROA
ATOM   2782  CE1 PHE P 172      18.065  -8.601   7.254  0.00  0.00      PROA
ATOM   2783  HE1 PHE P 172      19.116  -8.376   7.358  0.00  0.00      PROA
ATOM   2784  CZ  PHE P 172      17.550  -9.858   7.423  0.00  0.00      PROA
ATOM   2785  HZ  PHE P 172      18.140 -10.713   7.719  0.00  0.00      PROA
ATOM   2786  CD2 PHE P 172      15.326  -8.996   7.038  0.00  0.00      PROA
ATOM   2787  HD2 PHE P 172      14.292  -9.210   6.810  0.00  0.00      PROA
ATOM   2788  CE2 PHE P 172      16.181 -10.058   7.324  0.00  0.00      PROA
ATOM   2789  HE2 PHE P 172      15.768 -11.053   7.411  0.00  0.00      PROA
ATOM   2790  C   PHE P 172      14.718  -6.044   9.058  0.00  0.00      PROA
ATOM   2791  O   PHE P 172      14.797  -6.948   9.881  0.00  0.00      PROA
ATOM   2792  N   ILE P 173      15.408  -4.834   9.231  0.00  0.00      PROA
ATOM   2793  HN  ILE P 173      15.383  -4.117   8.539  0.00  0.00      PROA
ATOM   2794  CA  ILE P 173      16.131  -4.551  10.506  0.00  0.00      PROA
ATOM   2795  HA  ILE P 173      16.718  -5.450  10.621  0.00  0.00      PROA
ATOM   2796  CB  ILE P 173      17.149  -3.424  10.377  0.00  0.00      PROA
ATOM   2797  HB  ILE P 173      16.680  -2.434  10.189  0.00  0.00      PROA
ATOM   2798  CG2 ILE P 173      18.086  -3.343  11.589  0.00  0.00      PROA
ATOM   2799 HG21 ILE P 173      18.669  -2.407  11.456  0.00  0.00      PROA
ATOM   2800 HG22 ILE P 173      18.713  -4.252  11.708  0.00  0.00      PROA
ATOM   2801 HG23 ILE P 173      17.598  -3.119  12.562  0.00  0.00      PROA
ATOM   2802  CG1 ILE P 173      18.127  -3.851   9.224  0.00  0.00      PROA
```

ATOM 2803 HG11 ILE P 173 17.570 -4.210 8.332 0.00 0.00 PROA
ATOM 2804 HG12 ILE P 173 18.704 -4.692 9.666 0.00 0.00 PROA
ATOM 2805 CD ILE P 173 19.128 -2.790 8.703 0.00 0.00 PROA
ATOM 2806 HD1 ILE P 173 19.539 -2.277 9.599 0.00 0.00 PROA
ATOM 2807 HD2 ILE P 173 18.589 -2.063 8.058 0.00 0.00 PROA
ATOM 2808 HD3 ILE P 173 19.926 -3.330 8.149 0.00 0.00 PROA
ATOM 2809 C ILE P 173 15.330 -4.484 11.804 0.00 0.00 PROA
ATOM 2810 O ILE P 173 15.667 -5.036 12.878 0.00 0.00 PROA
ATOM 2811 N PHE P 174 14.091 -3.854 11.833 0.00 0.00 PROA
ATOM 2812 HN PHE P 174 13.690 -3.446 11.017 0.00 0.00 PROA
ATOM 2813 CA PHE P 174 13.415 -3.419 13.041 0.00 0.00 PROA
ATOM 2814 HA PHE P 174 14.117 -3.494 13.859 0.00 0.00 PROA
ATOM 2815 CB PHE P 174 13.057 -1.922 12.859 0.00 0.00 PROA
ATOM 2816 HB1 PHE P 174 12.370 -1.771 13.719 0.00 0.00 PROA
ATOM 2817 HB2 PHE P 174 12.364 -1.838 11.994 0.00 0.00 PROA
ATOM 2818 CG PHE P 174 14.208 -0.992 12.698 0.00 0.00 PROA
ATOM 2819 CD1 PHE P 174 15.154 -1.021 13.695 0.00 0.00 PROA
ATOM 2820 HD1 PHE P 174 15.173 -1.816 14.425 0.00 0.00 PROA
ATOM 2821 CE1 PHE P 174 16.227 -0.033 13.676 0.00 0.00 PROA
ATOM 2822 HE1 PHE P 174 16.986 -0.057 14.443 0.00 0.00 PROA
ATOM 2823 CZ PHE P 174 16.325 0.882 12.643 0.00 0.00 PROA
ATOM 2824 HZ PHE P 174 17.139 1.590 12.688 0.00 0.00 PROA
ATOM 2825 CD2 PHE P 174 14.181 0.051 11.737 0.00 0.00 PROA
ATOM 2826 HD2 PHE P 174 13.325 0.137 11.085 0.00 0.00 PROA
ATOM 2827 CE2 PHE P 174 15.295 0.977 11.672 0.00 0.00 PROA
ATOM 2828 HE2 PHE P 174 15.393 1.789 10.967 0.00 0.00 PROA
ATOM 2829 C PHE P 174 12.248 -4.325 13.372 0.00 0.00 PROA
ATOM 2830 O PHE P 174 11.764 -4.293 14.515 0.00 0.00 PROA
ATOM 2831 N ALA P 175 11.689 -5.100 12.419 0.00 0.00 PROA
ATOM 2832 HN ALA P 175 12.102 -5.167 11.515 0.00 0.00 PROA
ATOM 2833 CA ALA P 175 10.703 -6.071 12.823 0.00 0.00 PROA
ATOM 2834 HA ALA P 175 10.008 -5.525 13.444 0.00 0.00 PROA
ATOM 2835 CB ALA P 175 10.098 -6.631 11.539 0.00 0.00 PROA
ATOM 2836 HB1 ALA P 175 9.096 -7.091 11.671 0.00 0.00 PROA
ATOM 2837 HB2 ALA P 175 10.816 -7.285 11.000 0.00 0.00 PROA
ATOM 2838 HB3 ALA P 175 9.926 -5.895 10.725 0.00 0.00 PROA
ATOM 2839 C ALA P 175 11.423 -7.242 13.556 0.00 0.00 PROA
ATOM 2840 O ALA P 175 12.325 -7.884 13.012 0.00 0.00 PROA
ATOM 2841 N ASN P 176 10.956 -7.378 14.835 0.00 0.00 PROA
ATOM 2842 HN ASN P 176 10.339 -6.736 15.283 0.00 0.00 PROA
ATOM 2843 CA ASN P 176 11.344 -8.415 15.763 0.00 0.00 PROA
ATOM 2844 HA ASN P 176 11.635 -9.331 15.271 0.00 0.00 PROA
ATOM 2845 CB ASN P 176 12.538 -7.910 16.578 0.00 0.00 PROA
ATOM 2846 HB1 ASN P 176 12.717 -8.642 17.394 0.00 0.00 PROA
ATOM 2847 HB2 ASN P 176 12.185 -7.029 17.156 0.00 0.00 PROA
ATOM 2848 CG ASN P 176 13.797 -7.573 15.840 0.00 0.00 PROA
ATOM 2849 OD1 ASN P 176 14.315 -8.479 15.159 0.00 0.00 PROA
ATOM 2850 ND2 ASN P 176 14.256 -6.253 15.888 0.00 0.00 PROA
ATOM 2851 HD21 ASN P 176 15.086 -5.995 15.393 0.00 0.00 PROA
ATOM 2852 HD22 ASN P 176 13.735 -5.458 16.201 0.00 0.00 PROA
ATOM 2853 C ASN P 176 10.201 -8.790 16.684 0.00 0.00 PROA
ATOM 2854 O ASN P 176 9.216 -8.052 16.778 0.00 0.00 PROA
ATOM 2855 N VAL P 177 10.336 -9.970 17.345 0.00 0.00 PROA
ATOM 2856 HN VAL P 177 11.219 -10.430 17.402 0.00 0.00 PROA
ATOM 2857 CA VAL P 177 9.330 -10.461 18.247 0.00 0.00 PROA
ATOM 2858 HA VAL P 177 8.414 -9.965 17.963 0.00 0.00 PROA
ATOM 2859 CB VAL P 177 9.177 -11.980 18.219 0.00 0.00 PROA
ATOM 2860 HB VAL P 177 10.216 -12.364 18.297 0.00 0.00 PROA
ATOM 2861 CG1 VAL P 177 8.347 -12.510 19.454 0.00 0.00 PROA
ATOM 2862 HG11 VAL P 177 7.870 -13.488 19.227 0.00 0.00 PROA
ATOM 2863 HG12 VAL P 177 7.521 -11.820 19.728 0.00 0.00 PROA

```
ATOM 2864 HG13 VAL P 177 8.955 -12.746 20.354 0.00 0.00 PROA
ATOM 2865 CG2 VAL P 177 8.501 -12.360 16.948 0.00 0.00 PROA
ATOM 2866 HG21 VAL P 177 8.572 -13.450 16.743 0.00 0.00 PROA
ATOM 2867 HG22 VAL P 177 8.997 -11.961 16.037 0.00 0.00 PROA
ATOM 2868 HG23 VAL P 177 7.455 -11.988 16.923 0.00 0.00 PROA
ATOM 2869 C VAL P 177 9.642 -9.964 19.652 0.00 0.00 PROA
ATOM 2870 O VAL P 177 10.811 -10.025 20.076 0.00 0.00 PROA
ATOM 2871 N SER P 178 8.637 -9.546 20.358 0.00 0.00 PROA
ATOM 2872 HN SER P 178 7.722 -9.578 19.965 0.00 0.00 PROA
ATOM 2873 CA SER P 178 8.763 -9.083 21.754 0.00 0.00 PROA
ATOM 2874 HA SER P 178 9.789 -9.324 21.990 0.00 0.00 PROA
ATOM 2875 CB SER P 178 8.431 -7.611 21.814 0.00 0.00 PROA
ATOM 2876 HB1 SER P 178 7.338 -7.442 21.713 0.00 0.00 PROA
ATOM 2877 HB2 SER P 178 8.774 -7.096 20.891 0.00 0.00 PROA
ATOM 2878 OG SER P 178 8.773 -7.056 23.020 0.00 0.00 PROA
ATOM 2879 HG1 SER P 178 9.726 -7.067 23.136 0.00 0.00 PROA
ATOM 2880 C SER P 178 7.844 -9.994 22.550 0.00 0.00 PROA
ATOM 2881 O SER P 178 6.677 -9.962 22.193 0.00 0.00 PROA
ATOM 2882 N GLU P 179 8.359 -10.731 23.580 0.00 0.00 PROA
ATOM 2883 HN GLU P 179 9.356 -10.717 23.608 0.00 0.00 PROA
ATOM 2884 CA GLU P 179 7.557 -11.558 24.556 0.00 0.00 PROA
ATOM 2885 HA GLU P 179 6.876 -12.258 24.095 0.00 0.00 PROA
ATOM 2886 CB GLU P 179 8.484 -12.577 25.271 0.00 0.00 PROA
ATOM 2887 HB1 GLU P 179 8.192 -12.947 26.277 0.00 0.00 PROA
ATOM 2888 HB2 GLU P 179 9.365 -12.020 25.655 0.00 0.00 PROA
ATOM 2889 CG GLU P 179 9.009 -13.742 24.365 0.00 0.00 PROA
ATOM 2890 HG1 GLU P 179 9.184 -13.360 23.336 0.00 0.00 PROA
ATOM 2891 HG2 GLU P 179 8.170 -14.471 24.348 0.00 0.00 PROA
ATOM 2892 CD GLU P 179 10.267 -14.418 24.882 0.00 0.00 PROA
ATOM 2893 OE1 GLU P 179 11.328 -14.205 24.206 0.00 0.00 PROA
ATOM 2894 OE2 GLU P 179 10.248 -15.035 25.958 0.00 0.00 PROA
ATOM 2895 C GLU P 179 6.799 -10.705 25.525 0.00 0.00 PROA
ATOM 2896 O GLU P 179 7.447 -10.154 26.419 0.00 0.00 PROA
ATOM 2897 N ALA P 180 5.433 -10.646 25.460 0.00 0.00 PROA
ATOM 2898 HN ALA P 180 4.896 -11.112 24.761 0.00 0.00 PROA
ATOM 2899 CA ALA P 180 4.604 -10.355 26.625 0.00 0.00 PROA
ATOM 2900 HA ALA P 180 5.138 -9.846 27.413 0.00 0.00 PROA
ATOM 2901 CB ALA P 180 3.321 -9.444 26.266 0.00 0.00 PROA
ATOM 2902 HB1 ALA P 180 2.768 -9.206 27.199 0.00 0.00 PROA
ATOM 2903 HB2 ALA P 180 2.508 -9.955 25.708 0.00 0.00 PROA
ATOM 2904 HB3 ALA P 180 3.579 -8.481 25.774 0.00 0.00 PROA
ATOM 2905 C ALA P 180 4.253 -11.681 27.238 0.00 0.00 PROA
ATOM 2906 O ALA P 180 4.586 -12.745 26.752 0.00 0.00 PROA
ATOM 2907 N ASP P 181 3.591 -11.592 28.388 0.00 0.00 PROA
ATOM 2908 HN ASP P 181 3.506 -10.721 28.864 0.00 0.00 PROA
ATOM 2909 CA ASP P 181 3.112 -12.679 29.226 0.00 0.00 PROA
ATOM 2910 HA ASP P 181 3.931 -13.380 29.297 0.00 0.00 PROA
ATOM 2911 CB ASP P 181 2.455 -12.250 30.613 0.00 0.00 PROA
ATOM 2912 HB1 ASP P 181 2.000 -13.139 31.100 0.00 0.00 PROA
ATOM 2913 HB2 ASP P 181 1.747 -11.448 30.312 0.00 0.00 PROA
ATOM 2914 CG ASP P 181 3.460 -11.716 31.578 0.00 0.00 PROA
ATOM 2915 OD1 ASP P 181 4.673 -11.662 31.295 0.00 0.00 PROA
ATOM 2916 OD2 ASP P 181 3.122 -11.361 32.728 0.00 0.00 PROA
ATOM 2917 C ASP P 181 2.065 -13.458 28.441 0.00 0.00 PROA
ATOM 2918 O ASP P 181 0.915 -13.050 28.198 0.00 0.00 PROA
ATOM 2919 N ASP P 182 2.596 -14.611 28.013 0.00 0.00 PROA
ATOM 2920 HN ASP P 182 3.498 -14.904 28.320 0.00 0.00 PROA
ATOM 2921 CA ASP P 182 1.825 -15.646 27.288 0.00 0.00 PROA
ATOM 2922 HA ASP P 182 2.543 -16.409 27.026 0.00 0.00 PROA
ATOM 2923 CB ASP P 182 0.647 -16.113 28.209 0.00 0.00 PROA
ATOM 2924 HB1 ASP P 182 -0.010 -16.875 27.737 0.00 0.00 PROA
```

ATOM	2925	HB2	ASP	P	182	0.010	-15.249	28.499	0.00	0.00	PROA
ATOM	2926	CG	ASP	P	182	1.092	-16.742	29.498	0.00	0.00	PROA
ATOM	2927	OD1	ASP	P	182	0.364	-17.158	30.411	0.00	0.00	PROA
ATOM	2928	OD2	ASP	P	182	2.338	-16.813	29.672	0.00	0.00	PROA
ATOM	2929	C	ASP	P	182	1.347	-15.082	25.940	0.00	0.00	PROA
ATOM	2930	O	ASP	P	182	0.394	-15.515	25.330	0.00	0.00	PROA
ATOM	2931	N	ARG	P	183	2.150	-14.035	25.416	0.00	0.00	PROA
ATOM	2932	HN	ARG	P	183	2.914	-13.798	26.011	0.00	0.00	PROA
ATOM	2933	CA	ARG	P	183	1.892	-13.517	24.060	0.00	0.00	PROA
ATOM	2934	HA	ARG	P	183	1.509	-14.409	23.586	0.00	0.00	PROA
ATOM	2935	CB	ARG	P	183	0.917	-12.304	23.995	0.00	0.00	PROA
ATOM	2936	HB1	ARG	P	183	1.532	-11.390	24.137	0.00	0.00	PROA
ATOM	2937	HB2	ARG	P	183	0.299	-12.421	24.911	0.00	0.00	PROA
ATOM	2938	CG	ARG	P	183	0.182	-12.036	22.716	0.00	0.00	PROA
ATOM	2939	HG1	ARG	P	183	0.929	-11.824	21.921	0.00	0.00	PROA
ATOM	2940	HG2	ARG	P	183	-0.404	-11.144	23.025	0.00	0.00	PROA
ATOM	2941	CD	ARG	P	183	-0.785	-13.163	22.281	0.00	0.00	PROA
ATOM	2942	HD1	ARG	P	183	-1.340	-13.630	23.122	0.00	0.00	PROA
ATOM	2943	HD2	ARG	P	183	-0.331	-14.039	21.770	0.00	0.00	PROA
ATOM	2944	NE	ARG	P	183	-1.762	-12.537	21.389	0.00	0.00	PROA
ATOM	2945	HE	ARG	P	183	-2.671	-12.306	21.734	0.00	0.00	PROA
ATOM	2946	CZ	ARG	P	183	-1.400	-12.089	20.181	0.00	0.00	PROA
ATOM	2947	NH1	ARG	P	183	-0.264	-12.376	19.640	0.00	0.00	PROA
ATOM	2948	HH11	ARG	P	183	0.493	-12.763	20.168	0.00	0.00	PROA
ATOM	2949	HH12	ARG	P	183	-0.104	-11.938	18.756	0.00	0.00	PROA
ATOM	2950	NH2	ARG	P	183	-2.279	-11.396	19.475	0.00	0.00	PROA
ATOM	2951	HH21	ARG	P	183	-3.189	-11.676	19.781	0.00	0.00	PROA
ATOM	2952	HH22	ARG	P	183	-2.175	-11.230	18.494	0.00	0.00	PROA
ATOM	2953	C	ARG	P	183	3.222	-13.132	23.343	0.00	0.00	PROA
ATOM	2954	O	ARG	P	183	3.956	-12.139	23.683	0.00	0.00	PROA
ATOM	2955	N	TYR	P	184	3.418	-13.740	22.225	0.00	0.00	PROA
ATOM	2956	HN	TYR	P	184	2.748	-14.356	21.817	0.00	0.00	PROA
ATOM	2957	CA	TYR	P	184	4.465	-13.237	21.320	0.00	0.00	PROA
ATOM	2958	HA	TYR	P	184	5.344	-12.880	21.834	0.00	0.00	PROA
ATOM	2959	CB	TYR	P	184	4.942	-14.298	20.257	0.00	0.00	PROA
ATOM	2960	HB1	TYR	P	184	5.639	-13.841	19.523	0.00	0.00	PROA
ATOM	2961	HB2	TYR	P	184	4.072	-14.766	19.748	0.00	0.00	PROA
ATOM	2962	CG	TYR	P	184	5.554	-15.488	20.933	0.00	0.00	PROA
ATOM	2963	CD1	TYR	P	184	5.235	-16.867	20.610	0.00	0.00	PROA
ATOM	2964	HD1	TYR	P	184	4.448	-17.080	19.903	0.00	0.00	PROA
ATOM	2965	CE1	TYR	P	184	5.939	-17.943	21.121	0.00	0.00	PROA
ATOM	2966	HE1	TYR	P	184	5.566	-18.927	20.876	0.00	0.00	PROA
ATOM	2967	CZ	TYR	P	184	7.068	-17.738	21.873	0.00	0.00	PROA
ATOM	2968	OH	TYR	P	184	7.987	-18.721	22.123	0.00	0.00	PROA
ATOM	2969	HH	TYR	P	184	7.668	-19.502	21.664	0.00	0.00	PROA
ATOM	2970	CD2	TYR	P	184	6.605	-15.337	21.802	0.00	0.00	PROA
ATOM	2971	HD2	TYR	P	184	7.017	-14.358	21.998	0.00	0.00	PROA
ATOM	2972	CE2	TYR	P	184	7.417	-16.429	22.257	0.00	0.00	PROA
ATOM	2973	HE2	TYR	P	184	8.335	-16.263	22.801	0.00	0.00	PROA
ATOM	2974	C	TYR	P	184	3.903	-12.122	20.411	0.00	0.00	PROA
ATOM	2975	O	TYR	P	184	2.881	-12.344	19.785	0.00	0.00	PROA
ATOM	2976	N	ILE	P	185	4.483	-10.946	20.399	0.00	0.00	PROA
ATOM	2977	HN	ILE	P	185	5.185	-10.827	21.096	0.00	0.00	PROA
ATOM	2978	CA	ILE	P	185	4.116	-9.797	19.619	0.00	0.00	PROA
ATOM	2979	HA	ILE	P	185	3.174	-10.003	19.132	0.00	0.00	PROA
ATOM	2980	CB	ILE	P	185	3.798	-8.579	20.503	0.00	0.00	PROA
ATOM	2981	HB	ILE	P	185	4.709	-8.528	21.137	0.00	0.00	PROA
ATOM	2982	CG2	ILE	P	185	3.665	-7.305	19.762	0.00	0.00	PROA
ATOM	2983	HG21	ILE	P	185	4.648	-6.919	19.417	0.00	0.00	PROA
ATOM	2984	HG22	ILE	P	185	3.335	-6.556	20.514	0.00	0.00	PROA
ATOM	2985	HG23	ILE	P	185	2.974	-7.271	18.893	0.00	0.00	PROA

ATOM	2986	CG1	ILE	P	185	2.550	-8.834	21.404	0.00	0.00	PROA
ATOM	2987	HG11	ILE	P	185	2.438	-7.994	22.122	0.00	0.00	PROA
ATOM	2988	HG12	ILE	P	185	2.666	-9.780	21.976	0.00	0.00	PROA
ATOM	2989	CD	ILE	P	185	1.198	-8.934	20.632	0.00	0.00	PROA
ATOM	2990	HD1	ILE	P	185	0.222	-9.010	21.158	0.00	0.00	PROA
ATOM	2991	HD2	ILE	P	185	1.358	-9.843	20.013	0.00	0.00	PROA
ATOM	2992	HD3	ILE	P	185	1.104	-8.083	19.924	0.00	0.00	PROA
ATOM	2993	C	ILE	P	185	5.089	-9.503	18.531	0.00	0.00	PROA
ATOM	2994	O	ILE	P	185	6.264	-9.245	18.784	0.00	0.00	PROA
ATOM	2995	N	CYS	P	186	4.656	-9.512	17.212	0.00	0.00	PROA
ATOM	2996	HN	CYS	P	186	3.715	-9.666	16.920	0.00	0.00	PROA
ATOM	2997	CA	CYS	P	186	5.529	-9.319	16.090	0.00	0.00	PROA
ATOM	2998	HA	CYS	P	186	6.562	-9.469	16.369	0.00	0.00	PROA
ATOM	2999	CB	CYS	P	186	5.355	-10.313	14.931	0.00	0.00	PROA
ATOM	3000	HB1	CYS	P	186	4.267	-10.436	14.740	0.00	0.00	PROA
ATOM	3001	HB2	CYS	P	186	5.837	-11.247	15.292	0.00	0.00	PROA
ATOM	3002	SG	CYS	P	186	6.095	-9.779	13.307	0.00	0.00	PROA
ATOM	3003	C	CYS	P	186	5.285	-7.861	15.573	0.00	0.00	PROA
ATOM	3004	O	CYS	P	186	4.113	-7.489	15.289	0.00	0.00	PROA
ATOM	3005	N	ASP	P	187	6.361	-7.048	15.458	0.00	0.00	PROA
ATOM	3006	HN	ASP	P	187	7.260	-7.372	15.742	0.00	0.00	PROA
ATOM	3007	CA	ASP	P	187	6.164	-5.649	15.242	0.00	0.00	PROA
ATOM	3008	HA	ASP	P	187	5.610	-5.542	14.321	0.00	0.00	PROA
ATOM	3009	CB	ASP	P	187	5.272	-4.934	16.439	0.00	0.00	PROA
ATOM	3010	HB1	ASP	P	187	5.894	-4.962	17.359	0.00	0.00	PROA
ATOM	3011	HB2	ASP	P	187	4.315	-5.473	16.606	0.00	0.00	PROA
ATOM	3012	CG	ASP	P	187	4.871	-3.510	16.005	0.00	0.00	PROA
ATOM	3013	OD1	ASP	P	187	4.315	-2.786	16.875	0.00	0.00	PROA
ATOM	3014	OD2	ASP	P	187	4.844	-3.176	14.750	0.00	0.00	PROA
ATOM	3015	C	ASP	P	187	7.537	-4.969	15.074	0.00	0.00	PROA
ATOM	3016	O	ASP	P	187	8.593	-5.518	15.456	0.00	0.00	PROA
ATOM	3017	N	ARG	P	188	7.569	-3.802	14.398	0.00	0.00	PROA
ATOM	3018	HN	ARG	P	188	6.659	-3.466	14.166	0.00	0.00	PROA
ATOM	3019	CA	ARG	P	188	8.699	-2.926	14.299	0.00	0.00	PROA
ATOM	3020	HA	ARG	P	188	9.562	-3.453	13.920	0.00	0.00	PROA
ATOM	3021	CB	ARG	P	188	8.415	-1.808	13.286	0.00	0.00	PROA
ATOM	3022	HB1	ARG	P	188	9.312	-1.153	13.245	0.00	0.00	PROA
ATOM	3023	HB2	ARG	P	188	7.526	-1.244	13.641	0.00	0.00	PROA
ATOM	3024	CG	ARG	P	188	7.961	-2.184	11.847	0.00	0.00	PROA
ATOM	3025	HG1	ARG	P	188	6.966	-2.665	11.967	0.00	0.00	PROA
ATOM	3026	HG2	ARG	P	188	8.607	-2.965	11.391	0.00	0.00	PROA
ATOM	3027	CD	ARG	P	188	7.983	-0.983	10.866	0.00	0.00	PROA
ATOM	3028	HD1	ARG	P	188	9.065	-0.733	10.865	0.00	0.00	PROA
ATOM	3029	HD2	ARG	P	188	7.408	-0.141	11.307	0.00	0.00	PROA
ATOM	3030	NE	ARG	P	188	7.693	-1.502	9.530	0.00	0.00	PROA
ATOM	3031	HE	ARG	P	188	8.352	-1.434	8.781	0.00	0.00	PROA
ATOM	3032	CZ	ARG	P	188	6.420	-1.649	9.083	0.00	0.00	PROA
ATOM	3033	NH1	ARG	P	188	5.429	-1.684	9.884	0.00	0.00	PROA
ATOM	3034	HH11	ARG	P	188	5.614	-1.676	10.866	0.00	0.00	PROA
ATOM	3035	HH12	ARG	P	188	4.530	-2.001	9.580	0.00	0.00	PROA
ATOM	3036	NH2	ARG	P	188	6.178	-1.946	7.858	0.00	0.00	PROA
ATOM	3037	HH21	ARG	P	188	6.906	-2.198	7.220	0.00	0.00	PROA
ATOM	3038	HH22	ARG	P	188	5.258	-2.314	7.728	0.00	0.00	PROA
ATOM	3039	C	ARG	P	188	9.036	-2.288	15.646	0.00	0.00	PROA
ATOM	3040	O	ARG	P	188	8.155	-1.754	16.417	0.00	0.00	PROA
ATOM	3041	N	PHE	P	189	10.334	-2.399	15.958	0.00	0.00	PROA
ATOM	3042	HN	PHE	P	189	10.970	-2.933	15.406	0.00	0.00	PROA
ATOM	3043	CA	PHE	P	189	10.823	-1.775	17.183	0.00	0.00	PROA
ATOM	3044	HA	PHE	P	189	10.105	-1.018	17.463	0.00	0.00	PROA
ATOM	3045	CB	PHE	P	189	11.055	-2.865	18.281	0.00	0.00	PROA
ATOM	3046	HB1	PHE	P	189	11.620	-3.673	17.768	0.00	0.00	PROA

ATOM	3047	HB2	PHE	P	189	10.117	-3.337	18.642	0.00	0.00	PROA
ATOM	3048	CG	PHE	P	189	11.727	-2.426	19.529	0.00	0.00	PROA
ATOM	3049	CD1	PHE	P	189	10.860	-1.918	20.542	0.00	0.00	PROA
ATOM	3050	HD1	PHE	P	189	9.828	-1.738	20.281	0.00	0.00	PROA
ATOM	3051	CE1	PHE	P	189	11.474	-1.558	21.749	0.00	0.00	PROA
ATOM	3052	HE1	PHE	P	189	10.777	-1.250	22.515	0.00	0.00	PROA
ATOM	3053	CZ	PHE	P	189	12.836	-1.697	21.948	0.00	0.00	PROA
ATOM	3054	HZ	PHE	P	189	13.144	-1.198	22.854	0.00	0.00	PROA
ATOM	3055	CD2	PHE	P	189	13.090	-2.604	19.660	0.00	0.00	PROA
ATOM	3056	HD2	PHE	P	189	13.726	-3.050	18.909	0.00	0.00	PROA
ATOM	3057	CE2	PHE	P	189	13.720	-2.252	20.876	0.00	0.00	PROA
ATOM	3058	HE2	PHE	P	189	14.793	-2.324	20.979	0.00	0.00	PROA
ATOM	3059	C	PHE	P	189	12.067	-0.918	16.842	0.00	0.00	PROA
ATOM	3060	O	PHE	P	189	13.086	-1.369	16.336	0.00	0.00	PROA
ATOM	3061	N	TYR	P	190	11.967	0.335	17.157	0.00	0.00	PROA
ATOM	3062	HN	TYR	P	190	11.175	0.553	17.721	0.00	0.00	PROA
ATOM	3063	CA	TYR	P	190	12.866	1.397	16.785	0.00	0.00	PROA
ATOM	3064	HA	TYR	P	190	13.597	0.901	16.164	0.00	0.00	PROA
ATOM	3065	CB	TYR	P	190	12.103	2.557	15.989	0.00	0.00	PROA
ATOM	3066	HB1	TYR	P	190	12.882	3.268	15.641	0.00	0.00	PROA
ATOM	3067	HB2	TYR	P	190	11.455	3.087	16.720	0.00	0.00	PROA
ATOM	3068	CG	TYR	P	190	11.358	2.190	14.778	0.00	0.00	PROA
ATOM	3069	CD1	TYR	P	190	11.999	1.738	13.605	0.00	0.00	PROA
ATOM	3070	HD1	TYR	P	190	13.059	1.546	13.674	0.00	0.00	PROA
ATOM	3071	CE1	TYR	P	190	11.307	1.613	12.420	0.00	0.00	PROA
ATOM	3072	HE1	TYR	P	190	11.937	1.331	11.589	0.00	0.00	PROA
ATOM	3073	CZ	TYR	P	190	9.968	1.896	12.437	0.00	0.00	PROA
ATOM	3074	OH	TYR	P	190	9.380	1.723	11.153	0.00	0.00	PROA
ATOM	3075	HH	TYR	P	190	10.037	1.451	10.509	0.00	0.00	PROA
ATOM	3076	CD2	TYR	P	190	9.987	2.385	14.761	0.00	0.00	PROA
ATOM	3077	HD2	TYR	P	190	9.437	2.648	15.652	0.00	0.00	PROA
ATOM	3078	CE2	TYR	P	190	9.214	2.000	13.632	0.00	0.00	PROA
ATOM	3079	HE2	TYR	P	190	8.136	2.048	13.586	0.00	0.00	PROA
ATOM	3080	C	TYR	P	190	13.454	1.917	18.058	0.00	0.00	PROA
ATOM	3081	O	TYR	P	190	12.844	1.640	19.118	0.00	0.00	PROA
ATOM	3082	N	PRO	P	191	14.612	2.570	18.095	0.00	0.00	PROA
ATOM	3083	CD	PRO	P	191	15.483	2.851	16.943	0.00	0.00	PROA
ATOM	3084	HD1	PRO	P	191	14.854	3.390	16.203	0.00	0.00	PROA
ATOM	3085	HD2	PRO	P	191	15.990	1.899	16.676	0.00	0.00	PROA
ATOM	3086	CA	PRO	P	191	15.117	3.164	19.320	0.00	0.00	PROA
ATOM	3087	HA	PRO	P	191	15.085	2.444	20.124	0.00	0.00	PROA
ATOM	3088	CB	PRO	P	191	16.538	3.512	18.894	0.00	0.00	PROA
ATOM	3089	HB1	PRO	P	191	17.149	2.595	19.036	0.00	0.00	PROA
ATOM	3090	HB2	PRO	P	191	17.019	4.303	19.509	0.00	0.00	PROA
ATOM	3091	CG	PRO	P	191	16.449	3.923	17.386	0.00	0.00	PROA
ATOM	3092	HG1	PRO	P	191	16.002	4.921	17.189	0.00	0.00	PROA
ATOM	3093	HG2	PRO	P	191	17.382	3.746	16.809	0.00	0.00	PROA
ATOM	3094	C	PRO	P	191	14.349	4.412	19.771	0.00	0.00	PROA
ATOM	3095	O	PRO	P	191	14.542	4.846	20.894	0.00	0.00	PROA
ATOM	3096	N	ASN	P	192	13.579	5.107	18.930	0.00	0.00	PROA
ATOM	3097	HN	ASN	P	192	13.351	4.684	18.056	0.00	0.00	PROA
ATOM	3098	CA	ASN	P	192	13.039	6.386	19.207	0.00	0.00	PROA
ATOM	3099	HA	ASN	P	192	12.720	6.316	20.237	0.00	0.00	PROA
ATOM	3100	CB	ASN	P	192	14.162	7.468	19.145	0.00	0.00	PROA
ATOM	3101	HB1	ASN	P	192	15.011	7.254	19.829	0.00	0.00	PROA
ATOM	3102	HB2	ASN	P	192	13.631	8.387	19.473	0.00	0.00	PROA
ATOM	3103	CG	ASN	P	192	14.596	7.769	17.691	0.00	0.00	PROA
ATOM	3104	OD1	ASN	P	192	13.788	7.598	16.742	0.00	0.00	PROA
ATOM	3105	ND2	ASN	P	192	15.900	8.128	17.410	0.00	0.00	PROA
ATOM	3106	HD21	ASN	P	192	16.087	8.416	16.471	0.00	0.00	PROA
ATOM	3107	HD22	ASN	P	192	16.544	8.166	18.174	0.00	0.00	PROA

ATOM	3108	C	ASN	P	192	11.759	6.648	18.393	0.00	0.00	PROA
ATOM	3109	O	ASN	P	192	11.415	5.854	17.490	0.00	0.00	PROA
ATOM	3110	N	ASP	P	193	10.909	7.667	18.735	0.00	0.00	PROA
ATOM	3111	HN	ASP	P	193	10.998	8.284	19.513	0.00	0.00	PROA
ATOM	3112	CA	ASP	P	193	9.651	7.821	18.029	0.00	0.00	PROA
ATOM	3113	HA	ASP	P	193	9.203	6.919	17.638	0.00	0.00	PROA
ATOM	3114	CB	ASP	P	193	8.631	8.443	18.917	0.00	0.00	PROA
ATOM	3115	HB1	ASP	P	193	7.625	8.653	18.495	0.00	0.00	PROA
ATOM	3116	HB2	ASP	P	193	8.970	9.440	19.269	0.00	0.00	PROA
ATOM	3117	CG	ASP	P	193	8.458	7.576	20.228	0.00	0.00	PROA
ATOM	3118	OD1	ASP	P	193	8.507	6.336	20.179	0.00	0.00	PROA
ATOM	3119	OD2	ASP	P	193	8.391	8.135	21.297	0.00	0.00	PROA
ATOM	3120	C	ASP	P	193	9.850	8.746	16.861	0.00	0.00	PROA
ATOM	3121	O	ASP	P	193	8.970	9.035	16.045	0.00	0.00	PROA
ATOM	3122	N	LEU	P	194	11.041	9.367	16.705	0.00	0.00	PROA
ATOM	3123	HN	LEU	P	194	11.780	9.156	17.340	0.00	0.00	PROA
ATOM	3124	CA	LEU	P	194	11.395	10.266	15.685	0.00	0.00	PROA
ATOM	3125	HA	LEU	P	194	10.560	10.950	15.648	0.00	0.00	PROA
ATOM	3126	CB	LEU	P	194	12.747	10.918	16.070	0.00	0.00	PROA
ATOM	3127	HB1	LEU	P	194	13.443	10.150	16.469	0.00	0.00	PROA
ATOM	3128	HB2	LEU	P	194	12.524	11.612	16.908	0.00	0.00	PROA
ATOM	3129	CG	LEU	P	194	13.526	11.680	14.954	0.00	0.00	PROA
ATOM	3130	HG	LEU	P	194	13.846	11.034	14.108	0.00	0.00	PROA
ATOM	3131	CD1	LEU	P	194	12.767	12.860	14.365	0.00	0.00	PROA
ATOM	3132	HD11	LEU	P	194	13.330	13.360	13.548	0.00	0.00	PROA
ATOM	3133	HD12	LEU	P	194	12.562	13.565	15.199	0.00	0.00	PROA
ATOM	3134	HD13	LEU	P	194	11.826	12.529	13.877	0.00	0.00	PROA
ATOM	3135	CD2	LEU	P	194	14.946	12.193	15.478	0.00	0.00	PROA
ATOM	3136	HD21	LEU	P	194	14.939	12.951	16.291	0.00	0.00	PROA
ATOM	3137	HD22	LEU	P	194	15.512	12.704	14.670	0.00	0.00	PROA
ATOM	3138	HD23	LEU	P	194	15.580	11.296	15.645	0.00	0.00	PROA
ATOM	3139	C	LEU	P	194	11.518	9.397	14.395	0.00	0.00	PROA
ATOM	3140	O	LEU	P	194	11.062	9.781	13.327	0.00	0.00	PROA
ATOM	3141	N	TRP	P	195	11.906	8.130	14.550	0.00	0.00	PROA
ATOM	3142	HN	TRP	P	195	12.398	7.868	15.377	0.00	0.00	PROA
ATOM	3143	CA	TRP	P	195	11.703	7.160	13.437	0.00	0.00	PROA
ATOM	3144	HA	TRP	P	195	12.149	7.639	12.578	0.00	0.00	PROA
ATOM	3145	CB	TRP	P	195	12.276	5.735	13.813	0.00	0.00	PROA
ATOM	3146	HB1	TRP	P	195	11.649	4.859	13.540	0.00	0.00	PROA
ATOM	3147	HB2	TRP	P	195	12.220	5.678	14.921	0.00	0.00	PROA
ATOM	3148	CG	TRP	P	195	13.661	5.459	13.315	0.00	0.00	PROA
ATOM	3149	CD1	TRP	P	195	14.772	5.459	14.183	0.00	0.00	PROA
ATOM	3150	HD1	TRP	P	195	14.765	5.640	15.247	0.00	0.00	PROA
ATOM	3151	NE1	TRP	P	195	15.885	5.230	13.409	0.00	0.00	PROA
ATOM	3152	HE1	TRP	P	195	16.616	4.712	13.796	0.00	0.00	PROA
ATOM	3153	CE2	TRP	P	195	15.557	5.086	12.039	0.00	0.00	PROA
ATOM	3154	CD2	TRP	P	195	14.183	5.247	12.000	0.00	0.00	PROA
ATOM	3155	CE3	TRP	P	195	13.554	5.242	10.750	0.00	0.00	PROA
ATOM	3156	HE3	TRP	P	195	12.481	5.289	10.635	0.00	0.00	PROA
ATOM	3157	CZ3	TRP	P	195	14.356	5.103	9.662	0.00	0.00	PROA
ATOM	3158	HZ3	TRP	P	195	14.089	5.117	8.615	0.00	0.00	PROA
ATOM	3159	CZ2	TRP	P	195	16.379	5.044	10.929	0.00	0.00	PROA
ATOM	3160	HZ2	TRP	P	195	17.433	5.030	11.167	0.00	0.00	PROA
ATOM	3161	CH2	TRP	P	195	15.768	5.081	9.774	0.00	0.00	PROA
ATOM	3162	HH2	TRP	P	195	16.411	5.075	8.906	0.00	0.00	PROA
ATOM	3163	C	TRP	P	195	10.313	6.862	12.956	0.00	0.00	PROA
ATOM	3164	O	TRP	P	195	10.015	6.762	11.776	0.00	0.00	PROA
ATOM	3165	N	VAL	P	196	9.335	6.767	13.914	0.00	0.00	PROA
ATOM	3166	HN	VAL	P	196	9.578	6.901	14.871	0.00	0.00	PROA
ATOM	3167	CA	VAL	P	196	7.934	6.527	13.695	0.00	0.00	PROA
ATOM	3168	HA	VAL	P	196	7.885	5.677	13.031	0.00	0.00	PROA

ATOM	3169	CB	VAL	P	196	7.128	6.280	14.974	0.00	0.00	PROA
ATOM	3170	HB	VAL	P	196	7.202	7.136	15.679	0.00	0.00	PROA
ATOM	3171	CG1	VAL	P	196	5.650	6.045	14.636	0.00	0.00	PROA
ATOM	3172	HG11	VAL	P	196	5.408	5.496	13.701	0.00	0.00	PROA
ATOM	3173	HG12	VAL	P	196	5.053	6.982	14.636	0.00	0.00	PROA
ATOM	3174	HG13	VAL	P	196	5.192	5.412	15.426	0.00	0.00	PROA
ATOM	3175	CG2	VAL	P	196	7.680	4.994	15.622	0.00	0.00	PROA
ATOM	3176	HG21	VAL	P	196	7.615	4.184	14.863	0.00	0.00	PROA
ATOM	3177	HG22	VAL	P	196	6.943	4.840	16.439	0.00	0.00	PROA
ATOM	3178	HG23	VAL	P	196	8.657	5.085	16.143	0.00	0.00	PROA
ATOM	3179	C	VAL	P	196	7.393	7.688	12.868	0.00	0.00	PROA
ATOM	3180	O	VAL	P	196	6.582	7.430	11.971	0.00	0.00	PROA
ATOM	3181	N	VAL	P	197	7.738	8.979	13.125	0.00	0.00	PROA
ATOM	3182	HN	VAL	P	197	8.416	9.100	13.847	0.00	0.00	PROA
ATOM	3183	CA	VAL	P	197	7.416	10.082	12.233	0.00	0.00	PROA
ATOM	3184	HA	VAL	P	197	6.358	10.002	12.034	0.00	0.00	PROA
ATOM	3185	CB	VAL	P	197	7.877	11.399	12.786	0.00	0.00	PROA
ATOM	3186	HB	VAL	P	197	8.925	11.489	13.143	0.00	0.00	PROA
ATOM	3187	CG1	VAL	P	197	7.740	12.579	11.814	0.00	0.00	PROA
ATOM	3188	HG11	VAL	P	197	8.432	12.552	10.945	0.00	0.00	PROA
ATOM	3189	HG12	VAL	P	197	7.908	13.534	12.357	0.00	0.00	PROA
ATOM	3190	HG13	VAL	P	197	6.784	12.600	11.250	0.00	0.00	PROA
ATOM	3191	CG2	VAL	P	197	7.066	11.795	14.096	0.00	0.00	PROA
ATOM	3192	HG21	VAL	P	197	5.974	11.645	13.955	0.00	0.00	PROA
ATOM	3193	HG22	VAL	P	197	7.259	12.783	14.566	0.00	0.00	PROA
ATOM	3194	HG23	VAL	P	197	7.310	10.987	14.817	0.00	0.00	PROA
ATOM	3195	C	VAL	P	197	7.924	9.890	10.824	0.00	0.00	PROA
ATOM	3196	O	VAL	P	197	7.223	10.024	9.821	0.00	0.00	PROA
ATOM	3197	N	VAL	P	198	9.230	9.502	10.722	0.00	0.00	PROA
ATOM	3198	HN	VAL	P	198	9.851	9.562	11.499	0.00	0.00	PROA
ATOM	3199	CA	VAL	P	198	9.822	9.194	9.350	0.00	0.00	PROA
ATOM	3200	HA	VAL	P	198	9.789	10.092	8.750	0.00	0.00	PROA
ATOM	3201	CB	VAL	P	198	11.366	8.770	9.463	0.00	0.00	PROA
ATOM	3202	HB	VAL	P	198	11.550	8.017	10.259	0.00	0.00	PROA
ATOM	3203	CG1	VAL	P	198	11.985	8.250	8.157	0.00	0.00	PROA
ATOM	3204	HG11	VAL	P	198	11.671	8.924	7.332	0.00	0.00	PROA
ATOM	3205	HG12	VAL	P	198	11.656	7.205	7.972	0.00	0.00	PROA
ATOM	3206	HG13	VAL	P	198	13.090	8.242	8.277	0.00	0.00	PROA
ATOM	3207	CG2	VAL	P	198	12.005	10.011	9.966	0.00	0.00	PROA
ATOM	3208	HG21	VAL	P	198	11.723	10.139	11.033	0.00	0.00	PROA
ATOM	3209	HG22	VAL	P	198	11.536	10.869	9.439	0.00	0.00	PROA
ATOM	3210	HG23	VAL	P	198	13.101	10.071	9.794	0.00	0.00	PROA
ATOM	3211	C	VAL	P	198	9.162	8.083	8.550	0.00	0.00	PROA
ATOM	3212	O	VAL	P	198	8.936	8.207	7.363	0.00	0.00	PROA
ATOM	3213	N	PHE	P	199	8.870	6.886	9.146	0.00	0.00	PROA
ATOM	3214	HN	PHE	P	199	9.083	6.732	10.108	0.00	0.00	PROA
ATOM	3215	CA	PHE	P	199	8.124	5.835	8.660	0.00	0.00	PROA
ATOM	3216	HA	PHE	P	199	8.558	5.543	7.715	0.00	0.00	PROA
ATOM	3217	CB	PHE	P	199	8.282	4.785	9.820	0.00	0.00	PROA
ATOM	3218	HB1	PHE	P	199	8.100	5.265	10.806	0.00	0.00	PROA
ATOM	3219	HB2	PHE	P	199	9.227	4.202	9.776	0.00	0.00	PROA
ATOM	3220	CG	PHE	P	199	7.260	3.689	9.721	0.00	0.00	PROA
ATOM	3221	CD1	PHE	P	199	6.303	3.576	10.733	0.00	0.00	PROA
ATOM	3222	HD1	PHE	P	199	6.279	4.249	11.578	0.00	0.00	PROA
ATOM	3223	CE1	PHE	P	199	5.305	2.589	10.672	0.00	0.00	PROA
ATOM	3224	HE1	PHE	P	199	4.638	2.512	11.518	0.00	0.00	PROA
ATOM	3225	CZ	PHE	P	199	5.328	1.653	9.637	0.00	0.00	PROA
ATOM	3226	HZ	PHE	P	199	4.507	0.953	9.604	0.00	0.00	PROA
ATOM	3227	CD2	PHE	P	199	7.275	2.720	8.641	0.00	0.00	PROA
ATOM	3228	HD2	PHE	P	199	8.025	2.770	7.865	0.00	0.00	PROA
ATOM	3229	CE2	PHE	P	199	6.304	1.710	8.714	0.00	0.00	PROA

ATOM	3230	HE2	PHE	P	199	6.260	0.950	7.948	0.00	0.00	PROA
ATOM	3231	C	PHE	P	199	6.644	6.190	8.257	0.00	0.00	PROA
ATOM	3232	O	PHE	P	199	6.242	5.938	7.169	0.00	0.00	PROA
ATOM	3233	N	GLN	P	200	5.812	6.920	9.024	0.00	0.00	PROA
ATOM	3234	HN	GLN	P	200	6.054	7.143	9.965	0.00	0.00	PROA
ATOM	3235	CA	GLN	P	200	4.496	7.362	8.566	0.00	0.00	PROA
ATOM	3236	HA	GLN	P	200	4.071	6.489	8.092	0.00	0.00	PROA
ATOM	3237	CB	GLN	P	200	3.687	7.862	9.823	0.00	0.00	PROA
ATOM	3238	HB1	GLN	P	200	2.958	8.617	9.457	0.00	0.00	PROA
ATOM	3239	HB2	GLN	P	200	4.289	8.516	10.489	0.00	0.00	PROA
ATOM	3240	CG	GLN	P	200	3.076	6.786	10.787	0.00	0.00	PROA
ATOM	3241	HG1	GLN	P	200	2.299	7.336	11.359	0.00	0.00	PROA
ATOM	3242	HG2	GLN	P	200	3.863	6.610	11.552	0.00	0.00	PROA
ATOM	3243	CD	GLN	P	200	2.590	5.393	10.318	0.00	0.00	PROA
ATOM	3244	OE1	GLN	P	200	3.186	4.847	9.449	0.00	0.00	PROA
ATOM	3245	NE2	GLN	P	200	1.624	4.844	10.959	0.00	0.00	PROA
ATOM	3246	HE21	GLN	P	200	1.366	3.941	10.616	0.00	0.00	PROA
ATOM	3247	HE22	GLN	P	200	1.438	5.196	11.877	0.00	0.00	PROA
ATOM	3248	C	GLN	P	200	4.552	8.362	7.446	0.00	0.00	PROA
ATOM	3249	O	GLN	P	200	3.669	8.321	6.593	0.00	0.00	PROA
ATOM	3250	N	PHE	P	201	5.432	9.302	7.415	0.00	0.00	PROA
ATOM	3251	HN	PHE	P	201	6.022	9.402	8.212	0.00	0.00	PROA
ATOM	3252	CA	PHE	P	201	5.609	10.332	6.382	0.00	0.00	PROA
ATOM	3253	HA	PHE	P	201	4.614	10.576	6.039	0.00	0.00	PROA
ATOM	3254	CB	PHE	P	201	6.332	11.629	6.850	0.00	0.00	PROA
ATOM	3255	HB1	PHE	P	201	7.354	11.424	7.235	0.00	0.00	PROA
ATOM	3256	HB2	PHE	P	201	5.799	11.912	7.782	0.00	0.00	PROA
ATOM	3257	CG	PHE	P	201	6.400	12.829	5.902	0.00	0.00	PROA
ATOM	3258	CD1	PHE	P	201	7.487	13.770	5.996	0.00	0.00	PROA
ATOM	3259	HD1	PHE	P	201	8.229	13.461	6.717	0.00	0.00	PROA
ATOM	3260	CE1	PHE	P	201	7.496	15.054	5.384	0.00	0.00	PROA
ATOM	3261	HE1	PHE	P	201	8.251	15.803	5.572	0.00	0.00	PROA
ATOM	3262	CZ	PHE	P	201	6.339	15.338	4.613	0.00	0.00	PROA
ATOM	3263	HZ	PHE	P	201	6.170	16.318	4.194	0.00	0.00	PROA
ATOM	3264	CD2	PHE	P	201	5.431	13.105	4.987	0.00	0.00	PROA
ATOM	3265	HD2	PHE	P	201	4.575	12.450	4.911	0.00	0.00	PROA
ATOM	3266	CE2	PHE	P	201	5.360	14.358	4.404	0.00	0.00	PROA
ATOM	3267	HE2	PHE	P	201	4.529	14.462	3.722	0.00	0.00	PROA
ATOM	3268	C	PHE	P	201	6.264	9.788	5.045	0.00	0.00	PROA
ATOM	3269	O	PHE	P	201	6.020	10.170	3.924	0.00	0.00	PROA
ATOM	3270	N	GLN	P	202	7.137	8.686	5.198	0.00	0.00	PROA
ATOM	3271	HN	GLN	P	202	7.565	8.370	6.041	0.00	0.00	PROA
ATOM	3272	CA	GLN	P	202	7.574	7.771	4.131	0.00	0.00	PROA
ATOM	3273	HA	GLN	P	202	8.225	8.351	3.493	0.00	0.00	PROA
ATOM	3274	CB	GLN	P	202	8.559	6.686	4.567	0.00	0.00	PROA
ATOM	3275	HB1	GLN	P	202	8.062	6.295	5.480	0.00	0.00	PROA
ATOM	3276	HB2	GLN	P	202	9.472	7.158	4.990	0.00	0.00	PROA
ATOM	3277	CG	GLN	P	202	9.001	5.624	3.593	0.00	0.00	PROA
ATOM	3278	HG1	GLN	P	202	9.850	5.947	2.954	0.00	0.00	PROA
ATOM	3279	HG2	GLN	P	202	8.250	5.438	2.796	0.00	0.00	PROA
ATOM	3280	CD	GLN	P	202	9.436	4.267	4.261	0.00	0.00	PROA
ATOM	3281	OE1	GLN	P	202	10.577	3.990	4.681	0.00	0.00	PROA
ATOM	3282	NE2	GLN	P	202	8.440	3.364	4.340	0.00	0.00	PROA
ATOM	3283	HE21	GLN	P	202	8.710	2.515	4.795	0.00	0.00	PROA
ATOM	3284	HE22	GLN	P	202	7.567	3.715	4.001	0.00	0.00	PROA
ATOM	3285	C	GLN	P	202	6.463	7.090	3.295	0.00	0.00	PROA
ATOM	3286	O	GLN	P	202	6.550	7.016	2.083	0.00	0.00	PROA
ATOM	3287	N	HSD	P	203	5.356	6.714	3.883	0.00	0.00	PROA
ATOM	3288	HN	HSD	P	203	5.267	6.569	4.865	0.00	0.00	PROA
ATOM	3289	CA	HSD	P	203	4.183	6.269	3.109	0.00	0.00	PROA
ATOM	3290	HA	HSD	P	203	4.522	5.494	2.436	0.00	0.00	PROA

ATOM	3291	CB	HSD	P	203	2.961	5.952	4.014	0.00	0.00	PROA
ATOM	3292	HB1	HSD	P	203	2.040	5.727	3.436	0.00	0.00	PROA
ATOM	3293	HB2	HSD	P	203	2.773	6.781	4.730	0.00	0.00	PROA
ATOM	3294	ND1	HSD	P	203	4.221	4.495	5.720	0.00	0.00	PROA
ATOM	3295	HD1	HSD	P	203	4.792	5.240	6.067	0.00	0.00	PROA
ATOM	3296	CG	HSD	P	203	3.195	4.740	4.850	0.00	0.00	PROA
ATOM	3297	CE1	HSD	P	203	4.175	3.211	6.179	0.00	0.00	PROA
ATOM	3298	HE1	HSD	P	203	4.885	2.847	6.922	0.00	0.00	PROA
ATOM	3299	NE2	HSD	P	203	3.235	2.518	5.557	0.00	0.00	PROA
ATOM	3300	CD2	HSD	P	203	2.626	3.464	4.793	0.00	0.00	PROA
ATOM	3301	HD2	HSD	P	203	1.795	3.142	4.178	0.00	0.00	PROA
ATOM	3302	C	HSD	P	203	3.620	7.420	2.294	0.00	0.00	PROA
ATOM	3303	O	HSD	P	203	3.196	7.235	1.165	0.00	0.00	PROA
ATOM	3304	N	ILE	P	204	3.466	8.591	2.896	0.00	0.00	PROA
ATOM	3305	HN	ILE	P	204	3.853	8.730	3.804	0.00	0.00	PROA
ATOM	3306	CA	ILE	P	204	2.940	9.762	2.176	0.00	0.00	PROA
ATOM	3307	HA	ILE	P	204	1.937	9.582	1.817	0.00	0.00	PROA
ATOM	3308	CB	ILE	P	204	2.673	10.812	3.221	0.00	0.00	PROA
ATOM	3309	HB	ILE	P	204	3.655	10.985	3.711	0.00	0.00	PROA
ATOM	3310	CG2	ILE	P	204	2.095	12.146	2.542	0.00	0.00	PROA
ATOM	3311	HG21	ILE	P	204	1.160	11.911	1.990	0.00	0.00	PROA
ATOM	3312	HG22	ILE	P	204	2.822	12.728	1.936	0.00	0.00	PROA
ATOM	3313	HG23	ILE	P	204	1.626	12.749	3.348	0.00	0.00	PROA
ATOM	3314	CG1	ILE	P	204	1.746	10.341	4.437	0.00	0.00	PROA
ATOM	3315	HG11	ILE	P	204	1.931	11.003	5.309	0.00	0.00	PROA
ATOM	3316	HG12	ILE	P	204	1.985	9.358	4.896	0.00	0.00	PROA
ATOM	3317	CD	ILE	P	204	0.234	10.255	4.034	0.00	0.00	PROA
ATOM	3318	HD1	ILE	P	204	0.010	9.650	3.129	0.00	0.00	PROA
ATOM	3319	HD2	ILE	P	204	-0.214	11.253	3.840	0.00	0.00	PROA
ATOM	3320	HD3	ILE	P	204	-0.294	9.581	4.742	0.00	0.00	PROA
ATOM	3321	C	ILE	P	204	3.746	10.276	1.059	0.00	0.00	PROA
ATOM	3322	O	ILE	P	204	3.305	10.461	-0.119	0.00	0.00	PROA
ATOM	3323	N	MET	P	205	5.041	10.466	1.376	0.00	0.00	PROA
ATOM	3324	HN	MET	P	205	5.396	10.180	2.262	0.00	0.00	PROA
ATOM	3325	CA	MET	P	205	5.989	10.898	0.401	0.00	0.00	PROA
ATOM	3326	HA	MET	P	205	5.573	11.808	-0.004	0.00	0.00	PROA
ATOM	3327	CB	MET	P	205	7.368	11.326	1.007	0.00	0.00	PROA
ATOM	3328	HB1	MET	P	205	8.159	11.341	0.227	0.00	0.00	PROA
ATOM	3329	HB2	MET	P	205	7.714	10.568	1.742	0.00	0.00	PROA
ATOM	3330	CG	MET	P	205	7.316	12.742	1.673	0.00	0.00	PROA
ATOM	3331	HG1	MET	P	205	8.264	12.791	2.251	0.00	0.00	PROA
ATOM	3332	HG2	MET	P	205	6.477	12.911	2.381	0.00	0.00	PROA
ATOM	3333	SD	MET	P	205	7.243	14.146	0.551	0.00	0.00	PROA
ATOM	3334	CE	MET	P	205	8.907	14.151	-0.199	0.00	0.00	PROA
ATOM	3335	HE1	MET	P	205	8.922	15.051	-0.850	0.00	0.00	PROA
ATOM	3336	HE2	MET	P	205	9.096	13.243	-0.810	0.00	0.00	PROA
ATOM	3337	HE3	MET	P	205	9.629	14.093	0.644	0.00	0.00	PROA
ATOM	3338	C	MET	P	205	6.194	9.915	-0.755	0.00	0.00	PROA
ATOM	3339	O	MET	P	205	6.132	10.308	-1.898	0.00	0.00	PROA
ATOM	3340	N	VAL	P	206	6.393	8.627	-0.540	0.00	0.00	PROA
ATOM	3341	HN	VAL	P	206	6.224	8.316	0.392	0.00	0.00	PROA
ATOM	3342	CA	VAL	P	206	6.750	7.617	-1.540	0.00	0.00	PROA
ATOM	3343	HA	VAL	P	206	7.308	8.178	-2.275	0.00	0.00	PROA
ATOM	3344	CB	VAL	P	206	7.736	6.544	-1.088	0.00	0.00	PROA
ATOM	3345	HB	VAL	P	206	7.343	5.955	-0.232	0.00	0.00	PROA
ATOM	3346	CG1	VAL	P	206	8.247	5.755	-2.279	0.00	0.00	PROA
ATOM	3347	HG11	VAL	P	206	7.541	4.900	-2.343	0.00	0.00	PROA
ATOM	3348	HG12	VAL	P	206	9.283	5.355	-2.301	0.00	0.00	PROA
ATOM	3349	HG13	VAL	P	206	8.097	6.255	-3.260	0.00	0.00	PROA
ATOM	3350	CG2	VAL	P	206	9.021	7.283	-0.619	0.00	0.00	PROA
ATOM	3351	HG21	VAL	P	206	9.570	7.725	-1.478	0.00	0.00	PROA

```
ATOM   3352 HG22 VAL P 206       9.828   6.690  -0.138  0.00  0.00      PROA
ATOM   3353 HG23 VAL P 206       8.796   8.086   0.114  0.00  0.00      PROA
ATOM   3354  C   VAL P 206       5.504   6.958  -2.207  0.00  0.00      PROA
ATOM   3355  O   VAL P 206       5.615   6.487  -3.311  0.00  0.00      PROA
ATOM   3356  N   GLY P 207       4.353   6.901  -1.466  0.00  0.00      PROA
ATOM   3357  HN  GLY P 207       4.378   7.298  -0.552  0.00  0.00      PROA
ATOM   3358  CA  GLY P 207       3.063   6.281  -1.899  0.00  0.00      PROA
ATOM   3359  HA1 GLY P 207       2.629   5.868  -1.001  0.00  0.00      PROA
ATOM   3360  HA2 GLY P 207       3.259   5.573  -2.691  0.00  0.00      PROA
ATOM   3361  C   GLY P 207       2.018   7.255  -2.345  0.00  0.00      PROA
ATOM   3362  O   GLY P 207       0.937   6.894  -2.874  0.00  0.00      PROA
ATOM   3363  N   LEU P 208       2.299   8.582  -2.096  0.00  0.00      PROA
ATOM   3364  HN  LEU P 208       3.125   8.848  -1.604  0.00  0.00      PROA
ATOM   3365  CA  LEU P 208       1.422   9.662  -2.509  0.00  0.00      PROA
ATOM   3366  HA  LEU P 208       0.676   9.217  -3.151  0.00  0.00      PROA
ATOM   3367  CB  LEU P 208       0.610  10.302  -1.280  0.00  0.00      PROA
ATOM   3368  HB1 LEU P 208       1.099  11.241  -0.944  0.00  0.00      PROA
ATOM   3369  HB2 LEU P 208       0.602   9.546  -0.466  0.00  0.00      PROA
ATOM   3370  CG  LEU P 208      -0.862  10.599  -1.517  0.00  0.00      PROA
ATOM   3371  HG  LEU P 208      -1.362   9.718  -1.973  0.00  0.00      PROA
ATOM   3372  CD1 LEU P 208      -1.470  10.626  -0.111  0.00  0.00      PROA
ATOM   3373 HD11 LEU P 208      -2.532  10.949  -0.154  0.00  0.00      PROA
ATOM   3374 HD12 LEU P 208      -0.977  11.374   0.547  0.00  0.00      PROA
ATOM   3375 HD13 LEU P 208      -1.449   9.616   0.350  0.00  0.00      PROA
ATOM   3376  CD2 LEU P 208      -1.162  11.821  -2.381  0.00  0.00      PROA
ATOM   3377 HD21 LEU P 208      -0.914  11.719  -3.460  0.00  0.00      PROA
ATOM   3378 HD22 LEU P 208      -0.388  12.555  -2.070  0.00  0.00      PROA
ATOM   3379 HD23 LEU P 208      -2.192  12.195  -2.202  0.00  0.00      PROA
ATOM   3380  C   LEU P 208       2.155  10.688  -3.393  0.00  0.00      PROA
ATOM   3381  O   LEU P 208       1.951  10.691  -4.561  0.00  0.00      PROA
ATOM   3382  N   ILE P 209       2.886  11.566  -2.735  0.00  0.00      PROA
ATOM   3383  HN  ILE P 209       3.063  11.401  -1.767  0.00  0.00      PROA
ATOM   3384  CA  ILE P 209       3.462  12.751  -3.356  0.00  0.00      PROA
ATOM   3385  HA  ILE P 209       2.619  13.267  -3.792  0.00  0.00      PROA
ATOM   3386  CB  ILE P 209       4.065  13.668  -2.307  0.00  0.00      PROA
ATOM   3387  HB  ILE P 209       4.795  13.017  -1.781  0.00  0.00      PROA
ATOM   3388  CG2 ILE P 209       4.752  14.903  -3.034  0.00  0.00      PROA
ATOM   3389 HG21 ILE P 209       5.167  15.511  -2.202  0.00  0.00      PROA
ATOM   3390 HG22 ILE P 209       4.035  15.436  -3.695  0.00  0.00      PROA
ATOM   3391 HG23 ILE P 209       5.696  14.606  -3.539  0.00  0.00      PROA
ATOM   3392  CG1 ILE P 209       2.960  14.041  -1.293  0.00  0.00      PROA
ATOM   3393 HG11 ILE P 209       2.516  13.262  -0.637  0.00  0.00      PROA
ATOM   3394 HG12 ILE P 209       2.059  14.293  -1.892  0.00  0.00      PROA
ATOM   3395  CD  ILE P 209       3.302  15.133  -0.309  0.00  0.00      PROA
ATOM   3396  HD1 ILE P 209       4.399  15.244  -0.168  0.00  0.00      PROA
ATOM   3397  HD2 ILE P 209       2.794  14.893   0.650  0.00  0.00      PROA
ATOM   3398  HD3 ILE P 209       2.794  16.061  -0.646  0.00  0.00      PROA
ATOM   3399  C   ILE P 209       4.453  12.471  -4.523  0.00  0.00      PROA
ATOM   3400  O   ILE P 209       4.300  12.963  -5.602  0.00  0.00      PROA
ATOM   3401  N   LEU P 210       5.468  11.652  -4.297  0.00  0.00      PROA
ATOM   3402  HN  LEU P 210       5.562  11.243  -3.393  0.00  0.00      PROA
ATOM   3403  CA  LEU P 210       6.453  11.260  -5.329  0.00  0.00      PROA
ATOM   3404  HA  LEU P 210       6.825  12.163  -5.790  0.00  0.00      PROA
ATOM   3405  CB  LEU P 210       7.716  10.625  -4.716  0.00  0.00      PROA
ATOM   3406  HB1 LEU P 210       8.457  10.499  -5.535  0.00  0.00      PROA
ATOM   3407  HB2 LEU P 210       7.504   9.658  -4.211  0.00  0.00      PROA
ATOM   3408  CG  LEU P 210       8.406  11.446  -3.591  0.00  0.00      PROA
ATOM   3409  HG  LEU P 210       7.680  11.798  -2.828  0.00  0.00      PROA
ATOM   3410  CD1 LEU P 210       9.581  10.739  -2.826  0.00  0.00      PROA
ATOM   3411 HD11 LEU P 210       9.872  11.397  -1.979  0.00  0.00      PROA
ATOM   3412 HD12 LEU P 210      10.456  10.511  -3.471  0.00  0.00      PROA
```

```
ATOM   3413 HD13 LEU P 210       9.209   9.750  -2.485  0.00  0.00      PROA
ATOM   3414  CD2 LEU P 210       8.948  12.710  -4.258  0.00  0.00      PROA
ATOM   3415 HD21 LEU P 210       9.667  12.505  -5.080  0.00  0.00      PROA
ATOM   3416 HD22 LEU P 210       9.544  13.268  -3.505  0.00  0.00      PROA
ATOM   3417 HD23 LEU P 210       8.172  13.393  -4.664  0.00  0.00      PROA
ATOM   3418  C   LEU P 210       5.915  10.614  -6.620  0.00  0.00      PROA
ATOM   3419  O   LEU P 210       6.372  11.000  -7.746  0.00  0.00      PROA
ATOM   3420  N   PRO P 211       5.025   9.601  -6.607  0.00  0.00      PROA
ATOM   3421  CD  PRO P 211       5.011   8.672  -5.463  0.00  0.00      PROA
ATOM   3422  HD1 PRO P 211       4.746   9.117  -4.480  0.00  0.00      PROA
ATOM   3423  HD2 PRO P 211       5.985   8.139  -5.477  0.00  0.00      PROA
ATOM   3424  CA  PRO P 211       4.451   9.051  -7.795  0.00  0.00      PROA
ATOM   3425  HA  PRO P 211       5.175   9.043  -8.596  0.00  0.00      PROA
ATOM   3426  CB  PRO P 211       4.107   7.593  -7.266  0.00  0.00      PROA
ATOM   3427  HB1 PRO P 211       4.871   6.828  -7.520  0.00  0.00      PROA
ATOM   3428  HB2 PRO P 211       3.132   7.283  -7.699  0.00  0.00      PROA
ATOM   3429  CG  PRO P 211       3.864   7.706  -5.759  0.00  0.00      PROA
ATOM   3430  HG1 PRO P 211       2.873   8.109  -5.460  0.00  0.00      PROA
ATOM   3431  HG2 PRO P 211       4.027   6.688  -5.344  0.00  0.00      PROA
ATOM   3432  C   PRO P 211       3.316   9.886  -8.317  0.00  0.00      PROA
ATOM   3433  O   PRO P 211       3.051   9.890  -9.488  0.00  0.00      PROA
ATOM   3434  N   GLY P 212       2.580  10.644  -7.506  0.00  0.00      PROA
ATOM   3435  HN  GLY P 212       2.675  10.630  -6.514  0.00  0.00      PROA
ATOM   3436  CA  GLY P 212       1.496  11.565  -7.929  0.00  0.00      PROA
ATOM   3437  HA1 GLY P 212       1.009  11.905  -7.027  0.00  0.00      PROA
ATOM   3438  HA2 GLY P 212       0.844  11.098  -8.652  0.00  0.00      PROA
ATOM   3439  C   GLY P 212       2.048  12.630  -8.768  0.00  0.00      PROA
ATOM   3440  O   GLY P 212       1.469  12.998  -9.777  0.00  0.00      PROA
ATOM   3441  N   ILE P 213       3.214  13.228  -8.444  0.00  0.00      PROA
ATOM   3442  HN  ILE P 213       3.669  12.918  -7.613  0.00  0.00      PROA
ATOM   3443  CA  ILE P 213       3.909  14.153  -9.303  0.00  0.00      PROA
ATOM   3444  HA  ILE P 213       3.302  15.047  -9.317  0.00  0.00      PROA
ATOM   3445  CB  ILE P 213       5.294  14.479  -8.714  0.00  0.00      PROA
ATOM   3446  HB  ILE P 213       5.748  13.543  -8.325  0.00  0.00      PROA
ATOM   3447  CG2 ILE P 213       6.265  15.179  -9.703  0.00  0.00      PROA
ATOM   3448 HG21 ILE P 213       5.744  16.026 -10.198  0.00  0.00      PROA
ATOM   3449 HG22 ILE P 213       6.716  14.562 -10.509  0.00  0.00      PROA
ATOM   3450 HG23 ILE P 213       7.106  15.603  -9.113  0.00  0.00      PROA
ATOM   3451  CG1 ILE P 213       5.143  15.374  -7.449  0.00  0.00      PROA
ATOM   3452 HG11 ILE P 213       4.230  15.189  -6.844  0.00  0.00      PROA
ATOM   3453 HG12 ILE P 213       4.906  16.401  -7.801  0.00  0.00      PROA
ATOM   3454  CD  ILE P 213       6.392  15.373  -6.467  0.00  0.00      PROA
ATOM   3455  HD1 ILE P 213       6.208  16.195  -5.742  0.00  0.00      PROA
ATOM   3456  HD2 ILE P 213       7.385  15.494  -6.950  0.00  0.00      PROA
ATOM   3457  HD3 ILE P 213       6.441  14.362  -6.009  0.00  0.00      PROA
ATOM   3458  C   ILE P 213       4.133  13.566 -10.704  0.00  0.00      PROA
ATOM   3459  O   ILE P 213       3.844  14.260 -11.668  0.00  0.00      PROA
ATOM   3460  N   VAL P 214       4.649  12.277 -10.865  0.00  0.00      PROA
ATOM   3461  HN  VAL P 214       5.060  11.750 -10.126  0.00  0.00      PROA
ATOM   3462  CA  VAL P 214       4.829  11.699 -12.158  0.00  0.00      PROA
ATOM   3463  HA  VAL P 214       5.468  12.364 -12.721  0.00  0.00      PROA
ATOM   3464  CB  VAL P 214       5.370  10.266 -12.013  0.00  0.00      PROA
ATOM   3465  HB  VAL P 214       4.591   9.757 -11.406  0.00  0.00      PROA
ATOM   3466  CG1 VAL P 214       5.455   9.554 -13.386  0.00  0.00      PROA
ATOM   3467 HG11 VAL P 214       6.355   9.860 -13.961  0.00  0.00      PROA
ATOM   3468 HG12 VAL P 214       4.550   9.535 -14.031  0.00  0.00      PROA
ATOM   3469 HG13 VAL P 214       5.544   8.479 -13.122  0.00  0.00      PROA
ATOM   3470  CG2 VAL P 214       6.741  10.332 -11.268  0.00  0.00      PROA
ATOM   3471 HG21 VAL P 214       7.460  10.931 -11.867  0.00  0.00      PROA
ATOM   3472 HG22 VAL P 214       7.079   9.274 -11.233  0.00  0.00      PROA
ATOM   3473 HG23 VAL P 214       6.805  10.705 -10.224  0.00  0.00      PROA
```

```
ATOM   3474  C   VAL P 214       3.479  11.636 -12.944  0.00  0.00      PROA
ATOM   3475  O   VAL P 214       3.324  11.978 -14.106  0.00  0.00      PROA
ATOM   3476  N   ILE P 215       2.451  11.136 -12.246  0.00  0.00      PROA
ATOM   3477  HN  ILE P 215       2.610  10.812 -11.317  0.00  0.00      PROA
ATOM   3478  CA  ILE P 215       1.103  10.912 -12.829  0.00  0.00      PROA
ATOM   3479  HA  ILE P 215       1.357  10.361 -13.723  0.00  0.00      PROA
ATOM   3480  CB  ILE P 215       0.195  10.153 -11.739  0.00  0.00      PROA
ATOM   3481  HB  ILE P 215       0.348  10.698 -10.782  0.00  0.00      PROA
ATOM   3482  CG2 ILE P 215      -1.323  10.232 -12.065  0.00  0.00      PROA
ATOM   3483 HG21 ILE P 215      -1.627  11.300 -12.037  0.00  0.00      PROA
ATOM   3484 HG22 ILE P 215      -1.997   9.632 -11.416  0.00  0.00      PROA
ATOM   3485 HG23 ILE P 215      -1.517   9.930 -13.116  0.00  0.00      PROA
ATOM   3486  CG1 ILE P 215       0.751   8.723 -11.529  0.00  0.00      PROA
ATOM   3487 HG11 ILE P 215       1.827   8.612 -11.785  0.00  0.00      PROA
ATOM   3488 HG12 ILE P 215       0.165   8.120 -12.256  0.00  0.00      PROA
ATOM   3489  CD  ILE P 215       0.377   8.156 -10.166  0.00  0.00      PROA
ATOM   3490  HD1 ILE P 215      -0.700   8.179  -9.896  0.00  0.00      PROA
ATOM   3491  HD2 ILE P 215       0.964   8.584  -9.325  0.00  0.00      PROA
ATOM   3492  HD3 ILE P 215       0.695   7.098 -10.277  0.00  0.00      PROA
ATOM   3493  C   ILE P 215       0.463  12.186 -13.368  0.00  0.00      PROA
ATOM   3494  O   ILE P 215       0.017  12.224 -14.501  0.00  0.00      PROA
ATOM   3495  N   LEU P 216       0.600  13.264 -12.520  0.00  0.00      PROA
ATOM   3496  HN  LEU P 216       0.930  13.249 -11.579  0.00  0.00      PROA
ATOM   3497  CA  LEU P 216       0.251  14.624 -12.941  0.00  0.00      PROA
ATOM   3498  HA  LEU P 216      -0.715  14.551 -13.417  0.00  0.00      PROA
ATOM   3499  CB  LEU P 216       0.084  15.475 -11.674  0.00  0.00      PROA
ATOM   3500  HB1 LEU P 216      -0.017  16.517 -12.046  0.00  0.00      PROA
ATOM   3501  HB2 LEU P 216       1.048  15.417 -11.127  0.00  0.00      PROA
ATOM   3502  CG  LEU P 216      -1.170  14.960 -10.765  0.00  0.00      PROA
ATOM   3503  HG  LEU P 216      -1.073  13.872 -10.562  0.00  0.00      PROA
ATOM   3504  CD1 LEU P 216      -1.230  15.786  -9.473  0.00  0.00      PROA
ATOM   3505 HD11 LEU P 216      -0.262  15.783  -8.928  0.00  0.00      PROA
ATOM   3506 HD12 LEU P 216      -2.052  15.391  -8.837  0.00  0.00      PROA
ATOM   3507 HD13 LEU P 216      -1.392  16.852  -9.739  0.00  0.00      PROA
ATOM   3508  CD2 LEU P 216      -2.538  15.111 -11.527  0.00  0.00      PROA
ATOM   3509 HD21 LEU P 216      -2.674  16.173 -11.825  0.00  0.00      PROA
ATOM   3510 HD22 LEU P 216      -3.393  14.891 -10.852  0.00  0.00      PROA
ATOM   3511 HD23 LEU P 216      -2.639  14.422 -12.392  0.00  0.00      PROA
ATOM   3512  C   LEU P 216       1.047  15.249 -14.020  0.00  0.00      PROA
ATOM   3513  O   LEU P 216       0.512  15.982 -14.912  0.00  0.00      PROA
ATOM   3514  N   SER P 217       2.377  15.004 -14.053  0.00  0.00      PROA
ATOM   3515  HN  SER P 217       2.786  14.514 -13.287  0.00  0.00      PROA
ATOM   3516  CA  SER P 217       3.304  15.485 -15.138  0.00  0.00      PROA
ATOM   3517  HA  SER P 217       3.235  16.563 -15.172  0.00  0.00      PROA
ATOM   3518  CB  SER P 217       4.706  15.044 -14.857  0.00  0.00      PROA
ATOM   3519  HB1 SER P 217       5.351  15.468 -15.656  0.00  0.00      PROA
ATOM   3520  HB2 SER P 217       4.836  13.943 -14.920  0.00  0.00      PROA
ATOM   3521  OG  SER P 217       5.338  15.445 -13.652  0.00  0.00      PROA
ATOM   3522  HG1 SER P 217       4.886  15.065 -12.895  0.00  0.00      PROA
ATOM   3523  C   SER P 217       3.056  14.912 -16.471  0.00  0.00      PROA
ATOM   3524  O   SER P 217       3.228  15.594 -17.488  0.00  0.00      PROA
ATOM   3525  N   CYS P 218       2.731  13.610 -16.564  0.00  0.00      PROA
ATOM   3526  HN  CYS P 218       2.819  13.087 -15.720  0.00  0.00      PROA
ATOM   3527  CA  CYS P 218       2.207  12.964 -17.739  0.00  0.00      PROA
ATOM   3528  HA  CYS P 218       2.946  12.981 -18.526  0.00  0.00      PROA
ATOM   3529  CB  CYS P 218       2.037  11.456 -17.328  0.00  0.00      PROA
ATOM   3530  HB1 CYS P 218       1.574  10.871 -18.150  0.00  0.00      PROA
ATOM   3531  HB2 CYS P 218       1.308  11.400 -16.491  0.00  0.00      PROA
ATOM   3532  SG  CYS P 218       3.542  10.621 -16.878  0.00  0.00      PROA
ATOM   3533  HG1 CYS P 218       3.322  10.414 -15.588  0.00  0.00      PROA
ATOM   3534  C   CYS P 218       0.891  13.599 -18.273  0.00  0.00      PROA
```

ATOM 3535 O CYS P 218 0.743 13.882 -19.482 0.00 0.00 PROA
ATOM 3536 N TYR P 219 -0.089 13.896 -17.320 0.00 0.00 PROA
ATOM 3537 HN TYR P 219 -0.014 13.511 -16.404 0.00 0.00 PROA
ATOM 3538 CA TYR P 219 -1.380 14.522 -17.695 0.00 0.00 PROA
ATOM 3539 HA TYR P 219 -1.816 13.955 -18.504 0.00 0.00 PROA
ATOM 3540 CB TYR P 219 -2.526 14.483 -16.602 0.00 0.00 PROA
ATOM 3541 HB1 TYR P 219 -3.430 15.065 -16.878 0.00 0.00 PROA
ATOM 3542 HB2 TYR P 219 -2.140 14.996 -15.695 0.00 0.00 PROA
ATOM 3543 CG TYR P 219 -2.998 13.077 -16.086 0.00 0.00 PROA
ATOM 3544 CD1 TYR P 219 -3.636 12.991 -14.871 0.00 0.00 PROA
ATOM 3545 HD1 TYR P 219 -3.963 13.843 -14.294 0.00 0.00 PROA
ATOM 3546 CE1 TYR P 219 -3.761 11.719 -14.285 0.00 0.00 PROA
ATOM 3547 HE1 TYR P 219 -4.229 11.545 -13.328 0.00 0.00 PROA
ATOM 3548 CZ TYR P 219 -3.427 10.538 -14.969 0.00 0.00 PROA
ATOM 3549 OH TYR P 219 -3.534 9.290 -14.404 0.00 0.00 PROA
ATOM 3550 HH TYR P 219 -3.005 8.673 -14.915 0.00 0.00 PROA
ATOM 3551 CD2 TYR P 219 -2.660 11.893 -16.809 0.00 0.00 PROA
ATOM 3552 HD2 TYR P 219 -2.161 11.939 -17.765 0.00 0.00 PROA
ATOM 3553 CE2 TYR P 219 -2.860 10.635 -16.253 0.00 0.00 PROA
ATOM 3554 HE2 TYR P 219 -2.725 9.724 -16.816 0.00 0.00 PROA
ATOM 3555 C TYR P 219 -1.107 15.886 -18.273 0.00 0.00 PROA
ATOM 3556 O TYR P 219 -1.711 16.213 -19.297 0.00 0.00 PROA
ATOM 3557 N CYS P 220 -0.191 16.640 -17.699 0.00 0.00 PROA
ATOM 3558 HN CYS P 220 0.055 16.294 -16.797 0.00 0.00 PROA
ATOM 3559 CA CYS P 220 0.132 18.023 -18.206 0.00 0.00 PROA
ATOM 3560 HA CYS P 220 -0.781 18.600 -18.217 0.00 0.00 PROA
ATOM 3561 CB CYS P 220 1.190 18.687 -17.287 0.00 0.00 PROA
ATOM 3562 HB1 CYS P 220 2.111 18.066 -17.317 0.00 0.00 PROA
ATOM 3563 HB2 CYS P 220 0.896 18.658 -16.216 0.00 0.00 PROA
ATOM 3564 SG CYS P 220 1.654 20.481 -17.611 0.00 0.00 PROA
ATOM 3565 HG1 CYS P 220 2.710 20.349 -18.401 0.00 0.00 PROA
ATOM 3566 C CYS P 220 0.652 18.011 -19.628 0.00 0.00 PROA
ATOM 3567 O CYS P 220 0.337 18.839 -20.458 0.00 0.00 PROA
ATOM 3568 N ILE P 221 1.615 17.041 -19.932 0.00 0.00 PROA
ATOM 3569 HN ILE P 221 1.852 16.334 -19.270 0.00 0.00 PROA
ATOM 3570 CA ILE P 221 2.062 16.898 -21.310 0.00 0.00 PROA
ATOM 3571 HA ILE P 221 2.074 17.899 -21.716 0.00 0.00 PROA
ATOM 3572 CB ILE P 221 3.453 16.255 -21.471 0.00 0.00 PROA
ATOM 3573 HB ILE P 221 4.209 16.892 -20.965 0.00 0.00 PROA
ATOM 3574 CG2 ILE P 221 3.449 14.831 -20.887 0.00 0.00 PROA
ATOM 3575 HG21 ILE P 221 2.680 14.201 -21.384 0.00 0.00 PROA
ATOM 3576 HG22 ILE P 221 3.191 14.708 -19.813 0.00 0.00 PROA
ATOM 3577 HG23 ILE P 221 4.430 14.317 -20.971 0.00 0.00 PROA
ATOM 3578 CG1 ILE P 221 3.867 16.187 -22.922 0.00 0.00 PROA
ATOM 3579 HG11 ILE P 221 3.234 15.425 -23.425 0.00 0.00 PROA
ATOM 3580 HG12 ILE P 221 4.868 15.715 -22.833 0.00 0.00 PROA
ATOM 3581 CD ILE P 221 3.912 17.514 -23.644 0.00 0.00 PROA
ATOM 3582 HD1 ILE P 221 4.142 18.320 -22.915 0.00 0.00 PROA
ATOM 3583 HD2 ILE P 221 2.863 17.807 -23.862 0.00 0.00 PROA
ATOM 3584 HD3 ILE P 221 4.624 17.620 -24.490 0.00 0.00 PROA
ATOM 3585 C ILE P 221 0.882 16.341 -22.148 0.00 0.00 PROA
ATOM 3586 O ILE P 221 0.716 16.739 -23.252 0.00 0.00 PROA
ATOM 3587 N ILE P 222 0.082 15.366 -21.718 0.00 0.00 PROA
ATOM 3588 HN ILE P 222 0.203 14.935 -20.828 0.00 0.00 PROA
ATOM 3589 CA ILE P 222 -1.003 14.913 -22.595 0.00 0.00 PROA
ATOM 3590 HA ILE P 222 -0.603 14.755 -23.586 0.00 0.00 PROA
ATOM 3591 CB ILE P 222 -1.766 13.651 -21.996 0.00 0.00 PROA
ATOM 3592 HB ILE P 222 -2.094 13.938 -20.975 0.00 0.00 PROA
ATOM 3593 CG2 ILE P 222 -3.024 13.321 -22.814 0.00 0.00 PROA
ATOM 3594 HG21 ILE P 222 -3.830 14.084 -22.770 0.00 0.00 PROA
ATOM 3595 HG22 ILE P 222 -3.615 12.412 -22.575 0.00 0.00 PROA

ATOM	3596	HG23	ILE	P	222	-2.670	13.151	-23.854	0.00	0.00	PROA
ATOM	3597	CG1	ILE	P	222	-0.851	12.435	-21.933	0.00	0.00	PROA
ATOM	3598	HG11	ILE	P	222	0.174	12.692	-21.591	0.00	0.00	PROA
ATOM	3599	HG12	ILE	P	222	-0.893	12.020	-22.963	0.00	0.00	PROA
ATOM	3600	CD	ILE	P	222	-1.356	11.356	-21.001	0.00	0.00	PROA
ATOM	3601	HD1	ILE	P	222	-2.077	10.728	-21.566	0.00	0.00	PROA
ATOM	3602	HD2	ILE	P	222	-1.808	11.744	-20.063	0.00	0.00	PROA
ATOM	3603	HD3	ILE	P	222	-0.479	10.712	-20.774	0.00	0.00	PROA
ATOM	3604	C	ILE	P	222	-2.030	15.998	-22.906	0.00	0.00	PROA
ATOM	3605	O	ILE	P	222	-2.522	16.093	-23.984	0.00	0.00	PROA
ATOM	3606	N	ILE	P	223	-2.446	16.793	-21.952	0.00	0.00	PROA
ATOM	3607	HN	ILE	P	223	-2.085	16.577	-21.049	0.00	0.00	PROA
ATOM	3608	CA	ILE	P	223	-3.356	17.940	-22.050	0.00	0.00	PROA
ATOM	3609	HA	ILE	P	223	-4.250	17.563	-22.525	0.00	0.00	PROA
ATOM	3610	CB	ILE	P	223	-3.704	18.608	-20.717	0.00	0.00	PROA
ATOM	3611	HB	ILE	P	223	-2.754	18.599	-20.141	0.00	0.00	PROA
ATOM	3612	CG2	ILE	P	223	-4.186	20.082	-20.947	0.00	0.00	PROA
ATOM	3613	HG21	ILE	P	223	-4.555	20.542	-20.006	0.00	0.00	PROA
ATOM	3614	HG22	ILE	P	223	-5.039	20.161	-21.655	0.00	0.00	PROA
ATOM	3615	HG23	ILE	P	223	-3.315	20.673	-21.305	0.00	0.00	PROA
ATOM	3616	CG1	ILE	P	223	-4.637	17.667	-19.953	0.00	0.00	PROA
ATOM	3617	HG11	ILE	P	223	-4.234	16.632	-19.986	0.00	0.00	PROA
ATOM	3618	HG12	ILE	P	223	-5.568	17.673	-20.560	0.00	0.00	PROA
ATOM	3619	CD	ILE	P	223	-4.978	18.076	-18.599	0.00	0.00	PROA
ATOM	3620	HD1	ILE	P	223	-5.570	19.010	-18.493	0.00	0.00	PROA
ATOM	3621	HD2	ILE	P	223	-4.054	18.020	-17.985	0.00	0.00	PROA
ATOM	3622	HD3	ILE	P	223	-5.489	17.215	-18.116	0.00	0.00	PROA
ATOM	3623	C	ILE	P	223	-2.870	19.013	-23.061	0.00	0.00	PROA
ATOM	3624	O	ILE	P	223	-3.599	19.566	-23.919	0.00	0.00	PROA
ATOM	3625	N	SER	P	224	-1.515	19.393	-22.954	0.00	0.00	PROA
ATOM	3626	HN	SER	P	224	-0.929	18.919	-22.300	0.00	0.00	PROA
ATOM	3627	CA	SER	P	224	-0.777	20.303	-23.767	0.00	0.00	PROA
ATOM	3628	HA	SER	P	224	-1.375	21.201	-23.704	0.00	0.00	PROA
ATOM	3629	CB	SER	P	224	0.721	20.526	-23.410	0.00	0.00	PROA
ATOM	3630	HB1	SER	P	224	1.090	21.288	-24.130	0.00	0.00	PROA
ATOM	3631	HB2	SER	P	224	1.369	19.638	-23.572	0.00	0.00	PROA
ATOM	3632	OG	SER	P	224	0.908	21.029	-22.118	0.00	0.00	PROA
ATOM	3633	HG1	SER	P	224	0.875	20.318	-21.475	0.00	0.00	PROA
ATOM	3634	C	SER	P	224	-0.846	19.891	-25.301	0.00	0.00	PROA
ATOM	3635	O	SER	P	224	-0.851	20.741	-26.123	0.00	0.00	PROA
ATOM	3636	N	LYS	P	225	-0.859	18.568	-25.522	0.00	0.00	PROA
ATOM	3637	HN	LYS	P	225	-0.742	18.025	-24.694	0.00	0.00	PROA
ATOM	3638	CA	LYS	P	225	-0.893	17.934	-26.803	0.00	0.00	PROA
ATOM	3639	HA	LYS	P	225	-0.809	18.752	-27.503	0.00	0.00	PROA
ATOM	3640	CB	LYS	P	225	0.220	16.892	-27.009	0.00	0.00	PROA
ATOM	3641	HB1	LYS	P	225	0.126	16.575	-28.070	0.00	0.00	PROA
ATOM	3642	HB2	LYS	P	225	-0.052	16.032	-26.360	0.00	0.00	PROA
ATOM	3643	CG	LYS	P	225	1.663	17.469	-26.866	0.00	0.00	PROA
ATOM	3644	HG1	LYS	P	225	2.321	16.574	-26.836	0.00	0.00	PROA
ATOM	3645	HG2	LYS	P	225	1.851	17.910	-25.863	0.00	0.00	PROA
ATOM	3646	CD	LYS	P	225	2.180	18.353	-28.043	0.00	0.00	PROA
ATOM	3647	HD1	LYS	P	225	2.025	17.862	-29.027	0.00	0.00	PROA
ATOM	3648	HD2	LYS	P	225	3.276	18.370	-27.862	0.00	0.00	PROA
ATOM	3649	CE	LYS	P	225	1.629	19.763	-28.139	0.00	0.00	PROA
ATOM	3650	HE1	LYS	P	225	1.559	20.228	-27.132	0.00	0.00	PROA
ATOM	3651	HE2	LYS	P	225	0.686	19.713	-28.725	0.00	0.00	PROA
ATOM	3652	NZ	LYS	P	225	2.581	20.561	-28.881	0.00	0.00	PROA
ATOM	3653	HZ1	LYS	P	225	2.237	21.350	-29.465	0.00	0.00	PROA
ATOM	3654	HZ2	LYS	P	225	3.099	20.033	-29.612	0.00	0.00	PROA
ATOM	3655	HZ3	LYS	P	225	3.169	21.135	-28.244	0.00	0.00	PROA
ATOM	3656	C	LYS	P	225	-2.285	17.338	-27.191	0.00	0.00	PROA

ATOM	3657	O	LYS	P	225	-2.437	16.412	-27.999	0.00	0.00	PROA
ATOM	3658	N	LEU	P	226	-3.378	17.971	-26.630	0.00	0.00	PROA
ATOM	3659	HN	LEU	P	226	-3.186	18.694	-25.971	0.00	0.00	PROA
ATOM	3660	CA	LEU	P	226	-4.730	17.414	-26.586	0.00	0.00	PROA
ATOM	3661	HA	LEU	P	226	-4.605	16.376	-26.855	0.00	0.00	PROA
ATOM	3662	CB	LEU	P	226	-5.414	17.436	-25.224	0.00	0.00	PROA
ATOM	3663	HB1	LEU	P	226	-5.788	18.464	-25.027	0.00	0.00	PROA
ATOM	3664	HB2	LEU	P	226	-4.596	17.229	-24.501	0.00	0.00	PROA
ATOM	3665	CG	LEU	P	226	-6.593	16.554	-24.925	0.00	0.00	PROA
ATOM	3666	HG	LEU	P	226	-7.302	16.730	-25.762	0.00	0.00	PROA
ATOM	3667	CD1	LEU	P	226	-6.218	15.082	-24.967	0.00	0.00	PROA
ATOM	3668	HD11	LEU	P	226	-6.937	14.392	-24.475	0.00	0.00	PROA
ATOM	3669	HD12	LEU	P	226	-5.195	14.969	-24.547	0.00	0.00	PROA
ATOM	3670	HD13	LEU	P	226	-6.317	14.784	-26.032	0.00	0.00	PROA
ATOM	3671	CD2	LEU	P	226	-6.975	16.961	-23.535	0.00	0.00	PROA
ATOM	3672	HD21	LEU	P	226	-6.139	16.782	-22.826	0.00	0.00	PROA
ATOM	3673	HD22	LEU	P	226	-7.778	16.297	-23.152	0.00	0.00	PROA
ATOM	3674	HD23	LEU	P	226	-7.309	18.020	-23.564	0.00	0.00	PROA
ATOM	3675	C	LEU	P	226	-5.587	17.868	-27.672	0.00	0.00	PROA
ATOM	3676	O	LEU	P	226	-6.264	17.064	-28.292	0.00	0.00	PROA
ATOM	3677	N	SER	P	227	-5.503	19.132	-27.914	0.00	0.00	PROA
ATOM	3678	HN	SER	P	227	-4.962	19.733	-27.331	0.00	0.00	PROA
ATOM	3679	CA	SER	P	227	-6.063	19.845	-29.047	0.00	0.00	PROA
ATOM	3680	HA	SER	P	227	-7.060	19.451	-29.182	0.00	0.00	PROA
ATOM	3681	CB	SER	P	227	-6.160	21.428	-28.760	0.00	0.00	PROA
ATOM	3682	HB1	SER	P	227	-6.456	21.884	-29.729	0.00	0.00	PROA
ATOM	3683	HB2	SER	P	227	-5.131	21.754	-28.499	0.00	0.00	PROA
ATOM	3684	OG	SER	P	227	-7.088	21.635	-27.711	0.00	0.00	PROA
ATOM	3685	HG1	SER	P	227	-6.630	21.371	-26.910	0.00	0.00	PROA
ATOM	3686	C	SER	P	227	-5.342	19.614	-30.344	0.00	0.00	PROA
ATOM	3687	O	SER	P	227	-4.134	19.420	-30.426	0.00	0.00	PROA
ATOM	3688	N	HSD	P	228	-6.028	19.660	-31.476	0.00	0.00	PROA
ATOM	3689	HN	HSD	P	228	-6.927	20.089	-31.524	0.00	0.00	PROA
ATOM	3690	CA	HSD	P	228	-5.590	19.066	-32.801	0.00	0.00	PROA
ATOM	3691	HA	HSD	P	228	-6.472	19.190	-33.412	0.00	0.00	PROA
ATOM	3692	CB	HSD	P	228	-4.527	20.043	-33.464	0.00	0.00	PROA
ATOM	3693	HB1	HSD	P	228	-3.677	20.411	-32.851	0.00	0.00	PROA
ATOM	3694	HB2	HSD	P	228	-5.013	20.961	-33.858	0.00	0.00	PROA
ATOM	3695	ND1	HSD	P	228	-4.183	19.270	-35.889	0.00	0.00	PROA
ATOM	3696	HD1	HSD	P	228	-5.110	19.503	-36.184	0.00	0.00	PROA
ATOM	3697	CG	HSD	P	228	-3.706	19.419	-34.547	0.00	0.00	PROA
ATOM	3698	CE1	HSD	P	228	-3.097	19.026	-36.615	0.00	0.00	PROA
ATOM	3699	HE1	HSD	P	228	-3.088	18.861	-37.693	0.00	0.00	PROA
ATOM	3700	NE2	HSD	P	228	-1.973	18.972	-35.899	0.00	0.00	PROA
ATOM	3701	CD2	HSD	P	228	-2.340	19.245	-34.602	0.00	0.00	PROA
ATOM	3702	HD2	HSD	P	228	-1.715	19.459	-33.744	0.00	0.00	PROA
ATOM	3703	C	HSD	P	228	-5.130	17.629	-32.942	0.00	0.00	PROA
ATOM	3704	O	HSD	P	228	-4.583	17.031	-32.070	0.00	0.00	PROA
ATOM	3705	N	SER	P	229	-5.381	17.036	-34.073	0.00	0.00	PROA
ATOM	3706	HN	SER	P	229	-5.799	17.598	-34.782	0.00	0.00	PROA
ATOM	3707	CA	SER	P	229	-5.181	15.704	-34.545	0.00	0.00	PROA
ATOM	3708	HA	SER	P	229	-5.787	15.661	-35.438	0.00	0.00	PROA
ATOM	3709	CB	SER	P	229	-3.775	15.532	-35.088	0.00	0.00	PROA
ATOM	3710	HB1	SER	P	229	-3.028	15.393	-34.277	0.00	0.00	PROA
ATOM	3711	HB2	SER	P	229	-3.548	16.446	-35.678	0.00	0.00	PROA
ATOM	3712	OG	SER	P	229	-3.740	14.371	-35.989	0.00	0.00	PROA
ATOM	3713	HG1	SER	P	229	-3.924	13.665	-35.365	0.00	0.00	PROA
ATOM	3714	C	SER	P	229	-5.608	14.600	-33.567	0.00	0.00	PROA
ATOM	3715	O	SER	P	229	-4.790	13.891	-33.000	0.00	0.00	PROA
ATOM	3716	N	LYS	P	230	-6.963	14.378	-33.405	0.00	0.00	PROA
ATOM	3717	HN	LYS	P	230	-7.582	15.003	-33.874	0.00	0.00	PROA

ATOM	3718	CA	LYS	P	230	-7.666	13.300	-32.700	0.00	0.00	PROA
ATOM	3719	HA	LYS	P	230	-8.638	13.417	-33.156	0.00	0.00	PROA
ATOM	3720	CB	LYS	P	230	-7.187	11.815	-33.022	0.00	0.00	PROA
ATOM	3721	HB1	LYS	P	230	-7.876	11.128	-32.485	0.00	0.00	PROA
ATOM	3722	HB2	LYS	P	230	-6.227	11.581	-32.513	0.00	0.00	PROA
ATOM	3723	CG	LYS	P	230	-7.115	11.395	-34.486	0.00	0.00	PROA
ATOM	3724	HG1	LYS	P	230	-7.150	10.301	-34.677	0.00	0.00	PROA
ATOM	3725	HG2	LYS	P	230	-6.209	11.841	-34.948	0.00	0.00	PROA
ATOM	3726	CD	LYS	P	230	-8.238	11.925	-35.411	0.00	0.00	PROA
ATOM	3727	HD1	LYS	P	230	-7.967	12.974	-35.655	0.00	0.00	PROA
ATOM	3728	HD2	LYS	P	230	-9.213	11.976	-34.881	0.00	0.00	PROA
ATOM	3729	CE	LYS	P	230	-8.522	11.143	-36.720	0.00	0.00	PROA
ATOM	3730	HE1	LYS	P	230	-9.500	11.248	-37.237	0.00	0.00	PROA
ATOM	3731	HE2	LYS	P	230	-8.536	10.084	-36.382	0.00	0.00	PROA
ATOM	3732	NZ	LYS	P	230	-7.496	11.316	-37.688	0.00	0.00	PROA
ATOM	3733	HZ1	LYS	P	230	-6.667	10.774	-37.371	0.00	0.00	PROA
ATOM	3734	HZ2	LYS	P	230	-7.250	12.325	-37.745	0.00	0.00	PROA
ATOM	3735	HZ3	LYS	P	230	-7.814	11.037	-38.638	0.00	0.00	PROA
ATOM	3736	C	LYS	P	230	-8.005	13.604	-31.273	0.00	0.00	PROA
ATOM	3737	O	LYS	P	230	-8.270	12.654	-30.517	0.00	0.00	PROA
ATOM	3738	N	GLY	P	231	-8.167	14.876	-30.953	0.00	0.00	PROA
ATOM	3739	HN	GLY	P	231	-8.070	15.620	-31.610	0.00	0.00	PROA
ATOM	3740	CA	GLY	P	231	-8.411	15.390	-29.653	0.00	0.00	PROA
ATOM	3741	HA1	GLY	P	231	-8.501	16.466	-29.681	0.00	0.00	PROA
ATOM	3742	HA2	GLY	P	231	-7.625	15.112	-28.966	0.00	0.00	PROA
ATOM	3743	C	GLY	P	231	-9.616	14.786	-28.959	0.00	0.00	PROA
ATOM	3744	O	GLY	P	231	-9.509	14.337	-27.838	0.00	0.00	PROA
ATOM	3745	N	HSD	P	232	-10.793	14.714	-29.629	0.00	0.00	PROA
ATOM	3746	HN	HSD	P	232	-11.042	15.145	-30.493	0.00	0.00	PROA
ATOM	3747	CA	HSD	P	232	-11.918	13.955	-29.117	0.00	0.00	PROA
ATOM	3748	HA	HSD	P	232	-12.244	14.331	-28.159	0.00	0.00	PROA
ATOM	3749	CB	HSD	P	232	-13.206	14.207	-29.951	0.00	0.00	PROA
ATOM	3750	HB1	HSD	P	232	-12.814	14.226	-30.991	0.00	0.00	PROA
ATOM	3751	HB2	HSD	P	232	-13.568	15.226	-29.698	0.00	0.00	PROA
ATOM	3752	ND1	HSD	P	232	-14.367	12.025	-30.635	0.00	0.00	PROA
ATOM	3753	HD1	HSD	P	232	-13.568	11.480	-30.891	0.00	0.00	PROA
ATOM	3754	CG	HSD	P	232	-14.334	13.179	-29.863	0.00	0.00	PROA
ATOM	3755	CE1	HSD	P	232	-15.540	11.401	-30.316	0.00	0.00	PROA
ATOM	3756	HE1	HSD	P	232	-15.782	10.444	-30.778	0.00	0.00	PROA
ATOM	3757	NE2	HSD	P	232	-16.334	12.019	-29.465	0.00	0.00	PROA
ATOM	3758	CD2	HSD	P	232	-15.515	13.217	-29.210	0.00	0.00	PROA
ATOM	3759	HD2	HSD	P	232	-16.042	14.016	-28.702	0.00	0.00	PROA
ATOM	3760	C	HSD	P	232	-11.602	12.444	-28.851	0.00	0.00	PROA
ATOM	3761	O	HSD	P	232	-12.113	11.890	-27.821	0.00	0.00	PROA
ATOM	3762	N	GLN	P	233	-10.788	11.733	-29.722	0.00	0.00	PROA
ATOM	3763	HN	GLN	P	233	-10.311	12.265	-30.418	0.00	0.00	PROA
ATOM	3764	CA	GLN	P	233	-10.369	10.339	-29.305	0.00	0.00	PROA
ATOM	3765	HA	GLN	P	233	-11.300	9.883	-29.004	0.00	0.00	PROA
ATOM	3766	CB	GLN	P	233	-9.672	9.627	-30.485	0.00	0.00	PROA
ATOM	3767	HB1	GLN	P	233	-9.470	8.606	-30.096	0.00	0.00	PROA
ATOM	3768	HB2	GLN	P	233	-8.699	10.069	-30.789	0.00	0.00	PROA
ATOM	3769	CG	GLN	P	233	-10.482	9.444	-31.802	0.00	0.00	PROA
ATOM	3770	HG1	GLN	P	233	-10.061	8.589	-32.373	0.00	0.00	PROA
ATOM	3771	HG2	GLN	P	233	-10.450	10.339	-32.459	0.00	0.00	PROA
ATOM	3772	CD	GLN	P	233	-11.978	9.215	-31.591	0.00	0.00	PROA
ATOM	3773	OE1	GLN	P	233	-12.749	10.106	-31.864	0.00	0.00	PROA
ATOM	3774	NE2	GLN	P	233	-12.427	8.088	-31.049	0.00	0.00	PROA
ATOM	3775	HE21	GLN	P	233	-13.418	7.961	-31.005	0.00	0.00	PROA
ATOM	3776	HE22	GLN	P	233	-11.839	7.419	-30.595	0.00	0.00	PROA
ATOM	3777	C	GLN	P	233	-9.506	10.338	-28.066	0.00	0.00	PROA
ATOM	3778	O	GLN	P	233	-9.738	9.622	-27.085	0.00	0.00	PROA

ATOM	3779	N	LYS	P	234	-8.529	11.231	-28.112	0.00	0.00	PROA
ATOM	3780	HN	LYS	P	234	-8.461	11.847	-28.892	0.00	0.00	PROA
ATOM	3781	CA	LYS	P	234	-7.499	11.365	-27.115	0.00	0.00	PROA
ATOM	3782	HA	LYS	P	234	-6.941	10.445	-27.014	0.00	0.00	PROA
ATOM	3783	CB	LYS	P	234	-6.577	12.408	-27.640	0.00	0.00	PROA
ATOM	3784	HB1	LYS	P	234	-5.870	12.721	-26.842	0.00	0.00	PROA
ATOM	3785	HB2	LYS	P	234	-7.087	13.342	-27.959	0.00	0.00	PROA
ATOM	3786	CG	LYS	P	234	-5.608	11.901	-28.842	0.00	0.00	PROA
ATOM	3787	HG1	LYS	P	234	-6.149	11.592	-29.761	0.00	0.00	PROA
ATOM	3788	HG2	LYS	P	234	-5.097	11.011	-28.414	0.00	0.00	PROA
ATOM	3789	CD	LYS	P	234	-4.469	12.958	-29.098	0.00	0.00	PROA
ATOM	3790	HD1	LYS	P	234	-3.897	12.468	-29.914	0.00	0.00	PROA
ATOM	3791	HD2	LYS	P	234	-3.775	13.104	-28.243	0.00	0.00	PROA
ATOM	3792	CE	LYS	P	234	-4.901	14.346	-29.584	0.00	0.00	PROA
ATOM	3793	HE1	LYS	P	234	-5.367	14.897	-28.740	0.00	0.00	PROA
ATOM	3794	HE2	LYS	P	234	-5.605	14.188	-30.429	0.00	0.00	PROA
ATOM	3795	NZ	LYS	P	234	-3.733	15.181	-30.051	0.00	0.00	PROA
ATOM	3796	HZ1	LYS	P	234	-2.886	14.592	-30.177	0.00	0.00	PROA
ATOM	3797	HZ2	LYS	P	234	-3.477	15.771	-29.233	0.00	0.00	PROA
ATOM	3798	HZ3	LYS	P	234	-3.863	15.821	-30.860	0.00	0.00	PROA
ATOM	3799	C	LYS	P	234	-7.999	11.751	-25.783	0.00	0.00	PROA
ATOM	3800	O	LYS	P	234	-7.608	11.181	-24.772	0.00	0.00	PROA
ATOM	3801	N	ARG	P	235	-8.987	12.709	-25.705	0.00	0.00	PROA
ATOM	3802	HN	ARG	P	235	-9.191	13.263	-26.508	0.00	0.00	PROA
ATOM	3803	CA	ARG	P	235	-9.691	12.977	-24.535	0.00	0.00	PROA
ATOM	3804	HA	ARG	P	235	-8.977	13.210	-23.759	0.00	0.00	PROA
ATOM	3805	CB	ARG	P	235	-10.594	14.129	-24.815	0.00	0.00	PROA
ATOM	3806	HB1	ARG	P	235	-11.427	14.199	-24.084	0.00	0.00	PROA
ATOM	3807	HB2	ARG	P	235	-11.088	13.846	-25.769	0.00	0.00	PROA
ATOM	3808	CG	ARG	P	235	-9.976	15.489	-24.945	0.00	0.00	PROA
ATOM	3809	HG1	ARG	P	235	-9.171	15.309	-25.688	0.00	0.00	PROA
ATOM	3810	HG2	ARG	P	235	-9.491	15.694	-23.967	0.00	0.00	PROA
ATOM	3811	CD	ARG	P	235	-10.792	16.635	-25.433	0.00	0.00	PROA
ATOM	3812	HD1	ARG	P	235	-11.756	16.674	-24.881	0.00	0.00	PROA
ATOM	3813	HD2	ARG	P	235	-11.182	16.390	-26.444	0.00	0.00	PROA
ATOM	3814	NE	ARG	P	235	-10.101	17.951	-25.452	0.00	0.00	PROA
ATOM	3815	HE	ARG	P	235	-9.876	18.413	-26.310	0.00	0.00	PROA
ATOM	3816	CZ	ARG	P	235	-9.774	18.720	-24.387	0.00	0.00	PROA
ATOM	3817	NH1	ARG	P	235	-10.153	18.431	-23.183	0.00	0.00	PROA
ATOM	3818	HH11	ARG	P	235	-10.476	17.520	-22.927	0.00	0.00	PROA
ATOM	3819	HH12	ARG	P	235	-9.798	19.092	-22.521	0.00	0.00	PROA
ATOM	3820	NH2	ARG	P	235	-9.096	19.826	-24.625	0.00	0.00	PROA
ATOM	3821	HH21	ARG	P	235	-8.881	19.934	-25.595	0.00	0.00	PROA
ATOM	3822	HH22	ARG	P	235	-9.029	20.510	-23.898	0.00	0.00	PROA
ATOM	3823	C	ARG	P	235	-10.481	11.923	-23.885	0.00	0.00	PROA
ATOM	3824	O	ARG	P	235	-10.426	11.838	-22.674	0.00	0.00	PROA
ATOM	3825	N	LYS	P	236	-11.114	11.067	-24.673	0.00	0.00	PROA
ATOM	3826	HN	LYS	P	236	-11.191	11.232	-25.653	0.00	0.00	PROA
ATOM	3827	CA	LYS	P	236	-11.886	9.975	-24.255	0.00	0.00	PROA
ATOM	3828	HA	LYS	P	236	-12.578	10.228	-23.465	0.00	0.00	PROA
ATOM	3829	CB	LYS	P	236	-12.896	9.465	-25.349	0.00	0.00	PROA
ATOM	3830	HB1	LYS	P	236	-13.331	8.540	-24.912	0.00	0.00	PROA
ATOM	3831	HB2	LYS	P	236	-12.338	9.128	-26.249	0.00	0.00	PROA
ATOM	3832	CG	LYS	P	236	-13.916	10.561	-25.675	0.00	0.00	PROA
ATOM	3833	HG1	LYS	P	236	-13.508	11.591	-25.765	0.00	0.00	PROA
ATOM	3834	HG2	LYS	P	236	-14.513	10.609	-24.740	0.00	0.00	PROA
ATOM	3835	CD	LYS	P	236	-14.812	10.266	-26.895	0.00	0.00	PROA
ATOM	3836	HD1	LYS	P	236	-14.213	10.304	-27.830	0.00	0.00	PROA
ATOM	3837	HD2	LYS	P	236	-15.578	11.069	-26.849	0.00	0.00	PROA
ATOM	3838	CE	LYS	P	236	-15.641	8.968	-26.803	0.00	0.00	PROA
ATOM	3839	HE1	LYS	P	236	-16.505	9.132	-26.123	0.00	0.00	PROA

ATOM	3840	HE2	LYS	P	236	-15.133	8.052	-26.432	0.00	0.00	PROA
ATOM	3841	NZ	LYS	P	236	-16.130	8.628	-28.160	0.00	0.00	PROA
ATOM	3842	HZ1	LYS	P	236	-16.903	9.285	-28.389	0.00	0.00	PROA
ATOM	3843	HZ2	LYS	P	236	-16.404	7.628	-28.082	0.00	0.00	PROA
ATOM	3844	HZ3	LYS	P	236	-15.309	8.680	-28.796	0.00	0.00	PROA
ATOM	3845	C	LYS	P	236	-11.040	8.840	-23.666	0.00	0.00	PROA
ATOM	3846	O	LYS	P	236	-11.345	8.218	-22.664	0.00	0.00	PROA
ATOM	3847	N	ALA	P	237	-9.841	8.541	-24.307	0.00	0.00	PROA
ATOM	3848	HN	ALA	P	237	-9.704	8.842	-25.248	0.00	0.00	PROA
ATOM	3849	CA	ALA	P	237	-8.771	7.850	-23.687	0.00	0.00	PROA
ATOM	3850	HA	ALA	P	237	-9.219	6.911	-23.396	0.00	0.00	PROA
ATOM	3851	CB	ALA	P	237	-7.609	7.759	-24.682	0.00	0.00	PROA
ATOM	3852	HB1	ALA	P	237	-6.790	7.227	-24.152	0.00	0.00	PROA
ATOM	3853	HB2	ALA	P	237	-7.207	8.731	-25.039	0.00	0.00	PROA
ATOM	3854	HB3	ALA	P	237	-7.943	7.082	-25.498	0.00	0.00	PROA
ATOM	3855	C	ALA	P	237	-8.262	8.507	-22.385	0.00	0.00	PROA
ATOM	3856	O	ALA	P	237	-8.116	7.809	-21.341	0.00	0.00	PROA
ATOM	3857	N	LEU	P	238	-8.057	9.776	-22.348	0.00	0.00	PROA
ATOM	3858	HN	LEU	P	238	-8.126	10.269	-23.212	0.00	0.00	PROA
ATOM	3859	CA	LEU	P	238	-7.587	10.494	-21.144	0.00	0.00	PROA
ATOM	3860	HA	LEU	P	238	-6.677	9.993	-20.850	0.00	0.00	PROA
ATOM	3861	CB	LEU	P	238	-7.170	11.994	-21.431	0.00	0.00	PROA
ATOM	3862	HB1	LEU	P	238	-8.195	12.370	-21.637	0.00	0.00	PROA
ATOM	3863	HB2	LEU	P	238	-6.561	11.932	-22.358	0.00	0.00	PROA
ATOM	3864	CG	LEU	P	238	-6.515	12.748	-20.238	0.00	0.00	PROA
ATOM	3865	HG	LEU	P	238	-7.172	12.683	-19.344	0.00	0.00	PROA
ATOM	3866	CD1	LEU	P	238	-5.133	12.194	-19.889	0.00	0.00	PROA
ATOM	3867	HD11	LEU	P	238	-4.495	12.551	-20.726	0.00	0.00	PROA
ATOM	3868	HD12	LEU	P	238	-4.994	11.094	-19.946	0.00	0.00	PROA
ATOM	3869	HD13	LEU	P	238	-4.671	12.665	-18.995	0.00	0.00	PROA
ATOM	3870	CD2	LEU	P	238	-6.384	14.173	-20.711	0.00	0.00	PROA
ATOM	3871	HD21	LEU	P	238	-6.091	14.308	-21.774	0.00	0.00	PROA
ATOM	3872	HD22	LEU	P	238	-5.645	14.736	-20.101	0.00	0.00	PROA
ATOM	3873	HD23	LEU	P	238	-7.320	14.753	-20.565	0.00	0.00	PROA
ATOM	3874	C	LEU	P	238	-8.628	10.357	-20.019	0.00	0.00	PROA
ATOM	3875	O	LEU	P	238	-8.247	9.932	-18.906	0.00	0.00	PROA
ATOM	3876	N	LYS	P	239	-9.919	10.514	-20.306	0.00	0.00	PROA
ATOM	3877	HN	LYS	P	239	-10.268	10.891	-21.161	0.00	0.00	PROA
ATOM	3878	CA	LYS	P	239	-10.935	10.345	-19.326	0.00	0.00	PROA
ATOM	3879	HA	LYS	P	239	-10.757	11.110	-18.584	0.00	0.00	PROA
ATOM	3880	CB	LYS	P	239	-12.318	10.759	-19.939	0.00	0.00	PROA
ATOM	3881	HB1	LYS	P	239	-13.133	10.096	-19.578	0.00	0.00	PROA
ATOM	3882	HB2	LYS	P	239	-12.298	10.593	-21.037	0.00	0.00	PROA
ATOM	3883	CG	LYS	P	239	-12.731	12.279	-19.772	0.00	0.00	PROA
ATOM	3884	HG1	LYS	P	239	-13.786	12.401	-20.099	0.00	0.00	PROA
ATOM	3885	HG2	LYS	P	239	-12.074	12.967	-20.347	0.00	0.00	PROA
ATOM	3886	CD	LYS	P	239	-12.687	12.834	-18.383	0.00	0.00	PROA
ATOM	3887	HD1	LYS	P	239	-13.256	13.786	-18.444	0.00	0.00	PROA
ATOM	3888	HD2	LYS	P	239	-11.632	13.007	-18.080	0.00	0.00	PROA
ATOM	3889	CE	LYS	P	239	-13.472	12.052	-17.295	0.00	0.00	PROA
ATOM	3890	HE1	LYS	P	239	-13.337	12.514	-16.294	0.00	0.00	PROA
ATOM	3891	HE2	LYS	P	239	-13.127	11.004	-17.164	0.00	0.00	PROA
ATOM	3892	NZ	LYS	P	239	-14.867	12.044	-17.686	0.00	0.00	PROA
ATOM	3893	HZ1	LYS	P	239	-14.993	11.770	-18.681	0.00	0.00	PROA
ATOM	3894	HZ2	LYS	P	239	-15.243	12.992	-17.479	0.00	0.00	PROA
ATOM	3895	HZ3	LYS	P	239	-15.401	11.350	-17.124	0.00	0.00	PROA
ATOM	3896	C	LYS	P	239	-10.977	8.954	-18.670	0.00	0.00	PROA
ATOM	3897	O	LYS	P	239	-11.014	8.898	-17.404	0.00	0.00	PROA
ATOM	3898	N	THR	P	240	-10.992	7.848	-19.432	0.00	0.00	PROA
ATOM	3899	HN	THR	P	240	-11.012	7.946	-20.424	0.00	0.00	PROA
ATOM	3900	CA	THR	P	240	-11.063	6.538	-18.868	0.00	0.00	PROA

```
ATOM   3901  HA  THR P 240     -11.874   6.495 -18.157  0.00  0.00      PROA
ATOM   3902  CB  THR P 240     -11.355   5.452 -19.867  0.00  0.00      PROA
ATOM   3903  HB  THR P 240     -12.307   5.670 -20.396  0.00  0.00      PROA
ATOM   3904  OG1 THR P 240     -11.440   4.157 -19.207  0.00  0.00      PROA
ATOM   3905  HG1 THR P 240     -12.206   4.119 -18.629  0.00  0.00      PROA
ATOM   3906  CG2 THR P 240     -10.245   5.425 -20.959  0.00  0.00      PROA
ATOM   3907 HG21 THR P 240      -9.264   5.211 -20.483  0.00  0.00      PROA
ATOM   3908 HG22 THR P 240     -10.301   6.349 -21.573  0.00  0.00      PROA
ATOM   3909 HG23 THR P 240     -10.418   4.636 -21.721  0.00  0.00      PROA
ATOM   3910  C   THR P 240      -9.821   6.186 -17.949  0.00  0.00      PROA
ATOM   3911  O   THR P 240      -9.937   5.677 -16.823  0.00  0.00      PROA
ATOM   3912  N   THR P 241      -8.655   6.643 -18.372  0.00  0.00      PROA
ATOM   3913  HN  THR P 241      -8.511   7.073 -19.259  0.00  0.00      PROA
ATOM   3914  CA  THR P 241      -7.371   6.493 -17.631  0.00  0.00      PROA
ATOM   3915  HA  THR P 241      -7.320   5.446 -17.370  0.00  0.00      PROA
ATOM   3916  CB  THR P 241      -6.132   6.974 -18.454  0.00  0.00      PROA
ATOM   3917  HB  THR P 241      -6.076   8.080 -18.375  0.00  0.00      PROA
ATOM   3918  OG1 THR P 241      -6.140   6.794 -19.865  0.00  0.00      PROA
ATOM   3919  HG1 THR P 241      -6.906   7.258 -20.210  0.00  0.00      PROA
ATOM   3920  CG2 THR P 241      -4.895   6.384 -17.851  0.00  0.00      PROA
ATOM   3921 HG21 THR P 241      -4.816   5.296 -18.058  0.00  0.00      PROA
ATOM   3922 HG22 THR P 241      -4.844   6.572 -16.757  0.00  0.00      PROA
ATOM   3923 HG23 THR P 241      -3.998   6.907 -18.248  0.00  0.00      PROA
ATOM   3924  C   THR P 241      -7.338   7.167 -16.351  0.00  0.00      PROA
ATOM   3925  O   THR P 241      -6.927   6.544 -15.382  0.00  0.00      PROA
ATOM   3926  N   VAL P 242      -7.731   8.416 -16.354  0.00  0.00      PROA
ATOM   3927  HN  VAL P 242      -7.975   8.878 -17.203  0.00  0.00      PROA
ATOM   3928  CA  VAL P 242      -7.943   9.321 -15.242  0.00  0.00      PROA
ATOM   3929  HA  VAL P 242      -6.998   9.408 -14.726  0.00  0.00      PROA
ATOM   3930  CB  VAL P 242      -8.317  10.719 -15.816  0.00  0.00      PROA
ATOM   3931  HB  VAL P 242      -9.028  10.496 -16.639  0.00  0.00      PROA
ATOM   3932  CG1 VAL P 242      -8.924  11.657 -14.809  0.00  0.00      PROA
ATOM   3933 HG11 VAL P 242     -10.008  11.467 -14.661  0.00  0.00      PROA
ATOM   3934 HG12 VAL P 242      -8.817  12.690 -15.205  0.00  0.00      PROA
ATOM   3935 HG13 VAL P 242      -8.395  11.621 -13.833  0.00  0.00      PROA
ATOM   3936  CG2 VAL P 242      -7.074  11.387 -16.392  0.00  0.00      PROA
ATOM   3937 HG21 VAL P 242      -6.635  10.884 -17.280  0.00  0.00      PROA
ATOM   3938 HG22 VAL P 242      -6.293  11.590 -15.629  0.00  0.00      PROA
ATOM   3939 HG23 VAL P 242      -7.320  12.385 -16.814  0.00  0.00      PROA
ATOM   3940  C   VAL P 242      -8.837   8.807 -14.114  0.00  0.00      PROA
ATOM   3941  O   VAL P 242      -8.568   9.021 -12.955  0.00  0.00      PROA
ATOM   3942  N   ILE P 243      -9.970   8.235 -14.459  0.00  0.00      PROA
ATOM   3943  HN  ILE P 243     -10.180   7.998 -15.404  0.00  0.00      PROA
ATOM   3944  CA  ILE P 243     -10.921   7.724 -13.419  0.00  0.00      PROA
ATOM   3945  HA  ILE P 243     -10.863   8.394 -12.574  0.00  0.00      PROA
ATOM   3946  CB  ILE P 243     -12.316   7.622 -13.955  0.00  0.00      PROA
ATOM   3947  HB  ILE P 243     -12.352   6.939 -14.830  0.00  0.00      PROA
ATOM   3948  CG2 ILE P 243     -13.419   7.035 -12.934  0.00  0.00      PROA
ATOM   3949 HG21 ILE P 243     -14.451   7.196 -13.313  0.00  0.00      PROA
ATOM   3950 HG22 ILE P 243     -13.247   7.577 -11.979  0.00  0.00      PROA
ATOM   3951 HG23 ILE P 243     -13.360   5.938 -12.766  0.00  0.00      PROA
ATOM   3952  CG1 ILE P 243     -12.881   8.920 -14.557  0.00  0.00      PROA
ATOM   3953 HG11 ILE P 243     -13.803   8.583 -15.077  0.00  0.00      PROA
ATOM   3954 HG12 ILE P 243     -12.119   9.353 -15.240  0.00  0.00      PROA
ATOM   3955  CD  ILE P 243     -13.055  10.054 -13.560  0.00  0.00      PROA
ATOM   3956  HD1 ILE P 243     -13.196  11.044 -14.043  0.00  0.00      PROA
ATOM   3957  HD2 ILE P 243     -12.123  10.119 -12.959  0.00  0.00      PROA
ATOM   3958  HD3 ILE P 243     -13.858   9.956 -12.798  0.00  0.00      PROA
ATOM   3959  C   ILE P 243     -10.454   6.447 -12.829  0.00  0.00      PROA
ATOM   3960  O   ILE P 243     -10.592   6.159 -11.681  0.00  0.00      PROA
ATOM   3961  N   LEU P 244      -9.672   5.632 -13.595  0.00  0.00      PROA
```

ATOM 3962 HN LEU P 244 -9.427 5.960 -14.504 0.00 0.00 PROA
ATOM 3963 CA LEU P 244 -8.993 4.428 -13.176 0.00 0.00 PROA
ATOM 3964 HA LEU P 244 -9.771 3.808 -12.756 0.00 0.00 PROA
ATOM 3965 CB LEU P 244 -8.485 3.576 -14.368 0.00 0.00 PROA
ATOM 3966 HB1 LEU P 244 -7.765 4.170 -14.971 0.00 0.00 PROA
ATOM 3967 HB2 LEU P 244 -9.371 3.446 -15.025 0.00 0.00 PROA
ATOM 3968 CG LEU P 244 -7.815 2.268 -14.171 0.00 0.00 PROA
ATOM 3969 HG LEU P 244 -6.965 2.345 -13.458 0.00 0.00 PROA
ATOM 3970 CD1 LEU P 244 -8.793 1.263 -13.528 0.00 0.00 PROA
ATOM 3971 HD11 LEU P 244 -9.375 1.768 -12.727 0.00 0.00 PROA
ATOM 3972 HD12 LEU P 244 -8.355 0.259 -13.344 0.00 0.00 PROA
ATOM 3973 HD13 LEU P 244 -9.561 0.964 -14.272 0.00 0.00 PROA
ATOM 3974 CD2 LEU P 244 -7.198 1.661 -15.490 0.00 0.00 PROA
ATOM 3975 HD21 LEU P 244 -6.651 0.719 -15.274 0.00 0.00 PROA
ATOM 3976 HD22 LEU P 244 -6.513 2.369 -16.005 0.00 0.00 PROA
ATOM 3977 HD23 LEU P 244 -7.921 1.228 -16.213 0.00 0.00 PROA
ATOM 3978 C LEU P 244 -7.880 4.607 -12.054 0.00 0.00 PROA
ATOM 3979 O LEU P 244 -7.921 4.038 -10.982 0.00 0.00 PROA
ATOM 3980 N ILE P 245 -6.986 5.617 -12.296 0.00 0.00 PROA
ATOM 3981 HN ILE P 245 -6.907 6.118 -13.154 0.00 0.00 PROA
ATOM 3982 CA ILE P 245 -6.050 6.016 -11.275 0.00 0.00 PROA
ATOM 3983 HA ILE P 245 -5.568 5.108 -10.944 0.00 0.00 PROA
ATOM 3984 CB ILE P 245 -4.900 6.819 -11.903 0.00 0.00 PROA
ATOM 3985 HB ILE P 245 -5.331 7.797 -12.205 0.00 0.00 PROA
ATOM 3986 CG2 ILE P 245 -3.955 7.243 -10.808 0.00 0.00 PROA
ATOM 3987 HG21 ILE P 245 -4.418 7.967 -10.104 0.00 0.00 PROA
ATOM 3988 HG22 ILE P 245 -3.072 7.746 -11.256 0.00 0.00 PROA
ATOM 3989 HG23 ILE P 245 -3.684 6.376 -10.167 0.00 0.00 PROA
ATOM 3990 CG1 ILE P 245 -4.218 6.095 -13.131 0.00 0.00 PROA
ATOM 3991 HG11 ILE P 245 -3.330 6.681 -13.448 0.00 0.00 PROA
ATOM 3992 HG12 ILE P 245 -4.927 6.045 -13.986 0.00 0.00 PROA
ATOM 3993 CD ILE P 245 -3.714 4.609 -12.993 0.00 0.00 PROA
ATOM 3994 HD1 ILE P 245 -4.526 3.851 -13.020 0.00 0.00 PROA
ATOM 3995 HD2 ILE P 245 -3.218 4.355 -12.032 0.00 0.00 PROA
ATOM 3996 HD3 ILE P 245 -3.080 4.305 -13.854 0.00 0.00 PROA
ATOM 3997 C ILE P 245 -6.560 6.672 -10.059 0.00 0.00 PROA
ATOM 3998 O ILE P 245 -6.161 6.451 -8.915 0.00 0.00 PROA
ATOM 3999 N LEU P 246 -7.589 7.521 -10.220 0.00 0.00 PROA
ATOM 4000 HN LEU P 246 -7.943 7.831 -11.099 0.00 0.00 PROA
ATOM 4001 CA LEU P 246 -8.448 8.062 -9.220 0.00 0.00 PROA
ATOM 4002 HA LEU P 246 -7.828 8.624 -8.538 0.00 0.00 PROA
ATOM 4003 CB LEU P 246 -9.373 9.083 -9.944 0.00 0.00 PROA
ATOM 4004 HB1 LEU P 246 -10.032 8.493 -10.616 0.00 0.00 PROA
ATOM 4005 HB2 LEU P 246 -8.843 9.797 -10.610 0.00 0.00 PROA
ATOM 4006 CG LEU P 246 -10.302 9.977 -9.029 0.00 0.00 PROA
ATOM 4007 HG LEU P 246 -10.774 9.399 -8.206 0.00 0.00 PROA
ATOM 4008 CD1 LEU P 246 -9.381 11.042 -8.239 0.00 0.00 PROA
ATOM 4009 HD11 LEU P 246 -10.147 11.496 -7.574 0.00 0.00 PROA
ATOM 4010 HD12 LEU P 246 -8.892 11.768 -8.923 0.00 0.00 PROA
ATOM 4011 HD13 LEU P 246 -8.610 10.469 -7.681 0.00 0.00 PROA
ATOM 4012 CD2 LEU P 246 -11.255 10.840 -9.783 0.00 0.00 PROA
ATOM 4013 HD21 LEU P 246 -12.223 10.325 -9.965 0.00 0.00 PROA
ATOM 4014 HD22 LEU P 246 -10.853 11.359 -10.679 0.00 0.00 PROA
ATOM 4015 HD23 LEU P 246 -11.638 11.638 -9.112 0.00 0.00 PROA
ATOM 4016 C LEU P 246 -9.183 7.053 -8.404 0.00 0.00 PROA
ATOM 4017 O LEU P 246 -9.288 7.211 -7.192 0.00 0.00 PROA
ATOM 4018 N ALA P 247 -9.787 5.988 -8.977 0.00 0.00 PROA
ATOM 4019 HN ALA P 247 -9.621 5.884 -9.954 0.00 0.00 PROA
ATOM 4020 CA ALA P 247 -10.536 4.891 -8.483 0.00 0.00 PROA
ATOM 4021 HA ALA P 247 -11.406 5.231 -7.939 0.00 0.00 PROA
ATOM 4022 CB ALA P 247 -10.956 3.913 -9.656 0.00 0.00 PROA

ATOM	4023	HB1	ALA	P	247	-10.087	3.578	-10.261	0.00	0.00	PROA
ATOM	4024	HB2	ALA	P	247	-11.797	4.320	-10.257	0.00	0.00	PROA
ATOM	4025	HB3	ALA	P	247	-11.523	3.074	-9.198	0.00	0.00	PROA
ATOM	4026	C	ALA	P	247	-9.820	4.093	-7.425	0.00	0.00	PROA
ATOM	4027	O	ALA	P	247	-10.196	3.831	-6.319	0.00	0.00	PROA
ATOM	4028	N	PHE	P	248	-8.555	3.718	-7.842	0.00	0.00	PROA
ATOM	4029	HN	PHE	P	248	-8.165	3.914	-8.738	0.00	0.00	PROA
ATOM	4030	CA	PHE	P	248	-7.536	3.135	-7.032	0.00	0.00	PROA
ATOM	4031	HA	PHE	P	248	-7.914	2.218	-6.606	0.00	0.00	PROA
ATOM	4032	CB	PHE	P	248	-6.385	2.821	-7.964	0.00	0.00	PROA
ATOM	4033	HB1	PHE	P	248	-5.468	2.485	-7.433	0.00	0.00	PROA
ATOM	4034	HB2	PHE	P	248	-6.070	3.719	-8.536	0.00	0.00	PROA
ATOM	4035	CG	PHE	P	248	-6.615	1.580	-8.865	0.00	0.00	PROA
ATOM	4036	CD1	PHE	P	248	-5.992	1.622	-10.124	0.00	0.00	PROA
ATOM	4037	HD1	PHE	P	248	-5.471	2.515	-10.437	0.00	0.00	PROA
ATOM	4038	CE1	PHE	P	248	-5.911	0.586	-11.015	0.00	0.00	PROA
ATOM	4039	HE1	PHE	P	248	-5.633	0.777	-12.041	0.00	0.00	PROA
ATOM	4040	CZ	PHE	P	248	-6.671	-0.563	-10.770	0.00	0.00	PROA
ATOM	4041	HZ	PHE	P	248	-6.516	-1.395	-11.442	0.00	0.00	PROA
ATOM	4042	CD2	PHE	P	248	-7.283	0.377	-8.662	0.00	0.00	PROA
ATOM	4043	HD2	PHE	P	248	-7.863	0.240	-7.761	0.00	0.00	PROA
ATOM	4044	CE2	PHE	P	248	-7.438	-0.669	-9.624	0.00	0.00	PROA
ATOM	4045	HE2	PHE	P	248	-8.115	-1.493	-9.458	0.00	0.00	PROA
ATOM	4046	C	PHE	P	248	-7.065	4.090	-5.887	0.00	0.00	PROA
ATOM	4047	O	PHE	P	248	-6.970	3.692	-4.718	0.00	0.00	PROA
ATOM	4048	N	PHE	P	249	-6.867	5.399	-6.192	0.00	0.00	PROA
ATOM	4049	HN	PHE	P	249	-7.006	5.759	-7.111	0.00	0.00	PROA
ATOM	4050	CA	PHE	P	249	-6.411	6.278	-5.252	0.00	0.00	PROA
ATOM	4051	HA	PHE	P	249	-5.502	5.930	-4.785	0.00	0.00	PROA
ATOM	4052	CB	PHE	P	249	-6.129	7.679	-5.929	0.00	0.00	PROA
ATOM	4053	HB1	PHE	P	249	-7.053	7.876	-6.513	0.00	0.00	PROA
ATOM	4054	HB2	PHE	P	249	-5.336	7.586	-6.702	0.00	0.00	PROA
ATOM	4055	CG	PHE	P	249	-5.881	8.923	-5.055	0.00	0.00	PROA
ATOM	4056	CD1	PHE	P	249	-4.584	9.206	-4.495	0.00	0.00	PROA
ATOM	4057	HD1	PHE	P	249	-3.704	8.638	-4.756	0.00	0.00	PROA
ATOM	4058	CE1	PHE	P	249	-4.469	10.318	-3.629	0.00	0.00	PROA
ATOM	4059	HE1	PHE	P	249	-3.522	10.531	-3.157	0.00	0.00	PROA
ATOM	4060	CZ	PHE	P	249	-5.489	11.211	-3.419	0.00	0.00	PROA
ATOM	4061	HZ	PHE	P	249	-5.347	12.147	-2.899	0.00	0.00	PROA
ATOM	4062	CD2	PHE	P	249	-6.887	9.837	-4.854	0.00	0.00	PROA
ATOM	4063	HD2	PHE	P	249	-7.874	9.585	-5.213	0.00	0.00	PROA
ATOM	4064	CE2	PHE	P	249	-6.717	11.003	-4.073	0.00	0.00	PROA
ATOM	4065	HE2	PHE	P	249	-7.517	11.724	-3.991	0.00	0.00	PROA
ATOM	4066	C	PHE	P	249	-7.397	6.430	-4.014	0.00	0.00	PROA
ATOM	4067	O	PHE	P	249	-6.927	6.414	-2.851	0.00	0.00	PROA
ATOM	4068	N	ALA	P	250	-8.714	6.427	-4.317	0.00	0.00	PROA
ATOM	4069	HN	ALA	P	250	-8.845	6.484	-5.304	0.00	0.00	PROA
ATOM	4070	CA	ALA	P	250	-9.911	6.385	-3.489	0.00	0.00	PROA
ATOM	4071	HA	ALA	P	250	-9.879	7.199	-2.780	0.00	0.00	PROA
ATOM	4072	CB	ALA	P	250	-11.144	6.475	-4.382	0.00	0.00	PROA
ATOM	4073	HB1	ALA	P	250	-12.105	6.439	-3.826	0.00	0.00	PROA
ATOM	4074	HB2	ALA	P	250	-11.257	5.636	-5.102	0.00	0.00	PROA
ATOM	4075	HB3	ALA	P	250	-11.189	7.351	-5.063	0.00	0.00	PROA
ATOM	4076	C	ALA	P	250	-10.029	5.122	-2.612	0.00	0.00	PROA
ATOM	4077	O	ALA	P	250	-10.164	5.102	-1.388	0.00	0.00	PROA
ATOM	4078	N	CYS	P	251	-9.729	3.935	-3.145	0.00	0.00	PROA
ATOM	4079	HN	CYS	P	251	-9.592	3.871	-4.130	0.00	0.00	PROA
ATOM	4080	CA	CYS	P	251	-9.537	2.731	-2.358	0.00	0.00	PROA
ATOM	4081	HA	CYS	P	251	-10.450	2.509	-1.827	0.00	0.00	PROA
ATOM	4082	CB	CYS	P	251	-9.377	1.539	-3.290	0.00	0.00	PROA
ATOM	4083	HB1	CYS	P	251	-9.276	0.645	-2.637	0.00	0.00	PROA

ATOM 4084 HB2 CYS P 251 -8.425 1.532 -3.862 0.00 0.00 PROA
ATOM 4085 SG CYS P 251 -10.807 1.339 -4.355 0.00 0.00 PROA
ATOM 4086 HG1 CYS P 251 -10.539 2.447 -5.030 0.00 0.00 PROA
ATOM 4087 C CYS P 251 -8.386 2.839 -1.385 0.00 0.00 PROA
ATOM 4088 O CYS P 251 -8.580 2.393 -0.277 0.00 0.00 PROA
ATOM 4089 N TRP P 252 -7.220 3.492 -1.664 0.00 0.00 PROA
ATOM 4090 HN TRP P 252 -7.059 3.755 -2.612 0.00 0.00 PROA
ATOM 4091 CA TRP P 252 -6.106 3.823 -0.761 0.00 0.00 PROA
ATOM 4092 HA TRP P 252 -5.688 2.942 -0.297 0.00 0.00 PROA
ATOM 4093 CB TRP P 252 -4.789 4.252 -1.503 0.00 0.00 PROA
ATOM 4094 HB1 TRP P 252 -4.006 4.487 -0.751 0.00 0.00 PROA
ATOM 4095 HB2 TRP P 252 -5.089 5.190 -2.018 0.00 0.00 PROA
ATOM 4096 CG TRP P 252 -4.203 3.179 -2.253 0.00 0.00 PROA
ATOM 4097 CD1 TRP P 252 -3.946 3.343 -3.575 0.00 0.00 PROA
ATOM 4098 HD1 TRP P 252 -4.323 4.226 -4.071 0.00 0.00 PROA
ATOM 4099 NE1 TRP P 252 -3.156 2.318 -3.967 0.00 0.00 PROA
ATOM 4100 HE1 TRP P 252 -2.765 2.148 -4.845 0.00 0.00 PROA
ATOM 4101 CE2 TRP P 252 -2.938 1.491 -2.977 0.00 0.00 PROA
ATOM 4102 CD2 TRP P 252 -3.546 2.023 -1.801 0.00 0.00 PROA
ATOM 4103 CE3 TRP P 252 -3.352 1.353 -0.528 0.00 0.00 PROA
ATOM 4104 HE3 TRP P 252 -3.789 1.725 0.386 0.00 0.00 PROA
ATOM 4105 CZ3 TRP P 252 -2.750 0.034 -0.568 0.00 0.00 PROA
ATOM 4106 HZ3 TRP P 252 -2.490 -0.502 0.333 0.00 0.00 PROA
ATOM 4107 CZ2 TRP P 252 -2.252 0.319 -2.972 0.00 0.00 PROA
ATOM 4108 HZ2 TRP P 252 -1.674 0.075 -3.851 0.00 0.00 PROA
ATOM 4109 CH2 TRP P 252 -2.144 -0.318 -1.773 0.00 0.00 PROA
ATOM 4110 HH2 TRP P 252 -1.592 -1.246 -1.796 0.00 0.00 PROA
ATOM 4111 C TRP P 252 -6.396 4.758 0.400 0.00 0.00 PROA
ATOM 4112 O TRP P 252 -5.931 4.566 1.498 0.00 0.00 PROA
ATOM 4113 N LEU P 253 -7.189 5.758 0.102 0.00 0.00 PROA
ATOM 4114 HN LEU P 253 -7.850 5.729 -0.644 0.00 0.00 PROA
ATOM 4115 CA LEU P 253 -7.293 7.013 0.867 0.00 0.00 PROA
ATOM 4116 HA LEU P 253 -6.283 7.394 0.823 0.00 0.00 PROA
ATOM 4117 CB LEU P 253 -8.288 8.009 0.165 0.00 0.00 PROA
ATOM 4118 HB1 LEU P 253 -9.288 7.526 0.203 0.00 0.00 PROA
ATOM 4119 HB2 LEU P 253 -8.123 8.091 -0.930 0.00 0.00 PROA
ATOM 4120 CG LEU P 253 -8.423 9.397 0.716 0.00 0.00 PROA
ATOM 4121 HG LEU P 253 -8.726 9.290 1.780 0.00 0.00 PROA
ATOM 4122 CD1 LEU P 253 -7.018 10.067 0.570 0.00 0.00 PROA
ATOM 4123 HD11 LEU P 253 -6.823 10.148 -0.521 0.00 0.00 PROA
ATOM 4124 HD12 LEU P 253 -6.252 9.420 1.049 0.00 0.00 PROA
ATOM 4125 HD13 LEU P 253 -7.082 11.099 0.976 0.00 0.00 PROA
ATOM 4126 CD2 LEU P 253 -9.447 10.069 -0.128 0.00 0.00 PROA
ATOM 4127 HD21 LEU P 253 -9.461 11.161 0.074 0.00 0.00 PROA
ATOM 4128 HD22 LEU P 253 -10.490 9.731 0.051 0.00 0.00 PROA
ATOM 4129 HD23 LEU P 253 -9.108 9.970 -1.182 0.00 0.00 PROA
ATOM 4130 C LEU P 253 -7.540 6.983 2.427 0.00 0.00 PROA
ATOM 4131 O LEU P 253 -6.845 7.734 3.140 0.00 0.00 PROA
ATOM 4132 N PRO P 254 -8.388 6.163 2.963 0.00 0.00 PROA
ATOM 4133 CD PRO P 254 -9.384 5.351 2.308 0.00 0.00 PROA
ATOM 4134 HD1 PRO P 254 -8.806 4.562 1.781 0.00 0.00 PROA
ATOM 4135 HD2 PRO P 254 -9.944 6.007 1.607 0.00 0.00 PROA
ATOM 4136 CA PRO P 254 -8.575 6.239 4.432 0.00 0.00 PROA
ATOM 4137 HA PRO P 254 -8.603 7.278 4.725 0.00 0.00 PROA
ATOM 4138 CB PRO P 254 -9.961 5.638 4.575 0.00 0.00 PROA
ATOM 4139 HB1 PRO P 254 -10.798 6.369 4.542 0.00 0.00 PROA
ATOM 4140 HB2 PRO P 254 -10.105 4.994 5.468 0.00 0.00 PROA
ATOM 4141 CG PRO P 254 -10.250 4.734 3.408 0.00 0.00 PROA
ATOM 4142 HG1 PRO P 254 -9.807 3.719 3.494 0.00 0.00 PROA
ATOM 4143 HG2 PRO P 254 -11.319 4.505 3.211 0.00 0.00 PROA
ATOM 4144 C PRO P 254 -7.452 5.573 5.206 0.00 0.00 PROA

ATOM 4145 O PRO P 254 -7.262 6.019 6.379 0.00 0.00 PROA
ATOM 4146 N TYR P 255 -6.687 4.619 4.652 0.00 0.00 PROA
ATOM 4147 HN TYR P 255 -6.876 4.289 3.730 0.00 0.00 PROA
ATOM 4148 CA TYR P 255 -5.496 4.158 5.361 0.00 0.00 PROA
ATOM 4149 HA TYR P 255 -5.826 3.737 6.299 0.00 0.00 PROA
ATOM 4150 CB TYR P 255 -5.045 2.991 4.487 0.00 0.00 PROA
ATOM 4151 HB1 TYR P 255 -4.773 3.265 3.445 0.00 0.00 PROA
ATOM 4152 HB2 TYR P 255 -5.853 2.231 4.548 0.00 0.00 PROA
ATOM 4153 CG TYR P 255 -3.759 2.397 5.143 0.00 0.00 PROA
ATOM 4154 CD1 TYR P 255 -3.775 1.979 6.532 0.00 0.00 PROA
ATOM 4155 HD1 TYR P 255 -4.624 2.246 7.144 0.00 0.00 PROA
ATOM 4156 CE1 TYR P 255 -2.691 1.260 7.129 0.00 0.00 PROA
ATOM 4157 HE1 TYR P 255 -2.741 0.779 8.095 0.00 0.00 PROA
ATOM 4158 CZ TYR P 255 -1.554 0.961 6.359 0.00 0.00 PROA
ATOM 4159 OH TYR P 255 -0.489 0.227 6.922 0.00 0.00 PROA
ATOM 4160 HH TYR P 255 0.167 0.150 6.226 0.00 0.00 PROA
ATOM 4161 CD2 TYR P 255 -2.584 2.168 4.471 0.00 0.00 PROA
ATOM 4162 HD2 TYR P 255 -2.463 2.520 3.457 0.00 0.00 PROA
ATOM 4163 CE2 TYR P 255 -1.472 1.537 5.047 0.00 0.00 PROA
ATOM 4164 HE2 TYR P 255 -0.600 1.455 4.415 0.00 0.00 PROA
ATOM 4165 C TYR P 255 -4.471 5.280 5.506 0.00 0.00 PROA
ATOM 4166 O TYR P 255 -3.770 5.408 6.476 0.00 0.00 PROA
ATOM 4167 N TYR P 256 -4.295 6.225 4.473 0.00 0.00 PROA
ATOM 4168 HN TYR P 256 -4.684 6.167 3.557 0.00 0.00 PROA
ATOM 4169 CA TYR P 256 -3.430 7.398 4.617 0.00 0.00 PROA
ATOM 4170 HA TYR P 256 -2.482 7.023 4.973 0.00 0.00 PROA
ATOM 4171 CB TYR P 256 -3.327 8.088 3.196 0.00 0.00 PROA
ATOM 4172 HB1 TYR P 256 -2.711 9.011 3.150 0.00 0.00 PROA
ATOM 4173 HB2 TYR P 256 -4.351 8.253 2.797 0.00 0.00 PROA
ATOM 4174 CG TYR P 256 -2.648 7.291 2.072 0.00 0.00 PROA
ATOM 4175 CD1 TYR P 256 -1.491 6.566 2.340 0.00 0.00 PROA
ATOM 4176 HD1 TYR P 256 -1.035 6.561 3.318 0.00 0.00 PROA
ATOM 4177 CE1 TYR P 256 -0.794 5.889 1.364 0.00 0.00 PROA
ATOM 4178 HE1 TYR P 256 0.092 5.288 1.506 0.00 0.00 PROA
ATOM 4179 CZ TYR P 256 -1.307 5.903 0.033 0.00 0.00 PROA
ATOM 4180 OH TYR P 256 -0.669 5.132 -0.997 0.00 0.00 PROA
ATOM 4181 HH TYR P 256 -0.631 5.647 -1.807 0.00 0.00 PROA
ATOM 4182 CD2 TYR P 256 -2.946 7.458 0.686 0.00 0.00 PROA
ATOM 4183 HD2 TYR P 256 -3.717 8.191 0.499 0.00 0.00 PROA
ATOM 4184 CE2 TYR P 256 -2.267 6.793 -0.372 0.00 0.00 PROA
ATOM 4185 HE2 TYR P 256 -2.692 6.910 -1.359 0.00 0.00 PROA
ATOM 4186 C TYR P 256 -3.987 8.456 5.654 0.00 0.00 PROA
ATOM 4187 O TYR P 256 -3.264 9.176 6.388 0.00 0.00 PROA
ATOM 4188 N ILE P 257 -5.307 8.485 5.874 0.00 0.00 PROA
ATOM 4189 HN ILE P 257 -6.006 7.969 5.385 0.00 0.00 PROA
ATOM 4190 CA ILE P 257 -5.930 9.185 7.002 0.00 0.00 PROA
ATOM 4191 HA ILE P 257 -5.434 10.143 6.958 0.00 0.00 PROA
ATOM 4192 CB ILE P 257 -7.439 9.495 6.967 0.00 0.00 PROA
ATOM 4193 HB ILE P 257 -7.915 8.491 6.941 0.00 0.00 PROA
ATOM 4194 CG2 ILE P 257 -8.014 10.194 8.240 0.00 0.00 PROA
ATOM 4195 HG21 ILE P 257 -8.199 9.434 9.029 0.00 0.00 PROA
ATOM 4196 HG22 ILE P 257 -8.978 10.746 8.267 0.00 0.00 PROA
ATOM 4197 HG23 ILE P 257 -7.250 10.957 8.499 0.00 0.00 PROA
ATOM 4198 CG1 ILE P 257 -7.742 10.319 5.714 0.00 0.00 PROA
ATOM 4199 HG11 ILE P 257 -7.637 9.674 4.815 0.00 0.00 PROA
ATOM 4200 HG12 ILE P 257 -7.042 11.162 5.529 0.00 0.00 PROA
ATOM 4201 CD ILE P 257 -9.198 10.943 5.629 0.00 0.00 PROA
ATOM 4202 HD1 ILE P 257 -9.450 11.578 6.505 0.00 0.00 PROA
ATOM 4203 HD2 ILE P 257 -10.000 10.175 5.616 0.00 0.00 PROA
ATOM 4204 HD3 ILE P 257 -9.332 11.544 4.704 0.00 0.00 PROA
ATOM 4205 C ILE P 257 -5.637 8.549 8.385 0.00 0.00 PROA

ATOM	4206	O	ILE	P	257	-5.356	9.241	9.370	0.00	0.00	PROA
ATOM	4207	N	GLY	P	258	-5.651	7.192	8.407	0.00	0.00	PROA
ATOM	4208	HN	GLY	P	258	-5.751	6.696	7.548	0.00	0.00	PROA
ATOM	4209	CA	GLY	P	258	-5.257	6.419	9.567	0.00	0.00	PROA
ATOM	4210	HA1	GLY	P	258	-5.526	5.391	9.372	0.00	0.00	PROA
ATOM	4211	HA2	GLY	P	258	-5.831	6.685	10.443	0.00	0.00	PROA
ATOM	4212	C	GLY	P	258	-3.827	6.649	9.909	0.00	0.00	PROA
ATOM	4213	O	GLY	P	258	-3.595	7.012	11.076	0.00	0.00	PROA
ATOM	4214	N	ILE	P	259	-2.857	6.660	8.986	0.00	0.00	PROA
ATOM	4215	HN	ILE	P	259	-3.067	6.331	8.069	0.00	0.00	PROA
ATOM	4216	CA	ILE	P	259	-1.481	7.116	9.156	0.00	0.00	PROA
ATOM	4217	HA	ILE	P	259	-0.977	6.597	9.958	0.00	0.00	PROA
ATOM	4218	CB	ILE	P	259	-0.778	6.718	7.839	0.00	0.00	PROA
ATOM	4219	HB	ILE	P	259	-1.439	6.903	6.966	0.00	0.00	PROA
ATOM	4220	CG2	ILE	P	259	0.544	7.473	7.640	0.00	0.00	PROA
ATOM	4221	HG21	ILE	P	259	1.210	7.210	6.790	0.00	0.00	PROA
ATOM	4222	HG22	ILE	P	259	1.140	7.357	8.569	0.00	0.00	PROA
ATOM	4223	HG23	ILE	P	259	0.309	8.552	7.518	0.00	0.00	PROA
ATOM	4224	CG1	ILE	P	259	-0.491	5.209	7.737	0.00	0.00	PROA
ATOM	4225	HG11	ILE	P	259	-1.349	4.648	8.163	0.00	0.00	PROA
ATOM	4226	HG12	ILE	P	259	0.382	4.878	8.340	0.00	0.00	PROA
ATOM	4227	CD	ILE	P	259	-0.269	4.697	6.342	0.00	0.00	PROA
ATOM	4228	HD1	ILE	P	259	-0.099	3.605	6.232	0.00	0.00	PROA
ATOM	4229	HD2	ILE	P	259	0.583	5.212	5.849	0.00	0.00	PROA
ATOM	4230	HD3	ILE	P	259	-1.117	5.000	5.691	0.00	0.00	PROA
ATOM	4231	C	ILE	P	259	-1.374	8.579	9.494	0.00	0.00	PROA
ATOM	4232	O	ILE	P	259	-0.713	8.939	10.467	0.00	0.00	PROA
ATOM	4233	N	SER	P	260	-1.999	9.478	8.816	0.00	0.00	PROA
ATOM	4234	HN	SER	P	260	-2.550	9.047	8.105	0.00	0.00	PROA
ATOM	4235	CA	SER	P	260	-2.031	10.929	9.228	0.00	0.00	PROA
ATOM	4236	HA	SER	P	260	-0.982	11.152	9.099	0.00	0.00	PROA
ATOM	4237	CB	SER	P	260	-2.696	11.767	8.155	0.00	0.00	PROA
ATOM	4238	HB1	SER	P	260	-2.555	12.778	8.594	0.00	0.00	PROA
ATOM	4239	HB2	SER	P	260	-3.790	11.584	8.216	0.00	0.00	PROA
ATOM	4240	OG	SER	P	260	-2.074	11.601	6.889	0.00	0.00	PROA
ATOM	4241	HG1	SER	P	260	-2.262	10.764	6.459	0.00	0.00	PROA
ATOM	4242	C	SER	P	260	-2.527	11.223	10.601	0.00	0.00	PROA
ATOM	4243	O	SER	P	260	-1.868	11.961	11.394	0.00	0.00	PROA
ATOM	4244	N	ILE	P	261	-3.687	10.664	11.089	0.00	0.00	PROA
ATOM	4245	HN	ILE	P	261	-4.243	10.135	10.453	0.00	0.00	PROA
ATOM	4246	CA	ILE	P	261	-4.085	10.769	12.499	0.00	0.00	PROA
ATOM	4247	HA	ILE	P	261	-4.210	11.821	12.710	0.00	0.00	PROA
ATOM	4248	CB	ILE	P	261	-5.512	10.192	12.506	0.00	0.00	PROA
ATOM	4249	HB	ILE	P	261	-5.490	9.127	12.191	0.00	0.00	PROA
ATOM	4250	CG2	ILE	P	261	-5.974	10.075	13.934	0.00	0.00	PROA
ATOM	4251	HG21	ILE	P	261	-5.853	11.035	14.479	0.00	0.00	PROA
ATOM	4252	HG22	ILE	P	261	-5.505	9.194	14.422	0.00	0.00	PROA
ATOM	4253	HG23	ILE	P	261	-7.061	9.845	13.967	0.00	0.00	PROA
ATOM	4254	CG1	ILE	P	261	-6.417	10.969	11.560	0.00	0.00	PROA
ATOM	4255	HG11	ILE	P	261	-7.484	10.659	11.558	0.00	0.00	PROA
ATOM	4256	HG12	ILE	P	261	-6.035	10.898	10.519	0.00	0.00	PROA
ATOM	4257	CD	ILE	P	261	-6.485	12.471	11.887	0.00	0.00	PROA
ATOM	4258	HD1	ILE	P	261	-7.353	12.909	11.350	0.00	0.00	PROA
ATOM	4259	HD2	ILE	P	261	-5.563	13.023	11.607	0.00	0.00	PROA
ATOM	4260	HD3	ILE	P	261	-6.550	12.660	12.980	0.00	0.00	PROA
ATOM	4261	C	ILE	P	261	-3.175	10.171	13.555	0.00	0.00	PROA
ATOM	4262	O	ILE	P	261	-2.876	10.886	14.539	0.00	0.00	PROA
ATOM	4263	N	ASP	P	262	-2.650	8.988	13.276	0.00	0.00	PROA
ATOM	4264	HN	ASP	P	262	-2.894	8.506	12.438	0.00	0.00	PROA
ATOM	4265	CA	ASP	P	262	-1.635	8.346	14.058	0.00	0.00	PROA
ATOM	4266	HA	ASP	P	262	-2.080	8.159	15.025	0.00	0.00	PROA

```
ATOM   4267  CB  ASP P 262      -1.141   7.000  13.376  0.00  0.00      PROA
ATOM   4268  HB1 ASP P 262      -0.654   7.119  12.385  0.00  0.00      PROA
ATOM   4269  HB2 ASP P 262      -2.104   6.477  13.191  0.00  0.00      PROA
ATOM   4270  CG  ASP P 262      -0.267   6.119  14.197  0.00  0.00      PROA
ATOM   4271  OD1 ASP P 262       0.614   5.444  13.640  0.00  0.00      PROA
ATOM   4272  OD2 ASP P 262      -0.608   5.963  15.421  0.00  0.00      PROA
ATOM   4273  C   ASP P 262      -0.455   9.272  14.289  0.00  0.00      PROA
ATOM   4274  O   ASP P 262       0.135   9.309  15.333  0.00  0.00      PROA
ATOM   4275  N   SER P 263       0.012  10.005  13.297  0.00  0.00      PROA
ATOM   4276  HN  SER P 263      -0.289   9.960  12.348  0.00  0.00      PROA
ATOM   4277  CA  SER P 263       1.046  10.978  13.313  0.00  0.00      PROA
ATOM   4278  HA  SER P 263       1.813  10.408  13.818  0.00  0.00      PROA
ATOM   4279  CB  SER P 263       1.423  11.573  11.956  0.00  0.00      PROA
ATOM   4280  HB1 SER P 263       2.088  12.452  11.817  0.00  0.00      PROA
ATOM   4281  HB2 SER P 263       0.496  11.680  11.354  0.00  0.00      PROA
ATOM   4282  OG  SER P 263       2.109  10.616  11.194  0.00  0.00      PROA
ATOM   4283  HG1 SER P 263       2.782  10.204  11.742  0.00  0.00      PROA
ATOM   4284  C   SER P 263       0.736  12.123  14.229  0.00  0.00      PROA
ATOM   4285  O   SER P 263       1.547  12.524  15.055  0.00  0.00      PROA
ATOM   4286  N   PHE P 264      -0.518  12.659  14.136  0.00  0.00      PROA
ATOM   4287  HN  PHE P 264      -1.068  12.123  13.500  0.00  0.00      PROA
ATOM   4288  CA  PHE P 264      -0.981  13.805  14.939  0.00  0.00      PROA
ATOM   4289  HA  PHE P 264      -0.178  14.524  14.991  0.00  0.00      PROA
ATOM   4290  CB  PHE P 264      -2.189  14.532  14.247  0.00  0.00      PROA
ATOM   4291  HB1 PHE P 264      -2.439  15.458  14.808  0.00  0.00      PROA
ATOM   4292  HB2 PHE P 264      -3.053  13.836  14.201  0.00  0.00      PROA
ATOM   4293  CG  PHE P 264      -1.893  15.134  12.863  0.00  0.00      PROA
ATOM   4294  CD1 PHE P 264      -0.736  15.914  12.616  0.00  0.00      PROA
ATOM   4295  HD1 PHE P 264       0.052  16.031  13.344  0.00  0.00      PROA
ATOM   4296  CE1 PHE P 264      -0.397  16.362  11.354  0.00  0.00      PROA
ATOM   4297  HE1 PHE P 264       0.566  16.829  11.210  0.00  0.00      PROA
ATOM   4298  CZ  PHE P 264      -1.254  16.091  10.271  0.00  0.00      PROA
ATOM   4299  HZ  PHE P 264      -1.010  16.476   9.291  0.00  0.00      PROA
ATOM   4300  CD2 PHE P 264      -2.836  15.120  11.828  0.00  0.00      PROA
ATOM   4301  HD2 PHE P 264      -3.846  14.755  11.943  0.00  0.00      PROA
ATOM   4302  CE2 PHE P 264      -2.524  15.448  10.469  0.00  0.00      PROA
ATOM   4303  HE2 PHE P 264      -3.237  15.301   9.671  0.00  0.00      PROA
ATOM   4304  C   PHE P 264      -1.254  13.489  16.407  0.00  0.00      PROA
ATOM   4305  O   PHE P 264      -1.024  14.366  17.248  0.00  0.00      PROA
ATOM   4306  N   ILE P 265      -1.766  12.311  16.796  0.00  0.00      PROA
ATOM   4307  HN  ILE P 265      -2.079  11.683  16.088  0.00  0.00      PROA
ATOM   4308  CA  ILE P 265      -1.889  11.741  18.100  0.00  0.00      PROA
ATOM   4309  HA  ILE P 265      -2.377  12.497  18.697  0.00  0.00      PROA
ATOM   4310  CB  ILE P 265      -2.645  10.392  18.062  0.00  0.00      PROA
ATOM   4311  HB  ILE P 265      -2.117   9.732  17.341  0.00  0.00      PROA
ATOM   4312  CG2 ILE P 265      -2.614   9.636  19.362  0.00  0.00      PROA
ATOM   4313 HG21 ILE P 265      -3.533   9.845  19.950  0.00  0.00      PROA
ATOM   4314 HG22 ILE P 265      -1.712   9.952  19.929  0.00  0.00      PROA
ATOM   4315 HG23 ILE P 265      -2.578   8.531  19.260  0.00  0.00      PROA
ATOM   4316  CG1 ILE P 265      -4.089  10.522  17.612  0.00  0.00      PROA
ATOM   4317 HG11 ILE P 265      -4.178  11.152  16.702  0.00  0.00      PROA
ATOM   4318 HG12 ILE P 265      -4.681  11.050  18.390  0.00  0.00      PROA
ATOM   4319  CD  ILE P 265      -4.830   9.131  17.263  0.00  0.00      PROA
ATOM   4320  HD1 ILE P 265      -5.804   9.260  16.745  0.00  0.00      PROA
ATOM   4321  HD2 ILE P 265      -4.965   8.595  18.226  0.00  0.00      PROA
ATOM   4322  HD3 ILE P 265      -4.205   8.510  16.586  0.00  0.00      PROA
ATOM   4323  C   ILE P 265      -0.443  11.583  18.649  0.00  0.00      PROA
ATOM   4324  O   ILE P 265      -0.116  12.083  19.783  0.00  0.00      PROA
ATOM   4325  N   LEU P 266       0.414  11.014  17.900  0.00  0.00      PROA
ATOM   4326  HN  LEU P 266       0.170  10.655  17.003  0.00  0.00      PROA
ATOM   4327  CA  LEU P 266       1.722  10.775  18.318  0.00  0.00      PROA
```

ATOM	4328	HA	LEU	P	266	1.608	10.128	19.176	0.00	0.00	PROA
ATOM	4329	CB	LEU	P	266	2.468	10.009	17.180	0.00	0.00	PROA
ATOM	4330	HB1	LEU	P	266	2.248	10.587	16.257	0.00	0.00	PROA
ATOM	4331	HB2	LEU	P	266	2.030	9.014	16.950	0.00	0.00	PROA
ATOM	4332	CG	LEU	P	266	3.950	9.703	17.352	0.00	0.00	PROA
ATOM	4333	HG	LEU	P	266	4.428	10.462	18.007	0.00	0.00	PROA
ATOM	4334	CD1	LEU	P	266	4.105	8.449	18.152	0.00	0.00	PROA
ATOM	4335	HD11	LEU	P	266	5.158	8.110	18.262	0.00	0.00	PROA
ATOM	4336	HD12	LEU	P	266	3.697	7.626	17.527	0.00	0.00	PROA
ATOM	4337	HD13	LEU	P	266	3.561	8.402	19.119	0.00	0.00	PROA
ATOM	4338	CD2	LEU	P	266	4.744	9.628	16.057	0.00	0.00	PROA
ATOM	4339	HD21	LEU	P	266	4.038	9.266	15.279	0.00	0.00	PROA
ATOM	4340	HD22	LEU	P	266	5.679	9.039	16.176	0.00	0.00	PROA
ATOM	4341	HD23	LEU	P	266	4.986	10.644	15.678	0.00	0.00	PROA
ATOM	4342	C	LEU	P	266	2.562	11.988	18.764	0.00	0.00	PROA
ATOM	4343	O	LEU	P	266	3.262	11.968	19.763	0.00	0.00	PROA
ATOM	4344	N	LEU	P	267	2.420	13.158	18.027	0.00	0.00	PROA
ATOM	4345	HN	LEU	P	267	1.832	13.141	17.223	0.00	0.00	PROA
ATOM	4346	CA	LEU	P	267	3.101	14.429	18.261	0.00	0.00	PROA
ATOM	4347	HA	LEU	P	267	4.132	14.170	18.457	0.00	0.00	PROA
ATOM	4348	CB	LEU	P	267	3.047	15.262	16.940	0.00	0.00	PROA
ATOM	4349	HB1	LEU	P	267	3.342	16.324	17.080	0.00	0.00	PROA
ATOM	4350	HB2	LEU	P	267	2.000	15.274	16.567	0.00	0.00	PROA
ATOM	4351	CG	LEU	P	267	4.041	14.635	15.904	0.00	0.00	PROA
ATOM	4352	HG	LEU	P	267	3.951	13.528	15.859	0.00	0.00	PROA
ATOM	4353	CD1	LEU	P	267	3.729	15.185	14.413	0.00	0.00	PROA
ATOM	4354	HD11	LEU	P	267	4.180	16.175	14.186	0.00	0.00	PROA
ATOM	4355	HD12	LEU	P	267	2.620	15.226	14.366	0.00	0.00	PROA
ATOM	4356	HD13	LEU	P	267	4.071	14.450	13.653	0.00	0.00	PROA
ATOM	4357	CD2	LEU	P	267	5.474	14.925	16.271	0.00	0.00	PROA
ATOM	4358	HD21	LEU	P	267	5.622	15.983	16.578	0.00	0.00	PROA
ATOM	4359	HD22	LEU	P	267	6.236	14.650	15.511	0.00	0.00	PROA
ATOM	4360	HD23	LEU	P	267	5.607	14.313	17.188	0.00	0.00	PROA
ATOM	4361	C	LEU	P	267	2.505	15.253	19.457	0.00	0.00	PROA
ATOM	4362	O	LEU	P	267	3.050	16.204	19.956	0.00	0.00	PROA
ATOM	4363	N	GLU	P	268	1.276	14.836	19.885	0.00	0.00	PROA
ATOM	4364	HN	GLU	P	268	0.917	14.018	19.442	0.00	0.00	PROA
ATOM	4365	CA	GLU	P	268	0.393	15.463	20.866	0.00	0.00	PROA
ATOM	4366	HA	GLU	P	268	-0.346	14.678	20.943	0.00	0.00	PROA
ATOM	4367	CB	GLU	P	268	1.143	15.604	22.236	0.00	0.00	PROA
ATOM	4368	HB1	GLU	P	268	0.592	16.221	22.978	0.00	0.00	PROA
ATOM	4369	HB2	GLU	P	268	2.096	16.136	22.031	0.00	0.00	PROA
ATOM	4370	CG	GLU	P	268	1.376	14.259	22.926	0.00	0.00	PROA
ATOM	4371	HG1	GLU	P	268	2.262	13.696	22.562	0.00	0.00	PROA
ATOM	4372	HG2	GLU	P	268	0.452	13.644	22.888	0.00	0.00	PROA
ATOM	4373	CD	GLU	P	268	1.619	14.508	24.390	0.00	0.00	PROA
ATOM	4374	OE1	GLU	P	268	2.802	14.840	24.805	0.00	0.00	PROA
ATOM	4375	OE2	GLU	P	268	0.653	14.471	25.161	0.00	0.00	PROA
ATOM	4376	C	GLU	P	268	-0.365	16.718	20.362	0.00	0.00	PROA
ATOM	4377	O	GLU	P	268	-0.795	17.683	21.037	0.00	0.00	PROA
ATOM	4378	N	ILE	P	269	-0.560	16.786	19.028	0.00	0.00	PROA
ATOM	4379	HN	ILE	P	269	-0.302	16.059	18.397	0.00	0.00	PROA
ATOM	4380	CA	ILE	P	269	-1.479	17.714	18.386	0.00	0.00	PROA
ATOM	4381	HA	ILE	P	269	-1.279	18.729	18.693	0.00	0.00	PROA
ATOM	4382	CB	ILE	P	269	-1.280	17.692	16.925	0.00	0.00	PROA
ATOM	4383	HB	ILE	P	269	-1.466	16.651	16.586	0.00	0.00	PROA
ATOM	4384	CG2	ILE	P	269	-2.367	18.549	16.246	0.00	0.00	PROA
ATOM	4385	HG21	ILE	P	269	-3.408	18.170	16.324	0.00	0.00	PROA
ATOM	4386	HG22	ILE	P	269	-2.028	18.663	15.194	0.00	0.00	PROA
ATOM	4387	HG23	ILE	P	269	-2.316	19.521	16.781	0.00	0.00	PROA
ATOM	4388	CG1	ILE	P	269	0.142	18.103	16.543	0.00	0.00	PROA

ATOM 4389 HG11 ILE P 269 0.424 17.700 15.546 0.00 0.00 PROA
ATOM 4390 HG12 ILE P 269 0.879 17.745 17.292 0.00 0.00 PROA
ATOM 4391 CD ILE P 269 0.424 19.604 16.712 0.00 0.00 PROA
ATOM 4392 HD1 ILE P 269 0.041 19.931 17.703 0.00 0.00 PROA
ATOM 4393 HD2 ILE P 269 -0.149 20.147 15.930 0.00 0.00 PROA
ATOM 4394 HD3 ILE P 269 1.526 19.690 16.608 0.00 0.00 PROA
ATOM 4395 C ILE P 269 -2.888 17.396 18.807 0.00 0.00 PROA
ATOM 4396 O ILE P 269 -3.603 18.258 19.354 0.00 0.00 PROA
ATOM 4397 N ILE P 270 -3.317 16.153 18.613 0.00 0.00 PROA
ATOM 4398 HN ILE P 270 -2.656 15.500 18.250 0.00 0.00 PROA
ATOM 4399 CA ILE P 270 -4.511 15.419 19.174 0.00 0.00 PROA
ATOM 4400 HA ILE P 270 -5.290 16.098 19.490 0.00 0.00 PROA
ATOM 4401 CB ILE P 270 -4.979 14.233 18.282 0.00 0.00 PROA
ATOM 4402 HB ILE P 270 -4.122 13.526 18.276 0.00 0.00 PROA
ATOM 4403 CG2 ILE P 270 -6.318 13.591 18.761 0.00 0.00 PROA
ATOM 4404 HG21 ILE P 270 -6.552 12.681 18.167 0.00 0.00 PROA
ATOM 4405 HG22 ILE P 270 -7.076 14.381 18.569 0.00 0.00 PROA
ATOM 4406 HG23 ILE P 270 -6.316 13.453 19.863 0.00 0.00 PROA
ATOM 4407 CG1 ILE P 270 -5.294 14.766 16.856 0.00 0.00 PROA
ATOM 4408 HG11 ILE P 270 -4.347 15.104 16.384 0.00 0.00 PROA
ATOM 4409 HG12 ILE P 270 -5.951 15.643 17.041 0.00 0.00 PROA
ATOM 4410 CD ILE P 270 -5.859 13.838 15.837 0.00 0.00 PROA
ATOM 4411 HD1 ILE P 270 -5.447 12.808 15.792 0.00 0.00 PROA
ATOM 4412 HD2 ILE P 270 -5.793 14.344 14.850 0.00 0.00 PROA
ATOM 4413 HD3 ILE P 270 -6.928 13.732 16.123 0.00 0.00 PROA
ATOM 4414 C ILE P 270 -3.874 14.937 20.500 0.00 0.00 PROA
ATOM 4415 O ILE P 270 -2.725 14.462 20.632 0.00 0.00 PROA
ATOM 4416 N LYS P 271 -4.607 15.243 21.567 0.00 0.00 PROA
ATOM 4417 HN LYS P 271 -5.504 15.666 21.465 0.00 0.00 PROA
ATOM 4418 CA LYS P 271 -4.284 15.071 22.978 0.00 0.00 PROA
ATOM 4419 HA LYS P 271 -3.268 14.720 23.085 0.00 0.00 PROA
ATOM 4420 CB LYS P 271 -4.464 16.369 23.719 0.00 0.00 PROA
ATOM 4421 HB1 LYS P 271 -4.430 16.197 24.816 0.00 0.00 PROA
ATOM 4422 HB2 LYS P 271 -5.435 16.787 23.379 0.00 0.00 PROA
ATOM 4423 CG LYS P 271 -3.325 17.329 23.379 0.00 0.00 PROA
ATOM 4424 HG1 LYS P 271 -3.610 18.307 23.822 0.00 0.00 PROA
ATOM 4425 HG2 LYS P 271 -3.311 17.400 22.270 0.00 0.00 PROA
ATOM 4426 CD LYS P 271 -1.941 16.921 23.940 0.00 0.00 PROA
ATOM 4427 HD1 LYS P 271 -1.166 17.706 23.803 0.00 0.00 PROA
ATOM 4428 HD2 LYS P 271 -1.613 15.935 23.548 0.00 0.00 PROA
ATOM 4429 CE LYS P 271 -1.964 16.730 25.519 0.00 0.00 PROA
ATOM 4430 HE1 LYS P 271 -2.454 15.771 25.794 0.00 0.00 PROA
ATOM 4431 HE2 LYS P 271 -2.500 17.570 26.011 0.00 0.00 PROA
ATOM 4432 NZ LYS P 271 -0.627 16.659 26.147 0.00 0.00 PROA
ATOM 4433 HZ1 LYS P 271 -0.858 16.434 27.135 0.00 0.00 PROA
ATOM 4434 HZ2 LYS P 271 -0.111 17.560 26.081 0.00 0.00 PROA
ATOM 4435 HZ3 LYS P 271 0.024 15.953 25.747 0.00 0.00 PROA
ATOM 4436 C LYS P 271 -5.257 14.040 23.512 0.00 0.00 PROA
ATOM 4437 O LYS P 271 -5.193 13.645 24.693 0.00 0.00 PROA
ATOM 4438 N GLN P 272 -6.244 13.565 22.730 0.00 0.00 PROA
ATOM 4439 HN GLN P 272 -6.432 14.140 21.937 0.00 0.00 PROA
ATOM 4440 CA GLN P 272 -7.323 12.665 23.125 0.00 0.00 PROA
ATOM 4441 HA GLN P 272 -7.941 13.152 23.864 0.00 0.00 PROA
ATOM 4442 CB GLN P 272 -8.204 12.303 21.847 0.00 0.00 PROA
ATOM 4443 HB1 GLN P 272 -8.823 11.488 22.279 0.00 0.00 PROA
ATOM 4444 HB2 GLN P 272 -7.606 11.800 21.056 0.00 0.00 PROA
ATOM 4445 CG GLN P 272 -9.144 13.395 21.223 0.00 0.00 PROA
ATOM 4446 HG1 GLN P 272 -9.863 12.956 20.500 0.00 0.00 PROA
ATOM 4447 HG2 GLN P 272 -8.569 14.218 20.748 0.00 0.00 PROA
ATOM 4448 CD GLN P 272 -10.100 14.079 22.172 0.00 0.00 PROA
ATOM 4449 OE1 GLN P 272 -10.125 15.310 22.368 0.00 0.00 PROA

ATOM 4450 NE2 GLN P 272 -10.928 13.300 22.890 0.00 0.00 PROA
ATOM 4451 HE21 GLN P 272 -11.563 13.805 23.474 0.00 0.00 PROA
ATOM 4452 HE22 GLN P 272 -10.983 12.301 22.865 0.00 0.00 PROA
ATOM 4453 C GLN P 272 -6.788 11.372 23.875 0.00 0.00 PROA
ATOM 4454 O GLN P 272 -5.690 10.905 23.563 0.00 0.00 PROA
ATOM 4455 N GLY P 273 -7.448 10.923 24.914 0.00 0.00 PROA
ATOM 4456 HN GLY P 273 -8.336 11.335 25.103 0.00 0.00 PROA
ATOM 4457 CA GLY P 273 -6.950 9.904 25.772 0.00 0.00 PROA
ATOM 4458 HA1 GLY P 273 -7.622 9.861 26.616 0.00 0.00 PROA
ATOM 4459 HA2 GLY P 273 -5.997 10.232 26.160 0.00 0.00 PROA
ATOM 4460 C GLY P 273 -6.750 8.515 25.221 0.00 0.00 PROA
ATOM 4461 O GLY P 273 -6.978 8.238 24.047 0.00 0.00 PROA
ATOM 4462 N CYS P 274 -6.316 7.544 26.085 0.00 0.00 PROA
ATOM 4463 HN CYS P 274 -6.112 7.810 27.024 0.00 0.00 PROA
ATOM 4464 CA CYS P 274 -5.853 6.190 25.785 0.00 0.00 PROA
ATOM 4465 HA CYS P 274 -4.986 6.327 25.156 0.00 0.00 PROA
ATOM 4466 CB CYS P 274 -5.435 5.540 27.147 0.00 0.00 PROA
ATOM 4467 HB1 CYS P 274 -4.945 4.576 26.894 0.00 0.00 PROA
ATOM 4468 HB2 CYS P 274 -6.189 5.390 27.948 0.00 0.00 PROA
ATOM 4469 SG CYS P 274 -4.092 6.380 27.995 0.00 0.00 PROA
ATOM 4470 C CYS P 274 -6.883 5.365 25.125 0.00 0.00 PROA
ATOM 4471 O CYS P 274 -6.534 4.628 24.185 0.00 0.00 PROA
ATOM 4472 N GLU P 275 -8.060 5.453 25.642 0.00 0.00 PROA
ATOM 4473 HN GLU P 275 -8.147 5.837 26.558 0.00 0.00 PROA
ATOM 4474 CA GLU P 275 -9.302 4.931 25.097 0.00 0.00 PROA
ATOM 4475 HA GLU P 275 -9.156 3.877 24.910 0.00 0.00 PROA
ATOM 4476 CB GLU P 275 -10.512 5.257 25.979 0.00 0.00 PROA
ATOM 4477 HB1 GLU P 275 -11.433 4.779 25.582 0.00 0.00 PROA
ATOM 4478 HB2 GLU P 275 -10.767 6.320 26.181 0.00 0.00 PROA
ATOM 4479 CG GLU P 275 -10.316 4.625 27.425 0.00 0.00 PROA
ATOM 4480 HG1 GLU P 275 -9.269 4.739 27.778 0.00 0.00 PROA
ATOM 4481 HG2 GLU P 275 -10.381 3.526 27.279 0.00 0.00 PROA
ATOM 4482 CD GLU P 275 -11.241 5.223 28.465 0.00 0.00 PROA
ATOM 4483 OE1 GLU P 275 -10.721 6.119 29.132 0.00 0.00 PROA
ATOM 4484 OE2 GLU P 275 -12.366 4.688 28.603 0.00 0.00 PROA
ATOM 4485 C GLU P 275 -9.595 5.446 23.660 0.00 0.00 PROA
ATOM 4486 O GLU P 275 -10.067 4.630 22.818 0.00 0.00 PROA
ATOM 4487 N PHE P 276 -9.311 6.704 23.291 0.00 0.00 PROA
ATOM 4488 HN PHE P 276 -9.146 7.327 24.051 0.00 0.00 PROA
ATOM 4489 CA PHE P 276 -9.557 7.286 21.943 0.00 0.00 PROA
ATOM 4490 HA PHE P 276 -10.594 7.074 21.730 0.00 0.00 PROA
ATOM 4491 CB PHE P 276 -9.254 8.860 21.856 0.00 0.00 PROA
ATOM 4492 HB1 PHE P 276 -8.166 9.027 22.010 0.00 0.00 PROA
ATOM 4493 HB2 PHE P 276 -9.743 9.495 22.626 0.00 0.00 PROA
ATOM 4494 CG PHE P 276 -9.768 9.436 20.543 0.00 0.00 PROA
ATOM 4495 CD1 PHE P 276 -8.797 9.755 19.518 0.00 0.00 PROA
ATOM 4496 HD1 PHE P 276 -7.783 9.512 19.799 0.00 0.00 PROA
ATOM 4497 CE1 PHE P 276 -9.323 10.253 18.322 0.00 0.00 PROA
ATOM 4498 HE1 PHE P 276 -8.582 10.393 17.549 0.00 0.00 PROA
ATOM 4499 CZ PHE P 276 -10.678 10.530 18.143 0.00 0.00 PROA
ATOM 4500 HZ PHE P 276 -11.026 10.883 17.184 0.00 0.00 PROA
ATOM 4501 CD2 PHE P 276 -11.137 9.699 20.341 0.00 0.00 PROA
ATOM 4502 HD2 PHE P 276 -11.748 9.457 21.198 0.00 0.00 PROA
ATOM 4503 CE2 PHE P 276 -11.563 10.208 19.191 0.00 0.00 PROA
ATOM 4504 HE2 PHE P 276 -12.609 10.445 19.062 0.00 0.00 PROA
ATOM 4505 C PHE P 276 -8.683 6.574 20.930 0.00 0.00 PROA
ATOM 4506 O PHE P 276 -9.074 6.209 19.868 0.00 0.00 PROA
ATOM 4507 N GLU P 277 -7.431 6.393 21.404 0.00 0.00 PROA
ATOM 4508 HN GLU P 277 -7.212 6.890 22.240 0.00 0.00 PROA
ATOM 4509 CA GLU P 277 -6.370 5.682 20.701 0.00 0.00 PROA
ATOM 4510 HA GLU P 277 -6.276 6.127 19.722 0.00 0.00 PROA

ATOM 4511 CB GLU P 277 -4.982 5.893 21.365 0.00 0.00 PROA
ATOM 4512 HB1 GLU P 277 -4.215 5.290 20.835 0.00 0.00 PROA
ATOM 4513 HB2 GLU P 277 -5.119 5.566 22.418 0.00 0.00 PROA
ATOM 4514 CG GLU P 277 -4.437 7.352 21.379 0.00 0.00 PROA
ATOM 4515 HG1 GLU P 277 -5.050 7.998 22.043 0.00 0.00 PROA
ATOM 4516 HG2 GLU P 277 -4.440 7.757 20.344 0.00 0.00 PROA
ATOM 4517 CD GLU P 277 -3.002 7.317 21.926 0.00 0.00 PROA
ATOM 4518 OE1 GLU P 277 -2.795 7.874 22.982 0.00 0.00 PROA
ATOM 4519 OE2 GLU P 277 -2.146 6.828 21.147 0.00 0.00 PROA
ATOM 4520 C GLU P 277 -6.730 4.195 20.492 0.00 0.00 PROA
ATOM 4521 O GLU P 277 -6.617 3.682 19.368 0.00 0.00 PROA
ATOM 4522 N ASN P 278 -7.213 3.554 21.494 0.00 0.00 PROA
ATOM 4523 HN ASN P 278 -7.353 4.012 22.369 0.00 0.00 PROA
ATOM 4524 CA ASN P 278 -7.748 2.224 21.267 0.00 0.00 PROA
ATOM 4525 HA ASN P 278 -6.935 1.590 20.946 0.00 0.00 PROA
ATOM 4526 CB ASN P 278 -8.268 1.693 22.629 0.00 0.00 PROA
ATOM 4527 HB1 ASN P 278 -8.861 0.768 22.467 0.00 0.00 PROA
ATOM 4528 HB2 ASN P 278 -8.838 2.569 23.006 0.00 0.00 PROA
ATOM 4529 CG ASN P 278 -7.242 1.352 23.698 0.00 0.00 PROA
ATOM 4530 OD1 ASN P 278 -6.023 1.293 23.454 0.00 0.00 PROA
ATOM 4531 ND2 ASN P 278 -7.698 1.263 25.011 0.00 0.00 PROA
ATOM 4532 HD21 ASN P 278 -8.687 1.278 25.153 0.00 0.00 PROA
ATOM 4533 HD22 ASN P 278 -7.035 1.243 25.759 0.00 0.00 PROA
ATOM 4534 C ASN P 278 -8.877 2.178 20.235 0.00 0.00 PROA
ATOM 4535 O ASN P 278 -8.859 1.492 19.249 0.00 0.00 PROA
ATOM 4536 N THR P 279 -9.891 3.092 20.339 0.00 0.00 PROA
ATOM 4537 HN THR P 279 -9.914 3.727 21.108 0.00 0.00 PROA
ATOM 4538 CA THR P 279 -10.938 3.290 19.323 0.00 0.00 PROA
ATOM 4539 HA THR P 279 -11.386 2.309 19.272 0.00 0.00 PROA
ATOM 4540 CB THR P 279 -12.067 4.330 19.587 0.00 0.00 PROA
ATOM 4541 HB THR P 279 -11.699 5.378 19.551 0.00 0.00 PROA
ATOM 4542 OG1 THR P 279 -12.667 4.036 20.839 0.00 0.00 PROA
ATOM 4543 HG1 THR P 279 -12.546 4.806 21.401 0.00 0.00 PROA
ATOM 4544 CG2 THR P 279 -13.131 4.176 18.498 0.00 0.00 PROA
ATOM 4545 HG21 THR P 279 -14.045 4.605 18.962 0.00 0.00 PROA
ATOM 4546 HG22 THR P 279 -13.320 3.083 18.434 0.00 0.00 PROA
ATOM 4547 HG23 THR P 279 -12.885 4.536 17.476 0.00 0.00 PROA
ATOM 4548 C THR P 279 -10.401 3.539 17.883 0.00 0.00 PROA
ATOM 4549 O THR P 279 -10.812 3.002 16.844 0.00 0.00 PROA
ATOM 4550 N VAL P 280 -9.535 4.556 17.724 0.00 0.00 PROA
ATOM 4551 HN VAL P 280 -9.237 4.969 18.581 0.00 0.00 PROA
ATOM 4552 CA VAL P 280 -8.878 4.940 16.477 0.00 0.00 PROA
ATOM 4553 HA VAL P 280 -9.711 5.053 15.798 0.00 0.00 PROA
ATOM 4554 CB VAL P 280 -8.042 6.299 16.654 0.00 0.00 PROA
ATOM 4555 HB VAL P 280 -7.567 6.225 17.656 0.00 0.00 PROA
ATOM 4556 CG1 VAL P 280 -6.935 6.441 15.554 0.00 0.00 PROA
ATOM 4557 HG11 VAL P 280 -6.159 7.176 15.858 0.00 0.00 PROA
ATOM 4558 HG12 VAL P 280 -7.332 6.883 14.615 0.00 0.00 PROA
ATOM 4559 HG13 VAL P 280 -6.309 5.535 15.404 0.00 0.00 PROA
ATOM 4560 CG2 VAL P 280 -8.941 7.538 16.764 0.00 0.00 PROA
ATOM 4561 HG21 VAL P 280 -9.362 7.849 15.784 0.00 0.00 PROA
ATOM 4562 HG22 VAL P 280 -8.284 8.372 17.091 0.00 0.00 PROA
ATOM 4563 HG23 VAL P 280 -9.707 7.288 17.529 0.00 0.00 PROA
ATOM 4564 C VAL P 280 -8.174 3.790 15.853 0.00 0.00 PROA
ATOM 4565 O VAL P 280 -8.221 3.594 14.601 0.00 0.00 PROA
ATOM 4566 N HSE P 281 -7.476 2.935 16.700 0.00 0.00 PROA
ATOM 4567 HN HSE P 281 -7.291 3.178 17.649 0.00 0.00 PROA
ATOM 4568 CA HSE P 281 -6.837 1.741 16.278 0.00 0.00 PROA
ATOM 4569 HA HSE P 281 -6.187 2.028 15.465 0.00 0.00 PROA
ATOM 4570 CB HSE P 281 -6.037 1.049 17.399 0.00 0.00 PROA
ATOM 4571 HB1 HSE P 281 -6.647 0.698 18.259 0.00 0.00 PROA

ATOM	4572	HB2	HSE	P	281	-5.303	1.791	17.782	0.00	0.00	PROA
ATOM	4573	ND1	HSE	P	281	-5.006	-1.116	17.657	0.00	0.00	PROA
ATOM	4574	CG	HSE	P	281	-5.218	-0.085	16.868	0.00	0.00	PROA
ATOM	4575	CE1	HSE	P	281	-4.437	-2.005	16.843	0.00	0.00	PROA
ATOM	4576	HE1	HSE	P	281	-4.081	-3.022	17.008	0.00	0.00	PROA
ATOM	4577	NE2	HSE	P	281	-4.217	-1.563	15.621	0.00	0.00	PROA
ATOM	4578	HE2	HSE	P	281	-3.526	-1.875	14.968	0.00	0.00	PROA
ATOM	4579	CD2	HSE	P	281	-4.658	-0.221	15.642	0.00	0.00	PROA
ATOM	4580	HD2	HSE	P	281	-4.736	0.399	14.758	0.00	0.00	PROA
ATOM	4581	C	HSE	P	281	-7.734	0.709	15.561	0.00	0.00	PROA
ATOM	4582	O	HSE	P	281	-7.308	-0.052	14.722	0.00	0.00	PROA
ATOM	4583	N	LYS	P	282	-8.955	0.525	16.051	0.00	0.00	PROA
ATOM	4584	HN	LYS	P	282	-9.225	0.992	16.889	0.00	0.00	PROA
ATOM	4585	CA	LYS	P	282	-9.975	-0.362	15.402	0.00	0.00	PROA
ATOM	4586	HA	LYS	P	282	-9.682	-1.399	15.333	0.00	0.00	PROA
ATOM	4587	CB	LYS	P	282	-11.335	-0.375	16.111	0.00	0.00	PROA
ATOM	4588	HB1	LYS	P	282	-12.033	-1.016	15.531	0.00	0.00	PROA
ATOM	4589	HB2	LYS	P	282	-11.718	0.668	16.087	0.00	0.00	PROA
ATOM	4590	CG	LYS	P	282	-11.205	-0.688	17.529	0.00	0.00	PROA
ATOM	4591	HG1	LYS	P	282	-12.237	-0.804	17.925	0.00	0.00	PROA
ATOM	4592	HG2	LYS	P	282	-10.953	0.230	18.101	0.00	0.00	PROA
ATOM	4593	CD	LYS	P	282	-10.190	-1.758	17.941	0.00	0.00	PROA
ATOM	4594	HD1	LYS	P	282	-9.235	-1.582	17.402	0.00	0.00	PROA
ATOM	4595	HD2	LYS	P	282	-10.635	-2.730	17.640	0.00	0.00	PROA
ATOM	4596	CE	LYS	P	282	-9.769	-1.865	19.409	0.00	0.00	PROA
ATOM	4597	HE1	LYS	P	282	-10.708	-1.993	19.989	0.00	0.00	PROA
ATOM	4598	HE2	LYS	P	282	-9.408	-0.872	19.752	0.00	0.00	PROA
ATOM	4599	NZ	LYS	P	282	-8.824	-2.960	19.697	0.00	0.00	PROA
ATOM	4600	HZ1	LYS	P	282	-8.304	-2.771	20.578	0.00	0.00	PROA
ATOM	4601	HZ2	LYS	P	282	-8.215	-3.127	18.871	0.00	0.00	PROA
ATOM	4602	HZ3	LYS	P	282	-9.337	-3.817	19.987	0.00	0.00	PROA
ATOM	4603	C	LYS	P	282	-10.367	0.143	14.054	0.00	0.00	PROA
ATOM	4604	O	LYS	P	282	-10.492	-0.630	13.114	0.00	0.00	PROA
ATOM	4605	N	TRP	P	283	-10.620	1.479	13.953	0.00	0.00	PROA
ATOM	4606	HN	TRP	P	283	-10.683	2.083	14.743	0.00	0.00	PROA
ATOM	4607	CA	TRP	P	283	-10.768	2.146	12.666	0.00	0.00	PROA
ATOM	4608	HA	TRP	P	283	-11.590	1.751	12.088	0.00	0.00	PROA
ATOM	4609	CB	TRP	P	283	-10.966	3.742	12.916	0.00	0.00	PROA
ATOM	4610	HB1	TRP	P	283	-10.877	4.278	11.947	0.00	0.00	PROA
ATOM	4611	HB2	TRP	P	283	-10.281	4.079	13.724	0.00	0.00	PROA
ATOM	4612	CG	TRP	P	283	-12.403	3.990	13.326	0.00	0.00	PROA
ATOM	4613	CD1	TRP	P	283	-12.888	4.213	14.616	0.00	0.00	PROA
ATOM	4614	HD1	TRP	P	283	-12.218	4.405	15.441	0.00	0.00	PROA
ATOM	4615	NE1	TRP	P	283	-14.272	4.585	14.527	0.00	0.00	PROA
ATOM	4616	HE1	TRP	P	283	-14.868	4.701	15.292	0.00	0.00	PROA
ATOM	4617	CE2	TRP	P	283	-14.649	4.403	13.209	0.00	0.00	PROA
ATOM	4618	CD2	TRP	P	283	-13.529	4.029	12.500	0.00	0.00	PROA
ATOM	4619	CE3	TRP	P	283	-13.635	3.643	11.151	0.00	0.00	PROA
ATOM	4620	HE3	TRP	P	283	-12.723	3.504	10.590	0.00	0.00	PROA
ATOM	4621	CZ3	TRP	P	283	-14.864	3.800	10.526	0.00	0.00	PROA
ATOM	4622	HZ3	TRP	P	283	-14.857	3.609	9.464	0.00	0.00	PROA
ATOM	4623	CZ2	TRP	P	283	-15.858	4.586	12.611	0.00	0.00	PROA
ATOM	4624	HZ2	TRP	P	283	-16.667	4.957	13.223	0.00	0.00	PROA
ATOM	4625	CH2	TRP	P	283	-15.978	4.306	11.180	0.00	0.00	PROA
ATOM	4626	HH2	TRP	P	283	-16.936	4.498	10.721	0.00	0.00	PROA
ATOM	4627	C	TRP	P	283	-9.567	1.971	11.720	0.00	0.00	PROA
ATOM	4628	O	TRP	P	283	-9.562	1.682	10.531	0.00	0.00	PROA
ATOM	4629	N	ILE	P	284	-8.354	2.148	12.221	0.00	0.00	PROA
ATOM	4630	HN	ILE	P	284	-8.400	2.580	13.119	0.00	0.00	PROA
ATOM	4631	CA	ILE	P	284	-7.130	1.892	11.507	0.00	0.00	PROA
ATOM	4632	HA	ILE	P	284	-7.237	2.530	10.643	0.00	0.00	PROA

```
ATOM   4633  CB  ILE P 284      -5.939   2.304  12.309  0.00  0.00      PROA
ATOM   4634  HB  ILE P 284      -6.113   1.964  13.352  0.00  0.00      PROA
ATOM   4635  CG2 ILE P 284      -4.689   1.659  11.748  0.00  0.00      PROA
ATOM   4636 HG21 ILE P 284      -3.869   1.925  12.449  0.00  0.00      PROA
ATOM   4637 HG22 ILE P 284      -4.402   2.032  10.742  0.00  0.00      PROA
ATOM   4638 HG23 ILE P 284      -4.775   0.552  11.720  0.00  0.00      PROA
ATOM   4639  CG1 ILE P 284      -5.857   3.845  12.273  0.00  0.00      PROA
ATOM   4640 HG11 ILE P 284      -6.805   4.361  12.538  0.00  0.00      PROA
ATOM   4641 HG12 ILE P 284      -5.648   4.280  11.272  0.00  0.00      PROA
ATOM   4642  CD  ILE P 284      -4.671   4.384  13.198  0.00  0.00      PROA
ATOM   4643  HD1 ILE P 284      -3.704   4.213  12.679  0.00  0.00      PROA
ATOM   4644  HD2 ILE P 284      -4.641   3.831  14.162  0.00  0.00      PROA
ATOM   4645  HD3 ILE P 284      -4.918   5.457  13.348  0.00  0.00      PROA
ATOM   4646  C   ILE P 284      -7.026   0.448  10.996  0.00  0.00      PROA
ATOM   4647  O   ILE P 284      -6.782   0.174   9.833  0.00  0.00      PROA
ATOM   4648  N   SER P 285      -7.344  -0.549  11.843  0.00  0.00      PROA
ATOM   4649  HN  SER P 285      -7.582  -0.331  12.786  0.00  0.00      PROA
ATOM   4650  CA  SER P 285      -7.181  -1.949  11.577  0.00  0.00      PROA
ATOM   4651  HA  SER P 285      -6.182  -2.185  11.242  0.00  0.00      PROA
ATOM   4652  CB  SER P 285      -7.323  -2.781  12.892  0.00  0.00      PROA
ATOM   4653  HB1 SER P 285      -8.239  -2.518  13.463  0.00  0.00      PROA
ATOM   4654  HB2 SER P 285      -6.458  -2.478  13.520  0.00  0.00      PROA
ATOM   4655  OG  SER P 285      -7.186  -4.188  12.731  0.00  0.00      PROA
ATOM   4656  HG1 SER P 285      -6.248  -4.263  12.540  0.00  0.00      PROA
ATOM   4657  C   SER P 285      -8.096  -2.553  10.466  0.00  0.00      PROA
ATOM   4658  O   SER P 285      -7.631  -3.263   9.562  0.00  0.00      PROA
ATOM   4659  N   ILE P 286      -9.421  -2.166  10.426  0.00  0.00      PROA
ATOM   4660  HN  ILE P 286      -9.754  -1.761  11.273  0.00  0.00      PROA
ATOM   4661  CA  ILE P 286     -10.213  -2.367   9.244  0.00  0.00      PROA
ATOM   4662  HA  ILE P 286     -10.038  -3.407   9.010  0.00  0.00      PROA
ATOM   4663  CB  ILE P 286     -11.734  -2.244   9.360  0.00  0.00      PROA
ATOM   4664  HB  ILE P 286     -12.295  -2.282   8.402  0.00  0.00      PROA
ATOM   4665  CG2 ILE P 286     -12.259  -3.446  10.150  0.00  0.00      PROA
ATOM   4666 HG21 ILE P 286     -11.695  -3.501  11.106  0.00  0.00      PROA
ATOM   4667 HG22 ILE P 286     -12.006  -4.395   9.631  0.00  0.00      PROA
ATOM   4668 HG23 ILE P 286     -13.351  -3.517  10.343  0.00  0.00      PROA
ATOM   4669  CG1 ILE P 286     -12.224  -0.845   9.928  0.00  0.00      PROA
ATOM   4670 HG11 ILE P 286     -11.597  -0.546  10.795  0.00  0.00      PROA
ATOM   4671 HG12 ILE P 286     -13.155  -1.211  10.412  0.00  0.00      PROA
ATOM   4672  CD  ILE P 286     -12.473   0.212   8.830  0.00  0.00      PROA
ATOM   4673  HD1 ILE P 286     -11.475   0.624   8.565  0.00  0.00      PROA
ATOM   4674  HD2 ILE P 286     -13.045   1.029   9.321  0.00  0.00      PROA
ATOM   4675  HD3 ILE P 286     -12.838  -0.196   7.864  0.00  0.00      PROA
ATOM   4676  C   ILE P 286      -9.674  -1.598   8.052  0.00  0.00      PROA
ATOM   4677  O   ILE P 286      -9.535  -2.214   6.996  0.00  0.00      PROA
ATOM   4678  N   THR P 287      -9.265  -0.290   8.171  0.00  0.00      PROA
ATOM   4679  HN  THR P 287      -9.390   0.171   9.046  0.00  0.00      PROA
ATOM   4680  CA  THR P 287      -8.756   0.399   6.951  0.00  0.00      PROA
ATOM   4681  HA  THR P 287      -9.563   0.247   6.249  0.00  0.00      PROA
ATOM   4682  CB  THR P 287      -8.608   1.945   7.037  0.00  0.00      PROA
ATOM   4683  HB  THR P 287      -7.969   2.311   6.205  0.00  0.00      PROA
ATOM   4684  OG1 THR P 287      -7.969   2.399   8.269  0.00  0.00      PROA
ATOM   4685  HG1 THR P 287      -8.174   1.854   9.032  0.00  0.00      PROA
ATOM   4686  CG2 THR P 287     -10.054   2.571   7.078  0.00  0.00      PROA
ATOM   4687 HG21 THR P 287     -10.621   2.326   8.001  0.00  0.00      PROA
ATOM   4688 HG22 THR P 287     -10.518   2.158   6.157  0.00  0.00      PROA
ATOM   4689 HG23 THR P 287     -10.005   3.680   7.030  0.00  0.00      PROA
ATOM   4690  C   THR P 287      -7.444  -0.213   6.337  0.00  0.00      PROA
ATOM   4691  O   THR P 287      -7.365  -0.298   5.141  0.00  0.00      PROA
ATOM   4692  N   GLU P 288      -6.579  -0.758   7.220  0.00  0.00      PROA
ATOM   4693  HN  GLU P 288      -6.766  -0.535   8.174  0.00  0.00      PROA
```

ATOM	4694	CA	GLU	P	288	-5.503	-1.720	6.894	0.00	0.00	PROA
ATOM	4695	HA	GLU	P	288	-4.870	-1.201	6.190	0.00	0.00	PROA
ATOM	4696	CB	GLU	P	288	-4.717	-1.995	8.236	0.00	0.00	PROA
ATOM	4697	HB1	GLU	P	288	-5.310	-2.546	8.997	0.00	0.00	PROA
ATOM	4698	HB2	GLU	P	288	-4.505	-1.000	8.684	0.00	0.00	PROA
ATOM	4699	CG	GLU	P	288	-3.394	-2.701	8.101	0.00	0.00	PROA
ATOM	4700	HG1	GLU	P	288	-2.622	-1.930	7.893	0.00	0.00	PROA
ATOM	4701	HG2	GLU	P	288	-3.304	-3.528	7.364	0.00	0.00	PROA
ATOM	4702	CD	GLU	P	288	-3.057	-3.210	9.492	0.00	0.00	PROA
ATOM	4703	OE1	GLU	P	288	-2.383	-2.504	10.329	0.00	0.00	PROA
ATOM	4704	OE2	GLU	P	288	-3.424	-4.413	9.695	0.00	0.00	PROA
ATOM	4705	C	GLU	P	288	-5.904	-2.957	6.211	0.00	0.00	PROA
ATOM	4706	O	GLU	P	288	-5.356	-3.375	5.209	0.00	0.00	PROA
ATOM	4707	N	ALA	P	289	-6.981	-3.615	6.681	0.00	0.00	PROA
ATOM	4708	HN	ALA	P	289	-7.441	-3.293	7.504	0.00	0.00	PROA
ATOM	4709	CA	ALA	P	289	-7.615	-4.708	5.905	0.00	0.00	PROA
ATOM	4710	HA	ALA	P	289	-6.878	-5.460	5.663	0.00	0.00	PROA
ATOM	4711	CB	ALA	P	289	-8.735	-5.315	6.737	0.00	0.00	PROA
ATOM	4712	HB1	ALA	P	289	-8.611	-5.412	7.837	0.00	0.00	PROA
ATOM	4713	HB2	ALA	P	289	-9.030	-6.298	6.310	0.00	0.00	PROA
ATOM	4714	HB3	ALA	P	289	-9.674	-4.727	6.656	0.00	0.00	PROA
ATOM	4715	C	ALA	P	289	-8.087	-4.344	4.545	0.00	0.00	PROA
ATOM	4716	O	ALA	P	289	-8.062	-5.046	3.550	0.00	0.00	PROA
ATOM	4717	N	LEU	P	290	-8.688	-3.159	4.416	0.00	0.00	PROA
ATOM	4718	HN	LEU	P	290	-8.872	-2.704	5.285	0.00	0.00	PROA
ATOM	4719	CA	LEU	P	290	-9.103	-2.640	3.144	0.00	0.00	PROA
ATOM	4720	HA	LEU	P	290	-9.773	-3.365	2.707	0.00	0.00	PROA
ATOM	4721	CB	LEU	P	290	-10.097	-1.428	3.439	0.00	0.00	PROA
ATOM	4722	HB1	LEU	P	290	-9.572	-0.851	4.230	0.00	0.00	PROA
ATOM	4723	HB2	LEU	P	290	-10.935	-1.948	3.950	0.00	0.00	PROA
ATOM	4724	CG	LEU	P	290	-10.623	-0.722	2.216	0.00	0.00	PROA
ATOM	4725	HG	LEU	P	290	-9.754	-0.460	1.575	0.00	0.00	PROA
ATOM	4726	CD1	LEU	P	290	-11.479	-1.561	1.346	0.00	0.00	PROA
ATOM	4727	HD11	LEU	P	290	-11.285	-2.655	1.345	0.00	0.00	PROA
ATOM	4728	HD12	LEU	P	290	-11.591	-1.197	0.302	0.00	0.00	PROA
ATOM	4729	HD13	LEU	P	290	-12.514	-1.596	1.748	0.00	0.00	PROA
ATOM	4730	CD2	LEU	P	290	-11.329	0.571	2.410	0.00	0.00	PROA
ATOM	4731	HD21	LEU	P	290	-11.518	1.059	1.429	0.00	0.00	PROA
ATOM	4732	HD22	LEU	P	290	-10.744	1.229	3.087	0.00	0.00	PROA
ATOM	4733	HD23	LEU	P	290	-12.369	0.485	2.791	0.00	0.00	PROA
ATOM	4734	C	LEU	P	290	-7.943	-2.335	2.141	0.00	0.00	PROA
ATOM	4735	O	LEU	P	290	-8.051	-2.630	0.940	0.00	0.00	PROA
ATOM	4736	N	ALA	P	291	-6.871	-1.913	2.698	0.00	0.00	PROA
ATOM	4737	HN	ALA	P	291	-6.856	-1.563	3.632	0.00	0.00	PROA
ATOM	4738	CA	ALA	P	291	-5.570	-1.659	1.990	0.00	0.00	PROA
ATOM	4739	HA	ALA	P	291	-5.917	-1.101	1.133	0.00	0.00	PROA
ATOM	4740	CB	ALA	P	291	-4.626	-0.939	2.949	0.00	0.00	PROA
ATOM	4741	HB1	ALA	P	291	-5.256	-0.069	3.234	0.00	0.00	PROA
ATOM	4742	HB2	ALA	P	291	-3.776	-0.495	2.388	0.00	0.00	PROA
ATOM	4743	HB3	ALA	P	291	-4.209	-1.640	3.703	0.00	0.00	PROA
ATOM	4744	C	ALA	P	291	-4.866	-2.970	1.540	0.00	0.00	PROA
ATOM	4745	O	ALA	P	291	-4.410	-3.074	0.400	0.00	0.00	PROA
ATOM	4746	N	PHE	P	292	-4.942	-4.094	2.393	0.00	0.00	PROA
ATOM	4747	HN	PHE	P	292	-5.195	-3.942	3.345	0.00	0.00	PROA
ATOM	4748	CA	PHE	P	292	-4.719	-5.437	1.988	0.00	0.00	PROA
ATOM	4749	HA	PHE	P	292	-3.699	-5.668	1.718	0.00	0.00	PROA
ATOM	4750	CB	PHE	P	292	-4.879	-6.370	3.201	0.00	0.00	PROA
ATOM	4751	HB1	PHE	P	292	-4.854	-7.400	2.786	0.00	0.00	PROA
ATOM	4752	HB2	PHE	P	292	-5.825	-6.241	3.770	0.00	0.00	PROA
ATOM	4753	CG	PHE	P	292	-3.841	-6.346	4.299	0.00	0.00	PROA
ATOM	4754	CD1	PHE	P	292	-4.072	-7.111	5.474	0.00	0.00	PROA

ATOM	4755	HD1	PHE	P	292	-4.892	-7.814	5.459	0.00	0.00	PROA
ATOM	4756	CE1	PHE	P	292	-3.346	-6.884	6.604	0.00	0.00	PROA
ATOM	4757	HE1	PHE	P	292	-3.422	-7.394	7.554	0.00	0.00	PROA
ATOM	4758	CZ	PHE	P	292	-2.250	-5.953	6.602	0.00	0.00	PROA
ATOM	4759	HZ	PHE	P	292	-1.648	-5.957	7.499	0.00	0.00	PROA
ATOM	4760	CD2	PHE	P	292	-2.756	-5.518	4.310	0.00	0.00	PROA
ATOM	4761	HD2	PHE	P	292	-2.477	-4.972	3.421	0.00	0.00	PROA
ATOM	4762	CE2	PHE	P	292	-1.895	-5.377	5.384	0.00	0.00	PROA
ATOM	4763	HE2	PHE	P	292	-1.018	-4.755	5.286	0.00	0.00	PROA
ATOM	4764	C	PHE	P	292	-5.544	-5.988	0.904	0.00	0.00	PROA
ATOM	4765	O	PHE	P	292	-4.982	-6.522	-0.035	0.00	0.00	PROA
ATOM	4766	N	PHE	P	293	-6.844	-5.730	0.923	0.00	0.00	PROA
ATOM	4767	HN	PHE	P	293	-7.237	-5.215	1.681	0.00	0.00	PROA
ATOM	4768	CA	PHE	P	293	-7.834	-6.187	-0.089	0.00	0.00	PROA
ATOM	4769	HA	PHE	P	293	-7.719	-7.245	-0.269	0.00	0.00	PROA
ATOM	4770	CB	PHE	P	293	-9.206	-6.071	0.587	0.00	0.00	PROA
ATOM	4771	HB1	PHE	P	293	-9.375	-5.002	0.840	0.00	0.00	PROA
ATOM	4772	HB2	PHE	P	293	-9.276	-6.660	1.526	0.00	0.00	PROA
ATOM	4773	CG	PHE	P	293	-10.314	-6.450	-0.409	0.00	0.00	PROA
ATOM	4774	CD1	PHE	P	293	-10.289	-7.630	-1.173	0.00	0.00	PROA
ATOM	4775	HD1	PHE	P	293	-9.403	-8.247	-1.144	0.00	0.00	PROA
ATOM	4776	CE1	PHE	P	293	-11.228	-7.788	-2.143	0.00	0.00	PROA
ATOM	4777	HE1	PHE	P	293	-11.109	-8.697	-2.714	0.00	0.00	PROA
ATOM	4778	CZ	PHE	P	293	-12.308	-6.906	-2.299	0.00	0.00	PROA
ATOM	4779	HZ	PHE	P	293	-12.958	-7.001	-3.157	0.00	0.00	PROA
ATOM	4780	CD2	PHE	P	293	-11.483	-5.624	-0.375	0.00	0.00	PROA
ATOM	4781	HD2	PHE	P	293	-11.697	-4.809	0.301	0.00	0.00	PROA
ATOM	4782	CE2	PHE	P	293	-12.468	-5.896	-1.356	0.00	0.00	PROA
ATOM	4783	HE2	PHE	P	293	-13.347	-5.279	-1.469	0.00	0.00	PROA
ATOM	4784	C	PHE	P	293	-7.581	-5.423	-1.370	0.00	0.00	PROA
ATOM	4785	O	PHE	P	293	-7.851	-5.849	-2.484	0.00	0.00	PROA
ATOM	4786	N	HSD	P	294	-6.958	-4.188	-1.325	0.00	0.00	PROA
ATOM	4787	HN	HSD	P	294	-6.792	-3.785	-0.428	0.00	0.00	PROA
ATOM	4788	CA	HSD	P	294	-6.665	-3.354	-2.472	0.00	0.00	PROA
ATOM	4789	HA	HSD	P	294	-7.586	-3.376	-3.036	0.00	0.00	PROA
ATOM	4790	CB	HSD	P	294	-6.240	-1.886	-2.063	0.00	0.00	PROA
ATOM	4791	HB1	HSD	P	294	-5.148	-1.890	-1.861	0.00	0.00	PROA
ATOM	4792	HB2	HSD	P	294	-6.711	-1.575	-1.106	0.00	0.00	PROA
ATOM	4793	ND1	HSD	P	294	-5.800	0.333	-3.264	0.00	0.00	PROA
ATOM	4794	HD1	HSD	P	294	-5.014	0.517	-2.673	0.00	0.00	PROA
ATOM	4795	CG	HSD	P	294	-6.414	-0.905	-3.217	0.00	0.00	PROA
ATOM	4796	CE1	HSD	P	294	-6.081	0.903	-4.397	0.00	0.00	PROA
ATOM	4797	HE1	HSD	P	294	-5.758	1.909	-4.663	0.00	0.00	PROA
ATOM	4798	NE2	HSD	P	294	-6.811	0.088	-5.180	0.00	0.00	PROA
ATOM	4799	CD2	HSD	P	294	-7.014	-1.019	-4.424	0.00	0.00	PROA
ATOM	4800	HD2	HSD	P	294	-7.512	-1.827	-4.945	0.00	0.00	PROA
ATOM	4801	C	HSD	P	294	-5.598	-3.961	-3.348	0.00	0.00	PROA
ATOM	4802	O	HSD	P	294	-5.338	-3.606	-4.515	0.00	0.00	PROA
ATOM	4803	N	CYS	P	295	-4.883	-5.006	-2.795	0.00	0.00	PROA
ATOM	4804	HN	CYS	P	295	-4.966	-5.321	-1.852	0.00	0.00	PROA
ATOM	4805	CA	CYS	P	295	-4.058	-5.867	-3.634	0.00	0.00	PROA
ATOM	4806	HA	CYS	P	295	-3.361	-5.264	-4.197	0.00	0.00	PROA
ATOM	4807	CB	CYS	P	295	-3.361	-6.964	-2.716	0.00	0.00	PROA
ATOM	4808	HB1	CYS	P	295	-4.173	-7.557	-2.245	0.00	0.00	PROA
ATOM	4809	HB2	CYS	P	295	-2.830	-6.397	-1.922	0.00	0.00	PROA
ATOM	4810	SG	CYS	P	295	-2.322	-8.178	-3.638	0.00	0.00	PROA
ATOM	4811	HG1	CYS	P	295	-1.506	-7.190	-3.977	0.00	0.00	PROA
ATOM	4812	C	CYS	P	295	-4.909	-6.627	-4.673	0.00	0.00	PROA
ATOM	4813	O	CYS	P	295	-4.728	-6.491	-5.920	0.00	0.00	PROA
ATOM	4814	N	CYS	P	296	-5.814	-7.401	-4.175	0.00	0.00	PROA
ATOM	4815	HN	CYS	P	296	-6.113	-7.163	-3.254	0.00	0.00	PROA

ATOM	4816	CA	CYS	P	296	-6.611	-8.436	-4.838	0.00	0.00	PROA
ATOM	4817	HA	CYS	P	296	-6.013	-9.067	-5.479	0.00	0.00	PROA
ATOM	4818	CB	CYS	P	296	-7.215	-9.464	-3.790	0.00	0.00	PROA
ATOM	4819	HB1	CYS	P	296	-7.605	-10.345	-4.342	0.00	0.00	PROA
ATOM	4820	HB2	CYS	P	296	-8.025	-8.964	-3.217	0.00	0.00	PROA
ATOM	4821	SG	CYS	P	296	-5.847	-10.051	-2.683	0.00	0.00	PROA
ATOM	4822	HG1	CYS	P	296	-5.038	-10.654	-3.542	0.00	0.00	PROA
ATOM	4823	C	CYS	P	296	-7.688	-7.801	-5.661	0.00	0.00	PROA
ATOM	4824	O	CYS	P	296	-8.067	-8.276	-6.752	0.00	0.00	PROA
ATOM	4825	N	LEU	P	297	-8.281	-6.705	-5.169	0.00	0.00	PROA
ATOM	4826	HN	LEU	P	297	-8.107	-6.342	-4.257	0.00	0.00	PROA
ATOM	4827	CA	LEU	P	297	-9.334	-5.927	-5.785	0.00	0.00	PROA
ATOM	4828	HA	LEU	P	297	-10.228	-6.526	-5.876	0.00	0.00	PROA
ATOM	4829	CB	LEU	P	297	-9.734	-4.725	-4.908	0.00	0.00	PROA
ATOM	4830	HB1	LEU	P	297	-8.786	-4.156	-4.797	0.00	0.00	PROA
ATOM	4831	HB2	LEU	P	297	-10.040	-5.179	-3.941	0.00	0.00	PROA
ATOM	4832	CG	LEU	P	297	-10.833	-3.864	-5.343	0.00	0.00	PROA
ATOM	4833	HG	LEU	P	297	-10.701	-3.510	-6.388	0.00	0.00	PROA
ATOM	4834	CD1	LEU	P	297	-12.169	-4.671	-5.265	0.00	0.00	PROA
ATOM	4835	HD11	LEU	P	297	-12.362	-5.292	-6.165	0.00	0.00	PROA
ATOM	4836	HD12	LEU	P	297	-12.988	-3.937	-5.422	0.00	0.00	PROA
ATOM	4837	HD13	LEU	P	297	-12.281	-5.279	-4.342	0.00	0.00	PROA
ATOM	4838	CD2	LEU	P	297	-10.865	-2.494	-4.587	0.00	0.00	PROA
ATOM	4839	HD21	LEU	P	297	-11.131	-2.677	-3.524	0.00	0.00	PROA
ATOM	4840	HD22	LEU	P	297	-11.583	-1.788	-5.056	0.00	0.00	PROA
ATOM	4841	HD23	LEU	P	297	-9.878	-1.989	-4.660	0.00	0.00	PROA
ATOM	4842	C	LEU	P	297	-8.817	-5.427	-7.159	0.00	0.00	PROA
ATOM	4843	O	LEU	P	297	-9.520	-5.394	-8.190	0.00	0.00	PROA
ATOM	4844	N	ASN	P	298	-7.571	-4.940	-7.199	0.00	0.00	PROA
ATOM	4845	HN	ASN	P	298	-6.990	-5.096	-6.404	0.00	0.00	PROA
ATOM	4846	CA	ASN	P	298	-6.951	-4.342	-8.363	0.00	0.00	PROA
ATOM	4847	HA	ASN	P	298	-7.377	-3.351	-8.423	0.00	0.00	PROA
ATOM	4848	CB	ASN	P	298	-5.489	-3.939	-8.024	0.00	0.00	PROA
ATOM	4849	HB1	ASN	P	298	-4.699	-4.712	-7.906	0.00	0.00	PROA
ATOM	4850	HB2	ASN	P	298	-5.623	-3.491	-7.016	0.00	0.00	PROA
ATOM	4851	CG	ASN	P	298	-4.873	-2.952	-8.969	0.00	0.00	PROA
ATOM	4852	OD1	ASN	P	298	-4.763	-3.227	-10.176	0.00	0.00	PROA
ATOM	4853	ND2	ASN	P	298	-4.282	-1.795	-8.529	0.00	0.00	PROA
ATOM	4854	HD21	ASN	P	298	-4.085	-1.592	-7.570	0.00	0.00	PROA
ATOM	4855	HD22	ASN	P	298	-4.052	-1.074	-9.183	0.00	0.00	PROA
ATOM	4856	C	ASN	P	298	-7.148	-5.054	-9.734	0.00	0.00	PROA
ATOM	4857	O	ASN	P	298	-7.574	-4.459	-10.678	0.00	0.00	PROA
ATOM	4858	N	PRO	P	299	-6.861	-6.292	-10.033	0.00	0.00	PROA
ATOM	4859	CD	PRO	P	299	-6.013	-7.192	-9.247	0.00	0.00	PROA
ATOM	4860	HD1	PRO	P	299	-6.250	-7.141	-8.163	0.00	0.00	PROA
ATOM	4861	HD2	PRO	P	299	-4.940	-6.936	-9.376	0.00	0.00	PROA
ATOM	4862	CA	PRO	P	299	-7.259	-6.916	-11.370	0.00	0.00	PROA
ATOM	4863	HA	PRO	P	299	-6.929	-6.188	-12.095	0.00	0.00	PROA
ATOM	4864	CB	PRO	P	299	-6.409	-8.157	-11.415	0.00	0.00	PROA
ATOM	4865	HB1	PRO	P	299	-5.421	-7.822	-11.797	0.00	0.00	PROA
ATOM	4866	HB2	PRO	P	299	-6.735	-8.853	-12.217	0.00	0.00	PROA
ATOM	4867	CG	PRO	P	299	-6.345	-8.553	-9.926	0.00	0.00	PROA
ATOM	4868	HG1	PRO	P	299	-7.381	-8.858	-9.665	0.00	0.00	PROA
ATOM	4869	HG2	PRO	P	299	-5.664	-9.305	-9.472	0.00	0.00	PROA
ATOM	4870	C	PRO	P	299	-8.719	-7.138	-11.646	0.00	0.00	PROA
ATOM	4871	O	PRO	P	299	-9.123	-7.259	-12.737	0.00	0.00	PROA
ATOM	4872	N	ILE	P	300	-9.572	-7.187	-10.603	0.00	0.00	PROA
ATOM	4873	HN	ILE	P	300	-9.215	-7.016	-9.688	0.00	0.00	PROA
ATOM	4874	CA	ILE	P	300	-10.989	-7.343	-10.725	0.00	0.00	PROA
ATOM	4875	HA	ILE	P	300	-11.177	-8.046	-11.523	0.00	0.00	PROA
ATOM	4876	CB	ILE	P	300	-11.704	-7.890	-9.498	0.00	0.00	PROA

ATOM	4877	HB	ILE	P	300	-11.952	-7.079	-8.779	0.00	0.00	PROA
ATOM	4878	CG2	ILE	P	300	-13.041	-8.480	-9.890	0.00	0.00	PROA
ATOM	4879	HG21	ILE	P	300	-13.425	-8.918	-8.944	0.00	0.00	PROA
ATOM	4880	HG22	ILE	P	300	-12.945	-9.317	-10.614	0.00	0.00	PROA
ATOM	4881	HG23	ILE	P	300	-13.908	-7.884	-10.249	0.00	0.00	PROA
ATOM	4882	CG1	ILE	P	300	-10.705	-8.784	-8.781	0.00	0.00	PROA
ATOM	4883	HG11	ILE	P	300	-9.891	-8.104	-8.449	0.00	0.00	PROA
ATOM	4884	HG12	ILE	P	300	-10.301	-9.592	-9.428	0.00	0.00	PROA
ATOM	4885	CD	ILE	P	300	-11.285	-9.305	-7.466	0.00	0.00	PROA
ATOM	4886	HD1	ILE	P	300	-12.226	-9.894	-7.520	0.00	0.00	PROA
ATOM	4887	HD2	ILE	P	300	-11.492	-8.496	-6.734	0.00	0.00	PROA
ATOM	4888	HD3	ILE	P	300	-10.566	-9.963	-6.933	0.00	0.00	PROA
ATOM	4889	C	ILE	P	300	-11.715	-6.128	-11.262	0.00	0.00	PROA
ATOM	4890	O	ILE	P	300	-12.502	-6.202	-12.178	0.00	0.00	PROA
ATOM	4891	N	LEU	P	301	-11.231	-4.923	-10.806	0.00	0.00	PROA
ATOM	4892	HN	LEU	P	301	-10.616	-4.857	-10.024	0.00	0.00	PROA
ATOM	4893	CA	LEU	P	301	-11.724	-3.655	-11.451	0.00	0.00	PROA
ATOM	4894	HA	LEU	P	301	-12.801	-3.719	-11.502	0.00	0.00	PROA
ATOM	4895	CB	LEU	P	301	-11.319	-2.335	-10.774	0.00	0.00	PROA
ATOM	4896	HB1	LEU	P	301	-11.877	-1.476	-11.206	0.00	0.00	PROA
ATOM	4897	HB2	LEU	P	301	-10.239	-2.090	-10.864	0.00	0.00	PROA
ATOM	4898	CG	LEU	P	301	-11.424	-2.274	-9.207	0.00	0.00	PROA
ATOM	4899	HG	LEU	P	301	-10.773	-3.082	-8.809	0.00	0.00	PROA
ATOM	4900	CD1	LEU	P	301	-11.080	-0.861	-8.583	0.00	0.00	PROA
ATOM	4901	HD11	LEU	P	301	-11.860	-0.084	-8.731	0.00	0.00	PROA
ATOM	4902	HD12	LEU	P	301	-10.101	-0.576	-9.023	0.00	0.00	PROA
ATOM	4903	HD13	LEU	P	301	-10.926	-0.897	-7.484	0.00	0.00	PROA
ATOM	4904	CD2	LEU	P	301	-12.807	-2.640	-8.679	0.00	0.00	PROA
ATOM	4905	HD21	LEU	P	301	-13.055	-3.663	-9.033	0.00	0.00	PROA
ATOM	4906	HD22	LEU	P	301	-13.483	-1.925	-9.195	0.00	0.00	PROA
ATOM	4907	HD23	LEU	P	301	-12.898	-2.519	-7.578	0.00	0.00	PROA
ATOM	4908	C	LEU	P	301	-11.304	-3.483	-12.926	0.00	0.00	PROA
ATOM	4909	O	LEU	P	301	-12.139	-3.119	-13.787	0.00	0.00	PROA
ATOM	4910	N	TYR	P	302	-10.009	-3.823	-13.273	0.00	0.00	PROA
ATOM	4911	HN	TYR	P	302	-9.414	-4.305	-12.635	0.00	0.00	PROA
ATOM	4912	CA	TYR	P	302	-9.487	-3.716	-14.643	0.00	0.00	PROA
ATOM	4913	HA	TYR	P	302	-9.833	-2.745	-14.964	0.00	0.00	PROA
ATOM	4914	CB	TYR	P	302	-7.941	-3.739	-14.496	0.00	0.00	PROA
ATOM	4915	HB1	TYR	P	302	-7.520	-4.703	-14.136	0.00	0.00	PROA
ATOM	4916	HB2	TYR	P	302	-7.724	-2.947	-13.748	0.00	0.00	PROA
ATOM	4917	CG	TYR	P	302	-7.148	-3.478	-15.797	0.00	0.00	PROA
ATOM	4918	CD1	TYR	P	302	-6.462	-2.303	-15.922	0.00	0.00	PROA
ATOM	4919	HD1	TYR	P	302	-6.493	-1.691	-15.032	0.00	0.00	PROA
ATOM	4920	CE1	TYR	P	302	-5.633	-2.034	-16.968	0.00	0.00	PROA
ATOM	4921	HE1	TYR	P	302	-5.061	-1.118	-16.946	0.00	0.00	PROA
ATOM	4922	CZ	TYR	P	302	-5.511	-2.914	-17.994	0.00	0.00	PROA
ATOM	4923	OH	TYR	P	302	-4.628	-2.542	-19.056	0.00	0.00	PROA
ATOM	4924	HH	TYR	P	302	-4.666	-3.057	-19.865	0.00	0.00	PROA
ATOM	4925	CD2	TYR	P	302	-6.928	-4.480	-16.774	0.00	0.00	PROA
ATOM	4926	HD2	TYR	P	302	-7.414	-5.444	-16.779	0.00	0.00	PROA
ATOM	4927	CE2	TYR	P	302	-6.190	-4.180	-17.931	0.00	0.00	PROA
ATOM	4928	HE2	TYR	P	302	-6.031	-4.917	-18.704	0.00	0.00	PROA
ATOM	4929	C	TYR	P	302	-10.168	-4.748	-15.633	0.00	0.00	PROA
ATOM	4930	O	TYR	P	302	-10.355	-4.515	-16.885	0.00	0.00	PROA
ATOM	4931	N	ALA	P	303	-10.605	-5.922	-15.029	0.00	0.00	PROA
ATOM	4932	HN	ALA	P	303	-10.395	-6.233	-14.105	0.00	0.00	PROA
ATOM	4933	CA	ALA	P	303	-11.484	-6.971	-15.743	0.00	0.00	PROA
ATOM	4934	HA	ALA	P	303	-10.861	-7.418	-16.504	0.00	0.00	PROA
ATOM	4935	CB	ALA	P	303	-11.704	-8.174	-14.778	0.00	0.00	PROA
ATOM	4936	HB1	ALA	P	303	-10.976	-8.412	-13.973	0.00	0.00	PROA
ATOM	4937	HB2	ALA	P	303	-11.896	-9.079	-15.394	0.00	0.00	PROA

ATOM	4938	HB3	ALA	P	303	-12.663	-8.038	-14.233	0.00	0.00	PROA
ATOM	4939	C	ALA	P	303	-12.677	-6.389	-16.462	0.00	0.00	PROA
ATOM	4940	O	ALA	P	303	-13.539	-5.667	-15.913	0.00	0.00	PROA
ATOM	4941	N	PHE	P	304	-12.730	-6.624	-17.789	0.00	0.00	PROA
ATOM	4942	HN	PHE	P	304	-11.945	-7.043	-18.240	0.00	0.00	PROA
ATOM	4943	CA	PHE	P	304	-13.934	-6.256	-18.528	0.00	0.00	PROA
ATOM	4944	HA	PHE	P	304	-13.707	-6.560	-19.539	0.00	0.00	PROA
ATOM	4945	CB	PHE	P	304	-15.204	-6.969	-18.087	0.00	0.00	PROA
ATOM	4946	HB1	PHE	P	304	-15.992	-6.951	-18.870	0.00	0.00	PROA
ATOM	4947	HB2	PHE	P	304	-15.690	-6.527	-17.191	0.00	0.00	PROA
ATOM	4948	CG	PHE	P	304	-15.016	-8.525	-17.895	0.00	0.00	PROA
ATOM	4949	CD1	PHE	P	304	-14.848	-9.153	-16.675	0.00	0.00	PROA
ATOM	4950	HD1	PHE	P	304	-14.758	-8.525	-15.801	0.00	0.00	PROA
ATOM	4951	CE1	PHE	P	304	-14.805	-10.577	-16.684	0.00	0.00	PROA
ATOM	4952	HE1	PHE	P	304	-14.635	-11.004	-15.707	0.00	0.00	PROA
ATOM	4953	CZ	PHE	P	304	-15.038	-11.361	-17.811	0.00	0.00	PROA
ATOM	4954	HZ	PHE	P	304	-15.042	-12.438	-17.738	0.00	0.00	PROA
ATOM	4955	CD2	PHE	P	304	-15.179	-9.257	-19.053	0.00	0.00	PROA
ATOM	4956	HD2	PHE	P	304	-15.151	-8.880	-20.065	0.00	0.00	PROA
ATOM	4957	CE2	PHE	P	304	-15.213	-10.638	-19.002	0.00	0.00	PROA
ATOM	4958	HE2	PHE	P	304	-15.072	-11.216	-19.903	0.00	0.00	PROA
ATOM	4959	C	PHE	P	304	-14.080	-4.752	-18.727	0.00	0.00	PROA
ATOM	4960	O	PHE	P	304	-15.132	-4.263	-19.060	0.00	0.00	PROA
ATOM	4961	N	LEU	P	305	-12.950	-3.961	-18.604	0.00	0.00	PROA
ATOM	4962	HN	LEU	P	305	-12.131	-4.358	-18.196	0.00	0.00	PROA
ATOM	4963	CA	LEU	P	305	-13.034	-2.533	-18.904	0.00	0.00	PROA
ATOM	4964	HA	LEU	P	305	-13.997	-2.257	-18.502	0.00	0.00	PROA
ATOM	4965	CB	LEU	P	305	-11.956	-1.867	-18.023	0.00	0.00	PROA
ATOM	4966	HB1	LEU	P	305	-10.922	-2.132	-18.333	0.00	0.00	PROA
ATOM	4967	HB2	LEU	P	305	-11.974	-2.313	-17.006	0.00	0.00	PROA
ATOM	4968	CG	LEU	P	305	-12.029	-0.327	-17.815	0.00	0.00	PROA
ATOM	4969	HG	LEU	P	305	-11.824	0.215	-18.762	0.00	0.00	PROA
ATOM	4970	CD1	LEU	P	305	-13.403	0.139	-17.270	0.00	0.00	PROA
ATOM	4971	HD11	LEU	P	305	-13.559	-0.096	-16.196	0.00	0.00	PROA
ATOM	4972	HD12	LEU	P	305	-14.205	-0.363	-17.852	0.00	0.00	PROA
ATOM	4973	HD13	LEU	P	305	-13.493	1.236	-17.418	0.00	0.00	PROA
ATOM	4974	CD2	LEU	P	305	-10.887	0.056	-16.822	0.00	0.00	PROA
ATOM	4975	HD21	LEU	P	305	-11.068	-0.645	-15.979	0.00	0.00	PROA
ATOM	4976	HD22	LEU	P	305	-10.974	1.070	-16.376	0.00	0.00	PROA
ATOM	4977	HD23	LEU	P	305	-9.856	-0.057	-17.219	0.00	0.00	PROA
ATOM	4978	C	LEU	P	305	-12.887	-2.222	-20.362	0.00	0.00	PROA
ATOM	4979	O	LEU	P	305	-11.761	-2.352	-20.926	0.00	0.00	PROA
ATOM	4980	N	GLY	P	306	-14.029	-1.804	-20.979	0.00	0.00	PROA
ATOM	4981	HN	GLY	P	306	-14.854	-1.759	-20.421	0.00	0.00	PROA
ATOM	4982	CA	GLY	P	306	-14.190	-1.585	-22.394	0.00	0.00	PROA
ATOM	4983	HA1	GLY	P	306	-13.281	-1.081	-22.685	0.00	0.00	PROA
ATOM	4984	HA2	GLY	P	306	-15.054	-0.965	-22.584	0.00	0.00	PROA
ATOM	4985	C	GLY	P	306	-14.295	-2.853	-23.133	0.00	0.00	PROA
ATOM	4986	O	GLY	P	306	-14.124	-3.918	-22.598	0.00	0.00	PROA
ATOM	4987	N	ALA	P	307	-14.666	-2.698	-24.408	0.00	0.00	PROA
ATOM	4988	HN	ALA	P	307	-15.014	-1.803	-24.677	0.00	0.00	PROA
ATOM	4989	CA	ALA	P	307	-14.701	-3.702	-25.377	0.00	0.00	PROA
ATOM	4990	HA	ALA	P	307	-13.887	-4.398	-25.242	0.00	0.00	PROA
ATOM	4991	CB	ALA	P	307	-16.082	-4.257	-25.477	0.00	0.00	PROA
ATOM	4992	HB1	ALA	P	307	-16.169	-4.846	-26.415	0.00	0.00	PROA
ATOM	4993	HB2	ALA	P	307	-16.939	-3.586	-25.253	0.00	0.00	PROA
ATOM	4994	HB3	ALA	P	307	-16.175	-5.000	-24.656	0.00	0.00	PROA
ATOM	4995	C	ALA	P	307	-14.356	-2.947	-26.672	0.00	0.00	PROA
ATOM	4996	O	ALA	P	307	-14.832	-1.845	-26.934	0.00	0.00	PROA
ATOM	4997	N	LYS	P	308	-13.428	-3.493	-27.522	0.00	0.00	PROA
ATOM	4998	HN	LYS	P	308	-13.105	-4.428	-27.392	0.00	0.00	PROA

ATOM	4999	CA	LYS	P	308	-13.023	-2.796	-28.777	0.00	0.00	PROA
ATOM	5000	HA	LYS	P	308	-12.647	-1.841	-28.439	0.00	0.00	PROA
ATOM	5001	CB	LYS	P	308	-11.829	-3.657	-29.393	0.00	0.00	PROA
ATOM	5002	HB1	LYS	P	308	-11.560	-3.265	-30.398	0.00	0.00	PROA
ATOM	5003	HB2	LYS	P	308	-12.380	-4.610	-29.540	0.00	0.00	PROA
ATOM	5004	CG	LYS	P	308	-10.581	-3.795	-28.489	0.00	0.00	PROA
ATOM	5005	HG1	LYS	P	308	-9.915	-4.514	-29.012	0.00	0.00	PROA
ATOM	5006	HG2	LYS	P	308	-10.859	-4.311	-27.545	0.00	0.00	PROA
ATOM	5007	CD	LYS	P	308	-9.810	-2.471	-28.193	0.00	0.00	PROA
ATOM	5008	HD1	LYS	P	308	-10.335	-1.759	-27.521	0.00	0.00	PROA
ATOM	5009	HD2	LYS	P	308	-9.819	-2.018	-29.208	0.00	0.00	PROA
ATOM	5010	CE	LYS	P	308	-8.345	-2.583	-27.710	0.00	0.00	PROA
ATOM	5011	HE1	LYS	P	308	-7.836	-3.188	-28.491	0.00	0.00	PROA
ATOM	5012	HE2	LYS	P	308	-8.288	-3.178	-26.773	0.00	0.00	PROA
ATOM	5013	NZ	LYS	P	308	-7.662	-1.276	-27.466	0.00	0.00	PROA
ATOM	5014	HZ1	LYS	P	308	-6.680	-1.601	-27.573	0.00	0.00	PROA
ATOM	5015	HZ2	LYS	P	308	-7.980	-0.845	-26.575	0.00	0.00	PROA
ATOM	5016	HZ3	LYS	P	308	-7.864	-0.687	-28.300	0.00	0.00	PROA
ATOM	5017	C	LYS	P	308	-14.109	-2.504	-29.836	0.00	0.00	PROA
ATOM	5018	O	LYS	P	308	-14.920	-3.356	-30.105	0.00	0.00	PROA
ATOM	5019	N	PHE	P	309	-13.926	-1.402	-30.510	0.00	0.00	PROA
ATOM	5020	HN	PHE	P	309	-13.130	-0.862	-30.248	0.00	0.00	PROA
ATOM	5021	CA	PHE	P	309	-14.984	-0.888	-31.396	0.00	0.00	PROA
ATOM	5022	HA	PHE	P	309	-15.970	-1.036	-30.980	0.00	0.00	PROA
ATOM	5023	CB	PHE	P	309	-14.836	0.665	-31.538	0.00	0.00	PROA
ATOM	5024	HB1	PHE	P	309	-13.786	0.798	-31.874	0.00	0.00	PROA
ATOM	5025	HB2	PHE	P	309	-14.932	0.991	-30.480	0.00	0.00	PROA
ATOM	5026	CG	PHE	P	309	-15.873	1.389	-32.289	0.00	0.00	PROA
ATOM	5027	CD1	PHE	P	309	-15.501	2.425	-33.163	0.00	0.00	PROA
ATOM	5028	HD1	PHE	P	309	-14.478	2.749	-33.292	0.00	0.00	PROA
ATOM	5029	CE1	PHE	P	309	-16.404	3.285	-33.877	0.00	0.00	PROA
ATOM	5030	HE1	PHE	P	309	-16.011	4.084	-34.490	0.00	0.00	PROA
ATOM	5031	CZ	PHE	P	309	-17.785	3.034	-33.594	0.00	0.00	PROA
ATOM	5032	HZ	PHE	P	309	-18.589	3.586	-34.057	0.00	0.00	PROA
ATOM	5033	CD2	PHE	P	309	-17.272	1.157	-32.152	0.00	0.00	PROA
ATOM	5034	HD2	PHE	P	309	-17.541	0.365	-31.469	0.00	0.00	PROA
ATOM	5035	CE2	PHE	P	309	-18.187	2.036	-32.738	0.00	0.00	PROA
ATOM	5036	HE2	PHE	P	309	-19.256	1.887	-32.709	0.00	0.00	PROA
ATOM	5037	C	PHE	P	309	-15.012	-1.634	-32.700	0.00	0.00	PROA
ATOM	5038	O	PHE	P	309	-13.973	-2.075	-33.189	0.00	0.00	PROA
ATOM	5039	N	LYS	P	310	-16.209	-1.791	-33.288	0.00	0.00	PROA
ATOM	5040	HN	LYS	P	310	-17.015	-1.329	-32.927	0.00	0.00	PROA
ATOM	5041	CA	LYS	P	310	-16.440	-2.489	-34.480	0.00	0.00	PROA
ATOM	5042	HA	LYS	P	310	-16.218	-3.488	-34.134	0.00	0.00	PROA
ATOM	5043	CB	LYS	P	310	-17.892	-2.511	-34.894	0.00	0.00	PROA
ATOM	5044	HB1	LYS	P	310	-18.378	-3.284	-34.260	0.00	0.00	PROA
ATOM	5045	HB2	LYS	P	310	-18.075	-2.934	-35.906	0.00	0.00	PROA
ATOM	5046	CG	LYS	P	310	-18.779	-1.229	-34.884	0.00	0.00	PROA
ATOM	5047	HG1	LYS	P	310	-18.718	-0.763	-33.878	0.00	0.00	PROA
ATOM	5048	HG2	LYS	P	310	-19.804	-1.595	-35.107	0.00	0.00	PROA
ATOM	5049	CD	LYS	P	310	-18.541	-0.283	-36.072	0.00	0.00	PROA
ATOM	5050	HD1	LYS	P	310	-18.275	-0.832	-37.000	0.00	0.00	PROA
ATOM	5051	HD2	LYS	P	310	-17.674	0.382	-35.868	0.00	0.00	PROA
ATOM	5052	CE	LYS	P	310	-19.705	0.656	-36.516	0.00	0.00	PROA
ATOM	5053	HE1	LYS	P	310	-19.381	1.256	-37.393	0.00	0.00	PROA
ATOM	5054	HE2	LYS	P	310	-19.997	1.307	-35.665	0.00	0.00	PROA
ATOM	5055	NZ	LYS	P	310	-20.891	-0.051	-37.040	0.00	0.00	PROA
ATOM	5056	HZ1	LYS	P	310	-21.128	-0.969	-36.614	0.00	0.00	PROA
ATOM	5057	HZ2	LYS	P	310	-20.642	-0.313	-38.015	0.00	0.00	PROA
ATOM	5058	HZ3	LYS	P	310	-21.723	0.561	-37.162	0.00	0.00	PROA
ATOM	5059	C	LYS	P	310	-15.552	-2.171	-35.683	0.00	0.00	PROA

ATOM	5060	O	LYS	P	310	-15.186	-0.965	-35.882	0.00	0.00	PROA
ATOM	5061	N	THR	P	311	-15.255	-3.200	-36.492	0.00	0.00	PROA
ATOM	5062	HN	THR	P	311	-15.710	-4.074	-36.339	0.00	0.00	PROA
ATOM	5063	CA	THR	P	311	-14.304	-3.110	-37.641	0.00	0.00	PROA
ATOM	5064	HA	THR	P	311	-14.171	-2.060	-37.859	0.00	0.00	PROA
ATOM	5065	CB	THR	P	311	-12.889	-3.635	-37.343	0.00	0.00	PROA
ATOM	5066	HB	THR	P	311	-12.630	-3.313	-36.312	0.00	0.00	PROA
ATOM	5067	OG1	THR	P	311	-11.944	-3.224	-38.290	0.00	0.00	PROA
ATOM	5068	HG1	THR	P	311	-11.084	-3.443	-37.923	0.00	0.00	PROA
ATOM	5069	CG2	THR	P	311	-12.894	-5.172	-37.235	0.00	0.00	PROA
ATOM	5070	HG21	THR	P	311	-13.925	-5.446	-36.924	0.00	0.00	PROA
ATOM	5071	HG22	THR	P	311	-12.092	-5.751	-36.728	0.00	0.00	PROA
ATOM	5072	HG23	THR	P	311	-12.741	-5.540	-38.272	0.00	0.00	PROA
ATOM	5073	C	THR	P	311	-14.799	-3.611	-38.966	0.00	0.00	PROA
ATOM	5074	O	THR	P	311	-15.748	-4.345	-39.052	0.00	0.00	PROA
ATOM	5075	N	SER	P	312	-14.190	-3.132	-40.110	0.00	0.00	PROA
ATOM	5076	HN	SER	P	312	-13.339	-2.618	-40.026	0.00	0.00	PROA
ATOM	5077	CA	SER	P	312	-14.585	-3.572	-41.406	0.00	0.00	PROA
ATOM	5078	HA	SER	P	312	-14.706	-4.641	-41.321	0.00	0.00	PROA
ATOM	5079	CB	SER	P	312	-15.966	-2.973	-41.834	0.00	0.00	PROA
ATOM	5080	HB1	SER	P	312	-15.828	-1.870	-41.820	0.00	0.00	PROA
ATOM	5081	HB2	SER	P	312	-16.715	-3.296	-41.079	0.00	0.00	PROA
ATOM	5082	OG	SER	P	312	-16.317	-3.238	-43.194	0.00	0.00	PROA
ATOM	5083	HG1	SER	P	312	-16.557	-4.167	-43.147	0.00	0.00	PROA
ATOM	5084	C	SER	P	312	-13.286	-3.436	-42.276	0.00	0.00	PROA
ATOM	5085	O	SER	P	312	-12.495	-2.600	-41.895	0.00	0.00	PROA
ATOM	5086	N	ALA	P	313	-13.027	-4.291	-43.263	0.00	0.00	PROA
ATOM	5087	HN	ALA	P	313	-13.652	-5.016	-43.542	0.00	0.00	PROA
ATOM	5088	CA	ALA	P	313	-11.676	-4.399	-43.807	0.00	0.00	PROA
ATOM	5089	HA	ALA	P	313	-10.976	-3.861	-43.184	0.00	0.00	PROA
ATOM	5090	CB	ALA	P	313	-11.253	-5.898	-43.698	0.00	0.00	PROA
ATOM	5091	HB1	ALA	P	313	-10.682	-6.292	-44.567	0.00	0.00	PROA
ATOM	5092	HB2	ALA	P	313	-12.141	-6.560	-43.627	0.00	0.00	PROA
ATOM	5093	HB3	ALA	P	313	-10.579	-5.982	-42.819	0.00	0.00	PROA
ATOM	5094	C	ALA	P	313	-11.446	-3.970	-45.269	0.00	0.00	PROA
ATOM	5095	O	ALA	P	313	-10.267	-3.831	-45.652	0.00	0.00	PROA
ATOM	5096	N	GLN	P	314	-12.539	-3.805	-45.981	0.00	0.00	PROA
ATOM	5097	HN	GLN	P	314	-13.456	-3.982	-45.632	0.00	0.00	PROA
ATOM	5098	CA	GLN	P	314	-12.498	-3.453	-47.342	0.00	0.00	PROA
ATOM	5099	HA	GLN	P	314	-11.587	-3.004	-47.710	0.00	0.00	PROA
ATOM	5100	CB	GLN	P	314	-12.851	-4.698	-48.239	0.00	0.00	PROA
ATOM	5101	HB1	GLN	P	314	-12.991	-4.297	-49.265	0.00	0.00	PROA
ATOM	5102	HB2	GLN	P	314	-13.903	-4.920	-47.958	0.00	0.00	PROA
ATOM	5103	CG	GLN	P	314	-11.869	-5.828	-48.200	0.00	0.00	PROA
ATOM	5104	HG1	GLN	P	314	-12.153	-6.716	-48.804	0.00	0.00	PROA
ATOM	5105	HG2	GLN	P	314	-11.948	-6.265	-47.182	0.00	0.00	PROA
ATOM	5106	CD	GLN	P	314	-10.376	-5.552	-48.463	0.00	0.00	PROA
ATOM	5107	OE1	GLN	P	314	-10.015	-4.576	-49.109	0.00	0.00	PROA
ATOM	5108	NE2	GLN	P	314	-9.526	-6.407	-47.837	0.00	0.00	PROA
ATOM	5109	HE21	GLN	P	314	-8.550	-6.189	-47.866	0.00	0.00	PROA
ATOM	5110	HE22	GLN	P	314	-9.921	-7.080	-47.212	0.00	0.00	PROA
ATOM	5111	C	GLN	P	314	-13.577	-2.294	-47.440	0.00	0.00	PROA
ATOM	5112	O	GLN	P	314	-14.674	-2.399	-46.900	0.00	0.00	PROA
ATOM	5113	N	HSD	P	315	-13.334	-1.183	-48.190	0.00	0.00	PROA
ATOM	5114	HN	HSD	P	315	-12.490	-1.040	-48.700	0.00	0.00	PROA
ATOM	5115	CA	HSD	P	315	-14.255	-0.069	-48.183	0.00	0.00	PROA
ATOM	5116	HA	HSD	P	315	-15.233	-0.474	-47.968	0.00	0.00	PROA
ATOM	5117	CB	HSD	P	315	-13.714	0.855	-47.056	0.00	0.00	PROA
ATOM	5118	HB1	HSD	P	315	-12.616	0.977	-47.177	0.00	0.00	PROA
ATOM	5119	HB2	HSD	P	315	-13.842	0.439	-46.034	0.00	0.00	PROA
ATOM	5120	ND1	HSD	P	315	-15.541	2.547	-46.882	0.00	0.00	PROA

ATOM 5121 HD1 HSD P 315 -16.294 1.894 -46.798 0.00 0.00 PROA
ATOM 5122 CG HSD P 315 -14.196 2.287 -47.094 0.00 0.00 PROA
ATOM 5123 CE1 HSD P 315 -15.612 3.866 -46.829 0.00 0.00 PROA
ATOM 5124 HE1 HSD P 315 -16.590 4.343 -46.777 0.00 0.00 PROA
ATOM 5125 NE2 HSD P 315 -14.446 4.499 -46.935 0.00 0.00 PROA
ATOM 5126 CD2 HSD P 315 -13.543 3.458 -47.148 0.00 0.00 PROA
ATOM 5127 HD2 HSD P 315 -12.487 3.681 -47.236 0.00 0.00 PROA
ATOM 5128 C HSD P 315 -14.126 0.570 -49.458 0.00 0.00 PROA
ATOM 5129 O HSD P 315 -13.099 0.656 -50.130 0.00 0.00 PROA
ATOM 5130 N ALA P 316 -15.287 1.033 -49.920 0.00 0.00 PROA
ATOM 5131 HN ALA P 316 -16.106 1.107 -49.356 0.00 0.00 PROA
ATOM 5132 CA ALA P 316 -15.545 1.433 -51.335 0.00 0.00 PROA
ATOM 5133 HA ALA P 316 -14.632 1.284 -51.894 0.00 0.00 PROA
ATOM 5134 CB ALA P 316 -16.678 0.575 -51.966 0.00 0.00 PROA
ATOM 5135 HB1 ALA P 316 -17.597 0.804 -51.385 0.00 0.00 PROA
ATOM 5136 HB2 ALA P 316 -16.389 -0.474 -52.189 0.00 0.00 PROA
ATOM 5137 HB3 ALA P 316 -16.829 0.826 -53.038 0.00 0.00 PROA
ATOM 5138 C ALA P 316 -15.948 2.821 -51.585 0.00 0.00 PROA
ATOM 5139 O ALA P 316 -16.536 3.432 -50.668 0.00 0.00 PROA
ATOM 5140 N LEU P 317 -15.626 3.363 -52.728 0.00 0.00 PROA
ATOM 5141 HN LEU P 317 -15.064 2.934 -53.431 0.00 0.00 PROA
ATOM 5142 CA LEU P 317 -15.745 4.795 -52.969 0.00 0.00 PROA
ATOM 5143 HA LEU P 317 -15.176 5.322 -52.218 0.00 0.00 PROA
ATOM 5144 CB LEU P 317 -15.093 5.148 -54.360 0.00 0.00 PROA
ATOM 5145 HB1 LEU P 317 -15.565 4.525 -55.150 0.00 0.00 PROA
ATOM 5146 HB2 LEU P 317 -14.072 4.755 -54.167 0.00 0.00 PROA
ATOM 5147 CG LEU P 317 -15.176 6.626 -54.807 0.00 0.00 PROA
ATOM 5148 HG LEU P 317 -16.232 6.846 -55.074 0.00 0.00 PROA
ATOM 5149 CD1 LEU P 317 -14.508 7.568 -53.816 0.00 0.00 PROA
ATOM 5150 HD11 LEU P 317 -15.050 7.765 -52.867 0.00 0.00 PROA
ATOM 5151 HD12 LEU P 317 -14.132 8.553 -54.165 0.00 0.00 PROA
ATOM 5152 HD13 LEU P 317 -13.571 7.068 -53.487 0.00 0.00 PROA
ATOM 5153 CD2 LEU P 317 -14.347 6.691 -56.197 0.00 0.00 PROA
ATOM 5154 HD21 LEU P 317 -13.278 6.445 -56.019 0.00 0.00 PROA
ATOM 5155 HD22 LEU P 317 -14.438 7.694 -56.665 0.00 0.00 PROA
ATOM 5156 HD23 LEU P 317 -14.607 5.990 -57.018 0.00 0.00 PROA
ATOM 5157 C LEU P 317 -17.215 5.410 -53.123 0.00 0.00 PROA
ATOM 5158 O LEU P 317 -18.035 5.015 -53.899 0.00 0.00 PROA
ATOM 5159 N THR P 318 -17.532 6.429 -52.311 0.00 0.00 PROA
ATOM 5160 HN THR P 318 -16.869 6.659 -51.603 0.00 0.00 PROA
ATOM 5161 CA THR P 318 -18.762 7.250 -52.303 0.00 0.00 PROA
ATOM 5162 HA THR P 318 -19.598 6.628 -52.019 0.00 0.00 PROA
ATOM 5163 CB THR P 318 -18.628 8.353 -51.215 0.00 0.00 PROA
ATOM 5164 HB THR P 318 -18.205 7.756 -50.379 0.00 0.00 PROA
ATOM 5165 OG1 THR P 318 -19.931 8.780 -50.735 0.00 0.00 PROA
ATOM 5166 HG1 THR P 318 -20.217 8.051 -50.179 0.00 0.00 PROA
ATOM 5167 CG2 THR P 318 -17.684 9.580 -51.459 0.00 0.00 PROA
ATOM 5168 HG21 THR P 318 -17.863 10.066 -52.442 0.00 0.00 PROA
ATOM 5169 HG22 THR P 318 -16.691 9.127 -51.667 0.00 0.00 PROA
ATOM 5170 HG23 THR P 318 -17.727 10.322 -50.633 0.00 0.00 PROA
ATOM 5171 C THR P 318 -19.208 7.795 -53.606 0.00 0.00 PROA
ATOM 5172 O THR P 318 -18.441 8.471 -54.318 0.00 0.00 PROA
ATOM 5173 N SER P 319 -20.483 7.604 -53.920 0.00 0.00 PROA
ATOM 5174 HN SER P 319 -20.929 6.901 -53.371 0.00 0.00 PROA
ATOM 5175 CA SER P 319 -21.178 8.285 -55.066 0.00 0.00 PROA
ATOM 5176 HA SER P 319 -20.463 8.386 -55.870 0.00 0.00 PROA
ATOM 5177 CB SER P 319 -22.408 7.448 -55.552 0.00 0.00 PROA
ATOM 5178 HB1 SER P 319 -23.101 8.038 -56.188 0.00 0.00 PROA
ATOM 5179 HB2 SER P 319 -23.044 7.270 -54.658 0.00 0.00 PROA
ATOM 5180 OG SER P 319 -22.078 6.143 -56.107 0.00 0.00 PROA
ATOM 5181 HG1 SER P 319 -21.805 5.690 -55.306 0.00 0.00 PROA

ATOM	5182	C	SER	P	319	-21.815	9.632	-54.570	0.00	0.00	PROA
ATOM	5183	O	SER	P	319	-22.694	9.501	-53.688	0.00	0.00	PROA
ATOM	5184	O	SER	P	319	-21.480	10.713	-55.136	0.00	0.00	PROA
ATOM	5185	N	LEU	P	1	20.008	4.162	26.889	0.00	0.00	PROB
ATOM	5186	H1	LEU	P	1	20.757	4.375	27.579	0.00	0.00	PROB
ATOM	5187	H	LEU	P	1	20.393	4.614	26.035	0.00	0.00	PROB
ATOM	5188	H	LEU	P	1	19.945	3.131	26.763	0.00	0.00	PROB
ATOM	5189	CA	LEU	P	1	18.748	4.853	27.399	0.00	0.00	PROB
ATOM	5190	HA	LEU	P	1	18.908	5.366	28.336	0.00	0.00	PROB
ATOM	5191	CB	LEU	P	1	18.227	5.779	26.278	0.00	0.00	PROB
ATOM	5192	HB1	LEU	P	1	17.216	6.151	26.549	0.00	0.00	PROB
ATOM	5193	HB2	LEU	P	1	18.153	5.233	25.313	0.00	0.00	PROB
ATOM	5194	CG	LEU	P	1	19.136	6.969	25.916	0.00	0.00	PROB
ATOM	5195	HG	LEU	P	1	20.203	6.682	25.796	0.00	0.00	PROB
ATOM	5196	CD1	LEU	P	1	18.763	7.522	24.551	0.00	0.00	PROB
ATOM	5197	HD11	LEU	P	1	18.681	6.830	23.686	0.00	0.00	PROB
ATOM	5198	HD12	LEU	P	1	19.477	8.350	24.354	0.00	0.00	PROB
ATOM	5199	HD13	LEU	P	1	17.744	7.965	24.539	0.00	0.00	PROB
ATOM	5200	CD2	LEU	P	1	19.059	8.035	26.950	0.00	0.00	PROB
ATOM	5201	HD21	LEU	P	1	19.529	7.697	27.898	0.00	0.00	PROB
ATOM	5202	HD22	LEU	P	1	18.104	8.569	27.146	0.00	0.00	PROB
ATOM	5203	HD23	LEU	P	1	19.828	8.797	26.700	0.00	0.00	PROB
ATOM	5204	C	LEU	P	1	17.697	3.771	27.732	0.00	0.00	PROB
ATOM	5205	O	LEU	P	1	18.056	2.587	27.784	0.00	0.00	PROB
ATOM	5206	N	VAL	P	2	16.415	4.069	27.874	0.00	0.00	PROB
ATOM	5207	HN	VAL	P	2	16.080	5.008	27.843	0.00	0.00	PROB
ATOM	5208	CA	VAL	P	2	15.372	3.078	28.061	0.00	0.00	PROB
ATOM	5209	HA	VAL	P	2	15.725	2.113	27.727	0.00	0.00	PROB
ATOM	5210	CB	VAL	P	2	14.692	2.987	29.477	0.00	0.00	PROB
ATOM	5211	HB	VAL	P	2	13.881	2.229	29.476	0.00	0.00	PROB
ATOM	5212	CG1	VAL	P	2	15.764	2.497	30.433	0.00	0.00	PROB
ATOM	5213	HG11	VAL	P	2	15.182	2.092	31.289	0.00	0.00	PROB
ATOM	5214	HG12	VAL	P	2	16.477	3.292	30.740	0.00	0.00	PROB
ATOM	5215	HG13	VAL	P	2	16.418	1.658	30.112	0.00	0.00	PROB
ATOM	5216	CG2	VAL	P	2	14.102	4.405	29.853	0.00	0.00	PROB
ATOM	5217	HG21	VAL	P	2	14.910	5.125	30.104	0.00	0.00	PROB
ATOM	5218	HG22	VAL	P	2	13.398	4.277	30.703	0.00	0.00	PROB
ATOM	5219	HG23	VAL	P	2	13.508	4.772	28.989	0.00	0.00	PROB
ATOM	5220	C	VAL	P	2	14.322	3.415	26.955	0.00	0.00	PROB
ATOM	5221	O	VAL	P	2	14.250	4.520	26.402	0.00	0.00	PROB
ATOM	5222	N	ARG	P	3	13.390	2.477	26.700	0.00	0.00	PROB
ATOM	5223	HN	ARG	P	3	13.406	1.635	27.234	0.00	0.00	PROB
ATOM	5224	CA	ARG	P	3	12.403	2.744	25.692	0.00	0.00	PROB
ATOM	5225	HA	ARG	P	3	12.523	3.745	25.304	0.00	0.00	PROB
ATOM	5226	CB	ARG	P	3	12.456	1.676	24.583	0.00	0.00	PROB
ATOM	5227	HB1	ARG	P	3	12.024	0.754	25.028	0.00	0.00	PROB
ATOM	5228	HB2	ARG	P	3	13.476	1.480	24.188	0.00	0.00	PROB
ATOM	5229	CG	ARG	P	3	11.530	2.134	23.417	0.00	0.00	PROB
ATOM	5230	HG1	ARG	P	3	10.593	2.614	23.771	0.00	0.00	PROB
ATOM	5231	HG2	ARG	P	3	11.293	1.192	22.878	0.00	0.00	PROB
ATOM	5232	CD	ARG	P	3	12.340	2.992	22.426	0.00	0.00	PROB
ATOM	5233	HD1	ARG	P	3	13.187	2.298	22.240	0.00	0.00	PROB
ATOM	5234	HD2	ARG	P	3	12.765	3.952	22.791	0.00	0.00	PROB
ATOM	5235	NE	ARG	P	3	11.610	3.168	21.135	0.00	0.00	PROB
ATOM	5236	HE	ARG	P	3	11.909	2.654	20.331	0.00	0.00	PROB
ATOM	5237	CZ	ARG	P	3	10.731	4.134	20.858	0.00	0.00	PROB
ATOM	5238	NH1	ARG	P	3	10.631	5.268	21.516	0.00	0.00	PROB
ATOM	5239	HH11	ARG	P	3	11.026	5.367	22.430	0.00	0.00	PROB
ATOM	5240	HH12	ARG	P	3	9.896	5.824	21.128	0.00	0.00	PROB
ATOM	5241	NH2	ARG	P	3	10.092	4.068	19.688	0.00	0.00	PROB
ATOM	5242	HH21	ARG	P	3	9.482	4.855	19.601	0.00	0.00	PROB

ATOM 5243 HH22 ARG P 3 9.849 3.172 19.316 0.00 0.00 PROB
ATOM 5244 C ARG P 3 11.148 2.661 26.418 0.00 0.00 PROB
ATOM 5245 O ARG P 3 10.770 1.743 27.123 0.00 0.00 PROB
ATOM 5246 N TYR P 4 10.334 3.639 26.221 0.00 0.00 PROB
ATOM 5247 HN TYR P 4 10.586 4.393 25.618 0.00 0.00 PROB
ATOM 5248 CA TYR P 4 9.020 3.878 26.833 0.00 0.00 PROB
ATOM 5249 HA TYR P 4 9.076 3.362 27.780 0.00 0.00 PROB
ATOM 5250 CB TYR P 4 8.731 5.387 27.059 0.00 0.00 PROB
ATOM 5251 HB1 TYR P 4 7.790 5.478 27.644 0.00 0.00 PROB
ATOM 5252 HB2 TYR P 4 8.656 5.849 26.051 0.00 0.00 PROB
ATOM 5253 CG TYR P 4 9.733 6.092 27.819 0.00 0.00 PROB
ATOM 5254 CD1 TYR P 4 10.053 5.748 29.142 0.00 0.00 PROB
ATOM 5255 HD1 TYR P 4 9.401 5.128 29.740 0.00 0.00 PROB
ATOM 5256 CE1 TYR P 4 11.038 6.449 29.810 0.00 0.00 PROB
ATOM 5257 HE1 TYR P 4 11.396 6.071 30.756 0.00 0.00 PROB
ATOM 5258 CZ TYR P 4 11.764 7.477 29.193 0.00 0.00 PROB
ATOM 5259 OH TYR P 4 12.798 8.205 29.797 0.00 0.00 PROB
ATOM 5260 HH TYR P 4 12.946 7.930 30.705 0.00 0.00 PROB
ATOM 5261 CD2 TYR P 4 10.405 7.175 27.193 0.00 0.00 PROB
ATOM 5262 HD2 TYR P 4 10.076 7.505 26.219 0.00 0.00 PROB
ATOM 5263 CE2 TYR P 4 11.402 7.840 27.896 0.00 0.00 PROB
ATOM 5264 HE2 TYR P 4 11.942 8.615 27.372 0.00 0.00 PROB
ATOM 5265 C TYR P 4 7.790 3.302 26.106 0.00 0.00 PROB
ATOM 5266 O TYR P 4 6.642 3.767 26.293 0.00 0.00 PROB
ATOM 5267 N THR P 5 7.985 2.294 25.238 0.00 0.00 PROB
ATOM 5268 HN THR P 5 8.933 2.077 25.022 0.00 0.00 PROB
ATOM 5269 CA THR P 5 6.935 1.713 24.291 0.00 0.00 PROB
ATOM 5270 HA THR P 5 6.371 2.609 24.075 0.00 0.00 PROB
ATOM 5271 CB THR P 5 7.359 0.938 23.071 0.00 0.00 PROB
ATOM 5272 HB THR P 5 6.618 0.222 22.656 0.00 0.00 PROB
ATOM 5273 OG1 THR P 5 8.610 0.390 23.322 0.00 0.00 PROB
ATOM 5274 HG1 THR P 5 8.445 -0.554 23.267 0.00 0.00 PROB
ATOM 5275 CG2 THR P 5 7.647 1.972 21.915 0.00 0.00 PROB
ATOM 5276 HG21 THR P 5 6.685 2.514 21.794 0.00 0.00 PROB
ATOM 5277 HG22 THR P 5 7.840 1.539 20.910 0.00 0.00 PROB
ATOM 5278 HG23 THR P 5 8.556 2.542 22.202 0.00 0.00 PROB
ATOM 5279 C THR P 5 5.901 0.840 25.072 0.00 0.00 PROB
ATOM 5280 O THR P 5 6.145 -0.080 25.749 0.00 0.00 PROB
ATOM 5281 N LYS P 6 4.583 1.175 24.783 0.00 0.00 PROB
ATOM 5282 HN LYS P 6 4.328 1.927 24.180 0.00 0.00 PROB
ATOM 5283 CA LYS P 6 3.413 0.532 25.515 0.00 0.00 PROB
ATOM 5284 HA LYS P 6 3.811 -0.176 26.227 0.00 0.00 PROB
ATOM 5285 CB LYS P 6 2.580 1.697 26.118 0.00 0.00 PROB
ATOM 5286 HB1 LYS P 6 1.746 1.287 26.726 0.00 0.00 PROB
ATOM 5287 HB2 LYS P 6 2.168 2.231 25.235 0.00 0.00 PROB
ATOM 5288 CG LYS P 6 3.390 2.615 27.004 0.00 0.00 PROB
ATOM 5289 HG1 LYS P 6 4.146 3.117 26.364 0.00 0.00 PROB
ATOM 5290 HG2 LYS P 6 3.948 2.021 27.759 0.00 0.00 PROB
ATOM 5291 CD LYS P 6 2.439 3.558 27.721 0.00 0.00 PROB
ATOM 5292 HD1 LYS P 6 2.970 4.317 28.334 0.00 0.00 PROB
ATOM 5293 HD2 LYS P 6 1.695 3.054 28.375 0.00 0.00 PROB
ATOM 5294 CE LYS P 6 1.564 4.443 26.816 0.00 0.00 PROB
ATOM 5295 HE1 LYS P 6 0.681 4.749 27.418 0.00 0.00 PROB
ATOM 5296 HE2 LYS P 6 1.099 3.884 25.976 0.00 0.00 PROB
ATOM 5297 NZ LYS P 6 2.344 5.579 26.308 0.00 0.00 PROB
ATOM 5298 HZ1 LYS P 6 2.735 6.101 27.118 0.00 0.00 PROB
ATOM 5299 HZ2 LYS P 6 1.698 6.144 25.721 0.00 0.00 PROB
ATOM 5300 HZ3 LYS P 6 3.112 5.195 25.720 0.00 0.00 PROB
ATOM 5301 C LYS P 6 2.601 -0.364 24.602 0.00 0.00 PROB
ATOM 5302 O LYS P 6 1.577 -0.930 25.077 0.00 0.00 PROB
ATOM 5303 N LYS P 7 2.969 -0.594 23.293 0.00 0.00 PROB

ATOM 5304 HN LYS P 7 3.726 -0.124 22.845 0.00 0.00 PROB
ATOM 5305 CA LYS P 7 2.230 -1.455 22.463 0.00 0.00 PROB
ATOM 5306 HA LYS P 7 1.174 -1.251 22.567 0.00 0.00 PROB
ATOM 5307 CB LYS P 7 2.682 -1.185 21.008 0.00 0.00 PROB
ATOM 5308 HB1 LYS P 7 2.018 -1.823 20.386 0.00 0.00 PROB
ATOM 5309 HB2 LYS P 7 3.687 -1.610 20.800 0.00 0.00 PROB
ATOM 5310 CG LYS P 7 2.527 0.215 20.528 0.00 0.00 PROB
ATOM 5311 HG1 LYS P 7 2.839 0.157 19.463 0.00 0.00 PROB
ATOM 5312 HG2 LYS P 7 3.206 0.903 21.075 0.00 0.00 PROB
ATOM 5313 CD LYS P 7 1.094 0.760 20.491 0.00 0.00 PROB
ATOM 5314 HD1 LYS P 7 0.703 0.823 21.529 0.00 0.00 PROB
ATOM 5315 HD2 LYS P 7 0.427 0.062 19.941 0.00 0.00 PROB
ATOM 5316 CE LYS P 7 0.831 2.134 19.897 0.00 0.00 PROB
ATOM 5317 HE1 LYS P 7 1.263 2.912 20.561 0.00 0.00 PROB
ATOM 5318 HE2 LYS P 7 -0.255 2.329 19.770 0.00 0.00 PROB
ATOM 5319 NZ LYS P 7 1.524 2.316 18.584 0.00 0.00 PROB
ATOM 5320 HZ1 LYS P 7 1.392 1.548 17.896 0.00 0.00 PROB
ATOM 5321 HZ2 LYS P 7 2.562 2.357 18.638 0.00 0.00 PROB
ATOM 5322 HZ3 LYS P 7 1.268 3.265 18.246 0.00 0.00 PROB
ATOM 5323 C LYS P 7 2.420 -2.903 22.790 0.00 0.00 PROB
ATOM 5324 O LYS P 7 1.584 -3.765 22.454 0.00 0.00 PROB
ATOM 5325 N VAL P 8 3.591 -3.320 23.371 0.00 0.00 PROB
ATOM 5326 HN VAL P 8 4.324 -2.702 23.643 0.00 0.00 PROB
ATOM 5327 CA VAL P 8 3.658 -4.649 23.917 0.00 0.00 PROB
ATOM 5328 HA VAL P 8 2.986 -5.334 23.421 0.00 0.00 PROB
ATOM 5329 CB VAL P 8 5.101 -5.188 23.717 0.00 0.00 PROB
ATOM 5330 HB VAL P 8 5.823 -4.752 24.440 0.00 0.00 PROB
ATOM 5331 CG1 VAL P 8 5.279 -6.722 23.923 0.00 0.00 PROB
ATOM 5332 HG11 VAL P 8 4.989 -7.108 24.924 0.00 0.00 PROB
ATOM 5333 HG12 VAL P 8 6.341 -7.043 23.863 0.00 0.00 PROB
ATOM 5334 HG13 VAL P 8 4.747 -7.288 23.129 0.00 0.00 PROB
ATOM 5335 CG2 VAL P 8 5.574 -4.900 22.220 0.00 0.00 PROB
ATOM 5336 HG21 VAL P 8 5.865 -3.828 22.185 0.00 0.00 PROB
ATOM 5337 HG22 VAL P 8 4.758 -5.059 21.483 0.00 0.00 PROB
ATOM 5338 HG23 VAL P 8 6.543 -5.422 22.067 0.00 0.00 PROB
ATOM 5339 C VAL P 8 3.211 -4.523 25.348 0.00 0.00 PROB
ATOM 5340 O VAL P 8 3.907 -3.743 26.144 0.00 0.00 PROB
ATOM 5341 N PRO P 9 2.189 -5.162 25.883 0.00 0.00 PROB
ATOM 5342 CD PRO P 9 1.234 -6.074 25.113 0.00 0.00 PROB
ATOM 5343 HD1 PRO P 9 1.768 -6.950 24.687 0.00 0.00 PROB
ATOM 5344 HD2 PRO P 9 0.751 -5.509 24.288 0.00 0.00 PROB
ATOM 5345 CA PRO P 9 1.563 -4.672 27.166 0.00 0.00 PROB
ATOM 5346 HA PRO P 9 1.652 -3.607 27.321 0.00 0.00 PROB
ATOM 5347 CB PRO P 9 0.142 -5.181 27.107 0.00 0.00 PROB
ATOM 5348 HB1 PRO P 9 -0.434 -4.369 26.613 0.00 0.00 PROB
ATOM 5349 HB2 PRO P 9 -0.462 -5.330 28.027 0.00 0.00 PROB
ATOM 5350 CG PRO P 9 0.144 -6.396 26.162 0.00 0.00 PROB
ATOM 5351 HG1 PRO P 9 0.279 -7.326 26.754 0.00 0.00 PROB
ATOM 5352 HG2 PRO P 9 -0.820 -6.441 25.612 0.00 0.00 PROB
ATOM 5353 C PRO P 9 2.122 -5.332 28.406 0.00 0.00 PROB
ATOM 5354 O PRO P 9 1.494 -6.240 28.990 0.00 0.00 PROB
ATOM 5355 N GLN P 10 3.323 -4.901 28.817 0.00 0.00 PROB
ATOM 5356 HN GLN P 10 3.831 -4.217 28.300 0.00 0.00 PROB
ATOM 5357 CA GLN P 10 3.926 -5.266 30.053 0.00 0.00 PROB
ATOM 5358 HA GLN P 10 3.167 -5.504 30.784 0.00 0.00 PROB
ATOM 5359 CB GLN P 10 4.916 -6.482 29.907 0.00 0.00 PROB
ATOM 5360 HB1 GLN P 10 4.288 -7.320 29.535 0.00 0.00 PROB
ATOM 5361 HB2 GLN P 10 5.428 -6.780 30.848 0.00 0.00 PROB
ATOM 5362 CG GLN P 10 6.065 -6.156 28.974 0.00 0.00 PROB
ATOM 5363 HG1 GLN P 10 6.721 -5.374 29.411 0.00 0.00 PROB
ATOM 5364 HG2 GLN P 10 5.576 -5.876 28.017 0.00 0.00 PROB

```
ATOM   5365  CD  GLN P  10       6.836  -7.439  28.632  0.00  0.00      PROB
ATOM   5366  OE1 GLN P  10       6.575  -8.452  29.254  0.00  0.00      PROB
ATOM   5367  NE2 GLN P  10       7.824  -7.386  27.723  0.00  0.00      PROB
ATOM   5368 HE21 GLN P  10       7.961  -6.486  27.310  0.00  0.00      PROB
ATOM   5369 HE22 GLN P  10       8.155  -8.227  27.296  0.00  0.00      PROB
ATOM   5370  C   GLN P  10       4.676  -4.084  30.663  0.00  0.00      PROB
ATOM   5371  O   GLN P  10       5.125  -3.184  29.984  0.00  0.00      PROB
ATOM   5372  N   VAL P  11       4.870  -4.056  31.996  0.00  0.00      PROB
ATOM   5373  HN  VAL P  11       4.697  -4.888  32.516  0.00  0.00      PROB
ATOM   5374  CA  VAL P  11       5.486  -3.024  32.808  0.00  0.00      PROB
ATOM   5375  HA  VAL P  11       4.908  -2.141  32.578  0.00  0.00      PROB
ATOM   5376  CB  VAL P  11       5.175  -3.288  34.247  0.00  0.00      PROB
ATOM   5377  HB  VAL P  11       5.493  -4.301  34.573  0.00  0.00      PROB
ATOM   5378  CG1 VAL P  11       5.769  -2.234  35.141  0.00  0.00      PROB
ATOM   5379 HG11 VAL P  11       5.509  -1.212  34.793  0.00  0.00      PROB
ATOM   5380 HG12 VAL P  11       6.872  -2.315  35.249  0.00  0.00      PROB
ATOM   5381 HG13 VAL P  11       5.260  -2.253  36.128  0.00  0.00      PROB
ATOM   5382  CG2 VAL P  11       3.607  -3.307  34.412  0.00  0.00      PROB
ATOM   5383 HG21 VAL P  11       3.189  -2.414  33.901  0.00  0.00      PROB
ATOM   5384 HG22 VAL P  11       3.411  -3.165  35.497  0.00  0.00      PROB
ATOM   5385 HG23 VAL P  11       3.099  -4.227  34.052  0.00  0.00      PROB
ATOM   5386  C   VAL P  11       6.969  -2.653  32.636  0.00  0.00      PROB
ATOM   5387  O   VAL P  11       7.394  -1.550  32.846  0.00  0.00      PROB
ATOM   5388  N   SER P  12       7.791  -3.630  32.256  0.00  0.00      PROB
ATOM   5389  HN  SER P  12       7.451  -4.530  31.995  0.00  0.00      PROB
ATOM   5390  CA  SER P  12       9.169  -3.305  31.967  0.00  0.00      PROB
ATOM   5391  HA  SER P  12       9.463  -2.712  32.821  0.00  0.00      PROB
ATOM   5392  CB  SER P  12      10.064  -4.558  31.818  0.00  0.00      PROB
ATOM   5393  HB1 SER P  12      10.231  -5.087  32.781  0.00  0.00      PROB
ATOM   5394  HB2 SER P  12      11.108  -4.393  31.475  0.00  0.00      PROB
ATOM   5395  OG  SER P  12       9.365  -5.520  30.918  0.00  0.00      PROB
ATOM   5396  HG1 SER P  12       9.130  -6.245  31.502  0.00  0.00      PROB
ATOM   5397  C   SER P  12       9.420  -2.525  30.745  0.00  0.00      PROB
ATOM   5398  O   SER P  12       8.746  -2.555  29.677  0.00  0.00      PROB
ATOM   5399  N   THR P  13      10.488  -1.683  30.827  0.00  0.00      PROB
ATOM   5400  HN  THR P  13      11.075  -1.760  31.629  0.00  0.00      PROB
ATOM   5401  CA  THR P  13      10.888  -0.677  29.786  0.00  0.00      PROB
ATOM   5402  HA  THR P  13      10.141  -0.535  29.019  0.00  0.00      PROB
ATOM   5403  CB  THR P  13      11.221   0.727  30.388  0.00  0.00      PROB
ATOM   5404  HB  THR P  13      11.804   1.416  29.740  0.00  0.00      PROB
ATOM   5405  OG1 THR P  13      11.877   0.578  31.636  0.00  0.00      PROB
ATOM   5406  HG1 THR P  13      12.760   0.272  31.418  0.00  0.00      PROB
ATOM   5407  CG2 THR P  13       9.958   1.393  30.612  0.00  0.00      PROB
ATOM   5408 HG21 THR P  13       9.391   0.869  31.411  0.00  0.00      PROB
ATOM   5409 HG22 THR P  13       9.258   1.505  29.756  0.00  0.00      PROB
ATOM   5410 HG23 THR P  13      10.144   2.434  30.953  0.00  0.00      PROB
ATOM   5411  C   THR P  13      12.167  -1.259  29.211  0.00  0.00      PROB
ATOM   5412  O   THR P  13      13.112  -1.412  29.978  0.00  0.00      PROB
ATOM   5413  N   PRO P  14      12.298  -1.694  27.898  0.00  0.00      PROB
ATOM   5414  CD  PRO P  14      11.127  -1.826  26.973  0.00  0.00      PROB
ATOM   5415  HD1 PRO P  14      10.813  -0.809  26.656  0.00  0.00      PROB
ATOM   5416  HD2 PRO P  14      10.278  -2.339  27.473  0.00  0.00      PROB
ATOM   5417  CA  PRO P  14      13.461  -2.236  27.289  0.00  0.00      PROB
ATOM   5418  HA  PRO P  14      13.703  -3.113  27.870  0.00  0.00      PROB
ATOM   5419  CB  PRO P  14      13.082  -2.363  25.801  0.00  0.00      PROB
ATOM   5420  HB1 PRO P  14      13.656  -3.199  25.347  0.00  0.00      PROB
ATOM   5421  HB2 PRO P  14      13.240  -1.420  25.235  0.00  0.00      PROB
ATOM   5422  CG  PRO P  14      11.578  -2.647  25.763  0.00  0.00      PROB
ATOM   5423  HG1 PRO P  14      11.069  -2.289  24.842  0.00  0.00      PROB
ATOM   5424  HG2 PRO P  14      11.427  -3.745  25.827  0.00  0.00      PROB
ATOM   5425  C   PRO P  14      14.671  -1.273  27.420  0.00  0.00      PROB
```

```
ATOM   5426  O   PRO P  14      14.445  -0.053  27.247  0.00  0.00      PROB
ATOM   5427  N   THR P  15      15.884  -1.818  27.713  0.00  0.00      PROB
ATOM   5428  HN  THR P  15      15.947  -2.809  27.794  0.00  0.00      PROB
ATOM   5429  CA  THR P  15      17.019  -1.021  28.140  0.00  0.00      PROB
ATOM   5430  HA  THR P  15      16.704  -0.007  28.340  0.00  0.00      PROB
ATOM   5431  CB  THR P  15      17.642  -1.491  29.416  0.00  0.00      PROB
ATOM   5432  HB  THR P  15      17.996  -2.521  29.197  0.00  0.00      PROB
ATOM   5433  OG1 THR P  15      16.575  -1.607  30.379  0.00  0.00      PROB
ATOM   5434  HG1 THR P  15      15.800  -1.940  29.922  0.00  0.00      PROB
ATOM   5435  CG2 THR P  15      18.679  -0.543  29.943  0.00  0.00      PROB
ATOM   5436 HG21 THR P  15      19.242  -1.050  30.756  0.00  0.00      PROB
ATOM   5437 HG22 THR P  15      18.090   0.250  30.452  0.00  0.00      PROB
ATOM   5438 HG23 THR P  15      19.459  -0.278  29.198  0.00  0.00      PROB
ATOM   5439  C   THR P  15      18.009  -1.198  27.015  0.00  0.00      PROB
ATOM   5440  O   THR P  15      18.312  -2.285  26.508  0.00  0.00      PROB
ATOM   5441  N   LEU P  16      18.532  -0.036  26.568  0.00  0.00      PROB
ATOM   5442  HN  LEU P  16      18.249   0.834  26.966  0.00  0.00      PROB
ATOM   5443  CA  LEU P  16      19.336   0.220  25.401  0.00  0.00      PROB
ATOM   5444  HA  LEU P  16      19.221  -0.692  24.834  0.00  0.00      PROB
ATOM   5445  CB  LEU P  16      18.800   1.320  24.550  0.00  0.00      PROB
ATOM   5446  HB1 LEU P  16      19.228   1.219  23.530  0.00  0.00      PROB
ATOM   5447  HB2 LEU P  16      19.195   2.283  24.938  0.00  0.00      PROB
ATOM   5448  CG  LEU P  16      17.254   1.445  24.408  0.00  0.00      PROB
ATOM   5449  HG  LEU P  16      16.800   1.520  25.419  0.00  0.00      PROB
ATOM   5450  CD1 LEU P  16      16.882   2.595  23.489  0.00  0.00      PROB
ATOM   5451 HD11 LEU P  16      15.817   2.910  23.452  0.00  0.00      PROB
ATOM   5452 HD12 LEU P  16      17.144   2.309  22.448  0.00  0.00      PROB
ATOM   5453 HD13 LEU P  16      17.410   3.506  23.840  0.00  0.00      PROB
ATOM   5454  CD2 LEU P  16      16.618   0.098  23.835  0.00  0.00      PROB
ATOM   5455 HD21 LEU P  16      17.061   0.102  22.816  0.00  0.00      PROB
ATOM   5456 HD22 LEU P  16      15.508   0.132  23.874  0.00  0.00      PROB
ATOM   5457 HD23 LEU P  16      16.983  -0.796  24.385  0.00  0.00      PROB
ATOM   5458  C   LEU P  16      20.801   0.491  25.669  0.00  0.00      PROB
ATOM   5459  O   LEU P  16      21.151   1.540  26.307  0.00  0.00      PROB
ATOM   5460  O   LEU P  16      21.701  -0.285  25.199  0.00  0.00      PROB
END
```

2. A representative structure from the highest populated cluster of CTER-IN

```
CRYST1   90.602   92.132  141.001  90.00  90.00  90.00 P 1           1
ATOM      1  N   MET P   1      14.338  19.031  33.137  0.00  0.00      PROA
ATOM      2  HT1 MET P   1      13.953  19.934  33.481  0.00  0.00      PROA
ATOM      3  HT2 MET P   1      14.978  19.345  32.380  0.00  0.00      PROA
ATOM      4  HT3 MET P   1      14.783  18.648  33.996  0.00  0.00      PROA
ATOM      5  CA  MET P   1      13.320  18.144  32.574  0.00  0.00      PROA
ATOM      6  HA  MET P   1      13.546  17.160  32.957  0.00  0.00      PROA
ATOM      7  CB  MET P   1      11.951  18.640  32.920  0.00  0.00      PROA
ATOM      8  HB1 MET P   1      11.226  18.473  32.095  0.00  0.00      PROA
ATOM      9  HB2 MET P   1      11.986  19.748  32.996  0.00  0.00      PROA
ATOM     10  CG  MET P   1      11.320  17.890  34.176  0.00  0.00      PROA
ATOM     11  HG1 MET P   1      11.231  16.798  33.995  0.00  0.00      PROA
ATOM     12  HG2 MET P   1      10.265  18.238  34.189  0.00  0.00      PROA
ATOM     13  SD  MET P   1      12.193  18.275  35.705  0.00  0.00      PROA
ATOM     14  CE  MET P   1      11.171  19.812  35.861  0.00  0.00      PROA
ATOM     15  HE1 MET P   1      11.271  20.470  34.971  0.00  0.00      PROA
ATOM     16  HE2 MET P   1      11.413  20.403  36.770  0.00  0.00      PROA
ATOM     17  HE3 MET P   1      10.085  19.587  35.935  0.00  0.00      PROA
ATOM     18  C   MET P   1      13.477  18.140  31.093  0.00  0.00      PROA
ATOM     19  O   MET P   1      14.175  19.005  30.583  0.00  0.00      PROA
ATOM     20  N   GLU P   2      12.830  17.228  30.399  0.00  0.00      PROA
ATOM     21  HN  GLU P   2      12.145  16.610  30.776  0.00  0.00      PROA
ATOM     22  CA  GLU P   2      12.976  17.042  28.983  0.00  0.00      PROA
ATOM     23  HA  GLU P   2      13.916  17.475  28.675  0.00  0.00      PROA
ATOM     24  CB  GLU P   2      13.003  15.438  28.849  0.00  0.00      PROA
ATOM     25  HB1 GLU P   2      12.892  15.074  27.806  0.00  0.00      PROA
ATOM     26  HB2 GLU P   2      12.099  15.010  29.332  0.00  0.00      PROA
ATOM     27  CG  GLU P   2      14.310  14.787  29.377  0.00  0.00      PROA
ATOM     28  HG1 GLU P   2      14.548  15.194  30.383  0.00  0.00      PROA
ATOM     29  HG2 GLU P   2      15.069  15.068  28.616  0.00  0.00      PROA
ATOM     30  CD  GLU P   2      14.105  13.325  29.664  0.00  0.00      PROA
ATOM     31  OE1 GLU P   2      14.716  12.422  28.983  0.00  0.00      PROA
ATOM     32  OE2 GLU P   2      13.340  12.993  30.615  0.00  0.00      PROA
ATOM     33  C   GLU P   2      11.910  17.620  28.107  0.00  0.00      PROA
ATOM     34  O   GLU P   2      10.761  17.646  28.379  0.00  0.00      PROA
ATOM     35  N   GLY P   3      12.316  18.146  26.942  0.00  0.00      PROA
ATOM     36  HN  GLY P   3      13.294  18.173  26.749  0.00  0.00      PROA
ATOM     37  CA  GLY P   3      11.547  18.877  25.913  0.00  0.00      PROA
ATOM     38  HA1 GLY P   3      10.572  18.421  25.817  0.00  0.00      PROA
ATOM     39  HA2 GLY P   3      12.121  18.888  24.998  0.00  0.00      PROA
ATOM     40  C   GLY P   3      11.176  20.274  26.364  0.00  0.00      PROA
ATOM     41  O   GLY P   3      11.863  20.839  27.181  0.00  0.00      PROA
ATOM     42  N   ILE P   4      10.083  20.838  25.795  0.00  0.00      PROA
ATOM     43  HN  ILE P   4       9.449  20.347  25.202  0.00  0.00      PROA
ATOM     44  CA  ILE P   4       9.714  22.186  25.987  0.00  0.00      PROA
ATOM     45  HA  ILE P   4      10.591  22.814  25.936  0.00  0.00      PROA
ATOM     46  CB  ILE P   4       8.746  22.617  24.969  0.00  0.00      PROA
ATOM     47  HB  ILE P   4       7.874  21.937  24.863  0.00  0.00      PROA
ATOM     48  CG2 ILE P   4       8.123  24.010  25.273  0.00  0.00      PROA
ATOM     49 HG21 ILE P   4       8.845  24.818  25.516  0.00  0.00      PROA
ATOM     50 HG22 ILE P   4       7.443  24.041  26.151  0.00  0.00      PROA
ATOM     51 HG23 ILE P   4       7.592  24.417  24.386  0.00  0.00      PROA
ATOM     52  CG1 ILE P   4       9.362  22.592  23.499  0.00  0.00      PROA
ATOM     53 HG11 ILE P   4       8.503  22.861  22.848  0.00  0.00      PROA
ATOM     54 HG12 ILE P   4       9.685  21.569  23.212  0.00  0.00      PROA
ATOM     55  CD  ILE P   4      10.450  23.594  23.075  0.00  0.00      PROA
ATOM     56  HD1 ILE P   4      10.864  23.509  22.048  0.00  0.00      PROA
ATOM     57  HD2 ILE P   4      11.316  23.550  23.770  0.00  0.00      PROA
```

```
ATOM     58  HD3 ILE P   4      10.155  24.664  23.115  0.00  0.00      PROA
ATOM     59  C   ILE P   4       9.113  22.415  27.326  0.00  0.00      PROA
ATOM     60  O   ILE P   4       8.072  21.902  27.602  0.00  0.00      PROA
ATOM     61  N   SER P   5       9.752  23.190  28.195  0.00  0.00      PROA
ATOM     62  HN  SER P   5      10.697  23.445  28.004  0.00  0.00      PROA
ATOM     63  CA  SER P   5       9.143  23.682  29.348  0.00  0.00      PROA
ATOM     64  HA  SER P   5       8.174  24.074  29.077  0.00  0.00      PROA
ATOM     65  CB  SER P   5       9.102  22.630  30.500  0.00  0.00      PROA
ATOM     66  HB1 SER P   5       9.994  22.710  31.158  0.00  0.00      PROA
ATOM     67  HB2 SER P   5       9.130  21.682  29.922  0.00  0.00      PROA
ATOM     68  OG  SER P   5       7.837  22.672  31.174  0.00  0.00      PROA
ATOM     69  HG1 SER P   5       7.830  21.721  31.305  0.00  0.00      PROA
ATOM     70  C   SER P   5       9.871  24.931  29.835  0.00  0.00      PROA
ATOM     71  O   SER P   5      10.998  25.188  29.401  0.00  0.00      PROA
ATOM     72  N   ILE P   6       9.257  25.675  30.742  0.00  0.00      PROA
ATOM     73  HN  ILE P   6       8.370  25.537  31.176  0.00  0.00      PROA
ATOM     74  CA  ILE P   6       9.656  27.028  31.074  0.00  0.00      PROA
ATOM     75  HA  ILE P   6      10.249  27.432  30.267  0.00  0.00      PROA
ATOM     76  CB  ILE P   6       8.511  27.931  31.375  0.00  0.00      PROA
ATOM     77  HB  ILE P   6       8.016  27.474  32.258  0.00  0.00      PROA
ATOM     78  CG2 ILE P   6       8.933  29.310  31.895  0.00  0.00      PROA
ATOM     79 HG21 ILE P   6       9.484  29.147  32.846  0.00  0.00      PROA
ATOM     80 HG22 ILE P   6       8.042  29.920  32.156  0.00  0.00      PROA
ATOM     81 HG23 ILE P   6       9.614  29.819  31.181  0.00  0.00      PROA
ATOM     82  CG1 ILE P   6       7.460  28.115  30.216  0.00  0.00      PROA
ATOM     83 HG11 ILE P   6       6.808  28.920  30.619  0.00  0.00      PROA
ATOM     84 HG12 ILE P   6       6.898  27.159  30.158  0.00  0.00      PROA
ATOM     85  CD  ILE P   6       8.148  28.413  28.877  0.00  0.00      PROA
ATOM     86  HD1 ILE P   6       8.894  29.233  28.951  0.00  0.00      PROA
ATOM     87  HD2 ILE P   6       7.367  28.724  28.151  0.00  0.00      PROA
ATOM     88  HD3 ILE P   6       8.676  27.523  28.472  0.00  0.00      PROA
ATOM     89  C   ILE P   6      10.606  26.882  32.280  0.00  0.00      PROA
ATOM     90  O   ILE P   6      10.200  26.457  33.355  0.00  0.00      PROA
ATOM     91  N   TYR P   7      11.863  27.327  32.157  0.00  0.00      PROA
ATOM     92  HN  TYR P   7      12.186  27.688  31.285  0.00  0.00      PROA
ATOM     93  CA  TYR P   7      12.897  27.137  33.103  0.00  0.00      PROA
ATOM     94  HA  TYR P   7      12.645  26.301  33.738  0.00  0.00      PROA
ATOM     95  CB  TYR P   7      14.186  26.680  32.442  0.00  0.00      PROA
ATOM     96  HB1 TYR P   7      15.031  26.528  33.147  0.00  0.00      PROA
ATOM     97  HB2 TYR P   7      14.552  27.525  31.821  0.00  0.00      PROA
ATOM     98  CG  TYR P   7      14.147  25.354  31.715  0.00  0.00      PROA
ATOM     99  CD1 TYR P   7      13.334  24.145  31.948  0.00  0.00      PROA
ATOM    100  HD1 TYR P   7      12.544  24.266  32.674  0.00  0.00      PROA
ATOM    101  CE1 TYR P   7      13.358  22.972  31.172  0.00  0.00      PROA
ATOM    102  HE1 TYR P   7      12.646  22.186  31.379  0.00  0.00      PROA
ATOM    103  CZ  TYR P   7      14.149  23.067  30.049  0.00  0.00      PROA
ATOM    104  OH  TYR P   7      13.974  22.018  29.187  0.00  0.00      PROA
ATOM    105  HH  TYR P   7      13.442  21.360  29.641  0.00  0.00      PROA
ATOM    106  CD2 TYR P   7      15.071  25.224  30.671  0.00  0.00      PROA
ATOM    107  HD2 TYR P   7      15.764  25.987  30.349  0.00  0.00      PROA
ATOM    108  CE2 TYR P   7      15.080  24.095  29.862  0.00  0.00      PROA
ATOM    109  HE2 TYR P   7      15.652  24.203  28.953  0.00  0.00      PROA
ATOM    110  C   TYR P   7      13.078  28.436  33.919  0.00  0.00      PROA
ATOM    111  O   TYR P   7      13.672  29.444  33.469  0.00  0.00      PROA
ATOM    112  N   THR P   8      12.433  28.388  35.057  0.00  0.00      PROA
ATOM    113  HN  THR P   8      11.943  27.558  35.314  0.00  0.00      PROA
ATOM    114  CA  THR P   8      12.368  29.465  36.079  0.00  0.00      PROA
ATOM    115  HA  THR P   8      13.273  30.055  36.068  0.00  0.00      PROA
ATOM    116  CB  THR P   8      11.205  30.468  35.820  0.00  0.00      PROA
ATOM    117  HB  THR P   8      10.934  30.957  36.781  0.00  0.00      PROA
ATOM    118  OG1 THR P   8      10.023  29.853  35.480  0.00  0.00      PROA
```

ATOM	119	HG1	THR	P	8	9.741	29.346	36.245	0.00	0.00	PROA
ATOM	120	CG2	THR	P	8	11.574	31.490	34.768	0.00	0.00	PROA
ATOM	121	HG21	THR	P	8	11.592	31.081	33.735	0.00	0.00	PROA
ATOM	122	HG22	THR	P	8	12.481	32.055	35.069	0.00	0.00	PROA
ATOM	123	HG23	THR	P	8	10.757	32.242	34.781	0.00	0.00	PROA
ATOM	124	C	THR	P	8	12.243	29.021	37.486	0.00	0.00	PROA
ATOM	125	O	THR	P	8	11.827	29.722	38.445	0.00	0.00	PROA
ATOM	126	N	SER	P	9	12.549	27.723	37.656	0.00	0.00	PROA
ATOM	127	HN	SER	P	9	12.607	27.080	36.896	0.00	0.00	PROA
ATOM	128	CA	SER	P	9	12.597	27.035	38.961	0.00	0.00	PROA
ATOM	129	HA	SER	P	9	12.608	27.788	39.735	0.00	0.00	PROA
ATOM	130	CB	SER	P	9	11.338	26.092	39.265	0.00	0.00	PROA
ATOM	131	HB1	SER	P	9	11.067	25.353	38.481	0.00	0.00	PROA
ATOM	132	HB2	SER	P	9	10.594	26.906	39.132	0.00	0.00	PROA
ATOM	133	OG	SER	P	9	11.349	25.478	40.557	0.00	0.00	PROA
ATOM	134	HG1	SER	P	9	11.358	26.148	41.245	0.00	0.00	PROA
ATOM	135	C	SER	P	9	13.821	26.141	39.082	0.00	0.00	PROA
ATOM	136	O	SER	P	9	13.970	25.229	38.254	0.00	0.00	PROA
ATOM	137	N	ASP	P	10	14.737	26.310	40.106	0.00	0.00	PROA
ATOM	138	HN	ASP	P	10	14.627	27.124	40.671	0.00	0.00	PROA
ATOM	139	CA	ASP	P	10	15.985	25.517	40.283	0.00	0.00	PROA
ATOM	140	HA	ASP	P	10	16.426	25.206	39.348	0.00	0.00	PROA
ATOM	141	CB	ASP	P	10	17.040	26.380	40.923	0.00	0.00	PROA
ATOM	142	HB1	ASP	P	10	17.788	25.759	41.460	0.00	0.00	PROA
ATOM	143	HB2	ASP	P	10	16.694	26.928	41.825	0.00	0.00	PROA
ATOM	144	CG	ASP	P	10	17.773	27.265	39.905	0.00	0.00	PROA
ATOM	145	OD1	ASP	P	10	18.574	28.143	40.295	0.00	0.00	PROA
ATOM	146	OD2	ASP	P	10	17.578	27.097	38.644	0.00	0.00	PROA
ATOM	147	C	ASP	P	10	15.756	24.353	41.292	0.00	0.00	PROA
ATOM	148	O	ASP	P	10	16.590	23.463	41.358	0.00	0.00	PROA
ATOM	149	N	ASN	P	11	14.593	24.359	41.955	0.00	0.00	PROA
ATOM	150	HN	ASN	P	11	14.146	25.247	42.030	0.00	0.00	PROA
ATOM	151	CA	ASN	P	11	14.101	23.410	42.901	0.00	0.00	PROA
ATOM	152	HA	ASN	P	11	14.846	22.781	43.368	0.00	0.00	PROA
ATOM	153	CB	ASN	P	11	13.406	24.101	44.110	0.00	0.00	PROA
ATOM	154	HB1	ASN	P	11	13.262	23.365	44.929	0.00	0.00	PROA
ATOM	155	HB2	ASN	P	11	12.455	24.572	43.779	0.00	0.00	PROA
ATOM	156	CG	ASN	P	11	14.286	25.165	44.786	0.00	0.00	PROA
ATOM	157	OD1	ASN	P	11	15.509	25.094	44.747	0.00	0.00	PROA
ATOM	158	ND2	ASN	P	11	13.671	26.193	45.414	0.00	0.00	PROA
ATOM	159	HD21	ASN	P	11	12.804	26.502	45.023	0.00	0.00	PROA
ATOM	160	HD22	ASN	P	11	14.223	26.737	46.046	0.00	0.00	PROA
ATOM	161	C	ASN	P	11	13.099	22.545	42.272	0.00	0.00	PROA
ATOM	162	O	ASN	P	11	12.415	21.765	42.945	0.00	0.00	PROA
ATOM	163	N	TYR	P	12	13.065	22.495	40.851	0.00	0.00	PROA
ATOM	164	HN	TYR	P	12	13.660	23.153	40.395	0.00	0.00	PROA
ATOM	165	CA	TYR	P	12	12.262	21.631	39.989	0.00	0.00	PROA
ATOM	166	HA	TYR	P	12	12.368	22.075	39.010	0.00	0.00	PROA
ATOM	167	CB	TYR	P	12	12.789	20.138	39.997	0.00	0.00	PROA
ATOM	168	HB1	TYR	P	12	12.216	19.486	39.304	0.00	0.00	PROA
ATOM	169	HB2	TYR	P	12	12.800	19.761	41.042	0.00	0.00	PROA
ATOM	170	CG	TYR	P	12	14.194	20.116	39.514	0.00	0.00	PROA
ATOM	171	CD1	TYR	P	12	15.307	20.112	40.322	0.00	0.00	PROA
ATOM	172	HD1	TYR	P	12	15.276	20.046	41.399	0.00	0.00	PROA
ATOM	173	CE1	TYR	P	12	16.615	20.346	39.697	0.00	0.00	PROA
ATOM	174	HE1	TYR	P	12	17.467	20.366	40.360	0.00	0.00	PROA
ATOM	175	CZ	TYR	P	12	16.785	20.475	38.311	0.00	0.00	PROA
ATOM	176	OH	TYR	P	12	18.044	20.613	37.750	0.00	0.00	PROA
ATOM	177	HH	TYR	P	12	17.913	20.454	36.812	0.00	0.00	PROA
ATOM	178	CD2	TYR	P	12	14.383	20.187	38.135	0.00	0.00	PROA
ATOM	179	HD2	TYR	P	12	13.551	20.295	37.456	0.00	0.00	PROA

ATOM	180	CE2	TYR	P	12	15.612	20.440	37.547	0.00	0.00	PROA
ATOM	181	HE2	TYR	P	12	15.701	20.577	36.479	0.00	0.00	PROA
ATOM	182	C	TYR	P	12	10.727	21.714	40.300	0.00	0.00	PROA
ATOM	183	O	TYR	P	12	10.046	20.680	40.188	0.00	0.00	PROA
ATOM	184	N	THR	P	13	10.140	22.838	40.690	0.00	0.00	PROA
ATOM	185	HN	THR	P	13	10.643	23.699	40.689	0.00	0.00	PROA
ATOM	186	CA	THR	P	13	8.717	22.963	41.147	0.00	0.00	PROA
ATOM	187	HA	THR	P	13	8.590	22.385	42.051	0.00	0.00	PROA
ATOM	188	CB	THR	P	13	8.479	24.390	41.672	0.00	0.00	PROA
ATOM	189	HB	THR	P	13	8.978	25.133	41.013	0.00	0.00	PROA
ATOM	190	OG1	THR	P	13	9.120	24.514	42.921	0.00	0.00	PROA
ATOM	191	HG1	THR	P	13	10.052	24.308	42.816	0.00	0.00	PROA
ATOM	192	CG2	THR	P	13	6.961	24.618	41.908	0.00	0.00	PROA
ATOM	193	HG21	THR	P	13	6.840	25.646	42.313	0.00	0.00	PROA
ATOM	194	HG22	THR	P	13	6.496	23.813	42.517	0.00	0.00	PROA
ATOM	195	HG23	THR	P	13	6.376	24.540	40.967	0.00	0.00	PROA
ATOM	196	C	THR	P	13	7.734	22.597	40.131	0.00	0.00	PROA
ATOM	197	O	THR	P	13	7.900	22.960	38.964	0.00	0.00	PROA
ATOM	198	N	GLU	P	14	6.664	21.808	40.396	0.00	0.00	PROA
ATOM	199	HN	GLU	P	14	6.369	21.608	41.327	0.00	0.00	PROA
ATOM	200	CA	GLU	P	14	5.712	21.308	39.468	0.00	0.00	PROA
ATOM	201	HA	GLU	P	14	5.959	20.335	39.069	0.00	0.00	PROA
ATOM	202	CB	GLU	P	14	4.442	20.960	40.374	0.00	0.00	PROA
ATOM	203	HB1	GLU	P	14	4.205	21.892	40.930	0.00	0.00	PROA
ATOM	204	HB2	GLU	P	14	4.786	20.101	40.989	0.00	0.00	PROA
ATOM	205	CG	GLU	P	14	3.111	20.581	39.681	0.00	0.00	PROA
ATOM	206	HG1	GLU	P	14	3.278	19.865	38.848	0.00	0.00	PROA
ATOM	207	HG2	GLU	P	14	2.676	21.477	39.188	0.00	0.00	PROA
ATOM	208	CD	GLU	P	14	2.028	20.047	40.595	0.00	0.00	PROA
ATOM	209	OE1	GLU	P	14	0.900	19.789	40.086	0.00	0.00	PROA
ATOM	210	OE2	GLU	P	14	2.289	19.776	41.845	0.00	0.00	PROA
ATOM	211	C	GLU	P	14	5.253	22.202	38.267	0.00	0.00	PROA
ATOM	212	O	GLU	P	14	4.827	23.308	38.433	0.00	0.00	PROA
ATOM	213	N	GLU	P	15	5.277	21.650	37.091	0.00	0.00	PROA
ATOM	214	HN	GLU	P	15	5.664	20.731	37.068	0.00	0.00	PROA
ATOM	215	CA	GLU	P	15	4.798	22.255	35.828	0.00	0.00	PROA
ATOM	216	HA	GLU	P	15	5.272	23.220	35.727	0.00	0.00	PROA
ATOM	217	CB	GLU	P	15	5.116	21.407	34.641	0.00	0.00	PROA
ATOM	218	HB1	GLU	P	15	4.674	21.911	33.755	0.00	0.00	PROA
ATOM	219	HB2	GLU	P	15	4.611	20.429	34.790	0.00	0.00	PROA
ATOM	220	CG	GLU	P	15	6.638	21.293	34.515	0.00	0.00	PROA
ATOM	221	HG1	GLU	P	15	7.096	20.652	35.298	0.00	0.00	PROA
ATOM	222	HG2	GLU	P	15	7.142	22.276	34.631	0.00	0.00	PROA
ATOM	223	CD	GLU	P	15	7.026	20.675	33.163	0.00	0.00	PROA
ATOM	224	OE1	GLU	P	15	8.219	20.644	32.797	0.00	0.00	PROA
ATOM	225	OE2	GLU	P	15	6.078	20.084	32.493	0.00	0.00	PROA
ATOM	226	C	GLU	P	15	3.285	22.519	35.965	0.00	0.00	PROA
ATOM	227	O	GLU	P	15	2.475	21.861	36.525	0.00	0.00	PROA
ATOM	228	N	MET	P	16	2.835	23.725	35.493	0.00	0.00	PROA
ATOM	229	HN	MET	P	16	3.531	24.356	35.159	0.00	0.00	PROA
ATOM	230	CA	MET	P	16	1.456	24.106	35.600	0.00	0.00	PROA
ATOM	231	HA	MET	P	16	0.805	23.252	35.720	0.00	0.00	PROA
ATOM	232	CB	MET	P	16	1.265	25.267	36.657	0.00	0.00	PROA
ATOM	233	HB1	MET	P	16	0.239	25.685	36.572	0.00	0.00	PROA
ATOM	234	HB2	MET	P	16	1.979	26.115	36.590	0.00	0.00	PROA
ATOM	235	CG	MET	P	16	1.428	24.806	38.133	0.00	0.00	PROA
ATOM	236	HG1	MET	P	16	2.425	24.317	38.161	0.00	0.00	PROA
ATOM	237	HG2	MET	P	16	0.675	24.025	38.372	0.00	0.00	PROA
ATOM	238	SD	MET	P	16	1.477	26.120	39.377	0.00	0.00	PROA
ATOM	239	CE	MET	P	16	1.989	24.942	40.654	0.00	0.00	PROA
ATOM	240	HE1	MET	P	16	2.992	24.559	40.367	0.00	0.00	PROA

```
ATOM    241  HE2 MET P  16       1.296  24.106  40.887  0.00  0.00      PROA
ATOM    242  HE3 MET P  16       2.122  25.412  41.652  0.00  0.00      PROA
ATOM    243  C   MET P  16       0.937  24.622  34.221  0.00  0.00      PROA
ATOM    244  O   MET P  16       1.769  25.059  33.379  0.00  0.00      PROA
ATOM    245  N   GLY P  17      -0.434  24.614  34.091  0.00  0.00      PROA
ATOM    246  HN  GLY P  17      -0.992  24.424  34.895  0.00  0.00      PROA
ATOM    247  CA  GLY P  17      -1.153  25.081  32.924  0.00  0.00      PROA
ATOM    248  HA1 GLY P  17      -2.188  24.776  32.976  0.00  0.00      PROA
ATOM    249  HA2 GLY P  17      -0.645  24.572  32.118  0.00  0.00      PROA
ATOM    250  C   GLY P  17      -1.052  26.494  32.564  0.00  0.00      PROA
ATOM    251  O   GLY P  17      -1.225  27.350  33.446  0.00  0.00      PROA
ATOM    252  N   SER P  18      -0.837  26.737  31.275  0.00  0.00      PROA
ATOM    253  HN  SER P  18      -0.904  26.002  30.604  0.00  0.00      PROA
ATOM    254  CA  SER P  18      -0.675  28.148  30.815  0.00  0.00      PROA
ATOM    255  HA  SER P  18      -1.517  28.704  31.202  0.00  0.00      PROA
ATOM    256  CB  SER P  18       0.752  28.691  31.003  0.00  0.00      PROA
ATOM    257  HB1 SER P  18       1.496  28.129  30.399  0.00  0.00      PROA
ATOM    258  HB2 SER P  18       1.031  28.617  32.076  0.00  0.00      PROA
ATOM    259  OG  SER P  18       0.778  30.065  30.761  0.00  0.00      PROA
ATOM    260  HG1 SER P  18       0.255  30.394  31.496  0.00  0.00      PROA
ATOM    261  C   SER P  18      -0.956  28.236  29.349  0.00  0.00      PROA
ATOM    262  O   SER P  18      -1.079  27.215  28.671  0.00  0.00      PROA
ATOM    263  N   GLY P  19      -1.203  29.438  28.776  0.00  0.00      PROA
ATOM    264  HN  GLY P  19      -1.076  30.307  29.249  0.00  0.00      PROA
ATOM    265  CA  GLY P  19      -1.807  29.560  27.515  0.00  0.00      PROA
ATOM    266  HA1 GLY P  19      -1.091  29.168  26.808  0.00  0.00      PROA
ATOM    267  HA2 GLY P  19      -2.050  30.604  27.381  0.00  0.00      PROA
ATOM    268  C   GLY P  19      -3.025  28.660  27.277  0.00  0.00      PROA
ATOM    269  O   GLY P  19      -3.961  28.660  28.127  0.00  0.00      PROA
ATOM    270  N   ASP P  20      -3.034  27.858  26.268  0.00  0.00      PROA
ATOM    271  HN  ASP P  20      -2.400  28.055  25.524  0.00  0.00      PROA
ATOM    272  CA  ASP P  20      -3.987  26.764  26.022  0.00  0.00      PROA
ATOM    273  HA  ASP P  20      -4.917  27.250  25.769  0.00  0.00      PROA
ATOM    274  CB  ASP P  20      -3.512  25.950  24.735  0.00  0.00      PROA
ATOM    275  HB1 ASP P  20      -4.047  25.013  24.467  0.00  0.00      PROA
ATOM    276  HB2 ASP P  20      -2.468  25.628  24.935  0.00  0.00      PROA
ATOM    277  CG  ASP P  20      -3.563  26.882  23.477  0.00  0.00      PROA
ATOM    278  OD1 ASP P  20      -4.610  27.603  23.281  0.00  0.00      PROA
ATOM    279  OD2 ASP P  20      -2.452  26.980  22.862  0.00  0.00      PROA
ATOM    280  C   ASP P  20      -4.173  25.801  27.168  0.00  0.00      PROA
ATOM    281  O   ASP P  20      -3.245  25.340  27.836  0.00  0.00      PROA
ATOM    282  N   TYR P  21      -5.460  25.423  27.396  0.00  0.00      PROA
ATOM    283  HN  TYR P  21      -6.170  25.862  26.851  0.00  0.00      PROA
ATOM    284  CA  TYR P  21      -5.906  24.533  28.477  0.00  0.00      PROA
ATOM    285  HA  TYR P  21      -5.255  24.683  29.326  0.00  0.00      PROA
ATOM    286  CB  TYR P  21      -7.267  24.901  28.886  0.00  0.00      PROA
ATOM    287  HB1 TYR P  21      -7.884  25.172  28.003  0.00  0.00      PROA
ATOM    288  HB2 TYR P  21      -7.255  25.882  29.408  0.00  0.00      PROA
ATOM    289  CG  TYR P  21      -7.966  23.966  29.851  0.00  0.00      PROA
ATOM    290  CD1 TYR P  21      -9.150  23.356  29.323  0.00  0.00      PROA
ATOM    291  HD1 TYR P  21      -9.381  23.424  28.270  0.00  0.00      PROA
ATOM    292  CE1 TYR P  21     -10.056  22.743  30.147  0.00  0.00      PROA
ATOM    293  HE1 TYR P  21     -10.992  22.394  29.738  0.00  0.00      PROA
ATOM    294  CZ  TYR P  21      -9.684  22.535  31.506  0.00  0.00      PROA
ATOM    295  OH  TYR P  21     -10.513  21.899  32.427  0.00  0.00      PROA
ATOM    296  HH  TYR P  21     -10.169  22.084  33.304  0.00  0.00      PROA
ATOM    297  CD2 TYR P  21      -7.658  23.762  31.176  0.00  0.00      PROA
ATOM    298  HD2 TYR P  21      -6.753  24.126  31.640  0.00  0.00      PROA
ATOM    299  CE2 TYR P  21      -8.540  23.040  31.991  0.00  0.00      PROA
ATOM    300  HE2 TYR P  21      -8.194  22.831  32.993  0.00  0.00      PROA
ATOM    301  C   TYR P  21      -5.721  23.078  28.181  0.00  0.00      PROA
```

ATOM	302	O	TYR	P	21	-5.807	22.764	26.972	0.00	0.00	PROA
ATOM	303	N	ASP	P	22	-5.385	22.205	29.141	0.00	0.00	PROA
ATOM	304	HN	ASP	P	22	-5.252	22.745	29.969	0.00	0.00	PROA
ATOM	305	CA	ASP	P	22	-5.170	20.763	28.977	0.00	0.00	PROA
ATOM	306	HA	ASP	P	22	-4.117	20.646	28.768	0.00	0.00	PROA
ATOM	307	CB	ASP	P	22	-5.592	20.016	30.310	0.00	0.00	PROA
ATOM	308	HB1	ASP	P	22	-5.510	18.908	30.275	0.00	0.00	PROA
ATOM	309	HB2	ASP	P	22	-6.612	20.334	30.617	0.00	0.00	PROA
ATOM	310	CG	ASP	P	22	-4.669	20.362	31.464	0.00	0.00	PROA
ATOM	311	OD1	ASP	P	22	-5.026	20.137	32.691	0.00	0.00	PROA
ATOM	312	OD2	ASP	P	22	-3.522	20.814	31.187	0.00	0.00	PROA
ATOM	313	C	ASP	P	22	-5.831	20.078	27.821	0.00	0.00	PROA
ATOM	314	O	ASP	P	22	-7.013	20.138	27.579	0.00	0.00	PROA
ATOM	315	N	SER	P	23	-5.062	19.190	27.175	0.00	0.00	PROA
ATOM	316	HN	SER	P	23	-4.079	19.165	27.343	0.00	0.00	PROA
ATOM	317	CA	SER	P	23	-5.399	18.324	26.037	0.00	0.00	PROA
ATOM	318	HA	SER	P	23	-6.014	18.903	25.365	0.00	0.00	PROA
ATOM	319	CB	SER	P	23	-4.096	17.890	25.248	0.00	0.00	PROA
ATOM	320	HB1	SER	P	23	-3.750	18.759	24.649	0.00	0.00	PROA
ATOM	321	HB2	SER	P	23	-4.234	17.116	24.462	0.00	0.00	PROA
ATOM	322	OG	SER	P	23	-2.968	17.452	26.049	0.00	0.00	PROA
ATOM	323	HG1	SER	P	23	-2.659	18.190	26.580	0.00	0.00	PROA
ATOM	324	C	SER	P	23	-6.060	16.978	26.377	0.00	0.00	PROA
ATOM	325	O	SER	P	23	-6.711	16.356	25.564	0.00	0.00	PROA
ATOM	326	N	MET	P	24	-5.703	16.504	27.612	0.00	0.00	PROA
ATOM	327	HN	MET	P	24	-4.998	16.990	28.124	0.00	0.00	PROA
ATOM	328	CA	MET	P	24	-6.431	15.447	28.277	0.00	0.00	PROA
ATOM	329	HA	MET	P	24	-5.931	15.450	29.234	0.00	0.00	PROA
ATOM	330	CB	MET	P	24	-7.911	15.773	28.576	0.00	0.00	PROA
ATOM	331	HB1	MET	P	24	-8.336	14.894	29.105	0.00	0.00	PROA
ATOM	332	HB2	MET	P	24	-8.482	15.848	27.626	0.00	0.00	PROA
ATOM	333	CG	MET	P	24	-8.267	16.994	29.406	0.00	0.00	PROA
ATOM	334	HG1	MET	P	24	-9.362	17.124	29.546	0.00	0.00	PROA
ATOM	335	HG2	MET	P	24	-7.884	17.859	28.823	0.00	0.00	PROA
ATOM	336	SD	MET	P	24	-7.496	16.894	31.122	0.00	0.00	PROA
ATOM	337	CE	MET	P	24	-8.558	18.270	31.712	0.00	0.00	PROA
ATOM	338	HE1	MET	P	24	-8.427	19.175	31.081	0.00	0.00	PROA
ATOM	339	HE2	MET	P	24	-8.355	18.494	32.781	0.00	0.00	PROA
ATOM	340	HE3	MET	P	24	-9.617	17.958	31.586	0.00	0.00	PROA
ATOM	341	C	MET	P	24	-6.174	14.040	27.743	0.00	0.00	PROA
ATOM	342	O	MET	P	24	-7.158	13.315	27.518	0.00	0.00	PROA
ATOM	343	N	LYS	P	25	-4.932	13.687	27.456	0.00	0.00	PROA
ATOM	344	HN	LYS	P	25	-4.201	14.343	27.629	0.00	0.00	PROA
ATOM	345	CA	LYS	P	25	-4.731	12.470	26.644	0.00	0.00	PROA
ATOM	346	HA	LYS	P	25	-5.668	12.004	26.376	0.00	0.00	PROA
ATOM	347	CB	LYS	P	25	-3.986	12.726	25.303	0.00	0.00	PROA
ATOM	348	HB1	LYS	P	25	-3.984	11.748	24.777	0.00	0.00	PROA
ATOM	349	HB2	LYS	P	25	-2.980	13.173	25.450	0.00	0.00	PROA
ATOM	350	CG	LYS	P	25	-4.687	13.741	24.455	0.00	0.00	PROA
ATOM	351	HG1	LYS	P	25	-4.309	14.753	24.717	0.00	0.00	PROA
ATOM	352	HG2	LYS	P	25	-5.744	13.922	24.747	0.00	0.00	PROA
ATOM	353	CD	LYS	P	25	-4.622	13.398	23.021	0.00	0.00	PROA
ATOM	354	HD1	LYS	P	25	-5.157	14.246	22.541	0.00	0.00	PROA
ATOM	355	HD2	LYS	P	25	-5.157	12.454	22.781	0.00	0.00	PROA
ATOM	356	CE	LYS	P	25	-3.198	13.367	22.474	0.00	0.00	PROA
ATOM	357	HE1	LYS	P	25	-2.626	12.530	22.930	0.00	0.00	PROA
ATOM	358	HE2	LYS	P	25	-2.657	14.289	22.775	0.00	0.00	PROA
ATOM	359	NZ	LYS	P	25	-3.197	13.189	21.017	0.00	0.00	PROA
ATOM	360	HZ1	LYS	P	25	-3.556	12.279	20.661	0.00	0.00	PROA
ATOM	361	HZ2	LYS	P	25	-2.205	13.316	20.733	0.00	0.00	PROA
ATOM	362	HZ3	LYS	P	25	-3.702	13.934	20.496	0.00	0.00	PROA

ATOM	363	C	LYS	P	25	-3.884	11.497	27.386	0.00	0.00	PROA
ATOM	364	O	LYS	P	25	-3.430	10.396	26.889	0.00	0.00	PROA
ATOM	365	N	GLU	P	26	-3.445	11.826	28.626	0.00	0.00	PROA
ATOM	366	HN	GLU	P	26	-3.631	12.697	29.076	0.00	0.00	PROA
ATOM	367	CA	GLU	P	26	-2.541	10.987	29.430	0.00	0.00	PROA
ATOM	368	HA	GLU	P	26	-1.702	10.715	28.806	0.00	0.00	PROA
ATOM	369	CB	GLU	P	26	-1.853	11.699	30.642	0.00	0.00	PROA
ATOM	370	HB1	GLU	P	26	-1.328	11.008	31.336	0.00	0.00	PROA
ATOM	371	HB2	GLU	P	26	-2.579	12.228	31.295	0.00	0.00	PROA
ATOM	372	CG	GLU	P	26	-0.767	12.680	30.116	0.00	0.00	PROA
ATOM	373	HG1	GLU	P	26	-1.268	13.372	29.406	0.00	0.00	PROA
ATOM	374	HG2	GLU	P	26	0.074	12.079	29.708	0.00	0.00	PROA
ATOM	375	CD	GLU	P	26	-0.166	13.396	31.325	0.00	0.00	PROA
ATOM	376	OE1	GLU	P	26	0.604	12.699	32.011	0.00	0.00	PROA
ATOM	377	OE2	GLU	P	26	-0.334	14.606	31.483	0.00	0.00	PROA
ATOM	378	C	GLU	P	26	-3.055	9.616	29.819	0.00	0.00	PROA
ATOM	379	O	GLU	P	26	-2.325	8.638	29.638	0.00	0.00	PROA
ATOM	380	N	PRO	P	27	-4.314	9.452	30.236	0.00	0.00	PROA
ATOM	381	CD	PRO	P	27	-5.231	10.565	30.733	0.00	0.00	PROA
ATOM	382	HD1	PRO	P	27	-5.947	10.694	29.893	0.00	0.00	PROA
ATOM	383	HD2	PRO	P	27	-4.719	11.505	31.030	0.00	0.00	PROA
ATOM	384	CA	PRO	P	27	-4.822	8.131	30.617	0.00	0.00	PROA
ATOM	385	HA	PRO	P	27	-4.109	7.508	31.136	0.00	0.00	PROA
ATOM	386	CB	PRO	P	27	-6.057	8.456	31.487	0.00	0.00	PROA
ATOM	387	HB1	PRO	P	27	-6.094	7.799	32.382	0.00	0.00	PROA
ATOM	388	HB2	PRO	P	27	-6.971	8.298	30.875	0.00	0.00	PROA
ATOM	389	CG	PRO	P	27	-5.949	9.948	31.904	0.00	0.00	PROA
ATOM	390	HG1	PRO	P	27	-6.886	10.460	32.210	0.00	0.00	PROA
ATOM	391	HG2	PRO	P	27	-5.260	9.983	32.775	0.00	0.00	PROA
ATOM	392	C	PRO	P	27	-5.221	7.249	29.489	0.00	0.00	PROA
ATOM	393	O	PRO	P	27	-5.543	6.066	29.675	0.00	0.00	PROA
ATOM	394	N	CYS	P	28	-5.309	7.752	28.204	0.00	0.00	PROA
ATOM	395	HN	CYS	P	28	-4.939	8.672	28.101	0.00	0.00	PROA
ATOM	396	CA	CYS	P	28	-5.935	7.105	27.041	0.00	0.00	PROA
ATOM	397	HA	CYS	P	28	-6.899	6.773	27.396	0.00	0.00	PROA
ATOM	398	CB	CYS	P	28	-6.259	8.122	25.887	0.00	0.00	PROA
ATOM	399	HB1	CYS	P	28	-6.790	7.619	25.051	0.00	0.00	PROA
ATOM	400	HB2	CYS	P	28	-5.278	8.568	25.617	0.00	0.00	PROA
ATOM	401	SG	CYS	P	28	-7.306	9.487	26.501	0.00	0.00	PROA
ATOM	402	C	CYS	P	28	-5.215	5.879	26.506	0.00	0.00	PROA
ATOM	403	O	CYS	P	28	-4.006	5.643	26.753	0.00	0.00	PROA
ATOM	404	N	PHE	P	29	-6.016	4.984	25.829	0.00	0.00	PROA
ATOM	405	HN	PHE	P	29	-6.944	5.215	25.545	0.00	0.00	PROA
ATOM	406	CA	PHE	P	29	-5.533	3.757	25.176	0.00	0.00	PROA
ATOM	407	HA	PHE	P	29	-4.566	4.046	24.792	0.00	0.00	PROA
ATOM	408	CB	PHE	P	29	-5.412	2.570	26.160	0.00	0.00	PROA
ATOM	409	HB1	PHE	P	29	-6.425	2.251	26.487	0.00	0.00	PROA
ATOM	410	HB2	PHE	P	29	-4.856	2.952	27.043	0.00	0.00	PROA
ATOM	411	CG	PHE	P	29	-4.638	1.365	25.687	0.00	0.00	PROA
ATOM	412	CD1	PHE	P	29	-3.353	1.557	25.044	0.00	0.00	PROA
ATOM	413	HD1	PHE	P	29	-3.084	2.593	24.901	0.00	0.00	PROA
ATOM	414	CE1	PHE	P	29	-2.504	0.460	24.855	0.00	0.00	PROA
ATOM	415	HE1	PHE	P	29	-1.530	0.621	24.417	0.00	0.00	PROA
ATOM	416	CZ	PHE	P	29	-2.980	-0.829	25.034	0.00	0.00	PROA
ATOM	417	HZ	PHE	P	29	-2.235	-1.602	24.913	0.00	0.00	PROA
ATOM	418	CD2	PHE	P	29	-5.049	0.061	25.997	0.00	0.00	PROA
ATOM	419	HD2	PHE	P	29	-5.932	-0.179	26.571	0.00	0.00	PROA
ATOM	420	CE2	PHE	P	29	-4.214	-1.034	25.619	0.00	0.00	PROA
ATOM	421	HE2	PHE	P	29	-4.615	-2.023	25.783	0.00	0.00	PROA
ATOM	422	C	PHE	P	29	-6.523	3.476	24.074	0.00	0.00	PROA
ATOM	423	O	PHE	P	29	-7.665	3.993	24.100	0.00	0.00	PROA

ATOM	424	N	ARG	P	30	-6.155	2.648	23.148	0.00	0.00	PROA
ATOM	425	HN	ARG	P	30	-5.340	2.074	23.153	0.00	0.00	PROA
ATOM	426	CA	ARG	P	30	-6.880	2.223	22.022	0.00	0.00	PROA
ATOM	427	HA	ARG	P	30	-7.742	2.868	21.939	0.00	0.00	PROA
ATOM	428	CB	ARG	P	30	-6.202	2.320	20.652	0.00	0.00	PROA
ATOM	429	HB1	ARG	P	30	-6.821	1.964	19.800	0.00	0.00	PROA
ATOM	430	HB2	ARG	P	30	-5.279	1.701	20.639	0.00	0.00	PROA
ATOM	431	CG	ARG	P	30	-5.745	3.727	20.361	0.00	0.00	PROA
ATOM	432	HG1	ARG	P	30	-5.105	4.133	21.174	0.00	0.00	PROA
ATOM	433	HG2	ARG	P	30	-6.664	4.331	20.203	0.00	0.00	PROA
ATOM	434	CD	ARG	P	30	-4.920	3.853	18.969	0.00	0.00	PROA
ATOM	435	HD1	ARG	P	30	-5.589	3.794	18.084	0.00	0.00	PROA
ATOM	436	HD2	ARG	P	30	-4.137	3.071	19.067	0.00	0.00	PROA
ATOM	437	NE	ARG	P	30	-4.436	5.319	19.009	0.00	0.00	PROA
ATOM	438	HE	ARG	P	30	-4.609	5.873	19.824	0.00	0.00	PROA
ATOM	439	CZ	ARG	P	30	-3.889	5.834	17.937	0.00	0.00	PROA
ATOM	440	NH1	ARG	P	30	-3.960	5.383	16.718	0.00	0.00	PROA
ATOM	441	HH11	ARG	P	30	-4.209	4.414	16.742	0.00	0.00	PROA
ATOM	442	HH12	ARG	P	30	-3.344	5.715	16.004	0.00	0.00	PROA
ATOM	443	NH2	ARG	P	30	-3.239	6.986	18.118	0.00	0.00	PROA
ATOM	444	HH21	ARG	P	30	-2.676	7.321	17.363	0.00	0.00	PROA
ATOM	445	HH22	ARG	P	30	-3.438	7.485	18.961	0.00	0.00	PROA
ATOM	446	C	ARG	P	30	-7.477	0.820	22.194	0.00	0.00	PROA
ATOM	447	O	ARG	P	30	-6.747	-0.202	22.011	0.00	0.00	PROA
ATOM	448	N	GLU	P	31	-8.762	0.667	22.522	0.00	0.00	PROA
ATOM	449	HN	GLU	P	31	-9.248	1.487	22.815	0.00	0.00	PROA
ATOM	450	CA	GLU	P	31	-9.467	-0.573	22.675	0.00	0.00	PROA
ATOM	451	HA	GLU	P	31	-8.804	-1.107	23.340	0.00	0.00	PROA
ATOM	452	CB	GLU	P	31	-10.736	-0.460	23.412	0.00	0.00	PROA
ATOM	453	HB1	GLU	P	31	-11.306	-1.413	23.387	0.00	0.00	PROA
ATOM	454	HB2	GLU	P	31	-11.343	0.326	22.914	0.00	0.00	PROA
ATOM	455	CG	GLU	P	31	-10.493	-0.282	24.922	0.00	0.00	PROA
ATOM	456	HG1	GLU	P	31	-9.962	0.652	25.202	0.00	0.00	PROA
ATOM	457	HG2	GLU	P	31	-9.761	-1.088	25.145	0.00	0.00	PROA
ATOM	458	CD	GLU	P	31	-11.732	-0.233	25.766	0.00	0.00	PROA
ATOM	459	OE1	GLU	P	31	-12.097	0.914	26.204	0.00	0.00	PROA
ATOM	460	OE2	GLU	P	31	-12.151	-1.359	26.213	0.00	0.00	PROA
ATOM	461	C	GLU	P	31	-9.619	-1.416	21.388	0.00	0.00	PROA
ATOM	462	O	GLU	P	31	-10.331	-1.087	20.450	0.00	0.00	PROA
ATOM	463	N	GLU	P	32	-8.982	-2.644	21.420	0.00	0.00	PROA
ATOM	464	HN	GLU	P	32	-8.287	-2.799	22.117	0.00	0.00	PROA
ATOM	465	CA	GLU	P	32	-9.070	-3.723	20.428	0.00	0.00	PROA
ATOM	466	HA	GLU	P	32	-8.908	-3.292	19.451	0.00	0.00	PROA
ATOM	467	CB	GLU	P	32	-7.893	-4.775	20.585	0.00	0.00	PROA
ATOM	468	HB1	GLU	P	32	-6.964	-4.171	20.660	0.00	0.00	PROA
ATOM	469	HB2	GLU	P	32	-7.701	-5.445	19.720	0.00	0.00	PROA
ATOM	470	CG	GLU	P	32	-8.042	-5.664	21.831	0.00	0.00	PROA
ATOM	471	HG1	GLU	P	32	-9.046	-6.133	21.907	0.00	0.00	PROA
ATOM	472	HG2	GLU	P	32	-7.916	-5.009	22.720	0.00	0.00	PROA
ATOM	473	CD	GLU	P	32	-7.013	-6.779	21.749	0.00	0.00	PROA
ATOM	474	OE1	GLU	P	32	-7.103	-7.566	20.770	0.00	0.00	PROA
ATOM	475	OE2	GLU	P	32	-6.159	-6.956	22.686	0.00	0.00	PROA
ATOM	476	C	GLU	P	32	-10.434	-4.418	20.377	0.00	0.00	PROA
ATOM	477	O	GLU	P	32	-11.236	-4.285	21.339	0.00	0.00	PROA
ATOM	478	N	ASN	P	33	-10.824	-5.042	19.347	0.00	0.00	PROA
ATOM	479	HN	ASN	P	33	-10.299	-5.223	18.519	0.00	0.00	PROA
ATOM	480	CA	ASN	P	33	-12.145	-5.672	19.289	0.00	0.00	PROA
ATOM	481	HA	ASN	P	33	-12.864	-5.378	20.039	0.00	0.00	PROA
ATOM	482	CB	ASN	P	33	-12.861	-5.303	17.954	0.00	0.00	PROA
ATOM	483	HB1	ASN	P	33	-13.922	-5.630	17.923	0.00	0.00	PROA
ATOM	484	HB2	ASN	P	33	-12.284	-5.868	17.191	0.00	0.00	PROA

ATOM	485	CG	ASN	P	33	-12.835	-3.803	17.730	0.00	0.00	PROA
ATOM	486	OD1	ASN	P	33	-13.720	-3.090	18.238	0.00	0.00	PROA
ATOM	487	ND2	ASN	P	33	-11.881	-3.386	16.885	0.00	0.00	PROA
ATOM	488	HD21	ASN	P	33	-12.088	-2.543	16.390	0.00	0.00	PROA
ATOM	489	HD22	ASN	P	33	-11.138	-4.001	16.616	0.00	0.00	PROA
ATOM	490	C	ASN	P	33	-11.996	-7.169	19.431	0.00	0.00	PROA
ATOM	491	O	ASN	P	33	-10.872	-7.746	19.431	0.00	0.00	PROA
ATOM	492	N	ALA	P	34	-13.130	-7.851	19.711	0.00	0.00	PROA
ATOM	493	HN	ALA	P	34	-13.971	-7.333	19.847	0.00	0.00	PROA
ATOM	494	CA	ALA	P	34	-13.103	-9.322	19.660	0.00	0.00	PROA
ATOM	495	HA	ALA	P	34	-12.121	-9.768	19.704	0.00	0.00	PROA
ATOM	496	CB	ALA	P	34	-13.737	-9.941	20.926	0.00	0.00	PROA
ATOM	497	HB1	ALA	P	34	-13.003	-9.680	21.718	0.00	0.00	PROA
ATOM	498	HB2	ALA	P	34	-13.561	-11.023	20.745	0.00	0.00	PROA
ATOM	499	HB3	ALA	P	34	-14.825	-9.757	21.054	0.00	0.00	PROA
ATOM	500	C	ALA	P	34	-13.705	-9.929	18.364	0.00	0.00	PROA
ATOM	501	O	ALA	P	34	-13.293	-9.588	17.274	0.00	0.00	PROA
ATOM	502	N	ASN	P	35	-14.700	-10.767	18.475	0.00	0.00	PROA
ATOM	503	HN	ASN	P	35	-15.192	-10.891	19.333	0.00	0.00	PROA
ATOM	504	CA	ASN	P	35	-15.034	-11.809	17.512	0.00	0.00	PROA
ATOM	505	HA	ASN	P	35	-14.209	-12.501	17.593	0.00	0.00	PROA
ATOM	506	CB	ASN	P	35	-16.404	-12.448	17.977	0.00	0.00	PROA
ATOM	507	HB1	ASN	P	35	-16.811	-13.209	17.277	0.00	0.00	PROA
ATOM	508	HB2	ASN	P	35	-17.119	-11.602	18.060	0.00	0.00	PROA
ATOM	509	CG	ASN	P	35	-16.089	-13.132	19.276	0.00	0.00	PROA
ATOM	510	OD1	ASN	P	35	-15.059	-13.829	19.237	0.00	0.00	PROA
ATOM	511	ND2	ASN	P	35	-16.796	-12.982	20.382	0.00	0.00	PROA
ATOM	512	HD21	ASN	P	35	-16.329	-13.321	21.198	0.00	0.00	PROA
ATOM	513	HD22	ASN	P	35	-17.647	-12.458	20.367	0.00	0.00	PROA
ATOM	514	C	ASN	P	35	-15.151	-11.542	16.033	0.00	0.00	PROA
ATOM	515	O	ASN	P	35	-14.940	-12.389	15.186	0.00	0.00	PROA
ATOM	516	N	PHE	P	36	-15.647	-10.339	15.649	0.00	0.00	PROA
ATOM	517	HN	PHE	P	36	-15.977	-9.733	16.369	0.00	0.00	PROA
ATOM	518	CA	PHE	P	36	-15.628	-9.885	14.232	0.00	0.00	PROA
ATOM	519	HA	PHE	P	36	-16.255	-10.540	13.646	0.00	0.00	PROA
ATOM	520	CB	PHE	P	36	-16.201	-8.425	14.239	0.00	0.00	PROA
ATOM	521	HB1	PHE	P	36	-15.519	-7.750	14.799	0.00	0.00	PROA
ATOM	522	HB2	PHE	P	36	-17.167	-8.346	14.780	0.00	0.00	PROA
ATOM	523	CG	PHE	P	36	-16.477	-7.922	12.797	0.00	0.00	PROA
ATOM	524	CD1	PHE	P	36	-17.769	-8.228	12.277	0.00	0.00	PROA
ATOM	525	HD1	PHE	P	36	-18.385	-8.682	13.039	0.00	0.00	PROA
ATOM	526	CE1	PHE	P	36	-18.178	-7.693	11.065	0.00	0.00	PROA
ATOM	527	HE1	PHE	P	36	-19.197	-7.731	10.710	0.00	0.00	PROA
ATOM	528	CZ	PHE	P	36	-17.278	-6.977	10.284	0.00	0.00	PROA
ATOM	529	HZ	PHE	P	36	-17.623	-6.434	9.416	0.00	0.00	PROA
ATOM	530	CD2	PHE	P	36	-15.533	-7.243	12.039	0.00	0.00	PROA
ATOM	531	HD2	PHE	P	36	-14.604	-6.850	12.423	0.00	0.00	PROA
ATOM	532	CE2	PHE	P	36	-15.984	-6.764	10.753	0.00	0.00	PROA
ATOM	533	HE2	PHE	P	36	-15.277	-6.231	10.133	0.00	0.00	PROA
ATOM	534	C	PHE	P	36	-14.171	-9.866	13.709	0.00	0.00	PROA
ATOM	535	O	PHE	P	36	-13.767	-10.336	12.669	0.00	0.00	PROA
ATOM	536	N	ASN	P	37	-13.255	-9.339	14.575	0.00	0.00	PROA
ATOM	537	HN	ASN	P	37	-13.558	-9.138	15.504	0.00	0.00	PROA
ATOM	538	CA	ASN	P	37	-11.847	-9.128	14.296	0.00	0.00	PROA
ATOM	539	HA	ASN	P	37	-11.817	-9.038	13.220	0.00	0.00	PROA
ATOM	540	CB	ASN	P	37	-11.337	-7.795	15.030	0.00	0.00	PROA
ATOM	541	HB1	ASN	P	37	-10.243	-7.725	14.846	0.00	0.00	PROA
ATOM	542	HB2	ASN	P	37	-11.496	-7.757	16.129	0.00	0.00	PROA
ATOM	543	CG	ASN	P	37	-12.139	-6.568	14.476	0.00	0.00	PROA
ATOM	544	OD1	ASN	P	37	-13.313	-6.297	14.798	0.00	0.00	PROA
ATOM	545	ND2	ASN	P	37	-11.455	-5.722	13.625	0.00	0.00	PROA

```
ATOM    546 HD21 ASN P  37     -11.811  -4.797  13.491  0.00  0.00      PROA
ATOM    547 HD22 ASN P  37     -10.525  -5.989  13.374  0.00  0.00      PROA
ATOM    548  C   ASN P  37     -10.993 -10.281  14.658  0.00  0.00      PROA
ATOM    549  O   ASN P  37      -9.916 -10.288  14.178  0.00  0.00      PROA
ATOM    550  N   LYS P  38     -11.580 -11.285  15.339  0.00  0.00      PROA
ATOM    551  HN  LYS P  38     -12.412 -11.162  15.874  0.00  0.00      PROA
ATOM    552  CA  LYS P  38     -10.722 -12.383  15.830  0.00  0.00      PROA
ATOM    553  HA  LYS P  38      -9.691 -12.269  15.527  0.00  0.00      PROA
ATOM    554  CB  LYS P  38     -10.656 -12.453  17.332  0.00  0.00      PROA
ATOM    555  HB1 LYS P  38     -10.039 -13.346  17.568  0.00  0.00      PROA
ATOM    556  HB2 LYS P  38     -11.660 -12.578  17.792  0.00  0.00      PROA
ATOM    557  CG  LYS P  38     -10.014 -11.252  18.069  0.00  0.00      PROA
ATOM    558  HG1 LYS P  38      -9.977 -11.411  19.168  0.00  0.00      PROA
ATOM    559  HG2 LYS P  38     -10.718 -10.403  17.935  0.00  0.00      PROA
ATOM    560  CD  LYS P  38      -8.587 -11.013  17.727  0.00  0.00      PROA
ATOM    561  HD1 LYS P  38      -8.464 -10.660  16.681  0.00  0.00      PROA
ATOM    562  HD2 LYS P  38      -8.022 -11.969  17.756  0.00  0.00      PROA
ATOM    563  CE  LYS P  38      -7.724 -10.153  18.696  0.00  0.00      PROA
ATOM    564  HE1 LYS P  38      -6.632 -10.087  18.505  0.00  0.00      PROA
ATOM    565  HE2 LYS P  38      -7.837 -10.607  19.704  0.00  0.00      PROA
ATOM    566  NZ  LYS P  38      -8.281  -8.728  18.695  0.00  0.00      PROA
ATOM    567  HZ1 LYS P  38      -7.959  -8.266  19.570  0.00  0.00      PROA
ATOM    568  HZ2 LYS P  38      -9.319  -8.671  18.715  0.00  0.00      PROA
ATOM    569  HZ3 LYS P  38      -7.812  -8.334  17.855  0.00  0.00      PROA
ATOM    570  C   LYS P  38     -11.197 -13.713  15.151  0.00  0.00      PROA
ATOM    571  O   LYS P  38     -10.575 -14.771  15.271  0.00  0.00      PROA
ATOM    572  N   ILE P  39     -12.331 -13.710  14.439  0.00  0.00      PROA
ATOM    573  HN  ILE P  39     -12.883 -12.890  14.308  0.00  0.00      PROA
ATOM    574  CA  ILE P  39     -12.860 -14.901  13.753  0.00  0.00      PROA
ATOM    575  HA  ILE P  39     -12.107 -15.671  13.673  0.00  0.00      PROA
ATOM    576  CB  ILE P  39     -14.141 -15.527  14.450  0.00  0.00      PROA
ATOM    577  HB  ILE P  39     -15.027 -14.883  14.263  0.00  0.00      PROA
ATOM    578  CG2 ILE P  39     -14.492 -16.921  13.820  0.00  0.00      PROA
ATOM    579 HG21 ILE P  39     -14.541 -16.868  12.712  0.00  0.00      PROA
ATOM    580 HG22 ILE P  39     -15.431 -17.378  14.198  0.00  0.00      PROA
ATOM    581 HG23 ILE P  39     -13.584 -17.516  14.060  0.00  0.00      PROA
ATOM    582  CG1 ILE P  39     -13.952 -15.641  16.018  0.00  0.00      PROA
ATOM    583 HG11 ILE P  39     -13.987 -14.643  16.506  0.00  0.00      PROA
ATOM    584 HG12 ILE P  39     -12.931 -16.051  16.177  0.00  0.00      PROA
ATOM    585  CD  ILE P  39     -14.894 -16.492  16.865  0.00  0.00      PROA
ATOM    586  HD1 ILE P  39     -15.049 -17.512  16.452  0.00  0.00      PROA
ATOM    587  HD2 ILE P  39     -15.861 -15.966  17.015  0.00  0.00      PROA
ATOM    588  HD3 ILE P  39     -14.540 -16.571  17.915  0.00  0.00      PROA
ATOM    589  C   ILE P  39     -13.273 -14.523  12.359  0.00  0.00      PROA
ATOM    590  O   ILE P  39     -12.793 -15.177  11.407  0.00  0.00      PROA
ATOM    591  N   PHE P  40     -14.170 -13.545  12.253  0.00  0.00      PROA
ATOM    592  HN  PHE P  40     -14.463 -12.945  12.994  0.00  0.00      PROA
ATOM    593  CA  PHE P  40     -14.734 -13.206  10.922  0.00  0.00      PROA
ATOM    594  HA  PHE P  40     -15.174 -14.077  10.458  0.00  0.00      PROA
ATOM    595  CB  PHE P  40     -16.042 -12.320  11.134  0.00  0.00      PROA
ATOM    596  HB1 PHE P  40     -15.764 -11.336  11.569  0.00  0.00      PROA
ATOM    597  HB2 PHE P  40     -16.769 -12.861  11.778  0.00  0.00      PROA
ATOM    598  CG  PHE P  40     -16.667 -11.891   9.808  0.00  0.00      PROA
ATOM    599  CD1 PHE P  40     -16.323 -10.665   9.143  0.00  0.00      PROA
ATOM    600  HD1 PHE P  40     -15.509 -10.064   9.522  0.00  0.00      PROA
ATOM    601  CE1 PHE P  40     -16.826 -10.333   7.918  0.00  0.00      PROA
ATOM    602  HE1 PHE P  40     -16.562  -9.392   7.459  0.00  0.00      PROA
ATOM    603  CZ  PHE P  40     -17.631 -11.178   7.256  0.00  0.00      PROA
ATOM    604  HZ  PHE P  40     -17.977 -10.828   6.295  0.00  0.00      PROA
ATOM    605  CD2 PHE P  40     -17.546 -12.744   9.156  0.00  0.00      PROA
ATOM    606  HD2 PHE P  40     -17.896 -13.699   9.520  0.00  0.00      PROA
```

```
ATOM    607  CE2 PHE P  40     -17.995 -12.362   7.839  0.00  0.00      PROA
ATOM    608  HE2 PHE P  40     -18.600 -13.096   7.327  0.00  0.00      PROA
ATOM    609  C   PHE P  40     -13.716 -12.717   9.843  0.00  0.00      PROA
ATOM    610  O   PHE P  40     -13.487 -13.333   8.792  0.00  0.00      PROA
ATOM    611  N   LEU P  41     -12.968 -11.640  10.139  0.00  0.00      PROA
ATOM    612  HN  LEU P  41     -13.129 -11.108  10.966  0.00  0.00      PROA
ATOM    613  CA  LEU P  41     -11.886 -11.129   9.349  0.00  0.00      PROA
ATOM    614  HA  LEU P  41     -12.258 -10.946   8.352  0.00  0.00      PROA
ATOM    615  CB  LEU P  41     -11.426  -9.716   9.832  0.00  0.00      PROA
ATOM    616  HB1 LEU P  41     -10.610  -9.387   9.154  0.00  0.00      PROA
ATOM    617  HB2 LEU P  41     -11.219  -9.712  10.924  0.00  0.00      PROA
ATOM    618  CG  LEU P  41     -12.428  -8.553   9.542  0.00  0.00      PROA
ATOM    619  HG  LEU P  41     -13.403  -8.794  10.017  0.00  0.00      PROA
ATOM    620  CD1 LEU P  41     -11.956  -7.226  10.129  0.00  0.00      PROA
ATOM    621 HD11 LEU P  41     -12.162  -7.246  11.220  0.00  0.00      PROA
ATOM    622 HD12 LEU P  41     -12.414  -6.277   9.778  0.00  0.00      PROA
ATOM    623 HD13 LEU P  41     -10.857  -7.063  10.121  0.00  0.00      PROA
ATOM    624  CD2 LEU P  41     -12.695  -8.251   8.052  0.00  0.00      PROA
ATOM    625 HD21 LEU P  41     -13.718  -7.821   7.989  0.00  0.00      PROA
ATOM    626 HD22 LEU P  41     -12.580  -9.206   7.496  0.00  0.00      PROA
ATOM    627 HD23 LEU P  41     -11.954  -7.505   7.693  0.00  0.00      PROA
ATOM    628  C   LEU P  41     -10.789 -12.088   9.135  0.00  0.00      PROA
ATOM    629  O   LEU P  41     -10.385 -12.163   8.002  0.00  0.00      PROA
ATOM    630  N   PRO P  42     -10.267 -12.894  10.047  0.00  0.00      PROA
ATOM    631  CD  PRO P  42     -10.185 -12.552  11.481  0.00  0.00      PROA
ATOM    632  HD1 PRO P  42     -11.031 -12.888  12.118  0.00  0.00      PROA
ATOM    633  HD2 PRO P  42     -10.179 -11.447  11.595  0.00  0.00      PROA
ATOM    634  CA  PRO P  42      -9.118 -13.786   9.650  0.00  0.00      PROA
ATOM    635  HA  PRO P  42      -8.417 -13.283   9.000  0.00  0.00      PROA
ATOM    636  CB  PRO P  42      -8.614 -14.231  11.070  0.00  0.00      PROA
ATOM    637  HB1 PRO P  42      -7.529 -14.436  11.200  0.00  0.00      PROA
ATOM    638  HB2 PRO P  42      -9.155 -15.153  11.373  0.00  0.00      PROA
ATOM    639  CG  PRO P  42      -8.828 -13.028  11.976  0.00  0.00      PROA
ATOM    640  HG1 PRO P  42      -8.853 -13.291  13.055  0.00  0.00      PROA
ATOM    641  HG2 PRO P  42      -8.100 -12.244  11.677  0.00  0.00      PROA
ATOM    642  C   PRO P  42      -9.546 -14.934   8.742  0.00  0.00      PROA
ATOM    643  O   PRO P  42      -8.746 -15.385   7.863  0.00  0.00      PROA
ATOM    644  N   THR P  43     -10.769 -15.438   8.874  0.00  0.00      PROA
ATOM    645  HN  THR P  43     -11.368 -15.100   9.596  0.00  0.00      PROA
ATOM    646  CA  THR P  43     -11.410 -16.400   7.925  0.00  0.00      PROA
ATOM    647  HA  THR P  43     -10.921 -17.353   7.790  0.00  0.00      PROA
ATOM    648  CB  THR P  43     -12.788 -16.792   8.523  0.00  0.00      PROA
ATOM    649  HB  THR P  43     -13.444 -15.895   8.552  0.00  0.00      PROA
ATOM    650  OG1 THR P  43     -12.765 -17.405   9.854  0.00  0.00      PROA
ATOM    651  HG1 THR P  43     -12.551 -16.707  10.477  0.00  0.00      PROA
ATOM    652  CG2 THR P  43     -13.531 -17.769   7.647  0.00  0.00      PROA
ATOM    653 HG21 THR P  43     -13.695 -17.350   6.631  0.00  0.00      PROA
ATOM    654 HG22 THR P  43     -14.512 -18.044   8.089  0.00  0.00      PROA
ATOM    655 HG23 THR P  43     -12.936 -18.705   7.590  0.00  0.00      PROA
ATOM    656  C   THR P  43     -11.527 -15.899   6.478  0.00  0.00      PROA
ATOM    657  O   THR P  43     -11.186 -16.551   5.538  0.00  0.00      PROA
ATOM    658  N   ILE P  44     -11.993 -14.590   6.366  0.00  0.00      PROA
ATOM    659  HN  ILE P  44     -12.448 -14.277   7.196  0.00  0.00      PROA
ATOM    660  CA  ILE P  44     -12.229 -13.714   5.254  0.00  0.00      PROA
ATOM    661  HA  ILE P  44     -12.775 -14.289   4.521  0.00  0.00      PROA
ATOM    662  CB  ILE P  44     -12.968 -12.470   5.647  0.00  0.00      PROA
ATOM    663  HB  ILE P  44     -12.681 -12.026   6.624  0.00  0.00      PROA
ATOM    664  CG2 ILE P  44     -12.724 -11.234   4.786  0.00  0.00      PROA
ATOM    665 HG21 ILE P  44     -11.619 -11.119   4.754  0.00  0.00      PROA
ATOM    666 HG22 ILE P  44     -13.247 -10.476   5.407  0.00  0.00      PROA
ATOM    667 HG23 ILE P  44     -13.161 -11.272   3.766  0.00  0.00      PROA
```

```
ATOM    668  CG1 ILE P  44     -14.547 -12.611   5.759  0.00  0.00      PROA
ATOM    669 HG11 ILE P  44     -14.875 -11.580   6.014  0.00  0.00      PROA
ATOM    670 HG12 ILE P  44     -14.864 -13.197   6.648  0.00  0.00      PROA
ATOM    671  CD  ILE P  44     -15.234 -13.243   4.505  0.00  0.00      PROA
ATOM    672  HD1 ILE P  44     -16.327 -13.079   4.621  0.00  0.00      PROA
ATOM    673  HD2 ILE P  44     -15.141 -14.347   4.589  0.00  0.00      PROA
ATOM    674  HD3 ILE P  44     -14.788 -12.817   3.581  0.00  0.00      PROA
ATOM    675  C   ILE P  44     -10.911 -13.444   4.538  0.00  0.00      PROA
ATOM    676  O   ILE P  44     -10.722 -13.662   3.357  0.00  0.00      PROA
ATOM    677  N   TYR P  45      -9.915 -13.057   5.300  0.00  0.00      PROA
ATOM    678  HN  TYR P  45     -10.124 -13.009   6.273  0.00  0.00      PROA
ATOM    679  CA  TYR P  45      -8.481 -12.920   4.895  0.00  0.00      PROA
ATOM    680  HA  TYR P  45      -8.495 -12.160   4.128  0.00  0.00      PROA
ATOM    681  CB  TYR P  45      -7.563 -12.277   6.076  0.00  0.00      PROA
ATOM    682  HB1 TYR P  45      -6.509 -12.284   5.725  0.00  0.00      PROA
ATOM    683  HB2 TYR P  45      -7.824 -12.927   6.939  0.00  0.00      PROA
ATOM    684  CG  TYR P  45      -7.780 -10.875   6.390  0.00  0.00      PROA
ATOM    685  CD1 TYR P  45      -8.522  -9.920   5.688  0.00  0.00      PROA
ATOM    686  HD1 TYR P  45      -9.080 -10.244   4.823  0.00  0.00      PROA
ATOM    687  CE1 TYR P  45      -8.633  -8.569   6.071  0.00  0.00      PROA
ATOM    688  HE1 TYR P  45      -9.240  -7.935   5.442  0.00  0.00      PROA
ATOM    689  CZ  TYR P  45      -7.911  -8.155   7.212  0.00  0.00      PROA
ATOM    690  OH  TYR P  45      -7.916  -6.823   7.677  0.00  0.00      PROA
ATOM    691  HH  TYR P  45      -7.069  -6.717   8.115  0.00  0.00      PROA
ATOM    692  CD2 TYR P  45      -7.096 -10.438   7.531  0.00  0.00      PROA
ATOM    693  HD2 TYR P  45      -6.620 -11.197   8.134  0.00  0.00      PROA
ATOM    694  CE2 TYR P  45      -7.104  -9.070   7.858  0.00  0.00      PROA
ATOM    695  HE2 TYR P  45      -6.631  -8.699   8.756  0.00  0.00      PROA
ATOM    696  C   TYR P  45      -7.903 -14.198   4.365  0.00  0.00      PROA
ATOM    697  O   TYR P  45      -7.180 -14.186   3.372  0.00  0.00      PROA
ATOM    698  N   SER P  46      -8.209 -15.338   5.003  0.00  0.00      PROA
ATOM    699  HN  SER P  46      -8.816 -15.240   5.788  0.00  0.00      PROA
ATOM    700  CA  SER P  46      -7.943 -16.698   4.586  0.00  0.00      PROA
ATOM    701  HA  SER P  46      -6.884 -16.600   4.399  0.00  0.00      PROA
ATOM    702  CB  SER P  46      -8.192 -17.871   5.574  0.00  0.00      PROA
ATOM    703  HB1 SER P  46      -7.779 -18.804   5.133  0.00  0.00      PROA
ATOM    704  HB2 SER P  46      -9.244 -18.063   5.874  0.00  0.00      PROA
ATOM    705  OG  SER P  46      -7.462 -17.617   6.816  0.00  0.00      PROA
ATOM    706  HG1 SER P  46      -7.760 -16.798   7.220  0.00  0.00      PROA
ATOM    707  C   SER P  46      -8.600 -17.069   3.233  0.00  0.00      PROA
ATOM    708  O   SER P  46      -7.905 -17.484   2.309  0.00  0.00      PROA
ATOM    709  N   ILE P  47      -9.872 -16.800   3.039  0.00  0.00      PROA
ATOM    710  HN  ILE P  47     -10.549 -16.814   3.770  0.00  0.00      PROA
ATOM    711  CA  ILE P  47     -10.492 -16.926   1.740  0.00  0.00      PROA
ATOM    712  HA  ILE P  47     -10.249 -17.898   1.337  0.00  0.00      PROA
ATOM    713  CB  ILE P  47     -12.016 -16.943   1.981  0.00  0.00      PROA
ATOM    714  HB  ILE P  47     -12.252 -16.111   2.680  0.00  0.00      PROA
ATOM    715  CG2 ILE P  47     -12.755 -16.636   0.604  0.00  0.00      PROA
ATOM    716 HG21 ILE P  47     -13.856 -16.734   0.716  0.00  0.00      PROA
ATOM    717 HG22 ILE P  47     -12.450 -17.385  -0.157  0.00  0.00      PROA
ATOM    718 HG23 ILE P  47     -12.688 -15.575   0.280  0.00  0.00      PROA
ATOM    719  CG1 ILE P  47     -12.558 -18.225   2.690  0.00  0.00      PROA
ATOM    720 HG11 ILE P  47     -11.860 -18.506   3.507  0.00  0.00      PROA
ATOM    721 HG12 ILE P  47     -12.668 -19.043   1.947  0.00  0.00      PROA
ATOM    722  CD  ILE P  47     -13.881 -17.891   3.380  0.00  0.00      PROA
ATOM    723  HD1 ILE P  47     -14.547 -18.125   2.522  0.00  0.00      PROA
ATOM    724  HD2 ILE P  47     -13.985 -16.845   3.741  0.00  0.00      PROA
ATOM    725  HD3 ILE P  47     -14.183 -18.553   4.219  0.00  0.00      PROA
ATOM    726  C   ILE P  47      -9.993 -16.010   0.656  0.00  0.00      PROA
ATOM    727  O   ILE P  47      -9.791 -16.463  -0.508  0.00  0.00      PROA
ATOM    728  N   ILE P  48      -9.710 -14.728   0.946  0.00  0.00      PROA
```

ATOM	729	HN	ILE	P	48	-9.934	-14.378	1.853	0.00	0.00	PROA
ATOM	730	CA	ILE	P	48	-9.073	-13.808	0.068	0.00	0.00	PROA
ATOM	731	HA	ILE	P	48	-9.593	-13.743	-0.877	0.00	0.00	PROA
ATOM	732	CB	ILE	P	48	-9.111	-12.414	0.716	0.00	0.00	PROA
ATOM	733	HB	ILE	P	48	-8.871	-12.595	1.785	0.00	0.00	PROA
ATOM	734	CG2	ILE	P	48	-7.952	-11.452	0.302	0.00	0.00	PROA
ATOM	735	HG21	ILE	P	48	-7.536	-11.530	-0.725	0.00	0.00	PROA
ATOM	736	HG22	ILE	P	48	-7.073	-11.674	0.944	0.00	0.00	PROA
ATOM	737	HG23	ILE	P	48	-8.134	-10.373	0.498	0.00	0.00	PROA
ATOM	738	CG1	ILE	P	48	-10.482	-11.722	0.581	0.00	0.00	PROA
ATOM	739	HG11	ILE	P	48	-11.244	-12.443	0.946	0.00	0.00	PROA
ATOM	740	HG12	ILE	P	48	-10.793	-11.432	-0.446	0.00	0.00	PROA
ATOM	741	CD	ILE	P	48	-10.618	-10.472	1.524	0.00	0.00	PROA
ATOM	742	HD1	ILE	P	48	-11.534	-9.847	1.451	0.00	0.00	PROA
ATOM	743	HD2	ILE	P	48	-9.843	-9.745	1.200	0.00	0.00	PROA
ATOM	744	HD3	ILE	P	48	-10.513	-10.825	2.572	0.00	0.00	PROA
ATOM	745	C	ILE	P	48	-7.723	-14.254	-0.335	0.00	0.00	PROA
ATOM	746	O	ILE	P	48	-7.253	-14.098	-1.414	0.00	0.00	PROA
ATOM	747	N	PHE	P	49	-6.970	-14.832	0.680	0.00	0.00	PROA
ATOM	748	HN	PHE	P	49	-7.276	-14.695	1.619	0.00	0.00	PROA
ATOM	749	CA	PHE	P	49	-5.635	-15.295	0.553	0.00	0.00	PROA
ATOM	750	HA	PHE	P	49	-5.061	-14.472	0.154	0.00	0.00	PROA
ATOM	751	CB	PHE	P	49	-4.972	-15.683	1.967	0.00	0.00	PROA
ATOM	752	HB1	PHE	P	49	-5.899	-16.011	2.484	0.00	0.00	PROA
ATOM	753	HB2	PHE	P	49	-4.495	-14.798	2.438	0.00	0.00	PROA
ATOM	754	CG	PHE	P	49	-4.054	-16.881	1.855	0.00	0.00	PROA
ATOM	755	CD1	PHE	P	49	-2.823	-16.928	1.180	0.00	0.00	PROA
ATOM	756	HD1	PHE	P	49	-2.459	-15.999	0.766	0.00	0.00	PROA
ATOM	757	CE1	PHE	P	49	-2.259	-18.144	0.825	0.00	0.00	PROA
ATOM	758	HE1	PHE	P	49	-1.416	-18.027	0.160	0.00	0.00	PROA
ATOM	759	CZ	PHE	P	49	-2.825	-19.328	1.224	0.00	0.00	PROA
ATOM	760	HZ	PHE	P	49	-2.402	-20.303	1.031	0.00	0.00	PROA
ATOM	761	CD2	PHE	P	49	-4.508	-18.115	2.407	0.00	0.00	PROA
ATOM	762	HD2	PHE	P	49	-5.326	-18.122	3.112	0.00	0.00	PROA
ATOM	763	CE2	PHE	P	49	-3.884	-19.318	2.099	0.00	0.00	PROA
ATOM	764	HE2	PHE	P	49	-4.325	-20.250	2.418	0.00	0.00	PROA
ATOM	765	C	PHE	P	49	-5.579	-16.412	-0.515	0.00	0.00	PROA
ATOM	766	O	PHE	P	49	-4.872	-16.228	-1.521	0.00	0.00	PROA
ATOM	767	N	LEU	P	50	-6.451	-17.362	-0.386	0.00	0.00	PROA
ATOM	768	HN	LEU	P	50	-7.033	-17.320	0.422	0.00	0.00	PROA
ATOM	769	CA	LEU	P	50	-6.621	-18.491	-1.242	0.00	0.00	PROA
ATOM	770	HA	LEU	P	50	-5.780	-19.167	-1.277	0.00	0.00	PROA
ATOM	771	CB	LEU	P	50	-7.729	-19.301	-0.650	0.00	0.00	PROA
ATOM	772	HB1	LEU	P	50	-8.574	-18.585	-0.561	0.00	0.00	PROA
ATOM	773	HB2	LEU	P	50	-7.333	-19.645	0.330	0.00	0.00	PROA
ATOM	774	CG	LEU	P	50	-8.250	-20.461	-1.597	0.00	0.00	PROA
ATOM	775	HG	LEU	P	50	-8.665	-20.009	-2.523	0.00	0.00	PROA
ATOM	776	CD1	LEU	P	50	-7.175	-21.494	-1.985	0.00	0.00	PROA
ATOM	777	HD11	LEU	P	50	-6.265	-21.034	-2.427	0.00	0.00	PROA
ATOM	778	HD12	LEU	P	50	-7.571	-22.212	-2.735	0.00	0.00	PROA
ATOM	779	HD13	LEU	P	50	-6.941	-22.043	-1.048	0.00	0.00	PROA
ATOM	780	CD2	LEU	P	50	-9.416	-21.263	-0.852	0.00	0.00	PROA
ATOM	781	HD21	LEU	P	50	-9.126	-21.811	0.070	0.00	0.00	PROA
ATOM	782	HD22	LEU	P	50	-9.854	-21.958	-1.599	0.00	0.00	PROA
ATOM	783	HD23	LEU	P	50	-10.244	-20.562	-0.610	0.00	0.00	PROA
ATOM	784	C	LEU	P	50	-6.985	-18.035	-2.642	0.00	0.00	PROA
ATOM	785	O	LEU	P	50	-6.459	-18.429	-3.680	0.00	0.00	PROA
ATOM	786	N	THR	P	51	-7.945	-17.062	-2.733	0.00	0.00	PROA
ATOM	787	HN	THR	P	51	-8.305	-16.845	-1.829	0.00	0.00	PROA
ATOM	788	CA	THR	P	51	-8.501	-16.515	-4.027	0.00	0.00	PROA
ATOM	789	HA	THR	P	51	-8.591	-17.337	-4.721	0.00	0.00	PROA

```
ATOM    790  CB  THR P  51      -9.729 -15.627  -3.859  0.00  0.00      PROA
ATOM    791  HB  THR P  51      -9.474 -14.694  -3.313  0.00  0.00      PROA
ATOM    792  OG1 THR P  51     -10.802 -16.333  -3.310  0.00  0.00      PROA
ATOM    793  HG1 THR P  51     -10.507 -16.574  -2.428  0.00  0.00      PROA
ATOM    794  CG2 THR P  51     -10.389 -15.322  -5.218  0.00  0.00      PROA
ATOM    795 HG21 THR P  51      -9.713 -14.678  -5.820  0.00  0.00      PROA
ATOM    796 HG22 THR P  51     -11.352 -14.787  -5.074  0.00  0.00      PROA
ATOM    797 HG23 THR P  51     -10.534 -16.318  -5.690  0.00  0.00      PROA
ATOM    798  C   THR P  51      -7.424 -15.652  -4.778  0.00  0.00      PROA
ATOM    799  O   THR P  51      -7.359 -15.607  -5.978  0.00  0.00      PROA
ATOM    800  N   GLY P  52      -6.517 -14.935  -4.031  0.00  0.00      PROA
ATOM    801  HN  GLY P  52      -6.588 -15.049  -3.043  0.00  0.00      PROA
ATOM    802  CA  GLY P  52      -5.472 -14.084  -4.608  0.00  0.00      PROA
ATOM    803  HA1 GLY P  52      -5.015 -13.427  -3.882  0.00  0.00      PROA
ATOM    804  HA2 GLY P  52      -5.951 -13.637  -5.467  0.00  0.00      PROA
ATOM    805  C   GLY P  52      -4.341 -14.873  -5.055  0.00  0.00      PROA
ATOM    806  O   GLY P  52      -3.819 -14.636  -6.119  0.00  0.00      PROA
ATOM    807  N   ILE P  53      -3.978 -15.918  -4.326  0.00  0.00      PROA
ATOM    808  HN  ILE P  53      -4.295 -15.981  -3.382  0.00  0.00      PROA
ATOM    809  CA  ILE P  53      -3.139 -16.977  -4.859  0.00  0.00      PROA
ATOM    810  HA  ILE P  53      -2.289 -16.346  -5.073  0.00  0.00      PROA
ATOM    811  CB  ILE P  53      -2.570 -17.993  -3.790  0.00  0.00      PROA
ATOM    812  HB  ILE P  53      -3.523 -18.241  -3.276  0.00  0.00      PROA
ATOM    813  CG2 ILE P  53      -1.911 -19.269  -4.414  0.00  0.00      PROA
ATOM    814 HG21 ILE P  53      -0.987 -19.032  -4.984  0.00  0.00      PROA
ATOM    815 HG22 ILE P  53      -2.553 -19.910  -5.055  0.00  0.00      PROA
ATOM    816 HG23 ILE P  53      -1.764 -19.915  -3.523  0.00  0.00      PROA
ATOM    817  CG1 ILE P  53      -1.539 -17.439  -2.790  0.00  0.00      PROA
ATOM    818 HG11 ILE P  53      -0.878 -18.174  -2.283  0.00  0.00      PROA
ATOM    819 HG12 ILE P  53      -2.132 -16.990  -1.965  0.00  0.00      PROA
ATOM    820  CD  ILE P  53      -0.568 -16.286  -3.249  0.00  0.00      PROA
ATOM    821  HD1 ILE P  53       0.000 -16.682  -4.118  0.00  0.00      PROA
ATOM    822  HD2 ILE P  53       0.149 -16.040  -2.437  0.00  0.00      PROA
ATOM    823  HD3 ILE P  53      -1.212 -15.448  -3.592  0.00  0.00      PROA
ATOM    824  C   ILE P  53      -3.553 -17.717  -6.127  0.00  0.00      PROA
ATOM    825  O   ILE P  53      -2.838 -17.761  -7.124  0.00  0.00      PROA
ATOM    826  N   VAL P  54      -4.770 -18.259  -6.073  0.00  0.00      PROA
ATOM    827  HN  VAL P  54      -5.191 -18.396  -5.180  0.00  0.00      PROA
ATOM    828  CA  VAL P  54      -5.240 -18.978  -7.238  0.00  0.00      PROA
ATOM    829  HA  VAL P  54      -4.421 -19.553  -7.644  0.00  0.00      PROA
ATOM    830  CB  VAL P  54      -6.306 -20.019  -6.823  0.00  0.00      PROA
ATOM    831  HB  VAL P  54      -7.084 -19.541  -6.190  0.00  0.00      PROA
ATOM    832  CG1 VAL P  54      -6.889 -20.604  -8.113  0.00  0.00      PROA
ATOM    833 HG11 VAL P  54      -7.367 -21.595  -7.963  0.00  0.00      PROA
ATOM    834 HG12 VAL P  54      -6.185 -20.719  -8.965  0.00  0.00      PROA
ATOM    835 HG13 VAL P  54      -7.699 -19.945  -8.494  0.00  0.00      PROA
ATOM    836  CG2 VAL P  54      -5.592 -21.195  -5.995  0.00  0.00      PROA
ATOM    837 HG21 VAL P  54      -4.803 -21.729  -6.565  0.00  0.00      PROA
ATOM    838 HG22 VAL P  54      -6.357 -21.870  -5.555  0.00  0.00      PROA
ATOM    839 HG23 VAL P  54      -5.103 -20.609  -5.187  0.00  0.00      PROA
ATOM    840  C   VAL P  54      -5.588 -17.971  -8.371  0.00  0.00      PROA
ATOM    841  O   VAL P  54      -5.002 -17.972  -9.526  0.00  0.00      PROA
ATOM    842  N   GLY P  55      -6.322 -16.923  -8.107  0.00  0.00      PROA
ATOM    843  HN  GLY P  55      -6.792 -16.965  -7.229  0.00  0.00      PROA
ATOM    844  CA  GLY P  55      -6.583 -15.829  -9.042  0.00  0.00      PROA
ATOM    845  HA1 GLY P  55      -7.193 -15.123  -8.498  0.00  0.00      PROA
ATOM    846  HA2 GLY P  55      -7.221 -16.235  -9.813  0.00  0.00      PROA
ATOM    847  C   GLY P  55      -5.390 -15.150  -9.695  0.00  0.00      PROA
ATOM    848  O   GLY P  55      -5.395 -15.104 -10.919  0.00  0.00      PROA
ATOM    849  N   ASN P  56      -4.481 -14.606  -8.897  0.00  0.00      PROA
ATOM    850  HN  ASN P  56      -4.642 -14.571  -7.913  0.00  0.00      PROA
```

ATOM 851 CA ASN P 56 -3.292 -13.960 -9.608 0.00 0.00 PROA
ATOM 852 HA ASN P 56 -3.628 -13.405 -10.471 0.00 0.00 PROA
ATOM 853 CB ASN P 56 -2.382 -13.110 -8.631 0.00 0.00 PROA
ATOM 854 HB1 ASN P 56 -1.427 -12.887 -9.152 0.00 0.00 PROA
ATOM 855 HB2 ASN P 56 -2.121 -13.804 -7.804 0.00 0.00 PROA
ATOM 856 CG ASN P 56 -3.213 -11.935 -8.338 0.00 0.00 PROA
ATOM 857 OD1 ASN P 56 -4.006 -11.505 -9.172 0.00 0.00 PROA
ATOM 858 ND2 ASN P 56 -2.977 -11.244 -7.205 0.00 0.00 PROA
ATOM 859 HD21 ASN P 56 -3.358 -10.340 -7.008 0.00 0.00 PROA
ATOM 860 HD22 ASN P 56 -2.241 -11.523 -6.588 0.00 0.00 PROA
ATOM 861 C ASN P 56 -2.343 -14.983 -10.243 0.00 0.00 PROA
ATOM 862 O ASN P 56 -1.732 -14.717 -11.231 0.00 0.00 PROA
ATOM 863 N GLY P 57 -2.313 -16.227 -9.757 0.00 0.00 PROA
ATOM 864 HN GLY P 57 -2.876 -16.527 -8.991 0.00 0.00 PROA
ATOM 865 CA GLY P 57 -1.748 -17.343 -10.534 0.00 0.00 PROA
ATOM 866 HA1 GLY P 57 -1.837 -18.264 -9.976 0.00 0.00 PROA
ATOM 867 HA2 GLY P 57 -0.708 -17.099 -10.693 0.00 0.00 PROA
ATOM 868 C GLY P 57 -2.413 -17.452 -11.914 0.00 0.00 PROA
ATOM 869 O GLY P 57 -1.667 -17.531 -12.922 0.00 0.00 PROA
ATOM 870 N LEU P 58 -3.743 -17.418 -12.096 0.00 0.00 PROA
ATOM 871 HN LEU P 58 -4.420 -17.572 -11.381 0.00 0.00 PROA
ATOM 872 CA LEU P 58 -4.308 -17.491 -13.421 0.00 0.00 PROA
ATOM 873 HA LEU P 58 -3.778 -18.291 -13.917 0.00 0.00 PROA
ATOM 874 CB LEU P 58 -5.796 -17.858 -13.483 0.00 0.00 PROA
ATOM 875 HB1 LEU P 58 -6.111 -18.111 -14.518 0.00 0.00 PROA
ATOM 876 HB2 LEU P 58 -6.394 -16.952 -13.247 0.00 0.00 PROA
ATOM 877 CG LEU P 58 -6.199 -19.007 -12.542 0.00 0.00 PROA
ATOM 878 HG LEU P 58 -6.182 -18.677 -11.481 0.00 0.00 PROA
ATOM 879 CD1 LEU P 58 -7.740 -19.192 -12.745 0.00 0.00 PROA
ATOM 880 HD11 LEU P 58 -8.213 -19.897 -12.028 0.00 0.00 PROA
ATOM 881 HD12 LEU P 58 -8.074 -19.590 -13.727 0.00 0.00 PROA
ATOM 882 HD13 LEU P 58 -8.309 -18.244 -12.630 0.00 0.00 PROA
ATOM 883 CD2 LEU P 58 -5.442 -20.282 -12.819 0.00 0.00 PROA
ATOM 884 HD21 LEU P 58 -4.356 -20.180 -12.606 0.00 0.00 PROA
ATOM 885 HD22 LEU P 58 -5.573 -20.544 -13.890 0.00 0.00 PROA
ATOM 886 HD23 LEU P 58 -5.857 -21.124 -12.224 0.00 0.00 PROA
ATOM 887 C LEU P 58 -4.047 -16.180 -14.194 0.00 0.00 PROA
ATOM 888 O LEU P 58 -3.713 -16.244 -15.394 0.00 0.00 PROA
ATOM 889 N VAL P 59 -4.117 -14.979 -13.577 0.00 0.00 PROA
ATOM 890 HN VAL P 59 -4.450 -15.044 -12.639 0.00 0.00 PROA
ATOM 891 CA VAL P 59 -3.909 -13.768 -14.370 0.00 0.00 PROA
ATOM 892 HA VAL P 59 -4.641 -13.735 -15.164 0.00 0.00 PROA
ATOM 893 CB VAL P 59 -4.210 -12.526 -13.656 0.00 0.00 PROA
ATOM 894 HB VAL P 59 -3.487 -12.392 -12.823 0.00 0.00 PROA
ATOM 895 CG1 VAL P 59 -4.046 -11.253 -14.625 0.00 0.00 PROA
ATOM 896 HG11 VAL P 59 -2.979 -11.066 -14.871 0.00 0.00 PROA
ATOM 897 HG12 VAL P 59 -4.511 -10.405 -14.077 0.00 0.00 PROA
ATOM 898 HG13 VAL P 59 -4.607 -11.380 -15.575 0.00 0.00 PROA
ATOM 899 CG2 VAL P 59 -5.621 -12.602 -13.055 0.00 0.00 PROA
ATOM 900 HG21 VAL P 59 -6.419 -12.648 -13.826 0.00 0.00 PROA
ATOM 901 HG22 VAL P 59 -5.654 -11.685 -12.429 0.00 0.00 PROA
ATOM 902 HG23 VAL P 59 -5.830 -13.406 -12.317 0.00 0.00 PROA
ATOM 903 C VAL P 59 -2.536 -13.733 -15.029 0.00 0.00 PROA
ATOM 904 O VAL P 59 -2.298 -13.334 -16.164 0.00 0.00 PROA
ATOM 905 N ILE P 60 -1.457 -14.087 -14.311 0.00 0.00 PROA
ATOM 906 HN ILE P 60 -1.595 -14.354 -13.361 0.00 0.00 PROA
ATOM 907 CA ILE P 60 -0.030 -14.208 -14.790 0.00 0.00 PROA
ATOM 908 HA ILE P 60 0.289 -13.239 -15.145 0.00 0.00 PROA
ATOM 909 CB ILE P 60 0.846 -14.527 -13.608 0.00 0.00 PROA
ATOM 910 HB ILE P 60 0.339 -15.428 -13.199 0.00 0.00 PROA
ATOM 911 CG2 ILE P 60 2.256 -14.990 -13.934 0.00 0.00 PROA

```
ATOM    912 HG21 ILE P  60       2.576 -14.457 -14.855  0.00  0.00      PROA
ATOM    913 HG22 ILE P  60       2.396 -16.064 -14.179  0.00  0.00      PROA
ATOM    914 HG23 ILE P  60       2.996 -14.761 -13.137  0.00  0.00      PROA
ATOM    915  CG1 ILE P  60       0.757 -13.404 -12.546  0.00  0.00      PROA
ATOM    916 HG11 ILE P  60      -0.264 -13.030 -12.320  0.00  0.00      PROA
ATOM    917 HG12 ILE P  60       1.202 -12.534 -13.074  0.00  0.00      PROA
ATOM    918  CD  ILE P  60       1.460 -13.791 -11.193  0.00  0.00      PROA
ATOM    919  HD1 ILE P  60       1.349 -12.950 -10.475  0.00  0.00      PROA
ATOM    920  HD2 ILE P  60       2.521 -14.114 -11.124  0.00  0.00      PROA
ATOM    921  HD3 ILE P  60       0.857 -14.514 -10.603  0.00  0.00      PROA
ATOM    922  C   ILE P  60       0.064 -15.248 -15.871  0.00  0.00      PROA
ATOM    923  O   ILE P  60       0.656 -14.993 -16.928  0.00  0.00      PROA
ATOM    924  N   LEU P  61      -0.721 -16.323 -15.771  0.00  0.00      PROA
ATOM    925  HN  LEU P  61      -1.218 -16.586 -14.947  0.00  0.00      PROA
ATOM    926  CA  LEU P  61      -0.834 -17.383 -16.736  0.00  0.00      PROA
ATOM    927  HA  LEU P  61       0.150 -17.721 -17.027  0.00  0.00      PROA
ATOM    928  CB  LEU P  61      -1.615 -18.569 -16.049  0.00  0.00      PROA
ATOM    929  HB1 LEU P  61      -2.689 -18.393 -15.826  0.00  0.00      PROA
ATOM    930  HB2 LEU P  61      -1.081 -18.645 -15.078  0.00  0.00      PROA
ATOM    931  CG  LEU P  61      -1.672 -19.886 -16.819  0.00  0.00      PROA
ATOM    932  HG  LEU P  61      -1.963 -19.669 -17.869  0.00  0.00      PROA
ATOM    933  CD1 LEU P  61      -0.277 -20.609 -16.662  0.00  0.00      PROA
ATOM    934 HD11 LEU P  61      -0.497 -21.629 -17.044  0.00  0.00      PROA
ATOM    935 HD12 LEU P  61      -0.107 -20.572 -15.565  0.00  0.00      PROA
ATOM    936 HD13 LEU P  61       0.462 -20.030 -17.257  0.00  0.00      PROA
ATOM    937  CD2 LEU P  61      -2.735 -20.834 -16.215  0.00  0.00      PROA
ATOM    938 HD21 LEU P  61      -3.660 -20.243 -16.045  0.00  0.00      PROA
ATOM    939 HD22 LEU P  61      -2.376 -21.193 -15.227  0.00  0.00      PROA
ATOM    940 HD23 LEU P  61      -2.893 -21.623 -16.981  0.00  0.00      PROA
ATOM    941  C   LEU P  61      -1.346 -16.818 -18.064  0.00  0.00      PROA
ATOM    942  O   LEU P  61      -0.757 -17.082 -19.094  0.00  0.00      PROA
ATOM    943  N   VAL P  62      -2.436 -16.071 -17.954  0.00  0.00      PROA
ATOM    944  HN  VAL P  62      -2.835 -15.955 -17.047  0.00  0.00      PROA
ATOM    945  CA  VAL P  62      -3.194 -15.386 -18.986  0.00  0.00      PROA
ATOM    946  HA  VAL P  62      -3.621 -16.165 -19.601  0.00  0.00      PROA
ATOM    947  CB  VAL P  62      -4.370 -14.640 -18.437  0.00  0.00      PROA
ATOM    948  HB  VAL P  62      -4.129 -14.106 -17.493  0.00  0.00      PROA
ATOM    949  CG1 VAL P  62      -5.003 -13.665 -19.377  0.00  0.00      PROA
ATOM    950 HG11 VAL P  62      -5.813 -13.245 -18.744  0.00  0.00      PROA
ATOM    951 HG12 VAL P  62      -5.358 -14.075 -20.347  0.00  0.00      PROA
ATOM    952 HG13 VAL P  62      -4.356 -12.797 -19.629  0.00  0.00      PROA
ATOM    953  CG2 VAL P  62      -5.473 -15.676 -18.039  0.00  0.00      PROA
ATOM    954 HG21 VAL P  62      -5.960 -16.197 -18.891  0.00  0.00      PROA
ATOM    955 HG22 VAL P  62      -6.247 -15.215 -17.389  0.00  0.00      PROA
ATOM    956 HG23 VAL P  62      -5.117 -16.415 -17.290  0.00  0.00      PROA
ATOM    957  C   VAL P  62      -2.329 -14.431 -19.746  0.00  0.00      PROA
ATOM    958  O   VAL P  62      -2.228 -14.431 -20.970  0.00  0.00      PROA
ATOM    959  N   MET P  63      -1.501 -13.681 -19.028  0.00  0.00      PROA
ATOM    960  HN  MET P  63      -1.645 -13.817 -18.051  0.00  0.00      PROA
ATOM    961  CA  MET P  63      -0.478 -12.779 -19.507  0.00  0.00      PROA
ATOM    962  HA  MET P  63      -1.014 -12.133 -20.186  0.00  0.00      PROA
ATOM    963  CB  MET P  63       0.027 -11.922 -18.310  0.00  0.00      PROA
ATOM    964  HB1 MET P  63       0.950 -11.408 -18.655  0.00  0.00      PROA
ATOM    965  HB2 MET P  63       0.187 -12.585 -17.433  0.00  0.00      PROA
ATOM    966  CG  MET P  63      -1.100 -10.815 -17.827  0.00  0.00      PROA
ATOM    967  HG1 MET P  63      -0.621 -10.296 -16.970  0.00  0.00      PROA
ATOM    968  HG2 MET P  63      -1.960 -11.437 -17.498  0.00  0.00      PROA
ATOM    969  SD  MET P  63      -1.595  -9.488 -18.969  0.00  0.00      PROA
ATOM    970  CE  MET P  63      -2.610  -8.668 -17.773  0.00  0.00      PROA
ATOM    971  HE1 MET P  63      -2.036  -8.021 -17.075  0.00  0.00      PROA
ATOM    972  HE2 MET P  63      -3.211  -9.386 -17.175  0.00  0.00      PROA
```

ATOM	973	HE3	MET	P	63	-3.316	-7.998	-18.308	0.00	0.00	PROA
ATOM	974	C	MET	P	63	0.635	-13.434	-20.286	0.00	0.00	PROA
ATOM	975	O	MET	P	63	1.128	-12.995	-21.329	0.00	0.00	PROA
ATOM	976	N	GLY	P	64	1.125	-14.574	-19.818	0.00	0.00	PROA
ATOM	977	HN	GLY	P	64	0.791	-14.772	-18.900	0.00	0.00	PROA
ATOM	978	CA	GLY	P	64	1.987	-15.465	-20.596	0.00	0.00	PROA
ATOM	979	HA1	GLY	P	64	2.228	-16.266	-19.913	0.00	0.00	PROA
ATOM	980	HA2	GLY	P	64	2.862	-14.911	-20.902	0.00	0.00	PROA
ATOM	981	C	GLY	P	64	1.401	-16.170	-21.728	0.00	0.00	PROA
ATOM	982	O	GLY	P	64	2.001	-16.195	-22.811	0.00	0.00	PROA
ATOM	983	N	TYR	P	65	0.280	-16.854	-21.627	0.00	0.00	PROA
ATOM	984	HN	TYR	P	65	-0.176	-16.907	-20.742	0.00	0.00	PROA
ATOM	985	CA	TYR	P	65	-0.298	-17.727	-22.581	0.00	0.00	PROA
ATOM	986	HA	TYR	P	65	0.463	-18.057	-23.272	0.00	0.00	PROA
ATOM	987	CB	TYR	P	65	-0.895	-19.022	-21.874	0.00	0.00	PROA
ATOM	988	HB1	TYR	P	65	-1.510	-19.515	-22.656	0.00	0.00	PROA
ATOM	989	HB2	TYR	P	65	-1.512	-18.780	-20.982	0.00	0.00	PROA
ATOM	990	CG	TYR	P	65	0.149	-20.012	-21.527	0.00	0.00	PROA
ATOM	991	CD1	TYR	P	65	0.993	-20.567	-22.591	0.00	0.00	PROA
ATOM	992	HD1	TYR	P	65	0.982	-20.105	-23.568	0.00	0.00	PROA
ATOM	993	CE1	TYR	P	65	1.875	-21.578	-22.236	0.00	0.00	PROA
ATOM	994	HE1	TYR	P	65	2.354	-22.079	-23.064	0.00	0.00	PROA
ATOM	995	CZ	TYR	P	65	1.889	-22.145	-20.981	0.00	0.00	PROA
ATOM	996	OH	TYR	P	65	2.878	-23.082	-20.749	0.00	0.00	PROA
ATOM	997	HH	TYR	P	65	2.595	-23.444	-19.906	0.00	0.00	PROA
ATOM	998	CD2	TYR	P	65	0.197	-20.613	-20.240	0.00	0.00	PROA
ATOM	999	HD2	TYR	P	65	-0.400	-20.221	-19.430	0.00	0.00	PROA
ATOM	1000	CE2	TYR	P	65	0.979	-21.710	-19.985	0.00	0.00	PROA
ATOM	1001	HE2	TYR	P	65	1.026	-22.121	-18.987	0.00	0.00	PROA
ATOM	1002	C	TYR	P	65	-1.326	-17.062	-23.460	0.00	0.00	PROA
ATOM	1003	O	TYR	P	65	-1.059	-16.836	-24.625	0.00	0.00	PROA
ATOM	1004	N	GLN	P	66	-2.496	-16.776	-22.886	0.00	0.00	PROA
ATOM	1005	HN	GLN	P	66	-2.566	-16.890	-21.898	0.00	0.00	PROA
ATOM	1006	CA	GLN	P	66	-3.698	-16.256	-23.541	0.00	0.00	PROA
ATOM	1007	HA	GLN	P	66	-3.998	-16.957	-24.305	0.00	0.00	PROA
ATOM	1008	CB	GLN	P	66	-4.809	-16.323	-22.446	0.00	0.00	PROA
ATOM	1009	HB1	GLN	P	66	-5.773	-15.917	-22.821	0.00	0.00	PROA
ATOM	1010	HB2	GLN	P	66	-4.489	-15.583	-21.681	0.00	0.00	PROA
ATOM	1011	CG	GLN	P	66	-5.293	-17.732	-21.973	0.00	0.00	PROA
ATOM	1012	HG1	GLN	P	66	-6.342	-17.687	-21.608	0.00	0.00	PROA
ATOM	1013	HG2	GLN	P	66	-4.656	-17.901	-21.079	0.00	0.00	PROA
ATOM	1014	CD	GLN	P	66	-5.009	-18.905	-22.896	0.00	0.00	PROA
ATOM	1015	OE1	GLN	P	66	-4.048	-19.660	-22.892	0.00	0.00	PROA
ATOM	1016	NE2	GLN	P	66	-5.799	-19.001	-24.032	0.00	0.00	PROA
ATOM	1017	HE21	GLN	P	66	-5.603	-19.720	-24.699	0.00	0.00	PROA
ATOM	1018	HE22	GLN	P	66	-6.365	-18.195	-24.204	0.00	0.00	PROA
ATOM	1019	C	GLN	P	66	-3.455	-14.889	-24.203	0.00	0.00	PROA
ATOM	1020	O	GLN	P	66	-3.925	-14.694	-25.394	0.00	0.00	PROA
ATOM	1021	N	LYS	P	67	-2.750	-13.964	-23.568	0.00	0.00	PROA
ATOM	1022	HN	LYS	P	67	-2.467	-14.225	-22.649	0.00	0.00	PROA
ATOM	1023	CA	LYS	P	67	-2.359	-12.771	-24.256	0.00	0.00	PROA
ATOM	1024	HA	LYS	P	67	-3.242	-12.292	-24.653	0.00	0.00	PROA
ATOM	1025	CB	LYS	P	67	-1.795	-11.751	-23.279	0.00	0.00	PROA
ATOM	1026	HB1	LYS	P	67	-1.218	-10.981	-23.836	0.00	0.00	PROA
ATOM	1027	HB2	LYS	P	67	-0.928	-12.108	-22.683	0.00	0.00	PROA
ATOM	1028	CG	LYS	P	67	-2.801	-11.080	-22.370	0.00	0.00	PROA
ATOM	1029	HG1	LYS	P	67	-2.119	-10.626	-21.619	0.00	0.00	PROA
ATOM	1030	HG2	LYS	P	67	-3.442	-11.875	-21.933	0.00	0.00	PROA
ATOM	1031	CD	LYS	P	67	-3.633	-9.966	-22.990	0.00	0.00	PROA
ATOM	1032	HD1	LYS	P	67	-4.292	-10.402	-23.771	0.00	0.00	PROA
ATOM	1033	HD2	LYS	P	67	-2.925	-9.188	-23.349	0.00	0.00	PROA

ATOM 1034 CE LYS P 67 -4.664 -9.348 -22.062 0.00 0.00 PROA
ATOM 1035 HE1 LYS P 67 -4.228 -9.249 -21.045 0.00 0.00 PROA
ATOM 1036 HE2 LYS P 67 -5.459 -10.123 -22.103 0.00 0.00 PROA
ATOM 1037 NZ LYS P 67 -5.128 -8.122 -22.600 0.00 0.00 PROA
ATOM 1038 HZ1 LYS P 67 -4.268 -7.563 -22.772 0.00 0.00 PROA
ATOM 1039 HZ2 LYS P 67 -5.598 -7.470 -21.941 0.00 0.00 PROA
ATOM 1040 HZ3 LYS P 67 -5.781 -8.137 -23.409 0.00 0.00 PROA
ATOM 1041 C LYS P 67 -1.408 -12.906 -25.435 0.00 0.00 PROA
ATOM 1042 O LYS P 67 -0.314 -13.399 -25.339 0.00 0.00 PROA
ATOM 1043 N LYS P 68 -1.887 -12.583 -26.660 0.00 0.00 PROA
ATOM 1044 HN LYS P 68 -2.813 -12.235 -26.787 0.00 0.00 PROA
ATOM 1045 CA LYS P 68 -1.120 -12.643 -27.908 0.00 0.00 PROA
ATOM 1046 HA LYS P 68 -0.494 -13.517 -28.011 0.00 0.00 PROA
ATOM 1047 CB LYS P 68 -2.269 -12.516 -29.030 0.00 0.00 PROA
ATOM 1048 HB1 LYS P 68 -1.900 -12.234 -30.039 0.00 0.00 PROA
ATOM 1049 HB2 LYS P 68 -2.885 -11.662 -28.676 0.00 0.00 PROA
ATOM 1050 CG LYS P 68 -3.120 -13.764 -29.055 0.00 0.00 PROA
ATOM 1051 HG1 LYS P 68 -3.921 -13.356 -29.708 0.00 0.00 PROA
ATOM 1052 HG2 LYS P 68 -3.456 -14.026 -28.030 0.00 0.00 PROA
ATOM 1053 CD LYS P 68 -2.431 -14.837 -29.819 0.00 0.00 PROA
ATOM 1054 HD1 LYS P 68 -1.483 -15.117 -29.313 0.00 0.00 PROA
ATOM 1055 HD2 LYS P 68 -2.166 -14.445 -30.824 0.00 0.00 PROA
ATOM 1056 CE LYS P 68 -3.215 -16.146 -29.837 0.00 0.00 PROA
ATOM 1057 HE1 LYS P 68 -4.221 -15.886 -30.231 0.00 0.00 PROA
ATOM 1058 HE2 LYS P 68 -3.328 -16.544 -28.806 0.00 0.00 PROA
ATOM 1059 NZ LYS P 68 -2.557 -17.068 -30.719 0.00 0.00 PROA
ATOM 1060 HZ1 LYS P 68 -3.195 -17.792 -31.106 0.00 0.00 PROA
ATOM 1061 HZ2 LYS P 68 -1.767 -17.595 -30.296 0.00 0.00 PROA
ATOM 1062 HZ3 LYS P 68 -2.239 -16.583 -31.583 0.00 0.00 PROA
ATOM 1063 C LYS P 68 -0.140 -11.505 -28.089 0.00 0.00 PROA
ATOM 1064 O LYS P 68 1.057 -11.722 -28.127 0.00 0.00 PROA
ATOM 1065 N LEU P 69 -0.664 -10.208 -28.214 0.00 0.00 PROA
ATOM 1066 HN LEU P 69 -1.654 -10.101 -28.260 0.00 0.00 PROA
ATOM 1067 CA LEU P 69 0.082 -9.006 -28.258 0.00 0.00 PROA
ATOM 1068 HA LEU P 69 0.873 -9.141 -28.981 0.00 0.00 PROA
ATOM 1069 CB LEU P 69 -0.775 -7.849 -28.837 0.00 0.00 PROA
ATOM 1070 HB1 LEU P 69 -0.160 -6.945 -29.031 0.00 0.00 PROA
ATOM 1071 HB2 LEU P 69 -1.457 -7.481 -28.041 0.00 0.00 PROA
ATOM 1072 CG LEU P 69 -1.571 -8.116 -30.140 0.00 0.00 PROA
ATOM 1073 HG LEU P 69 -2.350 -8.849 -29.841 0.00 0.00 PROA
ATOM 1074 CD1 LEU P 69 -2.348 -6.863 -30.655 0.00 0.00 PROA
ATOM 1075 HD11 LEU P 69 -2.817 -6.309 -29.814 0.00 0.00 PROA
ATOM 1076 HD12 LEU P 69 -3.265 -7.114 -31.230 0.00 0.00 PROA
ATOM 1077 HD13 LEU P 69 -1.622 -6.179 -31.144 0.00 0.00 PROA
ATOM 1078 CD2 LEU P 69 -0.766 -8.692 -31.337 0.00 0.00 PROA
ATOM 1079 HD21 LEU P 69 -0.304 -9.643 -30.997 0.00 0.00 PROA
ATOM 1080 HD22 LEU P 69 0.085 -8.027 -31.597 0.00 0.00 PROA
ATOM 1081 HD23 LEU P 69 -1.382 -8.766 -32.259 0.00 0.00 PROA
ATOM 1082 C LEU P 69 0.618 -8.501 -26.987 0.00 0.00 PROA
ATOM 1083 O LEU P 69 -0.127 -8.302 -26.076 0.00 0.00 PROA
ATOM 1084 N ARG P 70 1.950 -8.099 -27.007 0.00 0.00 PROA
ATOM 1085 HN ARG P 70 2.514 -8.295 -27.805 0.00 0.00 PROA
ATOM 1086 CA ARG P 70 2.520 -7.424 -25.880 0.00 0.00 PROA
ATOM 1087 HA ARG P 70 2.263 -8.059 -25.045 0.00 0.00 PROA
ATOM 1088 CB ARG P 70 4.137 -7.470 -25.896 0.00 0.00 PROA
ATOM 1089 HB1 ARG P 70 4.432 -7.045 -24.913 0.00 0.00 PROA
ATOM 1090 HB2 ARG P 70 4.644 -6.963 -26.745 0.00 0.00 PROA
ATOM 1091 CG ARG P 70 4.841 -8.818 -25.839 0.00 0.00 PROA
ATOM 1092 HG1 ARG P 70 5.945 -8.699 -25.797 0.00 0.00 PROA
ATOM 1093 HG2 ARG P 70 4.575 -9.481 -26.690 0.00 0.00 PROA
ATOM 1094 CD ARG P 70 4.404 -9.733 -24.642 0.00 0.00 PROA

```
ATOM   1095  HD1 ARG P  70       3.436 -10.273 -24.719  0.00  0.00      PROA
ATOM   1096  HD2 ARG P  70       4.312  -9.150 -23.702  0.00  0.00      PROA
ATOM   1097  NE  ARG P  70       5.467 -10.833 -24.517  0.00  0.00      PROA
ATOM   1098  HE  ARG P  70       6.028 -11.002 -25.327  0.00  0.00      PROA
ATOM   1099  CZ  ARG P  70       5.700 -11.579 -23.504  0.00  0.00      PROA
ATOM   1100  NH1 ARG P  70       5.082 -11.426 -22.398  0.00  0.00      PROA
ATOM   1101 HH11 ARG P  70       4.403 -10.707 -22.543  0.00  0.00      PROA
ATOM   1102 HH12 ARG P  70       4.920 -12.160 -21.739  0.00  0.00      PROA
ATOM   1103  NH2 ARG P  70       6.551 -12.591 -23.653  0.00  0.00      PROA
ATOM   1104 HH21 ARG P  70       7.009 -12.701 -24.535  0.00  0.00      PROA
ATOM   1105 HH22 ARG P  70       6.820 -13.127 -22.853  0.00  0.00      PROA
ATOM   1106  C   ARG P  70       2.092  -5.925 -25.674  0.00  0.00      PROA
ATOM   1107  O   ARG P  70       2.043  -5.226 -26.684  0.00  0.00      PROA
ATOM   1108  N   SER P  71       1.797  -5.427 -24.527  0.00  0.00      PROA
ATOM   1109  HN  SER P  71       1.875  -5.955 -23.685  0.00  0.00      PROA
ATOM   1110  CA  SER P  71       1.510  -3.987 -24.394  0.00  0.00      PROA
ATOM   1111  HA  SER P  71       2.175  -3.485 -25.081  0.00  0.00      PROA
ATOM   1112  CB  SER P  71       0.037  -3.630 -24.699  0.00  0.00      PROA
ATOM   1113  HB1 SER P  71      -0.127  -3.984 -25.740  0.00  0.00      PROA
ATOM   1114  HB2 SER P  71      -0.081  -2.526 -24.710  0.00  0.00      PROA
ATOM   1115  OG  SER P  71      -0.922  -4.242 -23.856  0.00  0.00      PROA
ATOM   1116  HG1 SER P  71      -0.645  -5.159 -23.794  0.00  0.00      PROA
ATOM   1117  C   SER P  71       1.909  -3.585 -23.002  0.00  0.00      PROA
ATOM   1118  O   SER P  71       1.913  -4.459 -22.096  0.00  0.00      PROA
ATOM   1119  N   MET P  72       2.265  -2.289 -22.806  0.00  0.00      PROA
ATOM   1120  HN  MET P  72       2.127  -1.672 -23.577  0.00  0.00      PROA
ATOM   1121  CA  MET P  72       2.529  -1.702 -21.579  0.00  0.00      PROA
ATOM   1122  HA  MET P  72       3.359  -2.176 -21.076  0.00  0.00      PROA
ATOM   1123  CB  MET P  72       2.903  -0.223 -21.825  0.00  0.00      PROA
ATOM   1124  HB1 MET P  72       3.083   0.241 -20.832  0.00  0.00      PROA
ATOM   1125  HB2 MET P  72       2.060   0.274 -22.351  0.00  0.00      PROA
ATOM   1126  CG  MET P  72       4.249   0.049 -22.564  0.00  0.00      PROA
ATOM   1127  HG1 MET P  72       4.994  -0.457 -21.914  0.00  0.00      PROA
ATOM   1128  HG2 MET P  72       4.458   1.130 -22.415  0.00  0.00      PROA
ATOM   1129  SD  MET P  72       4.316  -0.443 -24.319  0.00  0.00      PROA
ATOM   1130  CE  MET P  72       3.125   0.755 -24.977  0.00  0.00      PROA
ATOM   1131  HE1 MET P  72       3.271   0.735 -26.078  0.00  0.00      PROA
ATOM   1132  HE2 MET P  72       3.260   1.818 -24.683  0.00  0.00      PROA
ATOM   1133  HE3 MET P  72       2.111   0.455 -24.636  0.00  0.00      PROA
ATOM   1134  C   MET P  72       1.428  -1.753 -20.570  0.00  0.00      PROA
ATOM   1135  O   MET P  72       1.719  -1.997 -19.413  0.00  0.00      PROA
ATOM   1136  N   THR P  73       0.171  -1.494 -20.979  0.00  0.00      PROA
ATOM   1137  HN  THR P  73       0.042  -1.384 -21.961  0.00  0.00      PROA
ATOM   1138  CA  THR P  73      -0.911  -1.728 -20.058  0.00  0.00      PROA
ATOM   1139  HA  THR P  73      -0.700  -1.150 -19.170  0.00  0.00      PROA
ATOM   1140  CB  THR P  73      -2.214  -1.242 -20.575  0.00  0.00      PROA
ATOM   1141  HB  THR P  73      -1.967  -0.272 -21.057  0.00  0.00      PROA
ATOM   1142  OG1 THR P  73      -3.117  -1.064 -19.512  0.00  0.00      PROA
ATOM   1143  HG1 THR P  73      -3.843  -0.671 -20.002  0.00  0.00      PROA
ATOM   1144  CG2 THR P  73      -2.780  -2.118 -21.725  0.00  0.00      PROA
ATOM   1145 HG21 THR P  73      -3.793  -1.753 -21.996  0.00  0.00      PROA
ATOM   1146 HG22 THR P  73      -3.169  -3.115 -21.426  0.00  0.00      PROA
ATOM   1147 HG23 THR P  73      -2.118  -2.258 -22.606  0.00  0.00      PROA
ATOM   1148  C   THR P  73      -1.018  -3.189 -19.424  0.00  0.00      PROA
ATOM   1149  O   THR P  73      -1.324  -3.354 -18.276  0.00  0.00      PROA
ATOM   1150  N   ASP P  74      -0.884  -4.263 -20.272  0.00  0.00      PROA
ATOM   1151  HN  ASP P  74      -0.762  -4.106 -21.249  0.00  0.00      PROA
ATOM   1152  CA  ASP P  74      -0.916  -5.588 -19.824  0.00  0.00      PROA
ATOM   1153  HA  ASP P  74      -1.764  -5.848 -19.209  0.00  0.00      PROA
ATOM   1154  CB  ASP P  74      -0.801  -6.447 -21.011  0.00  0.00      PROA
ATOM   1155  HB1 ASP P  74      -0.600  -7.505 -20.739  0.00  0.00      PROA
```

ATOM	1156	HB2	ASP	P	74	0.014	-6.185	-21.718	0.00	0.00	PROA
ATOM	1157	CG	ASP	P	74	-2.113	-6.449	-21.853	0.00	0.00	PROA
ATOM	1158	OD1	ASP	P	74	-2.015	-7.004	-22.951	0.00	0.00	PROA
ATOM	1159	OD2	ASP	P	74	-3.129	-5.888	-21.304	0.00	0.00	PROA
ATOM	1160	C	ASP	P	74	0.245	-5.853	-18.890	0.00	0.00	PROA
ATOM	1161	O	ASP	P	74	-0.001	-6.353	-17.788	0.00	0.00	PROA
ATOM	1162	N	LYS	P	75	1.489	-5.393	-19.184	0.00	0.00	PROA
ATOM	1163	HN	LYS	P	75	1.708	-5.324	-20.154	0.00	0.00	PROA
ATOM	1164	CA	LYS	P	75	2.607	-5.377	-18.280	0.00	0.00	PROA
ATOM	1165	HA	LYS	P	75	2.691	-6.396	-17.933	0.00	0.00	PROA
ATOM	1166	CB	LYS	P	75	3.916	-5.005	-19.062	0.00	0.00	PROA
ATOM	1167	HB1	LYS	P	75	3.703	-4.005	-19.498	0.00	0.00	PROA
ATOM	1168	HB2	LYS	P	75	4.082	-5.770	-19.851	0.00	0.00	PROA
ATOM	1169	CG	LYS	P	75	5.163	-4.993	-18.260	0.00	0.00	PROA
ATOM	1170	HG1	LYS	P	75	5.104	-4.212	-17.472	0.00	0.00	PROA
ATOM	1171	HG2	LYS	P	75	5.993	-4.643	-18.911	0.00	0.00	PROA
ATOM	1172	CD	LYS	P	75	5.593	-6.396	-17.678	0.00	0.00	PROA
ATOM	1173	HD1	LYS	P	75	5.452	-7.181	-18.452	0.00	0.00	PROA
ATOM	1174	HD2	LYS	P	75	4.999	-6.621	-16.767	0.00	0.00	PROA
ATOM	1175	CE	LYS	P	75	7.121	-6.511	-17.487	0.00	0.00	PROA
ATOM	1176	HE1	LYS	P	75	7.672	-5.776	-16.862	0.00	0.00	PROA
ATOM	1177	HE2	LYS	P	75	7.641	-6.493	-18.468	0.00	0.00	PROA
ATOM	1178	NZ	LYS	P	75	7.297	-7.834	-16.871	0.00	0.00	PROA
ATOM	1179	HZ1	LYS	P	75	6.612	-8.531	-17.228	0.00	0.00	PROA
ATOM	1180	HZ2	LYS	P	75	7.210	-7.719	-15.841	0.00	0.00	PROA
ATOM	1181	HZ3	LYS	P	75	8.213	-8.225	-17.169	0.00	0.00	PROA
ATOM	1182	C	LYS	P	75	2.453	-4.506	-16.954	0.00	0.00	PROA
ATOM	1183	O	LYS	P	75	2.761	-4.902	-15.838	0.00	0.00	PROA
ATOM	1184	N	TYR	P	76	1.830	-3.261	-17.019	0.00	0.00	PROA
ATOM	1185	HN	TYR	P	76	1.627	-2.759	-17.857	0.00	0.00	PROA
ATOM	1186	CA	TYR	P	76	1.311	-2.671	-15.770	0.00	0.00	PROA
ATOM	1187	HA	TYR	P	76	2.100	-2.646	-15.033	0.00	0.00	PROA
ATOM	1188	CB	TYR	P	76	0.812	-1.103	-15.945	0.00	0.00	PROA
ATOM	1189	HB1	TYR	P	76	0.352	-0.632	-15.050	0.00	0.00	PROA
ATOM	1190	HB2	TYR	P	76	0.118	-1.033	-16.811	0.00	0.00	PROA
ATOM	1191	CG	TYR	P	76	2.047	-0.298	-16.364	0.00	0.00	PROA
ATOM	1192	CD1	TYR	P	76	3.014	-0.108	-15.394	0.00	0.00	PROA
ATOM	1193	HD1	TYR	P	76	2.943	-0.542	-14.408	0.00	0.00	PROA
ATOM	1194	CE1	TYR	P	76	4.005	0.803	-15.706	0.00	0.00	PROA
ATOM	1195	HE1	TYR	P	76	4.750	1.113	-14.988	0.00	0.00	PROA
ATOM	1196	CZ	TYR	P	76	4.076	1.466	-16.930	0.00	0.00	PROA
ATOM	1197	OH	TYR	P	76	5.154	2.400	-17.072	0.00	0.00	PROA
ATOM	1198	HH	TYR	P	76	5.091	2.912	-17.882	0.00	0.00	PROA
ATOM	1199	CD2	TYR	P	76	2.015	0.372	-17.610	0.00	0.00	PROA
ATOM	1200	HD2	TYR	P	76	1.296	0.170	-18.390	0.00	0.00	PROA
ATOM	1201	CE2	TYR	P	76	3.034	1.300	-17.855	0.00	0.00	PROA
ATOM	1202	HE2	TYR	P	76	3.012	1.698	-18.859	0.00	0.00	PROA
ATOM	1203	C	TYR	P	76	0.318	-3.411	-14.905	0.00	0.00	PROA
ATOM	1204	O	TYR	P	76	0.450	-3.551	-13.694	0.00	0.00	PROA
ATOM	1205	N	ARG	P	77	-0.720	-4.005	-15.543	0.00	0.00	PROA
ATOM	1206	HN	ARG	P	77	-0.978	-3.868	-16.496	0.00	0.00	PROA
ATOM	1207	CA	ARG	P	77	-1.749	-4.756	-14.846	0.00	0.00	PROA
ATOM	1208	HA	ARG	P	77	-2.086	-4.084	-14.070	0.00	0.00	PROA
ATOM	1209	CB	ARG	P	77	-2.925	-5.115	-15.777	0.00	0.00	PROA
ATOM	1210	HB1	ARG	P	77	-2.453	-5.660	-16.622	0.00	0.00	PROA
ATOM	1211	HB2	ARG	P	77	-3.298	-4.098	-16.023	0.00	0.00	PROA
ATOM	1212	CG	ARG	P	77	-4.109	-6.017	-15.299	0.00	0.00	PROA
ATOM	1213	HG1	ARG	P	77	-3.694	-7.037	-15.155	0.00	0.00	PROA
ATOM	1214	HG2	ARG	P	77	-4.931	-6.174	-16.029	0.00	0.00	PROA
ATOM	1215	CD	ARG	P	77	-4.632	-5.553	-13.945	0.00	0.00	PROA
ATOM	1216	HD1	ARG	P	77	-4.942	-4.486	-13.917	0.00	0.00	PROA

ATOM 1217 HD2 ARG P 77 -3.921 -5.694 -13.103 0.00 0.00 PROA
ATOM 1218 NE ARG P 77 -5.805 -6.404 -13.584 0.00 0.00 PROA
ATOM 1219 HE ARG P 77 -5.684 -7.020 -12.805 0.00 0.00 PROA
ATOM 1220 CZ ARG P 77 -7.057 -5.990 -13.608 0.00 0.00 PROA
ATOM 1221 NH1 ARG P 77 -7.481 -5.096 -14.484 0.00 0.00 PROA
ATOM 1222 HH11 ARG P 77 -6.972 -4.876 -15.316 0.00 0.00 PROA
ATOM 1223 HH12 ARG P 77 -8.459 -4.887 -14.451 0.00 0.00 PROA
ATOM 1224 NH2 ARG P 77 -8.023 -6.514 -12.858 0.00 0.00 PROA
ATOM 1225 HH21 ARG P 77 -7.941 -7.407 -12.414 0.00 0.00 PROA
ATOM 1226 HH22 ARG P 77 -8.934 -6.136 -13.019 0.00 0.00 PROA
ATOM 1227 C ARG P 77 -1.061 -6.001 -14.230 0.00 0.00 PROA
ATOM 1228 O ARG P 77 -1.405 -6.478 -13.143 0.00 0.00 PROA
ATOM 1229 N LEU P 78 -0.048 -6.565 -14.925 0.00 0.00 PROA
ATOM 1230 HN LEU P 78 0.026 -6.435 -15.911 0.00 0.00 PROA
ATOM 1231 CA LEU P 78 0.794 -7.624 -14.390 0.00 0.00 PROA
ATOM 1232 HA LEU P 78 0.066 -8.376 -14.124 0.00 0.00 PROA
ATOM 1233 CB LEU P 78 1.738 -8.069 -15.433 0.00 0.00 PROA
ATOM 1234 HB1 LEU P 78 2.543 -7.305 -15.483 0.00 0.00 PROA
ATOM 1235 HB2 LEU P 78 1.261 -8.084 -16.436 0.00 0.00 PROA
ATOM 1236 CG LEU P 78 2.462 -9.450 -15.088 0.00 0.00 PROA
ATOM 1237 HG LEU P 78 3.048 -9.218 -14.174 0.00 0.00 PROA
ATOM 1238 CD1 LEU P 78 1.555 -10.582 -14.794 0.00 0.00 PROA
ATOM 1239 HD11 LEU P 78 0.937 -10.228 -13.941 0.00 0.00 PROA
ATOM 1240 HD12 LEU P 78 2.100 -11.459 -14.383 0.00 0.00 PROA
ATOM 1241 HD13 LEU P 78 0.964 -10.787 -15.712 0.00 0.00 PROA
ATOM 1242 CD2 LEU P 78 3.555 -9.818 -16.098 0.00 0.00 PROA
ATOM 1243 HD21 LEU P 78 4.198 -10.651 -15.743 0.00 0.00 PROA
ATOM 1244 HD22 LEU P 78 4.245 -8.962 -16.259 0.00 0.00 PROA
ATOM 1245 HD23 LEU P 78 3.072 -10.186 -17.028 0.00 0.00 PROA
ATOM 1246 C LEU P 78 1.441 -7.270 -13.090 0.00 0.00 PROA
ATOM 1247 O LEU P 78 1.302 -7.909 -12.091 0.00 0.00 PROA
ATOM 1248 N HSE P 79 2.071 -6.041 -13.085 0.00 0.00 PROA
ATOM 1249 HN HSE P 79 2.144 -5.579 -13.965 0.00 0.00 PROA
ATOM 1250 CA HSE P 79 2.781 -5.478 -11.945 0.00 0.00 PROA
ATOM 1251 HA HSE P 79 3.453 -6.241 -11.581 0.00 0.00 PROA
ATOM 1252 CB HSE P 79 3.538 -4.200 -12.364 0.00 0.00 PROA
ATOM 1253 HB1 HSE P 79 4.046 -3.730 -11.495 0.00 0.00 PROA
ATOM 1254 HB2 HSE P 79 2.817 -3.456 -12.764 0.00 0.00 PROA
ATOM 1255 ND1 HSE P 79 5.666 -5.287 -12.932 0.00 0.00 PROA
ATOM 1256 CG HSE P 79 4.726 -4.397 -13.321 0.00 0.00 PROA
ATOM 1257 CE1 HSE P 79 6.755 -5.105 -13.733 0.00 0.00 PROA
ATOM 1258 HE1 HSE P 79 7.738 -5.563 -13.625 0.00 0.00 PROA
ATOM 1259 NE2 HSE P 79 6.504 -4.048 -14.567 0.00 0.00 PROA
ATOM 1260 HE2 HSE P 79 7.067 -3.809 -15.358 0.00 0.00 PROA
ATOM 1261 CD2 HSE P 79 5.221 -3.601 -14.297 0.00 0.00 PROA
ATOM 1262 HD2 HSE P 79 4.752 -2.811 -14.871 0.00 0.00 PROA
ATOM 1263 C HSE P 79 1.868 -5.219 -10.740 0.00 0.00 PROA
ATOM 1264 O HSE P 79 2.142 -5.529 -9.616 0.00 0.00 PROA
ATOM 1265 N LEU P 80 0.655 -4.631 -10.962 0.00 0.00 PROA
ATOM 1266 HN LEU P 80 0.456 -4.237 -11.857 0.00 0.00 PROA
ATOM 1267 CA LEU P 80 -0.408 -4.627 -9.983 0.00 0.00 PROA
ATOM 1268 HA LEU P 80 -0.027 -4.038 -9.161 0.00 0.00 PROA
ATOM 1269 CB LEU P 80 -1.596 -3.782 -10.570 0.00 0.00 PROA
ATOM 1270 HB1 LEU P 80 -2.534 -3.683 -9.983 0.00 0.00 PROA
ATOM 1271 HB2 LEU P 80 -1.796 -4.175 -11.590 0.00 0.00 PROA
ATOM 1272 CG LEU P 80 -1.191 -2.324 -10.719 0.00 0.00 PROA
ATOM 1273 HG LEU P 80 -0.310 -2.274 -11.393 0.00 0.00 PROA
ATOM 1274 CD1 LEU P 80 -2.339 -1.480 -11.356 0.00 0.00 PROA
ATOM 1275 HD11 LEU P 80 -3.300 -1.472 -10.798 0.00 0.00 PROA
ATOM 1276 HD12 LEU P 80 -2.459 -1.836 -12.401 0.00 0.00 PROA
ATOM 1277 HD13 LEU P 80 -1.938 -0.460 -11.539 0.00 0.00 PROA

ATOM	1278	CD2	LEU	P	80	-0.833	-1.600	-9.429	0.00	0.00	PROA
ATOM	1279	HD21	LEU	P	80	-0.529	-0.558	-9.669	0.00	0.00	PROA
ATOM	1280	HD22	LEU	P	80	0.070	-2.069	-8.983	0.00	0.00	PROA
ATOM	1281	HD23	LEU	P	80	-1.722	-1.671	-8.768	0.00	0.00	PROA
ATOM	1282	C	LEU	P	80	-0.927	-6.000	-9.478	0.00	0.00	PROA
ATOM	1283	O	LEU	P	80	-1.186	-6.227	-8.305	0.00	0.00	PROA
ATOM	1284	N	SER	P	81	-1.062	-7.059	-10.378	0.00	0.00	PROA
ATOM	1285	HN	SER	P	81	-1.074	-6.913	-11.365	0.00	0.00	PROA
ATOM	1286	CA	SER	P	81	-1.409	-8.438	-10.036	0.00	0.00	PROA
ATOM	1287	HA	SER	P	81	-2.330	-8.370	-9.474	0.00	0.00	PROA
ATOM	1288	CB	SER	P	81	-1.614	-9.377	-11.269	0.00	0.00	PROA
ATOM	1289	HB1	SER	P	81	-0.640	-9.562	-11.771	0.00	0.00	PROA
ATOM	1290	HB2	SER	P	81	-2.254	-8.867	-12.021	0.00	0.00	PROA
ATOM	1291	OG	SER	P	81	-2.004	-10.707	-10.896	0.00	0.00	PROA
ATOM	1292	HG1	SER	P	81	-2.931	-10.639	-10.652	0.00	0.00	PROA
ATOM	1293	C	SER	P	81	-0.237	-8.995	-9.083	0.00	0.00	PROA
ATOM	1294	O	SER	P	81	-0.542	-9.350	-7.928	0.00	0.00	PROA
ATOM	1295	N	VAL	P	82	1.018	-8.808	-9.458	0.00	0.00	PROA
ATOM	1296	HN	VAL	P	82	1.107	-8.392	-10.359	0.00	0.00	PROA
ATOM	1297	CA	VAL	P	82	2.158	-9.138	-8.572	0.00	0.00	PROA
ATOM	1298	HA	VAL	P	82	2.015	-10.147	-8.214	0.00	0.00	PROA
ATOM	1299	CB	VAL	P	82	3.349	-8.800	-9.427	0.00	0.00	PROA
ATOM	1300	HB	VAL	P	82	3.286	-7.787	-9.879	0.00	0.00	PROA
ATOM	1301	CG1	VAL	P	82	4.692	-8.926	-8.748	0.00	0.00	PROA
ATOM	1302	HG11	VAL	P	82	4.848	-8.074	-8.052	0.00	0.00	PROA
ATOM	1303	HG12	VAL	P	82	5.400	-8.792	-9.593	0.00	0.00	PROA
ATOM	1304	HG13	VAL	P	82	4.704	-9.909	-8.231	0.00	0.00	PROA
ATOM	1305	CG2	VAL	P	82	3.477	-9.836	-10.619	0.00	0.00	PROA
ATOM	1306	HG21	VAL	P	82	3.971	-9.351	-11.488	0.00	0.00	PROA
ATOM	1307	HG22	VAL	P	82	2.552	-10.423	-10.801	0.00	0.00	PROA
ATOM	1308	HG23	VAL	P	82	4.016	-10.756	-10.307	0.00	0.00	PROA
ATOM	1309	C	VAL	P	82	2.154	-8.296	-7.298	0.00	0.00	PROA
ATOM	1310	O	VAL	P	82	2.470	-8.693	-6.246	0.00	0.00	PROA
ATOM	1311	N	ALA	P	83	1.730	-6.999	-7.374	0.00	0.00	PROA
ATOM	1312	HN	ALA	P	83	1.402	-6.611	-8.232	0.00	0.00	PROA
ATOM	1313	CA	ALA	P	83	1.610	-6.178	-6.148	0.00	0.00	PROA
ATOM	1314	HA	ALA	P	83	2.584	-6.229	-5.685	0.00	0.00	PROA
ATOM	1315	CB	ALA	P	83	1.280	-4.730	-6.573	0.00	0.00	PROA
ATOM	1316	HB1	ALA	P	83	2.142	-4.372	-7.176	0.00	0.00	PROA
ATOM	1317	HB2	ALA	P	83	1.119	-4.063	-5.699	0.00	0.00	PROA
ATOM	1318	HB3	ALA	P	83	0.311	-4.663	-7.111	0.00	0.00	PROA
ATOM	1319	C	ALA	P	83	0.553	-6.677	-5.178	0.00	0.00	PROA
ATOM	1320	O	ALA	P	83	0.754	-6.812	-4.000	0.00	0.00	PROA
ATOM	1321	N	ASP	P	84	-0.698	-7.047	-5.632	0.00	0.00	PROA
ATOM	1322	HN	ASP	P	84	-0.970	-6.795	-6.557	0.00	0.00	PROA
ATOM	1323	CA	ASP	P	84	-1.781	-7.652	-4.858	0.00	0.00	PROA
ATOM	1324	HA	ASP	P	84	-1.911	-6.981	-4.022	0.00	0.00	PROA
ATOM	1325	CB	ASP	P	84	-3.022	-7.750	-5.727	0.00	0.00	PROA
ATOM	1326	HB1	ASP	P	84	-3.730	-8.485	-5.289	0.00	0.00	PROA
ATOM	1327	HB2	ASP	P	84	-2.709	-7.996	-6.764	0.00	0.00	PROA
ATOM	1328	CG	ASP	P	84	-3.722	-6.405	-5.798	0.00	0.00	PROA
ATOM	1329	OD1	ASP	P	84	-3.659	-5.532	-4.891	0.00	0.00	PROA
ATOM	1330	OD2	ASP	P	84	-4.526	-6.184	-6.811	0.00	0.00	PROA
ATOM	1331	C	ASP	P	84	-1.308	-8.992	-4.278	0.00	0.00	PROA
ATOM	1332	O	ASP	P	84	-1.524	-9.242	-3.124	0.00	0.00	PROA
ATOM	1333	N	LEU	P	85	-0.582	-9.815	-5.087	0.00	0.00	PROA
ATOM	1334	HN	LEU	P	85	-0.336	-9.413	-5.966	0.00	0.00	PROA
ATOM	1335	CA	LEU	P	85	0.016	-11.085	-4.713	0.00	0.00	PROA
ATOM	1336	HA	LEU	P	85	-0.808	-11.699	-4.379	0.00	0.00	PROA
ATOM	1337	CB	LEU	P	85	0.640	-11.664	-6.014	0.00	0.00	PROA
ATOM	1338	HB1	LEU	P	85	1.324	-10.865	-6.373	0.00	0.00	PROA

```
ATOM   1339  HB2 LEU P  85      -0.074 -11.751  -6.861  0.00  0.00      PROA
ATOM   1340  CG  LEU P  85       1.296 -13.050  -5.946  0.00  0.00      PROA
ATOM   1341  HG  LEU P  85       2.135 -12.908  -5.232  0.00  0.00      PROA
ATOM   1342  CD1 LEU P  85       0.295 -14.145  -5.507  0.00  0.00      PROA
ATOM   1343 HD11 LEU P  85      -0.372 -14.122  -6.395  0.00  0.00      PROA
ATOM   1344 HD12 LEU P  85      -0.167 -13.865  -4.536  0.00  0.00      PROA
ATOM   1345 HD13 LEU P  85       0.774 -15.144  -5.428  0.00  0.00      PROA
ATOM   1346  CD2 LEU P  85       1.958 -13.405  -7.285  0.00  0.00      PROA
ATOM   1347 HD21 LEU P  85       1.131 -13.532  -8.016  0.00  0.00      PROA
ATOM   1348 HD22 LEU P  85       2.569 -14.327  -7.176  0.00  0.00      PROA
ATOM   1349 HD23 LEU P  85       2.571 -12.572  -7.692  0.00  0.00      PROA
ATOM   1350  C   LEU P  85       1.042 -11.029  -3.574  0.00  0.00      PROA
ATOM   1351  O   LEU P  85       0.964 -11.757  -2.568  0.00  0.00      PROA
ATOM   1352  N   LEU P  86       1.973 -10.056  -3.703  0.00  0.00      PROA
ATOM   1353  HN  LEU P  86       1.999  -9.670  -4.622  0.00  0.00      PROA
ATOM   1354  CA  LEU P  86       2.888  -9.645  -2.655  0.00  0.00      PROA
ATOM   1355  HA  LEU P  86       3.436 -10.532  -2.372  0.00  0.00      PROA
ATOM   1356  CB  LEU P  86       3.848  -8.632  -3.253  0.00  0.00      PROA
ATOM   1357  HB1 LEU P  86       4.359  -8.128  -2.405  0.00  0.00      PROA
ATOM   1358  HB2 LEU P  86       3.319  -7.951  -3.953  0.00  0.00      PROA
ATOM   1359  CG  LEU P  86       4.957  -9.233  -4.149  0.00  0.00      PROA
ATOM   1360  HG  LEU P  86       4.487 -10.013  -4.785  0.00  0.00      PROA
ATOM   1361  CD1 LEU P  86       5.552  -8.228  -5.108  0.00  0.00      PROA
ATOM   1362 HD11 LEU P  86       6.318  -8.760  -5.712  0.00  0.00      PROA
ATOM   1363 HD12 LEU P  86       6.081  -7.456  -4.510  0.00  0.00      PROA
ATOM   1364 HD13 LEU P  86       4.786  -7.714  -5.728  0.00  0.00      PROA
ATOM   1365  CD2 LEU P  86       6.083  -9.829  -3.283  0.00  0.00      PROA
ATOM   1366 HD21 LEU P  86       6.636  -8.977  -2.832  0.00  0.00      PROA
ATOM   1367 HD22 LEU P  86       6.801 -10.429  -3.882  0.00  0.00      PROA
ATOM   1368 HD23 LEU P  86       5.697 -10.555  -2.537  0.00  0.00      PROA
ATOM   1369  C   LEU P  86       2.179  -9.212  -1.285  0.00  0.00      PROA
ATOM   1370  O   LEU P  86       2.605  -9.640  -0.210  0.00  0.00      PROA
ATOM   1371  N   PHE P  87       1.076  -8.466  -1.387  0.00  0.00      PROA
ATOM   1372  HN  PHE P  87       0.716  -8.188  -2.274  0.00  0.00      PROA
ATOM   1373  CA  PHE P  87       0.333  -8.162  -0.155  0.00  0.00      PROA
ATOM   1374  HA  PHE P  87       1.088  -7.955   0.589  0.00  0.00      PROA
ATOM   1375  CB  PHE P  87      -0.631  -7.033  -0.416  0.00  0.00      PROA
ATOM   1376  HB1 PHE P  87      -1.334  -6.791   0.410  0.00  0.00      PROA
ATOM   1377  HB2 PHE P  87      -1.250  -7.281  -1.304  0.00  0.00      PROA
ATOM   1378  CG  PHE P  87       0.070  -5.712  -0.570  0.00  0.00      PROA
ATOM   1379  CD1 PHE P  87      -0.357  -4.865  -1.598  0.00  0.00      PROA
ATOM   1380  HD1 PHE P  87      -1.129  -5.194  -2.278  0.00  0.00      PROA
ATOM   1381  CE1 PHE P  87       0.278  -3.580  -1.785  0.00  0.00      PROA
ATOM   1382  HE1 PHE P  87      -0.093  -2.978  -2.602  0.00  0.00      PROA
ATOM   1383  CZ  PHE P  87       1.322  -3.165  -0.945  0.00  0.00      PROA
ATOM   1384  HZ  PHE P  87       1.672  -2.162  -1.139  0.00  0.00      PROA
ATOM   1385  CD2 PHE P  87       1.098  -5.288   0.338  0.00  0.00      PROA
ATOM   1386  HD2 PHE P  87       1.523  -5.863   1.148  0.00  0.00      PROA
ATOM   1387  CE2 PHE P  87       1.606  -3.939   0.160  0.00  0.00      PROA
ATOM   1388  HE2 PHE P  87       2.326  -3.609   0.895  0.00  0.00      PROA
ATOM   1389  C   PHE P  87      -0.420  -9.356   0.393  0.00  0.00      PROA
ATOM   1390  O   PHE P  87      -0.468  -9.578   1.603  0.00  0.00      PROA
ATOM   1391  N   VAL P  88      -1.067 -10.120  -0.506  0.00  0.00      PROA
ATOM   1392  HN  VAL P  88      -1.060  -9.855  -1.467  0.00  0.00      PROA
ATOM   1393  CA  VAL P  88      -1.841 -11.306  -0.178  0.00  0.00      PROA
ATOM   1394  HA  VAL P  88      -2.672 -11.088   0.476  0.00  0.00      PROA
ATOM   1395  CB  VAL P  88      -2.484 -11.954  -1.406  0.00  0.00      PROA
ATOM   1396  HB  VAL P  88      -1.763 -11.801  -2.237  0.00  0.00      PROA
ATOM   1397  CG1 VAL P  88      -2.813 -13.466  -1.276  0.00  0.00      PROA
ATOM   1398 HG11 VAL P  88      -3.377 -13.656  -0.338  0.00  0.00      PROA
ATOM   1399 HG12 VAL P  88      -1.910 -14.112  -1.233  0.00  0.00      PROA
```

ATOM 1400 HG13 VAL P 88 -3.306 -13.831 -2.202 0.00 0.00 PROA
ATOM 1401 CG2 VAL P 88 -3.718 -11.066 -1.816 0.00 0.00 PROA
ATOM 1402 HG21 VAL P 88 -4.106 -11.293 -2.832 0.00 0.00 PROA
ATOM 1403 HG22 VAL P 88 -3.369 -10.012 -1.832 0.00 0.00 PROA
ATOM 1404 HG23 VAL P 88 -4.490 -11.237 -1.035 0.00 0.00 PROA
ATOM 1405 C VAL P 88 -1.140 -12.398 0.713 0.00 0.00 PROA
ATOM 1406 O VAL P 88 -1.737 -12.922 1.645 0.00 0.00 PROA
ATOM 1407 N ILE P 89 0.191 -12.692 0.413 0.00 0.00 PROA
ATOM 1408 HN ILE P 89 0.556 -12.213 -0.381 0.00 0.00 PROA
ATOM 1409 CA ILE P 89 0.971 -13.732 1.110 0.00 0.00 PROA
ATOM 1410 HA ILE P 89 0.407 -14.653 1.104 0.00 0.00 PROA
ATOM 1411 CB ILE P 89 2.332 -14.065 0.477 0.00 0.00 PROA
ATOM 1412 HB ILE P 89 2.071 -14.176 -0.597 0.00 0.00 PROA
ATOM 1413 CG2 ILE P 89 3.269 -12.804 0.479 0.00 0.00 PROA
ATOM 1414 HG21 ILE P 89 2.702 -11.927 0.100 0.00 0.00 PROA
ATOM 1415 HG22 ILE P 89 4.199 -12.948 -0.112 0.00 0.00 PROA
ATOM 1416 HG23 ILE P 89 3.658 -12.515 1.479 0.00 0.00 PROA
ATOM 1417 CG1 ILE P 89 2.995 -15.356 0.896 0.00 0.00 PROA
ATOM 1418 HG11 ILE P 89 3.448 -15.394 1.910 0.00 0.00 PROA
ATOM 1419 HG12 ILE P 89 3.802 -15.498 0.146 0.00 0.00 PROA
ATOM 1420 CD ILE P 89 2.075 -16.626 0.884 0.00 0.00 PROA
ATOM 1421 HD1 ILE P 89 2.688 -17.469 1.266 0.00 0.00 PROA
ATOM 1422 HD2 ILE P 89 1.700 -16.781 -0.151 0.00 0.00 PROA
ATOM 1423 HD3 ILE P 89 1.294 -16.548 1.670 0.00 0.00 PROA
ATOM 1424 C ILE P 89 1.162 -13.475 2.578 0.00 0.00 PROA
ATOM 1425 O ILE P 89 1.322 -14.371 3.413 0.00 0.00 PROA
ATOM 1426 N THR P 90 1.232 -12.173 2.982 0.00 0.00 PROA
ATOM 1427 HN THR P 90 1.205 -11.561 2.196 0.00 0.00 PROA
ATOM 1428 CA THR P 90 1.346 -11.706 4.336 0.00 0.00 PROA
ATOM 1429 HA THR P 90 2.304 -12.095 4.648 0.00 0.00 PROA
ATOM 1430 CB THR P 90 1.538 -10.232 4.471 0.00 0.00 PROA
ATOM 1431 HB THR P 90 1.734 -9.960 5.530 0.00 0.00 PROA
ATOM 1432 OG1 THR P 90 0.394 -9.440 4.152 0.00 0.00 PROA
ATOM 1433 HG1 THR P 90 0.204 -9.564 3.220 0.00 0.00 PROA
ATOM 1434 CG2 THR P 90 2.688 -9.697 3.690 0.00 0.00 PROA
ATOM 1435 HG21 THR P 90 3.305 -8.938 4.217 0.00 0.00 PROA
ATOM 1436 HG22 THR P 90 2.383 -9.245 2.722 0.00 0.00 PROA
ATOM 1437 HG23 THR P 90 3.398 -10.506 3.414 0.00 0.00 PROA
ATOM 1438 C THR P 90 0.229 -12.166 5.305 0.00 0.00 PROA
ATOM 1439 O THR P 90 0.448 -12.489 6.458 0.00 0.00 PROA
ATOM 1440 N LEU P 91 -1.011 -12.243 4.756 0.00 0.00 PROA
ATOM 1441 HN LEU P 91 -1.146 -12.048 3.788 0.00 0.00 PROA
ATOM 1442 CA LEU P 91 -2.320 -12.497 5.402 0.00 0.00 PROA
ATOM 1443 HA LEU P 91 -2.447 -11.816 6.231 0.00 0.00 PROA
ATOM 1444 CB LEU P 91 -3.567 -12.490 4.481 0.00 0.00 PROA
ATOM 1445 HB1 LEU P 91 -4.462 -12.763 5.080 0.00 0.00 PROA
ATOM 1446 HB2 LEU P 91 -3.437 -13.293 3.724 0.00 0.00 PROA
ATOM 1447 CG LEU P 91 -3.829 -11.103 3.847 0.00 0.00 PROA
ATOM 1448 HG LEU P 91 -2.884 -10.750 3.382 0.00 0.00 PROA
ATOM 1449 CD1 LEU P 91 -4.919 -11.064 2.836 0.00 0.00 PROA
ATOM 1450 HD11 LEU P 91 -4.957 -10.136 2.227 0.00 0.00 PROA
ATOM 1451 HD12 LEU P 91 -5.953 -11.290 3.172 0.00 0.00 PROA
ATOM 1452 HD13 LEU P 91 -4.581 -11.795 2.071 0.00 0.00 PROA
ATOM 1453 CD2 LEU P 91 -4.055 -10.054 4.938 0.00 0.00 PROA
ATOM 1454 HD21 LEU P 91 -4.626 -10.420 5.819 0.00 0.00 PROA
ATOM 1455 HD22 LEU P 91 -4.572 -9.176 4.496 0.00 0.00 PROA
ATOM 1456 HD23 LEU P 91 -3.156 -9.500 5.282 0.00 0.00 PROA
ATOM 1457 C LEU P 91 -2.381 -13.814 6.223 0.00 0.00 PROA
ATOM 1458 O LEU P 91 -2.786 -13.690 7.351 0.00 0.00 PROA
ATOM 1459 N PRO P 92 -1.961 -14.975 5.799 0.00 0.00 PROA
ATOM 1460 CD PRO P 92 -1.774 -15.327 4.393 0.00 0.00 PROA

```
ATOM   1461  HD1 PRO P  92      -1.239 -14.580   3.769  0.00  0.00      PROA
ATOM   1462  HD2 PRO P  92      -2.803 -15.446   3.991  0.00  0.00      PROA
ATOM   1463  CA  PRO P  92      -2.126 -16.148   6.605  0.00  0.00      PROA
ATOM   1464  HA  PRO P  92      -3.163 -16.221   6.901  0.00  0.00      PROA
ATOM   1465  CB  PRO P  92      -1.756 -17.354   5.676  0.00  0.00      PROA
ATOM   1466  HB1 PRO P  92      -2.617 -17.971   5.340  0.00  0.00      PROA
ATOM   1467  HB2 PRO P  92      -1.039 -18.061   6.144  0.00  0.00      PROA
ATOM   1468  CG  PRO P  92      -1.026 -16.634   4.459  0.00  0.00      PROA
ATOM   1469  HG1 PRO P  92       0.013 -16.539   4.839  0.00  0.00      PROA
ATOM   1470  HG2 PRO P  92      -1.025 -17.183   3.493  0.00  0.00      PROA
ATOM   1471  C   PRO P  92      -1.255 -16.250   7.799  0.00  0.00      PROA
ATOM   1472  O   PRO P  92      -1.687 -16.758   8.867  0.00  0.00      PROA
ATOM   1473  N   PHE P  93      -0.021 -15.720   7.669  0.00  0.00      PROA
ATOM   1474  HN  PHE P  93       0.331 -15.389   6.796  0.00  0.00      PROA
ATOM   1475  CA  PHE P  93       0.877 -15.490   8.760  0.00  0.00      PROA
ATOM   1476  HA  PHE P  93       1.094 -16.459   9.186  0.00  0.00      PROA
ATOM   1477  CB  PHE P  93       2.268 -15.106   8.244  0.00  0.00      PROA
ATOM   1478  HB1 PHE P  93       2.870 -15.028   9.174  0.00  0.00      PROA
ATOM   1479  HB2 PHE P  93       2.352 -14.187   7.624  0.00  0.00      PROA
ATOM   1480  CG  PHE P  93       2.794 -16.178   7.382  0.00  0.00      PROA
ATOM   1481  CD1 PHE P  93       3.237 -17.369   8.007  0.00  0.00      PROA
ATOM   1482  HD1 PHE P  93       3.239 -17.590   9.064  0.00  0.00      PROA
ATOM   1483  CE1 PHE P  93       3.509 -18.465   7.191  0.00  0.00      PROA
ATOM   1484  HE1 PHE P  93       3.670 -19.433   7.641  0.00  0.00      PROA
ATOM   1485  CZ  PHE P  93       3.458 -18.342   5.841  0.00  0.00      PROA
ATOM   1486  HZ  PHE P  93       3.555 -19.245   5.255  0.00  0.00      PROA
ATOM   1487  CD2 PHE P  93       2.685 -16.080   5.976  0.00  0.00      PROA
ATOM   1488  HD2 PHE P  93       2.309 -15.182   5.509  0.00  0.00      PROA
ATOM   1489  CE2 PHE P  93       3.101 -17.164   5.211  0.00  0.00      PROA
ATOM   1490  HE2 PHE P  93       3.191 -17.183   4.135  0.00  0.00      PROA
ATOM   1491  C   PHE P  93       0.279 -14.628   9.839  0.00  0.00      PROA
ATOM   1492  O   PHE P  93       0.312 -15.119  10.960  0.00  0.00      PROA
ATOM   1493  N   TRP P  94      -0.311 -13.473   9.497  0.00  0.00      PROA
ATOM   1494  HN  TRP P  94      -0.135 -13.079   8.598  0.00  0.00      PROA
ATOM   1495  CA  TRP P  94      -0.967 -12.619  10.464  0.00  0.00      PROA
ATOM   1496  HA  TRP P  94      -0.419 -12.549  11.392  0.00  0.00      PROA
ATOM   1497  CB  TRP P  94      -1.198 -11.271   9.777  0.00  0.00      PROA
ATOM   1498  HB1 TRP P  94      -1.691 -10.547  10.460  0.00  0.00      PROA
ATOM   1499  HB2 TRP P  94      -1.787 -11.442   8.851  0.00  0.00      PROA
ATOM   1500  CG  TRP P  94       0.016 -10.586   9.363  0.00  0.00      PROA
ATOM   1501  CD1 TRP P  94       0.172 -10.035   8.147  0.00  0.00      PROA
ATOM   1502  HD1 TRP P  94      -0.568  -9.995   7.360  0.00  0.00      PROA
ATOM   1503  NE1 TRP P  94       1.452  -9.500   8.119  0.00  0.00      PROA
ATOM   1504  HE1 TRP P  94       1.770  -8.981   7.356  0.00  0.00      PROA
ATOM   1505  CE2 TRP P  94       2.072  -9.692   9.250  0.00  0.00      PROA
ATOM   1506  CD2 TRP P  94       1.221 -10.445  10.069  0.00  0.00      PROA
ATOM   1507  CE3 TRP P  94       1.623 -10.707  11.368  0.00  0.00      PROA
ATOM   1508  HE3 TRP P  94       0.935 -11.134  12.083  0.00  0.00      PROA
ATOM   1509  CZ3 TRP P  94       2.865 -10.380  11.762  0.00  0.00      PROA
ATOM   1510  HZ3 TRP P  94       3.167 -10.672  12.757  0.00  0.00      PROA
ATOM   1511  CZ2 TRP P  94       3.308  -9.347   9.641  0.00  0.00      PROA
ATOM   1512  HZ2 TRP P  94       4.053  -8.795   9.088  0.00  0.00      PROA
ATOM   1513  CH2 TRP P  94       3.776  -9.765  10.888  0.00  0.00      PROA
ATOM   1514  HH2 TRP P  94       4.749  -9.478  11.258  0.00  0.00      PROA
ATOM   1515  C   TRP P  94      -2.310 -13.099  10.986  0.00  0.00      PROA
ATOM   1516  O   TRP P  94      -2.602 -12.884  12.146  0.00  0.00      PROA
ATOM   1517  N   ALA P  95      -3.112 -13.845  10.206  0.00  0.00      PROA
ATOM   1518  HN  ALA P  95      -2.870 -13.890   9.240  0.00  0.00      PROA
ATOM   1519  CA  ALA P  95      -4.366 -14.426  10.625  0.00  0.00      PROA
ATOM   1520  HA  ALA P  95      -5.005 -13.728  11.147  0.00  0.00      PROA
ATOM   1521  CB  ALA P  95      -5.283 -14.878   9.451  0.00  0.00      PROA
```

ATOM 1522 HB1 ALA P 95 -5.759 -14.052 8.881 0.00 0.00 PROA
ATOM 1523 HB2 ALA P 95 -6.077 -15.541 9.857 0.00 0.00 PROA
ATOM 1524 HB3 ALA P 95 -4.629 -15.380 8.705 0.00 0.00 PROA
ATOM 1525 C ALA P 95 -4.214 -15.535 11.737 0.00 0.00 PROA
ATOM 1526 O ALA P 95 -5.040 -15.720 12.600 0.00 0.00 PROA
ATOM 1527 N VAL P 96 -3.167 -16.291 11.764 0.00 0.00 PROA
ATOM 1528 HN VAL P 96 -2.573 -16.371 10.967 0.00 0.00 PROA
ATOM 1529 CA VAL P 96 -2.791 -17.099 12.898 0.00 0.00 PROA
ATOM 1530 HA VAL P 96 -3.736 -17.549 13.165 0.00 0.00 PROA
ATOM 1531 CB VAL P 96 -1.801 -18.211 12.583 0.00 0.00 PROA
ATOM 1532 HB VAL P 96 -0.821 -17.755 12.325 0.00 0.00 PROA
ATOM 1533 CG1 VAL P 96 -1.614 -19.164 13.743 0.00 0.00 PROA
ATOM 1534 HG11 VAL P 96 -1.003 -18.783 14.589 0.00 0.00 PROA
ATOM 1535 HG12 VAL P 96 -1.187 -20.151 13.467 0.00 0.00 PROA
ATOM 1536 HG13 VAL P 96 -2.617 -19.287 14.204 0.00 0.00 PROA
ATOM 1537 CG2 VAL P 96 -2.373 -18.963 11.457 0.00 0.00 PROA
ATOM 1538 HG21 VAL P 96 -3.362 -19.340 11.796 0.00 0.00 PROA
ATOM 1539 HG22 VAL P 96 -1.712 -19.831 11.250 0.00 0.00 PROA
ATOM 1540 HG23 VAL P 96 -2.352 -18.372 10.516 0.00 0.00 PROA
ATOM 1541 C VAL P 96 -2.405 -16.347 14.187 0.00 0.00 PROA
ATOM 1542 O VAL P 96 -2.857 -16.532 15.284 0.00 0.00 PROA
ATOM 1543 N ASP P 97 -1.523 -15.297 14.161 0.00 0.00 PROA
ATOM 1544 HN ASP P 97 -1.122 -14.998 13.298 0.00 0.00 PROA
ATOM 1545 CA ASP P 97 -1.175 -14.428 15.183 0.00 0.00 PROA
ATOM 1546 HA ASP P 97 -0.924 -15.056 16.025 0.00 0.00 PROA
ATOM 1547 CB ASP P 97 0.049 -13.638 14.683 0.00 0.00 PROA
ATOM 1548 HB1 ASP P 97 -0.193 -13.378 13.630 0.00 0.00 PROA
ATOM 1549 HB2 ASP P 97 0.924 -14.317 14.772 0.00 0.00 PROA
ATOM 1550 CG ASP P 97 0.143 -12.341 15.470 0.00 0.00 PROA
ATOM 1551 OD1 ASP P 97 0.458 -12.313 16.718 0.00 0.00 PROA
ATOM 1552 OD2 ASP P 97 -0.287 -11.324 14.901 0.00 0.00 PROA
ATOM 1553 C ASP P 97 -2.365 -13.683 15.880 0.00 0.00 PROA
ATOM 1554 O ASP P 97 -2.322 -13.438 17.084 0.00 0.00 PROA
ATOM 1555 N ALA P 98 -3.433 -13.261 15.089 0.00 0.00 PROA
ATOM 1556 HN ALA P 98 -3.373 -13.458 14.113 0.00 0.00 PROA
ATOM 1557 CA ALA P 98 -4.694 -12.806 15.617 0.00 0.00 PROA
ATOM 1558 HA ALA P 98 -4.539 -11.858 16.110 0.00 0.00 PROA
ATOM 1559 CB ALA P 98 -5.604 -12.499 14.455 0.00 0.00 PROA
ATOM 1560 HB1 ALA P 98 -5.915 -13.401 13.886 0.00 0.00 PROA
ATOM 1561 HB2 ALA P 98 -5.173 -11.772 13.734 0.00 0.00 PROA
ATOM 1562 HB3 ALA P 98 -6.547 -12.028 14.804 0.00 0.00 PROA
ATOM 1563 C ALA P 98 -5.505 -13.667 16.617 0.00 0.00 PROA
ATOM 1564 O ALA P 98 -6.027 -13.230 17.615 0.00 0.00 PROA
ATOM 1565 N VAL P 99 -5.583 -14.928 16.193 0.00 0.00 PROA
ATOM 1566 HN VAL P 99 -5.157 -15.315 15.379 0.00 0.00 PROA
ATOM 1567 CA VAL P 99 -6.363 -15.869 16.939 0.00 0.00 PROA
ATOM 1568 HA VAL P 99 -6.937 -15.370 17.705 0.00 0.00 PROA
ATOM 1569 CB VAL P 99 -7.318 -16.505 15.863 0.00 0.00 PROA
ATOM 1570 HB VAL P 99 -7.829 -15.757 15.219 0.00 0.00 PROA
ATOM 1571 CG1 VAL P 99 -6.642 -17.532 14.985 0.00 0.00 PROA
ATOM 1572 HG11 VAL P 99 -5.811 -16.985 14.490 0.00 0.00 PROA
ATOM 1573 HG12 VAL P 99 -7.427 -17.902 14.292 0.00 0.00 PROA
ATOM 1574 HG13 VAL P 99 -6.261 -18.323 15.666 0.00 0.00 PROA
ATOM 1575 CG2 VAL P 99 -8.587 -17.108 16.539 0.00 0.00 PROA
ATOM 1576 HG21 VAL P 99 -9.186 -16.493 17.245 0.00 0.00 PROA
ATOM 1577 HG22 VAL P 99 -8.313 -17.971 17.183 0.00 0.00 PROA
ATOM 1578 HG23 VAL P 99 -9.237 -17.507 15.732 0.00 0.00 PROA
ATOM 1579 C VAL P 99 -5.565 -16.853 17.760 0.00 0.00 PROA
ATOM 1580 O VAL P 99 -6.111 -17.677 18.481 0.00 0.00 PROA
ATOM 1581 N ALA P 100 -4.266 -16.948 17.562 0.00 0.00 PROA
ATOM 1582 HN ALA P 100 -3.815 -16.472 16.811 0.00 0.00 PROA

ATOM	1583	CA	ALA	P	100	-3.489	-17.801	18.361	0.00	0.00	PROA
ATOM	1584	HA	ALA	P	100	-3.871	-18.021	19.347	0.00	0.00	PROA
ATOM	1585	CB	ALA	P	100	-3.331	-19.147	17.776	0.00	0.00	PROA
ATOM	1586	HB1	ALA	P	100	-4.302	-19.659	17.605	0.00	0.00	PROA
ATOM	1587	HB2	ALA	P	100	-2.761	-19.778	18.490	0.00	0.00	PROA
ATOM	1588	HB3	ALA	P	100	-2.847	-19.201	16.778	0.00	0.00	PROA
ATOM	1589	C	ALA	P	100	-2.084	-17.246	18.685	0.00	0.00	PROA
ATOM	1590	O	ALA	P	100	-1.900	-16.472	19.630	0.00	0.00	PROA
ATOM	1591	N	ASN	P	101	-1.006	-17.648	17.863	0.00	0.00	PROA
ATOM	1592	HN	ASN	P	101	-1.123	-18.130	16.998	0.00	0.00	PROA
ATOM	1593	CA	ASN	P	101	0.347	-17.537	18.431	0.00	0.00	PROA
ATOM	1594	HA	ASN	P	101	0.411	-16.586	18.939	0.00	0.00	PROA
ATOM	1595	CB	ASN	P	101	0.580	-18.768	19.431	0.00	0.00	PROA
ATOM	1596	HB1	ASN	P	101	0.380	-19.701	18.861	0.00	0.00	PROA
ATOM	1597	HB2	ASN	P	101	-0.208	-18.718	20.212	0.00	0.00	PROA
ATOM	1598	CG	ASN	P	101	1.988	-18.843	19.985	0.00	0.00	PROA
ATOM	1599	OD1	ASN	P	101	2.717	-17.859	20.092	0.00	0.00	PROA
ATOM	1600	ND2	ASN	P	101	2.387	-20.075	20.388	0.00	0.00	PROA
ATOM	1601	HD21	ASN	P	101	3.351	-20.153	20.642	0.00	0.00	PROA
ATOM	1602	HD22	ASN	P	101	1.714	-20.753	20.684	0.00	0.00	PROA
ATOM	1603	C	ASN	P	101	1.379	-17.489	17.263	0.00	0.00	PROA
ATOM	1604	O	ASN	P	101	1.109	-17.916	16.180	0.00	0.00	PROA
ATOM	1605	N	TRP	P	102	2.570	-16.988	17.294	0.00	0.00	PROA
ATOM	1606	HN	TRP	P	102	3.004	-16.924	18.189	0.00	0.00	PROA
ATOM	1607	CA	TRP	P	102	3.459	-17.081	16.125	0.00	0.00	PROA
ATOM	1608	HA	TRP	P	102	2.947	-16.998	15.178	0.00	0.00	PROA
ATOM	1609	CB	TRP	P	102	4.497	-15.894	16.113	0.00	0.00	PROA
ATOM	1610	HB1	TRP	P	102	5.176	-15.814	16.989	0.00	0.00	PROA
ATOM	1611	HB2	TRP	P	102	3.880	-14.980	16.256	0.00	0.00	PROA
ATOM	1612	CG	TRP	P	102	5.346	-15.809	14.861	0.00	0.00	PROA
ATOM	1613	CD1	TRP	P	102	6.607	-16.238	14.605	0.00	0.00	PROA
ATOM	1614	HD1	TRP	P	102	7.341	-16.595	15.313	0.00	0.00	PROA
ATOM	1615	NE1	TRP	P	102	6.826	-16.133	13.197	0.00	0.00	PROA
ATOM	1616	HE1	TRP	P	102	7.693	-16.256	12.765	0.00	0.00	PROA
ATOM	1617	CE2	TRP	P	102	5.780	-15.495	12.603	0.00	0.00	PROA
ATOM	1618	CD2	TRP	P	102	4.884	-15.204	13.642	0.00	0.00	PROA
ATOM	1619	CE3	TRP	P	102	3.698	-14.523	13.320	0.00	0.00	PROA
ATOM	1620	HE3	TRP	P	102	2.879	-14.243	13.967	0.00	0.00	PROA
ATOM	1621	CZ3	TRP	P	102	3.529	-13.951	12.024	0.00	0.00	PROA
ATOM	1622	HZ3	TRP	P	102	2.581	-13.440	11.946	0.00	0.00	PROA
ATOM	1623	CZ2	TRP	P	102	5.622	-14.921	11.345	0.00	0.00	PROA
ATOM	1624	HZ2	TRP	P	102	6.443	-15.163	10.687	0.00	0.00	PROA
ATOM	1625	CH2	TRP	P	102	4.445	-14.190	11.052	0.00	0.00	PROA
ATOM	1626	HH2	TRP	P	102	4.248	-13.910	10.027	0.00	0.00	PROA
ATOM	1627	C	TRP	P	102	4.326	-18.363	16.123	0.00	0.00	PROA
ATOM	1628	O	TRP	P	102	5.295	-18.508	16.852	0.00	0.00	PROA
ATOM	1629	N	TYR	P	103	4.088	-19.290	15.198	0.00	0.00	PROA
ATOM	1630	HN	TYR	P	103	3.238	-19.247	14.678	0.00	0.00	PROA
ATOM	1631	CA	TYR	P	103	4.662	-20.605	15.046	0.00	0.00	PROA
ATOM	1632	HA	TYR	P	103	5.218	-20.904	15.922	0.00	0.00	PROA
ATOM	1633	CB	TYR	P	103	3.562	-21.608	14.606	0.00	0.00	PROA
ATOM	1634	HB1	TYR	P	103	3.971	-22.602	14.327	0.00	0.00	PROA
ATOM	1635	HB2	TYR	P	103	3.140	-21.100	13.713	0.00	0.00	PROA
ATOM	1636	CG	TYR	P	103	2.533	-21.738	15.677	0.00	0.00	PROA
ATOM	1637	CD1	TYR	P	103	2.760	-22.367	16.863	0.00	0.00	PROA
ATOM	1638	HD1	TYR	P	103	3.741	-22.728	17.136	0.00	0.00	PROA
ATOM	1639	CE1	TYR	P	103	1.810	-22.582	17.837	0.00	0.00	PROA
ATOM	1640	HE1	TYR	P	103	2.064	-23.026	18.788	0.00	0.00	PROA
ATOM	1641	CZ	TYR	P	103	0.473	-22.245	17.516	0.00	0.00	PROA
ATOM	1642	OH	TYR	P	103	-0.635	-22.557	18.310	0.00	0.00	PROA
ATOM	1643	HH	TYR	P	103	-0.400	-23.382	18.742	0.00	0.00	PROA

```
ATOM   1644  CD2 TYR P 103       1.231 -21.288  15.450  0.00  0.00      PROA
ATOM   1645  HD2 TYR P 103       1.013 -20.705  14.568  0.00  0.00      PROA
ATOM   1646  CE2 TYR P 103       0.249 -21.511  16.324  0.00  0.00      PROA
ATOM   1647  HE2 TYR P 103      -0.756 -21.201  16.083  0.00  0.00      PROA
ATOM   1648  C   TYR P 103       5.726 -20.690  14.032  0.00  0.00      PROA
ATOM   1649  O   TYR P 103       6.386 -21.727  13.922  0.00  0.00      PROA
ATOM   1650  N   PHE P 104       5.860 -19.597  13.284  0.00  0.00      PROA
ATOM   1651  HN  PHE P 104       5.349 -18.783  13.549  0.00  0.00      PROA
ATOM   1652  CA  PHE P 104       6.425 -19.782  11.994  0.00  0.00      PROA
ATOM   1653  HA  PHE P 104       6.526 -20.795  11.634  0.00  0.00      PROA
ATOM   1654  CB  PHE P 104       5.669 -18.769  10.989  0.00  0.00      PROA
ATOM   1655  HB1 PHE P 104       5.982 -18.874   9.928  0.00  0.00      PROA
ATOM   1656  HB2 PHE P 104       5.926 -17.749  11.347  0.00  0.00      PROA
ATOM   1657  CG  PHE P 104       4.226 -19.084  11.000  0.00  0.00      PROA
ATOM   1658  CD1 PHE P 104       3.756 -20.404  10.665  0.00  0.00      PROA
ATOM   1659  HD1 PHE P 104       4.535 -21.126  10.473  0.00  0.00      PROA
ATOM   1660  CE1 PHE P 104       2.398 -20.704  10.814  0.00  0.00      PROA
ATOM   1661  HE1 PHE P 104       2.024 -21.716  10.770  0.00  0.00      PROA
ATOM   1662  CZ  PHE P 104       1.474 -19.695  11.165  0.00  0.00      PROA
ATOM   1663  HZ  PHE P 104       0.415 -19.908  11.185  0.00  0.00      PROA
ATOM   1664  CD2 PHE P 104       3.277 -18.138  11.454  0.00  0.00      PROA
ATOM   1665  HD2 PHE P 104       3.435 -17.141  11.839  0.00  0.00      PROA
ATOM   1666  CE2 PHE P 104       1.962 -18.408  11.446  0.00  0.00      PROA
ATOM   1667  HE2 PHE P 104       1.287 -17.603  11.696  0.00  0.00      PROA
ATOM   1668  C   PHE P 104       7.946 -19.225  11.938  0.00  0.00      PROA
ATOM   1669  O   PHE P 104       8.604 -19.248  10.901  0.00  0.00      PROA
ATOM   1670  N   GLY P 105       8.506 -18.946  13.123  0.00  0.00      PROA
ATOM   1671  HN  GLY P 105       7.886 -18.899  13.903  0.00  0.00      PROA
ATOM   1672  CA  GLY P 105       9.926 -18.590  13.327  0.00  0.00      PROA
ATOM   1673  HA1 GLY P 105      10.469 -19.426  12.911  0.00  0.00      PROA
ATOM   1674  HA2 GLY P 105      10.015 -18.558  14.403  0.00  0.00      PROA
ATOM   1675  C   GLY P 105      10.392 -17.245  12.693  0.00  0.00      PROA
ATOM   1676  O   GLY P 105       9.646 -16.668  11.878  0.00  0.00      PROA
ATOM   1677  N   ASN P 106      11.580 -16.767  13.052  0.00  0.00      PROA
ATOM   1678  HN  ASN P 106      12.087 -17.349  13.683  0.00  0.00      PROA
ATOM   1679  CA  ASN P 106      12.255 -15.479  12.682  0.00  0.00      PROA
ATOM   1680  HA  ASN P 106      11.649 -14.676  13.075  0.00  0.00      PROA
ATOM   1681  CB  ASN P 106      13.660 -15.605  13.372  0.00  0.00      PROA
ATOM   1682  HB1 ASN P 106      14.428 -14.982  12.865  0.00  0.00      PROA
ATOM   1683  HB2 ASN P 106      13.949 -16.677  13.318  0.00  0.00      PROA
ATOM   1684  CG  ASN P 106      13.563 -15.054  14.780  0.00  0.00      PROA
ATOM   1685  OD1 ASN P 106      13.302 -13.888  14.964  0.00  0.00      PROA
ATOM   1686  ND2 ASN P 106      13.806 -15.924  15.824  0.00  0.00      PROA
ATOM   1687 HD21 ASN P 106      13.882 -15.523  16.737  0.00  0.00      PROA
ATOM   1688 HD22 ASN P 106      14.271 -16.725  15.446  0.00  0.00      PROA
ATOM   1689  C   ASN P 106      12.482 -15.259  11.175  0.00  0.00      PROA
ATOM   1690  O   ASN P 106      12.260 -14.192  10.600  0.00  0.00      PROA
ATOM   1691  N   PHE P 107      12.901 -16.318  10.426  0.00  0.00      PROA
ATOM   1692  HN  PHE P 107      13.032 -17.200  10.873  0.00  0.00      PROA
ATOM   1693  CA  PHE P 107      13.159 -16.068   9.033  0.00  0.00      PROA
ATOM   1694  HA  PHE P 107      13.846 -15.241   8.931  0.00  0.00      PROA
ATOM   1695  CB  PHE P 107      13.681 -17.298   8.342  0.00  0.00      PROA
ATOM   1696  HB1 PHE P 107      12.930 -18.113   8.423  0.00  0.00      PROA
ATOM   1697  HB2 PHE P 107      14.638 -17.511   8.864  0.00  0.00      PROA
ATOM   1698  CG  PHE P 107      14.038 -17.216   6.892  0.00  0.00      PROA
ATOM   1699  CD1 PHE P 107      13.130 -17.619   5.899  0.00  0.00      PROA
ATOM   1700  HD1 PHE P 107      12.113 -17.814   6.204  0.00  0.00      PROA
ATOM   1701  CE1 PHE P 107      13.473 -17.681   4.559  0.00  0.00      PROA
ATOM   1702  HE1 PHE P 107      12.707 -18.058   3.897  0.00  0.00      PROA
ATOM   1703  CZ  PHE P 107      14.717 -17.237   4.097  0.00  0.00      PROA
ATOM   1704  HZ  PHE P 107      14.934 -17.097   3.048  0.00  0.00      PROA
```

```
ATOM 1705 CD2 PHE P 107 15.317 -16.785 6.443 0.00 0.00 PROA
ATOM 1706 HD2 PHE P 107 16.005 -16.374 7.167 0.00 0.00 PROA
ATOM 1707 CE2 PHE P 107 15.631 -16.747 5.057 0.00 0.00 PROA
ATOM 1708 HE2 PHE P 107 16.587 -16.372 4.725 0.00 0.00 PROA
ATOM 1709 C PHE P 107 11.833 -15.716 8.303 0.00 0.00 PROA
ATOM 1710 O PHE P 107 11.798 -14.745 7.546 0.00 0.00 PROA
ATOM 1711 N LEU P 108 10.664 -16.431 8.536 0.00 0.00 PROA
ATOM 1712 HN LEU P 108 10.699 -17.214 9.152 0.00 0.00 PROA
ATOM 1713 CA LEU P 108 9.406 -15.964 7.962 0.00 0.00 PROA
ATOM 1714 HA LEU P 108 9.659 -15.960 6.912 0.00 0.00 PROA
ATOM 1715 CB LEU P 108 8.305 -17.119 7.972 0.00 0.00 PROA
ATOM 1716 HB1 LEU P 108 7.399 -16.780 7.426 0.00 0.00 PROA
ATOM 1717 HB2 LEU P 108 8.066 -17.367 9.029 0.00 0.00 PROA
ATOM 1718 CG LEU P 108 8.647 -18.490 7.323 0.00 0.00 PROA
ATOM 1719 HG LEU P 108 9.418 -18.975 7.959 0.00 0.00 PROA
ATOM 1720 CD1 LEU P 108 7.523 -19.500 7.454 0.00 0.00 PROA
ATOM 1721 HD11 LEU P 108 7.402 -19.828 8.509 0.00 0.00 PROA
ATOM 1722 HD12 LEU P 108 7.813 -20.458 6.971 0.00 0.00 PROA
ATOM 1723 HD13 LEU P 108 6.555 -19.106 7.078 0.00 0.00 PROA
ATOM 1724 CD2 LEU P 108 8.983 -18.367 5.857 0.00 0.00 PROA
ATOM 1725 HD21 LEU P 108 10.027 -18.003 5.748 0.00 0.00 PROA
ATOM 1726 HD22 LEU P 108 8.246 -17.616 5.500 0.00 0.00 PROA
ATOM 1727 HD23 LEU P 108 8.856 -19.328 5.314 0.00 0.00 PROA
ATOM 1728 C LEU P 108 8.922 -14.620 8.371 0.00 0.00 PROA
ATOM 1729 O LEU P 108 8.438 -13.905 7.531 0.00 0.00 PROA
ATOM 1730 N CYS P 109 9.171 -14.351 9.645 0.00 0.00 PROA
ATOM 1731 HN CYS P 109 9.629 -15.063 10.172 0.00 0.00 PROA
ATOM 1732 CA CYS P 109 8.858 -13.094 10.306 0.00 0.00 PROA
ATOM 1733 HA CYS P 109 7.781 -13.072 10.239 0.00 0.00 PROA
ATOM 1734 CB CYS P 109 9.362 -13.144 11.738 0.00 0.00 PROA
ATOM 1735 HB1 CYS P 109 10.473 -13.160 11.762 0.00 0.00 PROA
ATOM 1736 HB2 CYS P 109 8.969 -14.026 12.288 0.00 0.00 PROA
ATOM 1737 SG CYS P 109 8.830 -11.672 12.737 0.00 0.00 PROA
ATOM 1738 C CYS P 109 9.554 -11.947 9.618 0.00 0.00 PROA
ATOM 1739 O CYS P 109 9.002 -10.844 9.360 0.00 0.00 PROA
ATOM 1740 N LYS P 110 10.822 -12.104 9.267 0.00 0.00 PROA
ATOM 1741 HN LYS P 110 11.286 -12.946 9.529 0.00 0.00 PROA
ATOM 1742 CA LYS P 110 11.603 -11.233 8.403 0.00 0.00 PROA
ATOM 1743 HA LYS P 110 11.522 -10.249 8.840 0.00 0.00 PROA
ATOM 1744 CB LYS P 110 13.104 -11.773 8.385 0.00 0.00 PROA
ATOM 1745 HB1 LYS P 110 13.692 -11.301 7.570 0.00 0.00 PROA
ATOM 1746 HB2 LYS P 110 12.930 -12.844 8.145 0.00 0.00 PROA
ATOM 1747 CG LYS P 110 13.863 -11.513 9.694 0.00 0.00 PROA
ATOM 1748 HG1 LYS P 110 13.358 -11.925 10.593 0.00 0.00 PROA
ATOM 1749 HG2 LYS P 110 13.874 -10.407 9.807 0.00 0.00 PROA
ATOM 1750 CD LYS P 110 15.269 -12.041 9.741 0.00 0.00 PROA
ATOM 1751 HD1 LYS P 110 15.923 -11.526 9.005 0.00 0.00 PROA
ATOM 1752 HD2 LYS P 110 15.343 -13.047 9.274 0.00 0.00 PROA
ATOM 1753 CE LYS P 110 15.877 -12.104 11.159 0.00 0.00 PROA
ATOM 1754 HE1 LYS P 110 16.942 -12.415 11.106 0.00 0.00 PROA
ATOM 1755 HE2 LYS P 110 15.421 -12.900 11.785 0.00 0.00 PROA
ATOM 1756 NZ LYS P 110 15.937 -10.825 11.832 0.00 0.00 PROA
ATOM 1757 HZ1 LYS P 110 16.375 -10.958 12.765 0.00 0.00 PROA
ATOM 1758 HZ2 LYS P 110 14.986 -10.434 11.992 0.00 0.00 PROA
ATOM 1759 HZ3 LYS P 110 16.547 -10.173 11.297 0.00 0.00 PROA
ATOM 1760 C LYS P 110 11.126 -11.102 7.004 0.00 0.00 PROA
ATOM 1761 O LYS P 110 10.830 -9.986 6.479 0.00 0.00 PROA
ATOM 1762 N ALA P 111 10.787 -12.240 6.338 0.00 0.00 PROA
ATOM 1763 HN ALA P 111 10.815 -13.109 6.826 0.00 0.00 PROA
ATOM 1764 CA ALA P 111 10.253 -12.253 5.019 0.00 0.00 PROA
ATOM 1765 HA ALA P 111 10.914 -11.719 4.353 0.00 0.00 PROA
```

ATOM	1766	CB	ALA	P	111	10.012	-13.763	4.539	0.00	0.00	PROA
ATOM	1767	HB1	ALA	P	111	9.707	-13.803	3.471	0.00	0.00	PROA
ATOM	1768	HB2	ALA	P	111	9.266	-14.296	5.167	0.00	0.00	PROA
ATOM	1769	HB3	ALA	P	111	10.928	-14.385	4.629	0.00	0.00	PROA
ATOM	1770	C	ALA	P	111	8.900	-11.507	4.853	0.00	0.00	PROA
ATOM	1771	O	ALA	P	111	8.798	-10.719	3.835	0.00	0.00	PROA
ATOM	1772	N	VAL	P	112	7.882	-11.618	5.694	0.00	0.00	PROA
ATOM	1773	HN	VAL	P	112	7.990	-12.124	6.547	0.00	0.00	PROA
ATOM	1774	CA	VAL	P	112	6.648	-10.928	5.554	0.00	0.00	PROA
ATOM	1775	HA	VAL	P	112	6.551	-10.876	4.479	0.00	0.00	PROA
ATOM	1776	CB	VAL	P	112	5.479	-11.735	6.053	0.00	0.00	PROA
ATOM	1777	HB	VAL	P	112	4.551	-11.170	5.820	0.00	0.00	PROA
ATOM	1778	CG1	VAL	P	112	5.493	-13.077	5.392	0.00	0.00	PROA
ATOM	1779	HG11	VAL	P	112	5.453	-13.154	4.284	0.00	0.00	PROA
ATOM	1780	HG12	VAL	P	112	4.637	-13.721	5.687	0.00	0.00	PROA
ATOM	1781	HG13	VAL	P	112	6.354	-13.740	5.621	0.00	0.00	PROA
ATOM	1782	CG2	VAL	P	112	5.554	-12.010	7.565	0.00	0.00	PROA
ATOM	1783	HG21	VAL	P	112	4.482	-12.077	7.850	0.00	0.00	PROA
ATOM	1784	HG22	VAL	P	112	5.973	-11.153	8.135	0.00	0.00	PROA
ATOM	1785	HG23	VAL	P	112	6.054	-12.951	7.882	0.00	0.00	PROA
ATOM	1786	C	VAL	P	112	6.654	-9.537	5.914	0.00	0.00	PROA
ATOM	1787	O	VAL	P	112	5.995	-8.750	5.286	0.00	0.00	PROA
ATOM	1788	N	HSD	P	113	7.499	-9.002	6.825	0.00	0.00	PROA
ATOM	1789	HN	HSD	P	113	8.163	-9.573	7.303	0.00	0.00	PROA
ATOM	1790	CA	HSD	P	113	7.659	-7.632	7.090	0.00	0.00	PROA
ATOM	1791	HA	HSD	P	113	6.724	-7.135	7.299	0.00	0.00	PROA
ATOM	1792	CB	HSD	P	113	8.521	-7.391	8.375	0.00	0.00	PROA
ATOM	1793	HB1	HSD	P	113	8.930	-6.369	8.529	0.00	0.00	PROA
ATOM	1794	HB2	HSD	P	113	9.264	-8.211	8.278	0.00	0.00	PROA
ATOM	1795	ND1	HSD	P	113	7.605	-8.639	10.369	0.00	0.00	PROA
ATOM	1796	HD1	HSD	P	113	8.150	-9.474	10.301	0.00	0.00	PROA
ATOM	1797	CG	HSD	P	113	7.738	-7.503	9.561	0.00	0.00	PROA
ATOM	1798	CE1	HSD	P	113	6.798	-8.286	11.397	0.00	0.00	PROA
ATOM	1799	HE1	HSD	P	113	6.467	-8.974	12.174	0.00	0.00	PROA
ATOM	1800	NE2	HSD	P	113	6.349	-7.060	11.242	0.00	0.00	PROA
ATOM	1801	CD2	HSD	P	113	7.020	-6.573	10.157	0.00	0.00	PROA
ATOM	1802	HD2	HSD	P	113	7.072	-5.519	9.913	0.00	0.00	PROA
ATOM	1803	C	HSD	P	113	8.390	-6.947	5.840	0.00	0.00	PROA
ATOM	1804	O	HSD	P	113	7.910	-5.956	5.313	0.00	0.00	PROA
ATOM	1805	N	VAL	P	114	9.549	-7.562	5.286	0.00	0.00	PROA
ATOM	1806	HN	VAL	P	114	9.779	-8.467	5.635	0.00	0.00	PROA
ATOM	1807	CA	VAL	P	114	10.423	-7.245	4.193	0.00	0.00	PROA
ATOM	1808	HA	VAL	P	114	10.832	-6.255	4.332	0.00	0.00	PROA
ATOM	1809	CB	VAL	P	114	11.790	-8.122	4.043	0.00	0.00	PROA
ATOM	1810	HB	VAL	P	114	11.481	-9.189	4.059	0.00	0.00	PROA
ATOM	1811	CG1	VAL	P	114	12.556	-7.824	2.715	0.00	0.00	PROA
ATOM	1812	HG11	VAL	P	114	12.821	-6.747	2.784	0.00	0.00	PROA
ATOM	1813	HG12	VAL	P	114	11.902	-7.968	1.828	0.00	0.00	PROA
ATOM	1814	HG13	VAL	P	114	13.458	-8.472	2.695	0.00	0.00	PROA
ATOM	1815	CG2	VAL	P	114	12.783	-7.860	5.165	0.00	0.00	PROA
ATOM	1816	HG21	VAL	P	114	12.301	-7.909	6.165	0.00	0.00	PROA
ATOM	1817	HG22	VAL	P	114	13.333	-6.896	5.120	0.00	0.00	PROA
ATOM	1818	HG23	VAL	P	114	13.584	-8.627	5.097	0.00	0.00	PROA
ATOM	1819	C	VAL	P	114	9.669	-7.188	2.879	0.00	0.00	PROA
ATOM	1820	O	VAL	P	114	9.829	-6.347	2.001	0.00	0.00	PROA
ATOM	1821	N	ILE	P	115	8.711	-8.178	2.619	0.00	0.00	PROA
ATOM	1822	HN	ILE	P	115	8.521	-8.950	3.220	0.00	0.00	PROA
ATOM	1823	CA	ILE	P	115	7.835	-8.184	1.424	0.00	0.00	PROA
ATOM	1824	HA	ILE	P	115	8.451	-8.084	0.542	0.00	0.00	PROA
ATOM	1825	CB	ILE	P	115	7.012	-9.519	1.308	0.00	0.00	PROA
ATOM	1826	HB	ILE	P	115	6.816	-9.842	2.353	0.00	0.00	PROA

```
ATOM   1827  CG2 ILE P 115       5.733  -9.402   0.525  0.00  0.00      PROA
ATOM   1828 HG21 ILE P 115       5.927  -9.164  -0.543  0.00  0.00      PROA
ATOM   1829 HG22 ILE P 115       4.984  -8.663   0.881  0.00  0.00      PROA
ATOM   1830 HG23 ILE P 115       5.358 -10.441   0.643  0.00  0.00      PROA
ATOM   1831  CG1 ILE P 115       7.998 -10.547   0.800  0.00  0.00      PROA
ATOM   1832 HG11 ILE P 115       9.023 -10.195   1.047  0.00  0.00      PROA
ATOM   1833 HG12 ILE P 115       8.009 -10.674  -0.303  0.00  0.00      PROA
ATOM   1834  CD  ILE P 115       7.800 -11.989   1.321  0.00  0.00      PROA
ATOM   1835  HD1 ILE P 115       7.084 -12.565   0.696  0.00  0.00      PROA
ATOM   1836  HD2 ILE P 115       7.444 -12.098   2.368  0.00  0.00      PROA
ATOM   1837  HD3 ILE P 115       8.770 -12.511   1.180  0.00  0.00      PROA
ATOM   1838  C   ILE P 115       6.824  -7.045   1.340  0.00  0.00      PROA
ATOM   1839  O   ILE P 115       6.548  -6.464   0.271  0.00  0.00      PROA
ATOM   1840  N   TYR P 116       6.169  -6.747   2.475  0.00  0.00      PROA
ATOM   1841  HN  TYR P 116       6.257  -7.322   3.285  0.00  0.00      PROA
ATOM   1842  CA  TYR P 116       5.162  -5.794   2.596  0.00  0.00      PROA
ATOM   1843  HA  TYR P 116       4.416  -5.976   1.836  0.00  0.00      PROA
ATOM   1844  CB  TYR P 116       4.472  -5.975   3.997  0.00  0.00      PROA
ATOM   1845  HB1 TYR P 116       5.134  -5.860   4.882  0.00  0.00      PROA
ATOM   1846  HB2 TYR P 116       4.038  -6.997   4.039  0.00  0.00      PROA
ATOM   1847  CG  TYR P 116       3.444  -4.889   4.119  0.00  0.00      PROA
ATOM   1848  CD1 TYR P 116       3.796  -3.603   4.726  0.00  0.00      PROA
ATOM   1849  HD1 TYR P 116       4.812  -3.290   4.918  0.00  0.00      PROA
ATOM   1850  CE1 TYR P 116       2.743  -2.704   5.036  0.00  0.00      PROA
ATOM   1851  HE1 TYR P 116       2.977  -1.806   5.588  0.00  0.00      PROA
ATOM   1852  CZ  TYR P 116       1.416  -3.076   4.748  0.00  0.00      PROA
ATOM   1853  OH  TYR P 116       0.448  -2.093   4.969  0.00  0.00      PROA
ATOM   1854  HH  TYR P 116      -0.391  -2.465   4.689  0.00  0.00      PROA
ATOM   1855  CD2 TYR P 116       2.067  -5.175   3.905  0.00  0.00      PROA
ATOM   1856  HD2 TYR P 116       1.804  -6.179   3.607  0.00  0.00      PROA
ATOM   1857  CE2 TYR P 116       1.108  -4.231   4.113  0.00  0.00      PROA
ATOM   1858  HE2 TYR P 116       0.097  -4.455   3.809  0.00  0.00      PROA
ATOM   1859  C   TYR P 116       5.705  -4.411   2.256  0.00  0.00      PROA
ATOM   1860  O   TYR P 116       5.088  -3.722   1.437  0.00  0.00      PROA
ATOM   1861  N   THR P 117       6.863  -4.033   2.874  0.00  0.00      PROA
ATOM   1862  HN  THR P 117       7.423  -4.600   3.473  0.00  0.00      PROA
ATOM   1863  CA  THR P 117       7.431  -2.766   2.613  0.00  0.00      PROA
ATOM   1864  HA  THR P 117       6.561  -2.127   2.576  0.00  0.00      PROA
ATOM   1865  CB  THR P 117       8.386  -2.361   3.715  0.00  0.00      PROA
ATOM   1866  HB  THR P 117       9.081  -1.581   3.338  0.00  0.00      PROA
ATOM   1867  OG1 THR P 117       9.165  -3.475   4.077  0.00  0.00      PROA
ATOM   1868  HG1 THR P 117       9.980  -3.141   4.459  0.00  0.00      PROA
ATOM   1869  CG2 THR P 117       7.541  -1.768   4.929  0.00  0.00      PROA
ATOM   1870 HG21 THR P 117       6.875  -0.951   4.577  0.00  0.00      PROA
ATOM   1871 HG22 THR P 117       8.298  -1.472   5.686  0.00  0.00      PROA
ATOM   1872 HG23 THR P 117       7.055  -2.698   5.293  0.00  0.00      PROA
ATOM   1873  C   THR P 117       8.146  -2.626   1.268  0.00  0.00      PROA
ATOM   1874  O   THR P 117       7.915  -1.673   0.553  0.00  0.00      PROA
ATOM   1875  N   VAL P 118       8.860  -3.684   0.754  0.00  0.00      PROA
ATOM   1876  HN  VAL P 118       9.017  -4.482   1.332  0.00  0.00      PROA
ATOM   1877  CA  VAL P 118       9.484  -3.552  -0.537  0.00  0.00      PROA
ATOM   1878  HA  VAL P 118      10.066  -2.642  -0.511  0.00  0.00      PROA
ATOM   1879  CB  VAL P 118      10.381  -4.740  -0.820  0.00  0.00      PROA
ATOM   1880  HB  VAL P 118      10.975  -4.921   0.101  0.00  0.00      PROA
ATOM   1881  CG1 VAL P 118       9.787  -6.134  -1.179  0.00  0.00      PROA
ATOM   1882 HG11 VAL P 118      10.633  -6.851  -1.252  0.00  0.00      PROA
ATOM   1883 HG12 VAL P 118       9.260  -6.055  -2.154  0.00  0.00      PROA
ATOM   1884 HG13 VAL P 118       9.076  -6.495  -0.406  0.00  0.00      PROA
ATOM   1885  CG2 VAL P 118      11.348  -4.400  -1.990  0.00  0.00      PROA
ATOM   1886 HG21 VAL P 118      11.940  -3.473  -1.831  0.00  0.00      PROA
ATOM   1887 HG22 VAL P 118      10.886  -4.355  -2.999  0.00  0.00      PROA
```

```
ATOM 1888 HG23 VAL P 118 12.156 -5.163 -1.970 0.00 0.00 PROA
ATOM 1889 C VAL P 118 8.413 -3.361 -1.645 0.00 0.00 PROA
ATOM 1890 O VAL P 118 8.588 -2.552 -2.531 0.00 0.00 PROA
ATOM 1891 N ASN P 119 7.278 -4.029 -1.516 0.00 0.00 PROA
ATOM 1892 HN ASN P 119 7.026 -4.584 -0.728 0.00 0.00 PROA
ATOM 1893 CA ASN P 119 6.172 -3.898 -2.488 0.00 0.00 PROA
ATOM 1894 HA ASN P 119 6.582 -4.021 -3.479 0.00 0.00 PROA
ATOM 1895 CB ASN P 119 5.119 -5.094 -2.289 0.00 0.00 PROA
ATOM 1896 HB1 ASN P 119 4.608 -5.067 -1.303 0.00 0.00 PROA
ATOM 1897 HB2 ASN P 119 5.724 -6.022 -2.368 0.00 0.00 PROA
ATOM 1898 CG ASN P 119 4.086 -5.111 -3.446 0.00 0.00 PROA
ATOM 1899 OD1 ASN P 119 4.363 -4.993 -4.638 0.00 0.00 PROA
ATOM 1900 ND2 ASN P 119 2.777 -5.272 -3.015 0.00 0.00 PROA
ATOM 1901 HD21 ASN P 119 2.075 -5.609 -3.642 0.00 0.00 PROA
ATOM 1902 HD22 ASN P 119 2.440 -5.172 -2.079 0.00 0.00 PROA
ATOM 1903 C ASN P 119 5.437 -2.606 -2.393 0.00 0.00 PROA
ATOM 1904 O ASN P 119 5.132 -1.937 -3.385 0.00 0.00 PROA
ATOM 1905 N LEU P 120 5.183 -2.106 -1.145 0.00 0.00 PROA
ATOM 1906 HN LEU P 120 5.339 -2.762 -0.410 0.00 0.00 PROA
ATOM 1907 CA LEU P 120 4.533 -0.925 -0.825 0.00 0.00 PROA
ATOM 1908 HA LEU P 120 3.746 -0.743 -1.541 0.00 0.00 PROA
ATOM 1909 CB LEU P 120 4.084 -0.987 0.634 0.00 0.00 PROA
ATOM 1910 HB1 LEU P 120 4.963 -1.281 1.247 0.00 0.00 PROA
ATOM 1911 HB2 LEU P 120 3.431 -1.844 0.902 0.00 0.00 PROA
ATOM 1912 CG LEU P 120 3.376 0.182 1.394 0.00 0.00 PROA
ATOM 1913 HG LEU P 120 4.037 1.066 1.514 0.00 0.00 PROA
ATOM 1914 CD1 LEU P 120 2.098 0.566 0.606 0.00 0.00 PROA
ATOM 1915 HD11 LEU P 120 2.191 0.687 -0.494 0.00 0.00 PROA
ATOM 1916 HD12 LEU P 120 1.736 1.509 1.069 0.00 0.00 PROA
ATOM 1917 HD13 LEU P 120 1.349 -0.250 0.691 0.00 0.00 PROA
ATOM 1918 CD2 LEU P 120 3.095 -0.083 2.864 0.00 0.00 PROA
ATOM 1919 HD21 LEU P 120 2.737 0.790 3.451 0.00 0.00 PROA
ATOM 1920 HD22 LEU P 120 4.065 -0.367 3.324 0.00 0.00 PROA
ATOM 1921 HD23 LEU P 120 2.422 -0.964 2.938 0.00 0.00 PROA
ATOM 1922 C LEU P 120 5.372 0.315 -1.036 0.00 0.00 PROA
ATOM 1923 O LEU P 120 4.879 1.352 -1.517 0.00 0.00 PROA
ATOM 1924 N TYR P 121 6.691 0.282 -0.923 0.00 0.00 PROA
ATOM 1925 HN TYR P 121 7.259 -0.455 -0.565 0.00 0.00 PROA
ATOM 1926 CA TYR P 121 7.564 1.374 -1.248 0.00 0.00 PROA
ATOM 1927 HA TYR P 121 6.967 2.156 -0.803 0.00 0.00 PROA
ATOM 1928 CB TYR P 121 8.910 1.275 -0.587 0.00 0.00 PROA
ATOM 1929 HB1 TYR P 121 9.380 2.219 -0.938 0.00 0.00 PROA
ATOM 1930 HB2 TYR P 121 9.459 0.382 -0.953 0.00 0.00 PROA
ATOM 1931 CG TYR P 121 8.982 1.301 0.959 0.00 0.00 PROA
ATOM 1932 CD1 TYR P 121 7.930 1.597 1.866 0.00 0.00 PROA
ATOM 1933 HD1 TYR P 121 6.925 1.850 1.562 0.00 0.00 PROA
ATOM 1934 CE1 TYR P 121 8.124 1.542 3.253 0.00 0.00 PROA
ATOM 1935 HE1 TYR P 121 7.299 1.773 3.911 0.00 0.00 PROA
ATOM 1936 CZ TYR P 121 9.390 1.186 3.785 0.00 0.00 PROA
ATOM 1937 OH TYR P 121 9.572 1.170 5.201 0.00 0.00 PROA
ATOM 1938 HH TYR P 121 10.426 0.767 5.373 0.00 0.00 PROA
ATOM 1939 CD2 TYR P 121 10.249 0.946 1.549 0.00 0.00 PROA
ATOM 1940 HD2 TYR P 121 11.154 0.687 1.019 0.00 0.00 PROA
ATOM 1941 CE2 TYR P 121 10.447 0.945 2.919 0.00 0.00 PROA
ATOM 1942 HE2 TYR P 121 11.386 0.676 3.379 0.00 0.00 PROA
ATOM 1943 C TYR P 121 7.585 1.596 -2.783 0.00 0.00 PROA
ATOM 1944 O TYR P 121 7.693 2.739 -3.243 0.00 0.00 PROA
ATOM 1945 N SER P 122 7.579 0.534 -3.546 0.00 0.00 PROA
ATOM 1946 HN SER P 122 7.526 -0.370 -3.129 0.00 0.00 PROA
ATOM 1947 CA SER P 122 7.611 0.579 -4.953 0.00 0.00 PROA
ATOM 1948 HA SER P 122 8.154 1.491 -5.155 0.00 0.00 PROA
```

```
ATOM   1949  CB  SER P 122       8.129  -0.749  -5.594  0.00  0.00      PROA
ATOM   1950  HB1 SER P 122       9.219  -0.774  -5.378  0.00  0.00      PROA
ATOM   1951  HB2 SER P 122       8.023  -0.656  -6.696  0.00  0.00      PROA
ATOM   1952  OG  SER P 122       7.460  -1.976  -5.348  0.00  0.00      PROA
ATOM   1953  HG1 SER P 122       7.956  -2.353  -4.618  0.00  0.00      PROA
ATOM   1954  C   SER P 122       6.276   0.925  -5.529  0.00  0.00      PROA
ATOM   1955  O   SER P 122       6.177   1.798  -6.383  0.00  0.00      PROA
ATOM   1956  N   SER P 123       5.155   0.500  -4.943  0.00  0.00      PROA
ATOM   1957  HN  SER P 123       5.389  -0.051  -4.146  0.00  0.00      PROA
ATOM   1958  CA  SER P 123       3.786   0.573  -5.417  0.00  0.00      PROA
ATOM   1959  HA  SER P 123       3.789   0.008  -6.338  0.00  0.00      PROA
ATOM   1960  CB  SER P 123       2.708   0.066  -4.350  0.00  0.00      PROA
ATOM   1961  HB1 SER P 123       2.939   0.564  -3.384  0.00  0.00      PROA
ATOM   1962  HB2 SER P 123       2.792  -1.035  -4.223  0.00  0.00      PROA
ATOM   1963  OG  SER P 123       1.337   0.097  -4.734  0.00  0.00      PROA
ATOM   1964  HG1 SER P 123       1.305  -0.628  -5.362  0.00  0.00      PROA
ATOM   1965  C   SER P 123       3.233   1.943  -5.810  0.00  0.00      PROA
ATOM   1966  O   SER P 123       2.705   2.177  -6.891  0.00  0.00      PROA
ATOM   1967  N   VAL P 124       3.446   2.978  -5.001  0.00  0.00      PROA
ATOM   1968  HN  VAL P 124       3.747   2.814  -4.065  0.00  0.00      PROA
ATOM   1969  CA  VAL P 124       3.034   4.390  -5.284  0.00  0.00      PROA
ATOM   1970  HA  VAL P 124       2.005   4.345  -5.610  0.00  0.00      PROA
ATOM   1971  CB  VAL P 124       3.138   5.154  -3.947  0.00  0.00      PROA
ATOM   1972  HB  VAL P 124       2.836   6.221  -4.017  0.00  0.00      PROA
ATOM   1973  CG1 VAL P 124       2.339   4.440  -2.871  0.00  0.00      PROA
ATOM   1974 HG11 VAL P 124       2.747   3.424  -2.687  0.00  0.00      PROA
ATOM   1975 HG12 VAL P 124       1.269   4.231  -3.084  0.00  0.00      PROA
ATOM   1976 HG13 VAL P 124       2.482   4.925  -1.882  0.00  0.00      PROA
ATOM   1977  CG2 VAL P 124       4.634   5.227  -3.501  0.00  0.00      PROA
ATOM   1978 HG21 VAL P 124       5.146   4.253  -3.347  0.00  0.00      PROA
ATOM   1979 HG22 VAL P 124       4.736   5.643  -2.476  0.00  0.00      PROA
ATOM   1980 HG23 VAL P 124       5.264   5.878  -4.144  0.00  0.00      PROA
ATOM   1981  C   VAL P 124       3.824   4.981  -6.464  0.00  0.00      PROA
ATOM   1982  O   VAL P 124       3.367   5.736  -7.370  0.00  0.00      PROA
ATOM   1983  N   TRP P 125       5.062   4.550  -6.597  0.00  0.00      PROA
ATOM   1984  HN  TRP P 125       5.348   3.844  -5.953  0.00  0.00      PROA
ATOM   1985  CA  TRP P 125       5.855   4.934  -7.782  0.00  0.00      PROA
ATOM   1986  HA  TRP P 125       5.727   6.002  -7.878  0.00  0.00      PROA
ATOM   1987  CB  TRP P 125       7.386   4.784  -7.532  0.00  0.00      PROA
ATOM   1988  HB1 TRP P 125       8.033   4.897  -8.428  0.00  0.00      PROA
ATOM   1989  HB2 TRP P 125       7.625   3.788  -7.102  0.00  0.00      PROA
ATOM   1990  CG  TRP P 125       7.795   5.817  -6.562  0.00  0.00      PROA
ATOM   1991  CD1 TRP P 125       8.078   5.858  -5.196  0.00  0.00      PROA
ATOM   1992  HD1 TRP P 125       7.984   4.967  -4.594  0.00  0.00      PROA
ATOM   1993  NE1 TRP P 125       8.258   7.120  -4.809  0.00  0.00      PROA
ATOM   1994  HE1 TRP P 125       8.428   7.435  -3.902  0.00  0.00      PROA
ATOM   1995  CE2 TRP P 125       8.152   7.960  -5.865  0.00  0.00      PROA
ATOM   1996  CD2 TRP P 125       7.844   7.177  -7.010  0.00  0.00      PROA
ATOM   1997  CE3 TRP P 125       7.654   7.843  -8.224  0.00  0.00      PROA
ATOM   1998  HE3 TRP P 125       7.527   7.264  -9.127  0.00  0.00      PROA
ATOM   1999  CZ3 TRP P 125       7.865   9.190  -8.343  0.00  0.00      PROA
ATOM   2000  HZ3 TRP P 125       7.829   9.741  -9.271  0.00  0.00      PROA
ATOM   2001  CZ2 TRP P 125       8.362   9.327  -5.997  0.00  0.00      PROA
ATOM   2002  HZ2 TRP P 125       8.982   9.824  -5.266  0.00  0.00      PROA
ATOM   2003  CH2 TRP P 125       8.258   9.953  -7.291  0.00  0.00      PROA
ATOM   2004  HH2 TRP P 125       8.610  10.972  -7.358  0.00  0.00      PROA
ATOM   2005  C   TRP P 125       5.567   4.231  -9.049  0.00  0.00      PROA
ATOM   2006  O   TRP P 125       5.608   4.798 -10.152  0.00  0.00      PROA
ATOM   2007  N   ILE P 126       5.201   2.940  -8.887  0.00  0.00      PROA
ATOM   2008  HN  ILE P 126       5.239   2.392  -8.055  0.00  0.00      PROA
ATOM   2009  CA  ILE P 126       4.530   2.218  -9.988  0.00  0.00      PROA
```

ATOM	2010	HA	ILE	P	126	5.117	2.241	-10.894	0.00	0.00	PROA
ATOM	2011	CB	ILE	P	126	4.264	0.706	-9.654	0.00	0.00	PROA
ATOM	2012	HB	ILE	P	126	3.482	0.563	-8.877	0.00	0.00	PROA
ATOM	2013	CG2	ILE	P	126	3.864	0.041	-11.017	0.00	0.00	PROA
ATOM	2014	HG21	ILE	P	126	4.585	0.275	-11.829	0.00	0.00	PROA
ATOM	2015	HG22	ILE	P	126	2.847	0.318	-11.370	0.00	0.00	PROA
ATOM	2016	HG23	ILE	P	126	3.944	-1.063	-10.927	0.00	0.00	PROA
ATOM	2017	CG1	ILE	P	126	5.379	-0.158	-9.079	0.00	0.00	PROA
ATOM	2018	HG11	ILE	P	126	5.052	-1.211	-9.218	0.00	0.00	PROA
ATOM	2019	HG12	ILE	P	126	5.433	-0.073	-7.973	0.00	0.00	PROA
ATOM	2020	CD	ILE	P	126	6.781	0.031	-9.662	0.00	0.00	PROA
ATOM	2021	HD1	ILE	P	126	7.400	0.855	-9.247	0.00	0.00	PROA
ATOM	2022	HD2	ILE	P	126	6.756	0.121	-10.769	0.00	0.00	PROA
ATOM	2023	HD3	ILE	P	126	7.423	-0.849	-9.444	0.00	0.00	PROA
ATOM	2024	C	ILE	P	126	3.248	2.938	-10.403	0.00	0.00	PROA
ATOM	2025	O	ILE	P	126	3.056	3.116	-11.606	0.00	0.00	PROA
ATOM	2026	N	LEU	P	127	2.434	3.474	-9.468	0.00	0.00	PROA
ATOM	2027	HN	LEU	P	127	2.697	3.300	-8.522	0.00	0.00	PROA
ATOM	2028	CA	LEU	P	127	1.260	4.316	-9.884	0.00	0.00	PROA
ATOM	2029	HA	LEU	P	127	0.641	3.870	-10.649	0.00	0.00	PROA
ATOM	2030	CB	LEU	P	127	0.330	4.478	-8.682	0.00	0.00	PROA
ATOM	2031	HB1	LEU	P	127	0.820	5.104	-7.904	0.00	0.00	PROA
ATOM	2032	HB2	LEU	P	127	0.056	3.466	-8.315	0.00	0.00	PROA
ATOM	2033	CG	LEU	P	127	-1.063	5.252	-9.030	0.00	0.00	PROA
ATOM	2034	HG	LEU	P	127	-0.854	6.326	-9.219	0.00	0.00	PROA
ATOM	2035	CD1	LEU	P	127	-1.858	4.719	-10.268	0.00	0.00	PROA
ATOM	2036	HD11	LEU	P	127	-1.301	4.946	-11.202	0.00	0.00	PROA
ATOM	2037	HD12	LEU	P	127	-2.851	5.209	-10.357	0.00	0.00	PROA
ATOM	2038	HD13	LEU	P	127	-2.107	3.643	-10.147	0.00	0.00	PROA
ATOM	2039	CD2	LEU	P	127	-1.938	5.135	-7.820	0.00	0.00	PROA
ATOM	2040	HD21	LEU	P	127	-2.920	5.645	-7.913	0.00	0.00	PROA
ATOM	2041	HD22	LEU	P	127	-1.426	5.612	-6.957	0.00	0.00	PROA
ATOM	2042	HD23	LEU	P	127	-2.267	4.087	-7.654	0.00	0.00	PROA
ATOM	2043	C	LEU	P	127	1.660	5.649	-10.468	0.00	0.00	PROA
ATOM	2044	O	LEU	P	127	1.038	6.142	-11.364	0.00	0.00	PROA
ATOM	2045	N	ALA	P	128	2.690	6.320	-9.995	0.00	0.00	PROA
ATOM	2046	HN	ALA	P	128	3.086	5.905	-9.180	0.00	0.00	PROA
ATOM	2047	CA	ALA	P	128	3.259	7.549	-10.531	0.00	0.00	PROA
ATOM	2048	HA	ALA	P	128	2.511	8.327	-10.510	0.00	0.00	PROA
ATOM	2049	CB	ALA	P	128	4.375	8.048	-9.622	0.00	0.00	PROA
ATOM	2050	HB1	ALA	P	128	3.992	8.222	-8.594	0.00	0.00	PROA
ATOM	2051	HB2	ALA	P	128	4.691	9.039	-10.012	0.00	0.00	PROA
ATOM	2052	HB3	ALA	P	128	5.209	7.323	-9.505	0.00	0.00	PROA
ATOM	2053	C	ALA	P	128	3.668	7.321	-11.940	0.00	0.00	PROA
ATOM	2054	O	ALA	P	128	3.191	8.041	-12.781	0.00	0.00	PROA
ATOM	2055	N	PHE	P	129	4.414	6.221	-12.284	0.00	0.00	PROA
ATOM	2056	HN	PHE	P	129	4.869	5.635	-11.618	0.00	0.00	PROA
ATOM	2057	CA	PHE	P	129	4.791	5.952	-13.681	0.00	0.00	PROA
ATOM	2058	HA	PHE	P	129	5.124	6.932	-13.986	0.00	0.00	PROA
ATOM	2059	CB	PHE	P	129	5.910	4.974	-13.775	0.00	0.00	PROA
ATOM	2060	HB1	PHE	P	129	6.062	4.549	-14.790	0.00	0.00	PROA
ATOM	2061	HB2	PHE	P	129	5.792	4.045	-13.178	0.00	0.00	PROA
ATOM	2062	CG	PHE	P	129	7.156	5.613	-13.403	0.00	0.00	PROA
ATOM	2063	CD1	PHE	P	129	7.676	6.705	-14.115	0.00	0.00	PROA
ATOM	2064	HD1	PHE	P	129	7.091	7.045	-14.957	0.00	0.00	PROA
ATOM	2065	CE1	PHE	P	129	8.920	7.300	-13.856	0.00	0.00	PROA
ATOM	2066	HE1	PHE	P	129	9.170	8.097	-14.540	0.00	0.00	PROA
ATOM	2067	CZ	PHE	P	129	9.700	6.828	-12.821	0.00	0.00	PROA
ATOM	2068	HZ	PHE	P	129	10.704	7.195	-12.664	0.00	0.00	PROA
ATOM	2069	CD2	PHE	P	129	7.966	5.147	-12.274	0.00	0.00	PROA
ATOM	2070	HD2	PHE	P	129	7.660	4.264	-11.732	0.00	0.00	PROA

ATOM	2071	CE2	PHE	P	129	9.223	5.773	-11.992	0.00	0.00	PROA
ATOM	2072	HE2	PHE	P	129	9.916	5.431	-11.237	0.00	0.00	PROA
ATOM	2073	C	PHE	P	129	3.633	5.583	-14.593	0.00	0.00	PROA
ATOM	2074	O	PHE	P	129	3.759	5.955	-15.798	0.00	0.00	PROA
ATOM	2075	N	ILE	P	130	2.589	4.848	-14.099	0.00	0.00	PROA
ATOM	2076	HN	ILE	P	130	2.690	4.512	-13.165	0.00	0.00	PROA
ATOM	2077	CA	ILE	P	130	1.314	4.599	-14.858	0.00	0.00	PROA
ATOM	2078	HA	ILE	P	130	1.645	4.078	-15.744	0.00	0.00	PROA
ATOM	2079	CB	ILE	P	130	0.397	3.742	-14.064	0.00	0.00	PROA
ATOM	2080	HB	ILE	P	130	0.237	4.151	-13.044	0.00	0.00	PROA
ATOM	2081	CG2	ILE	P	130	-1.022	3.600	-14.748	0.00	0.00	PROA
ATOM	2082	HG21	ILE	P	130	-1.457	2.729	-14.214	0.00	0.00	PROA
ATOM	2083	HG22	ILE	P	130	-0.884	3.382	-15.829	0.00	0.00	PROA
ATOM	2084	HG23	ILE	P	130	-1.671	4.497	-14.651	0.00	0.00	PROA
ATOM	2085	CG1	ILE	P	130	1.034	2.320	-13.951	0.00	0.00	PROA
ATOM	2086	HG11	ILE	P	130	2.113	2.495	-13.749	0.00	0.00	PROA
ATOM	2087	HG12	ILE	P	130	0.933	1.778	-14.916	0.00	0.00	PROA
ATOM	2088	CD	ILE	P	130	0.452	1.532	-12.788	0.00	0.00	PROA
ATOM	2089	HD1	ILE	P	130	0.541	2.051	-11.810	0.00	0.00	PROA
ATOM	2090	HD2	ILE	P	130	0.850	0.496	-12.743	0.00	0.00	PROA
ATOM	2091	HD3	ILE	P	130	-0.612	1.281	-12.985	0.00	0.00	PROA
ATOM	2092	C	ILE	P	130	0.661	5.961	-15.247	0.00	0.00	PROA
ATOM	2093	O	ILE	P	130	0.233	6.219	-16.347	0.00	0.00	PROA
ATOM	2094	N	SER	P	131	0.767	6.930	-14.347	0.00	0.00	PROA
ATOM	2095	HN	SER	P	131	1.343	6.675	-13.574	0.00	0.00	PROA
ATOM	2096	CA	SER	P	131	0.326	8.343	-14.585	0.00	0.00	PROA
ATOM	2097	HA	SER	P	131	-0.670	8.334	-15.002	0.00	0.00	PROA
ATOM	2098	CB	SER	P	131	0.265	9.221	-13.287	0.00	0.00	PROA
ATOM	2099	HB1	SER	P	131	-0.330	10.155	-13.374	0.00	0.00	PROA
ATOM	2100	HB2	SER	P	131	1.263	9.564	-12.938	0.00	0.00	PROA
ATOM	2101	OG	SER	P	131	-0.230	8.515	-12.140	0.00	0.00	PROA
ATOM	2102	HG1	SER	P	131	0.423	7.977	-11.688	0.00	0.00	PROA
ATOM	2103	C	SER	P	131	1.203	9.003	-15.638	0.00	0.00	PROA
ATOM	2104	O	SER	P	131	0.753	9.645	-16.607	0.00	0.00	PROA
ATOM	2105	N	LEU	P	132	2.530	8.912	-15.517	0.00	0.00	PROA
ATOM	2106	HN	LEU	P	132	2.956	8.458	-14.738	0.00	0.00	PROA
ATOM	2107	CA	LEU	P	132	3.591	9.451	-16.417	0.00	0.00	PROA
ATOM	2108	HA	LEU	P	132	3.456	10.522	-16.436	0.00	0.00	PROA
ATOM	2109	CB	LEU	P	132	5.041	9.294	-15.815	0.00	0.00	PROA
ATOM	2110	HB1	LEU	P	132	5.447	8.267	-15.932	0.00	0.00	PROA
ATOM	2111	HB2	LEU	P	132	4.954	9.427	-14.715	0.00	0.00	PROA
ATOM	2112	CG	LEU	P	132	6.055	10.338	-16.441	0.00	0.00	PROA
ATOM	2113	HG	LEU	P	132	5.857	10.342	-17.534	0.00	0.00	PROA
ATOM	2114	CD1	LEU	P	132	5.895	11.707	-15.821	0.00	0.00	PROA
ATOM	2115	HD11	LEU	P	132	6.735	12.276	-16.273	0.00	0.00	PROA
ATOM	2116	HD12	LEU	P	132	6.061	11.683	-14.722	0.00	0.00	PROA
ATOM	2117	HD13	LEU	P	132	4.905	12.156	-16.048	0.00	0.00	PROA
ATOM	2118	CD2	LEU	P	132	7.466	9.963	-16.187	0.00	0.00	PROA
ATOM	2119	HD21	LEU	P	132	8.188	10.611	-16.728	0.00	0.00	PROA
ATOM	2120	HD22	LEU	P	132	7.579	8.916	-16.542	0.00	0.00	PROA
ATOM	2121	HD23	LEU	P	132	7.752	10.210	-15.143	0.00	0.00	PROA
ATOM	2122	C	LEU	P	132	3.388	8.893	-17.817	0.00	0.00	PROA
ATOM	2123	O	LEU	P	132	3.460	9.613	-18.798	0.00	0.00	PROA
ATOM	2124	N	ASP	P	133	3.194	7.601	-17.928	0.00	0.00	PROA
ATOM	2125	HN	ASP	P	133	3.198	7.044	-17.101	0.00	0.00	PROA
ATOM	2126	CA	ASP	P	133	2.850	6.909	-19.227	0.00	0.00	PROA
ATOM	2127	HA	ASP	P	133	3.622	7.119	-19.952	0.00	0.00	PROA
ATOM	2128	CB	ASP	P	133	2.858	5.373	-19.024	0.00	0.00	PROA
ATOM	2129	HB1	ASP	P	133	2.032	5.112	-18.327	0.00	0.00	PROA
ATOM	2130	HB2	ASP	P	133	3.845	5.058	-18.624	0.00	0.00	PROA
ATOM	2131	CG	ASP	P	133	2.622	4.620	-20.341	0.00	0.00	PROA

```
ATOM   2132  OD1 ASP P 133      1.433   4.157 -20.496  0.00  0.00      PROA
ATOM   2133  OD2 ASP P 133      3.581   4.341 -21.038  0.00  0.00      PROA
ATOM   2134  C   ASP P 133      1.530   7.454 -19.834  0.00  0.00      PROA
ATOM   2135  O   ASP P 133      1.464   7.634 -21.025  0.00  0.00      PROA
ATOM   2136  N   ARG P 134      0.440   7.637 -19.013  0.00  0.00      PROA
ATOM   2137  HN  ARG P 134      0.496   7.555 -18.021  0.00  0.00      PROA
ATOM   2138  CA  ARG P 134     -0.780   8.180 -19.587  0.00  0.00      PROA
ATOM   2139  HA  ARG P 134     -1.133   7.516 -20.362  0.00  0.00      PROA
ATOM   2140  CB  ARG P 134     -1.856   8.236 -18.529  0.00  0.00      PROA
ATOM   2141  HB1 ARG P 134     -1.732   8.940 -17.679  0.00  0.00      PROA
ATOM   2142  HB2 ARG P 134     -1.815   7.254 -18.012  0.00  0.00      PROA
ATOM   2143  CG  ARG P 134     -3.283   8.277 -19.051  0.00  0.00      PROA
ATOM   2144  HG1 ARG P 134     -3.434   9.377 -19.075  0.00  0.00      PROA
ATOM   2145  HG2 ARG P 134     -4.001   7.986 -18.254  0.00  0.00      PROA
ATOM   2146  CD  ARG P 134     -3.607   7.575 -20.390  0.00  0.00      PROA
ATOM   2147  HD1 ARG P 134     -2.885   7.885 -21.175  0.00  0.00      PROA
ATOM   2148  HD2 ARG P 134     -4.543   7.993 -20.817  0.00  0.00      PROA
ATOM   2149  NE  ARG P 134     -3.556   6.062 -20.172  0.00  0.00      PROA
ATOM   2150  HE  ARG P 134     -3.877   5.712 -19.292  0.00  0.00      PROA
ATOM   2151  CZ  ARG P 134     -2.945   5.235 -20.973  0.00  0.00      PROA
ATOM   2152  NH1 ARG P 134     -2.403   5.498 -22.130  0.00  0.00      PROA
ATOM   2153 HH11 ARG P 134     -2.743   6.245 -22.701  0.00  0.00      PROA
ATOM   2154 HH12 ARG P 134     -2.260   4.634 -22.612  0.00  0.00      PROA
ATOM   2155  NH2 ARG P 134     -2.812   3.962 -20.674  0.00  0.00      PROA
ATOM   2156 HH21 ARG P 134     -2.498   3.335 -21.387  0.00  0.00      PROA
ATOM   2157 HH22 ARG P 134     -3.343   3.575 -19.920  0.00  0.00      PROA
ATOM   2158  C   ARG P 134     -0.489   9.630 -20.141  0.00  0.00      PROA
ATOM   2159  O   ARG P 134     -0.868   9.938 -21.268  0.00  0.00      PROA
ATOM   2160  N   TYR P 135      0.285  10.504 -19.435  0.00  0.00      PROA
ATOM   2161  HN  TYR P 135      0.470  10.247 -18.490  0.00  0.00      PROA
ATOM   2162  CA  TYR P 135      0.780  11.784 -19.923  0.00  0.00      PROA
ATOM   2163  HA  TYR P 135     -0.084  12.393 -20.144  0.00  0.00      PROA
ATOM   2164  CB  TYR P 135      1.580  12.587 -18.776  0.00  0.00      PROA
ATOM   2165  HB1 TYR P 135      2.060  11.894 -18.054  0.00  0.00      PROA
ATOM   2166  HB2 TYR P 135      0.925  13.128 -18.060  0.00  0.00      PROA
ATOM   2167  CG  TYR P 135      2.609  13.543 -19.153  0.00  0.00      PROA
ATOM   2168  CD1 TYR P 135      2.345  14.726 -19.897  0.00  0.00      PROA
ATOM   2169  HD1 TYR P 135      1.377  15.022 -20.272  0.00  0.00      PROA
ATOM   2170  CE1 TYR P 135      3.370  15.649 -20.140  0.00  0.00      PROA
ATOM   2171  HE1 TYR P 135      3.023  16.587 -20.549  0.00  0.00      PROA
ATOM   2172  CZ  TYR P 135      4.666  15.384 -19.773  0.00  0.00      PROA
ATOM   2173  OH  TYR P 135      5.696  16.261 -20.129  0.00  0.00      PROA
ATOM   2174  HH  TYR P 135      5.917  16.194 -21.061  0.00  0.00      PROA
ATOM   2175  CD2 TYR P 135      3.955  13.324 -18.777  0.00  0.00      PROA
ATOM   2176  HD2 TYR P 135      4.206  12.351 -18.379  0.00  0.00      PROA
ATOM   2177  CE2 TYR P 135      5.009  14.315 -19.089  0.00  0.00      PROA
ATOM   2178  HE2 TYR P 135      5.993  14.101 -18.697  0.00  0.00      PROA
ATOM   2179  C   TYR P 135      1.564  11.623 -21.170  0.00  0.00      PROA
ATOM   2180  O   TYR P 135      1.347  12.420 -22.082  0.00  0.00      PROA
ATOM   2181  N   LEU P 136      2.536  10.659 -21.369  0.00  0.00      PROA
ATOM   2182  HN  LEU P 136      2.658  10.021 -20.613  0.00  0.00      PROA
ATOM   2183  CA  LEU P 136      3.318  10.433 -22.502  0.00  0.00      PROA
ATOM   2184  HA  LEU P 136      3.646  11.403 -22.845  0.00  0.00      PROA
ATOM   2185  CB  LEU P 136      4.492   9.391 -22.113  0.00  0.00      PROA
ATOM   2186  HB1 LEU P 136      4.992   9.012 -23.030  0.00  0.00      PROA
ATOM   2187  HB2 LEU P 136      4.065   8.517 -21.576  0.00  0.00      PROA
ATOM   2188  CG  LEU P 136      5.509  10.079 -21.222  0.00  0.00      PROA
ATOM   2189  HG  LEU P 136      5.071  10.624 -20.358  0.00  0.00      PROA
ATOM   2190  CD1 LEU P 136      6.545   9.064 -20.674  0.00  0.00      PROA
ATOM   2191 HD11 LEU P 136      6.949   8.494 -21.538  0.00  0.00      PROA
ATOM   2192 HD12 LEU P 136      6.069   8.289 -20.035  0.00  0.00      PROA
```

```
ATOM   2193 HD13 LEU P 136       7.272   9.691 -20.117  0.00  0.00      PROA
ATOM   2194  CD2 LEU P 136       6.316  11.111 -22.023  0.00  0.00      PROA
ATOM   2195 HD21 LEU P 136       7.008  10.592 -22.720  0.00  0.00      PROA
ATOM   2196 HD22 LEU P 136       6.966  11.594 -21.262  0.00  0.00      PROA
ATOM   2197 HD23 LEU P 136       5.656  11.828 -22.556  0.00  0.00      PROA
ATOM   2198  C   LEU P 136       2.445   9.909 -23.619  0.00  0.00      PROA
ATOM   2199  O   LEU P 136       2.772  10.217 -24.773  0.00  0.00      PROA
ATOM   2200  N   ALA P 137       1.407   9.157 -23.367  0.00  0.00      PROA
ATOM   2201  HN  ALA P 137       1.126   8.946 -22.434  0.00  0.00      PROA
ATOM   2202  CA  ALA P 137       0.436   8.947 -24.473  0.00  0.00      PROA
ATOM   2203  HA  ALA P 137       0.980   8.518 -25.301  0.00  0.00      PROA
ATOM   2204  CB  ALA P 137      -0.681   7.920 -24.131  0.00  0.00      PROA
ATOM   2205  HB1 ALA P 137      -1.365   7.751 -24.990  0.00  0.00      PROA
ATOM   2206  HB2 ALA P 137      -1.268   8.281 -23.260  0.00  0.00      PROA
ATOM   2207  HB3 ALA P 137      -0.189   6.941 -23.943  0.00  0.00      PROA
ATOM   2208  C   ALA P 137      -0.283  10.196 -24.957  0.00  0.00      PROA
ATOM   2209  O   ALA P 137      -0.351  10.403 -26.160  0.00  0.00      PROA
ATOM   2210  N   ILE P 138      -0.882  10.883 -24.003  0.00  0.00      PROA
ATOM   2211  HN  ILE P 138      -0.821  10.603 -23.048  0.00  0.00      PROA
ATOM   2212  CA  ILE P 138      -1.790  11.989 -24.303  0.00  0.00      PROA
ATOM   2213  HA  ILE P 138      -2.505  11.575 -24.999  0.00  0.00      PROA
ATOM   2214  CB  ILE P 138      -2.621  12.475 -23.113  0.00  0.00      PROA
ATOM   2215  HB  ILE P 138      -1.877  12.621 -22.301  0.00  0.00      PROA
ATOM   2216  CG2 ILE P 138      -3.358  13.771 -23.368  0.00  0.00      PROA
ATOM   2217 HG21 ILE P 138      -3.794  14.078 -22.393  0.00  0.00      PROA
ATOM   2218 HG22 ILE P 138      -4.149  13.765 -24.148  0.00  0.00      PROA
ATOM   2219 HG23 ILE P 138      -2.689  14.604 -23.674  0.00  0.00      PROA
ATOM   2220  CG1 ILE P 138      -3.556  11.363 -22.640  0.00  0.00      PROA
ATOM   2221 HG11 ILE P 138      -4.138  11.599 -21.723  0.00  0.00      PROA
ATOM   2222 HG12 ILE P 138      -3.052  10.448 -22.261  0.00  0.00      PROA
ATOM   2223  CD  ILE P 138      -4.658  10.950 -23.619  0.00  0.00      PROA
ATOM   2224  HD1 ILE P 138      -5.466  10.387 -23.104  0.00  0.00      PROA
ATOM   2225  HD2 ILE P 138      -4.266  10.326 -24.450  0.00  0.00      PROA
ATOM   2226  HD3 ILE P 138      -5.226  11.793 -24.068  0.00  0.00      PROA
ATOM   2227  C   ILE P 138      -1.145  13.129 -25.153  0.00  0.00      PROA
ATOM   2228  O   ILE P 138      -1.723  13.757 -26.058  0.00  0.00      PROA
ATOM   2229  N   VAL P 139       0.152  13.437 -24.808  0.00  0.00      PROA
ATOM   2230  HN  VAL P 139       0.614  12.979 -24.053  0.00  0.00      PROA
ATOM   2231  CA  VAL P 139       0.900  14.541 -25.433  0.00  0.00      PROA
ATOM   2232  HA  VAL P 139       0.127  15.168 -25.854  0.00  0.00      PROA
ATOM   2233  CB  VAL P 139       1.711  15.416 -24.493  0.00  0.00      PROA
ATOM   2234  HB  VAL P 139       1.950  16.336 -25.068  0.00  0.00      PROA
ATOM   2235  CG1 VAL P 139       0.849  15.879 -23.326  0.00  0.00      PROA
ATOM   2236 HG11 VAL P 139       1.371  16.709 -22.804  0.00  0.00      PROA
ATOM   2237 HG12 VAL P 139       0.780  15.070 -22.568  0.00  0.00      PROA
ATOM   2238 HG13 VAL P 139      -0.154  16.169 -23.706  0.00  0.00      PROA
ATOM   2239  CG2 VAL P 139       3.031  14.774 -24.067  0.00  0.00      PROA
ATOM   2240 HG21 VAL P 139       3.603  15.242 -23.237  0.00  0.00      PROA
ATOM   2241 HG22 VAL P 139       3.777  14.796 -24.890  0.00  0.00      PROA
ATOM   2242 HG23 VAL P 139       2.818  13.733 -23.745  0.00  0.00      PROA
ATOM   2243  C   VAL P 139       1.721  14.300 -26.636  0.00  0.00      PROA
ATOM   2244  O   VAL P 139       2.366  15.210 -27.191  0.00  0.00      PROA
ATOM   2245  N   HSD P 140       1.732  13.081 -27.196  0.00  0.00      PROA
ATOM   2246  HN  HSD P 140       1.024  12.457 -26.876  0.00  0.00      PROA
ATOM   2247  CA  HSD P 140       2.516  12.707 -28.328  0.00  0.00      PROA
ATOM   2248  HA  HSD P 140       2.921  13.584 -28.810  0.00  0.00      PROA
ATOM   2249  CB  HSD P 140       3.723  11.853 -27.959  0.00  0.00      PROA
ATOM   2250  HB1 HSD P 140       4.320  11.696 -28.883  0.00  0.00      PROA
ATOM   2251  HB2 HSD P 140       3.402  10.840 -27.633  0.00  0.00      PROA
ATOM   2252  ND1 HSD P 140       5.269  13.665 -27.081  0.00  0.00      PROA
ATOM   2253  HD1 HSD P 140       5.367  14.348 -27.804  0.00  0.00      PROA
```

ATOM	2254	CG	HSD	P	140	4.634	12.408	-26.914	0.00	0.00	PROA
ATOM	2255	CE1	HSD	P	140	6.211	13.751	-26.097	0.00	0.00	PROA
ATOM	2256	HE1	HSD	P	140	6.949	14.552	-26.031	0.00	0.00	PROA
ATOM	2257	NE2	HSD	P	140	6.254	12.689	-25.344	0.00	0.00	PROA
ATOM	2258	CD2	HSD	P	140	5.242	11.837	-25.859	0.00	0.00	PROA
ATOM	2259	HD2	HSD	P	140	5.143	10.886	-25.351	0.00	0.00	PROA
ATOM	2260	C	HSD	P	140	1.689	11.953	-29.278	0.00	0.00	PROA
ATOM	2261	O	HSD	P	140	0.872	11.066	-28.961	0.00	0.00	PROA
ATOM	2262	N	ALA	P	141	1.886	12.227	-30.588	0.00	0.00	PROA
ATOM	2263	HN	ALA	P	141	2.564	12.894	-30.886	0.00	0.00	PROA
ATOM	2264	CA	ALA	P	141	1.184	11.622	-31.697	0.00	0.00	PROA
ATOM	2265	HA	ALA	P	141	0.408	10.993	-31.285	0.00	0.00	PROA
ATOM	2266	CB	ALA	P	141	0.586	12.613	-32.706	0.00	0.00	PROA
ATOM	2267	HB1	ALA	P	141	0.111	12.100	-33.569	0.00	0.00	PROA
ATOM	2268	HB2	ALA	P	141	1.437	13.195	-33.121	0.00	0.00	PROA
ATOM	2269	HB3	ALA	P	141	-0.096	13.342	-32.218	0.00	0.00	PROA
ATOM	2270	C	ALA	P	141	2.158	10.674	-32.456	0.00	0.00	PROA
ATOM	2271	O	ALA	P	141	1.716	10.029	-33.382	0.00	0.00	PROA
ATOM	2272	N	THR	P	142	3.407	10.374	-32.045	0.00	0.00	PROA
ATOM	2273	HN	THR	P	142	3.594	10.601	-31.092	0.00	0.00	PROA
ATOM	2274	CA	THR	P	142	4.435	9.488	-32.528	0.00	0.00	PROA
ATOM	2275	HA	THR	P	142	4.759	9.936	-33.455	0.00	0.00	PROA
ATOM	2276	CB	THR	P	142	5.589	9.362	-31.564	0.00	0.00	PROA
ATOM	2277	HB	THR	P	142	6.277	8.518	-31.785	0.00	0.00	PROA
ATOM	2278	OG1	THR	P	142	5.231	9.335	-30.188	0.00	0.00	PROA
ATOM	2279	HG1	THR	P	142	5.951	9.417	-29.559	0.00	0.00	PROA
ATOM	2280	CG2	THR	P	142	6.414	10.684	-31.664	0.00	0.00	PROA
ATOM	2281	HG21	THR	P	142	6.898	10.889	-32.642	0.00	0.00	PROA
ATOM	2282	HG22	THR	P	142	7.287	10.648	-30.978	0.00	0.00	PROA
ATOM	2283	HG23	THR	P	142	5.811	11.566	-31.360	0.00	0.00	PROA
ATOM	2284	C	THR	P	142	3.938	8.058	-32.752	0.00	0.00	PROA
ATOM	2285	O	THR	P	142	3.245	7.365	-31.960	0.00	0.00	PROA
ATOM	2286	N	ASN	P	143	4.385	7.477	-33.951	0.00	0.00	PROA
ATOM	2287	HN	ASN	P	143	4.927	8.113	-34.495	0.00	0.00	PROA
ATOM	2288	CA	ASN	P	143	4.115	6.131	-34.401	0.00	0.00	PROA
ATOM	2289	HA	ASN	P	143	3.048	5.967	-34.363	0.00	0.00	PROA
ATOM	2290	CB	ASN	P	143	4.589	5.961	-35.873	0.00	0.00	PROA
ATOM	2291	HB1	ASN	P	143	5.625	6.301	-36.085	0.00	0.00	PROA
ATOM	2292	HB2	ASN	P	143	3.875	6.463	-36.560	0.00	0.00	PROA
ATOM	2293	CG	ASN	P	143	4.603	4.445	-36.275	0.00	0.00	PROA
ATOM	2294	OD1	ASN	P	143	5.666	3.889	-36.443	0.00	0.00	PROA
ATOM	2295	ND2	ASN	P	143	3.414	3.798	-36.424	0.00	0.00	PROA
ATOM	2296	HD21	ASN	P	143	3.454	2.922	-36.905	0.00	0.00	PROA
ATOM	2297	HD22	ASN	P	143	2.556	4.221	-36.134	0.00	0.00	PROA
ATOM	2298	C	ASN	P	143	4.696	5.116	-33.407	0.00	0.00	PROA
ATOM	2299	O	ASN	P	143	4.035	4.257	-32.848	0.00	0.00	PROA
ATOM	2300	N	SER	P	144	6.024	5.224	-33.227	0.00	0.00	PROA
ATOM	2301	HN	SER	P	144	6.463	5.953	-33.745	0.00	0.00	PROA
ATOM	2302	CA	SER	P	144	6.828	4.392	-32.280	0.00	0.00	PROA
ATOM	2303	HA	SER	P	144	6.519	3.368	-32.429	0.00	0.00	PROA
ATOM	2304	CB	SER	P	144	8.319	4.632	-32.544	0.00	0.00	PROA
ATOM	2305	HB1	SER	P	144	8.456	5.716	-32.343	0.00	0.00	PROA
ATOM	2306	HB2	SER	P	144	8.532	4.451	-33.619	0.00	0.00	PROA
ATOM	2307	OG	SER	P	144	9.214	3.806	-31.763	0.00	0.00	PROA
ATOM	2308	HG1	SER	P	144	9.366	3.025	-32.299	0.00	0.00	PROA
ATOM	2309	C	SER	P	144	6.573	4.646	-30.847	0.00	0.00	PROA
ATOM	2310	O	SER	P	144	6.447	5.743	-30.347	0.00	0.00	PROA
ATOM	2311	N	GLN	P	145	6.522	3.540	-30.112	0.00	0.00	PROA
ATOM	2312	HN	GLN	P	145	6.855	2.696	-30.527	0.00	0.00	PROA
ATOM	2313	CA	GLN	P	145	6.205	3.463	-28.653	0.00	0.00	PROA
ATOM	2314	HA	GLN	P	145	5.930	4.448	-28.306	0.00	0.00	PROA

```
ATOM   2315  CB  GLN P 145       5.107   2.423 -28.275  0.00  0.00      PROA
ATOM   2316  HB1 GLN P 145       4.944   2.421 -27.177  0.00  0.00      PROA
ATOM   2317  HB2 GLN P 145       5.503   1.407 -28.486  0.00  0.00      PROA
ATOM   2318  CG  GLN P 145       3.820   2.679 -29.076  0.00  0.00      PROA
ATOM   2319  HG1 GLN P 145       3.119   1.862 -28.802  0.00  0.00      PROA
ATOM   2320  HG2 GLN P 145       4.052   2.614 -30.160  0.00  0.00      PROA
ATOM   2321  CD  GLN P 145       3.149   4.063 -28.794  0.00  0.00      PROA
ATOM   2322  OE1 GLN P 145       3.180   4.451 -27.595  0.00  0.00      PROA
ATOM   2323  NE2 GLN P 145       2.580   4.784 -29.798  0.00  0.00      PROA
ATOM   2324 HE21 GLN P 145       2.215   5.698 -29.622  0.00  0.00      PROA
ATOM   2325 HE22 GLN P 145       2.654   4.499 -30.754  0.00  0.00      PROA
ATOM   2326  C   GLN P 145       7.388   3.022 -27.973  0.00  0.00      PROA
ATOM   2327  O   GLN P 145       7.331   2.700 -26.774  0.00  0.00      PROA
ATOM   2328  N   ARG P 146       8.564   3.110 -28.698  0.00  0.00      PROA
ATOM   2329  HN  ARG P 146       8.570   3.190 -29.691  0.00  0.00      PROA
ATOM   2330  CA  ARG P 146       9.767   2.582 -28.037  0.00  0.00      PROA
ATOM   2331  HA  ARG P 146       9.471   1.563 -27.833  0.00  0.00      PROA
ATOM   2332  CB  ARG P 146      10.920   2.433 -29.035  0.00  0.00      PROA
ATOM   2333  HB1 ARG P 146      10.793   3.143 -29.880  0.00  0.00      PROA
ATOM   2334  HB2 ARG P 146      10.673   1.474 -29.537  0.00  0.00      PROA
ATOM   2335  CG  ARG P 146      12.481   2.515 -28.544  0.00  0.00      PROA
ATOM   2336  HG1 ARG P 146      12.616   3.368 -27.845  0.00  0.00      PROA
ATOM   2337  HG2 ARG P 146      13.066   2.899 -29.407  0.00  0.00      PROA
ATOM   2338  CD  ARG P 146      13.009   1.228 -27.928  0.00  0.00      PROA
ATOM   2339  HD1 ARG P 146      13.387   0.533 -28.708  0.00  0.00      PROA
ATOM   2340  HD2 ARG P 146      12.349   0.864 -27.112  0.00  0.00      PROA
ATOM   2341  NE  ARG P 146      14.188   1.601 -27.101  0.00  0.00      PROA
ATOM   2342  HE  ARG P 146      14.289   2.572 -26.883  0.00  0.00      PROA
ATOM   2343  CZ  ARG P 146      15.053   0.827 -26.487  0.00  0.00      PROA
ATOM   2344  NH1 ARG P 146      14.998  -0.422 -26.543  0.00  0.00      PROA
ATOM   2345 HH11 ARG P 146      14.691  -0.865 -27.385  0.00  0.00      PROA
ATOM   2346 HH12 ARG P 146      15.662  -1.008 -26.078  0.00  0.00      PROA
ATOM   2347  NH2 ARG P 146      15.852   1.447 -25.677  0.00  0.00      PROA
ATOM   2348 HH21 ARG P 146      15.735   2.436 -25.585  0.00  0.00      PROA
ATOM   2349 HH22 ARG P 146      16.484   0.859 -25.172  0.00  0.00      PROA
ATOM   2350  C   ARG P 146      10.226   3.125 -26.677  0.00  0.00      PROA
ATOM   2351  O   ARG P 146      10.541   2.232 -25.831  0.00  0.00      PROA
ATOM   2352  N   PRO P 147      10.331   4.412 -26.252  0.00  0.00      PROA
ATOM   2353  CD  PRO P 147      10.188   5.562 -27.117  0.00  0.00      PROA
ATOM   2354  HD1 PRO P 147       9.149   5.949 -27.053  0.00  0.00      PROA
ATOM   2355  HD2 PRO P 147      10.488   5.442 -28.180  0.00  0.00      PROA
ATOM   2356  CA  PRO P 147      10.724   4.769 -24.907  0.00  0.00      PROA
ATOM   2357  HA  PRO P 147      11.644   4.290 -24.606  0.00  0.00      PROA
ATOM   2358  CB  PRO P 147      11.032   6.267 -24.941  0.00  0.00      PROA
ATOM   2359  HB1 PRO P 147      12.011   6.406 -24.434  0.00  0.00      PROA
ATOM   2360  HB2 PRO P 147      10.310   6.816 -24.298  0.00  0.00      PROA
ATOM   2361  CG  PRO P 147      11.068   6.572 -26.413  0.00  0.00      PROA
ATOM   2362  HG1 PRO P 147      10.599   7.574 -26.508  0.00  0.00      PROA
ATOM   2363  HG2 PRO P 147      12.099   6.688 -26.812  0.00  0.00      PROA
ATOM   2364  C   PRO P 147       9.748   4.458 -23.762  0.00  0.00      PROA
ATOM   2365  O   PRO P 147      10.225   4.051 -22.704  0.00  0.00      PROA
ATOM   2366  N   ARG P 148       8.428   4.521 -24.063  0.00  0.00      PROA
ATOM   2367  HN  ARG P 148       8.209   4.825 -24.987  0.00  0.00      PROA
ATOM   2368  CA  ARG P 148       7.425   4.073 -23.082  0.00  0.00      PROA
ATOM   2369  HA  ARG P 148       7.489   4.579 -22.131  0.00  0.00      PROA
ATOM   2370  CB  ARG P 148       6.024   4.224 -23.668  0.00  0.00      PROA
ATOM   2371  HB1 ARG P 148       5.307   3.753 -22.962  0.00  0.00      PROA
ATOM   2372  HB2 ARG P 148       5.997   3.606 -24.591  0.00  0.00      PROA
ATOM   2373  CG  ARG P 148       5.464   5.607 -23.759  0.00  0.00      PROA
ATOM   2374  HG1 ARG P 148       6.145   6.350 -24.228  0.00  0.00      PROA
ATOM   2375  HG2 ARG P 148       5.391   6.053 -22.744  0.00  0.00      PROA
```

```
ATOM   2376  CD  ARG P 148       4.111   5.571 -24.518  0.00  0.00      PROA
ATOM   2377  HD1 ARG P 148       4.241   5.198 -25.557  0.00  0.00      PROA
ATOM   2378  HD2 ARG P 148       3.774   6.629 -24.495  0.00  0.00      PROA
ATOM   2379  NE  ARG P 148       3.161   4.765 -23.722  0.00  0.00      PROA
ATOM   2380  HE  ARG P 148       3.326   4.572 -22.755  0.00  0.00      PROA
ATOM   2381  CZ  ARG P 148       1.976   4.372 -24.147  0.00  0.00      PROA
ATOM   2382  NH1 ARG P 148       1.623   4.512 -25.378  0.00  0.00      PROA
ATOM   2383 HH11 ARG P 148       2.359   4.547 -26.054  0.00  0.00      PROA
ATOM   2384 HH12 ARG P 148       0.658   4.391 -25.611  0.00  0.00      PROA
ATOM   2385  NH2 ARG P 148       1.249   3.712 -23.241  0.00  0.00      PROA
ATOM   2386 HH21 ARG P 148       1.434   3.857 -22.270  0.00  0.00      PROA
ATOM   2387 HH22 ARG P 148       0.262   3.641 -23.391  0.00  0.00      PROA
ATOM   2388  C   ARG P 148       7.534   2.538 -22.686  0.00  0.00      PROA
ATOM   2389  O   ARG P 148       7.361   2.094 -21.576  0.00  0.00      PROA
ATOM   2390  N   LYS P 149       7.918   1.760 -23.680  0.00  0.00      PROA
ATOM   2391  HN  LYS P 149       8.170   1.972 -24.621  0.00  0.00      PROA
ATOM   2392  CA  LYS P 149       8.203   0.358 -23.441  0.00  0.00      PROA
ATOM   2393  HA  LYS P 149       7.331  -0.003 -22.916  0.00  0.00      PROA
ATOM   2394  CB  LYS P 149       8.252  -0.256 -24.899  0.00  0.00      PROA
ATOM   2395  HB1 LYS P 149       8.750   0.439 -25.609  0.00  0.00      PROA
ATOM   2396  HB2 LYS P 149       7.187  -0.319 -25.212  0.00  0.00      PROA
ATOM   2397  CG  LYS P 149       8.841  -1.709 -24.845  0.00  0.00      PROA
ATOM   2398  HG1 LYS P 149       8.484  -2.344 -25.683  0.00  0.00      PROA
ATOM   2399  HG2 LYS P 149       8.461  -2.064 -23.863  0.00  0.00      PROA
ATOM   2400  CD  LYS P 149      10.395  -1.718 -25.050  0.00  0.00      PROA
ATOM   2401  HD1 LYS P 149      10.745  -2.382 -24.231  0.00  0.00      PROA
ATOM   2402  HD2 LYS P 149      10.721  -0.657 -25.009  0.00  0.00      PROA
ATOM   2403  CE  LYS P 149      10.822  -2.174 -26.466  0.00  0.00      PROA
ATOM   2404  HE1 LYS P 149      11.883  -1.858 -26.559  0.00  0.00      PROA
ATOM   2405  HE2 LYS P 149      10.219  -1.964 -27.375  0.00  0.00      PROA
ATOM   2406  NZ  LYS P 149      10.819  -3.628 -26.533  0.00  0.00      PROA
ATOM   2407  HZ1 LYS P 149      10.067  -4.006 -25.921  0.00  0.00      PROA
ATOM   2408  HZ2 LYS P 149      11.733  -3.995 -26.199  0.00  0.00      PROA
ATOM   2409  HZ3 LYS P 149      10.581  -4.000 -27.474  0.00  0.00      PROA
ATOM   2410  C   LYS P 149       9.453   0.049 -22.561  0.00  0.00      PROA
ATOM   2411  O   LYS P 149       9.449  -0.756 -21.653  0.00  0.00      PROA
ATOM   2412  N   LEU P 150      10.504   0.834 -22.780  0.00  0.00      PROA
ATOM   2413  HN  LEU P 150      10.466   1.511 -23.511  0.00  0.00      PROA
ATOM   2414  CA  LEU P 150      11.674   0.877 -21.892  0.00  0.00      PROA
ATOM   2415  HA  LEU P 150      12.212  -0.059 -21.926  0.00  0.00      PROA
ATOM   2416  CB  LEU P 150      12.764   1.928 -22.379  0.00  0.00      PROA
ATOM   2417  HB1 LEU P 150      12.386   2.968 -22.282  0.00  0.00      PROA
ATOM   2418  HB2 LEU P 150      12.898   1.826 -23.478  0.00  0.00      PROA
ATOM   2419  CG  LEU P 150      14.196   1.840 -21.709  0.00  0.00      PROA
ATOM   2420  HG  LEU P 150      14.092   1.794 -20.604  0.00  0.00      PROA
ATOM   2421  CD1 LEU P 150      14.761   0.412 -22.063  0.00  0.00      PROA
ATOM   2422 HD11 LEU P 150      14.809   0.221 -23.156  0.00  0.00      PROA
ATOM   2423 HD12 LEU P 150      14.200  -0.454 -21.650  0.00  0.00      PROA
ATOM   2424 HD13 LEU P 150      15.791   0.474 -21.651  0.00  0.00      PROA
ATOM   2425  CD2 LEU P 150      15.094   2.892 -22.300  0.00  0.00      PROA
ATOM   2426 HD21 LEU P 150      14.562   3.866 -22.354  0.00  0.00      PROA
ATOM   2427 HD22 LEU P 150      15.213   2.652 -23.378  0.00  0.00      PROA
ATOM   2428 HD23 LEU P 150      16.126   3.060 -21.925  0.00  0.00      PROA
ATOM   2429  C   LEU P 150      11.334   1.330 -20.444  0.00  0.00      PROA
ATOM   2430  O   LEU P 150      11.875   0.845 -19.463  0.00  0.00      PROA
ATOM   2431  N   LEU P 151      10.440   2.338 -20.327  0.00  0.00      PROA
ATOM   2432  HN  LEU P 151      10.176   2.789 -21.176  0.00  0.00      PROA
ATOM   2433  CA  LEU P 151       9.893   2.845 -19.091  0.00  0.00      PROA
ATOM   2434  HA  LEU P 151      10.692   3.068 -18.400  0.00  0.00      PROA
ATOM   2435  CB  LEU P 151       9.209   4.260 -19.298  0.00  0.00      PROA
ATOM   2436  HB1 LEU P 151       8.392   4.137 -20.041  0.00  0.00      PROA
```

ATOM	2437	HB2	LEU	P	151	9.910	4.997	-19.745	0.00	0.00	PROA
ATOM	2438	CG	LEU	P	151	8.590	4.842	-18.025	0.00	0.00	PROA
ATOM	2439	HG	LEU	P	151	7.933	4.052	-17.603	0.00	0.00	PROA
ATOM	2440	CD1	LEU	P	151	9.639	5.172	-17.039	0.00	0.00	PROA
ATOM	2441	HD11	LEU	P	151	9.233	5.319	-16.016	0.00	0.00	PROA
ATOM	2442	HD12	LEU	P	151	10.189	6.114	-17.253	0.00	0.00	PROA
ATOM	2443	HD13	LEU	P	151	10.263	4.263	-16.906	0.00	0.00	PROA
ATOM	2444	CD2	LEU	P	151	7.806	6.150	-18.347	0.00	0.00	PROA
ATOM	2445	HD21	LEU	P	151	6.872	5.840	-18.862	0.00	0.00	PROA
ATOM	2446	HD22	LEU	P	151	8.414	6.716	-19.084	0.00	0.00	PROA
ATOM	2447	HD23	LEU	P	151	7.677	6.842	-17.487	0.00	0.00	PROA
ATOM	2448	C	LEU	P	151	9.048	1.898	-18.292	0.00	0.00	PROA
ATOM	2449	O	LEU	P	151	9.100	1.950	-17.048	0.00	0.00	PROA
ATOM	2450	N	ALA	P	152	8.327	0.938	-18.852	0.00	0.00	PROA
ATOM	2451	HN	ALA	P	152	8.250	0.890	-19.845	0.00	0.00	PROA
ATOM	2452	CA	ALA	P	152	7.584	-0.100	-18.060	0.00	0.00	PROA
ATOM	2453	HA	ALA	P	152	7.164	0.321	-17.159	0.00	0.00	PROA
ATOM	2454	CB	ALA	P	152	6.424	-0.655	-18.987	0.00	0.00	PROA
ATOM	2455	HB1	ALA	P	152	5.733	-1.248	-18.351	0.00	0.00	PROA
ATOM	2456	HB2	ALA	P	152	6.865	-1.400	-19.684	0.00	0.00	PROA
ATOM	2457	HB3	ALA	P	152	5.874	0.168	-19.492	0.00	0.00	PROA
ATOM	2458	C	ALA	P	152	8.407	-1.242	-17.490	0.00	0.00	PROA
ATOM	2459	O	ALA	P	152	7.908	-2.008	-16.745	0.00	0.00	PROA
ATOM	2460	N	GLU	P	153	9.581	-1.339	-17.863	0.00	0.00	PROA
ATOM	2461	HN	GLU	P	153	10.038	-0.790	-18.559	0.00	0.00	PROA
ATOM	2462	CA	GLU	P	153	10.424	-2.414	-17.417	0.00	0.00	PROA
ATOM	2463	HA	GLU	P	153	9.936	-3.001	-16.653	0.00	0.00	PROA
ATOM	2464	CB	GLU	P	153	10.715	-3.201	-18.726	0.00	0.00	PROA
ATOM	2465	HB1	GLU	P	153	11.504	-3.951	-18.503	0.00	0.00	PROA
ATOM	2466	HB2	GLU	P	153	11.114	-2.438	-19.428	0.00	0.00	PROA
ATOM	2467	CG	GLU	P	153	9.444	-3.812	-19.466	0.00	0.00	PROA
ATOM	2468	HG1	GLU	P	153	8.994	-3.067	-20.156	0.00	0.00	PROA
ATOM	2469	HG2	GLU	P	153	8.720	-4.220	-18.729	0.00	0.00	PROA
ATOM	2470	CD	GLU	P	153	9.795	-4.978	-20.373	0.00	0.00	PROA
ATOM	2471	OE1	GLU	P	153	10.622	-4.712	-21.289	0.00	0.00	PROA
ATOM	2472	OE2	GLU	P	153	9.281	-6.106	-20.345	0.00	0.00	PROA
ATOM	2473	C	GLU	P	153	11.757	-1.965	-16.755	0.00	0.00	PROA
ATOM	2474	O	GLU	P	153	11.818	-1.694	-15.598	0.00	0.00	PROA
ATOM	2475	N	LYS	P	154	12.820	-1.884	-17.612	0.00	0.00	PROA
ATOM	2476	HN	LYS	P	154	12.803	-2.094	-18.586	0.00	0.00	PROA
ATOM	2477	CA	LYS	P	154	14.251	-1.833	-17.196	0.00	0.00	PROA
ATOM	2478	HA	LYS	P	154	14.382	-2.819	-16.776	0.00	0.00	PROA
ATOM	2479	CB	LYS	P	154	15.187	-1.645	-18.418	0.00	0.00	PROA
ATOM	2480	HB1	LYS	P	154	16.234	-1.859	-18.113	0.00	0.00	PROA
ATOM	2481	HB2	LYS	P	154	15.135	-0.587	-18.752	0.00	0.00	PROA
ATOM	2482	CG	LYS	P	154	14.823	-2.530	-19.647	0.00	0.00	PROA
ATOM	2483	HG1	LYS	P	154	15.472	-2.265	-20.509	0.00	0.00	PROA
ATOM	2484	HG2	LYS	P	154	13.750	-2.448	-19.924	0.00	0.00	PROA
ATOM	2485	CD	LYS	P	154	15.057	-4.093	-19.438	0.00	0.00	PROA
ATOM	2486	HD1	LYS	P	154	14.953	-4.415	-18.380	0.00	0.00	PROA
ATOM	2487	HD2	LYS	P	154	16.157	-4.248	-19.450	0.00	0.00	PROA
ATOM	2488	CE	LYS	P	154	14.465	-4.946	-20.487	0.00	0.00	PROA
ATOM	2489	HE1	LYS	P	154	14.810	-4.603	-21.485	0.00	0.00	PROA
ATOM	2490	HE2	LYS	P	154	13.389	-4.815	-20.241	0.00	0.00	PROA
ATOM	2491	NZ	LYS	P	154	14.929	-6.329	-20.330	0.00	0.00	PROA
ATOM	2492	HZ1	LYS	P	154	15.958	-6.324	-20.182	0.00	0.00	PROA
ATOM	2493	HZ2	LYS	P	154	14.823	-6.809	-21.246	0.00	0.00	PROA
ATOM	2494	HZ3	LYS	P	154	14.445	-6.852	-19.572	0.00	0.00	PROA
ATOM	2495	C	LYS	P	154	14.543	-0.708	-16.247	0.00	0.00	PROA
ATOM	2496	O	LYS	P	154	15.266	-0.921	-15.272	0.00	0.00	PROA
ATOM	2497	N	VAL	P	155	13.967	0.452	-16.547	0.00	0.00	PROA

```
ATOM   2498  HN  VAL P 155      13.339   0.553 -17.315  0.00  0.00      PROA
ATOM   2499  CA  VAL P 155      14.068   1.676 -15.726  0.00  0.00      PROA
ATOM   2500  HA  VAL P 155      15.078   1.898 -15.416  0.00  0.00      PROA
ATOM   2501  CB  VAL P 155      13.482   2.812 -16.560  0.00  0.00      PROA
ATOM   2502  HB  VAL P 155      12.486   2.388 -16.810  0.00  0.00      PROA
ATOM   2503  CG1 VAL P 155      13.322   4.147 -15.741  0.00  0.00      PROA
ATOM   2504 HG11 VAL P 155      12.715   3.996 -14.822  0.00  0.00      PROA
ATOM   2505 HG12 VAL P 155      12.858   4.990 -16.297  0.00  0.00      PROA
ATOM   2506 HG13 VAL P 155      14.333   4.465 -15.407  0.00  0.00      PROA
ATOM   2507  CG2 VAL P 155      14.283   3.037 -17.847  0.00  0.00      PROA
ATOM   2508 HG21 VAL P 155      14.457   2.064 -18.353  0.00  0.00      PROA
ATOM   2509 HG22 VAL P 155      15.285   3.465 -17.631  0.00  0.00      PROA
ATOM   2510 HG23 VAL P 155      13.706   3.772 -18.448  0.00  0.00      PROA
ATOM   2511  C   VAL P 155      13.354   1.414 -14.392  0.00  0.00      PROA
ATOM   2512  O   VAL P 155      13.861   1.722 -13.262  0.00  0.00      PROA
ATOM   2513  N   VAL P 156      12.176   0.840 -14.358  0.00  0.00      PROA
ATOM   2514  HN  VAL P 156      11.723   0.503 -15.179  0.00  0.00      PROA
ATOM   2515  CA  VAL P 156      11.516   0.537 -13.156  0.00  0.00      PROA
ATOM   2516  HA  VAL P 156      11.483   1.402 -12.510  0.00  0.00      PROA
ATOM   2517  CB  VAL P 156      10.082  -0.091 -13.300  0.00  0.00      PROA
ATOM   2518  HB  VAL P 156      10.116  -1.172 -13.553  0.00  0.00      PROA
ATOM   2519  CG1 VAL P 156       9.370   0.097 -11.918  0.00  0.00      PROA
ATOM   2520 HG11 VAL P 156       8.311  -0.231 -11.990  0.00  0.00      PROA
ATOM   2521 HG12 VAL P 156       9.303   1.165 -11.620  0.00  0.00      PROA
ATOM   2522 HG13 VAL P 156       9.798  -0.522 -11.102  0.00  0.00      PROA
ATOM   2523  CG2 VAL P 156       9.299   0.584 -14.391  0.00  0.00      PROA
ATOM   2524 HG21 VAL P 156       8.270   0.165 -14.375  0.00  0.00      PROA
ATOM   2525 HG22 VAL P 156       9.740   0.205 -15.338  0.00  0.00      PROA
ATOM   2526 HG23 VAL P 156       9.358   1.692 -14.348  0.00  0.00      PROA
ATOM   2527  C   VAL P 156      12.319  -0.457 -12.279  0.00  0.00      PROA
ATOM   2528  O   VAL P 156      12.531  -0.314 -11.080  0.00  0.00      PROA
ATOM   2529  N   TYR P 157      12.922  -1.498 -12.894  0.00  0.00      PROA
ATOM   2530  HN  TYR P 157      12.764  -1.651 -13.866  0.00  0.00      PROA
ATOM   2531  CA  TYR P 157      13.751  -2.524 -12.177  0.00  0.00      PROA
ATOM   2532  HA  TYR P 157      13.013  -2.848 -11.458  0.00  0.00      PROA
ATOM   2533  CB  TYR P 157      14.170  -3.641 -13.145  0.00  0.00      PROA
ATOM   2534  HB1 TYR P 157      14.620  -4.415 -12.486  0.00  0.00      PROA
ATOM   2535  HB2 TYR P 157      14.896  -3.361 -13.937  0.00  0.00      PROA
ATOM   2536  CG  TYR P 157      12.887  -4.182 -13.872  0.00  0.00      PROA
ATOM   2537  CD1 TYR P 157      13.011  -4.825 -15.141  0.00  0.00      PROA
ATOM   2538  HD1 TYR P 157      13.973  -4.924 -15.623  0.00  0.00      PROA
ATOM   2539  CE1 TYR P 157      11.879  -5.447 -15.772  0.00  0.00      PROA
ATOM   2540  HE1 TYR P 157      11.939  -5.996 -16.700  0.00  0.00      PROA
ATOM   2541  CZ  TYR P 157      10.685  -5.357 -15.114  0.00  0.00      PROA
ATOM   2542  OH  TYR P 157       9.607  -5.836 -15.784  0.00  0.00      PROA
ATOM   2543  HH  TYR P 157       9.949  -6.186 -16.610  0.00  0.00      PROA
ATOM   2544  CD2 TYR P 157      11.565  -4.298 -13.206  0.00  0.00      PROA
ATOM   2545  HD2 TYR P 157      11.382  -3.908 -12.216  0.00  0.00      PROA
ATOM   2546  CE2 TYR P 157      10.536  -4.930 -13.834  0.00  0.00      PROA
ATOM   2547  HE2 TYR P 157       9.559  -4.878 -13.377  0.00  0.00      PROA
ATOM   2548  C   TYR P 157      14.963  -1.963 -11.474  0.00  0.00      PROA
ATOM   2549  O   TYR P 157      15.143  -2.365 -10.288  0.00  0.00      PROA
ATOM   2550  N   VAL P 158      15.726  -1.064 -11.983  0.00  0.00      PROA
ATOM   2551  HN  VAL P 158      15.632  -0.786 -12.936  0.00  0.00      PROA
ATOM   2552  CA  VAL P 158      16.813  -0.555 -11.213  0.00  0.00      PROA
ATOM   2553  HA  VAL P 158      17.217  -1.205 -10.451  0.00  0.00      PROA
ATOM   2554  CB  VAL P 158      18.037  -0.182 -12.085  0.00  0.00      PROA
ATOM   2555  HB  VAL P 158      18.786   0.230 -11.375  0.00  0.00      PROA
ATOM   2556  CG1 VAL P 158      18.555  -1.430 -12.864  0.00  0.00      PROA
ATOM   2557 HG11 VAL P 158      17.793  -1.598 -13.654  0.00  0.00      PROA
ATOM   2558 HG12 VAL P 158      18.611  -2.290 -12.164  0.00  0.00      PROA
```

ATOM	2559	HG13	VAL	P	158	19.528	-1.131	-13.309	0.00	0.00	PROA
ATOM	2560	CG2	VAL	P	158	17.766	0.986	-13.056	0.00	0.00	PROA
ATOM	2561	HG21	VAL	P	158	17.053	0.580	-13.806	0.00	0.00	PROA
ATOM	2562	HG22	VAL	P	158	18.648	1.414	-13.580	0.00	0.00	PROA
ATOM	2563	HG23	VAL	P	158	17.311	1.819	-12.477	0.00	0.00	PROA
ATOM	2564	C	VAL	P	158	16.330	0.806	-10.460	0.00	0.00	PROA
ATOM	2565	O	VAL	P	158	17.017	1.160	-9.540	0.00	0.00	PROA
ATOM	2566	N	GLY	P	159	15.195	1.398	-10.768	0.00	0.00	PROA
ATOM	2567	HN	GLY	P	159	14.626	0.955	-11.457	0.00	0.00	PROA
ATOM	2568	CA	GLY	P	159	14.961	2.803	-10.268	0.00	0.00	PROA
ATOM	2569	HA1	GLY	P	159	14.653	3.393	-11.119	0.00	0.00	PROA
ATOM	2570	HA2	GLY	P	159	15.838	3.298	-9.879	0.00	0.00	PROA
ATOM	2571	C	GLY	P	159	13.986	2.801	-9.166	0.00	0.00	PROA
ATOM	2572	O	GLY	P	159	13.982	3.671	-8.316	0.00	0.00	PROA
ATOM	2573	N	VAL	P	160	13.106	1.795	-9.146	0.00	0.00	PROA
ATOM	2574	HN	VAL	P	160	13.090	1.120	-9.880	0.00	0.00	PROA
ATOM	2575	CA	VAL	P	160	12.092	1.664	-8.144	0.00	0.00	PROA
ATOM	2576	HA	VAL	P	160	12.105	2.598	-7.602	0.00	0.00	PROA
ATOM	2577	CB	VAL	P	160	10.654	1.719	-8.695	0.00	0.00	PROA
ATOM	2578	HB	VAL	P	160	10.485	0.753	-9.216	0.00	0.00	PROA
ATOM	2579	CG1	VAL	P	160	9.641	1.761	-7.544	0.00	0.00	PROA
ATOM	2580	HG11	VAL	P	160	8.580	1.928	-7.829	0.00	0.00	PROA
ATOM	2581	HG12	VAL	P	160	9.950	2.564	-6.842	0.00	0.00	PROA
ATOM	2582	HG13	VAL	P	160	9.675	0.840	-6.924	0.00	0.00	PROA
ATOM	2583	CG2	VAL	P	160	10.339	2.922	-9.569	0.00	0.00	PROA
ATOM	2584	HG21	VAL	P	160	10.917	2.769	-10.506	0.00	0.00	PROA
ATOM	2585	HG22	VAL	P	160	10.677	3.829	-9.025	0.00	0.00	PROA
ATOM	2586	HG23	VAL	P	160	9.231	2.906	-9.659	0.00	0.00	PROA
ATOM	2587	C	VAL	P	160	12.390	0.475	-7.273	0.00	0.00	PROA
ATOM	2588	O	VAL	P	160	12.614	0.737	-6.100	0.00	0.00	PROA
ATOM	2589	N	TRP	P	161	12.521	-0.709	-7.836	0.00	0.00	PROA
ATOM	2590	HN	TRP	P	161	12.404	-0.875	-8.813	0.00	0.00	PROA
ATOM	2591	CA	TRP	P	161	12.839	-1.838	-7.019	0.00	0.00	PROA
ATOM	2592	HA	TRP	P	161	12.008	-1.892	-6.331	0.00	0.00	PROA
ATOM	2593	CB	TRP	P	161	12.641	-3.198	-7.803	0.00	0.00	PROA
ATOM	2594	HB1	TRP	P	161	12.991	-4.068	-7.207	0.00	0.00	PROA
ATOM	2595	HB2	TRP	P	161	13.161	-3.138	-8.783	0.00	0.00	PROA
ATOM	2596	CG	TRP	P	161	11.303	-3.569	-8.025	0.00	0.00	PROA
ATOM	2597	CD1	TRP	P	161	10.624	-3.520	-9.245	0.00	0.00	PROA
ATOM	2598	HD1	TRP	P	161	10.846	-2.913	-10.110	0.00	0.00	PROA
ATOM	2599	NE1	TRP	P	161	9.404	-4.076	-9.125	0.00	0.00	PROA
ATOM	2600	HE1	TRP	P	161	8.684	-4.206	-9.772	0.00	0.00	PROA
ATOM	2601	CE2	TRP	P	161	9.223	-4.473	-7.808	0.00	0.00	PROA
ATOM	2602	CD2	TRP	P	161	10.400	-4.183	-7.096	0.00	0.00	PROA
ATOM	2603	CE3	TRP	P	161	10.467	-4.541	-5.717	0.00	0.00	PROA
ATOM	2604	HE3	TRP	P	161	11.330	-4.291	-5.118	0.00	0.00	PROA
ATOM	2605	CZ3	TRP	P	161	9.321	-5.159	-5.142	0.00	0.00	PROA
ATOM	2606	HZ3	TRP	P	161	9.365	-5.422	-4.096	0.00	0.00	PROA
ATOM	2607	CZ2	TRP	P	161	8.119	-5.043	-7.212	0.00	0.00	PROA
ATOM	2608	HZ2	TRP	P	161	7.238	-5.224	-7.810	0.00	0.00	PROA
ATOM	2609	CH2	TRP	P	161	8.235	-5.401	-5.894	0.00	0.00	PROA
ATOM	2610	HH2	TRP	P	161	7.395	-5.836	-5.372	0.00	0.00	PROA
ATOM	2611	C	TRP	P	161	14.090	-1.917	-6.227	0.00	0.00	PROA
ATOM	2612	O	TRP	P	161	14.001	-2.180	-5.063	0.00	0.00	PROA
ATOM	2613	N	ILE	P	162	15.281	-1.642	-6.897	0.00	0.00	PROA
ATOM	2614	HN	ILE	P	162	15.325	-1.437	-7.872	0.00	0.00	PROA
ATOM	2615	CA	ILE	P	162	16.538	-1.641	-6.122	0.00	0.00	PROA
ATOM	2616	HA	ILE	P	162	16.507	-2.620	-5.669	0.00	0.00	PROA
ATOM	2617	CB	ILE	P	162	17.743	-1.546	-7.030	0.00	0.00	PROA
ATOM	2618	HB	ILE	P	162	17.437	-0.721	-7.707	0.00	0.00	PROA
ATOM	2619	CG2	ILE	P	162	19.079	-1.214	-6.290	0.00	0.00	PROA

ATOM	2620	HG21	ILE	P	162	19.336	-1.965	-5.512	0.00	0.00	PROA
ATOM	2621	HG22	ILE	P	162	18.983	-0.241	-5.762	0.00	0.00	PROA
ATOM	2622	HG23	ILE	P	162	19.884	-1.199	-7.056	0.00	0.00	PROA
ATOM	2623	CG1	ILE	P	162	17.810	-2.807	-7.952	0.00	0.00	PROA
ATOM	2624	HG11	ILE	P	162	16.941	-2.829	-8.644	0.00	0.00	PROA
ATOM	2625	HG12	ILE	P	162	17.719	-3.751	-7.372	0.00	0.00	PROA
ATOM	2626	CD	ILE	P	162	18.964	-2.922	-8.916	0.00	0.00	PROA
ATOM	2627	HD1	ILE	P	162	19.922	-2.922	-8.352	0.00	0.00	PROA
ATOM	2628	HD2	ILE	P	162	19.020	-2.149	-9.711	0.00	0.00	PROA
ATOM	2629	HD3	ILE	P	162	18.989	-3.949	-9.339	0.00	0.00	PROA
ATOM	2630	C	ILE	P	162	16.532	-0.623	-5.010	0.00	0.00	PROA
ATOM	2631	O	ILE	P	162	16.929	-0.983	-3.955	0.00	0.00	PROA
ATOM	2632	N	PRO	P	163	16.047	0.626	-5.103	0.00	0.00	PROA
ATOM	2633	CD	PRO	P	163	16.036	1.418	-6.356	0.00	0.00	PROA
ATOM	2634	HD1	PRO	P	163	15.085	1.386	-6.930	0.00	0.00	PROA
ATOM	2635	HD2	PRO	P	163	16.760	1.051	-7.115	0.00	0.00	PROA
ATOM	2636	CA	PRO	P	163	16.067	1.538	-3.962	0.00	0.00	PROA
ATOM	2637	HA	PRO	P	163	17.035	1.381	-3.511	0.00	0.00	PROA
ATOM	2638	CB	PRO	P	163	15.815	2.925	-4.558	0.00	0.00	PROA
ATOM	2639	HB1	PRO	P	163	16.329	3.637	-3.878	0.00	0.00	PROA
ATOM	2640	HB2	PRO	P	163	14.733	3.175	-4.558	0.00	0.00	PROA
ATOM	2641	CG	PRO	P	163	16.435	2.859	-5.957	0.00	0.00	PROA
ATOM	2642	HG1	PRO	P	163	16.036	3.578	-6.705	0.00	0.00	PROA
ATOM	2643	HG2	PRO	P	163	17.533	2.831	-5.793	0.00	0.00	PROA
ATOM	2644	C	PRO	P	163	15.066	1.183	-2.986	0.00	0.00	PROA
ATOM	2645	O	PRO	P	163	15.317	1.487	-1.844	0.00	0.00	PROA
ATOM	2646	N	ALA	P	164	13.947	0.601	-3.380	0.00	0.00	PROA
ATOM	2647	HN	ALA	P	164	13.797	0.624	-4.365	0.00	0.00	PROA
ATOM	2648	CA	ALA	P	164	12.987	0.018	-2.348	0.00	0.00	PROA
ATOM	2649	HA	ALA	P	164	12.744	0.772	-1.614	0.00	0.00	PROA
ATOM	2650	CB	ALA	P	164	11.713	-0.482	-3.191	0.00	0.00	PROA
ATOM	2651	HB1	ALA	P	164	11.964	-1.100	-4.080	0.00	0.00	PROA
ATOM	2652	HB2	ALA	P	164	11.168	0.410	-3.568	0.00	0.00	PROA
ATOM	2653	HB3	ALA	P	164	11.048	-1.118	-2.568	0.00	0.00	PROA
ATOM	2654	C	ALA	P	164	13.671	-1.133	-1.524	0.00	0.00	PROA
ATOM	2655	O	ALA	P	164	13.559	-1.162	-0.277	0.00	0.00	PROA
ATOM	2656	N	LEU	P	165	14.560	-2.000	-2.096	0.00	0.00	PROA
ATOM	2657	HN	LEU	P	165	14.662	-1.927	-3.085	0.00	0.00	PROA
ATOM	2658	CA	LEU	P	165	15.464	-2.936	-1.337	0.00	0.00	PROA
ATOM	2659	HA	LEU	P	165	14.942	-3.707	-0.790	0.00	0.00	PROA
ATOM	2660	CB	LEU	P	165	16.253	-3.808	-2.385	0.00	0.00	PROA
ATOM	2661	HB1	LEU	P	165	17.167	-4.241	-1.925	0.00	0.00	PROA
ATOM	2662	HB2	LEU	P	165	16.620	-3.273	-3.288	0.00	0.00	PROA
ATOM	2663	CG	LEU	P	165	15.447	-5.028	-2.930	0.00	0.00	PROA
ATOM	2664	HG	LEU	P	165	14.448	-4.641	-3.224	0.00	0.00	PROA
ATOM	2665	CD1	LEU	P	165	16.026	-5.564	-4.240	0.00	0.00	PROA
ATOM	2666	HD11	LEU	P	165	16.933	-6.132	-3.938	0.00	0.00	PROA
ATOM	2667	HD12	LEU	P	165	16.311	-4.808	-5.003	0.00	0.00	PROA
ATOM	2668	HD13	LEU	P	165	15.234	-6.239	-4.628	0.00	0.00	PROA
ATOM	2669	CD2	LEU	P	165	15.312	-6.119	-1.870	0.00	0.00	PROA
ATOM	2670	HD21	LEU	P	165	14.726	-5.734	-1.009	0.00	0.00	PROA
ATOM	2671	HD22	LEU	P	165	16.360	-6.204	-1.509	0.00	0.00	PROA
ATOM	2672	HD23	LEU	P	165	14.972	-7.097	-2.273	0.00	0.00	PROA
ATOM	2673	C	LEU	P	165	16.439	-2.300	-0.365	0.00	0.00	PROA
ATOM	2674	O	LEU	P	165	16.515	-2.717	0.775	0.00	0.00	PROA
ATOM	2675	N	LEU	P	166	17.081	-1.249	-0.745	0.00	0.00	PROA
ATOM	2676	HN	LEU	P	166	16.888	-0.778	-1.603	0.00	0.00	PROA
ATOM	2677	CA	LEU	P	166	17.800	-0.455	0.213	0.00	0.00	PROA
ATOM	2678	HA	LEU	P	166	18.611	-1.016	0.653	0.00	0.00	PROA
ATOM	2679	CB	LEU	P	166	18.443	0.856	-0.419	0.00	0.00	PROA
ATOM	2680	HB1	LEU	P	166	18.967	1.415	0.385	0.00	0.00	PROA

ATOM 2681 HB2 LEU P 166 17.628 1.490 -0.828 0.00 0.00 PROA
ATOM 2682 CG LEU P 166 19.408 0.476 -1.501 0.00 0.00 PROA
ATOM 2683 HG LEU P 166 18.867 0.019 -2.357 0.00 0.00 PROA
ATOM 2684 CD1 LEU P 166 20.179 1.707 -1.970 0.00 0.00 PROA
ATOM 2685 HD11 LEU P 166 19.446 2.376 -2.470 0.00 0.00 PROA
ATOM 2686 HD12 LEU P 166 20.876 1.278 -2.722 0.00 0.00 PROA
ATOM 2687 HD13 LEU P 166 20.864 2.271 -1.301 0.00 0.00 PROA
ATOM 2688 CD2 LEU P 166 20.482 -0.504 -1.077 0.00 0.00 PROA
ATOM 2689 HD21 LEU P 166 19.981 -1.489 -0.960 0.00 0.00 PROA
ATOM 2690 HD22 LEU P 166 20.897 -0.157 -0.107 0.00 0.00 PROA
ATOM 2691 HD23 LEU P 166 21.343 -0.689 -1.755 0.00 0.00 PROA
ATOM 2692 C LEU P 166 17.112 0.064 1.492 0.00 0.00 PROA
ATOM 2693 O LEU P 166 17.671 0.101 2.568 0.00 0.00 PROA
ATOM 2694 N LEU P 167 15.820 0.469 1.343 0.00 0.00 PROA
ATOM 2695 HN LEU P 167 15.283 0.527 0.506 0.00 0.00 PROA
ATOM 2696 CA LEU P 167 15.114 0.925 2.535 0.00 0.00 PROA
ATOM 2697 HA LEU P 167 15.728 1.495 3.216 0.00 0.00 PROA
ATOM 2698 CB LEU P 167 14.035 1.882 2.100 0.00 0.00 PROA
ATOM 2699 HB1 LEU P 167 13.259 2.155 2.847 0.00 0.00 PROA
ATOM 2700 HB2 LEU P 167 13.483 1.311 1.324 0.00 0.00 PROA
ATOM 2701 CG LEU P 167 14.474 3.210 1.333 0.00 0.00 PROA
ATOM 2702 HG LEU P 167 15.151 2.882 0.515 0.00 0.00 PROA
ATOM 2703 CD1 LEU P 167 13.246 3.954 0.753 0.00 0.00 PROA
ATOM 2704 HD11 LEU P 167 12.806 3.244 0.021 0.00 0.00 PROA
ATOM 2705 HD12 LEU P 167 13.384 4.978 0.346 0.00 0.00 PROA
ATOM 2706 HD13 LEU P 167 12.603 4.168 1.634 0.00 0.00 PROA
ATOM 2707 CD2 LEU P 167 15.154 4.166 2.337 0.00 0.00 PROA
ATOM 2708 HD21 LEU P 167 14.378 4.484 3.065 0.00 0.00 PROA
ATOM 2709 HD22 LEU P 167 15.340 5.176 1.913 0.00 0.00 PROA
ATOM 2710 HD23 LEU P 167 16.049 3.715 2.817 0.00 0.00 PROA
ATOM 2711 C LEU P 167 14.441 -0.270 3.244 0.00 0.00 PROA
ATOM 2712 O LEU P 167 13.906 -0.080 4.312 0.00 0.00 PROA
ATOM 2713 N THR P 168 14.500 -1.542 2.722 0.00 0.00 PROA
ATOM 2714 HN THR P 168 14.799 -1.705 1.785 0.00 0.00 PROA
ATOM 2715 CA THR P 168 13.982 -2.779 3.440 0.00 0.00 PROA
ATOM 2716 HA THR P 168 13.334 -2.366 4.199 0.00 0.00 PROA
ATOM 2717 CB THR P 168 13.235 -3.777 2.594 0.00 0.00 PROA
ATOM 2718 HB THR P 168 13.282 -4.797 3.031 0.00 0.00 PROA
ATOM 2719 OG1 THR P 168 13.708 -3.835 1.292 0.00 0.00 PROA
ATOM 2720 HG1 THR P 168 13.230 -3.122 0.862 0.00 0.00 PROA
ATOM 2721 CG2 THR P 168 11.727 -3.406 2.620 0.00 0.00 PROA
ATOM 2722 HG21 THR P 168 11.475 -2.612 1.885 0.00 0.00 PROA
ATOM 2723 HG22 THR P 168 11.409 -3.040 3.619 0.00 0.00 PROA
ATOM 2724 HG23 THR P 168 11.057 -4.258 2.372 0.00 0.00 PROA
ATOM 2725 C THR P 168 15.009 -3.638 4.208 0.00 0.00 PROA
ATOM 2726 O THR P 168 14.666 -4.490 5.012 0.00 0.00 PROA
ATOM 2727 N ILE P 169 16.327 -3.381 4.034 0.00 0.00 PROA
ATOM 2728 HN ILE P 169 16.723 -2.845 3.293 0.00 0.00 PROA
ATOM 2729 CA ILE P 169 17.307 -3.903 4.934 0.00 0.00 PROA
ATOM 2730 HA ILE P 169 17.200 -4.978 4.942 0.00 0.00 PROA
ATOM 2731 CB ILE P 169 18.774 -3.749 4.576 0.00 0.00 PROA
ATOM 2732 HB ILE P 169 19.136 -2.721 4.793 0.00 0.00 PROA
ATOM 2733 CG2 ILE P 169 19.686 -4.608 5.417 0.00 0.00 PROA
ATOM 2734 HG21 ILE P 169 19.620 -4.352 6.497 0.00 0.00 PROA
ATOM 2735 HG22 ILE P 169 20.763 -4.404 5.239 0.00 0.00 PROA
ATOM 2736 HG23 ILE P 169 19.471 -5.665 5.152 0.00 0.00 PROA
ATOM 2737 CG1 ILE P 169 19.096 -3.986 3.095 0.00 0.00 PROA
ATOM 2738 HG11 ILE P 169 18.163 -3.960 2.493 0.00 0.00 PROA
ATOM 2739 HG12 ILE P 169 19.410 -5.027 2.867 0.00 0.00 PROA
ATOM 2740 CD ILE P 169 20.061 -2.958 2.466 0.00 0.00 PROA
ATOM 2741 HD1 ILE P 169 21.016 -3.010 3.030 0.00 0.00 PROA

ATOM	2742	HD2	ILE	P	169	19.572	-1.968	2.586	0.00	0.00	PROA
ATOM	2743	HD3	ILE	P	169	20.251	-3.215	1.401	0.00	0.00	PROA
ATOM	2744	C	ILE	P	169	17.083	-3.539	6.444	0.00	0.00	PROA
ATOM	2745	O	ILE	P	169	17.162	-4.493	7.217	0.00	0.00	PROA
ATOM	2746	N	PRO	P	170	16.815	-2.363	6.915	0.00	0.00	PROA
ATOM	2747	CD	PRO	P	170	16.966	-1.168	6.175	0.00	0.00	PROA
ATOM	2748	HD1	PRO	P	170	16.085	-1.016	5.515	0.00	0.00	PROA
ATOM	2749	HD2	PRO	P	170	17.911	-1.064	5.600	0.00	0.00	PROA
ATOM	2750	CA	PRO	P	170	16.196	-2.128	8.225	0.00	0.00	PROA
ATOM	2751	HA	PRO	P	170	17.022	-2.269	8.906	0.00	0.00	PROA
ATOM	2752	CB	PRO	P	170	15.808	-0.655	8.238	0.00	0.00	PROA
ATOM	2753	HB1	PRO	P	170	15.901	-0.226	9.259	0.00	0.00	PROA
ATOM	2754	HB2	PRO	P	170	14.788	-0.523	7.819	0.00	0.00	PROA
ATOM	2755	CG	PRO	P	170	16.906	-0.127	7.309	0.00	0.00	PROA
ATOM	2756	HG1	PRO	P	170	16.555	0.875	6.980	0.00	0.00	PROA
ATOM	2757	HG2	PRO	P	170	17.899	-0.068	7.803	0.00	0.00	PROA
ATOM	2758	C	PRO	P	170	15.141	-3.196	8.665	0.00	0.00	PROA
ATOM	2759	O	PRO	P	170	15.059	-3.592	9.810	0.00	0.00	PROA
ATOM	2760	N	ASP	P	171	14.174	-3.598	7.818	0.00	0.00	PROA
ATOM	2761	HN	ASP	P	171	14.229	-3.297	6.869	0.00	0.00	PROA
ATOM	2762	CA	ASP	P	171	13.162	-4.586	8.121	0.00	0.00	PROA
ATOM	2763	HA	ASP	P	171	12.764	-4.402	9.108	0.00	0.00	PROA
ATOM	2764	CB	ASP	P	171	12.068	-4.655	6.998	0.00	0.00	PROA
ATOM	2765	HB1	ASP	P	171	11.274	-5.381	7.273	0.00	0.00	PROA
ATOM	2766	HB2	ASP	P	171	12.388	-4.929	5.970	0.00	0.00	PROA
ATOM	2767	CG	ASP	P	171	11.321	-3.293	6.963	0.00	0.00	PROA
ATOM	2768	OD1	ASP	P	171	10.998	-2.811	8.100	0.00	0.00	PROA
ATOM	2769	OD2	ASP	P	171	10.923	-2.874	5.861	0.00	0.00	PROA
ATOM	2770	C	ASP	P	171	13.768	-5.991	8.177	0.00	0.00	PROA
ATOM	2771	O	ASP	P	171	13.416	-6.831	9.095	0.00	0.00	PROA
ATOM	2772	N	PHE	P	172	14.759	-6.321	7.381	0.00	0.00	PROA
ATOM	2773	HN	PHE	P	172	15.072	-5.851	6.560	0.00	0.00	PROA
ATOM	2774	CA	PHE	P	172	15.519	-7.574	7.501	0.00	0.00	PROA
ATOM	2775	HA	PHE	P	172	14.753	-8.311	7.691	0.00	0.00	PROA
ATOM	2776	CB	PHE	P	172	16.491	-7.722	6.279	0.00	0.00	PROA
ATOM	2777	HB1	PHE	P	172	17.297	-6.959	6.310	0.00	0.00	PROA
ATOM	2778	HB2	PHE	P	172	15.933	-7.478	5.350	0.00	0.00	PROA
ATOM	2779	CG	PHE	P	172	16.953	-9.070	6.018	0.00	0.00	PROA
ATOM	2780	CD1	PHE	P	172	16.332	-10.260	6.502	0.00	0.00	PROA
ATOM	2781	HD1	PHE	P	172	15.537	-10.343	7.228	0.00	0.00	PROA
ATOM	2782	CE1	PHE	P	172	16.638	-11.481	5.897	0.00	0.00	PROA
ATOM	2783	HE1	PHE	P	172	16.149	-12.367	6.273	0.00	0.00	PROA
ATOM	2784	CZ	PHE	P	172	17.477	-11.540	4.780	0.00	0.00	PROA
ATOM	2785	HZ	PHE	P	172	17.937	-12.390	4.297	0.00	0.00	PROA
ATOM	2786	CD2	PHE	P	172	17.849	-9.205	4.885	0.00	0.00	PROA
ATOM	2787	HD2	PHE	P	172	18.387	-8.337	4.532	0.00	0.00	PROA
ATOM	2788	CE2	PHE	P	172	18.108	-10.417	4.374	0.00	0.00	PROA
ATOM	2789	HE2	PHE	P	172	18.804	-10.503	3.553	0.00	0.00	PROA
ATOM	2790	C	PHE	P	172	16.402	-7.665	8.794	0.00	0.00	PROA
ATOM	2791	O	PHE	P	172	16.394	-8.626	9.502	0.00	0.00	PROA
ATOM	2792	N	ILE	P	173	17.145	-6.559	9.116	0.00	0.00	PROA
ATOM	2793	HN	ILE	P	173	17.388	-5.974	8.346	0.00	0.00	PROA
ATOM	2794	CA	ILE	P	173	17.773	-6.382	10.437	0.00	0.00	PROA
ATOM	2795	HA	ILE	P	173	18.477	-7.190	10.569	0.00	0.00	PROA
ATOM	2796	CB	ILE	P	173	18.597	-5.119	10.321	0.00	0.00	PROA
ATOM	2797	HB	ILE	P	173	17.946	-4.286	9.981	0.00	0.00	PROA
ATOM	2798	CG2	ILE	P	173	19.107	-4.716	11.708	0.00	0.00	PROA
ATOM	2799	HG21	ILE	P	173	19.997	-5.374	11.803	0.00	0.00	PROA
ATOM	2800	HG22	ILE	P	173	18.448	-4.721	12.603	0.00	0.00	PROA
ATOM	2801	HG23	ILE	P	173	19.553	-3.699	11.668	0.00	0.00	PROA
ATOM	2802	CG1	ILE	P	173	19.731	-5.311	9.335	0.00	0.00	PROA

```
ATOM   2803 HG11 ILE P 173      19.351  -5.590   8.329  0.00  0.00      PROA
ATOM   2804 HG12 ILE P 173      20.489  -5.999   9.766  0.00  0.00      PROA
ATOM   2805  CD  ILE P 173      20.402  -3.940   9.142  0.00  0.00      PROA
ATOM   2806  HD1 ILE P 173      19.603  -3.168   9.181  0.00  0.00      PROA
ATOM   2807  HD2 ILE P 173      21.016  -3.903   8.217  0.00  0.00      PROA
ATOM   2808  HD3 ILE P 173      21.036  -3.701  10.022  0.00  0.00      PROA
ATOM   2809  C   ILE P 173      16.874  -6.407  11.649  0.00  0.00      PROA
ATOM   2810  O   ILE P 173      17.190  -7.037  12.682  0.00  0.00      PROA
ATOM   2811  N   PHE P 174      15.731  -5.662  11.658  0.00  0.00      PROA
ATOM   2812  HN  PHE P 174      15.391  -5.157  10.868  0.00  0.00      PROA
ATOM   2813  CA  PHE P 174      14.913  -5.478  12.841  0.00  0.00      PROA
ATOM   2814  HA  PHE P 174      15.532  -5.574  13.721  0.00  0.00      PROA
ATOM   2815  CB  PHE P 174      14.272  -4.031  12.804  0.00  0.00      PROA
ATOM   2816  HB1 PHE P 174      13.601  -3.919  13.682  0.00  0.00      PROA
ATOM   2817  HB2 PHE P 174      13.562  -3.898  11.959  0.00  0.00      PROA
ATOM   2818  CG  PHE P 174      15.259  -2.924  12.919  0.00  0.00      PROA
ATOM   2819  CD1 PHE P 174      16.461  -3.019  13.641  0.00  0.00      PROA
ATOM   2820  HD1 PHE P 174      16.657  -3.870  14.276  0.00  0.00      PROA
ATOM   2821  CE1 PHE P 174      17.482  -1.966  13.586  0.00  0.00      PROA
ATOM   2822  HE1 PHE P 174      18.396  -2.069  14.153  0.00  0.00      PROA
ATOM   2823  CZ  PHE P 174      17.169  -0.841  12.826  0.00  0.00      PROA
ATOM   2824  HZ  PHE P 174      17.965  -0.151  12.589  0.00  0.00      PROA
ATOM   2825  CD2 PHE P 174      15.013  -1.758  12.170  0.00  0.00      PROA
ATOM   2826  HD2 PHE P 174      14.104  -1.678  11.594  0.00  0.00      PROA
ATOM   2827  CE2 PHE P 174      15.994  -0.751  12.093  0.00  0.00      PROA
ATOM   2828  HE2 PHE P 174      15.704   0.175  11.619  0.00  0.00      PROA
ATOM   2829  C   PHE P 174      13.931  -6.613  13.163  0.00  0.00      PROA
ATOM   2830  O   PHE P 174      13.602  -6.872  14.334  0.00  0.00      PROA
ATOM   2831  N   ALA P 175      13.328  -7.222  12.124  0.00  0.00      PROA
ATOM   2832  HN  ALA P 175      13.695  -7.016  11.220  0.00  0.00      PROA
ATOM   2833  CA  ALA P 175      12.146  -8.108  12.193  0.00  0.00      PROA
ATOM   2834  HA  ALA P 175      11.475  -7.599  12.869  0.00  0.00      PROA
ATOM   2835  CB  ALA P 175      11.502  -8.404  10.833  0.00  0.00      PROA
ATOM   2836  HB1 ALA P 175      11.120  -7.478  10.353  0.00  0.00      PROA
ATOM   2837  HB2 ALA P 175      10.673  -9.133  10.954  0.00  0.00      PROA
ATOM   2838  HB3 ALA P 175      12.385  -8.717  10.235  0.00  0.00      PROA
ATOM   2839  C   ALA P 175      12.540  -9.389  12.813  0.00  0.00      PROA
ATOM   2840  O   ALA P 175      13.520 -10.070  12.504  0.00  0.00      PROA
ATOM   2841  N   ASN P 176      11.824  -9.708  13.878  0.00  0.00      PROA
ATOM   2842  HN  ASN P 176      11.076  -9.130  14.193  0.00  0.00      PROA
ATOM   2843  CA  ASN P 176      12.350 -10.693  14.737  0.00  0.00      PROA
ATOM   2844  HA  ASN P 176      12.605 -11.593  14.196  0.00  0.00      PROA
ATOM   2845  CB  ASN P 176      13.594 -10.277  15.565  0.00  0.00      PROA
ATOM   2846  HB1 ASN P 176      13.489  -9.190  15.769  0.00  0.00      PROA
ATOM   2847  HB2 ASN P 176      14.417 -10.334  14.821  0.00  0.00      PROA
ATOM   2848  CG  ASN P 176      14.148 -11.008  16.873  0.00  0.00      PROA
ATOM   2849  OD1 ASN P 176      14.015 -10.511  17.971  0.00  0.00      PROA
ATOM   2850  ND2 ASN P 176      14.759 -12.188  16.648  0.00  0.00      PROA
ATOM   2851 HD21 ASN P 176      15.099 -12.738  17.411  0.00  0.00      PROA
ATOM   2852 HD22 ASN P 176      14.891 -12.494  15.705  0.00  0.00      PROA
ATOM   2853  C   ASN P 176      11.199 -11.150  15.648  0.00  0.00      PROA
ATOM   2854  O   ASN P 176      10.147 -10.543  15.753  0.00  0.00      PROA
ATOM   2855  N   VAL P 177      11.380 -12.317  16.232  0.00  0.00      PROA
ATOM   2856  HN  VAL P 177      12.239 -12.809  16.107  0.00  0.00      PROA
ATOM   2857  CA  VAL P 177      10.399 -13.071  16.968  0.00  0.00      PROA
ATOM   2858  HA  VAL P 177       9.449 -12.571  16.849  0.00  0.00      PROA
ATOM   2859  CB  VAL P 177      10.141 -14.408  16.378  0.00  0.00      PROA
ATOM   2860  HB  VAL P 177      11.118 -14.927  16.279  0.00  0.00      PROA
ATOM   2861  CG1 VAL P 177       9.227 -15.301  17.326  0.00  0.00      PROA
ATOM   2862 HG11 VAL P 177       8.185 -14.928  17.421  0.00  0.00      PROA
ATOM   2863 HG12 VAL P 177       9.654 -15.531  18.325  0.00  0.00      PROA
```

```
ATOM 2864 HG13 VAL P 177 9.126 -16.269 16.789 0.00 0.00 PROA
ATOM 2865 CG2 VAL P 177 9.428 -14.243 15.060 0.00 0.00 PROA
ATOM 2866 HG21 VAL P 177 9.261 -15.264 14.654 0.00 0.00 PROA
ATOM 2867 HG22 VAL P 177 10.120 -13.669 14.407 0.00 0.00 PROA
ATOM 2868 HG23 VAL P 177 8.450 -13.732 15.184 0.00 0.00 PROA
ATOM 2869 C VAL P 177 10.791 -13.181 18.442 0.00 0.00 PROA
ATOM 2870 O VAL P 177 11.866 -13.540 18.756 0.00 0.00 PROA
ATOM 2871 N SER P 178 9.818 -12.974 19.357 0.00 0.00 PROA
ATOM 2872 HN SER P 178 8.866 -12.766 19.145 0.00 0.00 PROA
ATOM 2873 CA SER P 178 10.149 -13.226 20.748 0.00 0.00 PROA
ATOM 2874 HA SER P 178 10.736 -14.125 20.866 0.00 0.00 PROA
ATOM 2875 CB SER P 178 10.930 -12.001 21.281 0.00 0.00 PROA
ATOM 2876 HB1 SER P 178 10.263 -11.118 21.382 0.00 0.00 PROA
ATOM 2877 HB2 SER P 178 11.696 -11.780 20.507 0.00 0.00 PROA
ATOM 2878 OG SER P 178 11.563 -12.366 22.498 0.00 0.00 PROA
ATOM 2879 HG1 SER P 178 12.471 -12.666 22.423 0.00 0.00 PROA
ATOM 2880 C SER P 178 8.837 -13.338 21.559 0.00 0.00 PROA
ATOM 2881 O SER P 178 7.798 -13.526 20.962 0.00 0.00 PROA
ATOM 2882 N GLU P 179 8.826 -13.410 22.845 0.00 0.00 PROA
ATOM 2883 HN GLU P 179 9.711 -13.339 23.299 0.00 0.00 PROA
ATOM 2884 CA GLU P 179 7.645 -13.670 23.673 0.00 0.00 PROA
ATOM 2885 HA GLU P 179 6.919 -14.249 23.123 0.00 0.00 PROA
ATOM 2886 CB GLU P 179 8.034 -14.385 25.004 0.00 0.00 PROA
ATOM 2887 HB1 GLU P 179 8.730 -13.732 25.573 0.00 0.00 PROA
ATOM 2888 HB2 GLU P 179 8.676 -15.261 24.774 0.00 0.00 PROA
ATOM 2889 CG GLU P 179 6.827 -14.764 25.826 0.00 0.00 PROA
ATOM 2890 HG1 GLU P 179 6.154 -15.400 25.211 0.00 0.00 PROA
ATOM 2891 HG2 GLU P 179 6.260 -13.893 26.218 0.00 0.00 PROA
ATOM 2892 CD GLU P 179 7.291 -15.471 27.088 0.00 0.00 PROA
ATOM 2893 OE1 GLU P 179 7.021 -16.681 27.204 0.00 0.00 PROA
ATOM 2894 OE2 GLU P 179 8.044 -14.833 27.877 0.00 0.00 PROA
ATOM 2895 C GLU P 179 7.080 -12.337 24.083 0.00 0.00 PROA
ATOM 2896 O GLU P 179 7.765 -11.392 24.460 0.00 0.00 PROA
ATOM 2897 N ALA P 180 5.763 -12.256 24.063 0.00 0.00 PROA
ATOM 2898 HN ALA P 180 5.241 -13.097 23.943 0.00 0.00 PROA
ATOM 2899 CA ALA P 180 5.030 -11.167 24.678 0.00 0.00 PROA
ATOM 2900 HA ALA P 180 5.674 -10.708 25.414 0.00 0.00 PROA
ATOM 2901 CB ALA P 180 4.454 -10.120 23.654 0.00 0.00 PROA
ATOM 2902 HB1 ALA P 180 3.923 -9.302 24.187 0.00 0.00 PROA
ATOM 2903 HB2 ALA P 180 3.750 -10.639 22.969 0.00 0.00 PROA
ATOM 2904 HB3 ALA P 180 5.285 -9.664 23.073 0.00 0.00 PROA
ATOM 2905 C ALA P 180 3.805 -11.773 25.351 0.00 0.00 PROA
ATOM 2906 O ALA P 180 3.056 -12.593 24.753 0.00 0.00 PROA
ATOM 2907 N ASP P 181 3.552 -11.485 26.642 0.00 0.00 PROA
ATOM 2908 HN ASP P 181 4.122 -10.734 26.968 0.00 0.00 PROA
ATOM 2909 CA ASP P 181 2.572 -12.086 27.515 0.00 0.00 PROA
ATOM 2910 HA ASP P 181 2.987 -12.027 28.510 0.00 0.00 PROA
ATOM 2911 CB ASP P 181 1.238 -11.301 27.391 0.00 0.00 PROA
ATOM 2912 HB1 ASP P 181 0.473 -11.820 28.008 0.00 0.00 PROA
ATOM 2913 HB2 ASP P 181 0.902 -11.215 26.336 0.00 0.00 PROA
ATOM 2914 CG ASP P 181 1.245 -9.877 27.978 0.00 0.00 PROA
ATOM 2915 OD1 ASP P 181 0.083 -9.354 28.097 0.00 0.00 PROA
ATOM 2916 OD2 ASP P 181 2.290 -9.329 28.297 0.00 0.00 PROA
ATOM 2917 C ASP P 181 2.380 -13.610 27.440 0.00 0.00 PROA
ATOM 2918 O ASP P 181 1.237 -14.147 27.424 0.00 0.00 PROA
ATOM 2919 N ASP P 182 3.505 -14.466 27.393 0.00 0.00 PROA
ATOM 2920 HN ASP P 182 4.424 -14.096 27.280 0.00 0.00 PROA
ATOM 2921 CA ASP P 182 3.468 -15.905 27.471 0.00 0.00 PROA
ATOM 2922 HA ASP P 182 4.493 -16.143 27.712 0.00 0.00 PROA
ATOM 2923 CB ASP P 182 2.717 -16.568 28.644 0.00 0.00 PROA
ATOM 2924 HB1 ASP P 182 2.967 -17.641 28.784 0.00 0.00 PROA
```

ATOM	2925	HB2	ASP	P	182	1.619	-16.436	28.533	0.00	0.00	PROA
ATOM	2926	CG	ASP	P	182	3.246	-15.917	29.933	0.00	0.00	PROA
ATOM	2927	OD1	ASP	P	182	2.397	-15.729	30.855	0.00	0.00	PROA
ATOM	2928	OD2	ASP	P	182	4.433	-15.705	30.137	0.00	0.00	PROA
ATOM	2929	C	ASP	P	182	3.176	-16.649	26.130	0.00	0.00	PROA
ATOM	2930	O	ASP	P	182	2.869	-17.849	26.082	0.00	0.00	PROA
ATOM	2931	N	ARG	P	183	3.327	-15.985	24.998	0.00	0.00	PROA
ATOM	2932	HN	ARG	P	183	3.599	-15.026	24.993	0.00	0.00	PROA
ATOM	2933	CA	ARG	P	183	3.217	-16.534	23.635	0.00	0.00	PROA
ATOM	2934	HA	ARG	P	183	3.624	-17.532	23.582	0.00	0.00	PROA
ATOM	2935	CB	ARG	P	183	1.747	-16.653	23.159	0.00	0.00	PROA
ATOM	2936	HB1	ARG	P	183	1.190	-17.216	23.938	0.00	0.00	PROA
ATOM	2937	HB2	ARG	P	183	1.701	-17.383	22.324	0.00	0.00	PROA
ATOM	2938	CG	ARG	P	183	0.952	-15.346	22.886	0.00	0.00	PROA
ATOM	2939	HG1	ARG	P	183	1.110	-14.539	23.632	0.00	0.00	PROA
ATOM	2940	HG2	ARG	P	183	-0.094	-15.719	22.838	0.00	0.00	PROA
ATOM	2941	CD	ARG	P	183	1.301	-14.703	21.560	0.00	0.00	PROA
ATOM	2942	HD1	ARG	P	183	1.642	-15.447	20.808	0.00	0.00	PROA
ATOM	2943	HD2	ARG	P	183	1.948	-13.814	21.717	0.00	0.00	PROA
ATOM	2944	NE	ARG	P	183	0.072	-14.101	21.078	0.00	0.00	PROA
ATOM	2945	HE	ARG	P	183	-0.704	-14.108	21.708	0.00	0.00	PROA
ATOM	2946	CZ	ARG	P	183	-0.206	-13.886	19.808	0.00	0.00	PROA
ATOM	2947	NH1	ARG	P	183	0.655	-13.983	18.789	0.00	0.00	PROA
ATOM	2948	HH11	ARG	P	183	1.589	-14.274	18.998	0.00	0.00	PROA
ATOM	2949	HH12	ARG	P	183	0.552	-13.317	18.050	0.00	0.00	PROA
ATOM	2950	NH2	ARG	P	183	-1.391	-13.349	19.545	0.00	0.00	PROA
ATOM	2951	HH21	ARG	P	183	-2.074	-13.439	20.270	0.00	0.00	PROA
ATOM	2952	HH22	ARG	P	183	-1.642	-13.257	18.581	0.00	0.00	PROA
ATOM	2953	C	ARG	P	183	4.144	-15.664	22.758	0.00	0.00	PROA
ATOM	2954	O	ARG	P	183	4.596	-14.590	23.163	0.00	0.00	PROA
ATOM	2955	N	TYR	P	184	4.533	-16.184	21.565	0.00	0.00	PROA
ATOM	2956	HN	TYR	P	184	4.056	-17.004	21.260	0.00	0.00	PROA
ATOM	2957	CA	TYR	P	184	5.381	-15.548	20.596	0.00	0.00	PROA
ATOM	2958	HA	TYR	P	184	6.131	-14.933	21.071	0.00	0.00	PROA
ATOM	2959	CB	TYR	P	184	6.062	-16.476	19.568	0.00	0.00	PROA
ATOM	2960	HB1	TYR	P	184	6.630	-15.872	18.829	0.00	0.00	PROA
ATOM	2961	HB2	TYR	P	184	5.224	-17.048	19.116	0.00	0.00	PROA
ATOM	2962	CG	TYR	P	184	7.069	-17.492	20.239	0.00	0.00	PROA
ATOM	2963	CD1	TYR	P	184	7.650	-17.325	21.537	0.00	0.00	PROA
ATOM	2964	HD1	TYR	P	184	7.376	-16.491	22.166	0.00	0.00	PROA
ATOM	2965	CE1	TYR	P	184	8.570	-18.208	22.035	0.00	0.00	PROA
ATOM	2966	HE1	TYR	P	184	8.988	-18.076	23.022	0.00	0.00	PROA
ATOM	2967	CZ	TYR	P	184	9.019	-19.281	21.224	0.00	0.00	PROA
ATOM	2968	OH	TYR	P	184	10.000	-20.243	21.546	0.00	0.00	PROA
ATOM	2969	HH	TYR	P	184	10.632	-19.867	22.164	0.00	0.00	PROA
ATOM	2970	CD2	TYR	P	184	7.620	-18.446	19.397	0.00	0.00	PROA
ATOM	2971	HD2	TYR	P	184	7.272	-18.644	18.394	0.00	0.00	PROA
ATOM	2972	CE2	TYR	P	184	8.564	-19.356	19.865	0.00	0.00	PROA
ATOM	2973	HE2	TYR	P	184	8.967	-20.053	19.145	0.00	0.00	PROA
ATOM	2974	C	TYR	P	184	4.549	-14.507	19.741	0.00	0.00	PROA
ATOM	2975	O	TYR	P	184	3.414	-14.673	19.380	0.00	0.00	PROA
ATOM	2976	N	ILE	P	185	5.291	-13.461	19.331	0.00	0.00	PROA
ATOM	2977	HN	ILE	P	185	6.194	-13.257	19.702	0.00	0.00	PROA
ATOM	2978	CA	ILE	P	185	4.875	-12.400	18.419	0.00	0.00	PROA
ATOM	2979	HA	ILE	P	185	4.024	-12.689	17.820	0.00	0.00	PROA
ATOM	2980	CB	ILE	P	185	4.483	-11.095	19.137	0.00	0.00	PROA
ATOM	2981	HB	ILE	P	185	4.293	-10.309	18.376	0.00	0.00	PROA
ATOM	2982	CG2	ILE	P	185	3.230	-11.408	19.972	0.00	0.00	PROA
ATOM	2983	HG21	ILE	P	185	2.762	-10.527	20.461	0.00	0.00	PROA
ATOM	2984	HG22	ILE	P	185	3.569	-12.076	20.793	0.00	0.00	PROA
ATOM	2985	HG23	ILE	P	185	2.378	-11.866	19.426	0.00	0.00	PROA

ATOM	2986	CG1	ILE	P	185	5.475	-10.455	20.143	0.00	0.00	PROA
ATOM	2987	HG11	ILE	P	185	5.606	-11.303	20.849	0.00	0.00	PROA
ATOM	2988	HG12	ILE	P	185	4.945	-9.686	20.744	0.00	0.00	PROA
ATOM	2989	CD	ILE	P	185	6.741	-9.751	19.603	0.00	0.00	PROA
ATOM	2990	HD1	ILE	P	185	7.576	-10.479	19.519	0.00	0.00	PROA
ATOM	2991	HD2	ILE	P	185	7.035	-8.939	20.303	0.00	0.00	PROA
ATOM	2992	HD3	ILE	P	185	6.469	-9.230	18.661	0.00	0.00	PROA
ATOM	2993	C	ILE	P	185	6.036	-12.121	17.507	0.00	0.00	PROA
ATOM	2994	O	ILE	P	185	7.179	-12.564	17.779	0.00	0.00	PROA
ATOM	2995	N	CYS	P	186	5.728	-11.507	16.388	0.00	0.00	PROA
ATOM	2996	HN	CYS	P	186	4.785	-11.237	16.211	0.00	0.00	PROA
ATOM	2997	CA	CYS	P	186	6.653	-11.212	15.314	0.00	0.00	PROA
ATOM	2998	HA	CYS	P	186	7.629	-11.630	15.513	0.00	0.00	PROA
ATOM	2999	CB	CYS	P	186	6.112	-11.876	13.982	0.00	0.00	PROA
ATOM	3000	HB1	CYS	P	186	5.021	-11.679	14.044	0.00	0.00	PROA
ATOM	3001	HB2	CYS	P	186	6.189	-12.977	14.111	0.00	0.00	PROA
ATOM	3002	SG	CYS	P	186	6.916	-11.361	12.432	0.00	0.00	PROA
ATOM	3003	C	CYS	P	186	6.683	-9.698	15.079	0.00	0.00	PROA
ATOM	3004	O	CYS	P	186	5.732	-9.167	14.639	0.00	0.00	PROA
ATOM	3005	N	ASP	P	187	7.723	-9.020	15.461	0.00	0.00	PROA
ATOM	3006	HN	ASP	P	187	8.509	-9.588	15.692	0.00	0.00	PROA
ATOM	3007	CA	ASP	P	187	7.743	-7.606	15.396	0.00	0.00	PROA
ATOM	3008	HA	ASP	P	187	7.153	-7.232	14.573	0.00	0.00	PROA
ATOM	3009	CB	ASP	P	187	6.968	-7.036	16.641	0.00	0.00	PROA
ATOM	3010	HB1	ASP	P	187	7.337	-7.487	17.587	0.00	0.00	PROA
ATOM	3011	HB2	ASP	P	187	5.924	-7.417	16.628	0.00	0.00	PROA
ATOM	3012	CG	ASP	P	187	6.881	-5.508	16.706	0.00	0.00	PROA
ATOM	3013	OD1	ASP	P	187	7.004	-4.945	15.604	0.00	0.00	PROA
ATOM	3014	OD2	ASP	P	187	6.361	-4.978	17.721	0.00	0.00	PROA
ATOM	3015	C	ASP	P	187	9.202	-7.150	15.145	0.00	0.00	PROA
ATOM	3016	O	ASP	P	187	10.132	-7.892	15.261	0.00	0.00	PROA
ATOM	3017	N	ARG	P	188	9.301	-5.911	14.662	0.00	0.00	PROA
ATOM	3018	HN	ARG	P	188	8.457	-5.381	14.691	0.00	0.00	PROA
ATOM	3019	CA	ARG	P	188	10.538	-5.255	14.463	0.00	0.00	PROA
ATOM	3020	HA	ARG	P	188	11.247	-5.974	14.080	0.00	0.00	PROA
ATOM	3021	CB	ARG	P	188	10.458	-4.144	13.414	0.00	0.00	PROA
ATOM	3022	HB1	ARG	P	188	11.309	-3.430	13.398	0.00	0.00	PROA
ATOM	3023	HB2	ARG	P	188	9.603	-3.458	13.592	0.00	0.00	PROA
ATOM	3024	CG	ARG	P	188	10.327	-4.802	12.030	0.00	0.00	PROA
ATOM	3025	HG1	ARG	P	188	9.542	-5.578	11.901	0.00	0.00	PROA
ATOM	3026	HG2	ARG	P	188	11.241	-5.359	11.730	0.00	0.00	PROA
ATOM	3027	CD	ARG	P	188	10.043	-3.754	10.915	0.00	0.00	PROA
ATOM	3028	HD1	ARG	P	188	10.118	-4.334	9.970	0.00	0.00	PROA
ATOM	3029	HD2	ARG	P	188	10.833	-2.976	10.841	0.00	0.00	PROA
ATOM	3030	NE	ARG	P	188	8.614	-3.258	11.110	0.00	0.00	PROA
ATOM	3031	HE	ARG	P	188	7.891	-3.759	11.586	0.00	0.00	PROA
ATOM	3032	CZ	ARG	P	188	8.143	-2.225	10.418	0.00	0.00	PROA
ATOM	3033	NH1	ARG	P	188	8.794	-1.565	9.506	0.00	0.00	PROA
ATOM	3034	HH11	ARG	P	188	9.423	-2.023	8.878	0.00	0.00	PROA
ATOM	3035	HH12	ARG	P	188	8.240	-0.867	9.052	0.00	0.00	PROA
ATOM	3036	NH2	ARG	P	188	6.850	-1.912	10.618	0.00	0.00	PROA
ATOM	3037	HH21	ARG	P	188	6.372	-2.481	11.287	0.00	0.00	PROA
ATOM	3038	HH22	ARG	P	188	6.627	-0.952	10.449	0.00	0.00	PROA
ATOM	3039	C	ARG	P	188	11.086	-4.747	15.786	0.00	0.00	PROA
ATOM	3040	O	ARG	P	188	10.435	-4.062	16.569	0.00	0.00	PROA
ATOM	3041	N	PHE	P	189	12.316	-5.190	16.128	0.00	0.00	PROA
ATOM	3042	HN	PHE	P	189	12.769	-5.739	15.430	0.00	0.00	PROA
ATOM	3043	CA	PHE	P	189	12.998	-4.843	17.365	0.00	0.00	PROA
ATOM	3044	HA	PHE	P	189	12.222	-4.418	17.985	0.00	0.00	PROA
ATOM	3045	CB	PHE	P	189	13.509	-6.233	18.031	0.00	0.00	PROA
ATOM	3046	HB1	PHE	P	189	14.202	-5.991	18.865	0.00	0.00	PROA

ATOM	3047	HB2	PHE	P	189	14.025	-6.797	17.225	0.00	0.00	PROA
ATOM	3048	CG	PHE	P	189	12.457	-7.033	18.713	0.00	0.00	PROA
ATOM	3049	CD1	PHE	P	189	11.882	-8.223	18.197	0.00	0.00	PROA
ATOM	3050	HD1	PHE	P	189	12.080	-8.723	17.261	0.00	0.00	PROA
ATOM	3051	CE1	PHE	P	189	10.902	-8.969	18.824	0.00	0.00	PROA
ATOM	3052	HE1	PHE	P	189	10.502	-9.850	18.343	0.00	0.00	PROA
ATOM	3053	CZ	PHE	P	189	10.548	-8.543	20.136	0.00	0.00	PROA
ATOM	3054	HZ	PHE	P	189	9.758	-9.017	20.699	0.00	0.00	PROA
ATOM	3055	CD2	PHE	P	189	11.984	-6.590	19.964	0.00	0.00	PROA
ATOM	3056	HD2	PHE	P	189	12.389	-5.703	20.429	0.00	0.00	PROA
ATOM	3057	CE2	PHE	P	189	10.959	-7.291	20.678	0.00	0.00	PROA
ATOM	3058	HE2	PHE	P	189	10.622	-6.956	21.648	0.00	0.00	PROA
ATOM	3059	C	PHE	P	189	14.170	-3.855	17.236	0.00	0.00	PROA
ATOM	3060	O	PHE	P	189	15.172	-4.138	16.605	0.00	0.00	PROA
ATOM	3061	N	TYR	P	190	14.086	-2.662	17.851	0.00	0.00	PROA
ATOM	3062	HN	TYR	P	190	13.405	-2.438	18.544	0.00	0.00	PROA
ATOM	3063	CA	TYR	P	190	14.911	-1.491	17.620	0.00	0.00	PROA
ATOM	3064	HA	TYR	P	190	15.453	-1.646	16.699	0.00	0.00	PROA
ATOM	3065	CB	TYR	P	190	14.018	-0.263	17.479	0.00	0.00	PROA
ATOM	3066	HB1	TYR	P	190	14.652	0.647	17.544	0.00	0.00	PROA
ATOM	3067	HB2	TYR	P	190	13.366	-0.307	18.377	0.00	0.00	PROA
ATOM	3068	CG	TYR	P	190	13.240	-0.270	16.207	0.00	0.00	PROA
ATOM	3069	CD1	TYR	P	190	13.748	0.289	15.077	0.00	0.00	PROA
ATOM	3070	HD1	TYR	P	190	14.650	0.879	15.144	0.00	0.00	PROA
ATOM	3071	CE1	TYR	P	190	12.935	0.273	13.944	0.00	0.00	PROA
ATOM	3072	HE1	TYR	P	190	13.338	0.706	13.041	0.00	0.00	PROA
ATOM	3073	CZ	TYR	P	190	11.715	-0.371	13.879	0.00	0.00	PROA
ATOM	3074	OH	TYR	P	190	11.005	-0.314	12.651	0.00	0.00	PROA
ATOM	3075	HH	TYR	P	190	11.598	0.028	11.977	0.00	0.00	PROA
ATOM	3076	CD2	TYR	P	190	12.014	-1.009	16.214	0.00	0.00	PROA
ATOM	3077	HD2	TYR	P	190	11.596	-1.421	17.121	0.00	0.00	PROA
ATOM	3078	CE2	TYR	P	190	11.258	-1.052	15.057	0.00	0.00	PROA
ATOM	3079	HE2	TYR	P	190	10.303	-1.549	15.141	0.00	0.00	PROA
ATOM	3080	C	TYR	P	190	15.938	-1.248	18.771	0.00	0.00	PROA
ATOM	3081	O	TYR	P	190	15.583	-1.387	19.953	0.00	0.00	PROA
ATOM	3082	N	PRO	P	191	17.200	-0.959	18.513	0.00	0.00	PROA
ATOM	3083	CD	PRO	P	191	17.847	-0.766	17.210	0.00	0.00	PROA
ATOM	3084	HD1	PRO	P	191	17.249	-0.226	16.445	0.00	0.00	PROA
ATOM	3085	HD2	PRO	P	191	18.082	-1.769	16.792	0.00	0.00	PROA
ATOM	3086	CA	PRO	P	191	18.039	-0.440	19.660	0.00	0.00	PROA
ATOM	3087	HA	PRO	P	191	18.138	-1.199	20.421	0.00	0.00	PROA
ATOM	3088	CB	PRO	P	191	19.464	-0.382	18.963	0.00	0.00	PROA
ATOM	3089	HB1	PRO	P	191	20.024	-1.334	19.080	0.00	0.00	PROA
ATOM	3090	HB2	PRO	P	191	20.151	0.378	19.392	0.00	0.00	PROA
ATOM	3091	CG	PRO	P	191	19.179	-0.089	17.531	0.00	0.00	PROA
ATOM	3092	HG1	PRO	P	191	19.061	1.013	17.449	0.00	0.00	PROA
ATOM	3093	HG2	PRO	P	191	20.049	-0.417	16.922	0.00	0.00	PROA
ATOM	3094	C	PRO	P	191	17.596	0.871	20.229	0.00	0.00	PROA
ATOM	3095	O	PRO	P	191	17.813	0.952	21.447	0.00	0.00	PROA
ATOM	3096	N	ASN	P	192	17.019	1.804	19.425	0.00	0.00	PROA
ATOM	3097	HN	ASN	P	192	16.782	1.586	18.482	0.00	0.00	PROA
ATOM	3098	CA	ASN	P	192	16.393	3.043	19.907	0.00	0.00	PROA
ATOM	3099	HA	ASN	P	192	15.944	2.791	20.856	0.00	0.00	PROA
ATOM	3100	CB	ASN	P	192	17.609	3.999	20.371	0.00	0.00	PROA
ATOM	3101	HB1	ASN	P	192	18.427	3.421	20.852	0.00	0.00	PROA
ATOM	3102	HB2	ASN	P	192	17.200	4.787	21.038	0.00	0.00	PROA
ATOM	3103	CG	ASN	P	192	18.205	4.752	19.173	0.00	0.00	PROA
ATOM	3104	OD1	ASN	P	192	17.616	5.644	18.599	0.00	0.00	PROA
ATOM	3105	ND2	ASN	P	192	19.524	4.374	18.867	0.00	0.00	PROA
ATOM	3106	HD21	ASN	P	192	19.983	4.712	18.046	0.00	0.00	PROA
ATOM	3107	HD22	ASN	P	192	19.921	3.656	19.439	0.00	0.00	PROA

ATOM	3108	C	ASN	P	192	15.356	3.733	18.936	0.00	0.00	PROA
ATOM	3109	O	ASN	P	192	14.929	3.294	17.904	0.00	0.00	PROA
ATOM	3110	N	ASP	P	193	14.858	4.931	19.345	0.00	0.00	PROA
ATOM	3111	HN	ASP	P	193	15.265	5.313	20.171	0.00	0.00	PROA
ATOM	3112	CA	ASP	P	193	13.640	5.497	18.772	0.00	0.00	PROA
ATOM	3113	HA	ASP	P	193	12.884	4.781	18.484	0.00	0.00	PROA
ATOM	3114	CB	ASP	P	193	12.976	6.613	19.692	0.00	0.00	PROA
ATOM	3115	HB1	ASP	P	193	12.236	7.252	19.165	0.00	0.00	PROA
ATOM	3116	HB2	ASP	P	193	13.832	7.239	20.024	0.00	0.00	PROA
ATOM	3117	CG	ASP	P	193	12.242	5.916	20.843	0.00	0.00	PROA
ATOM	3118	OD1	ASP	P	193	11.407	5.045	20.553	0.00	0.00	PROA
ATOM	3119	OD2	ASP	P	193	12.370	6.362	22.007	0.00	0.00	PROA
ATOM	3120	C	ASP	P	193	13.974	6.255	17.426	0.00	0.00	PROA
ATOM	3121	O	ASP	P	193	13.186	6.455	16.512	0.00	0.00	PROA
ATOM	3122	N	LEU	P	194	15.276	6.596	17.182	0.00	0.00	PROA
ATOM	3123	HN	LEU	P	194	16.015	6.291	17.778	0.00	0.00	PROA
ATOM	3124	CA	LEU	P	194	15.551	7.516	16.112	0.00	0.00	PROA
ATOM	3125	HA	LEU	P	194	14.941	8.405	16.174	0.00	0.00	PROA
ATOM	3126	CB	LEU	P	194	16.978	8.096	16.332	0.00	0.00	PROA
ATOM	3127	HB1	LEU	P	194	17.759	7.315	16.210	0.00	0.00	PROA
ATOM	3128	HB2	LEU	P	194	17.038	8.556	17.341	0.00	0.00	PROA
ATOM	3129	CG	LEU	P	194	17.308	9.236	15.301	0.00	0.00	PROA
ATOM	3130	HG	LEU	P	194	17.284	8.815	14.273	0.00	0.00	PROA
ATOM	3131	CD1	LEU	P	194	16.279	10.456	15.208	0.00	0.00	PROA
ATOM	3132	HD11	LEU	P	194	15.256	10.249	14.826	0.00	0.00	PROA
ATOM	3133	HD12	LEU	P	194	16.766	11.294	14.665	0.00	0.00	PROA
ATOM	3134	HD13	LEU	P	194	16.175	10.849	16.242	0.00	0.00	PROA
ATOM	3135	CD2	LEU	P	194	18.671	9.779	15.632	0.00	0.00	PROA
ATOM	3136	HD21	LEU	P	194	19.342	8.900	15.732	0.00	0.00	PROA
ATOM	3137	HD22	LEU	P	194	18.692	10.514	16.465	0.00	0.00	PROA
ATOM	3138	HD23	LEU	P	194	18.956	10.306	14.697	0.00	0.00	PROA
ATOM	3139	C	LEU	P	194	15.354	6.940	14.769	0.00	0.00	PROA
ATOM	3140	O	LEU	P	194	15.050	7.584	13.818	0.00	0.00	PROA
ATOM	3141	N	TRP	P	195	15.491	5.629	14.667	0.00	0.00	PROA
ATOM	3142	HN	TRP	P	195	15.827	5.120	15.456	0.00	0.00	PROA
ATOM	3143	CA	TRP	P	195	15.153	4.849	13.489	0.00	0.00	PROA
ATOM	3144	HA	TRP	P	195	15.713	5.170	12.623	0.00	0.00	PROA
ATOM	3145	CB	TRP	P	195	15.442	3.339	13.771	0.00	0.00	PROA
ATOM	3146	HB1	TRP	P	195	14.969	2.689	13.004	0.00	0.00	PROA
ATOM	3147	HB2	TRP	P	195	14.818	3.081	14.653	0.00	0.00	PROA
ATOM	3148	CG	TRP	P	195	16.899	2.997	13.955	0.00	0.00	PROA
ATOM	3149	CD1	TRP	P	195	17.671	2.837	15.033	0.00	0.00	PROA
ATOM	3150	HD1	TRP	P	195	17.276	2.993	16.027	0.00	0.00	PROA
ATOM	3151	NE1	TRP	P	195	18.960	2.525	14.650	0.00	0.00	PROA
ATOM	3152	HE1	TRP	P	195	19.619	1.992	15.135	0.00	0.00	PROA
ATOM	3153	CE2	TRP	P	195	19.026	2.584	13.291	0.00	0.00	PROA
ATOM	3154	CD2	TRP	P	195	17.712	2.800	12.793	0.00	0.00	PROA
ATOM	3155	CE3	TRP	P	195	17.487	2.803	11.366	0.00	0.00	PROA
ATOM	3156	HE3	TRP	P	195	16.566	3.131	10.909	0.00	0.00	PROA
ATOM	3157	CZ3	TRP	P	195	18.586	2.580	10.506	0.00	0.00	PROA
ATOM	3158	HZ3	TRP	P	195	18.562	2.682	9.431	0.00	0.00	PROA
ATOM	3159	CZ2	TRP	P	195	20.114	2.328	12.405	0.00	0.00	PROA
ATOM	3160	HZ2	TRP	P	195	21.107	2.285	12.829	0.00	0.00	PROA
ATOM	3161	CH2	TRP	P	195	19.878	2.410	11.050	0.00	0.00	PROA
ATOM	3162	HH2	TRP	P	195	20.670	2.233	10.338	0.00	0.00	PROA
ATOM	3163	C	TRP	P	195	13.644	4.965	13.106	0.00	0.00	PROA
ATOM	3164	O	TRP	P	195	13.351	5.390	11.995	0.00	0.00	PROA
ATOM	3165	N	VAL	P	196	12.746	4.783	14.148	0.00	0.00	PROA
ATOM	3166	HN	VAL	P	196	13.116	4.599	15.055	0.00	0.00	PROA
ATOM	3167	CA	VAL	P	196	11.296	4.895	13.957	0.00	0.00	PROA
ATOM	3168	HA	VAL	P	196	10.950	4.345	13.094	0.00	0.00	PROA

ATOM	3169	CB	VAL	P	196	10.562	4.354	15.266	0.00	0.00	PROA
ATOM	3170	HB	VAL	P	196	10.956	4.852	16.178	0.00	0.00	PROA
ATOM	3171	CG1	VAL	P	196	9.093	4.421	15.120	0.00	0.00	PROA
ATOM	3172	HG11	VAL	P	196	8.585	3.650	14.502	0.00	0.00	PROA
ATOM	3173	HG12	VAL	P	196	8.689	5.454	15.055	0.00	0.00	PROA
ATOM	3174	HG13	VAL	P	196	8.683	4.107	16.103	0.00	0.00	PROA
ATOM	3175	CG2	VAL	P	196	10.931	2.850	15.335	0.00	0.00	PROA
ATOM	3176	HG21	VAL	P	196	10.355	2.317	14.548	0.00	0.00	PROA
ATOM	3177	HG22	VAL	P	196	10.559	2.441	16.298	0.00	0.00	PROA
ATOM	3178	HG23	VAL	P	196	12.011	2.590	15.362	0.00	0.00	PROA
ATOM	3179	C	VAL	P	196	10.914	6.316	13.628	0.00	0.00	PROA
ATOM	3180	O	VAL	P	196	10.052	6.508	12.761	0.00	0.00	PROA
ATOM	3181	N	VAL	P	197	11.567	7.341	14.253	0.00	0.00	PROA
ATOM	3182	HN	VAL	P	197	12.034	7.083	15.095	0.00	0.00	PROA
ATOM	3183	CA	VAL	P	197	11.401	8.729	14.013	0.00	0.00	PROA
ATOM	3184	HA	VAL	P	197	10.337	8.879	14.123	0.00	0.00	PROA
ATOM	3185	CB	VAL	P	197	12.060	9.578	15.083	0.00	0.00	PROA
ATOM	3186	HB	VAL	P	197	13.099	9.196	15.182	0.00	0.00	PROA
ATOM	3187	CG1	VAL	P	197	11.981	11.078	14.825	0.00	0.00	PROA
ATOM	3188	HG11	VAL	P	197	12.420	11.380	13.850	0.00	0.00	PROA
ATOM	3189	HG12	VAL	P	197	12.474	11.695	15.607	0.00	0.00	PROA
ATOM	3190	HG13	VAL	P	197	10.938	11.434	14.692	0.00	0.00	PROA
ATOM	3191	CG2	VAL	P	197	11.391	9.203	16.425	0.00	0.00	PROA
ATOM	3192	HG21	VAL	P	197	11.961	9.546	17.315	0.00	0.00	PROA
ATOM	3193	HG22	VAL	P	197	11.343	8.107	16.604	0.00	0.00	PROA
ATOM	3194	HG23	VAL	P	197	10.385	9.673	16.453	0.00	0.00	PROA
ATOM	3195	C	VAL	P	197	11.788	9.065	12.600	0.00	0.00	PROA
ATOM	3196	O	VAL	P	197	11.082	9.814	11.855	0.00	0.00	PROA
ATOM	3197	N	VAL	P	198	12.892	8.529	12.124	0.00	0.00	PROA
ATOM	3198	HN	VAL	P	198	13.426	7.887	12.669	0.00	0.00	PROA
ATOM	3199	CA	VAL	P	198	13.391	8.708	10.775	0.00	0.00	PROA
ATOM	3200	HA	VAL	P	198	13.319	9.709	10.378	0.00	0.00	PROA
ATOM	3201	CB	VAL	P	198	14.853	8.340	10.717	0.00	0.00	PROA
ATOM	3202	HB	VAL	P	198	15.030	7.341	11.171	0.00	0.00	PROA
ATOM	3203	CG1	VAL	P	198	15.336	8.315	9.210	0.00	0.00	PROA
ATOM	3204	HG11	VAL	P	198	14.909	9.163	8.634	0.00	0.00	PROA
ATOM	3205	HG12	VAL	P	198	15.034	7.344	8.761	0.00	0.00	PROA
ATOM	3206	HG13	VAL	P	198	16.437	8.408	9.096	0.00	0.00	PROA
ATOM	3207	CG2	VAL	P	198	15.684	9.421	11.441	0.00	0.00	PROA
ATOM	3208	HG21	VAL	P	198	16.746	9.102	11.513	0.00	0.00	PROA
ATOM	3209	HG22	VAL	P	198	15.485	9.578	12.523	0.00	0.00	PROA
ATOM	3210	HG23	VAL	P	198	15.699	10.419	10.953	0.00	0.00	PROA
ATOM	3211	C	VAL	P	198	12.515	7.985	9.634	0.00	0.00	PROA
ATOM	3212	O	VAL	P	198	12.170	8.613	8.653	0.00	0.00	PROA
ATOM	3213	N	PHE	P	199	12.047	6.716	9.898	0.00	0.00	PROA
ATOM	3214	HN	PHE	P	199	12.420	6.209	10.672	0.00	0.00	PROA
ATOM	3215	CA	PHE	P	199	11.023	6.114	9.049	0.00	0.00	PROA
ATOM	3216	HA	PHE	P	199	11.254	6.265	8.005	0.00	0.00	PROA
ATOM	3217	CB	PHE	P	199	10.910	4.543	9.247	0.00	0.00	PROA
ATOM	3218	HB1	PHE	P	199	9.924	4.066	9.062	0.00	0.00	PROA
ATOM	3219	HB2	PHE	P	199	11.069	4.326	10.325	0.00	0.00	PROA
ATOM	3220	CG	PHE	P	199	12.043	3.911	8.489	0.00	0.00	PROA
ATOM	3221	CD1	PHE	P	199	13.366	4.024	8.886	0.00	0.00	PROA
ATOM	3222	HD1	PHE	P	199	13.778	4.534	9.744	0.00	0.00	PROA
ATOM	3223	CE1	PHE	P	199	14.370	3.474	8.027	0.00	0.00	PROA
ATOM	3224	HE1	PHE	P	199	15.403	3.654	8.286	0.00	0.00	PROA
ATOM	3225	CZ	PHE	P	199	13.982	2.723	6.925	0.00	0.00	PROA
ATOM	3226	HZ	PHE	P	199	14.681	2.163	6.321	0.00	0.00	PROA
ATOM	3227	CD2	PHE	P	199	11.649	3.139	7.369	0.00	0.00	PROA
ATOM	3228	HD2	PHE	P	199	10.580	3.166	7.216	0.00	0.00	PROA
ATOM	3229	CE2	PHE	P	199	12.595	2.542	6.605	0.00	0.00	PROA

ATOM	3230	HE2	PHE	P	199	12.297	1.896	5.792	0.00	0.00	PROA
ATOM	3231	C	PHE	P	199	9.734	6.922	9.070	0.00	0.00	PROA
ATOM	3232	O	PHE	P	199	9.229	7.121	7.957	0.00	0.00	PROA
ATOM	3233	N	GLN	P	200	9.185	7.376	10.228	0.00	0.00	PROA
ATOM	3234	HN	GLN	P	200	9.444	7.157	11.165	0.00	0.00	PROA
ATOM	3235	CA	GLN	P	200	7.967	8.109	10.185	0.00	0.00	PROA
ATOM	3236	HA	GLN	P	200	7.226	7.443	9.767	0.00	0.00	PROA
ATOM	3237	CB	GLN	P	200	7.343	8.389	11.567	0.00	0.00	PROA
ATOM	3238	HB1	GLN	P	200	6.717	9.304	11.632	0.00	0.00	PROA
ATOM	3239	HB2	GLN	P	200	8.215	8.653	12.202	0.00	0.00	PROA
ATOM	3240	CG	GLN	P	200	6.713	7.180	12.292	0.00	0.00	PROA
ATOM	3241	HG1	GLN	P	200	7.520	6.489	12.618	0.00	0.00	PROA
ATOM	3242	HG2	GLN	P	200	5.992	6.606	11.672	0.00	0.00	PROA
ATOM	3243	CD	GLN	P	200	6.099	7.490	13.701	0.00	0.00	PROA
ATOM	3244	OE1	GLN	P	200	6.682	7.175	14.748	0.00	0.00	PROA
ATOM	3245	NE2	GLN	P	200	4.892	8.142	13.768	0.00	0.00	PROA
ATOM	3246	HE21	GLN	P	200	4.408	8.367	14.613	0.00	0.00	PROA
ATOM	3247	HE22	GLN	P	200	4.478	8.565	12.962	0.00	0.00	PROA
ATOM	3248	C	GLN	P	200	7.948	9.314	9.290	0.00	0.00	PROA
ATOM	3249	O	GLN	P	200	7.035	9.711	8.628	0.00	0.00	PROA
ATOM	3250	N	PHE	P	201	9.068	10.056	9.365	0.00	0.00	PROA
ATOM	3251	HN	PHE	P	201	9.802	9.935	10.029	0.00	0.00	PROA
ATOM	3252	CA	PHE	P	201	9.231	11.174	8.479	0.00	0.00	PROA
ATOM	3253	HA	PHE	P	201	8.337	11.780	8.511	0.00	0.00	PROA
ATOM	3254	CB	PHE	P	201	10.321	12.144	9.003	0.00	0.00	PROA
ATOM	3255	HB1	PHE	P	201	10.519	12.875	8.190	0.00	0.00	PROA
ATOM	3256	HB2	PHE	P	201	11.241	11.616	9.336	0.00	0.00	PROA
ATOM	3257	CG	PHE	P	201	9.806	13.002	10.130	0.00	0.00	PROA
ATOM	3258	CD1	PHE	P	201	10.565	13.118	11.364	0.00	0.00	PROA
ATOM	3259	HD1	PHE	P	201	11.439	12.506	11.531	0.00	0.00	PROA
ATOM	3260	CE1	PHE	P	201	10.113	13.986	12.357	0.00	0.00	PROA
ATOM	3261	HE1	PHE	P	201	10.737	14.035	13.238	0.00	0.00	PROA
ATOM	3262	CZ	PHE	P	201	8.989	14.719	12.227	0.00	0.00	PROA
ATOM	3263	HZ	PHE	P	201	8.775	15.438	13.005	0.00	0.00	PROA
ATOM	3264	CD2	PHE	P	201	8.633	13.784	10.013	0.00	0.00	PROA
ATOM	3265	HD2	PHE	P	201	8.138	13.847	9.055	0.00	0.00	PROA
ATOM	3266	CE2	PHE	P	201	8.276	14.698	11.063	0.00	0.00	PROA
ATOM	3267	HE2	PHE	P	201	7.390	15.313	11.000	0.00	0.00	PROA
ATOM	3268	C	PHE	P	201	9.502	10.847	6.945	0.00	0.00	PROA
ATOM	3269	O	PHE	P	201	9.111	11.519	5.955	0.00	0.00	PROA
ATOM	3270	N	GLN	P	202	10.283	9.771	6.626	0.00	0.00	PROA
ATOM	3271	HN	GLN	P	202	10.536	9.180	7.387	0.00	0.00	PROA
ATOM	3272	CA	GLN	P	202	10.540	9.214	5.252	0.00	0.00	PROA
ATOM	3273	HA	GLN	P	202	11.056	9.854	4.551	0.00	0.00	PROA
ATOM	3274	CB	GLN	P	202	11.581	8.106	5.412	0.00	0.00	PROA
ATOM	3275	HB1	GLN	P	202	11.237	7.475	6.259	0.00	0.00	PROA
ATOM	3276	HB2	GLN	P	202	12.539	8.510	5.802	0.00	0.00	PROA
ATOM	3277	CG	GLN	P	202	11.888	7.226	4.258	0.00	0.00	PROA
ATOM	3278	HG1	GLN	P	202	12.827	6.653	4.406	0.00	0.00	PROA
ATOM	3279	HG2	GLN	P	202	11.834	7.790	3.302	0.00	0.00	PROA
ATOM	3280	CD	GLN	P	202	10.811	6.183	3.873	0.00	0.00	PROA
ATOM	3281	OE1	GLN	P	202	10.575	6.013	2.650	0.00	0.00	PROA
ATOM	3282	NE2	GLN	P	202	10.332	5.277	4.796	0.00	0.00	PROA
ATOM	3283	HE21	GLN	P	202	9.597	4.650	4.537	0.00	0.00	PROA
ATOM	3284	HE22	GLN	P	202	10.606	5.445	5.743	0.00	0.00	PROA
ATOM	3285	C	GLN	P	202	9.316	8.697	4.538	0.00	0.00	PROA
ATOM	3286	O	GLN	P	202	9.044	8.922	3.357	0.00	0.00	PROA
ATOM	3287	N	HSD	P	203	8.470	8.002	5.298	0.00	0.00	PROA
ATOM	3288	HN	HSD	P	203	8.717	7.672	6.206	0.00	0.00	PROA
ATOM	3289	CA	HSD	P	203	7.225	7.385	4.790	0.00	0.00	PROA
ATOM	3290	HA	HSD	P	203	7.397	6.650	4.018	0.00	0.00	PROA

```
ATOM   3291  CB  HSD P 203       6.528   6.737   5.974  0.00  0.00      PROA
ATOM   3292  HB1 HSD P 203       5.474   6.641   5.633  0.00  0.00      PROA
ATOM   3293  HB2 HSD P 203       6.612   7.373   6.881  0.00  0.00      PROA
ATOM   3294  ND1 HSD P 203       7.101   4.200   5.591  0.00  0.00      PROA
ATOM   3295  HD1 HSD P 203       6.948   4.123   4.605  0.00  0.00      PROA
ATOM   3296  CG  HSD P 203       6.950   5.360   6.377  0.00  0.00      PROA
ATOM   3297  CE1 HSD P 203       7.375   3.165   6.403  0.00  0.00      PROA
ATOM   3298  HE1 HSD P 203       7.575   2.154   6.050  0.00  0.00      PROA
ATOM   3299  NE2 HSD P 203       7.381   3.563   7.670  0.00  0.00      PROA
ATOM   3300  CD2 HSD P 203       7.122   4.890   7.620  0.00  0.00      PROA
ATOM   3301  HD2 HSD P 203       7.147   5.403   8.574  0.00  0.00      PROA
ATOM   3302  C   HSD P 203       6.297   8.405   4.154  0.00  0.00      PROA
ATOM   3303  O   HSD P 203       5.608   8.224   3.147  0.00  0.00      PROA
ATOM   3304  N   ILE P 204       6.034   9.568   4.855  0.00  0.00      PROA
ATOM   3305  HN  ILE P 204       6.550   9.753   5.687  0.00  0.00      PROA
ATOM   3306  CA  ILE P 204       5.282  10.661   4.356  0.00  0.00      PROA
ATOM   3307  HA  ILE P 204       4.334  10.250   4.045  0.00  0.00      PROA
ATOM   3308  CB  ILE P 204       4.956  11.721   5.360  0.00  0.00      PROA
ATOM   3309  HB  ILE P 204       4.333  11.145   6.077  0.00  0.00      PROA
ATOM   3310  CG2 ILE P 204       6.198  12.312   6.094  0.00  0.00      PROA
ATOM   3311 HG21 ILE P 204       5.929  13.124   6.803  0.00  0.00      PROA
ATOM   3312 HG22 ILE P 204       6.914  12.811   5.407  0.00  0.00      PROA
ATOM   3313 HG23 ILE P 204       6.695  11.476   6.631  0.00  0.00      PROA
ATOM   3314  CG1 ILE P 204       4.096  12.939   4.912  0.00  0.00      PROA
ATOM   3315 HG11 ILE P 204       3.451  12.521   4.110  0.00  0.00      PROA
ATOM   3316 HG12 ILE P 204       4.757  13.609   4.321  0.00  0.00      PROA
ATOM   3317  CD  ILE P 204       3.167  13.422   5.949  0.00  0.00      PROA
ATOM   3318  HD1 ILE P 204       2.523  14.166   5.433  0.00  0.00      PROA
ATOM   3319  HD2 ILE P 204       3.718  13.946   6.758  0.00  0.00      PROA
ATOM   3320  HD3 ILE P 204       2.557  12.635   6.441  0.00  0.00      PROA
ATOM   3321  C   ILE P 204       5.879  11.260   3.110  0.00  0.00      PROA
ATOM   3322  O   ILE P 204       5.154  11.550   2.106  0.00  0.00      PROA
ATOM   3323  N   MET P 205       7.239  11.461   3.053  0.00  0.00      PROA
ATOM   3324  HN  MET P 205       7.733  11.359   3.913  0.00  0.00      PROA
ATOM   3325  CA  MET P 205       7.903  12.083   1.963  0.00  0.00      PROA
ATOM   3326  HA  MET P 205       7.483  13.022   1.635  0.00  0.00      PROA
ATOM   3327  CB  MET P 205       9.389  12.305   2.349  0.00  0.00      PROA
ATOM   3328  HB1 MET P 205       9.818  11.305   2.572  0.00  0.00      PROA
ATOM   3329  HB2 MET P 205       9.479  12.896   3.285  0.00  0.00      PROA
ATOM   3330  CG  MET P 205      10.346  12.938   1.282  0.00  0.00      PROA
ATOM   3331  HG1 MET P 205      10.304  12.134   0.515  0.00  0.00      PROA
ATOM   3332  HG2 MET P 205      11.344  13.012   1.764  0.00  0.00      PROA
ATOM   3333  SD  MET P 205       9.932  14.544   0.370  0.00  0.00      PROA
ATOM   3334  CE  MET P 205       9.532  15.399   1.934  0.00  0.00      PROA
ATOM   3335  HE1 MET P 205       8.634  14.936   2.396  0.00  0.00      PROA
ATOM   3336  HE2 MET P 205       9.360  16.480   1.742  0.00  0.00      PROA
ATOM   3337  HE3 MET P 205      10.318  15.172   2.685  0.00  0.00      PROA
ATOM   3338  C   MET P 205       7.911  11.127   0.733  0.00  0.00      PROA
ATOM   3339  O   MET P 205       7.380  11.464  -0.302  0.00  0.00      PROA
ATOM   3340  N   VAL P 206       8.469   9.940   0.832  0.00  0.00      PROA
ATOM   3341  HN  VAL P 206       8.860   9.658   1.705  0.00  0.00      PROA
ATOM   3342  CA  VAL P 206       8.709   9.081  -0.365  0.00  0.00      PROA
ATOM   3343  HA  VAL P 206       8.842   9.749  -1.203  0.00  0.00      PROA
ATOM   3344  CB  VAL P 206      10.018   8.288  -0.053  0.00  0.00      PROA
ATOM   3345  HB  VAL P 206       9.889   7.611   0.819  0.00  0.00      PROA
ATOM   3346  CG1 VAL P 206      10.306   7.326  -1.259  0.00  0.00      PROA
ATOM   3347 HG11 VAL P 206       9.536   6.525  -1.286  0.00  0.00      PROA
ATOM   3348 HG12 VAL P 206      11.280   6.818  -1.093  0.00  0.00      PROA
ATOM   3349 HG13 VAL P 206      10.468   8.005  -2.123  0.00  0.00      PROA
ATOM   3350  CG2 VAL P 206      11.236   9.278  -0.070  0.00  0.00      PROA
ATOM   3351 HG21 VAL P 206      12.216   8.756  -0.035  0.00  0.00      PROA
```

ATOM	3352	HG22	VAL	P	206	11.038	9.897	0.831	0.00	0.00	PROA
ATOM	3353	HG23	VAL	P	206	11.225	9.986	-0.927	0.00	0.00	PROA
ATOM	3354	C	VAL	P	206	7.471	8.168	-0.794	0.00	0.00	PROA
ATOM	3355	O	VAL	P	206	7.363	7.783	-1.949	0.00	0.00	PROA
ATOM	3356	N	GLY	P	207	6.427	8.004	0.135	0.00	0.00	PROA
ATOM	3357	HN	GLY	P	207	6.413	8.496	1.002	0.00	0.00	PROA
ATOM	3358	CA	GLY	P	207	5.289	7.143	-0.024	0.00	0.00	PROA
ATOM	3359	HA1	GLY	P	207	5.120	6.720	0.955	0.00	0.00	PROA
ATOM	3360	HA2	GLY	P	207	5.447	6.407	-0.798	0.00	0.00	PROA
ATOM	3361	C	GLY	P	207	3.984	7.884	-0.328	0.00	0.00	PROA
ATOM	3362	O	GLY	P	207	3.074	7.285	-0.892	0.00	0.00	PROA
ATOM	3363	N	LEU	P	208	4.046	9.235	-0.072	0.00	0.00	PROA
ATOM	3364	HN	LEU	P	208	4.947	9.538	0.230	0.00	0.00	PROA
ATOM	3365	CA	LEU	P	208	2.900	10.102	-0.290	0.00	0.00	PROA
ATOM	3366	HA	LEU	P	208	2.300	9.557	-1.003	0.00	0.00	PROA
ATOM	3367	CB	LEU	P	208	2.039	10.146	0.968	0.00	0.00	PROA
ATOM	3368	HB1	LEU	P	208	2.696	10.537	1.774	0.00	0.00	PROA
ATOM	3369	HB2	LEU	P	208	1.838	9.055	1.033	0.00	0.00	PROA
ATOM	3370	CG	LEU	P	208	0.750	10.988	1.014	0.00	0.00	PROA
ATOM	3371	HG	LEU	P	208	0.873	12.081	0.860	0.00	0.00	PROA
ATOM	3372	CD1	LEU	P	208	-0.266	10.355	0.125	0.00	0.00	PROA
ATOM	3373	HD11	LEU	P	208	0.026	10.503	-0.936	0.00	0.00	PROA
ATOM	3374	HD12	LEU	P	208	-1.277	10.814	0.182	0.00	0.00	PROA
ATOM	3375	HD13	LEU	P	208	-0.387	9.271	0.339	0.00	0.00	PROA
ATOM	3376	CD2	LEU	P	208	0.218	10.890	2.455	0.00	0.00	PROA
ATOM	3377	HD21	LEU	P	208	0.928	11.368	3.163	0.00	0.00	PROA
ATOM	3378	HD22	LEU	P	208	0.064	9.825	2.731	0.00	0.00	PROA
ATOM	3379	HD23	LEU	P	208	-0.672	11.536	2.612	0.00	0.00	PROA
ATOM	3380	C	LEU	P	208	3.122	11.442	-0.883	0.00	0.00	PROA
ATOM	3381	O	LEU	P	208	2.673	11.695	-2.021	0.00	0.00	PROA
ATOM	3382	N	ILE	P	209	3.850	12.314	-0.208	0.00	0.00	PROA
ATOM	3383	HN	ILE	P	209	4.258	12.146	0.685	0.00	0.00	PROA
ATOM	3384	CA	ILE	P	209	3.956	13.631	-0.768	0.00	0.00	PROA
ATOM	3385	HA	ILE	P	209	2.973	13.912	-1.118	0.00	0.00	PROA
ATOM	3386	CB	ILE	P	209	4.469	14.719	0.167	0.00	0.00	PROA
ATOM	3387	HB	ILE	P	209	5.560	14.525	0.246	0.00	0.00	PROA
ATOM	3388	CG2	ILE	P	209	4.278	16.166	-0.424	0.00	0.00	PROA
ATOM	3389	HG21	ILE	P	209	3.207	16.263	-0.703	0.00	0.00	PROA
ATOM	3390	HG22	ILE	P	209	4.911	16.327	-1.322	0.00	0.00	PROA
ATOM	3391	HG23	ILE	P	209	4.522	16.903	0.371	0.00	0.00	PROA
ATOM	3392	CG1	ILE	P	209	3.858	14.504	1.579	0.00	0.00	PROA
ATOM	3393	HG11	ILE	P	209	4.388	15.223	2.239	0.00	0.00	PROA
ATOM	3394	HG12	ILE	P	209	4.120	13.538	2.061	0.00	0.00	PROA
ATOM	3395	CD	ILE	P	209	2.264	14.610	1.755	0.00	0.00	PROA
ATOM	3396	HD1	ILE	P	209	1.801	14.325	0.786	0.00	0.00	PROA
ATOM	3397	HD2	ILE	P	209	1.788	15.594	1.953	0.00	0.00	PROA
ATOM	3398	HD3	ILE	P	209	1.914	13.931	2.562	0.00	0.00	PROA
ATOM	3399	C	ILE	P	209	4.651	13.784	-2.173	0.00	0.00	PROA
ATOM	3400	O	ILE	P	209	4.143	14.383	-3.101	0.00	0.00	PROA
ATOM	3401	N	LEU	P	210	5.879	13.233	-2.343	0.00	0.00	PROA
ATOM	3402	HN	LEU	P	210	6.353	12.768	-1.600	0.00	0.00	PROA
ATOM	3403	CA	LEU	P	210	6.534	13.176	-3.606	0.00	0.00	PROA
ATOM	3404	HA	LEU	P	210	6.559	14.209	-3.922	0.00	0.00	PROA
ATOM	3405	CB	LEU	P	210	8.003	12.681	-3.270	0.00	0.00	PROA
ATOM	3406	HB1	LEU	P	210	8.030	11.605	-2.993	0.00	0.00	PROA
ATOM	3407	HB2	LEU	P	210	8.415	13.121	-2.336	0.00	0.00	PROA
ATOM	3408	CG	LEU	P	210	8.968	12.806	-4.407	0.00	0.00	PROA
ATOM	3409	HG	LEU	P	210	8.678	12.090	-5.206	0.00	0.00	PROA
ATOM	3410	CD1	LEU	P	210	9.017	14.198	-5.004	0.00	0.00	PROA
ATOM	3411	HD11	LEU	P	210	9.252	14.940	-4.212	0.00	0.00	PROA
ATOM	3412	HD12	LEU	P	210	8.009	14.409	-5.420	0.00	0.00	PROA

ATOM 3413 HD13 LEU P 210 9.816 14.276 -5.772 0.00 0.00 PROA
ATOM 3414 CD2 LEU P 210 10.395 12.338 -4.010 0.00 0.00 PROA
ATOM 3415 HD21 LEU P 210 10.802 11.798 -4.891 0.00 0.00 PROA
ATOM 3416 HD22 LEU P 210 10.376 11.699 -3.100 0.00 0.00 PROA
ATOM 3417 HD23 LEU P 210 11.083 13.182 -3.792 0.00 0.00 PROA
ATOM 3418 C LEU P 210 5.876 12.369 -4.715 0.00 0.00 PROA
ATOM 3419 O LEU P 210 5.910 12.933 -5.809 0.00 0.00 PROA
ATOM 3420 N PRO P 211 5.301 11.216 -4.519 0.00 0.00 PROA
ATOM 3421 CD PRO P 211 5.773 10.146 -3.572 0.00 0.00 PROA
ATOM 3422 HD1 PRO P 211 5.778 10.405 -2.491 0.00 0.00 PROA
ATOM 3423 HD2 PRO P 211 6.774 9.813 -3.920 0.00 0.00 PROA
ATOM 3424 CA PRO P 211 4.635 10.578 -5.616 0.00 0.00 PROA
ATOM 3425 HA PRO P 211 5.299 10.786 -6.442 0.00 0.00 PROA
ATOM 3426 CB PRO P 211 4.606 9.047 -5.367 0.00 0.00 PROA
ATOM 3427 HB1 PRO P 211 5.494 8.576 -5.841 0.00 0.00 PROA
ATOM 3428 HB2 PRO P 211 3.636 8.560 -5.606 0.00 0.00 PROA
ATOM 3429 CG PRO P 211 4.880 8.945 -3.849 0.00 0.00 PROA
ATOM 3430 HG1 PRO P 211 3.901 9.112 -3.351 0.00 0.00 PROA
ATOM 3431 HG2 PRO P 211 5.195 7.958 -3.448 0.00 0.00 PROA
ATOM 3432 C PRO P 211 3.309 11.209 -5.955 0.00 0.00 PROA
ATOM 3433 O PRO P 211 2.909 11.334 -7.096 0.00 0.00 PROA
ATOM 3434 N GLY P 212 2.557 11.669 -4.925 0.00 0.00 PROA
ATOM 3435 HN GLY P 212 2.869 11.690 -3.978 0.00 0.00 PROA
ATOM 3436 CA GLY P 212 1.484 12.614 -4.979 0.00 0.00 PROA
ATOM 3437 HA1 GLY P 212 1.308 13.110 -4.036 0.00 0.00 PROA
ATOM 3438 HA2 GLY P 212 0.651 12.043 -5.361 0.00 0.00 PROA
ATOM 3439 C GLY P 212 1.556 13.738 -6.021 0.00 0.00 PROA
ATOM 3440 O GLY P 212 0.804 13.763 -6.977 0.00 0.00 PROA
ATOM 3441 N ILE P 213 2.588 14.592 -5.961 0.00 0.00 PROA
ATOM 3442 HN ILE P 213 3.270 14.476 -5.243 0.00 0.00 PROA
ATOM 3443 CA ILE P 213 2.757 15.650 -6.924 0.00 0.00 PROA
ATOM 3444 HA ILE P 213 1.802 16.140 -7.047 0.00 0.00 PROA
ATOM 3445 CB ILE P 213 3.833 16.716 -6.517 0.00 0.00 PROA
ATOM 3446 HB ILE P 213 3.933 17.449 -7.346 0.00 0.00 PROA
ATOM 3447 CG2 ILE P 213 3.360 17.447 -5.250 0.00 0.00 PROA
ATOM 3448 HG21 ILE P 213 2.367 17.928 -5.384 0.00 0.00 PROA
ATOM 3449 HG22 ILE P 213 4.073 18.288 -5.109 0.00 0.00 PROA
ATOM 3450 HG23 ILE P 213 3.441 16.851 -4.316 0.00 0.00 PROA
ATOM 3451 CG1 ILE P 213 5.343 16.222 -6.389 0.00 0.00 PROA
ATOM 3452 HG11 ILE P 213 5.665 15.433 -7.101 0.00 0.00 PROA
ATOM 3453 HG12 ILE P 213 5.472 15.845 -5.352 0.00 0.00 PROA
ATOM 3454 CD ILE P 213 6.311 17.373 -6.776 0.00 0.00 PROA
ATOM 3455 HD1 ILE P 213 7.370 17.165 -6.516 0.00 0.00 PROA
ATOM 3456 HD2 ILE P 213 6.212 18.217 -6.061 0.00 0.00 PROA
ATOM 3457 HD3 ILE P 213 6.187 17.680 -7.837 0.00 0.00 PROA
ATOM 3458 C ILE P 213 3.169 15.167 -8.331 0.00 0.00 PROA
ATOM 3459 O ILE P 213 2.684 15.686 -9.347 0.00 0.00 PROA
ATOM 3460 N VAL P 214 3.865 13.949 -8.435 0.00 0.00 PROA
ATOM 3461 HN VAL P 214 4.260 13.549 -7.611 0.00 0.00 PROA
ATOM 3462 CA VAL P 214 4.064 13.317 -9.747 0.00 0.00 PROA
ATOM 3463 HA VAL P 214 4.441 14.091 -10.399 0.00 0.00 PROA
ATOM 3464 CB VAL P 214 5.111 12.180 -9.752 0.00 0.00 PROA
ATOM 3465 HB VAL P 214 4.840 11.331 -9.089 0.00 0.00 PROA
ATOM 3466 CG1 VAL P 214 5.222 11.513 -11.195 0.00 0.00 PROA
ATOM 3467 HG11 VAL P 214 4.263 11.003 -11.432 0.00 0.00 PROA
ATOM 3468 HG12 VAL P 214 6.079 10.823 -11.349 0.00 0.00 PROA
ATOM 3469 HG13 VAL P 214 5.320 12.330 -11.941 0.00 0.00 PROA
ATOM 3470 CG2 VAL P 214 6.522 12.711 -9.292 0.00 0.00 PROA
ATOM 3471 HG21 VAL P 214 7.281 11.903 -9.357 0.00 0.00 PROA
ATOM 3472 HG22 VAL P 214 6.487 13.291 -8.345 0.00 0.00 PROA
ATOM 3473 HG23 VAL P 214 6.841 13.495 -10.010 0.00 0.00 PROA

ATOM	3474	C	VAL	P	214	2.779	12.860	-10.392	0.00	0.00	PROA
ATOM	3475	O	VAL	P	214	2.501	12.995	-11.590	0.00	0.00	PROA
ATOM	3476	N	ILE	P	215	1.873	12.274	-9.504	0.00	0.00	PROA
ATOM	3477	HN	ILE	P	215	2.170	11.922	-8.619	0.00	0.00	PROA
ATOM	3478	CA	ILE	P	215	0.474	11.814	-9.958	0.00	0.00	PROA
ATOM	3479	HA	ILE	P	215	0.615	11.279	-10.885	0.00	0.00	PROA
ATOM	3480	CB	ILE	P	215	-0.285	11.063	-8.824	0.00	0.00	PROA
ATOM	3481	HB	ILE	P	215	-0.272	11.637	-7.874	0.00	0.00	PROA
ATOM	3482	CG2	ILE	P	215	-1.780	10.821	-9.297	0.00	0.00	PROA
ATOM	3483	HG21	ILE	P	215	-1.599	10.461	-10.332	0.00	0.00	PROA
ATOM	3484	HG22	ILE	P	215	-2.389	11.750	-9.308	0.00	0.00	PROA
ATOM	3485	HG23	ILE	P	215	-2.196	10.004	-8.670	0.00	0.00	PROA
ATOM	3486	CG1	ILE	P	215	0.357	9.687	-8.648	0.00	0.00	PROA
ATOM	3487	HG11	ILE	P	215	1.456	9.765	-8.792	0.00	0.00	PROA
ATOM	3488	HG12	ILE	P	215	-0.050	8.968	-9.390	0.00	0.00	PROA
ATOM	3489	CD	ILE	P	215	0.063	9.112	-7.287	0.00	0.00	PROA
ATOM	3490	HD1	ILE	P	215	0.464	9.790	-6.503	0.00	0.00	PROA
ATOM	3491	HD2	ILE	P	215	0.652	8.179	-7.163	0.00	0.00	PROA
ATOM	3492	HD3	ILE	P	215	-1.008	8.974	-7.025	0.00	0.00	PROA
ATOM	3493	C	ILE	P	215	-0.347	13.026	-10.389	0.00	0.00	PROA
ATOM	3494	O	ILE	P	215	-1.027	13.043	-11.422	0.00	0.00	PROA
ATOM	3495	N	LEU	P	216	-0.251	14.120	-9.626	0.00	0.00	PROA
ATOM	3496	HN	LEU	P	216	0.249	14.038	-8.767	0.00	0.00	PROA
ATOM	3497	CA	LEU	P	216	-0.922	15.425	-9.889	0.00	0.00	PROA
ATOM	3498	HA	LEU	P	216	-1.923	15.175	-10.209	0.00	0.00	PROA
ATOM	3499	CB	LEU	P	216	-0.865	16.289	-8.542	0.00	0.00	PROA
ATOM	3500	HB1	LEU	P	216	0.152	16.721	-8.427	0.00	0.00	PROA
ATOM	3501	HB2	LEU	P	216	-1.083	15.610	-7.690	0.00	0.00	PROA
ATOM	3502	CG	LEU	P	216	-1.904	17.446	-8.489	0.00	0.00	PROA
ATOM	3503	HG	LEU	P	216	-1.695	18.103	-9.360	0.00	0.00	PROA
ATOM	3504	CD1	LEU	P	216	-3.355	16.971	-8.555	0.00	0.00	PROA
ATOM	3505	HD11	LEU	P	216	-3.575	16.442	-9.507	0.00	0.00	PROA
ATOM	3506	HD12	LEU	P	216	-4.031	17.815	-8.298	0.00	0.00	PROA
ATOM	3507	HD13	LEU	P	216	-3.629	16.284	-7.726	0.00	0.00	PROA
ATOM	3508	CD2	LEU	P	216	-1.699	18.345	-7.263	0.00	0.00	PROA
ATOM	3509	HD21	LEU	P	216	-0.685	18.781	-7.391	0.00	0.00	PROA
ATOM	3510	HD22	LEU	P	216	-1.786	17.772	-6.315	0.00	0.00	PROA
ATOM	3511	HD23	LEU	P	216	-2.501	19.108	-7.174	0.00	0.00	PROA
ATOM	3512	C	LEU	P	216	-0.386	16.208	-11.058	0.00	0.00	PROA
ATOM	3513	O	LEU	P	216	-1.221	16.678	-11.863	0.00	0.00	PROA
ATOM	3514	N	SER	P	217	0.916	16.318	-11.262	0.00	0.00	PROA
ATOM	3515	HN	SER	P	217	1.491	15.879	-10.576	0.00	0.00	PROA
ATOM	3516	CA	SER	P	217	1.419	16.995	-12.456	0.00	0.00	PROA
ATOM	3517	HA	SER	P	217	1.077	18.018	-12.404	0.00	0.00	PROA
ATOM	3518	CB	SER	P	217	2.963	17.344	-12.575	0.00	0.00	PROA
ATOM	3519	HB1	SER	P	217	3.474	16.360	-12.641	0.00	0.00	PROA
ATOM	3520	HB2	SER	P	217	3.369	17.701	-11.605	0.00	0.00	PROA
ATOM	3521	OG	SER	P	217	3.401	18.250	-13.688	0.00	0.00	PROA
ATOM	3522	HG1	SER	P	217	3.004	17.968	-14.515	0.00	0.00	PROA
ATOM	3523	C	SER	P	217	0.994	16.448	-13.830	0.00	0.00	PROA
ATOM	3524	O	SER	P	217	0.633	17.193	-14.699	0.00	0.00	PROA
ATOM	3525	N	CYS	P	218	0.961	15.059	-13.890	0.00	0.00	PROA
ATOM	3526	HN	CYS	P	218	1.285	14.436	-13.182	0.00	0.00	PROA
ATOM	3527	CA	CYS	P	218	0.415	14.327	-15.039	0.00	0.00	PROA
ATOM	3528	HA	CYS	P	218	0.971	14.726	-15.875	0.00	0.00	PROA
ATOM	3529	CB	CYS	P	218	0.572	12.872	-14.749	0.00	0.00	PROA
ATOM	3530	HB1	CYS	P	218	0.205	12.292	-15.622	0.00	0.00	PROA
ATOM	3531	HB2	CYS	P	218	-0.166	12.730	-13.930	0.00	0.00	PROA
ATOM	3532	SG	CYS	P	218	2.343	12.344	-14.585	0.00	0.00	PROA
ATOM	3533	HG1	CYS	P	218	2.438	12.552	-13.280	0.00	0.00	PROA
ATOM	3534	C	CYS	P	218	-1.035	14.617	-15.335	0.00	0.00	PROA

ATOM	3535	O	CYS	P	218	-1.357	14.981	-16.461	0.00	0.00	PROA
ATOM	3536	N	TYR	P	219	-1.932	14.581	-14.351	0.00	0.00	PROA
ATOM	3537	HN	TYR	P	219	-1.671	14.242	-13.450	0.00	0.00	PROA
ATOM	3538	CA	TYR	P	219	-3.290	15.094	-14.471	0.00	0.00	PROA
ATOM	3539	HA	TYR	P	219	-3.660	14.616	-15.366	0.00	0.00	PROA
ATOM	3540	CB	TYR	P	219	-4.226	14.684	-13.343	0.00	0.00	PROA
ATOM	3541	HB1	TYR	P	219	-5.262	15.081	-13.410	0.00	0.00	PROA
ATOM	3542	HB2	TYR	P	219	-3.689	14.777	-12.374	0.00	0.00	PROA
ATOM	3543	CG	TYR	P	219	-4.523	13.198	-13.553	0.00	0.00	PROA
ATOM	3544	CD1	TYR	P	219	-4.129	12.195	-12.671	0.00	0.00	PROA
ATOM	3545	HD1	TYR	P	219	-3.718	12.485	-11.716	0.00	0.00	PROA
ATOM	3546	CE1	TYR	P	219	-4.088	10.840	-13.069	0.00	0.00	PROA
ATOM	3547	HE1	TYR	P	219	-3.601	10.103	-12.448	0.00	0.00	PROA
ATOM	3548	CZ	TYR	P	219	-4.789	10.407	-14.196	0.00	0.00	PROA
ATOM	3549	OH	TYR	P	219	-5.076	9.086	-14.443	0.00	0.00	PROA
ATOM	3550	HH	TYR	P	219	-4.584	8.489	-13.874	0.00	0.00	PROA
ATOM	3551	CD2	TYR	P	219	-5.275	12.784	-14.708	0.00	0.00	PROA
ATOM	3552	HD2	TYR	P	219	-5.700	13.462	-15.433	0.00	0.00	PROA
ATOM	3553	CE2	TYR	P	219	-5.358	11.370	-15.025	0.00	0.00	PROA
ATOM	3554	HE2	TYR	P	219	-5.968	11.064	-15.862	0.00	0.00	PROA
ATOM	3555	C	TYR	P	219	-3.419	16.577	-14.825	0.00	0.00	PROA
ATOM	3556	O	TYR	P	219	-4.219	16.864	-15.712	0.00	0.00	PROA
ATOM	3557	N	CYS	P	220	-2.647	17.500	-14.232	0.00	0.00	PROA
ATOM	3558	HN	CYS	P	220	-2.126	17.183	-13.444	0.00	0.00	PROA
ATOM	3559	CA	CYS	P	220	-2.473	18.882	-14.587	0.00	0.00	PROA
ATOM	3560	HA	CYS	P	220	-3.464	19.205	-14.306	0.00	0.00	PROA
ATOM	3561	CB	CYS	P	220	-1.446	19.673	-13.813	0.00	0.00	PROA
ATOM	3562	HB1	CYS	P	220	-1.347	20.694	-14.239	0.00	0.00	PROA
ATOM	3563	HB2	CYS	P	220	-0.412	19.274	-13.888	0.00	0.00	PROA
ATOM	3564	SG	CYS	P	220	-1.849	19.916	-12.027	0.00	0.00	PROA
ATOM	3565	HG1	CYS	P	220	-1.399	18.717	-11.684	0.00	0.00	PROA
ATOM	3566	C	CYS	P	220	-2.272	18.969	-16.069	0.00	0.00	PROA
ATOM	3567	O	CYS	P	220	-3.016	19.628	-16.809	0.00	0.00	PROA
ATOM	3568	N	ILE	P	221	-1.198	18.400	-16.622	0.00	0.00	PROA
ATOM	3569	HN	ILE	P	221	-0.504	17.905	-16.105	0.00	0.00	PROA
ATOM	3570	CA	ILE	P	221	-0.909	18.394	-18.036	0.00	0.00	PROA
ATOM	3571	HA	ILE	P	221	-1.078	19.401	-18.388	0.00	0.00	PROA
ATOM	3572	CB	ILE	P	221	0.583	18.059	-18.294	0.00	0.00	PROA
ATOM	3573	HB	ILE	P	221	0.562	17.006	-17.940	0.00	0.00	PROA
ATOM	3574	CG2	ILE	P	221	0.883	18.167	-19.844	0.00	0.00	PROA
ATOM	3575	HG21	ILE	P	221	0.382	19.069	-20.258	0.00	0.00	PROA
ATOM	3576	HG22	ILE	P	221	0.532	17.270	-20.398	0.00	0.00	PROA
ATOM	3577	HG23	ILE	P	221	1.961	18.313	-20.071	0.00	0.00	PROA
ATOM	3578	CG1	ILE	P	221	1.655	18.878	-17.404	0.00	0.00	PROA
ATOM	3579	HG11	ILE	P	221	2.574	18.325	-17.693	0.00	0.00	PROA
ATOM	3580	HG12	ILE	P	221	1.544	18.686	-16.315	0.00	0.00	PROA
ATOM	3581	CD	ILE	P	221	1.806	20.388	-17.648	0.00	0.00	PROA
ATOM	3582	HD1	ILE	P	221	2.726	20.681	-17.098	0.00	0.00	PROA
ATOM	3583	HD2	ILE	P	221	0.927	20.976	-17.307	0.00	0.00	PROA
ATOM	3584	HD3	ILE	P	221	1.965	20.679	-18.708	0.00	0.00	PROA
ATOM	3585	C	ILE	P	221	-1.814	17.730	-19.028	0.00	0.00	PROA
ATOM	3586	O	ILE	P	221	-2.189	18.345	-20.041	0.00	0.00	PROA
ATOM	3587	N	ILE	P	222	-2.294	16.487	-18.654	0.00	0.00	PROA
ATOM	3588	HN	ILE	P	222	-2.001	16.072	-17.796	0.00	0.00	PROA
ATOM	3589	CA	ILE	P	222	-3.290	15.780	-19.391	0.00	0.00	PROA
ATOM	3590	HA	ILE	P	222	-2.721	15.530	-20.274	0.00	0.00	PROA
ATOM	3591	CB	ILE	P	222	-3.533	14.450	-18.679	0.00	0.00	PROA
ATOM	3592	HB	ILE	P	222	-3.575	14.764	-17.614	0.00	0.00	PROA
ATOM	3593	CG2	ILE	P	222	-4.746	13.752	-19.213	0.00	0.00	PROA
ATOM	3594	HG21	ILE	P	222	-4.829	12.800	-18.647	0.00	0.00	PROA
ATOM	3595	HG22	ILE	P	222	-4.622	13.502	-20.289	0.00	0.00	PROA

```
ATOM   3596 HG23 ILE P 222      -5.725  14.253 -19.057  0.00  0.00      PROA
ATOM   3597  CG1 ILE P 222      -2.345  13.520 -18.834  0.00  0.00      PROA
ATOM   3598 HG11 ILE P 222      -1.329  13.968 -18.776  0.00  0.00      PROA
ATOM   3599 HG12 ILE P 222      -2.357  13.188 -19.895  0.00  0.00      PROA
ATOM   3600  CD  ILE P 222      -2.347  12.322 -17.909  0.00  0.00      PROA
ATOM   3601  HD1 ILE P 222      -2.878  11.382 -18.173  0.00  0.00      PROA
ATOM   3602  HD2 ILE P 222      -2.655  12.673 -16.901  0.00  0.00      PROA
ATOM   3603  HD3 ILE P 222      -1.297  11.990 -17.756  0.00  0.00      PROA
ATOM   3604  C   ILE P 222      -4.586  16.574 -19.649  0.00  0.00      PROA
ATOM   3605  O   ILE P 222      -5.132  16.643 -20.729  0.00  0.00      PROA
ATOM   3606  N   ILE P 223      -5.067  17.271 -18.562  0.00  0.00      PROA
ATOM   3607  HN  ILE P 223      -4.583  17.258 -17.691  0.00  0.00      PROA
ATOM   3608  CA  ILE P 223      -6.276  18.063 -18.513  0.00  0.00      PROA
ATOM   3609  HA  ILE P 223      -7.199  17.577 -18.795  0.00  0.00      PROA
ATOM   3610  CB  ILE P 223      -6.725  18.356 -17.063  0.00  0.00      PROA
ATOM   3611  HB  ILE P 223      -5.747  18.674 -16.642  0.00  0.00      PROA
ATOM   3612  CG2 ILE P 223      -7.753  19.497 -17.020  0.00  0.00      PROA
ATOM   3613 HG21 ILE P 223      -8.711  19.323 -17.554  0.00  0.00      PROA
ATOM   3614 HG22 ILE P 223      -7.319  20.442 -17.410  0.00  0.00      PROA
ATOM   3615 HG23 ILE P 223      -7.959  19.644 -15.938  0.00  0.00      PROA
ATOM   3616  CG1 ILE P 223      -7.136  17.077 -16.215  0.00  0.00      PROA
ATOM   3617 HG11 ILE P 223      -7.132  17.347 -15.137  0.00  0.00      PROA
ATOM   3618 HG12 ILE P 223      -6.404  16.270 -16.427  0.00  0.00      PROA
ATOM   3619  CD  ILE P 223      -8.583  16.602 -16.615  0.00  0.00      PROA
ATOM   3620  HD1 ILE P 223      -8.699  16.492 -17.715  0.00  0.00      PROA
ATOM   3621  HD2 ILE P 223      -9.393  17.296 -16.302  0.00  0.00      PROA
ATOM   3622  HD3 ILE P 223      -8.874  15.669 -16.086  0.00  0.00      PROA
ATOM   3623  C   ILE P 223      -6.354  19.299 -19.473  0.00  0.00      PROA
ATOM   3624  O   ILE P 223      -7.255  19.364 -20.274  0.00  0.00      PROA
ATOM   3625  N   SER P 224      -5.231  20.016 -19.543  0.00  0.00      PROA
ATOM   3626  HN  SER P 224      -4.462  19.895 -18.920  0.00  0.00      PROA
ATOM   3627  CA  SER P 224      -4.928  21.021 -20.612  0.00  0.00      PROA
ATOM   3628  HA  SER P 224      -5.763  21.704 -20.663  0.00  0.00      PROA
ATOM   3629  CB  SER P 224      -3.653  21.917 -20.340  0.00  0.00      PROA
ATOM   3630  HB1 SER P 224      -3.434  22.627 -21.165  0.00  0.00      PROA
ATOM   3631  HB2 SER P 224      -2.776  21.309 -20.030  0.00  0.00      PROA
ATOM   3632  OG  SER P 224      -4.070  22.797 -19.291  0.00  0.00      PROA
ATOM   3633  HG1 SER P 224      -4.649  23.516 -19.556  0.00  0.00      PROA
ATOM   3634  C   SER P 224      -4.861  20.423 -22.106  0.00  0.00      PROA
ATOM   3635  O   SER P 224      -5.168  20.988 -23.141  0.00  0.00      PROA
ATOM   3636  N   LYS P 225      -4.521  19.109 -22.220  0.00  0.00      PROA
ATOM   3637  HN  LYS P 225      -4.177  18.475 -21.531  0.00  0.00      PROA
ATOM   3638  CA  LYS P 225      -4.437  18.482 -23.511  0.00  0.00      PROA
ATOM   3639  HA  LYS P 225      -4.554  19.147 -24.354  0.00  0.00      PROA
ATOM   3640  CB  LYS P 225      -2.982  17.927 -23.735  0.00  0.00      PROA
ATOM   3641  HB1 LYS P 225      -3.006  17.605 -24.798  0.00  0.00      PROA
ATOM   3642  HB2 LYS P 225      -2.890  17.023 -23.096  0.00  0.00      PROA
ATOM   3643  CG  LYS P 225      -1.848  18.993 -23.559  0.00  0.00      PROA
ATOM   3644  HG1 LYS P 225      -0.825  18.593 -23.722  0.00  0.00      PROA
ATOM   3645  HG2 LYS P 225      -1.949  19.500 -22.575  0.00  0.00      PROA
ATOM   3646  CD  LYS P 225      -2.056  20.104 -24.631  0.00  0.00      PROA
ATOM   3647  HD1 LYS P 225      -2.871  20.755 -24.246  0.00  0.00      PROA
ATOM   3648  HD2 LYS P 225      -2.477  19.601 -25.528  0.00  0.00      PROA
ATOM   3649  CE  LYS P 225      -0.809  20.939 -24.875  0.00  0.00      PROA
ATOM   3650  HE1 LYS P 225       0.157  20.408 -25.014  0.00  0.00      PROA
ATOM   3651  HE2 LYS P 225      -0.577  21.608 -24.019  0.00  0.00      PROA
ATOM   3652  NZ  LYS P 225      -0.925  21.879 -26.059  0.00  0.00      PROA
ATOM   3653  HZ1 LYS P 225      -1.943  22.087 -26.116  0.00  0.00      PROA
ATOM   3654  HZ2 LYS P 225      -0.476  21.518 -26.925  0.00  0.00      PROA
ATOM   3655  HZ3 LYS P 225      -0.503  22.815 -25.893  0.00  0.00      PROA
ATOM   3656  C   LYS P 225      -5.543  17.519 -23.826  0.00  0.00      PROA
```

```
ATOM   3657  O   LYS P 225      -5.524  16.821 -24.818  0.00  0.00      PROA
ATOM   3658  N   LEU P 226      -6.593  17.508 -23.026  0.00  0.00      PROA
ATOM   3659  HN  LEU P 226      -6.547  18.105 -22.229  0.00  0.00      PROA
ATOM   3660  CA  LEU P 226      -7.676  16.541 -23.148  0.00  0.00      PROA
ATOM   3661  HA  LEU P 226      -7.218  15.566 -23.217  0.00  0.00      PROA
ATOM   3662  CB  LEU P 226      -8.509  16.661 -21.921  0.00  0.00      PROA
ATOM   3663  HB1 LEU P 226      -8.829  17.716 -21.787  0.00  0.00      PROA
ATOM   3664  HB2 LEU P 226      -7.828  16.523 -21.054  0.00  0.00      PROA
ATOM   3665  CG  LEU P 226      -9.815  15.754 -21.936  0.00  0.00      PROA
ATOM   3666  HG  LEU P 226     -10.468  16.029 -22.792  0.00  0.00      PROA
ATOM   3667  CD1 LEU P 226      -9.525  14.270 -22.001  0.00  0.00      PROA
ATOM   3668 HD11 LEU P 226     -10.522  13.818 -22.191  0.00  0.00      PROA
ATOM   3669 HD12 LEU P 226      -9.104  13.933 -21.029  0.00  0.00      PROA
ATOM   3670 HD13 LEU P 226      -8.969  13.928 -22.900  0.00  0.00      PROA
ATOM   3671  CD2 LEU P 226     -10.637  15.905 -20.685  0.00  0.00      PROA
ATOM   3672 HD21 LEU P 226     -11.386  15.095 -20.558  0.00  0.00      PROA
ATOM   3673 HD22 LEU P 226     -11.058  16.933 -20.675  0.00  0.00      PROA
ATOM   3674 HD23 LEU P 226      -9.982  15.798 -19.794  0.00  0.00      PROA
ATOM   3675  C   LEU P 226      -8.603  16.896 -24.300  0.00  0.00      PROA
ATOM   3676  O   LEU P 226      -9.217  16.053 -24.999  0.00  0.00      PROA
ATOM   3677  N   SER P 227      -8.584  18.134 -24.688  0.00  0.00      PROA
ATOM   3678  HN  SER P 227      -8.029  18.767 -24.153  0.00  0.00      PROA
ATOM   3679  CA  SER P 227      -9.296  18.733 -25.825  0.00  0.00      PROA
ATOM   3680  HA  SER P 227     -10.168  18.131 -26.033  0.00  0.00      PROA
ATOM   3681  CB  SER P 227      -9.656  20.257 -25.524  0.00  0.00      PROA
ATOM   3682  HB1 SER P 227     -10.190  20.162 -24.554  0.00  0.00      PROA
ATOM   3683  HB2 SER P 227     -10.293  20.673 -26.333  0.00  0.00      PROA
ATOM   3684  OG  SER P 227      -8.430  21.015 -25.353  0.00  0.00      PROA
ATOM   3685  HG1 SER P 227      -8.136  20.843 -24.455  0.00  0.00      PROA
ATOM   3686  C   SER P 227      -8.490  18.735 -27.107  0.00  0.00      PROA
ATOM   3687  O   SER P 227      -8.972  19.351 -28.027  0.00  0.00      PROA
ATOM   3688  N   HSD P 228      -7.341  18.064 -27.163  0.00  0.00      PROA
ATOM   3689  HN  HSD P 228      -7.107  17.650 -26.287  0.00  0.00      PROA
ATOM   3690  CA  HSD P 228      -6.539  18.020 -28.316  0.00  0.00      PROA
ATOM   3691  HA  HSD P 228      -7.146  17.883 -29.199  0.00  0.00      PROA
ATOM   3692  CB  HSD P 228      -5.788  19.403 -28.644  0.00  0.00      PROA
ATOM   3693  HB1 HSD P 228      -4.984  19.535 -27.889  0.00  0.00      PROA
ATOM   3694  HB2 HSD P 228      -6.499  20.250 -28.535  0.00  0.00      PROA
ATOM   3695  ND1 HSD P 228      -5.719  19.210 -31.253  0.00  0.00      PROA
ATOM   3696  HD1 HSD P 228      -6.463  18.585 -31.488  0.00  0.00      PROA
ATOM   3697  CG  HSD P 228      -5.138  19.440 -30.000  0.00  0.00      PROA
ATOM   3698  CE1 HSD P 228      -4.905  19.688 -32.157  0.00  0.00      PROA
ATOM   3699  HE1 HSD P 228      -5.216  19.594 -33.197  0.00  0.00      PROA
ATOM   3700  NE2 HSD P 228      -3.890  20.290 -31.674  0.00  0.00      PROA
ATOM   3701  CD2 HSD P 228      -3.969  20.132 -30.325  0.00  0.00      PROA
ATOM   3702  HD2 HSD P 228      -3.129  20.274 -29.656  0.00  0.00      PROA
ATOM   3703  C   HSD P 228      -5.524  16.927 -28.293  0.00  0.00      PROA
ATOM   3704  O   HSD P 228      -4.324  17.222 -28.219  0.00  0.00      PROA
ATOM   3705  N   SER P 229      -6.026  15.664 -28.269  0.00  0.00      PROA
ATOM   3706  HN  SER P 229      -7.005  15.480 -28.236  0.00  0.00      PROA
ATOM   3707  CA  SER P 229      -5.247  14.460 -28.446  0.00  0.00      PROA
ATOM   3708  HA  SER P 229      -4.230  14.737 -28.680  0.00  0.00      PROA
ATOM   3709  CB  SER P 229      -5.343  13.589 -27.171  0.00  0.00      PROA
ATOM   3710  HB1 SER P 229      -4.932  14.262 -26.389  0.00  0.00      PROA
ATOM   3711  HB2 SER P 229      -4.779  12.632 -27.154  0.00  0.00      PROA
ATOM   3712  OG  SER P 229      -6.725  13.192 -26.876  0.00  0.00      PROA
ATOM   3713  HG1 SER P 229      -7.241  13.994 -26.993  0.00  0.00      PROA
ATOM   3714  C   SER P 229      -5.538  13.647 -29.675  0.00  0.00      PROA
ATOM   3715  O   SER P 229      -6.676  13.734 -30.128  0.00  0.00      PROA
ATOM   3716  N   LYS P 230      -4.554  12.997 -30.242  0.00  0.00      PROA
ATOM   3717  HN  LYS P 230      -3.637  12.983 -29.853  0.00  0.00      PROA
```

ATOM 3718 CA LYS P 230 -4.653 12.470 -31.575 0.00 0.00 PROA
ATOM 3719 HA LYS P 230 -5.558 12.663 -32.132 0.00 0.00 PROA
ATOM 3720 CB LYS P 230 -3.534 12.990 -32.437 0.00 0.00 PROA
ATOM 3721 HB1 LYS P 230 -3.707 12.483 -33.411 0.00 0.00 PROA
ATOM 3722 HB2 LYS P 230 -2.537 12.698 -32.043 0.00 0.00 PROA
ATOM 3723 CG LYS P 230 -3.628 14.539 -32.648 0.00 0.00 PROA
ATOM 3724 HG1 LYS P 230 -2.842 14.739 -33.407 0.00 0.00 PROA
ATOM 3725 HG2 LYS P 230 -3.184 15.089 -31.792 0.00 0.00 PROA
ATOM 3726 CD LYS P 230 -4.994 14.997 -33.174 0.00 0.00 PROA
ATOM 3727 HD1 LYS P 230 -4.813 16.092 -33.216 0.00 0.00 PROA
ATOM 3728 HD2 LYS P 230 -5.738 14.863 -32.360 0.00 0.00 PROA
ATOM 3729 CE LYS P 230 -5.435 14.443 -34.507 0.00 0.00 PROA
ATOM 3730 HE1 LYS P 230 -5.358 13.336 -34.554 0.00 0.00 PROA
ATOM 3731 HE2 LYS P 230 -4.821 14.903 -35.311 0.00 0.00 PROA
ATOM 3732 NZ LYS P 230 -6.808 14.885 -34.810 0.00 0.00 PROA
ATOM 3733 HZ1 LYS P 230 -7.155 14.463 -35.695 0.00 0.00 PROA
ATOM 3734 HZ2 LYS P 230 -6.930 15.916 -34.879 0.00 0.00 PROA
ATOM 3735 HZ3 LYS P 230 -7.453 14.626 -34.036 0.00 0.00 PROA
ATOM 3736 C LYS P 230 -4.464 10.931 -31.474 0.00 0.00 PROA
ATOM 3737 O LYS P 230 -3.507 10.408 -30.939 0.00 0.00 PROA
ATOM 3738 N GLY P 231 -5.378 10.126 -32.105 0.00 0.00 PROA
ATOM 3739 HN GLY P 231 -6.118 10.576 -32.598 0.00 0.00 PROA
ATOM 3740 CA GLY P 231 -5.417 8.700 -32.156 0.00 0.00 PROA
ATOM 3741 HA1 GLY P 231 -4.553 8.219 -31.723 0.00 0.00 PROA
ATOM 3742 HA2 GLY P 231 -5.565 8.428 -33.191 0.00 0.00 PROA
ATOM 3743 C GLY P 231 -6.590 8.222 -31.321 0.00 0.00 PROA
ATOM 3744 O GLY P 231 -6.955 8.768 -30.315 0.00 0.00 PROA
ATOM 3745 N HSD P 232 -7.371 7.204 -31.806 0.00 0.00 PROA
ATOM 3746 HN HSD P 232 -7.008 6.706 -32.590 0.00 0.00 PROA
ATOM 3747 CA HSD P 232 -8.656 6.854 -31.232 0.00 0.00 PROA
ATOM 3748 HA HSD P 232 -9.233 7.767 -31.190 0.00 0.00 PROA
ATOM 3749 CB HSD P 232 -9.343 5.813 -32.173 0.00 0.00 PROA
ATOM 3750 HB1 HSD P 232 -8.908 4.791 -32.207 0.00 0.00 PROA
ATOM 3751 HB2 HSD P 232 -9.351 6.230 -33.203 0.00 0.00 PROA
ATOM 3752 ND1 HSD P 232 -11.766 6.642 -32.045 0.00 0.00 PROA
ATOM 3753 HD1 HSD P 232 -11.548 7.509 -32.491 0.00 0.00 PROA
ATOM 3754 CG HSD P 232 -10.853 5.597 -31.870 0.00 0.00 PROA
ATOM 3755 CE1 HSD P 232 -13.023 6.078 -32.046 0.00 0.00 PROA
ATOM 3756 HE1 HSD P 232 -13.954 6.644 -32.099 0.00 0.00 PROA
ATOM 3757 NE2 HSD P 232 -12.930 4.771 -31.855 0.00 0.00 PROA
ATOM 3758 CD2 HSD P 232 -11.594 4.509 -31.769 0.00 0.00 PROA
ATOM 3759 HD2 HSD P 232 -11.315 3.467 -31.677 0.00 0.00 PROA
ATOM 3760 C HSD P 232 -8.611 6.195 -29.880 0.00 0.00 PROA
ATOM 3761 O HSD P 232 -9.638 6.132 -29.175 0.00 0.00 PROA
ATOM 3762 N GLN P 233 -7.445 5.769 -29.469 0.00 0.00 PROA
ATOM 3763 HN GLN P 233 -6.599 5.804 -29.995 0.00 0.00 PROA
ATOM 3764 CA GLN P 233 -7.300 5.213 -28.133 0.00 0.00 PROA
ATOM 3765 HA GLN P 233 -8.215 4.696 -27.881 0.00 0.00 PROA
ATOM 3766 CB GLN P 233 -6.252 4.104 -28.155 0.00 0.00 PROA
ATOM 3767 HB1 GLN P 233 -5.985 3.804 -27.120 0.00 0.00 PROA
ATOM 3768 HB2 GLN P 233 -5.314 4.588 -28.503 0.00 0.00 PROA
ATOM 3769 CG GLN P 233 -6.497 2.815 -29.073 0.00 0.00 PROA
ATOM 3770 HG1 GLN P 233 -5.784 1.963 -29.100 0.00 0.00 PROA
ATOM 3771 HG2 GLN P 233 -6.601 3.304 -30.065 0.00 0.00 PROA
ATOM 3772 CD GLN P 233 -7.919 2.274 -28.957 0.00 0.00 PROA
ATOM 3773 OE1 GLN P 233 -8.814 2.413 -29.838 0.00 0.00 PROA
ATOM 3774 NE2 GLN P 233 -8.244 1.569 -27.848 0.00 0.00 PROA
ATOM 3775 HE21 GLN P 233 -9.207 1.461 -27.601 0.00 0.00 PROA
ATOM 3776 HE22 GLN P 233 -7.544 1.465 -27.142 0.00 0.00 PROA
ATOM 3777 C GLN P 233 -7.015 6.192 -27.025 0.00 0.00 PROA
ATOM 3778 O GLN P 233 -7.256 5.952 -25.854 0.00 0.00 PROA

ATOM 3779 N LYS P 234 -6.651 7.501 -27.306 0.00 0.00 PROA
ATOM 3780 HN LYS P 234 -6.677 7.791 -28.259 0.00 0.00 PROA
ATOM 3781 CA LYS P 234 -6.237 8.479 -26.316 0.00 0.00 PROA
ATOM 3782 HA LYS P 234 -5.368 8.070 -25.822 0.00 0.00 PROA
ATOM 3783 CB LYS P 234 -5.752 9.852 -26.864 0.00 0.00 PROA
ATOM 3784 HB1 LYS P 234 -5.421 10.518 -26.039 0.00 0.00 PROA
ATOM 3785 HB2 LYS P 234 -6.500 10.474 -27.398 0.00 0.00 PROA
ATOM 3786 CG LYS P 234 -4.535 9.814 -27.768 0.00 0.00 PROA
ATOM 3787 HG1 LYS P 234 -4.334 10.831 -28.166 0.00 0.00 PROA
ATOM 3788 HG2 LYS P 234 -4.731 9.141 -28.631 0.00 0.00 PROA
ATOM 3789 CD LYS P 234 -3.219 9.392 -27.134 0.00 0.00 PROA
ATOM 3790 HD1 LYS P 234 -3.489 8.615 -26.387 0.00 0.00 PROA
ATOM 3791 HD2 LYS P 234 -2.798 10.238 -26.548 0.00 0.00 PROA
ATOM 3792 CE LYS P 234 -2.113 8.664 -27.991 0.00 0.00 PROA
ATOM 3793 HE1 LYS P 234 -2.538 7.854 -28.621 0.00 0.00 PROA
ATOM 3794 HE2 LYS P 234 -1.348 8.168 -27.355 0.00 0.00 PROA
ATOM 3795 NZ LYS P 234 -1.493 9.698 -28.854 0.00 0.00 PROA
ATOM 3796 HZ1 LYS P 234 -0.718 10.240 -28.423 0.00 0.00 PROA
ATOM 3797 HZ2 LYS P 234 -2.229 10.243 -29.347 0.00 0.00 PROA
ATOM 3798 HZ3 LYS P 234 -0.911 9.186 -29.547 0.00 0.00 PROA
ATOM 3799 C LYS P 234 -7.219 8.715 -25.204 0.00 0.00 PROA
ATOM 3800 O LYS P 234 -6.867 8.603 -24.003 0.00 0.00 PROA
ATOM 3801 N ARG P 235 -8.525 8.966 -25.515 0.00 0.00 PROA
ATOM 3802 HN ARG P 235 -8.820 9.021 -26.466 0.00 0.00 PROA
ATOM 3803 CA ARG P 235 -9.496 9.218 -24.495 0.00 0.00 PROA
ATOM 3804 HA ARG P 235 -8.977 9.811 -23.756 0.00 0.00 PROA
ATOM 3805 CB ARG P 235 -10.694 10.157 -24.899 0.00 0.00 PROA
ATOM 3806 HB1 ARG P 235 -11.377 10.289 -24.033 0.00 0.00 PROA
ATOM 3807 HB2 ARG P 235 -11.261 9.604 -25.678 0.00 0.00 PROA
ATOM 3808 CG ARG P 235 -10.109 11.388 -25.503 0.00 0.00 PROA
ATOM 3809 HG1 ARG P 235 -9.473 11.275 -26.406 0.00 0.00 PROA
ATOM 3810 HG2 ARG P 235 -9.558 12.002 -24.758 0.00 0.00 PROA
ATOM 3811 CD ARG P 235 -11.227 12.399 -26.032 0.00 0.00 PROA
ATOM 3812 HD1 ARG P 235 -12.003 11.888 -26.642 0.00 0.00 PROA
ATOM 3813 HD2 ARG P 235 -10.761 13.229 -26.605 0.00 0.00 PROA
ATOM 3814 NE ARG P 235 -12.029 12.865 -24.861 0.00 0.00 PROA
ATOM 3815 HE ARG P 235 -12.518 12.190 -24.309 0.00 0.00 PROA
ATOM 3816 CZ ARG P 235 -12.420 14.142 -24.641 0.00 0.00 PROA
ATOM 3817 NH1 ARG P 235 -11.919 15.123 -25.298 0.00 0.00 PROA
ATOM 3818 HH11 ARG P 235 -11.452 14.987 -26.172 0.00 0.00 PROA
ATOM 3819 HH12 ARG P 235 -12.475 15.950 -25.217 0.00 0.00 PROA
ATOM 3820 NH2 ARG P 235 -13.258 14.407 -23.643 0.00 0.00 PROA
ATOM 3821 HH21 ARG P 235 -13.555 13.673 -23.033 0.00 0.00 PROA
ATOM 3822 HH22 ARG P 235 -13.268 15.342 -23.289 0.00 0.00 PROA
ATOM 3823 C ARG P 235 -10.104 7.968 -23.875 0.00 0.00 PROA
ATOM 3824 O ARG P 235 -10.564 7.929 -22.742 0.00 0.00 PROA
ATOM 3825 N LYS P 236 -10.046 6.891 -24.654 0.00 0.00 PROA
ATOM 3826 HN LYS P 236 -9.620 6.905 -25.556 0.00 0.00 PROA
ATOM 3827 CA LYS P 236 -10.505 5.587 -24.066 0.00 0.00 PROA
ATOM 3828 HA LYS P 236 -11.462 5.736 -23.586 0.00 0.00 PROA
ATOM 3829 CB LYS P 236 -10.855 4.506 -25.195 0.00 0.00 PROA
ATOM 3830 HB1 LYS P 236 -10.649 3.468 -24.857 0.00 0.00 PROA
ATOM 3831 HB2 LYS P 236 -10.179 4.491 -26.076 0.00 0.00 PROA
ATOM 3832 CG LYS P 236 -12.160 4.603 -25.899 0.00 0.00 PROA
ATOM 3833 HG1 LYS P 236 -12.233 5.703 -26.037 0.00 0.00 PROA
ATOM 3834 HG2 LYS P 236 -13.021 4.311 -25.261 0.00 0.00 PROA
ATOM 3835 CD LYS P 236 -12.167 3.861 -27.311 0.00 0.00 PROA
ATOM 3836 HD1 LYS P 236 -11.297 3.179 -27.422 0.00 0.00 PROA
ATOM 3837 HD2 LYS P 236 -12.257 4.672 -28.065 0.00 0.00 PROA
ATOM 3838 CE LYS P 236 -13.395 2.986 -27.656 0.00 0.00 PROA
ATOM 3839 HE1 LYS P 236 -14.289 3.579 -27.370 0.00 0.00 PROA

ATOM	3840	HE2	LYS	P	236	-13.403	2.026	-27.097	0.00	0.00	PROA
ATOM	3841	NZ	LYS	P	236	-13.547	2.683	-29.165	0.00	0.00	PROA
ATOM	3842	HZ1	LYS	P	236	-13.829	3.540	-29.681	0.00	0.00	PROA
ATOM	3843	HZ2	LYS	P	236	-14.327	1.996	-29.210	0.00	0.00	PROA
ATOM	3844	HZ3	LYS	P	236	-12.713	2.190	-29.542	0.00	0.00	PROA
ATOM	3845	C	LYS	P	236	-9.659	5.008	-23.067	0.00	0.00	PROA
ATOM	3846	O	LYS	P	236	-10.204	4.482	-22.074	0.00	0.00	PROA
ATOM	3847	N	ALA	P	237	-8.329	4.999	-23.236	0.00	0.00	PROA
ATOM	3848	HN	ALA	P	237	-7.894	5.316	-24.075	0.00	0.00	PROA
ATOM	3849	CA	ALA	P	237	-7.460	4.393	-22.293	0.00	0.00	PROA
ATOM	3850	HA	ALA	P	237	-7.892	3.452	-21.985	0.00	0.00	PROA
ATOM	3851	CB	ALA	P	237	-6.135	4.182	-22.969	0.00	0.00	PROA
ATOM	3852	HB1	ALA	P	237	-5.735	4.990	-23.619	0.00	0.00	PROA
ATOM	3853	HB2	ALA	P	237	-6.383	3.511	-23.819	0.00	0.00	PROA
ATOM	3854	HB3	ALA	P	237	-5.323	3.679	-22.401	0.00	0.00	PROA
ATOM	3855	C	ALA	P	237	-7.172	5.286	-21.107	0.00	0.00	PROA
ATOM	3856	O	ALA	P	237	-6.737	4.883	-19.952	0.00	0.00	PROA
ATOM	3857	N	LEU	P	238	-7.355	6.575	-21.277	0.00	0.00	PROA
ATOM	3858	HN	LEU	P	238	-7.619	6.890	-22.186	0.00	0.00	PROA
ATOM	3859	CA	LEU	P	238	-7.399	7.536	-20.156	0.00	0.00	PROA
ATOM	3860	HA	LEU	P	238	-6.513	7.575	-19.539	0.00	0.00	PROA
ATOM	3861	CB	LEU	P	238	-7.667	8.910	-20.873	0.00	0.00	PROA
ATOM	3862	HB1	LEU	P	238	-8.638	8.944	-21.412	0.00	0.00	PROA
ATOM	3863	HB2	LEU	P	238	-6.813	9.058	-21.568	0.00	0.00	PROA
ATOM	3864	CG	LEU	P	238	-7.626	10.063	-19.879	0.00	0.00	PROA
ATOM	3865	HG	LEU	P	238	-8.521	10.099	-19.222	0.00	0.00	PROA
ATOM	3866	CD1	LEU	P	238	-6.402	10.278	-18.938	0.00	0.00	PROA
ATOM	3867	HD11	LEU	P	238	-6.380	9.503	-18.142	0.00	0.00	PROA
ATOM	3868	HD12	LEU	P	238	-6.441	11.321	-18.556	0.00	0.00	PROA
ATOM	3869	HD13	LEU	P	238	-5.422	10.324	-19.459	0.00	0.00	PROA
ATOM	3870	CD2	LEU	P	238	-7.885	11.309	-20.778	0.00	0.00	PROA
ATOM	3871	HD21	LEU	P	238	-8.807	11.087	-21.357	0.00	0.00	PROA
ATOM	3872	HD22	LEU	P	238	-7.053	11.461	-21.498	0.00	0.00	PROA
ATOM	3873	HD23	LEU	P	238	-7.956	12.308	-20.297	0.00	0.00	PROA
ATOM	3874	C	LEU	P	238	-8.515	7.322	-19.170	0.00	0.00	PROA
ATOM	3875	O	LEU	P	238	-8.324	7.386	-17.948	0.00	0.00	PROA
ATOM	3876	N	LYS	P	239	-9.717	6.951	-19.665	0.00	0.00	PROA
ATOM	3877	HN	LYS	P	239	-9.777	6.892	-20.658	0.00	0.00	PROA
ATOM	3878	CA	LYS	P	239	-10.873	6.583	-18.883	0.00	0.00	PROA
ATOM	3879	HA	LYS	P	239	-10.991	7.408	-18.196	0.00	0.00	PROA
ATOM	3880	CB	LYS	P	239	-12.148	6.293	-19.788	0.00	0.00	PROA
ATOM	3881	HB1	LYS	P	239	-12.837	5.600	-19.260	0.00	0.00	PROA
ATOM	3882	HB2	LYS	P	239	-11.704	5.734	-20.639	0.00	0.00	PROA
ATOM	3883	CG	LYS	P	239	-12.887	7.528	-20.304	0.00	0.00	PROA
ATOM	3884	HG1	LYS	P	239	-12.141	8.210	-20.765	0.00	0.00	PROA
ATOM	3885	HG2	LYS	P	239	-13.226	8.017	-19.366	0.00	0.00	PROA
ATOM	3886	CD	LYS	P	239	-14.119	7.301	-21.222	0.00	0.00	PROA
ATOM	3887	HD1	LYS	P	239	-14.429	8.364	-21.306	0.00	0.00	PROA
ATOM	3888	HD2	LYS	P	239	-14.859	6.768	-20.587	0.00	0.00	PROA
ATOM	3889	CE	LYS	P	239	-13.907	6.631	-22.542	0.00	0.00	PROA
ATOM	3890	HE1	LYS	P	239	-13.685	5.558	-22.358	0.00	0.00	PROA
ATOM	3891	HE2	LYS	P	239	-13.123	7.116	-23.163	0.00	0.00	PROA
ATOM	3892	NZ	LYS	P	239	-15.185	6.525	-23.314	0.00	0.00	PROA
ATOM	3893	HZ1	LYS	P	239	-15.070	5.720	-23.962	0.00	0.00	PROA
ATOM	3894	HZ2	LYS	P	239	-15.241	7.442	-23.802	0.00	0.00	PROA
ATOM	3895	HZ3	LYS	P	239	-16.097	6.438	-22.822	0.00	0.00	PROA
ATOM	3896	C	LYS	P	239	-10.625	5.343	-17.969	0.00	0.00	PROA
ATOM	3897	O	LYS	P	239	-10.985	5.413	-16.817	0.00	0.00	PROA
ATOM	3898	N	THR	P	240	-9.959	4.323	-18.570	0.00	0.00	PROA
ATOM	3899	HN	THR	P	240	-9.718	4.276	-19.536	0.00	0.00	PROA
ATOM	3900	CA	THR	P	240	-9.531	3.088	-17.844	0.00	0.00	PROA

ATOM	3901	HA	THR	P	240	-10.383	2.598	-17.397	0.00	0.00	PROA
ATOM	3902	CB	THR	P	240	-8.913	2.091	-18.846	0.00	0.00	PROA
ATOM	3903	HB	THR	P	240	-8.060	2.556	-19.386	0.00	0.00	PROA
ATOM	3904	OG1	THR	P	240	-9.884	1.673	-19.780	0.00	0.00	PROA
ATOM	3905	HG1	THR	P	240	-10.601	1.176	-19.380	0.00	0.00	PROA
ATOM	3906	CG2	THR	P	240	-8.414	0.842	-18.092	0.00	0.00	PROA
ATOM	3907	HG21	THR	P	240	-7.537	1.132	-17.474	0.00	0.00	PROA
ATOM	3908	HG22	THR	P	240	-7.995	0.079	-18.783	0.00	0.00	PROA
ATOM	3909	HG23	THR	P	240	-9.135	0.361	-17.398	0.00	0.00	PROA
ATOM	3910	C	THR	P	240	-8.519	3.494	-16.768	0.00	0.00	PROA
ATOM	3911	O	THR	P	240	-8.590	3.070	-15.605	0.00	0.00	PROA
ATOM	3912	N	THR	P	241	-7.548	4.443	-17.037	0.00	0.00	PROA
ATOM	3913	HN	THR	P	241	-7.306	4.651	-17.982	0.00	0.00	PROA
ATOM	3914	CA	THR	P	241	-6.563	4.920	-16.087	0.00	0.00	PROA
ATOM	3915	HA	THR	P	241	-6.073	4.042	-15.692	0.00	0.00	PROA
ATOM	3916	CB	THR	P	241	-5.395	5.799	-16.594	0.00	0.00	PROA
ATOM	3917	HB	THR	P	241	-5.858	6.757	-16.915	0.00	0.00	PROA
ATOM	3918	OG1	THR	P	241	-4.800	5.284	-17.796	0.00	0.00	PROA
ATOM	3919	HG1	THR	P	241	-5.061	4.360	-17.792	0.00	0.00	PROA
ATOM	3920	CG2	THR	P	241	-4.177	5.881	-15.635	0.00	0.00	PROA
ATOM	3921	HG21	THR	P	241	-3.364	6.563	-15.963	0.00	0.00	PROA
ATOM	3922	HG22	THR	P	241	-3.717	4.874	-15.535	0.00	0.00	PROA
ATOM	3923	HG23	THR	P	241	-4.513	6.184	-14.621	0.00	0.00	PROA
ATOM	3924	C	THR	P	241	-7.225	5.663	-14.946	0.00	0.00	PROA
ATOM	3925	O	THR	P	241	-6.821	5.471	-13.856	0.00	0.00	PROA
ATOM	3926	N	VAL	P	242	-8.226	6.549	-15.231	0.00	0.00	PROA
ATOM	3927	HN	VAL	P	242	-8.386	6.760	-16.193	0.00	0.00	PROA
ATOM	3928	CA	VAL	P	242	-9.097	7.309	-14.298	0.00	0.00	PROA
ATOM	3929	HA	VAL	P	242	-8.519	8.046	-13.761	0.00	0.00	PROA
ATOM	3930	CB	VAL	P	242	-10.110	8.171	-15.077	0.00	0.00	PROA
ATOM	3931	HB	VAL	P	242	-10.322	7.633	-16.025	0.00	0.00	PROA
ATOM	3932	CG1	VAL	P	242	-11.515	8.336	-14.511	0.00	0.00	PROA
ATOM	3933	HG11	VAL	P	242	-12.235	8.979	-15.061	0.00	0.00	PROA
ATOM	3934	HG12	VAL	P	242	-11.441	8.958	-13.594	0.00	0.00	PROA
ATOM	3935	HG13	VAL	P	242	-12.050	7.378	-14.334	0.00	0.00	PROA
ATOM	3936	CG2	VAL	P	242	-9.526	9.513	-15.531	0.00	0.00	PROA
ATOM	3937	HG21	VAL	P	242	-10.144	10.083	-16.258	0.00	0.00	PROA
ATOM	3938	HG22	VAL	P	242	-8.586	9.282	-16.076	0.00	0.00	PROA
ATOM	3939	HG23	VAL	P	242	-9.241	10.134	-14.655	0.00	0.00	PROA
ATOM	3940	C	VAL	P	242	-9.656	6.475	-13.120	0.00	0.00	PROA
ATOM	3941	O	VAL	P	242	-9.468	6.816	-11.927	0.00	0.00	PROA
ATOM	3942	N	ILE	P	243	-10.359	5.415	-13.485	0.00	0.00	PROA
ATOM	3943	HN	ILE	P	243	-10.389	5.449	-14.481	0.00	0.00	PROA
ATOM	3944	CA	ILE	P	243	-10.847	4.323	-12.582	0.00	0.00	PROA
ATOM	3945	HA	ILE	P	243	-11.607	4.831	-12.007	0.00	0.00	PROA
ATOM	3946	CB	ILE	P	243	-11.597	3.288	-13.495	0.00	0.00	PROA
ATOM	3947	HB	ILE	P	243	-10.888	2.877	-14.245	0.00	0.00	PROA
ATOM	3948	CG2	ILE	P	243	-12.191	2.067	-12.813	0.00	0.00	PROA
ATOM	3949	HG21	ILE	P	243	-13.201	2.449	-12.555	0.00	0.00	PROA
ATOM	3950	HG22	ILE	P	243	-11.616	1.661	-11.954	0.00	0.00	PROA
ATOM	3951	HG23	ILE	P	243	-12.270	1.293	-13.607	0.00	0.00	PROA
ATOM	3952	CG1	ILE	P	243	-12.653	3.986	-14.383	0.00	0.00	PROA
ATOM	3953	HG11	ILE	P	243	-13.314	3.344	-15.004	0.00	0.00	PROA
ATOM	3954	HG12	ILE	P	243	-12.149	4.668	-15.101	0.00	0.00	PROA
ATOM	3955	CD	ILE	P	243	-13.636	4.775	-13.568	0.00	0.00	PROA
ATOM	3956	HD1	ILE	P	243	-13.610	4.379	-12.531	0.00	0.00	PROA
ATOM	3957	HD2	ILE	P	243	-14.625	4.922	-14.053	0.00	0.00	PROA
ATOM	3958	HD3	ILE	P	243	-13.166	5.768	-13.409	0.00	0.00	PROA
ATOM	3959	C	ILE	P	243	-9.895	3.713	-11.617	0.00	0.00	PROA
ATOM	3960	O	ILE	P	243	-10.267	3.376	-10.462	0.00	0.00	PROA
ATOM	3961	N	LEU	P	244	-8.665	3.532	-12.077	0.00	0.00	PROA

ATOM	3962	HN	LEU	P	244	-8.558	3.824	-13.024	0.00	0.00	PROA
ATOM	3963	CA	LEU	P	244	-7.592	3.244	-11.131	0.00	0.00	PROA
ATOM	3964	HA	LEU	P	244	-8.039	2.516	-10.471	0.00	0.00	PROA
ATOM	3965	CB	LEU	P	244	-6.336	2.688	-11.853	0.00	0.00	PROA
ATOM	3966	HB1	LEU	P	244	-6.045	3.281	-12.746	0.00	0.00	PROA
ATOM	3967	HB2	LEU	P	244	-6.737	1.721	-12.225	0.00	0.00	PROA
ATOM	3968	CG	LEU	P	244	-5.061	2.507	-10.977	0.00	0.00	PROA
ATOM	3969	HG	LEU	P	244	-4.701	3.450	-10.512	0.00	0.00	PROA
ATOM	3970	CD1	LEU	P	244	-5.219	1.445	-9.856	0.00	0.00	PROA
ATOM	3971	HD11	LEU	P	244	-5.846	0.641	-10.296	0.00	0.00	PROA
ATOM	3972	HD12	LEU	P	244	-5.648	1.839	-8.909	0.00	0.00	PROA
ATOM	3973	HD13	LEU	P	244	-4.164	1.210	-9.602	0.00	0.00	PROA
ATOM	3974	CD2	LEU	P	244	-3.957	2.086	-11.911	0.00	0.00	PROA
ATOM	3975	HD21	LEU	P	244	-3.036	1.794	-11.364	0.00	0.00	PROA
ATOM	3976	HD22	LEU	P	244	-3.667	2.780	-12.729	0.00	0.00	PROA
ATOM	3977	HD23	LEU	P	244	-4.209	1.122	-12.401	0.00	0.00	PROA
ATOM	3978	C	LEU	P	244	-7.195	4.379	-10.145	0.00	0.00	PROA
ATOM	3979	O	LEU	P	244	-7.106	4.027	-8.981	0.00	0.00	PROA
ATOM	3980	N	ILE	P	245	-7.208	5.709	-10.473	0.00	0.00	PROA
ATOM	3981	HN	ILE	P	245	-7.425	6.026	-11.394	0.00	0.00	PROA
ATOM	3982	CA	ILE	P	245	-7.214	6.735	-9.507	0.00	0.00	PROA
ATOM	3983	HA	ILE	P	245	-6.412	6.618	-8.793	0.00	0.00	PROA
ATOM	3984	CB	ILE	P	245	-6.855	8.103	-10.229	0.00	0.00	PROA
ATOM	3985	HB	ILE	P	245	-7.537	8.162	-11.104	0.00	0.00	PROA
ATOM	3986	CG2	ILE	P	245	-7.286	9.277	-9.383	0.00	0.00	PROA
ATOM	3987	HG21	ILE	P	245	-6.734	9.284	-8.419	0.00	0.00	PROA
ATOM	3988	HG22	ILE	P	245	-8.388	9.214	-9.254	0.00	0.00	PROA
ATOM	3989	HG23	ILE	P	245	-7.060	10.242	-9.885	0.00	0.00	PROA
ATOM	3990	CG1	ILE	P	245	-5.447	8.323	-10.934	0.00	0.00	PROA
ATOM	3991	HG11	ILE	P	245	-5.424	9.350	-11.359	0.00	0.00	PROA
ATOM	3992	HG12	ILE	P	245	-5.373	7.693	-11.846	0.00	0.00	PROA
ATOM	3993	CD	ILE	P	245	-4.253	8.111	-9.950	0.00	0.00	PROA
ATOM	3994	HD1	ILE	P	245	-4.495	8.637	-9.001	0.00	0.00	PROA
ATOM	3995	HD2	ILE	P	245	-3.322	8.583	-10.331	0.00	0.00	PROA
ATOM	3996	HD3	ILE	P	245	-3.997	7.044	-9.780	0.00	0.00	PROA
ATOM	3997	C	ILE	P	245	-8.364	6.717	-8.531	0.00	0.00	PROA
ATOM	3998	O	ILE	P	245	-8.264	6.798	-7.334	0.00	0.00	PROA
ATOM	3999	N	LEU	P	246	-9.601	6.576	-9.051	0.00	0.00	PROA
ATOM	4000	HN	LEU	P	246	-9.690	6.367	-10.022	0.00	0.00	PROA
ATOM	4001	CA	LEU	P	246	-10.819	6.708	-8.391	0.00	0.00	PROA
ATOM	4002	HA	LEU	P	246	-10.891	7.608	-7.798	0.00	0.00	PROA
ATOM	4003	CB	LEU	P	246	-12.053	6.783	-9.431	0.00	0.00	PROA
ATOM	4004	HB1	LEU	P	246	-12.203	5.760	-9.839	0.00	0.00	PROA
ATOM	4005	HB2	LEU	P	246	-11.779	7.414	-10.304	0.00	0.00	PROA
ATOM	4006	CG	LEU	P	246	-13.349	7.467	-8.939	0.00	0.00	PROA
ATOM	4007	HG	LEU	P	246	-13.606	6.971	-7.979	0.00	0.00	PROA
ATOM	4008	CD1	LEU	P	246	-13.223	9.016	-8.722	0.00	0.00	PROA
ATOM	4009	HD11	LEU	P	246	-12.757	9.137	-7.721	0.00	0.00	PROA
ATOM	4010	HD12	LEU	P	246	-14.191	9.560	-8.724	0.00	0.00	PROA
ATOM	4011	HD13	LEU	P	246	-12.539	9.449	-9.483	0.00	0.00	PROA
ATOM	4012	CD2	LEU	P	246	-14.576	7.064	-9.820	0.00	0.00	PROA
ATOM	4013	HD21	LEU	P	246	-15.495	7.632	-9.562	0.00	0.00	PROA
ATOM	4014	HD22	LEU	P	246	-14.726	6.000	-9.538	0.00	0.00	PROA
ATOM	4015	HD23	LEU	P	246	-14.261	7.391	-10.834	0.00	0.00	PROA
ATOM	4016	C	LEU	P	246	-11.065	5.590	-7.428	0.00	0.00	PROA
ATOM	4017	O	LEU	P	246	-11.480	5.744	-6.298	0.00	0.00	PROA
ATOM	4018	N	ALA	P	247	-10.721	4.339	-7.855	0.00	0.00	PROA
ATOM	4019	HN	ALA	P	247	-10.328	4.203	-8.761	0.00	0.00	PROA
ATOM	4020	CA	ALA	P	247	-10.579	3.221	-6.934	0.00	0.00	PROA
ATOM	4021	HA	ALA	P	247	-11.515	3.162	-6.398	0.00	0.00	PROA
ATOM	4022	CB	ALA	P	247	-10.402	1.943	-7.748	0.00	0.00	PROA

ATOM	4023	HB1	ALA	P	247	-10.211	1.090	-7.062	0.00	0.00	PROA
ATOM	4024	HB2	ALA	P	247	-9.494	2.013	-8.384	0.00	0.00	PROA
ATOM	4025	HB3	ALA	P	247	-11.319	1.863	-8.371	0.00	0.00	PROA
ATOM	4026	C	ALA	P	247	-9.465	3.428	-5.882	0.00	0.00	PROA
ATOM	4027	O	ALA	P	247	-9.668	3.115	-4.661	0.00	0.00	PROA
ATOM	4028	N	PHE	P	248	-8.345	3.990	-6.246	0.00	0.00	PROA
ATOM	4029	HN	PHE	P	248	-8.195	4.439	-7.124	0.00	0.00	PROA
ATOM	4030	CA	PHE	P	248	-7.190	4.249	-5.344	0.00	0.00	PROA
ATOM	4031	HA	PHE	P	248	-6.984	3.367	-4.756	0.00	0.00	PROA
ATOM	4032	CB	PHE	P	248	-6.007	4.704	-6.112	0.00	0.00	PROA
ATOM	4033	HB1	PHE	P	248	-6.304	5.551	-6.766	0.00	0.00	PROA
ATOM	4034	HB2	PHE	P	248	-5.615	3.845	-6.698	0.00	0.00	PROA
ATOM	4035	CG	PHE	P	248	-4.806	5.183	-5.331	0.00	0.00	PROA
ATOM	4036	CD1	PHE	P	248	-4.293	6.499	-5.467	0.00	0.00	PROA
ATOM	4037	HD1	PHE	P	248	-4.878	7.115	-6.134	0.00	0.00	PROA
ATOM	4038	CE1	PHE	P	248	-3.237	7.013	-4.778	0.00	0.00	PROA
ATOM	4039	HE1	PHE	P	248	-3.034	8.065	-4.914	0.00	0.00	PROA
ATOM	4040	CZ	PHE	P	248	-2.464	6.055	-3.974	0.00	0.00	PROA
ATOM	4041	HZ	PHE	P	248	-1.633	6.484	-3.434	0.00	0.00	PROA
ATOM	4042	CD2	PHE	P	248	-4.137	4.335	-4.479	0.00	0.00	PROA
ATOM	4043	HD2	PHE	P	248	-4.497	3.328	-4.323	0.00	0.00	PROA
ATOM	4044	CE2	PHE	P	248	-2.878	4.700	-3.903	0.00	0.00	PROA
ATOM	4045	HE2	PHE	P	248	-2.383	3.952	-3.300	0.00	0.00	PROA
ATOM	4046	C	PHE	P	248	-7.637	5.222	-4.291	0.00	0.00	PROA
ATOM	4047	O	PHE	P	248	-7.340	5.100	-3.092	0.00	0.00	PROA
ATOM	4048	N	PHE	P	249	-8.622	6.178	-4.621	0.00	0.00	PROA
ATOM	4049	HN	PHE	P	249	-9.020	6.142	-5.535	0.00	0.00	PROA
ATOM	4050	CA	PHE	P	249	-9.316	7.034	-3.668	0.00	0.00	PROA
ATOM	4051	HA	PHE	P	249	-8.576	7.606	-3.129	0.00	0.00	PROA
ATOM	4052	CB	PHE	P	249	-10.125	8.185	-4.353	0.00	0.00	PROA
ATOM	4053	HB1	PHE	P	249	-10.882	8.651	-3.686	0.00	0.00	PROA
ATOM	4054	HB2	PHE	P	249	-10.717	7.783	-5.203	0.00	0.00	PROA
ATOM	4055	CG	PHE	P	249	-9.311	9.369	-4.853	0.00	0.00	PROA
ATOM	4056	CD1	PHE	P	249	-8.030	9.356	-5.394	0.00	0.00	PROA
ATOM	4057	HD1	PHE	P	249	-7.530	8.412	-5.239	0.00	0.00	PROA
ATOM	4058	CE1	PHE	P	249	-7.539	10.442	-6.010	0.00	0.00	PROA
ATOM	4059	HE1	PHE	P	249	-6.580	10.388	-6.504	0.00	0.00	PROA
ATOM	4060	CZ	PHE	P	249	-8.280	11.636	-6.189	0.00	0.00	PROA
ATOM	4061	HZ	PHE	P	249	-7.873	12.475	-6.734	0.00	0.00	PROA
ATOM	4062	CD2	PHE	P	249	-10.056	10.575	-4.948	0.00	0.00	PROA
ATOM	4063	HD2	PHE	P	249	-11.083	10.671	-4.627	0.00	0.00	PROA
ATOM	4064	CE2	PHE	P	249	-9.547	11.708	-5.568	0.00	0.00	PROA
ATOM	4065	HE2	PHE	P	249	-9.992	12.685	-5.449	0.00	0.00	PROA
ATOM	4066	C	PHE	P	249	-10.188	6.357	-2.605	0.00	0.00	PROA
ATOM	4067	O	PHE	P	249	-10.251	6.867	-1.480	0.00	0.00	PROA
ATOM	4068	N	ALA	P	250	-10.734	5.160	-2.909	0.00	0.00	PROA
ATOM	4069	HN	ALA	P	250	-10.630	4.777	-3.824	0.00	0.00	PROA
ATOM	4070	CA	ALA	P	250	-11.541	4.334	-1.992	0.00	0.00	PROA
ATOM	4071	HA	ALA	P	250	-12.050	4.956	-1.271	0.00	0.00	PROA
ATOM	4072	CB	ALA	P	250	-12.594	3.442	-2.766	0.00	0.00	PROA
ATOM	4073	HB1	ALA	P	250	-13.270	4.210	-3.199	0.00	0.00	PROA
ATOM	4074	HB2	ALA	P	250	-13.141	2.697	-2.150	0.00	0.00	PROA
ATOM	4075	HB3	ALA	P	250	-12.050	3.016	-3.636	0.00	0.00	PROA
ATOM	4076	C	ALA	P	250	-10.771	3.443	-0.934	0.00	0.00	PROA
ATOM	4077	O	ALA	P	250	-11.284	2.936	0.087	0.00	0.00	PROA
ATOM	4078	N	CYS	P	251	-9.470	3.195	-1.127	0.00	0.00	PROA
ATOM	4079	HN	CYS	P	251	-9.027	3.618	-1.914	0.00	0.00	PROA
ATOM	4080	CA	CYS	P	251	-8.757	2.283	-0.215	0.00	0.00	PROA
ATOM	4081	HA	CYS	P	251	-9.203	2.361	0.765	0.00	0.00	PROA
ATOM	4082	CB	CYS	P	251	-8.929	0.727	-0.509	0.00	0.00	PROA
ATOM	4083	HB1	CYS	P	251	-9.965	0.525	-0.161	0.00	0.00	PROA

```
ATOM   4084  HB2 CYS P 251      -8.240   0.077   0.071  0.00  0.00      PROA
ATOM   4085  SG  CYS P 251      -8.782   0.355  -2.283  0.00  0.00      PROA
ATOM   4086  HG1 CYS P 251      -9.856   1.069  -2.586  0.00  0.00      PROA
ATOM   4087  C   CYS P 251      -7.267   2.573   0.209  0.00  0.00      PROA
ATOM   4088  O   CYS P 251      -7.138   3.222   1.243  0.00  0.00      PROA
ATOM   4089  N   TRP P 252      -6.314   2.237  -0.607  0.00  0.00      PROA
ATOM   4090  HN  TRP P 252      -6.546   1.579  -1.320  0.00  0.00      PROA
ATOM   4091  CA  TRP P 252      -4.956   2.604  -0.243  0.00  0.00      PROA
ATOM   4092  HA  TRP P 252      -4.722   2.175   0.720  0.00  0.00      PROA
ATOM   4093  CB  TRP P 252      -4.205   2.139  -1.492  0.00  0.00      PROA
ATOM   4094  HB1 TRP P 252      -3.155   2.490  -1.398  0.00  0.00      PROA
ATOM   4095  HB2 TRP P 252      -4.616   2.588  -2.421  0.00  0.00      PROA
ATOM   4096  CG  TRP P 252      -4.181   0.622  -1.575  0.00  0.00      PROA
ATOM   4097  CD1 TRP P 252      -4.883  -0.155  -2.463  0.00  0.00      PROA
ATOM   4098  HD1 TRP P 252      -5.460   0.316  -3.245  0.00  0.00      PROA
ATOM   4099  NE1 TRP P 252      -4.573  -1.545  -2.284  0.00  0.00      PROA
ATOM   4100  HE1 TRP P 252      -4.719  -2.349  -2.818  0.00  0.00      PROA
ATOM   4101  CE2 TRP P 252      -3.781  -1.608  -1.171  0.00  0.00      PROA
ATOM   4102  CD2 TRP P 252      -3.580  -0.325  -0.650  0.00  0.00      PROA
ATOM   4103  CE3 TRP P 252      -2.825  -0.128   0.466  0.00  0.00      PROA
ATOM   4104  HE3 TRP P 252      -2.648   0.828   0.937  0.00  0.00      PROA
ATOM   4105  CZ3 TRP P 252      -2.283  -1.303   1.118  0.00  0.00      PROA
ATOM   4106  HZ3 TRP P 252      -1.652  -1.182   1.986  0.00  0.00      PROA
ATOM   4107  CZ2 TRP P 252      -3.324  -2.767  -0.498  0.00  0.00      PROA
ATOM   4108  HZ2 TRP P 252      -3.494  -3.752  -0.907  0.00  0.00      PROA
ATOM   4109  CH2 TRP P 252      -2.560  -2.594   0.614  0.00  0.00      PROA
ATOM   4110  HH2 TRP P 252      -2.036  -3.457   0.997  0.00  0.00      PROA
ATOM   4111  C   TRP P 252      -4.727   4.103   0.050  0.00  0.00      PROA
ATOM   4112  O   TRP P 252      -4.039   4.466   0.993  0.00  0.00      PROA
ATOM   4113  N   LEU P 253      -5.221   5.054  -0.786  0.00  0.00      PROA
ATOM   4114  HN  LEU P 253      -5.757   4.809  -1.590  0.00  0.00      PROA
ATOM   4115  CA  LEU P 253      -5.002   6.489  -0.630  0.00  0.00      PROA
ATOM   4116  HA  LEU P 253      -3.924   6.560  -0.637  0.00  0.00      PROA
ATOM   4117  CB  LEU P 253      -5.475   7.398  -1.849  0.00  0.00      PROA
ATOM   4118  HB1 LEU P 253      -6.576   7.487  -1.968  0.00  0.00      PROA
ATOM   4119  HB2 LEU P 253      -5.022   6.919  -2.743  0.00  0.00      PROA
ATOM   4120  CG  LEU P 253      -4.937   8.814  -1.850  0.00  0.00      PROA
ATOM   4121  HG  LEU P 253      -5.494   9.172  -0.958  0.00  0.00      PROA
ATOM   4122  CD1 LEU P 253      -3.393   8.931  -1.738  0.00  0.00      PROA
ATOM   4123 HD11 LEU P 253      -2.877   8.440  -2.591  0.00  0.00      PROA
ATOM   4124 HD12 LEU P 253      -3.083   8.450  -0.785  0.00  0.00      PROA
ATOM   4125 HD13 LEU P 253      -3.031   9.979  -1.674  0.00  0.00      PROA
ATOM   4126  CD2 LEU P 253      -5.433   9.576  -3.091  0.00  0.00      PROA
ATOM   4127 HD21 LEU P 253      -6.537   9.581  -3.212  0.00  0.00      PROA
ATOM   4128 HD22 LEU P 253      -5.007   9.013  -3.949  0.00  0.00      PROA
ATOM   4129 HD23 LEU P 253      -5.155  10.652  -3.096  0.00  0.00      PROA
ATOM   4130  C   LEU P 253      -5.518   7.101   0.641  0.00  0.00      PROA
ATOM   4131  O   LEU P 253      -4.750   7.803   1.284  0.00  0.00      PROA
ATOM   4132  N   PRO P 254      -6.734   6.901   1.100  0.00  0.00      PROA
ATOM   4133  CD  PRO P 254      -7.875   6.349   0.409  0.00  0.00      PROA
ATOM   4134  HD1 PRO P 254      -7.678   5.408  -0.148  0.00  0.00      PROA
ATOM   4135  HD2 PRO P 254      -8.228   7.124  -0.305  0.00  0.00      PROA
ATOM   4136  CA  PRO P 254      -7.065   7.292   2.448  0.00  0.00      PROA
ATOM   4137  HA  PRO P 254      -6.805   8.334   2.560  0.00  0.00      PROA
ATOM   4138  CB  PRO P 254      -8.611   7.122   2.584  0.00  0.00      PROA
ATOM   4139  HB1 PRO P 254      -9.046   8.111   2.322  0.00  0.00      PROA
ATOM   4140  HB2 PRO P 254      -8.897   6.647   3.547  0.00  0.00      PROA
ATOM   4141  CG  PRO P 254      -8.989   6.165   1.507  0.00  0.00      PROA
ATOM   4142  HG1 PRO P 254      -9.016   5.106   1.842  0.00  0.00      PROA
ATOM   4143  HG2 PRO P 254      -9.983   6.339   1.041  0.00  0.00      PROA
ATOM   4144  C   PRO P 254      -6.319   6.554   3.543  0.00  0.00      PROA
```

ATOM	4145	O	PRO	P	254	-6.079	7.118	4.632	0.00	0.00	PROA
ATOM	4146	N	TYR	P	255	-5.963	5.266	3.404	0.00	0.00	PROA
ATOM	4147	HN	TYR	P	255	-6.148	4.848	2.518	0.00	0.00	PROA
ATOM	4148	CA	TYR	P	255	-5.059	4.527	4.278	0.00	0.00	PROA
ATOM	4149	HA	TYR	P	255	-5.535	4.507	5.247	0.00	0.00	PROA
ATOM	4150	CB	TYR	P	255	-5.096	3.012	3.759	0.00	0.00	PROA
ATOM	4151	HB1	TYR	P	255	-4.779	3.148	2.703	0.00	0.00	PROA
ATOM	4152	HB2	TYR	P	255	-6.125	2.602	3.844	0.00	0.00	PROA
ATOM	4153	CG	TYR	P	255	-4.109	2.095	4.414	0.00	0.00	PROA
ATOM	4154	CD1	TYR	P	255	-2.862	1.674	3.878	0.00	0.00	PROA
ATOM	4155	HD1	TYR	P	255	-2.432	2.051	2.962	0.00	0.00	PROA
ATOM	4156	CE1	TYR	P	255	-2.099	0.775	4.663	0.00	0.00	PROA
ATOM	4157	HE1	TYR	P	255	-1.219	0.245	4.330	0.00	0.00	PROA
ATOM	4158	CZ	TYR	P	255	-2.604	0.284	5.871	0.00	0.00	PROA
ATOM	4159	OH	TYR	P	255	-1.704	-0.369	6.658	0.00	0.00	PROA
ATOM	4160	HH	TYR	P	255	-2.244	-0.776	7.341	0.00	0.00	PROA
ATOM	4161	CD2	TYR	P	255	-4.493	1.665	5.704	0.00	0.00	PROA
ATOM	4162	HD2	TYR	P	255	-5.410	1.966	6.189	0.00	0.00	PROA
ATOM	4163	CE2	TYR	P	255	-3.750	0.702	6.354	0.00	0.00	PROA
ATOM	4164	HE2	TYR	P	255	-4.197	0.338	7.267	0.00	0.00	PROA
ATOM	4165	C	TYR	P	255	-3.622	5.246	4.410	0.00	0.00	PROA
ATOM	4166	O	TYR	P	255	-3.123	5.482	5.513	0.00	0.00	PROA
ATOM	4167	N	TYR	P	256	-3.042	5.783	3.342	0.00	0.00	PROA
ATOM	4168	HN	TYR	P	256	-3.455	5.522	2.473	0.00	0.00	PROA
ATOM	4169	CA	TYR	P	256	-1.752	6.449	3.336	0.00	0.00	PROA
ATOM	4170	HA	TYR	P	256	-1.024	5.835	3.844	0.00	0.00	PROA
ATOM	4171	CB	TYR	P	256	-1.303	6.761	1.916	0.00	0.00	PROA
ATOM	4172	HB1	TYR	P	256	-0.390	7.384	1.804	0.00	0.00	PROA
ATOM	4173	HB2	TYR	P	256	-2.129	7.363	1.481	0.00	0.00	PROA
ATOM	4174	CG	TYR	P	256	-1.090	5.526	1.070	0.00	0.00	PROA
ATOM	4175	CD1	TYR	P	256	-0.933	4.205	1.630	0.00	0.00	PROA
ATOM	4176	HD1	TYR	P	256	-0.987	3.973	2.683	0.00	0.00	PROA
ATOM	4177	CE1	TYR	P	256	-0.717	3.093	0.819	0.00	0.00	PROA
ATOM	4178	HE1	TYR	P	256	-0.599	2.190	1.400	0.00	0.00	PROA
ATOM	4179	CZ	TYR	P	256	-0.578	3.297	-0.551	0.00	0.00	PROA
ATOM	4180	OH	TYR	P	256	-0.114	2.243	-1.326	0.00	0.00	PROA
ATOM	4181	HH	TYR	P	256	-0.138	2.524	-2.244	0.00	0.00	PROA
ATOM	4182	CD2	TYR	P	256	-1.020	5.663	-0.296	0.00	0.00	PROA
ATOM	4183	HD2	TYR	P	256	-1.017	6.658	-0.716	0.00	0.00	PROA
ATOM	4184	CE2	TYR	P	256	-0.742	4.595	-1.100	0.00	0.00	PROA
ATOM	4185	HE2	TYR	P	256	-0.570	4.662	-2.164	0.00	0.00	PROA
ATOM	4186	C	TYR	P	256	-1.822	7.779	4.077	0.00	0.00	PROA
ATOM	4187	O	TYR	P	256	-0.968	8.086	4.885	0.00	0.00	PROA
ATOM	4188	N	ILE	P	257	-2.899	8.535	3.821	0.00	0.00	PROA
ATOM	4189	HN	ILE	P	257	-3.552	8.197	3.148	0.00	0.00	PROA
ATOM	4190	CA	ILE	P	257	-3.253	9.679	4.524	0.00	0.00	PROA
ATOM	4191	HA	ILE	P	257	-2.394	10.335	4.528	0.00	0.00	PROA
ATOM	4192	CB	ILE	P	257	-4.468	10.219	3.743	0.00	0.00	PROA
ATOM	4193	HB	ILE	P	257	-5.226	9.426	3.564	0.00	0.00	PROA
ATOM	4194	CG2	ILE	P	257	-5.301	11.291	4.455	0.00	0.00	PROA
ATOM	4195	HG21	ILE	P	257	-5.610	10.934	5.460	0.00	0.00	PROA
ATOM	4196	HG22	ILE	P	257	-6.192	11.513	3.829	0.00	0.00	PROA
ATOM	4197	HG23	ILE	P	257	-4.715	12.229	4.558	0.00	0.00	PROA
ATOM	4198	CG1	ILE	P	257	-3.961	10.738	2.347	0.00	0.00	PROA
ATOM	4199	HG11	ILE	P	257	-3.400	9.910	1.862	0.00	0.00	PROA
ATOM	4200	HG12	ILE	P	257	-3.247	11.573	2.513	0.00	0.00	PROA
ATOM	4201	CD	ILE	P	257	-5.071	11.201	1.408	0.00	0.00	PROA
ATOM	4202	HD1	ILE	P	257	-5.711	12.041	1.754	0.00	0.00	PROA
ATOM	4203	HD2	ILE	P	257	-5.708	10.298	1.288	0.00	0.00	PROA
ATOM	4204	HD3	ILE	P	257	-4.704	11.531	0.412	0.00	0.00	PROA
ATOM	4205	C	ILE	P	257	-3.584	9.458	5.979	0.00	0.00	PROA

ATOM	4206	O	ILE	P	257	-3.132	10.128	6.916	0.00	0.00	PROA
ATOM	4207	N	GLY	P	258	-4.345	8.339	6.265	0.00	0.00	PROA
ATOM	4208	HN	GLY	P	258	-4.772	7.801	5.542	0.00	0.00	PROA
ATOM	4209	CA	GLY	P	258	-4.733	7.826	7.553	0.00	0.00	PROA
ATOM	4210	HA1	GLY	P	258	-5.353	6.950	7.434	0.00	0.00	PROA
ATOM	4211	HA2	GLY	P	258	-5.361	8.573	8.016	0.00	0.00	PROA
ATOM	4212	C	GLY	P	258	-3.604	7.579	8.452	0.00	0.00	PROA
ATOM	4213	O	GLY	P	258	-3.453	8.232	9.480	0.00	0.00	PROA
ATOM	4214	N	ILE	P	259	-2.605	6.676	8.070	0.00	0.00	PROA
ATOM	4215	HN	ILE	P	259	-2.673	6.192	7.201	0.00	0.00	PROA
ATOM	4216	CA	ILE	P	259	-1.376	6.443	8.851	0.00	0.00	PROA
ATOM	4217	HA	ILE	P	259	-1.705	6.084	9.815	0.00	0.00	PROA
ATOM	4218	CB	ILE	P	259	-0.517	5.359	8.181	0.00	0.00	PROA
ATOM	4219	HB	ILE	P	259	-0.521	5.577	7.092	0.00	0.00	PROA
ATOM	4220	CG2	ILE	P	259	0.947	5.395	8.735	0.00	0.00	PROA
ATOM	4221	HG21	ILE	P	259	1.510	6.348	8.641	0.00	0.00	PROA
ATOM	4222	HG22	ILE	P	259	1.562	4.585	8.289	0.00	0.00	PROA
ATOM	4223	HG23	ILE	P	259	0.895	5.138	9.815	0.00	0.00	PROA
ATOM	4224	CG1	ILE	P	259	-1.117	3.986	8.315	0.00	0.00	PROA
ATOM	4225	HG11	ILE	P	259	-2.215	3.974	8.150	0.00	0.00	PROA
ATOM	4226	HG12	ILE	P	259	-1.097	3.646	9.372	0.00	0.00	PROA
ATOM	4227	CD	ILE	P	259	-0.529	2.908	7.433	0.00	0.00	PROA
ATOM	4228	HD1	ILE	P	259	-1.136	1.982	7.528	0.00	0.00	PROA
ATOM	4229	HD2	ILE	P	259	0.470	2.592	7.803	0.00	0.00	PROA
ATOM	4230	HD3	ILE	P	259	-0.455	3.291	6.393	0.00	0.00	PROA
ATOM	4231	C	ILE	P	259	-0.553	7.648	9.068	0.00	0.00	PROA
ATOM	4232	O	ILE	P	259	-0.019	7.757	10.136	0.00	0.00	PROA
ATOM	4233	N	SER	P	260	-0.531	8.581	8.123	0.00	0.00	PROA
ATOM	4234	HN	SER	P	260	-1.042	8.390	7.288	0.00	0.00	PROA
ATOM	4235	CA	SER	P	260	0.113	9.858	8.219	0.00	0.00	PROA
ATOM	4236	HA	SER	P	260	1.115	9.602	8.532	0.00	0.00	PROA
ATOM	4237	CB	SER	P	260	0.186	10.494	6.817	0.00	0.00	PROA
ATOM	4238	HB1	SER	P	260	0.486	11.564	6.818	0.00	0.00	PROA
ATOM	4239	HB2	SER	P	260	-0.839	10.602	6.402	0.00	0.00	PROA
ATOM	4240	OG	SER	P	260	0.971	9.759	5.906	0.00	0.00	PROA
ATOM	4241	HG1	SER	P	260	0.436	9.123	5.426	0.00	0.00	PROA
ATOM	4242	C	SER	P	260	-0.537	10.833	9.228	0.00	0.00	PROA
ATOM	4243	O	SER	P	260	0.175	11.505	9.934	0.00	0.00	PROA
ATOM	4244	N	ILE	P	261	-1.871	10.942	9.292	0.00	0.00	PROA
ATOM	4245	HN	ILE	P	261	-2.365	10.370	8.641	0.00	0.00	PROA
ATOM	4246	CA	ILE	P	261	-2.583	11.588	10.341	0.00	0.00	PROA
ATOM	4247	HA	ILE	P	261	-2.196	12.596	10.331	0.00	0.00	PROA
ATOM	4248	CB	ILE	P	261	-4.080	11.585	10.097	0.00	0.00	PROA
ATOM	4249	HB	ILE	P	261	-4.472	10.547	10.146	0.00	0.00	PROA
ATOM	4250	CG2	ILE	P	261	-4.717	12.487	11.233	0.00	0.00	PROA
ATOM	4251	HG21	ILE	P	261	-4.132	13.414	11.414	0.00	0.00	PROA
ATOM	4252	HG22	ILE	P	261	-4.822	11.853	12.140	0.00	0.00	PROA
ATOM	4253	HG23	ILE	P	261	-5.684	12.964	10.965	0.00	0.00	PROA
ATOM	4254	CG1	ILE	P	261	-4.552	12.324	8.751	0.00	0.00	PROA
ATOM	4255	HG11	ILE	P	261	-3.780	11.910	8.068	0.00	0.00	PROA
ATOM	4256	HG12	ILE	P	261	-4.470	13.411	8.964	0.00	0.00	PROA
ATOM	4257	CD	ILE	P	261	-5.979	11.964	8.327	0.00	0.00	PROA
ATOM	4258	HD1	ILE	P	261	-6.697	12.423	9.040	0.00	0.00	PROA
ATOM	4259	HD2	ILE	P	261	-6.099	10.876	8.138	0.00	0.00	PROA
ATOM	4260	HD3	ILE	P	261	-6.155	12.370	7.308	0.00	0.00	PROA
ATOM	4261	C	ILE	P	261	-2.283	10.963	11.736	0.00	0.00	PROA
ATOM	4262	O	ILE	P	261	-1.664	11.619	12.599	0.00	0.00	PROA
ATOM	4263	N	ASP	P	262	-2.462	9.601	11.791	0.00	0.00	PROA
ATOM	4264	HN	ASP	P	262	-2.740	9.075	10.991	0.00	0.00	PROA
ATOM	4265	CA	ASP	P	262	-2.061	8.818	12.955	0.00	0.00	PROA
ATOM	4266	HA	ASP	P	262	-2.807	8.975	13.720	0.00	0.00	PROA

ATOM	4267	CB	ASP	P	262	-2.294	7.357	12.571	0.00	0.00	PROA
ATOM	4268	HB1	ASP	P	262	-1.475	6.853	12.015	0.00	0.00	PROA
ATOM	4269	HB2	ASP	P	262	-3.280	7.136	12.109	0.00	0.00	PROA
ATOM	4270	CG	ASP	P	262	-2.310	6.416	13.810	0.00	0.00	PROA
ATOM	4271	OD1	ASP	P	262	-2.271	5.186	13.557	0.00	0.00	PROA
ATOM	4272	OD2	ASP	P	262	-2.382	6.851	14.984	0.00	0.00	PROA
ATOM	4273	C	ASP	P	262	-0.619	9.112	13.482	0.00	0.00	PROA
ATOM	4274	O	ASP	P	262	-0.361	9.538	14.541	0.00	0.00	PROA
ATOM	4275	N	SER	P	263	0.299	9.056	12.494	0.00	0.00	PROA
ATOM	4276	HN	SER	P	263	0.030	8.802	11.569	0.00	0.00	PROA
ATOM	4277	CA	SER	P	263	1.753	9.252	12.601	0.00	0.00	PROA
ATOM	4278	HA	SER	P	263	2.064	8.456	13.262	0.00	0.00	PROA
ATOM	4279	CB	SER	P	263	2.492	8.934	11.226	0.00	0.00	PROA
ATOM	4280	HB1	SER	P	263	1.981	9.541	10.448	0.00	0.00	PROA
ATOM	4281	HB2	SER	P	263	2.326	7.852	11.033	0.00	0.00	PROA
ATOM	4282	OG	SER	P	263	3.881	9.200	11.200	0.00	0.00	PROA
ATOM	4283	HG1	SER	P	263	4.200	8.773	10.402	0.00	0.00	PROA
ATOM	4284	C	SER	P	263	2.135	10.641	13.146	0.00	0.00	PROA
ATOM	4285	O	SER	P	263	2.965	10.799	14.023	0.00	0.00	PROA
ATOM	4286	N	PHE	P	264	1.433	11.727	12.741	0.00	0.00	PROA
ATOM	4287	HN	PHE	P	264	0.728	11.554	12.058	0.00	0.00	PROA
ATOM	4288	CA	PHE	P	264	1.743	13.123	13.041	0.00	0.00	PROA
ATOM	4289	HA	PHE	P	264	2.817	13.183	13.137	0.00	0.00	PROA
ATOM	4290	CB	PHE	P	264	1.302	14.142	11.935	0.00	0.00	PROA
ATOM	4291	HB1	PHE	P	264	1.051	15.073	12.485	0.00	0.00	PROA
ATOM	4292	HB2	PHE	P	264	0.339	13.862	11.455	0.00	0.00	PROA
ATOM	4293	CG	PHE	P	264	2.361	14.412	10.922	0.00	0.00	PROA
ATOM	4294	CD1	PHE	P	264	2.309	15.559	10.166	0.00	0.00	PROA
ATOM	4295	HD1	PHE	P	264	1.393	16.128	10.217	0.00	0.00	PROA
ATOM	4296	CE1	PHE	P	264	3.202	15.821	9.146	0.00	0.00	PROA
ATOM	4297	HE1	PHE	P	264	2.961	16.677	8.532	0.00	0.00	PROA
ATOM	4298	CZ	PHE	P	264	4.398	14.975	8.997	0.00	0.00	PROA
ATOM	4299	HZ	PHE	P	264	5.197	15.166	8.296	0.00	0.00	PROA
ATOM	4300	CD2	PHE	P	264	3.495	13.569	10.718	0.00	0.00	PROA
ATOM	4301	HD2	PHE	P	264	3.493	12.649	11.284	0.00	0.00	PROA
ATOM	4302	CE2	PHE	P	264	4.474	13.823	9.754	0.00	0.00	PROA
ATOM	4303	HE2	PHE	P	264	5.341	13.182	9.826	0.00	0.00	PROA
ATOM	4304	C	PHE	P	264	1.235	13.460	14.390	0.00	0.00	PROA
ATOM	4305	O	PHE	P	264	1.753	14.385	15.095	0.00	0.00	PROA
ATOM	4306	N	ILE	P	265	0.252	12.608	14.915	0.00	0.00	PROA
ATOM	4307	HN	ILE	P	265	-0.074	11.918	14.273	0.00	0.00	PROA
ATOM	4308	CA	ILE	P	265	-0.237	12.674	16.272	0.00	0.00	PROA
ATOM	4309	HA	ILE	P	265	-0.325	13.702	16.591	0.00	0.00	PROA
ATOM	4310	CB	ILE	P	265	-1.589	11.988	16.516	0.00	0.00	PROA
ATOM	4311	HB	ILE	P	265	-1.513	10.920	16.217	0.00	0.00	PROA
ATOM	4312	CG2	ILE	P	265	-2.043	12.122	17.986	0.00	0.00	PROA
ATOM	4313	HG21	ILE	P	265	-2.231	13.198	18.187	0.00	0.00	PROA
ATOM	4314	HG22	ILE	P	265	-1.277	11.731	18.689	0.00	0.00	PROA
ATOM	4315	HG23	ILE	P	265	-3.023	11.609	18.087	0.00	0.00	PROA
ATOM	4316	CG1	ILE	P	265	-2.709	12.710	15.598	0.00	0.00	PROA
ATOM	4317	HG11	ILE	P	265	-2.254	13.009	14.630	0.00	0.00	PROA
ATOM	4318	HG12	ILE	P	265	-3.045	13.652	16.082	0.00	0.00	PROA
ATOM	4319	CD	ILE	P	265	-3.923	11.782	15.279	0.00	0.00	PROA
ATOM	4320	HD1	ILE	P	265	-4.834	12.264	14.865	0.00	0.00	PROA
ATOM	4321	HD2	ILE	P	265	-4.211	11.236	16.203	0.00	0.00	PROA
ATOM	4322	HD3	ILE	P	265	-3.336	11.153	14.577	0.00	0.00	PROA
ATOM	4323	C	ILE	P	265	0.837	12.087	17.143	0.00	0.00	PROA
ATOM	4324	O	ILE	P	265	1.292	12.694	18.124	0.00	0.00	PROA
ATOM	4325	N	LEU	P	266	1.318	10.882	16.781	0.00	0.00	PROA
ATOM	4326	HN	LEU	P	266	1.013	10.414	15.955	0.00	0.00	PROA
ATOM	4327	CA	LEU	P	266	2.440	10.141	17.476	0.00	0.00	PROA

ATOM	4328	HA	LEU	P	266	2.171	9.902	18.495	0.00	0.00	PROA
ATOM	4329	CB	LEU	P	266	2.547	8.724	16.730	0.00	0.00	PROA
ATOM	4330	HB1	LEU	P	266	2.859	8.946	15.688	0.00	0.00	PROA
ATOM	4331	HB2	LEU	P	266	1.557	8.222	16.676	0.00	0.00	PROA
ATOM	4332	CG	LEU	P	266	3.560	7.789	17.261	0.00	0.00	PROA
ATOM	4333	HG	LEU	P	266	4.577	8.112	16.952	0.00	0.00	PROA
ATOM	4334	CD1	LEU	P	266	3.302	7.478	18.760	0.00	0.00	PROA
ATOM	4335	HD11	LEU	P	266	2.334	6.946	18.878	0.00	0.00	PROA
ATOM	4336	HD12	LEU	P	266	3.251	8.367	19.425	0.00	0.00	PROA
ATOM	4337	HD13	LEU	P	266	4.150	6.802	19.001	0.00	0.00	PROA
ATOM	4338	CD2	LEU	P	266	3.336	6.448	16.670	0.00	0.00	PROA
ATOM	4339	HD21	LEU	P	266	2.248	6.225	16.672	0.00	0.00	PROA
ATOM	4340	HD22	LEU	P	266	3.918	5.598	17.085	0.00	0.00	PROA
ATOM	4341	HD23	LEU	P	266	3.641	6.568	15.609	0.00	0.00	PROA
ATOM	4342	C	LEU	P	266	3.721	10.970	17.607	0.00	0.00	PROA
ATOM	4343	O	LEU	P	266	4.422	10.927	18.629	0.00	0.00	PROA
ATOM	4344	N	LEU	P	267	4.100	11.803	16.615	0.00	0.00	PROA
ATOM	4345	HN	LEU	P	267	3.689	11.686	15.714	0.00	0.00	PROA
ATOM	4346	CA	LEU	P	267	5.231	12.749	16.618	0.00	0.00	PROA
ATOM	4347	HA	LEU	P	267	6.098	12.367	17.135	0.00	0.00	PROA
ATOM	4348	CB	LEU	P	267	5.725	13.006	15.201	0.00	0.00	PROA
ATOM	4349	HB1	LEU	P	267	6.363	13.892	14.993	0.00	0.00	PROA
ATOM	4350	HB2	LEU	P	267	4.801	13.204	14.616	0.00	0.00	PROA
ATOM	4351	CG	LEU	P	267	6.343	11.688	14.635	0.00	0.00	PROA
ATOM	4352	HG	LEU	P	267	5.492	11.003	14.434	0.00	0.00	PROA
ATOM	4353	CD1	LEU	P	267	6.938	11.920	13.306	0.00	0.00	PROA
ATOM	4354	HD11	LEU	P	267	6.196	12.106	12.500	0.00	0.00	PROA
ATOM	4355	HD12	LEU	P	267	7.464	10.986	13.016	0.00	0.00	PROA
ATOM	4356	HD13	LEU	P	267	7.685	12.737	13.395	0.00	0.00	PROA
ATOM	4357	CD2	LEU	P	267	7.418	10.953	15.492	0.00	0.00	PROA
ATOM	4358	HD21	LEU	P	267	7.850	10.135	14.877	0.00	0.00	PROA
ATOM	4359	HD22	LEU	P	267	6.881	10.532	16.369	0.00	0.00	PROA
ATOM	4360	HD23	LEU	P	267	8.149	11.786	15.574	0.00	0.00	PROA
ATOM	4361	C	LEU	P	267	4.835	14.060	17.308	0.00	0.00	PROA
ATOM	4362	O	LEU	P	267	5.657	14.994	17.443	0.00	0.00	PROA
ATOM	4363	N	GLU	P	268	3.676	14.199	17.882	0.00	0.00	PROA
ATOM	4364	HN	GLU	P	268	3.022	13.461	17.737	0.00	0.00	PROA
ATOM	4365	CA	GLU	P	268	3.276	15.359	18.628	0.00	0.00	PROA
ATOM	4366	HA	GLU	P	268	2.271	15.151	18.963	0.00	0.00	PROA
ATOM	4367	CB	GLU	P	268	4.058	15.390	19.969	0.00	0.00	PROA
ATOM	4368	HB1	GLU	P	268	3.763	16.316	20.507	0.00	0.00	PROA
ATOM	4369	HB2	GLU	P	268	5.151	15.412	19.776	0.00	0.00	PROA
ATOM	4370	CG	GLU	P	268	3.639	14.173	20.821	0.00	0.00	PROA
ATOM	4371	HG1	GLU	P	268	3.961	13.228	20.335	0.00	0.00	PROA
ATOM	4372	HG2	GLU	P	268	2.534	14.056	20.843	0.00	0.00	PROA
ATOM	4373	CD	GLU	P	268	4.225	14.252	22.231	0.00	0.00	PROA
ATOM	4374	OE1	GLU	P	268	3.450	14.453	23.249	0.00	0.00	PROA
ATOM	4375	OE2	GLU	P	268	5.446	14.101	22.472	0.00	0.00	PROA
ATOM	4376	C	GLU	P	268	3.189	16.652	17.880	0.00	0.00	PROA
ATOM	4377	O	GLU	P	268	3.077	17.826	18.430	0.00	0.00	PROA
ATOM	4378	N	ILE	P	269	3.187	16.544	16.553	0.00	0.00	PROA
ATOM	4379	HN	ILE	P	269	3.127	15.639	16.139	0.00	0.00	PROA
ATOM	4380	CA	ILE	P	269	2.900	17.665	15.625	0.00	0.00	PROA
ATOM	4381	HA	ILE	P	269	3.424	18.585	15.837	0.00	0.00	PROA
ATOM	4382	CB	ILE	P	269	3.105	17.251	14.197	0.00	0.00	PROA
ATOM	4383	HB	ILE	P	269	2.602	16.269	14.067	0.00	0.00	PROA
ATOM	4384	CG2	ILE	P	269	2.525	18.313	13.181	0.00	0.00	PROA
ATOM	4385	HG21	ILE	P	269	2.730	18.019	12.130	0.00	0.00	PROA
ATOM	4386	HG22	ILE	P	269	3.011	19.289	13.398	0.00	0.00	PROA
ATOM	4387	HG23	ILE	P	269	1.426	18.383	13.327	0.00	0.00	PROA
ATOM	4388	CG1	ILE	P	269	4.618	17.148	13.965	0.00	0.00	PROA

```
ATOM   4389 HG11 ILE P 269      5.241  16.596  14.701  0.00  0.00      PROA
ATOM   4390 HG12 ILE P 269      5.100  18.145  13.866  0.00  0.00      PROA
ATOM   4391  CD  ILE P 269      4.867  16.352  12.641  0.00  0.00      PROA
ATOM   4392  HD1 ILE P 269      5.964  16.175  12.647  0.00  0.00      PROA
ATOM   4393  HD2 ILE P 269      4.533  16.932  11.755  0.00  0.00      PROA
ATOM   4394  HD3 ILE P 269      4.349  15.373  12.733  0.00  0.00      PROA
ATOM   4395  C   ILE P 269      1.426  18.071  15.765  0.00  0.00      PROA
ATOM   4396  O   ILE P 269      1.005  19.237  15.688  0.00  0.00      PROA
ATOM   4397  N   ILE P 270      0.481  17.098  15.962  0.00  0.00      PROA
ATOM   4398  HN  ILE P 270      0.778  16.151  15.864  0.00  0.00      PROA
ATOM   4399  CA  ILE P 270     -0.967  17.318  16.146  0.00  0.00      PROA
ATOM   4400  HA  ILE P 270     -1.169  18.368  15.995  0.00  0.00      PROA
ATOM   4401  CB  ILE P 270     -1.756  16.448  15.051  0.00  0.00      PROA
ATOM   4402  HB  ILE P 270     -1.283  15.447  15.133  0.00  0.00      PROA
ATOM   4403  CG2 ILE P 270     -3.235  16.352  15.298  0.00  0.00      PROA
ATOM   4404 HG21 ILE P 270     -3.740  15.823  14.461  0.00  0.00      PROA
ATOM   4405 HG22 ILE P 270     -3.698  17.334  15.535  0.00  0.00      PROA
ATOM   4406 HG23 ILE P 270     -3.458  15.641  16.122  0.00  0.00      PROA
ATOM   4407  CG1 ILE P 270     -1.460  17.020  13.643  0.00  0.00      PROA
ATOM   4408 HG11 ILE P 270     -0.371  17.229  13.574  0.00  0.00      PROA
ATOM   4409 HG12 ILE P 270     -1.995  17.994  13.634  0.00  0.00      PROA
ATOM   4410  CD  ILE P 270     -1.942  16.215  12.433  0.00  0.00      PROA
ATOM   4411  HD1 ILE P 270     -3.001  15.886  12.493  0.00  0.00      PROA
ATOM   4412  HD2 ILE P 270     -1.389  15.251  12.435  0.00  0.00      PROA
ATOM   4413  HD3 ILE P 270     -1.715  16.635  11.430  0.00  0.00      PROA
ATOM   4414  C   ILE P 270     -1.337  16.854  17.580  0.00  0.00      PROA
ATOM   4415  O   ILE P 270     -1.048  15.745  17.984  0.00  0.00      PROA
ATOM   4416  N   LYS P 271     -2.081  17.711  18.327  0.00  0.00      PROA
ATOM   4417  HN  LYS P 271     -2.425  18.594  18.015  0.00  0.00      PROA
ATOM   4418  CA  LYS P 271     -2.430  17.368  19.753  0.00  0.00      PROA
ATOM   4419  HA  LYS P 271     -1.856  16.501  20.042  0.00  0.00      PROA
ATOM   4420  CB  LYS P 271     -2.085  18.685  20.626  0.00  0.00      PROA
ATOM   4421  HB1 LYS P 271     -2.420  18.700  21.685  0.00  0.00      PROA
ATOM   4422  HB2 LYS P 271     -2.681  19.478  20.125  0.00  0.00      PROA
ATOM   4423  CG  LYS P 271     -0.587  19.156  20.682  0.00  0.00      PROA
ATOM   4424  HG1 LYS P 271     -0.476  20.174  21.114  0.00  0.00      PROA
ATOM   4425  HG2 LYS P 271     -0.130  19.165  19.670  0.00  0.00      PROA
ATOM   4426  CD  LYS P 271      0.381  18.194  21.359  0.00  0.00      PROA
ATOM   4427  HD1 LYS P 271      0.429  17.228  20.812  0.00  0.00      PROA
ATOM   4428  HD2 LYS P 271     -0.187  17.969  22.287  0.00  0.00      PROA
ATOM   4429  CE  LYS P 271      1.795  18.577  21.612  0.00  0.00      PROA
ATOM   4430  HE1 LYS P 271      2.342  18.920  20.708  0.00  0.00      PROA
ATOM   4431  HE2 LYS P 271      2.317  17.694  22.037  0.00  0.00      PROA
ATOM   4432  NZ  LYS P 271      1.861  19.625  22.621  0.00  0.00      PROA
ATOM   4433  HZ1 LYS P 271      1.125  19.649  23.355  0.00  0.00      PROA
ATOM   4434  HZ2 LYS P 271      1.861  20.504  22.064  0.00  0.00      PROA
ATOM   4435  HZ3 LYS P 271      2.787  19.599  23.095  0.00  0.00      PROA
ATOM   4436  C   LYS P 271     -3.848  16.909  20.028  0.00  0.00      PROA
ATOM   4437  O   LYS P 271     -4.279  16.654  21.174  0.00  0.00      PROA
ATOM   4438  N   GLN P 272     -4.672  16.831  19.010  0.00  0.00      PROA
ATOM   4439  HN  GLN P 272     -4.367  17.137  18.111  0.00  0.00      PROA
ATOM   4440  CA  GLN P 272     -6.070  16.424  19.054  0.00  0.00      PROA
ATOM   4441  HA  GLN P 272     -6.593  17.130  19.683  0.00  0.00      PROA
ATOM   4442  CB  GLN P 272     -6.801  16.497  17.657  0.00  0.00      PROA
ATOM   4443  HB1 GLN P 272     -7.884  16.266  17.739  0.00  0.00      PROA
ATOM   4444  HB2 GLN P 272     -6.453  15.680  16.990  0.00  0.00      PROA
ATOM   4445  CG  GLN P 272     -6.697  17.828  17.023  0.00  0.00      PROA
ATOM   4446  HG1 GLN P 272     -7.437  17.940  16.201  0.00  0.00      PROA
ATOM   4447  HG2 GLN P 272     -5.665  18.032  16.664  0.00  0.00      PROA
ATOM   4448  CD  GLN P 272     -7.041  18.881  18.041  0.00  0.00      PROA
ATOM   4449  OE1 GLN P 272     -6.216  19.582  18.535  0.00  0.00      PROA
```

```
ATOM   4450  NE2 GLN P 272      -8.341  19.053  18.299  0.00  0.00      PROA
ATOM   4451 HE21 GLN P 272      -8.701  19.813  18.841  0.00  0.00      PROA
ATOM   4452 HE22 GLN P 272      -8.972  18.404  17.873  0.00  0.00      PROA
ATOM   4453  C   GLN P 272      -6.295  15.081  19.665  0.00  0.00      PROA
ATOM   4454  O   GLN P 272      -5.488  14.147  19.655  0.00  0.00      PROA
ATOM   4455  N   GLY P 273      -7.461  15.063  20.333  0.00  0.00      PROA
ATOM   4456  HN  GLY P 273      -7.990  15.897  20.195  0.00  0.00      PROA
ATOM   4457  CA  GLY P 273      -7.959  14.175  21.408  0.00  0.00      PROA
ATOM   4458  HA1 GLY P 273      -8.988  14.420  21.623  0.00  0.00      PROA
ATOM   4459  HA2 GLY P 273      -7.394  14.325  22.317  0.00  0.00      PROA
ATOM   4460  C   GLY P 273      -7.898  12.732  21.229  0.00  0.00      PROA
ATOM   4461  O   GLY P 273      -7.757  12.168  20.160  0.00  0.00      PROA
ATOM   4462  N   CYS P 274      -8.151  11.929  22.320  0.00  0.00      PROA
ATOM   4463  HN  CYS P 274      -8.404  12.304  23.208  0.00  0.00      PROA
ATOM   4464  CA  CYS P 274      -8.121  10.529  22.231  0.00  0.00      PROA
ATOM   4465  HA  CYS P 274      -7.167  10.276  21.794  0.00  0.00      PROA
ATOM   4466  CB  CYS P 274      -8.258   9.912  23.602  0.00  0.00      PROA
ATOM   4467  HB1 CYS P 274      -8.072   8.817  23.576  0.00  0.00      PROA
ATOM   4468  HB2 CYS P 274      -9.294  10.050  23.978  0.00  0.00      PROA
ATOM   4469  SG  CYS P 274      -7.320  10.738  24.932  0.00  0.00      PROA
ATOM   4470  C   CYS P 274      -9.195   9.888  21.269  0.00  0.00      PROA
ATOM   4471  O   CYS P 274      -8.899   8.969  20.539  0.00  0.00      PROA
ATOM   4472  N   GLU P 275     -10.460  10.419  21.307  0.00  0.00      PROA
ATOM   4473  HN  GLU P 275     -10.674  11.031  22.065  0.00  0.00      PROA
ATOM   4474  CA  GLU P 275     -11.586  10.178  20.423  0.00  0.00      PROA
ATOM   4475  HA  GLU P 275     -11.706   9.105  20.400  0.00  0.00      PROA
ATOM   4476  CB  GLU P 275     -12.836  10.943  20.855  0.00  0.00      PROA
ATOM   4477  HB1 GLU P 275     -13.653  10.808  20.114  0.00  0.00      PROA
ATOM   4478  HB2 GLU P 275     -12.508  11.989  21.035  0.00  0.00      PROA
ATOM   4479  CG  GLU P 275     -13.427  10.567  22.261  0.00  0.00      PROA
ATOM   4480  HG1 GLU P 275     -12.745  10.810  23.104  0.00  0.00      PROA
ATOM   4481  HG2 GLU P 275     -13.699   9.502  22.419  0.00  0.00      PROA
ATOM   4482  CD  GLU P 275     -14.713  11.312  22.591  0.00  0.00      PROA
ATOM   4483  OE1 GLU P 275     -15.435  11.639  21.588  0.00  0.00      PROA
ATOM   4484  OE2 GLU P 275     -15.032  11.539  23.747  0.00  0.00      PROA
ATOM   4485  C   GLU P 275     -11.212  10.551  18.991  0.00  0.00      PROA
ATOM   4486  O   GLU P 275     -11.499   9.736  18.090  0.00  0.00      PROA
ATOM   4487  N   PHE P 276     -10.437  11.618  18.637  0.00  0.00      PROA
ATOM   4488  HN  PHE P 276     -10.172  12.343  19.269  0.00  0.00      PROA
ATOM   4489  CA  PHE P 276      -9.954  11.889  17.254  0.00  0.00      PROA
ATOM   4490  HA  PHE P 276     -10.866  11.830  16.678  0.00  0.00      PROA
ATOM   4491  CB  PHE P 276      -9.172  13.252  17.090  0.00  0.00      PROA
ATOM   4492  HB1 PHE P 276      -8.400  13.121  17.879  0.00  0.00      PROA
ATOM   4493  HB2 PHE P 276      -9.918  13.954  17.522  0.00  0.00      PROA
ATOM   4494  CG  PHE P 276      -8.718  13.771  15.749  0.00  0.00      PROA
ATOM   4495  CD1 PHE P 276      -7.404  13.469  15.291  0.00  0.00      PROA
ATOM   4496  HD1 PHE P 276      -6.749  12.812  15.844  0.00  0.00      PROA
ATOM   4497  CE1 PHE P 276      -6.927  13.959  14.059  0.00  0.00      PROA
ATOM   4498  HE1 PHE P 276      -5.940  13.795  13.653  0.00  0.00      PROA
ATOM   4499  CZ  PHE P 276      -7.803  14.609  13.238  0.00  0.00      PROA
ATOM   4500  HZ  PHE P 276      -7.422  14.926  12.278  0.00  0.00      PROA
ATOM   4501  CD2 PHE P 276      -9.574  14.499  14.918  0.00  0.00      PROA
ATOM   4502  HD2 PHE P 276     -10.541  14.810  15.284  0.00  0.00      PROA
ATOM   4503  CE2 PHE P 276      -9.134  14.956  13.642  0.00  0.00      PROA
ATOM   4504  HE2 PHE P 276      -9.783  15.543  13.009  0.00  0.00      PROA
ATOM   4505  C   PHE P 276      -9.055  10.747  16.835  0.00  0.00      PROA
ATOM   4506  O   PHE P 276      -9.162  10.209  15.714  0.00  0.00      PROA
ATOM   4507  N   GLU P 277      -8.143  10.297  17.791  0.00  0.00      PROA
ATOM   4508  HN  GLU P 277      -8.075  10.682  18.709  0.00  0.00      PROA
ATOM   4509  CA  GLU P 277      -7.272   9.154  17.520  0.00  0.00      PROA
ATOM   4510  HA  GLU P 277      -6.757   9.339  16.588  0.00  0.00      PROA
```

ATOM	4511	CB	GLU	P	277	-6.255	8.820	18.692	0.00	0.00	PROA
ATOM	4512	HB1	GLU	P	277	-5.759	7.867	18.407	0.00	0.00	PROA
ATOM	4513	HB2	GLU	P	277	-6.872	8.747	19.613	0.00	0.00	PROA
ATOM	4514	CG	GLU	P	277	-5.191	9.866	18.824	0.00	0.00	PROA
ATOM	4515	HG1	GLU	P	277	-5.680	10.847	19.005	0.00	0.00	PROA
ATOM	4516	HG2	GLU	P	277	-4.731	9.970	17.818	0.00	0.00	PROA
ATOM	4517	CD	GLU	P	277	-4.191	9.700	19.964	0.00	0.00	PROA
ATOM	4518	OE1	GLU	P	277	-3.665	8.611	20.195	0.00	0.00	PROA
ATOM	4519	OE2	GLU	P	277	-3.890	10.717	20.640	0.00	0.00	PROA
ATOM	4520	C	GLU	P	277	-7.951	7.881	17.254	0.00	0.00	PROA
ATOM	4521	O	GLU	P	277	-7.695	7.106	16.240	0.00	0.00	PROA
ATOM	4522	N	ASN	P	278	-8.953	7.552	18.062	0.00	0.00	PROA
ATOM	4523	HN	ASN	P	278	-8.952	7.973	18.965	0.00	0.00	PROA
ATOM	4524	CA	ASN	P	278	-9.840	6.383	17.932	0.00	0.00	PROA
ATOM	4525	HA	ASN	P	278	-9.272	5.464	17.936	0.00	0.00	PROA
ATOM	4526	CB	ASN	P	278	-10.916	6.440	19.057	0.00	0.00	PROA
ATOM	4527	HB1	ASN	P	278	-11.722	5.736	18.757	0.00	0.00	PROA
ATOM	4528	HB2	ASN	P	278	-11.312	7.450	19.297	0.00	0.00	PROA
ATOM	4529	CG	ASN	P	278	-10.268	5.852	20.294	0.00	0.00	PROA
ATOM	4530	OD1	ASN	P	278	-9.045	5.617	20.338	0.00	0.00	PROA
ATOM	4531	ND2	ASN	P	278	-11.094	5.442	21.332	0.00	0.00	PROA
ATOM	4532	HD21	ASN	P	278	-12.088	5.514	21.419	0.00	0.00	PROA
ATOM	4533	HD22	ASN	P	278	-10.711	4.628	21.769	0.00	0.00	PROA
ATOM	4534	C	ASN	P	278	-10.561	6.451	16.602	0.00	0.00	PROA
ATOM	4535	O	ASN	P	278	-10.816	5.460	15.925	0.00	0.00	PROA
ATOM	4536	N	THR	P	279	-10.933	7.642	16.222	0.00	0.00	PROA
ATOM	4537	HN	THR	P	279	-10.865	8.416	16.847	0.00	0.00	PROA
ATOM	4538	CA	THR	P	279	-11.623	7.952	14.945	0.00	0.00	PROA
ATOM	4539	HA	THR	P	279	-12.352	7.155	14.925	0.00	0.00	PROA
ATOM	4540	CB	THR	P	279	-12.352	9.291	14.816	0.00	0.00	PROA
ATOM	4541	HB	THR	P	279	-11.568	10.057	14.637	0.00	0.00	PROA
ATOM	4542	OG1	THR	P	279	-13.127	9.488	15.951	0.00	0.00	PROA
ATOM	4543	HG1	THR	P	279	-12.473	9.487	16.654	0.00	0.00	PROA
ATOM	4544	CG2	THR	P	279	-13.434	9.377	13.657	0.00	0.00	PROA
ATOM	4545	HG21	THR	P	279	-14.162	10.153	13.976	0.00	0.00	PROA
ATOM	4546	HG22	THR	P	279	-13.947	8.394	13.730	0.00	0.00	PROA
ATOM	4547	HG23	THR	P	279	-13.051	9.505	12.622	0.00	0.00	PROA
ATOM	4548	C	THR	P	279	-10.811	7.699	13.698	0.00	0.00	PROA
ATOM	4549	O	THR	P	279	-11.213	7.065	12.717	0.00	0.00	PROA
ATOM	4550	N	VAL	P	280	-9.560	8.107	13.818	0.00	0.00	PROA
ATOM	4551	HN	VAL	P	280	-9.277	8.620	14.624	0.00	0.00	PROA
ATOM	4552	CA	VAL	P	280	-8.496	7.960	12.832	0.00	0.00	PROA
ATOM	4553	HA	VAL	P	280	-8.912	8.159	11.855	0.00	0.00	PROA
ATOM	4554	CB	VAL	P	280	-7.332	8.845	13.026	0.00	0.00	PROA
ATOM	4555	HB	VAL	P	280	-7.103	8.797	14.112	0.00	0.00	PROA
ATOM	4556	CG1	VAL	P	280	-5.992	8.460	12.298	0.00	0.00	PROA
ATOM	4557	HG11	VAL	P	280	-6.190	8.527	11.207	0.00	0.00	PROA
ATOM	4558	HG12	VAL	P	280	-5.549	7.526	12.708	0.00	0.00	PROA
ATOM	4559	HG13	VAL	P	280	-5.221	9.242	12.467	0.00	0.00	PROA
ATOM	4560	CG2	VAL	P	280	-7.815	10.260	12.596	0.00	0.00	PROA
ATOM	4561	HG21	VAL	P	280	-8.103	10.287	11.523	0.00	0.00	PROA
ATOM	4562	HG22	VAL	P	280	-7.060	11.070	12.685	0.00	0.00	PROA
ATOM	4563	HG23	VAL	P	280	-8.682	10.538	13.234	0.00	0.00	PROA
ATOM	4564	C	VAL	P	280	-8.016	6.502	12.648	0.00	0.00	PROA
ATOM	4565	O	VAL	P	280	-7.975	6.074	11.501	0.00	0.00	PROA
ATOM	4566	N	HSE	P	281	-7.832	5.674	13.688	0.00	0.00	PROA
ATOM	4567	HN	HSE	P	281	-7.814	6.081	14.598	0.00	0.00	PROA
ATOM	4568	CA	HSE	P	281	-7.617	4.238	13.735	0.00	0.00	PROA
ATOM	4569	HA	HSE	P	281	-6.717	3.830	13.300	0.00	0.00	PROA
ATOM	4570	CB	HSE	P	281	-7.599	3.691	15.197	0.00	0.00	PROA
ATOM	4571	HB1	HSE	P	281	-8.532	3.791	15.791	0.00	0.00	PROA

ATOM	4572	HB2	HSE	P	281	-6.744	4.196	15.696	0.00	0.00	PROA
ATOM	4573	ND1	HSE	P	281	-7.175	1.746	16.599	0.00	0.00	PROA
ATOM	4574	CG	HSE	P	281	-7.117	2.304	15.308	0.00	0.00	PROA
ATOM	4575	CE1	HSE	P	281	-6.721	0.512	16.418	0.00	0.00	PROA
ATOM	4576	HE1	HSE	P	281	-6.803	-0.308	17.132	0.00	0.00	PROA
ATOM	4577	NE2	HSE	P	281	-6.403	0.252	15.125	0.00	0.00	PROA
ATOM	4578	HE2	HSE	P	281	-5.912	-0.549	14.784	0.00	0.00	PROA
ATOM	4579	CD2	HSE	P	281	-6.511	1.433	14.416	0.00	0.00	PROA
ATOM	4580	HD2	HSE	P	281	-6.260	1.608	13.378	0.00	0.00	PROA
ATOM	4581	C	HSE	P	281	-8.734	3.440	13.076	0.00	0.00	PROA
ATOM	4582	O	HSE	P	281	-8.468	2.471	12.379	0.00	0.00	PROA
ATOM	4583	N	LYS	P	282	-10.006	3.953	13.306	0.00	0.00	PROA
ATOM	4584	HN	LYS	P	282	-10.089	4.832	13.770	0.00	0.00	PROA
ATOM	4585	CA	LYS	P	282	-11.234	3.275	12.926	0.00	0.00	PROA
ATOM	4586	HA	LYS	P	282	-11.196	2.271	13.321	0.00	0.00	PROA
ATOM	4587	CB	LYS	P	282	-12.476	4.000	13.525	0.00	0.00	PROA
ATOM	4588	HB1	LYS	P	282	-12.437	5.100	13.379	0.00	0.00	PROA
ATOM	4589	HB2	LYS	P	282	-12.365	3.885	14.624	0.00	0.00	PROA
ATOM	4590	CG	LYS	P	282	-13.934	3.598	13.171	0.00	0.00	PROA
ATOM	4591	HG1	LYS	P	282	-14.167	3.792	12.103	0.00	0.00	PROA
ATOM	4592	HG2	LYS	P	282	-14.673	4.183	13.760	0.00	0.00	PROA
ATOM	4593	CD	LYS	P	282	-14.224	2.143	13.429	0.00	0.00	PROA
ATOM	4594	HD1	LYS	P	282	-13.455	1.539	12.902	0.00	0.00	PROA
ATOM	4595	HD2	LYS	P	282	-15.220	1.897	13.003	0.00	0.00	PROA
ATOM	4596	CE	LYS	P	282	-14.298	1.706	14.927	0.00	0.00	PROA
ATOM	4597	HE1	LYS	P	282	-15.141	2.321	15.307	0.00	0.00	PROA
ATOM	4598	HE2	LYS	P	282	-13.346	1.684	15.499	0.00	0.00	PROA
ATOM	4599	NZ	LYS	P	282	-14.730	0.314	15.060	0.00	0.00	PROA
ATOM	4600	HZ1	LYS	P	282	-13.948	-0.295	14.745	0.00	0.00	PROA
ATOM	4601	HZ2	LYS	P	282	-15.685	0.080	14.719	0.00	0.00	PROA
ATOM	4602	HZ3	LYS	P	282	-14.788	0.138	16.083	0.00	0.00	PROA
ATOM	4603	C	LYS	P	282	-11.376	3.111	11.483	0.00	0.00	PROA
ATOM	4604	O	LYS	P	282	-11.669	2.089	10.964	0.00	0.00	PROA
ATOM	4605	N	TRP	P	283	-11.095	4.224	10.726	0.00	0.00	PROA
ATOM	4606	HN	TRP	P	283	-10.932	5.069	11.231	0.00	0.00	PROA
ATOM	4607	CA	TRP	P	283	-10.979	4.255	9.311	0.00	0.00	PROA
ATOM	4608	HA	TRP	P	283	-11.897	3.814	8.950	0.00	0.00	PROA
ATOM	4609	CB	TRP	P	283	-11.077	5.724	8.797	0.00	0.00	PROA
ATOM	4610	HB1	TRP	P	283	-10.814	5.942	7.740	0.00	0.00	PROA
ATOM	4611	HB2	TRP	P	283	-10.302	6.246	9.398	0.00	0.00	PROA
ATOM	4612	CG	TRP	P	283	-12.474	6.262	9.080	0.00	0.00	PROA
ATOM	4613	CD1	TRP	P	283	-12.881	7.089	10.122	0.00	0.00	PROA
ATOM	4614	HD1	TRP	P	283	-12.160	7.602	10.741	0.00	0.00	PROA
ATOM	4615	NE1	TRP	P	283	-14.252	7.183	9.983	0.00	0.00	PROA
ATOM	4616	HE1	TRP	P	283	-14.878	7.624	10.589	0.00	0.00	PROA
ATOM	4617	CE2	TRP	P	283	-14.704	6.466	8.878	0.00	0.00	PROA
ATOM	4618	CD2	TRP	P	283	-13.616	5.877	8.282	0.00	0.00	PROA
ATOM	4619	CE3	TRP	P	283	-13.767	5.077	7.144	0.00	0.00	PROA
ATOM	4620	HE3	TRP	P	283	-12.948	4.678	6.564	0.00	0.00	PROA
ATOM	4621	CZ3	TRP	P	283	-15.095	4.968	6.643	0.00	0.00	PROA
ATOM	4622	HZ3	TRP	P	283	-15.280	4.291	5.822	0.00	0.00	PROA
ATOM	4623	CZ2	TRP	P	283	-16.016	6.300	8.434	0.00	0.00	PROA
ATOM	4624	HZ2	TRP	P	283	-16.926	6.643	8.904	0.00	0.00	PROA
ATOM	4625	CH2	TRP	P	283	-16.164	5.495	7.342	0.00	0.00	PROA
ATOM	4626	HH2	TRP	P	283	-17.132	5.185	6.977	0.00	0.00	PROA
ATOM	4627	C	TRP	P	283	-9.840	3.490	8.648	0.00	0.00	PROA
ATOM	4628	O	TRP	P	283	-10.055	2.754	7.628	0.00	0.00	PROA
ATOM	4629	N	ILE	P	284	-8.662	3.420	9.333	0.00	0.00	PROA
ATOM	4630	HN	ILE	P	284	-8.559	3.972	10.157	0.00	0.00	PROA
ATOM	4631	CA	ILE	P	284	-7.549	2.535	8.978	0.00	0.00	PROA
ATOM	4632	HA	ILE	P	284	-7.235	2.837	7.989	0.00	0.00	PROA

ATOM	4633	CB	ILE	P	284	-6.436	2.888	9.884	0.00	0.00	PROA
ATOM	4634	HB	ILE	P	284	-6.841	3.038	10.907	0.00	0.00	PROA
ATOM	4635	CG2	ILE	P	284	-5.336	1.806	9.827	0.00	0.00	PROA
ATOM	4636	HG21	ILE	P	284	-4.895	1.828	8.808	0.00	0.00	PROA
ATOM	4637	HG22	ILE	P	284	-5.653	0.767	10.060	0.00	0.00	PROA
ATOM	4638	HG23	ILE	P	284	-4.584	2.077	10.599	0.00	0.00	PROA
ATOM	4639	CG1	ILE	P	284	-5.784	4.209	9.569	0.00	0.00	PROA
ATOM	4640	HG11	ILE	P	284	-6.683	4.861	9.585	0.00	0.00	PROA
ATOM	4641	HG12	ILE	P	284	-5.353	4.280	8.547	0.00	0.00	PROA
ATOM	4642	CD	ILE	P	284	-4.803	4.723	10.601	0.00	0.00	PROA
ATOM	4643	HD1	ILE	P	284	-5.304	4.731	11.592	0.00	0.00	PROA
ATOM	4644	HD2	ILE	P	284	-4.440	5.745	10.361	0.00	0.00	PROA
ATOM	4645	HD3	ILE	P	284	-3.857	4.141	10.614	0.00	0.00	PROA
ATOM	4646	C	ILE	P	284	-7.924	1.070	8.974	0.00	0.00	PROA
ATOM	4647	O	ILE	P	284	-7.693	0.357	8.009	0.00	0.00	PROA
ATOM	4648	N	SER	P	285	-8.670	0.608	9.988	0.00	0.00	PROA
ATOM	4649	HN	SER	P	285	-8.623	1.192	10.795	0.00	0.00	PROA
ATOM	4650	CA	SER	P	285	-9.126	-0.772	10.140	0.00	0.00	PROA
ATOM	4651	HA	SER	P	285	-8.318	-1.471	9.978	0.00	0.00	PROA
ATOM	4652	CB	SER	P	285	-9.789	-1.080	11.534	0.00	0.00	PROA
ATOM	4653	HB1	SER	P	285	-9.929	-2.161	11.749	0.00	0.00	PROA
ATOM	4654	HB2	SER	P	285	-10.777	-0.581	11.634	0.00	0.00	PROA
ATOM	4655	OG	SER	P	285	-8.999	-0.587	12.613	0.00	0.00	PROA
ATOM	4656	HG1	SER	P	285	-8.109	-0.768	12.301	0.00	0.00	PROA
ATOM	4657	C	SER	P	285	-10.136	-1.098	9.003	0.00	0.00	PROA
ATOM	4658	O	SER	P	285	-10.182	-2.171	8.393	0.00	0.00	PROA
ATOM	4659	N	ILE	P	286	-11.067	-0.187	8.715	0.00	0.00	PROA
ATOM	4660	HN	ILE	P	286	-11.016	0.653	9.250	0.00	0.00	PROA
ATOM	4661	CA	ILE	P	286	-12.030	-0.373	7.599	0.00	0.00	PROA
ATOM	4662	HA	ILE	P	286	-12.506	-1.337	7.701	0.00	0.00	PROA
ATOM	4663	CB	ILE	P	286	-13.057	0.776	7.601	0.00	0.00	PROA
ATOM	4664	HB	ILE	P	286	-12.500	1.735	7.671	0.00	0.00	PROA
ATOM	4665	CG2	ILE	P	286	-13.905	0.860	6.347	0.00	0.00	PROA
ATOM	4666	HG21	ILE	P	286	-14.447	-0.070	6.072	0.00	0.00	PROA
ATOM	4667	HG22	ILE	P	286	-13.224	1.082	5.498	0.00	0.00	PROA
ATOM	4668	HG23	ILE	P	286	-14.589	1.735	6.333	0.00	0.00	PROA
ATOM	4669	CG1	ILE	P	286	-14.019	0.695	8.770	0.00	0.00	PROA
ATOM	4670	HG11	ILE	P	286	-13.525	0.132	9.591	0.00	0.00	PROA
ATOM	4671	HG12	ILE	P	286	-14.951	0.179	8.454	0.00	0.00	PROA
ATOM	4672	CD	ILE	P	286	-14.425	2.065	9.372	0.00	0.00	PROA
ATOM	4673	HD1	ILE	P	286	-15.053	1.849	10.262	0.00	0.00	PROA
ATOM	4674	HD2	ILE	P	286	-14.976	2.660	8.613	0.00	0.00	PROA
ATOM	4675	HD3	ILE	P	286	-13.602	2.793	9.537	0.00	0.00	PROA
ATOM	4676	C	ILE	P	286	-11.320	-0.389	6.223	0.00	0.00	PROA
ATOM	4677	O	ILE	P	286	-11.482	-1.302	5.439	0.00	0.00	PROA
ATOM	4678	N	THR	P	287	-10.526	0.608	5.923	0.00	0.00	PROA
ATOM	4679	HN	THR	P	287	-10.390	1.378	6.542	0.00	0.00	PROA
ATOM	4680	CA	THR	P	287	-9.852	0.724	4.647	0.00	0.00	PROA
ATOM	4681	HA	THR	P	287	-10.623	0.509	3.922	0.00	0.00	PROA
ATOM	4682	CB	THR	P	287	-9.395	2.172	4.334	0.00	0.00	PROA
ATOM	4683	HB	THR	P	287	-8.756	2.227	3.427	0.00	0.00	PROA
ATOM	4684	OG1	THR	P	287	-8.647	2.807	5.429	0.00	0.00	PROA
ATOM	4685	HG1	THR	P	287	-9.319	3.137	6.029	0.00	0.00	PROA
ATOM	4686	CG2	THR	P	287	-10.683	2.978	3.985	0.00	0.00	PROA
ATOM	4687	HG21	THR	P	287	-11.352	2.987	4.872	0.00	0.00	PROA
ATOM	4688	HG22	THR	P	287	-11.282	2.628	3.118	0.00	0.00	PROA
ATOM	4689	HG23	THR	P	287	-10.456	4.032	3.718	0.00	0.00	PROA
ATOM	4690	C	THR	P	287	-8.676	-0.204	4.396	0.00	0.00	PROA
ATOM	4691	O	THR	P	287	-8.420	-0.635	3.239	0.00	0.00	PROA
ATOM	4692	N	GLU	P	288	-8.035	-0.676	5.485	0.00	0.00	PROA
ATOM	4693	HN	GLU	P	288	-8.187	-0.266	6.381	0.00	0.00	PROA

ATOM	4694	CA	GLU	P	288	-7.001	-1.665	5.421	0.00	0.00	PROA
ATOM	4695	HA	GLU	P	288	-6.322	-1.291	4.668	0.00	0.00	PROA
ATOM	4696	CB	GLU	P	288	-6.355	-1.946	6.840	0.00	0.00	PROA
ATOM	4697	HB1	GLU	P	288	-7.027	-2.552	7.484	0.00	0.00	PROA
ATOM	4698	HB2	GLU	P	288	-6.064	-0.984	7.314	0.00	0.00	PROA
ATOM	4699	CG	GLU	P	288	-4.955	-2.641	6.704	0.00	0.00	PROA
ATOM	4700	HG1	GLU	P	288	-4.341	-2.022	6.016	0.00	0.00	PROA
ATOM	4701	HG2	GLU	P	288	-4.982	-3.658	6.257	0.00	0.00	PROA
ATOM	4702	CD	GLU	P	288	-4.199	-2.801	8.009	0.00	0.00	PROA
ATOM	4703	OE1	GLU	P	288	-4.477	-3.781	8.752	0.00	0.00	PROA
ATOM	4704	OE2	GLU	P	288	-3.216	-2.006	8.391	0.00	0.00	PROA
ATOM	4705	C	GLU	P	288	-7.528	-3.031	4.994	0.00	0.00	PROA
ATOM	4706	O	GLU	P	288	-6.988	-3.744	4.158	0.00	0.00	PROA
ATOM	4707	N	ALA	P	289	-8.635	-3.417	5.620	0.00	0.00	PROA
ATOM	4708	HN	ALA	P	289	-9.077	-3.031	6.426	0.00	0.00	PROA
ATOM	4709	CA	ALA	P	289	-9.459	-4.555	5.166	0.00	0.00	PROA
ATOM	4710	HA	ALA	P	289	-8.801	-5.411	5.192	0.00	0.00	PROA
ATOM	4711	CB	ALA	P	289	-10.530	-4.680	6.277	0.00	0.00	PROA
ATOM	4712	HB1	ALA	P	289	-11.161	-3.771	6.379	0.00	0.00	PROA
ATOM	4713	HB2	ALA	P	289	-10.071	-4.718	7.288	0.00	0.00	PROA
ATOM	4714	HB3	ALA	P	289	-11.128	-5.589	6.050	0.00	0.00	PROA
ATOM	4715	C	ALA	P	289	-10.104	-4.499	3.815	0.00	0.00	PROA
ATOM	4716	O	ALA	P	289	-10.096	-5.422	2.982	0.00	0.00	PROA
ATOM	4717	N	LEU	P	290	-10.599	-3.323	3.451	0.00	0.00	PROA
ATOM	4718	HN	LEU	P	290	-10.623	-2.579	4.114	0.00	0.00	PROA
ATOM	4719	CA	LEU	P	290	-11.136	-3.070	2.103	0.00	0.00	PROA
ATOM	4720	HA	LEU	P	290	-11.839	-3.816	1.763	0.00	0.00	PROA
ATOM	4721	CB	LEU	P	290	-11.805	-1.641	2.102	0.00	0.00	PROA
ATOM	4722	HB1	LEU	P	290	-11.047	-0.873	2.366	0.00	0.00	PROA
ATOM	4723	HB2	LEU	P	290	-12.510	-1.647	2.960	0.00	0.00	PROA
ATOM	4724	CG	LEU	P	290	-12.402	-1.130	0.752	0.00	0.00	PROA
ATOM	4725	HG	LEU	P	290	-11.571	-0.891	0.055	0.00	0.00	PROA
ATOM	4726	CD1	LEU	P	290	-13.279	-2.233	0.175	0.00	0.00	PROA
ATOM	4727	HD11	LEU	P	290	-14.156	-2.423	0.830	0.00	0.00	PROA
ATOM	4728	HD12	LEU	P	290	-12.716	-3.178	0.025	0.00	0.00	PROA
ATOM	4729	HD13	LEU	P	290	-13.658	-1.880	-0.808	0.00	0.00	PROA
ATOM	4730	CD2	LEU	P	290	-13.120	0.211	0.982	0.00	0.00	PROA
ATOM	4731	HD21	LEU	P	290	-13.906	0.108	1.760	0.00	0.00	PROA
ATOM	4732	HD22	LEU	P	290	-13.586	0.520	0.022	0.00	0.00	PROA
ATOM	4733	HD23	LEU	P	290	-12.387	0.962	1.348	0.00	0.00	PROA
ATOM	4734	C	LEU	P	290	-10.105	-3.029	1.043	0.00	0.00	PROA
ATOM	4735	O	LEU	P	290	-10.358	-3.497	-0.081	0.00	0.00	PROA
ATOM	4736	N	ALA	P	291	-8.811	-2.670	1.340	0.00	0.00	PROA
ATOM	4737	HN	ALA	P	291	-8.632	-2.226	2.214	0.00	0.00	PROA
ATOM	4738	CA	ALA	P	291	-7.605	-2.804	0.523	0.00	0.00	PROA
ATOM	4739	HA	ALA	P	291	-7.730	-2.271	-0.408	0.00	0.00	PROA
ATOM	4740	CB	ALA	P	291	-6.445	-2.258	1.264	0.00	0.00	PROA
ATOM	4741	HB1	ALA	P	291	-5.592	-2.970	1.293	0.00	0.00	PROA
ATOM	4742	HB2	ALA	P	291	-6.680	-2.063	2.332	0.00	0.00	PROA
ATOM	4743	HB3	ALA	P	291	-6.033	-1.326	0.821	0.00	0.00	PROA
ATOM	4744	C	ALA	P	291	-7.364	-4.268	0.170	0.00	0.00	PROA
ATOM	4745	O	ALA	P	291	-7.254	-4.597	-0.971	0.00	0.00	PROA
ATOM	4746	N	PHE	P	292	-7.407	-5.188	1.080	0.00	0.00	PROA
ATOM	4747	HN	PHE	P	292	-7.244	-5.123	2.061	0.00	0.00	PROA
ATOM	4748	CA	PHE	P	292	-7.420	-6.588	0.881	0.00	0.00	PROA
ATOM	4749	HA	PHE	P	292	-6.585	-6.881	0.261	0.00	0.00	PROA
ATOM	4750	CB	PHE	P	292	-7.131	-7.314	2.217	0.00	0.00	PROA
ATOM	4751	HB1	PHE	P	292	-7.045	-8.365	1.866	0.00	0.00	PROA
ATOM	4752	HB2	PHE	P	292	-7.951	-7.172	2.953	0.00	0.00	PROA
ATOM	4753	CG	PHE	P	292	-5.818	-7.003	2.906	0.00	0.00	PROA
ATOM	4754	CD1	PHE	P	292	-5.857	-6.492	4.271	0.00	0.00	PROA

ATOM	4755	HD1	PHE	P	292	-6.814	-6.142	4.629	0.00	0.00	PROA
ATOM	4756	CE1	PHE	P	292	-4.637	-6.331	4.967	0.00	0.00	PROA
ATOM	4757	HE1	PHE	P	292	-4.678	-5.913	5.962	0.00	0.00	PROA
ATOM	4758	CZ	PHE	P	292	-3.440	-6.535	4.310	0.00	0.00	PROA
ATOM	4759	HZ	PHE	P	292	-2.502	-6.185	4.713	0.00	0.00	PROA
ATOM	4760	CD2	PHE	P	292	-4.609	-7.277	2.280	0.00	0.00	PROA
ATOM	4761	HD2	PHE	P	292	-4.591	-7.630	1.259	0.00	0.00	PROA
ATOM	4762	CE2	PHE	P	292	-3.418	-7.044	3.016	0.00	0.00	PROA
ATOM	4763	HE2	PHE	P	292	-2.489	-7.359	2.563	0.00	0.00	PROA
ATOM	4764	C	PHE	P	292	-8.628	-7.218	0.244	0.00	0.00	PROA
ATOM	4765	O	PHE	P	292	-8.469	-8.148	-0.542	0.00	0.00	PROA
ATOM	4766	N	PHE	P	293	-9.863	-6.694	0.408	0.00	0.00	PROA
ATOM	4767	HN	PHE	P	293	-9.991	-5.980	1.092	0.00	0.00	PROA
ATOM	4768	CA	PHE	P	293	-10.927	-6.990	-0.598	0.00	0.00	PROA
ATOM	4769	HA	PHE	P	293	-10.914	-8.065	-0.694	0.00	0.00	PROA
ATOM	4770	CB	PHE	P	293	-12.300	-6.792	0.081	0.00	0.00	PROA
ATOM	4771	HB1	PHE	P	293	-12.343	-5.757	0.484	0.00	0.00	PROA
ATOM	4772	HB2	PHE	P	293	-12.395	-7.459	0.964	0.00	0.00	PROA
ATOM	4773	CG	PHE	P	293	-13.497	-7.061	-0.859	0.00	0.00	PROA
ATOM	4774	CD1	PHE	P	293	-13.650	-8.313	-1.426	0.00	0.00	PROA
ATOM	4775	HD1	PHE	P	293	-12.978	-9.110	-1.145	0.00	0.00	PROA
ATOM	4776	CE1	PHE	P	293	-14.750	-8.530	-2.238	0.00	0.00	PROA
ATOM	4777	HE1	PHE	P	293	-14.884	-9.471	-2.751	0.00	0.00	PROA
ATOM	4778	CZ	PHE	P	293	-15.756	-7.504	-2.465	0.00	0.00	PROA
ATOM	4779	HZ	PHE	P	293	-16.657	-7.674	-3.035	0.00	0.00	PROA
ATOM	4780	CD2	PHE	P	293	-14.437	-6.055	-0.992	0.00	0.00	PROA
ATOM	4781	HD2	PHE	P	293	-14.328	-5.101	-0.498	0.00	0.00	PROA
ATOM	4782	CE2	PHE	P	293	-15.567	-6.289	-1.876	0.00	0.00	PROA
ATOM	4783	HE2	PHE	P	293	-16.387	-5.588	-1.924	0.00	0.00	PROA
ATOM	4784	C	PHE	P	293	-10.727	-6.483	-2.024	0.00	0.00	PROA
ATOM	4785	O	PHE	P	293	-10.833	-7.273	-2.950	0.00	0.00	PROA
ATOM	4786	N	HSD	P	294	-10.258	-5.226	-2.199	0.00	0.00	PROA
ATOM	4787	HN	HSD	P	294	-9.996	-4.652	-1.427	0.00	0.00	PROA
ATOM	4788	CA	HSD	P	294	-9.843	-4.668	-3.504	0.00	0.00	PROA
ATOM	4789	HA	HSD	P	294	-10.561	-4.806	-4.299	0.00	0.00	PROA
ATOM	4790	CB	HSD	P	294	-9.322	-3.196	-3.301	0.00	0.00	PROA
ATOM	4791	HB1	HSD	P	294	-8.387	-3.184	-2.700	0.00	0.00	PROA
ATOM	4792	HB2	HSD	P	294	-10.014	-2.572	-2.696	0.00	0.00	PROA
ATOM	4793	ND1	HSD	P	294	-7.847	-2.144	-5.092	0.00	0.00	PROA
ATOM	4794	HD1	HSD	P	294	-6.951	-2.363	-4.707	0.00	0.00	PROA
ATOM	4795	CG	HSD	P	294	-9.089	-2.510	-4.632	0.00	0.00	PROA
ATOM	4796	CE1	HSD	P	294	-7.975	-1.478	-6.223	0.00	0.00	PROA
ATOM	4797	HE1	HSD	P	294	-7.124	-1.033	-6.739	0.00	0.00	PROA
ATOM	4798	NE2	HSD	P	294	-9.258	-1.488	-6.631	0.00	0.00	PROA
ATOM	4799	CD2	HSD	P	294	-9.972	-2.197	-5.660	0.00	0.00	PROA
ATOM	4800	HD2	HSD	P	294	-11.054	-2.175	-5.669	0.00	0.00	PROA
ATOM	4801	C	HSD	P	294	-8.700	-5.487	-4.205	0.00	0.00	PROA
ATOM	4802	O	HSD	P	294	-8.634	-5.679	-5.411	0.00	0.00	PROA
ATOM	4803	N	CYS	P	295	-7.758	-6.118	-3.432	0.00	0.00	PROA
ATOM	4804	HN	CYS	P	295	-7.703	-5.920	-2.456	0.00	0.00	PROA
ATOM	4805	CA	CYS	P	295	-6.789	-7.056	-3.962	0.00	0.00	PROA
ATOM	4806	HA	CYS	P	295	-6.176	-6.669	-4.763	0.00	0.00	PROA
ATOM	4807	CB	CYS	P	295	-5.792	-7.643	-2.897	0.00	0.00	PROA
ATOM	4808	HB1	CYS	P	295	-5.184	-8.470	-3.320	0.00	0.00	PROA
ATOM	4809	HB2	CYS	P	295	-6.361	-8.082	-2.049	0.00	0.00	PROA
ATOM	4810	SG	CYS	P	295	-4.766	-6.303	-2.065	0.00	0.00	PROA
ATOM	4811	HG1	CYS	P	295	-4.024	-6.037	-3.130	0.00	0.00	PROA
ATOM	4812	C	CYS	P	295	-7.433	-8.354	-4.576	0.00	0.00	PROA
ATOM	4813	O	CYS	P	295	-6.995	-8.971	-5.602	0.00	0.00	PROA
ATOM	4814	N	CYS	P	296	-8.509	-8.888	-3.884	0.00	0.00	PROA
ATOM	4815	HN	CYS	P	296	-8.870	-8.349	-3.127	0.00	0.00	PROA

```
ATOM   4816  CA  CYS P 296      -9.394 -10.002  -4.227  0.00  0.00      PROA
ATOM   4817  HA  CYS P 296      -8.746 -10.860  -4.332  0.00  0.00      PROA
ATOM   4818  CB  CYS P 296     -10.331 -10.266  -2.992  0.00  0.00      PROA
ATOM   4819  HB1 CYS P 296     -11.121  -9.490  -2.914  0.00  0.00      PROA
ATOM   4820  HB2 CYS P 296      -9.691 -10.325  -2.086  0.00  0.00      PROA
ATOM   4821  SG  CYS P 296     -10.944 -11.937  -3.032  0.00  0.00      PROA
ATOM   4822  HG1 CYS P 296     -11.482 -11.843  -4.239  0.00  0.00      PROA
ATOM   4823  C   CYS P 296     -10.095  -9.731  -5.580  0.00  0.00      PROA
ATOM   4824  O   CYS P 296     -10.220 -10.591  -6.420  0.00  0.00      PROA
ATOM   4825  N   LEU P 297     -10.549  -8.516  -5.838  0.00  0.00      PROA
ATOM   4826  HN  LEU P 297     -10.286  -7.783  -5.214  0.00  0.00      PROA
ATOM   4827  CA  LEU P 297     -11.365  -8.149  -6.993  0.00  0.00      PROA
ATOM   4828  HA  LEU P 297     -12.094  -8.936  -7.120  0.00  0.00      PROA
ATOM   4829  CB  LEU P 297     -12.136  -6.905  -6.673  0.00  0.00      PROA
ATOM   4830  HB1 LEU P 297     -12.655  -6.498  -7.567  0.00  0.00      PROA
ATOM   4831  HB2 LEU P 297     -11.472  -6.170  -6.170  0.00  0.00      PROA
ATOM   4832  CG  LEU P 297     -13.196  -7.089  -5.558  0.00  0.00      PROA
ATOM   4833  HG  LEU P 297     -12.880  -7.730  -4.707  0.00  0.00      PROA
ATOM   4834  CD1 LEU P 297     -13.528  -5.702  -4.981  0.00  0.00      PROA
ATOM   4835 HD11 LEU P 297     -13.877  -5.044  -5.805  0.00  0.00      PROA
ATOM   4836 HD12 LEU P 297     -12.608  -5.298  -4.506  0.00  0.00      PROA
ATOM   4837 HD13 LEU P 297     -14.382  -5.791  -4.276  0.00  0.00      PROA
ATOM   4838  CD2 LEU P 297     -14.450  -7.802  -6.037  0.00  0.00      PROA
ATOM   4839 HD21 LEU P 297     -14.213  -8.853  -6.310  0.00  0.00      PROA
ATOM   4840 HD22 LEU P 297     -14.767  -7.124  -6.857  0.00  0.00      PROA
ATOM   4841 HD23 LEU P 297     -15.218  -7.877  -5.238  0.00  0.00      PROA
ATOM   4842  C   LEU P 297     -10.571  -7.996  -8.351  0.00  0.00      PROA
ATOM   4843  O   LEU P 297     -11.086  -8.003  -9.436  0.00  0.00      PROA
ATOM   4844  N   ASN P 298      -9.241  -7.880  -8.272  0.00  0.00      PROA
ATOM   4845  HN  ASN P 298      -8.802  -7.950  -7.380  0.00  0.00      PROA
ATOM   4846  CA  ASN P 298      -8.340  -7.791  -9.422  0.00  0.00      PROA
ATOM   4847  HA  ASN P 298      -8.728  -7.051 -10.107  0.00  0.00      PROA
ATOM   4848  CB  ASN P 298      -6.876  -7.357  -8.981  0.00  0.00      PROA
ATOM   4849  HB1 ASN P 298      -6.539  -7.943  -8.099  0.00  0.00      PROA
ATOM   4850  HB2 ASN P 298      -7.010  -6.288  -8.708  0.00  0.00      PROA
ATOM   4851  CG  ASN P 298      -5.716  -7.392  -9.921  0.00  0.00      PROA
ATOM   4852  OD1 ASN P 298      -5.904  -7.740 -11.116  0.00  0.00      PROA
ATOM   4853  ND2 ASN P 298      -4.657  -6.691  -9.496  0.00  0.00      PROA
ATOM   4854 HD21 ASN P 298      -4.607  -6.427  -8.532  0.00  0.00      PROA
ATOM   4855 HD22 ASN P 298      -4.006  -6.538 -10.239  0.00  0.00      PROA
ATOM   4856  C   ASN P 298      -8.337  -9.058 -10.306  0.00  0.00      PROA
ATOM   4857  O   ASN P 298      -8.569  -8.911 -11.482  0.00  0.00      PROA
ATOM   4858  N   PRO P 299      -8.090 -10.335  -9.826  0.00  0.00      PROA
ATOM   4859  CD  PRO P 299      -7.275 -10.629  -8.651  0.00  0.00      PROA
ATOM   4860  HD1 PRO P 299      -7.747 -10.083  -7.807  0.00  0.00      PROA
ATOM   4861  HD2 PRO P 299      -6.199 -10.366  -8.741  0.00  0.00      PROA
ATOM   4862  CA  PRO P 299      -8.321 -11.478 -10.670  0.00  0.00      PROA
ATOM   4863  HA  PRO P 299      -7.806 -11.235 -11.587  0.00  0.00      PROA
ATOM   4864  CB  PRO P 299      -7.713 -12.656  -9.885  0.00  0.00      PROA
ATOM   4865  HB1 PRO P 299      -6.701 -12.829 -10.310  0.00  0.00      PROA
ATOM   4866  HB2 PRO P 299      -8.214 -13.629 -10.077  0.00  0.00      PROA
ATOM   4867  CG  PRO P 299      -7.492 -12.144  -8.452  0.00  0.00      PROA
ATOM   4868  HG1 PRO P 299      -8.314 -12.265  -7.714  0.00  0.00      PROA
ATOM   4869  HG2 PRO P 299      -6.645 -12.629  -7.922  0.00  0.00      PROA
ATOM   4870  C   PRO P 299      -9.851 -11.710 -10.878  0.00  0.00      PROA
ATOM   4871  O   PRO P 299     -10.133 -12.301 -11.903  0.00  0.00      PROA
ATOM   4872  N   ILE P 300     -10.695 -11.354  -9.895  0.00  0.00      PROA
ATOM   4873  HN  ILE P 300     -10.377 -10.991  -9.023  0.00  0.00      PROA
ATOM   4874  CA  ILE P 300     -12.173 -11.601 -10.053  0.00  0.00      PROA
ATOM   4875  HA  ILE P 300     -12.291 -12.655 -10.257  0.00  0.00      PROA
ATOM   4876  CB  ILE P 300     -12.860 -11.366  -8.643  0.00  0.00      PROA
```

```
ATOM   4877  HB  ILE P 300    -12.387 -10.510  -8.115  0.00  0.00      PROA
ATOM   4878  CG2 ILE P 300    -14.439 -11.039  -8.769  0.00  0.00      PROA
ATOM   4879 HG21 ILE P 300    -14.562 -10.038  -9.234  0.00  0.00      PROA
ATOM   4880 HG22 ILE P 300    -14.851 -10.916  -7.745  0.00  0.00      PROA
ATOM   4881 HG23 ILE P 300    -14.970 -11.835  -9.333  0.00  0.00      PROA
ATOM   4882  CG1 ILE P 300    -12.575 -12.534  -7.686  0.00  0.00      PROA
ATOM   4883 HG11 ILE P 300    -11.478 -12.549  -7.512  0.00  0.00      PROA
ATOM   4884 HG12 ILE P 300    -12.724 -13.490  -8.231  0.00  0.00      PROA
ATOM   4885  CD  ILE P 300    -13.406 -12.516  -6.392  0.00  0.00      PROA
ATOM   4886  HD1 ILE P 300    -14.490 -12.620  -6.616  0.00  0.00      PROA
ATOM   4887  HD2 ILE P 300    -13.204 -11.572  -5.842  0.00  0.00      PROA
ATOM   4888  HD3 ILE P 300    -13.088 -13.419  -5.829  0.00  0.00      PROA
ATOM   4889  C   ILE P 300    -12.917 -10.785 -11.150  0.00  0.00      PROA
ATOM   4890  O   ILE P 300    -13.844 -11.275 -11.798  0.00  0.00      PROA
ATOM   4891  N   LEU P 301    -12.504  -9.521 -11.352  0.00  0.00      PROA
ATOM   4892  HN  LEU P 301    -11.904  -9.101 -10.675  0.00  0.00      PROA
ATOM   4893  CA  LEU P 301    -13.132  -8.644 -12.246  0.00  0.00      PROA
ATOM   4894  HA  LEU P 301    -14.146  -8.950 -12.456  0.00  0.00      PROA
ATOM   4895  CB  LEU P 301    -13.060  -7.158 -11.700  0.00  0.00      PROA
ATOM   4896  HB1 LEU P 301    -13.134  -6.465 -12.566  0.00  0.00      PROA
ATOM   4897  HB2 LEU P 301    -12.025  -6.938 -11.362  0.00  0.00      PROA
ATOM   4898  CG  LEU P 301    -13.964  -6.785 -10.537  0.00  0.00      PROA
ATOM   4899  HG  LEU P 301    -13.819  -7.601  -9.798  0.00  0.00      PROA
ATOM   4900  CD1 LEU P 301    -13.583  -5.391 -10.099  0.00  0.00      PROA
ATOM   4901 HD11 LEU P 301    -12.478  -5.316 -10.021  0.00  0.00      PROA
ATOM   4902 HD12 LEU P 301    -13.929  -5.193  -9.061  0.00  0.00      PROA
ATOM   4903 HD13 LEU P 301    -13.945  -4.628 -10.820  0.00  0.00      PROA
ATOM   4904  CD2 LEU P 301    -15.467  -7.021 -11.018  0.00  0.00      PROA
ATOM   4905 HD21 LEU P 301    -15.548  -8.101 -11.265  0.00  0.00      PROA
ATOM   4906 HD22 LEU P 301    -15.721  -6.397 -11.902  0.00  0.00      PROA
ATOM   4907 HD23 LEU P 301    -16.155  -6.800 -10.174  0.00  0.00      PROA
ATOM   4908  C   LEU P 301    -12.453  -8.746 -13.515  0.00  0.00      PROA
ATOM   4909  O   LEU P 301    -13.039  -8.293 -14.497  0.00  0.00      PROA
ATOM   4910  N   TYR P 302    -11.339  -9.408 -13.661  0.00  0.00      PROA
ATOM   4911  HN  TYR P 302    -10.915  -9.818 -12.857  0.00  0.00      PROA
ATOM   4912  CA  TYR P 302    -10.727  -9.749 -14.867  0.00  0.00      PROA
ATOM   4913  HA  TYR P 302    -10.943  -8.998 -15.612  0.00  0.00      PROA
ATOM   4914  CB  TYR P 302     -9.219  -9.865 -14.616  0.00  0.00      PROA
ATOM   4915  HB1 TYR P 302     -8.975 -10.854 -14.175  0.00  0.00      PROA
ATOM   4916  HB2 TYR P 302     -8.962  -9.039 -13.918  0.00  0.00      PROA
ATOM   4917  CG  TYR P 302     -8.468  -9.579 -15.899  0.00  0.00      PROA
ATOM   4918  CD1 TYR P 302     -8.597  -8.351 -16.498  0.00  0.00      PROA
ATOM   4919  HD1 TYR P 302     -9.202  -7.611 -15.996  0.00  0.00      PROA
ATOM   4920  CE1 TYR P 302     -8.089  -8.114 -17.736  0.00  0.00      PROA
ATOM   4921  HE1 TYR P 302     -8.171  -7.140 -18.196  0.00  0.00      PROA
ATOM   4922  CZ  TYR P 302     -7.190  -9.134 -18.339  0.00  0.00      PROA
ATOM   4923  OH  TYR P 302     -6.383  -8.906 -19.514  0.00  0.00      PROA
ATOM   4924  HH  TYR P 302     -6.556  -8.052 -19.917  0.00  0.00      PROA
ATOM   4925  CD2 TYR P 302     -7.670 -10.604 -16.492  0.00  0.00      PROA
ATOM   4926  HD2 TYR P 302     -7.579 -11.524 -15.933  0.00  0.00      PROA
ATOM   4927  CE2 TYR P 302     -7.116 -10.378 -17.772  0.00  0.00      PROA
ATOM   4928  HE2 TYR P 302     -6.518 -11.198 -18.140  0.00  0.00      PROA
ATOM   4929  C   TYR P 302    -11.326 -10.992 -15.446  0.00  0.00      PROA
ATOM   4930  O   TYR P 302    -11.025 -11.360 -16.571  0.00  0.00      PROA
ATOM   4931  N   ALA P 303    -12.210 -11.656 -14.713  0.00  0.00      PROA
ATOM   4932  HN  ALA P 303    -12.544 -11.215 -13.883  0.00  0.00      PROA
ATOM   4933  CA  ALA P 303    -12.825 -12.924 -15.095  0.00  0.00      PROA
ATOM   4934  HA  ALA P 303    -12.116 -13.616 -15.526  0.00  0.00      PROA
ATOM   4935  CB  ALA P 303    -13.373 -13.525 -13.775  0.00  0.00      PROA
ATOM   4936  HB1 ALA P 303    -14.307 -13.011 -13.464  0.00  0.00      PROA
ATOM   4937  HB2 ALA P 303    -12.672 -13.249 -12.959  0.00  0.00      PROA
```

ATOM 4938 HB3 ALA P 303 -13.499 -14.625 -13.866 0.00 0.00 PROA
ATOM 4939 C ALA P 303 -13.949 -12.728 -16.119 0.00 0.00 PROA
ATOM 4940 O ALA P 303 -14.829 -11.952 -15.940 0.00 0.00 PROA
ATOM 4941 N PHE P 304 -13.762 -13.416 -17.231 0.00 0.00 PROA
ATOM 4942 HN PHE P 304 -13.025 -14.085 -17.286 0.00 0.00 PROA
ATOM 4943 CA PHE P 304 -14.690 -13.560 -18.342 0.00 0.00 PROA
ATOM 4944 HA PHE P 304 -14.367 -14.435 -18.885 0.00 0.00 PROA
ATOM 4945 CB PHE P 304 -16.136 -13.924 -17.875 0.00 0.00 PROA
ATOM 4946 HB1 PHE P 304 -16.716 -14.120 -18.802 0.00 0.00 PROA
ATOM 4947 HB2 PHE P 304 -16.623 -13.039 -17.415 0.00 0.00 PROA
ATOM 4948 CG PHE P 304 -16.281 -15.087 -16.904 0.00 0.00 PROA
ATOM 4949 CD1 PHE P 304 -15.793 -16.361 -17.167 0.00 0.00 PROA
ATOM 4950 HD1 PHE P 304 -15.225 -16.423 -18.084 0.00 0.00 PROA
ATOM 4951 CE1 PHE P 304 -16.128 -17.564 -16.418 0.00 0.00 PROA
ATOM 4952 HE1 PHE P 304 -15.824 -18.540 -16.768 0.00 0.00 PROA
ATOM 4953 CZ PHE P 304 -16.804 -17.376 -15.250 0.00 0.00 PROA
ATOM 4954 HZ PHE P 304 -17.079 -18.211 -14.622 0.00 0.00 PROA
ATOM 4955 CD2 PHE P 304 -17.000 -14.972 -15.739 0.00 0.00 PROA
ATOM 4956 HD2 PHE P 304 -17.353 -13.992 -15.454 0.00 0.00 PROA
ATOM 4957 CE2 PHE P 304 -17.259 -16.083 -14.921 0.00 0.00 PROA
ATOM 4958 HE2 PHE P 304 -17.866 -16.041 -14.029 0.00 0.00 PROA
ATOM 4959 C PHE P 304 -14.630 -12.345 -19.202 0.00 0.00 PROA
ATOM 4960 O PHE P 304 -14.580 -12.419 -20.474 0.00 0.00 PROA
ATOM 4961 N LEU P 305 -14.684 -11.162 -18.563 0.00 0.00 PROA
ATOM 4962 HN LEU P 305 -14.786 -11.221 -17.573 0.00 0.00 PROA
ATOM 4963 CA LEU P 305 -14.652 -9.806 -19.142 0.00 0.00 PROA
ATOM 4964 HA LEU P 305 -15.422 -9.813 -19.898 0.00 0.00 PROA
ATOM 4965 CB LEU P 305 -14.984 -8.709 -18.096 0.00 0.00 PROA
ATOM 4966 HB1 LEU P 305 -15.029 -7.736 -18.629 0.00 0.00 PROA
ATOM 4967 HB2 LEU P 305 -14.045 -8.728 -17.503 0.00 0.00 PROA
ATOM 4968 CG LEU P 305 -16.187 -8.912 -17.230 0.00 0.00 PROA
ATOM 4969 HG LEU P 305 -15.990 -9.853 -16.673 0.00 0.00 PROA
ATOM 4970 CD1 LEU P 305 -16.354 -7.755 -16.244 0.00 0.00 PROA
ATOM 4971 HD11 LEU P 305 -15.430 -7.696 -15.630 0.00 0.00 PROA
ATOM 4972 HD12 LEU P 305 -17.227 -7.996 -15.600 0.00 0.00 PROA
ATOM 4973 HD13 LEU P 305 -16.523 -6.797 -16.780 0.00 0.00 PROA
ATOM 4974 CD2 LEU P 305 -17.508 -9.169 -18.059 0.00 0.00 PROA
ATOM 4975 HD21 LEU P 305 -17.560 -10.007 -18.786 0.00 0.00 PROA
ATOM 4976 HD22 LEU P 305 -17.775 -8.268 -18.653 0.00 0.00 PROA
ATOM 4977 HD23 LEU P 305 -18.260 -9.350 -17.261 0.00 0.00 PROA
ATOM 4978 C LEU P 305 -13.376 -9.482 -19.827 0.00 0.00 PROA
ATOM 4979 O LEU P 305 -13.299 -8.768 -20.851 0.00 0.00 PROA
ATOM 4980 N GLY P 306 -12.284 -10.092 -19.302 0.00 0.00 PROA
ATOM 4981 HN GLY P 306 -12.531 -10.655 -18.517 0.00 0.00 PROA
ATOM 4982 CA GLY P 306 -10.900 -10.149 -19.761 0.00 0.00 PROA
ATOM 4983 HA1 GLY P 306 -10.272 -9.715 -18.997 0.00 0.00 PROA
ATOM 4984 HA2 GLY P 306 -10.753 -9.610 -20.685 0.00 0.00 PROA
ATOM 4985 C GLY P 306 -10.400 -11.488 -20.085 0.00 0.00 PROA
ATOM 4986 O GLY P 306 -10.174 -11.885 -21.231 0.00 0.00 PROA
ATOM 4987 N ALA P 307 -10.400 -12.352 -19.031 0.00 0.00 PROA
ATOM 4988 HN ALA P 307 -10.712 -12.129 -18.111 0.00 0.00 PROA
ATOM 4989 CA ALA P 307 -9.795 -13.683 -19.102 0.00 0.00 PROA
ATOM 4990 HA ALA P 307 -9.122 -13.764 -19.943 0.00 0.00 PROA
ATOM 4991 CB ALA P 307 -8.936 -13.921 -17.761 0.00 0.00 PROA
ATOM 4992 HB1 ALA P 307 -9.519 -13.789 -16.825 0.00 0.00 PROA
ATOM 4993 HB2 ALA P 307 -8.099 -13.191 -17.724 0.00 0.00 PROA
ATOM 4994 HB3 ALA P 307 -8.426 -14.907 -17.712 0.00 0.00 PROA
ATOM 4995 C ALA P 307 -10.862 -14.763 -19.123 0.00 0.00 PROA
ATOM 4996 O ALA P 307 -11.592 -14.982 -18.106 0.00 0.00 PROA
ATOM 4997 N LYS P 308 -10.978 -15.440 -20.228 0.00 0.00 PROA
ATOM 4998 HN LYS P 308 -10.441 -15.202 -21.034 0.00 0.00 PROA

```
ATOM   4999  CA  LYS P 308     -11.883 -16.594 -20.336  0.00  0.00      PROA
ATOM   5000  HA  LYS P 308     -12.501 -16.632 -19.451  0.00  0.00      PROA
ATOM   5001  CB  LYS P 308     -12.895 -16.622 -21.487  0.00  0.00      PROA
ATOM   5002  HB1 LYS P 308     -13.387 -17.580 -21.760  0.00  0.00      PROA
ATOM   5003  HB2 LYS P 308     -12.219 -16.393 -22.339  0.00  0.00      PROA
ATOM   5004  CG  LYS P 308     -13.990 -15.570 -21.406  0.00  0.00      PROA
ATOM   5005  HG1 LYS P 308     -13.454 -14.599 -21.344  0.00  0.00      PROA
ATOM   5006  HG2 LYS P 308     -14.656 -15.702 -20.526  0.00  0.00      PROA
ATOM   5007  CD  LYS P 308     -14.781 -15.630 -22.707  0.00  0.00      PROA
ATOM   5008  HD1 LYS P 308     -15.200 -16.659 -22.695  0.00  0.00      PROA
ATOM   5009  HD2 LYS P 308     -14.074 -15.627 -23.564  0.00  0.00      PROA
ATOM   5010  CE  LYS P 308     -15.900 -14.574 -22.947  0.00  0.00      PROA
ATOM   5011  HE1 LYS P 308     -16.606 -14.698 -22.098  0.00  0.00      PROA
ATOM   5012  HE2 LYS P 308     -16.428 -14.706 -23.916  0.00  0.00      PROA
ATOM   5013  NZ  LYS P 308     -15.418 -13.125 -22.972  0.00  0.00      PROA
ATOM   5014  HZ1 LYS P 308     -14.700 -12.959 -23.706  0.00  0.00      PROA
ATOM   5015  HZ2 LYS P 308     -14.857 -12.951 -22.114  0.00  0.00      PROA
ATOM   5016  HZ3 LYS P 308     -16.161 -12.400 -23.029  0.00  0.00      PROA
ATOM   5017  C   LYS P 308     -11.219 -17.954 -20.057  0.00  0.00      PROA
ATOM   5018  O   LYS P 308     -10.036 -18.203 -20.298  0.00  0.00      PROA
ATOM   5019  N   PHE P 309     -11.924 -18.856 -19.459  0.00  0.00      PROA
ATOM   5020  HN  PHE P 309     -12.900 -18.738 -19.297  0.00  0.00      PROA
ATOM   5021  CA  PHE P 309     -11.359 -20.075 -18.880  0.00  0.00      PROA
ATOM   5022  HA  PHE P 309     -10.295 -20.083 -19.064  0.00  0.00      PROA
ATOM   5023  CB  PHE P 309     -11.532 -20.085 -17.330  0.00  0.00      PROA
ATOM   5024  HB1 PHE P 309     -11.115 -21.003 -16.864  0.00  0.00      PROA
ATOM   5025  HB2 PHE P 309     -12.626 -20.111 -17.137  0.00  0.00      PROA
ATOM   5026  CG  PHE P 309     -10.851 -18.960 -16.680  0.00  0.00      PROA
ATOM   5027  CD1 PHE P 309     -11.494 -18.148 -15.692  0.00  0.00      PROA
ATOM   5028  HD1 PHE P 309     -12.554 -18.188 -15.487  0.00  0.00      PROA
ATOM   5029  CE1 PHE P 309     -10.694 -17.139 -15.029  0.00  0.00      PROA
ATOM   5030  HE1 PHE P 309     -11.302 -16.471 -14.437  0.00  0.00      PROA
ATOM   5031  CZ  PHE P 309      -9.303 -17.016 -15.193  0.00  0.00      PROA
ATOM   5032  HZ  PHE P 309      -8.775 -16.242 -14.655  0.00  0.00      PROA
ATOM   5033  CD2 PHE P 309      -9.467 -18.781 -16.897  0.00  0.00      PROA
ATOM   5034  HD2 PHE P 309      -9.032 -19.394 -17.673  0.00  0.00      PROA
ATOM   5035  CE2 PHE P 309      -8.672 -17.882 -16.160  0.00  0.00      PROA
ATOM   5036  HE2 PHE P 309      -7.595 -17.956 -16.138  0.00  0.00      PROA
ATOM   5037  C   PHE P 309     -11.931 -21.345 -19.496  0.00  0.00      PROA
ATOM   5038  O   PHE P 309     -11.765 -22.445 -19.014  0.00  0.00      PROA
ATOM   5039  N   LYS P 310     -12.634 -21.254 -20.656  0.00  0.00      PROA
ATOM   5040  HN  LYS P 310     -12.829 -20.291 -20.820  0.00  0.00      PROA
ATOM   5041  CA  LYS P 310     -13.195 -22.360 -21.413  0.00  0.00      PROA
ATOM   5042  HA  LYS P 310     -12.745 -23.316 -21.187  0.00  0.00      PROA
ATOM   5043  CB  LYS P 310     -14.729 -22.350 -21.231  0.00  0.00      PROA
ATOM   5044  HB1 LYS P 310     -15.227 -23.194 -21.756  0.00  0.00      PROA
ATOM   5045  HB2 LYS P 310     -15.114 -21.397 -21.653  0.00  0.00      PROA
ATOM   5046  CG  LYS P 310     -15.186 -22.403 -19.749  0.00  0.00      PROA
ATOM   5047  HG1 LYS P 310     -16.296 -22.416 -19.804  0.00  0.00      PROA
ATOM   5048  HG2 LYS P 310     -14.938 -21.435 -19.263  0.00  0.00      PROA
ATOM   5049  CD  LYS P 310     -14.684 -23.618 -18.966  0.00  0.00      PROA
ATOM   5050  HD1 LYS P 310     -14.175 -23.337 -18.020  0.00  0.00      PROA
ATOM   5051  HD2 LYS P 310     -13.830 -24.079 -19.507  0.00  0.00      PROA
ATOM   5052  CE  LYS P 310     -15.698 -24.779 -18.643  0.00  0.00      PROA
ATOM   5053  HE1 LYS P 310     -16.456 -24.593 -17.853  0.00  0.00      PROA
ATOM   5054  HE2 LYS P 310     -15.071 -25.474 -18.045  0.00  0.00      PROA
ATOM   5055  NZ  LYS P 310     -16.314 -25.388 -19.871  0.00  0.00      PROA
ATOM   5056  HZ1 LYS P 310     -15.541 -25.515 -20.555  0.00  0.00      PROA
ATOM   5057  HZ2 LYS P 310     -16.992 -24.673 -20.203  0.00  0.00      PROA
ATOM   5058  HZ3 LYS P 310     -16.823 -26.257 -19.612  0.00  0.00      PROA
ATOM   5059  C   LYS P 310     -12.898 -22.132 -22.776  0.00  0.00      PROA
```

ATOM 5060 O LYS P 310 -13.065 -21.079 -23.360 0.00 0.00 PROA
ATOM 5061 N THR P 311 -12.425 -23.166 -23.481 0.00 0.00 PROA
ATOM 5062 HN THR P 311 -12.430 -24.032 -22.987 0.00 0.00 PROA
ATOM 5063 CA THR P 311 -11.826 -23.071 -24.838 0.00 0.00 PROA
ATOM 5064 HA THR P 311 -11.021 -22.366 -24.694 0.00 0.00 PROA
ATOM 5065 CB THR P 311 -11.207 -24.389 -25.118 0.00 0.00 PROA
ATOM 5066 HB THR P 311 -10.648 -24.797 -24.249 0.00 0.00 PROA
ATOM 5067 OG1 THR P 311 -10.246 -24.227 -26.152 0.00 0.00 PROA
ATOM 5068 HG1 THR P 311 -9.486 -23.791 -25.760 0.00 0.00 PROA
ATOM 5069 CG2 THR P 311 -12.157 -25.527 -25.605 0.00 0.00 PROA
ATOM 5070 HG21 THR P 311 -13.035 -25.544 -24.925 0.00 0.00 PROA
ATOM 5071 HG22 THR P 311 -11.597 -26.472 -25.437 0.00 0.00 PROA
ATOM 5072 HG23 THR P 311 -12.426 -25.289 -26.656 0.00 0.00 PROA
ATOM 5073 C THR P 311 -12.745 -22.566 -25.971 0.00 0.00 PROA
ATOM 5074 O THR P 311 -13.971 -22.583 -26.020 0.00 0.00 PROA
ATOM 5075 N SER P 312 -12.060 -22.044 -27.003 0.00 0.00 PROA
ATOM 5076 HN SER P 312 -11.078 -21.926 -26.877 0.00 0.00 PROA
ATOM 5077 CA SER P 312 -12.601 -21.761 -28.286 0.00 0.00 PROA
ATOM 5078 HA SER P 312 -13.403 -22.418 -28.588 0.00 0.00 PROA
ATOM 5079 CB SER P 312 -13.032 -20.222 -28.301 0.00 0.00 PROA
ATOM 5080 HB1 SER P 312 -12.267 -19.521 -27.904 0.00 0.00 PROA
ATOM 5081 HB2 SER P 312 -13.883 -20.210 -27.586 0.00 0.00 PROA
ATOM 5082 OG SER P 312 -13.405 -19.747 -29.591 0.00 0.00 PROA
ATOM 5083 HG1 SER P 312 -14.362 -19.804 -29.540 0.00 0.00 PROA
ATOM 5084 C SER P 312 -11.533 -22.096 -29.387 0.00 0.00 PROA
ATOM 5085 O SER P 312 -10.351 -21.942 -29.185 0.00 0.00 PROA
ATOM 5086 N ALA P 313 -11.889 -22.460 -30.594 0.00 0.00 PROA
ATOM 5087 HN ALA P 313 -12.739 -22.980 -30.608 0.00 0.00 PROA
ATOM 5088 CA ALA P 313 -10.898 -22.646 -31.665 0.00 0.00 PROA
ATOM 5089 HA ALA P 313 -9.889 -22.818 -31.320 0.00 0.00 PROA
ATOM 5090 CB ALA P 313 -11.358 -23.760 -32.675 0.00 0.00 PROA
ATOM 5091 HB1 ALA P 313 -12.173 -23.409 -33.343 0.00 0.00 PROA
ATOM 5092 HB2 ALA P 313 -11.830 -24.514 -32.010 0.00 0.00 PROA
ATOM 5093 HB3 ALA P 313 -10.505 -24.124 -33.287 0.00 0.00 PROA
ATOM 5094 C ALA P 313 -10.664 -21.370 -32.486 0.00 0.00 PROA
ATOM 5095 O ALA P 313 -9.676 -21.363 -33.262 0.00 0.00 PROA
ATOM 5096 N GLN P 314 -11.545 -20.354 -32.304 0.00 0.00 PROA
ATOM 5097 HN GLN P 314 -12.295 -20.494 -31.663 0.00 0.00 PROA
ATOM 5098 CA GLN P 314 -11.566 -19.045 -33.047 0.00 0.00 PROA
ATOM 5099 HA GLN P 314 -10.874 -19.020 -33.876 0.00 0.00 PROA
ATOM 5100 CB GLN P 314 -13.097 -18.782 -33.488 0.00 0.00 PROA
ATOM 5101 HB1 GLN P 314 -13.072 -17.799 -34.006 0.00 0.00 PROA
ATOM 5102 HB2 GLN P 314 -13.818 -18.602 -32.662 0.00 0.00 PROA
ATOM 5103 CG GLN P 314 -13.730 -19.865 -34.365 0.00 0.00 PROA
ATOM 5104 HG1 GLN P 314 -14.672 -19.460 -34.792 0.00 0.00 PROA
ATOM 5105 HG2 GLN P 314 -14.072 -20.722 -33.745 0.00 0.00 PROA
ATOM 5106 CD GLN P 314 -12.799 -20.326 -35.465 0.00 0.00 PROA
ATOM 5107 OE1 GLN P 314 -12.786 -21.600 -35.632 0.00 0.00 PROA
ATOM 5108 NE2 GLN P 314 -12.135 -19.457 -36.324 0.00 0.00 PROA
ATOM 5109 HE21 GLN P 314 -11.406 -19.847 -36.887 0.00 0.00 PROA
ATOM 5110 HE22 GLN P 314 -12.293 -18.480 -36.181 0.00 0.00 PROA
ATOM 5111 C GLN P 314 -11.045 -17.918 -32.177 0.00 0.00 PROA
ATOM 5112 O GLN P 314 -10.964 -16.762 -32.586 0.00 0.00 PROA
ATOM 5113 N HSD P 315 -10.728 -18.189 -30.941 0.00 0.00 PROA
ATOM 5114 HN HSD P 315 -10.749 -19.155 -30.695 0.00 0.00 PROA
ATOM 5115 CA HSD P 315 -10.410 -17.221 -29.962 0.00 0.00 PROA
ATOM 5116 HA HSD P 315 -9.881 -16.430 -30.471 0.00 0.00 PROA
ATOM 5117 CB HSD P 315 -11.535 -16.659 -29.084 0.00 0.00 PROA
ATOM 5118 HB1 HSD P 315 -11.061 -16.101 -28.249 0.00 0.00 PROA
ATOM 5119 HB2 HSD P 315 -12.064 -17.562 -28.711 0.00 0.00 PROA
ATOM 5120 ND1 HSD P 315 -12.422 -14.332 -29.633 0.00 0.00 PROA

```
ATOM   5121  HD1 HSD P 315     -11.714 -13.923 -29.058  0.00  0.00      PROA
ATOM   5122  CG  HSD P 315     -12.461 -15.661 -29.836  0.00  0.00      PROA
ATOM   5123  CE1 HSD P 315     -13.591 -13.822 -30.060  0.00  0.00      PROA
ATOM   5124  HE1 HSD P 315     -13.907 -12.800 -29.852  0.00  0.00      PROA
ATOM   5125  NE2 HSD P 315     -14.368 -14.739 -30.577  0.00  0.00      PROA
ATOM   5126  CD2 HSD P 315     -13.597 -15.897 -30.476  0.00  0.00      PROA
ATOM   5127  HD2 HSD P 315     -14.135 -16.799 -30.741  0.00  0.00      PROA
ATOM   5128  C   HSD P 315      -9.408 -17.655 -28.916  0.00  0.00      PROA
ATOM   5129  O   HSD P 315      -9.375 -18.791 -28.449  0.00  0.00      PROA
ATOM   5130  N   ALA P 316      -8.519 -16.740 -28.452  0.00  0.00      PROA
ATOM   5131  HN  ALA P 316      -8.634 -15.816 -28.809  0.00  0.00      PROA
ATOM   5132  CA  ALA P 316      -7.429 -17.057 -27.586  0.00  0.00      PROA
ATOM   5133  HA  ALA P 316      -7.307 -18.130 -27.581  0.00  0.00      PROA
ATOM   5134  CB  ALA P 316      -6.117 -16.590 -28.149  0.00  0.00      PROA
ATOM   5135  HB1 ALA P 316      -5.313 -16.937 -27.465  0.00  0.00      PROA
ATOM   5136  HB2 ALA P 316      -5.949 -15.492 -28.148  0.00  0.00      PROA
ATOM   5137  HB3 ALA P 316      -5.920 -17.040 -29.145  0.00  0.00      PROA
ATOM   5138  C   ALA P 316      -7.714 -16.666 -26.193  0.00  0.00      PROA
ATOM   5139  O   ALA P 316      -6.873 -16.570 -25.321  0.00  0.00      PROA
ATOM   5140  N   LEU P 317      -9.054 -16.396 -26.008  0.00  0.00      PROA
ATOM   5141  HN  LEU P 317      -9.656 -16.284 -26.795  0.00  0.00      PROA
ATOM   5142  CA  LEU P 317      -9.779 -16.179 -24.713  0.00  0.00      PROA
ATOM   5143  HA  LEU P 317     -10.807 -16.092 -25.032  0.00  0.00      PROA
ATOM   5144  CB  LEU P 317      -9.556 -17.425 -23.795  0.00  0.00      PROA
ATOM   5145  HB1 LEU P 317     -10.281 -17.360 -22.956  0.00  0.00      PROA
ATOM   5146  HB2 LEU P 317      -8.529 -17.448 -23.370  0.00  0.00      PROA
ATOM   5147  CG  LEU P 317      -9.845 -18.738 -24.510  0.00  0.00      PROA
ATOM   5148  HG  LEU P 317      -9.017 -18.958 -25.218  0.00  0.00      PROA
ATOM   5149  CD1 LEU P 317      -9.804 -19.782 -23.340  0.00  0.00      PROA
ATOM   5150 HD11 LEU P 317      -8.778 -19.822 -22.914  0.00  0.00      PROA
ATOM   5151 HD12 LEU P 317      -9.970 -20.811 -23.725  0.00  0.00      PROA
ATOM   5152 HD13 LEU P 317     -10.574 -19.458 -22.608  0.00  0.00      PROA
ATOM   5153  CD2 LEU P 317     -11.217 -18.785 -25.182  0.00  0.00      PROA
ATOM   5154 HD21 LEU P 317     -11.483 -19.821 -25.481  0.00  0.00      PROA
ATOM   5155 HD22 LEU P 317     -11.304 -18.282 -26.170  0.00  0.00      PROA
ATOM   5156 HD23 LEU P 317     -12.068 -18.328 -24.633  0.00  0.00      PROA
ATOM   5157  C   LEU P 317      -9.523 -14.923 -23.875  0.00  0.00      PROA
ATOM   5158  O   LEU P 317      -9.996 -14.808 -22.695  0.00  0.00      PROA
ATOM   5159  N   THR P 318      -8.825 -13.931 -24.472  0.00  0.00      PROA
ATOM   5160  HN  THR P 318      -8.512 -14.087 -25.405  0.00  0.00      PROA
ATOM   5161  CA  THR P 318      -8.531 -12.613 -23.892  0.00  0.00      PROA
ATOM   5162  HA  THR P 318      -9.518 -12.221 -23.698  0.00  0.00      PROA
ATOM   5163  CB  THR P 318      -7.589 -12.605 -22.708  0.00  0.00      PROA
ATOM   5164  HB  THR P 318      -7.977 -13.341 -21.971  0.00  0.00      PROA
ATOM   5165  OG1 THR P 318      -7.591 -11.383 -22.053  0.00  0.00      PROA
ATOM   5166  HG1 THR P 318      -8.488 -11.157 -21.797  0.00  0.00      PROA
ATOM   5167  CG2 THR P 318      -6.175 -12.952 -23.113  0.00  0.00      PROA
ATOM   5168 HG21 THR P 318      -5.606 -13.276 -22.216  0.00  0.00      PROA
ATOM   5169 HG22 THR P 318      -5.641 -12.085 -23.558  0.00  0.00      PROA
ATOM   5170 HG23 THR P 318      -6.294 -13.754 -23.874  0.00  0.00      PROA
ATOM   5171  C   THR P 318      -7.954 -11.802 -24.982  0.00  0.00      PROA
ATOM   5172  O   THR P 318      -7.634 -12.337 -26.058  0.00  0.00      PROA
ATOM   5173  N   SER P 319      -7.952 -10.500 -24.905  0.00  0.00      PROA
ATOM   5174  HN  SER P 319      -8.101 -10.059 -24.024  0.00  0.00      PROA
ATOM   5175  CA  SER P 319      -7.759  -9.688 -26.060  0.00  0.00      PROA
ATOM   5176  HA  SER P 319      -7.152 -10.042 -26.880  0.00  0.00      PROA
ATOM   5177  CB  SER P 319      -9.079  -9.217 -26.743  0.00  0.00      PROA
ATOM   5178  HB1 SER P 319      -8.965  -8.420 -27.509  0.00  0.00      PROA
ATOM   5179  HB2 SER P 319      -9.817  -8.917 -25.969  0.00  0.00      PROA
ATOM   5180  OG  SER P 319      -9.670 -10.260 -27.510  0.00  0.00      PROA
ATOM   5181  HG1 SER P 319     -10.036 -10.962 -26.967  0.00  0.00      PROA
```

```
ATOM   5182  C   SER P 319      -7.102  -8.389 -25.574  0.00  0.00      PROA
ATOM   5183  OT1 SER P 319      -6.443  -7.651 -26.337  0.00  0.00      PROA
ATOM   5184  OT2 SER P 319      -7.169  -8.117 -24.338  0.00  0.00      PROA
ATOM   5185  N   LEU P   1       1.193  -6.782  28.115  0.00  0.00      PROB
ATOM   5186  HT1 LEU P   1       0.800  -6.399  28.998  0.00  0.00      PROB
ATOM   5187  HT2 LEU P   1       2.112  -6.298  28.163  0.00  0.00      PROB
ATOM   5188  HT3 LEU P   1       1.225  -7.815  28.237  0.00  0.00      PROB
ATOM   5189  CA  LEU P   1       0.347  -6.455  26.956  0.00  0.00      PROB
ATOM   5190  HA  LEU P   1      -0.675  -6.790  27.050  0.00  0.00      PROB
ATOM   5191  CB  LEU P   1       0.331  -4.946  26.849  0.00  0.00      PROB
ATOM   5192  HB1 LEU P   1      -0.098  -4.717  25.850  0.00  0.00      PROB
ATOM   5193  HB2 LEU P   1       1.360  -4.544  26.967  0.00  0.00      PROB
ATOM   5194  CG  LEU P   1      -0.588  -4.224  27.822  0.00  0.00      PROB
ATOM   5195  HG  LEU P   1      -0.553  -4.634  28.854  0.00  0.00      PROB
ATOM   5196  CD1 LEU P   1      -0.144  -2.768  28.035  0.00  0.00      PROB
ATOM   5197 HD11 LEU P   1      -0.258  -2.201  27.086  0.00  0.00      PROB
ATOM   5198 HD12 LEU P   1       0.913  -2.665  28.362  0.00  0.00      PROB
ATOM   5199 HD13 LEU P   1      -0.833  -2.392  28.821  0.00  0.00      PROB
ATOM   5200  CD2 LEU P   1      -1.983  -4.245  27.270  0.00  0.00      PROB
ATOM   5201 HD21 LEU P   1      -2.038  -3.861  26.229  0.00  0.00      PROB
ATOM   5202 HD22 LEU P   1      -2.697  -3.709  27.931  0.00  0.00      PROB
ATOM   5203 HD23 LEU P   1      -2.405  -5.273  27.232  0.00  0.00      PROB
ATOM   5204  C   LEU P   1       0.898  -7.034  25.692  0.00  0.00      PROB
ATOM   5205  O   LEU P   1       2.058  -6.729  25.376  0.00  0.00      PROB
ATOM   5206  N   VAL P   2       0.126  -7.848  24.882  0.00  0.00      PROB
ATOM   5207  HN  VAL P   2      -0.733  -8.235  25.207  0.00  0.00      PROB
ATOM   5208  CA  VAL P   2       0.764  -8.606  23.769  0.00  0.00      PROB
ATOM   5209  HA  VAL P   2       1.560  -9.196  24.198  0.00  0.00      PROB
ATOM   5210  CB  VAL P   2      -0.303  -9.621  23.241  0.00  0.00      PROB
ATOM   5211  HB  VAL P   2      -1.155  -8.974  22.940  0.00  0.00      PROB
ATOM   5212  CG1 VAL P   2       0.255 -10.554  22.134  0.00  0.00      PROB
ATOM   5213 HG11 VAL P   2      -0.438 -11.390  21.895  0.00  0.00      PROB
ATOM   5214 HG12 VAL P   2       1.205 -11.048  22.430  0.00  0.00      PROB
ATOM   5215 HG13 VAL P   2       0.348  -9.908  21.235  0.00  0.00      PROB
ATOM   5216  CG2 VAL P   2      -0.785 -10.675  24.264  0.00  0.00      PROB
ATOM   5217 HG21 VAL P   2      -0.220 -11.616  24.093  0.00  0.00      PROB
ATOM   5218 HG22 VAL P   2      -1.788 -11.048  23.969  0.00  0.00      PROB
ATOM   5219 HG23 VAL P   2      -0.606 -10.371  25.318  0.00  0.00      PROB
ATOM   5220  C   VAL P   2       1.271  -7.658  22.657  0.00  0.00      PROB
ATOM   5221  O   VAL P   2       2.340  -7.847  22.212  0.00  0.00      PROB
ATOM   5222  N   ARG P   3       0.491  -6.609  22.438  0.00  0.00      PROB
ATOM   5223  HN  ARG P   3      -0.304  -6.485  23.027  0.00  0.00      PROB
ATOM   5224  CA  ARG P   3       0.788  -5.614  21.405  0.00  0.00      PROB
ATOM   5225  HA  ARG P   3       1.238  -6.082  20.542  0.00  0.00      PROB
ATOM   5226  CB  ARG P   3      -0.537  -5.238  20.706  0.00  0.00      PROB
ATOM   5227  HB1 ARG P   3      -0.521  -4.307  20.099  0.00  0.00      PROB
ATOM   5228  HB2 ARG P   3      -1.274  -4.934  21.479  0.00  0.00      PROB
ATOM   5229  CG  ARG P   3      -1.251  -6.286  19.892  0.00  0.00      PROB
ATOM   5230  HG1 ARG P   3      -1.815  -5.708  19.128  0.00  0.00      PROB
ATOM   5231  HG2 ARG P   3      -2.038  -6.804  20.479  0.00  0.00      PROB
ATOM   5232  CD  ARG P   3      -0.348  -7.275  19.040  0.00  0.00      PROB
ATOM   5233  HD1 ARG P   3       0.246  -7.808  19.813  0.00  0.00      PROB
ATOM   5234  HD2 ARG P   3       0.337  -6.801  18.304  0.00  0.00      PROB
ATOM   5235  NE  ARG P   3      -1.312  -8.354  18.398  0.00  0.00      PROB
ATOM   5236  HE  ARG P   3      -2.308  -8.375  18.478  0.00  0.00      PROB
ATOM   5237  CZ  ARG P   3      -0.808  -9.496  17.949  0.00  0.00      PROB
ATOM   5238  NH1 ARG P   3       0.462  -9.818  17.964  0.00  0.00      PROB
ATOM   5239 HH11 ARG P   3       1.094  -9.050  18.069  0.00  0.00      PROB
ATOM   5240 HH12 ARG P   3       0.774 -10.674  17.552  0.00  0.00      PROB
ATOM   5241  NH2 ARG P   3      -1.585 -10.455  17.519  0.00  0.00      PROB
ATOM   5242 HH21 ARG P   3      -2.514 -10.225  17.229  0.00  0.00      PROB
```

ATOM 5243 HH22 ARG P 3 -1.009 -11.221 17.234 0.00 0.00 PROB
ATOM 5244 C ARG P 3 1.582 -4.453 21.936 0.00 0.00 PROB
ATOM 5245 O ARG P 3 2.075 -3.560 21.297 0.00 0.00 PROB
ATOM 5246 N TYR P 4 1.827 -4.514 23.284 0.00 0.00 PROB
ATOM 5247 HN TYR P 4 1.479 -5.239 23.872 0.00 0.00 PROB
ATOM 5248 CA TYR P 4 2.593 -3.439 23.902 0.00 0.00 PROB
ATOM 5249 HA TYR P 4 3.169 -2.873 23.186 0.00 0.00 PROB
ATOM 5250 CB TYR P 4 1.637 -2.402 24.618 0.00 0.00 PROB
ATOM 5251 HB1 TYR P 4 2.222 -1.573 25.070 0.00 0.00 PROB
ATOM 5252 HB2 TYR P 4 0.945 -2.971 25.275 0.00 0.00 PROB
ATOM 5253 CG TYR P 4 0.843 -1.760 23.448 0.00 0.00 PROB
ATOM 5254 CD1 TYR P 4 1.451 -0.770 22.637 0.00 0.00 PROB
ATOM 5255 HD1 TYR P 4 2.463 -0.486 22.886 0.00 0.00 PROB
ATOM 5256 CE1 TYR P 4 0.672 -0.179 21.600 0.00 0.00 PROB
ATOM 5257 HE1 TYR P 4 1.149 0.549 20.961 0.00 0.00 PROB
ATOM 5258 CZ TYR P 4 -0.677 -0.575 21.441 0.00 0.00 PROB
ATOM 5259 OH TYR P 4 -1.552 0.118 20.577 0.00 0.00 PROB
ATOM 5260 HH TYR P 4 -1.077 0.914 20.329 0.00 0.00 PROB
ATOM 5261 CD2 TYR P 4 -0.492 -2.216 23.177 0.00 0.00 PROB
ATOM 5262 HD2 TYR P 4 -0.866 -3.023 23.789 0.00 0.00 PROB
ATOM 5263 CE2 TYR P 4 -1.267 -1.548 22.194 0.00 0.00 PROB
ATOM 5264 HE2 TYR P 4 -2.319 -1.770 22.100 0.00 0.00 PROB
ATOM 5265 C TYR P 4 3.609 -3.877 24.914 0.00 0.00 PROB
ATOM 5266 O TYR P 4 3.804 -3.169 25.894 0.00 0.00 PROB
ATOM 5267 N THR P 5 4.339 -4.991 24.693 0.00 0.00 PROB
ATOM 5268 HN THR P 5 4.224 -5.568 23.889 0.00 0.00 PROB
ATOM 5269 CA THR P 5 5.378 -5.501 25.615 0.00 0.00 PROB
ATOM 5270 HA THR P 5 4.874 -5.461 26.570 0.00 0.00 PROB
ATOM 5271 CB THR P 5 5.751 -6.952 25.381 0.00 0.00 PROB
ATOM 5272 HB THR P 5 5.796 -7.071 24.277 0.00 0.00 PROB
ATOM 5273 OG1 THR P 5 4.660 -7.725 25.849 0.00 0.00 PROB
ATOM 5274 HG1 THR P 5 3.893 -7.194 25.623 0.00 0.00 PROB
ATOM 5275 CG2 THR P 5 7.091 -7.560 26.036 0.00 0.00 PROB
ATOM 5276 HG21 THR P 5 7.152 -8.666 26.130 0.00 0.00 PROB
ATOM 5277 HG22 THR P 5 7.303 -6.964 26.949 0.00 0.00 PROB
ATOM 5278 HG23 THR P 5 7.831 -7.355 25.233 0.00 0.00 PROB
ATOM 5279 C THR P 5 6.528 -4.542 25.702 0.00 0.00 PROB
ATOM 5280 O THR P 5 7.095 -4.344 26.773 0.00 0.00 PROB
ATOM 5281 N LYS P 6 6.933 -3.983 24.512 0.00 0.00 PROB
ATOM 5282 HN LYS P 6 6.486 -4.131 23.633 0.00 0.00 PROB
ATOM 5283 CA LYS P 6 8.023 -3.081 24.333 0.00 0.00 PROB
ATOM 5284 HA LYS P 6 8.442 -2.872 25.307 0.00 0.00 PROB
ATOM 5285 CB LYS P 6 9.023 -3.682 23.289 0.00 0.00 PROB
ATOM 5286 HB1 LYS P 6 9.851 -2.956 23.146 0.00 0.00 PROB
ATOM 5287 HB2 LYS P 6 8.465 -3.934 22.362 0.00 0.00 PROB
ATOM 5288 CG LYS P 6 9.754 -4.983 23.737 0.00 0.00 PROB
ATOM 5289 HG1 LYS P 6 10.564 -5.193 23.006 0.00 0.00 PROB
ATOM 5290 HG2 LYS P 6 9.019 -5.816 23.768 0.00 0.00 PROB
ATOM 5291 CD LYS P 6 10.350 -4.905 25.200 0.00 0.00 PROB
ATOM 5292 HD1 LYS P 6 9.518 -4.825 25.931 0.00 0.00 PROB
ATOM 5293 HD2 LYS P 6 10.910 -3.946 25.206 0.00 0.00 PROB
ATOM 5294 CE LYS P 6 11.385 -6.060 25.384 0.00 0.00 PROB
ATOM 5295 HE1 LYS P 6 12.211 -5.999 24.644 0.00 0.00 PROB
ATOM 5296 HE2 LYS P 6 10.864 -7.032 25.251 0.00 0.00 PROB
ATOM 5297 NZ LYS P 6 12.083 -5.996 26.677 0.00 0.00 PROB
ATOM 5298 HZ1 LYS P 6 12.601 -5.132 26.936 0.00 0.00 PROB
ATOM 5299 HZ2 LYS P 6 12.682 -6.828 26.848 0.00 0.00 PROB
ATOM 5300 HZ3 LYS P 6 11.338 -6.176 27.380 0.00 0.00 PROB
ATOM 5301 C LYS P 6 7.447 -1.723 23.896 0.00 0.00 PROB
ATOM 5302 O LYS P 6 7.865 -0.678 24.442 0.00 0.00 PROB
ATOM 5303 N LYS P 7 6.517 -1.623 22.922 0.00 0.00 PROB

ATOM	5304	HN	LYS	P	7	6.050	-2.441	22.594	0.00	0.00	PROB
ATOM	5305	CA	LYS	P	7	5.985	-0.304	22.552	0.00	0.00	PROB
ATOM	5306	HA	LYS	P	7	6.803	0.384	22.392	0.00	0.00	PROB
ATOM	5307	CB	LYS	P	7	4.956	-0.452	21.380	0.00	0.00	PROB
ATOM	5308	HB1	LYS	P	7	4.364	0.468	21.187	0.00	0.00	PROB
ATOM	5309	HB2	LYS	P	7	4.287	-1.310	21.604	0.00	0.00	PROB
ATOM	5310	CG	LYS	P	7	5.718	-0.695	20.047	0.00	0.00	PROB
ATOM	5311	HG1	LYS	P	7	6.613	-1.312	20.276	0.00	0.00	PROB
ATOM	5312	HG2	LYS	P	7	5.973	0.300	19.624	0.00	0.00	PROB
ATOM	5313	CD	LYS	P	7	4.909	-1.524	18.993	0.00	0.00	PROB
ATOM	5314	HD1	LYS	P	7	3.905	-1.127	18.732	0.00	0.00	PROB
ATOM	5315	HD2	LYS	P	7	4.728	-2.522	19.445	0.00	0.00	PROB
ATOM	5316	CE	LYS	P	7	5.785	-1.705	17.724	0.00	0.00	PROB
ATOM	5317	HE1	LYS	P	7	6.871	-1.895	17.860	0.00	0.00	PROB
ATOM	5318	HE2	LYS	P	7	5.773	-0.853	17.012	0.00	0.00	PROB
ATOM	5319	NZ	LYS	P	7	5.242	-2.706	16.832	0.00	0.00	PROB
ATOM	5320	HZ1	LYS	P	7	4.269	-2.903	17.144	0.00	0.00	PROB
ATOM	5321	HZ2	LYS	P	7	5.742	-3.601	17.006	0.00	0.00	PROB
ATOM	5322	HZ3	LYS	P	7	5.198	-2.470	15.820	0.00	0.00	PROB
ATOM	5323	C	LYS	P	7	5.281	0.433	23.725	0.00	0.00	PROB
ATOM	5324	O	LYS	P	7	4.608	-0.173	24.544	0.00	0.00	PROB
ATOM	5325	N	VAL	P	8	5.438	1.795	23.740	0.00	0.00	PROB
ATOM	5326	HN	VAL	P	8	6.083	2.279	23.154	0.00	0.00	PROB
ATOM	5327	CA	VAL	P	8	4.593	2.685	24.516	0.00	0.00	PROB
ATOM	5328	HA	VAL	P	8	4.886	2.495	25.538	0.00	0.00	PROB
ATOM	5329	CB	VAL	P	8	4.919	4.188	24.267	0.00	0.00	PROB
ATOM	5330	HB	VAL	P	8	5.142	4.189	23.178	0.00	0.00	PROB
ATOM	5331	CG1	VAL	P	8	3.910	5.224	24.667	0.00	0.00	PROB
ATOM	5332	HG11	VAL	P	8	4.204	6.294	24.598	0.00	0.00	PROB
ATOM	5333	HG12	VAL	P	8	3.571	4.974	25.694	0.00	0.00	PROB
ATOM	5334	HG13	VAL	P	8	3.210	5.182	23.805	0.00	0.00	PROB
ATOM	5335	CG2	VAL	P	8	6.315	4.381	24.932	0.00	0.00	PROB
ATOM	5336	HG21	VAL	P	8	6.761	5.387	24.780	0.00	0.00	PROB
ATOM	5337	HG22	VAL	P	8	7.094	3.701	24.525	0.00	0.00	PROB
ATOM	5338	HG23	VAL	P	8	6.282	4.094	26.004	0.00	0.00	PROB
ATOM	5339	C	VAL	P	8	3.016	2.503	24.196	0.00	0.00	PROB
ATOM	5340	O	VAL	P	8	2.636	2.156	23.087	0.00	0.00	PROB
ATOM	5341	N	PRO	P	9	2.067	2.626	25.148	0.00	0.00	PROB
ATOM	5342	CD	PRO	P	9	2.376	2.480	26.571	0.00	0.00	PROB
ATOM	5343	HD1	PRO	P	9	2.771	3.452	26.936	0.00	0.00	PROB
ATOM	5344	HD2	PRO	P	9	3.194	1.742	26.716	0.00	0.00	PROB
ATOM	5345	CA	PRO	P	9	0.645	2.429	24.882	0.00	0.00	PROB
ATOM	5346	HA	PRO	P	9	0.519	1.458	24.426	0.00	0.00	PROB
ATOM	5347	CB	PRO	P	9	-0.013	2.522	26.318	0.00	0.00	PROB
ATOM	5348	HB1	PRO	P	9	-0.985	1.992	26.412	0.00	0.00	PROB
ATOM	5349	HB2	PRO	P	9	-0.196	3.605	26.485	0.00	0.00	PROB
ATOM	5350	CG	PRO	P	9	1.115	1.919	27.180	0.00	0.00	PROB
ATOM	5351	HG1	PRO	P	9	1.040	2.171	28.260	0.00	0.00	PROB
ATOM	5352	HG2	PRO	P	9	1.284	0.825	27.276	0.00	0.00	PROB
ATOM	5353	C	PRO	P	9	0.059	3.465	23.861	0.00	0.00	PROB
ATOM	5354	O	PRO	P	9	-0.998	3.218	23.345	0.00	0.00	PROB
ATOM	5355	N	GLN	P	10	0.716	4.590	23.562	0.00	0.00	PROB
ATOM	5356	HN	GLN	P	10	1.590	4.824	23.980	0.00	0.00	PROB
ATOM	5357	CA	GLN	P	10	0.177	5.649	22.604	0.00	0.00	PROB
ATOM	5358	HA	GLN	P	10	-0.899	5.628	22.685	0.00	0.00	PROB
ATOM	5359	CB	GLN	P	10	0.709	7.045	22.899	0.00	0.00	PROB
ATOM	5360	HB1	GLN	P	10	0.150	7.745	22.242	0.00	0.00	PROB
ATOM	5361	HB2	GLN	P	10	1.779	7.101	22.602	0.00	0.00	PROB
ATOM	5362	CG	GLN	P	10	0.559	7.515	24.370	0.00	0.00	PROB
ATOM	5363	HG1	GLN	P	10	0.527	8.621	24.277	0.00	0.00	PROB
ATOM	5364	HG2	GLN	P	10	1.433	7.035	24.861	0.00	0.00	PROB

ATOM 5365 CD GLN P 10 -0.572 6.922 25.177 0.00 0.00 PROB
ATOM 5366 OE1 GLN P 10 -0.308 6.204 26.140 0.00 0.00 PROB
ATOM 5367 NE2 GLN P 10 -1.874 7.198 24.883 0.00 0.00 PROB
ATOM 5368 HE21 GLN P 10 -2.556 6.723 25.438 0.00 0.00 PROB
ATOM 5369 HE22 GLN P 10 -2.137 7.959 24.289 0.00 0.00 PROB
ATOM 5370 C GLN P 10 0.416 5.293 21.140 0.00 0.00 PROB
ATOM 5371 O GLN P 10 -0.306 5.841 20.325 0.00 0.00 PROB
ATOM 5372 N VAL P 11 1.430 4.451 20.868 0.00 0.00 PROB
ATOM 5373 HN VAL P 11 1.996 4.085 21.602 0.00 0.00 PROB
ATOM 5374 CA VAL P 11 1.583 3.801 19.542 0.00 0.00 PROB
ATOM 5375 HA VAL P 11 1.666 4.590 18.809 0.00 0.00 PROB
ATOM 5376 CB VAL P 11 2.888 2.965 19.597 0.00 0.00 PROB
ATOM 5377 HB VAL P 11 2.840 2.115 20.311 0.00 0.00 PROB
ATOM 5378 CG1 VAL P 11 3.205 2.322 18.243 0.00 0.00 PROB
ATOM 5379 HG11 VAL P 11 3.351 3.085 17.448 0.00 0.00 PROB
ATOM 5380 HG12 VAL P 11 2.500 1.502 17.986 0.00 0.00 PROB
ATOM 5381 HG13 VAL P 11 4.199 1.860 18.423 0.00 0.00 PROB
ATOM 5382 CG2 VAL P 11 4.079 3.830 20.019 0.00 0.00 PROB
ATOM 5383 HG21 VAL P 11 4.938 3.192 19.723 0.00 0.00 PROB
ATOM 5384 HG22 VAL P 11 4.101 3.933 21.125 0.00 0.00 PROB
ATOM 5385 HG23 VAL P 11 4.112 4.822 19.519 0.00 0.00 PROB
ATOM 5386 C VAL P 11 0.421 2.916 19.071 0.00 0.00 PROB
ATOM 5387 O VAL P 11 -0.095 2.172 19.888 0.00 0.00 PROB
ATOM 5388 N SER P 12 -0.017 3.183 17.823 0.00 0.00 PROB
ATOM 5389 HN SER P 12 0.405 3.917 17.296 0.00 0.00 PROB
ATOM 5390 CA SER P 12 -0.983 2.365 17.129 0.00 0.00 PROB
ATOM 5391 HA SER P 12 -1.921 2.799 17.444 0.00 0.00 PROB
ATOM 5392 CB SER P 12 -0.857 2.661 15.593 0.00 0.00 PROB
ATOM 5393 HB1 SER P 12 -1.884 2.550 15.184 0.00 0.00 PROB
ATOM 5394 HB2 SER P 12 -0.128 1.902 15.234 0.00 0.00 PROB
ATOM 5395 OG SER P 12 -0.457 4.002 15.315 0.00 0.00 PROB
ATOM 5396 HG1 SER P 12 -1.089 4.404 14.714 0.00 0.00 PROB
ATOM 5397 C SER P 12 -1.142 0.810 17.393 0.00 0.00 PROB
ATOM 5398 O SER P 12 -0.256 0.046 17.700 0.00 0.00 PROB
ATOM 5399 N THR P 13 -2.430 0.313 17.240 0.00 0.00 PROB
ATOM 5400 HN THR P 13 -3.209 0.921 17.107 0.00 0.00 PROB
ATOM 5401 CA THR P 13 -2.780 -1.062 17.492 0.00 0.00 PROB
ATOM 5402 HA THR P 13 -1.944 -1.458 18.049 0.00 0.00 PROB
ATOM 5403 CB THR P 13 -3.998 -1.323 18.399 0.00 0.00 PROB
ATOM 5404 HB THR P 13 -4.872 -1.344 17.713 0.00 0.00 PROB
ATOM 5405 OG1 THR P 13 -4.121 -0.278 19.330 0.00 0.00 PROB
ATOM 5406 HG1 THR P 13 -3.313 -0.192 19.841 0.00 0.00 PROB
ATOM 5407 CG2 THR P 13 -3.873 -2.582 19.204 0.00 0.00 PROB
ATOM 5408 HG21 THR P 13 -4.886 -2.768 19.618 0.00 0.00 PROB
ATOM 5409 HG22 THR P 13 -3.112 -2.456 20.003 0.00 0.00 PROB
ATOM 5410 HG23 THR P 13 -3.636 -3.438 18.536 0.00 0.00 PROB
ATOM 5411 C THR P 13 -3.082 -1.813 16.189 0.00 0.00 PROB
ATOM 5412 O THR P 13 -3.830 -1.284 15.337 0.00 0.00 PROB
ATOM 5413 N PRO P 14 -2.517 -3.060 15.964 0.00 0.00 PROB
ATOM 5414 CD PRO P 14 -1.461 -3.708 16.731 0.00 0.00 PROB
ATOM 5415 HD1 PRO P 14 -2.100 -4.311 17.411 0.00 0.00 PROB
ATOM 5416 HD2 PRO P 14 -0.715 -2.980 17.118 0.00 0.00 PROB
ATOM 5417 CA PRO P 14 -2.916 -3.954 14.802 0.00 0.00 PROB
ATOM 5418 HA PRO P 14 -2.576 -3.448 13.910 0.00 0.00 PROB
ATOM 5419 CB PRO P 14 -2.161 -5.186 15.007 0.00 0.00 PROB
ATOM 5420 HB1 PRO P 14 -2.009 -5.619 13.995 0.00 0.00 PROB
ATOM 5421 HB2 PRO P 14 -2.531 -5.972 15.699 0.00 0.00 PROB
ATOM 5422 CG PRO P 14 -0.856 -4.779 15.785 0.00 0.00 PROB
ATOM 5423 HG1 PRO P 14 -0.337 -5.570 16.366 0.00 0.00 PROB
ATOM 5424 HG2 PRO P 14 -0.089 -4.413 15.069 0.00 0.00 PROB
ATOM 5425 C PRO P 14 -4.392 -4.152 14.601 0.00 0.00 PROB

```
ATOM   5426  O   PRO P  14      -5.089  -3.819  15.528  0.00  0.00      PROB
ATOM   5427  N   THR P  15      -4.862  -4.494  13.341  0.00  0.00      PROB
ATOM   5428  HN  THR P  15      -4.239  -4.697  12.590  0.00  0.00      PROB
ATOM   5429  CA  THR P  15      -6.215  -4.374  12.962  0.00  0.00      PROB
ATOM   5430  HA  THR P  15      -6.596  -3.419  13.292  0.00  0.00      PROB
ATOM   5431  CB  THR P  15      -6.416  -4.325  11.392  0.00  0.00      PROB
ATOM   5432  HB  THR P  15      -5.816  -5.165  10.981  0.00  0.00      PROB
ATOM   5433  OG1 THR P  15      -5.960  -3.076  10.756  0.00  0.00      PROB
ATOM   5434  HG1 THR P  15      -5.643  -3.373   9.900  0.00  0.00      PROB
ATOM   5435  CG2 THR P  15      -7.948  -4.477  11.098  0.00  0.00      PROB
ATOM   5436 HG21 THR P  15      -8.197  -4.433  10.016  0.00  0.00      PROB
ATOM   5437 HG22 THR P  15      -8.540  -3.706  11.636  0.00  0.00      PROB
ATOM   5438 HG23 THR P  15      -8.321  -5.478  11.403  0.00  0.00      PROB
ATOM   5439  C   THR P  15      -7.108  -5.565  13.526  0.00  0.00      PROB
ATOM   5440  O   THR P  15      -8.379  -5.549  13.712  0.00  0.00      PROB
ATOM   5441  N   LEU P  16      -6.426  -6.685  13.893  0.00  0.00      PROB
ATOM   5442  HN  LEU P  16      -5.440  -6.712  13.752  0.00  0.00      PROB
ATOM   5443  CA  LEU P  16      -7.109  -7.832  14.347  0.00  0.00      PROB
ATOM   5444  HA  LEU P  16      -8.176  -7.835  14.175  0.00  0.00      PROB
ATOM   5445  CB  LEU P  16      -6.488  -9.054  13.544  0.00  0.00      PROB
ATOM   5446  HB1 LEU P  16      -7.007  -9.982  13.867  0.00  0.00      PROB
ATOM   5447  HB2 LEU P  16      -5.421  -9.198  13.820  0.00  0.00      PROB
ATOM   5448  CG  LEU P  16      -6.485  -9.019  12.002  0.00  0.00      PROB
ATOM   5449  HG  LEU P  16      -6.127  -7.976  11.864  0.00  0.00      PROB
ATOM   5450  CD1 LEU P  16      -5.518 -10.068  11.390  0.00  0.00      PROB
ATOM   5451 HD11 LEU P  16      -5.808 -10.171  10.323  0.00  0.00      PROB
ATOM   5452 HD12 LEU P  16      -5.856 -11.047  11.794  0.00  0.00      PROB
ATOM   5453 HD13 LEU P  16      -4.456  -9.771  11.521  0.00  0.00      PROB
ATOM   5454  CD2 LEU P  16      -7.913  -9.030  11.527  0.00  0.00      PROB
ATOM   5455 HD21 LEU P  16      -8.492  -9.846  12.009  0.00  0.00      PROB
ATOM   5456 HD22 LEU P  16      -8.080  -9.349  10.475  0.00  0.00      PROB
ATOM   5457 HD23 LEU P  16      -8.370  -8.032  11.692  0.00  0.00      PROB
ATOM   5458  C   LEU P  16      -6.970  -8.007  15.810  0.00  0.00      PROB
ATOM   5459  OT1 LEU P  16      -6.361  -9.056  16.322  0.00  0.00      PROB
ATOM   5460  OT2 LEU P  16      -7.537  -7.161  16.557  0.00  0.00      PROB
END
```

3. A representative structure from the second highly populated cluster of CTER-IN

```
CRYST1   90.078   91.599  142.948  90.00  90.00  90.00 P 1          1
ATOM      1  N   MET P   1      -4.240   7.389  42.932  0.00  0.00      PROA
ATOM      2  HT1 MET P   1      -3.905   6.417  43.086  0.00  0.00      PROA
ATOM      3  HT2 MET P   1      -5.222   7.483  43.262  0.00  0.00      PROA
ATOM      4  HT3 MET P   1      -3.635   8.013  43.504  0.00  0.00      PROA
ATOM      5  CA  MET P   1      -3.993   7.781  41.474  0.00  0.00      PROA
ATOM      6  HA  MET P   1      -3.011   7.391  41.248  0.00  0.00      PROA
ATOM      7  CB  MET P   1      -4.980   7.132  40.456  0.00  0.00      PROA
ATOM      8  HB1 MET P   1      -5.960   7.654  40.416  0.00  0.00      PROA
ATOM      9  HB2 MET P   1      -5.325   6.193  40.939  0.00  0.00      PROA
ATOM     10  CG  MET P   1      -4.421   6.628  39.180  0.00  0.00      PROA
ATOM     11  HG1 MET P   1      -5.195   6.032  38.651  0.00  0.00      PROA
ATOM     12  HG2 MET P   1      -3.670   5.825  39.342  0.00  0.00      PROA
ATOM     13  SD  MET P   1      -3.717   7.861  38.044  0.00  0.00      PROA
ATOM     14  CE  MET P   1      -5.334   8.250  37.350  0.00  0.00      PROA
ATOM     15  HE1 MET P   1      -5.081   8.950  36.525  0.00  0.00      PROA
ATOM     16  HE2 MET P   1      -6.053   8.686  38.076  0.00  0.00      PROA
ATOM     17  HE3 MET P   1      -5.805   7.358  36.883  0.00  0.00      PROA
ATOM     18  C   MET P   1      -3.953   9.293  41.346  0.00  0.00      PROA
ATOM     19  O   MET P   1      -4.797   9.908  41.972  0.00  0.00      PROA
ATOM     20  N   GLU P   2      -2.928   9.906  40.639  0.00  0.00      PROA
ATOM     21  HN  GLU P   2      -2.235   9.297  40.260  0.00  0.00      PROA
ATOM     22  CA  GLU P   2      -2.595  11.323  40.649  0.00  0.00      PROA
ATOM     23  HA  GLU P   2      -2.986  11.765  41.553  0.00  0.00      PROA
ATOM     24  CB  GLU P   2      -1.037  11.594  40.565  0.00  0.00      PROA
ATOM     25  HB1 GLU P   2      -0.901  12.682  40.749  0.00  0.00      PROA
ATOM     26  HB2 GLU P   2      -0.594  11.298  39.590  0.00  0.00      PROA
ATOM     27  CG  GLU P   2      -0.450  10.986  41.883  0.00  0.00      PROA
ATOM     28  HG1 GLU P   2      -0.471   9.878  41.802  0.00  0.00      PROA
ATOM     29  HG2 GLU P   2      -0.995  11.250  42.815  0.00  0.00      PROA
ATOM     30  CD  GLU P   2       1.008  11.385  42.062  0.00  0.00      PROA
ATOM     31  OE1 GLU P   2       1.878  10.700  41.440  0.00  0.00      PROA
ATOM     32  OE2 GLU P   2       1.309  12.267  42.927  0.00  0.00      PROA
ATOM     33  C   GLU P   2      -3.181  12.188  39.450  0.00  0.00      PROA
ATOM     34  O   GLU P   2      -3.129  13.467  39.339  0.00  0.00      PROA
ATOM     35  N   GLY P   3      -3.924  11.629  38.506  0.00  0.00      PROA
ATOM     36  HN  GLY P   3      -4.104  10.649  38.538  0.00  0.00      PROA
ATOM     37  CA  GLY P   3      -4.715  12.436  37.530  0.00  0.00      PROA
ATOM     38  HA1 GLY P   3      -5.273  11.698  36.973  0.00  0.00      PROA
ATOM     39  HA2 GLY P   3      -3.941  12.978  37.006  0.00  0.00      PROA
ATOM     40  C   GLY P   3      -5.724  13.480  38.054  0.00  0.00      PROA
ATOM     41  O   GLY P   3      -6.381  13.307  39.107  0.00  0.00      PROA
ATOM     42  N   ILE P   4      -6.053  14.479  37.160  0.00  0.00      PROA
ATOM     43  HN  ILE P   4      -5.479  14.566  36.349  0.00  0.00      PROA
ATOM     44  CA  ILE P   4      -7.257  15.326  37.172  0.00  0.00      PROA
ATOM     45  HA  ILE P   4      -7.066  15.938  38.041  0.00  0.00      PROA
ATOM     46  CB  ILE P   4      -7.295  16.307  36.020  0.00  0.00      PROA
ATOM     47  HB  ILE P   4      -6.241  16.646  35.925  0.00  0.00      PROA
ATOM     48  CG2 ILE P   4      -7.801  15.704  34.713  0.00  0.00      PROA
ATOM     49 HG21 ILE P   4      -7.479  16.433  33.939  0.00  0.00      PROA
ATOM     50 HG22 ILE P   4      -8.904  15.572  34.684  0.00  0.00      PROA
ATOM     51 HG23 ILE P   4      -7.240  14.791  34.419  0.00  0.00      PROA
ATOM     52  CG1 ILE P   4      -8.005  17.646  36.316  0.00  0.00      PROA
ATOM     53 HG11 ILE P   4      -7.869  18.042  37.345  0.00  0.00      PROA
ATOM     54 HG12 ILE P   4      -9.083  17.395  36.223  0.00  0.00      PROA
ATOM     55  CD  ILE P   4      -7.677  18.848  35.346  0.00  0.00      PROA
ATOM     56  HD1 ILE P   4      -8.057  18.721  34.310  0.00  0.00      PROA
```

```
ATOM     57  HD2 ILE P   4      -6.574  18.984  35.338  0.00  0.00      PROA
ATOM     58  HD3 ILE P   4      -8.127  19.747  35.820  0.00  0.00      PROA
ATOM     59  C   ILE P   4      -8.636  14.742  37.343  0.00  0.00      PROA
ATOM     60  O   ILE P   4      -9.012  13.721  36.653  0.00  0.00      PROA
ATOM     61  N   SER P   5      -9.335  15.254  38.346  0.00  0.00      PROA
ATOM     62  HN  SER P   5      -8.917  15.942  38.935  0.00  0.00      PROA
ATOM     63  CA  SER P   5     -10.738  14.859  38.525  0.00  0.00      PROA
ATOM     64  HA  SER P   5     -10.768  13.791  38.365  0.00  0.00      PROA
ATOM     65  CB  SER P   5     -11.145  14.982  40.020  0.00  0.00      PROA
ATOM     66  HB1 SER P   5     -12.227  14.755  40.130  0.00  0.00      PROA
ATOM     67  HB2 SER P   5     -10.973  16.013  40.396  0.00  0.00      PROA
ATOM     68  OG  SER P   5     -10.384  14.170  40.865  0.00  0.00      PROA
ATOM     69  HG1 SER P   5     -10.584  13.265  40.613  0.00  0.00      PROA
ATOM     70  C   SER P   5     -11.844  15.553  37.711  0.00  0.00      PROA
ATOM     71  O   SER P   5     -11.718  16.729  37.487  0.00  0.00      PROA
ATOM     72  N   ILE P   6     -12.970  14.848  37.416  0.00  0.00      PROA
ATOM     73  HN  ILE P   6     -13.031  13.867  37.583  0.00  0.00      PROA
ATOM     74  CA  ILE P   6     -14.166  15.383  36.979  0.00  0.00      PROA
ATOM     75  HA  ILE P   6     -13.987  16.436  36.818  0.00  0.00      PROA
ATOM     76  CB  ILE P   6     -14.649  14.907  35.587  0.00  0.00      PROA
ATOM     77  HB  ILE P   6     -13.796  15.046  34.889  0.00  0.00      PROA
ATOM     78  CG2 ILE P   6     -15.047  13.392  35.590  0.00  0.00      PROA
ATOM     79 HG21 ILE P   6     -14.198  12.795  35.986  0.00  0.00      PROA
ATOM     80 HG22 ILE P   6     -15.287  13.114  34.542  0.00  0.00      PROA
ATOM     81 HG23 ILE P   6     -15.909  13.269  36.281  0.00  0.00      PROA
ATOM     82  CG1 ILE P   6     -15.777  15.846  35.128  0.00  0.00      PROA
ATOM     83 HG11 ILE P   6     -16.486  16.130  35.935  0.00  0.00      PROA
ATOM     84 HG12 ILE P   6     -16.246  15.274  34.299  0.00  0.00      PROA
ATOM     85  CD  ILE P   6     -15.306  17.173  34.528  0.00  0.00      PROA
ATOM     86  HD1 ILE P   6     -14.723  17.003  33.598  0.00  0.00      PROA
ATOM     87  HD2 ILE P   6     -14.721  17.771  35.259  0.00  0.00      PROA
ATOM     88  HD3 ILE P   6     -16.174  17.785  34.201  0.00  0.00      PROA
ATOM     89  C   ILE P   6     -15.295  15.280  38.050  0.00  0.00      PROA
ATOM     90  O   ILE P   6     -15.621  14.270  38.596  0.00  0.00      PROA
ATOM     91  N   TYR P   7     -15.873  16.452  38.422  0.00  0.00      PROA
ATOM     92  HN  TYR P   7     -15.621  17.210  37.826  0.00  0.00      PROA
ATOM     93  CA  TYR P   7     -16.946  16.715  39.464  0.00  0.00      PROA
ATOM     94  HA  TYR P   7     -16.797  15.954  40.215  0.00  0.00      PROA
ATOM     95  CB  TYR P   7     -16.899  18.152  40.142  0.00  0.00      PROA
ATOM     96  HB1 TYR P   7     -17.792  18.269  40.792  0.00  0.00      PROA
ATOM     97  HB2 TYR P   7     -16.922  18.988  39.411  0.00  0.00      PROA
ATOM     98  CG  TYR P   7     -15.622  18.382  40.843  0.00  0.00      PROA
ATOM     99  CD1 TYR P   7     -14.844  19.628  40.774  0.00  0.00      PROA
ATOM    100  HD1 TYR P   7     -15.230  20.480  40.235  0.00  0.00      PROA
ATOM    101  CE1 TYR P   7     -13.741  19.854  41.602  0.00  0.00      PROA
ATOM    102  HE1 TYR P   7     -13.181  20.775  41.535  0.00  0.00      PROA
ATOM    103  CZ  TYR P   7     -13.193  18.796  42.285  0.00  0.00      PROA
ATOM    104  OH  TYR P   7     -12.017  18.929  43.022  0.00  0.00      PROA
ATOM    105  HH  TYR P   7     -11.928  18.053  43.404  0.00  0.00      PROA
ATOM    106  CD2 TYR P   7     -15.088  17.355  41.632  0.00  0.00      PROA
ATOM    107  HD2 TYR P   7     -15.643  16.430  41.688  0.00  0.00      PROA
ATOM    108  CE2 TYR P   7     -13.886  17.556  42.331  0.00  0.00      PROA
ATOM    109  HE2 TYR P   7     -13.481  16.824  43.014  0.00  0.00      PROA
ATOM    110  C   TYR P   7     -18.339  16.615  38.835  0.00  0.00      PROA
ATOM    111  O   TYR P   7     -18.658  17.419  37.992  0.00  0.00      PROA
ATOM    112  N   THR P   8     -19.142  15.662  39.357  0.00  0.00      PROA
ATOM    113  HN  THR P   8     -18.828  15.150  40.153  0.00  0.00      PROA
ATOM    114  CA  THR P   8     -20.524  15.292  38.909  0.00  0.00      PROA
ATOM    115  HA  THR P   8     -20.834  15.959  38.119  0.00  0.00      PROA
ATOM    116  CB  THR P   8     -20.714  13.874  38.429  0.00  0.00      PROA
ATOM    117  HB  THR P   8     -21.718  13.528  38.101  0.00  0.00      PROA
```

```
ATOM    118  OG1 THR P   8     -20.133  12.947  39.309  0.00  0.00      PROA
ATOM    119  HG1 THR P   8     -20.535  13.064  40.173  0.00  0.00      PROA
ATOM    120  CG2 THR P   8     -19.896  13.727  37.183  0.00  0.00      PROA
ATOM    121 HG21 THR P   8     -18.809  13.840  37.380  0.00  0.00      PROA
ATOM    122 HG22 THR P   8     -20.211  14.410  36.366  0.00  0.00      PROA
ATOM    123 HG23 THR P   8     -19.913  12.677  36.819  0.00  0.00      PROA
ATOM    124  C   THR P   8     -21.461  15.552  40.111  0.00  0.00      PROA
ATOM    125  O   THR P   8     -21.257  15.037  41.306  0.00  0.00      PROA
ATOM    126  N   SER P   9     -22.533  16.323  39.840  0.00  0.00      PROA
ATOM    127  HN  SER P   9     -22.714  16.555  38.888  0.00  0.00      PROA
ATOM    128  CA  SER P   9     -23.442  16.842  40.873  0.00  0.00      PROA
ATOM    129  HA  SER P   9     -23.353  16.277  41.789  0.00  0.00      PROA
ATOM    130  CB  SER P   9     -23.273  18.365  41.158  0.00  0.00      PROA
ATOM    131  HB1 SER P   9     -23.373  18.855  40.166  0.00  0.00      PROA
ATOM    132  HB2 SER P   9     -22.227  18.526  41.498  0.00  0.00      PROA
ATOM    133  OG  SER P   9     -24.050  18.897  42.227  0.00  0.00      PROA
ATOM    134  HG1 SER P   9     -23.872  18.388  43.020  0.00  0.00      PROA
ATOM    135  C   SER P   9     -24.842  16.630  40.270  0.00  0.00      PROA
ATOM    136  O   SER P   9     -25.015  16.843  39.047  0.00  0.00      PROA
ATOM    137  N   ASP P  10     -25.902  16.177  41.035  0.00  0.00      PROA
ATOM    138  HN  ASP P  10     -25.848  16.076  42.026  0.00  0.00      PROA
ATOM    139  CA  ASP P  10     -27.218  15.848  40.462  0.00  0.00      PROA
ATOM    140  HA  ASP P  10     -27.084  15.241  39.579  0.00  0.00      PROA
ATOM    141  CB  ASP P  10     -28.012  15.085  41.528  0.00  0.00      PROA
ATOM    142  HB1 ASP P  10     -29.010  14.875  41.087  0.00  0.00      PROA
ATOM    143  HB2 ASP P  10     -28.130  15.721  42.432  0.00  0.00      PROA
ATOM    144  CG  ASP P  10     -27.212  13.854  41.786  0.00  0.00      PROA
ATOM    145  OD1 ASP P  10     -26.883  13.031  40.863  0.00  0.00      PROA
ATOM    146  OD2 ASP P  10     -26.979  13.592  42.958  0.00  0.00      PROA
ATOM    147  C   ASP P  10     -27.944  17.067  39.928  0.00  0.00      PROA
ATOM    148  O   ASP P  10     -28.212  18.012  40.684  0.00  0.00      PROA
ATOM    149  N   ASN P  11     -28.273  17.084  38.622  0.00  0.00      PROA
ATOM    150  HN  ASN P  11     -27.785  16.472  38.005  0.00  0.00      PROA
ATOM    151  CA  ASN P  11     -28.937  18.222  37.965  0.00  0.00      PROA
ATOM    152  HA  ASN P  11     -29.129  17.791  36.994  0.00  0.00      PROA
ATOM    153  CB  ASN P  11     -30.350  18.621  38.668  0.00  0.00      PROA
ATOM    154  HB1 ASN P  11     -30.855  19.267  37.918  0.00  0.00      PROA
ATOM    155  HB2 ASN P  11     -30.245  19.141  39.644  0.00  0.00      PROA
ATOM    156  CG  ASN P  11     -31.319  17.456  38.860  0.00  0.00      PROA
ATOM    157  OD1 ASN P  11     -31.256  16.445  38.218  0.00  0.00      PROA
ATOM    158  ND2 ASN P  11     -32.348  17.664  39.756  0.00  0.00      PROA
ATOM    159 HD21 ASN P  11     -32.791  18.560  39.764  0.00  0.00      PROA
ATOM    160 HD22 ASN P  11     -32.896  16.840  39.903  0.00  0.00      PROA
ATOM    161  C   ASN P  11     -28.027  19.488  37.726  0.00  0.00      PROA
ATOM    162  O   ASN P  11     -28.532  20.593  37.408  0.00  0.00      PROA
ATOM    163  N   TYR P  12     -26.586  19.426  37.768  0.00  0.00      PROA
ATOM    164  HN  TYR P  12     -26.096  18.631  38.116  0.00  0.00      PROA
ATOM    165  CA  TYR P  12     -25.768  20.591  37.445  0.00  0.00      PROA
ATOM    166  HA  TYR P  12     -26.344  21.322  36.898  0.00  0.00      PROA
ATOM    167  CB  TYR P  12     -25.240  21.202  38.869  0.00  0.00      PROA
ATOM    168  HB1 TYR P  12     -24.761  20.458  39.540  0.00  0.00      PROA
ATOM    169  HB2 TYR P  12     -26.104  21.501  39.500  0.00  0.00      PROA
ATOM    170  CG  TYR P  12     -24.197  22.310  38.848  0.00  0.00      PROA
ATOM    171  CD1 TYR P  12     -22.852  22.089  39.016  0.00  0.00      PROA
ATOM    172  HD1 TYR P  12     -22.455  21.141  39.348  0.00  0.00      PROA
ATOM    173  CE1 TYR P  12     -21.851  23.081  38.710  0.00  0.00      PROA
ATOM    174  HE1 TYR P  12     -20.825  22.868  38.974  0.00  0.00      PROA
ATOM    175  CZ  TYR P  12     -22.292  24.386  38.345  0.00  0.00      PROA
ATOM    176  OH  TYR P  12     -21.369  25.503  38.211  0.00  0.00      PROA
ATOM    177  HH  TYR P  12     -20.486  25.132  38.274  0.00  0.00      PROA
ATOM    178  CD2 TYR P  12     -24.612  23.596  38.470  0.00  0.00      PROA
```

```
ATOM    179  HD2 TYR P  12     -25.646  23.807  38.242  0.00  0.00      PROA
ATOM    180  CE2 TYR P  12     -23.709  24.659  38.282  0.00  0.00      PROA
ATOM    181  HE2 TYR P  12     -24.018  25.692  38.233  0.00  0.00      PROA
ATOM    182  C   TYR P  12     -24.588  20.205  36.578  0.00  0.00      PROA
ATOM    183  O   TYR P  12     -24.105  19.086  36.674  0.00  0.00      PROA
ATOM    184  N   THR P  13     -24.109  21.155  35.712  0.00  0.00      PROA
ATOM    185  HN  THR P  13     -24.620  22.009  35.779  0.00  0.00      PROA
ATOM    186  CA  THR P  13     -23.047  20.985  34.726  0.00  0.00      PROA
ATOM    187  HA  THR P  13     -23.336  20.261  33.979  0.00  0.00      PROA
ATOM    188  CB  THR P  13     -22.804  22.258  33.866  0.00  0.00      PROA
ATOM    189  HB  THR P  13     -21.910  22.277  33.207  0.00  0.00      PROA
ATOM    190  OG1 THR P  13     -22.708  23.449  34.606  0.00  0.00      PROA
ATOM    191  HG1 THR P  13     -22.022  23.274  35.254  0.00  0.00      PROA
ATOM    192  CG2 THR P  13     -24.064  22.365  33.007  0.00  0.00      PROA
ATOM    193 HG21 THR P  13     -25.010  22.424  33.587  0.00  0.00      PROA
ATOM    194 HG22 THR P  13     -24.218  21.509  32.317  0.00  0.00      PROA
ATOM    195 HG23 THR P  13     -24.026  23.264  32.356  0.00  0.00      PROA
ATOM    196  C   THR P  13     -21.743  20.562  35.423  0.00  0.00      PROA
ATOM    197  O   THR P  13     -21.453  20.789  36.583  0.00  0.00      PROA
ATOM    198  N   GLU P  14     -20.965  19.750  34.675  0.00  0.00      PROA
ATOM    199  HN  GLU P  14     -21.322  19.520  33.773  0.00  0.00      PROA
ATOM    200  CA  GLU P  14     -19.704  19.093  34.984  0.00  0.00      PROA
ATOM    201  HA  GLU P  14     -19.829  18.583  35.928  0.00  0.00      PROA
ATOM    202  CB  GLU P  14     -19.311  17.955  34.015  0.00  0.00      PROA
ATOM    203  HB1 GLU P  14     -18.427  17.445  34.455  0.00  0.00      PROA
ATOM    204  HB2 GLU P  14     -19.081  18.272  32.975  0.00  0.00      PROA
ATOM    205  CG  GLU P  14     -20.405  16.783  33.881  0.00  0.00      PROA
ATOM    206  HG1 GLU P  14     -21.443  17.098  33.642  0.00  0.00      PROA
ATOM    207  HG2 GLU P  14     -20.458  16.385  34.917  0.00  0.00      PROA
ATOM    208  CD  GLU P  14     -20.007  15.792  32.831  0.00  0.00      PROA
ATOM    209  OE1 GLU P  14     -18.942  15.118  33.037  0.00  0.00      PROA
ATOM    210  OE2 GLU P  14     -20.744  15.587  31.844  0.00  0.00      PROA
ATOM    211  C   GLU P  14     -18.586  20.087  35.304  0.00  0.00      PROA
ATOM    212  O   GLU P  14     -18.361  21.025  34.574  0.00  0.00      PROA
ATOM    213  N   GLU P  15     -17.742  19.790  36.259  0.00  0.00      PROA
ATOM    214  HN  GLU P  15     -17.938  18.953  36.764  0.00  0.00      PROA
ATOM    215  CA  GLU P  15     -16.578  20.645  36.593  0.00  0.00      PROA
ATOM    216  HA  GLU P  15     -16.448  21.405  35.837  0.00  0.00      PROA
ATOM    217  CB  GLU P  15     -16.879  21.522  37.840  0.00  0.00      PROA
ATOM    218  HB1 GLU P  15     -15.931  22.069  38.029  0.00  0.00      PROA
ATOM    219  HB2 GLU P  15     -17.136  21.038  38.806  0.00  0.00      PROA
ATOM    220  CG  GLU P  15     -17.954  22.575  37.432  0.00  0.00      PROA
ATOM    221  HG1 GLU P  15     -18.955  22.174  37.161  0.00  0.00      PROA
ATOM    222  HG2 GLU P  15     -17.612  23.140  36.538  0.00  0.00      PROA
ATOM    223  CD  GLU P  15     -18.253  23.604  38.526  0.00  0.00      PROA
ATOM    224  OE1 GLU P  15     -17.741  23.565  39.663  0.00  0.00      PROA
ATOM    225  OE2 GLU P  15     -18.873  24.611  37.987  0.00  0.00      PROA
ATOM    226  C   GLU P  15     -15.271  19.889  36.731  0.00  0.00      PROA
ATOM    227  O   GLU P  15     -15.123  18.867  37.346  0.00  0.00      PROA
ATOM    228  N   MET P  16     -14.154  20.403  36.141  0.00  0.00      PROA
ATOM    229  HN  MET P  16     -14.172  21.225  35.578  0.00  0.00      PROA
ATOM    230  CA  MET P  16     -12.808  19.865  36.321  0.00  0.00      PROA
ATOM    231  HA  MET P  16     -12.885  18.799  36.161  0.00  0.00      PROA
ATOM    232  CB  MET P  16     -11.821  20.105  35.157  0.00  0.00      PROA
ATOM    233  HB1 MET P  16     -10.776  19.904  35.476  0.00  0.00      PROA
ATOM    234  HB2 MET P  16     -11.873  21.201  34.977  0.00  0.00      PROA
ATOM    235  CG  MET P  16     -11.982  19.248  33.834  0.00  0.00      PROA
ATOM    236  HG1 MET P  16     -11.404  19.868  33.115  0.00  0.00      PROA
ATOM    237  HG2 MET P  16     -13.064  19.173  33.595  0.00  0.00      PROA
ATOM    238  SD  MET P  16     -11.307  17.510  33.847  0.00  0.00      PROA
ATOM    239  CE  MET P  16     -11.255  16.992  32.105  0.00  0.00      PROA
```

ATOM	240	HE1	MET	P	16	-11.173	15.910	31.869	0.00	0.00	PROA
ATOM	241	HE2	MET	P	16	-10.633	17.678	31.492	0.00	0.00	PROA
ATOM	242	HE3	MET	P	16	-12.226	17.278	31.647	0.00	0.00	PROA
ATOM	243	C	MET	P	16	-12.176	20.236	37.609	0.00	0.00	PROA
ATOM	244	O	MET	P	16	-12.436	21.371	38.036	0.00	0.00	PROA
ATOM	245	N	GLY	P	17	-11.441	19.351	38.287	0.00	0.00	PROA
ATOM	246	HN	GLY	P	17	-11.294	18.492	37.803	0.00	0.00	PROA
ATOM	247	CA	GLY	P	17	-10.908	19.529	39.612	0.00	0.00	PROA
ATOM	248	HA1	GLY	P	17	-11.093	18.650	40.213	0.00	0.00	PROA
ATOM	249	HA2	GLY	P	17	-11.450	20.354	40.049	0.00	0.00	PROA
ATOM	250	C	GLY	P	17	-9.487	19.848	39.598	0.00	0.00	PROA
ATOM	251	O	GLY	P	17	-8.835	20.413	38.680	0.00	0.00	PROA
ATOM	252	N	SER	P	18	-8.902	19.287	40.701	0.00	0.00	PROA
ATOM	253	HN	SER	P	18	-9.415	18.944	41.484	0.00	0.00	PROA
ATOM	254	CA	SER	P	18	-7.465	19.380	40.951	0.00	0.00	PROA
ATOM	255	HA	SER	P	18	-7.111	20.193	40.334	0.00	0.00	PROA
ATOM	256	CB	SER	P	18	-7.209	19.715	42.380	0.00	0.00	PROA
ATOM	257	HB1	SER	P	18	-7.619	19.000	43.125	0.00	0.00	PROA
ATOM	258	HB2	SER	P	18	-7.700	20.652	42.723	0.00	0.00	PROA
ATOM	259	OG	SER	P	18	-5.782	19.825	42.568	0.00	0.00	PROA
ATOM	260	HG1	SER	P	18	-5.675	19.324	43.380	0.00	0.00	PROA
ATOM	261	C	SER	P	18	-6.788	17.992	40.486	0.00	0.00	PROA
ATOM	262	O	SER	P	18	-7.566	17.074	40.192	0.00	0.00	PROA
ATOM	263	N	GLY	P	19	-5.490	17.862	40.427	0.00	0.00	PROA
ATOM	264	HN	GLY	P	19	-4.970	18.657	40.730	0.00	0.00	PROA
ATOM	265	CA	GLY	P	19	-4.709	16.707	39.897	0.00	0.00	PROA
ATOM	266	HA1	GLY	P	19	-5.266	15.782	39.870	0.00	0.00	PROA
ATOM	267	HA2	GLY	P	19	-3.833	16.742	40.528	0.00	0.00	PROA
ATOM	268	C	GLY	P	19	-4.245	17.107	38.470	0.00	0.00	PROA
ATOM	269	O	GLY	P	19	-4.663	18.040	37.838	0.00	0.00	PROA
ATOM	270	N	ASP	P	20	-3.217	16.334	37.967	0.00	0.00	PROA
ATOM	271	HN	ASP	P	20	-3.043	15.463	38.422	0.00	0.00	PROA
ATOM	272	CA	ASP	P	20	-2.437	16.470	36.768	0.00	0.00	PROA
ATOM	273	HA	ASP	P	20	-2.477	17.540	36.634	0.00	0.00	PROA
ATOM	274	CB	ASP	P	20	-0.960	16.099	37.105	0.00	0.00	PROA
ATOM	275	HB1	ASP	P	20	-0.933	15.021	37.371	0.00	0.00	PROA
ATOM	276	HB2	ASP	P	20	-0.474	16.673	37.922	0.00	0.00	PROA
ATOM	277	CG	ASP	P	20	-0.143	16.383	35.934	0.00	0.00	PROA
ATOM	278	OD1	ASP	P	20	0.436	15.434	35.322	0.00	0.00	PROA
ATOM	279	OD2	ASP	P	20	-0.044	17.571	35.539	0.00	0.00	PROA
ATOM	280	C	ASP	P	20	-3.006	15.846	35.542	0.00	0.00	PROA
ATOM	281	O	ASP	P	20	-3.346	14.636	35.508	0.00	0.00	PROA
ATOM	282	N	TYR	P	21	-3.293	16.603	34.424	0.00	0.00	PROA
ATOM	283	HN	TYR	P	21	-3.266	17.597	34.499	0.00	0.00	PROA
ATOM	284	CA	TYR	P	21	-3.848	16.059	33.197	0.00	0.00	PROA
ATOM	285	HA	TYR	P	21	-4.817	15.643	33.431	0.00	0.00	PROA
ATOM	286	CB	TYR	P	21	-4.017	17.231	32.141	0.00	0.00	PROA
ATOM	287	HB1	TYR	P	21	-3.069	17.555	31.661	0.00	0.00	PROA
ATOM	288	HB2	TYR	P	21	-4.392	18.159	32.623	0.00	0.00	PROA
ATOM	289	CG	TYR	P	21	-4.972	16.746	30.996	0.00	0.00	PROA
ATOM	290	CD1	TYR	P	21	-4.548	15.971	29.972	0.00	0.00	PROA
ATOM	291	HD1	TYR	P	21	-3.506	15.698	29.898	0.00	0.00	PROA
ATOM	292	CE1	TYR	P	21	-5.357	15.653	28.929	0.00	0.00	PROA
ATOM	293	HE1	TYR	P	21	-4.902	15.189	28.067	0.00	0.00	PROA
ATOM	294	CZ	TYR	P	21	-6.743	16.002	29.006	0.00	0.00	PROA
ATOM	295	OH	TYR	P	21	-7.579	15.575	27.974	0.00	0.00	PROA
ATOM	296	HH	TYR	P	21	-8.458	15.937	28.111	0.00	0.00	PROA
ATOM	297	CD2	TYR	P	21	-6.300	17.238	31.085	0.00	0.00	PROA
ATOM	298	HD2	TYR	P	21	-6.653	17.827	31.918	0.00	0.00	PROA
ATOM	299	CE2	TYR	P	21	-7.224	16.862	30.038	0.00	0.00	PROA
ATOM	300	HE2	TYR	P	21	-8.266	17.146	30.040	0.00	0.00	PROA

```
ATOM    301  C   TYR P  21      -2.861  15.034  32.528  0.00  0.00      PROA
ATOM    302  O   TYR P  21      -3.230  13.941  32.173  0.00  0.00      PROA
ATOM    303  N   ASP P  22      -1.566  15.418  32.404  0.00  0.00      PROA
ATOM    304  HN  ASP P  22      -1.315  16.338  32.696  0.00  0.00      PROA
ATOM    305  CA  ASP P  22      -0.464  14.595  31.847  0.00  0.00      PROA
ATOM    306  HA  ASP P  22      -0.809  14.402  30.842  0.00  0.00      PROA
ATOM    307  CB  ASP P  22       0.959  15.286  31.873  0.00  0.00      PROA
ATOM    308  HB1 ASP P  22       1.719  14.539  31.559  0.00  0.00      PROA
ATOM    309  HB2 ASP P  22       1.156  15.694  32.887  0.00  0.00      PROA
ATOM    310  CG  ASP P  22       1.027  16.543  31.028  0.00  0.00      PROA
ATOM    311  OD1 ASP P  22       1.579  17.542  31.485  0.00  0.00      PROA
ATOM    312  OD2 ASP P  22       0.470  16.469  29.920  0.00  0.00      PROA
ATOM    313  C   ASP P  22      -0.330  13.192  32.578  0.00  0.00      PROA
ATOM    314  O   ASP P  22      -0.229  12.093  32.009  0.00  0.00      PROA
ATOM    315  N   SER P  23      -0.460  13.264  33.936  0.00  0.00      PROA
ATOM    316  HN  SER P  23      -0.433  14.161  34.369  0.00  0.00      PROA
ATOM    317  CA  SER P  23      -0.569  12.028  34.759  0.00  0.00      PROA
ATOM    318  HA  SER P  23       0.346  11.471  34.624  0.00  0.00      PROA
ATOM    319  CB  SER P  23      -0.603  12.378  36.215  0.00  0.00      PROA
ATOM    320  HB1 SER P  23      -0.660  11.479  36.866  0.00  0.00      PROA
ATOM    321  HB2 SER P  23      -1.542  12.907  36.485  0.00  0.00      PROA
ATOM    322  OG  SER P  23       0.520  13.176  36.587  0.00  0.00      PROA
ATOM    323  HG1 SER P  23       0.518  14.001  36.096  0.00  0.00      PROA
ATOM    324  C   SER P  23      -1.813  11.114  34.488  0.00  0.00      PROA
ATOM    325  O   SER P  23      -1.777   9.881  34.495  0.00  0.00      PROA
ATOM    326  N   MET P  24      -3.025  11.718  34.230  0.00  0.00      PROA
ATOM    327  HN  MET P  24      -2.949  12.707  34.138  0.00  0.00      PROA
ATOM    328  CA  MET P  24      -4.313  11.086  33.950  0.00  0.00      PROA
ATOM    329  HA  MET P  24      -4.532  10.476  34.813  0.00  0.00      PROA
ATOM    330  CB  MET P  24      -5.423  12.202  33.845  0.00  0.00      PROA
ATOM    331  HB1 MET P  24      -5.238  12.854  32.964  0.00  0.00      PROA
ATOM    332  HB2 MET P  24      -5.341  12.880  34.721  0.00  0.00      PROA
ATOM    333  CG  MET P  24      -6.940  11.803  33.819  0.00  0.00      PROA
ATOM    334  HG1 MET P  24      -7.416  12.713  34.243  0.00  0.00      PROA
ATOM    335  HG2 MET P  24      -7.051  10.996  34.574  0.00  0.00      PROA
ATOM    336  SD  MET P  24      -7.795  11.485  32.255  0.00  0.00      PROA
ATOM    337  CE  MET P  24      -7.883  13.200  31.719  0.00  0.00      PROA
ATOM    338  HE1 MET P  24      -8.317  13.186  30.697  0.00  0.00      PROA
ATOM    339  HE2 MET P  24      -6.908  13.704  31.547  0.00  0.00      PROA
ATOM    340  HE3 MET P  24      -8.556  13.843  32.326  0.00  0.00      PROA
ATOM    341  C   MET P  24      -4.239  10.243  32.637  0.00  0.00      PROA
ATOM    342  O   MET P  24      -4.812   9.185  32.511  0.00  0.00      PROA
ATOM    343  N   LYS P  25      -3.320  10.742  31.736  0.00  0.00      PROA
ATOM    344  HN  LYS P  25      -2.838  11.580  31.980  0.00  0.00      PROA
ATOM    345  CA  LYS P  25      -3.117  10.046  30.474  0.00  0.00      PROA
ATOM    346  HA  LYS P  25      -4.030   9.597  30.109  0.00  0.00      PROA
ATOM    347  CB  LYS P  25      -2.588  10.939  29.327  0.00  0.00      PROA
ATOM    348  HB1 LYS P  25      -2.371  10.224  28.506  0.00  0.00      PROA
ATOM    349  HB2 LYS P  25      -1.617  11.363  29.664  0.00  0.00      PROA
ATOM    350  CG  LYS P  25      -3.503  12.063  28.913  0.00  0.00      PROA
ATOM    351  HG1 LYS P  25      -2.837  12.623  28.222  0.00  0.00      PROA
ATOM    352  HG2 LYS P  25      -3.840  12.732  29.734  0.00  0.00      PROA
ATOM    353  CD  LYS P  25      -4.734  11.702  28.081  0.00  0.00      PROA
ATOM    354  HD1 LYS P  25      -5.200  12.625  27.674  0.00  0.00      PROA
ATOM    355  HD2 LYS P  25      -5.468  11.183  28.733  0.00  0.00      PROA
ATOM    356  CE  LYS P  25      -4.471  10.860  26.809  0.00  0.00      PROA
ATOM    357  HE1 LYS P  25      -5.397  10.618  26.244  0.00  0.00      PROA
ATOM    358  HE2 LYS P  25      -3.999   9.886  27.057  0.00  0.00      PROA
ATOM    359  NZ  LYS P  25      -3.582  11.470  25.772  0.00  0.00      PROA
ATOM    360  HZ1 LYS P  25      -3.659  10.940  24.880  0.00  0.00      PROA
ATOM    361  HZ2 LYS P  25      -2.617  11.394  26.150  0.00  0.00      PROA
```

ATOM	362	HZ3	LYS	P	25	-3.791	12.477	25.621	0.00	0.00	PROA
ATOM	363	C	LYS	P	25	-2.068	8.918	30.578	0.00	0.00	PROA
ATOM	364	O	LYS	P	25	-2.009	8.093	29.710	0.00	0.00	PROA
ATOM	365	N	GLU	P	26	-1.237	8.781	31.656	0.00	0.00	PROA
ATOM	366	HN	GLU	P	26	-1.132	9.462	32.376	0.00	0.00	PROA
ATOM	367	CA	GLU	P	26	-0.457	7.614	31.877	0.00	0.00	PROA
ATOM	368	HA	GLU	P	26	0.178	7.637	31.004	0.00	0.00	PROA
ATOM	369	CB	GLU	P	26	0.461	7.922	33.092	0.00	0.00	PROA
ATOM	370	HB1	GLU	P	26	-0.190	8.237	33.935	0.00	0.00	PROA
ATOM	371	HB2	GLU	P	26	1.140	8.745	32.780	0.00	0.00	PROA
ATOM	372	CG	GLU	P	26	1.281	6.678	33.508	0.00	0.00	PROA
ATOM	373	HG1	GLU	P	26	1.875	6.244	32.676	0.00	0.00	PROA
ATOM	374	HG2	GLU	P	26	0.659	5.868	33.945	0.00	0.00	PROA
ATOM	375	CD	GLU	P	26	2.307	6.952	34.578	0.00	0.00	PROA
ATOM	376	OE1	GLU	P	26	2.031	6.509	35.722	0.00	0.00	PROA
ATOM	377	OE2	GLU	P	26	3.386	7.455	34.213	0.00	0.00	PROA
ATOM	378	C	GLU	P	26	-0.990	6.188	31.892	0.00	0.00	PROA
ATOM	379	O	GLU	P	26	-0.415	5.261	31.219	0.00	0.00	PROA
ATOM	380	N	PRO	P	27	-2.055	5.895	32.606	0.00	0.00	PROA
ATOM	381	CD	PRO	P	27	-2.632	6.715	33.740	0.00	0.00	PROA
ATOM	382	HD1	PRO	P	27	-2.925	7.765	33.526	0.00	0.00	PROA
ATOM	383	HD2	PRO	P	27	-1.867	6.694	34.545	0.00	0.00	PROA
ATOM	384	CA	PRO	P	27	-2.639	4.519	32.638	0.00	0.00	PROA
ATOM	385	HA	PRO	P	27	-1.833	3.829	32.836	0.00	0.00	PROA
ATOM	386	CB	PRO	P	27	-3.513	4.418	33.890	0.00	0.00	PROA
ATOM	387	HB1	PRO	P	27	-2.939	4.137	34.798	0.00	0.00	PROA
ATOM	388	HB2	PRO	P	27	-4.438	3.801	33.899	0.00	0.00	PROA
ATOM	389	CG	PRO	P	27	-3.844	5.932	34.142	0.00	0.00	PROA
ATOM	390	HG1	PRO	P	27	-4.642	6.047	33.378	0.00	0.00	PROA
ATOM	391	HG2	PRO	P	27	-4.206	6.048	35.186	0.00	0.00	PROA
ATOM	392	C	PRO	P	27	-3.419	4.086	31.425	0.00	0.00	PROA
ATOM	393	O	PRO	P	27	-3.656	2.879	31.218	0.00	0.00	PROA
ATOM	394	N	CYS	P	28	-3.930	5.034	30.688	0.00	0.00	PROA
ATOM	395	HN	CYS	P	28	-3.684	5.959	30.967	0.00	0.00	PROA
ATOM	396	CA	CYS	P	28	-4.638	4.928	29.432	0.00	0.00	PROA
ATOM	397	HA	CYS	P	28	-5.427	4.243	29.706	0.00	0.00	PROA
ATOM	398	CB	CYS	P	28	-5.163	6.291	28.843	0.00	0.00	PROA
ATOM	399	HB1	CYS	P	28	-5.657	6.216	27.851	0.00	0.00	PROA
ATOM	400	HB2	CYS	P	28	-4.296	6.968	28.688	0.00	0.00	PROA
ATOM	401	SG	CYS	P	28	-6.271	7.107	30.058	0.00	0.00	PROA
ATOM	402	C	CYS	P	28	-3.748	4.234	28.340	0.00	0.00	PROA
ATOM	403	O	CYS	P	28	-2.515	4.374	28.227	0.00	0.00	PROA
ATOM	404	N	PHE	P	29	-4.389	3.392	27.477	0.00	0.00	PROA
ATOM	405	HN	PHE	P	29	-5.385	3.363	27.444	0.00	0.00	PROA
ATOM	406	CA	PHE	P	29	-3.784	2.704	26.394	0.00	0.00	PROA
ATOM	407	HA	PHE	P	29	-2.858	3.137	26.047	0.00	0.00	PROA
ATOM	408	CB	PHE	P	29	-3.174	1.329	26.873	0.00	0.00	PROA
ATOM	409	HB1	PHE	P	29	-3.949	0.596	27.182	0.00	0.00	PROA
ATOM	410	HB2	PHE	P	29	-2.663	1.518	27.841	0.00	0.00	PROA
ATOM	411	CG	PHE	P	29	-2.158	0.670	26.006	0.00	0.00	PROA
ATOM	412	CD1	PHE	P	29	-2.471	-0.671	25.507	0.00	0.00	PROA
ATOM	413	HD1	PHE	P	29	-3.309	-1.271	25.829	0.00	0.00	PROA
ATOM	414	CE1	PHE	P	29	-1.511	-1.314	24.650	0.00	0.00	PROA
ATOM	415	HE1	PHE	P	29	-1.542	-2.341	24.320	0.00	0.00	PROA
ATOM	416	CZ	PHE	P	29	-0.253	-0.633	24.273	0.00	0.00	PROA
ATOM	417	HZ	PHE	P	29	0.317	-1.167	23.527	0.00	0.00	PROA
ATOM	418	CD2	PHE	P	29	-0.985	1.265	25.566	0.00	0.00	PROA
ATOM	419	HD2	PHE	P	29	-0.833	2.296	25.849	0.00	0.00	PROA
ATOM	420	CE2	PHE	P	29	-0.126	0.681	24.644	0.00	0.00	PROA
ATOM	421	HE2	PHE	P	29	0.748	1.211	24.294	0.00	0.00	PROA
ATOM	422	C	PHE	P	29	-4.734	2.590	25.227	0.00	0.00	PROA

ATOM	423	O	PHE	P	29	-5.909	2.564	25.323	0.00	0.00	PROA
ATOM	424	N	ARG	P	30	-4.236	2.778	23.984	0.00	0.00	PROA
ATOM	425	HN	ARG	P	30	-3.300	3.109	23.890	0.00	0.00	PROA
ATOM	426	CA	ARG	P	30	-4.816	2.395	22.729	0.00	0.00	PROA
ATOM	427	HA	ARG	P	30	-5.885	2.544	22.716	0.00	0.00	PROA
ATOM	428	CB	ARG	P	30	-4.182	3.286	21.565	0.00	0.00	PROA
ATOM	429	HB1	ARG	P	30	-4.784	2.842	20.744	0.00	0.00	PROA
ATOM	430	HB2	ARG	P	30	-3.103	3.093	21.381	0.00	0.00	PROA
ATOM	431	CG	ARG	P	30	-4.503	4.832	21.666	0.00	0.00	PROA
ATOM	432	HG1	ARG	P	30	-4.275	5.270	22.661	0.00	0.00	PROA
ATOM	433	HG2	ARG	P	30	-5.593	4.865	21.456	0.00	0.00	PROA
ATOM	434	CD	ARG	P	30	-3.686	5.790	20.762	0.00	0.00	PROA
ATOM	435	HD1	ARG	P	30	-2.600	5.594	20.887	0.00	0.00	PROA
ATOM	436	HD2	ARG	P	30	-3.809	6.871	20.987	0.00	0.00	PROA
ATOM	437	NE	ARG	P	30	-4.107	5.538	19.347	0.00	0.00	PROA
ATOM	438	HE	ARG	P	30	-4.493	4.665	19.048	0.00	0.00	PROA
ATOM	439	CZ	ARG	P	30	-3.377	6.042	18.395	0.00	0.00	PROA
ATOM	440	NH1	ARG	P	30	-2.613	7.127	18.435	0.00	0.00	PROA
ATOM	441	HH11	ARG	P	30	-2.700	7.701	19.250	0.00	0.00	PROA
ATOM	442	HH12	ARG	P	30	-1.999	7.382	17.688	0.00	0.00	PROA
ATOM	443	NH2	ARG	P	30	-3.516	5.442	17.249	0.00	0.00	PROA
ATOM	444	HH21	ARG	P	30	-3.133	5.918	16.458	0.00	0.00	PROA
ATOM	445	HH22	ARG	P	30	-4.266	4.791	17.130	0.00	0.00	PROA
ATOM	446	C	ARG	P	30	-4.677	0.846	22.446	0.00	0.00	PROA
ATOM	447	O	ARG	P	30	-3.609	0.282	22.511	0.00	0.00	PROA
ATOM	448	N	GLU	P	31	-5.782	0.051	22.281	0.00	0.00	PROA
ATOM	449	HN	GLU	P	31	-6.663	0.502	22.161	0.00	0.00	PROA
ATOM	450	CA	GLU	P	31	-5.691	-1.318	22.023	0.00	0.00	PROA
ATOM	451	HA	GLU	P	31	-4.760	-1.513	21.513	0.00	0.00	PROA
ATOM	452	CB	GLU	P	31	-5.535	-2.184	23.321	0.00	0.00	PROA
ATOM	453	HB1	GLU	P	31	-6.447	-1.849	23.859	0.00	0.00	PROA
ATOM	454	HB2	GLU	P	31	-4.608	-1.800	23.799	0.00	0.00	PROA
ATOM	455	CG	GLU	P	31	-5.496	-3.735	23.226	0.00	0.00	PROA
ATOM	456	HG1	GLU	P	31	-6.505	-4.040	22.874	0.00	0.00	PROA
ATOM	457	HG2	GLU	P	31	-5.458	-4.058	24.288	0.00	0.00	PROA
ATOM	458	CD	GLU	P	31	-4.356	-4.296	22.396	0.00	0.00	PROA
ATOM	459	OE1	GLU	P	31	-3.508	-4.990	22.983	0.00	0.00	PROA
ATOM	460	OE2	GLU	P	31	-4.230	-3.965	21.152	0.00	0.00	PROA
ATOM	461	C	GLU	P	31	-6.813	-1.853	21.136	0.00	0.00	PROA
ATOM	462	O	GLU	P	31	-7.904	-1.301	21.074	0.00	0.00	PROA
ATOM	463	N	GLU	P	32	-6.485	-2.930	20.347	0.00	0.00	PROA
ATOM	464	HN	GLU	P	32	-5.554	-3.252	20.504	0.00	0.00	PROA
ATOM	465	CA	GLU	P	32	-7.276	-3.457	19.191	0.00	0.00	PROA
ATOM	466	HA	GLU	P	32	-7.532	-2.604	18.580	0.00	0.00	PROA
ATOM	467	CB	GLU	P	32	-6.448	-4.402	18.321	0.00	0.00	PROA
ATOM	468	HB1	GLU	P	32	-7.089	-4.677	17.456	0.00	0.00	PROA
ATOM	469	HB2	GLU	P	32	-6.012	-5.321	18.768	0.00	0.00	PROA
ATOM	470	CG	GLU	P	32	-5.219	-3.607	17.807	0.00	0.00	PROA
ATOM	471	HG1	GLU	P	32	-4.574	-3.287	18.653	0.00	0.00	PROA
ATOM	472	HG2	GLU	P	32	-5.640	-2.685	17.352	0.00	0.00	PROA
ATOM	473	CD	GLU	P	32	-4.493	-4.420	16.818	0.00	0.00	PROA
ATOM	474	OE1	GLU	P	32	-4.443	-4.242	15.608	0.00	0.00	PROA
ATOM	475	OE2	GLU	P	32	-3.877	-5.473	17.308	0.00	0.00	PROA
ATOM	476	C	GLU	P	32	-8.537	-4.199	19.741	0.00	0.00	PROA
ATOM	477	O	GLU	P	32	-8.749	-4.437	20.933	0.00	0.00	PROA
ATOM	478	N	ASN	P	33	-9.447	-4.498	18.794	0.00	0.00	PROA
ATOM	479	HN	ASN	P	33	-9.320	-4.281	17.829	0.00	0.00	PROA
ATOM	480	CA	ASN	P	33	-10.630	-5.215	19.185	0.00	0.00	PROA
ATOM	481	HA	ASN	P	33	-10.890	-4.971	20.204	0.00	0.00	PROA
ATOM	482	CB	ASN	P	33	-11.764	-4.736	18.231	0.00	0.00	PROA
ATOM	483	HB1	ASN	P	33	-11.528	-4.675	17.147	0.00	0.00	PROA

ATOM 484 HB2 ASN P 33 -11.967 -3.656 18.395 0.00 0.00 PROA
ATOM 485 CG ASN P 33 -13.119 -5.575 18.285 0.00 0.00 PROA
ATOM 486 OD1 ASN P 33 -13.126 -6.521 19.134 0.00 0.00 PROA
ATOM 487 ND2 ASN P 33 -14.124 -5.236 17.502 0.00 0.00 PROA
ATOM 488 HD21 ASN P 33 -14.946 -5.800 17.422 0.00 0.00 PROA
ATOM 489 HD22 ASN P 33 -14.055 -4.481 16.850 0.00 0.00 PROA
ATOM 490 C ASN P 33 -10.306 -6.718 19.037 0.00 0.00 PROA
ATOM 491 O ASN P 33 -10.049 -7.307 18.000 0.00 0.00 PROA
ATOM 492 N ALA P 34 -10.367 -7.519 20.154 0.00 0.00 PROA
ATOM 493 HN ALA P 34 -10.565 -7.021 20.995 0.00 0.00 PROA
ATOM 494 CA ALA P 34 -10.115 -8.907 20.198 0.00 0.00 PROA
ATOM 495 HA ALA P 34 -9.112 -9.154 19.884 0.00 0.00 PROA
ATOM 496 CB ALA P 34 -10.039 -9.425 21.682 0.00 0.00 PROA
ATOM 497 HB1 ALA P 34 -9.500 -10.397 21.685 0.00 0.00 PROA
ATOM 498 HB2 ALA P 34 -11.028 -9.449 22.187 0.00 0.00 PROA
ATOM 499 HB3 ALA P 34 -9.397 -8.694 22.218 0.00 0.00 PROA
ATOM 500 C ALA P 34 -11.121 -9.761 19.433 0.00 0.00 PROA
ATOM 501 O ALA P 34 -10.771 -10.746 18.796 0.00 0.00 PROA
ATOM 502 N ASN P 35 -12.419 -9.486 19.483 0.00 0.00 PROA
ATOM 503 HN ASN P 35 -12.655 -8.741 20.102 0.00 0.00 PROA
ATOM 504 CA ASN P 35 -13.499 -10.147 18.742 0.00 0.00 PROA
ATOM 505 HA ASN P 35 -13.442 -11.187 19.028 0.00 0.00 PROA
ATOM 506 CB ASN P 35 -14.962 -9.747 19.326 0.00 0.00 PROA
ATOM 507 HB1 ASN P 35 -14.842 -8.649 19.447 0.00 0.00 PROA
ATOM 508 HB2 ASN P 35 -15.082 -10.213 20.327 0.00 0.00 PROA
ATOM 509 CG ASN P 35 -16.147 -10.052 18.407 0.00 0.00 PROA
ATOM 510 OD1 ASN P 35 -16.276 -9.669 17.253 0.00 0.00 PROA
ATOM 511 ND2 ASN P 35 -17.020 -10.945 18.973 0.00 0.00 PROA
ATOM 512 HD21 ASN P 35 -17.026 -11.309 19.905 0.00 0.00 PROA
ATOM 513 HD22 ASN P 35 -17.726 -11.307 18.364 0.00 0.00 PROA
ATOM 514 C ASN P 35 -13.279 -10.152 17.227 0.00 0.00 PROA
ATOM 515 O ASN P 35 -13.391 -11.155 16.545 0.00 0.00 PROA
ATOM 516 N PHE P 36 -12.838 -8.976 16.708 0.00 0.00 PROA
ATOM 517 HN PHE P 36 -12.755 -8.282 17.419 0.00 0.00 PROA
ATOM 518 CA PHE P 36 -12.512 -8.792 15.313 0.00 0.00 PROA
ATOM 519 HA PHE P 36 -13.431 -9.083 14.826 0.00 0.00 PROA
ATOM 520 CB PHE P 36 -12.304 -7.268 15.174 0.00 0.00 PROA
ATOM 521 HB1 PHE P 36 -11.428 -7.014 15.809 0.00 0.00 PROA
ATOM 522 HB2 PHE P 36 -13.238 -6.802 15.554 0.00 0.00 PROA
ATOM 523 CG PHE P 36 -12.034 -6.786 13.733 0.00 0.00 PROA
ATOM 524 CD1 PHE P 36 -13.187 -6.793 12.859 0.00 0.00 PROA
ATOM 525 HD1 PHE P 36 -14.168 -7.028 13.244 0.00 0.00 PROA
ATOM 526 CE1 PHE P 36 -13.045 -6.236 11.568 0.00 0.00 PROA
ATOM 527 HE1 PHE P 36 -13.915 -6.154 10.934 0.00 0.00 PROA
ATOM 528 CZ PHE P 36 -11.846 -5.585 11.242 0.00 0.00 PROA
ATOM 529 HZ PHE P 36 -11.605 -5.142 10.287 0.00 0.00 PROA
ATOM 530 CD2 PHE P 36 -10.795 -6.348 13.329 0.00 0.00 PROA
ATOM 531 HD2 PHE P 36 -9.958 -6.371 14.011 0.00 0.00 PROA
ATOM 532 CE2 PHE P 36 -10.715 -5.631 12.092 0.00 0.00 PROA
ATOM 533 HE2 PHE P 36 -9.747 -5.211 11.859 0.00 0.00 PROA
ATOM 534 C PHE P 36 -11.403 -9.640 14.875 0.00 0.00 PROA
ATOM 535 O PHE P 36 -11.563 -10.266 13.804 0.00 0.00 PROA
ATOM 536 N ASN P 37 -10.307 -9.696 15.638 0.00 0.00 PROA
ATOM 537 HN ASN P 37 -10.232 -9.000 16.347 0.00 0.00 PROA
ATOM 538 CA ASN P 37 -9.181 -10.432 15.388 0.00 0.00 PROA
ATOM 539 HA ASN P 37 -8.979 -10.222 14.348 0.00 0.00 PROA
ATOM 540 CB ASN P 37 -7.971 -9.941 16.137 0.00 0.00 PROA
ATOM 541 HB1 ASN P 37 -7.142 -10.678 16.080 0.00 0.00 PROA
ATOM 542 HB2 ASN P 37 -8.210 -9.866 17.220 0.00 0.00 PROA
ATOM 543 CG ASN P 37 -7.495 -8.667 15.545 0.00 0.00 PROA
ATOM 544 OD1 ASN P 37 -6.504 -8.650 14.849 0.00 0.00 PROA

ATOM	545	ND2	ASN	P	37	-8.162	-7.527	15.882	0.00	0.00	PROA
ATOM	546	HD21	ASN	P	37	-8.799	-7.560	16.652	0.00	0.00	PROA
ATOM	547	HD22	ASN	P	37	-7.760	-6.665	15.572	0.00	0.00	PROA
ATOM	548	C	ASN	P	37	-9.418	-11.942	15.548	0.00	0.00	PROA
ATOM	549	O	ASN	P	37	-8.933	-12.695	14.725	0.00	0.00	PROA
ATOM	550	N	LYS	P	38	-10.125	-12.385	16.557	0.00	0.00	PROA
ATOM	551	HN	LYS	P	38	-10.449	-11.678	17.182	0.00	0.00	PROA
ATOM	552	CA	LYS	P	38	-10.457	-13.782	16.774	0.00	0.00	PROA
ATOM	553	HA	LYS	P	38	-9.554	-14.294	16.474	0.00	0.00	PROA
ATOM	554	CB	LYS	P	38	-10.827	-14.062	18.250	0.00	0.00	PROA
ATOM	555	HB1	LYS	P	38	-11.275	-15.070	18.381	0.00	0.00	PROA
ATOM	556	HB2	LYS	P	38	-11.622	-13.309	18.438	0.00	0.00	PROA
ATOM	557	CG	LYS	P	38	-9.616	-13.826	19.259	0.00	0.00	PROA
ATOM	558	HG1	LYS	P	38	-9.132	-12.838	19.105	0.00	0.00	PROA
ATOM	559	HG2	LYS	P	38	-8.854	-14.634	19.278	0.00	0.00	PROA
ATOM	560	CD	LYS	P	38	-10.102	-13.843	20.797	0.00	0.00	PROA
ATOM	561	HD1	LYS	P	38	-10.498	-14.868	20.958	0.00	0.00	PROA
ATOM	562	HD2	LYS	P	38	-10.886	-13.078	20.985	0.00	0.00	PROA
ATOM	563	CE	LYS	P	38	-8.966	-13.484	21.815	0.00	0.00	PROA
ATOM	564	HE1	LYS	P	38	-8.610	-12.435	21.726	0.00	0.00	PROA
ATOM	565	HE2	LYS	P	38	-8.172	-14.221	21.570	0.00	0.00	PROA
ATOM	566	NZ	LYS	P	38	-9.480	-13.713	23.168	0.00	0.00	PROA
ATOM	567	HZ1	LYS	P	38	-9.967	-12.849	23.482	0.00	0.00	PROA
ATOM	568	HZ2	LYS	P	38	-8.675	-13.938	23.786	0.00	0.00	PROA
ATOM	569	HZ3	LYS	P	38	-10.153	-14.504	23.225	0.00	0.00	PROA
ATOM	570	C	LYS	P	38	-11.618	-14.377	15.922	0.00	0.00	PROA
ATOM	571	O	LYS	P	38	-11.567	-15.593	15.658	0.00	0.00	PROA
ATOM	572	N	ILE	P	39	-12.696	-13.611	15.502	0.00	0.00	PROA
ATOM	573	HN	ILE	P	39	-12.780	-12.655	15.772	0.00	0.00	PROA
ATOM	574	CA	ILE	P	39	-13.959	-14.162	14.892	0.00	0.00	PROA
ATOM	575	HA	ILE	P	39	-13.872	-15.238	14.849	0.00	0.00	PROA
ATOM	576	CB	ILE	P	39	-15.143	-13.807	15.786	0.00	0.00	PROA
ATOM	577	HB	ILE	P	39	-15.369	-12.727	15.651	0.00	0.00	PROA
ATOM	578	CG2	ILE	P	39	-16.332	-14.620	15.257	0.00	0.00	PROA
ATOM	579	HG21	ILE	P	39	-17.172	-14.379	15.943	0.00	0.00	PROA
ATOM	580	HG22	ILE	P	39	-16.055	-15.687	15.397	0.00	0.00	PROA
ATOM	581	HG23	ILE	P	39	-16.531	-14.469	14.174	0.00	0.00	PROA
ATOM	582	CG1	ILE	P	39	-14.919	-14.200	17.237	0.00	0.00	PROA
ATOM	583	HG11	ILE	P	39	-15.880	-13.945	17.732	0.00	0.00	PROA
ATOM	584	HG12	ILE	P	39	-14.085	-13.632	17.702	0.00	0.00	PROA
ATOM	585	CD	ILE	P	39	-14.769	-15.740	17.452	0.00	0.00	PROA
ATOM	586	HD1	ILE	P	39	-14.341	-15.979	18.448	0.00	0.00	PROA
ATOM	587	HD2	ILE	P	39	-14.022	-16.193	16.765	0.00	0.00	PROA
ATOM	588	HD3	ILE	P	39	-15.721	-16.311	17.390	0.00	0.00	PROA
ATOM	589	C	ILE	P	39	-14.169	-13.699	13.505	0.00	0.00	PROA
ATOM	590	O	ILE	P	39	-14.552	-14.464	12.647	0.00	0.00	PROA
ATOM	591	N	PHE	P	40	-13.829	-12.402	13.154	0.00	0.00	PROA
ATOM	592	HN	PHE	P	40	-13.397	-11.865	13.875	0.00	0.00	PROA
ATOM	593	CA	PHE	P	40	-14.275	-11.769	12.010	0.00	0.00	PROA
ATOM	594	HA	PHE	P	40	-15.080	-12.286	11.508	0.00	0.00	PROA
ATOM	595	CB	PHE	P	40	-14.734	-10.356	12.362	0.00	0.00	PROA
ATOM	596	HB1	PHE	P	40	-14.744	-9.725	11.448	0.00	0.00	PROA
ATOM	597	HB2	PHE	P	40	-14.084	-9.912	13.146	0.00	0.00	PROA
ATOM	598	CG	PHE	P	40	-16.158	-10.476	12.781	0.00	0.00	PROA
ATOM	599	CD1	PHE	P	40	-16.502	-10.241	14.126	0.00	0.00	PROA
ATOM	600	HD1	PHE	P	40	-15.696	-9.933	14.776	0.00	0.00	PROA
ATOM	601	CE1	PHE	P	40	-17.870	-10.288	14.503	0.00	0.00	PROA
ATOM	602	HE1	PHE	P	40	-18.164	-10.161	15.534	0.00	0.00	PROA
ATOM	603	CZ	PHE	P	40	-18.854	-10.391	13.519	0.00	0.00	PROA
ATOM	604	HZ	PHE	P	40	-19.904	-10.393	13.769	0.00	0.00	PROA
ATOM	605	CD2	PHE	P	40	-17.157	-10.588	11.835	0.00	0.00	PROA

```
ATOM    606  HD2 PHE P  40     -16.852 -10.605  10.799  0.00  0.00      PROA
ATOM    607  CE2 PHE P  40     -18.503 -10.598  12.147  0.00  0.00      PROA
ATOM    608  HE2 PHE P  40     -19.345 -10.610  11.471  0.00  0.00      PROA
ATOM    609  C   PHE P  40     -13.168 -11.817  10.927  0.00  0.00      PROA
ATOM    610  O   PHE P  40     -13.295 -12.289   9.777  0.00  0.00      PROA
ATOM    611  N   LEU P  41     -11.986 -11.311  11.266  0.00  0.00      PROA
ATOM    612  HN  LEU P  41     -11.886 -10.964  12.195  0.00  0.00      PROA
ATOM    613  CA  LEU P  41     -10.792 -11.272  10.363  0.00  0.00      PROA
ATOM    614  HA  LEU P  41     -11.020 -10.704   9.473  0.00  0.00      PROA
ATOM    615  CB  LEU P  41      -9.506 -10.651  10.984  0.00  0.00      PROA
ATOM    616  HB1 LEU P  41      -8.671 -11.161  10.457  0.00  0.00      PROA
ATOM    617  HB2 LEU P  41      -9.341 -11.033  12.014  0.00  0.00      PROA
ATOM    618  CG  LEU P  41      -9.386  -9.093  11.086  0.00  0.00      PROA
ATOM    619  HG  LEU P  41     -10.340  -8.773  11.558  0.00  0.00      PROA
ATOM    620  CD1 LEU P  41      -8.180  -8.562  11.849  0.00  0.00      PROA
ATOM    621 HD11 LEU P  41      -7.957  -9.158  12.760  0.00  0.00      PROA
ATOM    622 HD12 LEU P  41      -8.299  -7.487  12.104  0.00  0.00      PROA
ATOM    623 HD13 LEU P  41      -7.213  -8.727  11.327  0.00  0.00      PROA
ATOM    624  CD2 LEU P  41      -9.327  -8.510   9.724  0.00  0.00      PROA
ATOM    625 HD21 LEU P  41      -8.400  -8.840   9.208  0.00  0.00      PROA
ATOM    626 HD22 LEU P  41      -9.371  -7.402   9.798  0.00  0.00      PROA
ATOM    627 HD23 LEU P  41     -10.043  -8.946   8.995  0.00  0.00      PROA
ATOM    628  C   LEU P  41     -10.410 -12.639   9.820  0.00  0.00      PROA
ATOM    629  O   LEU P  41     -10.188 -12.627   8.613  0.00  0.00      PROA
ATOM    630  N   PRO P  42     -10.264 -13.810  10.483  0.00  0.00      PROA
ATOM    631  CD  PRO P  42     -10.418 -13.926  11.969  0.00  0.00      PROA
ATOM    632  HD1 PRO P  42     -11.505 -13.989  12.189  0.00  0.00      PROA
ATOM    633  HD2 PRO P  42      -9.850 -13.139  12.510  0.00  0.00      PROA
ATOM    634  CA  PRO P  42     -10.059 -15.113   9.822  0.00  0.00      PROA
ATOM    635  HA  PRO P  42      -9.068 -15.208   9.403  0.00  0.00      PROA
ATOM    636  CB  PRO P  42     -10.152 -16.143  11.064  0.00  0.00      PROA
ATOM    637  HB1 PRO P  42      -9.464 -16.954  10.743  0.00  0.00      PROA
ATOM    638  HB2 PRO P  42     -11.186 -16.524  11.205  0.00  0.00      PROA
ATOM    639  CG  PRO P  42      -9.819 -15.302  12.275  0.00  0.00      PROA
ATOM    640  HG1 PRO P  42     -10.224 -15.756  13.204  0.00  0.00      PROA
ATOM    641  HG2 PRO P  42      -8.717 -15.201  12.372  0.00  0.00      PROA
ATOM    642  C   PRO P  42     -10.981 -15.463   8.652  0.00  0.00      PROA
ATOM    643  O   PRO P  42     -10.480 -15.972   7.571  0.00  0.00      PROA
ATOM    644  N   THR P  43     -12.334 -15.311   8.692  0.00  0.00      PROA
ATOM    645  HN  THR P  43     -12.806 -14.965   9.499  0.00  0.00      PROA
ATOM    646  CA  THR P  43     -13.273 -15.422   7.601  0.00  0.00      PROA
ATOM    647  HA  THR P  43     -13.033 -16.422   7.272  0.00  0.00      PROA
ATOM    648  CB  THR P  43     -14.712 -15.338   8.037  0.00  0.00      PROA
ATOM    649  HB  THR P  43     -14.913 -14.569   8.813  0.00  0.00      PROA
ATOM    650  OG1 THR P  43     -15.199 -16.497   8.576  0.00  0.00      PROA
ATOM    651  HG1 THR P  43     -14.742 -16.735   9.387  0.00  0.00      PROA
ATOM    652  CG2 THR P  43     -15.766 -15.066   6.953  0.00  0.00      PROA
ATOM    653 HG21 THR P  43     -16.690 -14.778   7.500  0.00  0.00      PROA
ATOM    654 HG22 THR P  43     -15.963 -15.945   6.303  0.00  0.00      PROA
ATOM    655 HG23 THR P  43     -15.586 -14.181   6.306  0.00  0.00      PROA
ATOM    656  C   THR P  43     -12.917 -14.509   6.434  0.00  0.00      PROA
ATOM    657  O   THR P  43     -12.776 -14.889   5.270  0.00  0.00      PROA
ATOM    658  N   ILE P  44     -12.642 -13.211   6.765  0.00  0.00      PROA
ATOM    659  HN  ILE P  44     -12.778 -12.802   7.664  0.00  0.00      PROA
ATOM    660  CA  ILE P  44     -12.263 -12.229   5.764  0.00  0.00      PROA
ATOM    661  HA  ILE P  44     -12.989 -12.255   4.965  0.00  0.00      PROA
ATOM    662  CB  ILE P  44     -12.225 -10.859   6.373  0.00  0.00      PROA
ATOM    663  HB  ILE P  44     -11.559 -10.926   7.259  0.00  0.00      PROA
ATOM    664  CG2 ILE P  44     -11.634  -9.815   5.372  0.00  0.00      PROA
ATOM    665 HG21 ILE P  44     -11.747  -8.827   5.866  0.00  0.00      PROA
ATOM    666 HG22 ILE P  44     -12.275  -9.779   4.466  0.00  0.00      PROA
```

```
ATOM    667 HG23 ILE P  44     -10.578 -10.010   5.086  0.00  0.00      PROA
ATOM    668  CG1 ILE P  44     -13.683 -10.423   6.884  0.00  0.00      PROA
ATOM    669 HG11 ILE P  44     -14.209 -11.298   7.323  0.00  0.00      PROA
ATOM    670 HG12 ILE P  44     -14.117 -10.013   5.947  0.00  0.00      PROA
ATOM    671  CD  ILE P  44     -13.684  -9.394   7.998  0.00  0.00      PROA
ATOM    672  HD1 ILE P  44     -13.063  -9.731   8.856  0.00  0.00      PROA
ATOM    673  HD2 ILE P  44     -14.732  -9.240   8.335  0.00  0.00      PROA
ATOM    674  HD3 ILE P  44     -13.229  -8.455   7.617  0.00  0.00      PROA
ATOM    675  C   ILE P  44     -10.915 -12.645   5.060  0.00  0.00      PROA
ATOM    676  O   ILE P  44     -10.708 -12.568   3.884  0.00  0.00      PROA
ATOM    677  N   TYR P  45      -9.879 -13.057   5.810  0.00  0.00      PROA
ATOM    678  HN  TYR P  45     -10.074 -12.914   6.777  0.00  0.00      PROA
ATOM    679  CA  TYR P  45      -8.573 -13.604   5.475  0.00  0.00      PROA
ATOM    680  HA  TYR P  45      -8.042 -12.968   4.782  0.00  0.00      PROA
ATOM    681  CB  TYR P  45      -7.661 -13.886   6.664  0.00  0.00      PROA
ATOM    682  HB1 TYR P  45      -6.764 -14.386   6.241  0.00  0.00      PROA
ATOM    683  HB2 TYR P  45      -8.213 -14.505   7.403  0.00  0.00      PROA
ATOM    684  CG  TYR P  45      -7.211 -12.657   7.302  0.00  0.00      PROA
ATOM    685  CD1 TYR P  45      -6.378 -12.847   8.499  0.00  0.00      PROA
ATOM    686  HD1 TYR P  45      -6.063 -13.832   8.810  0.00  0.00      PROA
ATOM    687  CE1 TYR P  45      -5.839 -11.698   9.107  0.00  0.00      PROA
ATOM    688  HE1 TYR P  45      -5.306 -11.751  10.044  0.00  0.00      PROA
ATOM    689  CZ  TYR P  45      -5.984 -10.471   8.512  0.00  0.00      PROA
ATOM    690  OH  TYR P  45      -5.447  -9.317   9.085  0.00  0.00      PROA
ATOM    691  HH  TYR P  45      -5.705  -8.553   8.563  0.00  0.00      PROA
ATOM    692  CD2 TYR P  45      -7.448 -11.358   6.827  0.00  0.00      PROA
ATOM    693  HD2 TYR P  45      -8.038 -11.231   5.931  0.00  0.00      PROA
ATOM    694  CE2 TYR P  45      -6.800 -10.305   7.400  0.00  0.00      PROA
ATOM    695  HE2 TYR P  45      -6.902  -9.339   6.928  0.00  0.00      PROA
ATOM    696  C   TYR P  45      -8.856 -14.969   4.655  0.00  0.00      PROA
ATOM    697  O   TYR P  45      -8.214 -15.165   3.658  0.00  0.00      PROA
ATOM    698  N   SER P  46      -9.835 -15.813   5.046  0.00  0.00      PROA
ATOM    699  HN  SER P  46     -10.138 -15.732   5.993  0.00  0.00      PROA
ATOM    700  CA  SER P  46     -10.175 -16.982   4.277  0.00  0.00      PROA
ATOM    701  HA  SER P  46      -9.233 -17.510   4.247  0.00  0.00      PROA
ATOM    702  CB  SER P  46     -11.264 -17.886   4.945  0.00  0.00      PROA
ATOM    703  HB1 SER P  46     -11.188 -18.753   4.254  0.00  0.00      PROA
ATOM    704  HB2 SER P  46     -12.277 -17.431   4.981  0.00  0.00      PROA
ATOM    705  OG  SER P  46     -10.857 -18.385   6.211  0.00  0.00      PROA
ATOM    706  HG1 SER P  46     -10.505 -17.632   6.692  0.00  0.00      PROA
ATOM    707  C   SER P  46     -10.536 -16.548   2.798  0.00  0.00      PROA
ATOM    708  O   SER P  46      -9.937 -16.995   1.829  0.00  0.00      PROA
ATOM    709  N   ILE P  47     -11.445 -15.560   2.598  0.00  0.00      PROA
ATOM    710  HN  ILE P  47     -12.031 -15.222   3.331  0.00  0.00      PROA
ATOM    711  CA  ILE P  47     -11.969 -15.213   1.329  0.00  0.00      PROA
ATOM    712  HA  ILE P  47     -12.059 -16.072   0.680  0.00  0.00      PROA
ATOM    713  CB  ILE P  47     -13.367 -14.587   1.431  0.00  0.00      PROA
ATOM    714  HB  ILE P  47     -13.234 -13.553   1.817  0.00  0.00      PROA
ATOM    715  CG2 ILE P  47     -14.033 -14.514   0.021  0.00  0.00      PROA
ATOM    716 HG21 ILE P  47     -14.013 -15.479  -0.530  0.00  0.00      PROA
ATOM    717 HG22 ILE P  47     -13.544 -13.757  -0.629  0.00  0.00      PROA
ATOM    718 HG23 ILE P  47     -15.097 -14.197   0.047  0.00  0.00      PROA
ATOM    719  CG1 ILE P  47     -14.329 -15.367   2.392  0.00  0.00      PROA
ATOM    720 HG11 ILE P  47     -15.331 -15.052   2.031  0.00  0.00      PROA
ATOM    721 HG12 ILE P  47     -14.294 -14.947   3.420  0.00  0.00      PROA
ATOM    722  CD  ILE P  47     -14.410 -16.843   2.369  0.00  0.00      PROA
ATOM    723  HD1 ILE P  47     -13.523 -17.379   2.769  0.00  0.00      PROA
ATOM    724  HD2 ILE P  47     -14.559 -17.312   1.373  0.00  0.00      PROA
ATOM    725  HD3 ILE P  47     -15.232 -17.175   3.038  0.00  0.00      PROA
ATOM    726  C   ILE P  47     -11.026 -14.256   0.549  0.00  0.00      PROA
ATOM    727  O   ILE P  47     -10.973 -14.348  -0.693  0.00  0.00      PROA
```

```
ATOM    728  N   ILE P  48     -10.103 -13.484   1.230  0.00  0.00      PROA
ATOM    729  HN  ILE P  48     -10.257 -13.613   2.206  0.00  0.00      PROA
ATOM    730  CA  ILE P  48      -8.962 -12.742   0.681  0.00  0.00      PROA
ATOM    731  HA  ILE P  48      -9.335 -12.093  -0.097  0.00  0.00      PROA
ATOM    732  CB  ILE P  48      -8.322 -11.740   1.689  0.00  0.00      PROA
ATOM    733  HB  ILE P  48      -8.367 -12.318   2.637  0.00  0.00      PROA
ATOM    734  CG2 ILE P  48      -6.850 -11.460   1.252  0.00  0.00      PROA
ATOM    735 HG21 ILE P  48      -6.191 -12.347   1.367  0.00  0.00      PROA
ATOM    736 HG22 ILE P  48      -6.405 -10.816   2.041  0.00  0.00      PROA
ATOM    737 HG23 ILE P  48      -6.704 -11.072   0.222  0.00  0.00      PROA
ATOM    738  CG1 ILE P  48      -9.109 -10.463   1.879  0.00  0.00      PROA
ATOM    739 HG11 ILE P  48     -10.140 -10.747   2.179  0.00  0.00      PROA
ATOM    740 HG12 ILE P  48      -9.252  -9.835   0.973  0.00  0.00      PROA
ATOM    741  CD  ILE P  48      -8.558  -9.631   2.975  0.00  0.00      PROA
ATOM    742  HD1 ILE P  48      -7.656  -9.024   2.750  0.00  0.00      PROA
ATOM    743  HD2 ILE P  48      -8.205 -10.216   3.851  0.00  0.00      PROA
ATOM    744  HD3 ILE P  48      -9.345  -8.903   3.270  0.00  0.00      PROA
ATOM    745  C   ILE P  48      -8.036 -13.656   0.044  0.00  0.00      PROA
ATOM    746  O   ILE P  48      -7.549 -13.391  -1.073  0.00  0.00      PROA
ATOM    747  N   PHE P  49      -7.824 -14.877   0.671  0.00  0.00      PROA
ATOM    748  HN  PHE P  49      -8.152 -14.903   1.612  0.00  0.00      PROA
ATOM    749  CA  PHE P  49      -7.087 -15.980   0.099  0.00  0.00      PROA
ATOM    750  HA  PHE P  49      -6.153 -15.582  -0.268  0.00  0.00      PROA
ATOM    751  CB  PHE P  49      -6.779 -17.161   1.150  0.00  0.00      PROA
ATOM    752  HB1 PHE P  49      -7.643 -17.814   1.397  0.00  0.00      PROA
ATOM    753  HB2 PHE P  49      -6.501 -16.762   2.149  0.00  0.00      PROA
ATOM    754  CG  PHE P  49      -5.596 -17.991   0.695  0.00  0.00      PROA
ATOM    755  CD1 PHE P  49      -5.865 -19.145  -0.172  0.00  0.00      PROA
ATOM    756  HD1 PHE P  49      -6.872 -19.351  -0.504  0.00  0.00      PROA
ATOM    757  CE1 PHE P  49      -4.815 -19.963  -0.458  0.00  0.00      PROA
ATOM    758  HE1 PHE P  49      -5.115 -20.854  -0.988  0.00  0.00      PROA
ATOM    759  CZ  PHE P  49      -3.477 -19.783   0.085  0.00  0.00      PROA
ATOM    760  HZ  PHE P  49      -2.681 -20.339  -0.387  0.00  0.00      PROA
ATOM    761  CD2 PHE P  49      -4.384 -17.931   1.360  0.00  0.00      PROA
ATOM    762  HD2 PHE P  49      -4.270 -17.212   2.157  0.00  0.00      PROA
ATOM    763  CE2 PHE P  49      -3.318 -18.747   1.078  0.00  0.00      PROA
ATOM    764  HE2 PHE P  49      -2.426 -18.800   1.685  0.00  0.00      PROA
ATOM    765  C   PHE P  49      -7.728 -16.572  -1.159  0.00  0.00      PROA
ATOM    766  O   PHE P  49      -7.053 -16.956  -2.125  0.00  0.00      PROA
ATOM    767  N   LEU P  50      -9.025 -16.778  -1.137  0.00  0.00      PROA
ATOM    768  HN  LEU P  50      -9.415 -16.507  -0.260  0.00  0.00      PROA
ATOM    769  CA  LEU P  50      -9.837 -17.334  -2.247  0.00  0.00      PROA
ATOM    770  HA  LEU P  50      -9.274 -18.219  -2.502  0.00  0.00      PROA
ATOM    771  CB  LEU P  50     -11.282 -17.581  -1.738  0.00  0.00      PROA
ATOM    772  HB1 LEU P  50     -11.819 -16.693  -1.341  0.00  0.00      PROA
ATOM    773  HB2 LEU P  50     -11.184 -18.274  -0.875  0.00  0.00      PROA
ATOM    774  CG  LEU P  50     -12.273 -18.167  -2.744  0.00  0.00      PROA
ATOM    775  HG  LEU P  50     -12.679 -17.347  -3.374  0.00  0.00      PROA
ATOM    776  CD1 LEU P  50     -11.773 -19.158  -3.770  0.00  0.00      PROA
ATOM    777 HD11 LEU P  50     -10.801 -18.833  -4.199  0.00  0.00      PROA
ATOM    778 HD12 LEU P  50     -12.423 -19.367  -4.646  0.00  0.00      PROA
ATOM    779 HD13 LEU P  50     -11.636 -20.140  -3.270  0.00  0.00      PROA
ATOM    780  CD2 LEU P  50     -13.450 -18.712  -1.958  0.00  0.00      PROA
ATOM    781 HD21 LEU P  50     -14.234 -19.109  -2.639  0.00  0.00      PROA
ATOM    782 HD22 LEU P  50     -13.797 -17.826  -1.384  0.00  0.00      PROA
ATOM    783 HD23 LEU P  50     -12.968 -19.512  -1.355  0.00  0.00      PROA
ATOM    784  C   LEU P  50      -9.780 -16.432  -3.472  0.00  0.00      PROA
ATOM    785  O   LEU P  50      -9.455 -16.856  -4.539  0.00  0.00      PROA
ATOM    786  N   THR P  51     -10.066 -15.137  -3.187  0.00  0.00      PROA
ATOM    787  HN  THR P  51     -10.438 -14.878  -2.298  0.00  0.00      PROA
ATOM    788  CA  THR P  51      -9.981 -14.078  -4.149  0.00  0.00      PROA
```

```
ATOM    789  HA  THR P  51     -10.642 -14.392  -4.943  0.00  0.00      PROA
ATOM    790  CB  THR P  51     -10.481 -12.699  -3.702  0.00  0.00      PROA
ATOM    791  HB  THR P  51      -9.692 -12.180  -3.117  0.00  0.00      PROA
ATOM    792  OG1 THR P  51     -11.679 -12.763  -2.947  0.00  0.00      PROA
ATOM    793  HG1 THR P  51     -11.416 -13.020  -2.060  0.00  0.00      PROA
ATOM    794  CG2 THR P  51     -10.878 -11.785  -4.940  0.00  0.00      PROA
ATOM    795 HG21 THR P  51     -11.107 -10.716  -4.744  0.00  0.00      PROA
ATOM    796 HG22 THR P  51     -11.750 -12.210  -5.482  0.00  0.00      PROA
ATOM    797 HG23 THR P  51      -9.990 -11.860  -5.603  0.00  0.00      PROA
ATOM    798  C   THR P  51      -8.550 -13.920  -4.666  0.00  0.00      PROA
ATOM    799  O   THR P  51      -8.345 -13.820  -5.875  0.00  0.00      PROA
ATOM    800  N   GLY P  52      -7.547 -13.935  -3.791  0.00  0.00      PROA
ATOM    801  HN  GLY P  52      -7.799 -14.044  -2.833  0.00  0.00      PROA
ATOM    802  CA  GLY P  52      -6.118 -13.930  -4.159  0.00  0.00      PROA
ATOM    803  HA1 GLY P  52      -5.610 -14.188  -3.241  0.00  0.00      PROA
ATOM    804  HA2 GLY P  52      -5.920 -12.934  -4.528  0.00  0.00      PROA
ATOM    805  C   GLY P  52      -5.683 -14.889  -5.202  0.00  0.00      PROA
ATOM    806  O   GLY P  52      -5.098 -14.515  -6.206  0.00  0.00      PROA
ATOM    807  N   ILE P  53      -5.984 -16.207  -4.995  0.00  0.00      PROA
ATOM    808  HN  ILE P  53      -6.554 -16.386  -4.197  0.00  0.00      PROA
ATOM    809  CA  ILE P  53      -5.573 -17.281  -5.908  0.00  0.00      PROA
ATOM    810  HA  ILE P  53      -4.547 -17.122  -6.206  0.00  0.00      PROA
ATOM    811  CB  ILE P  53      -5.706 -18.534  -5.124  0.00  0.00      PROA
ATOM    812  HB  ILE P  53      -6.735 -18.532  -4.703  0.00  0.00      PROA
ATOM    813  CG2 ILE P  53      -5.516 -19.786  -5.973  0.00  0.00      PROA
ATOM    814 HG21 ILE P  53      -4.700 -19.773  -6.727  0.00  0.00      PROA
ATOM    815 HG22 ILE P  53      -6.418 -19.930  -6.604  0.00  0.00      PROA
ATOM    816 HG23 ILE P  53      -5.552 -20.717  -5.367  0.00  0.00      PROA
ATOM    817  CG1 ILE P  53      -4.843 -18.649  -3.836  0.00  0.00      PROA
ATOM    818 HG11 ILE P  53      -5.228 -19.380  -3.093  0.00  0.00      PROA
ATOM    819 HG12 ILE P  53      -5.085 -17.663  -3.385  0.00  0.00      PROA
ATOM    820  CD  ILE P  53      -3.365 -18.820  -4.200  0.00  0.00      PROA
ATOM    821  HD1 ILE P  53      -3.027 -18.393  -5.168  0.00  0.00      PROA
ATOM    822  HD2 ILE P  53      -3.367 -19.921  -4.345  0.00  0.00      PROA
ATOM    823  HD3 ILE P  53      -2.716 -18.392  -3.405  0.00  0.00      PROA
ATOM    824  C   ILE P  53      -6.339 -17.362  -7.326  0.00  0.00      PROA
ATOM    825  O   ILE P  53      -5.804 -17.354  -8.396  0.00  0.00      PROA
ATOM    826  N   VAL P  54      -7.697 -17.362  -7.270  0.00  0.00      PROA
ATOM    827  HN  VAL P  54      -8.194 -17.456  -6.411  0.00  0.00      PROA
ATOM    828  CA  VAL P  54      -8.601 -17.432  -8.445  0.00  0.00      PROA
ATOM    829  HA  VAL P  54      -8.305 -18.298  -9.020  0.00  0.00      PROA
ATOM    830  CB  VAL P  54     -10.047 -17.739  -7.962  0.00  0.00      PROA
ATOM    831  HB  VAL P  54     -10.353 -16.940  -7.253  0.00  0.00      PROA
ATOM    832  CG1 VAL P  54     -11.029 -17.667  -9.137  0.00  0.00      PROA
ATOM    833 HG11 VAL P  54     -10.620 -18.379  -9.886  0.00  0.00      PROA
ATOM    834 HG12 VAL P  54     -11.064 -16.609  -9.474  0.00  0.00      PROA
ATOM    835 HG13 VAL P  54     -12.062 -17.898  -8.797  0.00  0.00      PROA
ATOM    836  CG2 VAL P  54     -10.144 -19.110  -7.329  0.00  0.00      PROA
ATOM    837 HG21 VAL P  54     -11.193 -19.462  -7.230  0.00  0.00      PROA
ATOM    838 HG22 VAL P  54      -9.768 -19.034  -6.286  0.00  0.00      PROA
ATOM    839 HG23 VAL P  54      -9.684 -19.949  -7.894  0.00  0.00      PROA
ATOM    840  C   VAL P  54      -8.506 -16.195  -9.326  0.00  0.00      PROA
ATOM    841  O   VAL P  54      -8.403 -16.322 -10.603  0.00  0.00      PROA
ATOM    842  N   GLY P  55      -8.527 -15.094  -8.675  0.00  0.00      PROA
ATOM    843  HN  GLY P  55      -8.669 -15.028  -7.690  0.00  0.00      PROA
ATOM    844  CA  GLY P  55      -8.491 -13.849  -9.363  0.00  0.00      PROA
ATOM    845  HA1 GLY P  55      -8.639 -13.007  -8.704  0.00  0.00      PROA
ATOM    846  HA2 GLY P  55      -9.203 -13.843 -10.175  0.00  0.00      PROA
ATOM    847  C   GLY P  55      -7.206 -13.419  -9.924  0.00  0.00      PROA
ATOM    848  O   GLY P  55      -7.118 -13.098 -11.104  0.00  0.00      PROA
ATOM    849  N   ASN P  56      -6.126 -13.439  -9.161  0.00  0.00      PROA
```

```
ATOM    850  HN  ASN P  56      -6.228 -13.625  -8.187  0.00  0.00      PROA
ATOM    851  CA  ASN P  56      -4.771 -13.244  -9.795  0.00  0.00      PROA
ATOM    852  HA  ASN P  56      -4.691 -12.310 -10.332  0.00  0.00      PROA
ATOM    853  CB  ASN P  56      -3.633 -13.009  -8.739  0.00  0.00      PROA
ATOM    854  HB1 ASN P  56      -2.698 -12.971  -9.337  0.00  0.00      PROA
ATOM    855  HB2 ASN P  56      -3.659 -13.917  -8.099  0.00  0.00      PROA
ATOM    856  CG  ASN P  56      -3.776 -11.655  -8.105  0.00  0.00      PROA
ATOM    857  OD1 ASN P  56      -3.650 -10.618  -8.739  0.00  0.00      PROA
ATOM    858  ND2 ASN P  56      -3.962 -11.795  -6.732  0.00  0.00      PROA
ATOM    859 HD21 ASN P  56      -3.969 -11.068  -6.045  0.00  0.00      PROA
ATOM    860 HD22 ASN P  56      -4.116 -12.736  -6.430  0.00  0.00      PROA
ATOM    861  C   ASN P  56      -4.286 -14.333 -10.817  0.00  0.00      PROA
ATOM    862  O   ASN P  56      -3.683 -14.091 -11.838  0.00  0.00      PROA
ATOM    863  N   GLY P  57      -4.651 -15.597 -10.438  0.00  0.00      PROA
ATOM    864  HN  GLY P  57      -5.097 -15.756  -9.561  0.00  0.00      PROA
ATOM    865  CA  GLY P  57      -4.533 -16.646 -11.483  0.00  0.00      PROA
ATOM    866  HA1 GLY P  57      -5.174 -17.401 -11.053  0.00  0.00      PROA
ATOM    867  HA2 GLY P  57      -3.494 -16.856 -11.689  0.00  0.00      PROA
ATOM    868  C   GLY P  57      -5.169 -16.386 -12.855  0.00  0.00      PROA
ATOM    869  O   GLY P  57      -4.529 -16.346 -13.891  0.00  0.00      PROA
ATOM    870  N   LEU P  58      -6.471 -16.108 -12.896  0.00  0.00      PROA
ATOM    871  HN  LEU P  58      -6.975 -16.146 -12.037  0.00  0.00      PROA
ATOM    872  CA  LEU P  58      -7.170 -15.652 -14.086  0.00  0.00      PROA
ATOM    873  HA  LEU P  58      -7.131 -16.485 -14.772  0.00  0.00      PROA
ATOM    874  CB  LEU P  58      -8.685 -15.382 -13.832  0.00  0.00      PROA
ATOM    875  HB1 LEU P  58      -8.688 -14.598 -13.045  0.00  0.00      PROA
ATOM    876  HB2 LEU P  58      -8.930 -16.367 -13.380  0.00  0.00      PROA
ATOM    877  CG  LEU P  58      -9.547 -14.865 -15.029  0.00  0.00      PROA
ATOM    878  HG  LEU P  58      -9.049 -13.906 -15.289  0.00  0.00      PROA
ATOM    879  CD1 LEU P  58      -9.721 -15.913 -16.191  0.00  0.00      PROA
ATOM    880 HD11 LEU P  58      -8.697 -16.122 -16.568  0.00  0.00      PROA
ATOM    881 HD12 LEU P  58     -10.265 -15.504 -17.069  0.00  0.00      PROA
ATOM    882 HD13 LEU P  58     -10.231 -16.847 -15.870  0.00  0.00      PROA
ATOM    883  CD2 LEU P  58     -10.900 -14.344 -14.561  0.00  0.00      PROA
ATOM    884 HD21 LEU P  58     -10.786 -13.718 -13.650  0.00  0.00      PROA
ATOM    885 HD22 LEU P  58     -11.604 -15.169 -14.319  0.00  0.00      PROA
ATOM    886 HD23 LEU P  58     -11.458 -13.691 -15.265  0.00  0.00      PROA
ATOM    887  C   LEU P  58      -6.524 -14.473 -14.868  0.00  0.00      PROA
ATOM    888  O   LEU P  58      -6.293 -14.544 -16.087  0.00  0.00      PROA
ATOM    889  N   VAL P  59      -6.120 -13.384 -14.148  0.00  0.00      PROA
ATOM    890  HN  VAL P  59      -6.287 -13.204 -13.182  0.00  0.00      PROA
ATOM    891  CA  VAL P  59      -5.410 -12.203 -14.743  0.00  0.00      PROA
ATOM    892  HA  VAL P  59      -6.020 -11.864 -15.567  0.00  0.00      PROA
ATOM    893  CB  VAL P  59      -5.266 -11.037 -13.764  0.00  0.00      PROA
ATOM    894  HB  VAL P  59      -4.744 -11.288 -12.816  0.00  0.00      PROA
ATOM    895  CG1 VAL P  59      -4.572  -9.755 -14.407  0.00  0.00      PROA
ATOM    896 HG11 VAL P  59      -5.102  -9.332 -15.287  0.00  0.00      PROA
ATOM    897 HG12 VAL P  59      -3.572 -10.110 -14.736  0.00  0.00      PROA
ATOM    898 HG13 VAL P  59      -4.402  -8.989 -13.621  0.00  0.00      PROA
ATOM    899  CG2 VAL P  59      -6.627 -10.568 -13.315  0.00  0.00      PROA
ATOM    900 HG21 VAL P  59      -7.429 -11.296 -13.068  0.00  0.00      PROA
ATOM    901 HG22 VAL P  59      -6.946  -9.888 -14.134  0.00  0.00      PROA
ATOM    902 HG23 VAL P  59      -6.523  -9.919 -12.420  0.00  0.00      PROA
ATOM    903  C   VAL P  59      -3.995 -12.694 -15.392  0.00  0.00      PROA
ATOM    904  O   VAL P  59      -3.633 -12.329 -16.504  0.00  0.00      PROA
ATOM    905  N   ILE P  60      -3.209 -13.568 -14.720  0.00  0.00      PROA
ATOM    906  HN  ILE P  60      -3.344 -13.778 -13.755  0.00  0.00      PROA
ATOM    907  CA  ILE P  60      -1.917 -14.032 -15.261  0.00  0.00      PROA
ATOM    908  HA  ILE P  60      -1.353 -13.245 -15.739  0.00  0.00      PROA
ATOM    909  CB  ILE P  60      -1.077 -14.760 -14.206  0.00  0.00      PROA
ATOM    910  HB  ILE P  60      -1.783 -15.496 -13.765  0.00  0.00      PROA
```

```
ATOM    911  CG2 ILE P  60       0.081 -15.465 -14.898  0.00  0.00      PROA
ATOM    912 HG21 ILE P  60       0.743 -14.738 -15.417  0.00  0.00      PROA
ATOM    913 HG22 ILE P  60      -0.247 -16.282 -15.576  0.00  0.00      PROA
ATOM    914 HG23 ILE P  60       0.693 -15.989 -14.134  0.00  0.00      PROA
ATOM    915  CG1 ILE P  60      -0.696 -13.819 -13.003  0.00  0.00      PROA
ATOM    916 HG11 ILE P  60      -1.530 -13.200 -12.608  0.00  0.00      PROA
ATOM    917 HG12 ILE P  60      -0.067 -12.998 -13.410  0.00  0.00      PROA
ATOM    918  CD  ILE P  60       0.011 -14.485 -11.806  0.00  0.00      PROA
ATOM    919  HD1 ILE P  60       0.760 -15.223 -12.164  0.00  0.00      PROA
ATOM    920  HD2 ILE P  60      -0.683 -14.915 -11.052  0.00  0.00      PROA
ATOM    921  HD3 ILE P  60       0.573 -13.644 -11.347  0.00  0.00      PROA
ATOM    922  C   ILE P  60      -2.174 -14.932 -16.552  0.00  0.00      PROA
ATOM    923  O   ILE P  60      -1.439 -14.729 -17.525  0.00  0.00      PROA
ATOM    924  N   LEU P  61      -3.286 -15.833 -16.573  0.00  0.00      PROA
ATOM    925  HN  LEU P  61      -3.871 -15.914 -15.769  0.00  0.00      PROA
ATOM    926  CA  LEU P  61      -3.673 -16.560 -17.764  0.00  0.00      PROA
ATOM    927  HA  LEU P  61      -2.804 -17.091 -18.126  0.00  0.00      PROA
ATOM    928  CB  LEU P  61      -4.823 -17.573 -17.368  0.00  0.00      PROA
ATOM    929  HB1 LEU P  61      -5.588 -16.903 -16.921  0.00  0.00      PROA
ATOM    930  HB2 LEU P  61      -4.305 -18.168 -16.586  0.00  0.00      PROA
ATOM    931  CG  LEU P  61      -5.407 -18.512 -18.440  0.00  0.00      PROA
ATOM    932  HG  LEU P  61      -5.820 -17.971 -19.319  0.00  0.00      PROA
ATOM    933  CD1 LEU P  61      -4.311 -19.307 -19.019  0.00  0.00      PROA
ATOM    934 HD11 LEU P  61      -3.734 -18.830 -19.840  0.00  0.00      PROA
ATOM    935 HD12 LEU P  61      -4.724 -20.171 -19.581  0.00  0.00      PROA
ATOM    936 HD13 LEU P  61      -3.678 -19.728 -18.208  0.00  0.00      PROA
ATOM    937  CD2 LEU P  61      -6.549 -19.390 -17.902  0.00  0.00      PROA
ATOM    938 HD21 LEU P  61      -6.781 -20.198 -18.629  0.00  0.00      PROA
ATOM    939 HD22 LEU P  61      -7.439 -18.781 -17.636  0.00  0.00      PROA
ATOM    940 HD23 LEU P  61      -6.191 -19.897 -16.980  0.00  0.00      PROA
ATOM    941  C   LEU P  61      -4.059 -15.679 -18.932  0.00  0.00      PROA
ATOM    942  O   LEU P  61      -3.642 -15.822 -20.126  0.00  0.00      PROA
ATOM    943  N   VAL P  62      -4.903 -14.616 -18.703  0.00  0.00      PROA
ATOM    944  HN  VAL P  62      -5.318 -14.480 -17.806  0.00  0.00      PROA
ATOM    945  CA  VAL P  62      -5.250 -13.624 -19.740  0.00  0.00      PROA
ATOM    946  HA  VAL P  62      -5.550 -14.227 -20.585  0.00  0.00      PROA
ATOM    947  CB  VAL P  62      -6.498 -12.819 -19.248  0.00  0.00      PROA
ATOM    948  HB  VAL P  62      -6.406 -12.416 -18.217  0.00  0.00      PROA
ATOM    949  CG1 VAL P  62      -6.774 -11.642 -20.211  0.00  0.00      PROA
ATOM    950 HG11 VAL P  62      -6.719 -12.000 -21.261  0.00  0.00      PROA
ATOM    951 HG12 VAL P  62      -6.052 -10.805 -20.110  0.00  0.00      PROA
ATOM    952 HG13 VAL P  62      -7.827 -11.340 -20.026  0.00  0.00      PROA
ATOM    953  CG2 VAL P  62      -7.676 -13.820 -19.253  0.00  0.00      PROA
ATOM    954 HG21 VAL P  62      -7.884 -14.362 -20.201  0.00  0.00      PROA
ATOM    955 HG22 VAL P  62      -8.627 -13.379 -18.885  0.00  0.00      PROA
ATOM    956 HG23 VAL P  62      -7.339 -14.601 -18.539  0.00  0.00      PROA
ATOM    957  C   VAL P  62      -4.028 -12.800 -20.251  0.00  0.00      PROA
ATOM    958  O   VAL P  62      -3.887 -12.612 -21.462  0.00  0.00      PROA
ATOM    959  N   MET P  63      -3.156 -12.319 -19.324  0.00  0.00      PROA
ATOM    960  HN  MET P  63      -3.313 -12.640 -18.393  0.00  0.00      PROA
ATOM    961  CA  MET P  63      -2.210 -11.281 -19.592  0.00  0.00      PROA
ATOM    962  HA  MET P  63      -2.531 -10.809 -20.509  0.00  0.00      PROA
ATOM    963  CB  MET P  63      -2.114 -10.295 -18.429  0.00  0.00      PROA
ATOM    964  HB1 MET P  63      -1.318  -9.529 -18.542  0.00  0.00      PROA
ATOM    965  HB2 MET P  63      -1.795 -10.798 -17.491  0.00  0.00      PROA
ATOM    966  CG  MET P  63      -3.467  -9.545 -18.090  0.00  0.00      PROA
ATOM    967  HG1 MET P  63      -3.353  -8.932 -17.171  0.00  0.00      PROA
ATOM    968  HG2 MET P  63      -4.175 -10.332 -17.754  0.00  0.00      PROA
ATOM    969  SD  MET P  63      -4.139  -8.506 -19.380  0.00  0.00      PROA
ATOM    970  CE  MET P  63      -2.955  -7.141 -19.091  0.00  0.00      PROA
ATOM    971  HE1 MET P  63      -3.176  -6.428 -18.269  0.00  0.00      PROA
```

ATOM 972 HE2 MET P 63 -2.880 -6.513 -20.005 0.00 0.00 PROA
ATOM 973 HE3 MET P 63 -2.004 -7.690 -18.919 0.00 0.00 PROA
ATOM 974 C MET P 63 -0.816 -11.788 -20.004 0.00 0.00 PROA
ATOM 975 O MET P 63 -0.037 -11.088 -20.693 0.00 0.00 PROA
ATOM 976 N GLY P 64 -0.431 -12.950 -19.543 0.00 0.00 PROA
ATOM 977 HN GLY P 64 -1.112 -13.453 -19.016 0.00 0.00 PROA
ATOM 978 CA GLY P 64 0.744 -13.685 -19.832 0.00 0.00 PROA
ATOM 979 HA1 GLY P 64 0.849 -14.400 -19.029 0.00 0.00 PROA
ATOM 980 HA2 GLY P 64 1.537 -12.977 -20.026 0.00 0.00 PROA
ATOM 981 C GLY P 64 0.655 -14.637 -21.076 0.00 0.00 PROA
ATOM 982 O GLY P 64 1.622 -14.847 -21.791 0.00 0.00 PROA
ATOM 983 N TYR P 65 -0.526 -15.215 -21.335 0.00 0.00 PROA
ATOM 984 HN TYR P 65 -1.286 -14.950 -20.747 0.00 0.00 PROA
ATOM 985 CA TYR P 65 -0.801 -16.158 -22.412 0.00 0.00 PROA
ATOM 986 HA TYR P 65 0.070 -16.314 -23.032 0.00 0.00 PROA
ATOM 987 CB TYR P 65 -1.214 -17.544 -21.799 0.00 0.00 PROA
ATOM 988 HB1 TYR P 65 -1.552 -18.267 -22.572 0.00 0.00 PROA
ATOM 989 HB2 TYR P 65 -2.092 -17.370 -21.141 0.00 0.00 PROA
ATOM 990 CG TYR P 65 -0.090 -18.181 -21.128 0.00 0.00 PROA
ATOM 991 CD1 TYR P 65 -0.096 -18.262 -19.742 0.00 0.00 PROA
ATOM 992 HD1 TYR P 65 -0.857 -17.691 -19.231 0.00 0.00 PROA
ATOM 993 CE1 TYR P 65 0.777 -19.103 -19.002 0.00 0.00 PROA
ATOM 994 HE1 TYR P 65 0.801 -19.000 -17.927 0.00 0.00 PROA
ATOM 995 CZ TYR P 65 1.718 -19.886 -19.742 0.00 0.00 PROA
ATOM 996 OH TYR P 65 2.462 -20.811 -19.099 0.00 0.00 PROA
ATOM 997 HH TYR P 65 3.099 -21.137 -19.740 0.00 0.00 PROA
ATOM 998 CD2 TYR P 65 0.845 -18.945 -21.859 0.00 0.00 PROA
ATOM 999 HD2 TYR P 65 0.874 -18.771 -22.925 0.00 0.00 PROA
ATOM 1000 CE2 TYR P 65 1.764 -19.837 -21.173 0.00 0.00 PROA
ATOM 1001 HE2 TYR P 65 2.428 -20.542 -21.653 0.00 0.00 PROA
ATOM 1002 C TYR P 65 -1.732 -15.651 -23.419 0.00 0.00 PROA
ATOM 1003 O TYR P 65 -1.302 -15.478 -24.549 0.00 0.00 PROA
ATOM 1004 N GLN P 66 -3.055 -15.478 -23.076 0.00 0.00 PROA
ATOM 1005 HN GLN P 66 -3.394 -15.793 -22.192 0.00 0.00 PROA
ATOM 1006 CA GLN P 66 -4.118 -15.168 -24.061 0.00 0.00 PROA
ATOM 1007 HA GLN P 66 -4.095 -15.965 -24.789 0.00 0.00 PROA
ATOM 1008 CB GLN P 66 -5.519 -15.178 -23.362 0.00 0.00 PROA
ATOM 1009 HB1 GLN P 66 -6.314 -14.887 -24.082 0.00 0.00 PROA
ATOM 1010 HB2 GLN P 66 -5.400 -14.356 -22.625 0.00 0.00 PROA
ATOM 1011 CG GLN P 66 -5.952 -16.547 -22.727 0.00 0.00 PROA
ATOM 1012 HG1 GLN P 66 -6.705 -16.279 -21.956 0.00 0.00 PROA
ATOM 1013 HG2 GLN P 66 -5.097 -17.096 -22.278 0.00 0.00 PROA
ATOM 1014 CD GLN P 66 -6.622 -17.476 -23.817 0.00 0.00 PROA
ATOM 1015 OE1 GLN P 66 -7.636 -17.198 -24.501 0.00 0.00 PROA
ATOM 1016 NE2 GLN P 66 -5.882 -18.641 -24.013 0.00 0.00 PROA
ATOM 1017 HE21 GLN P 66 -6.362 -19.339 -24.545 0.00 0.00 PROA
ATOM 1018 HE22 GLN P 66 -5.052 -18.831 -23.489 0.00 0.00 PROA
ATOM 1019 C GLN P 66 -4.024 -13.876 -24.920 0.00 0.00 PROA
ATOM 1020 O GLN P 66 -4.136 -13.907 -26.144 0.00 0.00 PROA
ATOM 1021 N LYS P 67 -3.632 -12.757 -24.299 0.00 0.00 PROA
ATOM 1022 HN LYS P 67 -3.548 -12.700 -23.307 0.00 0.00 PROA
ATOM 1023 CA LYS P 67 -3.144 -11.700 -25.171 0.00 0.00 PROA
ATOM 1024 HA LYS P 67 -3.812 -11.496 -25.995 0.00 0.00 PROA
ATOM 1025 CB LYS P 67 -3.040 -10.338 -24.420 0.00 0.00 PROA
ATOM 1026 HB1 LYS P 67 -2.377 -10.378 -23.530 0.00 0.00 PROA
ATOM 1027 HB2 LYS P 67 -4.077 -10.202 -24.044 0.00 0.00 PROA
ATOM 1028 CG LYS P 67 -2.640 -9.209 -25.360 0.00 0.00 PROA
ATOM 1029 HG1 LYS P 67 -3.263 -9.054 -26.266 0.00 0.00 PROA
ATOM 1030 HG2 LYS P 67 -1.571 -9.388 -25.603 0.00 0.00 PROA
ATOM 1031 CD LYS P 67 -2.764 -7.818 -24.516 0.00 0.00 PROA
ATOM 1032 HD1 LYS P 67 -2.071 -7.920 -23.654 0.00 0.00 PROA

```
ATOM   1033  HD2 LYS P  67      -3.791  -7.675 -24.119  0.00  0.00      PROA
ATOM   1034  CE  LYS P  67      -2.472  -6.606 -25.427  0.00  0.00      PROA
ATOM   1035  HE1 LYS P  67      -3.325  -6.591 -26.139  0.00  0.00      PROA
ATOM   1036  HE2 LYS P  67      -1.490  -6.642 -25.945  0.00  0.00      PROA
ATOM   1037  NZ  LYS P  67      -2.591  -5.378 -24.646  0.00  0.00      PROA
ATOM   1038  HZ1 LYS P  67      -1.811  -5.327 -23.961  0.00  0.00      PROA
ATOM   1039  HZ2 LYS P  67      -3.449  -5.418 -24.060  0.00  0.00      PROA
ATOM   1040  HZ3 LYS P  67      -2.665  -4.547 -25.267  0.00  0.00      PROA
ATOM   1041  C   LYS P  67      -1.843 -12.039 -25.836  0.00  0.00      PROA
ATOM   1042  O   LYS P  67      -0.951 -12.520 -25.095  0.00  0.00      PROA
ATOM   1043  N   LYS P  68      -1.786 -11.817 -27.143  0.00  0.00      PROA
ATOM   1044  HN  LYS P  68      -2.602 -11.620 -27.680  0.00  0.00      PROA
ATOM   1045  CA  LYS P  68      -0.608 -11.764 -27.955  0.00  0.00      PROA
ATOM   1046  HA  LYS P  68       0.197 -12.258 -27.431  0.00  0.00      PROA
ATOM   1047  CB  LYS P  68      -0.815 -12.480 -29.364  0.00  0.00      PROA
ATOM   1048  HB1 LYS P  68       0.069 -12.408 -30.033  0.00  0.00      PROA
ATOM   1049  HB2 LYS P  68      -1.606 -11.999 -29.979  0.00  0.00      PROA
ATOM   1050  CG  LYS P  68      -1.137 -14.008 -29.252  0.00  0.00      PROA
ATOM   1051  HG1 LYS P  68      -1.230 -14.364 -30.300  0.00  0.00      PROA
ATOM   1052  HG2 LYS P  68      -2.130 -14.065 -28.756  0.00  0.00      PROA
ATOM   1053  CD  LYS P  68      -0.064 -14.735 -28.370  0.00  0.00      PROA
ATOM   1054  HD1 LYS P  68       0.159 -14.349 -27.352  0.00  0.00      PROA
ATOM   1055  HD2 LYS P  68       0.812 -14.672 -29.051  0.00  0.00      PROA
ATOM   1056  CE  LYS P  68      -0.258 -16.252 -28.265  0.00  0.00      PROA
ATOM   1057  HE1 LYS P  68       0.591 -16.753 -27.753  0.00  0.00      PROA
ATOM   1058  HE2 LYS P  68      -0.518 -16.678 -29.258  0.00  0.00      PROA
ATOM   1059  NZ  LYS P  68      -1.398 -16.526 -27.359  0.00  0.00      PROA
ATOM   1060  HZ1 LYS P  68      -2.330 -16.269 -27.741  0.00  0.00      PROA
ATOM   1061  HZ2 LYS P  68      -1.279 -16.001 -26.469  0.00  0.00      PROA
ATOM   1062  HZ3 LYS P  68      -1.375 -17.536 -27.110  0.00  0.00      PROA
ATOM   1063  C   LYS P  68      -0.223 -10.298 -28.257  0.00  0.00      PROA
ATOM   1064  O   LYS P  68      -1.141  -9.453 -28.191  0.00  0.00      PROA
ATOM   1065  N   LEU P  69       1.048 -10.131 -28.565  0.00  0.00      PROA
ATOM   1066  HN  LEU P  69       1.512 -11.008 -28.663  0.00  0.00      PROA
ATOM   1067  CA  LEU P  69       1.823  -8.912 -28.691  0.00  0.00      PROA
ATOM   1068  HA  LEU P  69       2.798  -9.353 -28.835  0.00  0.00      PROA
ATOM   1069  CB  LEU P  69       1.380  -8.005 -29.892  0.00  0.00      PROA
ATOM   1070  HB1 LEU P  69       2.016  -7.095 -29.871  0.00  0.00      PROA
ATOM   1071  HB2 LEU P  69       0.310  -7.716 -29.808  0.00  0.00      PROA
ATOM   1072  CG  LEU P  69       1.665  -8.616 -31.344  0.00  0.00      PROA
ATOM   1073  HG  LEU P  69       1.041  -9.505 -31.576  0.00  0.00      PROA
ATOM   1074  CD1 LEU P  69       1.357  -7.585 -32.390  0.00  0.00      PROA
ATOM   1075 HD11 LEU P  69       0.288  -7.285 -32.414  0.00  0.00      PROA
ATOM   1076 HD12 LEU P  69       1.793  -7.987 -33.330  0.00  0.00      PROA
ATOM   1077 HD13 LEU P  69       1.892  -6.704 -31.976  0.00  0.00      PROA
ATOM   1078  CD2 LEU P  69       3.146  -9.125 -31.499  0.00  0.00      PROA
ATOM   1079 HD21 LEU P  69       3.762  -8.317 -31.050  0.00  0.00      PROA
ATOM   1080 HD22 LEU P  69       3.278  -9.383 -32.571  0.00  0.00      PROA
ATOM   1081 HD23 LEU P  69       3.408 -10.019 -30.894  0.00  0.00      PROA
ATOM   1082  C   LEU P  69       1.861  -8.015 -27.483  0.00  0.00      PROA
ATOM   1083  O   LEU P  69       1.823  -6.804 -27.460  0.00  0.00      PROA
ATOM   1084  N   ARG P  70       2.139  -8.680 -26.347  0.00  0.00      PROA
ATOM   1085  HN  ARG P  70       2.409  -9.639 -26.376  0.00  0.00      PROA
ATOM   1086  CA  ARG P  70       2.081  -8.065 -25.033  0.00  0.00      PROA
ATOM   1087  HA  ARG P  70       1.039  -7.802 -24.926  0.00  0.00      PROA
ATOM   1088  CB  ARG P  70       2.380  -9.116 -23.974  0.00  0.00      PROA
ATOM   1089  HB1 ARG P  70       2.454  -8.715 -22.941  0.00  0.00      PROA
ATOM   1090  HB2 ARG P  70       3.360  -9.576 -24.225  0.00  0.00      PROA
ATOM   1091  CG  ARG P  70       1.267 -10.227 -23.973  0.00  0.00      PROA
ATOM   1092  HG1 ARG P  70       1.014 -10.631 -24.976  0.00  0.00      PROA
ATOM   1093  HG2 ARG P  70       0.323  -9.811 -23.561  0.00  0.00      PROA
```

ATOM	1094	CD	ARG	P	70	1.603	-11.422	-23.106	0.00	0.00	PROA
ATOM	1095	HD1	ARG	P	70	0.760	-12.071	-22.784	0.00	0.00	PROA
ATOM	1096	HD2	ARG	P	70	1.950	-10.977	-22.149	0.00	0.00	PROA
ATOM	1097	NE	ARG	P	70	2.684	-12.307	-23.688	0.00	0.00	PROA
ATOM	1098	HE	ARG	P	70	3.614	-12.135	-23.363	0.00	0.00	PROA
ATOM	1099	CZ	ARG	P	70	2.551	-13.323	-24.557	0.00	0.00	PROA
ATOM	1100	NH1	ARG	P	70	1.413	-13.818	-25.039	0.00	0.00	PROA
ATOM	1101	HH11	ARG	P	70	0.531	-13.435	-24.763	0.00	0.00	PROA
ATOM	1102	HH12	ARG	P	70	1.546	-14.807	-25.106	0.00	0.00	PROA
ATOM	1103	NH2	ARG	P	70	3.689	-13.935	-24.852	0.00	0.00	PROA
ATOM	1104	HH21	ARG	P	70	4.513	-13.492	-24.499	0.00	0.00	PROA
ATOM	1105	HH22	ARG	P	70	3.771	-14.377	-25.745	0.00	0.00	PROA
ATOM	1106	C	ARG	P	70	2.987	-6.862	-24.830	0.00	0.00	PROA
ATOM	1107	O	ARG	P	70	4.168	-6.969	-25.121	0.00	0.00	PROA
ATOM	1108	N	SER	P	71	2.429	-5.741	-24.345	0.00	0.00	PROA
ATOM	1109	HN	SER	P	71	1.452	-5.652	-24.166	0.00	0.00	PROA
ATOM	1110	CA	SER	P	71	3.189	-4.537	-24.116	0.00	0.00	PROA
ATOM	1111	HA	SER	P	71	3.984	-4.650	-24.839	0.00	0.00	PROA
ATOM	1112	CB	SER	P	71	2.347	-3.340	-24.438	0.00	0.00	PROA
ATOM	1113	HB1	SER	P	71	1.637	-3.103	-23.617	0.00	0.00	PROA
ATOM	1114	HB2	SER	P	71	1.732	-3.601	-25.326	0.00	0.00	PROA
ATOM	1115	OG	SER	P	71	3.063	-2.124	-24.724	0.00	0.00	PROA
ATOM	1116	HG1	SER	P	71	3.589	-2.339	-25.498	0.00	0.00	PROA
ATOM	1117	C	SER	P	71	3.626	-4.421	-22.746	0.00	0.00	PROA
ATOM	1118	O	SER	P	71	3.338	-5.310	-21.915	0.00	0.00	PROA
ATOM	1119	N	MET	P	72	4.428	-3.386	-22.467	0.00	0.00	PROA
ATOM	1120	HN	MET	P	72	4.768	-2.909	-23.275	0.00	0.00	PROA
ATOM	1121	CA	MET	P	72	5.173	-3.202	-21.163	0.00	0.00	PROA
ATOM	1122	HA	MET	P	72	5.759	-4.097	-21.014	0.00	0.00	PROA
ATOM	1123	CB	MET	P	72	6.108	-1.969	-21.182	0.00	0.00	PROA
ATOM	1124	HB1	MET	P	72	6.495	-1.781	-20.157	0.00	0.00	PROA
ATOM	1125	HB2	MET	P	72	5.386	-1.179	-21.478	0.00	0.00	PROA
ATOM	1126	CG	MET	P	72	7.363	-2.017	-22.046	0.00	0.00	PROA
ATOM	1127	HG1	MET	P	72	8.038	-2.867	-21.807	0.00	0.00	PROA
ATOM	1128	HG2	MET	P	72	8.021	-1.132	-21.916	0.00	0.00	PROA
ATOM	1129	SD	MET	P	72	7.098	-2.103	-23.729	0.00	0.00	PROA
ATOM	1130	CE	MET	P	72	8.844	-2.145	-24.144	0.00	0.00	PROA
ATOM	1131	HE1	MET	P	72	9.044	-1.556	-25.065	0.00	0.00	PROA
ATOM	1132	HE2	MET	P	72	9.233	-3.159	-24.376	0.00	0.00	PROA
ATOM	1133	HE3	MET	P	72	9.445	-1.638	-23.359	0.00	0.00	PROA
ATOM	1134	C	MET	P	72	4.126	-3.218	-20.010	0.00	0.00	PROA
ATOM	1135	O	MET	P	72	4.310	-3.825	-18.990	0.00	0.00	PROA
ATOM	1136	N	THR	P	73	2.997	-2.492	-20.195	0.00	0.00	PROA
ATOM	1137	HN	THR	P	73	2.771	-2.155	-21.106	0.00	0.00	PROA
ATOM	1138	CA	THR	P	73	1.958	-2.325	-19.175	0.00	0.00	PROA
ATOM	1139	HA	THR	P	73	2.482	-2.188	-18.240	0.00	0.00	PROA
ATOM	1140	CB	THR	P	73	0.854	-1.290	-19.406	0.00	0.00	PROA
ATOM	1141	HB	THR	P	73	0.068	-1.416	-18.631	0.00	0.00	PROA
ATOM	1142	OG1	THR	P	73	0.278	-1.542	-20.649	0.00	0.00	PROA
ATOM	1143	HG1	THR	P	73	0.774	-1.014	-21.279	0.00	0.00	PROA
ATOM	1144	CG2	THR	P	73	1.374	0.107	-19.482	0.00	0.00	PROA
ATOM	1145	HG21	THR	P	73	2.037	0.202	-20.368	0.00	0.00	PROA
ATOM	1146	HG22	THR	P	73	1.952	0.447	-18.596	0.00	0.00	PROA
ATOM	1147	HG23	THR	P	73	0.555	0.835	-19.667	0.00	0.00	PROA
ATOM	1148	C	THR	P	73	1.321	-3.654	-18.897	0.00	0.00	PROA
ATOM	1149	O	THR	P	73	1.020	-3.928	-17.752	0.00	0.00	PROA
ATOM	1150	N	ASP	P	74	1.136	-4.501	-19.916	0.00	0.00	PROA
ATOM	1151	HN	ASP	P	74	1.529	-4.280	-20.805	0.00	0.00	PROA
ATOM	1152	CA	ASP	P	74	0.612	-5.832	-19.842	0.00	0.00	PROA
ATOM	1153	HA	ASP	P	74	-0.310	-5.821	-19.279	0.00	0.00	PROA
ATOM	1154	CB	ASP	P	74	0.445	-6.456	-21.256	0.00	0.00	PROA

ATOM	1155	HB1	ASP	P	74	-0.146	-7.392	-21.161	0.00	0.00	PROA
ATOM	1156	HB2	ASP	P	74	1.464	-6.655	-21.653	0.00	0.00	PROA
ATOM	1157	CG	ASP	P	74	-0.418	-5.456	-22.104	0.00	0.00	PROA
ATOM	1158	OD1	ASP	P	74	-1.388	-4.860	-21.546	0.00	0.00	PROA
ATOM	1159	OD2	ASP	P	74	-0.191	-5.424	-23.305	0.00	0.00	PROA
ATOM	1160	C	ASP	P	74	1.586	-6.668	-19.018	0.00	0.00	PROA
ATOM	1161	O	ASP	P	74	1.165	-7.523	-18.199	0.00	0.00	PROA
ATOM	1162	N	LYS	P	75	2.909	-6.557	-19.272	0.00	0.00	PROA
ATOM	1163	HN	LYS	P	75	3.246	-6.032	-20.050	0.00	0.00	PROA
ATOM	1164	CA	LYS	P	75	3.882	-7.243	-18.416	0.00	0.00	PROA
ATOM	1165	HA	LYS	P	75	3.633	-8.294	-18.445	0.00	0.00	PROA
ATOM	1166	CB	LYS	P	75	5.306	-7.219	-19.000	0.00	0.00	PROA
ATOM	1167	HB1	LYS	P	75	6.117	-7.779	-18.488	0.00	0.00	PROA
ATOM	1168	HB2	LYS	P	75	5.709	-6.185	-19.065	0.00	0.00	PROA
ATOM	1169	CG	LYS	P	75	5.261	-7.725	-20.478	0.00	0.00	PROA
ATOM	1170	HG1	LYS	P	75	4.628	-6.967	-20.988	0.00	0.00	PROA
ATOM	1171	HG2	LYS	P	75	4.712	-8.690	-20.505	0.00	0.00	PROA
ATOM	1172	CD	LYS	P	75	6.555	-7.761	-21.347	0.00	0.00	PROA
ATOM	1173	HD1	LYS	P	75	6.940	-8.772	-21.093	0.00	0.00	PROA
ATOM	1174	HD2	LYS	P	75	7.216	-7.002	-20.876	0.00	0.00	PROA
ATOM	1175	CE	LYS	P	75	6.313	-7.539	-22.810	0.00	0.00	PROA
ATOM	1176	HE1	LYS	P	75	5.855	-6.560	-23.068	0.00	0.00	PROA
ATOM	1177	HE2	LYS	P	75	5.731	-8.374	-23.255	0.00	0.00	PROA
ATOM	1178	NZ	LYS	P	75	7.540	-7.603	-23.611	0.00	0.00	PROA
ATOM	1179	HZ1	LYS	P	75	7.352	-7.353	-24.603	0.00	0.00	PROA
ATOM	1180	HZ2	LYS	P	75	7.687	-8.631	-23.556	0.00	0.00	PROA
ATOM	1181	HZ3	LYS	P	75	8.390	-7.148	-23.221	0.00	0.00	PROA
ATOM	1182	C	LYS	P	75	3.876	-6.756	-17.015	0.00	0.00	PROA
ATOM	1183	O	LYS	P	75	4.002	-7.574	-16.074	0.00	0.00	PROA
ATOM	1184	N	TYR	P	76	3.800	-5.397	-16.755	0.00	0.00	PROA
ATOM	1185	HN	TYR	P	76	3.873	-4.803	-17.552	0.00	0.00	PROA
ATOM	1186	CA	TYR	P	76	3.703	-4.791	-15.469	0.00	0.00	PROA
ATOM	1187	HA	TYR	P	76	4.634	-4.936	-14.940	0.00	0.00	PROA
ATOM	1188	CB	TYR	P	76	3.650	-3.217	-15.477	0.00	0.00	PROA
ATOM	1189	HB1	TYR	P	76	3.610	-2.854	-14.428	0.00	0.00	PROA
ATOM	1190	HB2	TYR	P	76	2.840	-2.857	-16.147	0.00	0.00	PROA
ATOM	1191	CG	TYR	P	76	4.920	-2.532	-15.929	0.00	0.00	PROA
ATOM	1192	CD1	TYR	P	76	6.164	-2.864	-15.321	0.00	0.00	PROA
ATOM	1193	HD1	TYR	P	76	6.250	-3.702	-14.645	0.00	0.00	PROA
ATOM	1194	CE1	TYR	P	76	7.322	-2.131	-15.654	0.00	0.00	PROA
ATOM	1195	HE1	TYR	P	76	8.249	-2.415	-15.179	0.00	0.00	PROA
ATOM	1196	CZ	TYR	P	76	7.287	-1.009	-16.499	0.00	0.00	PROA
ATOM	1197	OH	TYR	P	76	8.447	-0.416	-16.848	0.00	0.00	PROA
ATOM	1198	HH	TYR	P	76	8.257	0.449	-17.219	0.00	0.00	PROA
ATOM	1199	CD2	TYR	P	76	4.899	-1.422	-16.876	0.00	0.00	PROA
ATOM	1200	HD2	TYR	P	76	3.973	-1.095	-17.325	0.00	0.00	PROA
ATOM	1201	CE2	TYR	P	76	6.016	-0.660	-17.086	0.00	0.00	PROA
ATOM	1202	HE2	TYR	P	76	5.883	0.174	-17.759	0.00	0.00	PROA
ATOM	1203	C	TYR	P	76	2.586	-5.256	-14.626	0.00	0.00	PROA
ATOM	1204	O	TYR	P	76	2.710	-5.542	-13.416	0.00	0.00	PROA
ATOM	1205	N	ARG	P	77	1.378	-5.425	-15.270	0.00	0.00	PROA
ATOM	1206	HN	ARG	P	77	1.141	-5.235	-16.220	0.00	0.00	PROA
ATOM	1207	CA	ARG	P	77	0.213	-6.043	-14.538	0.00	0.00	PROA
ATOM	1208	HA	ARG	P	77	0.062	-5.372	-13.706	0.00	0.00	PROA
ATOM	1209	CB	ARG	P	77	-0.887	-6.046	-15.603	0.00	0.00	PROA
ATOM	1210	HB1	ARG	P	77	-0.488	-6.562	-16.502	0.00	0.00	PROA
ATOM	1211	HB2	ARG	P	77	-1.174	-5.056	-16.020	0.00	0.00	PROA
ATOM	1212	CG	ARG	P	77	-2.195	-6.719	-15.219	0.00	0.00	PROA
ATOM	1213	HG1	ARG	P	77	-2.028	-7.803	-15.042	0.00	0.00	PROA
ATOM	1214	HG2	ARG	P	77	-2.979	-6.673	-16.005	0.00	0.00	PROA
ATOM	1215	CD	ARG	P	77	-2.837	-6.069	-13.945	0.00	0.00	PROA

ATOM	1216	HD1	ARG	P	77	-2.094	-6.132	-13.121	0.00	0.00	PROA
ATOM	1217	HD2	ARG	P	77	-3.818	-6.519	-13.684	0.00	0.00	PROA
ATOM	1218	NE	ARG	P	77	-3.001	-4.601	-14.284	0.00	0.00	PROA
ATOM	1219	HE	ARG	P	77	-3.535	-4.274	-15.064	0.00	0.00	PROA
ATOM	1220	CZ	ARG	P	77	-2.597	-3.571	-13.541	0.00	0.00	PROA
ATOM	1221	NH1	ARG	P	77	-2.317	-3.646	-12.262	0.00	0.00	PROA
ATOM	1222	HH11	ARG	P	77	-2.396	-4.523	-11.788	0.00	0.00	PROA
ATOM	1223	HH12	ARG	P	77	-2.195	-2.766	-11.802	0.00	0.00	PROA
ATOM	1224	NH2	ARG	P	77	-2.392	-2.385	-14.007	0.00	0.00	PROA
ATOM	1225	HH21	ARG	P	77	-2.404	-2.273	-15.001	0.00	0.00	PROA
ATOM	1226	HH22	ARG	P	77	-2.152	-1.672	-13.348	0.00	0.00	PROA
ATOM	1227	C	ARG	P	77	0.494	-7.501	-14.017	0.00	0.00	PROA
ATOM	1228	O	ARG	P	77	0.114	-7.953	-12.907	0.00	0.00	PROA
ATOM	1229	N	LEU	P	78	1.187	-8.310	-14.852	0.00	0.00	PROA
ATOM	1230	HN	LEU	P	78	1.665	-8.045	-15.686	0.00	0.00	PROA
ATOM	1231	CA	LEU	P	78	1.609	-9.628	-14.342	0.00	0.00	PROA
ATOM	1232	HA	LEU	P	78	0.782	-10.177	-13.916	0.00	0.00	PROA
ATOM	1233	CB	LEU	P	78	2.341	-10.415	-15.566	0.00	0.00	PROA
ATOM	1234	HB1	LEU	P	78	3.344	-10.045	-15.868	0.00	0.00	PROA
ATOM	1235	HB2	LEU	P	78	1.678	-10.165	-16.421	0.00	0.00	PROA
ATOM	1236	CG	LEU	P	78	2.393	-11.997	-15.403	0.00	0.00	PROA
ATOM	1237	HG	LEU	P	78	1.551	-12.305	-14.746	0.00	0.00	PROA
ATOM	1238	CD1	LEU	P	78	2.244	-12.590	-16.827	0.00	0.00	PROA
ATOM	1239	HD11	LEU	P	78	2.998	-12.122	-17.495	0.00	0.00	PROA
ATOM	1240	HD12	LEU	P	78	1.221	-12.482	-17.248	0.00	0.00	PROA
ATOM	1241	HD13	LEU	P	78	2.274	-13.700	-16.873	0.00	0.00	PROA
ATOM	1242	CD2	LEU	P	78	3.742	-12.529	-14.846	0.00	0.00	PROA
ATOM	1243	HD21	LEU	P	78	4.031	-12.027	-13.898	0.00	0.00	PROA
ATOM	1244	HD22	LEU	P	78	4.551	-12.463	-15.605	0.00	0.00	PROA
ATOM	1245	HD23	LEU	P	78	3.711	-13.623	-14.653	0.00	0.00	PROA
ATOM	1246	C	LEU	P	78	2.660	-9.491	-13.216	0.00	0.00	PROA
ATOM	1247	O	LEU	P	78	2.526	-10.123	-12.198	0.00	0.00	PROA
ATOM	1248	N	HSE	P	79	3.685	-8.587	-13.349	0.00	0.00	PROA
ATOM	1249	HN	HSE	P	79	3.805	-8.095	-14.208	0.00	0.00	PROA
ATOM	1250	CA	HSE	P	79	4.634	-8.282	-12.250	0.00	0.00	PROA
ATOM	1251	HA	HSE	P	79	5.277	-9.133	-12.080	0.00	0.00	PROA
ATOM	1252	CB	HSE	P	79	5.604	-7.103	-12.580	0.00	0.00	PROA
ATOM	1253	HB1	HSE	P	79	6.350	-7.031	-11.761	0.00	0.00	PROA
ATOM	1254	HB2	HSE	P	79	4.913	-6.242	-12.703	0.00	0.00	PROA
ATOM	1255	ND1	HSE	P	79	7.056	-6.112	-14.402	0.00	0.00	PROA
ATOM	1256	CG	HSE	P	79	6.332	-7.236	-13.830	0.00	0.00	PROA
ATOM	1257	CE1	HSE	P	79	7.683	-6.599	-15.473	0.00	0.00	PROA
ATOM	1258	HE1	HSE	P	79	8.310	-6.054	-16.179	0.00	0.00	PROA
ATOM	1259	NE2	HSE	P	79	7.445	-7.984	-15.568	0.00	0.00	PROA
ATOM	1260	HE2	HSE	P	79	7.710	-8.634	-16.280	0.00	0.00	PROA
ATOM	1261	CD2	HSE	P	79	6.600	-8.408	-14.604	0.00	0.00	PROA
ATOM	1262	HD2	HSE	P	79	6.289	-9.443	-14.529	0.00	0.00	PROA
ATOM	1263	C	HSE	P	79	3.925	-7.863	-10.971	0.00	0.00	PROA
ATOM	1264	O	HSE	P	79	4.307	-8.374	-9.947	0.00	0.00	PROA
ATOM	1265	N	LEU	P	80	2.904	-6.928	-10.912	0.00	0.00	PROA
ATOM	1266	HN	LEU	P	80	2.729	-6.463	-11.776	0.00	0.00	PROA
ATOM	1267	CA	LEU	P	80	2.086	-6.594	-9.714	0.00	0.00	PROA
ATOM	1268	HA	LEU	P	80	2.738	-6.364	-8.885	0.00	0.00	PROA
ATOM	1269	CB	LEU	P	80	1.072	-5.568	-10.041	0.00	0.00	PROA
ATOM	1270	HB1	LEU	P	80	0.340	-5.436	-9.215	0.00	0.00	PROA
ATOM	1271	HB2	LEU	P	80	0.549	-5.719	-11.010	0.00	0.00	PROA
ATOM	1272	CG	LEU	P	80	1.739	-4.114	-10.094	0.00	0.00	PROA
ATOM	1273	HG	LEU	P	80	2.680	-4.136	-10.684	0.00	0.00	PROA
ATOM	1274	CD1	LEU	P	80	0.920	-3.120	-10.854	0.00	0.00	PROA
ATOM	1275	HD11	LEU	P	80	-0.109	-3.001	-10.452	0.00	0.00	PROA
ATOM	1276	HD12	LEU	P	80	0.957	-3.384	-11.932	0.00	0.00	PROA

```
ATOM   1277 HD13 LEU P  80       1.427  -2.147 -10.678  0.00  0.00      PROA
ATOM   1278  CD2 LEU P  80       2.132  -3.567  -8.711  0.00  0.00      PROA
ATOM   1279 HD21 LEU P  80       2.900  -4.262  -8.309  0.00  0.00      PROA
ATOM   1280 HD22 LEU P  80       1.239  -3.556  -8.050  0.00  0.00      PROA
ATOM   1281 HD23 LEU P  80       2.641  -2.593  -8.872  0.00  0.00      PROA
ATOM   1282  C   LEU P  80       1.346  -7.865  -9.252  0.00  0.00      PROA
ATOM   1283  O   LEU P  80       1.432  -8.219  -8.086  0.00  0.00      PROA
ATOM   1284  N   SER P  81       0.805  -8.651 -10.130  0.00  0.00      PROA
ATOM   1285  HN  SER P  81       0.818  -8.363 -11.085  0.00  0.00      PROA
ATOM   1286  CA  SER P  81       0.050  -9.874  -9.762  0.00  0.00      PROA
ATOM   1287  HA  SER P  81      -0.679  -9.633  -9.003  0.00  0.00      PROA
ATOM   1288  CB  SER P  81      -0.559 -10.547 -10.975  0.00  0.00      PROA
ATOM   1289  HB1 SER P  81      -1.133 -11.487 -10.823  0.00  0.00      PROA
ATOM   1290  HB2 SER P  81       0.173 -10.846 -11.755  0.00  0.00      PROA
ATOM   1291  OG  SER P  81      -1.464  -9.641 -11.538  0.00  0.00      PROA
ATOM   1292  HG1 SER P  81      -0.952  -8.941 -11.951  0.00  0.00      PROA
ATOM   1293  C   SER P  81       0.967 -10.851  -9.144  0.00  0.00      PROA
ATOM   1294  O   SER P  81       0.513 -11.567  -8.306  0.00  0.00      PROA
ATOM   1295  N   VAL P  82       2.192 -11.015  -9.599  0.00  0.00      PROA
ATOM   1296  HN  VAL P  82       2.576 -10.415 -10.297  0.00  0.00      PROA
ATOM   1297  CA  VAL P  82       3.117 -11.854  -9.052  0.00  0.00      PROA
ATOM   1298  HA  VAL P  82       2.622 -12.798  -8.882  0.00  0.00      PROA
ATOM   1299  CB  VAL P  82       4.333 -12.261  -9.951  0.00  0.00      PROA
ATOM   1300  HB  VAL P  82       4.832 -11.306 -10.221  0.00  0.00      PROA
ATOM   1301  CG1 VAL P  82       5.407 -13.022  -9.107  0.00  0.00      PROA
ATOM   1302 HG11 VAL P  82       5.718 -12.493  -8.181  0.00  0.00      PROA
ATOM   1303 HG12 VAL P  82       6.174 -13.430  -9.800  0.00  0.00      PROA
ATOM   1304 HG13 VAL P  82       4.766 -13.904  -8.894  0.00  0.00      PROA
ATOM   1305  CG2 VAL P  82       3.838 -13.035 -11.191  0.00  0.00      PROA
ATOM   1306 HG21 VAL P  82       4.716 -13.309 -11.814  0.00  0.00      PROA
ATOM   1307 HG22 VAL P  82       3.206 -12.297 -11.731  0.00  0.00      PROA
ATOM   1308 HG23 VAL P  82       3.225 -13.917 -10.907  0.00  0.00      PROA
ATOM   1309  C   VAL P  82       3.546 -11.502  -7.634  0.00  0.00      PROA
ATOM   1310  O   VAL P  82       3.621 -12.330  -6.702  0.00  0.00      PROA
ATOM   1311  N   ALA P  83       3.726 -10.194  -7.469  0.00  0.00      PROA
ATOM   1312  HN  ALA P  83       3.593  -9.573  -8.238  0.00  0.00      PROA
ATOM   1313  CA  ALA P  83       4.014  -9.637  -6.176  0.00  0.00      PROA
ATOM   1314  HA  ALA P  83       4.852 -10.195  -5.784  0.00  0.00      PROA
ATOM   1315  CB  ALA P  83       4.453  -8.145  -6.403  0.00  0.00      PROA
ATOM   1316  HB1 ALA P  83       3.681  -7.559  -6.946  0.00  0.00      PROA
ATOM   1317  HB2 ALA P  83       5.421  -8.063  -6.943  0.00  0.00      PROA
ATOM   1318  HB3 ALA P  83       4.573  -7.603  -5.441  0.00  0.00      PROA
ATOM   1319  C   ALA P  83       2.889  -9.665  -5.128  0.00  0.00      PROA
ATOM   1320  O   ALA P  83       3.133  -9.704  -3.950  0.00  0.00      PROA
ATOM   1321  N   ASP P  84       1.599  -9.663  -5.568  0.00  0.00      PROA
ATOM   1322  HN  ASP P  84       1.417  -9.549  -6.542  0.00  0.00      PROA
ATOM   1323  CA  ASP P  84       0.384  -9.724  -4.720  0.00  0.00      PROA
ATOM   1324  HA  ASP P  84       0.539  -9.194  -3.792  0.00  0.00      PROA
ATOM   1325  CB  ASP P  84      -0.847  -9.094  -5.519  0.00  0.00      PROA
ATOM   1326  HB1 ASP P  84      -1.701  -9.516  -4.946  0.00  0.00      PROA
ATOM   1327  HB2 ASP P  84      -0.636  -9.540  -6.514  0.00  0.00      PROA
ATOM   1328  CG  ASP P  84      -1.078  -7.567  -5.493  0.00  0.00      PROA
ATOM   1329  OD1 ASP P  84      -0.497  -6.785  -4.702  0.00  0.00      PROA
ATOM   1330  OD2 ASP P  84      -2.074  -7.196  -6.180  0.00  0.00      PROA
ATOM   1331  C   ASP P  84       0.080 -11.149  -4.436  0.00  0.00      PROA
ATOM   1332  O   ASP P  84      -0.324 -11.379  -3.300  0.00  0.00      PROA
ATOM   1333  N   LEU P  85       0.310 -12.124  -5.310  0.00  0.00      PROA
ATOM   1334  HN  LEU P  85       0.533 -11.929  -6.262  0.00  0.00      PROA
ATOM   1335  CA  LEU P  85       0.296 -13.537  -4.921  0.00  0.00      PROA
ATOM   1336  HA  LEU P  85      -0.621 -13.794  -4.412  0.00  0.00      PROA
ATOM   1337  CB  LEU P  85       0.510 -14.436  -6.117  0.00  0.00      PROA
```

```
ATOM   1338  HB1 LEU P  85       0.789 -15.450  -5.759  0.00  0.00      PROA
ATOM   1339  HB2 LEU P  85       1.260 -13.907  -6.742  0.00  0.00      PROA
ATOM   1340  CG  LEU P  85      -0.709 -14.615  -7.034  0.00  0.00      PROA
ATOM   1341  HG  LEU P  85      -0.947 -13.574  -7.339  0.00  0.00      PROA
ATOM   1342  CD1 LEU P  85      -0.318 -15.393  -8.269  0.00  0.00      PROA
ATOM   1343 HD11 LEU P  85       0.061 -16.427  -8.121  0.00  0.00      PROA
ATOM   1344 HD12 LEU P  85       0.526 -14.820  -8.709  0.00  0.00      PROA
ATOM   1345 HD13 LEU P  85      -1.292 -15.534  -8.784  0.00  0.00      PROA
ATOM   1346  CD2 LEU P  85      -1.900 -15.175  -6.254  0.00  0.00      PROA
ATOM   1347 HD21 LEU P  85      -2.326 -14.404  -5.577  0.00  0.00      PROA
ATOM   1348 HD22 LEU P  85      -1.656 -16.031  -5.589  0.00  0.00      PROA
ATOM   1349 HD23 LEU P  85      -2.685 -15.467  -6.985  0.00  0.00      PROA
ATOM   1350  C   LEU P  85       1.352 -13.869  -3.859  0.00  0.00      PROA
ATOM   1351  O   LEU P  85       1.029 -14.603  -2.918  0.00  0.00      PROA
ATOM   1352  N   LEU P  86       2.601 -13.243  -3.900  0.00  0.00      PROA
ATOM   1353  HN  LEU P  86       2.706 -12.549  -4.608  0.00  0.00      PROA
ATOM   1354  CA  LEU P  86       3.537 -13.428  -2.754  0.00  0.00      PROA
ATOM   1355  HA  LEU P  86       3.628 -14.503  -2.699  0.00  0.00      PROA
ATOM   1356  CB  LEU P  86       4.997 -12.896  -2.999  0.00  0.00      PROA
ATOM   1357  HB1 LEU P  86       5.029 -11.837  -3.334  0.00  0.00      PROA
ATOM   1358  HB2 LEU P  86       5.345 -13.539  -3.836  0.00  0.00      PROA
ATOM   1359  CG  LEU P  86       6.090 -13.020  -1.919  0.00  0.00      PROA
ATOM   1360  HG  LEU P  86       5.911 -12.211  -1.179  0.00  0.00      PROA
ATOM   1361  CD1 LEU P  86       6.087 -14.184  -0.896  0.00  0.00      PROA
ATOM   1362 HD11 LEU P  86       6.173 -15.161  -1.418  0.00  0.00      PROA
ATOM   1363 HD12 LEU P  86       5.160 -14.091  -0.291  0.00  0.00      PROA
ATOM   1364 HD13 LEU P  86       7.105 -14.131  -0.454  0.00  0.00      PROA
ATOM   1365  CD2 LEU P  86       7.459 -12.752  -2.678  0.00  0.00      PROA
ATOM   1366 HD21 LEU P  86       7.387 -11.912  -3.402  0.00  0.00      PROA
ATOM   1367 HD22 LEU P  86       7.722 -13.671  -3.244  0.00  0.00      PROA
ATOM   1368 HD23 LEU P  86       8.196 -12.504  -1.884  0.00  0.00      PROA
ATOM   1369  C   LEU P  86       2.911 -12.915  -1.402  0.00  0.00      PROA
ATOM   1370  O   LEU P  86       2.994 -13.520  -0.355  0.00  0.00      PROA
ATOM   1371  N   PHE P  87       2.170 -11.765  -1.385  0.00  0.00      PROA
ATOM   1372  HN  PHE P  87       2.009 -11.257  -2.229  0.00  0.00      PROA
ATOM   1373  CA  PHE P  87       1.454 -11.270  -0.225  0.00  0.00      PROA
ATOM   1374  HA  PHE P  87       2.232 -11.230   0.523  0.00  0.00      PROA
ATOM   1375  CB  PHE P  87       0.993  -9.803  -0.415  0.00  0.00      PROA
ATOM   1376  HB1 PHE P  87       0.253  -9.879  -1.240  0.00  0.00      PROA
ATOM   1377  HB2 PHE P  87       1.905  -9.191  -0.579  0.00  0.00      PROA
ATOM   1378  CG  PHE P  87       0.240  -9.222   0.757  0.00  0.00      PROA
ATOM   1379  CD1 PHE P  87       0.944  -8.353   1.656  0.00  0.00      PROA
ATOM   1380  HD1 PHE P  87       1.930  -7.979   1.419  0.00  0.00      PROA
ATOM   1381  CE1 PHE P  87       0.256  -7.881   2.809  0.00  0.00      PROA
ATOM   1382  HE1 PHE P  87       0.812  -7.266   3.501  0.00  0.00      PROA
ATOM   1383  CZ  PHE P  87      -1.091  -8.266   3.086  0.00  0.00      PROA
ATOM   1384  HZ  PHE P  87      -1.523  -7.864   3.990  0.00  0.00      PROA
ATOM   1385  CD2 PHE P  87      -1.130  -9.485   0.964  0.00  0.00      PROA
ATOM   1386  HD2 PHE P  87      -1.672 -10.161   0.318  0.00  0.00      PROA
ATOM   1387  CE2 PHE P  87      -1.773  -9.021   2.139  0.00  0.00      PROA
ATOM   1388  HE2 PHE P  87      -2.783  -9.384   2.255  0.00  0.00      PROA
ATOM   1389  C   PHE P  87       0.479 -12.363   0.307  0.00  0.00      PROA
ATOM   1390  O   PHE P  87       0.553 -12.697   1.534  0.00  0.00      PROA
ATOM   1391  N   VAL P  88      -0.398 -12.987  -0.571  0.00  0.00      PROA
ATOM   1392  HN  VAL P  88      -0.365 -12.646  -1.508  0.00  0.00      PROA
ATOM   1393  CA  VAL P  88      -1.292 -14.045  -0.219  0.00  0.00      PROA
ATOM   1394  HA  VAL P  88      -1.976 -13.518   0.430  0.00  0.00      PROA
ATOM   1395  CB  VAL P  88      -2.178 -14.534  -1.361  0.00  0.00      PROA
ATOM   1396  HB  VAL P  88      -1.594 -14.920  -2.224  0.00  0.00      PROA
ATOM   1397  CG1 VAL P  88      -3.079 -15.802  -1.038  0.00  0.00      PROA
ATOM   1398 HG11 VAL P  88      -3.873 -16.009  -1.787  0.00  0.00      PROA
```

```
ATOM   1399 HG12 VAL P  88      -3.707 -15.578  -0.149  0.00  0.00      PROA
ATOM   1400 HG13 VAL P  88      -2.529 -16.754  -0.885  0.00  0.00      PROA
ATOM   1401  CG2 VAL P  88      -3.158 -13.373  -1.778  0.00  0.00      PROA
ATOM   1402 HG21 VAL P  88      -3.455 -13.611  -2.822  0.00  0.00      PROA
ATOM   1403 HG22 VAL P  88      -2.593 -12.432  -1.951  0.00  0.00      PROA
ATOM   1404 HG23 VAL P  88      -4.084 -13.287  -1.171  0.00  0.00      PROA
ATOM   1405  C   VAL P  88      -0.723 -15.290   0.476  0.00  0.00      PROA
ATOM   1406  O   VAL P  88      -1.277 -15.761   1.453  0.00  0.00      PROA
ATOM   1407  N   ILE P  89       0.505 -15.753   0.101  0.00  0.00      PROA
ATOM   1408  HN  ILE P  89       1.112 -15.392  -0.604  0.00  0.00      PROA
ATOM   1409  CA  ILE P  89       1.172 -16.875   0.797  0.00  0.00      PROA
ATOM   1410  HA  ILE P  89       0.360 -17.538   1.055  0.00  0.00      PROA
ATOM   1411  CB  ILE P  89       2.164 -17.693  -0.054  0.00  0.00      PROA
ATOM   1412  HB  ILE P  89       2.621 -18.491   0.568  0.00  0.00      PROA
ATOM   1413  CG2 ILE P  89       1.337 -18.340  -1.165  0.00  0.00      PROA
ATOM   1414 HG21 ILE P  89       0.251 -18.279  -0.941  0.00  0.00      PROA
ATOM   1415 HG22 ILE P  89       1.794 -19.283  -1.533  0.00  0.00      PROA
ATOM   1416 HG23 ILE P  89       1.477 -17.709  -2.069  0.00  0.00      PROA
ATOM   1417  CG1 ILE P  89       3.277 -16.785  -0.618  0.00  0.00      PROA
ATOM   1418 HG11 ILE P  89       3.743 -16.023   0.043  0.00  0.00      PROA
ATOM   1419 HG12 ILE P  89       2.791 -16.109  -1.353  0.00  0.00      PROA
ATOM   1420  CD  ILE P  89       4.432 -17.580  -1.220  0.00  0.00      PROA
ATOM   1421  HD1 ILE P  89       4.015 -18.347  -1.908  0.00  0.00      PROA
ATOM   1422  HD2 ILE P  89       5.120 -18.121  -0.535  0.00  0.00      PROA
ATOM   1423  HD3 ILE P  89       5.003 -16.879  -1.865  0.00  0.00      PROA
ATOM   1424  C   ILE P  89       1.810 -16.442   2.128  0.00  0.00      PROA
ATOM   1425  O   ILE P  89       1.952 -17.260   3.028  0.00  0.00      PROA
ATOM   1426  N   THR P  90       2.055 -15.108   2.316  0.00  0.00      PROA
ATOM   1427  HN  THR P  90       1.980 -14.583   1.472  0.00  0.00      PROA
ATOM   1428  CA  THR P  90       2.626 -14.578   3.553  0.00  0.00      PROA
ATOM   1429  HA  THR P  90       3.259 -15.316   4.024  0.00  0.00      PROA
ATOM   1430  CB  THR P  90       3.403 -13.301   3.254  0.00  0.00      PROA
ATOM   1431  HB  THR P  90       2.677 -12.472   3.117  0.00  0.00      PROA
ATOM   1432  OG1 THR P  90       4.309 -13.374   2.144  0.00  0.00      PROA
ATOM   1433  HG1 THR P  90       3.831 -13.327   1.313  0.00  0.00      PROA
ATOM   1434  CG2 THR P  90       4.335 -13.017   4.431  0.00  0.00      PROA
ATOM   1435 HG21 THR P  90       5.100 -12.230   4.258  0.00  0.00      PROA
ATOM   1436 HG22 THR P  90       4.876 -13.891   4.852  0.00  0.00      PROA
ATOM   1437 HG23 THR P  90       3.883 -12.609   5.360  0.00  0.00      PROA
ATOM   1438  C   THR P  90       1.529 -14.303   4.609  0.00  0.00      PROA
ATOM   1439  O   THR P  90       1.774 -14.362   5.797  0.00  0.00      PROA
ATOM   1440  N   LEU P  91       0.301 -14.280   4.157  0.00  0.00      PROA
ATOM   1441  HN  LEU P  91       0.153 -14.350   3.174  0.00  0.00      PROA
ATOM   1442  CA  LEU P  91      -0.970 -14.047   4.878  0.00  0.00      PROA
ATOM   1443  HA  LEU P  91      -0.773 -13.130   5.413  0.00  0.00      PROA
ATOM   1444  CB  LEU P  91      -2.065 -13.930   3.817  0.00  0.00      PROA
ATOM   1445  HB1 LEU P  91      -2.325 -14.856   3.261  0.00  0.00      PROA
ATOM   1446  HB2 LEU P  91      -1.701 -13.188   3.075  0.00  0.00      PROA
ATOM   1447  CG  LEU P  91      -3.449 -13.484   4.414  0.00  0.00      PROA
ATOM   1448  HG  LEU P  91      -3.965 -14.213   5.075  0.00  0.00      PROA
ATOM   1449  CD1 LEU P  91      -3.231 -12.193   5.190  0.00  0.00      PROA
ATOM   1450 HD11 LEU P  91      -2.707 -12.433   6.140  0.00  0.00      PROA
ATOM   1451 HD12 LEU P  91      -4.242 -11.816   5.455  0.00  0.00      PROA
ATOM   1452 HD13 LEU P  91      -2.604 -11.479   4.615  0.00  0.00      PROA
ATOM   1453  CD2 LEU P  91      -4.547 -13.364   3.328  0.00  0.00      PROA
ATOM   1454 HD21 LEU P  91      -4.242 -12.507   2.690  0.00  0.00      PROA
ATOM   1455 HD22 LEU P  91      -5.530 -13.093   3.770  0.00  0.00      PROA
ATOM   1456 HD23 LEU P  91      -4.725 -14.252   2.686  0.00  0.00      PROA
ATOM   1457  C   LEU P  91      -1.287 -15.066   6.015  0.00  0.00      PROA
ATOM   1458  O   LEU P  91      -1.491 -14.565   7.101  0.00  0.00      PROA
ATOM   1459  N   PRO P  92      -1.117 -16.419   5.882  0.00  0.00      PROA
```

ATOM	1460	CD	PRO	P	92	-1.022	-17.140	4.657	0.00	0.00	PROA
ATOM	1461	HD1	PRO	P	92	-0.405	-16.603	3.905	0.00	0.00	PROA
ATOM	1462	HD2	PRO	P	92	-2.009	-17.434	4.241	0.00	0.00	PROA
ATOM	1463	CA	PRO	P	92	-1.186	-17.339	6.985	0.00	0.00	PROA
ATOM	1464	HA	PRO	P	92	-2.222	-17.198	7.256	0.00	0.00	PROA
ATOM	1465	CB	PRO	P	92	-0.942	-18.703	6.431	0.00	0.00	PROA
ATOM	1466	HB1	PRO	P	92	-1.831	-19.355	6.294	0.00	0.00	PROA
ATOM	1467	HB2	PRO	P	92	-0.309	-19.257	7.156	0.00	0.00	PROA
ATOM	1468	CG	PRO	P	92	-0.300	-18.427	5.136	0.00	0.00	PROA
ATOM	1469	HG1	PRO	P	92	0.779	-18.234	5.316	0.00	0.00	PROA
ATOM	1470	HG2	PRO	P	92	-0.474	-19.282	4.448	0.00	0.00	PROA
ATOM	1471	C	PRO	P	92	-0.468	-17.041	8.220	0.00	0.00	PROA
ATOM	1472	O	PRO	P	92	-1.085	-17.148	9.290	0.00	0.00	PROA
ATOM	1473	N	PHE	P	93	0.851	-16.707	8.266	0.00	0.00	PROA
ATOM	1474	HN	PHE	P	93	1.371	-16.618	7.420	0.00	0.00	PROA
ATOM	1475	CA	PHE	P	93	1.683	-16.379	9.426	0.00	0.00	PROA
ATOM	1476	HA	PHE	P	93	1.699	-17.244	10.072	0.00	0.00	PROA
ATOM	1477	CB	PHE	P	93	3.160	-16.041	9.021	0.00	0.00	PROA
ATOM	1478	HB1	PHE	P	93	3.865	-16.056	9.880	0.00	0.00	PROA
ATOM	1479	HB2	PHE	P	93	3.187	-15.010	8.608	0.00	0.00	PROA
ATOM	1480	CG	PHE	P	93	3.781	-17.093	8.045	0.00	0.00	PROA
ATOM	1481	CD1	PHE	P	93	3.976	-18.449	8.310	0.00	0.00	PROA
ATOM	1482	HD1	PHE	P	93	3.660	-18.828	9.271	0.00	0.00	PROA
ATOM	1483	CE1	PHE	P	93	4.459	-19.361	7.337	0.00	0.00	PROA
ATOM	1484	HE1	PHE	P	93	4.454	-20.410	7.594	0.00	0.00	PROA
ATOM	1485	CZ	PHE	P	93	4.633	-18.856	6.088	0.00	0.00	PROA
ATOM	1486	HZ	PHE	P	93	4.962	-19.563	5.341	0.00	0.00	PROA
ATOM	1487	CD2	PHE	P	93	4.058	-16.629	6.779	0.00	0.00	PROA
ATOM	1488	HD2	PHE	P	93	3.978	-15.579	6.541	0.00	0.00	PROA
ATOM	1489	CE2	PHE	P	93	4.468	-17.540	5.742	0.00	0.00	PROA
ATOM	1490	HE2	PHE	P	93	4.627	-17.210	4.726	0.00	0.00	PROA
ATOM	1491	C	PHE	P	93	1.106	-15.188	10.179	0.00	0.00	PROA
ATOM	1492	O	PHE	P	93	1.049	-15.263	11.399	0.00	0.00	PROA
ATOM	1493	N	TRP	P	94	0.703	-14.156	9.372	0.00	0.00	PROA
ATOM	1494	HN	TRP	P	94	0.765	-14.244	8.381	0.00	0.00	PROA
ATOM	1495	CA	TRP	P	94	0.007	-12.914	9.780	0.00	0.00	PROA
ATOM	1496	HA	TRP	P	94	0.542	-12.502	10.623	0.00	0.00	PROA
ATOM	1497	CB	TRP	P	94	-0.084	-11.904	8.620	0.00	0.00	PROA
ATOM	1498	HB1	TRP	P	94	-0.689	-11.039	8.966	0.00	0.00	PROA
ATOM	1499	HB2	TRP	P	94	-0.486	-12.423	7.724	0.00	0.00	PROA
ATOM	1500	CG	TRP	P	94	1.194	-11.292	8.274	0.00	0.00	PROA
ATOM	1501	CD1	TRP	P	94	2.322	-11.019	9.035	0.00	0.00	PROA
ATOM	1502	HD1	TRP	P	94	2.424	-11.378	10.048	0.00	0.00	PROA
ATOM	1503	NE1	TRP	P	94	3.212	-10.365	8.227	0.00	0.00	PROA
ATOM	1504	HE1	TRP	P	94	3.973	-9.769	8.365	0.00	0.00	PROA
ATOM	1505	CE2	TRP	P	94	2.758	-10.299	7.003	0.00	0.00	PROA
ATOM	1506	CD2	TRP	P	94	1.441	-10.778	6.949	0.00	0.00	PROA
ATOM	1507	CE3	TRP	P	94	0.729	-10.774	5.799	0.00	0.00	PROA
ATOM	1508	HE3	TRP	P	94	-0.304	-11.032	5.618	0.00	0.00	PROA
ATOM	1509	CZ3	TRP	P	94	1.331	-10.243	4.642	0.00	0.00	PROA
ATOM	1510	HZ3	TRP	P	94	0.798	-10.268	3.703	0.00	0.00	PROA
ATOM	1511	CZ2	TRP	P	94	3.382	-9.788	5.877	0.00	0.00	PROA
ATOM	1512	HZ2	TRP	P	94	4.350	-9.308	5.867	0.00	0.00	PROA
ATOM	1513	CH2	TRP	P	94	2.648	-9.737	4.697	0.00	0.00	PROA
ATOM	1514	HH2	TRP	P	94	3.113	-9.408	3.779	0.00	0.00	PROA
ATOM	1515	C	TRP	P	94	-1.393	-13.273	10.346	0.00	0.00	PROA
ATOM	1516	O	TRP	P	94	-1.884	-12.724	11.310	0.00	0.00	PROA
ATOM	1517	N	ALA	P	95	-2.079	-14.311	9.781	0.00	0.00	PROA
ATOM	1518	HN	ALA	P	95	-1.687	-14.885	9.066	0.00	0.00	PROA
ATOM	1519	CA	ALA	P	95	-3.332	-14.778	10.387	0.00	0.00	PROA
ATOM	1520	HA	ALA	P	95	-3.921	-13.898	10.598	0.00	0.00	PROA

```
ATOM   1521  CB  ALA P  95      -4.216 -15.583   9.346  0.00  0.00      PROA
ATOM   1522  HB1 ALA P  95      -3.666 -16.517   9.099  0.00  0.00      PROA
ATOM   1523  HB2 ALA P  95      -4.382 -14.934   8.460  0.00  0.00      PROA
ATOM   1524  HB3 ALA P  95      -5.168 -15.847   9.854  0.00  0.00      PROA
ATOM   1525  C   ALA P  95      -3.093 -15.525  11.776  0.00  0.00      PROA
ATOM   1526  O   ALA P  95      -3.775 -15.198  12.716  0.00  0.00      PROA
ATOM   1527  N   VAL P  96      -2.174 -16.443  11.903  0.00  0.00      PROA
ATOM   1528  HN  VAL P  96      -1.704 -16.822  11.110  0.00  0.00      PROA
ATOM   1529  CA  VAL P  96      -1.943 -17.024  13.183  0.00  0.00      PROA
ATOM   1530  HA  VAL P  96      -2.847 -17.561  13.428  0.00  0.00      PROA
ATOM   1531  CB  VAL P  96      -0.837 -18.056  13.083  0.00  0.00      PROA
ATOM   1532  HB  VAL P  96       0.048 -17.799  12.463  0.00  0.00      PROA
ATOM   1533  CG1 VAL P  96      -0.498 -18.750  14.356  0.00  0.00      PROA
ATOM   1534 HG11 VAL P  96       0.242 -18.146  14.923  0.00  0.00      PROA
ATOM   1535 HG12 VAL P  96       0.153 -19.632  14.174  0.00  0.00      PROA
ATOM   1536 HG13 VAL P  96      -1.389 -19.053  14.947  0.00  0.00      PROA
ATOM   1537  CG2 VAL P  96      -1.470 -19.133  12.192  0.00  0.00      PROA
ATOM   1538 HG21 VAL P  96      -2.257 -19.639  12.791  0.00  0.00      PROA
ATOM   1539 HG22 VAL P  96      -0.653 -19.865  12.019  0.00  0.00      PROA
ATOM   1540 HG23 VAL P  96      -1.894 -18.699  11.261  0.00  0.00      PROA
ATOM   1541  C   VAL P  96      -1.682 -16.035  14.343  0.00  0.00      PROA
ATOM   1542  O   VAL P  96      -2.191 -16.071  15.452  0.00  0.00      PROA
ATOM   1543  N   ASP P  97      -0.856 -15.054  14.002  0.00  0.00      PROA
ATOM   1544  HN  ASP P  97      -0.498 -14.970  13.075  0.00  0.00      PROA
ATOM   1545  CA  ASP P  97      -0.506 -13.805  14.695  0.00  0.00      PROA
ATOM   1546  HA  ASP P  97       0.003 -14.069  15.610  0.00  0.00      PROA
ATOM   1547  CB  ASP P  97       0.455 -12.891  13.828  0.00  0.00      PROA
ATOM   1548  HB1 ASP P  97      -0.054 -12.479  12.931  0.00  0.00      PROA
ATOM   1549  HB2 ASP P  97       1.200 -13.590  13.391  0.00  0.00      PROA
ATOM   1550  CG  ASP P  97       1.251 -11.786  14.588  0.00  0.00      PROA
ATOM   1551  OD1 ASP P  97       2.447 -11.632  14.250  0.00  0.00      PROA
ATOM   1552  OD2 ASP P  97       0.560 -10.964  15.249  0.00  0.00      PROA
ATOM   1553  C   ASP P  97      -1.614 -12.978  15.125  0.00  0.00      PROA
ATOM   1554  O   ASP P  97      -1.819 -12.698  16.303  0.00  0.00      PROA
ATOM   1555  N   ALA P  98      -2.499 -12.624  14.202  0.00  0.00      PROA
ATOM   1556  HN  ALA P  98      -2.347 -12.939  13.268  0.00  0.00      PROA
ATOM   1557  CA  ALA P  98      -3.703 -11.813  14.525  0.00  0.00      PROA
ATOM   1558  HA  ALA P  98      -3.363 -10.853  14.886  0.00  0.00      PROA
ATOM   1559  CB  ALA P  98      -4.466 -11.599  13.151  0.00  0.00      PROA
ATOM   1560  HB1 ALA P  98      -3.659 -11.183  12.512  0.00  0.00      PROA
ATOM   1561  HB2 ALA P  98      -5.303 -10.871  13.222  0.00  0.00      PROA
ATOM   1562  HB3 ALA P  98      -4.661 -12.595  12.700  0.00  0.00      PROA
ATOM   1563  C   ALA P  98      -4.690 -12.456  15.510  0.00  0.00      PROA
ATOM   1564  O   ALA P  98      -5.158 -11.877  16.440  0.00  0.00      PROA
ATOM   1565  N   VAL P  99      -4.948 -13.746  15.354  0.00  0.00      PROA
ATOM   1566  HN  VAL P  99      -4.563 -14.199  14.553  0.00  0.00      PROA
ATOM   1567  CA  VAL P  99      -5.604 -14.600  16.347  0.00  0.00      PROA
ATOM   1568  HA  VAL P  99      -6.484 -14.105  16.730  0.00  0.00      PROA
ATOM   1569  CB  VAL P  99      -5.935 -15.996  15.753  0.00  0.00      PROA
ATOM   1570  HB  VAL P  99      -4.989 -16.467  15.409  0.00  0.00      PROA
ATOM   1571  CG1 VAL P  99      -6.747 -16.852  16.697  0.00  0.00      PROA
ATOM   1572 HG11 VAL P  99      -7.445 -16.246  17.313  0.00  0.00      PROA
ATOM   1573 HG12 VAL P  99      -6.104 -17.364  17.444  0.00  0.00      PROA
ATOM   1574 HG13 VAL P  99      -7.281 -17.658  16.151  0.00  0.00      PROA
ATOM   1575  CG2 VAL P  99      -6.772 -15.687  14.445  0.00  0.00      PROA
ATOM   1576 HG21 VAL P  99      -7.552 -14.971  14.781  0.00  0.00      PROA
ATOM   1577 HG22 VAL P  99      -7.350 -16.490  13.939  0.00  0.00      PROA
ATOM   1578 HG23 VAL P  99      -6.025 -15.248  13.750  0.00  0.00      PROA
ATOM   1579  C   VAL P  99      -4.830 -14.763  17.652  0.00  0.00      PROA
ATOM   1580  O   VAL P  99      -5.385 -14.548  18.740  0.00  0.00      PROA
ATOM   1581  N   ALA P 100      -3.569 -15.214  17.676  0.00  0.00      PROA
```

ATOM	1582	HN	ALA	P	100	-3.112	-15.314	16.795	0.00	0.00	PROA
ATOM	1583	CA	ALA	P	100	-2.834	-15.583	18.857	0.00	0.00	PROA
ATOM	1584	HA	ALA	P	100	-3.460	-15.258	19.674	0.00	0.00	PROA
ATOM	1585	CB	ALA	P	100	-2.772	-17.136	18.931	0.00	0.00	PROA
ATOM	1586	HB1	ALA	P	100	-3.766	-17.633	18.951	0.00	0.00	PROA
ATOM	1587	HB2	ALA	P	100	-2.197	-17.400	19.844	0.00	0.00	PROA
ATOM	1588	HB3	ALA	P	100	-2.321	-17.536	17.997	0.00	0.00	PROA
ATOM	1589	C	ALA	P	100	-1.478	-15.114	19.123	0.00	0.00	PROA
ATOM	1590	O	ALA	P	100	-1.324	-14.160	19.857	0.00	0.00	PROA
ATOM	1591	N	ASN	P	101	-0.363	-15.772	18.643	0.00	0.00	PROA
ATOM	1592	HN	ASN	P	101	-0.457	-16.478	17.945	0.00	0.00	PROA
ATOM	1593	CA	ASN	P	101	0.999	-15.389	18.950	0.00	0.00	PROA
ATOM	1594	HA	ASN	P	101	1.191	-14.329	19.026	0.00	0.00	PROA
ATOM	1595	CB	ASN	P	101	1.509	-16.123	20.263	0.00	0.00	PROA
ATOM	1596	HB1	ASN	P	101	2.590	-16.376	20.278	0.00	0.00	PROA
ATOM	1597	HB2	ASN	P	101	0.972	-17.095	20.222	0.00	0.00	PROA
ATOM	1598	CG	ASN	P	101	1.104	-15.233	21.451	0.00	0.00	PROA
ATOM	1599	OD1	ASN	P	101	0.281	-15.663	22.269	0.00	0.00	PROA
ATOM	1600	ND2	ASN	P	101	1.838	-14.097	21.677	0.00	0.00	PROA
ATOM	1601	HD21	ASN	P	101	1.605	-13.576	22.498	0.00	0.00	PROA
ATOM	1602	HD22	ASN	P	101	2.692	-13.827	21.232	0.00	0.00	PROA
ATOM	1603	C	ASN	P	101	1.805	-15.911	17.793	0.00	0.00	PROA
ATOM	1604	O	ASN	P	101	1.349	-16.703	16.912	0.00	0.00	PROA
ATOM	1605	N	TRP	P	102	3.007	-15.395	17.642	0.00	0.00	PROA
ATOM	1606	HN	TRP	P	102	3.335	-14.642	18.208	0.00	0.00	PROA
ATOM	1607	CA	TRP	P	102	3.897	-15.940	16.606	0.00	0.00	PROA
ATOM	1608	HA	TRP	P	102	3.343	-16.086	15.691	0.00	0.00	PROA
ATOM	1609	CB	TRP	P	102	5.044	-14.937	16.238	0.00	0.00	PROA
ATOM	1610	HB1	TRP	P	102	5.641	-14.698	17.144	0.00	0.00	PROA
ATOM	1611	HB2	TRP	P	102	4.475	-14.033	15.930	0.00	0.00	PROA
ATOM	1612	CG	TRP	P	102	5.943	-15.181	15.092	0.00	0.00	PROA
ATOM	1613	CD1	TRP	P	102	7.167	-15.727	15.204	0.00	0.00	PROA
ATOM	1614	HD1	TRP	P	102	7.585	-16.289	16.026	0.00	0.00	PROA
ATOM	1615	NE1	TRP	P	102	7.720	-15.789	13.930	0.00	0.00	PROA
ATOM	1616	HE1	TRP	P	102	8.655	-15.963	13.709	0.00	0.00	PROA
ATOM	1617	CE2	TRP	P	102	6.920	-15.115	13.093	0.00	0.00	PROA
ATOM	1618	CD2	TRP	P	102	5.738	-14.825	13.743	0.00	0.00	PROA
ATOM	1619	CE3	TRP	P	102	4.652	-14.372	13.036	0.00	0.00	PROA
ATOM	1620	HE3	TRP	P	102	3.686	-14.191	13.483	0.00	0.00	PROA
ATOM	1621	CZ3	TRP	P	102	4.792	-14.037	11.694	0.00	0.00	PROA
ATOM	1622	HZ3	TRP	P	102	3.917	-13.582	11.254	0.00	0.00	PROA
ATOM	1623	CZ2	TRP	P	102	7.128	-14.764	11.685	0.00	0.00	PROA
ATOM	1624	HZ2	TRP	P	102	8.041	-15.020	11.167	0.00	0.00	PROA
ATOM	1625	CH2	TRP	P	102	6.028	-14.128	11.045	0.00	0.00	PROA
ATOM	1626	HH2	TRP	P	102	6.140	-13.757	10.036	0.00	0.00	PROA
ATOM	1627	C	TRP	P	102	4.584	-17.268	16.933	0.00	0.00	PROA
ATOM	1628	O	TRP	P	102	4.966	-17.559	18.068	0.00	0.00	PROA
ATOM	1629	N	TYR	P	103	4.753	-18.174	15.923	0.00	0.00	PROA
ATOM	1630	HN	TYR	P	103	4.380	-18.052	15.007	0.00	0.00	PROA
ATOM	1631	CA	TYR	P	103	5.243	-19.610	16.137	0.00	0.00	PROA
ATOM	1632	HA	TYR	P	103	5.595	-19.734	17.150	0.00	0.00	PROA
ATOM	1633	CB	TYR	P	103	4.134	-20.651	16.004	0.00	0.00	PROA
ATOM	1634	HB1	TYR	P	103	4.518	-21.662	16.261	0.00	0.00	PROA
ATOM	1635	HB2	TYR	P	103	3.743	-20.766	14.971	0.00	0.00	PROA
ATOM	1636	CG	TYR	P	103	3.015	-20.468	17.022	0.00	0.00	PROA
ATOM	1637	CD1	TYR	P	103	3.151	-20.939	18.339	0.00	0.00	PROA
ATOM	1638	HD1	TYR	P	103	4.077	-21.406	18.640	0.00	0.00	PROA
ATOM	1639	CE1	TYR	P	103	2.075	-20.934	19.253	0.00	0.00	PROA
ATOM	1640	HE1	TYR	P	103	2.240	-21.326	20.245	0.00	0.00	PROA
ATOM	1641	CZ	TYR	P	103	0.900	-20.315	18.888	0.00	0.00	PROA
ATOM	1642	OH	TYR	P	103	-0.125	-20.185	19.837	0.00	0.00	PROA

ATOM	1643	HH	TYR	P	103	0.165	-20.616	20.644	0.00	0.00	PROA
ATOM	1644	CD2	TYR	P	103	1.767	-19.845	16.679	0.00	0.00	PROA
ATOM	1645	HD2	TYR	P	103	1.788	-19.171	15.835	0.00	0.00	PROA
ATOM	1646	CE2	TYR	P	103	0.731	-19.802	17.572	0.00	0.00	PROA
ATOM	1647	HE2	TYR	P	103	-0.151	-19.265	17.256	0.00	0.00	PROA
ATOM	1648	C	TYR	P	103	6.428	-19.949	15.128	0.00	0.00	PROA
ATOM	1649	O	TYR	P	103	6.857	-21.060	14.964	0.00	0.00	PROA
ATOM	1650	N	PHE	P	104	6.940	-18.922	14.392	0.00	0.00	PROA
ATOM	1651	HN	PHE	P	104	6.570	-17.998	14.443	0.00	0.00	PROA
ATOM	1652	CA	PHE	P	104	7.762	-19.077	13.191	0.00	0.00	PROA
ATOM	1653	HA	PHE	P	104	7.952	-20.134	13.074	0.00	0.00	PROA
ATOM	1654	CB	PHE	P	104	7.159	-18.646	11.853	0.00	0.00	PROA
ATOM	1655	HB1	PHE	P	104	7.568	-19.134	10.943	0.00	0.00	PROA
ATOM	1656	HB2	PHE	P	104	7.215	-17.537	11.901	0.00	0.00	PROA
ATOM	1657	CG	PHE	P	104	5.683	-18.981	11.898	0.00	0.00	PROA
ATOM	1658	CD1	PHE	P	104	5.259	-20.283	11.871	0.00	0.00	PROA
ATOM	1659	HD1	PHE	P	104	5.901	-21.099	11.574	0.00	0.00	PROA
ATOM	1660	CE1	PHE	P	104	3.885	-20.632	12.003	0.00	0.00	PROA
ATOM	1661	HE1	PHE	P	104	3.641	-21.680	11.918	0.00	0.00	PROA
ATOM	1662	CZ	PHE	P	104	2.940	-19.628	12.063	0.00	0.00	PROA
ATOM	1663	HZ	PHE	P	104	1.878	-19.783	12.187	0.00	0.00	PROA
ATOM	1664	CD2	PHE	P	104	4.720	-18.005	12.059	0.00	0.00	PROA
ATOM	1665	HD2	PHE	P	104	5.048	-16.976	12.030	0.00	0.00	PROA
ATOM	1666	CE2	PHE	P	104	3.373	-18.276	12.128	0.00	0.00	PROA
ATOM	1667	HE2	PHE	P	104	2.656	-17.496	12.341	0.00	0.00	PROA
ATOM	1668	C	PHE	P	104	9.219	-18.596	13.336	0.00	0.00	PROA
ATOM	1669	O	PHE	P	104	9.893	-18.427	12.299	0.00	0.00	PROA
ATOM	1670	N	GLY	P	105	9.692	-18.383	14.550	0.00	0.00	PROA
ATOM	1671	HN	GLY	P	105	9.183	-18.481	15.401	0.00	0.00	PROA
ATOM	1672	CA	GLY	P	105	11.079	-17.935	14.682	0.00	0.00	PROA
ATOM	1673	HA1	GLY	P	105	11.675	-18.678	14.173	0.00	0.00	PROA
ATOM	1674	HA2	GLY	P	105	11.316	-17.873	15.734	0.00	0.00	PROA
ATOM	1675	C	GLY	P	105	11.420	-16.502	14.164	0.00	0.00	PROA
ATOM	1676	O	GLY	P	105	10.649	-15.671	13.590	0.00	0.00	PROA
ATOM	1677	N	ASN	P	106	12.645	-16.074	14.433	0.00	0.00	PROA
ATOM	1678	HN	ASN	P	106	13.227	-16.666	14.985	0.00	0.00	PROA
ATOM	1679	CA	ASN	P	106	13.141	-14.721	14.199	0.00	0.00	PROA
ATOM	1680	HA	ASN	P	106	12.338	-14.055	14.476	0.00	0.00	PROA
ATOM	1681	CB	ASN	P	106	14.425	-14.612	15.132	0.00	0.00	PROA
ATOM	1682	HB1	ASN	P	106	15.228	-15.308	14.808	0.00	0.00	PROA
ATOM	1683	HB2	ASN	P	106	14.180	-14.901	16.177	0.00	0.00	PROA
ATOM	1684	CG	ASN	P	106	14.754	-13.133	15.237	0.00	0.00	PROA
ATOM	1685	OD1	ASN	P	106	14.110	-12.401	15.988	0.00	0.00	PROA
ATOM	1686	ND2	ASN	P	106	15.715	-12.652	14.388	0.00	0.00	PROA
ATOM	1687	HD21	ASN	P	106	15.968	-11.694	14.523	0.00	0.00	PROA
ATOM	1688	HD22	ASN	P	106	16.399	-13.251	13.971	0.00	0.00	PROA
ATOM	1689	C	ASN	P	106	13.536	-14.371	12.773	0.00	0.00	PROA
ATOM	1690	O	ASN	P	106	13.240	-13.214	12.303	0.00	0.00	PROA
ATOM	1691	N	PHE	P	107	14.143	-15.245	11.917	0.00	0.00	PROA
ATOM	1692	HN	PHE	P	107	14.444	-16.111	12.308	0.00	0.00	PROA
ATOM	1693	CA	PHE	P	107	14.507	-14.924	10.538	0.00	0.00	PROA
ATOM	1694	HA	PHE	P	107	15.079	-14.013	10.630	0.00	0.00	PROA
ATOM	1695	CB	PHE	P	107	15.439	-15.969	9.813	0.00	0.00	PROA
ATOM	1696	HB1	PHE	P	107	14.846	-16.908	9.776	0.00	0.00	PROA
ATOM	1697	HB2	PHE	P	107	16.293	-16.016	10.522	0.00	0.00	PROA
ATOM	1698	CG	PHE	P	107	15.864	-15.685	8.399	0.00	0.00	PROA
ATOM	1699	CD1	PHE	P	107	15.688	-16.727	7.449	0.00	0.00	PROA
ATOM	1700	HD1	PHE	P	107	15.221	-17.680	7.649	0.00	0.00	PROA
ATOM	1701	CE1	PHE	P	107	16.086	-16.597	6.046	0.00	0.00	PROA
ATOM	1702	HE1	PHE	P	107	16.026	-17.463	5.403	0.00	0.00	PROA
ATOM	1703	CZ	PHE	P	107	16.892	-15.521	5.717	0.00	0.00	PROA

ATOM 1704 HZ PHE P 107 17.342 -15.394 4.744 0.00 0.00 PROA
ATOM 1705 CD2 PHE P 107 16.742 -14.667 8.026 0.00 0.00 PROA
ATOM 1706 HD2 PHE P 107 17.102 -13.968 8.766 0.00 0.00 PROA
ATOM 1707 CE2 PHE P 107 17.274 -14.580 6.717 0.00 0.00 PROA
ATOM 1708 HE2 PHE P 107 18.004 -13.810 6.516 0.00 0.00 PROA
ATOM 1709 C PHE P 107 13.370 -14.552 9.616 0.00 0.00 PROA
ATOM 1710 O PHE P 107 13.386 -13.563 8.872 0.00 0.00 PROA
ATOM 1711 N LEU P 108 12.231 -15.372 9.670 0.00 0.00 PROA
ATOM 1712 HN LEU P 108 12.498 -16.216 10.128 0.00 0.00 PROA
ATOM 1713 CA LEU P 108 11.007 -15.065 8.983 0.00 0.00 PROA
ATOM 1714 HA LEU P 108 11.302 -14.827 7.972 0.00 0.00 PROA
ATOM 1715 CB LEU P 108 10.043 -16.263 9.140 0.00 0.00 PROA
ATOM 1716 HB1 LEU P 108 9.718 -16.325 10.201 0.00 0.00 PROA
ATOM 1717 HB2 LEU P 108 10.640 -17.153 8.848 0.00 0.00 PROA
ATOM 1718 CG LEU P 108 8.868 -16.215 8.196 0.00 0.00 PROA
ATOM 1719 HG LEU P 108 8.302 -15.315 8.518 0.00 0.00 PROA
ATOM 1720 CD1 LEU P 108 9.206 -16.219 6.662 0.00 0.00 PROA
ATOM 1721 HD11 LEU P 108 8.422 -16.455 5.911 0.00 0.00 PROA
ATOM 1722 HD12 LEU P 108 10.063 -16.918 6.561 0.00 0.00 PROA
ATOM 1723 HD13 LEU P 108 9.599 -15.208 6.419 0.00 0.00 PROA
ATOM 1724 CD2 LEU P 108 7.949 -17.437 8.442 0.00 0.00 PROA
ATOM 1725 HD21 LEU P 108 8.425 -18.441 8.450 0.00 0.00 PROA
ATOM 1726 HD22 LEU P 108 7.146 -17.338 7.681 0.00 0.00 PROA
ATOM 1727 HD23 LEU P 108 7.394 -17.401 9.403 0.00 0.00 PROA
ATOM 1728 C LEU P 108 10.376 -13.769 9.558 0.00 0.00 PROA
ATOM 1729 O LEU P 108 9.767 -13.086 8.720 0.00 0.00 PROA
ATOM 1730 N CYS P 109 10.618 -13.421 10.865 0.00 0.00 PROA
ATOM 1731 HN CYS P 109 11.326 -13.821 11.442 0.00 0.00 PROA
ATOM 1732 CA CYS P 109 10.031 -12.246 11.396 0.00 0.00 PROA
ATOM 1733 HA CYS P 109 9.015 -12.272 11.031 0.00 0.00 PROA
ATOM 1734 CB CYS P 109 10.147 -12.208 12.937 0.00 0.00 PROA
ATOM 1735 HB1 CYS P 109 11.197 -12.077 13.276 0.00 0.00 PROA
ATOM 1736 HB2 CYS P 109 9.745 -13.120 13.428 0.00 0.00 PROA
ATOM 1737 SG CYS P 109 9.245 -10.815 13.636 0.00 0.00 PROA
ATOM 1738 C CYS P 109 10.690 -10.964 10.797 0.00 0.00 PROA
ATOM 1739 O CYS P 109 10.036 -9.904 10.557 0.00 0.00 PROA
ATOM 1740 N LYS P 110 11.957 -11.028 10.357 0.00 0.00 PROA
ATOM 1741 HN LYS P 110 12.433 -11.810 10.752 0.00 0.00 PROA
ATOM 1742 CA LYS P 110 12.662 -10.083 9.493 0.00 0.00 PROA
ATOM 1743 HA LYS P 110 12.477 -9.090 9.874 0.00 0.00 PROA
ATOM 1744 CB LYS P 110 14.241 -10.239 9.438 0.00 0.00 PROA
ATOM 1745 HB1 LYS P 110 14.536 -9.324 8.881 0.00 0.00 PROA
ATOM 1746 HB2 LYS P 110 14.502 -11.088 8.770 0.00 0.00 PROA
ATOM 1747 CG LYS P 110 14.857 -10.156 10.851 0.00 0.00 PROA
ATOM 1748 HG1 LYS P 110 14.700 -11.078 11.450 0.00 0.00 PROA
ATOM 1749 HG2 LYS P 110 14.314 -9.358 11.400 0.00 0.00 PROA
ATOM 1750 CD LYS P 110 16.271 -9.796 10.926 0.00 0.00 PROA
ATOM 1751 HD1 LYS P 110 16.430 -8.902 10.286 0.00 0.00 PROA
ATOM 1752 HD2 LYS P 110 16.878 -10.638 10.528 0.00 0.00 PROA
ATOM 1753 CE LYS P 110 17.010 -9.586 12.273 0.00 0.00 PROA
ATOM 1754 HE1 LYS P 110 18.095 -9.500 12.053 0.00 0.00 PROA
ATOM 1755 HE2 LYS P 110 16.777 -10.437 12.949 0.00 0.00 PROA
ATOM 1756 NZ LYS P 110 16.513 -8.312 12.894 0.00 0.00 PROA
ATOM 1757 HZ1 LYS P 110 15.505 -8.358 13.145 0.00 0.00 PROA
ATOM 1758 HZ2 LYS P 110 16.745 -7.534 12.244 0.00 0.00 PROA
ATOM 1759 HZ3 LYS P 110 17.122 -8.082 13.706 0.00 0.00 PROA
ATOM 1760 C LYS P 110 12.109 -10.091 8.041 0.00 0.00 PROA
ATOM 1761 O LYS P 110 11.688 -9.048 7.462 0.00 0.00 PROA
ATOM 1762 N ALA P 111 12.009 -11.301 7.408 0.00 0.00 PROA
ATOM 1763 HN ALA P 111 12.250 -12.126 7.914 0.00 0.00 PROA
ATOM 1764 CA ALA P 111 11.626 -11.518 5.999 0.00 0.00 PROA

ATOM 1765 HA ALA P 111 12.246 -10.791 5.495 0.00 0.00 PROA
ATOM 1766 CB ALA P 111 11.987 -12.978 5.565 0.00 0.00 PROA
ATOM 1767 HB1 ALA P 111 13.003 -13.109 5.996 0.00 0.00 PROA
ATOM 1768 HB2 ALA P 111 11.991 -13.159 4.469 0.00 0.00 PROA
ATOM 1769 HB3 ALA P 111 11.344 -13.649 6.173 0.00 0.00 PROA
ATOM 1770 C ALA P 111 10.161 -11.112 5.721 0.00 0.00 PROA
ATOM 1771 O ALA P 111 9.909 -10.447 4.720 0.00 0.00 PROA
ATOM 1772 N VAL P 112 9.087 -11.440 6.486 0.00 0.00 PROA
ATOM 1773 HN VAL P 112 9.200 -11.934 7.344 0.00 0.00 PROA
ATOM 1774 CA VAL P 112 7.676 -11.052 6.213 0.00 0.00 PROA
ATOM 1775 HA VAL P 112 7.481 -11.498 5.249 0.00 0.00 PROA
ATOM 1776 CB VAL P 112 6.701 -11.688 7.275 0.00 0.00 PROA
ATOM 1777 HB VAL P 112 5.662 -11.486 6.936 0.00 0.00 PROA
ATOM 1778 CG1 VAL P 112 6.798 -13.238 7.074 0.00 0.00 PROA
ATOM 1779 HG11 VAL P 112 5.909 -13.802 7.428 0.00 0.00 PROA
ATOM 1780 HG12 VAL P 112 7.688 -13.661 7.587 0.00 0.00 PROA
ATOM 1781 HG13 VAL P 112 6.880 -13.502 5.998 0.00 0.00 PROA
ATOM 1782 CG2 VAL P 112 6.875 -11.272 8.753 0.00 0.00 PROA
ATOM 1783 HG21 VAL P 112 6.087 -11.782 9.347 0.00 0.00 PROA
ATOM 1784 HG22 VAL P 112 6.885 -10.169 8.885 0.00 0.00 PROA
ATOM 1785 HG23 VAL P 112 7.854 -11.605 9.159 0.00 0.00 PROA
ATOM 1786 C VAL P 112 7.471 -9.547 6.083 0.00 0.00 PROA
ATOM 1787 O VAL P 112 6.817 -9.133 5.168 0.00 0.00 PROA
ATOM 1788 N HSD P 113 8.047 -8.650 7.019 0.00 0.00 PROA
ATOM 1789 HN HSD P 113 8.592 -9.038 7.758 0.00 0.00 PROA
ATOM 1790 CA HSD P 113 8.029 -7.215 6.939 0.00 0.00 PROA
ATOM 1791 HA HSD P 113 6.999 -6.905 7.033 0.00 0.00 PROA
ATOM 1792 CB HSD P 113 8.570 -6.619 8.264 0.00 0.00 PROA
ATOM 1793 HB1 HSD P 113 8.745 -5.551 8.014 0.00 0.00 PROA
ATOM 1794 HB2 HSD P 113 9.579 -7.022 8.492 0.00 0.00 PROA
ATOM 1795 ND1 HSD P 113 8.089 -7.706 10.539 0.00 0.00 PROA
ATOM 1796 HD1 HSD P 113 8.884 -8.312 10.509 0.00 0.00 PROA
ATOM 1797 CG HSD P 113 7.760 -6.807 9.558 0.00 0.00 PROA
ATOM 1798 CE1 HSD P 113 7.125 -7.639 11.421 0.00 0.00 PROA
ATOM 1799 HE1 HSD P 113 7.056 -8.379 12.218 0.00 0.00 PROA
ATOM 1800 NE2 HSD P 113 6.234 -6.705 11.176 0.00 0.00 PROA
ATOM 1801 CD2 HSD P 113 6.633 -6.205 9.975 0.00 0.00 PROA
ATOM 1802 HD2 HSD P 113 5.947 -5.556 9.445 0.00 0.00 PROA
ATOM 1803 C HSD P 113 8.475 -6.589 5.623 0.00 0.00 PROA
ATOM 1804 O HSD P 113 7.825 -5.689 5.007 0.00 0.00 PROA
ATOM 1805 N VAL P 114 9.562 -7.154 5.045 0.00 0.00 PROA
ATOM 1806 HN VAL P 114 10.098 -7.826 5.551 0.00 0.00 PROA
ATOM 1807 CA VAL P 114 10.053 -6.908 3.705 0.00 0.00 PROA
ATOM 1808 HA VAL P 114 10.418 -5.893 3.647 0.00 0.00 PROA
ATOM 1809 CB VAL P 114 11.274 -7.752 3.548 0.00 0.00 PROA
ATOM 1810 HB VAL P 114 11.023 -8.834 3.528 0.00 0.00 PROA
ATOM 1811 CG1 VAL P 114 11.854 -7.563 2.141 0.00 0.00 PROA
ATOM 1812 HG11 VAL P 114 11.206 -7.989 1.345 0.00 0.00 PROA
ATOM 1813 HG12 VAL P 114 12.849 -8.056 2.102 0.00 0.00 PROA
ATOM 1814 HG13 VAL P 114 11.950 -6.458 2.080 0.00 0.00 PROA
ATOM 1815 CG2 VAL P 114 12.324 -7.594 4.675 0.00 0.00 PROA
ATOM 1816 HG21 VAL P 114 13.180 -8.265 4.448 0.00 0.00 PROA
ATOM 1817 HG22 VAL P 114 11.834 -7.889 5.628 0.00 0.00 PROA
ATOM 1818 HG23 VAL P 114 12.784 -6.586 4.593 0.00 0.00 PROA
ATOM 1819 C VAL P 114 9.021 -7.115 2.566 0.00 0.00 PROA
ATOM 1820 O VAL P 114 8.739 -6.329 1.732 0.00 0.00 PROA
ATOM 1821 N ILE P 115 8.266 -8.261 2.612 0.00 0.00 PROA
ATOM 1822 HN ILE P 115 8.348 -8.935 3.342 0.00 0.00 PROA
ATOM 1823 CA ILE P 115 7.208 -8.592 1.600 0.00 0.00 PROA
ATOM 1824 HA ILE P 115 7.684 -8.508 0.634 0.00 0.00 PROA
ATOM 1825 CB ILE P 115 6.574 -9.995 1.878 0.00 0.00 PROA

```
ATOM   1826  HB  ILE P 115       6.078 -10.053   2.870  0.00  0.00      PROA
ATOM   1827  CG2 ILE P 115       5.370 -10.256   0.926  0.00  0.00      PROA
ATOM   1828 HG21 ILE P 115       5.053 -11.301   0.721  0.00  0.00      PROA
ATOM   1829 HG22 ILE P 115       5.778  -9.831  -0.016  0.00  0.00      PROA
ATOM   1830 HG23 ILE P 115       4.463  -9.684   1.218  0.00  0.00      PROA
ATOM   1831  CG1 ILE P 115       7.674 -11.114   1.730  0.00  0.00      PROA
ATOM   1832 HG11 ILE P 115       7.221 -12.080   1.422  0.00  0.00      PROA
ATOM   1833 HG12 ILE P 115       8.197 -11.378   2.674  0.00  0.00      PROA
ATOM   1834  CD  ILE P 115       8.793 -10.870   0.651  0.00  0.00      PROA
ATOM   1835  HD1 ILE P 115       9.395 -11.767   0.908  0.00  0.00      PROA
ATOM   1836  HD2 ILE P 115       9.469  -9.991   0.721  0.00  0.00      PROA
ATOM   1837  HD3 ILE P 115       8.481 -10.869  -0.415  0.00  0.00      PROA
ATOM   1838  C   ILE P 115       6.121  -7.556   1.639  0.00  0.00      PROA
ATOM   1839  O   ILE P 115       5.706  -7.074   0.600  0.00  0.00      PROA
ATOM   1840  N   TYR P 116       5.649  -7.141   2.830  0.00  0.00      PROA
ATOM   1841  HN  TYR P 116       5.975  -7.529   3.689  0.00  0.00      PROA
ATOM   1842  CA  TYR P 116       4.726  -6.086   2.965  0.00  0.00      PROA
ATOM   1843  HA  TYR P 116       3.833  -6.342   2.413  0.00  0.00      PROA
ATOM   1844  CB  TYR P 116       4.335  -5.929   4.507  0.00  0.00      PROA
ATOM   1845  HB1 TYR P 116       5.334  -5.704   4.938  0.00  0.00      PROA
ATOM   1846  HB2 TYR P 116       3.778  -6.839   4.816  0.00  0.00      PROA
ATOM   1847  CG  TYR P 116       3.567  -4.694   4.810  0.00  0.00      PROA
ATOM   1848  CD1 TYR P 116       4.132  -3.739   5.719  0.00  0.00      PROA
ATOM   1849  HD1 TYR P 116       5.099  -3.888   6.176  0.00  0.00      PROA
ATOM   1850  CE1 TYR P 116       3.505  -2.528   5.864  0.00  0.00      PROA
ATOM   1851  HE1 TYR P 116       4.015  -1.794   6.472  0.00  0.00      PROA
ATOM   1852  CZ  TYR P 116       2.321  -2.290   5.273  0.00  0.00      PROA
ATOM   1853  OH  TYR P 116       1.702  -1.064   5.411  0.00  0.00      PROA
ATOM   1854  HH  TYR P 116       0.827  -1.099   5.018  0.00  0.00      PROA
ATOM   1855  CD2 TYR P 116       2.394  -4.404   4.103  0.00  0.00      PROA
ATOM   1856  HD2 TYR P 116       2.152  -5.069   3.287  0.00  0.00      PROA
ATOM   1857  CE2 TYR P 116       1.736  -3.176   4.350  0.00  0.00      PROA
ATOM   1858  HE2 TYR P 116       0.806  -2.992   3.833  0.00  0.00      PROA
ATOM   1859  C   TYR P 116       5.198  -4.704   2.405  0.00  0.00      PROA
ATOM   1860  O   TYR P 116       4.585  -4.087   1.567  0.00  0.00      PROA
ATOM   1861  N   THR P 117       6.397  -4.237   2.782  0.00  0.00      PROA
ATOM   1862  HN  THR P 117       7.017  -4.690   3.418  0.00  0.00      PROA
ATOM   1863  CA  THR P 117       6.945  -2.964   2.389  0.00  0.00      PROA
ATOM   1864  HA  THR P 117       6.319  -2.122   2.644  0.00  0.00      PROA
ATOM   1865  CB  THR P 117       8.204  -2.742   3.213  0.00  0.00      PROA
ATOM   1866  HB  THR P 117       9.046  -3.456   3.091  0.00  0.00      PROA
ATOM   1867  OG1 THR P 117       7.826  -2.747   4.551  0.00  0.00      PROA
ATOM   1868  HG1 THR P 117       8.666  -2.975   4.955  0.00  0.00      PROA
ATOM   1869  CG2 THR P 117       8.660  -1.328   2.860  0.00  0.00      PROA
ATOM   1870 HG21 THR P 117       9.182  -1.377   1.881  0.00  0.00      PROA
ATOM   1871 HG22 THR P 117       9.419  -1.176   3.657  0.00  0.00      PROA
ATOM   1872 HG23 THR P 117       7.847  -0.571   2.864  0.00  0.00      PROA
ATOM   1873  C   THR P 117       7.262  -2.853   1.020  0.00  0.00      PROA
ATOM   1874  O   THR P 117       7.010  -1.881   0.277  0.00  0.00      PROA
ATOM   1875  N   VAL P 118       7.845  -3.907   0.481  0.00  0.00      PROA
ATOM   1876  HN  VAL P 118       8.005  -4.742   1.002  0.00  0.00      PROA
ATOM   1877  CA  VAL P 118       8.192  -3.994  -0.899  0.00  0.00      PROA
ATOM   1878  HA  VAL P 118       8.620  -3.048  -1.194  0.00  0.00      PROA
ATOM   1879  CB  VAL P 118       9.228  -4.960  -1.331  0.00  0.00      PROA
ATOM   1880  HB  VAL P 118       9.001  -6.012  -1.053  0.00  0.00      PROA
ATOM   1881  CG1 VAL P 118       9.354  -4.912  -2.890  0.00  0.00      PROA
ATOM   1882 HG11 VAL P 118       8.460  -5.383  -3.352  0.00  0.00      PROA
ATOM   1883 HG12 VAL P 118      10.175  -5.570  -3.246  0.00  0.00      PROA
ATOM   1884 HG13 VAL P 118       9.490  -3.905  -3.338  0.00  0.00      PROA
ATOM   1885  CG2 VAL P 118      10.600  -4.593  -0.760  0.00  0.00      PROA
ATOM   1886 HG21 VAL P 118      11.324  -5.411  -0.962  0.00  0.00      PROA
```

```
ATOM   1887 HG22 VAL P 118      10.495  -4.377   0.324  0.00  0.00      PROA
ATOM   1888 HG23 VAL P 118      10.935  -3.583  -1.079  0.00  0.00      PROA
ATOM   1889  C   VAL P 118       6.937  -3.957  -1.795  0.00  0.00      PROA
ATOM   1890  O   VAL P 118       6.826  -3.199  -2.749  0.00  0.00      PROA
ATOM   1891  N   ASN P 119       5.971  -4.775  -1.444  0.00  0.00      PROA
ATOM   1892  HN  ASN P 119       5.993  -5.407  -0.673  0.00  0.00      PROA
ATOM   1893  CA  ASN P 119       4.714  -4.716  -2.172  0.00  0.00      PROA
ATOM   1894  HA  ASN P 119       4.906  -4.863  -3.225  0.00  0.00      PROA
ATOM   1895  CB  ASN P 119       3.759  -5.903  -1.723  0.00  0.00      PROA
ATOM   1896  HB1 ASN P 119       3.551  -5.669  -0.658  0.00  0.00      PROA
ATOM   1897  HB2 ASN P 119       4.271  -6.887  -1.780  0.00  0.00      PROA
ATOM   1898  CG  ASN P 119       2.435  -6.243  -2.342  0.00  0.00      PROA
ATOM   1899  OD1 ASN P 119       1.451  -6.420  -1.592  0.00  0.00      PROA
ATOM   1900  ND2 ASN P 119       2.361  -6.219  -3.706  0.00  0.00      PROA
ATOM   1901 HD21 ASN P 119       1.442  -6.469  -4.012  0.00  0.00      PROA
ATOM   1902 HD22 ASN P 119       3.102  -5.978  -4.333  0.00  0.00      PROA
ATOM   1903  C   ASN P 119       3.922  -3.394  -2.102  0.00  0.00      PROA
ATOM   1904  O   ASN P 119       3.357  -2.922  -3.115  0.00  0.00      PROA
ATOM   1905  N   LEU P 120       3.830  -2.673  -0.966  0.00  0.00      PROA
ATOM   1906  HN  LEU P 120       4.058  -2.997  -0.051  0.00  0.00      PROA
ATOM   1907  CA  LEU P 120       3.223  -1.294  -0.950  0.00  0.00      PROA
ATOM   1908  HA  LEU P 120       2.205  -1.369  -1.305  0.00  0.00      PROA
ATOM   1909  CB  LEU P 120       3.122  -0.783   0.561  0.00  0.00      PROA
ATOM   1910  HB1 LEU P 120       4.188  -0.763   0.876  0.00  0.00      PROA
ATOM   1911  HB2 LEU P 120       2.564  -1.534   1.160  0.00  0.00      PROA
ATOM   1912  CG  LEU P 120       2.386   0.547   0.720  0.00  0.00      PROA
ATOM   1913  HG  LEU P 120       2.750   1.221  -0.085  0.00  0.00      PROA
ATOM   1914  CD1 LEU P 120       0.836   0.268   0.630  0.00  0.00      PROA
ATOM   1915 HD11 LEU P 120       0.591  -0.526   1.367  0.00  0.00      PROA
ATOM   1916 HD12 LEU P 120       0.552  -0.007  -0.409  0.00  0.00      PROA
ATOM   1917 HD13 LEU P 120       0.223   1.169   0.845  0.00  0.00      PROA
ATOM   1918  CD2 LEU P 120       2.788   1.151   2.089  0.00  0.00      PROA
ATOM   1919 HD21 LEU P 120       2.162   2.016   2.395  0.00  0.00      PROA
ATOM   1920 HD22 LEU P 120       3.879   1.336   2.186  0.00  0.00      PROA
ATOM   1921 HD23 LEU P 120       2.460   0.369   2.807  0.00  0.00      PROA
ATOM   1922  C   LEU P 120       3.937  -0.248  -1.768  0.00  0.00      PROA
ATOM   1923  O   LEU P 120       3.297   0.581  -2.441  0.00  0.00      PROA
ATOM   1924  N   TYR P 121       5.282  -0.126  -1.671  0.00  0.00      PROA
ATOM   1925  HN  TYR P 121       5.797  -0.787  -1.131  0.00  0.00      PROA
ATOM   1926  CA  TYR P 121       6.092   0.862  -2.392  0.00  0.00      PROA
ATOM   1927  HA  TYR P 121       5.498   1.758  -2.485  0.00  0.00      PROA
ATOM   1928  CB  TYR P 121       7.475   1.197  -1.674  0.00  0.00      PROA
ATOM   1929  HB1 TYR P 121       8.090   1.858  -2.321  0.00  0.00      PROA
ATOM   1930  HB2 TYR P 121       8.030   0.235  -1.647  0.00  0.00      PROA
ATOM   1931  CG  TYR P 121       7.310   1.947  -0.357  0.00  0.00      PROA
ATOM   1932  CD1 TYR P 121       8.441   2.049   0.505  0.00  0.00      PROA
ATOM   1933  HD1 TYR P 121       9.325   1.507   0.203  0.00  0.00      PROA
ATOM   1934  CE1 TYR P 121       8.438   2.666   1.769  0.00  0.00      PROA
ATOM   1935  HE1 TYR P 121       9.277   2.643   2.448  0.00  0.00      PROA
ATOM   1936  CZ  TYR P 121       7.237   3.290   2.214  0.00  0.00      PROA
ATOM   1937  OH  TYR P 121       7.213   3.916   3.450  0.00  0.00      PROA
ATOM   1938  HH  TYR P 121       8.012   3.648   3.911  0.00  0.00      PROA
ATOM   1939  CD2 TYR P 121       6.182   2.688   0.068  0.00  0.00      PROA
ATOM   1940  HD2 TYR P 121       5.265   2.786  -0.494  0.00  0.00      PROA
ATOM   1941  CE2 TYR P 121       6.188   3.286   1.326  0.00  0.00      PROA
ATOM   1942  HE2 TYR P 121       5.276   3.782   1.623  0.00  0.00      PROA
ATOM   1943  C   TYR P 121       6.372   0.575  -3.868  0.00  0.00      PROA
ATOM   1944  O   TYR P 121       6.377   1.487  -4.704  0.00  0.00      PROA
ATOM   1945  N   SER P 122       6.531  -0.680  -4.308  0.00  0.00      PROA
ATOM   1946  HN  SER P 122       6.619  -1.451  -3.681  0.00  0.00      PROA
ATOM   1947  CA  SER P 122       6.674  -1.124  -5.680  0.00  0.00      PROA
```

ATOM	1948	HA	SER	P	122	7.425	-0.523	-6.170	0.00	0.00	PROA
ATOM	1949	CB	SER	P	122	7.059	-2.603	-5.799	0.00	0.00	PROA
ATOM	1950	HB1	SER	P	122	7.980	-2.801	-5.210	0.00	0.00	PROA
ATOM	1951	HB2	SER	P	122	7.215	-2.952	-6.842	0.00	0.00	PROA
ATOM	1952	OG	SER	P	122	6.067	-3.475	-5.293	0.00	0.00	PROA
ATOM	1953	HG1	SER	P	122	6.185	-3.382	-4.345	0.00	0.00	PROA
ATOM	1954	C	SER	P	122	5.346	-0.914	-6.368	0.00	0.00	PROA
ATOM	1955	O	SER	P	122	5.329	-0.506	-7.556	0.00	0.00	PROA
ATOM	1956	N	SER	P	123	4.224	-0.989	-5.604	0.00	0.00	PROA
ATOM	1957	HN	SER	P	123	4.391	-1.470	-4.746	0.00	0.00	PROA
ATOM	1958	CA	SER	P	123	2.950	-0.880	-6.206	0.00	0.00	PROA
ATOM	1959	HA	SER	P	123	3.028	-1.444	-7.123	0.00	0.00	PROA
ATOM	1960	CB	SER	P	123	1.728	-1.449	-5.293	0.00	0.00	PROA
ATOM	1961	HB1	SER	P	123	0.714	-1.203	-5.674	0.00	0.00	PROA
ATOM	1962	HB2	SER	P	123	1.789	-1.010	-4.274	0.00	0.00	PROA
ATOM	1963	OG	SER	P	123	1.711	-2.880	-5.234	0.00	0.00	PROA
ATOM	1964	HG1	SER	P	123	2.223	-3.094	-4.451	0.00	0.00	PROA
ATOM	1965	C	SER	P	123	2.598	0.481	-6.720	0.00	0.00	PROA
ATOM	1966	O	SER	P	123	2.060	0.686	-7.815	0.00	0.00	PROA
ATOM	1967	N	VAL	P	124	2.859	1.484	-5.892	0.00	0.00	PROA
ATOM	1968	HN	VAL	P	124	3.326	1.320	-5.027	0.00	0.00	PROA
ATOM	1969	CA	VAL	P	124	2.809	2.876	-6.289	0.00	0.00	PROA
ATOM	1970	HA	VAL	P	124	1.839	3.046	-6.733	0.00	0.00	PROA
ATOM	1971	CB	VAL	P	124	2.875	3.902	-5.170	0.00	0.00	PROA
ATOM	1972	HB	VAL	P	124	2.966	4.864	-5.718	0.00	0.00	PROA
ATOM	1973	CG1	VAL	P	124	1.508	3.855	-4.477	0.00	0.00	PROA
ATOM	1974	HG11	VAL	P	124	1.484	4.618	-3.670	0.00	0.00	PROA
ATOM	1975	HG12	VAL	P	124	1.450	2.782	-4.194	0.00	0.00	PROA
ATOM	1976	HG13	VAL	P	124	0.735	4.008	-5.260	0.00	0.00	PROA
ATOM	1977	CG2	VAL	P	124	4.086	3.744	-4.321	0.00	0.00	PROA
ATOM	1978	HG21	VAL	P	124	4.999	3.672	-4.949	0.00	0.00	PROA
ATOM	1979	HG22	VAL	P	124	3.956	2.888	-3.625	0.00	0.00	PROA
ATOM	1980	HG23	VAL	P	124	4.244	4.538	-3.560	0.00	0.00	PROA
ATOM	1981	C	VAL	P	124	3.822	3.206	-7.427	0.00	0.00	PROA
ATOM	1982	O	VAL	P	124	3.458	3.766	-8.456	0.00	0.00	PROA
ATOM	1983	N	TRP	P	125	5.042	2.743	-7.283	0.00	0.00	PROA
ATOM	1984	HN	TRP	P	125	5.205	2.223	-6.448	0.00	0.00	PROA
ATOM	1985	CA	TRP	P	125	6.103	2.962	-8.259	0.00	0.00	PROA
ATOM	1986	HA	TRP	P	125	6.031	4.030	-8.398	0.00	0.00	PROA
ATOM	1987	CB	TRP	P	125	7.502	2.803	-7.668	0.00	0.00	PROA
ATOM	1988	HB1	TRP	P	125	8.327	2.759	-8.411	0.00	0.00	PROA
ATOM	1989	HB2	TRP	P	125	7.487	1.810	-7.170	0.00	0.00	PROA
ATOM	1990	CG	TRP	P	125	7.980	3.913	-6.801	0.00	0.00	PROA
ATOM	1991	CD1	TRP	P	125	8.216	3.848	-5.468	0.00	0.00	PROA
ATOM	1992	HD1	TRP	P	125	8.043	3.031	-4.783	0.00	0.00	PROA
ATOM	1993	NE1	TRP	P	125	8.818	5.065	-5.109	0.00	0.00	PROA
ATOM	1994	HE1	TRP	P	125	8.795	5.415	-4.198	0.00	0.00	PROA
ATOM	1995	CE2	TRP	P	125	8.991	5.837	-6.204	0.00	0.00	PROA
ATOM	1996	CD2	TRP	P	125	8.507	5.115	-7.304	0.00	0.00	PROA
ATOM	1997	CE3	TRP	P	125	8.682	5.573	-8.583	0.00	0.00	PROA
ATOM	1998	HE3	TRP	P	125	8.486	4.900	-9.405	0.00	0.00	PROA
ATOM	1999	CZ3	TRP	P	125	9.212	6.828	-8.813	0.00	0.00	PROA
ATOM	2000	HZ3	TRP	P	125	9.377	7.195	-9.815	0.00	0.00	PROA
ATOM	2001	CZ2	TRP	P	125	9.537	7.100	-6.428	0.00	0.00	PROA
ATOM	2002	HZ2	TRP	P	125	10.008	7.685	-5.651	0.00	0.00	PROA
ATOM	2003	CH2	TRP	P	125	9.469	7.667	-7.701	0.00	0.00	PROA
ATOM	2004	HH2	TRP	P	125	9.634	8.695	-7.990	0.00	0.00	PROA
ATOM	2005	C	TRP	P	125	5.888	2.324	-9.665	0.00	0.00	PROA
ATOM	2006	O	TRP	P	125	6.147	2.913	-10.704	0.00	0.00	PROA
ATOM	2007	N	ILE	P	126	5.343	1.101	-9.717	0.00	0.00	PROA
ATOM	2008	HN	ILE	P	126	5.276	0.557	-8.884	0.00	0.00	PROA

ATOM 2009 CA ILE P 126 4.897 0.369 -10.893 0.00 0.00 PROA
ATOM 2010 HA ILE P 126 5.680 0.395 -11.637 0.00 0.00 PROA
ATOM 2011 CB ILE P 126 4.652 -1.062 -10.577 0.00 0.00 PROA
ATOM 2012 HB ILE P 126 3.929 -1.071 -9.734 0.00 0.00 PROA
ATOM 2013 CG2 ILE P 126 3.934 -1.681 -11.756 0.00 0.00 PROA
ATOM 2014 HG21 ILE P 126 4.371 -1.354 -12.724 0.00 0.00 PROA
ATOM 2015 HG22 ILE P 126 2.852 -1.429 -11.759 0.00 0.00 PROA
ATOM 2016 HG23 ILE P 126 4.002 -2.789 -11.806 0.00 0.00 PROA
ATOM 2017 CG1 ILE P 126 5.978 -1.789 -10.225 0.00 0.00 PROA
ATOM 2018 HG11 ILE P 126 5.869 -2.663 -9.548 0.00 0.00 PROA
ATOM 2019 HG12 ILE P 126 6.565 -1.035 -9.657 0.00 0.00 PROA
ATOM 2020 CD ILE P 126 6.785 -2.274 -11.396 0.00 0.00 PROA
ATOM 2021 HD1 ILE P 126 6.309 -3.121 -11.935 0.00 0.00 PROA
ATOM 2022 HD2 ILE P 126 7.862 -2.294 -11.123 0.00 0.00 PROA
ATOM 2023 HD3 ILE P 126 6.808 -1.529 -12.219 0.00 0.00 PROA
ATOM 2024 C ILE P 126 3.685 1.138 -11.540 0.00 0.00 PROA
ATOM 2025 O ILE P 126 3.700 1.356 -12.755 0.00 0.00 PROA
ATOM 2026 N LEU P 127 2.756 1.665 -10.786 0.00 0.00 PROA
ATOM 2027 HN LEU P 127 2.811 1.660 -9.791 0.00 0.00 PROA
ATOM 2028 CA LEU P 127 1.723 2.528 -11.349 0.00 0.00 PROA
ATOM 2029 HA LEU P 127 1.285 1.905 -12.115 0.00 0.00 PROA
ATOM 2030 CB LEU P 127 0.593 2.582 -10.274 0.00 0.00 PROA
ATOM 2031 HB1 LEU P 127 0.933 2.982 -9.294 0.00 0.00 PROA
ATOM 2032 HB2 LEU P 127 0.265 1.533 -10.108 0.00 0.00 PROA
ATOM 2033 CG LEU P 127 -0.688 3.333 -10.749 0.00 0.00 PROA
ATOM 2034 HG LEU P 127 -0.351 4.379 -10.911 0.00 0.00 PROA
ATOM 2035 CD1 LEU P 127 -1.376 2.623 -11.979 0.00 0.00 PROA
ATOM 2036 HD11 LEU P 127 -0.884 2.817 -12.957 0.00 0.00 PROA
ATOM 2037 HD12 LEU P 127 -2.470 2.809 -12.023 0.00 0.00 PROA
ATOM 2038 HD13 LEU P 127 -1.235 1.521 -11.953 0.00 0.00 PROA
ATOM 2039 CD2 LEU P 127 -1.738 3.479 -9.611 0.00 0.00 PROA
ATOM 2040 HD21 LEU P 127 -1.995 2.538 -9.080 0.00 0.00 PROA
ATOM 2041 HD22 LEU P 127 -2.705 3.927 -9.923 0.00 0.00 PROA
ATOM 2042 HD23 LEU P 127 -1.424 4.174 -8.804 0.00 0.00 PROA
ATOM 2043 C LEU P 127 2.254 3.782 -11.968 0.00 0.00 PROA
ATOM 2044 O LEU P 127 1.853 4.158 -13.107 0.00 0.00 PROA
ATOM 2045 N ALA P 128 3.305 4.351 -11.280 0.00 0.00 PROA
ATOM 2046 HN ALA P 128 3.505 4.087 -10.340 0.00 0.00 PROA
ATOM 2047 CA ALA P 128 3.929 5.494 -11.818 0.00 0.00 PROA
ATOM 2048 HA ALA P 128 3.201 6.212 -12.165 0.00 0.00 PROA
ATOM 2049 CB ALA P 128 4.950 6.065 -10.878 0.00 0.00 PROA
ATOM 2050 HB1 ALA P 128 5.718 5.277 -10.726 0.00 0.00 PROA
ATOM 2051 HB2 ALA P 128 4.544 6.411 -9.904 0.00 0.00 PROA
ATOM 2052 HB3 ALA P 128 5.340 6.998 -11.337 0.00 0.00 PROA
ATOM 2053 C ALA P 128 4.660 5.213 -13.150 0.00 0.00 PROA
ATOM 2054 O ALA P 128 4.677 6.027 -14.083 0.00 0.00 PROA
ATOM 2055 N PHE P 129 5.327 4.019 -13.258 0.00 0.00 PROA
ATOM 2056 HN PHE P 129 5.362 3.368 -12.504 0.00 0.00 PROA
ATOM 2057 CA PHE P 129 5.909 3.549 -14.467 0.00 0.00 PROA
ATOM 2058 HA PHE P 129 6.483 4.375 -14.860 0.00 0.00 PROA
ATOM 2059 CB PHE P 129 6.904 2.442 -14.080 0.00 0.00 PROA
ATOM 2060 HB1 PHE P 129 7.029 1.665 -14.865 0.00 0.00 PROA
ATOM 2061 HB2 PHE P 129 6.482 1.900 -13.207 0.00 0.00 PROA
ATOM 2062 CG PHE P 129 8.295 3.002 -13.742 0.00 0.00 PROA
ATOM 2063 CD1 PHE P 129 8.433 3.894 -12.626 0.00 0.00 PROA
ATOM 2064 HD1 PHE P 129 7.562 4.203 -12.067 0.00 0.00 PROA
ATOM 2065 CE1 PHE P 129 9.681 4.437 -12.202 0.00 0.00 PROA
ATOM 2066 HE1 PHE P 129 9.678 5.115 -11.362 0.00 0.00 PROA
ATOM 2067 CZ PHE P 129 10.836 3.959 -12.750 0.00 0.00 PROA
ATOM 2068 HZ PHE P 129 11.824 4.321 -12.505 0.00 0.00 PROA
ATOM 2069 CD2 PHE P 129 9.504 2.639 -14.339 0.00 0.00 PROA

ATOM	2070	HD2	PHE	P	129	9.342	1.987	-15.184	0.00	0.00	PROA
ATOM	2071	CE2	PHE	P	129	10.720	3.035	-13.867	0.00	0.00	PROA
ATOM	2072	HE2	PHE	P	129	11.564	2.870	-14.519	0.00	0.00	PROA
ATOM	2073	C	PHE	P	129	4.964	3.123	-15.633	0.00	0.00	PROA
ATOM	2074	O	PHE	P	129	5.153	3.273	-16.822	0.00	0.00	PROA
ATOM	2075	N	ILE	P	130	3.816	2.526	-15.251	0.00	0.00	PROA
ATOM	2076	HN	ILE	P	130	3.664	2.365	-14.279	0.00	0.00	PROA
ATOM	2077	CA	ILE	P	130	2.693	2.199	-16.095	0.00	0.00	PROA
ATOM	2078	HA	ILE	P	130	3.047	1.524	-16.860	0.00	0.00	PROA
ATOM	2079	CB	ILE	P	130	1.487	1.454	-15.367	0.00	0.00	PROA
ATOM	2080	HB	ILE	P	130	1.516	1.901	-14.350	0.00	0.00	PROA
ATOM	2081	CG2	ILE	P	130	0.099	1.662	-15.969	0.00	0.00	PROA
ATOM	2082	HG21	ILE	P	130	0.147	1.203	-16.980	0.00	0.00	PROA
ATOM	2083	HG22	ILE	P	130	-0.213	2.728	-15.959	0.00	0.00	PROA
ATOM	2084	HG23	ILE	P	130	-0.643	1.137	-15.331	0.00	0.00	PROA
ATOM	2085	CG1	ILE	P	130	1.759	-0.081	-15.196	0.00	0.00	PROA
ATOM	2086	HG11	ILE	P	130	2.824	-0.366	-15.057	0.00	0.00	PROA
ATOM	2087	HG12	ILE	P	130	1.382	-0.472	-16.165	0.00	0.00	PROA
ATOM	2088	CD	ILE	P	130	0.881	-0.548	-14.083	0.00	0.00	PROA
ATOM	2089	HD1	ILE	P	130	0.217	-1.319	-14.530	0.00	0.00	PROA
ATOM	2090	HD2	ILE	P	130	0.347	0.208	-13.469	0.00	0.00	PROA
ATOM	2091	HD3	ILE	P	130	1.499	-1.100	-13.344	0.00	0.00	PROA
ATOM	2092	C	ILE	P	130	2.182	3.433	-16.795	0.00	0.00	PROA
ATOM	2093	O	ILE	P	130	2.050	3.507	-18.025	0.00	0.00	PROA
ATOM	2094	N	SER	P	131	2.004	4.504	-15.971	0.00	0.00	PROA
ATOM	2095	HN	SER	P	131	2.087	4.382	-14.985	0.00	0.00	PROA
ATOM	2096	CA	SER	P	131	1.641	5.846	-16.276	0.00	0.00	PROA
ATOM	2097	HA	SER	P	131	0.766	5.865	-16.909	0.00	0.00	PROA
ATOM	2098	CB	SER	P	131	1.622	6.840	-15.106	0.00	0.00	PROA
ATOM	2099	HB1	SER	P	131	1.064	7.761	-15.379	0.00	0.00	PROA
ATOM	2100	HB2	SER	P	131	2.629	7.170	-14.773	0.00	0.00	PROA
ATOM	2101	OG	SER	P	131	0.759	6.392	-14.049	0.00	0.00	PROA
ATOM	2102	HG1	SER	P	131	1.130	5.658	-13.555	0.00	0.00	PROA
ATOM	2103	C	SER	P	131	2.705	6.501	-17.210	0.00	0.00	PROA
ATOM	2104	O	SER	P	131	2.352	7.105	-18.225	0.00	0.00	PROA
ATOM	2105	N	LEU	P	132	3.984	6.362	-16.983	0.00	0.00	PROA
ATOM	2106	HN	LEU	P	132	4.304	6.013	-16.105	0.00	0.00	PROA
ATOM	2107	CA	LEU	P	132	5.004	6.768	-17.890	0.00	0.00	PROA
ATOM	2108	HA	LEU	P	132	4.897	7.839	-17.977	0.00	0.00	PROA
ATOM	2109	CB	LEU	P	132	6.448	6.361	-17.447	0.00	0.00	PROA
ATOM	2110	HB1	LEU	P	132	7.210	6.139	-18.225	0.00	0.00	PROA
ATOM	2111	HB2	LEU	P	132	6.315	5.410	-16.887	0.00	0.00	PROA
ATOM	2112	CG	LEU	P	132	7.048	7.528	-16.585	0.00	0.00	PROA
ATOM	2113	HG	LEU	P	132	6.169	8.056	-16.157	0.00	0.00	PROA
ATOM	2114	CD1	LEU	P	132	7.970	7.063	-15.466	0.00	0.00	PROA
ATOM	2115	HD11	LEU	P	132	7.419	6.465	-14.709	0.00	0.00	PROA
ATOM	2116	HD12	LEU	P	132	8.285	7.969	-14.904	0.00	0.00	PROA
ATOM	2117	HD13	LEU	P	132	8.862	6.522	-15.846	0.00	0.00	PROA
ATOM	2118	CD2	LEU	P	132	7.734	8.633	-17.353	0.00	0.00	PROA
ATOM	2119	HD21	LEU	P	132	8.643	8.308	-17.902	0.00	0.00	PROA
ATOM	2120	HD22	LEU	P	132	7.818	9.614	-16.837	0.00	0.00	PROA
ATOM	2121	HD23	LEU	P	132	7.137	8.854	-18.264	0.00	0.00	PROA
ATOM	2122	C	LEU	P	132	4.926	6.239	-19.288	0.00	0.00	PROA
ATOM	2123	O	LEU	P	132	4.987	6.965	-20.344	0.00	0.00	PROA
ATOM	2124	N	ASP	P	133	4.716	4.910	-19.411	0.00	0.00	PROA
ATOM	2125	HN	ASP	P	133	4.625	4.309	-18.621	0.00	0.00	PROA
ATOM	2126	CA	ASP	P	133	4.613	4.195	-20.622	0.00	0.00	PROA
ATOM	2127	HA	ASP	P	133	5.534	4.242	-21.184	0.00	0.00	PROA
ATOM	2128	CB	ASP	P	133	4.376	2.655	-20.273	0.00	0.00	PROA
ATOM	2129	HB1	ASP	P	133	3.315	2.460	-20.008	0.00	0.00	PROA
ATOM	2130	HB2	ASP	P	133	5.016	2.333	-19.424	0.00	0.00	PROA

ATOM	2131	CG	ASP	P	133	4.668	1.762	-21.427	0.00	0.00	PROA
ATOM	2132	OD1	ASP	P	133	5.806	1.785	-21.870	0.00	0.00	PROA
ATOM	2133	OD2	ASP	P	133	3.777	1.049	-21.997	0.00	0.00	PROA
ATOM	2134	C	ASP	P	133	3.435	4.722	-21.417	0.00	0.00	PROA
ATOM	2135	O	ASP	P	133	3.540	5.157	-22.564	0.00	0.00	PROA
ATOM	2136	N	ARG	P	134	2.236	4.889	-20.781	0.00	0.00	PROA
ATOM	2137	HN	ARG	P	134	2.021	4.758	-19.816	0.00	0.00	PROA
ATOM	2138	CA	ARG	P	134	1.113	5.378	-21.520	0.00	0.00	PROA
ATOM	2139	HA	ARG	P	134	1.026	4.671	-22.332	0.00	0.00	PROA
ATOM	2140	CB	ARG	P	134	-0.157	5.272	-20.597	0.00	0.00	PROA
ATOM	2141	HB1	ARG	P	134	-0.944	5.974	-20.945	0.00	0.00	PROA
ATOM	2142	HB2	ARG	P	134	0.142	5.685	-19.610	0.00	0.00	PROA
ATOM	2143	CG	ARG	P	134	-0.725	3.873	-20.384	0.00	0.00	PROA
ATOM	2144	HG1	ARG	P	134	-1.418	4.065	-19.538	0.00	0.00	PROA
ATOM	2145	HG2	ARG	P	134	0.056	3.161	-20.042	0.00	0.00	PROA
ATOM	2146	CD	ARG	P	134	-1.493	3.279	-21.599	0.00	0.00	PROA
ATOM	2147	HD1	ARG	P	134	-0.934	3.291	-22.559	0.00	0.00	PROA
ATOM	2148	HD2	ARG	P	134	-2.465	3.795	-21.753	0.00	0.00	PROA
ATOM	2149	NE	ARG	P	134	-1.731	1.861	-21.079	0.00	0.00	PROA
ATOM	2150	HE	ARG	P	134	-2.238	1.701	-20.232	0.00	0.00	PROA
ATOM	2151	CZ	ARG	P	134	-1.485	0.775	-21.759	0.00	0.00	PROA
ATOM	2152	NH1	ARG	P	134	-0.599	0.642	-22.737	0.00	0.00	PROA
ATOM	2153	HH11	ARG	P	134	-0.007	1.383	-23.054	0.00	0.00	PROA
ATOM	2154	HH12	ARG	P	134	-0.453	-0.294	-23.058	0.00	0.00	PROA
ATOM	2155	NH2	ARG	P	134	-2.209	-0.304	-21.463	0.00	0.00	PROA
ATOM	2156	HH21	ARG	P	134	-2.569	-0.303	-20.530	0.00	0.00	PROA
ATOM	2157	HH22	ARG	P	134	-1.909	-1.188	-21.821	0.00	0.00	PROA
ATOM	2158	C	ARG	P	134	1.205	6.712	-22.171	0.00	0.00	PROA
ATOM	2159	O	ARG	P	134	0.830	6.892	-23.311	0.00	0.00	PROA
ATOM	2160	N	TYR	P	135	1.893	7.600	-21.392	0.00	0.00	PROA
ATOM	2161	HN	TYR	P	135	2.060	7.417	-20.426	0.00	0.00	PROA
ATOM	2162	CA	TYR	P	135	2.304	8.894	-21.920	0.00	0.00	PROA
ATOM	2163	HA	TYR	P	135	1.458	9.415	-22.344	0.00	0.00	PROA
ATOM	2164	CB	TYR	P	135	2.663	9.825	-20.664	0.00	0.00	PROA
ATOM	2165	HB1	TYR	P	135	2.848	9.230	-19.744	0.00	0.00	PROA
ATOM	2166	HB2	TYR	P	135	1.715	10.331	-20.382	0.00	0.00	PROA
ATOM	2167	CG	TYR	P	135	3.885	10.771	-20.983	0.00	0.00	PROA
ATOM	2168	CD1	TYR	P	135	3.724	11.893	-21.761	0.00	0.00	PROA
ATOM	2169	HD1	TYR	P	135	2.728	12.226	-22.009	0.00	0.00	PROA
ATOM	2170	CE1	TYR	P	135	4.934	12.509	-22.258	0.00	0.00	PROA
ATOM	2171	HE1	TYR	P	135	4.875	13.438	-22.805	0.00	0.00	PROA
ATOM	2172	CZ	TYR	P	135	6.190	12.129	-21.894	0.00	0.00	PROA
ATOM	2173	OH	TYR	P	135	7.208	12.886	-22.392	0.00	0.00	PROA
ATOM	2174	HH	TYR	P	135	8.067	12.496	-22.217	0.00	0.00	PROA
ATOM	2175	CD2	TYR	P	135	5.212	10.386	-20.599	0.00	0.00	PROA
ATOM	2176	HD2	TYR	P	135	5.387	9.494	-20.016	0.00	0.00	PROA
ATOM	2177	CE2	TYR	P	135	6.360	11.073	-20.998	0.00	0.00	PROA
ATOM	2178	HE2	TYR	P	135	7.335	10.738	-20.675	0.00	0.00	PROA
ATOM	2179	C	TYR	P	135	3.249	8.860	-23.165	0.00	0.00	PROA
ATOM	2180	O	TYR	P	135	2.985	9.600	-24.148	0.00	0.00	PROA
ATOM	2181	N	LEU	P	136	4.211	7.936	-23.271	0.00	0.00	PROA
ATOM	2182	HN	LEU	P	136	4.462	7.297	-22.548	0.00	0.00	PROA
ATOM	2183	CA	LEU	P	136	5.105	7.714	-24.414	0.00	0.00	PROA
ATOM	2184	HA	LEU	P	136	5.507	8.670	-24.717	0.00	0.00	PROA
ATOM	2185	CB	LEU	P	136	6.323	6.882	-23.855	0.00	0.00	PROA
ATOM	2186	HB1	LEU	P	136	6.923	6.479	-24.698	0.00	0.00	PROA
ATOM	2187	HB2	LEU	P	136	5.835	6.015	-23.361	0.00	0.00	PROA
ATOM	2188	CG	LEU	P	136	7.308	7.758	-23.041	0.00	0.00	PROA
ATOM	2189	HG	LEU	P	136	6.690	8.419	-22.397	0.00	0.00	PROA
ATOM	2190	CD1	LEU	P	136	8.167	6.944	-22.066	0.00	0.00	PROA
ATOM	2191	HD11	LEU	P	136	7.552	6.260	-21.442	0.00	0.00	PROA

```
ATOM   2192 HD12 LEU P 136       8.672   7.648 -21.371  0.00  0.00      PROA
ATOM   2193 HD13 LEU P 136       8.902   6.353 -22.653  0.00  0.00      PROA
ATOM   2194  CD2 LEU P 136       8.145   8.720 -23.918  0.00  0.00      PROA
ATOM   2195 HD21 LEU P 136       7.479   9.542 -24.258  0.00  0.00      PROA
ATOM   2196 HD22 LEU P 136       8.698   8.167 -24.707  0.00  0.00      PROA
ATOM   2197 HD23 LEU P 136       8.949   9.199 -23.319  0.00  0.00      PROA
ATOM   2198  C   LEU P 136       4.297   7.057 -25.551  0.00  0.00      PROA
ATOM   2199  O   LEU P 136       4.784   7.089 -26.647  0.00  0.00      PROA
ATOM   2200  N   ALA P 137       3.216   6.317 -25.251  0.00  0.00      PROA
ATOM   2201  HN  ALA P 137       2.942   6.062 -24.327  0.00  0.00      PROA
ATOM   2202  CA  ALA P 137       2.337   5.794 -26.275  0.00  0.00      PROA
ATOM   2203  HA  ALA P 137       2.799   5.456 -27.191  0.00  0.00      PROA
ATOM   2204  CB  ALA P 137       1.411   4.611 -25.849  0.00  0.00      PROA
ATOM   2205  HB1 ALA P 137       1.967   3.692 -25.564  0.00  0.00      PROA
ATOM   2206  HB2 ALA P 137       0.753   4.245 -26.666  0.00  0.00      PROA
ATOM   2207  HB3 ALA P 137       0.824   4.974 -24.979  0.00  0.00      PROA
ATOM   2208  C   ALA P 137       1.270   6.820 -26.701  0.00  0.00      PROA
ATOM   2209  O   ALA P 137       0.489   6.516 -27.596  0.00  0.00      PROA
ATOM   2210  N   ILE P 138       1.305   8.041 -26.131  0.00  0.00      PROA
ATOM   2211  HN  ILE P 138       1.843   8.236 -25.315  0.00  0.00      PROA
ATOM   2212  CA  ILE P 138       0.441   9.129 -26.523  0.00  0.00      PROA
ATOM   2213  HA  ILE P 138      -0.253   8.795 -27.280  0.00  0.00      PROA
ATOM   2214  CB  ILE P 138      -0.236   9.914 -25.400  0.00  0.00      PROA
ATOM   2215  HB  ILE P 138       0.556  10.051 -24.633  0.00  0.00      PROA
ATOM   2216  CG2 ILE P 138      -0.726  11.342 -25.685  0.00  0.00      PROA
ATOM   2217 HG21 ILE P 138      -1.636  11.245 -26.316  0.00  0.00      PROA
ATOM   2218 HG22 ILE P 138      -0.088  11.980 -26.333  0.00  0.00      PROA
ATOM   2219 HG23 ILE P 138      -0.987  11.913 -24.768  0.00  0.00      PROA
ATOM   2220  CG1 ILE P 138      -1.400   9.089 -24.777  0.00  0.00      PROA
ATOM   2221 HG11 ILE P 138      -1.745   9.722 -23.931  0.00  0.00      PROA
ATOM   2222 HG12 ILE P 138      -1.006   8.095 -24.475  0.00  0.00      PROA
ATOM   2223  CD  ILE P 138      -2.553   8.846 -25.786  0.00  0.00      PROA
ATOM   2224  HD1 ILE P 138      -2.419   8.002 -26.495  0.00  0.00      PROA
ATOM   2225  HD2 ILE P 138      -2.863   9.779 -26.303  0.00  0.00      PROA
ATOM   2226  HD3 ILE P 138      -3.502   8.600 -25.263  0.00  0.00      PROA
ATOM   2227  C   ILE P 138       1.308  10.174 -27.331  0.00  0.00      PROA
ATOM   2228  O   ILE P 138       0.848  10.722 -28.367  0.00  0.00      PROA
ATOM   2229  N   VAL P 139       2.491  10.502 -26.836  0.00  0.00      PROA
ATOM   2230  HN  VAL P 139       2.809  10.111 -25.975  0.00  0.00      PROA
ATOM   2231  CA  VAL P 139       3.355  11.383 -27.500  0.00  0.00      PROA
ATOM   2232  HA  VAL P 139       2.802  11.980 -28.210  0.00  0.00      PROA
ATOM   2233  CB  VAL P 139       4.052  12.307 -26.522  0.00  0.00      PROA
ATOM   2234  HB  VAL P 139       4.797  11.717 -25.946  0.00  0.00      PROA
ATOM   2235  CG1 VAL P 139       4.733  13.393 -27.342  0.00  0.00      PROA
ATOM   2236 HG11 VAL P 139       4.909  14.241 -26.646  0.00  0.00      PROA
ATOM   2237 HG12 VAL P 139       4.041  13.842 -28.085  0.00  0.00      PROA
ATOM   2238 HG13 VAL P 139       5.784  13.186 -27.638  0.00  0.00      PROA
ATOM   2239  CG2 VAL P 139       3.048  12.847 -25.533  0.00  0.00      PROA
ATOM   2240 HG21 VAL P 139       2.319  13.498 -26.061  0.00  0.00      PROA
ATOM   2241 HG22 VAL P 139       3.487  13.503 -24.751  0.00  0.00      PROA
ATOM   2242 HG23 VAL P 139       2.501  12.037 -25.005  0.00  0.00      PROA
ATOM   2243  C   VAL P 139       4.388  10.679 -28.425  0.00  0.00      PROA
ATOM   2244  O   VAL P 139       5.478  10.208 -27.976  0.00  0.00      PROA
ATOM   2245  N   HSD P 140       4.149  10.666 -29.746  0.00  0.00      PROA
ATOM   2246  HN  HSD P 140       3.341  11.132 -30.098  0.00  0.00      PROA
ATOM   2247  CA  HSD P 140       5.180  10.317 -30.754  0.00  0.00      PROA
ATOM   2248  HA  HSD P 140       4.638  10.393 -31.686  0.00  0.00      PROA
ATOM   2249  CB  HSD P 140       6.462  11.248 -30.694  0.00  0.00      PROA
ATOM   2250  HB1 HSD P 140       7.222  10.810 -31.375  0.00  0.00      PROA
ATOM   2251  HB2 HSD P 140       6.939  11.269 -29.691  0.00  0.00      PROA
ATOM   2252  ND1 HSD P 140       5.113  13.428 -30.858  0.00  0.00      PROA
```

```
ATOM   2253  HD1 HSD P 140       4.207  13.045 -30.677  0.00  0.00      PROA
ATOM   2254  CG  HSD P 140       6.299  12.706 -30.946  0.00  0.00      PROA
ATOM   2255  CE1 HSD P 140       5.381  14.691 -31.240  0.00  0.00      PROA
ATOM   2256  HE1 HSD P 140       4.766  15.569 -31.434  0.00  0.00      PROA
ATOM   2257  NE2 HSD P 140       6.668  14.856 -31.551  0.00  0.00      PROA
ATOM   2258  CD2 HSD P 140       7.264  13.632 -31.362  0.00  0.00      PROA
ATOM   2259  HD2 HSD P 140       8.320  13.462 -31.527  0.00  0.00      PROA
ATOM   2260  C   HSD P 140       5.473   8.831 -30.650  0.00  0.00      PROA
ATOM   2261  O   HSD P 140       6.637   8.356 -30.668  0.00  0.00      PROA
ATOM   2262  N   ALA P 141       4.432   7.978 -30.579  0.00  0.00      PROA
ATOM   2263  HN  ALA P 141       3.545   8.430 -30.523  0.00  0.00      PROA
ATOM   2264  CA  ALA P 141       4.419   6.534 -30.272  0.00  0.00      PROA
ATOM   2265  HA  ALA P 141       4.856   6.330 -29.306  0.00  0.00      PROA
ATOM   2266  CB  ALA P 141       2.956   6.119 -30.194  0.00  0.00      PROA
ATOM   2267  HB1 ALA P 141       2.910   5.012 -30.283  0.00  0.00      PROA
ATOM   2268  HB2 ALA P 141       2.364   6.309 -31.115  0.00  0.00      PROA
ATOM   2269  HB3 ALA P 141       2.555   6.538 -29.246  0.00  0.00      PROA
ATOM   2270  C   ALA P 141       5.265   5.735 -31.243  0.00  0.00      PROA
ATOM   2271  O   ALA P 141       5.898   4.832 -30.878  0.00  0.00      PROA
ATOM   2272  N   THR P 142       5.280   6.125 -32.595  0.00  0.00      PROA
ATOM   2273  HN  THR P 142       4.739   6.856 -33.004  0.00  0.00      PROA
ATOM   2274  CA  THR P 142       6.140   5.403 -33.586  0.00  0.00      PROA
ATOM   2275  HA  THR P 142       6.045   4.327 -33.584  0.00  0.00      PROA
ATOM   2276  CB  THR P 142       5.730   5.744 -35.061  0.00  0.00      PROA
ATOM   2277  HB  THR P 142       6.397   5.372 -35.868  0.00  0.00      PROA
ATOM   2278  OG1 THR P 142       5.538   7.112 -35.240  0.00  0.00      PROA
ATOM   2279  HG1 THR P 142       5.113   7.300 -36.080  0.00  0.00      PROA
ATOM   2280  CG2 THR P 142       4.339   5.144 -35.241  0.00  0.00      PROA
ATOM   2281 HG21 THR P 142       3.593   5.679 -34.615  0.00  0.00      PROA
ATOM   2282 HG22 THR P 142       4.295   4.053 -35.036  0.00  0.00      PROA
ATOM   2283 HG23 THR P 142       4.091   5.298 -36.313  0.00  0.00      PROA
ATOM   2284  C   THR P 142       7.597   5.741 -33.527  0.00  0.00      PROA
ATOM   2285  O   THR P 142       8.433   5.071 -34.153  0.00  0.00      PROA
ATOM   2286  N   ASN P 143       7.962   6.737 -32.662  0.00  0.00      PROA
ATOM   2287  HN  ASN P 143       7.259   7.210 -32.135  0.00  0.00      PROA
ATOM   2288  CA  ASN P 143       9.377   7.101 -32.378  0.00  0.00      PROA
ATOM   2289  HA  ASN P 143      10.097   6.585 -32.996  0.00  0.00      PROA
ATOM   2290  CB  ASN P 143       9.614   8.630 -32.519  0.00  0.00      PROA
ATOM   2291  HB1 ASN P 143      10.624   8.973 -32.207  0.00  0.00      PROA
ATOM   2292  HB2 ASN P 143       8.910   9.078 -31.785  0.00  0.00      PROA
ATOM   2293  CG  ASN P 143       9.235   9.122 -33.949  0.00  0.00      PROA
ATOM   2294  OD1 ASN P 143       8.259   9.824 -34.133  0.00  0.00      PROA
ATOM   2295  ND2 ASN P 143      10.056   8.767 -34.976  0.00  0.00      PROA
ATOM   2296 HD21 ASN P 143       9.886   9.280 -35.818  0.00  0.00      PROA
ATOM   2297 HD22 ASN P 143      10.765   8.107 -34.728  0.00  0.00      PROA
ATOM   2298  C   ASN P 143       9.706   6.762 -30.926  0.00  0.00      PROA
ATOM   2299  O   ASN P 143      10.852   7.044 -30.586  0.00  0.00      PROA
ATOM   2300  N   SER P 144       8.867   6.078 -30.078  0.00  0.00      PROA
ATOM   2301  HN  SER P 144       7.945   5.770 -30.301  0.00  0.00      PROA
ATOM   2302  CA  SER P 144       9.090   6.036 -28.604  0.00  0.00      PROA
ATOM   2303  HA  SER P 144       9.961   6.617 -28.338  0.00  0.00      PROA
ATOM   2304  CB  SER P 144       7.940   6.607 -27.676  0.00  0.00      PROA
ATOM   2305  HB1 SER P 144       7.725   7.603 -28.120  0.00  0.00      PROA
ATOM   2306  HB2 SER P 144       8.242   6.680 -26.609  0.00  0.00      PROA
ATOM   2307  OG  SER P 144       6.770   5.854 -27.887  0.00  0.00      PROA
ATOM   2308  HG1 SER P 144       6.039   6.473 -27.830  0.00  0.00      PROA
ATOM   2309  C   SER P 144       9.595   4.704 -28.180  0.00  0.00      PROA
ATOM   2310  O   SER P 144       9.613   4.433 -26.988  0.00  0.00      PROA
ATOM   2311  N   GLN P 145      10.085   3.828 -29.108  0.00  0.00      PROA
ATOM   2312  HN  GLN P 145      10.171   4.073 -30.071  0.00  0.00      PROA
ATOM   2313  CA  GLN P 145      10.435   2.460 -28.716  0.00  0.00      PROA
```

ATOM	2314	HA	GLN	P	145	9.588	1.997	-28.232	0.00	0.00	PROA
ATOM	2315	CB	GLN	P	145	10.775	1.549	-29.928	0.00	0.00	PROA
ATOM	2316	HB1	GLN	P	145	11.477	0.762	-29.578	0.00	0.00	PROA
ATOM	2317	HB2	GLN	P	145	11.222	2.168	-30.735	0.00	0.00	PROA
ATOM	2318	CG	GLN	P	145	9.508	0.942	-30.562	0.00	0.00	PROA
ATOM	2319	HG1	GLN	P	145	8.909	0.376	-29.818	0.00	0.00	PROA
ATOM	2320	HG2	GLN	P	145	9.876	0.398	-31.458	0.00	0.00	PROA
ATOM	2321	CD	GLN	P	145	8.551	2.069	-31.001	0.00	0.00	PROA
ATOM	2322	OE1	GLN	P	145	8.791	2.625	-32.065	0.00	0.00	PROA
ATOM	2323	NE2	GLN	P	145	7.434	2.405	-30.259	0.00	0.00	PROA
ATOM	2324	HE21	GLN	P	145	6.859	3.190	-30.489	0.00	0.00	PROA
ATOM	2325	HE22	GLN	P	145	7.356	1.926	-29.384	0.00	0.00	PROA
ATOM	2326	C	GLN	P	145	11.612	2.364	-27.733	0.00	0.00	PROA
ATOM	2327	O	GLN	P	145	11.582	1.554	-26.759	0.00	0.00	PROA
ATOM	2328	N	ARG	P	146	12.672	3.199	-27.930	0.00	0.00	PROA
ATOM	2329	HN	ARG	P	146	12.719	3.843	-28.689	0.00	0.00	PROA
ATOM	2330	CA	ARG	P	146	13.766	3.297	-26.989	0.00	0.00	PROA
ATOM	2331	HA	ARG	P	146	14.075	2.262	-26.961	0.00	0.00	PROA
ATOM	2332	CB	ARG	P	146	14.922	4.039	-27.666	0.00	0.00	PROA
ATOM	2333	HB1	ARG	P	146	14.570	5.032	-28.019	0.00	0.00	PROA
ATOM	2334	HB2	ARG	P	146	15.262	3.483	-28.566	0.00	0.00	PROA
ATOM	2335	CG	ARG	P	146	16.106	4.407	-26.683	0.00	0.00	PROA
ATOM	2336	HG1	ARG	P	146	15.804	5.012	-25.802	0.00	0.00	PROA
ATOM	2337	HG2	ARG	P	146	16.827	5.071	-27.208	0.00	0.00	PROA
ATOM	2338	CD	ARG	P	146	16.959	3.259	-26.195	0.00	0.00	PROA
ATOM	2339	HD1	ARG	P	146	17.558	2.944	-27.076	0.00	0.00	PROA
ATOM	2340	HD2	ARG	P	146	16.274	2.452	-25.857	0.00	0.00	PROA
ATOM	2341	NE	ARG	P	146	17.864	3.739	-25.060	0.00	0.00	PROA
ATOM	2342	HE	ARG	P	146	18.029	4.705	-24.859	0.00	0.00	PROA
ATOM	2343	CZ	ARG	P	146	18.837	2.990	-24.491	0.00	0.00	PROA
ATOM	2344	NH1	ARG	P	146	18.955	1.725	-24.608	0.00	0.00	PROA
ATOM	2345	HH11	ARG	P	146	18.383	1.160	-25.203	0.00	0.00	PROA
ATOM	2346	HH12	ARG	P	146	19.611	1.259	-24.014	0.00	0.00	PROA
ATOM	2347	NH2	ARG	P	146	19.597	3.621	-23.579	0.00	0.00	PROA
ATOM	2348	HH21	ARG	P	146	19.564	4.604	-23.400	0.00	0.00	PROA
ATOM	2349	HH22	ARG	P	146	20.350	3.074	-23.212	0.00	0.00	PROA
ATOM	2350	C	ARG	P	146	13.534	3.669	-25.542	0.00	0.00	PROA
ATOM	2351	O	ARG	P	146	14.077	2.939	-24.732	0.00	0.00	PROA
ATOM	2352	N	PRO	P	147	12.791	4.709	-25.154	0.00	0.00	PROA
ATOM	2353	CD	PRO	P	147	12.541	5.898	-26.002	0.00	0.00	PROA
ATOM	2354	HD1	PRO	P	147	12.187	5.649	-27.026	0.00	0.00	PROA
ATOM	2355	HD2	PRO	P	147	13.475	6.494	-26.087	0.00	0.00	PROA
ATOM	2356	CA	PRO	P	147	12.497	5.091	-23.705	0.00	0.00	PROA
ATOM	2357	HA	PRO	P	147	13.428	5.154	-23.161	0.00	0.00	PROA
ATOM	2358	CB	PRO	P	147	11.780	6.464	-23.734	0.00	0.00	PROA
ATOM	2359	HB1	PRO	P	147	12.457	7.279	-23.400	0.00	0.00	PROA
ATOM	2360	HB2	PRO	P	147	10.830	6.567	-23.168	0.00	0.00	PROA
ATOM	2361	CG	PRO	P	147	11.526	6.738	-25.178	0.00	0.00	PROA
ATOM	2362	HG1	PRO	P	147	10.523	6.372	-25.483	0.00	0.00	PROA
ATOM	2363	HG2	PRO	P	147	11.564	7.820	-25.428	0.00	0.00	PROA
ATOM	2364	C	PRO	P	147	11.614	4.110	-23.045	0.00	0.00	PROA
ATOM	2365	O	PRO	P	147	11.897	3.746	-21.917	0.00	0.00	PROA
ATOM	2366	N	ARG	P	148	10.597	3.525	-23.703	0.00	0.00	PROA
ATOM	2367	HN	ARG	P	148	10.484	3.626	-24.689	0.00	0.00	PROA
ATOM	2368	CA	ARG	P	148	9.750	2.461	-23.137	0.00	0.00	PROA
ATOM	2369	HA	ARG	P	148	9.325	2.837	-22.218	0.00	0.00	PROA
ATOM	2370	CB	ARG	P	148	8.620	2.143	-24.139	0.00	0.00	PROA
ATOM	2371	HB1	ARG	P	148	7.998	1.339	-23.691	0.00	0.00	PROA
ATOM	2372	HB2	ARG	P	148	9.039	1.712	-25.074	0.00	0.00	PROA
ATOM	2373	CG	ARG	P	148	7.777	3.453	-24.460	0.00	0.00	PROA
ATOM	2374	HG1	ARG	P	148	8.433	4.316	-24.705	0.00	0.00	PROA

ATOM	2375	HG2	ARG	P	148	7.226	3.815	-23.566	0.00	0.00	PROA
ATOM	2376	CD	ARG	P	148	6.613	3.203	-25.470	0.00	0.00	PROA
ATOM	2377	HD1	ARG	P	148	6.978	2.862	-26.462	0.00	0.00	PROA
ATOM	2378	HD2	ARG	P	148	5.966	4.081	-25.677	0.00	0.00	PROA
ATOM	2379	NE	ARG	P	148	5.773	2.134	-24.813	0.00	0.00	PROA
ATOM	2380	HE	ARG	P	148	5.875	1.845	-23.861	0.00	0.00	PROA
ATOM	2381	CZ	ARG	P	148	4.687	1.600	-25.393	0.00	0.00	PROA
ATOM	2382	NH1	ARG	P	148	4.277	1.757	-26.616	0.00	0.00	PROA
ATOM	2383	HH11	ARG	P	148	4.858	2.311	-27.212	0.00	0.00	PROA
ATOM	2384	HH12	ARG	P	148	3.452	1.259	-26.885	0.00	0.00	PROA
ATOM	2385	NH2	ARG	P	148	3.956	0.783	-24.666	0.00	0.00	PROA
ATOM	2386	HH21	ARG	P	148	4.134	0.757	-23.682	0.00	0.00	PROA
ATOM	2387	HH22	ARG	P	148	3.175	0.338	-25.105	0.00	0.00	PROA
ATOM	2388	C	ARG	P	148	10.451	1.158	-22.845	0.00	0.00	PROA
ATOM	2389	O	ARG	P	148	10.185	0.376	-21.970	0.00	0.00	PROA
ATOM	2390	N	LYS	P	149	11.476	0.833	-23.692	0.00	0.00	PROA
ATOM	2391	HN	LYS	P	149	11.657	1.463	-24.444	0.00	0.00	PROA
ATOM	2392	CA	LYS	P	149	12.364	-0.274	-23.566	0.00	0.00	PROA
ATOM	2393	HA	LYS	P	149	11.856	-1.168	-23.235	0.00	0.00	PROA
ATOM	2394	CB	LYS	P	149	12.995	-0.476	-24.944	0.00	0.00	PROA
ATOM	2395	HB1	LYS	P	149	13.552	0.438	-25.241	0.00	0.00	PROA
ATOM	2396	HB2	LYS	P	149	12.121	-0.680	-25.600	0.00	0.00	PROA
ATOM	2397	CG	LYS	P	149	14.088	-1.560	-25.180	0.00	0.00	PROA
ATOM	2398	HG1	LYS	P	149	14.932	-1.623	-24.461	0.00	0.00	PROA
ATOM	2399	HG2	LYS	P	149	14.544	-1.419	-26.183	0.00	0.00	PROA
ATOM	2400	CD	LYS	P	149	13.502	-2.963	-25.215	0.00	0.00	PROA
ATOM	2401	HD1	LYS	P	149	12.709	-3.100	-25.981	0.00	0.00	PROA
ATOM	2402	HD2	LYS	P	149	13.100	-3.192	-24.205	0.00	0.00	PROA
ATOM	2403	CE	LYS	P	149	14.548	-3.954	-25.727	0.00	0.00	PROA
ATOM	2404	HE1	LYS	P	149	15.483	-3.871	-25.133	0.00	0.00	PROA
ATOM	2405	HE2	LYS	P	149	14.824	-3.763	-26.787	0.00	0.00	PROA
ATOM	2406	NZ	LYS	P	149	14.101	-5.374	-25.522	0.00	0.00	PROA
ATOM	2407	HZ1	LYS	P	149	13.123	-5.531	-25.838	0.00	0.00	PROA
ATOM	2408	HZ2	LYS	P	149	14.158	-5.542	-24.497	0.00	0.00	PROA
ATOM	2409	HZ3	LYS	P	149	14.890	-5.909	-25.937	0.00	0.00	PROA
ATOM	2410	C	LYS	P	149	13.318	0.002	-22.491	0.00	0.00	PROA
ATOM	2411	O	LYS	P	149	13.711	-0.909	-21.729	0.00	0.00	PROA
ATOM	2412	N	LEU	P	150	13.830	1.253	-22.362	0.00	0.00	PROA
ATOM	2413	HN	LEU	P	150	13.527	2.010	-22.935	0.00	0.00	PROA
ATOM	2414	CA	LEU	P	150	14.789	1.563	-21.277	0.00	0.00	PROA
ATOM	2415	HA	LEU	P	150	15.474	0.743	-21.439	0.00	0.00	PROA
ATOM	2416	CB	LEU	P	150	15.432	3.005	-21.648	0.00	0.00	PROA
ATOM	2417	HB1	LEU	P	150	14.558	3.673	-21.803	0.00	0.00	PROA
ATOM	2418	HB2	LEU	P	150	15.961	2.902	-22.619	0.00	0.00	PROA
ATOM	2419	CG	LEU	P	150	16.394	3.542	-20.678	0.00	0.00	PROA
ATOM	2420	HG	LEU	P	150	15.858	3.590	-19.706	0.00	0.00	PROA
ATOM	2421	CD1	LEU	P	150	17.668	2.679	-20.524	0.00	0.00	PROA
ATOM	2422	HD11	LEU	P	150	18.486	3.306	-20.108	0.00	0.00	PROA
ATOM	2423	HD12	LEU	P	150	17.985	2.315	-21.525	0.00	0.00	PROA
ATOM	2424	HD13	LEU	P	150	17.404	1.843	-19.842	0.00	0.00	PROA
ATOM	2425	CD2	LEU	P	150	16.777	4.974	-21.156	0.00	0.00	PROA
ATOM	2426	HD21	LEU	P	150	17.587	5.413	-20.535	0.00	0.00	PROA
ATOM	2427	HD22	LEU	P	150	15.841	5.569	-21.218	0.00	0.00	PROA
ATOM	2428	HD23	LEU	P	150	17.270	4.903	-22.149	0.00	0.00	PROA
ATOM	2429	C	LEU	P	150	14.135	1.564	-19.962	0.00	0.00	PROA
ATOM	2430	O	LEU	P	150	14.837	1.349	-18.908	0.00	0.00	PROA
ATOM	2431	N	LEU	P	151	12.844	1.901	-19.814	0.00	0.00	PROA
ATOM	2432	HN	LEU	P	151	12.296	2.399	-20.482	0.00	0.00	PROA
ATOM	2433	CA	LEU	P	151	12.231	1.855	-18.543	0.00	0.00	PROA
ATOM	2434	HA	LEU	P	151	12.882	2.222	-17.764	0.00	0.00	PROA
ATOM	2435	CB	LEU	P	151	10.881	2.677	-18.527	0.00	0.00	PROA

ATOM	2436	HB1	LEU	P	151	10.304	2.396	-17.620	0.00	0.00	PROA
ATOM	2437	HB2	LEU	P	151	10.210	2.280	-19.318	0.00	0.00	PROA
ATOM	2438	CG	LEU	P	151	11.153	4.149	-18.571	0.00	0.00	PROA
ATOM	2439	HG	LEU	P	151	11.388	4.529	-19.588	0.00	0.00	PROA
ATOM	2440	CD1	LEU	P	151	9.897	4.853	-18.214	0.00	0.00	PROA
ATOM	2441	HD11	LEU	P	151	9.492	4.509	-17.238	0.00	0.00	PROA
ATOM	2442	HD12	LEU	P	151	9.141	4.506	-18.949	0.00	0.00	PROA
ATOM	2443	HD13	LEU	P	151	10.015	5.958	-18.243	0.00	0.00	PROA
ATOM	2444	CD2	LEU	P	151	12.167	4.768	-17.580	0.00	0.00	PROA
ATOM	2445	HD21	LEU	P	151	11.903	4.402	-16.564	0.00	0.00	PROA
ATOM	2446	HD22	LEU	P	151	12.134	5.874	-17.480	0.00	0.00	PROA
ATOM	2447	HD23	LEU	P	151	13.191	4.405	-17.812	0.00	0.00	PROA
ATOM	2448	C	LEU	P	151	11.878	0.418	-18.087	0.00	0.00	PROA
ATOM	2449	O	LEU	P	151	12.275	-0.016	-16.995	0.00	0.00	PROA
ATOM	2450	N	ALA	P	152	11.244	-0.380	-18.943	0.00	0.00	PROA
ATOM	2451	HN	ALA	P	152	11.001	0.031	-19.818	0.00	0.00	PROA
ATOM	2452	CA	ALA	P	152	10.726	-1.687	-18.509	0.00	0.00	PROA
ATOM	2453	HA	ALA	P	152	10.281	-1.567	-17.532	0.00	0.00	PROA
ATOM	2454	CB	ALA	P	152	9.666	-2.055	-19.560	0.00	0.00	PROA
ATOM	2455	HB1	ALA	P	152	9.286	-3.095	-19.468	0.00	0.00	PROA
ATOM	2456	HB2	ALA	P	152	10.190	-1.822	-20.511	0.00	0.00	PROA
ATOM	2457	HB3	ALA	P	152	8.857	-1.293	-19.552	0.00	0.00	PROA
ATOM	2458	C	ALA	P	152	11.803	-2.710	-18.347	0.00	0.00	PROA
ATOM	2459	O	ALA	P	152	11.696	-3.627	-17.574	0.00	0.00	PROA
ATOM	2460	N	GLU	P	153	12.981	-2.602	-18.971	0.00	0.00	PROA
ATOM	2461	HN	GLU	P	153	13.050	-1.880	-19.655	0.00	0.00	PROA
ATOM	2462	CA	GLU	P	153	13.984	-3.693	-18.958	0.00	0.00	PROA
ATOM	2463	HA	GLU	P	153	13.636	-4.526	-18.366	0.00	0.00	PROA
ATOM	2464	CB	GLU	P	153	14.292	-4.112	-20.421	0.00	0.00	PROA
ATOM	2465	HB1	GLU	P	153	15.141	-4.830	-20.407	0.00	0.00	PROA
ATOM	2466	HB2	GLU	P	153	14.554	-3.214	-21.019	0.00	0.00	PROA
ATOM	2467	CG	GLU	P	153	13.076	-4.839	-21.057	0.00	0.00	PROA
ATOM	2468	HG1	GLU	P	153	12.339	-4.016	-21.178	0.00	0.00	PROA
ATOM	2469	HG2	GLU	P	153	12.644	-5.489	-20.266	0.00	0.00	PROA
ATOM	2470	CD	GLU	P	153	13.273	-5.500	-22.337	0.00	0.00	PROA
ATOM	2471	OE1	GLU	P	153	14.467	-5.748	-22.686	0.00	0.00	PROA
ATOM	2472	OE2	GLU	P	153	12.349	-5.849	-23.055	0.00	0.00	PROA
ATOM	2473	C	GLU	P	153	15.209	-3.231	-18.193	0.00	0.00	PROA
ATOM	2474	O	GLU	P	153	16.154	-3.952	-18.008	0.00	0.00	PROA
ATOM	2475	N	LYS	P	154	15.190	-2.034	-17.612	0.00	0.00	PROA
ATOM	2476	HN	LYS	P	154	14.293	-1.600	-17.635	0.00	0.00	PROA
ATOM	2477	CA	LYS	P	154	16.418	-1.570	-16.896	0.00	0.00	PROA
ATOM	2478	HA	LYS	P	154	16.857	-2.418	-16.393	0.00	0.00	PROA
ATOM	2479	CB	LYS	P	154	17.356	-0.917	-17.858	0.00	0.00	PROA
ATOM	2480	HB1	LYS	P	154	17.048	0.101	-18.179	0.00	0.00	PROA
ATOM	2481	HB2	LYS	P	154	17.406	-1.434	-18.840	0.00	0.00	PROA
ATOM	2482	CG	LYS	P	154	18.802	-0.609	-17.272	0.00	0.00	PROA
ATOM	2483	HG1	LYS	P	154	18.805	0.148	-16.458	0.00	0.00	PROA
ATOM	2484	HG2	LYS	P	154	19.310	-0.047	-18.085	0.00	0.00	PROA
ATOM	2485	CD	LYS	P	154	19.492	-1.898	-16.811	0.00	0.00	PROA
ATOM	2486	HD1	LYS	P	154	19.193	-2.636	-17.586	0.00	0.00	PROA
ATOM	2487	HD2	LYS	P	154	19.147	-2.200	-15.799	0.00	0.00	PROA
ATOM	2488	CE	LYS	P	154	20.991	-1.690	-16.877	0.00	0.00	PROA
ATOM	2489	HE1	LYS	P	154	21.495	-1.084	-16.094	0.00	0.00	PROA
ATOM	2490	HE2	LYS	P	154	21.312	-1.203	-17.823	0.00	0.00	PROA
ATOM	2491	NZ	LYS	P	154	21.795	-2.981	-16.838	0.00	0.00	PROA
ATOM	2492	HZ1	LYS	P	154	21.322	-3.616	-17.512	0.00	0.00	PROA
ATOM	2493	HZ2	LYS	P	154	21.808	-3.463	-15.916	0.00	0.00	PROA
ATOM	2494	HZ3	LYS	P	154	22.775	-2.840	-17.154	0.00	0.00	PROA
ATOM	2495	C	LYS	P	154	16.076	-0.635	-15.696	0.00	0.00	PROA
ATOM	2496	O	LYS	P	154	16.291	-0.948	-14.495	0.00	0.00	PROA

ATOM	2497	N	VAL	P	155	15.592	0.617	-16.032	0.00	0.00	PROA
ATOM	2498	HN	VAL	P	155	15.417	0.719	-17.008	0.00	0.00	PROA
ATOM	2499	CA	VAL	P	155	15.317	1.697	-14.991	0.00	0.00	PROA
ATOM	2500	HA	VAL	P	155	16.245	1.850	-14.460	0.00	0.00	PROA
ATOM	2501	CB	VAL	P	155	15.120	3.054	-15.747	0.00	0.00	PROA
ATOM	2502	HB	VAL	P	155	14.300	3.030	-16.497	0.00	0.00	PROA
ATOM	2503	CG1	VAL	P	155	14.847	4.080	-14.628	0.00	0.00	PROA
ATOM	2504	HG11	VAL	P	155	13.820	3.914	-14.240	0.00	0.00	PROA
ATOM	2505	HG12	VAL	P	155	15.031	5.073	-15.092	0.00	0.00	PROA
ATOM	2506	HG13	VAL	P	155	15.613	4.026	-13.825	0.00	0.00	PROA
ATOM	2507	CG2	VAL	P	155	16.423	3.398	-16.535	0.00	0.00	PROA
ATOM	2508	HG21	VAL	P	155	17.249	3.544	-15.806	0.00	0.00	PROA
ATOM	2509	HG22	VAL	P	155	16.292	4.449	-16.871	0.00	0.00	PROA
ATOM	2510	HG23	VAL	P	155	16.555	2.750	-17.428	0.00	0.00	PROA
ATOM	2511	C	VAL	P	155	14.329	1.363	-13.843	0.00	0.00	PROA
ATOM	2512	O	VAL	P	155	14.459	1.777	-12.699	0.00	0.00	PROA
ATOM	2513	N	VAL	P	156	13.391	0.484	-14.171	0.00	0.00	PROA
ATOM	2514	HN	VAL	P	156	13.373	0.255	-15.141	0.00	0.00	PROA
ATOM	2515	CA	VAL	P	156	12.538	-0.167	-13.183	0.00	0.00	PROA
ATOM	2516	HA	VAL	P	156	12.001	0.605	-12.651	0.00	0.00	PROA
ATOM	2517	CB	VAL	P	156	11.400	-0.971	-13.796	0.00	0.00	PROA
ATOM	2518	HB	VAL	P	156	10.888	-0.236	-14.453	0.00	0.00	PROA
ATOM	2519	CG1	VAL	P	156	11.958	-2.129	-14.710	0.00	0.00	PROA
ATOM	2520	HG11	VAL	P	156	12.763	-1.794	-15.398	0.00	0.00	PROA
ATOM	2521	HG12	VAL	P	156	11.082	-2.348	-15.358	0.00	0.00	PROA
ATOM	2522	HG13	VAL	P	156	12.179	-3.112	-14.242	0.00	0.00	PROA
ATOM	2523	CG2	VAL	P	156	10.489	-1.506	-12.705	0.00	0.00	PROA
ATOM	2524	HG21	VAL	P	156	10.259	-0.686	-11.992	0.00	0.00	PROA
ATOM	2525	HG22	VAL	P	156	10.980	-2.283	-12.080	0.00	0.00	PROA
ATOM	2526	HG23	VAL	P	156	9.519	-1.941	-13.028	0.00	0.00	PROA
ATOM	2527	C	VAL	P	156	13.272	-1.049	-12.178	0.00	0.00	PROA
ATOM	2528	O	VAL	P	156	12.924	-1.135	-10.978	0.00	0.00	PROA
ATOM	2529	N	TYR	P	157	14.388	-1.663	-12.600	0.00	0.00	PROA
ATOM	2530	HN	TYR	P	157	14.565	-1.506	-13.568	0.00	0.00	PROA
ATOM	2531	CA	TYR	P	157	15.194	-2.452	-11.635	0.00	0.00	PROA
ATOM	2532	HA	TYR	P	157	14.642	-2.960	-10.859	0.00	0.00	PROA
ATOM	2533	CB	TYR	P	157	16.001	-3.545	-12.343	0.00	0.00	PROA
ATOM	2534	HB1	TYR	P	157	16.692	-4.082	-11.659	0.00	0.00	PROA
ATOM	2535	HB2	TYR	P	157	16.497	-2.924	-13.120	0.00	0.00	PROA
ATOM	2536	CG	TYR	P	157	15.000	-4.485	-12.970	0.00	0.00	PROA
ATOM	2537	CD1	TYR	P	157	14.550	-4.315	-14.284	0.00	0.00	PROA
ATOM	2538	HD1	TYR	P	157	14.860	-3.497	-14.917	0.00	0.00	PROA
ATOM	2539	CE1	TYR	P	157	13.633	-5.245	-14.875	0.00	0.00	PROA
ATOM	2540	HE1	TYR	P	157	13.277	-5.093	-15.883	0.00	0.00	PROA
ATOM	2541	CZ	TYR	P	157	13.151	-6.290	-14.053	0.00	0.00	PROA
ATOM	2542	OH	TYR	P	157	11.995	-6.974	-14.497	0.00	0.00	PROA
ATOM	2543	HH	TYR	P	157	11.821	-6.635	-15.378	0.00	0.00	PROA
ATOM	2544	CD2	TYR	P	157	14.458	-5.537	-12.161	0.00	0.00	PROA
ATOM	2545	HD2	TYR	P	157	14.945	-5.650	-11.204	0.00	0.00	PROA
ATOM	2546	CE2	TYR	P	157	13.520	-6.391	-12.730	0.00	0.00	PROA
ATOM	2547	HE2	TYR	P	157	13.003	-7.175	-12.196	0.00	0.00	PROA
ATOM	2548	C	TYR	P	157	16.218	-1.608	-10.845	0.00	0.00	PROA
ATOM	2549	O	TYR	P	157	16.205	-1.723	-9.663	0.00	0.00	PROA
ATOM	2550	N	VAL	P	158	16.852	-0.605	-11.440	0.00	0.00	PROA
ATOM	2551	HN	VAL	P	158	16.903	-0.618	-12.436	0.00	0.00	PROA
ATOM	2552	CA	VAL	P	158	17.740	0.310	-10.766	0.00	0.00	PROA
ATOM	2553	HA	VAL	P	158	18.353	-0.225	-10.056	0.00	0.00	PROA
ATOM	2554	CB	VAL	P	158	18.505	0.861	-11.955	0.00	0.00	PROA
ATOM	2555	HB	VAL	P	158	17.829	1.248	-12.747	0.00	0.00	PROA
ATOM	2556	CG1	VAL	P	158	19.376	2.048	-11.452	0.00	0.00	PROA
ATOM	2557	HG11	VAL	P	158	19.952	1.863	-10.520	0.00	0.00	PROA

```
ATOM   2558 HG12 VAL P 158      18.672   2.873 -11.211  0.00  0.00      PROA
ATOM   2559 HG13 VAL P 158      20.062   2.490 -12.206  0.00  0.00      PROA
ATOM   2560  CG2 VAL P 158      19.479  -0.202 -12.499  0.00  0.00      PROA
ATOM   2561 HG21 VAL P 158      18.873  -1.099 -12.749  0.00  0.00      PROA
ATOM   2562 HG22 VAL P 158      20.306  -0.443 -11.798  0.00  0.00      PROA
ATOM   2563 HG23 VAL P 158      19.865   0.185 -13.467  0.00  0.00      PROA
ATOM   2564  C   VAL P 158      17.044   1.352  -9.945  0.00  0.00      PROA
ATOM   2565  O   VAL P 158      17.639   1.800  -9.018  0.00  0.00      PROA
ATOM   2566  N   GLY P 159      15.723   1.589 -10.188  0.00  0.00      PROA
ATOM   2567  HN  GLY P 159      15.278   1.235 -11.007  0.00  0.00      PROA
ATOM   2568  CA  GLY P 159      15.154   2.786  -9.633  0.00  0.00      PROA
ATOM   2569  HA1 GLY P 159      15.026   3.538 -10.397  0.00  0.00      PROA
ATOM   2570  HA2 GLY P 159      15.724   3.140  -8.787  0.00  0.00      PROA
ATOM   2571  C   GLY P 159      13.796   2.610  -8.996  0.00  0.00      PROA
ATOM   2572  O   GLY P 159      13.280   3.524  -8.330  0.00  0.00      PROA
ATOM   2573  N   VAL P 160      13.213   1.399  -9.071  0.00  0.00      PROA
ATOM   2574  HN  VAL P 160      13.584   0.672  -9.644  0.00  0.00      PROA
ATOM   2575  CA  VAL P 160      12.108   0.860  -8.246  0.00  0.00      PROA
ATOM   2576  HA  VAL P 160      11.749   1.565  -7.510  0.00  0.00      PROA
ATOM   2577  CB  VAL P 160      10.813   0.483  -8.966  0.00  0.00      PROA
ATOM   2578  HB  VAL P 160      10.934  -0.487  -9.494  0.00  0.00      PROA
ATOM   2579  CG1 VAL P 160       9.744   0.231  -7.812  0.00  0.00      PROA
ATOM   2580 HG11 VAL P 160      10.032  -0.733  -7.342  0.00  0.00      PROA
ATOM   2581 HG12 VAL P 160       8.714   0.114  -8.210  0.00  0.00      PROA
ATOM   2582 HG13 VAL P 160       9.747   0.978  -6.989  0.00  0.00      PROA
ATOM   2583  CG2 VAL P 160      10.375   1.493 -10.056  0.00  0.00      PROA
ATOM   2584 HG21 VAL P 160       9.452   1.213 -10.607  0.00  0.00      PROA
ATOM   2585 HG22 VAL P 160      11.172   1.425 -10.828  0.00  0.00      PROA
ATOM   2586 HG23 VAL P 160      10.248   2.468  -9.539  0.00  0.00      PROA
ATOM   2587  C   VAL P 160      12.655  -0.341  -7.455  0.00  0.00      PROA
ATOM   2588  O   VAL P 160      12.764  -0.298  -6.275  0.00  0.00      PROA
ATOM   2589  N   TRP P 161      12.784  -1.540  -8.094  0.00  0.00      PROA
ATOM   2590  HN  TRP P 161      12.726  -1.692  -9.078  0.00  0.00      PROA
ATOM   2591  CA  TRP P 161      12.878  -2.806  -7.308  0.00  0.00      PROA
ATOM   2592  HA  TRP P 161      12.009  -2.874  -6.671  0.00  0.00      PROA
ATOM   2593  CB  TRP P 161      12.855  -4.060  -8.179  0.00  0.00      PROA
ATOM   2594  HB1 TRP P 161      13.063  -5.026  -7.671  0.00  0.00      PROA
ATOM   2595  HB2 TRP P 161      13.709  -4.066  -8.889  0.00  0.00      PROA
ATOM   2596  CG  TRP P 161      11.607  -4.258  -8.939  0.00  0.00      PROA
ATOM   2597  CD1 TRP P 161      11.473  -4.136 -10.296  0.00  0.00      PROA
ATOM   2598  HD1 TRP P 161      12.258  -3.748 -10.928  0.00  0.00      PROA
ATOM   2599  NE1 TRP P 161      10.178  -4.454 -10.630  0.00  0.00      PROA
ATOM   2600  HE1 TRP P 161       9.741  -4.284 -11.486  0.00  0.00      PROA
ATOM   2601  CE2 TRP P 161       9.431  -4.756  -9.513  0.00  0.00      PROA
ATOM   2602  CD2 TRP P 161      10.306  -4.689  -8.412  0.00  0.00      PROA
ATOM   2603  CE3 TRP P 161       9.892  -5.042  -7.100  0.00  0.00      PROA
ATOM   2604  HE3 TRP P 161      10.539  -5.064  -6.236  0.00  0.00      PROA
ATOM   2605  CZ3 TRP P 161       8.588  -5.545  -6.892  0.00  0.00      PROA
ATOM   2606  HZ3 TRP P 161       8.258  -5.851  -5.911  0.00  0.00      PROA
ATOM   2607  CZ2 TRP P 161       8.137  -5.066  -9.281  0.00  0.00      PROA
ATOM   2608  HZ2 TRP P 161       7.501  -5.220 -10.140  0.00  0.00      PROA
ATOM   2609  CH2 TRP P 161       7.750  -5.582  -8.008  0.00  0.00      PROA
ATOM   2610  HH2 TRP P 161       6.731  -5.933  -7.940  0.00  0.00      PROA
ATOM   2611  C   TRP P 161      14.161  -2.971  -6.458  0.00  0.00      PROA
ATOM   2612  O   TRP P 161      14.098  -3.255  -5.277  0.00  0.00      PROA
ATOM   2613  N   ILE P 162      15.400  -2.722  -6.985  0.00  0.00      PROA
ATOM   2614  HN  ILE P 162      15.481  -2.324  -7.896  0.00  0.00      PROA
ATOM   2615  CA  ILE P 162      16.527  -2.566  -6.120  0.00  0.00      PROA
ATOM   2616  HA  ILE P 162      16.532  -3.486  -5.555  0.00  0.00      PROA
ATOM   2617  CB  ILE P 162      17.804  -2.582  -6.927  0.00  0.00      PROA
ATOM   2618  HB  ILE P 162      17.846  -1.801  -7.717  0.00  0.00      PROA
```

ATOM 2619 CG2 ILE P 162 19.004 -2.556 -5.903 0.00 0.00 PROA
ATOM 2620 HG21 ILE P 162 19.980 -2.388 -6.407 0.00 0.00 PROA
ATOM 2621 HG22 ILE P 162 19.019 -3.329 -5.105 0.00 0.00 PROA
ATOM 2622 HG23 ILE P 162 18.985 -1.586 -5.361 0.00 0.00 PROA
ATOM 2623 CG1 ILE P 162 17.982 -3.909 -7.780 0.00 0.00 PROA
ATOM 2624 HG11 ILE P 162 17.100 -3.900 -8.455 0.00 0.00 PROA
ATOM 2625 HG12 ILE P 162 17.998 -4.731 -7.033 0.00 0.00 PROA
ATOM 2626 CD ILE P 162 19.230 -3.927 -8.645 0.00 0.00 PROA
ATOM 2627 HD1 ILE P 162 19.141 -4.735 -9.402 0.00 0.00 PROA
ATOM 2628 HD2 ILE P 162 20.183 -4.054 -8.088 0.00 0.00 PROA
ATOM 2629 HD3 ILE P 162 19.271 -2.983 -9.230 0.00 0.00 PROA
ATOM 2630 C ILE P 162 16.494 -1.468 -5.002 0.00 0.00 PROA
ATOM 2631 O ILE P 162 16.739 -1.827 -3.831 0.00 0.00 PROA
ATOM 2632 N PRO P 163 16.113 -0.214 -5.189 0.00 0.00 PROA
ATOM 2633 CD PRO P 163 16.486 0.468 -6.412 0.00 0.00 PROA
ATOM 2634 HD1 PRO P 163 15.659 0.317 -7.139 0.00 0.00 PROA
ATOM 2635 HD2 PRO P 163 17.446 0.177 -6.888 0.00 0.00 PROA
ATOM 2636 CA PRO P 163 15.806 0.759 -4.125 0.00 0.00 PROA
ATOM 2637 HA PRO P 163 16.715 0.946 -3.572 0.00 0.00 PROA
ATOM 2638 CB PRO P 163 15.471 1.951 -5.000 0.00 0.00 PROA
ATOM 2639 HB1 PRO P 163 15.507 2.864 -4.369 0.00 0.00 PROA
ATOM 2640 HB2 PRO P 163 14.535 1.903 -5.597 0.00 0.00 PROA
ATOM 2641 CG PRO P 163 16.567 1.884 -5.982 0.00 0.00 PROA
ATOM 2642 HG1 PRO P 163 16.394 2.609 -6.806 0.00 0.00 PROA
ATOM 2643 HG2 PRO P 163 17.574 2.077 -5.554 0.00 0.00 PROA
ATOM 2644 C PRO P 163 14.783 0.342 -2.999 0.00 0.00 PROA
ATOM 2645 O PRO P 163 15.017 0.665 -1.856 0.00 0.00 PROA
ATOM 2646 N ALA P 164 13.809 -0.495 -3.414 0.00 0.00 PROA
ATOM 2647 HN ALA P 164 13.575 -0.676 -4.366 0.00 0.00 PROA
ATOM 2648 CA ALA P 164 12.833 -1.095 -2.538 0.00 0.00 PROA
ATOM 2649 HA ALA P 164 12.313 -0.328 -1.982 0.00 0.00 PROA
ATOM 2650 CB ALA P 164 11.762 -1.925 -3.350 0.00 0.00 PROA
ATOM 2651 HB1 ALA P 164 11.389 -1.332 -4.213 0.00 0.00 PROA
ATOM 2652 HB2 ALA P 164 10.911 -2.086 -2.655 0.00 0.00 PROA
ATOM 2653 HB3 ALA P 164 12.170 -2.861 -3.788 0.00 0.00 PROA
ATOM 2654 C ALA P 164 13.542 -1.912 -1.447 0.00 0.00 PROA
ATOM 2655 O ALA P 164 13.119 -1.766 -0.299 0.00 0.00 PROA
ATOM 2656 N LEU P 165 14.594 -2.617 -1.719 0.00 0.00 PROA
ATOM 2657 HN LEU P 165 15.017 -2.689 -2.619 0.00 0.00 PROA
ATOM 2658 CA LEU P 165 15.390 -3.300 -0.757 0.00 0.00 PROA
ATOM 2659 HA LEU P 165 14.687 -3.919 -0.219 0.00 0.00 PROA
ATOM 2660 CB LEU P 165 16.373 -4.320 -1.439 0.00 0.00 PROA
ATOM 2661 HB1 LEU P 165 17.220 -4.696 -0.827 0.00 0.00 PROA
ATOM 2662 HB2 LEU P 165 16.857 -3.820 -2.305 0.00 0.00 PROA
ATOM 2663 CG LEU P 165 15.592 -5.571 -1.808 0.00 0.00 PROA
ATOM 2664 HG LEU P 165 14.624 -5.204 -2.211 0.00 0.00 PROA
ATOM 2665 CD1 LEU P 165 16.256 -6.178 -3.051 0.00 0.00 PROA
ATOM 2666 HD11 LEU P 165 16.073 -5.479 -3.895 0.00 0.00 PROA
ATOM 2667 HD12 LEU P 165 15.785 -7.136 -3.358 0.00 0.00 PROA
ATOM 2668 HD13 LEU P 165 17.330 -6.354 -2.828 0.00 0.00 PROA
ATOM 2669 CD2 LEU P 165 15.370 -6.558 -0.632 0.00 0.00 PROA
ATOM 2670 HD21 LEU P 165 14.557 -7.302 -0.775 0.00 0.00 PROA
ATOM 2671 HD22 LEU P 165 15.123 -6.012 0.304 0.00 0.00 PROA
ATOM 2672 HD23 LEU P 165 16.319 -7.103 -0.441 0.00 0.00 PROA
ATOM 2673 C LEU P 165 16.137 -2.439 0.217 0.00 0.00 PROA
ATOM 2674 O LEU P 165 16.156 -2.723 1.435 0.00 0.00 PROA
ATOM 2675 N LEU P 166 16.741 -1.332 -0.279 0.00 0.00 PROA
ATOM 2676 HN LEU P 166 16.625 -1.342 -1.269 0.00 0.00 PROA
ATOM 2677 CA LEU P 166 17.347 -0.210 0.456 0.00 0.00 PROA
ATOM 2678 HA LEU P 166 17.994 -0.741 1.139 0.00 0.00 PROA
ATOM 2679 CB LEU P 166 18.130 0.686 -0.463 0.00 0.00 PROA

```
ATOM   2680  HB1 LEU P 166      18.823   1.301   0.150  0.00  0.00      PROA
ATOM   2681  HB2 LEU P 166      17.406   1.343  -0.990  0.00  0.00      PROA
ATOM   2682  CG  LEU P 166      18.973   0.008  -1.565  0.00  0.00      PROA
ATOM   2683  HG  LEU P 166      18.244  -0.609  -2.133  0.00  0.00      PROA
ATOM   2684  CD1 LEU P 166      19.418   1.270  -2.434  0.00  0.00      PROA
ATOM   2685 HD11 LEU P 166      20.191   1.837  -1.873  0.00  0.00      PROA
ATOM   2686 HD12 LEU P 166      18.558   1.964  -2.557  0.00  0.00      PROA
ATOM   2687 HD13 LEU P 166      19.819   0.946  -3.418  0.00  0.00      PROA
ATOM   2688  CD2 LEU P 166      20.125  -0.861  -1.055  0.00  0.00      PROA
ATOM   2689 HD21 LEU P 166      20.856  -0.143  -0.626  0.00  0.00      PROA
ATOM   2690 HD22 LEU P 166      20.666  -1.173  -1.974  0.00  0.00      PROA
ATOM   2691 HD23 LEU P 166      19.853  -1.666  -0.339  0.00  0.00      PROA
ATOM   2692  C   LEU P 166      16.354   0.596   1.374  0.00  0.00      PROA
ATOM   2693  O   LEU P 166      16.675   0.988   2.508  0.00  0.00      PROA
ATOM   2694  N   LEU P 167      15.116   0.708   0.923  0.00  0.00      PROA
ATOM   2695  HN  LEU P 167      14.978   0.348   0.004  0.00  0.00      PROA
ATOM   2696  CA  LEU P 167      13.998   1.255   1.685  0.00  0.00      PROA
ATOM   2697  HA  LEU P 167      14.317   2.160   2.180  0.00  0.00      PROA
ATOM   2698  CB  LEU P 167      12.722   1.566   0.826  0.00  0.00      PROA
ATOM   2699  HB1 LEU P 167      11.873   1.708   1.528  0.00  0.00      PROA
ATOM   2700  HB2 LEU P 167      12.611   0.702   0.137  0.00  0.00      PROA
ATOM   2701  CG  LEU P 167      12.746   2.814  -0.121  0.00  0.00      PROA
ATOM   2702  HG  LEU P 167      13.761   2.904  -0.564  0.00  0.00      PROA
ATOM   2703  CD1 LEU P 167      11.840   2.882  -1.330  0.00  0.00      PROA
ATOM   2704 HD11 LEU P 167      12.216   2.100  -2.024  0.00  0.00      PROA
ATOM   2705 HD12 LEU P 167      11.949   3.898  -1.765  0.00  0.00      PROA
ATOM   2706 HD13 LEU P 167      10.782   2.718  -1.031  0.00  0.00      PROA
ATOM   2707  CD2 LEU P 167      12.437   4.047   0.741  0.00  0.00      PROA
ATOM   2708 HD21 LEU P 167      12.679   4.947   0.136  0.00  0.00      PROA
ATOM   2709 HD22 LEU P 167      13.021   3.923   1.678  0.00  0.00      PROA
ATOM   2710 HD23 LEU P 167      11.348   4.042   0.959  0.00  0.00      PROA
ATOM   2711  C   LEU P 167      13.636   0.434   2.975  0.00  0.00      PROA
ATOM   2712  O   LEU P 167      13.305   1.003   3.980  0.00  0.00      PROA
ATOM   2713  N   THR P 168      13.626  -0.904   2.911  0.00  0.00      PROA
ATOM   2714  HN  THR P 168      14.082  -1.209   2.078  0.00  0.00      PROA
ATOM   2715  CA  THR P 168      13.194  -1.862   3.996  0.00  0.00      PROA
ATOM   2716  HA  THR P 168      12.534  -1.316   4.653  0.00  0.00      PROA
ATOM   2717  CB  THR P 168      12.510  -3.096   3.295  0.00  0.00      PROA
ATOM   2718  HB  THR P 168      11.890  -2.562   2.544  0.00  0.00      PROA
ATOM   2719  OG1 THR P 168      11.697  -3.877   4.197  0.00  0.00      PROA
ATOM   2720  HG1 THR P 168      11.110  -3.248   4.622  0.00  0.00      PROA
ATOM   2721  CG2 THR P 168      13.496  -4.066   2.565  0.00  0.00      PROA
ATOM   2722 HG21 THR P 168      14.090  -3.320   1.995  0.00  0.00      PROA
ATOM   2723 HG22 THR P 168      12.921  -4.797   1.957  0.00  0.00      PROA
ATOM   2724 HG23 THR P 168      14.085  -4.660   3.295  0.00  0.00      PROA
ATOM   2725  C   THR P 168      14.368  -2.233   4.842  0.00  0.00      PROA
ATOM   2726  O   THR P 168      14.256  -3.101   5.668  0.00  0.00      PROA
ATOM   2727  N   ILE P 169      15.564  -1.489   4.756  0.00  0.00      PROA
ATOM   2728  HN  ILE P 169      15.645  -0.829   4.013  0.00  0.00      PROA
ATOM   2729  CA  ILE P 169      16.675  -1.554   5.789  0.00  0.00      PROA
ATOM   2730  HA  ILE P 169      17.002  -2.575   5.659  0.00  0.00      PROA
ATOM   2731  CB  ILE P 169      17.891  -0.679   5.493  0.00  0.00      PROA
ATOM   2732  HB  ILE P 169      17.427   0.241   5.077  0.00  0.00      PROA
ATOM   2733  CG2 ILE P 169      18.816  -0.264   6.635  0.00  0.00      PROA
ATOM   2734 HG21 ILE P 169      19.132  -1.151   7.224  0.00  0.00      PROA
ATOM   2735 HG22 ILE P 169      18.298   0.365   7.391  0.00  0.00      PROA
ATOM   2736 HG23 ILE P 169      19.713   0.295   6.294  0.00  0.00      PROA
ATOM   2737  CG1 ILE P 169      18.739  -1.225   4.271  0.00  0.00      PROA
ATOM   2738 HG11 ILE P 169      17.973  -1.457   3.500  0.00  0.00      PROA
ATOM   2739 HG12 ILE P 169      19.225  -2.192   4.525  0.00  0.00      PROA
ATOM   2740  CD  ILE P 169      19.853  -0.322   3.774  0.00  0.00      PROA
```

ATOM	2741	HD1	ILE	P	169	20.812	-0.580	4.273	0.00	0.00	PROA
ATOM	2742	HD2	ILE	P	169	19.592	0.746	3.936	0.00	0.00	PROA
ATOM	2743	HD3	ILE	P	169	20.045	-0.416	2.684	0.00	0.00	PROA
ATOM	2744	C	ILE	P	169	16.269	-1.395	7.197	0.00	0.00	PROA
ATOM	2745	O	ILE	P	169	16.800	-2.189	8.018	0.00	0.00	PROA
ATOM	2746	N	PRO	P	170	15.327	-0.468	7.659	0.00	0.00	PROA
ATOM	2747	CD	PRO	P	170	15.111	0.882	7.093	0.00	0.00	PROA
ATOM	2748	HD1	PRO	P	170	14.298	0.884	6.336	0.00	0.00	PROA
ATOM	2749	HD2	PRO	P	170	16.057	1.179	6.592	0.00	0.00	PROA
ATOM	2750	CA	PRO	P	170	14.892	-0.491	9.048	0.00	0.00	PROA
ATOM	2751	HA	PRO	P	170	15.769	-0.550	9.675	0.00	0.00	PROA
ATOM	2752	CB	PRO	P	170	14.148	0.826	9.275	0.00	0.00	PROA
ATOM	2753	HB1	PRO	P	170	14.245	1.317	10.267	0.00	0.00	PROA
ATOM	2754	HB2	PRO	P	170	13.096	0.643	8.968	0.00	0.00	PROA
ATOM	2755	CG	PRO	P	170	14.833	1.741	8.290	0.00	0.00	PROA
ATOM	2756	HG1	PRO	P	170	14.200	2.605	7.993	0.00	0.00	PROA
ATOM	2757	HG2	PRO	P	170	15.660	2.227	8.850	0.00	0.00	PROA
ATOM	2758	C	PRO	P	170	13.974	-1.634	9.449	0.00	0.00	PROA
ATOM	2759	O	PRO	P	170	14.145	-2.116	10.526	0.00	0.00	PROA
ATOM	2760	N	ASP	P	171	13.109	-2.069	8.527	0.00	0.00	PROA
ATOM	2761	HN	ASP	P	171	12.881	-1.501	7.741	0.00	0.00	PROA
ATOM	2762	CA	ASP	P	171	12.275	-3.236	8.702	0.00	0.00	PROA
ATOM	2763	HA	ASP	P	171	11.590	-3.061	9.518	0.00	0.00	PROA
ATOM	2764	CB	ASP	P	171	11.531	-3.533	7.357	0.00	0.00	PROA
ATOM	2765	HB1	ASP	P	171	10.912	-4.415	7.628	0.00	0.00	PROA
ATOM	2766	HB2	ASP	P	171	12.280	-3.784	6.575	0.00	0.00	PROA
ATOM	2767	CG	ASP	P	171	10.564	-2.459	6.910	0.00	0.00	PROA
ATOM	2768	OD1	ASP	P	171	9.914	-1.861	7.758	0.00	0.00	PROA
ATOM	2769	OD2	ASP	P	171	10.364	-2.412	5.655	0.00	0.00	PROA
ATOM	2770	C	ASP	P	171	13.072	-4.531	9.046	0.00	0.00	PROA
ATOM	2771	O	ASP	P	171	12.651	-5.264	9.892	0.00	0.00	PROA
ATOM	2772	N	PHE	P	172	14.218	-4.792	8.298	0.00	0.00	PROA
ATOM	2773	HN	PHE	P	172	14.319	-4.316	7.428	0.00	0.00	PROA
ATOM	2774	CA	PHE	P	172	15.187	-5.816	8.585	0.00	0.00	PROA
ATOM	2775	HA	PHE	P	172	14.613	-6.680	8.885	0.00	0.00	PROA
ATOM	2776	CB	PHE	P	172	16.060	-6.007	7.368	0.00	0.00	PROA
ATOM	2777	HB1	PHE	P	172	16.840	-5.217	7.321	0.00	0.00	PROA
ATOM	2778	HB2	PHE	P	172	15.430	-5.954	6.454	0.00	0.00	PROA
ATOM	2779	CG	PHE	P	172	16.694	-7.435	7.544	0.00	0.00	PROA
ATOM	2780	CD1	PHE	P	172	16.264	-8.546	6.842	0.00	0.00	PROA
ATOM	2781	HD1	PHE	P	172	15.392	-8.516	6.205	0.00	0.00	PROA
ATOM	2782	CE1	PHE	P	172	16.890	-9.741	6.914	0.00	0.00	PROA
ATOM	2783	HE1	PHE	P	172	16.516	-10.551	6.304	0.00	0.00	PROA
ATOM	2784	CZ	PHE	P	172	18.005	-9.897	7.722	0.00	0.00	PROA
ATOM	2785	HZ	PHE	P	172	18.631	-10.770	7.610	0.00	0.00	PROA
ATOM	2786	CD2	PHE	P	172	17.849	-7.574	8.330	0.00	0.00	PROA
ATOM	2787	HD2	PHE	P	172	18.154	-6.655	8.808	0.00	0.00	PROA
ATOM	2788	CE2	PHE	P	172	18.481	-8.782	8.424	0.00	0.00	PROA
ATOM	2789	HE2	PHE	P	172	19.395	-8.855	8.996	0.00	0.00	PROA
ATOM	2790	C	PHE	P	172	15.893	-5.510	9.975	0.00	0.00	PROA
ATOM	2791	O	PHE	P	172	16.066	-6.422	10.795	0.00	0.00	PROA
ATOM	2792	N	ILE	P	173	16.381	-4.277	10.208	0.00	0.00	PROA
ATOM	2793	HN	ILE	P	173	16.241	-3.547	9.544	0.00	0.00	PROA
ATOM	2794	CA	ILE	P	173	17.022	-3.945	11.431	0.00	0.00	PROA
ATOM	2795	HA	ILE	P	173	17.769	-4.723	11.375	0.00	0.00	PROA
ATOM	2796	CB	ILE	P	173	17.640	-2.565	11.472	0.00	0.00	PROA
ATOM	2797	HB	ILE	P	173	16.906	-1.895	10.974	0.00	0.00	PROA
ATOM	2798	CG2	ILE	P	173	17.929	-1.948	12.872	0.00	0.00	PROA
ATOM	2799	HG21	ILE	P	173	16.985	-1.912	13.457	0.00	0.00	PROA
ATOM	2800	HG22	ILE	P	173	18.376	-0.935	12.781	0.00	0.00	PROA
ATOM	2801	HG23	ILE	P	173	18.660	-2.579	13.422	0.00	0.00	PROA

ATOM 2802 CG1 ILE P 173 18.951 -2.613 10.517 0.00 0.00 PROA
ATOM 2803 HG11 ILE P 173 18.579 -3.101 9.591 0.00 0.00 PROA
ATOM 2804 HG12 ILE P 173 19.705 -3.212 11.072 0.00 0.00 PROA
ATOM 2805 CD ILE P 173 19.563 -1.216 10.097 0.00 0.00 PROA
ATOM 2806 HD1 ILE P 173 18.766 -0.623 9.600 0.00 0.00 PROA
ATOM 2807 HD2 ILE P 173 20.472 -1.383 9.480 0.00 0.00 PROA
ATOM 2808 HD3 ILE P 173 19.903 -0.739 11.041 0.00 0.00 PROA
ATOM 2809 C ILE P 173 16.147 -4.156 12.697 0.00 0.00 PROA
ATOM 2810 O ILE P 173 16.523 -4.743 13.767 0.00 0.00 PROA
ATOM 2811 N PHE P 174 14.826 -3.752 12.614 0.00 0.00 PROA
ATOM 2812 HN PHE P 174 14.537 -3.347 11.751 0.00 0.00 PROA
ATOM 2813 CA PHE P 174 14.055 -3.529 13.757 0.00 0.00 PROA
ATOM 2814 HA PHE P 174 14.618 -3.448 14.675 0.00 0.00 PROA
ATOM 2815 CB PHE P 174 13.221 -2.226 13.739 0.00 0.00 PROA
ATOM 2816 HB1 PHE P 174 12.497 -2.253 14.581 0.00 0.00 PROA
ATOM 2817 HB2 PHE P 174 12.589 -1.996 12.854 0.00 0.00 PROA
ATOM 2818 CG PHE P 174 14.069 -0.977 14.069 0.00 0.00 PROA
ATOM 2819 CD1 PHE P 174 13.883 0.265 13.266 0.00 0.00 PROA
ATOM 2820 HD1 PHE P 174 13.264 0.262 12.381 0.00 0.00 PROA
ATOM 2821 CE1 PHE P 174 14.619 1.349 13.573 0.00 0.00 PROA
ATOM 2822 HE1 PHE P 174 14.512 2.191 12.905 0.00 0.00 PROA
ATOM 2823 CZ PHE P 174 15.375 1.471 14.760 0.00 0.00 PROA
ATOM 2824 HZ PHE P 174 15.757 2.455 14.988 0.00 0.00 PROA
ATOM 2825 CD2 PHE P 174 15.030 -0.938 15.168 0.00 0.00 PROA
ATOM 2826 HD2 PHE P 174 15.438 -1.807 15.662 0.00 0.00 PROA
ATOM 2827 CE2 PHE P 174 15.614 0.289 15.502 0.00 0.00 PROA
ATOM 2828 HE2 PHE P 174 16.145 0.326 16.442 0.00 0.00 PROA
ATOM 2829 C PHE P 174 13.025 -4.642 14.014 0.00 0.00 PROA
ATOM 2830 O PHE P 174 12.690 -4.884 15.214 0.00 0.00 PROA
ATOM 2831 N ALA P 175 12.557 -5.444 13.057 0.00 0.00 PROA
ATOM 2832 HN ALA P 175 12.937 -5.343 12.141 0.00 0.00 PROA
ATOM 2833 CA ALA P 175 11.811 -6.635 13.232 0.00 0.00 PROA
ATOM 2834 HA ALA P 175 10.974 -6.443 13.887 0.00 0.00 PROA
ATOM 2835 CB ALA P 175 11.360 -7.328 11.857 0.00 0.00 PROA
ATOM 2836 HB1 ALA P 175 12.184 -7.569 11.152 0.00 0.00 PROA
ATOM 2837 HB2 ALA P 175 10.625 -6.656 11.365 0.00 0.00 PROA
ATOM 2838 HB3 ALA P 175 10.810 -8.264 12.096 0.00 0.00 PROA
ATOM 2839 C ALA P 175 12.612 -7.686 13.984 0.00 0.00 PROA
ATOM 2840 O ALA P 175 13.796 -7.968 13.645 0.00 0.00 PROA
ATOM 2841 N ASN P 176 11.991 -8.374 14.982 0.00 0.00 PROA
ATOM 2842 HN ASN P 176 11.089 -8.077 15.285 0.00 0.00 PROA
ATOM 2843 CA ASN P 176 12.580 -9.154 15.974 0.00 0.00 PROA
ATOM 2844 HA ASN P 176 13.220 -9.891 15.512 0.00 0.00 PROA
ATOM 2845 CB ASN P 176 13.348 -8.131 16.921 0.00 0.00 PROA
ATOM 2846 HB1 ASN P 176 12.722 -7.289 17.287 0.00 0.00 PROA
ATOM 2847 HB2 ASN P 176 14.287 -7.801 16.428 0.00 0.00 PROA
ATOM 2848 CG ASN P 176 13.818 -8.733 18.204 0.00 0.00 PROA
ATOM 2849 OD1 ASN P 176 13.440 -8.289 19.290 0.00 0.00 PROA
ATOM 2850 ND2 ASN P 176 14.552 -9.867 18.175 0.00 0.00 PROA
ATOM 2851 HD21 ASN P 176 14.560 -10.382 19.032 0.00 0.00 PROA
ATOM 2852 HD22 ASN P 176 14.877 -10.274 17.321 0.00 0.00 PROA
ATOM 2853 C ASN P 176 11.485 -9.984 16.585 0.00 0.00 PROA
ATOM 2854 O ASN P 176 10.364 -9.522 16.535 0.00 0.00 PROA
ATOM 2855 N VAL P 177 11.797 -11.110 17.127 0.00 0.00 PROA
ATOM 2856 HN VAL P 177 12.688 -11.507 16.919 0.00 0.00 PROA
ATOM 2857 CA VAL P 177 10.913 -11.778 18.059 0.00 0.00 PROA
ATOM 2858 HA VAL P 177 9.919 -11.403 17.865 0.00 0.00 PROA
ATOM 2859 CB VAL P 177 10.756 -13.338 17.828 0.00 0.00 PROA
ATOM 2860 HB VAL P 177 9.922 -13.638 18.498 0.00 0.00 PROA
ATOM 2861 CG1 VAL P 177 10.269 -13.605 16.387 0.00 0.00 PROA
ATOM 2862 HG11 VAL P 177 9.218 -13.291 16.207 0.00 0.00 PROA

```
ATOM   2863 HG12 VAL P 177      10.307 -14.709  16.271  0.00  0.00      PROA
ATOM   2864 HG13 VAL P 177      10.921 -12.989  15.731  0.00  0.00      PROA
ATOM   2865  CG2 VAL P 177      12.043 -14.113  18.098  0.00  0.00      PROA
ATOM   2866 HG21 VAL P 177      11.979 -15.201  17.884  0.00  0.00      PROA
ATOM   2867 HG22 VAL P 177      12.328 -13.885  19.148  0.00  0.00      PROA
ATOM   2868 HG23 VAL P 177      12.763 -13.643  17.395  0.00  0.00      PROA
ATOM   2869  C   VAL P 177      11.177 -11.551  19.552  0.00  0.00      PROA
ATOM   2870  O   VAL P 177      12.329 -11.580  20.000  0.00  0.00      PROA
ATOM   2871  N   SER P 178      10.051 -11.273  20.316  0.00  0.00      PROA
ATOM   2872  HN  SER P 178       9.183 -11.119  19.850  0.00  0.00      PROA
ATOM   2873  CA  SER P 178      10.122 -11.150  21.772  0.00  0.00      PROA
ATOM   2874  HA  SER P 178      11.007 -11.664  22.117  0.00  0.00      PROA
ATOM   2875  CB  SER P 178      10.117  -9.703  22.332  0.00  0.00      PROA
ATOM   2876  HB1 SER P 178      10.081  -9.616  23.439  0.00  0.00      PROA
ATOM   2877  HB2 SER P 178       9.201  -9.200  21.954  0.00  0.00      PROA
ATOM   2878  OG  SER P 178      11.303  -8.958  21.905  0.00  0.00      PROA
ATOM   2879  HG1 SER P 178      11.458  -9.291  21.018  0.00  0.00      PROA
ATOM   2880  C   SER P 178       8.973 -11.777  22.501  0.00  0.00      PROA
ATOM   2881  O   SER P 178       7.983 -11.976  21.807  0.00  0.00      PROA
ATOM   2882  N   GLU P 179       9.075 -12.068  23.835  0.00  0.00      PROA
ATOM   2883  HN  GLU P 179       9.881 -11.765  24.338  0.00  0.00      PROA
ATOM   2884  CA  GLU P 179       8.070 -12.831  24.558  0.00  0.00      PROA
ATOM   2885  HA  GLU P 179       7.326 -13.167  23.852  0.00  0.00      PROA
ATOM   2886  CB  GLU P 179       8.658 -14.002  25.375  0.00  0.00      PROA
ATOM   2887  HB1 GLU P 179       7.794 -14.479  25.884  0.00  0.00      PROA
ATOM   2888  HB2 GLU P 179       9.309 -13.646  26.201  0.00  0.00      PROA
ATOM   2889  CG  GLU P 179       9.370 -15.064  24.519  0.00  0.00      PROA
ATOM   2890  HG1 GLU P 179      10.227 -14.664  23.935  0.00  0.00      PROA
ATOM   2891  HG2 GLU P 179       8.698 -15.435  23.715  0.00  0.00      PROA
ATOM   2892  CD  GLU P 179       9.987 -16.218  25.281  0.00  0.00      PROA
ATOM   2893  OE1 GLU P 179      10.931 -15.977  26.097  0.00  0.00      PROA
ATOM   2894  OE2 GLU P 179       9.528 -17.378  25.202  0.00  0.00      PROA
ATOM   2895  C   GLU P 179       7.208 -11.982  25.506  0.00  0.00      PROA
ATOM   2896  O   GLU P 179       7.698 -11.182  26.321  0.00  0.00      PROA
ATOM   2897  N   ALA P 180       5.893 -12.032  25.285  0.00  0.00      PROA
ATOM   2898  HN  ALA P 180       5.591 -12.649  24.562  0.00  0.00      PROA
ATOM   2899  CA  ALA P 180       4.947 -11.296  26.094  0.00  0.00      PROA
ATOM   2900  HA  ALA P 180       5.470 -10.792  26.894  0.00  0.00      PROA
ATOM   2901  CB  ALA P 180       4.291 -10.256  25.215  0.00  0.00      PROA
ATOM   2902  HB1 ALA P 180       4.004 -10.622  24.205  0.00  0.00      PROA
ATOM   2903  HB2 ALA P 180       4.960  -9.400  24.985  0.00  0.00      PROA
ATOM   2904  HB3 ALA P 180       3.309  -9.978  25.655  0.00  0.00      PROA
ATOM   2905  C   ALA P 180       3.886 -12.317  26.665  0.00  0.00      PROA
ATOM   2906  O   ALA P 180       3.328 -13.058  25.845  0.00  0.00      PROA
ATOM   2907  N   ASP P 181       3.686 -12.401  27.988  0.00  0.00      PROA
ATOM   2908  HN  ASP P 181       3.862 -11.594  28.546  0.00  0.00      PROA
ATOM   2909  CA  ASP P 181       2.911 -13.500  28.579  0.00  0.00      PROA
ATOM   2910  HA  ASP P 181       3.171 -13.451  29.626  0.00  0.00      PROA
ATOM   2911  CB  ASP P 181       1.410 -13.466  28.318  0.00  0.00      PROA
ATOM   2912  HB1 ASP P 181       0.928 -14.284  28.895  0.00  0.00      PROA
ATOM   2913  HB2 ASP P 181       1.176 -13.697  27.256  0.00  0.00      PROA
ATOM   2914  CG  ASP P 181       0.899 -12.127  28.730  0.00  0.00      PROA
ATOM   2915  OD1 ASP P 181       1.258 -11.657  29.820  0.00  0.00      PROA
ATOM   2916  OD2 ASP P 181       0.068 -11.500  28.022  0.00  0.00      PROA
ATOM   2917  C   ASP P 181       3.535 -14.914  28.383  0.00  0.00      PROA
ATOM   2918  O   ASP P 181       2.906 -15.932  28.449  0.00  0.00      PROA
ATOM   2919  N   ASP P 182       4.851 -14.919  28.197  0.00  0.00      PROA
ATOM   2920  HN  ASP P 182       5.357 -14.088  28.417  0.00  0.00      PROA
ATOM   2921  CA  ASP P 182       5.684 -16.036  27.833  0.00  0.00      PROA
ATOM   2922  HA  ASP P 182       6.721 -15.752  27.933  0.00  0.00      PROA
ATOM   2923  CB  ASP P 182       5.552 -17.176  28.883  0.00  0.00      PROA
```

```
ATOM   2924  HB1 ASP P 182       6.222 -17.982  28.516  0.00  0.00      PROA
ATOM   2925  HB2 ASP P 182       4.556 -17.665  28.846  0.00  0.00      PROA
ATOM   2926  CG  ASP P 182       5.950 -16.779  30.284  0.00  0.00      PROA
ATOM   2927  OD1 ASP P 182       6.736 -15.753  30.433  0.00  0.00      PROA
ATOM   2928  OD2 ASP P 182       5.611 -17.569  31.185  0.00  0.00      PROA
ATOM   2929  C   ASP P 182       5.345 -16.467  26.421  0.00  0.00      PROA
ATOM   2930  O   ASP P 182       5.639 -17.589  26.051  0.00  0.00      PROA
ATOM   2931  N   ARG P 183       4.672 -15.693  25.623  0.00  0.00      PROA
ATOM   2932  HN  ARG P 183       4.134 -14.924  25.958  0.00  0.00      PROA
ATOM   2933  CA  ARG P 183       4.414 -16.133  24.270  0.00  0.00      PROA
ATOM   2934  HA  ARG P 183       4.938 -17.049  24.040  0.00  0.00      PROA
ATOM   2935  CB  ARG P 183       2.907 -16.323  24.087  0.00  0.00      PROA
ATOM   2936  HB1 ARG P 183       2.724 -16.646  23.040  0.00  0.00      PROA
ATOM   2937  HB2 ARG P 183       2.285 -15.403  24.123  0.00  0.00      PROA
ATOM   2938  CG  ARG P 183       2.264 -17.310  25.056  0.00  0.00      PROA
ATOM   2939  HG1 ARG P 183       2.311 -16.887  26.083  0.00  0.00      PROA
ATOM   2940  HG2 ARG P 183       2.967 -18.168  24.993  0.00  0.00      PROA
ATOM   2941  CD  ARG P 183       0.808 -17.788  24.857  0.00  0.00      PROA
ATOM   2942  HD1 ARG P 183       0.558 -18.569  25.607  0.00  0.00      PROA
ATOM   2943  HD2 ARG P 183       0.762 -18.177  23.818  0.00  0.00      PROA
ATOM   2944  NE  ARG P 183      -0.128 -16.603  24.863  0.00  0.00      PROA
ATOM   2945  HE  ARG P 183      -0.503 -16.269  23.998  0.00  0.00      PROA
ATOM   2946  CZ  ARG P 183      -0.441 -15.862  25.847  0.00  0.00      PROA
ATOM   2947  NH1 ARG P 183      -0.169 -16.076  27.106  0.00  0.00      PROA
ATOM   2948 HH11 ARG P 183       0.306 -16.946  27.239  0.00  0.00      PROA
ATOM   2949 HH12 ARG P 183      -0.818 -15.732  27.784  0.00  0.00      PROA
ATOM   2950  NH2 ARG P 183      -1.237 -14.806  25.581  0.00  0.00      PROA
ATOM   2951 HH21 ARG P 183      -1.254 -14.321  24.707  0.00  0.00      PROA
ATOM   2952 HH22 ARG P 183      -1.490 -14.209  26.343  0.00  0.00      PROA
ATOM   2953  C   ARG P 183       4.949 -15.168  23.243  0.00  0.00      PROA
ATOM   2954  O   ARG P 183       4.998 -14.009  23.421  0.00  0.00      PROA
ATOM   2955  N   TYR P 184       5.612 -15.624  22.110  0.00  0.00      PROA
ATOM   2956  HN  TYR P 184       5.811 -16.599  22.045  0.00  0.00      PROA
ATOM   2957  CA  TYR P 184       6.209 -14.757  21.073  0.00  0.00      PROA
ATOM   2958  HA  TYR P 184       6.889 -14.051  21.527  0.00  0.00      PROA
ATOM   2959  CB  TYR P 184       6.873 -15.529  19.894  0.00  0.00      PROA
ATOM   2960  HB1 TYR P 184       7.219 -14.853  19.083  0.00  0.00      PROA
ATOM   2961  HB2 TYR P 184       6.140 -16.191  19.385  0.00  0.00      PROA
ATOM   2962  CG  TYR P 184       8.001 -16.318  20.311  0.00  0.00      PROA
ATOM   2963  CD1 TYR P 184       9.221 -15.615  20.555  0.00  0.00      PROA
ATOM   2964  HD1 TYR P 184       9.165 -14.566  20.304  0.00  0.00      PROA
ATOM   2965  CE1 TYR P 184      10.433 -16.348  20.973  0.00  0.00      PROA
ATOM   2966  HE1 TYR P 184      11.324 -15.793  21.227  0.00  0.00      PROA
ATOM   2967  CZ  TYR P 184      10.254 -17.726  21.177  0.00  0.00      PROA
ATOM   2968  OH  TYR P 184      11.416 -18.343  21.806  0.00  0.00      PROA
ATOM   2969  HH  TYR P 184      11.865 -17.601  22.217  0.00  0.00      PROA
ATOM   2970  CD2 TYR P 184       8.001 -17.679  20.504  0.00  0.00      PROA
ATOM   2971  HD2 TYR P 184       7.057 -18.173  20.330  0.00  0.00      PROA
ATOM   2972  CE2 TYR P 184       9.096 -18.400  20.888  0.00  0.00      PROA
ATOM   2973  HE2 TYR P 184       8.995 -19.465  21.029  0.00  0.00      PROA
ATOM   2974  C   TYR P 184       5.228 -13.719  20.447  0.00  0.00      PROA
ATOM   2975  O   TYR P 184       4.130 -14.006  19.969  0.00  0.00      PROA
ATOM   2976  N   ILE P 185       5.783 -12.488  20.326  0.00  0.00      PROA
ATOM   2977  HN  ILE P 185       6.614 -12.160  20.768  0.00  0.00      PROA
ATOM   2978  CA  ILE P 185       5.319 -11.462  19.451  0.00  0.00      PROA
ATOM   2979  HA  ILE P 185       4.413 -11.715  18.920  0.00  0.00      PROA
ATOM   2980  CB  ILE P 185       5.188 -10.229  20.257  0.00  0.00      PROA
ATOM   2981  HB  ILE P 185       6.178 -10.051  20.729  0.00  0.00      PROA
ATOM   2982  CG2 ILE P 185       4.785  -8.992  19.433  0.00  0.00      PROA
ATOM   2983 HG21 ILE P 185       3.769  -9.201  19.035  0.00  0.00      PROA
ATOM   2984 HG22 ILE P 185       5.458  -8.925  18.552  0.00  0.00      PROA
```

ATOM 2985 HG23 ILE P 185 4.885 -8.053 20.018 0.00 0.00 PROA
ATOM 2986 CG1 ILE P 185 4.249 -10.430 21.438 0.00 0.00 PROA
ATOM 2987 HG11 ILE P 185 4.149 -9.437 21.927 0.00 0.00 PROA
ATOM 2988 HG12 ILE P 185 4.741 -11.118 22.158 0.00 0.00 PROA
ATOM 2989 CD ILE P 185 2.838 -10.946 20.976 0.00 0.00 PROA
ATOM 2990 HD1 ILE P 185 2.894 -11.804 20.272 0.00 0.00 PROA
ATOM 2991 HD2 ILE P 185 2.407 -10.092 20.411 0.00 0.00 PROA
ATOM 2992 HD3 ILE P 185 2.154 -11.180 21.820 0.00 0.00 PROA
ATOM 2993 C ILE P 185 6.361 -11.179 18.387 0.00 0.00 PROA
ATOM 2994 O ILE P 185 7.570 -10.957 18.714 0.00 0.00 PROA
ATOM 2995 N CYS P 186 5.899 -11.146 17.106 0.00 0.00 PROA
ATOM 2996 HN CYS P 186 4.931 -11.308 16.934 0.00 0.00 PROA
ATOM 2997 CA CYS P 186 6.709 -10.747 15.987 0.00 0.00 PROA
ATOM 2998 HA CYS P 186 7.763 -10.711 16.219 0.00 0.00 PROA
ATOM 2999 CB CYS P 186 6.503 -11.682 14.804 0.00 0.00 PROA
ATOM 3000 HB1 CYS P 186 5.414 -11.814 14.631 0.00 0.00 PROA
ATOM 3001 HB2 CYS P 186 7.116 -12.589 14.992 0.00 0.00 PROA
ATOM 3002 SG CYS P 186 7.221 -11.040 13.225 0.00 0.00 PROA
ATOM 3003 C CYS P 186 6.220 -9.359 15.516 0.00 0.00 PROA
ATOM 3004 O CYS P 186 5.146 -9.277 14.935 0.00 0.00 PROA
ATOM 3005 N ASP P 187 6.930 -8.312 15.745 0.00 0.00 PROA
ATOM 3006 HN ASP P 187 7.871 -8.421 16.057 0.00 0.00 PROA
ATOM 3007 CA ASP P 187 6.528 -7.010 15.283 0.00 0.00 PROA
ATOM 3008 HA ASP P 187 6.069 -7.072 14.307 0.00 0.00 PROA
ATOM 3009 CB ASP P 187 5.608 -6.366 16.440 0.00 0.00 PROA
ATOM 3010 HB1 ASP P 187 6.267 -6.220 17.322 0.00 0.00 PROA
ATOM 3011 HB2 ASP P 187 4.907 -7.189 16.697 0.00 0.00 PROA
ATOM 3012 CG ASP P 187 4.841 -5.145 16.036 0.00 0.00 PROA
ATOM 3013 OD1 ASP P 187 4.044 -4.601 16.822 0.00 0.00 PROA
ATOM 3014 OD2 ASP P 187 5.097 -4.646 14.891 0.00 0.00 PROA
ATOM 3015 C ASP P 187 7.831 -6.309 15.039 0.00 0.00 PROA
ATOM 3016 O ASP P 187 8.911 -6.900 15.303 0.00 0.00 PROA
ATOM 3017 N ARG P 188 7.824 -5.045 14.572 0.00 0.00 PROA
ATOM 3018 HN ARG P 188 6.934 -4.626 14.410 0.00 0.00 PROA
ATOM 3019 CA ARG P 188 8.977 -4.192 14.427 0.00 0.00 PROA
ATOM 3020 HA ARG P 188 9.871 -4.788 14.542 0.00 0.00 PROA
ATOM 3021 CB ARG P 188 8.973 -3.313 13.147 0.00 0.00 PROA
ATOM 3022 HB1 ARG P 188 9.881 -2.682 13.039 0.00 0.00 PROA
ATOM 3023 HB2 ARG P 188 8.135 -2.586 13.075 0.00 0.00 PROA
ATOM 3024 CG ARG P 188 9.006 -4.158 11.844 0.00 0.00 PROA
ATOM 3025 HG1 ARG P 188 8.271 -4.990 11.870 0.00 0.00 PROA
ATOM 3026 HG2 ARG P 188 9.971 -4.680 11.668 0.00 0.00 PROA
ATOM 3027 CD ARG P 188 8.706 -3.293 10.585 0.00 0.00 PROA
ATOM 3028 HD1 ARG P 188 9.010 -4.000 9.784 0.00 0.00 PROA
ATOM 3029 HD2 ARG P 188 9.322 -2.382 10.430 0.00 0.00 PROA
ATOM 3030 NE ARG P 188 7.229 -3.051 10.486 0.00 0.00 PROA
ATOM 3031 HE ARG P 188 6.679 -3.535 11.168 0.00 0.00 PROA
ATOM 3032 CZ ARG P 188 6.738 -2.367 9.514 0.00 0.00 PROA
ATOM 3033 NH1 ARG P 188 7.470 -1.627 8.663 0.00 0.00 PROA
ATOM 3034 HH11 ARG P 188 8.390 -1.892 8.375 0.00 0.00 PROA
ATOM 3035 HH12 ARG P 188 6.934 -1.211 7.928 0.00 0.00 PROA
ATOM 3036 NH2 ARG P 188 5.420 -2.331 9.145 0.00 0.00 PROA
ATOM 3037 HH21 ARG P 188 4.764 -2.938 9.594 0.00 0.00 PROA
ATOM 3038 HH22 ARG P 188 5.104 -1.436 8.830 0.00 0.00 PROA
ATOM 3039 C ARG P 188 9.047 -3.246 15.598 0.00 0.00 PROA
ATOM 3040 O ARG P 188 8.141 -2.396 15.731 0.00 0.00 PROA
ATOM 3041 N PHE P 189 10.141 -3.208 16.374 0.00 0.00 PROA
ATOM 3042 HN PHE P 189 10.852 -3.899 16.275 0.00 0.00 PROA
ATOM 3043 CA PHE P 189 10.150 -2.365 17.608 0.00 0.00 PROA
ATOM 3044 HA PHE P 189 9.140 -2.147 17.924 0.00 0.00 PROA
ATOM 3045 CB PHE P 189 10.816 -3.172 18.702 0.00 0.00 PROA

ATOM	3046	HB1	PHE	P	189	10.971	-2.663	19.677	0.00	0.00	PROA
ATOM	3047	HB2	PHE	P	189	11.860	-3.454	18.447	0.00	0.00	PROA
ATOM	3048	CG	PHE	P	189	10.001	-4.422	18.888	0.00	0.00	PROA
ATOM	3049	CD1	PHE	P	189	8.769	-4.407	19.450	0.00	0.00	PROA
ATOM	3050	HD1	PHE	P	189	8.377	-3.466	19.806	0.00	0.00	PROA
ATOM	3051	CE1	PHE	P	189	7.958	-5.516	19.563	0.00	0.00	PROA
ATOM	3052	HE1	PHE	P	189	7.001	-5.390	20.048	0.00	0.00	PROA
ATOM	3053	CZ	PHE	P	189	8.428	-6.774	19.115	0.00	0.00	PROA
ATOM	3054	HZ	PHE	P	189	7.862	-7.694	19.101	0.00	0.00	PROA
ATOM	3055	CD2	PHE	P	189	10.533	-5.692	18.541	0.00	0.00	PROA
ATOM	3056	HD2	PHE	P	189	11.467	-5.748	18.002	0.00	0.00	PROA
ATOM	3057	CE2	PHE	P	189	9.787	-6.823	18.702	0.00	0.00	PROA
ATOM	3058	HE2	PHE	P	189	10.190	-7.765	18.361	0.00	0.00	PROA
ATOM	3059	C	PHE	P	189	10.875	-1.006	17.385	0.00	0.00	PROA
ATOM	3060	O	PHE	P	189	12.098	-0.926	17.384	0.00	0.00	PROA
ATOM	3061	N	TYR	P	190	10.117	0.075	17.340	0.00	0.00	PROA
ATOM	3062	HN	TYR	P	190	9.121	0.070	17.376	0.00	0.00	PROA
ATOM	3063	CA	TYR	P	190	10.635	1.467	17.165	0.00	0.00	PROA
ATOM	3064	HA	TYR	P	190	11.534	1.374	16.574	0.00	0.00	PROA
ATOM	3065	CB	TYR	P	190	9.724	2.486	16.410	0.00	0.00	PROA
ATOM	3066	HB1	TYR	P	190	10.194	3.483	16.272	0.00	0.00	PROA
ATOM	3067	HB2	TYR	P	190	8.855	2.685	17.074	0.00	0.00	PROA
ATOM	3068	CG	TYR	P	190	9.251	1.959	15.119	0.00	0.00	PROA
ATOM	3069	CD1	TYR	P	190	7.911	2.111	14.746	0.00	0.00	PROA
ATOM	3070	HD1	TYR	P	190	7.294	2.819	15.279	0.00	0.00	PROA
ATOM	3071	CE1	TYR	P	190	7.388	1.440	13.638	0.00	0.00	PROA
ATOM	3072	HE1	TYR	P	190	6.392	1.645	13.275	0.00	0.00	PROA
ATOM	3073	CZ	TYR	P	190	8.186	0.555	12.903	0.00	0.00	PROA
ATOM	3074	OH	TYR	P	190	7.551	-0.100	11.786	0.00	0.00	PROA
ATOM	3075	HH	TYR	P	190	8.270	-0.148	11.152	0.00	0.00	PROA
ATOM	3076	CD2	TYR	P	190	10.098	1.041	14.275	0.00	0.00	PROA
ATOM	3077	HD2	TYR	P	190	11.102	0.788	14.583	0.00	0.00	PROA
ATOM	3078	CE2	TYR	P	190	9.562	0.364	13.161	0.00	0.00	PROA
ATOM	3079	HE2	TYR	P	190	10.243	-0.167	12.512	0.00	0.00	PROA
ATOM	3080	C	TYR	P	190	11.349	1.980	18.498	0.00	0.00	PROA
ATOM	3081	O	TYR	P	190	10.746	1.767	19.546	0.00	0.00	PROA
ATOM	3082	N	PRO	P	191	12.508	2.629	18.527	0.00	0.00	PROA
ATOM	3083	CD	PRO	P	191	13.205	2.982	17.359	0.00	0.00	PROA
ATOM	3084	HD1	PRO	P	191	12.678	3.637	16.633	0.00	0.00	PROA
ATOM	3085	HD2	PRO	P	191	13.544	2.062	16.835	0.00	0.00	PROA
ATOM	3086	CA	PRO	P	191	13.233	2.984	19.792	0.00	0.00	PROA
ATOM	3087	HA	PRO	P	191	13.257	2.098	20.408	0.00	0.00	PROA
ATOM	3088	CB	PRO	P	191	14.596	3.419	19.332	0.00	0.00	PROA
ATOM	3089	HB1	PRO	P	191	15.426	2.681	19.377	0.00	0.00	PROA
ATOM	3090	HB2	PRO	P	191	15.034	4.347	19.758	0.00	0.00	PROA
ATOM	3091	CG	PRO	P	191	14.453	3.711	17.829	0.00	0.00	PROA
ATOM	3092	HG1	PRO	P	191	14.360	4.812	17.717	0.00	0.00	PROA
ATOM	3093	HG2	PRO	P	191	15.339	3.420	17.224	0.00	0.00	PROA
ATOM	3094	C	PRO	P	191	12.534	3.980	20.511	0.00	0.00	PROA
ATOM	3095	O	PRO	P	191	12.429	3.760	21.710	0.00	0.00	PROA
ATOM	3096	N	ASN	P	192	12.011	4.967	19.841	0.00	0.00	PROA
ATOM	3097	HN	ASN	P	192	12.159	5.013	18.856	0.00	0.00	PROA
ATOM	3098	CA	ASN	P	192	11.317	6.090	20.458	0.00	0.00	PROA
ATOM	3099	HA	ASN	P	192	10.565	5.787	21.171	0.00	0.00	PROA
ATOM	3100	CB	ASN	P	192	12.402	6.986	21.047	0.00	0.00	PROA
ATOM	3101	HB1	ASN	P	192	13.004	6.484	21.835	0.00	0.00	PROA
ATOM	3102	HB2	ASN	P	192	11.921	7.854	21.545	0.00	0.00	PROA
ATOM	3103	CG	ASN	P	192	13.409	7.659	20.064	0.00	0.00	PROA
ATOM	3104	OD1	ASN	P	192	13.266	7.716	18.854	0.00	0.00	PROA
ATOM	3105	ND2	ASN	P	192	14.352	8.373	20.703	0.00	0.00	PROA
ATOM	3106	HD21	ASN	P	192	14.759	9.180	20.275	0.00	0.00	PROA

ATOM	3107	HD22	ASN	P	192	14.510	8.275	21.686	0.00	0.00	PROA
ATOM	3108	C	ASN	P	192	10.500	6.902	19.425	0.00	0.00	PROA
ATOM	3109	O	ASN	P	192	10.660	6.753	18.208	0.00	0.00	PROA
ATOM	3110	N	ASP	P	193	9.689	7.909	19.969	0.00	0.00	PROA
ATOM	3111	HN	ASP	P	193	9.852	8.125	20.929	0.00	0.00	PROA
ATOM	3112	CA	ASP	P	193	8.825	8.857	19.190	0.00	0.00	PROA
ATOM	3113	HA	ASP	P	193	8.151	8.296	18.559	0.00	0.00	PROA
ATOM	3114	CB	ASP	P	193	7.965	9.615	20.204	0.00	0.00	PROA
ATOM	3115	HB1	ASP	P	193	7.327	8.891	20.754	0.00	0.00	PROA
ATOM	3116	HB2	ASP	P	193	7.374	10.425	19.725	0.00	0.00	PROA
ATOM	3117	CG	ASP	P	193	8.688	10.284	21.426	0.00	0.00	PROA
ATOM	3118	OD1	ASP	P	193	9.917	10.350	21.431	0.00	0.00	PROA
ATOM	3119	OD2	ASP	P	193	7.983	10.767	22.375	0.00	0.00	PROA
ATOM	3120	C	ASP	P	193	9.422	9.691	18.095	0.00	0.00	PROA
ATOM	3121	O	ASP	P	193	8.880	9.810	16.951	0.00	0.00	PROA
ATOM	3122	N	LEU	P	194	10.643	10.143	18.363	0.00	0.00	PROA
ATOM	3123	HN	LEU	P	194	10.942	10.167	19.314	0.00	0.00	PROA
ATOM	3124	CA	LEU	P	194	11.503	10.860	17.368	0.00	0.00	PROA
ATOM	3125	HA	LEU	P	194	10.835	11.656	17.073	0.00	0.00	PROA
ATOM	3126	CB	LEU	P	194	12.738	11.549	18.013	0.00	0.00	PROA
ATOM	3127	HB1	LEU	P	194	13.463	10.927	18.580	0.00	0.00	PROA
ATOM	3128	HB2	LEU	P	194	12.386	12.394	18.644	0.00	0.00	PROA
ATOM	3129	CG	LEU	P	194	13.615	12.173	16.910	0.00	0.00	PROA
ATOM	3130	HG	LEU	P	194	14.047	11.421	16.216	0.00	0.00	PROA
ATOM	3131	CD1	LEU	P	194	12.806	13.277	16.102	0.00	0.00	PROA
ATOM	3132	HD11	LEU	P	194	12.165	12.897	15.277	0.00	0.00	PROA
ATOM	3133	HD12	LEU	P	194	13.479	13.971	15.554	0.00	0.00	PROA
ATOM	3134	HD13	LEU	P	194	12.087	13.827	16.745	0.00	0.00	PROA
ATOM	3135	CD2	LEU	P	194	14.907	12.813	17.492	0.00	0.00	PROA
ATOM	3136	HD21	LEU	P	194	15.490	13.508	16.851	0.00	0.00	PROA
ATOM	3137	HD22	LEU	P	194	15.555	11.926	17.656	0.00	0.00	PROA
ATOM	3138	HD23	LEU	P	194	14.643	13.279	18.466	0.00	0.00	PROA
ATOM	3139	C	LEU	P	194	11.745	9.896	16.213	0.00	0.00	PROA
ATOM	3140	O	LEU	P	194	11.642	10.300	15.063	0.00	0.00	PROA
ATOM	3141	N	TRP	P	195	12.085	8.592	16.351	0.00	0.00	PROA
ATOM	3142	HN	TRP	P	195	12.435	8.165	17.181	0.00	0.00	PROA
ATOM	3143	CA	TRP	P	195	12.151	7.785	15.174	0.00	0.00	PROA
ATOM	3144	HA	TRP	P	195	12.783	8.318	14.480	0.00	0.00	PROA
ATOM	3145	CB	TRP	P	195	12.811	6.352	15.382	0.00	0.00	PROA
ATOM	3146	HB1	TRP	P	195	12.135	5.678	15.950	0.00	0.00	PROA
ATOM	3147	HB2	TRP	P	195	13.717	6.388	16.025	0.00	0.00	PROA
ATOM	3148	CG	TRP	P	195	13.215	5.724	14.096	0.00	0.00	PROA
ATOM	3149	CD1	TRP	P	195	14.413	5.794	13.491	0.00	0.00	PROA
ATOM	3150	HD1	TRP	P	195	15.291	6.258	13.916	0.00	0.00	PROA
ATOM	3151	NE1	TRP	P	195	14.344	5.207	12.270	0.00	0.00	PROA
ATOM	3152	HE1	TRP	P	195	15.058	5.224	11.605	0.00	0.00	PROA
ATOM	3153	CE2	TRP	P	195	13.179	4.655	12.056	0.00	0.00	PROA
ATOM	3154	CD2	TRP	P	195	12.442	4.920	13.200	0.00	0.00	PROA
ATOM	3155	CE3	TRP	P	195	11.149	4.378	13.246	0.00	0.00	PROA
ATOM	3156	HE3	TRP	P	195	10.581	4.659	14.121	0.00	0.00	PROA
ATOM	3157	CZ3	TRP	P	195	10.698	3.611	12.184	0.00	0.00	PROA
ATOM	3158	HZ3	TRP	P	195	9.705	3.188	12.222	0.00	0.00	PROA
ATOM	3159	CZ2	TRP	P	195	12.742	3.953	10.925	0.00	0.00	PROA
ATOM	3160	HZ2	TRP	P	195	13.423	3.820	10.098	0.00	0.00	PROA
ATOM	3161	CH2	TRP	P	195	11.415	3.448	11.032	0.00	0.00	PROA
ATOM	3162	HH2	TRP	P	195	11.029	2.901	10.185	0.00	0.00	PROA
ATOM	3163	C	TRP	P	195	10.834	7.598	14.401	0.00	0.00	PROA
ATOM	3164	O	TRP	P	195	10.763	7.492	13.214	0.00	0.00	PROA
ATOM	3165	N	VAL	P	196	9.703	7.497	15.147	0.00	0.00	PROA
ATOM	3166	HN	VAL	P	196	9.818	7.634	16.128	0.00	0.00	PROA
ATOM	3167	CA	VAL	P	196	8.390	7.229	14.634	0.00	0.00	PROA

ATOM	3168	HA	VAL	P	196	8.545	6.338	14.045	0.00	0.00	PROA
ATOM	3169	CB	VAL	P	196	7.333	6.864	15.603	0.00	0.00	PROA
ATOM	3170	HB	VAL	P	196	7.132	7.678	16.331	0.00	0.00	PROA
ATOM	3171	CG1	VAL	P	196	5.999	6.548	14.886	0.00	0.00	PROA
ATOM	3172	HG11	VAL	P	196	5.193	6.062	15.477	0.00	0.00	PROA
ATOM	3173	HG12	VAL	P	196	6.313	5.743	14.188	0.00	0.00	PROA
ATOM	3174	HG13	VAL	P	196	5.522	7.385	14.332	0.00	0.00	PROA
ATOM	3175	CG2	VAL	P	196	7.782	5.683	16.429	0.00	0.00	PROA
ATOM	3176	HG21	VAL	P	196	7.172	5.348	17.295	0.00	0.00	PROA
ATOM	3177	HG22	VAL	P	196	8.755	5.877	16.929	0.00	0.00	PROA
ATOM	3178	HG23	VAL	P	196	7.947	4.784	15.797	0.00	0.00	PROA
ATOM	3179	C	VAL	P	196	7.943	8.351	13.714	0.00	0.00	PROA
ATOM	3180	O	VAL	P	196	7.605	8.208	12.556	0.00	0.00	PROA
ATOM	3181	N	VAL	P	197	8.037	9.651	14.089	0.00	0.00	PROA
ATOM	3182	HN	VAL	P	197	8.486	9.867	14.953	0.00	0.00	PROA
ATOM	3183	CA	VAL	P	197	7.741	10.761	13.251	0.00	0.00	PROA
ATOM	3184	HA	VAL	P	197	6.694	10.606	13.034	0.00	0.00	PROA
ATOM	3185	CB	VAL	P	197	7.733	12.164	13.881	0.00	0.00	PROA
ATOM	3186	HB	VAL	P	197	7.441	12.929	13.131	0.00	0.00	PROA
ATOM	3187	CG1	VAL	P	197	6.601	12.134	14.988	0.00	0.00	PROA
ATOM	3188	HG11	VAL	P	197	6.632	13.080	15.570	0.00	0.00	PROA
ATOM	3189	HG12	VAL	P	197	6.790	11.327	15.727	0.00	0.00	PROA
ATOM	3190	HG13	VAL	P	197	5.621	11.901	14.519	0.00	0.00	PROA
ATOM	3191	CG2	VAL	P	197	9.125	12.564	14.575	0.00	0.00	PROA
ATOM	3192	HG21	VAL	P	197	8.975	13.469	15.202	0.00	0.00	PROA
ATOM	3193	HG22	VAL	P	197	9.884	12.707	13.777	0.00	0.00	PROA
ATOM	3194	HG23	VAL	P	197	9.516	11.804	15.285	0.00	0.00	PROA
ATOM	3195	C	VAL	P	197	8.449	10.852	11.888	0.00	0.00	PROA
ATOM	3196	O	VAL	P	197	8.019	11.159	10.790	0.00	0.00	PROA
ATOM	3197	N	VAL	P	198	9.743	10.492	11.993	0.00	0.00	PROA
ATOM	3198	HN	VAL	P	198	10.081	10.202	12.885	0.00	0.00	PROA
ATOM	3199	CA	VAL	P	198	10.760	10.513	10.990	0.00	0.00	PROA
ATOM	3200	HA	VAL	P	198	10.777	11.460	10.471	0.00	0.00	PROA
ATOM	3201	CB	VAL	P	198	12.192	10.327	11.617	0.00	0.00	PROA
ATOM	3202	HB	VAL	P	198	12.174	9.534	12.395	0.00	0.00	PROA
ATOM	3203	CG1	VAL	P	198	13.226	9.884	10.464	0.00	0.00	PROA
ATOM	3204	HG11	VAL	P	198	14.255	9.896	10.881	0.00	0.00	PROA
ATOM	3205	HG12	VAL	P	198	13.137	10.524	9.561	0.00	0.00	PROA
ATOM	3206	HG13	VAL	P	198	13.009	8.842	10.146	0.00	0.00	PROA
ATOM	3207	CG2	VAL	P	198	12.818	11.609	12.130	0.00	0.00	PROA
ATOM	3208	HG21	VAL	P	198	13.151	12.237	11.276	0.00	0.00	PROA
ATOM	3209	HG22	VAL	P	198	13.656	11.376	12.822	0.00	0.00	PROA
ATOM	3210	HG23	VAL	P	198	12.140	12.233	12.750	0.00	0.00	PROA
ATOM	3211	C	VAL	P	198	10.526	9.486	9.874	0.00	0.00	PROA
ATOM	3212	O	VAL	P	198	10.579	9.918	8.696	0.00	0.00	PROA
ATOM	3213	N	PHE	P	199	10.120	8.263	10.239	0.00	0.00	PROA
ATOM	3214	HN	PHE	P	199	10.353	8.041	11.183	0.00	0.00	PROA
ATOM	3215	CA	PHE	P	199	9.328	7.323	9.449	0.00	0.00	PROA
ATOM	3216	HA	PHE	P	199	9.786	7.177	8.482	0.00	0.00	PROA
ATOM	3217	CB	PHE	P	199	9.490	5.993	10.117	0.00	0.00	PROA
ATOM	3218	HB1	PHE	P	199	9.773	6.219	11.167	0.00	0.00	PROA
ATOM	3219	HB2	PHE	P	199	10.364	5.466	9.678	0.00	0.00	PROA
ATOM	3220	CG	PHE	P	199	8.279	5.080	10.065	0.00	0.00	PROA
ATOM	3221	CD1	PHE	P	199	7.136	5.254	10.818	0.00	0.00	PROA
ATOM	3222	HD1	PHE	P	199	6.963	6.096	11.471	0.00	0.00	PROA
ATOM	3223	CE1	PHE	P	199	6.030	4.351	10.832	0.00	0.00	PROA
ATOM	3224	HE1	PHE	P	199	5.140	4.576	11.401	0.00	0.00	PROA
ATOM	3225	CZ	PHE	P	199	6.170	3.190	10.009	0.00	0.00	PROA
ATOM	3226	HZ	PHE	P	199	5.398	2.439	10.083	0.00	0.00	PROA
ATOM	3227	CD2	PHE	P	199	8.348	3.874	9.372	0.00	0.00	PROA
ATOM	3228	HD2	PHE	P	199	9.274	3.742	8.832	0.00	0.00	PROA

ATOM	3229	CE2	PHE	P	199	7.310	2.953	9.293	0.00	0.00	PROA
ATOM	3230	HE2	PHE	P	199	7.336	2.120	8.606	0.00	0.00	PROA
ATOM	3231	C	PHE	P	199	8.017	7.758	9.056	0.00	0.00	PROA
ATOM	3232	O	PHE	P	199	7.689	7.470	7.938	0.00	0.00	PROA
ATOM	3233	N	GLN	P	200	7.097	8.379	9.843	0.00	0.00	PROA
ATOM	3234	HN	GLN	P	200	7.327	8.704	10.757	0.00	0.00	PROA
ATOM	3235	CA	GLN	P	200	5.842	8.893	9.236	0.00	0.00	PROA
ATOM	3236	HA	GLN	P	200	5.502	7.958	8.815	0.00	0.00	PROA
ATOM	3237	CB	GLN	P	200	4.956	9.521	10.355	0.00	0.00	PROA
ATOM	3238	HB1	GLN	P	200	4.132	10.128	9.924	0.00	0.00	PROA
ATOM	3239	HB2	GLN	P	200	5.603	10.253	10.885	0.00	0.00	PROA
ATOM	3240	CG	GLN	P	200	4.432	8.443	11.354	0.00	0.00	PROA
ATOM	3241	HG1	GLN	P	200	5.306	7.909	11.786	0.00	0.00	PROA
ATOM	3242	HG2	GLN	P	200	3.779	7.682	10.876	0.00	0.00	PROA
ATOM	3243	CD	GLN	P	200	3.737	9.136	12.549	0.00	0.00	PROA
ATOM	3244	OE1	GLN	P	200	4.056	10.286	12.910	0.00	0.00	PROA
ATOM	3245	NE2	GLN	P	200	2.693	8.457	13.155	0.00	0.00	PROA
ATOM	3246	HE21	GLN	P	200	2.235	8.827	13.964	0.00	0.00	PROA
ATOM	3247	HE22	GLN	P	200	2.489	7.505	12.925	0.00	0.00	PROA
ATOM	3248	C	GLN	P	200	6.010	9.797	8.051	0.00	0.00	PROA
ATOM	3249	O	GLN	P	200	5.500	9.442	6.949	0.00	0.00	PROA
ATOM	3250	N	PHE	P	201	6.831	10.852	8.158	0.00	0.00	PROA
ATOM	3251	HN	PHE	P	201	7.236	11.105	9.034	0.00	0.00	PROA
ATOM	3252	CA	PHE	P	201	7.130	11.715	7.118	0.00	0.00	PROA
ATOM	3253	HA	PHE	P	201	6.194	12.141	6.789	0.00	0.00	PROA
ATOM	3254	CB	PHE	P	201	8.105	12.848	7.626	0.00	0.00	PROA
ATOM	3255	HB1	PHE	P	201	8.963	12.403	8.174	0.00	0.00	PROA
ATOM	3256	HB2	PHE	P	201	7.486	13.465	8.311	0.00	0.00	PROA
ATOM	3257	CG	PHE	P	201	8.528	13.792	6.470	0.00	0.00	PROA
ATOM	3258	CD1	PHE	P	201	9.867	14.036	6.311	0.00	0.00	PROA
ATOM	3259	HD1	PHE	P	201	10.579	13.488	6.910	0.00	0.00	PROA
ATOM	3260	CE1	PHE	P	201	10.270	14.919	5.233	0.00	0.00	PROA
ATOM	3261	HE1	PHE	P	201	11.300	15.215	5.101	0.00	0.00	PROA
ATOM	3262	CZ	PHE	P	201	9.329	15.541	4.375	0.00	0.00	PROA
ATOM	3263	HZ	PHE	P	201	9.690	16.195	3.595	0.00	0.00	PROA
ATOM	3264	CD2	PHE	P	201	7.584	14.358	5.611	0.00	0.00	PROA
ATOM	3265	HD2	PHE	P	201	6.538	14.112	5.724	0.00	0.00	PROA
ATOM	3266	CE2	PHE	P	201	7.975	15.132	4.544	0.00	0.00	PROA
ATOM	3267	HE2	PHE	P	201	7.241	15.468	3.826	0.00	0.00	PROA
ATOM	3268	C	PHE	P	201	7.798	10.953	5.928	0.00	0.00	PROA
ATOM	3269	O	PHE	P	201	7.312	11.144	4.841	0.00	0.00	PROA
ATOM	3270	N	GLN	P	202	8.734	10.027	6.171	0.00	0.00	PROA
ATOM	3271	HN	GLN	P	202	9.066	9.870	7.097	0.00	0.00	PROA
ATOM	3272	CA	GLN	P	202	9.330	9.258	5.119	0.00	0.00	PROA
ATOM	3273	HA	GLN	P	202	9.650	9.907	4.318	0.00	0.00	PROA
ATOM	3274	CB	GLN	P	202	10.575	8.528	5.778	0.00	0.00	PROA
ATOM	3275	HB1	GLN	P	202	10.188	7.780	6.503	0.00	0.00	PROA
ATOM	3276	HB2	GLN	P	202	11.165	9.245	6.387	0.00	0.00	PROA
ATOM	3277	CG	GLN	P	202	11.584	7.809	4.817	0.00	0.00	PROA
ATOM	3278	HG1	GLN	P	202	12.656	7.799	5.109	0.00	0.00	PROA
ATOM	3279	HG2	GLN	P	202	11.578	8.381	3.864	0.00	0.00	PROA
ATOM	3280	CD	GLN	P	202	11.213	6.406	4.497	0.00	0.00	PROA
ATOM	3281	OE1	GLN	P	202	11.388	5.498	5.254	0.00	0.00	PROA
ATOM	3282	NE2	GLN	P	202	10.666	6.077	3.304	0.00	0.00	PROA
ATOM	3283	HE21	GLN	P	202	10.370	5.126	3.218	0.00	0.00	PROA
ATOM	3284	HE22	GLN	P	202	10.231	6.813	2.786	0.00	0.00	PROA
ATOM	3285	C	GLN	P	202	8.449	8.260	4.427	0.00	0.00	PROA
ATOM	3286	O	GLN	P	202	8.445	8.213	3.225	0.00	0.00	PROA
ATOM	3287	N	HSD	P	203	7.481	7.674	5.205	0.00	0.00	PROA
ATOM	3288	HN	HSD	P	203	7.392	7.846	6.183	0.00	0.00	PROA
ATOM	3289	CA	HSD	P	203	6.494	6.905	4.585	0.00	0.00	PROA

ATOM	3290	HA	HSD	P	203	7.002	6.110	4.060	0.00	0.00	PROA
ATOM	3291	CB	HSD	P	203	5.718	6.227	5.700	0.00	0.00	PROA
ATOM	3292	HB1	HSD	P	203	5.238	7.015	6.319	0.00	0.00	PROA
ATOM	3293	HB2	HSD	P	203	6.525	5.666	6.218	0.00	0.00	PROA
ATOM	3294	ND1	HSD	P	203	4.493	4.641	4.041	0.00	0.00	PROA
ATOM	3295	HD1	HSD	P	203	5.038	4.859	3.231	0.00	0.00	PROA
ATOM	3296	CG	HSD	P	203	4.666	5.274	5.265	0.00	0.00	PROA
ATOM	3297	CE1	HSD	P	203	3.450	3.791	4.089	0.00	0.00	PROA
ATOM	3298	HE1	HSD	P	203	3.054	3.380	3.160	0.00	0.00	PROA
ATOM	3299	NE2	HSD	P	203	2.911	3.801	5.271	0.00	0.00	PROA
ATOM	3300	CD2	HSD	P	203	3.712	4.735	6.049	0.00	0.00	PROA
ATOM	3301	HD2	HSD	P	203	3.683	4.731	7.132	0.00	0.00	PROA
ATOM	3302	C	HSD	P	203	5.542	7.595	3.624	0.00	0.00	PROA
ATOM	3303	O	HSD	P	203	5.439	7.049	2.518	0.00	0.00	PROA
ATOM	3304	N	ILE	P	204	5.004	8.755	3.930	0.00	0.00	PROA
ATOM	3305	HN	ILE	P	204	5.252	9.398	4.651	0.00	0.00	PROA
ATOM	3306	CA	ILE	P	204	4.069	9.410	3.087	0.00	0.00	PROA
ATOM	3307	HA	ILE	P	204	3.319	8.707	2.755	0.00	0.00	PROA
ATOM	3308	CB	ILE	P	204	3.160	10.457	3.801	0.00	0.00	PROA
ATOM	3309	HB	ILE	P	204	2.664	9.950	4.656	0.00	0.00	PROA
ATOM	3310	CG2	ILE	P	204	4.050	11.555	4.558	0.00	0.00	PROA
ATOM	3311	HG21	ILE	P	204	4.952	11.161	5.075	0.00	0.00	PROA
ATOM	3312	HG22	ILE	P	204	3.422	11.910	5.403	0.00	0.00	PROA
ATOM	3313	HG23	ILE	P	204	4.339	12.369	3.859	0.00	0.00	PROA
ATOM	3314	CG1	ILE	P	204	2.140	10.990	2.799	0.00	0.00	PROA
ATOM	3315	HG11	ILE	P	204	1.769	10.115	2.224	0.00	0.00	PROA
ATOM	3316	HG12	ILE	P	204	2.600	11.708	2.086	0.00	0.00	PROA
ATOM	3317	CD	ILE	P	204	0.887	11.644	3.441	0.00	0.00	PROA
ATOM	3318	HD1	ILE	P	204	1.246	12.445	4.122	0.00	0.00	PROA
ATOM	3319	HD2	ILE	P	204	0.365	10.942	4.126	0.00	0.00	PROA
ATOM	3320	HD3	ILE	P	204	0.126	12.072	2.754	0.00	0.00	PROA
ATOM	3321	C	ILE	P	204	4.788	9.934	1.847	0.00	0.00	PROA
ATOM	3322	O	ILE	P	204	4.301	9.921	0.723	0.00	0.00	PROA
ATOM	3323	N	MET	P	205	6.050	10.375	2.027	0.00	0.00	PROA
ATOM	3324	HN	MET	P	205	6.473	10.404	2.929	0.00	0.00	PROA
ATOM	3325	CA	MET	P	205	6.891	10.837	0.980	0.00	0.00	PROA
ATOM	3326	HA	MET	P	205	6.407	11.602	0.391	0.00	0.00	PROA
ATOM	3327	CB	MET	P	205	8.262	11.518	1.535	0.00	0.00	PROA
ATOM	3328	HB1	MET	P	205	8.875	11.648	0.618	0.00	0.00	PROA
ATOM	3329	HB2	MET	P	205	8.725	10.762	2.205	0.00	0.00	PROA
ATOM	3330	CG	MET	P	205	8.114	12.916	2.173	0.00	0.00	PROA
ATOM	3331	HG1	MET	P	205	9.066	13.181	2.680	0.00	0.00	PROA
ATOM	3332	HG2	MET	P	205	7.404	12.690	2.997	0.00	0.00	PROA
ATOM	3333	SD	MET	P	205	7.569	14.295	0.993	0.00	0.00	PROA
ATOM	3334	CE	MET	P	205	9.048	14.900	0.258	0.00	0.00	PROA
ATOM	3335	HE1	MET	P	205	8.858	15.611	-0.575	0.00	0.00	PROA
ATOM	3336	HE2	MET	P	205	9.741	14.067	0.013	0.00	0.00	PROA
ATOM	3337	HE3	MET	P	205	9.537	15.619	0.950	0.00	0.00	PROA
ATOM	3338	C	MET	P	205	7.190	9.769	-0.117	0.00	0.00	PROA
ATOM	3339	O	MET	P	205	7.114	10.094	-1.282	0.00	0.00	PROA
ATOM	3340	N	VAL	P	206	7.460	8.561	0.311	0.00	0.00	PROA
ATOM	3341	HN	VAL	P	206	7.569	8.472	1.298	0.00	0.00	PROA
ATOM	3342	CA	VAL	P	206	7.837	7.535	-0.631	0.00	0.00	PROA
ATOM	3343	HA	VAL	P	206	8.308	7.972	-1.498	0.00	0.00	PROA
ATOM	3344	CB	VAL	P	206	8.855	6.564	-0.061	0.00	0.00	PROA
ATOM	3345	HB	VAL	P	206	8.509	6.427	0.986	0.00	0.00	PROA
ATOM	3346	CG1	VAL	P	206	8.863	5.162	-0.755	0.00	0.00	PROA
ATOM	3347	HG11	VAL	P	206	9.462	4.444	-0.156	0.00	0.00	PROA
ATOM	3348	HG12	VAL	P	206	9.367	5.323	-1.732	0.00	0.00	PROA
ATOM	3349	HG13	VAL	P	206	7.958	4.524	-0.842	0.00	0.00	PROA
ATOM	3350	CG2	VAL	P	206	10.221	7.362	-0.046	0.00	0.00	PROA

```
ATOM   3351 HG21 VAL P 206      10.027   8.347   0.429  0.00  0.00      PROA
ATOM   3352 HG22 VAL P 206      10.593   7.351  -1.094  0.00  0.00      PROA
ATOM   3353 HG23 VAL P 206      11.014   6.862   0.550  0.00  0.00      PROA
ATOM   3354  C   VAL P 206       6.702   6.766  -1.128  0.00  0.00      PROA
ATOM   3355  O   VAL P 206       6.559   6.329  -2.238  0.00  0.00      PROA
ATOM   3356  N   GLY P 207       5.702   6.553  -0.168  0.00  0.00      PROA
ATOM   3357  HN  GLY P 207       5.727   6.923   0.757  0.00  0.00      PROA
ATOM   3358  CA  GLY P 207       4.540   5.782  -0.588  0.00  0.00      PROA
ATOM   3359  HA1 GLY P 207       4.061   5.424   0.311  0.00  0.00      PROA
ATOM   3360  HA2 GLY P 207       4.850   5.050  -1.319  0.00  0.00      PROA
ATOM   3361  C   GLY P 207       3.470   6.555  -1.333  0.00  0.00      PROA
ATOM   3362  O   GLY P 207       2.647   6.120  -2.129  0.00  0.00      PROA
ATOM   3363  N   LEU P 208       3.524   7.918  -1.239  0.00  0.00      PROA
ATOM   3364  HN  LEU P 208       4.182   8.466  -0.729  0.00  0.00      PROA
ATOM   3365  CA  LEU P 208       2.471   8.710  -1.895  0.00  0.00      PROA
ATOM   3366  HA  LEU P 208       1.924   8.141  -2.633  0.00  0.00      PROA
ATOM   3367  CB  LEU P 208       1.506   9.359  -0.854  0.00  0.00      PROA
ATOM   3368  HB1 LEU P 208       2.008   9.803   0.032  0.00  0.00      PROA
ATOM   3369  HB2 LEU P 208       0.977   8.504  -0.381  0.00  0.00      PROA
ATOM   3370  CG  LEU P 208       0.322  10.269  -1.346  0.00  0.00      PROA
ATOM   3371  HG  LEU P 208       0.778  11.220  -1.696  0.00  0.00      PROA
ATOM   3372  CD1 LEU P 208      -0.512   9.484  -2.373  0.00  0.00      PROA
ATOM   3373 HD11 LEU P 208      -0.934   8.637  -1.790  0.00  0.00      PROA
ATOM   3374 HD12 LEU P 208       0.066   9.143  -3.258  0.00  0.00      PROA
ATOM   3375 HD13 LEU P 208      -1.337  10.094  -2.799  0.00  0.00      PROA
ATOM   3376  CD2 LEU P 208      -0.504  10.577  -0.145  0.00  0.00      PROA
ATOM   3377 HD21 LEU P 208       0.011  11.009   0.739  0.00  0.00      PROA
ATOM   3378 HD22 LEU P 208      -1.126   9.716   0.184  0.00  0.00      PROA
ATOM   3379 HD23 LEU P 208      -1.325  11.276  -0.413  0.00  0.00      PROA
ATOM   3380  C   LEU P 208       3.106   9.771  -2.650  0.00  0.00      PROA
ATOM   3381  O   LEU P 208       2.802   9.930  -3.849  0.00  0.00      PROA
ATOM   3382  N   ILE P 209       3.979  10.631  -2.044  0.00  0.00      PROA
ATOM   3383  HN  ILE P 209       4.269  10.560  -1.093  0.00  0.00      PROA
ATOM   3384  CA  ILE P 209       4.360  11.919  -2.674  0.00  0.00      PROA
ATOM   3385  HA  ILE P 209       3.416  12.317  -3.017  0.00  0.00      PROA
ATOM   3386  CB  ILE P 209       4.813  13.050  -1.730  0.00  0.00      PROA
ATOM   3387  HB  ILE P 209       5.751  12.756  -1.213  0.00  0.00      PROA
ATOM   3388  CG2 ILE P 209       5.131  14.397  -2.425  0.00  0.00      PROA
ATOM   3389 HG21 ILE P 209       4.213  14.724  -2.958  0.00  0.00      PROA
ATOM   3390 HG22 ILE P 209       5.972  14.468  -3.148  0.00  0.00      PROA
ATOM   3391 HG23 ILE P 209       5.382  15.099  -1.601  0.00  0.00      PROA
ATOM   3392  CG1 ILE P 209       3.639  13.103  -0.694  0.00  0.00      PROA
ATOM   3393 HG11 ILE P 209       3.386  12.112  -0.260  0.00  0.00      PROA
ATOM   3394 HG12 ILE P 209       2.745  13.356  -1.302  0.00  0.00      PROA
ATOM   3395  CD  ILE P 209       3.898  14.188   0.382  0.00  0.00      PROA
ATOM   3396  HD1 ILE P 209       4.730  13.879   1.051  0.00  0.00      PROA
ATOM   3397  HD2 ILE P 209       3.012  14.420   1.010  0.00  0.00      PROA
ATOM   3398  HD3 ILE P 209       4.205  15.178  -0.018  0.00  0.00      PROA
ATOM   3399  C   ILE P 209       5.186  11.842  -3.939  0.00  0.00      PROA
ATOM   3400  O   ILE P 209       4.727  12.303  -5.044  0.00  0.00      PROA
ATOM   3401  N   LEU P 210       6.392  11.223  -3.915  0.00  0.00      PROA
ATOM   3402  HN  LEU P 210       6.484  10.709  -3.066  0.00  0.00      PROA
ATOM   3403  CA  LEU P 210       7.292  10.957  -5.007  0.00  0.00      PROA
ATOM   3404  HA  LEU P 210       7.653  11.875  -5.448  0.00  0.00      PROA
ATOM   3405  CB  LEU P 210       8.586  10.219  -4.533  0.00  0.00      PROA
ATOM   3406  HB1 LEU P 210       9.280  10.028  -5.379  0.00  0.00      PROA
ATOM   3407  HB2 LEU P 210       8.310   9.345  -3.905  0.00  0.00      PROA
ATOM   3408  CG  LEU P 210       9.419  11.108  -3.570  0.00  0.00      PROA
ATOM   3409  HG  LEU P 210       8.795  11.345  -2.683  0.00  0.00      PROA
ATOM   3410  CD1 LEU P 210      10.617  10.248  -3.063  0.00  0.00      PROA
ATOM   3411 HD11 LEU P 210      11.069   9.734  -3.938  0.00  0.00      PROA
```

```
ATOM   3412 HD12 LEU P 210      10.341   9.486  -2.304  0.00  0.00      PROA
ATOM   3413 HD13 LEU P 210      11.244  10.970  -2.498  0.00  0.00      PROA
ATOM   3414  CD2 LEU P 210      10.215  12.314  -4.093  0.00  0.00      PROA
ATOM   3415 HD21 LEU P 210       9.602  12.851  -4.847  0.00  0.00      PROA
ATOM   3416 HD22 LEU P 210      11.075  11.923  -4.678  0.00  0.00      PROA
ATOM   3417 HD23 LEU P 210      10.549  13.099  -3.381  0.00  0.00      PROA
ATOM   3418  C   LEU P 210       6.861  10.171  -6.235  0.00  0.00      PROA
ATOM   3419  O   LEU P 210       7.164  10.648  -7.338  0.00  0.00      PROA
ATOM   3420  N   PRO P 211       6.115   9.067  -6.189  0.00  0.00      PROA
ATOM   3421  CD  PRO P 211       5.970   8.328  -4.987  0.00  0.00      PROA
ATOM   3422  HD1 PRO P 211       5.436   8.817  -4.144  0.00  0.00      PROA
ATOM   3423  HD2 PRO P 211       6.946   7.848  -4.760  0.00  0.00      PROA
ATOM   3424  CA  PRO P 211       5.448   8.481  -7.373  0.00  0.00      PROA
ATOM   3425  HA  PRO P 211       6.154   8.396  -8.186  0.00  0.00      PROA
ATOM   3426  CB  PRO P 211       5.191   7.109  -6.895  0.00  0.00      PROA
ATOM   3427  HB1 PRO P 211       6.053   6.428  -7.062  0.00  0.00      PROA
ATOM   3428  HB2 PRO P 211       4.271   6.611  -7.268  0.00  0.00      PROA
ATOM   3429  CG  PRO P 211       4.992   7.284  -5.404  0.00  0.00      PROA
ATOM   3430  HG1 PRO P 211       3.975   7.682  -5.198  0.00  0.00      PROA
ATOM   3431  HG2 PRO P 211       5.102   6.365  -4.789  0.00  0.00      PROA
ATOM   3432  C   PRO P 211       4.250   9.359  -7.782  0.00  0.00      PROA
ATOM   3433  O   PRO P 211       3.919   9.370  -8.937  0.00  0.00      PROA
ATOM   3434  N   GLY P 212       3.510   9.970  -6.838  0.00  0.00      PROA
ATOM   3435  HN  GLY P 212       3.710   9.740  -5.889  0.00  0.00      PROA
ATOM   3436  CA  GLY P 212       2.323  10.766  -7.016  0.00  0.00      PROA
ATOM   3437  HA1 GLY P 212       1.835  11.039  -6.093  0.00  0.00      PROA
ATOM   3438  HA2 GLY P 212       1.680  10.097  -7.568  0.00  0.00      PROA
ATOM   3439  C   GLY P 212       2.592  11.960  -7.861  0.00  0.00      PROA
ATOM   3440  O   GLY P 212       1.775  12.267  -8.732  0.00  0.00      PROA
ATOM   3441  N   ILE P 213       3.675  12.674  -7.684  0.00  0.00      PROA
ATOM   3442  HN  ILE P 213       4.264  12.371  -6.939  0.00  0.00      PROA
ATOM   3443  CA  ILE P 213       4.081  13.779  -8.654  0.00  0.00      PROA
ATOM   3444  HA  ILE P 213       3.277  14.496  -8.733  0.00  0.00      PROA
ATOM   3445  CB  ILE P 213       5.296  14.696  -8.288  0.00  0.00      PROA
ATOM   3446  HB  ILE P 213       5.534  15.260  -9.215  0.00  0.00      PROA
ATOM   3447  CG2 ILE P 213       4.679  15.618  -7.285  0.00  0.00      PROA
ATOM   3448 HG21 ILE P 213       4.024  16.371  -7.774  0.00  0.00      PROA
ATOM   3449 HG22 ILE P 213       5.453  16.228  -6.773  0.00  0.00      PROA
ATOM   3450 HG23 ILE P 213       4.039  14.987  -6.632  0.00  0.00      PROA
ATOM   3451  CG1 ILE P 213       6.515  13.934  -7.765  0.00  0.00      PROA
ATOM   3452 HG11 ILE P 213       6.755  13.071  -8.423  0.00  0.00      PROA
ATOM   3453 HG12 ILE P 213       6.357  13.399  -6.804  0.00  0.00      PROA
ATOM   3454  CD  ILE P 213       7.835  14.679  -7.769  0.00  0.00      PROA
ATOM   3455  HD1 ILE P 213       7.767  15.567  -7.106  0.00  0.00      PROA
ATOM   3456  HD2 ILE P 213       7.863  15.199  -8.750  0.00  0.00      PROA
ATOM   3457  HD3 ILE P 213       8.743  14.132  -7.436  0.00  0.00      PROA
ATOM   3458  C   ILE P 213       4.304  13.191 -10.065  0.00  0.00      PROA
ATOM   3459  O   ILE P 213       3.942  13.803 -11.032  0.00  0.00      PROA
ATOM   3460  N   VAL P 214       4.990  12.064 -10.316  0.00  0.00      PROA
ATOM   3461  HN  VAL P 214       5.474  11.474  -9.675  0.00  0.00      PROA
ATOM   3462  CA  VAL P 214       5.229  11.465 -11.626  0.00  0.00      PROA
ATOM   3463  HA  VAL P 214       5.644  12.272 -12.212  0.00  0.00      PROA
ATOM   3464  CB  VAL P 214       6.196  10.237 -11.534  0.00  0.00      PROA
ATOM   3465  HB  VAL P 214       5.759   9.519 -10.807  0.00  0.00      PROA
ATOM   3466  CG1 VAL P 214       6.191   9.566 -12.892  0.00  0.00      PROA
ATOM   3467 HG11 VAL P 214       6.618  10.176 -13.717  0.00  0.00      PROA
ATOM   3468 HG12 VAL P 214       5.208   9.200 -13.258  0.00  0.00      PROA
ATOM   3469 HG13 VAL P 214       6.844   8.680 -12.742  0.00  0.00      PROA
ATOM   3470  CG2 VAL P 214       7.604  10.716 -11.212  0.00  0.00      PROA
ATOM   3471 HG21 VAL P 214       8.045  10.047 -10.443  0.00  0.00      PROA
ATOM   3472 HG22 VAL P 214       7.499  11.755 -10.833  0.00  0.00      PROA
```

ATOM	3473	HG23	VAL	P	214	8.274	10.557	-12.084	0.00	0.00	PROA
ATOM	3474	C	VAL	P	214	3.875	11.073	-12.243	0.00	0.00	PROA
ATOM	3475	O	VAL	P	214	3.626	11.325	-13.453	0.00	0.00	PROA
ATOM	3476	N	ILE	P	215	2.903	10.480	-11.508	0.00	0.00	PROA
ATOM	3477	HN	ILE	P	215	3.245	10.100	-10.652	0.00	0.00	PROA
ATOM	3478	CA	ILE	P	215	1.575	10.089	-11.865	0.00	0.00	PROA
ATOM	3479	HA	ILE	P	215	1.675	9.498	-12.763	0.00	0.00	PROA
ATOM	3480	CB	ILE	P	215	0.757	9.165	-10.827	0.00	0.00	PROA
ATOM	3481	HB	ILE	P	215	0.513	9.691	-9.880	0.00	0.00	PROA
ATOM	3482	CG2	ILE	P	215	-0.550	8.750	-11.523	0.00	0.00	PROA
ATOM	3483	HG21	ILE	P	215	-0.356	8.622	-12.609	0.00	0.00	PROA
ATOM	3484	HG22	ILE	P	215	-1.259	9.604	-11.489	0.00	0.00	PROA
ATOM	3485	HG23	ILE	P	215	-1.039	7.848	-11.097	0.00	0.00	PROA
ATOM	3486	CG1	ILE	P	215	1.589	7.833	-10.573	0.00	0.00	PROA
ATOM	3487	HG11	ILE	P	215	2.665	8.079	-10.451	0.00	0.00	PROA
ATOM	3488	HG12	ILE	P	215	1.435	7.201	-11.474	0.00	0.00	PROA
ATOM	3489	CD	ILE	P	215	1.023	7.035	-9.397	0.00	0.00	PROA
ATOM	3490	HD1	ILE	P	215	0.219	6.323	-9.680	0.00	0.00	PROA
ATOM	3491	HD2	ILE	P	215	0.578	7.652	-8.587	0.00	0.00	PROA
ATOM	3492	HD3	ILE	P	215	1.826	6.411	-8.948	0.00	0.00	PROA
ATOM	3493	C	ILE	P	215	0.811	11.252	-12.435	0.00	0.00	PROA
ATOM	3494	O	ILE	P	215	0.242	11.343	-13.584	0.00	0.00	PROA
ATOM	3495	N	LEU	P	216	0.849	12.386	-11.638	0.00	0.00	PROA
ATOM	3496	HN	LEU	P	216	1.237	12.340	-10.721	0.00	0.00	PROA
ATOM	3497	CA	LEU	P	216	0.013	13.560	-11.964	0.00	0.00	PROA
ATOM	3498	HA	LEU	P	216	-0.963	13.231	-12.291	0.00	0.00	PROA
ATOM	3499	CB	LEU	P	216	0.041	14.701	-10.783	0.00	0.00	PROA
ATOM	3500	HB1	LEU	P	216	1.121	14.817	-10.550	0.00	0.00	PROA
ATOM	3501	HB2	LEU	P	216	-0.515	14.284	-9.916	0.00	0.00	PROA
ATOM	3502	CG	LEU	P	216	-0.593	16.033	-11.060	0.00	0.00	PROA
ATOM	3503	HG	LEU	P	216	-0.120	16.557	-11.918	0.00	0.00	PROA
ATOM	3504	CD1	LEU	P	216	-2.077	16.004	-11.301	0.00	0.00	PROA
ATOM	3505	HD11	LEU	P	216	-2.382	17.072	-11.252	0.00	0.00	PROA
ATOM	3506	HD12	LEU	P	216	-2.719	15.508	-10.542	0.00	0.00	PROA
ATOM	3507	HD13	LEU	P	216	-2.242	15.603	-12.324	0.00	0.00	PROA
ATOM	3508	CD2	LEU	P	216	-0.272	16.937	-9.831	0.00	0.00	PROA
ATOM	3509	HD21	LEU	P	216	-0.767	17.921	-9.977	0.00	0.00	PROA
ATOM	3510	HD22	LEU	P	216	0.832	16.995	-9.722	0.00	0.00	PROA
ATOM	3511	HD23	LEU	P	216	-0.605	16.435	-8.897	0.00	0.00	PROA
ATOM	3512	C	LEU	P	216	0.674	14.197	-13.199	0.00	0.00	PROA
ATOM	3513	O	LEU	P	216	-0.028	14.767	-14.047	0.00	0.00	PROA
ATOM	3514	N	SER	P	217	2.020	14.191	-13.300	0.00	0.00	PROA
ATOM	3515	HN	SER	P	217	2.480	13.743	-12.537	0.00	0.00	PROA
ATOM	3516	CA	SER	P	217	2.760	14.917	-14.397	0.00	0.00	PROA
ATOM	3517	HA	SER	P	217	2.365	15.922	-14.440	0.00	0.00	PROA
ATOM	3518	CB	SER	P	217	4.278	15.051	-14.171	0.00	0.00	PROA
ATOM	3519	HB1	SER	P	217	4.785	15.459	-15.071	0.00	0.00	PROA
ATOM	3520	HB2	SER	P	217	4.707	14.040	-14.002	0.00	0.00	PROA
ATOM	3521	OG	SER	P	217	4.610	15.874	-12.984	0.00	0.00	PROA
ATOM	3522	HG1	SER	P	217	4.371	15.318	-12.239	0.00	0.00	PROA
ATOM	3523	C	SER	P	217	2.539	14.265	-15.727	0.00	0.00	PROA
ATOM	3524	O	SER	P	217	2.293	14.839	-16.746	0.00	0.00	PROA
ATOM	3525	N	CYS	P	218	2.500	12.913	-15.682	0.00	0.00	PROA
ATOM	3526	HN	CYS	P	218	2.719	12.461	-14.821	0.00	0.00	PROA
ATOM	3527	CA	CYS	P	218	2.226	12.092	-16.881	0.00	0.00	PROA
ATOM	3528	HA	CYS	P	218	2.776	12.564	-17.682	0.00	0.00	PROA
ATOM	3529	CB	CYS	P	218	2.500	10.569	-16.873	0.00	0.00	PROA
ATOM	3530	HB1	CYS	P	218	2.026	10.002	-17.703	0.00	0.00	PROA
ATOM	3531	HB2	CYS	P	218	2.043	10.060	-15.998	0.00	0.00	PROA
ATOM	3532	SG	CYS	P	218	4.297	10.361	-16.934	0.00	0.00	PROA
ATOM	3533	HG1	CYS	P	218	4.391	11.223	-15.931	0.00	0.00	PROA

ATOM	3534	C	CYS	P	218	0.842	12.372	-17.402	0.00	0.00	PROA
ATOM	3535	O	CYS	P	218	0.648	12.678	-18.590	0.00	0.00	PROA
ATOM	3536	N	TYR	P	219	-0.191	12.301	-16.591	0.00	0.00	PROA
ATOM	3537	HN	TYR	P	219	-0.011	12.067	-15.638	0.00	0.00	PROA
ATOM	3538	CA	TYR	P	219	-1.608	12.721	-16.899	0.00	0.00	PROA
ATOM	3539	HA	TYR	P	219	-1.928	12.076	-17.703	0.00	0.00	PROA
ATOM	3540	CB	TYR	P	219	-2.498	12.473	-15.621	0.00	0.00	PROA
ATOM	3541	HB1	TYR	P	219	-3.412	13.105	-15.614	0.00	0.00	PROA
ATOM	3542	HB2	TYR	P	219	-1.951	12.651	-14.670	0.00	0.00	PROA
ATOM	3543	CG	TYR	P	219	-3.079	11.081	-15.645	0.00	0.00	PROA
ATOM	3544	CD1	TYR	P	219	-2.813	10.220	-14.523	0.00	0.00	PROA
ATOM	3545	HD1	TYR	P	219	-2.217	10.671	-13.744	0.00	0.00	PROA
ATOM	3546	CE1	TYR	P	219	-3.200	8.927	-14.605	0.00	0.00	PROA
ATOM	3547	HE1	TYR	P	219	-2.970	8.263	-13.784	0.00	0.00	PROA
ATOM	3548	CZ	TYR	P	219	-3.740	8.332	-15.732	0.00	0.00	PROA
ATOM	3549	OH	TYR	P	219	-3.864	6.917	-15.848	0.00	0.00	PROA
ATOM	3550	HH	TYR	P	219	-3.126	6.512	-15.386	0.00	0.00	PROA
ATOM	3551	CD2	TYR	P	219	-3.756	10.546	-16.737	0.00	0.00	PROA
ATOM	3552	HD2	TYR	P	219	-3.917	11.210	-17.573	0.00	0.00	PROA
ATOM	3553	CE2	TYR	P	219	-4.110	9.213	-16.771	0.00	0.00	PROA
ATOM	3554	HE2	TYR	P	219	-4.689	8.769	-17.567	0.00	0.00	PROA
ATOM	3555	C	TYR	P	219	-1.845	14.046	-17.371	0.00	0.00	PROA
ATOM	3556	O	TYR	P	219	-2.622	14.163	-18.294	0.00	0.00	PROA
ATOM	3557	N	CYS	P	220	-1.234	15.093	-16.766	0.00	0.00	PROA
ATOM	3558	HN	CYS	P	220	-0.608	14.962	-16.001	0.00	0.00	PROA
ATOM	3559	CA	CYS	P	220	-1.528	16.395	-17.211	0.00	0.00	PROA
ATOM	3560	HA	CYS	P	220	-2.594	16.548	-17.138	0.00	0.00	PROA
ATOM	3561	CB	CYS	P	220	-0.913	17.462	-16.243	0.00	0.00	PROA
ATOM	3562	HB1	CYS	P	220	-0.910	18.402	-16.835	0.00	0.00	PROA
ATOM	3563	HB2	CYS	P	220	0.119	17.144	-15.983	0.00	0.00	PROA
ATOM	3564	SG	CYS	P	220	-1.991	17.463	-14.752	0.00	0.00	PROA
ATOM	3565	HG1	CYS	P	220	-2.944	18.231	-15.258	0.00	0.00	PROA
ATOM	3566	C	CYS	P	220	-1.108	16.641	-18.635	0.00	0.00	PROA
ATOM	3567	O	CYS	P	220	-1.820	17.238	-19.449	0.00	0.00	PROA
ATOM	3568	N	ILE	P	221	0.047	16.124	-19.114	0.00	0.00	PROA
ATOM	3569	HN	ILE	P	221	0.675	15.712	-18.459	0.00	0.00	PROA
ATOM	3570	CA	ILE	P	221	0.505	16.077	-20.588	0.00	0.00	PROA
ATOM	3571	HA	ILE	P	221	0.565	17.075	-20.997	0.00	0.00	PROA
ATOM	3572	CB	ILE	P	221	1.823	15.453	-20.782	0.00	0.00	PROA
ATOM	3573	HB	ILE	P	221	1.838	14.484	-20.238	0.00	0.00	PROA
ATOM	3574	CG2	ILE	P	221	2.086	15.120	-22.252	0.00	0.00	PROA
ATOM	3575	HG21	ILE	P	221	2.180	16.085	-22.795	0.00	0.00	PROA
ATOM	3576	HG22	ILE	P	221	1.386	14.445	-22.790	0.00	0.00	PROA
ATOM	3577	HG23	ILE	P	221	3.089	14.644	-22.295	0.00	0.00	PROA
ATOM	3578	CG1	ILE	P	221	2.937	16.293	-20.212	0.00	0.00	PROA
ATOM	3579	HG11	ILE	P	221	2.630	16.850	-19.301	0.00	0.00	PROA
ATOM	3580	HG12	ILE	P	221	3.259	17.043	-20.966	0.00	0.00	PROA
ATOM	3581	CD	ILE	P	221	4.155	15.531	-19.670	0.00	0.00	PROA
ATOM	3582	HD1	ILE	P	221	4.720	16.278	-19.071	0.00	0.00	PROA
ATOM	3583	HD2	ILE	P	221	4.763	15.048	-20.464	0.00	0.00	PROA
ATOM	3584	HD3	ILE	P	221	3.920	14.814	-18.855	0.00	0.00	PROA
ATOM	3585	C	ILE	P	221	-0.477	15.231	-21.386	0.00	0.00	PROA
ATOM	3586	O	ILE	P	221	-0.920	15.749	-22.377	0.00	0.00	PROA
ATOM	3587	N	ILE	P	222	-0.897	14.029	-20.930	0.00	0.00	PROA
ATOM	3588	HN	ILE	P	222	-0.391	13.530	-20.232	0.00	0.00	PROA
ATOM	3589	CA	ILE	P	222	-1.967	13.344	-21.583	0.00	0.00	PROA
ATOM	3590	HA	ILE	P	222	-1.680	13.016	-22.571	0.00	0.00	PROA
ATOM	3591	CB	ILE	P	222	-2.278	11.979	-20.839	0.00	0.00	PROA
ATOM	3592	HB	ILE	P	222	-2.370	12.171	-19.749	0.00	0.00	PROA
ATOM	3593	CG2	ILE	P	222	-3.546	11.250	-21.340	0.00	0.00	PROA
ATOM	3594	HG21	ILE	P	222	-3.524	11.075	-22.437	0.00	0.00	PROA

```
ATOM   3595 HG22 ILE P 222      -4.475  11.847 -21.218  0.00  0.00      PROA
ATOM   3596 HG23 ILE P 222      -3.679  10.276 -20.823  0.00  0.00      PROA
ATOM   3597  CG1 ILE P 222      -1.028  11.105 -21.069  0.00  0.00      PROA
ATOM   3598 HG11 ILE P 222      -0.107  11.671 -20.811  0.00  0.00      PROA
ATOM   3599 HG12 ILE P 222      -0.913  10.839 -22.141  0.00  0.00      PROA
ATOM   3600  CD  ILE P 222      -1.130   9.729 -20.388  0.00  0.00      PROA
ATOM   3601  HD1 ILE P 222      -1.796   9.008 -20.908  0.00  0.00      PROA
ATOM   3602  HD2 ILE P 222      -1.634   9.799 -19.400  0.00  0.00      PROA
ATOM   3603  HD3 ILE P 222      -0.142   9.247 -20.232  0.00  0.00      PROA
ATOM   3604  C   ILE P 222      -3.267  14.047 -21.757  0.00  0.00      PROA
ATOM   3605  O   ILE P 222      -3.870  13.816 -22.745  0.00  0.00      PROA
ATOM   3606  N   ILE P 223      -3.695  14.850 -20.785  0.00  0.00      PROA
ATOM   3607  HN  ILE P 223      -3.213  14.990 -19.923  0.00  0.00      PROA
ATOM   3608  CA  ILE P 223      -4.788  15.766 -20.912  0.00  0.00      PROA
ATOM   3609  HA  ILE P 223      -5.593  15.150 -21.285  0.00  0.00      PROA
ATOM   3610  CB  ILE P 223      -5.200  16.449 -19.569  0.00  0.00      PROA
ATOM   3611  HB  ILE P 223      -4.290  16.749 -19.006  0.00  0.00      PROA
ATOM   3612  CG2 ILE P 223      -6.051  17.669 -19.729  0.00  0.00      PROA
ATOM   3613 HG21 ILE P 223      -6.941  17.450 -20.357  0.00  0.00      PROA
ATOM   3614 HG22 ILE P 223      -5.474  18.501 -20.186  0.00  0.00      PROA
ATOM   3615 HG23 ILE P 223      -6.384  18.064 -18.745  0.00  0.00      PROA
ATOM   3616  CG1 ILE P 223      -5.840  15.519 -18.555  0.00  0.00      PROA
ATOM   3617 HG11 ILE P 223      -5.511  15.795 -17.530  0.00  0.00      PROA
ATOM   3618 HG12 ILE P 223      -5.441  14.495 -18.712  0.00  0.00      PROA
ATOM   3619  CD  ILE P 223      -7.351  15.459 -18.480  0.00  0.00      PROA
ATOM   3620  HD1 ILE P 223      -7.668  15.305 -19.533  0.00  0.00      PROA
ATOM   3621  HD2 ILE P 223      -7.800  16.424 -18.162  0.00  0.00      PROA
ATOM   3622  HD3 ILE P 223      -7.725  14.622 -17.852  0.00  0.00      PROA
ATOM   3623  C   ILE P 223      -4.536  16.810 -21.996  0.00  0.00      PROA
ATOM   3624  O   ILE P 223      -5.305  16.862 -22.962  0.00  0.00      PROA
ATOM   3625  N   SER P 224      -3.436  17.500 -21.847  0.00  0.00      PROA
ATOM   3626  HN  SER P 224      -2.815  17.345 -21.083  0.00  0.00      PROA
ATOM   3627  CA  SER P 224      -3.005  18.489 -22.766  0.00  0.00      PROA
ATOM   3628  HA  SER P 224      -3.690  19.322 -22.718  0.00  0.00      PROA
ATOM   3629  CB  SER P 224      -1.576  18.977 -22.425  0.00  0.00      PROA
ATOM   3630  HB1 SER P 224      -0.824  18.297 -22.878  0.00  0.00      PROA
ATOM   3631  HB2 SER P 224      -1.532  18.962 -21.315  0.00  0.00      PROA
ATOM   3632  OG  SER P 224      -1.430  20.283 -22.973  0.00  0.00      PROA
ATOM   3633  HG1 SER P 224      -1.589  20.880 -22.238  0.00  0.00      PROA
ATOM   3634  C   SER P 224      -2.950  18.036 -24.258  0.00  0.00      PROA
ATOM   3635  O   SER P 224      -3.398  18.782 -25.106  0.00  0.00      PROA
ATOM   3636  N   LYS P 225      -2.544  16.834 -24.515  0.00  0.00      PROA
ATOM   3637  HN  LYS P 225      -2.182  16.210 -23.826  0.00  0.00      PROA
ATOM   3638  CA  LYS P 225      -2.712  16.161 -25.858  0.00  0.00      PROA
ATOM   3639  HA  LYS P 225      -2.379  16.837 -26.631  0.00  0.00      PROA
ATOM   3640  CB  LYS P 225      -1.715  15.035 -25.920  0.00  0.00      PROA
ATOM   3641  HB1 LYS P 225      -1.761  14.695 -26.977  0.00  0.00      PROA
ATOM   3642  HB2 LYS P 225      -2.074  14.262 -25.207  0.00  0.00      PROA
ATOM   3643  CG  LYS P 225      -0.294  15.351 -25.693  0.00  0.00      PROA
ATOM   3644  HG1 LYS P 225       0.369  14.459 -25.709  0.00  0.00      PROA
ATOM   3645  HG2 LYS P 225      -0.111  15.873 -24.730  0.00  0.00      PROA
ATOM   3646  CD  LYS P 225       0.194  16.362 -26.767  0.00  0.00      PROA
ATOM   3647  HD1 LYS P 225      -0.264  17.367 -26.647  0.00  0.00      PROA
ATOM   3648  HD2 LYS P 225      -0.212  15.926 -27.705  0.00  0.00      PROA
ATOM   3649  CE  LYS P 225       1.694  16.605 -26.776  0.00  0.00      PROA
ATOM   3650  HE1 LYS P 225       2.305  15.679 -26.847  0.00  0.00      PROA
ATOM   3651  HE2 LYS P 225       2.055  17.107 -25.853  0.00  0.00      PROA
ATOM   3652  NZ  LYS P 225       1.970  17.551 -27.894  0.00  0.00      PROA
ATOM   3653  HZ1 LYS P 225       1.570  18.499 -27.742  0.00  0.00      PROA
ATOM   3654  HZ2 LYS P 225       1.694  17.074 -28.776  0.00  0.00      PROA
ATOM   3655  HZ3 LYS P 225       3.005  17.644 -27.855  0.00  0.00      PROA
```

ATOM	3656	C	LYS P 225	-4.140	15.799	-26.211	0.00	0.00	PROA
ATOM	3657	O	LYS P 225	-4.483	16.129	-27.341	0.00	0.00	PROA
ATOM	3658	N	LEU P 226	-4.932	15.214	-25.324	0.00	0.00	PROA
ATOM	3659	HN	LEU P 226	-4.615	15.041	-24.394	0.00	0.00	PROA
ATOM	3660	CA	LEU P 226	-6.305	14.721	-25.666	0.00	0.00	PROA
ATOM	3661	HA	LEU P 226	-6.233	14.466	-26.713	0.00	0.00	PROA
ATOM	3662	CB	LEU P 226	-6.737	13.540	-24.731	0.00	0.00	PROA
ATOM	3663	HB1	LEU P 226	-7.733	13.146	-25.025	0.00	0.00	PROA
ATOM	3664	HB2	LEU P 226	-6.702	13.832	-23.660	0.00	0.00	PROA
ATOM	3665	CG	LEU P 226	-5.805	12.260	-24.924	0.00	0.00	PROA
ATOM	3666	HG	LEU P 226	-4.860	12.463	-24.375	0.00	0.00	PROA
ATOM	3667	CD1	LEU P 226	-6.341	10.960	-24.262	0.00	0.00	PROA
ATOM	3668	HD11	LEU P 226	-6.268	10.995	-23.154	0.00	0.00	PROA
ATOM	3669	HD12	LEU P 226	-5.580	10.204	-24.551	0.00	0.00	PROA
ATOM	3670	HD13	LEU P 226	-7.393	10.703	-24.512	0.00	0.00	PROA
ATOM	3671	CD2	LEU P 226	-5.422	11.959	-26.372	0.00	0.00	PROA
ATOM	3672	HD21	LEU P 226	-6.346	11.999	-26.988	0.00	0.00	PROA
ATOM	3673	HD22	LEU P 226	-4.987	10.948	-26.522	0.00	0.00	PROA
ATOM	3674	HD23	LEU P 226	-4.694	12.696	-26.773	0.00	0.00	PROA
ATOM	3675	C	LEU P 226	-7.369	15.806	-25.748	0.00	0.00	PROA
ATOM	3676	O	LEU P 226	-8.245	15.776	-26.629	0.00	0.00	PROA
ATOM	3677	N	SER P 227	-7.135	16.992	-25.017	0.00	0.00	PROA
ATOM	3678	HN	SER P 227	-6.311	16.973	-24.456	0.00	0.00	PROA
ATOM	3679	CA	SER P 227	-8.002	18.125	-25.038	0.00	0.00	PROA
ATOM	3680	HA	SER P 227	-9.059	17.913	-25.103	0.00	0.00	PROA
ATOM	3681	CB	SER P 227	-7.863	18.909	-23.692	0.00	0.00	PROA
ATOM	3682	HB1	SER P 227	-8.019	18.270	-22.796	0.00	0.00	PROA
ATOM	3683	HB2	SER P 227	-8.610	19.731	-23.706	0.00	0.00	PROA
ATOM	3684	OG	SER P 227	-6.580	19.542	-23.672	0.00	0.00	PROA
ATOM	3685	HG1	SER P 227	-5.928	18.843	-23.580	0.00	0.00	PROA
ATOM	3686	C	SER P 227	-7.709	19.020	-26.270	0.00	0.00	PROA
ATOM	3687	O	SER P 227	-8.387	19.969	-26.623	0.00	0.00	PROA
ATOM	3688	N	HSD P 228	-6.619	18.645	-27.062	0.00	0.00	PROA
ATOM	3689	HN	HSD P 228	-6.120	17.842	-26.743	0.00	0.00	PROA
ATOM	3690	CA	HSD P 228	-6.190	19.222	-28.290	0.00	0.00	PROA
ATOM	3691	HA	HSD P 228	-6.909	20.002	-28.496	0.00	0.00	PROA
ATOM	3692	CB	HSD P 228	-4.746	19.775	-28.050	0.00	0.00	PROA
ATOM	3693	HB1	HSD P 228	-4.364	20.184	-29.010	0.00	0.00	PROA
ATOM	3694	HB2	HSD P 228	-4.126	18.898	-27.766	0.00	0.00	PROA
ATOM	3695	ND1	HSD P 228	-4.761	20.945	-25.718	0.00	0.00	PROA
ATOM	3696	HD1	HSD P 228	-4.579	20.177	-25.105	0.00	0.00	PROA
ATOM	3697	CG	HSD P 228	-4.827	20.938	-27.109	0.00	0.00	PROA
ATOM	3698	CE1	HSD P 228	-4.726	22.220	-25.319	0.00	0.00	PROA
ATOM	3699	HE1	HSD P 228	-4.665	22.483	-24.263	0.00	0.00	PROA
ATOM	3700	NE2	HSD P 228	-4.706	23.068	-26.330	0.00	0.00	PROA
ATOM	3701	CD2	HSD P 228	-4.795	22.243	-27.446	0.00	0.00	PROA
ATOM	3702	HD2	HSD P 228	-4.817	22.727	-28.415	0.00	0.00	PROA
ATOM	3703	C	HSD P 228	-6.221	18.235	-29.457	0.00	0.00	PROA
ATOM	3704	O	HSD P 228	-5.674	18.494	-30.549	0.00	0.00	PROA
ATOM	3705	N	SER P 229	-7.027	17.184	-29.240	0.00	0.00	PROA
ATOM	3706	HN	SER P 229	-7.400	17.109	-28.318	0.00	0.00	PROA
ATOM	3707	CA	SER P 229	-7.233	16.027	-30.112	0.00	0.00	PROA
ATOM	3708	HA	SER P 229	-6.572	16.148	-30.958	0.00	0.00	PROA
ATOM	3709	CB	SER P 229	-6.891	14.658	-29.452	0.00	0.00	PROA
ATOM	3710	HB1	SER P 229	-7.643	14.465	-28.658	0.00	0.00	PROA
ATOM	3711	HB2	SER P 229	-5.887	14.764	-28.989	0.00	0.00	PROA
ATOM	3712	OG	SER P 229	-6.752	13.615	-30.366	0.00	0.00	PROA
ATOM	3713	HG1	SER P 229	-5.868	13.726	-30.723	0.00	0.00	PROA
ATOM	3714	C	SER P 229	-8.676	15.947	-30.558	0.00	0.00	PROA
ATOM	3715	O	SER P 229	-9.548	16.054	-29.706	0.00	0.00	PROA
ATOM	3716	N	LYS P 230	-8.961	15.800	-31.812	0.00	0.00	PROA

```
ATOM   3717  HN  LYS P 230      -8.221  15.618 -32.454  0.00  0.00      PROA
ATOM   3718  CA  LYS P 230     -10.278  15.919 -32.427  0.00  0.00      PROA
ATOM   3719  HA  LYS P 230     -10.505  16.974 -32.371  0.00  0.00      PROA
ATOM   3720  CB  LYS P 230     -10.102  15.536 -33.873  0.00  0.00      PROA
ATOM   3721  HB1 LYS P 230     -11.054  15.661 -34.433  0.00  0.00      PROA
ATOM   3722  HB2 LYS P 230      -9.751  14.483 -33.907  0.00  0.00      PROA
ATOM   3723  CG  LYS P 230      -9.108  16.457 -34.700  0.00  0.00      PROA
ATOM   3724  HG1 LYS P 230      -8.089  16.253 -34.306  0.00  0.00      PROA
ATOM   3725  HG2 LYS P 230      -9.327  17.530 -34.513  0.00  0.00      PROA
ATOM   3726  CD  LYS P 230      -9.194  16.249 -36.257  0.00  0.00      PROA
ATOM   3727  HD1 LYS P 230      -8.317  16.801 -36.659  0.00  0.00      PROA
ATOM   3728  HD2 LYS P 230     -10.020  16.758 -36.799  0.00  0.00      PROA
ATOM   3729  CE  LYS P 230      -9.137  14.820 -36.703  0.00  0.00      PROA
ATOM   3730  HE1 LYS P 230     -10.181  14.441 -36.676  0.00  0.00      PROA
ATOM   3731  HE2 LYS P 230      -8.662  14.113 -35.989  0.00  0.00      PROA
ATOM   3732  NZ  LYS P 230      -8.686  14.642 -38.076  0.00  0.00      PROA
ATOM   3733  HZ1 LYS P 230      -8.873  13.695 -38.463  0.00  0.00      PROA
ATOM   3734  HZ2 LYS P 230      -7.675  14.845 -38.213  0.00  0.00      PROA
ATOM   3735  HZ3 LYS P 230      -9.162  15.295 -38.732  0.00  0.00      PROA
ATOM   3736  C   LYS P 230     -11.438  15.060 -31.866  0.00  0.00      PROA
ATOM   3737  O   LYS P 230     -11.262  13.942 -31.520  0.00  0.00      PROA
ATOM   3738  N   GLY P 231     -12.729  15.586 -31.842  0.00  0.00      PROA
ATOM   3739  HN  GLY P 231     -12.881  16.414 -32.377  0.00  0.00      PROA
ATOM   3740  CA  GLY P 231     -13.793  14.992 -31.090  0.00  0.00      PROA
ATOM   3741  HA1 GLY P 231     -13.919  14.005 -31.512  0.00  0.00      PROA
ATOM   3742  HA2 GLY P 231     -14.716  15.530 -31.243  0.00  0.00      PROA
ATOM   3743  C   GLY P 231     -13.669  14.917 -29.536  0.00  0.00      PROA
ATOM   3744  O   GLY P 231     -12.915  15.559 -28.853  0.00  0.00      PROA
ATOM   3745  N   HSD P 232     -14.498  14.120 -28.951  0.00  0.00      PROA
ATOM   3746  HN  HSD P 232     -15.186  13.645 -29.495  0.00  0.00      PROA
ATOM   3747  CA  HSD P 232     -14.368  13.887 -27.495  0.00  0.00      PROA
ATOM   3748  HA  HSD P 232     -13.384  14.242 -27.225  0.00  0.00      PROA
ATOM   3749  CB  HSD P 232     -15.563  14.546 -26.667  0.00  0.00      PROA
ATOM   3750  HB1 HSD P 232     -15.515  14.331 -25.578  0.00  0.00      PROA
ATOM   3751  HB2 HSD P 232     -16.512  14.200 -27.128  0.00  0.00      PROA
ATOM   3752  ND1 HSD P 232     -16.856  16.781 -26.861  0.00  0.00      PROA
ATOM   3753  HD1 HSD P 232     -17.768  16.424 -26.655  0.00  0.00      PROA
ATOM   3754  CG  HSD P 232     -15.643  16.058 -26.760  0.00  0.00      PROA
ATOM   3755  CE1 HSD P 232     -16.527  18.107 -26.881  0.00  0.00      PROA
ATOM   3756  HE1 HSD P 232     -17.277  18.891 -26.772  0.00  0.00      PROA
ATOM   3757  NE2 HSD P 232     -15.196  18.248 -26.870  0.00  0.00      PROA
ATOM   3758  CD2 HSD P 232     -14.681  16.972 -26.817  0.00  0.00      PROA
ATOM   3759  HD2 HSD P 232     -13.603  16.879 -26.863  0.00  0.00      PROA
ATOM   3760  C   HSD P 232     -14.305  12.481 -27.173  0.00  0.00      PROA
ATOM   3761  O   HSD P 232     -14.628  12.128 -26.071  0.00  0.00      PROA
ATOM   3762  N   GLN P 233     -14.028  11.574 -28.151  0.00  0.00      PROA
ATOM   3763  HN  GLN P 233     -13.690  11.838 -29.051  0.00  0.00      PROA
ATOM   3764  CA  GLN P 233     -14.070  10.121 -27.840  0.00  0.00      PROA
ATOM   3765  HA  GLN P 233     -15.021   9.901 -27.378  0.00  0.00      PROA
ATOM   3766  CB  GLN P 233     -13.848   9.254 -29.116  0.00  0.00      PROA
ATOM   3767  HB1 GLN P 233     -14.327   8.256 -29.016  0.00  0.00      PROA
ATOM   3768  HB2 GLN P 233     -12.773   9.057 -29.313  0.00  0.00      PROA
ATOM   3769  CG  GLN P 233     -14.386   9.950 -30.441  0.00  0.00      PROA
ATOM   3770  HG1 GLN P 233     -14.320   9.252 -31.303  0.00  0.00      PROA
ATOM   3771  HG2 GLN P 233     -13.712  10.778 -30.750  0.00  0.00      PROA
ATOM   3772  CD  GLN P 233     -15.823  10.486 -30.407  0.00  0.00      PROA
ATOM   3773  OE1 GLN P 233     -16.066  11.691 -30.290  0.00  0.00      PROA
ATOM   3774  NE2 GLN P 233     -16.795   9.577 -30.607  0.00  0.00      PROA
ATOM   3775 HE21 GLN P 233     -17.770   9.784 -30.530  0.00  0.00      PROA
ATOM   3776 HE22 GLN P 233     -16.580   8.614 -30.770  0.00  0.00      PROA
ATOM   3777  C   GLN P 233     -13.021   9.748 -26.854  0.00  0.00      PROA
```

ATOM	3778	O	GLN	P	233	-13.223	9.224	-25.767	0.00	0.00	PROA
ATOM	3779	N	LYS	P	234	-11.799	10.243	-27.209	0.00	0.00	PROA
ATOM	3780	HN	LYS	P	234	-11.733	10.716	-28.084	0.00	0.00	PROA
ATOM	3781	CA	LYS	P	234	-10.630	10.202	-26.405	0.00	0.00	PROA
ATOM	3782	HA	LYS	P	234	-10.527	9.133	-26.291	0.00	0.00	PROA
ATOM	3783	CB	LYS	P	234	-9.323	10.663	-27.153	0.00	0.00	PROA
ATOM	3784	HB1	LYS	P	234	-8.529	10.661	-26.375	0.00	0.00	PROA
ATOM	3785	HB2	LYS	P	234	-9.333	11.720	-27.494	0.00	0.00	PROA
ATOM	3786	CG	LYS	P	234	-8.850	9.705	-28.226	0.00	0.00	PROA
ATOM	3787	HG1	LYS	P	234	-9.653	9.628	-28.990	0.00	0.00	PROA
ATOM	3788	HG2	LYS	P	234	-8.681	8.739	-27.705	0.00	0.00	PROA
ATOM	3789	CD	LYS	P	234	-7.515	10.014	-28.933	0.00	0.00	PROA
ATOM	3790	HD1	LYS	P	234	-6.600	10.088	-28.308	0.00	0.00	PROA
ATOM	3791	HD2	LYS	P	234	-7.596	11.037	-29.359	0.00	0.00	PROA
ATOM	3792	CE	LYS	P	234	-7.314	8.881	-29.984	0.00	0.00	PROA
ATOM	3793	HE1	LYS	P	234	-8.074	8.806	-30.791	0.00	0.00	PROA
ATOM	3794	HE2	LYS	P	234	-7.309	7.924	-29.419	0.00	0.00	PROA
ATOM	3795	NZ	LYS	P	234	-6.048	9.053	-30.706	0.00	0.00	PROA
ATOM	3796	HZ1	LYS	P	234	-6.066	8.451	-31.554	0.00	0.00	PROA
ATOM	3797	HZ2	LYS	P	234	-5.279	8.828	-30.043	0.00	0.00	PROA
ATOM	3798	HZ3	LYS	P	234	-5.915	10.047	-30.981	0.00	0.00	PROA
ATOM	3799	C	LYS	P	234	-10.723	10.836	-24.972	0.00	0.00	PROA
ATOM	3800	O	LYS	P	234	-10.129	10.296	-24.047	0.00	0.00	PROA
ATOM	3801	N	ARG	P	235	-11.437	11.956	-24.897	0.00	0.00	PROA
ATOM	3802	HN	ARG	P	235	-11.724	12.402	-25.741	0.00	0.00	PROA
ATOM	3803	CA	ARG	P	235	-11.730	12.621	-23.660	0.00	0.00	PROA
ATOM	3804	HA	ARG	P	235	-10.780	12.702	-23.152	0.00	0.00	PROA
ATOM	3805	CB	ARG	P	235	-12.189	14.133	-23.818	0.00	0.00	PROA
ATOM	3806	HB1	ARG	P	235	-12.463	14.564	-22.832	0.00	0.00	PROA
ATOM	3807	HB2	ARG	P	235	-13.083	14.407	-24.419	0.00	0.00	PROA
ATOM	3808	CG	ARG	P	235	-11.142	15.072	-24.307	0.00	0.00	PROA
ATOM	3809	HG1	ARG	P	235	-11.054	14.809	-25.383	0.00	0.00	PROA
ATOM	3810	HG2	ARG	P	235	-10.115	15.058	-23.884	0.00	0.00	PROA
ATOM	3811	CD	ARG	P	235	-11.661	16.537	-24.269	0.00	0.00	PROA
ATOM	3812	HD1	ARG	P	235	-12.691	16.706	-24.650	0.00	0.00	PROA
ATOM	3813	HD2	ARG	P	235	-10.957	17.138	-24.883	0.00	0.00	PROA
ATOM	3814	NE	ARG	P	235	-11.698	16.901	-22.843	0.00	0.00	PROA
ATOM	3815	HE	ARG	P	235	-11.434	16.195	-22.187	0.00	0.00	PROA
ATOM	3816	CZ	ARG	P	235	-11.684	18.144	-22.373	0.00	0.00	PROA
ATOM	3817	NH1	ARG	P	235	-11.598	19.253	-23.097	0.00	0.00	PROA
ATOM	3818	HH11	ARG	P	235	-11.792	19.184	-24.075	0.00	0.00	PROA
ATOM	3819	HH12	ARG	P	235	-11.412	20.157	-22.713	0.00	0.00	PROA
ATOM	3820	NH2	ARG	P	235	-11.450	18.274	-21.077	0.00	0.00	PROA
ATOM	3821	HH21	ARG	P	235	-11.717	17.499	-20.504	0.00	0.00	PROA
ATOM	3822	HH22	ARG	P	235	-11.628	19.133	-20.596	0.00	0.00	PROA
ATOM	3823	C	ARG	P	235	-12.717	11.860	-22.785	0.00	0.00	PROA
ATOM	3824	O	ARG	P	235	-12.622	11.935	-21.587	0.00	0.00	PROA
ATOM	3825	N	LYS	P	236	-13.680	11.098	-23.275	0.00	0.00	PROA
ATOM	3826	HN	LYS	P	236	-13.796	11.189	-24.261	0.00	0.00	PROA
ATOM	3827	CA	LYS	P	236	-14.577	10.277	-22.546	0.00	0.00	PROA
ATOM	3828	HA	LYS	P	236	-14.972	10.834	-21.710	0.00	0.00	PROA
ATOM	3829	CB	LYS	P	236	-15.851	9.992	-23.220	0.00	0.00	PROA
ATOM	3830	HB1	LYS	P	236	-15.712	10.062	-24.321	0.00	0.00	PROA
ATOM	3831	HB2	LYS	P	236	-16.531	10.867	-23.144	0.00	0.00	PROA
ATOM	3832	CG	LYS	P	236	-16.614	8.656	-23.173	0.00	0.00	PROA
ATOM	3833	HG1	LYS	P	236	-15.877	7.959	-23.626	0.00	0.00	PROA
ATOM	3834	HG2	LYS	P	236	-17.417	8.811	-23.926	0.00	0.00	PROA
ATOM	3835	CD	LYS	P	236	-17.228	8.274	-21.798	0.00	0.00	PROA
ATOM	3836	HD1	LYS	P	236	-18.112	8.881	-21.508	0.00	0.00	PROA
ATOM	3837	HD2	LYS	P	236	-16.458	8.545	-21.044	0.00	0.00	PROA
ATOM	3838	CE	LYS	P	236	-17.624	6.834	-21.544	0.00	0.00	PROA

ATOM	3839	HE1	LYS	P	236	-18.027	6.714	-20.516	0.00	0.00	PROA
ATOM	3840	HE2	LYS	P	236	-16.738	6.164	-21.559	0.00	0.00	PROA
ATOM	3841	NZ	LYS	P	236	-18.650	6.497	-22.548	0.00	0.00	PROA
ATOM	3842	HZ1	LYS	P	236	-19.165	5.680	-22.161	0.00	0.00	PROA
ATOM	3843	HZ2	LYS	P	236	-18.244	6.289	-23.482	0.00	0.00	PROA
ATOM	3844	HZ3	LYS	P	236	-19.335	7.269	-22.679	0.00	0.00	PROA
ATOM	3845	C	LYS	P	236	-13.855	9.006	-22.187	0.00	0.00	PROA
ATOM	3846	O	LYS	P	236	-14.005	8.546	-21.070	0.00	0.00	PROA
ATOM	3847	N	ALA	P	237	-13.033	8.437	-23.062	0.00	0.00	PROA
ATOM	3848	HN	ALA	P	237	-12.941	8.779	-23.995	0.00	0.00	PROA
ATOM	3849	CA	ALA	P	237	-12.212	7.244	-22.687	0.00	0.00	PROA
ATOM	3850	HA	ALA	P	237	-12.813	6.425	-22.321	0.00	0.00	PROA
ATOM	3851	CB	ALA	P	237	-11.449	6.784	-23.924	0.00	0.00	PROA
ATOM	3852	HB1	ALA	P	237	-12.056	6.570	-24.830	0.00	0.00	PROA
ATOM	3853	HB2	ALA	P	237	-10.693	5.984	-23.773	0.00	0.00	PROA
ATOM	3854	HB3	ALA	P	237	-10.857	7.645	-24.299	0.00	0.00	PROA
ATOM	3855	C	ALA	P	237	-11.239	7.536	-21.510	0.00	0.00	PROA
ATOM	3856	O	ALA	P	237	-11.161	6.740	-20.518	0.00	0.00	PROA
ATOM	3857	N	LEU	P	238	-10.574	8.765	-21.613	0.00	0.00	PROA
ATOM	3858	HN	LEU	P	238	-10.715	9.292	-22.447	0.00	0.00	PROA
ATOM	3859	CA	LEU	P	238	-9.712	9.278	-20.572	0.00	0.00	PROA
ATOM	3860	HA	LEU	P	238	-8.911	8.562	-20.461	0.00	0.00	PROA
ATOM	3861	CB	LEU	P	238	-9.003	10.534	-21.035	0.00	0.00	PROA
ATOM	3862	HB1	LEU	P	238	-9.841	11.125	-21.460	0.00	0.00	PROA
ATOM	3863	HB2	LEU	P	238	-8.402	10.226	-21.917	0.00	0.00	PROA
ATOM	3864	CG	LEU	P	238	-8.098	11.310	-19.993	0.00	0.00	PROA
ATOM	3865	HG	LEU	P	238	-8.618	11.620	-19.062	0.00	0.00	PROA
ATOM	3866	CD1	LEU	P	238	-6.872	10.420	-19.498	0.00	0.00	PROA
ATOM	3867	HD11	LEU	P	238	-6.229	10.033	-20.318	0.00	0.00	PROA
ATOM	3868	HD12	LEU	P	238	-7.211	9.509	-18.960	0.00	0.00	PROA
ATOM	3869	HD13	LEU	P	238	-6.149	11.020	-18.905	0.00	0.00	PROA
ATOM	3870	CD2	LEU	P	238	-7.617	12.568	-20.693	0.00	0.00	PROA
ATOM	3871	HD21	LEU	P	238	-6.940	13.144	-20.027	0.00	0.00	PROA
ATOM	3872	HD22	LEU	P	238	-8.492	13.127	-21.088	0.00	0.00	PROA
ATOM	3873	HD23	LEU	P	238	-6.953	12.320	-21.549	0.00	0.00	PROA
ATOM	3874	C	LEU	P	238	-10.483	9.430	-19.197	0.00	0.00	PROA
ATOM	3875	O	LEU	P	238	-9.979	9.050	-18.137	0.00	0.00	PROA
ATOM	3876	N	LYS	P	239	-11.697	9.994	-19.212	0.00	0.00	PROA
ATOM	3877	HN	LYS	P	239	-12.157	10.337	-20.028	0.00	0.00	PROA
ATOM	3878	CA	LYS	P	239	-12.461	10.000	-17.990	0.00	0.00	PROA
ATOM	3879	HA	LYS	P	239	-11.966	10.644	-17.278	0.00	0.00	PROA
ATOM	3880	CB	LYS	P	239	-13.831	10.685	-18.172	0.00	0.00	PROA
ATOM	3881	HB1	LYS	P	239	-14.443	10.579	-17.250	0.00	0.00	PROA
ATOM	3882	HB2	LYS	P	239	-14.324	10.265	-19.075	0.00	0.00	PROA
ATOM	3883	CG	LYS	P	239	-13.761	12.195	-18.205	0.00	0.00	PROA
ATOM	3884	HG1	LYS	P	239	-13.078	12.441	-19.045	0.00	0.00	PROA
ATOM	3885	HG2	LYS	P	239	-13.474	12.706	-17.261	0.00	0.00	PROA
ATOM	3886	CD	LYS	P	239	-15.106	12.737	-18.567	0.00	0.00	PROA
ATOM	3887	HD1	LYS	P	239	-15.683	12.726	-17.618	0.00	0.00	PROA
ATOM	3888	HD2	LYS	P	239	-15.519	12.084	-19.366	0.00	0.00	PROA
ATOM	3889	CE	LYS	P	239	-15.188	14.140	-19.064	0.00	0.00	PROA
ATOM	3890	HE1	LYS	P	239	-14.882	14.079	-20.130	0.00	0.00	PROA
ATOM	3891	HE2	LYS	P	239	-14.411	14.756	-18.564	0.00	0.00	PROA
ATOM	3892	NZ	LYS	P	239	-16.443	14.936	-18.894	0.00	0.00	PROA
ATOM	3893	HZ1	LYS	P	239	-17.195	14.462	-19.435	0.00	0.00	PROA
ATOM	3894	HZ2	LYS	P	239	-16.550	15.940	-19.143	0.00	0.00	PROA
ATOM	3895	HZ3	LYS	P	239	-16.804	14.840	-17.924	0.00	0.00	PROA
ATOM	3896	C	LYS	P	239	-12.708	8.702	-17.389	0.00	0.00	PROA
ATOM	3897	O	LYS	P	239	-12.428	8.566	-16.212	0.00	0.00	PROA
ATOM	3898	N	THR	P	240	-13.053	7.610	-18.182	0.00	0.00	PROA
ATOM	3899	HN	THR	P	240	-13.191	7.884	-19.130	0.00	0.00	PROA

ATOM	3900	CA	THR	P	240	-13.145	6.213	-17.710	0.00	0.00	PROA
ATOM	3901	HA	THR	P	240	-13.908	6.211	-16.946	0.00	0.00	PROA
ATOM	3902	CB	THR	P	240	-13.691	5.344	-18.818	0.00	0.00	PROA
ATOM	3903	HB	THR	P	240	-13.019	5.266	-19.700	0.00	0.00	PROA
ATOM	3904	OG1	THR	P	240	-15.031	5.709	-19.177	0.00	0.00	PROA
ATOM	3905	HG1	THR	P	240	-15.208	6.609	-18.894	0.00	0.00	PROA
ATOM	3906	CG2	THR	P	240	-13.742	3.889	-18.386	0.00	0.00	PROA
ATOM	3907	HG21	THR	P	240	-12.799	3.433	-18.016	0.00	0.00	PROA
ATOM	3908	HG22	THR	P	240	-14.081	3.200	-19.189	0.00	0.00	PROA
ATOM	3909	HG23	THR	P	240	-14.312	3.743	-17.443	0.00	0.00	PROA
ATOM	3910	C	THR	P	240	-11.805	5.734	-17.198	0.00	0.00	PROA
ATOM	3911	O	THR	P	240	-11.787	5.089	-16.158	0.00	0.00	PROA
ATOM	3912	N	THR	P	241	-10.682	6.013	-17.877	0.00	0.00	PROA
ATOM	3913	HN	THR	P	241	-10.762	6.369	-18.805	0.00	0.00	PROA
ATOM	3914	CA	THR	P	241	-9.332	5.731	-17.359	0.00	0.00	PROA
ATOM	3915	HA	THR	P	241	-9.239	4.677	-17.142	0.00	0.00	PROA
ATOM	3916	CB	THR	P	241	-8.295	6.146	-18.363	0.00	0.00	PROA
ATOM	3917	HB	THR	P	241	-8.512	7.213	-18.583	0.00	0.00	PROA
ATOM	3918	OG1	THR	P	241	-8.359	5.352	-19.570	0.00	0.00	PROA
ATOM	3919	HG1	THR	P	241	-8.349	4.431	-19.298	0.00	0.00	PROA
ATOM	3920	CG2	THR	P	241	-6.845	6.111	-17.850	0.00	0.00	PROA
ATOM	3921	HG21	THR	P	241	-6.475	5.105	-17.558	0.00	0.00	PROA
ATOM	3922	HG22	THR	P	241	-6.704	6.974	-17.164	0.00	0.00	PROA
ATOM	3923	HG23	THR	P	241	-6.183	6.462	-18.671	0.00	0.00	PROA
ATOM	3924	C	THR	P	241	-9.007	6.373	-16.042	0.00	0.00	PROA
ATOM	3925	O	THR	P	241	-8.646	5.660	-15.078	0.00	0.00	PROA
ATOM	3926	N	VAL	P	242	-9.410	7.668	-15.808	0.00	0.00	PROA
ATOM	3927	HN	VAL	P	242	-9.962	8.157	-16.478	0.00	0.00	PROA
ATOM	3928	CA	VAL	P	242	-9.212	8.460	-14.610	0.00	0.00	PROA
ATOM	3929	HA	VAL	P	242	-8.147	8.348	-14.470	0.00	0.00	PROA
ATOM	3930	CB	VAL	P	242	-9.313	9.919	-14.896	0.00	0.00	PROA
ATOM	3931	HB	VAL	P	242	-10.093	10.006	-15.682	0.00	0.00	PROA
ATOM	3932	CG1	VAL	P	242	-9.909	10.662	-13.678	0.00	0.00	PROA
ATOM	3933	HG11	VAL	P	242	-10.939	10.292	-13.487	0.00	0.00	PROA
ATOM	3934	HG12	VAL	P	242	-10.004	11.752	-13.870	0.00	0.00	PROA
ATOM	3935	HG13	VAL	P	242	-9.300	10.588	-12.751	0.00	0.00	PROA
ATOM	3936	CG2	VAL	P	242	-8.065	10.498	-15.425	0.00	0.00	PROA
ATOM	3937	HG21	VAL	P	242	-8.132	11.596	-15.580	0.00	0.00	PROA
ATOM	3938	HG22	VAL	P	242	-7.672	10.061	-16.368	0.00	0.00	PROA
ATOM	3939	HG23	VAL	P	242	-7.277	10.422	-14.646	0.00	0.00	PROA
ATOM	3940	C	VAL	P	242	-9.960	7.934	-13.404	0.00	0.00	PROA
ATOM	3941	O	VAL	P	242	-9.389	7.846	-12.306	0.00	0.00	PROA
ATOM	3942	N	ILE	P	243	-11.168	7.249	-13.618	0.00	0.00	PROA
ATOM	3943	HN	ILE	P	243	-11.521	7.247	-14.551	0.00	0.00	PROA
ATOM	3944	CA	ILE	P	243	-11.935	6.699	-12.571	0.00	0.00	PROA
ATOM	3945	HA	ILE	P	243	-12.054	7.496	-11.851	0.00	0.00	PROA
ATOM	3946	CB	ILE	P	243	-13.329	6.294	-12.982	0.00	0.00	PROA
ATOM	3947	HB	ILE	P	243	-13.118	5.564	-13.793	0.00	0.00	PROA
ATOM	3948	CG2	ILE	P	243	-14.163	5.615	-11.875	0.00	0.00	PROA
ATOM	3949	HG21	ILE	P	243	-13.762	4.629	-11.556	0.00	0.00	PROA
ATOM	3950	HG22	ILE	P	243	-15.196	5.310	-12.147	0.00	0.00	PROA
ATOM	3951	HG23	ILE	P	243	-14.082	6.331	-11.029	0.00	0.00	PROA
ATOM	3952	CG1	ILE	P	243	-14.150	7.507	-13.643	0.00	0.00	PROA
ATOM	3953	HG11	ILE	P	243	-13.451	8.031	-14.330	0.00	0.00	PROA
ATOM	3954	HG12	ILE	P	243	-14.447	8.215	-12.840	0.00	0.00	PROA
ATOM	3955	CD	ILE	P	243	-15.386	7.081	-14.460	0.00	0.00	PROA
ATOM	3956	HD1	ILE	P	243	-16.203	6.885	-13.733	0.00	0.00	PROA
ATOM	3957	HD2	ILE	P	243	-15.147	6.215	-15.113	0.00	0.00	PROA
ATOM	3958	HD3	ILE	P	243	-15.844	7.867	-15.097	0.00	0.00	PROA
ATOM	3959	C	ILE	P	243	-11.212	5.627	-11.760	0.00	0.00	PROA
ATOM	3960	O	ILE	P	243	-11.327	5.586	-10.532	0.00	0.00	PROA

ATOM	3961	N	LEU	P	244	-10.441	4.737	-12.373	0.00	0.00	PROA
ATOM	3962	HN	LEU	P	244	-10.428	4.745	-13.369	0.00	0.00	PROA
ATOM	3963	CA	LEU	P	244	-9.535	3.881	-11.609	0.00	0.00	PROA
ATOM	3964	HA	LEU	P	244	-10.180	3.265	-10.999	0.00	0.00	PROA
ATOM	3965	CB	LEU	P	244	-8.922	2.801	-12.553	0.00	0.00	PROA
ATOM	3966	HB1	LEU	P	244	-8.419	3.240	-13.441	0.00	0.00	PROA
ATOM	3967	HB2	LEU	P	244	-9.814	2.289	-12.972	0.00	0.00	PROA
ATOM	3968	CG	LEU	P	244	-8.051	1.718	-11.899	0.00	0.00	PROA
ATOM	3969	HG	LEU	P	244	-7.131	2.205	-11.513	0.00	0.00	PROA
ATOM	3970	CD1	LEU	P	244	-8.719	0.963	-10.759	0.00	0.00	PROA
ATOM	3971	HD11	LEU	P	244	-8.095	0.179	-10.278	0.00	0.00	PROA
ATOM	3972	HD12	LEU	P	244	-9.678	0.497	-11.071	0.00	0.00	PROA
ATOM	3973	HD13	LEU	P	244	-9.096	1.779	-10.106	0.00	0.00	PROA
ATOM	3974	CD2	LEU	P	244	-7.577	0.759	-13.018	0.00	0.00	PROA
ATOM	3975	HD21	LEU	P	244	-8.474	0.546	-13.638	0.00	0.00	PROA
ATOM	3976	HD22	LEU	P	244	-7.068	-0.160	-12.657	0.00	0.00	PROA
ATOM	3977	HD23	LEU	P	244	-6.943	1.248	-13.788	0.00	0.00	PROA
ATOM	3978	C	LEU	P	244	-8.433	4.565	-10.688	0.00	0.00	PROA
ATOM	3979	O	LEU	P	244	-8.144	4.198	-9.571	0.00	0.00	PROA
ATOM	3980	N	ILE	P	245	-7.828	5.661	-11.181	0.00	0.00	PROA
ATOM	3981	HN	ILE	P	245	-8.103	5.941	-12.097	0.00	0.00	PROA
ATOM	3982	CA	ILE	P	245	-6.822	6.490	-10.513	0.00	0.00	PROA
ATOM	3983	HA	ILE	P	245	-6.076	5.818	-10.116	0.00	0.00	PROA
ATOM	3984	CB	ILE	P	245	-6.076	7.502	-11.418	0.00	0.00	PROA
ATOM	3985	HB	ILE	P	245	-6.743	8.338	-11.719	0.00	0.00	PROA
ATOM	3986	CG2	ILE	P	245	-5.067	8.303	-10.646	0.00	0.00	PROA
ATOM	3987	HG21	ILE	P	245	-5.574	9.016	-9.962	0.00	0.00	PROA
ATOM	3988	HG22	ILE	P	245	-4.525	8.986	-11.334	0.00	0.00	PROA
ATOM	3989	HG23	ILE	P	245	-4.351	7.692	-10.056	0.00	0.00	PROA
ATOM	3990	CG1	ILE	P	245	-5.467	6.767	-12.683	0.00	0.00	PROA
ATOM	3991	HG11	ILE	P	245	-5.186	7.541	-13.429	0.00	0.00	PROA
ATOM	3992	HG12	ILE	P	245	-6.272	6.162	-13.153	0.00	0.00	PROA
ATOM	3993	CD	ILE	P	245	-4.363	5.804	-12.383	0.00	0.00	PROA
ATOM	3994	HD1	ILE	P	245	-4.167	5.081	-13.203	0.00	0.00	PROA
ATOM	3995	HD2	ILE	P	245	-4.659	5.058	-11.614	0.00	0.00	PROA
ATOM	3996	HD3	ILE	P	245	-3.430	6.296	-12.034	0.00	0.00	PROA
ATOM	3997	C	ILE	P	245	-7.350	7.174	-9.256	0.00	0.00	PROA
ATOM	3998	O	ILE	P	245	-6.812	6.980	-8.156	0.00	0.00	PROA
ATOM	3999	N	LEU	P	246	-8.426	7.972	-9.337	0.00	0.00	PROA
ATOM	4000	HN	LEU	P	246	-8.844	8.160	-10.222	0.00	0.00	PROA
ATOM	4001	CA	LEU	P	246	-9.259	8.495	-8.236	0.00	0.00	PROA
ATOM	4002	HA	LEU	P	246	-8.616	9.205	-7.738	0.00	0.00	PROA
ATOM	4003	CB	LEU	P	246	-10.617	9.212	-8.806	0.00	0.00	PROA
ATOM	4004	HB1	LEU	P	246	-11.306	9.496	-7.981	0.00	0.00	PROA
ATOM	4005	HB2	LEU	P	246	-11.034	8.320	-9.321	0.00	0.00	PROA
ATOM	4006	CG	LEU	P	246	-10.338	10.356	-9.740	0.00	0.00	PROA
ATOM	4007	HG	LEU	P	246	-9.711	10.033	-10.599	0.00	0.00	PROA
ATOM	4008	CD1	LEU	P	246	-11.718	10.793	-10.325	0.00	0.00	PROA
ATOM	4009	HD11	LEU	P	246	-12.305	9.933	-10.714	0.00	0.00	PROA
ATOM	4010	HD12	LEU	P	246	-11.528	11.534	-11.131	0.00	0.00	PROA
ATOM	4011	HD13	LEU	P	246	-12.292	11.310	-9.527	0.00	0.00	PROA
ATOM	4012	CD2	LEU	P	246	-9.681	11.510	-9.087	0.00	0.00	PROA
ATOM	4013	HD21	LEU	P	246	-10.206	11.682	-8.123	0.00	0.00	PROA
ATOM	4014	HD22	LEU	P	246	-9.691	12.389	-9.767	0.00	0.00	PROA
ATOM	4015	HD23	LEU	P	246	-8.648	11.250	-8.770	0.00	0.00	PROA
ATOM	4016	C	LEU	P	246	-9.660	7.400	-7.181	0.00	0.00	PROA
ATOM	4017	O	LEU	P	246	-9.591	7.541	-5.981	0.00	0.00	PROA
ATOM	4018	N	ALA	P	247	-10.169	6.274	-7.736	0.00	0.00	PROA
ATOM	4019	HN	ALA	P	247	-10.276	6.103	-8.713	0.00	0.00	PROA
ATOM	4020	CA	ALA	P	247	-10.497	5.144	-6.855	0.00	0.00	PROA
ATOM	4021	HA	ALA	P	247	-11.292	5.425	-6.181	0.00	0.00	PROA

ATOM	4022	CB	ALA	P	247	-11.187	4.018	-7.581	0.00	0.00	PROA
ATOM	4023	HB1	ALA	P	247	-10.569	3.424	-8.288	0.00	0.00	PROA
ATOM	4024	HB2	ALA	P	247	-12.056	4.453	-8.120	0.00	0.00	PROA
ATOM	4025	HB3	ALA	P	247	-11.654	3.319	-6.855	0.00	0.00	PROA
ATOM	4026	C	ALA	P	247	-9.423	4.538	-6.007	0.00	0.00	PROA
ATOM	4027	O	ALA	P	247	-9.617	4.200	-4.871	0.00	0.00	PROA
ATOM	4028	N	PHE	P	248	-8.225	4.280	-6.670	0.00	0.00	PROA
ATOM	4029	HN	PHE	P	248	-8.078	4.453	-7.641	0.00	0.00	PROA
ATOM	4030	CA	PHE	P	248	-7.074	3.677	-6.039	0.00	0.00	PROA
ATOM	4031	HA	PHE	P	248	-7.390	2.881	-5.381	0.00	0.00	PROA
ATOM	4032	CB	PHE	P	248	-6.110	3.169	-7.158	0.00	0.00	PROA
ATOM	4033	HB1	PHE	P	248	-6.186	3.989	-7.904	0.00	0.00	PROA
ATOM	4034	HB2	PHE	P	248	-6.490	2.225	-7.605	0.00	0.00	PROA
ATOM	4035	CG	PHE	P	248	-4.679	3.030	-6.794	0.00	0.00	PROA
ATOM	4036	CD1	PHE	P	248	-3.661	4.044	-6.989	0.00	0.00	PROA
ATOM	4037	HD1	PHE	P	248	-3.835	4.986	-7.487	0.00	0.00	PROA
ATOM	4038	CE1	PHE	P	248	-2.406	3.869	-6.472	0.00	0.00	PROA
ATOM	4039	HE1	PHE	P	248	-1.648	4.636	-6.521	0.00	0.00	PROA
ATOM	4040	CZ	PHE	P	248	-2.020	2.609	-5.931	0.00	0.00	PROA
ATOM	4041	HZ	PHE	P	248	-1.005	2.422	-5.613	0.00	0.00	PROA
ATOM	4042	CD2	PHE	P	248	-4.294	1.856	-6.279	0.00	0.00	PROA
ATOM	4043	HD2	PHE	P	248	-5.038	1.086	-6.140	0.00	0.00	PROA
ATOM	4044	CE2	PHE	P	248	-2.962	1.645	-5.832	0.00	0.00	PROA
ATOM	4045	HE2	PHE	P	248	-2.805	0.660	-5.417	0.00	0.00	PROA
ATOM	4046	C	PHE	P	248	-6.479	4.600	-4.918	0.00	0.00	PROA
ATOM	4047	O	PHE	P	248	-6.116	4.268	-3.810	0.00	0.00	PROA
ATOM	4048	N	PHE	P	249	-6.449	5.921	-5.230	0.00	0.00	PROA
ATOM	4049	HN	PHE	P	249	-6.696	6.246	-6.140	0.00	0.00	PROA
ATOM	4050	CA	PHE	P	249	-5.885	7.011	-4.450	0.00	0.00	PROA
ATOM	4051	HA	PHE	P	249	-4.836	6.913	-4.210	0.00	0.00	PROA
ATOM	4052	CB	PHE	P	249	-5.941	8.237	-5.498	0.00	0.00	PROA
ATOM	4053	HB1	PHE	P	249	-7.009	8.313	-5.794	0.00	0.00	PROA
ATOM	4054	HB2	PHE	P	249	-5.295	8.007	-6.371	0.00	0.00	PROA
ATOM	4055	CG	PHE	P	249	-5.616	9.519	-4.762	0.00	0.00	PROA
ATOM	4056	CD1	PHE	P	249	-6.345	10.704	-5.153	0.00	0.00	PROA
ATOM	4057	HD1	PHE	P	249	-7.037	10.713	-5.982	0.00	0.00	PROA
ATOM	4058	CE1	PHE	P	249	-5.902	11.954	-4.600	0.00	0.00	PROA
ATOM	4059	HE1	PHE	P	249	-6.506	12.806	-4.874	0.00	0.00	PROA
ATOM	4060	CZ	PHE	P	249	-4.823	11.959	-3.744	0.00	0.00	PROA
ATOM	4061	HZ	PHE	P	249	-4.505	12.904	-3.330	0.00	0.00	PROA
ATOM	4062	CD2	PHE	P	249	-4.509	9.620	-3.930	0.00	0.00	PROA
ATOM	4063	HD2	PHE	P	249	-3.917	8.753	-3.676	0.00	0.00	PROA
ATOM	4064	CE2	PHE	P	249	-4.106	10.842	-3.444	0.00	0.00	PROA
ATOM	4065	HE2	PHE	P	249	-3.278	10.767	-2.754	0.00	0.00	PROA
ATOM	4066	C	PHE	P	249	-6.723	7.246	-3.241	0.00	0.00	PROA
ATOM	4067	O	PHE	P	249	-6.296	7.226	-2.115	0.00	0.00	PROA
ATOM	4068	N	ALA	P	250	-8.035	7.220	-3.488	0.00	0.00	PROA
ATOM	4069	HN	ALA	P	250	-8.396	7.058	-4.404	0.00	0.00	PROA
ATOM	4070	CA	ALA	P	250	-9.162	7.287	-2.551	0.00	0.00	PROA
ATOM	4071	HA	ALA	P	250	-8.957	8.099	-1.869	0.00	0.00	PROA
ATOM	4072	CB	ALA	P	250	-10.538	7.624	-3.150	0.00	0.00	PROA
ATOM	4073	HB1	ALA	P	250	-10.848	6.828	-3.861	0.00	0.00	PROA
ATOM	4074	HB2	ALA	P	250	-10.524	8.603	-3.675	0.00	0.00	PROA
ATOM	4075	HB3	ALA	P	250	-11.286	7.907	-2.379	0.00	0.00	PROA
ATOM	4076	C	ALA	P	250	-9.205	6.113	-1.618	0.00	0.00	PROA
ATOM	4077	O	ALA	P	250	-9.503	6.323	-0.406	0.00	0.00	PROA
ATOM	4078	N	CYS	P	251	-8.908	4.874	-1.971	0.00	0.00	PROA
ATOM	4079	HN	CYS	P	251	-8.847	4.649	-2.941	0.00	0.00	PROA
ATOM	4080	CA	CYS	P	251	-8.717	3.755	-0.986	0.00	0.00	PROA
ATOM	4081	HA	CYS	P	251	-9.634	3.762	-0.415	0.00	0.00	PROA
ATOM	4082	CB	CYS	P	251	-8.520	2.343	-1.594	0.00	0.00	PROA

ATOM	4083	HB1	CYS P	251	-8.363	1.569	-0.812	0.00	0.00	PROA
ATOM	4084	HB2	CYS P	251	-7.643	2.303	-2.275	0.00	0.00	PROA
ATOM	4085	SG	CYS P	251	-9.842	1.713	-2.602	0.00	0.00	PROA
ATOM	4086	HG1	CYS P	251	-9.753	2.504	-3.662	0.00	0.00	PROA
ATOM	4087	C	CYS P	251	-7.577	3.910	0.103	0.00	0.00	PROA
ATOM	4088	O	CYS P	251	-7.802	3.515	1.237	0.00	0.00	PROA
ATOM	4089	N	TRP P	252	-6.377	4.356	-0.308	0.00	0.00	PROA
ATOM	4090	HN	TRP P	252	-6.254	4.426	-1.295	0.00	0.00	PROA
ATOM	4091	CA	TRP P	252	-5.275	4.723	0.611	0.00	0.00	PROA
ATOM	4092	HA	TRP P	252	-5.276	3.994	1.408	0.00	0.00	PROA
ATOM	4093	CB	TRP P	252	-3.899	4.731	-0.116	0.00	0.00	PROA
ATOM	4094	HB1	TRP P	252	-3.059	5.068	0.529	0.00	0.00	PROA
ATOM	4095	HB2	TRP P	252	-4.055	5.332	-1.037	0.00	0.00	PROA
ATOM	4096	CG	TRP P	252	-3.641	3.318	-0.594	0.00	0.00	PROA
ATOM	4097	CD1	TRP P	252	-3.582	2.886	-1.907	0.00	0.00	PROA
ATOM	4098	HD1	TRP P	252	-3.786	3.414	-2.827	0.00	0.00	PROA
ATOM	4099	NE1	TRP P	252	-3.351	1.548	-1.850	0.00	0.00	PROA
ATOM	4100	HE1	TRP P	252	-3.482	0.942	-2.604	0.00	0.00	PROA
ATOM	4101	CE2	TRP P	252	-3.272	1.090	-0.555	0.00	0.00	PROA
ATOM	4102	CD2	TRP P	252	-3.480	2.173	0.300	0.00	0.00	PROA
ATOM	4103	CE3	TRP P	252	-3.392	2.030	1.695	0.00	0.00	PROA
ATOM	4104	HE3	TRP P	252	-3.646	2.841	2.362	0.00	0.00	PROA
ATOM	4105	CZ3	TRP P	252	-3.284	0.716	2.214	0.00	0.00	PROA
ATOM	4106	HZ3	TRP P	252	-3.537	0.555	3.251	0.00	0.00	PROA
ATOM	4107	CZ2	TRP P	252	-3.007	-0.201	-0.046	0.00	0.00	PROA
ATOM	4108	HZ2	TRP P	252	-2.895	-1.035	-0.723	0.00	0.00	PROA
ATOM	4109	CH2	TRP P	252	-3.093	-0.346	1.363	0.00	0.00	PROA
ATOM	4110	HH2	TRP P	252	-2.904	-1.275	1.881	0.00	0.00	PROA
ATOM	4111	C	TRP P	252	-5.318	6.141	1.243	0.00	0.00	PROA
ATOM	4112	O	TRP P	252	-4.636	6.423	2.254	0.00	0.00	PROA
ATOM	4113	N	LEU P	253	-6.147	7.066	0.659	0.00	0.00	PROA
ATOM	4114	HN	LEU P	253	-6.540	6.891	-0.240	0.00	0.00	PROA
ATOM	4115	CA	LEU P	253	-6.322	8.434	1.217	0.00	0.00	PROA
ATOM	4116	HA	LEU P	253	-5.314	8.822	1.185	0.00	0.00	PROA
ATOM	4117	CB	LEU P	253	-7.145	9.268	0.093	0.00	0.00	PROA
ATOM	4118	HB1	LEU P	253	-8.190	8.900	0.009	0.00	0.00	PROA
ATOM	4119	HB2	LEU P	253	-6.658	9.149	-0.898	0.00	0.00	PROA
ATOM	4120	CG	LEU P	253	-7.250	10.792	0.402	0.00	0.00	PROA
ATOM	4121	HG	LEU P	253	-7.954	10.944	1.248	0.00	0.00	PROA
ATOM	4122	CD1	LEU P	253	-5.857	11.428	0.676	0.00	0.00	PROA
ATOM	4123	HD11	LEU P	253	-5.526	11.121	1.692	0.00	0.00	PROA
ATOM	4124	HD12	LEU P	253	-6.045	12.516	0.794	0.00	0.00	PROA
ATOM	4125	HD13	LEU P	253	-5.015	11.208	-0.015	0.00	0.00	PROA
ATOM	4126	CD2	LEU P	253	-7.985	11.451	-0.773	0.00	0.00	PROA
ATOM	4127	HD21	LEU P	253	-8.323	12.430	-0.371	0.00	0.00	PROA
ATOM	4128	HD22	LEU P	253	-8.911	10.891	-1.026	0.00	0.00	PROA
ATOM	4129	HD23	LEU P	253	-7.353	11.432	-1.687	0.00	0.00	PROA
ATOM	4130	C	LEU P	253	-6.843	8.525	2.615	0.00	0.00	PROA
ATOM	4131	O	LEU P	253	-6.277	9.311	3.379	0.00	0.00	PROA
ATOM	4132	N	PRO P	254	-7.855	7.729	3.110	0.00	0.00	PROA
ATOM	4133	CD	PRO P	254	-8.964	7.190	2.361	0.00	0.00	PROA
ATOM	4134	HD1	PRO P	254	-8.685	6.301	1.756	0.00	0.00	PROA
ATOM	4135	HD2	PRO P	254	-9.350	7.971	1.671	0.00	0.00	PROA
ATOM	4136	CA	PRO P	254	-8.116	7.670	4.541	0.00	0.00	PROA
ATOM	4137	HA	PRO P	254	-8.395	8.640	4.925	0.00	0.00	PROA
ATOM	4138	CB	PRO P	254	-9.476	6.879	4.745	0.00	0.00	PROA
ATOM	4139	HB1	PRO P	254	-10.034	7.489	5.487	0.00	0.00	PROA
ATOM	4140	HB2	PRO P	254	-9.184	5.879	5.132	0.00	0.00	PROA
ATOM	4141	CG	PRO P	254	-10.082	6.836	3.328	0.00	0.00	PROA
ATOM	4142	HG1	PRO P	254	-10.366	5.766	3.231	0.00	0.00	PROA
ATOM	4143	HG2	PRO P	254	-10.889	7.598	3.376	0.00	0.00	PROA

ATOM	4144	C	PRO	P	254	-7.086	7.076	5.461	0.00	0.00	PROA
ATOM	4145	O	PRO	P	254	-6.812	7.547	6.565	0.00	0.00	PROA
ATOM	4146	N	TYR	P	255	-6.385	6.056	4.988	0.00	0.00	PROA
ATOM	4147	HN	TYR	P	255	-6.803	5.537	4.246	0.00	0.00	PROA
ATOM	4148	CA	TYR	P	255	-5.232	5.504	5.681	0.00	0.00	PROA
ATOM	4149	HA	TYR	P	255	-5.487	5.214	6.690	0.00	0.00	PROA
ATOM	4150	CB	TYR	P	255	-4.743	4.161	5.010	0.00	0.00	PROA
ATOM	4151	HB1	TYR	P	255	-4.823	4.258	3.906	0.00	0.00	PROA
ATOM	4152	HB2	TYR	P	255	-5.439	3.308	5.161	0.00	0.00	PROA
ATOM	4153	CG	TYR	P	255	-3.324	3.673	5.280	0.00	0.00	PROA
ATOM	4154	CD1	TYR	P	255	-2.970	2.908	6.477	0.00	0.00	PROA
ATOM	4155	HD1	TYR	P	255	-3.583	2.657	7.330	0.00	0.00	PROA
ATOM	4156	CE1	TYR	P	255	-1.615	2.467	6.671	0.00	0.00	PROA
ATOM	4157	HE1	TYR	P	255	-1.328	1.827	7.491	0.00	0.00	PROA
ATOM	4158	CZ	TYR	P	255	-0.593	2.693	5.722	0.00	0.00	PROA
ATOM	4159	OH	TYR	P	255	0.671	2.328	6.086	0.00	0.00	PROA
ATOM	4160	HH	TYR	P	255	1.358	2.843	5.656	0.00	0.00	PROA
ATOM	4161	CD2	TYR	P	255	-2.267	3.957	4.427	0.00	0.00	PROA
ATOM	4162	HD2	TYR	P	255	-2.661	4.477	3.566	0.00	0.00	PROA
ATOM	4163	CE2	TYR	P	255	-0.933	3.536	4.629	0.00	0.00	PROA
ATOM	4164	HE2	TYR	P	255	-0.201	3.876	3.912	0.00	0.00	PROA
ATOM	4165	C	TYR	P	255	-4.082	6.490	5.862	0.00	0.00	PROA
ATOM	4166	O	TYR	P	255	-3.419	6.603	6.924	0.00	0.00	PROA
ATOM	4167	N	TYR	P	256	-3.792	7.333	4.827	0.00	0.00	PROA
ATOM	4168	HN	TYR	P	256	-4.244	7.135	3.960	0.00	0.00	PROA
ATOM	4169	CA	TYR	P	256	-2.855	8.342	4.888	0.00	0.00	PROA
ATOM	4170	HA	TYR	P	256	-1.979	8.101	5.471	0.00	0.00	PROA
ATOM	4171	CB	TYR	P	256	-2.344	8.729	3.466	0.00	0.00	PROA
ATOM	4172	HB1	TYR	P	256	-1.906	9.748	3.398	0.00	0.00	PROA
ATOM	4173	HB2	TYR	P	256	-3.265	8.770	2.845	0.00	0.00	PROA
ATOM	4174	CG	TYR	P	256	-1.316	7.721	3.009	0.00	0.00	PROA
ATOM	4175	CD1	TYR	P	256	-1.439	6.887	1.894	0.00	0.00	PROA
ATOM	4176	HD1	TYR	P	256	-2.309	7.064	1.279	0.00	0.00	PROA
ATOM	4177	CE1	TYR	P	256	-0.436	5.991	1.460	0.00	0.00	PROA
ATOM	4178	HE1	TYR	P	256	-0.548	5.293	0.644	0.00	0.00	PROA
ATOM	4179	CZ	TYR	P	256	0.781	6.103	2.175	0.00	0.00	PROA
ATOM	4180	OH	TYR	P	256	1.912	5.258	1.808	0.00	0.00	PROA
ATOM	4181	HH	TYR	P	256	1.690	4.894	0.948	0.00	0.00	PROA
ATOM	4182	CD2	TYR	P	256	-0.124	7.655	3.747	0.00	0.00	PROA
ATOM	4183	HD2	TYR	P	256	-0.019	8.256	4.638	0.00	0.00	PROA
ATOM	4184	CE2	TYR	P	256	0.978	6.842	3.308	0.00	0.00	PROA
ATOM	4185	HE2	TYR	P	256	1.852	6.764	3.938	0.00	0.00	PROA
ATOM	4186	C	TYR	P	256	-3.303	9.583	5.695	0.00	0.00	PROA
ATOM	4187	O	TYR	P	256	-2.426	10.253	6.183	0.00	0.00	PROA
ATOM	4188	N	ILE	P	257	-4.595	9.839	5.851	0.00	0.00	PROA
ATOM	4189	HN	ILE	P	257	-5.328	9.362	5.372	0.00	0.00	PROA
ATOM	4190	CA	ILE	P	257	-5.023	10.982	6.649	0.00	0.00	PROA
ATOM	4191	HA	ILE	P	257	-4.356	11.831	6.641	0.00	0.00	PROA
ATOM	4192	CB	ILE	P	257	-6.293	11.471	6.059	0.00	0.00	PROA
ATOM	4193	HB	ILE	P	257	-6.950	10.613	5.804	0.00	0.00	PROA
ATOM	4194	CG2	ILE	P	257	-6.984	12.351	7.101	0.00	0.00	PROA
ATOM	4195	HG21	ILE	P	257	-7.205	11.803	8.042	0.00	0.00	PROA
ATOM	4196	HG22	ILE	P	257	-7.947	12.765	6.733	0.00	0.00	PROA
ATOM	4197	HG23	ILE	P	257	-6.360	13.252	7.282	0.00	0.00	PROA
ATOM	4198	CG1	ILE	P	257	-6.103	12.184	4.673	0.00	0.00	PROA
ATOM	4199	HG11	ILE	P	257	-5.502	11.584	3.957	0.00	0.00	PROA
ATOM	4200	HG12	ILE	P	257	-5.526	13.123	4.813	0.00	0.00	PROA
ATOM	4201	CD	ILE	P	257	-7.384	12.479	3.920	0.00	0.00	PROA
ATOM	4202	HD1	ILE	P	257	-7.913	13.221	4.556	0.00	0.00	PROA
ATOM	4203	HD2	ILE	P	257	-7.998	11.558	3.819	0.00	0.00	PROA
ATOM	4204	HD3	ILE	P	257	-7.108	12.913	2.935	0.00	0.00	PROA

ATOM	4205	C	ILE	P	257	-5.104	10.451	8.113	0.00	0.00	PROA
ATOM	4206	O	ILE	P	257	-4.883	11.245	9.035	0.00	0.00	PROA
ATOM	4207	N	GLY	P	258	-5.227	9.113	8.399	0.00	0.00	PROA
ATOM	4208	HN	GLY	P	258	-5.266	8.492	7.621	0.00	0.00	PROA
ATOM	4209	CA	GLY	P	258	-5.241	8.467	9.691	0.00	0.00	PROA
ATOM	4210	HA1	GLY	P	258	-5.415	7.415	9.518	0.00	0.00	PROA
ATOM	4211	HA2	GLY	P	258	-5.994	8.839	10.371	0.00	0.00	PROA
ATOM	4212	C	GLY	P	258	-3.884	8.541	10.320	0.00	0.00	PROA
ATOM	4213	O	GLY	P	258	-3.715	9.033	11.489	0.00	0.00	PROA
ATOM	4214	N	ILE	P	259	-2.821	8.064	9.572	0.00	0.00	PROA
ATOM	4215	HN	ILE	P	259	-3.025	7.657	8.685	0.00	0.00	PROA
ATOM	4216	CA	ILE	P	259	-1.393	8.350	9.868	0.00	0.00	PROA
ATOM	4217	HA	ILE	P	259	-1.200	7.870	10.815	0.00	0.00	PROA
ATOM	4218	CB	ILE	P	259	-0.504	7.834	8.772	0.00	0.00	PROA
ATOM	4219	HB	ILE	P	259	-0.865	8.323	7.842	0.00	0.00	PROA
ATOM	4220	CG2	ILE	P	259	0.957	8.248	9.020	0.00	0.00	PROA
ATOM	4221	HG21	ILE	P	259	1.163	9.339	9.056	0.00	0.00	PROA
ATOM	4222	HG22	ILE	P	259	1.655	7.827	8.265	0.00	0.00	PROA
ATOM	4223	HG23	ILE	P	259	1.355	7.822	9.966	0.00	0.00	PROA
ATOM	4224	CG1	ILE	P	259	-0.611	6.234	8.582	0.00	0.00	PROA
ATOM	4225	HG11	ILE	P	259	-1.695	5.992	8.558	0.00	0.00	PROA
ATOM	4226	HG12	ILE	P	259	-0.381	5.758	9.559	0.00	0.00	PROA
ATOM	4227	CD	ILE	P	259	0.209	5.581	7.477	0.00	0.00	PROA
ATOM	4228	HD1	ILE	P	259	0.252	4.472	7.503	0.00	0.00	PROA
ATOM	4229	HD2	ILE	P	259	1.286	5.853	7.494	0.00	0.00	PROA
ATOM	4230	HD3	ILE	P	259	-0.145	5.807	6.448	0.00	0.00	PROA
ATOM	4231	C	ILE	P	259	-1.162	9.836	10.209	0.00	0.00	PROA
ATOM	4232	O	ILE	P	259	-0.534	10.134	11.164	0.00	0.00	PROA
ATOM	4233	N	SER	P	260	-1.629	10.799	9.391	0.00	0.00	PROA
ATOM	4234	HN	SER	P	260	-1.953	10.557	8.479	0.00	0.00	PROA
ATOM	4235	CA	SER	P	260	-1.527	12.190	9.728	0.00	0.00	PROA
ATOM	4236	HA	SER	P	260	-0.480	12.433	9.833	0.00	0.00	PROA
ATOM	4237	CB	SER	P	260	-2.145	13.208	8.710	0.00	0.00	PROA
ATOM	4238	HB1	SER	P	260	-1.933	14.276	8.930	0.00	0.00	PROA
ATOM	4239	HB2	SER	P	260	-3.179	13.099	8.318	0.00	0.00	PROA
ATOM	4240	OG	SER	P	260	-1.316	13.108	7.580	0.00	0.00	PROA
ATOM	4241	HG1	SER	P	260	-1.635	12.427	6.983	0.00	0.00	PROA
ATOM	4242	C	SER	P	260	-2.218	12.660	11.016	0.00	0.00	PROA
ATOM	4243	O	SER	P	260	-1.543	13.370	11.774	0.00	0.00	PROA
ATOM	4244	N	ILE	P	261	-3.384	12.278	11.404	0.00	0.00	PROA
ATOM	4245	HN	ILE	P	261	-3.895	11.793	10.698	0.00	0.00	PROA
ATOM	4246	CA	ILE	P	261	-4.126	12.609	12.676	0.00	0.00	PROA
ATOM	4247	HA	ILE	P	261	-3.976	13.661	12.866	0.00	0.00	PROA
ATOM	4248	CB	ILE	P	261	-5.577	12.185	12.828	0.00	0.00	PROA
ATOM	4249	HB	ILE	P	261	-5.690	11.109	12.573	0.00	0.00	PROA
ATOM	4250	CG2	ILE	P	261	-6.116	12.338	14.293	0.00	0.00	PROA
ATOM	4251	HG21	ILE	P	261	-5.620	11.713	15.067	0.00	0.00	PROA
ATOM	4252	HG22	ILE	P	261	-7.175	12.006	14.343	0.00	0.00	PROA
ATOM	4253	HG23	ILE	P	261	-6.096	13.428	14.507	0.00	0.00	PROA
ATOM	4254	CG1	ILE	P	261	-6.393	12.975	11.732	0.00	0.00	PROA
ATOM	4255	HG11	ILE	P	261	-7.426	12.599	11.889	0.00	0.00	PROA
ATOM	4256	HG12	ILE	P	261	-6.058	12.675	10.716	0.00	0.00	PROA
ATOM	4257	CD	ILE	P	261	-6.519	14.514	11.840	0.00	0.00	PROA
ATOM	4258	HD1	ILE	P	261	-5.529	14.910	11.527	0.00	0.00	PROA
ATOM	4259	HD2	ILE	P	261	-6.823	14.876	12.846	0.00	0.00	PROA
ATOM	4260	HD3	ILE	P	261	-7.177	14.853	11.012	0.00	0.00	PROA
ATOM	4261	C	ILE	P	261	-3.301	12.036	13.832	0.00	0.00	PROA
ATOM	4262	O	ILE	P	261	-3.109	12.666	14.839	0.00	0.00	PROA
ATOM	4263	N	ASP	P	262	-2.827	10.799	13.639	0.00	0.00	PROA
ATOM	4264	HN	ASP	P	262	-3.024	10.278	12.812	0.00	0.00	PROA
ATOM	4265	CA	ASP	P	262	-2.070	10.077	14.588	0.00	0.00	PROA

ATOM	4266	HA	ASP	P	262	-2.570	10.137	15.544	0.00	0.00	PROA
ATOM	4267	CB	ASP	P	262	-1.922	8.589	14.058	0.00	0.00	PROA
ATOM	4268	HB1	ASP	P	262	-1.147	8.465	13.271	0.00	0.00	PROA
ATOM	4269	HB2	ASP	P	262	-2.846	8.236	13.551	0.00	0.00	PROA
ATOM	4270	CG	ASP	P	262	-1.744	7.694	15.250	0.00	0.00	PROA
ATOM	4271	OD1	ASP	P	262	-1.251	8.197	16.278	0.00	0.00	PROA
ATOM	4272	OD2	ASP	P	262	-2.103	6.469	15.179	0.00	0.00	PROA
ATOM	4273	C	ASP	P	262	-0.708	10.743	14.839	0.00	0.00	PROA
ATOM	4274	O	ASP	P	262	-0.324	11.128	15.964	0.00	0.00	PROA
ATOM	4275	N	SER	P	263	-0.072	11.135	13.799	0.00	0.00	PROA
ATOM	4276	HN	SER	P	263	-0.509	10.817	12.961	0.00	0.00	PROA
ATOM	4277	CA	SER	P	263	1.149	11.982	13.771	0.00	0.00	PROA
ATOM	4278	HA	SER	P	263	1.929	11.341	14.154	0.00	0.00	PROA
ATOM	4279	CB	SER	P	263	1.734	12.261	12.401	0.00	0.00	PROA
ATOM	4280	HB1	SER	P	263	1.033	12.763	11.700	0.00	0.00	PROA
ATOM	4281	HB2	SER	P	263	1.791	11.342	11.778	0.00	0.00	PROA
ATOM	4282	OG	SER	P	263	3.007	12.809	12.458	0.00	0.00	PROA
ATOM	4283	HG1	SER	P	263	3.527	12.129	12.892	0.00	0.00	PROA
ATOM	4284	C	SER	P	263	0.984	13.347	14.464	0.00	0.00	PROA
ATOM	4285	O	SER	P	263	1.926	13.907	15.072	0.00	0.00	PROA
ATOM	4286	N	PHE	P	264	-0.167	14.070	14.178	0.00	0.00	PROA
ATOM	4287	HN	PHE	P	264	-0.890	13.778	13.556	0.00	0.00	PROA
ATOM	4288	CA	PHE	P	264	-0.486	15.301	14.732	0.00	0.00	PROA
ATOM	4289	HA	PHE	P	264	0.442	15.852	14.697	0.00	0.00	PROA
ATOM	4290	CB	PHE	P	264	-1.800	15.869	14.146	0.00	0.00	PROA
ATOM	4291	HB1	PHE	P	264	-2.321	16.570	14.832	0.00	0.00	PROA
ATOM	4292	HB2	PHE	P	264	-2.558	15.067	14.014	0.00	0.00	PROA
ATOM	4293	CG	PHE	P	264	-1.536	16.396	12.703	0.00	0.00	PROA
ATOM	4294	CD1	PHE	P	264	-0.288	16.378	12.012	0.00	0.00	PROA
ATOM	4295	HD1	PHE	P	264	0.620	15.987	12.448	0.00	0.00	PROA
ATOM	4296	CE1	PHE	P	264	-0.131	16.812	10.641	0.00	0.00	PROA
ATOM	4297	HE1	PHE	P	264	0.834	16.666	10.178	0.00	0.00	PROA
ATOM	4298	CZ	PHE	P	264	-1.263	17.102	9.934	0.00	0.00	PROA
ATOM	4299	HZ	PHE	P	264	-1.378	17.257	8.872	0.00	0.00	PROA
ATOM	4300	CD2	PHE	P	264	-2.634	16.865	12.011	0.00	0.00	PROA
ATOM	4301	HD2	PHE	P	264	-3.517	17.049	12.606	0.00	0.00	PROA
ATOM	4302	CE2	PHE	P	264	-2.480	17.255	10.641	0.00	0.00	PROA
ATOM	4303	HE2	PHE	P	264	-3.254	17.659	10.005	0.00	0.00	PROA
ATOM	4304	C	PHE	P	264	-0.660	15.190	16.183	0.00	0.00	PROA
ATOM	4305	O	PHE	P	264	-0.173	16.041	16.890	0.00	0.00	PROA
ATOM	4306	N	ILE	P	265	-1.301	14.147	16.738	0.00	0.00	PROA
ATOM	4307	HN	ILE	P	265	-1.801	13.540	16.125	0.00	0.00	PROA
ATOM	4308	CA	ILE	P	265	-1.263	13.814	18.171	0.00	0.00	PROA
ATOM	4309	HA	ILE	P	265	-1.512	14.728	18.689	0.00	0.00	PROA
ATOM	4310	CB	ILE	P	265	-2.254	12.552	18.293	0.00	0.00	PROA
ATOM	4311	HB	ILE	P	265	-1.850	11.915	17.478	0.00	0.00	PROA
ATOM	4312	CG2	ILE	P	265	-2.065	11.993	19.690	0.00	0.00	PROA
ATOM	4313	HG21	ILE	P	265	-1.006	11.712	19.874	0.00	0.00	PROA
ATOM	4314	HG22	ILE	P	265	-2.732	11.121	19.863	0.00	0.00	PROA
ATOM	4315	HG23	ILE	P	265	-2.266	12.784	20.444	0.00	0.00	PROA
ATOM	4316	CG1	ILE	P	265	-3.739	12.966	18.075	0.00	0.00	PROA
ATOM	4317	HG11	ILE	P	265	-3.751	13.641	17.193	0.00	0.00	PROA
ATOM	4318	HG12	ILE	P	265	-4.133	13.668	18.840	0.00	0.00	PROA
ATOM	4319	CD	ILE	P	265	-4.669	11.836	17.730	0.00	0.00	PROA
ATOM	4320	HD1	ILE	P	265	-4.791	11.145	18.591	0.00	0.00	PROA
ATOM	4321	HD2	ILE	P	265	-4.253	11.136	16.974	0.00	0.00	PROA
ATOM	4322	HD3	ILE	P	265	-5.649	12.242	17.397	0.00	0.00	PROA
ATOM	4323	C	ILE	P	265	0.127	13.519	18.602	0.00	0.00	PROA
ATOM	4324	O	ILE	P	265	0.630	14.058	19.612	0.00	0.00	PROA
ATOM	4325	N	LEU	P	266	0.894	12.776	17.879	0.00	0.00	PROA
ATOM	4326	HN	LEU	P	266	0.537	12.431	17.015	0.00	0.00	PROA

ATOM	4327	CA	LEU	P	266	2.283	12.396	18.293	0.00	0.00	PROA
ATOM	4328	HA	LEU	P	266	2.287	12.064	19.320	0.00	0.00	PROA
ATOM	4329	CB	LEU	P	266	2.835	11.373	17.338	0.00	0.00	PROA
ATOM	4330	HB1	LEU	P	266	2.838	11.735	16.288	0.00	0.00	PROA
ATOM	4331	HB2	LEU	P	266	2.099	10.540	17.364	0.00	0.00	PROA
ATOM	4332	CG	LEU	P	266	4.175	10.724	17.727	0.00	0.00	PROA
ATOM	4333	HG	LEU	P	266	5.050	11.399	17.605	0.00	0.00	PROA
ATOM	4334	CD1	LEU	P	266	4.274	10.155	19.147	0.00	0.00	PROA
ATOM	4335	HD11	LEU	P	266	4.168	10.982	19.882	0.00	0.00	PROA
ATOM	4336	HD12	LEU	P	266	5.202	9.625	19.453	0.00	0.00	PROA
ATOM	4337	HD13	LEU	P	266	3.362	9.586	19.428	0.00	0.00	PROA
ATOM	4338	CD2	LEU	P	266	4.496	9.607	16.678	0.00	0.00	PROA
ATOM	4339	HD21	LEU	P	266	4.151	9.878	15.657	0.00	0.00	PROA
ATOM	4340	HD22	LEU	P	266	3.962	8.652	16.870	0.00	0.00	PROA
ATOM	4341	HD23	LEU	P	266	5.590	9.414	16.655	0.00	0.00	PROA
ATOM	4342	C	LEU	P	266	3.215	13.632	18.352	0.00	0.00	PROA
ATOM	4343	O	LEU	P	266	4.072	13.696	19.196	0.00	0.00	PROA
ATOM	4344	N	LEU	P	267	3.226	14.603	17.434	0.00	0.00	PROA
ATOM	4345	HN	LEU	P	267	2.744	14.449	16.576	0.00	0.00	PROA
ATOM	4346	CA	LEU	P	267	3.987	15.907	17.516	0.00	0.00	PROA
ATOM	4347	HA	LEU	P	267	4.965	15.587	17.844	0.00	0.00	PROA
ATOM	4348	CB	LEU	P	267	4.136	16.574	16.115	0.00	0.00	PROA
ATOM	4349	HB1	LEU	P	267	4.706	17.525	16.047	0.00	0.00	PROA
ATOM	4350	HB2	LEU	P	267	3.119	16.742	15.701	0.00	0.00	PROA
ATOM	4351	CG	LEU	P	267	5.002	15.768	15.074	0.00	0.00	PROA
ATOM	4352	HG	LEU	P	267	4.826	14.713	15.373	0.00	0.00	PROA
ATOM	4353	CD1	LEU	P	267	4.594	15.863	13.639	0.00	0.00	PROA
ATOM	4354	HD11	LEU	P	267	5.149	15.138	13.008	0.00	0.00	PROA
ATOM	4355	HD12	LEU	P	267	4.792	16.872	13.219	0.00	0.00	PROA
ATOM	4356	HD13	LEU	P	267	3.503	15.714	13.487	0.00	0.00	PROA
ATOM	4357	CD2	LEU	P	267	6.535	16.187	15.283	0.00	0.00	PROA
ATOM	4358	HD21	LEU	P	267	6.768	15.872	16.323	0.00	0.00	PROA
ATOM	4359	HD22	LEU	P	267	6.752	17.261	15.099	0.00	0.00	PROA
ATOM	4360	HD23	LEU	P	267	7.186	15.617	14.586	0.00	0.00	PROA
ATOM	4361	C	LEU	P	267	3.488	17.011	18.546	0.00	0.00	PROA
ATOM	4362	O	LEU	P	267	4.081	18.035	18.731	0.00	0.00	PROA
ATOM	4363	N	GLU	P	268	2.378	16.716	19.207	0.00	0.00	PROA
ATOM	4364	HN	GLU	P	268	1.895	15.891	18.927	0.00	0.00	PROA
ATOM	4365	CA	GLU	P	268	1.725	17.598	20.172	0.00	0.00	PROA
ATOM	4366	HA	GLU	P	268	0.983	17.028	20.711	0.00	0.00	PROA
ATOM	4367	CB	GLU	P	268	2.535	17.916	21.464	0.00	0.00	PROA
ATOM	4368	HB1	GLU	P	268	2.030	18.827	21.850	0.00	0.00	PROA
ATOM	4369	HB2	GLU	P	268	3.594	18.125	21.199	0.00	0.00	PROA
ATOM	4370	CG	GLU	P	268	2.518	16.872	22.597	0.00	0.00	PROA
ATOM	4371	HG1	GLU	P	268	3.135	15.999	22.295	0.00	0.00	PROA
ATOM	4372	HG2	GLU	P	268	1.502	16.657	22.991	0.00	0.00	PROA
ATOM	4373	CD	GLU	P	268	3.310	17.413	23.788	0.00	0.00	PROA
ATOM	4374	OE1	GLU	P	268	4.422	16.845	23.957	0.00	0.00	PROA
ATOM	4375	OE2	GLU	P	268	2.876	18.345	24.532	0.00	0.00	PROA
ATOM	4376	C	GLU	P	268	1.075	18.836	19.548	0.00	0.00	PROA
ATOM	4377	O	GLU	P	268	0.862	19.832	20.197	0.00	0.00	PROA
ATOM	4378	N	ILE	P	269	0.614	18.772	18.239	0.00	0.00	PROA
ATOM	4379	HN	ILE	P	269	0.987	18.082	17.624	0.00	0.00	PROA
ATOM	4380	CA	ILE	P	269	-0.418	19.610	17.606	0.00	0.00	PROA
ATOM	4381	HA	ILE	P	269	-0.184	20.652	17.764	0.00	0.00	PROA
ATOM	4382	CB	ILE	P	269	-0.335	19.382	16.095	0.00	0.00	PROA
ATOM	4383	HB	ILE	P	269	-0.560	18.300	15.973	0.00	0.00	PROA
ATOM	4384	CG2	ILE	P	269	-1.507	20.118	15.313	0.00	0.00	PROA
ATOM	4385	HG21	ILE	P	269	-1.220	21.191	15.361	0.00	0.00	PROA
ATOM	4386	HG22	ILE	P	269	-2.496	20.077	15.817	0.00	0.00	PROA
ATOM	4387	HG23	ILE	P	269	-1.671	19.757	14.276	0.00	0.00	PROA

```
ATOM   4388  CG1 ILE P 269       1.051  19.687  15.442  0.00  0.00      PROA
ATOM   4389 HG11 ILE P 269       1.913  19.624  16.141  0.00  0.00      PROA
ATOM   4390 HG12 ILE P 269       1.265  20.723  15.103  0.00  0.00      PROA
ATOM   4391  CD  ILE P 269       1.424  18.731  14.327  0.00  0.00      PROA
ATOM   4392  HD1 ILE P 269       2.515  18.698  14.121  0.00  0.00      PROA
ATOM   4393  HD2 ILE P 269       0.897  18.888  13.362  0.00  0.00      PROA
ATOM   4394  HD3 ILE P 269       1.089  17.733  14.681  0.00  0.00      PROA
ATOM   4395  C   ILE P 269      -1.770  19.381  18.260  0.00  0.00      PROA
ATOM   4396  O   ILE P 269      -2.562  20.334  18.513  0.00  0.00      PROA
ATOM   4397  N   ILE P 270      -2.106  18.134  18.548  0.00  0.00      PROA
ATOM   4398  HN  ILE P 270      -1.447  17.421  18.322  0.00  0.00      PROA
ATOM   4399  CA  ILE P 270      -3.396  17.725  19.252  0.00  0.00      PROA
ATOM   4400  HA  ILE P 270      -4.090  18.540  19.387  0.00  0.00      PROA
ATOM   4401  CB  ILE P 270      -4.176  16.701  18.419  0.00  0.00      PROA
ATOM   4402  HB  ILE P 270      -3.523  15.813  18.285  0.00  0.00      PROA
ATOM   4403  CG2 ILE P 270      -5.399  16.121  19.246  0.00  0.00      PROA
ATOM   4404 HG21 ILE P 270      -4.914  15.517  20.043  0.00  0.00      PROA
ATOM   4405 HG22 ILE P 270      -6.035  15.439  18.642  0.00  0.00      PROA
ATOM   4406 HG23 ILE P 270      -6.038  16.898  19.718  0.00  0.00      PROA
ATOM   4407  CG1 ILE P 270      -4.547  17.193  16.967  0.00  0.00      PROA
ATOM   4408 HG11 ILE P 270      -3.738  17.754  16.452  0.00  0.00      PROA
ATOM   4409 HG12 ILE P 270      -5.301  17.961  17.242  0.00  0.00      PROA
ATOM   4410  CD  ILE P 270      -5.147  16.152  16.009  0.00  0.00      PROA
ATOM   4411  HD1 ILE P 270      -6.251  16.049  16.087  0.00  0.00      PROA
ATOM   4412  HD2 ILE P 270      -4.669  15.155  16.122  0.00  0.00      PROA
ATOM   4413  HD3 ILE P 270      -5.101  16.505  14.957  0.00  0.00      PROA
ATOM   4414  C   ILE P 270      -3.078  17.168  20.545  0.00  0.00      PROA
ATOM   4415  O   ILE P 270      -2.388  16.194  20.746  0.00  0.00      PROA
ATOM   4416  N   LYS P 271      -3.611  17.799  21.581  0.00  0.00      PROA
ATOM   4417  HN  LYS P 271      -3.863  18.760  21.502  0.00  0.00      PROA
ATOM   4418  CA  LYS P 271      -3.518  17.314  22.927  0.00  0.00      PROA
ATOM   4419  HA  LYS P 271      -3.028  16.352  22.916  0.00  0.00      PROA
ATOM   4420  CB  LYS P 271      -2.602  18.225  23.812  0.00  0.00      PROA
ATOM   4421  HB1 LYS P 271      -2.660  17.737  24.808  0.00  0.00      PROA
ATOM   4422  HB2 LYS P 271      -3.120  19.170  24.080  0.00  0.00      PROA
ATOM   4423  CG  LYS P 271      -1.158  18.453  23.269  0.00  0.00      PROA
ATOM   4424  HG1 LYS P 271      -1.202  18.951  22.277  0.00  0.00      PROA
ATOM   4425  HG2 LYS P 271      -0.727  17.470  22.982  0.00  0.00      PROA
ATOM   4426  CD  LYS P 271      -0.255  19.094  24.363  0.00  0.00      PROA
ATOM   4427  HD1 LYS P 271      -0.762  20.071  24.515  0.00  0.00      PROA
ATOM   4428  HD2 LYS P 271       0.751  19.179  23.900  0.00  0.00      PROA
ATOM   4429  CE  LYS P 271      -0.184  18.281  25.676  0.00  0.00      PROA
ATOM   4430  HE1 LYS P 271      -0.092  17.191  25.486  0.00  0.00      PROA
ATOM   4431  HE2 LYS P 271      -1.036  18.544  26.339  0.00  0.00      PROA
ATOM   4432  NZ  LYS P 271       1.034  18.624  26.351  0.00  0.00      PROA
ATOM   4433  HZ1 LYS P 271       1.308  17.992  27.130  0.00  0.00      PROA
ATOM   4434  HZ2 LYS P 271       1.072  19.621  26.645  0.00  0.00      PROA
ATOM   4435  HZ3 LYS P 271       1.838  18.510  25.702  0.00  0.00      PROA
ATOM   4436  C   LYS P 271      -4.895  17.048  23.608  0.00  0.00      PROA
ATOM   4437  O   LYS P 271      -5.677  17.927  23.719  0.00  0.00      PROA
ATOM   4438  N   GLN P 272      -5.096  15.820  24.143  0.00  0.00      PROA
ATOM   4439  HN  GLN P 272      -4.403  15.111  24.251  0.00  0.00      PROA
ATOM   4440  CA  GLN P 272      -6.428  15.278  24.246  0.00  0.00      PROA
ATOM   4441  HA  GLN P 272      -7.116  15.974  24.704  0.00  0.00      PROA
ATOM   4442  CB  GLN P 272      -7.143  15.132  22.845  0.00  0.00      PROA
ATOM   4443  HB1 GLN P 272      -7.477  14.077  22.735  0.00  0.00      PROA
ATOM   4444  HB2 GLN P 272      -6.286  15.230  22.145  0.00  0.00      PROA
ATOM   4445  CG  GLN P 272      -8.105  16.307  22.375  0.00  0.00      PROA
ATOM   4446  HG1 GLN P 272      -8.240  16.143  21.285  0.00  0.00      PROA
ATOM   4447  HG2 GLN P 272      -7.684  17.327  22.509  0.00  0.00      PROA
ATOM   4448  CD  GLN P 272      -9.495  16.308  22.934  0.00  0.00      PROA
```

ATOM 4449 OE1 GLN P 272 -9.740 15.667 23.997 0.00 0.00 PROA
ATOM 4450 NE2 GLN P 272 -10.420 16.896 22.145 0.00 0.00 PROA
ATOM 4451 HE21 GLN P 272 -11.405 16.783 22.272 0.00 0.00 PROA
ATOM 4452 HE22 GLN P 272 -10.110 17.393 21.334 0.00 0.00 PROA
ATOM 4453 C GLN P 272 -6.616 14.087 25.161 0.00 0.00 PROA
ATOM 4454 O GLN P 272 -5.642 13.537 25.584 0.00 0.00 PROA
ATOM 4455 N GLY P 273 -7.914 13.883 25.499 0.00 0.00 PROA
ATOM 4456 HN GLY P 273 -8.643 14.417 25.077 0.00 0.00 PROA
ATOM 4457 CA GLY P 273 -8.347 12.912 26.409 0.00 0.00 PROA
ATOM 4458 HA1 GLY P 273 -9.415 13.018 26.533 0.00 0.00 PROA
ATOM 4459 HA2 GLY P 273 -7.813 13.115 27.325 0.00 0.00 PROA
ATOM 4460 C GLY P 273 -8.107 11.447 25.978 0.00 0.00 PROA
ATOM 4461 O GLY P 273 -7.865 11.043 24.841 0.00 0.00 PROA
ATOM 4462 N CYS P 274 -8.200 10.522 26.960 0.00 0.00 PROA
ATOM 4463 HN CYS P 274 -8.480 10.746 27.890 0.00 0.00 PROA
ATOM 4464 CA CYS P 274 -8.214 9.059 26.751 0.00 0.00 PROA
ATOM 4465 HA CYS P 274 -7.311 8.746 26.249 0.00 0.00 PROA
ATOM 4466 CB CYS P 274 -8.309 8.421 28.170 0.00 0.00 PROA
ATOM 4467 HB1 CYS P 274 -8.511 7.329 28.144 0.00 0.00 PROA
ATOM 4468 HB2 CYS P 274 -9.222 8.905 28.579 0.00 0.00 PROA
ATOM 4469 SG CYS P 274 -6.887 8.798 29.225 0.00 0.00 PROA
ATOM 4470 C CYS P 274 -9.429 8.576 25.844 0.00 0.00 PROA
ATOM 4471 O CYS P 274 -9.341 7.737 24.922 0.00 0.00 PROA
ATOM 4472 N GLU P 275 -10.603 9.159 26.149 0.00 0.00 PROA
ATOM 4473 HN GLU P 275 -10.765 9.723 26.955 0.00 0.00 PROA
ATOM 4474 CA GLU P 275 -11.797 9.001 25.369 0.00 0.00 PROA
ATOM 4475 HA GLU P 275 -11.997 7.943 25.287 0.00 0.00 PROA
ATOM 4476 CB GLU P 275 -13.050 9.779 25.935 0.00 0.00 PROA
ATOM 4477 HB1 GLU P 275 -13.853 9.864 25.173 0.00 0.00 PROA
ATOM 4478 HB2 GLU P 275 -12.749 10.819 26.185 0.00 0.00 PROA
ATOM 4479 CG GLU P 275 -13.730 9.196 27.232 0.00 0.00 PROA
ATOM 4480 HG1 GLU P 275 -14.634 9.824 27.385 0.00 0.00 PROA
ATOM 4481 HG2 GLU P 275 -13.012 9.300 28.073 0.00 0.00 PROA
ATOM 4482 CD GLU P 275 -14.155 7.706 27.038 0.00 0.00 PROA
ATOM 4483 OE1 GLU P 275 -13.627 6.856 27.772 0.00 0.00 PROA
ATOM 4484 OE2 GLU P 275 -15.128 7.422 26.278 0.00 0.00 PROA
ATOM 4485 C GLU P 275 -11.682 9.327 23.829 0.00 0.00 PROA
ATOM 4486 O GLU P 275 -11.957 8.491 22.906 0.00 0.00 PROA
ATOM 4487 N PHE P 276 -11.131 10.493 23.573 0.00 0.00 PROA
ATOM 4488 HN PHE P 276 -11.101 11.101 24.362 0.00 0.00 PROA
ATOM 4489 CA PHE P 276 -10.870 10.950 22.215 0.00 0.00 PROA
ATOM 4490 HA PHE P 276 -11.719 10.853 21.555 0.00 0.00 PROA
ATOM 4491 CB PHE P 276 -10.317 12.412 22.179 0.00 0.00 PROA
ATOM 4492 HB1 PHE P 276 -9.634 12.549 23.045 0.00 0.00 PROA
ATOM 4493 HB2 PHE P 276 -11.091 13.118 22.549 0.00 0.00 PROA
ATOM 4494 CG PHE P 276 -9.740 12.823 20.834 0.00 0.00 PROA
ATOM 4495 CD1 PHE P 276 -10.620 13.116 19.781 0.00 0.00 PROA
ATOM 4496 HD1 PHE P 276 -11.696 13.212 19.794 0.00 0.00 PROA
ATOM 4497 CE1 PHE P 276 -9.998 13.525 18.576 0.00 0.00 PROA
ATOM 4498 HE1 PHE P 276 -10.598 13.750 17.707 0.00 0.00 PROA
ATOM 4499 CZ PHE P 276 -8.660 13.374 18.348 0.00 0.00 PROA
ATOM 4500 HZ PHE P 276 -8.171 13.552 17.401 0.00 0.00 PROA
ATOM 4501 CD2 PHE P 276 -8.356 12.757 20.643 0.00 0.00 PROA
ATOM 4502 HD2 PHE P 276 -7.754 12.355 21.445 0.00 0.00 PROA
ATOM 4503 CE2 PHE P 276 -7.837 13.096 19.410 0.00 0.00 PROA
ATOM 4504 HE2 PHE P 276 -6.769 13.135 19.251 0.00 0.00 PROA
ATOM 4505 C PHE P 276 -9.843 10.031 21.538 0.00 0.00 PROA
ATOM 4506 O PHE P 276 -10.091 9.542 20.423 0.00 0.00 PROA
ATOM 4507 N GLU P 277 -8.708 9.760 22.255 0.00 0.00 PROA
ATOM 4508 HN GLU P 277 -8.490 10.230 23.107 0.00 0.00 PROA
ATOM 4509 CA GLU P 277 -7.806 8.705 21.805 0.00 0.00 PROA

ATOM 4510 HA GLU P 277 -7.543 9.033 20.810 0.00 0.00 PROA
ATOM 4511 CB GLU P 277 -6.460 8.716 22.644 0.00 0.00 PROA
ATOM 4512 HB1 GLU P 277 -5.856 7.838 22.333 0.00 0.00 PROA
ATOM 4513 HB2 GLU P 277 -6.785 8.573 23.697 0.00 0.00 PROA
ATOM 4514 CG GLU P 277 -5.621 9.933 22.356 0.00 0.00 PROA
ATOM 4515 HG1 GLU P 277 -6.153 10.850 22.688 0.00 0.00 PROA
ATOM 4516 HG2 GLU P 277 -5.386 10.054 21.276 0.00 0.00 PROA
ATOM 4517 CD GLU P 277 -4.285 9.866 23.110 0.00 0.00 PROA
ATOM 4518 OE1 GLU P 277 -3.265 10.326 22.549 0.00 0.00 PROA
ATOM 4519 OE2 GLU P 277 -4.241 9.306 24.221 0.00 0.00 PROA
ATOM 4520 C GLU P 277 -8.331 7.250 21.523 0.00 0.00 PROA
ATOM 4521 O GLU P 277 -8.094 6.727 20.466 0.00 0.00 PROA
ATOM 4522 N ASN P 278 -9.212 6.644 22.350 0.00 0.00 PROA
ATOM 4523 HN ASN P 278 -9.414 7.132 23.195 0.00 0.00 PROA
ATOM 4524 CA ASN P 278 -10.106 5.514 21.963 0.00 0.00 PROA
ATOM 4525 HA ASN P 278 -9.606 4.594 21.700 0.00 0.00 PROA
ATOM 4526 CB ASN P 278 -10.989 5.022 23.096 0.00 0.00 PROA
ATOM 4527 HB1 ASN P 278 -11.612 4.115 22.947 0.00 0.00 PROA
ATOM 4528 HB2 ASN P 278 -11.573 5.924 23.380 0.00 0.00 PROA
ATOM 4529 CG ASN P 278 -10.039 4.584 24.242 0.00 0.00 PROA
ATOM 4530 OD1 ASN P 278 -8.964 3.966 24.113 0.00 0.00 PROA
ATOM 4531 ND2 ASN P 278 -10.376 4.993 25.477 0.00 0.00 PROA
ATOM 4532 HD21 ASN P 278 -11.305 5.294 25.692 0.00 0.00 PROA
ATOM 4533 HD22 ASN P 278 -9.947 4.561 26.270 0.00 0.00 PROA
ATOM 4534 C ASN P 278 -10.941 5.835 20.677 0.00 0.00 PROA
ATOM 4535 O ASN P 278 -11.020 4.947 19.827 0.00 0.00 PROA
ATOM 4536 N THR P 279 -11.598 7.013 20.479 0.00 0.00 PROA
ATOM 4537 HN THR P 279 -11.677 7.703 21.195 0.00 0.00 PROA
ATOM 4538 CA THR P 279 -12.382 7.171 19.198 0.00 0.00 PROA
ATOM 4539 HA THR P 279 -12.956 6.268 19.053 0.00 0.00 PROA
ATOM 4540 CB THR P 279 -13.100 8.493 19.154 0.00 0.00 PROA
ATOM 4541 HB THR P 279 -12.374 9.302 18.929 0.00 0.00 PROA
ATOM 4542 OG1 THR P 279 -13.559 8.840 20.401 0.00 0.00 PROA
ATOM 4543 HG1 THR P 279 -13.769 9.769 20.284 0.00 0.00 PROA
ATOM 4544 CG2 THR P 279 -14.162 8.595 18.081 0.00 0.00 PROA
ATOM 4545 HG21 THR P 279 -14.802 9.503 18.060 0.00 0.00 PROA
ATOM 4546 HG22 THR P 279 -14.848 7.722 18.125 0.00 0.00 PROA
ATOM 4547 HG23 THR P 279 -13.712 8.429 17.079 0.00 0.00 PROA
ATOM 4548 C THR P 279 -11.434 7.215 17.953 0.00 0.00 PROA
ATOM 4549 O THR P 279 -11.672 6.540 16.979 0.00 0.00 PROA
ATOM 4550 N VAL P 280 -10.276 7.916 18.103 0.00 0.00 PROA
ATOM 4551 HN VAL P 280 -10.094 8.290 19.009 0.00 0.00 PROA
ATOM 4552 CA VAL P 280 -9.213 7.889 17.099 0.00 0.00 PROA
ATOM 4553 HA VAL P 280 -9.580 8.378 16.208 0.00 0.00 PROA
ATOM 4554 CB VAL P 280 -7.959 8.731 17.456 0.00 0.00 PROA
ATOM 4555 HB VAL P 280 -7.572 8.587 18.487 0.00 0.00 PROA
ATOM 4556 CG1 VAL P 280 -6.651 8.398 16.647 0.00 0.00 PROA
ATOM 4557 HG11 VAL P 280 -6.847 8.231 15.566 0.00 0.00 PROA
ATOM 4558 HG12 VAL P 280 -6.254 7.477 17.126 0.00 0.00 PROA
ATOM 4559 HG13 VAL P 280 -5.878 9.174 16.831 0.00 0.00 PROA
ATOM 4560 CG2 VAL P 280 -8.295 10.210 17.254 0.00 0.00 PROA
ATOM 4561 HG21 VAL P 280 -7.488 10.888 17.604 0.00 0.00 PROA
ATOM 4562 HG22 VAL P 280 -9.257 10.514 17.720 0.00 0.00 PROA
ATOM 4563 HG23 VAL P 280 -8.450 10.366 16.165 0.00 0.00 PROA
ATOM 4564 C VAL P 280 -8.733 6.451 16.761 0.00 0.00 PROA
ATOM 4565 O VAL P 280 -8.618 6.153 15.569 0.00 0.00 PROA
ATOM 4566 N HSE P 281 -8.548 5.531 17.761 0.00 0.00 PROA
ATOM 4567 HN HSE P 281 -8.797 5.915 18.647 0.00 0.00 PROA
ATOM 4568 CA HSE P 281 -7.956 4.167 17.593 0.00 0.00 PROA
ATOM 4569 HA HSE P 281 -7.186 4.265 16.842 0.00 0.00 PROA
ATOM 4570 CB HSE P 281 -7.450 3.708 19.014 0.00 0.00 PROA

ATOM	4571	HB1	HSE	P	281	-8.230	3.144	19.568	0.00	0.00	PROA
ATOM	4572	HB2	HSE	P	281	-6.954	4.553	19.537	0.00	0.00	PROA
ATOM	4573	ND1	HSE	P	281	-5.255	3.144	17.977	0.00	0.00	PROA
ATOM	4574	CG	HSE	P	281	-6.304	2.746	18.775	0.00	0.00	PROA
ATOM	4575	CE1	HSE	P	281	-4.608	2.084	17.761	0.00	0.00	PROA
ATOM	4576	HE1	HSE	P	281	-3.809	1.996	17.025	0.00	0.00	PROA
ATOM	4577	NE2	HSE	P	281	-5.041	1.037	18.465	0.00	0.00	PROA
ATOM	4578	HE2	HSE	P	281	-4.709	0.098	18.370	0.00	0.00	PROA
ATOM	4579	CD2	HSE	P	281	-6.158	1.425	19.143	0.00	0.00	PROA
ATOM	4580	HD2	HSE	P	281	-6.943	0.825	19.586	0.00	0.00	PROA
ATOM	4581	C	HSE	P	281	-8.966	3.220	17.004	0.00	0.00	PROA
ATOM	4582	O	HSE	P	281	-8.585	2.298	16.333	0.00	0.00	PROA
ATOM	4583	N	LYS	P	282	-10.295	3.473	17.235	0.00	0.00	PROA
ATOM	4584	HN	LYS	P	282	-10.585	4.127	17.929	0.00	0.00	PROA
ATOM	4585	CA	LYS	P	282	-11.372	2.810	16.606	0.00	0.00	PROA
ATOM	4586	HA	LYS	P	282	-11.240	1.738	16.610	0.00	0.00	PROA
ATOM	4587	CB	LYS	P	282	-12.654	3.267	17.377	0.00	0.00	PROA
ATOM	4588	HB1	LYS	P	282	-12.692	4.377	17.383	0.00	0.00	PROA
ATOM	4589	HB2	LYS	P	282	-12.512	2.879	18.408	0.00	0.00	PROA
ATOM	4590	CG	LYS	P	282	-13.982	2.698	16.850	0.00	0.00	PROA
ATOM	4591	HG1	LYS	P	282	-14.817	2.793	17.577	0.00	0.00	PROA
ATOM	4592	HG2	LYS	P	282	-13.789	1.622	16.655	0.00	0.00	PROA
ATOM	4593	CD	LYS	P	282	-14.576	3.181	15.462	0.00	0.00	PROA
ATOM	4594	HD1	LYS	P	282	-15.424	2.500	15.237	0.00	0.00	PROA
ATOM	4595	HD2	LYS	P	282	-13.884	2.766	14.698	0.00	0.00	PROA
ATOM	4596	CE	LYS	P	282	-14.780	4.668	15.345	0.00	0.00	PROA
ATOM	4597	HE1	LYS	P	282	-14.993	4.941	14.290	0.00	0.00	PROA
ATOM	4598	HE2	LYS	P	282	-13.817	5.109	15.682	0.00	0.00	PROA
ATOM	4599	NZ	LYS	P	282	-15.886	5.351	16.155	0.00	0.00	PROA
ATOM	4600	HZ1	LYS	P	282	-15.651	5.170	17.152	0.00	0.00	PROA
ATOM	4601	HZ2	LYS	P	282	-16.796	4.902	15.930	0.00	0.00	PROA
ATOM	4602	HZ3	LYS	P	282	-15.890	6.389	16.095	0.00	0.00	PROA
ATOM	4603	C	LYS	P	282	-11.427	3.028	15.135	0.00	0.00	PROA
ATOM	4604	O	LYS	P	282	-11.493	2.082	14.342	0.00	0.00	PROA
ATOM	4605	N	TRP	P	283	-11.254	4.250	14.818	0.00	0.00	PROA
ATOM	4606	HN	TRP	P	283	-11.236	4.996	15.479	0.00	0.00	PROA
ATOM	4607	CA	TRP	P	283	-11.354	4.632	13.404	0.00	0.00	PROA
ATOM	4608	HA	TRP	P	283	-12.198	4.117	12.968	0.00	0.00	PROA
ATOM	4609	CB	TRP	P	283	-11.499	6.122	13.107	0.00	0.00	PROA
ATOM	4610	HB1	TRP	P	283	-11.115	6.417	12.108	0.00	0.00	PROA
ATOM	4611	HB2	TRP	P	283	-10.885	6.622	13.886	0.00	0.00	PROA
ATOM	4612	CG	TRP	P	283	-12.847	6.750	13.261	0.00	0.00	PROA
ATOM	4613	CD1	TRP	P	283	-13.282	7.475	14.321	0.00	0.00	PROA
ATOM	4614	HD1	TRP	P	283	-12.669	7.607	15.200	0.00	0.00	PROA
ATOM	4615	NE1	TRP	P	283	-14.588	7.765	14.132	0.00	0.00	PROA
ATOM	4616	HE1	TRP	P	283	-15.199	8.269	14.702	0.00	0.00	PROA
ATOM	4617	CE2	TRP	P	283	-15.002	7.273	12.888	0.00	0.00	PROA
ATOM	4618	CD2	TRP	P	283	-13.887	6.650	12.283	0.00	0.00	PROA
ATOM	4619	CE3	TRP	P	283	-14.024	6.179	10.977	0.00	0.00	PROA
ATOM	4620	HE3	TRP	P	283	-13.270	5.479	10.647	0.00	0.00	PROA
ATOM	4621	CZ3	TRP	P	283	-15.249	6.220	10.275	0.00	0.00	PROA
ATOM	4622	HZ3	TRP	P	283	-15.378	5.685	9.346	0.00	0.00	PROA
ATOM	4623	CZ2	TRP	P	283	-16.157	7.437	12.170	0.00	0.00	PROA
ATOM	4624	HZ2	TRP	P	283	-17.021	7.839	12.678	0.00	0.00	PROA
ATOM	4625	CH2	TRP	P	283	-16.269	6.894	10.901	0.00	0.00	PROA
ATOM	4626	HH2	TRP	P	283	-17.246	7.047	10.467	0.00	0.00	PROA
ATOM	4627	C	TRP	P	283	-10.122	4.117	12.584	0.00	0.00	PROA
ATOM	4628	O	TRP	P	283	-10.213	3.852	11.371	0.00	0.00	PROA
ATOM	4629	N	ILE	P	284	-8.906	4.016	13.249	0.00	0.00	PROA
ATOM	4630	HN	ILE	P	284	-8.787	4.413	14.156	0.00	0.00	PROA
ATOM	4631	CA	ILE	P	284	-7.706	3.484	12.634	0.00	0.00	PROA

ATOM	4632	HA	ILE	P	284	-7.521	3.980	11.692	0.00	0.00	PROA
ATOM	4633	CB	ILE	P	284	-6.570	3.791	13.621	0.00	0.00	PROA
ATOM	4634	HB	ILE	P	284	-7.020	3.734	14.635	0.00	0.00	PROA
ATOM	4635	CG2	ILE	P	284	-5.449	2.757	13.464	0.00	0.00	PROA
ATOM	4636	HG21	ILE	P	284	-5.044	2.702	12.431	0.00	0.00	PROA
ATOM	4637	HG22	ILE	P	284	-5.796	1.745	13.764	0.00	0.00	PROA
ATOM	4638	HG23	ILE	P	284	-4.593	3.143	14.058	0.00	0.00	PROA
ATOM	4639	CG1	ILE	P	284	-6.261	5.309	13.414	0.00	0.00	PROA
ATOM	4640	HG11	ILE	P	284	-7.179	5.922	13.284	0.00	0.00	PROA
ATOM	4641	HG12	ILE	P	284	-5.835	5.421	12.394	0.00	0.00	PROA
ATOM	4642	CD	ILE	P	284	-5.327	5.868	14.454	0.00	0.00	PROA
ATOM	4643	HD1	ILE	P	284	-5.838	5.977	15.434	0.00	0.00	PROA
ATOM	4644	HD2	ILE	P	284	-4.961	6.881	14.181	0.00	0.00	PROA
ATOM	4645	HD3	ILE	P	284	-4.535	5.089	14.480	0.00	0.00	PROA
ATOM	4646	C	ILE	P	284	-7.929	2.091	12.157	0.00	0.00	PROA
ATOM	4647	O	ILE	P	284	-7.642	1.706	10.998	0.00	0.00	PROA
ATOM	4648	N	SER	P	285	-8.606	1.276	13.019	0.00	0.00	PROA
ATOM	4649	HN	SER	P	285	-8.879	1.659	13.899	0.00	0.00	PROA
ATOM	4650	CA	SER	P	285	-9.044	-0.079	12.690	0.00	0.00	PROA
ATOM	4651	HA	SER	P	285	-8.153	-0.521	12.269	0.00	0.00	PROA
ATOM	4652	CB	SER	P	285	-9.573	-0.955	13.914	0.00	0.00	PROA
ATOM	4653	HB1	SER	P	285	-10.427	-0.316	14.223	0.00	0.00	PROA
ATOM	4654	HB2	SER	P	285	-8.743	-0.953	14.652	0.00	0.00	PROA
ATOM	4655	OG	SER	P	285	-9.990	-2.286	13.655	0.00	0.00	PROA
ATOM	4656	HG1	SER	P	285	-9.344	-2.681	13.065	0.00	0.00	PROA
ATOM	4657	C	SER	P	285	-9.945	-0.175	11.465	0.00	0.00	PROA
ATOM	4658	O	SER	P	285	-9.622	-0.991	10.629	0.00	0.00	PROA
ATOM	4659	N	ILE	P	286	-10.827	0.793	11.208	0.00	0.00	PROA
ATOM	4660	HN	ILE	P	286	-10.922	1.539	11.862	0.00	0.00	PROA
ATOM	4661	CA	ILE	P	286	-11.593	0.830	10.052	0.00	0.00	PROA
ATOM	4662	HA	ILE	P	286	-12.041	-0.129	9.838	0.00	0.00	PROA
ATOM	4663	CB	ILE	P	286	-12.730	1.934	10.046	0.00	0.00	PROA
ATOM	4664	HB	ILE	P	286	-12.182	2.895	10.155	0.00	0.00	PROA
ATOM	4665	CG2	ILE	P	286	-13.630	1.944	8.898	0.00	0.00	PROA
ATOM	4666	HG21	ILE	P	286	-13.655	0.918	8.471	0.00	0.00	PROA
ATOM	4667	HG22	ILE	P	286	-13.177	2.508	8.055	0.00	0.00	PROA
ATOM	4668	HG23	ILE	P	286	-14.631	2.392	9.074	0.00	0.00	PROA
ATOM	4669	CG1	ILE	P	286	-13.588	1.740	11.350	0.00	0.00	PROA
ATOM	4670	HG11	ILE	P	286	-13.022	1.685	12.305	0.00	0.00	PROA
ATOM	4671	HG12	ILE	P	286	-14.060	0.740	11.246	0.00	0.00	PROA
ATOM	4672	CD	ILE	P	286	-14.751	2.765	11.594	0.00	0.00	PROA
ATOM	4673	HD1	ILE	P	286	-15.358	2.761	10.663	0.00	0.00	PROA
ATOM	4674	HD2	ILE	P	286	-14.405	3.794	11.828	0.00	0.00	PROA
ATOM	4675	HD3	ILE	P	286	-15.390	2.421	12.435	0.00	0.00	PROA
ATOM	4676	C	ILE	P	286	-10.795	1.088	8.850	0.00	0.00	PROA
ATOM	4677	O	ILE	P	286	-11.002	0.485	7.835	0.00	0.00	PROA
ATOM	4678	N	THR	P	287	-9.773	1.937	8.894	0.00	0.00	PROA
ATOM	4679	HN	THR	P	287	-9.563	2.417	9.743	0.00	0.00	PROA
ATOM	4680	CA	THR	P	287	-8.757	2.184	7.770	0.00	0.00	PROA
ATOM	4681	HA	THR	P	287	-9.357	2.373	6.892	0.00	0.00	PROA
ATOM	4682	CB	THR	P	287	-7.847	3.406	7.827	0.00	0.00	PROA
ATOM	4683	HB	THR	P	287	-7.185	3.644	6.968	0.00	0.00	PROA
ATOM	4684	OG1	THR	P	287	-6.792	3.345	8.821	0.00	0.00	PROA
ATOM	4685	HG1	THR	P	287	-7.125	2.893	9.600	0.00	0.00	PROA
ATOM	4686	CG2	THR	P	287	-8.638	4.657	8.205	0.00	0.00	PROA
ATOM	4687	HG21	THR	P	287	-9.345	4.541	9.055	0.00	0.00	PROA
ATOM	4688	HG22	THR	P	287	-9.398	4.778	7.404	0.00	0.00	PROA
ATOM	4689	HG23	THR	P	287	-7.969	5.536	8.326	0.00	0.00	PROA
ATOM	4690	C	THR	P	287	-7.824	0.996	7.388	0.00	0.00	PROA
ATOM	4691	O	THR	P	287	-7.591	0.764	6.213	0.00	0.00	PROA
ATOM	4692	N	GLU	P	288	-7.326	0.205	8.422	0.00	0.00	PROA

ATOM	4693	HN	GLU	P	288	-7.620	0.605	9.287	0.00	0.00	PROA
ATOM	4694	CA	GLU	P	288	-6.505	-1.025	8.426	0.00	0.00	PROA
ATOM	4695	HA	GLU	P	288	-5.673	-0.850	7.760	0.00	0.00	PROA
ATOM	4696	CB	GLU	P	288	-6.056	-1.513	9.858	0.00	0.00	PROA
ATOM	4697	HB1	GLU	P	288	-5.834	-2.590	9.701	0.00	0.00	PROA
ATOM	4698	HB2	GLU	P	288	-7.031	-1.496	10.389	0.00	0.00	PROA
ATOM	4699	CG	GLU	P	288	-4.922	-0.705	10.492	0.00	0.00	PROA
ATOM	4700	HG1	GLU	P	288	-5.092	0.379	10.314	0.00	0.00	PROA
ATOM	4701	HG2	GLU	P	288	-3.923	-0.953	10.074	0.00	0.00	PROA
ATOM	4702	CD	GLU	P	288	-4.884	-0.739	12.013	0.00	0.00	PROA
ATOM	4703	OE1	GLU	P	288	-5.768	-1.231	12.729	0.00	0.00	PROA
ATOM	4704	OE2	GLU	P	288	-3.822	-0.338	12.526	0.00	0.00	PROA
ATOM	4705	C	GLU	P	288	-7.402	-2.069	7.733	0.00	0.00	PROA
ATOM	4706	O	GLU	P	288	-7.039	-2.825	6.849	0.00	0.00	PROA
ATOM	4707	N	ALA	P	289	-8.662	-2.025	8.128	0.00	0.00	PROA
ATOM	4708	HN	ALA	P	289	-8.773	-1.468	8.947	0.00	0.00	PROA
ATOM	4709	CA	ALA	P	289	-9.701	-2.847	7.490	0.00	0.00	PROA
ATOM	4710	HA	ALA	P	289	-9.380	-3.876	7.431	0.00	0.00	PROA
ATOM	4711	CB	ALA	P	289	-10.999	-2.653	8.274	0.00	0.00	PROA
ATOM	4712	HB1	ALA	P	289	-11.819	-3.312	7.919	0.00	0.00	PROA
ATOM	4713	HB2	ALA	P	289	-11.422	-1.629	8.195	0.00	0.00	PROA
ATOM	4714	HB3	ALA	P	289	-10.823	-2.791	9.362	0.00	0.00	PROA
ATOM	4715	C	ALA	P	289	-9.862	-2.597	5.976	0.00	0.00	PROA
ATOM	4716	O	ALA	P	289	-9.908	-3.480	5.181	0.00	0.00	PROA
ATOM	4717	N	LEU	P	290	-9.928	-1.287	5.532	0.00	0.00	PROA
ATOM	4718	HN	LEU	P	290	-9.993	-0.538	6.187	0.00	0.00	PROA
ATOM	4719	CA	LEU	P	290	-9.948	-0.980	4.087	0.00	0.00	PROA
ATOM	4720	HA	LEU	P	290	-10.743	-1.544	3.624	0.00	0.00	PROA
ATOM	4721	CB	LEU	P	290	-10.252	0.496	3.994	0.00	0.00	PROA
ATOM	4722	HB1	LEU	P	290	-9.512	0.956	4.682	0.00	0.00	PROA
ATOM	4723	HB2	LEU	P	290	-11.315	0.720	4.226	0.00	0.00	PROA
ATOM	4724	CG	LEU	P	290	-10.159	1.174	2.640	0.00	0.00	PROA
ATOM	4725	HG	LEU	P	290	-9.102	1.310	2.324	0.00	0.00	PROA
ATOM	4726	CD1	LEU	P	290	-10.838	0.418	1.546	0.00	0.00	PROA
ATOM	4727	HD11	LEU	P	290	-11.878	0.122	1.801	0.00	0.00	PROA
ATOM	4728	HD12	LEU	P	290	-10.297	-0.527	1.324	0.00	0.00	PROA
ATOM	4729	HD13	LEU	P	290	-10.852	1.066	0.644	0.00	0.00	PROA
ATOM	4730	CD2	LEU	P	290	-10.792	2.543	2.735	0.00	0.00	PROA
ATOM	4731	HD21	LEU	P	290	-10.334	3.073	3.597	0.00	0.00	PROA
ATOM	4732	HD22	LEU	P	290	-11.894	2.405	2.760	0.00	0.00	PROA
ATOM	4733	HD23	LEU	P	290	-10.566	3.126	1.816	0.00	0.00	PROA
ATOM	4734	C	LEU	P	290	-8.652	-1.419	3.410	0.00	0.00	PROA
ATOM	4735	O	LEU	P	290	-8.592	-1.907	2.234	0.00	0.00	PROA
ATOM	4736	N	ALA	P	291	-7.529	-1.385	4.103	0.00	0.00	PROA
ATOM	4737	HN	ALA	P	291	-7.554	-0.951	5.000	0.00	0.00	PROA
ATOM	4738	CA	ALA	P	291	-6.311	-1.900	3.461	0.00	0.00	PROA
ATOM	4739	HA	ALA	P	291	-6.124	-1.332	2.561	0.00	0.00	PROA
ATOM	4740	CB	ALA	P	291	-5.050	-1.816	4.451	0.00	0.00	PROA
ATOM	4741	HB1	ALA	P	291	-4.078	-1.661	3.937	0.00	0.00	PROA
ATOM	4742	HB2	ALA	P	291	-4.938	-2.639	5.189	0.00	0.00	PROA
ATOM	4743	HB3	ALA	P	291	-5.265	-0.960	5.125	0.00	0.00	PROA
ATOM	4744	C	ALA	P	291	-6.426	-3.447	3.148	0.00	0.00	PROA
ATOM	4745	O	ALA	P	291	-6.089	-3.842	2.026	0.00	0.00	PROA
ATOM	4746	N	PHE	P	292	-6.979	-4.343	4.077	0.00	0.00	PROA
ATOM	4747	HN	PHE	P	292	-7.162	-3.995	4.994	0.00	0.00	PROA
ATOM	4748	CA	PHE	P	292	-7.336	-5.757	3.783	0.00	0.00	PROA
ATOM	4749	HA	PHE	P	292	-6.463	-6.323	3.491	0.00	0.00	PROA
ATOM	4750	CB	PHE	P	292	-8.073	-6.410	5.093	0.00	0.00	PROA
ATOM	4751	HB1	PHE	P	292	-8.328	-7.447	4.788	0.00	0.00	PROA
ATOM	4752	HB2	PHE	P	292	-9.026	-5.844	5.170	0.00	0.00	PROA
ATOM	4753	CG	PHE	P	292	-7.111	-6.435	6.290	0.00	0.00	PROA

ATOM	4754	CD1	PHE	P	292	-5.793	-6.929	6.209	0.00	0.00	PROA
ATOM	4755	HD1	PHE	P	292	-5.452	-7.379	5.288	0.00	0.00	PROA
ATOM	4756	CE1	PHE	P	292	-4.911	-6.776	7.365	0.00	0.00	PROA
ATOM	4757	HE1	PHE	P	292	-3.847	-6.939	7.283	0.00	0.00	PROA
ATOM	4758	CZ	PHE	P	292	-5.426	-6.270	8.577	0.00	0.00	PROA
ATOM	4759	HZ	PHE	P	292	-4.723	-6.066	9.371	0.00	0.00	PROA
ATOM	4760	CD2	PHE	P	292	-7.618	-5.971	7.502	0.00	0.00	PROA
ATOM	4761	HD2	PHE	P	292	-8.655	-5.733	7.689	0.00	0.00	PROA
ATOM	4762	CE2	PHE	P	292	-6.743	-5.877	8.632	0.00	0.00	PROA
ATOM	4763	HE2	PHE	P	292	-7.121	-5.624	9.612	0.00	0.00	PROA
ATOM	4764	C	PHE	P	292	-8.205	-5.912	2.616	0.00	0.00	PROA
ATOM	4765	O	PHE	P	292	-7.971	-6.697	1.699	0.00	0.00	PROA
ATOM	4766	N	PHE	P	293	-9.280	-5.152	2.615	0.00	0.00	PROA
ATOM	4767	HN	PHE	P	293	-9.484	-4.567	3.396	0.00	0.00	PROA
ATOM	4768	CA	PHE	P	293	-10.258	-5.188	1.550	0.00	0.00	PROA
ATOM	4769	HA	PHE	P	293	-10.465	-6.236	1.387	0.00	0.00	PROA
ATOM	4770	CB	PHE	P	293	-11.603	-4.453	1.866	0.00	0.00	PROA
ATOM	4771	HB1	PHE	P	293	-12.243	-4.406	0.958	0.00	0.00	PROA
ATOM	4772	HB2	PHE	P	293	-11.277	-3.409	2.058	0.00	0.00	PROA
ATOM	4773	CG	PHE	P	293	-12.424	-5.091	2.976	0.00	0.00	PROA
ATOM	4774	CD1	PHE	P	293	-12.918	-6.452	2.859	0.00	0.00	PROA
ATOM	4775	HD1	PHE	P	293	-12.707	-7.055	1.987	0.00	0.00	PROA
ATOM	4776	CE1	PHE	P	293	-13.689	-6.923	3.913	0.00	0.00	PROA
ATOM	4777	HE1	PHE	P	293	-14.086	-7.926	3.870	0.00	0.00	PROA
ATOM	4778	CZ	PHE	P	293	-13.940	-6.147	5.075	0.00	0.00	PROA
ATOM	4779	HZ	PHE	P	293	-14.569	-6.604	5.825	0.00	0.00	PROA
ATOM	4780	CD2	PHE	P	293	-12.550	-4.349	4.153	0.00	0.00	PROA
ATOM	4781	HD2	PHE	P	293	-12.168	-3.344	4.252	0.00	0.00	PROA
ATOM	4782	CE2	PHE	P	293	-13.326	-4.886	5.222	0.00	0.00	PROA
ATOM	4783	HE2	PHE	P	293	-13.529	-4.307	6.111	0.00	0.00	PROA
ATOM	4784	C	PHE	P	293	-9.754	-4.859	0.147	0.00	0.00	PROA
ATOM	4785	O	PHE	P	293	-10.344	-5.284	-0.878	0.00	0.00	PROA
ATOM	4786	N	HSD	P	294	-8.742	-3.989	0.105	0.00	0.00	PROA
ATOM	4787	HN	HSD	P	294	-8.350	-3.503	0.882	0.00	0.00	PROA
ATOM	4788	CA	HSD	P	294	-8.142	-3.550	-1.159	0.00	0.00	PROA
ATOM	4789	HA	HSD	P	294	-8.932	-3.200	-1.808	0.00	0.00	PROA
ATOM	4790	CB	HSD	P	294	-7.154	-2.415	-1.012	0.00	0.00	PROA
ATOM	4791	HB1	HSD	P	294	-6.648	-2.641	-0.049	0.00	0.00	PROA
ATOM	4792	HB2	HSD	P	294	-7.733	-1.501	-0.757	0.00	0.00	PROA
ATOM	4793	ND1	HSD	P	294	-6.543	-1.242	-3.204	0.00	0.00	PROA
ATOM	4794	HD1	HSD	P	294	-7.369	-0.679	-3.245	0.00	0.00	PROA
ATOM	4795	CG	HSD	P	294	-6.222	-2.134	-2.155	0.00	0.00	PROA
ATOM	4796	CE1	HSD	P	294	-5.399	-0.952	-3.855	0.00	0.00	PROA
ATOM	4797	HE1	HSD	P	294	-5.243	-0.253	-4.678	0.00	0.00	PROA
ATOM	4798	NE2	HSD	P	294	-4.338	-1.581	-3.343	0.00	0.00	PROA
ATOM	4799	CD2	HSD	P	294	-4.858	-2.313	-2.261	0.00	0.00	PROA
ATOM	4800	HD2	HSD	P	294	-4.195	-2.930	-1.668	0.00	0.00	PROA
ATOM	4801	C	HSD	P	294	-7.428	-4.646	-1.991	0.00	0.00	PROA
ATOM	4802	O	HSD	P	294	-7.623	-4.836	-3.210	0.00	0.00	PROA
ATOM	4803	N	CYS	P	295	-6.689	-5.567	-1.344	0.00	0.00	PROA
ATOM	4804	HN	CYS	P	295	-6.734	-5.433	-0.357	0.00	0.00	PROA
ATOM	4805	CA	CYS	P	295	-6.061	-6.666	-2.049	0.00	0.00	PROA
ATOM	4806	HA	CYS	P	295	-5.569	-6.180	-2.879	0.00	0.00	PROA
ATOM	4807	CB	CYS	P	295	-5.014	-7.329	-1.120	0.00	0.00	PROA
ATOM	4808	HB1	CYS	P	295	-4.389	-7.985	-1.763	0.00	0.00	PROA
ATOM	4809	HB2	CYS	P	295	-5.417	-8.083	-0.410	0.00	0.00	PROA
ATOM	4810	SG	CYS	P	295	-3.891	-6.038	-0.272	0.00	0.00	PROA
ATOM	4811	HG1	CYS	P	295	-2.892	-6.111	-1.139	0.00	0.00	PROA
ATOM	4812	C	CYS	P	295	-6.987	-7.721	-2.650	0.00	0.00	PROA
ATOM	4813	O	CYS	P	295	-6.638	-8.449	-3.570	0.00	0.00	PROA
ATOM	4814	N	CYS	P	296	-8.230	-7.803	-2.219	0.00	0.00	PROA

ATOM	4815	HN	CYS	P	296	-8.581	-7.216	-1.493	0.00	0.00	PROA
ATOM	4816	CA	CYS	P	296	-9.271	-8.651	-2.777	0.00	0.00	PROA
ATOM	4817	HA	CYS	P	296	-8.799	-9.354	-3.448	0.00	0.00	PROA
ATOM	4818	CB	CYS	P	296	-10.080	-9.444	-1.867	0.00	0.00	PROA
ATOM	4819	HB1	CYS	P	296	-9.340	-10.067	-1.321	0.00	0.00	PROA
ATOM	4820	HB2	CYS	P	296	-10.796	-10.123	-2.377	0.00	0.00	PROA
ATOM	4821	SG	CYS	P	296	-11.001	-8.474	-0.696	0.00	0.00	PROA
ATOM	4822	HG1	CYS	P	296	-12.036	-8.155	-1.460	0.00	0.00	PROA
ATOM	4823	C	CYS	P	296	-10.135	-7.848	-3.745	0.00	0.00	PROA
ATOM	4824	O	CYS	P	296	-11.099	-8.361	-4.295	0.00	0.00	PROA
ATOM	4825	N	LEU	P	297	-9.885	-6.541	-4.078	0.00	0.00	PROA
ATOM	4826	HN	LEU	P	297	-9.315	-5.991	-3.472	0.00	0.00	PROA
ATOM	4827	CA	LEU	P	297	-10.484	-5.870	-5.148	0.00	0.00	PROA
ATOM	4828	HA	LEU	P	297	-11.425	-6.302	-5.454	0.00	0.00	PROA
ATOM	4829	CB	LEU	P	297	-10.802	-4.394	-4.681	0.00	0.00	PROA
ATOM	4830	HB1	LEU	P	297	-9.828	-3.922	-4.431	0.00	0.00	PROA
ATOM	4831	HB2	LEU	P	297	-11.368	-4.442	-3.727	0.00	0.00	PROA
ATOM	4832	CG	LEU	P	297	-11.651	-3.518	-5.631	0.00	0.00	PROA
ATOM	4833	HG	LEU	P	297	-11.123	-3.508	-6.608	0.00	0.00	PROA
ATOM	4834	CD1	LEU	P	297	-13.120	-4.080	-5.811	0.00	0.00	PROA
ATOM	4835	HD11	LEU	P	297	-13.693	-3.672	-4.952	0.00	0.00	PROA
ATOM	4836	HD12	LEU	P	297	-13.077	-5.190	-5.787	0.00	0.00	PROA
ATOM	4837	HD13	LEU	P	297	-13.613	-3.710	-6.736	0.00	0.00	PROA
ATOM	4838	CD2	LEU	P	297	-11.697	-2.083	-5.236	0.00	0.00	PROA
ATOM	4839	HD21	LEU	P	297	-11.850	-1.923	-4.147	0.00	0.00	PROA
ATOM	4840	HD22	LEU	P	297	-12.489	-1.518	-5.773	0.00	0.00	PROA
ATOM	4841	HD23	LEU	P	297	-10.717	-1.596	-5.428	0.00	0.00	PROA
ATOM	4842	C	LEU	P	297	-9.600	-5.776	-6.379	0.00	0.00	PROA
ATOM	4843	O	LEU	P	297	-10.147	-5.800	-7.480	0.00	0.00	PROA
ATOM	4844	N	ASN	P	298	-8.269	-5.493	-6.222	0.00	0.00	PROA
ATOM	4845	HN	ASN	P	298	-7.882	-5.281	-5.328	0.00	0.00	PROA
ATOM	4846	CA	ASN	P	298	-7.491	-5.239	-7.350	0.00	0.00	PROA
ATOM	4847	HA	ASN	P	298	-7.958	-4.425	-7.883	0.00	0.00	PROA
ATOM	4848	CB	ASN	P	298	-6.028	-4.979	-6.949	0.00	0.00	PROA
ATOM	4849	HB1	ASN	P	298	-5.491	-4.833	-7.911	0.00	0.00	PROA
ATOM	4850	HB2	ASN	P	298	-5.577	-5.893	-6.508	0.00	0.00	PROA
ATOM	4851	CG	ASN	P	298	-5.788	-3.794	-6.047	0.00	0.00	PROA
ATOM	4852	OD1	ASN	P	298	-6.447	-2.809	-6.288	0.00	0.00	PROA
ATOM	4853	ND2	ASN	P	298	-4.771	-3.932	-5.223	0.00	0.00	PROA
ATOM	4854	HD21	ASN	P	298	-4.677	-3.230	-4.517	0.00	0.00	PROA
ATOM	4855	HD22	ASN	P	298	-4.262	-4.788	-5.137	0.00	0.00	PROA
ATOM	4856	C	ASN	P	298	-7.431	-6.409	-8.360	0.00	0.00	PROA
ATOM	4857	O	ASN	P	298	-7.840	-6.026	-9.453	0.00	0.00	PROA
ATOM	4858	N	PRO	P	299	-7.236	-7.761	-8.225	0.00	0.00	PROA
ATOM	4859	CD	PRO	P	299	-6.981	-8.389	-6.910	0.00	0.00	PROA
ATOM	4860	HD1	PRO	P	299	-7.663	-8.049	-6.100	0.00	0.00	PROA
ATOM	4861	HD2	PRO	P	299	-5.952	-8.113	-6.594	0.00	0.00	PROA
ATOM	4862	CA	PRO	P	299	-7.562	-8.752	-9.279	0.00	0.00	PROA
ATOM	4863	HA	PRO	P	299	-6.901	-8.365	-10.040	0.00	0.00	PROA
ATOM	4864	CB	PRO	P	299	-7.131	-10.129	-8.629	0.00	0.00	PROA
ATOM	4865	HB1	PRO	P	299	-6.063	-10.094	-8.933	0.00	0.00	PROA
ATOM	4866	HB2	PRO	P	299	-7.682	-11.057	-8.893	0.00	0.00	PROA
ATOM	4867	CG	PRO	P	299	-7.114	-9.927	-7.037	0.00	0.00	PROA
ATOM	4868	HG1	PRO	P	299	-8.086	-10.298	-6.647	0.00	0.00	PROA
ATOM	4869	HG2	PRO	P	299	-6.312	-10.547	-6.583	0.00	0.00	PROA
ATOM	4870	C	PRO	P	299	-8.984	-8.846	-9.790	0.00	0.00	PROA
ATOM	4871	O	PRO	P	299	-9.098	-9.283	-10.932	0.00	0.00	PROA
ATOM	4872	N	ILE	P	300	-10.057	-8.373	-9.123	0.00	0.00	PROA
ATOM	4873	HN	ILE	P	300	-9.921	-8.071	-8.183	0.00	0.00	PROA
ATOM	4874	CA	ILE	P	300	-11.383	-8.377	-9.795	0.00	0.00	PROA
ATOM	4875	HA	ILE	P	300	-11.552	-9.271	-10.377	0.00	0.00	PROA

```
ATOM   4876  CB  ILE P 300     -12.472  -8.267  -8.697  0.00  0.00      PROA
ATOM   4877  HB  ILE P 300     -12.339  -7.358  -8.072  0.00  0.00      PROA
ATOM   4878  CG2 ILE P 300     -13.870  -8.207  -9.375  0.00  0.00      PROA
ATOM   4879 HG21 ILE P 300     -14.020  -8.976 -10.163  0.00  0.00      PROA
ATOM   4880 HG22 ILE P 300     -13.938  -7.190  -9.818  0.00  0.00      PROA
ATOM   4881 HG23 ILE P 300     -14.793  -8.337  -8.770  0.00  0.00      PROA
ATOM   4882  CG1 ILE P 300     -12.484  -9.469  -7.706  0.00  0.00      PROA
ATOM   4883 HG11 ILE P 300     -13.472  -9.333  -7.217  0.00  0.00      PROA
ATOM   4884 HG12 ILE P 300     -11.602  -9.351  -7.042  0.00  0.00      PROA
ATOM   4885  CD  ILE P 300     -12.506 -10.899  -8.267  0.00  0.00      PROA
ATOM   4886  HD1 ILE P 300     -13.410 -11.012  -8.904  0.00  0.00      PROA
ATOM   4887  HD2 ILE P 300     -12.572 -11.705  -7.505  0.00  0.00      PROA
ATOM   4888  HD3 ILE P 300     -11.613 -10.968  -8.925  0.00  0.00      PROA
ATOM   4889  C   ILE P 300     -11.531  -7.170 -10.840  0.00  0.00      PROA
ATOM   4890  O   ILE P 300     -12.200  -7.318 -11.879  0.00  0.00      PROA
ATOM   4891  N   LEU P 301     -10.827  -6.028 -10.669  0.00  0.00      PROA
ATOM   4892  HN  LEU P 301     -10.277  -5.906  -9.847  0.00  0.00      PROA
ATOM   4893  CA  LEU P 301     -10.654  -4.921 -11.611  0.00  0.00      PROA
ATOM   4894  HA  LEU P 301     -11.609  -4.600 -12.001  0.00  0.00      PROA
ATOM   4895  CB  LEU P 301      -9.865  -3.670 -10.985  0.00  0.00      PROA
ATOM   4896  HB1 LEU P 301      -9.721  -2.860 -11.732  0.00  0.00      PROA
ATOM   4897  HB2 LEU P 301      -8.847  -4.039 -10.734  0.00  0.00      PROA
ATOM   4898  CG  LEU P 301     -10.642  -3.137  -9.754  0.00  0.00      PROA
ATOM   4899  HG  LEU P 301     -10.981  -3.984  -9.120  0.00  0.00      PROA
ATOM   4900  CD1 LEU P 301      -9.797  -2.225  -8.810  0.00  0.00      PROA
ATOM   4901 HD11 LEU P 301      -9.497  -1.327  -9.390  0.00  0.00      PROA
ATOM   4902 HD12 LEU P 301      -8.894  -2.633  -8.308  0.00  0.00      PROA
ATOM   4903 HD13 LEU P 301     -10.345  -1.823  -7.931  0.00  0.00      PROA
ATOM   4904  CD2 LEU P 301     -11.910  -2.473 -10.143  0.00  0.00      PROA
ATOM   4905 HD21 LEU P 301     -11.650  -1.585 -10.757  0.00  0.00      PROA
ATOM   4906 HD22 LEU P 301     -12.494  -2.052  -9.297  0.00  0.00      PROA
ATOM   4907 HD23 LEU P 301     -12.609  -3.103 -10.734  0.00  0.00      PROA
ATOM   4908  C   LEU P 301      -9.994  -5.290 -12.955  0.00  0.00      PROA
ATOM   4909  O   LEU P 301     -10.116  -4.691 -14.036  0.00  0.00      PROA
ATOM   4910  N   TYR P 302      -9.241  -6.396 -12.979  0.00  0.00      PROA
ATOM   4911  HN  TYR P 302      -9.121  -6.869 -12.110  0.00  0.00      PROA
ATOM   4912  CA  TYR P 302      -8.293  -6.673 -14.082  0.00  0.00      PROA
ATOM   4913  HA  TYR P 302      -8.434  -5.933 -14.856  0.00  0.00      PROA
ATOM   4914  CB  TYR P 302      -6.870  -6.671 -13.542  0.00  0.00      PROA
ATOM   4915  HB1 TYR P 302      -6.291  -6.762 -14.486  0.00  0.00      PROA
ATOM   4916  HB2 TYR P 302      -6.687  -7.484 -12.807  0.00  0.00      PROA
ATOM   4917  CG  TYR P 302      -6.402  -5.379 -13.004  0.00  0.00      PROA
ATOM   4918  CD1 TYR P 302      -6.576  -4.069 -13.543  0.00  0.00      PROA
ATOM   4919  HD1 TYR P 302      -7.191  -4.062 -14.430  0.00  0.00      PROA
ATOM   4920  CE1 TYR P 302      -6.134  -2.980 -12.869  0.00  0.00      PROA
ATOM   4921  HE1 TYR P 302      -6.164  -1.985 -13.288  0.00  0.00      PROA
ATOM   4922  CZ  TYR P 302      -5.549  -3.130 -11.604  0.00  0.00      PROA
ATOM   4923  OH  TYR P 302      -4.900  -1.976 -11.164  0.00  0.00      PROA
ATOM   4924  HH  TYR P 302      -4.238  -2.190 -10.503  0.00  0.00      PROA
ATOM   4925  CD2 TYR P 302      -5.768  -5.471 -11.738  0.00  0.00      PROA
ATOM   4926  HD2 TYR P 302      -5.655  -6.502 -11.434  0.00  0.00      PROA
ATOM   4927  CE2 TYR P 302      -5.441  -4.366 -10.977  0.00  0.00      PROA
ATOM   4928  HE2 TYR P 302      -5.080  -4.432  -9.961  0.00  0.00      PROA
ATOM   4929  C   TYR P 302      -8.678  -8.018 -14.779  0.00  0.00      PROA
ATOM   4930  O   TYR P 302      -8.089  -8.457 -15.764  0.00  0.00      PROA
ATOM   4931  N   ALA P 303      -9.734  -8.676 -14.341  0.00  0.00      PROA
ATOM   4932  HN  ALA P 303     -10.220  -8.482 -13.493  0.00  0.00      PROA
ATOM   4933  CA  ALA P 303     -10.212  -9.923 -15.000  0.00  0.00      PROA
ATOM   4934  HA  ALA P 303      -9.302 -10.488 -15.138  0.00  0.00      PROA
ATOM   4935  CB  ALA P 303     -11.196 -10.667 -14.084  0.00  0.00      PROA
ATOM   4936  HB1 ALA P 303     -11.693 -11.522 -14.590  0.00  0.00      PROA
```

ATOM 4937 HB2 ALA P 303 -11.913 -9.916 -13.690 0.00 0.00 PROA
ATOM 4938 HB3 ALA P 303 -10.767 -11.134 -13.171 0.00 0.00 PROA
ATOM 4939 C ALA P 303 -10.748 -9.825 -16.390 0.00 0.00 PROA
ATOM 4940 O ALA P 303 -11.423 -8.871 -16.779 0.00 0.00 PROA
ATOM 4941 N PHE P 304 -10.381 -10.857 -17.213 0.00 0.00 PROA
ATOM 4942 HN PHE P 304 -9.912 -11.652 -16.835 0.00 0.00 PROA
ATOM 4943 CA PHE P 304 -11.018 -11.124 -18.550 0.00 0.00 PROA
ATOM 4944 HA PHE P 304 -10.794 -12.155 -18.782 0.00 0.00 PROA
ATOM 4945 CB PHE P 304 -12.614 -11.315 -18.518 0.00 0.00 PROA
ATOM 4946 HB1 PHE P 304 -12.958 -11.451 -19.566 0.00 0.00 PROA
ATOM 4947 HB2 PHE P 304 -13.086 -10.337 -18.284 0.00 0.00 PROA
ATOM 4948 CG PHE P 304 -13.212 -12.483 -17.729 0.00 0.00 PROA
ATOM 4949 CD1 PHE P 304 -12.947 -13.796 -18.099 0.00 0.00 PROA
ATOM 4950 HD1 PHE P 304 -12.194 -14.076 -18.821 0.00 0.00 PROA
ATOM 4951 CE1 PHE P 304 -13.584 -14.831 -17.476 0.00 0.00 PROA
ATOM 4952 HE1 PHE P 304 -13.338 -15.875 -17.599 0.00 0.00 PROA
ATOM 4953 CZ PHE P 304 -14.512 -14.563 -16.439 0.00 0.00 PROA
ATOM 4954 HZ PHE P 304 -14.972 -15.359 -15.871 0.00 0.00 PROA
ATOM 4955 CD2 PHE P 304 -14.164 -12.199 -16.747 0.00 0.00 PROA
ATOM 4956 HD2 PHE P 304 -14.383 -11.173 -16.490 0.00 0.00 PROA
ATOM 4957 CE2 PHE P 304 -14.807 -13.243 -16.096 0.00 0.00 PROA
ATOM 4958 HE2 PHE P 304 -15.523 -13.144 -15.293 0.00 0.00 PROA
ATOM 4959 C PHE P 304 -10.615 -10.301 -19.757 0.00 0.00 PROA
ATOM 4960 O PHE P 304 -10.286 -9.114 -19.629 0.00 0.00 PROA
ATOM 4961 N LEU P 305 -10.598 -10.892 -20.973 0.00 0.00 PROA
ATOM 4962 HN LEU P 305 -11.069 -11.769 -20.929 0.00 0.00 PROA
ATOM 4963 CA LEU P 305 -10.105 -10.422 -22.203 0.00 0.00 PROA
ATOM 4964 HA LEU P 305 -9.329 -9.711 -21.958 0.00 0.00 PROA
ATOM 4965 CB LEU P 305 -9.319 -11.552 -22.953 0.00 0.00 PROA
ATOM 4966 HB1 LEU P 305 -10.056 -12.278 -23.356 0.00 0.00 PROA
ATOM 4967 HB2 LEU P 305 -8.710 -11.966 -22.122 0.00 0.00 PROA
ATOM 4968 CG LEU P 305 -8.366 -10.982 -24.130 0.00 0.00 PROA
ATOM 4969 HG LEU P 305 -9.052 -10.287 -24.660 0.00 0.00 PROA
ATOM 4970 CD1 LEU P 305 -7.136 -10.418 -23.624 0.00 0.00 PROA
ATOM 4971 HD11 LEU P 305 -7.289 -9.724 -22.770 0.00 0.00 PROA
ATOM 4972 HD12 LEU P 305 -6.573 -9.957 -24.463 0.00 0.00 PROA
ATOM 4973 HD13 LEU P 305 -6.504 -11.183 -23.123 0.00 0.00 PROA
ATOM 4974 CD2 LEU P 305 -7.950 -12.077 -25.180 0.00 0.00 PROA
ATOM 4975 HD21 LEU P 305 -8.803 -12.438 -25.794 0.00 0.00 PROA
ATOM 4976 HD22 LEU P 305 -7.388 -12.868 -24.638 0.00 0.00 PROA
ATOM 4977 HD23 LEU P 305 -7.376 -11.628 -26.019 0.00 0.00 PROA
ATOM 4978 C LEU P 305 -11.231 -9.816 -23.056 0.00 0.00 PROA
ATOM 4979 O LEU P 305 -12.268 -10.418 -23.290 0.00 0.00 PROA
ATOM 4980 N GLY P 306 -11.068 -8.570 -23.550 0.00 0.00 PROA
ATOM 4981 HN GLY P 306 -10.247 -8.062 -23.301 0.00 0.00 PROA
ATOM 4982 CA GLY P 306 -12.174 -7.813 -24.189 0.00 0.00 PROA
ATOM 4983 HA1 GLY P 306 -12.121 -6.804 -23.809 0.00 0.00 PROA
ATOM 4984 HA2 GLY P 306 -13.119 -8.258 -23.915 0.00 0.00 PROA
ATOM 4985 C GLY P 306 -12.071 -7.546 -25.759 0.00 0.00 PROA
ATOM 4986 O GLY P 306 -10.987 -7.562 -26.325 0.00 0.00 PROA
ATOM 4987 N ALA P 307 -13.163 -7.316 -26.463 0.00 0.00 PROA
ATOM 4988 HN ALA P 307 -14.057 -7.375 -26.025 0.00 0.00 PROA
ATOM 4989 CA ALA P 307 -13.194 -6.772 -27.818 0.00 0.00 PROA
ATOM 4990 HA ALA P 307 -12.652 -7.482 -28.426 0.00 0.00 PROA
ATOM 4991 CB ALA P 307 -14.540 -6.706 -28.535 0.00 0.00 PROA
ATOM 4992 HB1 ALA P 307 -15.053 -5.781 -28.196 0.00 0.00 PROA
ATOM 4993 HB2 ALA P 307 -15.178 -7.589 -28.315 0.00 0.00 PROA
ATOM 4994 HB3 ALA P 307 -14.490 -6.527 -29.631 0.00 0.00 PROA
ATOM 4995 C ALA P 307 -12.554 -5.332 -27.926 0.00 0.00 PROA
ATOM 4996 O ALA P 307 -12.791 -4.384 -27.200 0.00 0.00 PROA
ATOM 4997 N LYS P 308 -11.685 -5.192 -28.941 0.00 0.00 PROA

ATOM	4998	HN	LYS	P	308	-11.498	-5.927	-29.588	0.00	0.00	PROA
ATOM	4999	CA	LYS	P	308	-10.904	-4.073	-29.150	0.00	0.00	PROA
ATOM	5000	HA	LYS	P	308	-10.768	-3.513	-28.236	0.00	0.00	PROA
ATOM	5001	CB	LYS	P	308	-9.424	-4.524	-29.554	0.00	0.00	PROA
ATOM	5002	HB1	LYS	P	308	-8.811	-4.874	-28.696	0.00	0.00	PROA
ATOM	5003	HB2	LYS	P	308	-8.972	-3.561	-29.875	0.00	0.00	PROA
ATOM	5004	CG	LYS	P	308	-9.385	-5.637	-30.629	0.00	0.00	PROA
ATOM	5005	HG1	LYS	P	308	-10.296	-5.660	-31.265	0.00	0.00	PROA
ATOM	5006	HG2	LYS	P	308	-9.678	-6.578	-30.116	0.00	0.00	PROA
ATOM	5007	CD	LYS	P	308	-8.093	-5.755	-31.556	0.00	0.00	PROA
ATOM	5008	HD1	LYS	P	308	-7.232	-5.892	-30.867	0.00	0.00	PROA
ATOM	5009	HD2	LYS	P	308	-7.971	-4.843	-32.178	0.00	0.00	PROA
ATOM	5010	CE	LYS	P	308	-7.998	-6.987	-32.587	0.00	0.00	PROA
ATOM	5011	HE1	LYS	P	308	-8.605	-6.890	-33.512	0.00	0.00	PROA
ATOM	5012	HE2	LYS	P	308	-8.374	-7.930	-32.135	0.00	0.00	PROA
ATOM	5013	NZ	LYS	P	308	-6.601	-7.340	-32.944	0.00	0.00	PROA
ATOM	5014	HZ1	LYS	P	308	-6.167	-6.418	-33.151	0.00	0.00	PROA
ATOM	5015	HZ2	LYS	P	308	-6.545	-7.985	-33.758	0.00	0.00	PROA
ATOM	5016	HZ3	LYS	P	308	-6.038	-7.773	-32.183	0.00	0.00	PROA
ATOM	5017	C	LYS	P	308	-11.396	-3.078	-30.243	0.00	0.00	PROA
ATOM	5018	O	LYS	P	308	-11.023	-1.929	-30.215	0.00	0.00	PROA
ATOM	5019	N	PHE	P	309	-12.197	-3.530	-31.199	0.00	0.00	PROA
ATOM	5020	HN	PHE	P	309	-12.361	-4.510	-31.128	0.00	0.00	PROA
ATOM	5021	CA	PHE	P	309	-12.670	-2.768	-32.372	0.00	0.00	PROA
ATOM	5022	HA	PHE	P	309	-12.108	-1.858	-32.520	0.00	0.00	PROA
ATOM	5023	CB	PHE	P	309	-12.702	-3.607	-33.695	0.00	0.00	PROA
ATOM	5024	HB1	PHE	P	309	-12.939	-2.966	-34.571	0.00	0.00	PROA
ATOM	5025	HB2	PHE	P	309	-13.287	-4.542	-33.562	0.00	0.00	PROA
ATOM	5026	CG	PHE	P	309	-11.344	-3.910	-34.143	0.00	0.00	PROA
ATOM	5027	CD1	PHE	P	309	-11.118	-4.964	-35.006	0.00	0.00	PROA
ATOM	5028	HD1	PHE	P	309	-11.935	-5.641	-35.209	0.00	0.00	PROA
ATOM	5029	CE1	PHE	P	309	-9.887	-5.218	-35.627	0.00	0.00	PROA
ATOM	5030	HE1	PHE	P	309	-9.870	-6.031	-36.338	0.00	0.00	PROA
ATOM	5031	CZ	PHE	P	309	-8.713	-4.441	-35.313	0.00	0.00	PROA
ATOM	5032	HZ	PHE	P	309	-7.737	-4.602	-35.748	0.00	0.00	PROA
ATOM	5033	CD2	PHE	P	309	-10.164	-3.240	-33.813	0.00	0.00	PROA
ATOM	5034	HD2	PHE	P	309	-10.278	-2.472	-33.061	0.00	0.00	PROA
ATOM	5035	CE2	PHE	P	309	-8.944	-3.458	-34.331	0.00	0.00	PROA
ATOM	5036	HE2	PHE	P	309	-8.124	-2.834	-34.008	0.00	0.00	PROA
ATOM	5037	C	PHE	P	309	-14.104	-2.171	-32.187	0.00	0.00	PROA
ATOM	5038	O	PHE	P	309	-14.877	-1.900	-33.076	0.00	0.00	PROA
ATOM	5039	N	LYS	P	310	-14.442	-1.946	-30.923	0.00	0.00	PROA
ATOM	5040	HN	LYS	P	310	-13.911	-2.224	-30.127	0.00	0.00	PROA
ATOM	5041	CA	LYS	P	310	-15.724	-1.362	-30.420	0.00	0.00	PROA
ATOM	5042	HA	LYS	P	310	-16.489	-2.024	-30.799	0.00	0.00	PROA
ATOM	5043	CB	LYS	P	310	-15.802	-1.445	-28.830	0.00	0.00	PROA
ATOM	5044	HB1	LYS	P	310	-16.709	-0.871	-28.540	0.00	0.00	PROA
ATOM	5045	HB2	LYS	P	310	-14.931	-0.878	-28.437	0.00	0.00	PROA
ATOM	5046	CG	LYS	P	310	-15.722	-2.813	-28.210	0.00	0.00	PROA
ATOM	5047	HG1	LYS	P	310	-14.664	-3.138	-28.121	0.00	0.00	PROA
ATOM	5048	HG2	LYS	P	310	-16.282	-3.621	-28.728	0.00	0.00	PROA
ATOM	5049	CD	LYS	P	310	-16.324	-2.954	-26.768	0.00	0.00	PROA
ATOM	5050	HD1	LYS	P	310	-17.380	-3.296	-26.812	0.00	0.00	PROA
ATOM	5051	HD2	LYS	P	310	-16.274	-1.932	-26.337	0.00	0.00	PROA
ATOM	5052	CE	LYS	P	310	-15.658	-4.017	-25.846	0.00	0.00	PROA
ATOM	5053	HE1	LYS	P	310	-14.578	-3.824	-25.670	0.00	0.00	PROA
ATOM	5054	HE2	LYS	P	310	-15.771	-5.036	-26.273	0.00	0.00	PROA
ATOM	5055	NZ	LYS	P	310	-16.303	-4.069	-24.497	0.00	0.00	PROA
ATOM	5056	HZ1	LYS	P	310	-15.885	-4.855	-23.960	0.00	0.00	PROA
ATOM	5057	HZ2	LYS	P	310	-17.326	-4.219	-24.611	0.00	0.00	PROA
ATOM	5058	HZ3	LYS	P	310	-16.078	-3.179	-24.008	0.00	0.00	PROA

ATOM 5059 C LYS P 310 -16.164 -0.031 -31.080 0.00 0.00 PROA
ATOM 5060 O LYS P 310 -15.401 0.656 -31.753 0.00 0.00 PROA
ATOM 5061 N THR P 311 -17.431 0.357 -30.791 0.00 0.00 PROA
ATOM 5062 HN THR P 311 -18.035 -0.196 -30.222 0.00 0.00 PROA
ATOM 5063 CA THR P 311 -17.860 1.769 -30.987 0.00 0.00 PROA
ATOM 5064 HA THR P 311 -17.647 1.802 -32.045 0.00 0.00 PROA
ATOM 5065 CB THR P 311 -19.310 2.110 -30.705 0.00 0.00 PROA
ATOM 5066 HB THR P 311 -19.448 2.953 -29.994 0.00 0.00 PROA
ATOM 5067 OG1 THR P 311 -20.099 0.957 -30.365 0.00 0.00 PROA
ATOM 5068 HG1 THR P 311 -19.963 0.859 -29.420 0.00 0.00 PROA
ATOM 5069 CG2 THR P 311 -19.940 2.763 -31.955 0.00 0.00 PROA
ATOM 5070 HG21 THR P 311 -20.972 3.099 -31.718 0.00 0.00 PROA
ATOM 5071 HG22 THR P 311 -19.941 2.061 -32.816 0.00 0.00 PROA
ATOM 5072 HG23 THR P 311 -19.425 3.724 -32.169 0.00 0.00 PROA
ATOM 5073 C THR P 311 -16.956 2.773 -30.319 0.00 0.00 PROA
ATOM 5074 O THR P 311 -16.512 2.559 -29.211 0.00 0.00 PROA
ATOM 5075 N SER P 312 -16.665 3.891 -30.976 0.00 0.00 PROA
ATOM 5076 HN SER P 312 -16.994 4.201 -31.865 0.00 0.00 PROA
ATOM 5077 CA SER P 312 -15.937 4.980 -30.301 0.00 0.00 PROA
ATOM 5078 HA SER P 312 -15.057 4.467 -29.944 0.00 0.00 PROA
ATOM 5079 CB SER P 312 -15.296 5.999 -31.236 0.00 0.00 PROA
ATOM 5080 HB1 SER P 312 -14.871 5.445 -32.100 0.00 0.00 PROA
ATOM 5081 HB2 SER P 312 -14.485 6.541 -30.705 0.00 0.00 PROA
ATOM 5082 OG SER P 312 -16.332 6.869 -31.813 0.00 0.00 PROA
ATOM 5083 HG1 SER P 312 -16.943 6.383 -32.372 0.00 0.00 PROA
ATOM 5084 C SER P 312 -16.706 5.568 -29.117 0.00 0.00 PROA
ATOM 5085 O SER P 312 -17.918 5.614 -29.100 0.00 0.00 PROA
ATOM 5086 N ALA P 313 -15.925 5.767 -28.075 0.00 0.00 PROA
ATOM 5087 HN ALA P 313 -14.950 5.588 -28.178 0.00 0.00 PROA
ATOM 5088 CA ALA P 313 -16.366 6.346 -26.803 0.00 0.00 PROA
ATOM 5089 HA ALA P 313 -15.514 6.776 -26.298 0.00 0.00 PROA
ATOM 5090 CB ALA P 313 -17.399 7.493 -26.998 0.00 0.00 PROA
ATOM 5091 HB1 ALA P 313 -17.035 8.364 -27.583 0.00 0.00 PROA
ATOM 5092 HB2 ALA P 313 -17.650 7.913 -26.001 0.00 0.00 PROA
ATOM 5093 HB3 ALA P 313 -18.352 7.149 -27.455 0.00 0.00 PROA
ATOM 5094 C ALA P 313 -16.870 5.349 -25.826 0.00 0.00 PROA
ATOM 5095 O ALA P 313 -17.427 5.664 -24.806 0.00 0.00 PROA
ATOM 5096 N GLN P 314 -16.510 4.096 -26.010 0.00 0.00 PROA
ATOM 5097 HN GLN P 314 -15.901 3.734 -26.712 0.00 0.00 PROA
ATOM 5098 CA GLN P 314 -16.727 3.074 -24.955 0.00 0.00 PROA
ATOM 5099 HA GLN P 314 -16.905 3.491 -23.975 0.00 0.00 PROA
ATOM 5100 CB GLN P 314 -17.923 2.114 -25.324 0.00 0.00 PROA
ATOM 5101 HB1 GLN P 314 -18.869 2.670 -25.153 0.00 0.00 PROA
ATOM 5102 HB2 GLN P 314 -17.918 1.276 -24.595 0.00 0.00 PROA
ATOM 5103 CG GLN P 314 -17.855 1.550 -26.785 0.00 0.00 PROA
ATOM 5104 HG1 GLN P 314 -16.889 1.141 -27.149 0.00 0.00 PROA
ATOM 5105 HG2 GLN P 314 -18.148 2.395 -27.445 0.00 0.00 PROA
ATOM 5106 CD GLN P 314 -18.910 0.485 -26.886 0.00 0.00 PROA
ATOM 5107 OE1 GLN P 314 -19.895 0.825 -27.593 0.00 0.00 PROA
ATOM 5108 NE2 GLN P 314 -18.879 -0.697 -26.144 0.00 0.00 PROA
ATOM 5109 HE21 GLN P 314 -19.685 -1.285 -26.079 0.00 0.00 PROA
ATOM 5110 HE22 GLN P 314 -18.135 -0.891 -25.504 0.00 0.00 PROA
ATOM 5111 C GLN P 314 -15.517 2.176 -24.701 0.00 0.00 PROA
ATOM 5112 O GLN P 314 -14.621 2.024 -25.567 0.00 0.00 PROA
ATOM 5113 N HSD P 315 -15.461 1.629 -23.480 0.00 0.00 PROA
ATOM 5114 HN HSD P 315 -16.109 1.932 -22.785 0.00 0.00 PROA
ATOM 5115 CA HSD P 315 -14.438 0.785 -23.083 0.00 0.00 PROA
ATOM 5116 HA HSD P 315 -13.518 1.197 -23.469 0.00 0.00 PROA
ATOM 5117 CB HSD P 315 -14.479 0.413 -21.587 0.00 0.00 PROA
ATOM 5118 HB1 HSD P 315 -15.529 0.148 -21.339 0.00 0.00 PROA
ATOM 5119 HB2 HSD P 315 -14.212 1.423 -21.207 0.00 0.00 PROA

ATOM	5120	ND1	HSD	P	315	-12.274	-0.280	-20.671	0.00	0.00	PROA
ATOM	5121	HD1	HSD	P	315	-11.761	0.570	-20.787	0.00	0.00	PROA
ATOM	5122	CG	HSD	P	315	-13.513	-0.633	-21.103	0.00	0.00	PROA
ATOM	5123	CE1	HSD	P	315	-11.731	-1.378	-20.081	0.00	0.00	PROA
ATOM	5124	HE1	HSD	P	315	-10.747	-1.448	-19.618	0.00	0.00	PROA
ATOM	5125	NE2	HSD	P	315	-12.609	-2.361	-20.131	0.00	0.00	PROA
ATOM	5126	CD2	HSD	P	315	-13.769	-1.874	-20.679	0.00	0.00	PROA
ATOM	5127	HD2	HSD	P	315	-14.621	-2.497	-20.922	0.00	0.00	PROA
ATOM	5128	C	HSD	P	315	-14.274	-0.471	-23.925	0.00	0.00	PROA
ATOM	5129	O	HSD	P	315	-15.196	-1.107	-24.372	0.00	0.00	PROA
ATOM	5130	N	ALA	P	316	-13.025	-0.814	-24.159	0.00	0.00	PROA
ATOM	5131	HN	ALA	P	316	-12.328	-0.366	-23.604	0.00	0.00	PROA
ATOM	5132	CA	ALA	P	316	-12.543	-1.867	-25.007	0.00	0.00	PROA
ATOM	5133	HA	ALA	P	316	-13.040	-2.819	-24.890	0.00	0.00	PROA
ATOM	5134	CB	ALA	P	316	-12.622	-1.421	-26.505	0.00	0.00	PROA
ATOM	5135	HB1	ALA	P	316	-13.681	-1.411	-26.842	0.00	0.00	PROA
ATOM	5136	HB2	ALA	P	316	-12.005	-2.040	-27.191	0.00	0.00	PROA
ATOM	5137	HB3	ALA	P	316	-12.259	-0.371	-26.479	0.00	0.00	PROA
ATOM	5138	C	ALA	P	316	-11.116	-2.128	-24.567	0.00	0.00	PROA
ATOM	5139	O	ALA	P	316	-10.396	-1.297	-23.998	0.00	0.00	PROA
ATOM	5140	N	LEU	P	317	-10.554	-3.305	-24.775	0.00	0.00	PROA
ATOM	5141	HN	LEU	P	317	-11.093	-4.028	-25.200	0.00	0.00	PROA
ATOM	5142	CA	LEU	P	317	-9.219	-3.735	-24.377	0.00	0.00	PROA
ATOM	5143	HA	LEU	P	317	-9.070	-3.417	-23.355	0.00	0.00	PROA
ATOM	5144	CB	LEU	P	317	-9.121	-5.299	-24.433	0.00	0.00	PROA
ATOM	5145	HB1	LEU	P	317	-9.192	-5.519	-25.520	0.00	0.00	PROA
ATOM	5146	HB2	LEU	P	317	-9.975	-5.889	-24.037	0.00	0.00	PROA
ATOM	5147	CG	LEU	P	317	-7.925	-5.933	-23.777	0.00	0.00	PROA
ATOM	5148	HG	LEU	P	317	-7.080	-5.225	-23.918	0.00	0.00	PROA
ATOM	5149	CD1	LEU	P	317	-8.105	-6.149	-22.308	0.00	0.00	PROA
ATOM	5150	HD11	LEU	P	317	-7.804	-5.264	-21.708	0.00	0.00	PROA
ATOM	5151	HD12	LEU	P	317	-7.456	-6.987	-21.976	0.00	0.00	PROA
ATOM	5152	HD13	LEU	P	317	-9.171	-6.368	-22.085	0.00	0.00	PROA
ATOM	5153	CD2	LEU	P	317	-7.697	-7.324	-24.443	0.00	0.00	PROA
ATOM	5154	HD21	LEU	P	317	-8.618	-7.862	-24.755	0.00	0.00	PROA
ATOM	5155	HD22	LEU	P	317	-7.003	-7.868	-23.767	0.00	0.00	PROA
ATOM	5156	HD23	LEU	P	317	-7.135	-7.039	-25.358	0.00	0.00	PROA
ATOM	5157	C	LEU	P	317	-8.221	-3.040	-25.247	0.00	0.00	PROA
ATOM	5158	O	LEU	P	317	-8.458	-2.791	-26.440	0.00	0.00	PROA
ATOM	5159	N	THR	P	318	-6.994	-2.808	-24.739	0.00	0.00	PROA
ATOM	5160	HN	THR	P	318	-6.789	-2.902	-23.768	0.00	0.00	PROA
ATOM	5161	CA	THR	P	318	-5.950	-2.151	-25.460	0.00	0.00	PROA
ATOM	5162	HA	THR	P	318	-6.349	-1.394	-26.117	0.00	0.00	PROA
ATOM	5163	CB	THR	P	318	-4.841	-1.540	-24.490	0.00	0.00	PROA
ATOM	5164	HB	THR	P	318	-4.170	-2.304	-24.042	0.00	0.00	PROA
ATOM	5165	OG1	THR	P	318	-5.477	-0.780	-23.427	0.00	0.00	PROA
ATOM	5166	HG1	THR	P	318	-5.654	-1.416	-22.730	0.00	0.00	PROA
ATOM	5167	CG2	THR	P	318	-3.978	-0.544	-25.163	0.00	0.00	PROA
ATOM	5168	HG21	THR	P	318	-3.298	-1.153	-25.796	0.00	0.00	PROA
ATOM	5169	HG22	THR	P	318	-3.424	0.002	-24.370	0.00	0.00	PROA
ATOM	5170	HG23	THR	P	318	-4.537	0.145	-25.832	0.00	0.00	PROA
ATOM	5171	C	THR	P	318	-5.242	-3.270	-26.268	0.00	0.00	PROA
ATOM	5172	O	THR	P	318	-5.004	-4.331	-25.728	0.00	0.00	PROA
ATOM	5173	N	SER	P	319	-4.984	-2.995	-27.561	0.00	0.00	PROA
ATOM	5174	HN	SER	P	319	-5.102	-2.036	-27.808	0.00	0.00	PROA
ATOM	5175	CA	SER	P	319	-4.407	-3.929	-28.554	0.00	0.00	PROA
ATOM	5176	HA	SER	P	319	-3.613	-4.433	-28.023	0.00	0.00	PROA
ATOM	5177	CB	SER	P	319	-5.508	-4.868	-29.112	0.00	0.00	PROA
ATOM	5178	HB1	SER	P	319	-5.185	-5.506	-29.963	0.00	0.00	PROA
ATOM	5179	HB2	SER	P	319	-6.275	-4.204	-29.565	0.00	0.00	PROA
ATOM	5180	OG	SER	P	319	-6.100	-5.679	-28.087	0.00	0.00	PROA

```
ATOM   5181  HG1 SER P 319      -6.199  -5.003 -27.412  0.00  0.00      PROA
ATOM   5182  C   SER P 319      -3.808  -3.096 -29.672  0.00  0.00      PROA
ATOM   5183  OT1 SER P 319      -4.026  -1.857 -29.603  0.00  0.00      PROA
ATOM   5184  OT2 SER P 319      -3.210  -3.588 -30.618  0.00  0.00      PROA
ATOM   5185  N   LEU P   1       7.154  -1.280  27.781  0.00  0.00      PROB
ATOM   5186  HT1 LEU P   1       7.171  -0.712  26.910  0.00  0.00      PROB
ATOM   5187  HT2 LEU P   1       8.141  -1.321  28.106  0.00  0.00      PROB
ATOM   5188  HT3 LEU P   1       6.746  -0.728  28.563  0.00  0.00      PROB
ATOM   5189  CA  LEU P   1       6.557  -2.584  27.494  0.00  0.00      PROB
ATOM   5190  HA  LEU P   1       6.647  -3.102  28.437  0.00  0.00      PROB
ATOM   5191  CB  LEU P   1       5.017  -2.445  27.017  0.00  0.00      PROB
ATOM   5192  HB1 LEU P   1       4.646  -3.464  26.774  0.00  0.00      PROB
ATOM   5193  HB2 LEU P   1       4.888  -1.767  26.147  0.00  0.00      PROB
ATOM   5194  CG  LEU P   1       4.061  -1.953  28.109  0.00  0.00      PROB
ATOM   5195  HG  LEU P   1       4.347  -0.882  28.184  0.00  0.00      PROB
ATOM   5196  CD1 LEU P   1       2.621  -2.159  27.544  0.00  0.00      PROB
ATOM   5197 HD11 LEU P   1       1.826  -1.529  27.996  0.00  0.00      PROB
ATOM   5198 HD12 LEU P   1       2.296  -3.202  27.742  0.00  0.00      PROB
ATOM   5199 HD13 LEU P   1       2.600  -1.918  26.459  0.00  0.00      PROB
ATOM   5200  CD2 LEU P   1       4.175  -2.485  29.528  0.00  0.00      PROB
ATOM   5201 HD21 LEU P   1       3.341  -2.095  30.151  0.00  0.00      PROB
ATOM   5202 HD22 LEU P   1       5.135  -2.181  29.997  0.00  0.00      PROB
ATOM   5203 HD23 LEU P   1       4.072  -3.581  29.679  0.00  0.00      PROB
ATOM   5204  C   LEU P   1       7.376  -3.465  26.458  0.00  0.00      PROB
ATOM   5205  O   LEU P   1       8.432  -3.009  25.971  0.00  0.00      PROB
ATOM   5206  N   VAL P   2       6.952  -4.757  26.216  0.00  0.00      PROB
ATOM   5207  HN  VAL P   2       6.291  -5.242  26.782  0.00  0.00      PROB
ATOM   5208  CA  VAL P   2       7.605  -5.447  25.149  0.00  0.00      PROB
ATOM   5209  HA  VAL P   2       8.680  -5.392  25.236  0.00  0.00      PROB
ATOM   5210  CB  VAL P   2       7.060  -6.878  25.107  0.00  0.00      PROB
ATOM   5211  HB  VAL P   2       5.952  -6.791  25.113  0.00  0.00      PROB
ATOM   5212  CG1 VAL P   2       7.578  -7.704  23.868  0.00  0.00      PROB
ATOM   5213 HG11 VAL P   2       7.124  -8.701  24.055  0.00  0.00      PROB
ATOM   5214 HG12 VAL P   2       8.683  -7.773  23.966  0.00  0.00      PROB
ATOM   5215 HG13 VAL P   2       7.368  -7.265  22.869  0.00  0.00      PROB
ATOM   5216  CG2 VAL P   2       7.395  -7.580  26.398  0.00  0.00      PROB
ATOM   5217 HG21 VAL P   2       8.497  -7.723  26.424  0.00  0.00      PROB
ATOM   5218 HG22 VAL P   2       6.979  -8.609  26.430  0.00  0.00      PROB
ATOM   5219 HG23 VAL P   2       6.915  -7.147  27.301  0.00  0.00      PROB
ATOM   5220  C   VAL P   2       7.501  -4.741  23.700  0.00  0.00      PROB
ATOM   5221  O   VAL P   2       8.414  -4.505  22.877  0.00  0.00      PROB
ATOM   5222  N   ARG P   3       6.249  -4.275  23.424  0.00  0.00      PROB
ATOM   5223  HN  ARG P   3       5.432  -4.624  23.876  0.00  0.00      PROB
ATOM   5224  CA  ARG P   3       5.857  -3.573  22.198  0.00  0.00      PROB
ATOM   5225  HA  ARG P   3       6.490  -3.913  21.391  0.00  0.00      PROB
ATOM   5226  CB  ARG P   3       4.379  -3.857  21.763  0.00  0.00      PROB
ATOM   5227  HB1 ARG P   3       4.128  -3.346  20.809  0.00  0.00      PROB
ATOM   5228  HB2 ARG P   3       3.791  -3.433  22.605  0.00  0.00      PROB
ATOM   5229  CG  ARG P   3       4.121  -5.297  21.378  0.00  0.00      PROB
ATOM   5230  HG1 ARG P   3       4.534  -6.060  22.073  0.00  0.00      PROB
ATOM   5231  HG2 ARG P   3       4.711  -5.601  20.487  0.00  0.00      PROB
ATOM   5232  CD  ARG P   3       2.652  -5.551  21.085  0.00  0.00      PROB
ATOM   5233  HD1 ARG P   3       1.919  -5.155  21.820  0.00  0.00      PROB
ATOM   5234  HD2 ARG P   3       2.476  -6.646  21.014  0.00  0.00      PROB
ATOM   5235  NE  ARG P   3       2.447  -5.051  19.656  0.00  0.00      PROB
ATOM   5236  HE  ARG P   3       3.254  -4.846  19.102  0.00  0.00      PROB
ATOM   5237  CZ  ARG P   3       1.243  -4.743  19.216  0.00  0.00      PROB
ATOM   5238  NH1 ARG P   3       0.250  -4.703  19.997  0.00  0.00      PROB
ATOM   5239 HH11 ARG P   3       0.245  -4.888  20.980  0.00  0.00      PROB
ATOM   5240 HH12 ARG P   3      -0.566  -4.581  19.433  0.00  0.00      PROB
ATOM   5241  NH2 ARG P   3       1.123  -4.445  17.941  0.00  0.00      PROB
```

```
ATOM   5242 HH21 ARG P   3       1.946  -4.519  17.379  0.00  0.00      PROB
ATOM   5243 HH22 ARG P   3       0.369  -3.811  17.771  0.00  0.00      PROB
ATOM   5244  C   ARG P   3       6.070  -2.090  22.308  0.00  0.00      PROB
ATOM   5245  O   ARG P   3       6.142  -1.498  23.328  0.00  0.00      PROB
ATOM   5246  N   TYR P   4       6.120  -1.519  21.123  0.00  0.00      PROB
ATOM   5247  HN  TYR P   4       6.007  -2.171  20.377  0.00  0.00      PROB
ATOM   5248  CA  TYR P   4       6.153  -0.111  20.799  0.00  0.00      PROB
ATOM   5249  HA  TYR P   4       6.134  -0.032  19.722  0.00  0.00      PROB
ATOM   5250  CB  TYR P   4       4.861   0.555  21.389  0.00  0.00      PROB
ATOM   5251  HB1 TYR P   4       4.805   1.619  21.076  0.00  0.00      PROB
ATOM   5252  HB2 TYR P   4       4.857   0.617  22.499  0.00  0.00      PROB
ATOM   5253  CG  TYR P   4       3.549   0.003  20.901  0.00  0.00      PROB
ATOM   5254  CD1 TYR P   4       3.050   0.293  19.566  0.00  0.00      PROB
ATOM   5255  HD1 TYR P   4       3.652   0.983  18.993  0.00  0.00      PROB
ATOM   5256  CE1 TYR P   4       1.940  -0.389  19.084  0.00  0.00      PROB
ATOM   5257  HE1 TYR P   4       1.505  -0.087  18.143  0.00  0.00      PROB
ATOM   5258  CZ  TYR P   4       1.281  -1.194  19.928  0.00  0.00      PROB
ATOM   5259  OH  TYR P   4      -0.028  -1.569  19.564  0.00  0.00      PROB
ATOM   5260  HH  TYR P   4      -0.456  -2.009  20.303  0.00  0.00      PROB
ATOM   5261  CD2 TYR P   4       2.897  -0.972  21.673  0.00  0.00      PROB
ATOM   5262  HD2 TYR P   4       3.178  -1.234  22.682  0.00  0.00      PROB
ATOM   5263  CE2 TYR P   4       1.728  -1.560  21.219  0.00  0.00      PROB
ATOM   5264  HE2 TYR P   4       1.196  -2.352  21.725  0.00  0.00      PROB
ATOM   5265  C   TYR P   4       7.369   0.663  21.207  0.00  0.00      PROB
ATOM   5266  O   TYR P   4       8.420   0.069  21.513  0.00  0.00      PROB
ATOM   5267  N   THR P   5       7.313   1.985  21.458  0.00  0.00      PROB
ATOM   5268  HN  THR P   5       6.463   2.503  21.405  0.00  0.00      PROB
ATOM   5269  CA  THR P   5       8.393   2.825  21.932  0.00  0.00      PROB
ATOM   5270  HA  THR P   5       9.286   2.521  21.406  0.00  0.00      PROB
ATOM   5271  CB  THR P   5       8.207   4.329  21.674  0.00  0.00      PROB
ATOM   5272  HB  THR P   5       9.041   4.822  22.217  0.00  0.00      PROB
ATOM   5273  OG1 THR P   5       6.977   4.831  22.178  0.00  0.00      PROB
ATOM   5274  HG1 THR P   5       7.019   4.799  23.137  0.00  0.00      PROB
ATOM   5275  CG2 THR P   5       8.317   4.456  20.168  0.00  0.00      PROB
ATOM   5276 HG21 THR P   5       7.550   3.812  19.688  0.00  0.00      PROB
ATOM   5277 HG22 THR P   5       9.364   4.242  19.867  0.00  0.00      PROB
ATOM   5278 HG23 THR P   5       8.177   5.517  19.867  0.00  0.00      PROB
ATOM   5279  C   THR P   5       8.720   2.486  23.443  0.00  0.00      PROB
ATOM   5280  O   THR P   5       7.875   2.231  24.263  0.00  0.00      PROB
ATOM   5281  N   LYS P   6      10.016   2.456  23.829  0.00  0.00      PROB
ATOM   5282  HN  LYS P   6      10.684   2.445  23.089  0.00  0.00      PROB
ATOM   5283  CA  LYS P   6      10.353   2.240  25.228  0.00  0.00      PROB
ATOM   5284  HA  LYS P   6       9.908   1.301  25.522  0.00  0.00      PROB
ATOM   5285  CB  LYS P   6      11.843   2.034  25.462  0.00  0.00      PROB
ATOM   5286  HB1 LYS P   6      12.158   1.880  26.517  0.00  0.00      PROB
ATOM   5287  HB2 LYS P   6      12.530   2.826  25.095  0.00  0.00      PROB
ATOM   5288  CG  LYS P   6      12.392   0.850  24.661  0.00  0.00      PROB
ATOM   5289  HG1 LYS P   6      13.484   0.842  24.870  0.00  0.00      PROB
ATOM   5290  HG2 LYS P   6      12.344   1.209  23.611  0.00  0.00      PROB
ATOM   5291  CD  LYS P   6      11.849  -0.561  24.850  0.00  0.00      PROB
ATOM   5292  HD1 LYS P   6      10.976  -0.569  24.162  0.00  0.00      PROB
ATOM   5293  HD2 LYS P   6      11.390  -0.664  25.857  0.00  0.00      PROB
ATOM   5294  CE  LYS P   6      12.803  -1.640  24.351  0.00  0.00      PROB
ATOM   5295  HE1 LYS P   6      12.495  -2.651  24.694  0.00  0.00      PROB
ATOM   5296  HE2 LYS P   6      13.834  -1.428  24.707  0.00  0.00      PROB
ATOM   5297  NZ  LYS P   6      12.820  -1.586  22.917  0.00  0.00      PROB
ATOM   5298  HZ1 LYS P   6      12.788  -0.592  22.612  0.00  0.00      PROB
ATOM   5299  HZ2 LYS P   6      11.967  -2.089  22.599  0.00  0.00      PROB
ATOM   5300  HZ3 LYS P   6      13.690  -2.004  22.530  0.00  0.00      PROB
ATOM   5301  C   LYS P   6       9.799   3.292  26.267  0.00  0.00      PROB
ATOM   5302  O   LYS P   6       9.577   4.451  25.896  0.00  0.00      PROB
```

ATOM	5303	N	LYS	P	7	9.619	3.007	27.555	0.00	0.00	PROB
ATOM	5304	HN	LYS	P	7	9.932	2.096	27.810	0.00	0.00	PROB
ATOM	5305	CA	LYS	P	7	9.147	3.870	28.552	0.00	0.00	PROB
ATOM	5306	HA	LYS	P	7	9.144	3.329	29.487	0.00	0.00	PROB
ATOM	5307	CB	LYS	P	7	10.100	5.053	28.824	0.00	0.00	PROB
ATOM	5308	HB1	LYS	P	7	10.388	5.639	27.926	0.00	0.00	PROB
ATOM	5309	HB2	LYS	P	7	11.074	4.558	29.026	0.00	0.00	PROB
ATOM	5310	CG	LYS	P	7	9.730	6.092	29.872	0.00	0.00	PROB
ATOM	5311	HG1	LYS	P	7	9.463	5.522	30.787	0.00	0.00	PROB
ATOM	5312	HG2	LYS	P	7	8.740	6.487	29.559	0.00	0.00	PROB
ATOM	5313	CD	LYS	P	7	10.689	7.213	30.181	0.00	0.00	PROB
ATOM	5314	HD1	LYS	P	7	10.917	7.645	29.183	0.00	0.00	PROB
ATOM	5315	HD2	LYS	P	7	11.602	6.650	30.470	0.00	0.00	PROB
ATOM	5316	CE	LYS	P	7	10.444	8.112	31.367	0.00	0.00	PROB
ATOM	5317	HE1	LYS	P	7	10.046	7.598	32.268	0.00	0.00	PROB
ATOM	5318	HE2	LYS	P	7	9.885	9.011	31.031	0.00	0.00	PROB
ATOM	5319	NZ	LYS	P	7	11.763	8.688	31.891	0.00	0.00	PROB
ATOM	5320	HZ1	LYS	P	7	12.352	9.095	31.136	0.00	0.00	PROB
ATOM	5321	HZ2	LYS	P	7	12.294	7.961	32.411	0.00	0.00	PROB
ATOM	5322	HZ3	LYS	P	7	11.502	9.400	32.603	0.00	0.00	PROB
ATOM	5323	C	LYS	P	7	7.694	4.176	28.382	0.00	0.00	PROB
ATOM	5324	O	LYS	P	7	6.791	3.660	29.018	0.00	0.00	PROB
ATOM	5325	N	VAL	P	8	7.307	5.032	27.396	0.00	0.00	PROB
ATOM	5326	HN	VAL	P	8	8.037	5.289	26.768	0.00	0.00	PROB
ATOM	5327	CA	VAL	P	8	5.951	5.301	27.072	0.00	0.00	PROB
ATOM	5328	HA	VAL	P	8	5.233	5.096	27.852	0.00	0.00	PROB
ATOM	5329	CB	VAL	P	8	5.577	6.770	26.780	0.00	0.00	PROB
ATOM	5330	HB	VAL	P	8	6.207	7.240	25.994	0.00	0.00	PROB
ATOM	5331	CG1	VAL	P	8	4.102	6.852	26.330	0.00	0.00	PROB
ATOM	5332	HG11	VAL	P	8	3.917	6.334	25.365	0.00	0.00	PROB
ATOM	5333	HG12	VAL	P	8	3.802	7.904	26.132	0.00	0.00	PROB
ATOM	5334	HG13	VAL	P	8	3.478	6.484	27.172	0.00	0.00	PROB
ATOM	5335	CG2	VAL	P	8	5.664	7.595	28.060	0.00	0.00	PROB
ATOM	5336	HG21	VAL	P	8	5.700	8.665	27.764	0.00	0.00	PROB
ATOM	5337	HG22	VAL	P	8	6.553	7.355	28.682	0.00	0.00	PROB
ATOM	5338	HG23	VAL	P	8	4.778	7.333	28.676	0.00	0.00	PROB
ATOM	5339	C	VAL	P	8	5.681	4.547	25.779	0.00	0.00	PROB
ATOM	5340	O	VAL	P	8	6.275	4.827	24.695	0.00	0.00	PROB
ATOM	5341	N	PRO	P	9	4.621	3.640	25.772	0.00	0.00	PROB
ATOM	5342	CD	PRO	P	9	3.955	3.206	26.962	0.00	0.00	PROB
ATOM	5343	HD1	PRO	P	9	3.385	4.040	27.425	0.00	0.00	PROB
ATOM	5344	HD2	PRO	P	9	4.701	2.759	27.653	0.00	0.00	PROB
ATOM	5345	CA	PRO	P	9	4.491	2.782	24.629	0.00	0.00	PROB
ATOM	5346	HA	PRO	P	9	5.443	2.544	24.178	0.00	0.00	PROB
ATOM	5347	CB	PRO	P	9	3.800	1.591	25.265	0.00	0.00	PROB
ATOM	5348	HB1	PRO	P	9	4.585	0.889	25.620	0.00	0.00	PROB
ATOM	5349	HB2	PRO	P	9	3.066	1.049	24.632	0.00	0.00	PROB
ATOM	5350	CG	PRO	P	9	3.018	2.132	26.427	0.00	0.00	PROB
ATOM	5351	HG1	PRO	P	9	2.098	2.609	26.027	0.00	0.00	PROB
ATOM	5352	HG2	PRO	P	9	2.586	1.381	27.122	0.00	0.00	PROB
ATOM	5353	C	PRO	P	9	3.617	3.527	23.590	0.00	0.00	PROB
ATOM	5354	O	PRO	P	9	2.383	3.324	23.509	0.00	0.00	PROB
ATOM	5355	N	GLN	P	10	4.118	4.464	22.730	0.00	0.00	PROB
ATOM	5356	HN	GLN	P	10	5.045	4.808	22.858	0.00	0.00	PROB
ATOM	5357	CA	GLN	P	10	3.342	5.177	21.724	0.00	0.00	PROB
ATOM	5358	HA	GLN	P	10	2.413	5.571	22.109	0.00	0.00	PROB
ATOM	5359	CB	GLN	P	10	4.166	6.333	21.133	0.00	0.00	PROB
ATOM	5360	HB1	GLN	P	10	3.663	6.795	20.257	0.00	0.00	PROB
ATOM	5361	HB2	GLN	P	10	5.064	5.729	20.882	0.00	0.00	PROB
ATOM	5362	CG	GLN	P	10	4.493	7.470	22.148	0.00	0.00	PROB
ATOM	5363	HG1	GLN	P	10	5.225	8.136	21.641	0.00	0.00	PROB

ATOM	5364	HG2	GLN	P	10	4.899	6.995	23.067	0.00	0.00	PROB
ATOM	5365	CD	GLN	P	10	3.174	8.201	22.480	0.00	0.00	PROB
ATOM	5366	OE1	GLN	P	10	2.082	7.727	22.169	0.00	0.00	PROB
ATOM	5367	NE2	GLN	P	10	3.261	9.398	22.994	0.00	0.00	PROB
ATOM	5368	HE21	GLN	P	10	2.406	9.776	23.351	0.00	0.00	PROB
ATOM	5369	HE22	GLN	P	10	4.197	9.647	23.241	0.00	0.00	PROB
ATOM	5370	C	GLN	P	10	2.795	4.270	20.539	0.00	0.00	PROB
ATOM	5371	O	GLN	P	10	3.493	3.573	19.801	0.00	0.00	PROB
ATOM	5372	N	VAL	P	11	1.446	4.148	20.374	0.00	0.00	PROB
ATOM	5373	HN	VAL	P	11	0.778	4.746	20.809	0.00	0.00	PROB
ATOM	5374	CA	VAL	P	11	0.805	3.118	19.558	0.00	0.00	PROB
ATOM	5375	HA	VAL	P	11	1.505	2.301	19.461	0.00	0.00	PROB
ATOM	5376	CB	VAL	P	11	-0.536	2.695	20.195	0.00	0.00	PROB
ATOM	5377	HB	VAL	P	11	-1.312	3.487	20.269	0.00	0.00	PROB
ATOM	5378	CG1	VAL	P	11	-1.116	1.605	19.310	0.00	0.00	PROB
ATOM	5379	HG11	VAL	P	11	-1.950	1.083	19.826	0.00	0.00	PROB
ATOM	5380	HG12	VAL	P	11	-0.397	0.789	19.083	0.00	0.00	PROB
ATOM	5381	HG13	VAL	P	11	-1.614	1.989	18.394	0.00	0.00	PROB
ATOM	5382	CG2	VAL	P	11	-0.328	2.129	21.613	0.00	0.00	PROB
ATOM	5383	HG21	VAL	P	11	0.437	1.322	21.618	0.00	0.00	PROB
ATOM	5384	HG22	VAL	P	11	-1.314	1.784	21.992	0.00	0.00	PROB
ATOM	5385	HG23	VAL	P	11	0.026	2.885	22.345	0.00	0.00	PROB
ATOM	5386	C	VAL	P	11	0.564	3.681	18.176	0.00	0.00	PROB
ATOM	5387	O	VAL	P	11	0.117	3.091	17.220	0.00	0.00	PROB
ATOM	5388	N	SER	P	12	1.111	4.930	17.977	0.00	0.00	PROB
ATOM	5389	HN	SER	P	12	1.824	5.237	18.603	0.00	0.00	PROB
ATOM	5390	CA	SER	P	12	0.879	5.839	16.844	0.00	0.00	PROB
ATOM	5391	HA	SER	P	12	-0.176	6.068	16.888	0.00	0.00	PROB
ATOM	5392	CB	SER	P	12	1.784	7.138	16.821	0.00	0.00	PROB
ATOM	5393	HB1	SER	P	12	2.848	6.899	16.605	0.00	0.00	PROB
ATOM	5394	HB2	SER	P	12	1.678	7.588	17.831	0.00	0.00	PROB
ATOM	5395	OG	SER	P	12	1.312	8.049	15.821	0.00	0.00	PROB
ATOM	5396	HG1	SER	P	12	0.455	8.339	16.141	0.00	0.00	PROB
ATOM	5397	C	SER	P	12	1.252	5.184	15.555	0.00	0.00	PROB
ATOM	5398	O	SER	P	12	2.305	4.495	15.437	0.00	0.00	PROB
ATOM	5399	N	THR	P	13	0.323	5.309	14.601	0.00	0.00	PROB
ATOM	5400	HN	THR	P	13	-0.515	5.822	14.773	0.00	0.00	PROB
ATOM	5401	CA	THR	P	13	0.430	4.837	13.227	0.00	0.00	PROB
ATOM	5402	HA	THR	P	13	-0.030	5.707	12.782	0.00	0.00	PROB
ATOM	5403	CB	THR	P	13	1.825	4.621	12.547	0.00	0.00	PROB
ATOM	5404	HB	THR	P	13	2.273	3.732	13.039	0.00	0.00	PROB
ATOM	5405	OG1	THR	P	13	2.744	5.691	12.895	0.00	0.00	PROB
ATOM	5406	HG1	THR	P	13	2.942	5.394	13.786	0.00	0.00	PROB
ATOM	5407	CG2	THR	P	13	1.801	4.604	10.998	0.00	0.00	PROB
ATOM	5408	HG21	THR	P	13	1.227	5.488	10.648	0.00	0.00	PROB
ATOM	5409	HG22	THR	P	13	1.301	3.666	10.675	0.00	0.00	PROB
ATOM	5410	HG23	THR	P	13	2.829	4.622	10.577	0.00	0.00	PROB
ATOM	5411	C	THR	P	13	-0.409	3.599	12.898	0.00	0.00	PROB
ATOM	5412	O	THR	P	13	-0.146	2.528	13.419	0.00	0.00	PROB
ATOM	5413	N	PRO	P	14	-1.425	3.662	11.976	0.00	0.00	PROB
ATOM	5414	CD	PRO	P	14	-2.238	4.928	11.844	0.00	0.00	PROB
ATOM	5415	HD1	PRO	P	14	-1.495	5.628	11.405	0.00	0.00	PROB
ATOM	5416	HD2	PRO	P	14	-2.766	5.206	12.782	0.00	0.00	PROB
ATOM	5417	CA	PRO	P	14	-2.093	2.549	11.312	0.00	0.00	PROB
ATOM	5418	HA	PRO	P	14	-2.644	2.025	12.079	0.00	0.00	PROB
ATOM	5419	CB	PRO	P	14	-2.906	3.270	10.255	0.00	0.00	PROB
ATOM	5420	HB1	PRO	P	14	-3.821	2.712	9.962	0.00	0.00	PROB
ATOM	5421	HB2	PRO	P	14	-2.265	3.480	9.372	0.00	0.00	PROB
ATOM	5422	CG	PRO	P	14	-3.317	4.576	10.839	0.00	0.00	PROB
ATOM	5423	HG1	PRO	P	14	-3.480	5.233	9.958	0.00	0.00	PROB
ATOM	5424	HG2	PRO	P	14	-4.292	4.533	11.369	0.00	0.00	PROB

```
ATOM   5425  C   PRO P  14      -1.198   1.464  10.671  0.00  0.00      PROB
ATOM   5426  O   PRO P  14      -0.315   1.677   9.852  0.00  0.00      PROB
ATOM   5427  N   THR P  15      -1.421   0.205  11.025  0.00  0.00      PROB
ATOM   5428  HN  THR P  15      -2.223  -0.005  11.579  0.00  0.00      PROB
ATOM   5429  CA  THR P  15      -0.472  -0.878  10.803  0.00  0.00      PROB
ATOM   5430  HA  THR P  15       0.086  -0.603   9.920  0.00  0.00      PROB
ATOM   5431  CB  THR P  15       0.661  -0.845  11.828  0.00  0.00      PROB
ATOM   5432  HB  THR P  15       0.985   0.215  11.747  0.00  0.00      PROB
ATOM   5433  OG1 THR P  15       1.666  -1.777  11.591  0.00  0.00      PROB
ATOM   5434  HG1 THR P  15       2.499  -1.301  11.565  0.00  0.00      PROB
ATOM   5435  CG2 THR P  15       0.213  -0.991  13.336  0.00  0.00      PROB
ATOM   5436 HG21 THR P  15       1.064  -0.945  14.048  0.00  0.00      PROB
ATOM   5437 HG22 THR P  15      -0.321  -1.965  13.353  0.00  0.00      PROB
ATOM   5438 HG23 THR P  15      -0.541  -0.229  13.629  0.00  0.00      PROB
ATOM   5439  C   THR P  15      -1.113  -2.276  10.719  0.00  0.00      PROB
ATOM   5440  O   THR P  15      -2.059  -2.585  11.459  0.00  0.00      PROB
ATOM   5441  N   LEU P  16      -0.584  -3.179   9.890  0.00  0.00      PROB
ATOM   5442  HN  LEU P  16       0.237  -2.852   9.429  0.00  0.00      PROB
ATOM   5443  CA  LEU P  16      -1.058  -4.545   9.713  0.00  0.00      PROB
ATOM   5444  HA  LEU P  16      -2.087  -4.538  10.041  0.00  0.00      PROB
ATOM   5445  CB  LEU P  16      -1.172  -4.760   8.159  0.00  0.00      PROB
ATOM   5446  HB1 LEU P  16      -1.722  -5.722   8.079  0.00  0.00      PROB
ATOM   5447  HB2 LEU P  16      -0.211  -4.900   7.618  0.00  0.00      PROB
ATOM   5448  CG  LEU P  16      -1.957  -3.661   7.437  0.00  0.00      PROB
ATOM   5449  HG  LEU P  16      -1.358  -2.770   7.725  0.00  0.00      PROB
ATOM   5450  CD1 LEU P  16      -1.843  -3.746   5.956  0.00  0.00      PROB
ATOM   5451 HD11 LEU P  16      -2.429  -2.948   5.454  0.00  0.00      PROB
ATOM   5452 HD12 LEU P  16      -2.343  -4.693   5.659  0.00  0.00      PROB
ATOM   5453 HD13 LEU P  16      -0.771  -3.693   5.669  0.00  0.00      PROB
ATOM   5454  CD2 LEU P  16      -3.466  -3.499   7.815  0.00  0.00      PROB
ATOM   5455 HD21 LEU P  16      -4.158  -4.336   7.583  0.00  0.00      PROB
ATOM   5456 HD22 LEU P  16      -3.943  -2.605   7.359  0.00  0.00      PROB
ATOM   5457 HD23 LEU P  16      -3.533  -3.267   8.900  0.00  0.00      PROB
ATOM   5458  C   LEU P  16      -0.232  -5.596  10.489  0.00  0.00      PROB
ATOM   5459  OT1 LEU P  16       0.898  -6.007  10.020  0.00  0.00      PROB
ATOM   5460  OT2 LEU P  16      -0.639  -6.009  11.612  0.00  0.00      PROB
END
```

4. A representative structure from highly populated cluster of NTER-IN

```
CRYST1   97.683   97.683  169.220  90.00  90.00  90.00 P 1          1
ATOM      1  N   MET P   1       8.029 -31.058  51.818  0.00  0.00      PROA
ATOM      2  HT1 MET P   1       8.746 -30.338  51.593  0.00  0.00      PROA
ATOM      3  HT2 MET P   1       7.981 -31.695  50.998  0.00  0.00      PROA
ATOM      4  HT3 MET P   1       8.477 -31.620  52.570  0.00  0.00      PROA
ATOM      5  CA  MET P   1       6.687 -30.486  52.111  0.00  0.00      PROA
ATOM      6  HA  MET P   1       6.105 -31.354  52.386  0.00  0.00      PROA
ATOM      7  CB  MET P   1       6.607 -29.371  53.213  0.00  0.00      PROA
ATOM      8  HB1 MET P   1       7.315 -29.589  54.041  0.00  0.00      PROA
ATOM      9  HB2 MET P   1       5.544 -29.306  53.531  0.00  0.00      PROA
ATOM     10  CG  MET P   1       6.907 -27.941  52.691  0.00  0.00      PROA
ATOM     11  HG1 MET P   1       6.576 -27.133  53.378  0.00  0.00      PROA
ATOM     12  HG2 MET P   1       6.387 -27.759  51.727  0.00  0.00      PROA
ATOM     13  SD  MET P   1       8.693 -27.767  52.423  0.00  0.00      PROA
ATOM     14  CE  MET P   1       8.623 -26.028  52.036  0.00  0.00      PROA
ATOM     15  HE1 MET P   1       7.819 -25.473  52.564  0.00  0.00      PROA
ATOM     16  HE2 MET P   1       8.390 -25.856  50.963  0.00  0.00      PROA
ATOM     17  HE3 MET P   1       9.554 -25.514  52.357  0.00  0.00      PROA
ATOM     18  C   MET P   1       5.984 -30.009  50.802  0.00  0.00      PROA
ATOM     19  O   MET P   1       6.662 -29.781  49.778  0.00  0.00      PROA
ATOM     20  N   GLU P   2       4.672 -29.891  50.868  0.00  0.00      PROA
ATOM     21  HN  GLU P   2       4.205 -30.151  51.709  0.00  0.00      PROA
ATOM     22  CA  GLU P   2       3.802 -29.374  49.831  0.00  0.00      PROA
ATOM     23  HA  GLU P   2       3.966 -29.996  48.964  0.00  0.00      PROA
ATOM     24  CB  GLU P   2       2.368 -29.587  50.239  0.00  0.00      PROA
ATOM     25  HB1 GLU P   2       1.777 -29.317  49.337  0.00  0.00      PROA
ATOM     26  HB2 GLU P   2       2.079 -28.949  51.101  0.00  0.00      PROA
ATOM     27  CG  GLU P   2       1.882 -31.040  50.598  0.00  0.00      PROA
ATOM     28  HG1 GLU P   2       2.332 -31.741  49.863  0.00  0.00      PROA
ATOM     29  HG2 GLU P   2       0.796 -31.063  50.367  0.00  0.00      PROA
ATOM     30  CD  GLU P   2       2.184 -31.513  51.983  0.00  0.00      PROA
ATOM     31  OE1 GLU P   2       1.676 -32.566  52.304  0.00  0.00      PROA
ATOM     32  OE2 GLU P   2       2.777 -30.742  52.776  0.00  0.00      PROA
ATOM     33  C   GLU P   2       4.094 -27.912  49.419  0.00  0.00      PROA
ATOM     34  O   GLU P   2       4.194 -26.960  50.209  0.00  0.00      PROA
ATOM     35  N   GLY P   3       4.266 -27.715  48.136  0.00  0.00      PROA
ATOM     36  HN  GLY P   3       4.245 -28.424  47.435  0.00  0.00      PROA
ATOM     37  CA  GLY P   3       4.390 -26.397  47.476  0.00  0.00      PROA
ATOM     38  HA1 GLY P   3       4.390 -26.713  46.444  0.00  0.00      PROA
ATOM     39  HA2 GLY P   3       5.374 -26.019  47.715  0.00  0.00      PROA
ATOM     40  C   GLY P   3       3.317 -25.398  47.838  0.00  0.00      PROA
ATOM     41  O   GLY P   3       2.185 -25.690  48.172  0.00  0.00      PROA
ATOM     42  N   ILE P   4       3.631 -24.044  47.743  0.00  0.00      PROA
ATOM     43  HN  ILE P   4       4.554 -23.800  47.457  0.00  0.00      PROA
ATOM     44  CA  ILE P   4       2.680 -22.998  47.891  0.00  0.00      PROA
ATOM     45  HA  ILE P   4       1.695 -23.260  48.248  0.00  0.00      PROA
ATOM     46  CB  ILE P   4       3.171 -21.990  48.972  0.00  0.00      PROA
ATOM     47  HB  ILE P   4       3.147 -22.497  49.961  0.00  0.00      PROA
ATOM     48  CG2 ILE P   4       4.641 -21.490  48.698  0.00  0.00      PROA
ATOM     49 HG21 ILE P   4       5.346 -22.308  48.960  0.00  0.00      PROA
ATOM     50 HG22 ILE P   4       4.782 -20.567  49.300  0.00  0.00      PROA
ATOM     51 HG23 ILE P   4       4.808 -21.185  47.643  0.00  0.00      PROA
ATOM     52  CG1 ILE P   4       2.289 -20.774  49.117  0.00  0.00      PROA
ATOM     53 HG11 ILE P   4       2.448 -20.130  48.226  0.00  0.00      PROA
ATOM     54 HG12 ILE P   4       2.750 -20.233  49.972  0.00  0.00      PROA
ATOM     55  CD  ILE P   4       0.816 -21.056  49.531  0.00  0.00      PROA
ATOM     56  HD1 ILE P   4       0.267 -20.116  49.750  0.00  0.00      PROA
ATOM     57  HD2 ILE P   4       0.799 -21.702  50.435  0.00  0.00      PROA
```

```
ATOM     58  HD3 ILE P   4       0.276 -21.732  48.835  0.00  0.00      PROA
ATOM     59  C   ILE P   4       2.408 -22.265  46.552  0.00  0.00      PROA
ATOM     60  O   ILE P   4       3.253 -21.995  45.704  0.00  0.00      PROA
ATOM     61  N   SER P   5       1.085 -21.971  46.298  0.00  0.00      PROA
ATOM     62  HN  SER P   5       0.446 -22.181  47.034  0.00  0.00      PROA
ATOM     63  CA  SER P   5       0.586 -21.289  45.031  0.00  0.00      PROA
ATOM     64  HA  SER P   5      -0.398 -20.957  45.329  0.00  0.00      PROA
ATOM     65  CB  SER P   5       1.346 -20.131  44.385  0.00  0.00      PROA
ATOM     66  HB1 SER P   5       2.316 -20.516  44.005  0.00  0.00      PROA
ATOM     67  HB2 SER P   5       1.515 -19.403  45.207  0.00  0.00      PROA
ATOM     68  OG  SER P   5       0.673 -19.468  43.275  0.00  0.00      PROA
ATOM     69  HG1 SER P   5      -0.067 -18.946  43.595  0.00  0.00      PROA
ATOM     70  C   SER P   5       0.288 -22.446  44.082  0.00  0.00      PROA
ATOM     71  O   SER P   5       0.677 -22.581  42.964  0.00  0.00      PROA
ATOM     72  N   ILE P   6      -0.596 -23.400  44.492  0.00  0.00      PROA
ATOM     73  HN  ILE P   6      -1.143 -23.388  45.326  0.00  0.00      PROA
ATOM     74  CA  ILE P   6      -0.881 -24.598  43.724  0.00  0.00      PROA
ATOM     75  HA  ILE P   6      -0.305 -24.680  42.815  0.00  0.00      PROA
ATOM     76  CB  ILE P   6      -0.964 -25.925  44.629  0.00  0.00      PROA
ATOM     77  HB  ILE P   6      -1.813 -25.852  45.342  0.00  0.00      PROA
ATOM     78  CG2 ILE P   6      -1.391 -27.045  43.621  0.00  0.00      PROA
ATOM     79 HG21 ILE P   6      -2.411 -26.895  43.207  0.00  0.00      PROA
ATOM     80 HG22 ILE P   6      -1.446 -27.937  44.281  0.00  0.00      PROA
ATOM     81 HG23 ILE P   6      -0.625 -27.294  42.855  0.00  0.00      PROA
ATOM     82  CG1 ILE P   6       0.397 -26.192  45.206  0.00  0.00      PROA
ATOM     83 HG11 ILE P   6       0.367 -27.240  45.573  0.00  0.00      PROA
ATOM     84 HG12 ILE P   6       0.601 -25.534  46.078  0.00  0.00      PROA
ATOM     85  CD  ILE P   6       1.576 -26.046  44.241  0.00  0.00      PROA
ATOM     86  HD1 ILE P   6       1.670 -24.973  43.969  0.00  0.00      PROA
ATOM     87  HD2 ILE P   6       1.408 -26.612  43.300  0.00  0.00      PROA
ATOM     88  HD3 ILE P   6       2.552 -26.341  44.682  0.00  0.00      PROA
ATOM     89  C   ILE P   6      -2.239 -24.353  43.091  0.00  0.00      PROA
ATOM     90  O   ILE P   6      -3.197 -23.938  43.783  0.00  0.00      PROA
ATOM     91  N   TYR P   7      -2.322 -24.577  41.816  0.00  0.00      PROA
ATOM     92  HN  TYR P   7      -1.519 -24.901  41.321  0.00  0.00      PROA
ATOM     93  CA  TYR P   7      -3.584 -24.653  41.049  0.00  0.00      PROA
ATOM     94  HA  TYR P   7      -4.426 -24.944  41.660  0.00  0.00      PROA
ATOM     95  CB  TYR P   7      -3.972 -23.320  40.314  0.00  0.00      PROA
ATOM     96  HB1 TYR P   7      -3.178 -22.989  39.611  0.00  0.00      PROA
ATOM     97  HB2 TYR P   7      -4.047 -22.576  41.135  0.00  0.00      PROA
ATOM     98  CG  TYR P   7      -5.343 -23.319  39.592  0.00  0.00      PROA
ATOM     99  CD1 TYR P   7      -5.527 -22.993  38.293  0.00  0.00      PROA
ATOM    100  HD1 TYR P   7      -4.693 -22.832  37.625  0.00  0.00      PROA
ATOM    101  CE1 TYR P   7      -6.844 -22.824  37.772  0.00  0.00      PROA
ATOM    102  HE1 TYR P   7      -7.101 -22.651  36.738  0.00  0.00      PROA
ATOM    103  CZ  TYR P   7      -7.982 -23.059  38.598  0.00  0.00      PROA
ATOM    104  OH  TYR P   7      -9.300 -22.840  38.117  0.00  0.00      PROA
ATOM    105  HH  TYR P   7      -9.209 -22.945  37.167  0.00  0.00      PROA
ATOM    106  CD2 TYR P   7      -6.507 -23.613  40.374  0.00  0.00      PROA
ATOM    107  HD2 TYR P   7      -6.260 -23.938  41.374  0.00  0.00      PROA
ATOM    108  CE2 TYR P   7      -7.796 -23.407  39.885  0.00  0.00      PROA
ATOM    109  HE2 TYR P   7      -8.698 -23.600  40.446  0.00  0.00      PROA
ATOM    110  C   TYR P   7      -3.281 -25.712  40.001  0.00  0.00      PROA
ATOM    111  O   TYR P   7      -2.100 -25.952  39.643  0.00  0.00      PROA
ATOM    112  N   THR P   8      -4.308 -26.391  39.523  0.00  0.00      PROA
ATOM    113  HN  THR P   8      -5.216 -26.310  39.927  0.00  0.00      PROA
ATOM    114  CA  THR P   8      -4.268 -27.358  38.486  0.00  0.00      PROA
ATOM    115  HA  THR P   8      -3.756 -28.249  38.818  0.00  0.00      PROA
ATOM    116  CB  THR P   8      -5.672 -27.938  38.093  0.00  0.00      PROA
ATOM    117  HB  THR P   8      -6.104 -28.277  39.059  0.00  0.00      PROA
ATOM    118  OG1 THR P   8      -5.596 -29.038  37.189  0.00  0.00      PROA
```

```
ATOM    119  HG1 THR P   8      -5.128 -29.825  37.478  0.00  0.00      PROA
ATOM    120  CG2 THR P   8      -6.531 -26.840  37.482  0.00  0.00      PROA
ATOM    121 HG21 THR P   8      -6.007 -26.519  36.557  0.00  0.00      PROA
ATOM    122 HG22 THR P   8      -6.514 -26.000  38.209  0.00  0.00      PROA
ATOM    123 HG23 THR P   8      -7.561 -27.175  37.233  0.00  0.00      PROA
ATOM    124  C   THR P   8      -3.479 -26.931  37.192  0.00  0.00      PROA
ATOM    125  O   THR P   8      -3.495 -25.809  36.758  0.00  0.00      PROA
ATOM    126  N   SER P   9      -2.648 -27.891  36.711  0.00  0.00      PROA
ATOM    127  HN  SER P   9      -2.600 -28.795  37.128  0.00  0.00      PROA
ATOM    128  CA  SER P   9      -1.688 -27.650  35.595  0.00  0.00      PROA
ATOM    129  HA  SER P   9      -2.182 -27.130  34.788  0.00  0.00      PROA
ATOM    130  CB  SER P   9      -0.428 -26.874  36.032  0.00  0.00      PROA
ATOM    131  HB1 SER P   9       0.382 -27.447  36.532  0.00  0.00      PROA
ATOM    132  HB2 SER P   9      -0.869 -26.133  36.732  0.00  0.00      PROA
ATOM    133  OG  SER P   9       0.036 -26.052  34.961  0.00  0.00      PROA
ATOM    134  HG1 SER P   9       0.727 -26.527  34.493  0.00  0.00      PROA
ATOM    135  C   SER P   9      -1.225 -29.009  35.134  0.00  0.00      PROA
ATOM    136  O   SER P   9      -1.178 -29.982  35.824  0.00  0.00      PROA
ATOM    137  N   ASP P  10      -0.859 -29.156  33.836  0.00  0.00      PROA
ATOM    138  HN  ASP P  10      -0.968 -28.405  33.189  0.00  0.00      PROA
ATOM    139  CA  ASP P  10      -0.425 -30.474  33.328  0.00  0.00      PROA
ATOM    140  HA  ASP P  10      -1.011 -31.249  33.799  0.00  0.00      PROA
ATOM    141  CB  ASP P  10      -0.535 -30.404  31.799  0.00  0.00      PROA
ATOM    142  HB1 ASP P  10      -0.037 -31.257  31.290  0.00  0.00      PROA
ATOM    143  HB2 ASP P  10      -0.058 -29.469  31.436  0.00  0.00      PROA
ATOM    144  CG  ASP P  10      -1.973 -30.330  31.300  0.00  0.00      PROA
ATOM    145  OD1 ASP P  10      -2.915 -30.860  32.018  0.00  0.00      PROA
ATOM    146  OD2 ASP P  10      -2.179 -29.921  30.137  0.00  0.00      PROA
ATOM    147  C   ASP P  10       0.973 -30.787  33.760  0.00  0.00      PROA
ATOM    148  O   ASP P  10       1.244 -31.918  34.190  0.00  0.00      PROA
ATOM    149  N   ASN P  11       1.949 -29.848  33.725  0.00  0.00      PROA
ATOM    150  HN  ASN P  11       1.721 -28.885  33.604  0.00  0.00      PROA
ATOM    151  CA  ASN P  11       3.283 -30.166  34.178  0.00  0.00      PROA
ATOM    152  HA  ASN P  11       3.191 -31.018  34.836  0.00  0.00      PROA
ATOM    153  CB  ASN P  11       4.260 -30.589  32.963  0.00  0.00      PROA
ATOM    154  HB1 ASN P  11       4.423 -29.733  32.274  0.00  0.00      PROA
ATOM    155  HB2 ASN P  11       3.724 -31.350  32.357  0.00  0.00      PROA
ATOM    156  CG  ASN P  11       5.583 -31.048  33.445  0.00  0.00      PROA
ATOM    157  OD1 ASN P  11       6.628 -30.394  33.160  0.00  0.00      PROA
ATOM    158  ND2 ASN P  11       5.617 -32.192  34.154  0.00  0.00      PROA
ATOM    159 HD21 ASN P  11       6.512 -32.372  34.562  0.00  0.00      PROA
ATOM    160 HD22 ASN P  11       4.779 -32.459  34.630  0.00  0.00      PROA
ATOM    161  C   ASN P  11       3.971 -29.120  35.044  0.00  0.00      PROA
ATOM    162  O   ASN P  11       4.373 -28.100  34.520  0.00  0.00      PROA
ATOM    163  N   TYR P  12       4.177 -29.432  36.332  0.00  0.00      PROA
ATOM    164  HN  TYR P  12       3.969 -30.281  36.811  0.00  0.00      PROA
ATOM    165  CA  TYR P  12       4.811 -28.426  37.198  0.00  0.00      PROA
ATOM    166  HA  TYR P  12       4.369 -27.470  36.960  0.00  0.00      PROA
ATOM    167  CB  TYR P  12       4.672 -28.897  38.659  0.00  0.00      PROA
ATOM    168  HB1 TYR P  12       5.190 -28.323  39.456  0.00  0.00      PROA
ATOM    169  HB2 TYR P  12       5.001 -29.947  38.809  0.00  0.00      PROA
ATOM    170  CG  TYR P  12       3.192 -28.687  38.939  0.00  0.00      PROA
ATOM    171  CD1 TYR P  12       2.534 -27.436  38.992  0.00  0.00      PROA
ATOM    172  HD1 TYR P  12       3.149 -26.551  38.925  0.00  0.00      PROA
ATOM    173  CE1 TYR P  12       1.203 -27.320  39.251  0.00  0.00      PROA
ATOM    174  HE1 TYR P  12       0.770 -26.333  39.191  0.00  0.00      PROA
ATOM    175  CZ  TYR P  12       0.360 -28.450  39.329  0.00  0.00      PROA
ATOM    176  OH  TYR P  12      -1.017 -28.334  39.647  0.00  0.00      PROA
ATOM    177  HH  TYR P  12      -1.239 -27.414  39.810  0.00  0.00      PROA
ATOM    178  CD2 TYR P  12       2.347 -29.866  38.934  0.00  0.00      PROA
ATOM    179  HD2 TYR P  12       2.766 -30.818  38.644  0.00  0.00      PROA
```

ATOM	180	CE2	TYR	P	12	0.946	-29.720	39.170	0.00	0.00	PROA
ATOM	181	HE2	TYR	P	12	0.320	-30.600	39.139	0.00	0.00	PROA
ATOM	182	C	TYR	P	12	6.301	-28.314	36.845	0.00	0.00	PROA
ATOM	183	O	TYR	P	12	6.923	-29.313	36.628	0.00	0.00	PROA
ATOM	184	N	THR	P	13	6.829	-27.094	36.796	0.00	0.00	PROA
ATOM	185	HN	THR	P	13	6.275	-26.369	37.198	0.00	0.00	PROA
ATOM	186	CA	THR	P	13	8.177	-26.844	36.367	0.00	0.00	PROA
ATOM	187	HA	THR	P	13	8.224	-27.303	35.390	0.00	0.00	PROA
ATOM	188	CB	THR	P	13	8.373	-25.314	36.127	0.00	0.00	PROA
ATOM	189	HB	THR	P	13	7.556	-24.996	35.445	0.00	0.00	PROA
ATOM	190	OG1	THR	P	13	9.548	-24.985	35.444	0.00	0.00	PROA
ATOM	191	HG1	THR	P	13	9.452	-25.358	34.565	0.00	0.00	PROA
ATOM	192	CG2	THR	P	13	8.334	-24.411	37.418	0.00	0.00	PROA
ATOM	193	HG21	THR	P	13	9.306	-24.546	37.941	0.00	0.00	PROA
ATOM	194	HG22	THR	P	13	7.514	-24.851	38.024	0.00	0.00	PROA
ATOM	195	HG23	THR	P	13	8.124	-23.340	37.210	0.00	0.00	PROA
ATOM	196	C	THR	P	13	9.355	-27.496	37.048	0.00	0.00	PROA
ATOM	197	O	THR	P	13	9.483	-27.619	38.265	0.00	0.00	PROA
ATOM	198	N	GLU	P	14	10.377	-27.993	36.283	0.00	0.00	PROA
ATOM	199	HN	GLU	P	14	10.283	-28.008	35.290	0.00	0.00	PROA
ATOM	200	CA	GLU	P	14	11.685	-28.513	36.768	0.00	0.00	PROA
ATOM	201	HA	GLU	P	14	11.507	-29.003	37.714	0.00	0.00	PROA
ATOM	202	CB	GLU	P	14	12.012	-29.622	35.780	0.00	0.00	PROA
ATOM	203	HB1	GLU	P	14	12.992	-30.092	36.011	0.00	0.00	PROA
ATOM	204	HB2	GLU	P	14	12.152	-29.211	34.758	0.00	0.00	PROA
ATOM	205	CG	GLU	P	14	10.986	-30.723	35.573	0.00	0.00	PROA
ATOM	206	HG1	GLU	P	14	9.961	-30.305	35.665	0.00	0.00	PROA
ATOM	207	HG2	GLU	P	14	11.049	-31.439	36.421	0.00	0.00	PROA
ATOM	208	CD	GLU	P	14	11.210	-31.440	34.204	0.00	0.00	PROA
ATOM	209	OE1	GLU	P	14	12.206	-32.141	33.973	0.00	0.00	PROA
ATOM	210	OE2	GLU	P	14	10.343	-31.282	33.346	0.00	0.00	PROA
ATOM	211	C	GLU	P	14	12.706	-27.360	36.798	0.00	0.00	PROA
ATOM	212	O	GLU	P	14	12.595	-26.345	36.094	0.00	0.00	PROA
ATOM	213	N	GLU	P	15	13.718	-27.460	37.664	0.00	0.00	PROA
ATOM	214	HN	GLU	P	15	13.829	-28.315	38.165	0.00	0.00	PROA
ATOM	215	CA	GLU	P	15	14.842	-26.571	37.786	0.00	0.00	PROA
ATOM	216	HA	GLU	P	15	14.463	-25.561	37.812	0.00	0.00	PROA
ATOM	217	CB	GLU	P	15	15.546	-26.896	39.162	0.00	0.00	PROA
ATOM	218	HB1	GLU	P	15	15.279	-27.922	39.496	0.00	0.00	PROA
ATOM	219	HB2	GLU	P	15	14.977	-26.238	39.852	0.00	0.00	PROA
ATOM	220	CG	GLU	P	15	17.069	-26.776	39.260	0.00	0.00	PROA
ATOM	221	HG1	GLU	P	15	17.510	-25.811	38.931	0.00	0.00	PROA
ATOM	222	HG2	GLU	P	15	17.602	-27.601	38.741	0.00	0.00	PROA
ATOM	223	CD	GLU	P	15	17.462	-26.975	40.661	0.00	0.00	PROA
ATOM	224	OE1	GLU	P	15	17.387	-28.045	41.311	0.00	0.00	PROA
ATOM	225	OE2	GLU	P	15	17.903	-25.936	41.265	0.00	0.00	PROA
ATOM	226	C	GLU	P	15	15.741	-26.526	36.549	0.00	0.00	PROA
ATOM	227	O	GLU	P	15	15.893	-27.593	35.953	0.00	0.00	PROA
ATOM	228	N	MET	P	16	16.401	-25.376	36.168	0.00	0.00	PROA
ATOM	229	HN	MET	P	16	16.442	-24.567	36.750	0.00	0.00	PROA
ATOM	230	CA	MET	P	16	17.285	-25.518	35.025	0.00	0.00	PROA
ATOM	231	HA	MET	P	16	16.650	-25.922	34.250	0.00	0.00	PROA
ATOM	232	CB	MET	P	16	17.822	-24.168	34.445	0.00	0.00	PROA
ATOM	233	HB1	MET	P	16	18.524	-24.408	33.617	0.00	0.00	PROA
ATOM	234	HB2	MET	P	16	18.372	-23.594	35.221	0.00	0.00	PROA
ATOM	235	CG	MET	P	16	16.831	-23.259	33.744	0.00	0.00	PROA
ATOM	236	HG1	MET	P	16	16.235	-23.925	33.083	0.00	0.00	PROA
ATOM	237	HG2	MET	P	16	17.301	-22.593	32.989	0.00	0.00	PROA
ATOM	238	SD	MET	P	16	15.673	-22.389	34.845	0.00	0.00	PROA
ATOM	239	CE	MET	P	16	14.632	-21.762	33.437	0.00	0.00	PROA
ATOM	240	HE1	MET	P	16	14.558	-22.551	32.657	0.00	0.00	PROA

```
ATOM    241  HE2 MET P  16      15.067 -20.851  32.973  0.00  0.00      PROA
ATOM    242  HE3 MET P  16      13.640 -21.480  33.849  0.00  0.00      PROA
ATOM    243  C   MET P  16      18.467 -26.538  35.084  0.00  0.00      PROA
ATOM    244  O   MET P  16      19.292 -26.614  35.978  0.00  0.00      PROA
ATOM    245  N   GLY P  17      18.650 -27.382  34.066  0.00  0.00      PROA
ATOM    246  HN  GLY P  17      17.963 -27.388  33.344  0.00  0.00      PROA
ATOM    247  CA  GLY P  17      19.935 -28.040  33.808  0.00  0.00      PROA
ATOM    248  HA1 GLY P  17      19.679 -28.738  33.025  0.00  0.00      PROA
ATOM    249  HA2 GLY P  17      20.350 -28.440  34.721  0.00  0.00      PROA
ATOM    250  C   GLY P  17      21.079 -27.114  33.370  0.00  0.00      PROA
ATOM    251  O   GLY P  17      21.070 -25.887  33.650  0.00  0.00      PROA
ATOM    252  N   SER P  18      22.020 -27.707  32.553  0.00  0.00      PROA
ATOM    253  HN  SER P  18      21.978 -28.650  32.230  0.00  0.00      PROA
ATOM    254  CA  SER P  18      23.173 -26.979  31.939  0.00  0.00      PROA
ATOM    255  HA  SER P  18      23.272 -25.955  32.265  0.00  0.00      PROA
ATOM    256  CB  SER P  18      24.522 -27.790  32.066  0.00  0.00      PROA
ATOM    257  HB1 SER P  18      25.445 -27.175  32.011  0.00  0.00      PROA
ATOM    258  HB2 SER P  18      24.706 -28.621  31.352  0.00  0.00      PROA
ATOM    259  OG  SER P  18      24.569 -28.442  33.329  0.00  0.00      PROA
ATOM    260  HG1 SER P  18      25.397 -28.252  33.777  0.00  0.00      PROA
ATOM    261  C   SER P  18      22.872 -26.908  30.454  0.00  0.00      PROA
ATOM    262  O   SER P  18      23.816 -26.947  29.703  0.00  0.00      PROA
ATOM    263  N   GLY P  19      21.621 -26.884  30.042  0.00  0.00      PROA
ATOM    264  HN  GLY P  19      20.882 -27.002  30.701  0.00  0.00      PROA
ATOM    265  CA  GLY P  19      21.274 -27.091  28.612  0.00  0.00      PROA
ATOM    266  HA1 GLY P  19      20.426 -27.761  28.604  0.00  0.00      PROA
ATOM    267  HA2 GLY P  19      22.084 -27.516  28.038  0.00  0.00      PROA
ATOM    268  C   GLY P  19      20.872 -25.803  27.887  0.00  0.00      PROA
ATOM    269  O   GLY P  19      21.141 -24.675  28.298  0.00  0.00      PROA
ATOM    270  N   ASP P  20      19.980 -25.961  26.907  0.00  0.00      PROA
ATOM    271  HN  ASP P  20      19.646 -26.818  26.522  0.00  0.00      PROA
ATOM    272  CA  ASP P  20      19.540 -24.775  26.183  0.00  0.00      PROA
ATOM    273  HA  ASP P  20      20.192 -23.919  26.273  0.00  0.00      PROA
ATOM    274  CB  ASP P  20      19.359 -25.165  24.680  0.00  0.00      PROA
ATOM    275  HB1 ASP P  20      18.588 -25.965  24.642  0.00  0.00      PROA
ATOM    276  HB2 ASP P  20      20.291 -25.664  24.339  0.00  0.00      PROA
ATOM    277  CG  ASP P  20      19.230 -24.022  23.783  0.00  0.00      PROA
ATOM    278  OD1 ASP P  20      19.721 -22.874  24.101  0.00  0.00      PROA
ATOM    279  OD2 ASP P  20      18.927 -24.252  22.629  0.00  0.00      PROA
ATOM    280  C   ASP P  20      18.203 -24.371  26.880  0.00  0.00      PROA
ATOM    281  O   ASP P  20      17.260 -25.119  27.005  0.00  0.00      PROA
ATOM    282  N   TYR P  21      18.144 -23.093  27.340  0.00  0.00      PROA
ATOM    283  HN  TYR P  21      18.869 -22.509  26.983  0.00  0.00      PROA
ATOM    284  CA  TYR P  21      17.051 -22.499  28.105  0.00  0.00      PROA
ATOM    285  HA  TYR P  21      16.134 -23.067  28.064  0.00  0.00      PROA
ATOM    286  CB  TYR P  21      17.271 -22.475  29.627  0.00  0.00      PROA
ATOM    287  HB1 TYR P  21      16.392 -21.962  30.072  0.00  0.00      PROA
ATOM    288  HB2 TYR P  21      18.245 -22.004  29.882  0.00  0.00      PROA
ATOM    289  CG  TYR P  21      17.212 -23.857  30.179  0.00  0.00      PROA
ATOM    290  CD1 TYR P  21      15.991 -24.523  30.284  0.00  0.00      PROA
ATOM    291  HD1 TYR P  21      15.108 -24.082  29.845  0.00  0.00      PROA
ATOM    292  CE1 TYR P  21      15.975 -25.883  30.769  0.00  0.00      PROA
ATOM    293  HE1 TYR P  21      15.013 -26.374  30.742  0.00  0.00      PROA
ATOM    294  CZ  TYR P  21      17.146 -26.579  31.141  0.00  0.00      PROA
ATOM    295  OH  TYR P  21      17.212 -27.880  31.655  0.00  0.00      PROA
ATOM    296  HH  TYR P  21      16.357 -28.233  31.912  0.00  0.00      PROA
ATOM    297  CD2 TYR P  21      18.380 -24.579  30.528  0.00  0.00      PROA
ATOM    298  HD2 TYR P  21      19.344 -24.100  30.443  0.00  0.00      PROA
ATOM    299  CE2 TYR P  21      18.313 -25.817  31.027  0.00  0.00      PROA
ATOM    300  HE2 TYR P  21      19.205 -26.370  31.278  0.00  0.00      PROA
ATOM    301  C   TYR P  21      16.729 -21.093  27.613  0.00  0.00      PROA
```

```
ATOM    302  O   TYR P  21      17.568 -20.412  26.974  0.00  0.00      PROA
ATOM    303  N   ASP P  22      15.551 -20.605  27.857  0.00  0.00      PROA
ATOM    304  HN  ASP P  22      15.069 -20.986  28.643  0.00  0.00      PROA
ATOM    305  CA  ASP P  22      14.951 -19.448  27.125  0.00  0.00      PROA
ATOM    306  HA  ASP P  22      15.249 -19.569  26.095  0.00  0.00      PROA
ATOM    307  CB  ASP P  22      13.401 -19.415  27.136  0.00  0.00      PROA
ATOM    308  HB1 ASP P  22      13.002 -18.461  26.731  0.00  0.00      PROA
ATOM    309  HB2 ASP P  22      13.020 -19.478  28.178  0.00  0.00      PROA
ATOM    310  CG  ASP P  22      12.809 -20.531  26.298  0.00  0.00      PROA
ATOM    311  OD1 ASP P  22      13.553 -21.467  25.946  0.00  0.00      PROA
ATOM    312  OD2 ASP P  22      11.525 -20.519  26.214  0.00  0.00      PROA
ATOM    313  C   ASP P  22      15.436 -18.077  27.662  0.00  0.00      PROA
ATOM    314  O   ASP P  22      15.574 -17.026  27.050  0.00  0.00      PROA
ATOM    315  N   SER P  23      15.935 -18.137  28.925  0.00  0.00      PROA
ATOM    316  HN  SER P  23      15.866 -18.940  29.511  0.00  0.00      PROA
ATOM    317  CA  SER P  23      16.409 -17.062  29.767  0.00  0.00      PROA
ATOM    318  HA  SER P  23      16.048 -16.112  29.403  0.00  0.00      PROA
ATOM    319  CB  SER P  23      15.797 -17.115  31.149  0.00  0.00      PROA
ATOM    320  HB1 SER P  23      14.688 -17.116  31.083  0.00  0.00      PROA
ATOM    321  HB2 SER P  23      16.311 -16.298  31.700  0.00  0.00      PROA
ATOM    322  OG  SER P  23      16.225 -18.296  31.785  0.00  0.00      PROA
ATOM    323  HG1 SER P  23      16.298 -18.058  32.713  0.00  0.00      PROA
ATOM    324  C   SER P  23      17.918 -16.976  29.737  0.00  0.00      PROA
ATOM    325  O   SER P  23      18.534 -16.084  30.306  0.00  0.00      PROA
ATOM    326  N   MET P  24      18.547 -17.981  29.108  0.00  0.00      PROA
ATOM    327  HN  MET P  24      17.936 -18.661  28.711  0.00  0.00      PROA
ATOM    328  CA  MET P  24      19.990 -18.154  29.175  0.00  0.00      PROA
ATOM    329  HA  MET P  24      20.402 -17.453  29.886  0.00  0.00      PROA
ATOM    330  CB  MET P  24      20.216 -19.620  29.585  0.00  0.00      PROA
ATOM    331  HB1 MET P  24      21.304 -19.830  29.505  0.00  0.00      PROA
ATOM    332  HB2 MET P  24      19.720 -20.353  28.913  0.00  0.00      PROA
ATOM    333  CG  MET P  24      19.861 -19.968  31.076  0.00  0.00      PROA
ATOM    334  HG1 MET P  24      18.779 -19.880  31.310  0.00  0.00      PROA
ATOM    335  HG2 MET P  24      20.393 -19.219  31.700  0.00  0.00      PROA
ATOM    336  SD  MET P  24      20.386 -21.600  31.605  0.00  0.00      PROA
ATOM    337  CE  MET P  24      19.833 -21.443  33.341  0.00  0.00      PROA
ATOM    338  HE1 MET P  24      20.084 -22.287  34.019  0.00  0.00      PROA
ATOM    339  HE2 MET P  24      18.740 -21.333  33.506  0.00  0.00      PROA
ATOM    340  HE3 MET P  24      20.333 -20.602  33.868  0.00  0.00      PROA
ATOM    341  C   MET P  24      20.825 -17.829  27.928  0.00  0.00      PROA
ATOM    342  O   MET P  24      22.070 -17.879  27.950  0.00  0.00      PROA
ATOM    343  N   LYS P  25      20.056 -17.644  26.817  0.00  0.00      PROA
ATOM    344  HN  LYS P  25      19.062 -17.628  26.749  0.00  0.00      PROA
ATOM    345  CA  LYS P  25      20.542 -17.486  25.451  0.00  0.00      PROA
ATOM    346  HA  LYS P  25      20.752 -18.485  25.099  0.00  0.00      PROA
ATOM    347  CB  LYS P  25      19.408 -17.003  24.423  0.00  0.00      PROA
ATOM    348  HB1 LYS P  25      19.696 -16.842  23.362  0.00  0.00      PROA
ATOM    349  HB2 LYS P  25      19.015 -16.072  24.884  0.00  0.00      PROA
ATOM    350  CG  LYS P  25      18.246 -18.040  24.266  0.00  0.00      PROA
ATOM    351  HG1 LYS P  25      17.377 -17.624  23.713  0.00  0.00      PROA
ATOM    352  HG2 LYS P  25      17.889 -18.301  25.286  0.00  0.00      PROA
ATOM    353  CD  LYS P  25      18.647 -19.410  23.788  0.00  0.00      PROA
ATOM    354  HD1 LYS P  25      19.235 -19.846  24.624  0.00  0.00      PROA
ATOM    355  HD2 LYS P  25      19.274 -19.299  22.877  0.00  0.00      PROA
ATOM    356  CE  LYS P  25      17.387 -20.142  23.402  0.00  0.00      PROA
ATOM    357  HE1 LYS P  25      16.917 -19.612  22.546  0.00  0.00      PROA
ATOM    358  HE2 LYS P  25      16.729 -20.303  24.283  0.00  0.00      PROA
ATOM    359  NZ  LYS P  25      17.735 -21.450  22.792  0.00  0.00      PROA
ATOM    360  HZ1 LYS P  25      16.881 -22.041  22.851  0.00  0.00      PROA
ATOM    361  HZ2 LYS P  25      18.556 -21.931  23.212  0.00  0.00      PROA
ATOM    362  HZ3 LYS P  25      18.015 -21.273  21.806  0.00  0.00      PROA
```

ATOM	363	C	LYS	P	25	21.859	-16.663	25.180	0.00	0.00	PROA
ATOM	364	O	LYS	P	25	22.039	-15.561	25.657	0.00	0.00	PROA
ATOM	365	N	GLU	P	26	22.844	-17.274	24.510	0.00	0.00	PROA
ATOM	366	HN	GLU	P	26	22.813	-18.153	24.040	0.00	0.00	PROA
ATOM	367	CA	GLU	P	26	24.127	-16.683	24.271	0.00	0.00	PROA
ATOM	368	HA	GLU	P	26	24.421	-16.434	25.279	0.00	0.00	PROA
ATOM	369	CB	GLU	P	26	25.030	-17.803	23.598	0.00	0.00	PROA
ATOM	370	HB1	GLU	P	26	24.991	-18.664	24.300	0.00	0.00	PROA
ATOM	371	HB2	GLU	P	26	26.113	-17.558	23.547	0.00	0.00	PROA
ATOM	372	CG	GLU	P	26	24.674	-17.997	22.064	0.00	0.00	PROA
ATOM	373	HG1	GLU	P	26	24.789	-17.005	21.577	0.00	0.00	PROA
ATOM	374	HG2	GLU	P	26	23.608	-18.286	21.941	0.00	0.00	PROA
ATOM	375	CD	GLU	P	26	25.364	-19.100	21.320	0.00	0.00	PROA
ATOM	376	OE1	GLU	P	26	24.630	-20.151	21.121	0.00	0.00	PROA
ATOM	377	OE2	GLU	P	26	26.569	-19.092	21.096	0.00	0.00	PROA
ATOM	378	C	GLU	P	26	23.967	-15.371	23.494	0.00	0.00	PROA
ATOM	379	O	GLU	P	26	23.194	-15.375	22.498	0.00	0.00	PROA
ATOM	380	N	PRO	P	27	24.622	-14.200	23.842	0.00	0.00	PROA
ATOM	381	CD	PRO	P	27	25.538	-14.153	24.954	0.00	0.00	PROA
ATOM	382	HD1	PRO	P	27	26.225	-15.016	24.819	0.00	0.00	PROA
ATOM	383	HD2	PRO	P	27	25.154	-14.355	25.977	0.00	0.00	PROA
ATOM	384	CA	PRO	P	27	24.630	-12.916	23.078	0.00	0.00	PROA
ATOM	385	HA	PRO	P	27	23.670	-12.425	23.143	0.00	0.00	PROA
ATOM	386	CB	PRO	P	27	25.394	-11.949	24.096	0.00	0.00	PROA
ATOM	387	HB1	PRO	P	27	24.727	-11.368	24.768	0.00	0.00	PROA
ATOM	388	HB2	PRO	P	27	26.008	-11.222	23.523	0.00	0.00	PROA
ATOM	389	CG	PRO	P	27	26.311	-12.836	24.801	0.00	0.00	PROA
ATOM	390	HG1	PRO	P	27	27.173	-13.094	24.149	0.00	0.00	PROA
ATOM	391	HG2	PRO	P	27	26.749	-12.476	25.756	0.00	0.00	PROA
ATOM	392	C	PRO	P	27	25.190	-13.018	21.669	0.00	0.00	PROA
ATOM	393	O	PRO	P	27	26.287	-13.515	21.564	0.00	0.00	PROA
ATOM	394	N	CYS	P	28	24.450	-12.422	20.688	0.00	0.00	PROA
ATOM	395	HN	CYS	P	28	23.488	-12.194	20.818	0.00	0.00	PROA
ATOM	396	CA	CYS	P	28	24.899	-12.222	19.351	0.00	0.00	PROA
ATOM	397	HA	CYS	P	28	25.723	-12.870	19.091	0.00	0.00	PROA
ATOM	398	CB	CYS	P	28	23.679	-12.399	18.465	0.00	0.00	PROA
ATOM	399	HB1	CYS	P	28	22.943	-11.592	18.668	0.00	0.00	PROA
ATOM	400	HB2	CYS	P	28	23.162	-13.371	18.619	0.00	0.00	PROA
ATOM	401	SG	CYS	P	28	24.032	-12.266	16.688	0.00	0.00	PROA
ATOM	402	C	CYS	P	28	25.402	-10.776	19.232	0.00	0.00	PROA
ATOM	403	O	CYS	P	28	24.750	-9.833	19.738	0.00	0.00	PROA
ATOM	404	N	PHE	P	29	26.559	-10.552	18.548	0.00	0.00	PROA
ATOM	405	HN	PHE	P	29	27.193	-11.289	18.329	0.00	0.00	PROA
ATOM	406	CA	PHE	P	29	27.188	-9.226	18.411	0.00	0.00	PROA
ATOM	407	HA	PHE	P	29	26.502	-8.534	18.877	0.00	0.00	PROA
ATOM	408	CB	PHE	P	29	28.598	-9.020	19.000	0.00	0.00	PROA
ATOM	409	HB1	PHE	P	29	28.857	-7.940	18.965	0.00	0.00	PROA
ATOM	410	HB2	PHE	P	29	29.324	-9.673	18.469	0.00	0.00	PROA
ATOM	411	CG	PHE	P	29	28.511	-9.413	20.443	0.00	0.00	PROA
ATOM	412	CD1	PHE	P	29	28.794	-10.730	20.811	0.00	0.00	PROA
ATOM	413	HD1	PHE	P	29	28.918	-11.541	20.109	0.00	0.00	PROA
ATOM	414	CE1	PHE	P	29	28.793	-11.047	22.196	0.00	0.00	PROA
ATOM	415	HE1	PHE	P	29	28.987	-12.041	22.573	0.00	0.00	PROA
ATOM	416	CZ	PHE	P	29	28.706	-10.075	23.190	0.00	0.00	PROA
ATOM	417	HZ	PHE	P	29	28.781	-10.383	24.222	0.00	0.00	PROA
ATOM	418	CD2	PHE	P	29	28.292	-8.439	21.463	0.00	0.00	PROA
ATOM	419	HD2	PHE	P	29	28.126	-7.427	21.125	0.00	0.00	PROA
ATOM	420	CE2	PHE	P	29	28.470	-8.764	22.806	0.00	0.00	PROA
ATOM	421	HE2	PHE	P	29	28.086	-7.959	23.415	0.00	0.00	PROA
ATOM	422	C	PHE	P	29	27.146	-8.910	16.892	0.00	0.00	PROA
ATOM	423	O	PHE	P	29	27.595	-9.738	16.074	0.00	0.00	PROA

ATOM	424	N	ARG	P	30	26.615	-7.735	16.454	0.00	0.00	PROA
ATOM	425	HN	ARG	P	30	26.224	-7.039	17.052	0.00	0.00	PROA
ATOM	426	CA	ARG	P	30	26.639	-7.270	15.148	0.00	0.00	PROA
ATOM	427	HA	ARG	P	30	26.933	-8.098	14.519	0.00	0.00	PROA
ATOM	428	CB	ARG	P	30	25.216	-6.797	14.701	0.00	0.00	PROA
ATOM	429	HB1	ARG	P	30	25.213	-6.413	13.659	0.00	0.00	PROA
ATOM	430	HB2	ARG	P	30	24.839	-6.000	15.377	0.00	0.00	PROA
ATOM	431	CG	ARG	P	30	24.157	-7.928	14.703	0.00	0.00	PROA
ATOM	432	HG1	ARG	P	30	24.604	-8.877	14.338	0.00	0.00	PROA
ATOM	433	HG2	ARG	P	30	23.373	-7.590	13.992	0.00	0.00	PROA
ATOM	434	CD	ARG	P	30	23.334	-8.214	15.961	0.00	0.00	PROA
ATOM	435	HD1	ARG	P	30	22.948	-7.344	16.534	0.00	0.00	PROA
ATOM	436	HD2	ARG	P	30	23.922	-8.876	16.631	0.00	0.00	PROA
ATOM	437	NE	ARG	P	30	22.165	-9.067	15.490	0.00	0.00	PROA
ATOM	438	HE	ARG	P	30	22.241	-9.809	14.825	0.00	0.00	PROA
ATOM	439	CZ	ARG	P	30	20.905	-8.695	15.578	0.00	0.00	PROA
ATOM	440	NH1	ARG	P	30	20.513	-7.579	16.209	0.00	0.00	PROA
ATOM	441	HH11	ARG	P	30	21.064	-6.939	16.746	0.00	0.00	PROA
ATOM	442	HH12	ARG	P	30	19.529	-7.435	16.319	0.00	0.00	PROA
ATOM	443	NH2	ARG	P	30	19.914	-9.419	15.073	0.00	0.00	PROA
ATOM	444	HH21	ARG	P	30	20.063	-10.323	14.670	0.00	0.00	PROA
ATOM	445	HH22	ARG	P	30	18.966	-9.113	15.154	0.00	0.00	PROA
ATOM	446	C	ARG	P	30	27.561	-6.081	14.938	0.00	0.00	PROA
ATOM	447	O	ARG	P	30	27.387	-5.356	13.984	0.00	0.00	PROA
ATOM	448	N	GLU	P	31	28.509	-5.855	15.894	0.00	0.00	PROA
ATOM	449	HN	GLU	P	31	28.801	-6.506	16.590	0.00	0.00	PROA
ATOM	450	CA	GLU	P	31	29.106	-4.587	16.042	0.00	0.00	PROA
ATOM	451	HA	GLU	P	31	28.295	-3.885	15.917	0.00	0.00	PROA
ATOM	452	CB	GLU	P	31	29.750	-4.601	17.472	0.00	0.00	PROA
ATOM	453	HB1	GLU	P	31	30.528	-3.809	17.418	0.00	0.00	PROA
ATOM	454	HB2	GLU	P	31	30.236	-5.588	17.630	0.00	0.00	PROA
ATOM	455	CG	GLU	P	31	28.574	-4.499	18.526	0.00	0.00	PROA
ATOM	456	HG1	GLU	P	31	27.998	-5.450	18.537	0.00	0.00	PROA
ATOM	457	HG2	GLU	P	31	27.941	-3.678	18.128	0.00	0.00	PROA
ATOM	458	CD	GLU	P	31	28.997	-4.132	19.927	0.00	0.00	PROA
ATOM	459	OE1	GLU	P	31	29.172	-4.995	20.840	0.00	0.00	PROA
ATOM	460	OE2	GLU	P	31	28.996	-2.875	20.133	0.00	0.00	PROA
ATOM	461	C	GLU	P	31	29.990	-4.192	14.870	0.00	0.00	PROA
ATOM	462	O	GLU	P	31	30.009	-3.034	14.481	0.00	0.00	PROA
ATOM	463	N	GLU	P	32	30.727	-5.099	14.256	0.00	0.00	PROA
ATOM	464	HN	GLU	P	32	30.887	-6.026	14.588	0.00	0.00	PROA
ATOM	465	CA	GLU	P	32	31.640	-4.789	13.181	0.00	0.00	PROA
ATOM	466	HA	GLU	P	32	32.200	-3.907	13.454	0.00	0.00	PROA
ATOM	467	CB	GLU	P	32	32.667	-5.919	13.014	0.00	0.00	PROA
ATOM	468	HB1	GLU	P	32	33.142	-5.749	12.025	0.00	0.00	PROA
ATOM	469	HB2	GLU	P	32	32.095	-6.870	12.981	0.00	0.00	PROA
ATOM	470	CG	GLU	P	32	33.582	-6.059	14.203	0.00	0.00	PROA
ATOM	471	HG1	GLU	P	32	32.950	-6.224	15.102	0.00	0.00	PROA
ATOM	472	HG2	GLU	P	32	34.145	-5.116	14.367	0.00	0.00	PROA
ATOM	473	CD	GLU	P	32	34.623	-7.110	14.028	0.00	0.00	PROA
ATOM	474	OE1	GLU	P	32	35.438	-7.080	13.037	0.00	0.00	PROA
ATOM	475	OE2	GLU	P	32	34.735	-7.964	14.978	0.00	0.00	PROA
ATOM	476	C	GLU	P	32	30.928	-4.401	11.906	0.00	0.00	PROA
ATOM	477	O	GLU	P	32	31.421	-3.534	11.199	0.00	0.00	PROA
ATOM	478	N	ASN	P	33	29.796	-5.079	11.590	0.00	0.00	PROA
ATOM	479	HN	ASN	P	33	29.351	-5.759	12.168	0.00	0.00	PROA
ATOM	480	CA	ASN	P	33	28.998	-4.695	10.453	0.00	0.00	PROA
ATOM	481	HA	ASN	P	33	29.724	-4.783	9.658	0.00	0.00	PROA
ATOM	482	CB	ASN	P	33	27.757	-5.696	10.286	0.00	0.00	PROA
ATOM	483	HB1	ASN	P	33	27.065	-5.237	9.548	0.00	0.00	PROA
ATOM	484	HB2	ASN	P	33	27.257	-5.942	11.247	0.00	0.00	PROA

ATOM	485	CG	ASN	P	33	28.234	-7.005	9.670	0.00	0.00	PROA
ATOM	486	OD1	ASN	P	33	29.417	-7.126	9.263	0.00	0.00	PROA
ATOM	487	ND2	ASN	P	33	27.448	-8.125	9.731	0.00	0.00	PROA
ATOM	488	HD21	ASN	P	33	27.775	-8.748	9.020	0.00	0.00	PROA
ATOM	489	HD22	ASN	P	33	26.550	-8.102	10.169	0.00	0.00	PROA
ATOM	490	C	ASN	P	33	28.531	-3.210	10.445	0.00	0.00	PROA
ATOM	491	O	ASN	P	33	28.684	-2.607	9.408	0.00	0.00	PROA
ATOM	492	N	ALA	P	34	27.990	-2.608	11.547	0.00	0.00	PROA
ATOM	493	HN	ALA	P	34	27.794	-3.091	12.396	0.00	0.00	PROA
ATOM	494	CA	ALA	P	34	27.655	-1.199	11.712	0.00	0.00	PROA
ATOM	495	HA	ALA	P	34	26.947	-0.924	10.944	0.00	0.00	PROA
ATOM	496	CB	ALA	P	34	27.080	-0.901	13.133	0.00	0.00	PROA
ATOM	497	HB1	ALA	P	34	26.987	0.188	13.330	0.00	0.00	PROA
ATOM	498	HB2	ALA	P	34	27.838	-1.194	13.891	0.00	0.00	PROA
ATOM	499	HB3	ALA	P	34	26.184	-1.510	13.378	0.00	0.00	PROA
ATOM	500	C	ALA	P	34	28.910	-0.302	11.554	0.00	0.00	PROA
ATOM	501	O	ALA	P	34	28.993	0.778	10.952	0.00	0.00	PROA
ATOM	502	N	ASN	P	35	30.091	-0.693	12.140	0.00	0.00	PROA
ATOM	503	HN	ASN	P	35	30.156	-1.405	12.835	0.00	0.00	PROA
ATOM	504	CA	ASN	P	35	31.409	-0.009	11.969	0.00	0.00	PROA
ATOM	505	HA	ASN	P	35	31.389	1.054	12.157	0.00	0.00	PROA
ATOM	506	CB	ASN	P	35	32.559	-0.734	12.899	0.00	0.00	PROA
ATOM	507	HB1	ASN	P	35	33.567	-0.307	12.708	0.00	0.00	PROA
ATOM	508	HB2	ASN	P	35	32.452	-1.806	12.625	0.00	0.00	PROA
ATOM	509	CG	ASN	P	35	32.294	-0.576	14.398	0.00	0.00	PROA
ATOM	510	OD1	ASN	P	35	31.479	0.190	14.898	0.00	0.00	PROA
ATOM	511	ND2	ASN	P	35	33.034	-1.443	15.072	0.00	0.00	PROA
ATOM	512	HD21	ASN	P	35	33.004	-1.433	16.072	0.00	0.00	PROA
ATOM	513	HD22	ASN	P	35	33.491	-2.161	14.547	0.00	0.00	PROA
ATOM	514	C	ASN	P	35	31.833	-0.110	10.507	0.00	0.00	PROA
ATOM	515	O	ASN	P	35	32.371	0.842	10.015	0.00	0.00	PROA
ATOM	516	N	PHE	P	36	31.714	-1.242	9.805	0.00	0.00	PROA
ATOM	517	HN	PHE	P	36	31.306	-2.005	10.300	0.00	0.00	PROA
ATOM	518	CA	PHE	P	36	31.991	-1.469	8.438	0.00	0.00	PROA
ATOM	519	HA	PHE	P	36	33.017	-1.241	8.189	0.00	0.00	PROA
ATOM	520	CB	PHE	P	36	31.873	-2.940	7.980	0.00	0.00	PROA
ATOM	521	HB1	PHE	P	36	30.895	-3.367	8.290	0.00	0.00	PROA
ATOM	522	HB2	PHE	P	36	32.583	-3.539	8.590	0.00	0.00	PROA
ATOM	523	CG	PHE	P	36	32.222	-3.166	6.556	0.00	0.00	PROA
ATOM	524	CD1	PHE	P	36	31.142	-3.340	5.598	0.00	0.00	PROA
ATOM	525	HD1	PHE	P	36	30.120	-3.350	5.946	0.00	0.00	PROA
ATOM	526	CE1	PHE	P	36	31.388	-3.451	4.273	0.00	0.00	PROA
ATOM	527	HE1	PHE	P	36	30.530	-3.542	3.624	0.00	0.00	PROA
ATOM	528	CZ	PHE	P	36	32.692	-3.507	3.804	0.00	0.00	PROA
ATOM	529	HZ	PHE	P	36	32.909	-3.461	2.747	0.00	0.00	PROA
ATOM	530	CD2	PHE	P	36	33.554	-3.378	6.035	0.00	0.00	PROA
ATOM	531	HD2	PHE	P	36	34.382	-3.190	6.701	0.00	0.00	PROA
ATOM	532	CE2	PHE	P	36	33.759	-3.410	4.693	0.00	0.00	PROA
ATOM	533	HE2	PHE	P	36	34.746	-3.302	4.268	0.00	0.00	PROA
ATOM	534	C	PHE	P	36	31.119	-0.528	7.581	0.00	0.00	PROA
ATOM	535	O	PHE	P	36	31.572	0.310	6.805	0.00	0.00	PROA
ATOM	536	N	ASN	P	37	29.813	-0.555	7.820	0.00	0.00	PROA
ATOM	537	HN	ASN	P	37	29.334	-1.309	8.263	0.00	0.00	PROA
ATOM	538	CA	ASN	P	37	28.886	0.172	6.960	0.00	0.00	PROA
ATOM	539	HA	ASN	P	37	29.101	-0.033	5.922	0.00	0.00	PROA
ATOM	540	CB	ASN	P	37	27.456	-0.218	7.190	0.00	0.00	PROA
ATOM	541	HB1	ASN	P	37	26.692	0.569	7.014	0.00	0.00	PROA
ATOM	542	HB2	ASN	P	37	27.418	-0.436	8.279	0.00	0.00	PROA
ATOM	543	CG	ASN	P	37	27.104	-1.540	6.454	0.00	0.00	PROA
ATOM	544	OD1	ASN	P	37	27.829	-2.094	5.598	0.00	0.00	PROA
ATOM	545	ND2	ASN	P	37	25.853	-1.957	6.624	0.00	0.00	PROA

ATOM	546	HD21	ASN	P	37	25.422	-2.497	5.901	0.00	0.00	PROA
ATOM	547	HD22	ASN	P	37	25.322	-1.651	7.415	0.00	0.00	PROA
ATOM	548	C	ASN	P	37	28.987	1.714	7.067	0.00	0.00	PROA
ATOM	549	O	ASN	P	37	28.672	2.417	6.096	0.00	0.00	PROA
ATOM	550	N	LYS	P	38	29.416	2.339	8.228	0.00	0.00	PROA
ATOM	551	HN	LYS	P	38	29.698	1.689	8.930	0.00	0.00	PROA
ATOM	552	CA	LYS	P	38	29.454	3.761	8.495	0.00	0.00	PROA
ATOM	553	HA	LYS	P	38	28.528	4.249	8.227	0.00	0.00	PROA
ATOM	554	CB	LYS	P	38	29.552	3.940	10.056	0.00	0.00	PROA
ATOM	555	HB1	LYS	P	38	30.591	3.754	10.404	0.00	0.00	PROA
ATOM	556	HB2	LYS	P	38	28.915	3.244	10.642	0.00	0.00	PROA
ATOM	557	CG	LYS	P	38	29.053	5.370	10.346	0.00	0.00	PROA
ATOM	558	HG1	LYS	P	38	27.942	5.377	10.333	0.00	0.00	PROA
ATOM	559	HG2	LYS	P	38	29.344	6.081	9.544	0.00	0.00	PROA
ATOM	560	CD	LYS	P	38	29.474	5.990	11.688	0.00	0.00	PROA
ATOM	561	HD1	LYS	P	38	29.141	5.363	12.543	0.00	0.00	PROA
ATOM	562	HD2	LYS	P	38	28.846	6.906	11.689	0.00	0.00	PROA
ATOM	563	CE	LYS	P	38	30.934	6.459	11.782	0.00	0.00	PROA
ATOM	564	HE1	LYS	P	38	31.193	6.970	10.830	0.00	0.00	PROA
ATOM	565	HE2	LYS	P	38	31.564	5.557	11.938	0.00	0.00	PROA
ATOM	566	NZ	LYS	P	38	31.019	7.388	12.932	0.00	0.00	PROA
ATOM	567	HZ1	LYS	P	38	30.630	6.887	13.757	0.00	0.00	PROA
ATOM	568	HZ2	LYS	P	38	30.508	8.270	12.725	0.00	0.00	PROA
ATOM	569	HZ3	LYS	P	38	32.005	7.675	13.095	0.00	0.00	PROA
ATOM	570	C	LYS	P	38	30.640	4.394	7.868	0.00	0.00	PROA
ATOM	571	O	LYS	P	38	30.775	5.585	7.852	0.00	0.00	PROA
ATOM	572	N	ILE	P	39	31.526	3.567	7.177	0.00	0.00	PROA
ATOM	573	HN	ILE	P	39	31.402	2.578	7.219	0.00	0.00	PROA
ATOM	574	CA	ILE	P	39	32.624	4.139	6.319	0.00	0.00	PROA
ATOM	575	HA	ILE	P	39	32.536	5.203	6.159	0.00	0.00	PROA
ATOM	576	CB	ILE	P	39	34.035	3.736	6.863	0.00	0.00	PROA
ATOM	577	HB	ILE	P	39	34.063	2.629	6.957	0.00	0.00	PROA
ATOM	578	CG2	ILE	P	39	35.093	4.236	5.848	0.00	0.00	PROA
ATOM	579	HG21	ILE	P	39	36.027	4.172	6.446	0.00	0.00	PROA
ATOM	580	HG22	ILE	P	39	34.941	5.285	5.515	0.00	0.00	PROA
ATOM	581	HG23	ILE	P	39	35.194	3.509	5.014	0.00	0.00	PROA
ATOM	582	CG1	ILE	P	39	34.231	4.379	8.255	0.00	0.00	PROA
ATOM	583	HG11	ILE	P	39	35.229	3.988	8.551	0.00	0.00	PROA
ATOM	584	HG12	ILE	P	39	33.499	3.967	8.982	0.00	0.00	PROA
ATOM	585	CD	ILE	P	39	34.247	5.917	8.259	0.00	0.00	PROA
ATOM	586	HD1	ILE	P	39	33.243	6.379	8.140	0.00	0.00	PROA
ATOM	587	HD2	ILE	P	39	34.956	6.334	7.512	0.00	0.00	PROA
ATOM	588	HD3	ILE	P	39	34.654	6.290	9.223	0.00	0.00	PROA
ATOM	589	C	ILE	P	39	32.479	3.618	4.908	0.00	0.00	PROA
ATOM	590	O	ILE	P	39	32.747	4.335	3.948	0.00	0.00	PROA
ATOM	591	N	PHE	P	40	32.123	2.284	4.785	0.00	0.00	PROA
ATOM	592	HN	PHE	P	40	31.988	1.685	5.570	0.00	0.00	PROA
ATOM	593	CA	PHE	P	40	32.168	1.704	3.424	0.00	0.00	PROA
ATOM	594	HA	PHE	P	40	33.117	1.773	2.912	0.00	0.00	PROA
ATOM	595	CB	PHE	P	40	31.842	0.181	3.541	0.00	0.00	PROA
ATOM	596	HB1	PHE	P	40	31.080	0.068	4.342	0.00	0.00	PROA
ATOM	597	HB2	PHE	P	40	32.720	-0.313	4.008	0.00	0.00	PROA
ATOM	598	CG	PHE	P	40	31.525	-0.646	2.360	0.00	0.00	PROA
ATOM	599	CD1	PHE	P	40	30.196	-1.146	2.285	0.00	0.00	PROA
ATOM	600	HD1	PHE	P	40	29.380	-1.027	2.983	0.00	0.00	PROA
ATOM	601	CE1	PHE	P	40	29.858	-1.988	1.233	0.00	0.00	PROA
ATOM	602	HE1	PHE	P	40	28.879	-2.436	1.153	0.00	0.00	PROA
ATOM	603	CZ	PHE	P	40	30.770	-2.253	0.196	0.00	0.00	PROA
ATOM	604	HZ	PHE	P	40	30.441	-2.929	-0.579	0.00	0.00	PROA
ATOM	605	CD2	PHE	P	40	32.487	-1.109	1.421	0.00	0.00	PROA
ATOM	606	HD2	PHE	P	40	33.520	-0.884	1.645	0.00	0.00	PROA

ATOM	607	CE2	PHE	P	40	32.119	-1.954	0.391	0.00	0.00	PROA
ATOM	608	HE2	PHE	P	40	32.866	-2.371	-0.267	0.00	0.00	PROA
ATOM	609	C	PHE	P	40	31.176	2.406	2.508	0.00	0.00	PROA
ATOM	610	O	PHE	P	40	31.530	2.779	1.417	0.00	0.00	PROA
ATOM	611	N	LEU	P	41	29.959	2.525	2.969	0.00	0.00	PROA
ATOM	612	HN	LEU	P	41	29.660	2.172	3.852	0.00	0.00	PROA
ATOM	613	CA	LEU	P	41	28.821	3.135	2.253	0.00	0.00	PROA
ATOM	614	HA	LEU	P	41	28.733	2.562	1.341	0.00	0.00	PROA
ATOM	615	CB	LEU	P	41	27.470	2.780	3.045	0.00	0.00	PROA
ATOM	616	HB1	LEU	P	41	26.565	3.239	2.593	0.00	0.00	PROA
ATOM	617	HB2	LEU	P	41	27.394	3.193	4.073	0.00	0.00	PROA
ATOM	618	CG	LEU	P	41	27.174	1.232	3.043	0.00	0.00	PROA
ATOM	619	HG	LEU	P	41	27.992	0.785	3.646	0.00	0.00	PROA
ATOM	620	CD1	LEU	P	41	25.805	0.930	3.588	0.00	0.00	PROA
ATOM	621	HD11	LEU	P	41	25.509	1.505	4.492	0.00	0.00	PROA
ATOM	622	HD12	LEU	P	41	25.807	-0.165	3.776	0.00	0.00	PROA
ATOM	623	HD13	LEU	P	41	25.032	1.126	2.813	0.00	0.00	PROA
ATOM	624	CD2	LEU	P	41	27.226	0.581	1.664	0.00	0.00	PROA
ATOM	625	HD21	LEU	P	41	26.815	-0.450	1.686	0.00	0.00	PROA
ATOM	626	HD22	LEU	P	41	28.246	0.564	1.224	0.00	0.00	PROA
ATOM	627	HD23	LEU	P	41	26.531	1.086	0.961	0.00	0.00	PROA
ATOM	628	C	LEU	P	41	28.942	4.646	1.849	0.00	0.00	PROA
ATOM	629	O	LEU	P	41	28.651	4.899	0.660	0.00	0.00	PROA
ATOM	630	N	PRO	P	42	29.396	5.657	2.632	0.00	0.00	PROA
ATOM	631	CD	PRO	P	42	29.525	5.504	4.096	0.00	0.00	PROA
ATOM	632	HD1	PRO	P	42	30.097	4.594	4.380	0.00	0.00	PROA
ATOM	633	HD2	PRO	P	42	28.495	5.451	4.508	0.00	0.00	PROA
ATOM	634	CA	PRO	P	42	29.512	7.088	2.197	0.00	0.00	PROA
ATOM	635	HA	PRO	P	42	28.590	7.260	1.661	0.00	0.00	PROA
ATOM	636	CB	PRO	P	42	29.788	7.840	3.547	0.00	0.00	PROA
ATOM	637	HB1	PRO	P	42	28.819	8.305	3.828	0.00	0.00	PROA
ATOM	638	HB2	PRO	P	42	30.446	8.733	3.476	0.00	0.00	PROA
ATOM	639	CG	PRO	P	42	30.252	6.777	4.543	0.00	0.00	PROA
ATOM	640	HG1	PRO	P	42	31.349	6.707	4.383	0.00	0.00	PROA
ATOM	641	HG2	PRO	P	42	29.851	7.014	5.551	0.00	0.00	PROA
ATOM	642	C	PRO	P	42	30.678	7.182	1.261	0.00	0.00	PROA
ATOM	643	O	PRO	P	42	30.736	8.097	0.440	0.00	0.00	PROA
ATOM	644	N	THR	P	43	31.752	6.371	1.358	0.00	0.00	PROA
ATOM	645	HN	THR	P	43	31.771	5.679	2.076	0.00	0.00	PROA
ATOM	646	CA	THR	P	43	32.829	6.416	0.320	0.00	0.00	PROA
ATOM	647	HA	THR	P	43	32.965	7.482	0.220	0.00	0.00	PROA
ATOM	648	CB	THR	P	43	33.991	5.494	0.608	0.00	0.00	PROA
ATOM	649	HB	THR	P	43	33.766	4.421	0.424	0.00	0.00	PROA
ATOM	650	OG1	THR	P	43	34.462	5.625	1.937	0.00	0.00	PROA
ATOM	651	HG1	THR	P	43	33.905	5.253	2.624	0.00	0.00	PROA
ATOM	652	CG2	THR	P	43	35.118	5.828	-0.429	0.00	0.00	PROA
ATOM	653	HG21	THR	P	43	35.626	6.789	-0.201	0.00	0.00	PROA
ATOM	654	HG22	THR	P	43	34.901	5.752	-1.516	0.00	0.00	PROA
ATOM	655	HG23	THR	P	43	35.881	5.081	-0.124	0.00	0.00	PROA
ATOM	656	C	THR	P	43	32.133	5.874	-0.985	0.00	0.00	PROA
ATOM	657	O	THR	P	43	32.321	6.462	-2.133	0.00	0.00	PROA
ATOM	658	N	ILE	P	44	31.310	4.825	-0.922	0.00	0.00	PROA
ATOM	659	HN	ILE	P	44	31.264	4.296	-0.078	0.00	0.00	PROA
ATOM	660	CA	ILE	P	44	30.667	4.274	-2.129	0.00	0.00	PROA
ATOM	661	HA	ILE	P	44	31.355	4.258	-2.962	0.00	0.00	PROA
ATOM	662	CB	ILE	P	44	30.077	2.891	-1.728	0.00	0.00	PROA
ATOM	663	HB	ILE	P	44	29.644	3.033	-0.714	0.00	0.00	PROA
ATOM	664	CG2	ILE	P	44	28.969	2.389	-2.694	0.00	0.00	PROA
ATOM	665	HG21	ILE	P	44	28.501	1.487	-2.245	0.00	0.00	PROA
ATOM	666	HG22	ILE	P	44	29.290	2.203	-3.741	0.00	0.00	PROA
ATOM	667	HG23	ILE	P	44	28.101	3.075	-2.794	0.00	0.00	PROA

```
ATOM    668  CG1 ILE P  44      31.259   1.894  -1.456  0.00  0.00      PROA
ATOM    669 HG11 ILE P  44      31.084   0.968  -0.868  0.00  0.00      PROA
ATOM    670 HG12 ILE P  44      31.994   2.497  -0.881  0.00  0.00      PROA
ATOM    671  CD  ILE P  44      32.163   1.504  -2.669  0.00  0.00      PROA
ATOM    672  HD1 ILE P  44      31.587   0.849  -3.356  0.00  0.00      PROA
ATOM    673  HD2 ILE P  44      33.006   0.890  -2.288  0.00  0.00      PROA
ATOM    674  HD3 ILE P  44      32.611   2.381  -3.184  0.00  0.00      PROA
ATOM    675  C   ILE P  44      29.636   5.164  -2.764  0.00  0.00      PROA
ATOM    676  O   ILE P  44      29.632   5.297  -3.978  0.00  0.00      PROA
ATOM    677  N   TYR P  45      28.782   5.866  -2.007  0.00  0.00      PROA
ATOM    678  HN  TYR P  45      28.615   5.517  -1.089  0.00  0.00      PROA
ATOM    679  CA  TYR P  45      27.834   6.872  -2.438  0.00  0.00      PROA
ATOM    680  HA  TYR P  45      27.277   6.435  -3.254  0.00  0.00      PROA
ATOM    681  CB  TYR P  45      26.892   7.391  -1.323  0.00  0.00      PROA
ATOM    682  HB1 TYR P  45      26.304   8.251  -1.709  0.00  0.00      PROA
ATOM    683  HB2 TYR P  45      27.441   7.868  -0.483  0.00  0.00      PROA
ATOM    684  CG  TYR P  45      26.048   6.334  -0.716  0.00  0.00      PROA
ATOM    685  CD1 TYR P  45      25.826   6.250   0.678  0.00  0.00      PROA
ATOM    686  HD1 TYR P  45      26.368   6.953   1.293  0.00  0.00      PROA
ATOM    687  CE1 TYR P  45      25.010   5.281   1.319  0.00  0.00      PROA
ATOM    688  HE1 TYR P  45      24.893   5.357   2.390  0.00  0.00      PROA
ATOM    689  CZ  TYR P  45      24.460   4.272   0.532  0.00  0.00      PROA
ATOM    690  OH  TYR P  45      23.695   3.185   1.225  0.00  0.00      PROA
ATOM    691  HH  TYR P  45      23.512   2.489   0.589  0.00  0.00      PROA
ATOM    692  CD2 TYR P  45      25.499   5.238  -1.482  0.00  0.00      PROA
ATOM    693  HD2 TYR P  45      25.709   5.151  -2.537  0.00  0.00      PROA
ATOM    694  CE2 TYR P  45      24.748   4.234  -0.844  0.00  0.00      PROA
ATOM    695  HE2 TYR P  45      24.398   3.327  -1.316  0.00  0.00      PROA
ATOM    696  C   TYR P  45      28.511   8.057  -2.990  0.00  0.00      PROA
ATOM    697  O   TYR P  45      27.976   8.695  -3.825  0.00  0.00      PROA
ATOM    698  N   SER P  46      29.666   8.409  -2.364  0.00  0.00      PROA
ATOM    699  HN  SER P  46      29.987   7.785  -1.656  0.00  0.00      PROA
ATOM    700  CA  SER P  46      30.502   9.466  -2.889  0.00  0.00      PROA
ATOM    701  HA  SER P  46      29.915  10.369  -2.962  0.00  0.00      PROA
ATOM    702  CB  SER P  46      31.787   9.856  -2.085  0.00  0.00      PROA
ATOM    703  HB1 SER P  46      32.176  10.821  -2.473  0.00  0.00      PROA
ATOM    704  HB2 SER P  46      32.537   9.042  -1.980  0.00  0.00      PROA
ATOM    705  OG  SER P  46      31.381  10.282  -0.829  0.00  0.00      PROA
ATOM    706  HG1 SER P  46      31.107   9.532  -0.295  0.00  0.00      PROA
ATOM    707  C   SER P  46      30.979   9.305  -4.284  0.00  0.00      PROA
ATOM    708  O   SER P  46      30.938  10.228  -5.084  0.00  0.00      PROA
ATOM    709  N   ILE P  47      31.521   8.141  -4.628  0.00  0.00      PROA
ATOM    710  HN  ILE P  47      31.647   7.570  -3.821  0.00  0.00      PROA
ATOM    711  CA  ILE P  47      32.015   7.752  -5.946  0.00  0.00      PROA
ATOM    712  HA  ILE P  47      32.651   8.563  -6.269  0.00  0.00      PROA
ATOM    713  CB  ILE P  47      32.751   6.405  -5.895  0.00  0.00      PROA
ATOM    714  HB  ILE P  47      32.185   5.600  -5.380  0.00  0.00      PROA
ATOM    715  CG2 ILE P  47      33.113   5.940  -7.350  0.00  0.00      PROA
ATOM    716 HG21 ILE P  47      33.806   5.073  -7.304  0.00  0.00      PROA
ATOM    717 HG22 ILE P  47      33.586   6.703  -8.004  0.00  0.00      PROA
ATOM    718 HG23 ILE P  47      32.164   5.645  -7.846  0.00  0.00      PROA
ATOM    719  CG1 ILE P  47      34.047   6.617  -5.048  0.00  0.00      PROA
ATOM    720 HG11 ILE P  47      33.737   6.800  -3.997  0.00  0.00      PROA
ATOM    721 HG12 ILE P  47      34.562   7.506  -5.471  0.00  0.00      PROA
ATOM    722  CD  ILE P  47      35.000   5.395  -4.986  0.00  0.00      PROA
ATOM    723  HD1 ILE P  47      35.645   5.652  -4.119  0.00  0.00      PROA
ATOM    724  HD2 ILE P  47      35.592   5.363  -5.925  0.00  0.00      PROA
ATOM    725  HD3 ILE P  47      34.458   4.467  -4.704  0.00  0.00      PROA
ATOM    726  C   ILE P  47      30.886   7.685  -6.934  0.00  0.00      PROA
ATOM    727  O   ILE P  47      30.901   8.292  -7.991  0.00  0.00      PROA
ATOM    728  N   ILE P  48      29.837   7.034  -6.542  0.00  0.00      PROA
```

ATOM	729	HN	ILE	P	48	29.801	6.653	-5.622	0.00	0.00	PROA
ATOM	730	CA	ILE	P	48	28.575	7.005	-7.259	0.00	0.00	PROA
ATOM	731	HA	ILE	P	48	28.773	6.461	-8.170	0.00	0.00	PROA
ATOM	732	CB	ILE	P	48	27.569	5.982	-6.699	0.00	0.00	PROA
ATOM	733	HB	ILE	P	48	27.607	6.142	-5.601	0.00	0.00	PROA
ATOM	734	CG2	ILE	P	48	26.084	6.126	-7.078	0.00	0.00	PROA
ATOM	735	HG21	ILE	P	48	25.738	7.077	-6.620	0.00	0.00	PROA
ATOM	736	HG22	ILE	P	48	25.564	5.209	-6.729	0.00	0.00	PROA
ATOM	737	HG23	ILE	P	48	25.781	6.072	-8.145	0.00	0.00	PROA
ATOM	738	CG1	ILE	P	48	28.058	4.515	-6.941	0.00	0.00	PROA
ATOM	739	HG11	ILE	P	48	29.161	4.464	-6.820	0.00	0.00	PROA
ATOM	740	HG12	ILE	P	48	27.945	4.243	-8.012	0.00	0.00	PROA
ATOM	741	CD	ILE	P	48	27.481	3.449	-6.045	0.00	0.00	PROA
ATOM	742	HD1	ILE	P	48	28.033	2.487	-6.114	0.00	0.00	PROA
ATOM	743	HD2	ILE	P	48	26.400	3.223	-6.168	0.00	0.00	PROA
ATOM	744	HD3	ILE	P	48	27.592	3.798	-4.996	0.00	0.00	PROA
ATOM	745	C	ILE	P	48	28.021	8.415	-7.636	0.00	0.00	PROA
ATOM	746	O	ILE	P	48	27.881	8.678	-8.806	0.00	0.00	PROA
ATOM	747	N	PHE	P	49	27.816	9.251	-6.628	0.00	0.00	PROA
ATOM	748	HN	PHE	P	49	28.022	8.979	-5.692	0.00	0.00	PROA
ATOM	749	CA	PHE	P	49	27.147	10.480	-6.836	0.00	0.00	PROA
ATOM	750	HA	PHE	P	49	26.144	10.443	-7.234	0.00	0.00	PROA
ATOM	751	CB	PHE	P	49	26.785	11.216	-5.511	0.00	0.00	PROA
ATOM	752	HB1	PHE	P	49	27.780	11.280	-5.019	0.00	0.00	PROA
ATOM	753	HB2	PHE	P	49	26.174	10.557	-4.858	0.00	0.00	PROA
ATOM	754	CG	PHE	P	49	26.272	12.644	-5.718	0.00	0.00	PROA
ATOM	755	CD1	PHE	P	49	25.046	12.769	-6.384	0.00	0.00	PROA
ATOM	756	HD1	PHE	P	49	24.473	11.940	-6.775	0.00	0.00	PROA
ATOM	757	CE1	PHE	P	49	24.643	14.007	-6.786	0.00	0.00	PROA
ATOM	758	HE1	PHE	P	49	23.779	14.197	-7.405	0.00	0.00	PROA
ATOM	759	CZ	PHE	P	49	25.292	15.176	-6.390	0.00	0.00	PROA
ATOM	760	HZ	PHE	P	49	24.905	16.149	-6.655	0.00	0.00	PROA
ATOM	761	CD2	PHE	P	49	26.990	13.796	-5.378	0.00	0.00	PROA
ATOM	762	HD2	PHE	P	49	27.980	13.746	-4.947	0.00	0.00	PROA
ATOM	763	CE2	PHE	P	49	26.418	15.072	-5.534	0.00	0.00	PROA
ATOM	764	HE2	PHE	P	49	26.866	15.895	-4.997	0.00	0.00	PROA
ATOM	765	C	PHE	P	49	27.771	11.364	-7.884	0.00	0.00	PROA
ATOM	766	O	PHE	P	49	27.064	11.611	-8.860	0.00	0.00	PROA
ATOM	767	N	LEU	P	50	29.089	11.576	-7.728	0.00	0.00	PROA
ATOM	768	HN	LEU	P	50	29.395	11.312	-6.817	0.00	0.00	PROA
ATOM	769	CA	LEU	P	50	29.932	12.375	-8.555	0.00	0.00	PROA
ATOM	770	HA	LEU	P	50	29.429	13.305	-8.776	0.00	0.00	PROA
ATOM	771	CB	LEU	P	50	31.305	12.691	-8.020	0.00	0.00	PROA
ATOM	772	HB1	LEU	P	50	31.883	13.339	-8.712	0.00	0.00	PROA
ATOM	773	HB2	LEU	P	50	31.879	11.813	-7.654	0.00	0.00	PROA
ATOM	774	CG	LEU	P	50	31.218	13.518	-6.712	0.00	0.00	PROA
ATOM	775	HG	LEU	P	50	30.664	12.858	-6.011	0.00	0.00	PROA
ATOM	776	CD1	LEU	P	50	32.608	13.719	-6.015	0.00	0.00	PROA
ATOM	777	HD11	LEU	P	50	33.399	14.016	-6.736	0.00	0.00	PROA
ATOM	778	HD12	LEU	P	50	32.933	12.738	-5.607	0.00	0.00	PROA
ATOM	779	HD13	LEU	P	50	32.681	14.485	-5.213	0.00	0.00	PROA
ATOM	780	CD2	LEU	P	50	30.415	14.782	-6.838	0.00	0.00	PROA
ATOM	781	HD21	LEU	P	50	30.494	15.469	-5.969	0.00	0.00	PROA
ATOM	782	HD22	LEU	P	50	29.345	14.534	-7.002	0.00	0.00	PROA
ATOM	783	HD23	LEU	P	50	30.821	15.320	-7.721	0.00	0.00	PROA
ATOM	784	C	LEU	P	50	30.139	11.838	-9.981	0.00	0.00	PROA
ATOM	785	O	LEU	P	50	30.112	12.625	-10.958	0.00	0.00	PROA
ATOM	786	N	THR	P	51	30.171	10.518	-10.233	0.00	0.00	PROA
ATOM	787	HN	THR	P	51	30.192	9.901	-9.450	0.00	0.00	PROA
ATOM	788	CA	THR	P	51	30.012	9.972	-11.630	0.00	0.00	PROA
ATOM	789	HA	THR	P	51	30.783	10.342	-12.290	0.00	0.00	PROA

```
ATOM    790  CB  THR P  51      30.216   8.483 -11.679  0.00  0.00      PROA
ATOM    791  HB  THR P  51      29.336   7.979 -11.224  0.00  0.00      PROA
ATOM    792  OG1 THR P  51      31.378   8.091 -11.042  0.00  0.00      PROA
ATOM    793  HG1 THR P  51      31.394   8.504 -10.176  0.00  0.00      PROA
ATOM    794  CG2 THR P  51      30.395   7.908 -13.157  0.00  0.00      PROA
ATOM    795 HG21 THR P  51      31.172   8.559 -13.611  0.00  0.00      PROA
ATOM    796 HG22 THR P  51      29.525   8.056 -13.832  0.00  0.00      PROA
ATOM    797 HG23 THR P  51      30.752   6.861 -13.264  0.00  0.00      PROA
ATOM    798  C   THR P  51      28.656  10.316 -12.215  0.00  0.00      PROA
ATOM    799  O   THR P  51      28.531  10.825 -13.302  0.00  0.00      PROA
ATOM    800  N   GLY P  52      27.558   9.960 -11.509  0.00  0.00      PROA
ATOM    801  HN  GLY P  52      27.827   9.571 -10.631  0.00  0.00      PROA
ATOM    802  CA  GLY P  52      26.203   9.905 -11.929  0.00  0.00      PROA
ATOM    803  HA1 GLY P  52      25.594   9.827 -11.041  0.00  0.00      PROA
ATOM    804  HA2 GLY P  52      26.107   9.172 -12.716  0.00  0.00      PROA
ATOM    805  C   GLY P  52      25.746  11.140 -12.644  0.00  0.00      PROA
ATOM    806  O   GLY P  52      25.244  11.138 -13.811  0.00  0.00      PROA
ATOM    807  N   ILE P  53      25.867  12.246 -11.852  0.00  0.00      PROA
ATOM    808  HN  ILE P  53      26.177  12.132 -10.911  0.00  0.00      PROA
ATOM    809  CA  ILE P  53      25.406  13.587 -12.191  0.00  0.00      PROA
ATOM    810  HA  ILE P  53      24.334  13.680 -12.276  0.00  0.00      PROA
ATOM    811  CB  ILE P  53      25.843  14.646 -11.183  0.00  0.00      PROA
ATOM    812  HB  ILE P  53      25.783  15.631 -11.693  0.00  0.00      PROA
ATOM    813  CG2 ILE P  53      24.814  14.541 -10.049  0.00  0.00      PROA
ATOM    814 HG21 ILE P  53      24.854  13.499  -9.663  0.00  0.00      PROA
ATOM    815 HG22 ILE P  53      23.813  14.838 -10.429  0.00  0.00      PROA
ATOM    816 HG23 ILE P  53      25.028  15.267  -9.236  0.00  0.00      PROA
ATOM    817  CG1 ILE P  53      27.335  14.413 -10.649  0.00  0.00      PROA
ATOM    818 HG11 ILE P  53      27.923  13.919 -11.452  0.00  0.00      PROA
ATOM    819 HG12 ILE P  53      27.233  13.767  -9.751  0.00  0.00      PROA
ATOM    820  CD  ILE P  53      28.108  15.703 -10.296  0.00  0.00      PROA
ATOM    821  HD1 ILE P  53      27.425  16.471  -9.876  0.00  0.00      PROA
ATOM    822  HD2 ILE P  53      28.587  16.086 -11.222  0.00  0.00      PROA
ATOM    823  HD3 ILE P  53      29.020  15.609  -9.668  0.00  0.00      PROA
ATOM    824  C   ILE P  53      25.893  14.051 -13.581  0.00  0.00      PROA
ATOM    825  O   ILE P  53      25.179  14.615 -14.433  0.00  0.00      PROA
ATOM    826  N   VAL P  54      27.144  13.725 -13.949  0.00  0.00      PROA
ATOM    827  HN  VAL P  54      27.630  13.115 -13.328  0.00  0.00      PROA
ATOM    828  CA  VAL P  54      27.763  14.208 -15.162  0.00  0.00      PROA
ATOM    829  HA  VAL P  54      27.155  15.000 -15.574  0.00  0.00      PROA
ATOM    830  CB  VAL P  54      29.169  14.785 -14.870  0.00  0.00      PROA
ATOM    831  HB  VAL P  54      29.052  15.678 -14.219  0.00  0.00      PROA
ATOM    832  CG1 VAL P  54      30.067  13.753 -14.189  0.00  0.00      PROA
ATOM    833 HG11 VAL P  54      30.012  13.724 -13.080  0.00  0.00      PROA
ATOM    834 HG12 VAL P  54      31.071  14.097 -14.516  0.00  0.00      PROA
ATOM    835 HG13 VAL P  54      29.944  12.748 -14.646  0.00  0.00      PROA
ATOM    836  CG2 VAL P  54      29.752  15.375 -16.179  0.00  0.00      PROA
ATOM    837 HG21 VAL P  54      28.909  15.806 -16.759  0.00  0.00      PROA
ATOM    838 HG22 VAL P  54      30.124  14.481 -16.723  0.00  0.00      PROA
ATOM    839 HG23 VAL P  54      30.657  16.013 -16.085  0.00  0.00      PROA
ATOM    840  C   VAL P  54      27.768  13.063 -16.222  0.00  0.00      PROA
ATOM    841  O   VAL P  54      27.704  13.312 -17.437  0.00  0.00      PROA
ATOM    842  N   GLY P  55      27.886  11.790 -15.790  0.00  0.00      PROA
ATOM    843  HN  GLY P  55      28.170  11.601 -14.854  0.00  0.00      PROA
ATOM    844  CA  GLY P  55      27.660  10.677 -16.741  0.00  0.00      PROA
ATOM    845  HA1 GLY P  55      27.690   9.671 -16.350  0.00  0.00      PROA
ATOM    846  HA2 GLY P  55      28.275  10.817 -17.618  0.00  0.00      PROA
ATOM    847  C   GLY P  55      26.264  10.651 -17.339  0.00  0.00      PROA
ATOM    848  O   GLY P  55      26.208  10.772 -18.567  0.00  0.00      PROA
ATOM    849  N   ASN P  56      25.205  10.600 -16.531  0.00  0.00      PROA
ATOM    850  HN  ASN P  56      25.215  10.544 -15.536  0.00  0.00      PROA
```

ATOM	851	CA	ASN	P	56	23.805	10.777	-16.983	0.00	0.00	PROA
ATOM	852	HA	ASN	P	56	23.699	10.046	-17.771	0.00	0.00	PROA
ATOM	853	CB	ASN	P	56	22.776	10.429	-15.813	0.00	0.00	PROA
ATOM	854	HB1	ASN	P	56	21.749	10.814	-15.993	0.00	0.00	PROA
ATOM	855	HB2	ASN	P	56	23.068	10.929	-14.865	0.00	0.00	PROA
ATOM	856	CG	ASN	P	56	22.750	8.961	-15.465	0.00	0.00	PROA
ATOM	857	OD1	ASN	P	56	21.754	8.391	-15.989	0.00	0.00	PROA
ATOM	858	ND2	ASN	P	56	23.652	8.425	-14.640	0.00	0.00	PROA
ATOM	859	HD21	ASN	P	56	23.329	7.587	-14.201	0.00	0.00	PROA
ATOM	860	HD22	ASN	P	56	24.404	9.027	-14.371	0.00	0.00	PROA
ATOM	861	C	ASN	P	56	23.557	12.095	-17.668	0.00	0.00	PROA
ATOM	862	O	ASN	P	56	22.713	12.159	-18.546	0.00	0.00	PROA
ATOM	863	N	GLY	P	57	24.301	13.164	-17.379	0.00	0.00	PROA
ATOM	864	HN	GLY	P	57	24.986	12.984	-16.678	0.00	0.00	PROA
ATOM	865	CA	GLY	P	57	24.145	14.485	-17.937	0.00	0.00	PROA
ATOM	866	HA1	GLY	P	57	24.672	15.161	-17.279	0.00	0.00	PROA
ATOM	867	HA2	GLY	P	57	23.121	14.682	-18.215	0.00	0.00	PROA
ATOM	868	C	GLY	P	57	24.760	14.629	-19.319	0.00	0.00	PROA
ATOM	869	O	GLY	P	57	24.226	15.335	-20.153	0.00	0.00	PROA
ATOM	870	N	LEU	P	58	25.915	13.859	-19.608	0.00	0.00	PROA
ATOM	871	HN	LEU	P	58	26.412	13.432	-18.856	0.00	0.00	PROA
ATOM	872	CA	LEU	P	58	26.290	13.521	-20.941	0.00	0.00	PROA
ATOM	873	HA	LEU	P	58	26.334	14.417	-21.543	0.00	0.00	PROA
ATOM	874	CB	LEU	P	58	27.720	12.959	-20.873	0.00	0.00	PROA
ATOM	875	HB1	LEU	P	58	28.046	12.491	-21.826	0.00	0.00	PROA
ATOM	876	HB2	LEU	P	58	27.740	12.161	-20.101	0.00	0.00	PROA
ATOM	877	CG	LEU	P	58	28.759	14.037	-20.531	0.00	0.00	PROA
ATOM	878	HG	LEU	P	58	28.269	14.833	-19.930	0.00	0.00	PROA
ATOM	879	CD1	LEU	P	58	29.840	13.314	-19.697	0.00	0.00	PROA
ATOM	880	HD11	LEU	P	58	30.368	12.587	-20.350	0.00	0.00	PROA
ATOM	881	HD12	LEU	P	58	29.342	12.709	-18.908	0.00	0.00	PROA
ATOM	882	HD13	LEU	P	58	30.481	14.083	-19.216	0.00	0.00	PROA
ATOM	883	CD2	LEU	P	58	29.432	14.736	-21.713	0.00	0.00	PROA
ATOM	884	HD21	LEU	P	58	29.929	13.954	-22.326	0.00	0.00	PROA
ATOM	885	HD22	LEU	P	58	30.141	15.498	-21.324	0.00	0.00	PROA
ATOM	886	HD23	LEU	P	58	28.715	15.331	-22.319	0.00	0.00	PROA
ATOM	887	C	LEU	P	58	25.361	12.603	-21.742	0.00	0.00	PROA
ATOM	888	O	LEU	P	58	25.018	13.045	-22.818	0.00	0.00	PROA
ATOM	889	N	VAL	P	59	24.798	11.475	-21.189	0.00	0.00	PROA
ATOM	890	HN	VAL	P	59	25.143	11.312	-20.268	0.00	0.00	PROA
ATOM	891	CA	VAL	P	59	23.741	10.671	-21.876	0.00	0.00	PROA
ATOM	892	HA	VAL	P	59	24.124	10.367	-22.839	0.00	0.00	PROA
ATOM	893	CB	VAL	P	59	23.354	9.399	-21.156	0.00	0.00	PROA
ATOM	894	HB	VAL	P	59	23.033	9.727	-20.145	0.00	0.00	PROA
ATOM	895	CG1	VAL	P	59	22.290	8.547	-21.908	0.00	0.00	PROA
ATOM	896	HG11	VAL	P	59	21.908	7.773	-21.208	0.00	0.00	PROA
ATOM	897	HG12	VAL	P	59	22.743	8.185	-22.856	0.00	0.00	PROA
ATOM	898	HG13	VAL	P	59	21.329	9.076	-22.086	0.00	0.00	PROA
ATOM	899	CG2	VAL	P	59	24.591	8.581	-21.066	0.00	0.00	PROA
ATOM	900	HG21	VAL	P	59	25.374	8.955	-20.372	0.00	0.00	PROA
ATOM	901	HG22	VAL	P	59	25.080	8.610	-22.063	0.00	0.00	PROA
ATOM	902	HG23	VAL	P	59	24.343	7.506	-20.929	0.00	0.00	PROA
ATOM	903	C	VAL	P	59	22.470	11.424	-22.216	0.00	0.00	PROA
ATOM	904	O	VAL	P	59	21.966	11.366	-23.343	0.00	0.00	PROA
ATOM	905	N	ILE	P	60	21.894	12.266	-21.332	0.00	0.00	PROA
ATOM	906	HN	ILE	P	60	22.282	12.286	-20.415	0.00	0.00	PROA
ATOM	907	CA	ILE	P	60	20.707	13.057	-21.458	0.00	0.00	PROA
ATOM	908	HA	ILE	P	60	19.927	12.445	-21.885	0.00	0.00	PROA
ATOM	909	CB	ILE	P	60	20.200	13.622	-20.136	0.00	0.00	PROA
ATOM	910	HB	ILE	P	60	21.052	14.114	-19.621	0.00	0.00	PROA
ATOM	911	CG2	ILE	P	60	19.075	14.548	-20.256	0.00	0.00	PROA

ATOM	912	HG21	ILE	P	60	18.242	14.161	-20.882	0.00	0.00	PROA
ATOM	913	HG22	ILE	P	60	19.271	15.596	-20.567	0.00	0.00	PROA
ATOM	914	HG23	ILE	P	60	18.635	14.680	-19.244	0.00	0.00	PROA
ATOM	915	CG1	ILE	P	60	19.777	12.539	-19.078	0.00	0.00	PROA
ATOM	916	HG11	ILE	P	60	20.464	11.682	-19.240	0.00	0.00	PROA
ATOM	917	HG12	ILE	P	60	18.763	12.277	-19.450	0.00	0.00	PROA
ATOM	918	CD	ILE	P	60	19.798	12.872	-17.599	0.00	0.00	PROA
ATOM	919	HD1	ILE	P	60	20.817	13.183	-17.282	0.00	0.00	PROA
ATOM	920	HD2	ILE	P	60	19.408	12.056	-16.953	0.00	0.00	PROA
ATOM	921	HD3	ILE	P	60	19.000	13.594	-17.323	0.00	0.00	PROA
ATOM	922	C	ILE	P	60	20.814	14.120	-22.494	0.00	0.00	PROA
ATOM	923	O	ILE	P	60	19.883	14.360	-23.221	0.00	0.00	PROA
ATOM	924	N	LEU	P	61	22.027	14.743	-22.520	0.00	0.00	PROA
ATOM	925	HN	LEU	P	61	22.721	14.621	-21.815	0.00	0.00	PROA
ATOM	926	CA	LEU	P	61	22.434	15.616	-23.655	0.00	0.00	PROA
ATOM	927	HA	LEU	P	61	21.662	16.366	-23.757	0.00	0.00	PROA
ATOM	928	CB	LEU	P	61	23.787	16.350	-23.346	0.00	0.00	PROA
ATOM	929	HB1	LEU	P	61	24.433	15.528	-22.968	0.00	0.00	PROA
ATOM	930	HB2	LEU	P	61	23.525	17.023	-22.502	0.00	0.00	PROA
ATOM	931	CG	LEU	P	61	24.532	17.049	-24.516	0.00	0.00	PROA
ATOM	932	HG	LEU	P	61	24.668	16.306	-25.330	0.00	0.00	PROA
ATOM	933	CD1	LEU	P	61	23.722	18.256	-25.040	0.00	0.00	PROA
ATOM	934	HD11	LEU	P	61	24.167	18.784	-25.910	0.00	0.00	PROA
ATOM	935	HD12	LEU	P	61	23.823	18.933	-24.164	0.00	0.00	PROA
ATOM	936	HD13	LEU	P	61	22.679	17.935	-25.251	0.00	0.00	PROA
ATOM	937	CD2	LEU	P	61	25.945	17.513	-24.139	0.00	0.00	PROA
ATOM	938	HD21	LEU	P	61	26.551	17.758	-25.037	0.00	0.00	PROA
ATOM	939	HD22	LEU	P	61	26.540	16.741	-23.605	0.00	0.00	PROA
ATOM	940	HD23	LEU	P	61	25.830	18.329	-23.394	0.00	0.00	PROA
ATOM	941	C	LEU	P	61	22.556	14.956	-25.045	0.00	0.00	PROA
ATOM	942	O	LEU	P	61	22.066	15.482	-26.064	0.00	0.00	PROA
ATOM	943	N	VAL	P	62	23.080	13.756	-25.113	0.00	0.00	PROA
ATOM	944	HN	VAL	P	62	23.320	13.259	-24.282	0.00	0.00	PROA
ATOM	945	CA	VAL	P	62	23.269	13.052	-26.322	0.00	0.00	PROA
ATOM	946	HA	VAL	P	62	23.591	13.797	-27.035	0.00	0.00	PROA
ATOM	947	CB	VAL	P	62	24.272	11.848	-26.145	0.00	0.00	PROA
ATOM	948	HB	VAL	P	62	24.136	11.393	-25.141	0.00	0.00	PROA
ATOM	949	CG1	VAL	P	62	24.017	10.690	-27.126	0.00	0.00	PROA
ATOM	950	HG11	VAL	P	62	23.075	10.191	-26.815	0.00	0.00	PROA
ATOM	951	HG12	VAL	P	62	24.820	9.922	-27.106	0.00	0.00	PROA
ATOM	952	HG13	VAL	P	62	23.875	11.020	-28.178	0.00	0.00	PROA
ATOM	953	CG2	VAL	P	62	25.726	12.265	-26.232	0.00	0.00	PROA
ATOM	954	HG21	VAL	P	62	26.006	12.609	-27.251	0.00	0.00	PROA
ATOM	955	HG22	VAL	P	62	26.347	11.397	-25.922	0.00	0.00	PROA
ATOM	956	HG23	VAL	P	62	25.984	13.073	-25.514	0.00	0.00	PROA
ATOM	957	C	VAL	P	62	21.909	12.595	-26.825	0.00	0.00	PROA
ATOM	958	O	VAL	P	62	21.472	12.875	-27.956	0.00	0.00	PROA
ATOM	959	N	MET	P	63	21.148	11.873	-26.080	0.00	0.00	PROA
ATOM	960	HN	MET	P	63	21.477	11.744	-25.148	0.00	0.00	PROA
ATOM	961	CA	MET	P	63	19.847	11.283	-26.520	0.00	0.00	PROA
ATOM	962	HA	MET	P	63	19.999	10.721	-27.429	0.00	0.00	PROA
ATOM	963	CB	MET	P	63	19.175	10.187	-25.603	0.00	0.00	PROA
ATOM	964	HB1	MET	P	63	18.216	9.861	-26.061	0.00	0.00	PROA
ATOM	965	HB2	MET	P	63	18.897	10.712	-24.665	0.00	0.00	PROA
ATOM	966	CG	MET	P	63	19.997	8.889	-25.291	0.00	0.00	PROA
ATOM	967	HG1	MET	P	63	19.389	8.297	-24.573	0.00	0.00	PROA
ATOM	968	HG2	MET	P	63	20.978	9.123	-24.826	0.00	0.00	PROA
ATOM	969	SD	MET	P	63	20.475	7.946	-26.783	0.00	0.00	PROA
ATOM	970	CE	MET	P	63	21.062	6.424	-25.977	0.00	0.00	PROA
ATOM	971	HE1	MET	P	63	20.312	5.606	-25.932	0.00	0.00	PROA
ATOM	972	HE2	MET	P	63	21.429	6.699	-24.965	0.00	0.00	PROA

```
ATOM    973  HE3 MET P  63      21.949   5.981 -26.478  0.00  0.00      PROA
ATOM    974  C   MET P  63      18.731  12.348 -26.823  0.00  0.00      PROA
ATOM    975  O   MET P  63      17.897  12.260 -27.741  0.00  0.00      PROA
ATOM    976  N   GLY P  64      18.593  13.319 -25.958  0.00  0.00      PROA
ATOM    977  HN  GLY P  64      19.105  13.291 -25.103  0.00  0.00      PROA
ATOM    978  CA  GLY P  64      17.462  14.276 -25.960  0.00  0.00      PROA
ATOM    979  HA1 GLY P  64      17.412  14.575 -24.924  0.00  0.00      PROA
ATOM    980  HA2 GLY P  64      16.522  13.821 -26.235  0.00  0.00      PROA
ATOM    981  C   GLY P  64      17.624  15.460 -26.815  0.00  0.00      PROA
ATOM    982  O   GLY P  64      18.611  15.639 -27.588  0.00  0.00      PROA
ATOM    983  N   TYR P  65      16.722  16.455 -26.673  0.00  0.00      PROA
ATOM    984  HN  TYR P  65      15.836  16.280 -26.250  0.00  0.00      PROA
ATOM    985  CA  TYR P  65      16.734  17.840 -27.212  0.00  0.00      PROA
ATOM    986  HA  TYR P  65      15.869  18.322 -26.782  0.00  0.00      PROA
ATOM    987  CB  TYR P  65      17.869  18.795 -26.835  0.00  0.00      PROA
ATOM    988  HB1 TYR P  65      17.663  19.764 -27.337  0.00  0.00      PROA
ATOM    989  HB2 TYR P  65      18.874  18.440 -27.151  0.00  0.00      PROA
ATOM    990  CG  TYR P  65      17.950  18.949 -25.382  0.00  0.00      PROA
ATOM    991  CD1 TYR P  65      17.006  19.721 -24.715  0.00  0.00      PROA
ATOM    992  HD1 TYR P  65      16.284  20.308 -25.263  0.00  0.00      PROA
ATOM    993  CE1 TYR P  65      16.993  19.956 -23.301  0.00  0.00      PROA
ATOM    994  HE1 TYR P  65      16.165  20.467 -22.830  0.00  0.00      PROA
ATOM    995  CZ  TYR P  65      18.055  19.411 -22.609  0.00  0.00      PROA
ATOM    996  OH  TYR P  65      18.200  19.533 -21.221  0.00  0.00      PROA
ATOM    997  HH  TYR P  65      17.510  20.100 -20.870  0.00  0.00      PROA
ATOM    998  CD2 TYR P  65      19.071  18.552 -24.669  0.00  0.00      PROA
ATOM    999  HD2 TYR P  65      19.953  18.189 -25.175  0.00  0.00      PROA
ATOM   1000  CE2 TYR P  65      19.122  18.810 -23.304  0.00  0.00      PROA
ATOM   1001  HE2 TYR P  65      19.956  18.504 -22.690  0.00  0.00      PROA
ATOM   1002  C   TYR P  65      16.526  17.785 -28.736  0.00  0.00      PROA
ATOM   1003  O   TYR P  65      17.188  18.503 -29.513  0.00  0.00      PROA
ATOM   1004  N   GLN P  66      15.512  17.063 -29.185  0.00  0.00      PROA
ATOM   1005  HN  GLN P  66      14.959  16.481 -28.594  0.00  0.00      PROA
ATOM   1006  CA  GLN P  66      14.821  17.413 -30.425  0.00  0.00      PROA
ATOM   1007  HA  GLN P  66      13.905  16.849 -30.513  0.00  0.00      PROA
ATOM   1008  CB  GLN P  66      14.285  18.887 -30.516  0.00  0.00      PROA
ATOM   1009  HB1 GLN P  66      13.602  18.962 -31.389  0.00  0.00      PROA
ATOM   1010  HB2 GLN P  66      15.100  19.639 -30.455  0.00  0.00      PROA
ATOM   1011  CG  GLN P  66      13.538  19.184 -29.235  0.00  0.00      PROA
ATOM   1012  HG1 GLN P  66      14.185  19.024 -28.346  0.00  0.00      PROA
ATOM   1013  HG2 GLN P  66      12.591  18.630 -29.064  0.00  0.00      PROA
ATOM   1014  CD  GLN P  66      13.175  20.624 -29.227  0.00  0.00      PROA
ATOM   1015  OE1 GLN P  66      13.206  21.391 -30.173  0.00  0.00      PROA
ATOM   1016  NE2 GLN P  66      12.675  21.048 -28.004  0.00  0.00      PROA
ATOM   1017 HE21 GLN P  66      12.313  21.980 -27.997  0.00  0.00      PROA
ATOM   1018 HE22 GLN P  66      12.360  20.401 -27.309  0.00  0.00      PROA
ATOM   1019  C   GLN P  66      15.611  17.059 -31.689  0.00  0.00      PROA
ATOM   1020  O   GLN P  66      15.463  17.658 -32.723  0.00  0.00      PROA
ATOM   1021  N   LYS P  67      16.597  16.115 -31.540  0.00  0.00      PROA
ATOM   1022  HN  LYS P  67      16.592  15.735 -30.618  0.00  0.00      PROA
ATOM   1023  CA  LYS P  67      17.530  15.635 -32.522  0.00  0.00      PROA
ATOM   1024  HA  LYS P  67      17.935  16.502 -33.021  0.00  0.00      PROA
ATOM   1025  CB  LYS P  67      18.750  14.921 -31.813  0.00  0.00      PROA
ATOM   1026  HB1 LYS P  67      19.295  14.309 -32.564  0.00  0.00      PROA
ATOM   1027  HB2 LYS P  67      18.276  14.263 -31.054  0.00  0.00      PROA
ATOM   1028  CG  LYS P  67      19.757  15.926 -31.190  0.00  0.00      PROA
ATOM   1029  HG1 LYS P  67      19.140  16.401 -30.398  0.00  0.00      PROA
ATOM   1030  HG2 LYS P  67      20.030  16.692 -31.947  0.00  0.00      PROA
ATOM   1031  CD  LYS P  67      21.029  15.238 -30.642  0.00  0.00      PROA
ATOM   1032  HD1 LYS P  67      21.604  14.892 -31.528  0.00  0.00      PROA
ATOM   1033  HD2 LYS P  67      20.726  14.295 -30.140  0.00  0.00      PROA
```

ATOM	1034	CE	LYS	P	67	21.959	16.191	-29.808	0.00	0.00	PROA
ATOM	1035	HE1	LYS	P	67	22.043	17.166	-30.332	0.00	0.00	PROA
ATOM	1036	HE2	LYS	P	67	22.982	15.760	-29.852	0.00	0.00	PROA
ATOM	1037	NZ	LYS	P	67	21.427	16.336	-28.433	0.00	0.00	PROA
ATOM	1038	HZ1	LYS	P	67	20.391	16.261	-28.370	0.00	0.00	PROA
ATOM	1039	HZ2	LYS	P	67	21.687	17.285	-28.097	0.00	0.00	PROA
ATOM	1040	HZ3	LYS	P	67	21.798	15.707	-27.692	0.00	0.00	PROA
ATOM	1041	C	LYS	P	67	16.998	14.606	-33.490	0.00	0.00	PROA
ATOM	1042	O	LYS	P	67	16.040	13.881	-33.241	0.00	0.00	PROA
ATOM	1043	N	LYS	P	68	17.598	14.517	-34.719	0.00	0.00	PROA
ATOM	1044	HN	LYS	P	68	18.474	14.963	-34.885	0.00	0.00	PROA
ATOM	1045	CA	LYS	P	68	17.133	13.625	-35.786	0.00	0.00	PROA
ATOM	1046	HA	LYS	P	68	16.083	13.423	-35.634	0.00	0.00	PROA
ATOM	1047	CB	LYS	P	68	17.272	14.239	-37.244	0.00	0.00	PROA
ATOM	1048	HB1	LYS	P	68	16.686	13.648	-37.980	0.00	0.00	PROA
ATOM	1049	HB2	LYS	P	68	18.319	14.110	-37.592	0.00	0.00	PROA
ATOM	1050	CG	LYS	P	68	16.936	15.812	-37.427	0.00	0.00	PROA
ATOM	1051	HG1	LYS	P	68	17.163	16.092	-38.478	0.00	0.00	PROA
ATOM	1052	HG2	LYS	P	68	17.667	16.393	-36.825	0.00	0.00	PROA
ATOM	1053	CD	LYS	P	68	15.484	16.211	-37.140	0.00	0.00	PROA
ATOM	1054	HD1	LYS	P	68	15.275	15.874	-36.102	0.00	0.00	PROA
ATOM	1055	HD2	LYS	P	68	14.883	15.605	-37.852	0.00	0.00	PROA
ATOM	1056	CE	LYS	P	68	15.202	17.681	-37.245	0.00	0.00	PROA
ATOM	1057	HE1	LYS	P	68	15.299	18.025	-38.297	0.00	0.00	PROA
ATOM	1058	HE2	LYS	P	68	15.835	18.392	-36.672	0.00	0.00	PROA
ATOM	1059	NZ	LYS	P	68	13.809	18.014	-36.902	0.00	0.00	PROA
ATOM	1060	HZ1	LYS	P	68	13.181	17.217	-37.131	0.00	0.00	PROA
ATOM	1061	HZ2	LYS	P	68	13.489	18.873	-37.393	0.00	0.00	PROA
ATOM	1062	HZ3	LYS	P	68	13.676	18.175	-35.883	0.00	0.00	PROA
ATOM	1063	C	LYS	P	68	17.870	12.289	-35.621	0.00	0.00	PROA
ATOM	1064	O	LYS	P	68	18.885	12.137	-34.942	0.00	0.00	PROA
ATOM	1065	N	LEU	P	69	17.344	11.292	-36.375	0.00	0.00	PROA
ATOM	1066	HN	LEU	P	69	16.621	11.585	-36.996	0.00	0.00	PROA
ATOM	1067	CA	LEU	P	69	17.967	9.970	-36.460	0.00	0.00	PROA
ATOM	1068	HA	LEU	P	69	17.408	9.401	-37.189	0.00	0.00	PROA
ATOM	1069	CB	LEU	P	69	19.426	10.047	-37.108	0.00	0.00	PROA
ATOM	1070	HB1	LEU	P	69	19.721	8.976	-37.131	0.00	0.00	PROA
ATOM	1071	HB2	LEU	P	69	20.121	10.581	-36.426	0.00	0.00	PROA
ATOM	1072	CG	LEU	P	69	19.578	10.513	-38.555	0.00	0.00	PROA
ATOM	1073	HG	LEU	P	69	19.124	11.522	-38.651	0.00	0.00	PROA
ATOM	1074	CD1	LEU	P	69	21.074	10.819	-38.921	0.00	0.00	PROA
ATOM	1075	HD11	LEU	P	69	21.625	9.878	-39.131	0.00	0.00	PROA
ATOM	1076	HD12	LEU	P	69	21.455	11.296	-37.993	0.00	0.00	PROA
ATOM	1077	HD13	LEU	P	69	21.189	11.506	-39.787	0.00	0.00	PROA
ATOM	1078	CD2	LEU	P	69	18.944	9.563	-39.582	0.00	0.00	PROA
ATOM	1079	HD21	LEU	P	69	18.154	10.152	-40.095	0.00	0.00	PROA
ATOM	1080	HD22	LEU	P	69	18.489	8.641	-39.161	0.00	0.00	PROA
ATOM	1081	HD23	LEU	P	69	19.625	9.089	-40.320	0.00	0.00	PROA
ATOM	1082	C	LEU	P	69	17.898	9.153	-35.204	0.00	0.00	PROA
ATOM	1083	O	LEU	P	69	18.837	8.472	-34.736	0.00	0.00	PROA
ATOM	1084	N	ARG	P	70	16.743	9.207	-34.596	0.00	0.00	PROA
ATOM	1085	HN	ARG	P	70	15.989	9.731	-34.985	0.00	0.00	PROA
ATOM	1086	CA	ARG	P	70	16.667	8.741	-33.195	0.00	0.00	PROA
ATOM	1087	HA	ARG	P	70	17.632	8.560	-32.744	0.00	0.00	PROA
ATOM	1088	CB	ARG	P	70	16.085	9.752	-32.130	0.00	0.00	PROA
ATOM	1089	HB1	ARG	P	70	16.008	9.241	-31.147	0.00	0.00	PROA
ATOM	1090	HB2	ARG	P	70	15.139	10.194	-32.511	0.00	0.00	PROA
ATOM	1091	CG	ARG	P	70	17.050	10.909	-31.894	0.00	0.00	PROA
ATOM	1092	HG1	ARG	P	70	17.077	11.689	-32.685	0.00	0.00	PROA
ATOM	1093	HG2	ARG	P	70	18.072	10.505	-31.729	0.00	0.00	PROA
ATOM	1094	CD	ARG	P	70	16.811	11.699	-30.577	0.00	0.00	PROA

```
ATOM   1095  HD1 ARG P  70      17.613  12.430 -30.340  0.00  0.00      PROA
ATOM   1096  HD2 ARG P  70      16.688  10.963 -29.754  0.00  0.00      PROA
ATOM   1097  NE  ARG P  70      15.513  12.367 -30.739  0.00  0.00      PROA
ATOM   1098  HE  ARG P  70      15.129  12.325 -31.662  0.00  0.00      PROA
ATOM   1099  CZ  ARG P  70      14.818  13.045 -29.899  0.00  0.00      PROA
ATOM   1100  NH1 ARG P  70      15.300  13.031 -28.672  0.00  0.00      PROA
ATOM   1101 HH11 ARG P  70      16.106  12.439 -28.692  0.00  0.00      PROA
ATOM   1102 HH12 ARG P  70      14.747  13.346 -27.901  0.00  0.00      PROA
ATOM   1103  NH2 ARG P  70      13.630  13.592 -30.220  0.00  0.00      PROA
ATOM   1104 HH21 ARG P  70      13.324  13.456 -31.163  0.00  0.00      PROA
ATOM   1105 HH22 ARG P  70      12.975  13.815 -29.499  0.00  0.00      PROA
ATOM   1106  C   ARG P  70      15.731   7.485 -33.254  0.00  0.00      PROA
ATOM   1107  O   ARG P  70      14.892   7.422 -34.093  0.00  0.00      PROA
ATOM   1108  N   SER P  71      16.008   6.460 -32.386  0.00  0.00      PROA
ATOM   1109  HN  SER P  71      16.736   6.600 -31.719  0.00  0.00      PROA
ATOM   1110  CA  SER P  71      15.172   5.278 -32.256  0.00  0.00      PROA
ATOM   1111  HA  SER P  71      14.583   5.266 -33.162  0.00  0.00      PROA
ATOM   1112  CB  SER P  71      15.992   3.938 -32.150  0.00  0.00      PROA
ATOM   1113  HB1 SER P  71      16.673   3.784 -33.013  0.00  0.00      PROA
ATOM   1114  HB2 SER P  71      15.317   3.057 -32.196  0.00  0.00      PROA
ATOM   1115  OG  SER P  71      16.698   3.828 -30.944  0.00  0.00      PROA
ATOM   1116  HG1 SER P  71      17.437   4.437 -31.005  0.00  0.00      PROA
ATOM   1117  C   SER P  71      14.224   5.476 -31.102  0.00  0.00      PROA
ATOM   1118  O   SER P  71      14.399   6.407 -30.341  0.00  0.00      PROA
ATOM   1119  N   MET P  72      13.224   4.548 -30.912  0.00  0.00      PROA
ATOM   1120  HN  MET P  72      13.057   3.893 -31.645  0.00  0.00      PROA
ATOM   1121  CA  MET P  72      12.513   4.385 -29.685  0.00  0.00      PROA
ATOM   1122  HA  MET P  72      12.162   5.360 -29.379  0.00  0.00      PROA
ATOM   1123  CB  MET P  72      11.339   3.383 -29.622  0.00  0.00      PROA
ATOM   1124  HB1 MET P  72      10.974   3.253 -28.581  0.00  0.00      PROA
ATOM   1125  HB2 MET P  72      11.619   2.378 -30.003  0.00  0.00      PROA
ATOM   1126  CG  MET P  72      10.184   3.671 -30.618  0.00  0.00      PROA
ATOM   1127  HG1 MET P  72       9.390   2.924 -30.402  0.00  0.00      PROA
ATOM   1128  HG2 MET P  72      10.508   3.680 -31.681  0.00  0.00      PROA
ATOM   1129  SD  MET P  72       9.595   5.383 -30.411  0.00  0.00      PROA
ATOM   1130  CE  MET P  72       8.680   5.162 -28.894  0.00  0.00      PROA
ATOM   1131  HE1 MET P  72       7.844   4.432 -28.938  0.00  0.00      PROA
ATOM   1132  HE2 MET P  72       8.219   6.140 -28.640  0.00  0.00      PROA
ATOM   1133  HE3 MET P  72       9.345   4.896 -28.045  0.00  0.00      PROA
ATOM   1134  C   MET P  72      13.414   4.006 -28.509  0.00  0.00      PROA
ATOM   1135  O   MET P  72      13.154   4.570 -27.457  0.00  0.00      PROA
ATOM   1136  N   THR P  73      14.448   3.182 -28.736  0.00  0.00      PROA
ATOM   1137  HN  THR P  73      14.546   2.723 -29.616  0.00  0.00      PROA
ATOM   1138  CA  THR P  73      15.424   2.761 -27.799  0.00  0.00      PROA
ATOM   1139  HA  THR P  73      14.886   2.367 -26.949  0.00  0.00      PROA
ATOM   1140  CB  THR P  73      16.433   1.679 -28.314  0.00  0.00      PROA
ATOM   1141  HB  THR P  73      16.657   1.874 -29.384  0.00  0.00      PROA
ATOM   1142  OG1 THR P  73      15.850   0.438 -28.263  0.00  0.00      PROA
ATOM   1143  HG1 THR P  73      15.094   0.548 -28.844  0.00  0.00      PROA
ATOM   1144  CG2 THR P  73      17.742   1.658 -27.575  0.00  0.00      PROA
ATOM   1145 HG21 THR P  73      18.277   0.700 -27.749  0.00  0.00      PROA
ATOM   1146 HG22 THR P  73      17.443   1.592 -26.508  0.00  0.00      PROA
ATOM   1147 HG23 THR P  73      18.340   2.580 -27.737  0.00  0.00      PROA
ATOM   1148  C   THR P  73      16.166   3.966 -27.174  0.00  0.00      PROA
ATOM   1149  O   THR P  73      16.245   4.138 -26.006  0.00  0.00      PROA
ATOM   1150  N   ASP P  74      16.579   4.930 -28.016  0.00  0.00      PROA
ATOM   1151  HN  ASP P  74      16.612   4.858 -29.010  0.00  0.00      PROA
ATOM   1152  CA  ASP P  74      17.117   6.204 -27.575  0.00  0.00      PROA
ATOM   1153  HA  ASP P  74      18.036   6.053 -27.028  0.00  0.00      PROA
ATOM   1154  CB  ASP P  74      17.419   7.041 -28.811  0.00  0.00      PROA
ATOM   1155  HB1 ASP P  74      17.903   8.006 -28.549  0.00  0.00      PROA
```

ATOM 1156 HB2 ASP P 74 16.492 7.103 -29.420 0.00 0.00 PROA
ATOM 1157 CG ASP P 74 18.297 6.572 -29.929 0.00 0.00 PROA
ATOM 1158 OD1 ASP P 74 18.859 5.464 -29.890 0.00 0.00 PROA
ATOM 1159 OD2 ASP P 74 18.399 7.248 -31.019 0.00 0.00 PROA
ATOM 1160 C ASP P 74 16.267 7.024 -26.620 0.00 0.00 PROA
ATOM 1161 O ASP P 74 16.772 7.657 -25.724 0.00 0.00 PROA
ATOM 1162 N LYS P 75 15.007 7.060 -26.866 0.00 0.00 PROA
ATOM 1163 HN LYS P 75 14.594 6.597 -27.647 0.00 0.00 PROA
ATOM 1164 CA LYS P 75 13.934 7.628 -26.022 0.00 0.00 PROA
ATOM 1165 HA LYS P 75 14.220 8.606 -25.663 0.00 0.00 PROA
ATOM 1166 CB LYS P 75 12.601 7.847 -26.726 0.00 0.00 PROA
ATOM 1167 HB1 LYS P 75 12.043 8.402 -25.942 0.00 0.00 PROA
ATOM 1168 HB2 LYS P 75 12.147 6.861 -26.962 0.00 0.00 PROA
ATOM 1169 CG LYS P 75 12.757 8.660 -28.033 0.00 0.00 PROA
ATOM 1170 HG1 LYS P 75 13.487 8.127 -28.677 0.00 0.00 PROA
ATOM 1171 HG2 LYS P 75 13.280 9.596 -27.743 0.00 0.00 PROA
ATOM 1172 CD LYS P 75 11.514 8.998 -28.909 0.00 0.00 PROA
ATOM 1173 HD1 LYS P 75 10.923 9.762 -28.361 0.00 0.00 PROA
ATOM 1174 HD2 LYS P 75 10.946 8.087 -29.196 0.00 0.00 PROA
ATOM 1175 CE LYS P 75 11.874 9.726 -30.242 0.00 0.00 PROA
ATOM 1176 HE1 LYS P 75 12.509 9.154 -30.951 0.00 0.00 PROA
ATOM 1177 HE2 LYS P 75 12.404 10.669 -29.988 0.00 0.00 PROA
ATOM 1178 NZ LYS P 75 10.719 10.135 -31.030 0.00 0.00 PROA
ATOM 1179 HZ1 LYS P 75 10.095 9.424 -31.462 0.00 0.00 PROA
ATOM 1180 HZ2 LYS P 75 11.127 10.662 -31.828 0.00 0.00 PROA
ATOM 1181 HZ3 LYS P 75 10.223 10.830 -30.436 0.00 0.00 PROA
ATOM 1182 C LYS P 75 13.777 6.741 -24.742 0.00 0.00 PROA
ATOM 1183 O LYS P 75 13.615 7.256 -23.657 0.00 0.00 PROA
ATOM 1184 N TYR P 76 13.855 5.391 -24.889 0.00 0.00 PROA
ATOM 1185 HN TYR P 76 13.856 4.938 -25.777 0.00 0.00 PROA
ATOM 1186 CA TYR P 76 13.861 4.537 -23.737 0.00 0.00 PROA
ATOM 1187 HA TYR P 76 13.015 4.813 -23.124 0.00 0.00 PROA
ATOM 1188 CB TYR P 76 13.755 2.974 -24.140 0.00 0.00 PROA
ATOM 1189 HB1 TYR P 76 13.928 2.359 -23.231 0.00 0.00 PROA
ATOM 1190 HB2 TYR P 76 14.573 2.849 -24.881 0.00 0.00 PROA
ATOM 1191 CG TYR P 76 12.427 2.611 -24.759 0.00 0.00 PROA
ATOM 1192 CD1 TYR P 76 11.185 3.250 -24.466 0.00 0.00 PROA
ATOM 1193 HD1 TYR P 76 11.201 4.000 -23.689 0.00 0.00 PROA
ATOM 1194 CE1 TYR P 76 10.063 2.986 -25.217 0.00 0.00 PROA
ATOM 1195 HE1 TYR P 76 9.186 3.560 -24.956 0.00 0.00 PROA
ATOM 1196 CZ TYR P 76 10.129 2.005 -26.188 0.00 0.00 PROA
ATOM 1197 OH TYR P 76 9.069 1.756 -26.933 0.00 0.00 PROA
ATOM 1198 HH TYR P 76 8.601 2.551 -27.199 0.00 0.00 PROA
ATOM 1199 CD2 TYR P 76 12.412 1.598 -25.711 0.00 0.00 PROA
ATOM 1200 HD2 TYR P 76 13.340 1.242 -26.134 0.00 0.00 PROA
ATOM 1201 CE2 TYR P 76 11.309 1.326 -26.473 0.00 0.00 PROA
ATOM 1202 HE2 TYR P 76 11.255 0.584 -27.256 0.00 0.00 PROA
ATOM 1203 C TYR P 76 15.052 4.775 -22.746 0.00 0.00 PROA
ATOM 1204 O TYR P 76 14.893 4.877 -21.537 0.00 0.00 PROA
ATOM 1205 N ARG P 77 16.255 4.956 -23.289 0.00 0.00 PROA
ATOM 1206 HN ARG P 77 16.337 4.921 -24.282 0.00 0.00 PROA
ATOM 1207 CA ARG P 77 17.499 5.344 -22.595 0.00 0.00 PROA
ATOM 1208 HA ARG P 77 17.684 4.625 -21.811 0.00 0.00 PROA
ATOM 1209 CB ARG P 77 18.796 5.118 -23.493 0.00 0.00 PROA
ATOM 1210 HB1 ARG P 77 19.697 5.434 -22.925 0.00 0.00 PROA
ATOM 1211 HB2 ARG P 77 18.790 5.642 -24.473 0.00 0.00 PROA
ATOM 1212 CG ARG P 77 19.012 3.580 -23.855 0.00 0.00 PROA
ATOM 1213 HG1 ARG P 77 19.794 3.657 -24.639 0.00 0.00 PROA
ATOM 1214 HG2 ARG P 77 18.122 3.295 -24.455 0.00 0.00 PROA
ATOM 1215 CD ARG P 77 19.347 2.642 -22.675 0.00 0.00 PROA
ATOM 1216 HD1 ARG P 77 19.633 1.633 -23.040 0.00 0.00 PROA

```
ATOM   1217  HD2 ARG P  77      18.517   2.589 -21.938  0.00  0.00      PROA
ATOM   1218  NE  ARG P  77      20.580   3.161 -22.048  0.00  0.00      PROA
ATOM   1219  HE  ARG P  77      20.530   3.922 -21.401  0.00  0.00      PROA
ATOM   1220  CZ  ARG P  77      21.800   2.530 -22.030  0.00  0.00      PROA
ATOM   1221  NH1 ARG P  77      22.169   1.726 -22.984  0.00  0.00      PROA
ATOM   1222 HH11 ARG P  77      21.623   1.745 -23.822  0.00  0.00      PROA
ATOM   1223 HH12 ARG P  77      22.998   1.176 -22.886  0.00  0.00      PROA
ATOM   1224  NH2 ARG P  77      22.670   2.703 -21.004  0.00  0.00      PROA
ATOM   1225 HH21 ARG P  77      22.345   3.195 -20.196  0.00  0.00      PROA
ATOM   1226 HH22 ARG P  77      23.525   2.195 -21.109  0.00  0.00      PROA
ATOM   1227  C   ARG P  77      17.473   6.755 -21.990  0.00  0.00      PROA
ATOM   1228  O   ARG P  77      17.958   6.972 -20.902  0.00  0.00      PROA
ATOM   1229  N   LEU P  78      16.855   7.733 -22.652  0.00  0.00      PROA
ATOM   1230  HN  LEU P  78      16.636   7.528 -23.603  0.00  0.00      PROA
ATOM   1231  CA  LEU P  78      16.573   9.074 -22.113  0.00  0.00      PROA
ATOM   1232  HA  LEU P  78      17.497   9.563 -21.842  0.00  0.00      PROA
ATOM   1233  CB  LEU P  78      15.743   9.930 -23.169  0.00  0.00      PROA
ATOM   1234  HB1 LEU P  78      14.797   9.371 -23.330  0.00  0.00      PROA
ATOM   1235  HB2 LEU P  78      16.278   9.949 -24.142  0.00  0.00      PROA
ATOM   1236  CG  LEU P  78      15.424  11.319 -22.689  0.00  0.00      PROA
ATOM   1237  HG  LEU P  78      14.784  11.159 -21.796  0.00  0.00      PROA
ATOM   1238  CD1 LEU P  78      16.653  12.128 -22.224  0.00  0.00      PROA
ATOM   1239 HD11 LEU P  78      17.029  11.848 -21.217  0.00  0.00      PROA
ATOM   1240 HD12 LEU P  78      16.387  13.206 -22.258  0.00  0.00      PROA
ATOM   1241 HD13 LEU P  78      17.499  12.009 -22.934  0.00  0.00      PROA
ATOM   1242  CD2 LEU P  78      14.752  12.137 -23.760  0.00  0.00      PROA
ATOM   1243 HD21 LEU P  78      14.034  11.459 -24.269  0.00  0.00      PROA
ATOM   1244 HD22 LEU P  78      15.417  12.422 -24.603  0.00  0.00      PROA
ATOM   1245 HD23 LEU P  78      14.301  13.030 -23.276  0.00  0.00      PROA
ATOM   1246  C   LEU P  78      15.703   9.021 -20.840  0.00  0.00      PROA
ATOM   1247  O   LEU P  78      16.075   9.610 -19.882  0.00  0.00      PROA
ATOM   1248  N   HSD P  79      14.634   8.277 -20.779  0.00  0.00      PROA
ATOM   1249  HN  HSD P  79      14.108   7.959 -21.564  0.00  0.00      PROA
ATOM   1250  CA  HSD P  79      13.823   8.050 -19.542  0.00  0.00      PROA
ATOM   1251  HA  HSD P  79      13.576   9.013 -19.119  0.00  0.00      PROA
ATOM   1252  CB  HSD P  79      12.518   7.196 -19.814  0.00  0.00      PROA
ATOM   1253  HB1 HSD P  79      12.691   6.150 -20.147  0.00  0.00      PROA
ATOM   1254  HB2 HSD P  79      11.904   7.639 -20.627  0.00  0.00      PROA
ATOM   1255  ND1 HSD P  79      11.722   6.598 -17.474  0.00  0.00      PROA
ATOM   1256  HD1 HSD P  79      12.579   6.242 -17.100  0.00  0.00      PROA
ATOM   1257  CG  HSD P  79      11.488   7.137 -18.721  0.00  0.00      PROA
ATOM   1258  CE1 HSD P  79      10.554   6.718 -16.818  0.00  0.00      PROA
ATOM   1259  HE1 HSD P  79      10.476   6.245 -15.839  0.00  0.00      PROA
ATOM   1260  NE2 HSD P  79       9.618   7.215 -17.524  0.00  0.00      PROA
ATOM   1261  CD2 HSD P  79      10.188   7.435 -18.789  0.00  0.00      PROA
ATOM   1262  HD2 HSD P  79       9.521   7.897 -19.507  0.00  0.00      PROA
ATOM   1263  C   HSD P  79      14.641   7.426 -18.463  0.00  0.00      PROA
ATOM   1264  O   HSD P  79      14.605   7.904 -17.341  0.00  0.00      PROA
ATOM   1265  N   LEU P  80      15.385   6.399 -18.873  0.00  0.00      PROA
ATOM   1266  HN  LEU P  80      15.530   6.333 -19.857  0.00  0.00      PROA
ATOM   1267  CA  LEU P  80      16.232   5.518 -18.102  0.00  0.00      PROA
ATOM   1268  HA  LEU P  80      15.523   5.096 -17.404  0.00  0.00      PROA
ATOM   1269  CB  LEU P  80      16.890   4.369 -18.860  0.00  0.00      PROA
ATOM   1270  HB1 LEU P  80      17.536   4.774 -19.668  0.00  0.00      PROA
ATOM   1271  HB2 LEU P  80      16.096   3.769 -19.353  0.00  0.00      PROA
ATOM   1272  CG  LEU P  80      17.702   3.366 -18.030  0.00  0.00      PROA
ATOM   1273  HG  LEU P  80      18.437   3.942 -17.429  0.00  0.00      PROA
ATOM   1274  CD1 LEU P  80      16.835   2.651 -16.957  0.00  0.00      PROA
ATOM   1275 HD11 LEU P  80      16.422   3.325 -16.176  0.00  0.00      PROA
ATOM   1276 HD12 LEU P  80      17.367   1.878 -16.361  0.00  0.00      PROA
ATOM   1277 HD13 LEU P  80      16.018   2.167 -17.533  0.00  0.00      PROA
```

```
ATOM   1278  CD2 LEU P  80      18.374   2.278 -18.857  0.00  0.00      PROA
ATOM   1279 HD21 LEU P  80      17.634   1.578 -19.300  0.00  0.00      PROA
ATOM   1280 HD22 LEU P  80      19.037   1.689 -18.188  0.00  0.00      PROA
ATOM   1281 HD23 LEU P  80      18.980   2.664 -19.705  0.00  0.00      PROA
ATOM   1282  C   LEU P  80      17.293   6.277 -17.308  0.00  0.00      PROA
ATOM   1283  O   LEU P  80      17.398   6.274 -16.080  0.00  0.00      PROA
ATOM   1284  N   SER P  81      18.077   7.071 -18.080  0.00  0.00      PROA
ATOM   1285  HN  SER P  81      18.050   6.984 -19.073  0.00  0.00      PROA
ATOM   1286  CA  SER P  81      18.960   8.127 -17.485  0.00  0.00      PROA
ATOM   1287  HA  SER P  81      19.560   7.705 -16.693  0.00  0.00      PROA
ATOM   1288  CB  SER P  81      19.963   8.749 -18.447  0.00  0.00      PROA
ATOM   1289  HB1 SER P  81      20.326   9.788 -18.292  0.00  0.00      PROA
ATOM   1290  HB2 SER P  81      19.403   8.731 -19.407  0.00  0.00      PROA
ATOM   1291  OG  SER P  81      21.188   8.007 -18.720  0.00  0.00      PROA
ATOM   1292  HG1 SER P  81      21.536   7.959 -17.827  0.00  0.00      PROA
ATOM   1293  C   SER P  81      18.296   9.256 -16.664  0.00  0.00      PROA
ATOM   1294  O   SER P  81      18.876   9.724 -15.716  0.00  0.00      PROA
ATOM   1295  N   VAL P  82      17.103   9.860 -17.019  0.00  0.00      PROA
ATOM   1296  HN  VAL P  82      16.644   9.546 -17.847  0.00  0.00      PROA
ATOM   1297  CA  VAL P  82      16.416  10.867 -16.173  0.00  0.00      PROA
ATOM   1298  HA  VAL P  82      17.171  11.571 -15.854  0.00  0.00      PROA
ATOM   1299  CB  VAL P  82      15.310  11.479 -17.085  0.00  0.00      PROA
ATOM   1300  HB  VAL P  82      14.734  10.624 -17.498  0.00  0.00      PROA
ATOM   1301  CG1 VAL P  82      14.397  12.406 -16.271  0.00  0.00      PROA
ATOM   1302 HG11 VAL P  82      13.800  11.849 -15.517  0.00  0.00      PROA
ATOM   1303 HG12 VAL P  82      13.712  12.950 -16.957  0.00  0.00      PROA
ATOM   1304 HG13 VAL P  82      14.941  13.205 -15.723  0.00  0.00      PROA
ATOM   1305  CG2 VAL P  82      16.064  12.359 -18.061  0.00  0.00      PROA
ATOM   1306 HG21 VAL P  82      16.801  11.803 -18.679  0.00  0.00      PROA
ATOM   1307 HG22 VAL P  82      16.587  13.175 -17.518  0.00  0.00      PROA
ATOM   1308 HG23 VAL P  82      15.331  12.771 -18.787  0.00  0.00      PROA
ATOM   1309  C   VAL P  82      15.886  10.274 -14.925  0.00  0.00      PROA
ATOM   1310  O   VAL P  82      15.987  10.846 -13.843  0.00  0.00      PROA
ATOM   1311  N   ALA P  83      15.330   9.016 -15.000  0.00  0.00      PROA
ATOM   1312  HN  ALA P  83      15.134   8.658 -15.909  0.00  0.00      PROA
ATOM   1313  CA  ALA P  83      14.930   8.226 -13.802  0.00  0.00      PROA
ATOM   1314  HA  ALA P  83      14.275   8.902 -13.272  0.00  0.00      PROA
ATOM   1315  CB  ALA P  83      14.163   7.019 -14.293  0.00  0.00      PROA
ATOM   1316  HB1 ALA P  83      13.312   7.378 -14.910  0.00  0.00      PROA
ATOM   1317  HB2 ALA P  83      13.792   6.362 -13.477  0.00  0.00      PROA
ATOM   1318  HB3 ALA P  83      14.727   6.414 -15.035  0.00  0.00      PROA
ATOM   1319  C   ALA P  83      16.042   7.902 -12.857  0.00  0.00      PROA
ATOM   1320  O   ALA P  83      15.875   8.217 -11.700  0.00  0.00      PROA
ATOM   1321  N   ASP P  84      17.180   7.336 -13.367  0.00  0.00      PROA
ATOM   1322  HN  ASP P  84      17.207   6.863 -14.244  0.00  0.00      PROA
ATOM   1323  CA  ASP P  84      18.387   7.089 -12.549  0.00  0.00      PROA
ATOM   1324  HA  ASP P  84      18.126   6.514 -11.673  0.00  0.00      PROA
ATOM   1325  CB  ASP P  84      19.450   6.282 -13.367  0.00  0.00      PROA
ATOM   1326  HB1 ASP P  84      20.362   6.245 -12.733  0.00  0.00      PROA
ATOM   1327  HB2 ASP P  84      19.611   6.717 -14.377  0.00  0.00      PROA
ATOM   1328  CG  ASP P  84      19.176   4.786 -13.468  0.00  0.00      PROA
ATOM   1329  OD1 ASP P  84      18.430   4.302 -12.627  0.00  0.00      PROA
ATOM   1330  OD2 ASP P  84      19.841   4.119 -14.340  0.00  0.00      PROA
ATOM   1331  C   ASP P  84      18.889   8.349 -12.012  0.00  0.00      PROA
ATOM   1332  O   ASP P  84      19.277   8.291 -10.842  0.00  0.00      PROA
ATOM   1333  N   LEU P  85      18.874   9.477 -12.717  0.00  0.00      PROA
ATOM   1334  HN  LEU P  85      18.645   9.440 -13.686  0.00  0.00      PROA
ATOM   1335  CA  LEU P  85      19.283  10.740 -12.149  0.00  0.00      PROA
ATOM   1336  HA  LEU P  85      20.254  10.555 -11.716  0.00  0.00      PROA
ATOM   1337  CB  LEU P  85      19.388  11.840 -13.353  0.00  0.00      PROA
ATOM   1338  HB1 LEU P  85      18.389  11.892 -13.839  0.00  0.00      PROA
```

ATOM 1339 HB2 LEU P 85 20.099 11.446 -14.110 0.00 0.00 PROA
ATOM 1340 CG LEU P 85 19.957 13.223 -12.957 0.00 0.00 PROA
ATOM 1341 HG LEU P 85 19.259 13.755 -12.276 0.00 0.00 PROA
ATOM 1342 CD1 LEU P 85 21.234 13.029 -12.161 0.00 0.00 PROA
ATOM 1343 HD11 LEU P 85 21.586 14.049 -11.896 0.00 0.00 PROA
ATOM 1344 HD12 LEU P 85 22.016 12.610 -12.829 0.00 0.00 PROA
ATOM 1345 HD13 LEU P 85 21.023 12.533 -11.189 0.00 0.00 PROA
ATOM 1346 CD2 LEU P 85 20.199 14.045 -14.212 0.00 0.00 PROA
ATOM 1347 HD21 LEU P 85 20.695 15.031 -14.084 0.00 0.00 PROA
ATOM 1348 HD22 LEU P 85 19.230 14.284 -14.698 0.00 0.00 PROA
ATOM 1349 HD23 LEU P 85 20.770 13.547 -15.024 0.00 0.00 PROA
ATOM 1350 C LEU P 85 18.379 11.220 -10.981 0.00 0.00 PROA
ATOM 1351 O LEU P 85 18.794 11.761 -9.958 0.00 0.00 PROA
ATOM 1352 N LEU P 86 17.041 11.036 -11.101 0.00 0.00 PROA
ATOM 1353 HN LEU P 86 16.540 10.803 -11.931 0.00 0.00 PROA
ATOM 1354 CA LEU P 86 16.154 11.310 -9.938 0.00 0.00 PROA
ATOM 1355 HA LEU P 86 16.449 12.279 -9.562 0.00 0.00 PROA
ATOM 1356 CB LEU P 86 14.657 11.268 -10.275 0.00 0.00 PROA
ATOM 1357 HB1 LEU P 86 14.336 10.205 -10.314 0.00 0.00 PROA
ATOM 1358 HB2 LEU P 86 14.515 11.650 -11.308 0.00 0.00 PROA
ATOM 1359 CG LEU P 86 13.681 12.006 -9.310 0.00 0.00 PROA
ATOM 1360 HG LEU P 86 13.767 11.699 -8.246 0.00 0.00 PROA
ATOM 1361 CD1 LEU P 86 13.755 13.479 -9.322 0.00 0.00 PROA
ATOM 1362 HD11 LEU P 86 12.968 13.956 -8.700 0.00 0.00 PROA
ATOM 1363 HD12 LEU P 86 13.672 13.913 -10.341 0.00 0.00 PROA
ATOM 1364 HD13 LEU P 86 14.737 13.774 -8.893 0.00 0.00 PROA
ATOM 1365 CD2 LEU P 86 12.280 11.519 -9.755 0.00 0.00 PROA
ATOM 1366 HD21 LEU P 86 12.307 10.418 -9.897 0.00 0.00 PROA
ATOM 1367 HD22 LEU P 86 11.753 12.062 -10.568 0.00 0.00 PROA
ATOM 1368 HD23 LEU P 86 11.674 11.624 -8.830 0.00 0.00 PROA
ATOM 1369 C LEU P 86 16.409 10.452 -8.702 0.00 0.00 PROA
ATOM 1370 O LEU P 86 16.288 10.922 -7.560 0.00 0.00 PROA
ATOM 1371 N PHE P 87 16.881 9.126 -8.882 0.00 0.00 PROA
ATOM 1372 HN PHE P 87 16.868 8.751 -9.805 0.00 0.00 PROA
ATOM 1373 CA PHE P 87 17.384 8.362 -7.781 0.00 0.00 PROA
ATOM 1374 HA PHE P 87 16.715 8.529 -6.950 0.00 0.00 PROA
ATOM 1375 CB PHE P 87 17.395 6.797 -8.238 0.00 0.00 PROA
ATOM 1376 HB1 PHE P 87 17.736 6.829 -9.295 0.00 0.00 PROA
ATOM 1377 HB2 PHE P 87 16.381 6.373 -8.397 0.00 0.00 PROA
ATOM 1378 CG PHE P 87 18.265 5.983 -7.350 0.00 0.00 PROA
ATOM 1379 CD1 PHE P 87 17.898 5.588 -6.066 0.00 0.00 PROA
ATOM 1380 HD1 PHE P 87 17.023 5.985 -5.572 0.00 0.00 PROA
ATOM 1381 CE1 PHE P 87 18.804 4.939 -5.223 0.00 0.00 PROA
ATOM 1382 HE1 PHE P 87 18.523 4.579 -4.244 0.00 0.00 PROA
ATOM 1383 CZ PHE P 87 20.070 4.652 -5.641 0.00 0.00 PROA
ATOM 1384 HZ PHE P 87 20.741 4.203 -4.924 0.00 0.00 PROA
ATOM 1385 CD2 PHE P 87 19.506 5.617 -7.814 0.00 0.00 PROA
ATOM 1386 HD2 PHE P 87 19.745 5.767 -8.856 0.00 0.00 PROA
ATOM 1387 CE2 PHE P 87 20.414 4.923 -6.955 0.00 0.00 PROA
ATOM 1388 HE2 PHE P 87 21.432 4.785 -7.288 0.00 0.00 PROA
ATOM 1389 C PHE P 87 18.739 8.936 -7.288 0.00 0.00 PROA
ATOM 1390 O PHE P 87 18.936 9.124 -6.106 0.00 0.00 PROA
ATOM 1391 N VAL P 88 19.575 9.400 -8.225 0.00 0.00 PROA
ATOM 1392 HN VAL P 88 19.292 9.328 -9.178 0.00 0.00 PROA
ATOM 1393 CA VAL P 88 20.903 9.845 -7.740 0.00 0.00 PROA
ATOM 1394 HA VAL P 88 21.237 9.228 -6.919 0.00 0.00 PROA
ATOM 1395 CB VAL P 88 21.889 9.755 -8.899 0.00 0.00 PROA
ATOM 1396 HB VAL P 88 21.307 9.911 -9.832 0.00 0.00 PROA
ATOM 1397 CG1 VAL P 88 22.999 10.816 -8.994 0.00 0.00 PROA
ATOM 1398 HG11 VAL P 88 22.528 11.800 -8.784 0.00 0.00 PROA
ATOM 1399 HG12 VAL P 88 23.398 10.860 -10.030 0.00 0.00 PROA

ATOM 1400 HG13 VAL P 88 23.785 10.602 -8.238 0.00 0.00 PROA
ATOM 1401 CG2 VAL P 88 22.424 8.320 -8.971 0.00 0.00 PROA
ATOM 1402 HG21 VAL P 88 21.716 7.464 -8.965 0.00 0.00 PROA
ATOM 1403 HG22 VAL P 88 23.010 8.141 -8.044 0.00 0.00 PROA
ATOM 1404 HG23 VAL P 88 23.178 8.154 -9.770 0.00 0.00 PROA
ATOM 1405 C VAL P 88 21.014 11.162 -7.016 0.00 0.00 PROA
ATOM 1406 O VAL P 88 21.708 11.345 -6.001 0.00 0.00 PROA
ATOM 1407 N ILE P 89 20.114 12.094 -7.400 0.00 0.00 PROA
ATOM 1408 HN ILE P 89 19.478 11.936 -8.151 0.00 0.00 PROA
ATOM 1409 CA ILE P 89 19.760 13.285 -6.539 0.00 0.00 PROA
ATOM 1410 HA ILE P 89 20.698 13.786 -6.348 0.00 0.00 PROA
ATOM 1411 CB ILE P 89 18.764 14.098 -7.201 0.00 0.00 PROA
ATOM 1412 HB ILE P 89 17.950 13.435 -7.564 0.00 0.00 PROA
ATOM 1413 CG2 ILE P 89 18.162 15.184 -6.309 0.00 0.00 PROA
ATOM 1414 HG21 ILE P 89 18.948 15.751 -5.766 0.00 0.00 PROA
ATOM 1415 HG22 ILE P 89 17.545 14.625 -5.572 0.00 0.00 PROA
ATOM 1416 HG23 ILE P 89 17.461 15.852 -6.853 0.00 0.00 PROA
ATOM 1417 CG1 ILE P 89 19.613 14.882 -8.327 0.00 0.00 PROA
ATOM 1418 HG11 ILE P 89 18.919 15.551 -8.879 0.00 0.00 PROA
ATOM 1419 HG12 ILE P 89 19.946 14.017 -8.940 0.00 0.00 PROA
ATOM 1420 CD ILE P 89 20.744 15.729 -8.002 0.00 0.00 PROA
ATOM 1421 HD1 ILE P 89 21.084 16.554 -8.663 0.00 0.00 PROA
ATOM 1422 HD2 ILE P 89 21.672 15.130 -7.882 0.00 0.00 PROA
ATOM 1423 HD3 ILE P 89 20.599 16.200 -7.006 0.00 0.00 PROA
ATOM 1424 C ILE P 89 19.316 12.848 -5.113 0.00 0.00 PROA
ATOM 1425 O ILE P 89 19.546 13.631 -4.178 0.00 0.00 PROA
ATOM 1426 N THR P 90 18.629 11.695 -4.880 0.00 0.00 PROA
ATOM 1427 HN THR P 90 18.575 10.902 -5.481 0.00 0.00 PROA
ATOM 1428 CA THR P 90 18.266 11.322 -3.504 0.00 0.00 PROA
ATOM 1429 HA THR P 90 17.988 12.193 -2.929 0.00 0.00 PROA
ATOM 1430 CB THR P 90 17.000 10.415 -3.400 0.00 0.00 PROA
ATOM 1431 HB THR P 90 16.815 10.297 -2.311 0.00 0.00 PROA
ATOM 1432 OG1 THR P 90 17.143 9.148 -4.011 0.00 0.00 PROA
ATOM 1433 HG1 THR P 90 17.496 9.268 -4.896 0.00 0.00 PROA
ATOM 1434 CG2 THR P 90 15.757 11.107 -3.982 0.00 0.00 PROA
ATOM 1435 HG21 THR P 90 15.887 11.164 -5.084 0.00 0.00 PROA
ATOM 1436 HG22 THR P 90 15.552 12.136 -3.616 0.00 0.00 PROA
ATOM 1437 HG23 THR P 90 14.825 10.511 -3.881 0.00 0.00 PROA
ATOM 1438 C THR P 90 19.358 10.644 -2.674 0.00 0.00 PROA
ATOM 1439 O THR P 90 19.165 10.432 -1.470 0.00 0.00 PROA
ATOM 1440 N LEU P 91 20.528 10.220 -3.219 0.00 0.00 PROA
ATOM 1441 HN LEU P 91 20.645 10.425 -4.188 0.00 0.00 PROA
ATOM 1442 CA LEU P 91 21.538 9.428 -2.502 0.00 0.00 PROA
ATOM 1443 HA LEU P 91 20.975 8.549 -2.226 0.00 0.00 PROA
ATOM 1444 CB LEU P 91 22.666 8.901 -3.434 0.00 0.00 PROA
ATOM 1445 HB1 LEU P 91 23.596 8.720 -2.855 0.00 0.00 PROA
ATOM 1446 HB2 LEU P 91 22.965 9.648 -4.199 0.00 0.00 PROA
ATOM 1447 CG LEU P 91 22.279 7.608 -4.297 0.00 0.00 PROA
ATOM 1448 HG LEU P 91 21.336 7.839 -4.837 0.00 0.00 PROA
ATOM 1449 CD1 LEU P 91 23.380 7.281 -5.332 0.00 0.00 PROA
ATOM 1450 HD11 LEU P 91 24.428 7.266 -4.964 0.00 0.00 PROA
ATOM 1451 HD12 LEU P 91 23.405 8.203 -5.950 0.00 0.00 PROA
ATOM 1452 HD13 LEU P 91 23.228 6.468 -6.073 0.00 0.00 PROA
ATOM 1453 CD2 LEU P 91 21.885 6.498 -3.334 0.00 0.00 PROA
ATOM 1454 HD21 LEU P 91 21.829 5.553 -3.916 0.00 0.00 PROA
ATOM 1455 HD22 LEU P 91 20.870 6.633 -2.904 0.00 0.00 PROA
ATOM 1456 HD23 LEU P 91 22.607 6.425 -2.494 0.00 0.00 PROA
ATOM 1457 C LEU P 91 22.142 10.043 -1.215 0.00 0.00 PROA
ATOM 1458 O LEU P 91 22.310 9.329 -0.255 0.00 0.00 PROA
ATOM 1459 N PRO P 92 22.471 11.384 -1.121 0.00 0.00 PROA
ATOM 1460 CD PRO P 92 22.736 12.236 -2.299 0.00 0.00 PROA

ATOM	1461	HD1	PRO	P	92	21.997	12.054	-3.108	0.00	0.00	PROA
ATOM	1462	HD2	PRO	P	92	23.733	11.881	-2.637	0.00	0.00	PROA
ATOM	1463	CA	PRO	P	92	22.732	12.057	0.136	0.00	0.00	PROA
ATOM	1464	HA	PRO	P	92	23.750	11.963	0.487	0.00	0.00	PROA
ATOM	1465	CB	PRO	P	92	22.532	13.544	-0.269	0.00	0.00	PROA
ATOM	1466	HB1	PRO	P	92	23.162	14.142	0.424	0.00	0.00	PROA
ATOM	1467	HB2	PRO	P	92	21.442	13.744	-0.188	0.00	0.00	PROA
ATOM	1468	CG	PRO	P	92	22.825	13.691	-1.815	0.00	0.00	PROA
ATOM	1469	HG1	PRO	P	92	22.026	14.409	-2.100	0.00	0.00	PROA
ATOM	1470	HG2	PRO	P	92	23.795	14.186	-2.037	0.00	0.00	PROA
ATOM	1471	C	PRO	P	92	21.823	11.682	1.312	0.00	0.00	PROA
ATOM	1472	O	PRO	P	92	22.362	11.475	2.391	0.00	0.00	PROA
ATOM	1473	N	PHE	P	93	20.548	11.432	1.063	0.00	0.00	PROA
ATOM	1474	HN	PHE	P	93	20.055	11.535	0.203	0.00	0.00	PROA
ATOM	1475	CA	PHE	P	93	19.612	11.017	2.122	0.00	0.00	PROA
ATOM	1476	HA	PHE	P	93	19.709	11.798	2.862	0.00	0.00	PROA
ATOM	1477	CB	PHE	P	93	18.110	11.033	1.687	0.00	0.00	PROA
ATOM	1478	HB1	PHE	P	93	17.458	10.563	2.454	0.00	0.00	PROA
ATOM	1479	HB2	PHE	P	93	18.009	10.428	0.761	0.00	0.00	PROA
ATOM	1480	CG	PHE	P	93	17.686	12.507	1.465	0.00	0.00	PROA
ATOM	1481	CD1	PHE	P	93	17.958	13.071	0.198	0.00	0.00	PROA
ATOM	1482	HD1	PHE	P	93	18.424	12.511	-0.598	0.00	0.00	PROA
ATOM	1483	CE1	PHE	P	93	17.629	14.425	-0.010	0.00	0.00	PROA
ATOM	1484	HE1	PHE	P	93	17.886	14.886	-0.952	0.00	0.00	PROA
ATOM	1485	CZ	PHE	P	93	16.917	15.105	0.984	0.00	0.00	PROA
ATOM	1486	HZ	PHE	P	93	16.518	16.091	0.797	0.00	0.00	PROA
ATOM	1487	CD2	PHE	P	93	17.182	13.250	2.489	0.00	0.00	PROA
ATOM	1488	HD2	PHE	P	93	17.087	12.884	3.500	0.00	0.00	PROA
ATOM	1489	CE2	PHE	P	93	16.713	14.547	2.238	0.00	0.00	PROA
ATOM	1490	HE2	PHE	P	93	16.110	15.108	2.936	0.00	0.00	PROA
ATOM	1491	C	PHE	P	93	19.878	9.566	2.630	0.00	0.00	PROA
ATOM	1492	O	PHE	P	93	20.008	9.355	3.856	0.00	0.00	PROA
ATOM	1493	N	TRP	P	94	20.273	8.614	1.731	0.00	0.00	PROA
ATOM	1494	HN	TRP	P	94	20.232	8.815	0.755	0.00	0.00	PROA
ATOM	1495	CA	TRP	P	94	20.956	7.370	2.148	0.00	0.00	PROA
ATOM	1496	HA	TRP	P	94	20.381	6.903	2.934	0.00	0.00	PROA
ATOM	1497	CB	TRP	P	94	21.093	6.371	0.893	0.00	0.00	PROA
ATOM	1498	HB1	TRP	P	94	21.451	5.380	1.245	0.00	0.00	PROA
ATOM	1499	HB2	TRP	P	94	21.867	6.694	0.165	0.00	0.00	PROA
ATOM	1500	CG	TRP	P	94	19.869	6.038	0.080	0.00	0.00	PROA
ATOM	1501	CD1	TRP	P	94	19.149	6.785	-0.788	0.00	0.00	PROA
ATOM	1502	HD1	TRP	P	94	19.250	7.794	-1.162	0.00	0.00	PROA
ATOM	1503	NE1	TRP	P	94	18.102	5.994	-1.224	0.00	0.00	PROA
ATOM	1504	HE1	TRP	P	94	17.395	6.400	-1.759	0.00	0.00	PROA
ATOM	1505	CE2	TRP	P	94	18.049	4.820	-0.580	0.00	0.00	PROA
ATOM	1506	CD2	TRP	P	94	19.232	4.785	0.156	0.00	0.00	PROA
ATOM	1507	CE3	TRP	P	94	19.402	3.584	0.966	0.00	0.00	PROA
ATOM	1508	HE3	TRP	P	94	20.279	3.500	1.591	0.00	0.00	PROA
ATOM	1509	CZ3	TRP	P	94	18.587	2.489	0.696	0.00	0.00	PROA
ATOM	1510	HZ3	TRP	P	94	18.907	1.597	1.214	0.00	0.00	PROA
ATOM	1511	CZ2	TRP	P	94	17.110	3.848	-0.565	0.00	0.00	PROA
ATOM	1512	HZ2	TRP	P	94	16.161	3.932	-1.075	0.00	0.00	PROA
ATOM	1513	CH2	TRP	P	94	17.437	2.555	-0.085	0.00	0.00	PROA
ATOM	1514	HH2	TRP	P	94	16.627	1.844	-0.152	0.00	0.00	PROA
ATOM	1515	C	TRP	P	94	22.292	7.614	2.806	0.00	0.00	PROA
ATOM	1516	O	TRP	P	94	22.627	6.889	3.749	0.00	0.00	PROA
ATOM	1517	N	ALA	P	95	23.130	8.522	2.231	0.00	0.00	PROA
ATOM	1518	HN	ALA	P	95	22.952	9.057	1.408	0.00	0.00	PROA
ATOM	1519	CA	ALA	P	95	24.430	8.907	2.822	0.00	0.00	PROA
ATOM	1520	HA	ALA	P	95	24.857	7.916	2.838	0.00	0.00	PROA
ATOM	1521	CB	ALA	P	95	25.234	9.829	1.862	0.00	0.00	PROA

ATOM 1522 HB1 ALA P 95 24.984 10.893 2.062 0.00 0.00 PROA
ATOM 1523 HB2 ALA P 95 24.964 9.635 0.802 0.00 0.00 PROA
ATOM 1524 HB3 ALA P 95 26.317 9.699 2.073 0.00 0.00 PROA
ATOM 1525 C ALA P 95 24.515 9.436 4.253 0.00 0.00 PROA
ATOM 1526 O ALA P 95 25.306 9.056 5.103 0.00 0.00 PROA
ATOM 1527 N VAL P 96 23.539 10.281 4.573 0.00 0.00 PROA
ATOM 1528 HN VAL P 96 22.913 10.447 3.816 0.00 0.00 PROA
ATOM 1529 CA VAL P 96 23.306 10.851 5.911 0.00 0.00 PROA
ATOM 1530 HA VAL P 96 24.290 11.034 6.318 0.00 0.00 PROA
ATOM 1531 CB VAL P 96 22.409 12.043 5.812 0.00 0.00 PROA
ATOM 1532 HB VAL P 96 21.491 11.770 5.248 0.00 0.00 PROA
ATOM 1533 CG1 VAL P 96 21.894 12.525 7.207 0.00 0.00 PROA
ATOM 1534 HG11 VAL P 96 21.323 13.470 7.085 0.00 0.00 PROA
ATOM 1535 HG12 VAL P 96 22.776 12.648 7.871 0.00 0.00 PROA
ATOM 1536 HG13 VAL P 96 21.101 11.893 7.662 0.00 0.00 PROA
ATOM 1537 CG2 VAL P 96 23.065 13.169 5.013 0.00 0.00 PROA
ATOM 1538 HG21 VAL P 96 22.394 14.038 5.182 0.00 0.00 PROA
ATOM 1539 HG22 VAL P 96 23.067 12.970 3.920 0.00 0.00 PROA
ATOM 1540 HG23 VAL P 96 23.998 13.591 5.443 0.00 0.00 PROA
ATOM 1541 C VAL P 96 22.833 9.754 6.925 0.00 0.00 PROA
ATOM 1542 O VAL P 96 23.191 9.762 8.076 0.00 0.00 PROA
ATOM 1543 N ASP P 97 21.849 8.848 6.494 0.00 0.00 PROA
ATOM 1544 HN ASP P 97 21.630 8.892 5.523 0.00 0.00 PROA
ATOM 1545 CA ASP P 97 21.222 7.773 7.265 0.00 0.00 PROA
ATOM 1546 HA ASP P 97 20.966 8.269 8.189 0.00 0.00 PROA
ATOM 1547 CB ASP P 97 19.984 7.222 6.540 0.00 0.00 PROA
ATOM 1548 HB1 ASP P 97 20.301 6.704 5.610 0.00 0.00 PROA
ATOM 1549 HB2 ASP P 97 19.463 8.106 6.114 0.00 0.00 PROA
ATOM 1550 CG ASP P 97 19.113 6.300 7.349 0.00 0.00 PROA
ATOM 1551 OD1 ASP P 97 19.079 6.388 8.576 0.00 0.00 PROA
ATOM 1552 OD2 ASP P 97 18.305 5.646 6.711 0.00 0.00 PROA
ATOM 1553 C ASP P 97 22.222 6.677 7.611 0.00 0.00 PROA
ATOM 1554 O ASP P 97 22.156 6.089 8.672 0.00 0.00 PROA
ATOM 1555 N ALA P 98 23.188 6.549 6.735 0.00 0.00 PROA
ATOM 1556 HN ALA P 98 23.197 6.868 5.791 0.00 0.00 PROA
ATOM 1557 CA ALA P 98 24.344 5.722 7.080 0.00 0.00 PROA
ATOM 1558 HA ALA P 98 24.014 4.748 7.410 0.00 0.00 PROA
ATOM 1559 CB ALA P 98 25.119 5.384 5.737 0.00 0.00 PROA
ATOM 1560 HB1 ALA P 98 26.121 4.938 5.917 0.00 0.00 PROA
ATOM 1561 HB2 ALA P 98 25.337 6.297 5.143 0.00 0.00 PROA
ATOM 1562 HB3 ALA P 98 24.589 4.659 5.083 0.00 0.00 PROA
ATOM 1563 C ALA P 98 25.314 6.313 8.109 0.00 0.00 PROA
ATOM 1564 O ALA P 98 26.152 5.600 8.686 0.00 0.00 PROA
ATOM 1565 N VAL P 99 25.357 7.634 8.343 0.00 0.00 PROA
ATOM 1566 HN VAL P 99 24.622 8.158 7.918 0.00 0.00 PROA
ATOM 1567 CA VAL P 99 26.416 8.270 9.077 0.00 0.00 PROA
ATOM 1568 HA VAL P 99 27.196 7.583 9.368 0.00 0.00 PROA
ATOM 1569 CB VAL P 99 27.219 9.346 8.250 0.00 0.00 PROA
ATOM 1570 HB VAL P 99 26.525 10.145 7.912 0.00 0.00 PROA
ATOM 1571 CG1 VAL P 99 28.253 10.111 9.114 0.00 0.00 PROA
ATOM 1572 HG11 VAL P 99 29.111 9.431 9.302 0.00 0.00 PROA
ATOM 1573 HG12 VAL P 99 27.872 10.501 10.082 0.00 0.00 PROA
ATOM 1574 HG13 VAL P 99 28.678 10.968 8.549 0.00 0.00 PROA
ATOM 1575 CG2 VAL P 99 27.864 8.641 7.105 0.00 0.00 PROA
ATOM 1576 HG21 VAL P 99 28.585 7.851 7.406 0.00 0.00 PROA
ATOM 1577 HG22 VAL P 99 28.346 9.379 6.428 0.00 0.00 PROA
ATOM 1578 HG23 VAL P 99 27.059 8.206 6.475 0.00 0.00 PROA
ATOM 1579 C VAL P 99 25.950 8.935 10.293 0.00 0.00 PROA
ATOM 1580 O VAL P 99 26.590 8.814 11.396 0.00 0.00 PROA
ATOM 1581 N ALA P 100 24.789 9.660 10.300 0.00 0.00 PROA
ATOM 1582 HN ALA P 100 24.238 9.606 9.471 0.00 0.00 PROA

ATOM	1583	CA	ALA	P	100	24.401	10.597	11.391	0.00	0.00	PROA
ATOM	1584	HA	ALA	P	100	25.077	10.550	12.232	0.00	0.00	PROA
ATOM	1585	CB	ALA	P	100	24.405	12.017	10.753	0.00	0.00	PROA
ATOM	1586	HB1	ALA	P	100	25.457	12.318	10.559	0.00	0.00	PROA
ATOM	1587	HB2	ALA	P	100	23.992	12.692	11.532	0.00	0.00	PROA
ATOM	1588	HB3	ALA	P	100	23.830	12.273	9.837	0.00	0.00	PROA
ATOM	1589	C	ALA	P	100	22.910	10.235	11.871	0.00	0.00	PROA
ATOM	1590	O	ALA	P	100	22.712	9.171	12.448	0.00	0.00	PROA
ATOM	1591	N	ASN	P	101	21.879	11.109	11.515	0.00	0.00	PROA
ATOM	1592	HN	ASN	P	101	22.026	11.929	10.968	0.00	0.00	PROA
ATOM	1593	CA	ASN	P	101	20.544	10.993	12.080	0.00	0.00	PROA
ATOM	1594	HA	ASN	P	101	20.487	9.934	12.283	0.00	0.00	PROA
ATOM	1595	CB	ASN	P	101	20.551	11.817	13.395	0.00	0.00	PROA
ATOM	1596	HB1	ASN	P	101	20.989	12.821	13.210	0.00	0.00	PROA
ATOM	1597	HB2	ASN	P	101	21.250	11.332	14.109	0.00	0.00	PROA
ATOM	1598	CG	ASN	P	101	19.223	11.948	14.090	0.00	0.00	PROA
ATOM	1599	OD1	ASN	P	101	18.230	11.252	13.903	0.00	0.00	PROA
ATOM	1600	ND2	ASN	P	101	19.143	12.969	15.019	0.00	0.00	PROA
ATOM	1601	HD21	ASN	P	101	18.432	12.985	15.721	0.00	0.00	PROA
ATOM	1602	HD22	ASN	P	101	19.795	13.727	15.031	0.00	0.00	PROA
ATOM	1603	C	ASN	P	101	19.629	11.425	10.943	0.00	0.00	PROA
ATOM	1604	O	ASN	P	101	20.226	11.875	9.928	0.00	0.00	PROA
ATOM	1605	N	TRP	P	102	18.307	11.374	11.060	0.00	0.00	PROA
ATOM	1606	HN	TRP	P	102	17.801	11.224	11.906	0.00	0.00	PROA
ATOM	1607	CA	TRP	P	102	17.419	11.772	10.064	0.00	0.00	PROA
ATOM	1608	HA	TRP	P	102	17.945	11.873	9.127	0.00	0.00	PROA
ATOM	1609	CB	TRP	P	102	16.252	10.707	9.965	0.00	0.00	PROA
ATOM	1610	HB1	TRP	P	102	15.829	10.600	10.987	0.00	0.00	PROA
ATOM	1611	HB2	TRP	P	102	16.677	9.716	9.697	0.00	0.00	PROA
ATOM	1612	CG	TRP	P	102	15.158	10.843	8.995	0.00	0.00	PROA
ATOM	1613	CD1	TRP	P	102	13.975	11.494	9.160	0.00	0.00	PROA
ATOM	1614	HD1	TRP	P	102	13.427	11.693	10.069	0.00	0.00	PROA
ATOM	1615	NE1	TRP	P	102	13.423	11.675	7.890	0.00	0.00	PROA
ATOM	1616	HE1	TRP	P	102	12.619	12.193	7.694	0.00	0.00	PROA
ATOM	1617	CE2	TRP	P	102	14.283	11.176	6.921	0.00	0.00	PROA
ATOM	1618	CD2	TRP	P	102	15.382	10.654	7.607	0.00	0.00	PROA
ATOM	1619	CE3	TRP	P	102	16.401	9.968	6.925	0.00	0.00	PROA
ATOM	1620	HE3	TRP	P	102	17.154	9.548	7.576	0.00	0.00	PROA
ATOM	1621	CZ3	TRP	P	102	16.494	10.217	5.557	0.00	0.00	PROA
ATOM	1622	HZ3	TRP	P	102	17.414	9.937	5.065	0.00	0.00	PROA
ATOM	1623	CZ2	TRP	P	102	14.248	11.172	5.560	0.00	0.00	PROA
ATOM	1624	HZ2	TRP	P	102	13.394	11.665	5.120	0.00	0.00	PROA
ATOM	1625	CH2	TRP	P	102	15.399	10.819	4.915	0.00	0.00	PROA
ATOM	1626	HH2	TRP	P	102	15.444	10.910	3.840	0.00	0.00	PROA
ATOM	1627	C	TRP	P	102	16.921	13.165	10.528	0.00	0.00	PROA
ATOM	1628	O	TRP	P	102	16.198	13.325	11.565	0.00	0.00	PROA
ATOM	1629	N	TYR	P	103	17.184	14.196	9.737	0.00	0.00	PROA
ATOM	1630	HN	TYR	P	103	17.711	14.116	8.895	0.00	0.00	PROA
ATOM	1631	CA	TYR	P	103	16.888	15.581	10.069	0.00	0.00	PROA
ATOM	1632	HA	TYR	P	103	16.599	15.774	11.092	0.00	0.00	PROA
ATOM	1633	CB	TYR	P	103	18.234	16.371	9.804	0.00	0.00	PROA
ATOM	1634	HB1	TYR	P	103	18.140	17.423	10.148	0.00	0.00	PROA
ATOM	1635	HB2	TYR	P	103	18.440	16.362	8.712	0.00	0.00	PROA
ATOM	1636	CG	TYR	P	103	19.377	15.852	10.538	0.00	0.00	PROA
ATOM	1637	CD1	TYR	P	103	19.410	16.101	11.955	0.00	0.00	PROA
ATOM	1638	HD1	TYR	P	103	18.539	16.504	12.450	0.00	0.00	PROA
ATOM	1639	CE1	TYR	P	103	20.513	15.663	12.610	0.00	0.00	PROA
ATOM	1640	HE1	TYR	P	103	20.586	15.821	13.676	0.00	0.00	PROA
ATOM	1641	CZ	TYR	P	103	21.626	15.125	11.945	0.00	0.00	PROA
ATOM	1642	OH	TYR	P	103	22.830	14.708	12.666	0.00	0.00	PROA
ATOM	1643	HH	TYR	P	103	22.562	14.523	13.569	0.00	0.00	PROA

ATOM	1644	CD2	TYR	P	103	20.392	15.267	9.877	0.00	0.00	PROA
ATOM	1645	HD2	TYR	P	103	20.366	15.092	8.812	0.00	0.00	PROA
ATOM	1646	CE2	TYR	P	103	21.518	14.903	10.583	0.00	0.00	PROA
ATOM	1647	HE2	TYR	P	103	22.243	14.285	10.075	0.00	0.00	PROA
ATOM	1648	C	TYR	P	103	15.797	16.154	9.179	0.00	0.00	PROA
ATOM	1649	O	TYR	P	103	15.685	17.345	8.914	0.00	0.00	PROA
ATOM	1650	N	PHE	P	104	15.036	15.287	8.566	0.00	0.00	PROA
ATOM	1651	HN	PHE	P	104	15.048	14.368	8.953	0.00	0.00	PROA
ATOM	1652	CA	PHE	P	104	14.269	15.588	7.317	0.00	0.00	PROA
ATOM	1653	HA	PHE	P	104	14.427	16.591	6.949	0.00	0.00	PROA
ATOM	1654	CB	PHE	P	104	14.931	14.741	6.223	0.00	0.00	PROA
ATOM	1655	HB1	PHE	P	104	14.571	15.063	5.223	0.00	0.00	PROA
ATOM	1656	HB2	PHE	P	104	14.659	13.666	6.166	0.00	0.00	PROA
ATOM	1657	CG	PHE	P	104	16.398	14.924	6.133	0.00	0.00	PROA
ATOM	1658	CD1	PHE	P	104	16.964	16.200	5.845	0.00	0.00	PROA
ATOM	1659	HD1	PHE	P	104	16.216	16.979	5.844	0.00	0.00	PROA
ATOM	1660	CE1	PHE	P	104	18.344	16.318	5.578	0.00	0.00	PROA
ATOM	1661	HE1	PHE	P	104	18.787	17.272	5.332	0.00	0.00	PROA
ATOM	1662	CZ	PHE	P	104	19.229	15.317	5.833	0.00	0.00	PROA
ATOM	1663	HZ	PHE	P	104	20.292	15.488	5.753	0.00	0.00	PROA
ATOM	1664	CD2	PHE	P	104	17.329	13.855	6.223	0.00	0.00	PROA
ATOM	1665	HD2	PHE	P	104	16.948	12.853	6.356	0.00	0.00	PROA
ATOM	1666	CE2	PHE	P	104	18.705	14.019	6.140	0.00	0.00	PROA
ATOM	1667	HE2	PHE	P	104	19.323	13.145	6.281	0.00	0.00	PROA
ATOM	1668	C	PHE	P	104	12.745	15.324	7.382	0.00	0.00	PROA
ATOM	1669	O	PHE	P	104	12.032	15.549	6.396	0.00	0.00	PROA
ATOM	1670	N	GLY	P	105	12.192	14.962	8.556	0.00	0.00	PROA
ATOM	1671	HN	GLY	P	105	12.820	14.869	9.324	0.00	0.00	PROA
ATOM	1672	CA	GLY	P	105	10.775	14.664	8.951	0.00	0.00	PROA
ATOM	1673	HA1	GLY	P	105	10.220	15.585	8.844	0.00	0.00	PROA
ATOM	1674	HA2	GLY	P	105	10.780	14.326	9.976	0.00	0.00	PROA
ATOM	1675	C	GLY	P	105	10.209	13.657	8.051	0.00	0.00	PROA
ATOM	1676	O	GLY	P	105	10.797	12.820	7.317	0.00	0.00	PROA
ATOM	1677	N	ASN	P	106	8.858	13.598	8.210	0.00	0.00	PROA
ATOM	1678	HN	ASN	P	106	8.419	14.215	8.859	0.00	0.00	PROA
ATOM	1679	CA	ASN	P	106	7.955	12.629	7.622	0.00	0.00	PROA
ATOM	1680	HA	ASN	P	106	8.483	11.699	7.770	0.00	0.00	PROA
ATOM	1681	CB	ASN	P	106	6.717	12.833	8.459	0.00	0.00	PROA
ATOM	1682	HB1	ASN	P	106	5.885	12.407	7.859	0.00	0.00	PROA
ATOM	1683	HB2	ASN	P	106	6.469	13.888	8.704	0.00	0.00	PROA
ATOM	1684	CG	ASN	P	106	6.804	12.056	9.822	0.00	0.00	PROA
ATOM	1685	OD1	ASN	P	106	6.177	11.004	9.958	0.00	0.00	PROA
ATOM	1686	ND2	ASN	P	106	7.366	12.691	10.841	0.00	0.00	PROA
ATOM	1687	HD21	ASN	P	106	7.444	12.170	11.691	0.00	0.00	PROA
ATOM	1688	HD22	ASN	P	106	7.674	13.639	10.758	0.00	0.00	PROA
ATOM	1689	C	ASN	P	106	7.758	12.766	6.133	0.00	0.00	PROA
ATOM	1690	O	ASN	P	106	7.769	11.832	5.396	0.00	0.00	PROA
ATOM	1691	N	PHE	P	107	7.483	14.043	5.652	0.00	0.00	PROA
ATOM	1692	HN	PHE	P	107	7.455	14.756	6.348	0.00	0.00	PROA
ATOM	1693	CA	PHE	P	107	7.146	14.285	4.203	0.00	0.00	PROA
ATOM	1694	HA	PHE	P	107	6.268	13.713	3.944	0.00	0.00	PROA
ATOM	1695	CB	PHE	P	107	6.623	15.831	4.026	0.00	0.00	PROA
ATOM	1696	HB1	PHE	P	107	7.455	16.555	4.166	0.00	0.00	PROA
ATOM	1697	HB2	PHE	P	107	5.787	16.183	4.668	0.00	0.00	PROA
ATOM	1698	CG	PHE	P	107	6.022	16.030	2.609	0.00	0.00	PROA
ATOM	1699	CD1	PHE	P	107	6.776	16.660	1.606	0.00	0.00	PROA
ATOM	1700	HD1	PHE	P	107	7.761	17.078	1.751	0.00	0.00	PROA
ATOM	1701	CE1	PHE	P	107	6.141	16.933	0.329	0.00	0.00	PROA
ATOM	1702	HE1	PHE	P	107	6.789	17.428	-0.379	0.00	0.00	PROA
ATOM	1703	CZ	PHE	P	107	4.855	16.581	0.110	0.00	0.00	PROA
ATOM	1704	HZ	PHE	P	107	4.345	16.807	-0.815	0.00	0.00	PROA

ATOM	1705	CD2	PHE	P	107	4.709	15.616	2.332	0.00	0.00	PROA
ATOM	1706	HD2	PHE	P	107	4.290	15.074	3.166	0.00	0.00	PROA
ATOM	1707	CE2	PHE	P	107	4.186	15.869	1.062	0.00	0.00	PROA
ATOM	1708	HE2	PHE	P	107	3.159	15.568	0.911	0.00	0.00	PROA
ATOM	1709	C	PHE	P	107	8.285	13.925	3.284	0.00	0.00	PROA
ATOM	1710	O	PHE	P	107	8.089	13.227	2.269	0.00	0.00	PROA
ATOM	1711	N	LEU	P	108	9.533	14.215	3.593	0.00	0.00	PROA
ATOM	1712	HN	LEU	P	108	9.616	14.823	4.378	0.00	0.00	PROA
ATOM	1713	CA	LEU	P	108	10.691	13.734	2.834	0.00	0.00	PROA
ATOM	1714	HA	LEU	P	108	10.600	13.817	1.761	0.00	0.00	PROA
ATOM	1715	CB	LEU	P	108	11.947	14.543	3.021	0.00	0.00	PROA
ATOM	1716	HB1	LEU	P	108	12.670	14.321	2.207	0.00	0.00	PROA
ATOM	1717	HB2	LEU	P	108	12.468	14.262	3.961	0.00	0.00	PROA
ATOM	1718	CG	LEU	P	108	11.777	16.066	3.020	0.00	0.00	PROA
ATOM	1719	HG	LEU	P	108	11.252	16.437	3.926	0.00	0.00	PROA
ATOM	1720	CD1	LEU	P	108	13.184	16.681	2.965	0.00	0.00	PROA
ATOM	1721	HD11	LEU	P	108	13.228	17.789	2.890	0.00	0.00	PROA
ATOM	1722	HD12	LEU	P	108	13.669	16.147	2.121	0.00	0.00	PROA
ATOM	1723	HD13	LEU	P	108	13.665	16.403	3.927	0.00	0.00	PROA
ATOM	1724	CD2	LEU	P	108	10.981	16.408	1.734	0.00	0.00	PROA
ATOM	1725	HD21	LEU	P	108	11.087	17.472	1.434	0.00	0.00	PROA
ATOM	1726	HD22	LEU	P	108	9.891	16.438	1.947	0.00	0.00	PROA
ATOM	1727	HD23	LEU	P	108	11.269	15.763	0.876	0.00	0.00	PROA
ATOM	1728	C	LEU	P	108	10.985	12.237	3.054	0.00	0.00	PROA
ATOM	1729	O	LEU	P	108	11.422	11.485	2.183	0.00	0.00	PROA
ATOM	1730	N	CYS	P	109	10.552	11.680	4.153	0.00	0.00	PROA
ATOM	1731	HN	CYS	P	109	9.977	12.120	4.839	0.00	0.00	PROA
ATOM	1732	CA	CYS	P	109	10.551	10.305	4.365	0.00	0.00	PROA
ATOM	1733	HA	CYS	P	109	11.497	9.916	4.019	0.00	0.00	PROA
ATOM	1734	CB	CYS	P	109	10.422	9.892	5.877	0.00	0.00	PROA
ATOM	1735	HB1	CYS	P	109	9.486	10.311	6.306	0.00	0.00	PROA
ATOM	1736	HB2	CYS	P	109	11.263	10.394	6.402	0.00	0.00	PROA
ATOM	1737	SG	CYS	P	109	10.405	8.094	6.347	0.00	0.00	PROA
ATOM	1738	C	CYS	P	109	9.548	9.557	3.422	0.00	0.00	PROA
ATOM	1739	O	CYS	P	109	9.848	8.462	2.908	0.00	0.00	PROA
ATOM	1740	N	LYS	P	110	8.323	10.146	3.155	0.00	0.00	PROA
ATOM	1741	HN	LYS	P	110	8.070	11.010	3.582	0.00	0.00	PROA
ATOM	1742	CA	LYS	P	110	7.454	9.531	2.258	0.00	0.00	PROA
ATOM	1743	HA	LYS	P	110	7.257	8.494	2.490	0.00	0.00	PROA
ATOM	1744	CB	LYS	P	110	6.019	10.240	2.363	0.00	0.00	PROA
ATOM	1745	HB1	LYS	P	110	5.419	9.767	1.556	0.00	0.00	PROA
ATOM	1746	HB2	LYS	P	110	6.095	11.324	2.133	0.00	0.00	PROA
ATOM	1747	CG	LYS	P	110	5.141	9.892	3.562	0.00	0.00	PROA
ATOM	1748	HG1	LYS	P	110	5.656	10.480	4.352	0.00	0.00	PROA
ATOM	1749	HG2	LYS	P	110	5.220	8.818	3.834	0.00	0.00	PROA
ATOM	1750	CD	LYS	P	110	3.698	10.359	3.519	0.00	0.00	PROA
ATOM	1751	HD1	LYS	P	110	3.218	9.634	2.828	0.00	0.00	PROA
ATOM	1752	HD2	LYS	P	110	3.639	11.395	3.121	0.00	0.00	PROA
ATOM	1753	CE	LYS	P	110	2.911	10.348	4.849	0.00	0.00	PROA
ATOM	1754	HE1	LYS	P	110	1.905	10.786	4.674	0.00	0.00	PROA
ATOM	1755	HE2	LYS	P	110	3.381	10.950	5.656	0.00	0.00	PROA
ATOM	1756	NZ	LYS	P	110	2.686	9.019	5.343	0.00	0.00	PROA
ATOM	1757	HZ1	LYS	P	110	2.181	8.367	4.709	0.00	0.00	PROA
ATOM	1758	HZ2	LYS	P	110	2.076	9.102	6.181	0.00	0.00	PROA
ATOM	1759	HZ3	LYS	P	110	3.544	8.632	5.784	0.00	0.00	PROA
ATOM	1760	C	LYS	P	110	7.889	9.658	0.818	0.00	0.00	PROA
ATOM	1761	O	LYS	P	110	7.918	8.683	0.058	0.00	0.00	PROA
ATOM	1762	N	ALA	P	111	8.380	10.857	0.419	0.00	0.00	PROA
ATOM	1763	HN	ALA	P	111	8.469	11.561	1.119	0.00	0.00	PROA
ATOM	1764	CA	ALA	P	111	8.861	11.109	-0.931	0.00	0.00	PROA
ATOM	1765	HA	ALA	P	111	8.072	10.919	-1.643	0.00	0.00	PROA

ATOM	1766	CB	ALA	P	111	9.316	12.534	-1.033	0.00	0.00	PROA
ATOM	1767	HB1	ALA	P	111	9.927	12.718	-1.943	0.00	0.00	PROA
ATOM	1768	HB2	ALA	P	111	9.947	12.774	-0.151	0.00	0.00	PROA
ATOM	1769	HB3	ALA	P	111	8.513	13.299	-0.972	0.00	0.00	PROA
ATOM	1770	C	ALA	P	111	10.012	10.190	-1.317	0.00	0.00	PROA
ATOM	1771	O	ALA	P	111	10.123	9.737	-2.497	0.00	0.00	PROA
ATOM	1772	N	VAL	P	112	10.915	9.860	-0.372	0.00	0.00	PROA
ATOM	1773	HN	VAL	P	112	10.672	10.108	0.562	0.00	0.00	PROA
ATOM	1774	CA	VAL	P	112	12.166	9.163	-0.688	0.00	0.00	PROA
ATOM	1775	HA	VAL	P	112	12.435	9.691	-1.591	0.00	0.00	PROA
ATOM	1776	CB	VAL	P	112	13.295	9.066	0.354	0.00	0.00	PROA
ATOM	1777	HB	VAL	P	112	14.066	8.343	0.010	0.00	0.00	PROA
ATOM	1778	CG1	VAL	P	112	13.997	10.439	0.519	0.00	0.00	PROA
ATOM	1779	HG11	VAL	P	112	13.360	11.295	0.831	0.00	0.00	PROA
ATOM	1780	HG12	VAL	P	112	14.442	10.825	-0.423	0.00	0.00	PROA
ATOM	1781	HG13	VAL	P	112	14.855	10.298	1.211	0.00	0.00	PROA
ATOM	1782	CG2	VAL	P	112	12.820	8.526	1.731	0.00	0.00	PROA
ATOM	1783	HG21	VAL	P	112	12.603	7.439	1.663	0.00	0.00	PROA
ATOM	1784	HG22	VAL	P	112	11.848	9.046	1.869	0.00	0.00	PROA
ATOM	1785	HG23	VAL	P	112	13.495	8.787	2.575	0.00	0.00	PROA
ATOM	1786	C	VAL	P	112	11.871	7.746	-1.167	0.00	0.00	PROA
ATOM	1787	O	VAL	P	112	12.535	7.144	-2.052	0.00	0.00	PROA
ATOM	1788	N	HSD	P	113	10.950	7.150	-0.446	0.00	0.00	PROA
ATOM	1789	HN	HSD	P	113	10.676	7.594	0.404	0.00	0.00	PROA
ATOM	1790	CA	HSD	P	113	10.280	5.902	-0.662	0.00	0.00	PROA
ATOM	1791	HA	HSD	P	113	11.072	5.168	-0.695	0.00	0.00	PROA
ATOM	1792	CB	HSD	P	113	9.524	5.491	0.626	0.00	0.00	PROA
ATOM	1793	HB1	HSD	P	113	8.828	4.645	0.443	0.00	0.00	PROA
ATOM	1794	HB2	HSD	P	113	8.911	6.335	1.007	0.00	0.00	PROA
ATOM	1795	ND1	HSD	P	113	11.095	3.817	1.521	0.00	0.00	PROA
ATOM	1796	HD1	HSD	P	113	11.228	3.345	0.650	0.00	0.00	PROA
ATOM	1797	CG	HSD	P	113	10.464	5.062	1.653	0.00	0.00	PROA
ATOM	1798	CE1	HSD	P	113	11.651	3.487	2.719	0.00	0.00	PROA
ATOM	1799	HE1	HSD	P	113	12.306	2.627	2.856	0.00	0.00	PROA
ATOM	1800	NE2	HSD	P	113	11.507	4.500	3.598	0.00	0.00	PROA
ATOM	1801	CD2	HSD	P	113	10.809	5.477	2.902	0.00	0.00	PROA
ATOM	1802	HD2	HSD	P	113	10.535	6.303	3.546	0.00	0.00	PROA
ATOM	1803	C	HSD	P	113	9.496	5.729	-1.925	0.00	0.00	PROA
ATOM	1804	O	HSD	P	113	9.633	4.743	-2.658	0.00	0.00	PROA
ATOM	1805	N	VAL	P	114	8.683	6.742	-2.229	0.00	0.00	PROA
ATOM	1806	HN	VAL	P	114	8.552	7.421	-1.511	0.00	0.00	PROA
ATOM	1807	CA	VAL	P	114	7.987	6.888	-3.464	0.00	0.00	PROA
ATOM	1808	HA	VAL	P	114	7.331	6.057	-3.673	0.00	0.00	PROA
ATOM	1809	CB	VAL	P	114	6.996	7.964	-3.249	0.00	0.00	PROA
ATOM	1810	HB	VAL	P	114	7.393	8.902	-2.806	0.00	0.00	PROA
ATOM	1811	CG1	VAL	P	114	6.265	8.360	-4.563	0.00	0.00	PROA
ATOM	1812	HG11	VAL	P	114	5.660	9.289	-4.487	0.00	0.00	PROA
ATOM	1813	HG12	VAL	P	114	5.536	7.581	-4.872	0.00	0.00	PROA
ATOM	1814	HG13	VAL	P	114	7.010	8.470	-5.380	0.00	0.00	PROA
ATOM	1815	CG2	VAL	P	114	5.870	7.469	-2.223	0.00	0.00	PROA
ATOM	1816	HG21	VAL	P	114	6.173	7.157	-1.201	0.00	0.00	PROA
ATOM	1817	HG22	VAL	P	114	5.330	6.628	-2.708	0.00	0.00	PROA
ATOM	1818	HG23	VAL	P	114	5.166	8.324	-2.132	0.00	0.00	PROA
ATOM	1819	C	VAL	P	114	8.865	6.898	-4.680	0.00	0.00	PROA
ATOM	1820	O	VAL	P	114	8.634	6.218	-5.642	0.00	0.00	PROA
ATOM	1821	N	ILE	P	115	9.902	7.787	-4.582	0.00	0.00	PROA
ATOM	1822	HN	ILE	P	115	9.803	8.443	-3.838	0.00	0.00	PROA
ATOM	1823	CA	ILE	P	115	10.912	7.998	-5.602	0.00	0.00	PROA
ATOM	1824	HA	ILE	P	115	10.412	8.172	-6.543	0.00	0.00	PROA
ATOM	1825	CB	ILE	P	115	11.870	9.237	-5.235	0.00	0.00	PROA
ATOM	1826	HB	ILE	P	115	12.196	9.091	-4.184	0.00	0.00	PROA

ATOM	1827	CG2	ILE	P	115	13.230	9.220	-6.008	0.00	0.00	PROA
ATOM	1828	HG21	ILE	P	115	13.134	8.826	-7.042	0.00	0.00	PROA
ATOM	1829	HG22	ILE	P	115	14.008	8.672	-5.434	0.00	0.00	PROA
ATOM	1830	HG23	ILE	P	115	13.541	10.272	-6.183	0.00	0.00	PROA
ATOM	1831	CG1	ILE	P	115	11.007	10.519	-5.471	0.00	0.00	PROA
ATOM	1832	HG11	ILE	P	115	10.042	10.369	-4.942	0.00	0.00	PROA
ATOM	1833	HG12	ILE	P	115	10.547	10.631	-6.477	0.00	0.00	PROA
ATOM	1834	CD	ILE	P	115	11.638	11.802	-5.020	0.00	0.00	PROA
ATOM	1835	HD1	ILE	P	115	12.162	11.717	-4.044	0.00	0.00	PROA
ATOM	1836	HD2	ILE	P	115	10.919	12.648	-5.046	0.00	0.00	PROA
ATOM	1837	HD3	ILE	P	115	12.423	12.149	-5.727	0.00	0.00	PROA
ATOM	1838	C	ILE	P	115	11.793	6.721	-5.832	0.00	0.00	PROA
ATOM	1839	O	ILE	P	115	12.019	6.364	-6.976	0.00	0.00	PROA
ATOM	1840	N	TYR	P	116	12.333	6.088	-4.772	0.00	0.00	PROA
ATOM	1841	HN	TYR	P	116	12.151	6.381	-3.837	0.00	0.00	PROA
ATOM	1842	CA	TYR	P	116	13.138	4.846	-4.828	0.00	0.00	PROA
ATOM	1843	HA	TYR	P	116	13.944	5.187	-5.462	0.00	0.00	PROA
ATOM	1844	CB	TYR	P	116	13.646	4.410	-3.391	0.00	0.00	PROA
ATOM	1845	HB1	TYR	P	116	12.761	4.315	-2.726	0.00	0.00	PROA
ATOM	1846	HB2	TYR	P	116	14.214	5.257	-2.950	0.00	0.00	PROA
ATOM	1847	CG	TYR	P	116	14.458	3.236	-3.347	0.00	0.00	PROA
ATOM	1848	CD1	TYR	P	116	14.240	2.283	-2.351	0.00	0.00	PROA
ATOM	1849	HD1	TYR	P	116	13.439	2.392	-1.634	0.00	0.00	PROA
ATOM	1850	CE1	TYR	P	116	15.052	1.072	-2.177	0.00	0.00	PROA
ATOM	1851	HE1	TYR	P	116	14.910	0.297	-1.438	0.00	0.00	PROA
ATOM	1852	CZ	TYR	P	116	16.082	0.914	-3.100	0.00	0.00	PROA
ATOM	1853	OH	TYR	P	116	16.996	-0.134	-3.061	0.00	0.00	PROA
ATOM	1854	HH	TYR	P	116	17.312	-0.120	-3.968	0.00	0.00	PROA
ATOM	1855	CD2	TYR	P	116	15.514	3.061	-4.241	0.00	0.00	PROA
ATOM	1856	HD2	TYR	P	116	15.701	3.644	-5.131	0.00	0.00	PROA
ATOM	1857	CE2	TYR	P	116	16.229	1.846	-4.133	0.00	0.00	PROA
ATOM	1858	HE2	TYR	P	116	17.138	1.683	-4.693	0.00	0.00	PROA
ATOM	1859	C	TYR	P	116	12.397	3.676	-5.545	0.00	0.00	PROA
ATOM	1860	O	TYR	P	116	12.888	3.080	-6.509	0.00	0.00	PROA
ATOM	1861	N	THR	P	117	11.153	3.395	-5.116	0.00	0.00	PROA
ATOM	1862	HN	THR	P	117	10.798	3.834	-4.294	0.00	0.00	PROA
ATOM	1863	CA	THR	P	117	10.343	2.294	-5.680	0.00	0.00	PROA
ATOM	1864	HA	THR	P	117	10.891	1.367	-5.764	0.00	0.00	PROA
ATOM	1865	CB	THR	P	117	9.077	2.088	-4.945	0.00	0.00	PROA
ATOM	1866	HB	THR	P	117	8.524	3.048	-4.863	0.00	0.00	PROA
ATOM	1867	OG1	THR	P	117	9.335	1.621	-3.630	0.00	0.00	PROA
ATOM	1868	HG1	THR	P	117	8.570	1.842	-3.094	0.00	0.00	PROA
ATOM	1869	CG2	THR	P	117	7.979	1.111	-5.423	0.00	0.00	PROA
ATOM	1870	HG21	THR	P	117	7.058	1.174	-4.805	0.00	0.00	PROA
ATOM	1871	HG22	THR	P	117	8.340	0.065	-5.320	0.00	0.00	PROA
ATOM	1872	HG23	THR	P	117	7.528	1.282	-6.424	0.00	0.00	PROA
ATOM	1873	C	THR	P	117	9.863	2.584	-7.090	0.00	0.00	PROA
ATOM	1874	O	THR	P	117	9.713	1.702	-7.931	0.00	0.00	PROA
ATOM	1875	N	VAL	P	118	9.667	3.895	-7.349	0.00	0.00	PROA
ATOM	1876	HN	VAL	P	118	9.612	4.500	-6.559	0.00	0.00	PROA
ATOM	1877	CA	VAL	P	118	9.586	4.326	-8.767	0.00	0.00	PROA
ATOM	1878	HA	VAL	P	118	8.701	3.921	-9.235	0.00	0.00	PROA
ATOM	1879	CB	VAL	P	118	9.263	5.861	-8.697	0.00	0.00	PROA
ATOM	1880	HB	VAL	P	118	9.756	6.248	-7.779	0.00	0.00	PROA
ATOM	1881	CG1	VAL	P	118	9.785	6.592	-9.928	0.00	0.00	PROA
ATOM	1882	HG11	VAL	P	118	10.891	6.557	-9.825	0.00	0.00	PROA
ATOM	1883	HG12	VAL	P	118	9.561	7.679	-9.950	0.00	0.00	PROA
ATOM	1884	HG13	VAL	P	118	9.423	6.057	-10.832	0.00	0.00	PROA
ATOM	1885	CG2	VAL	P	118	7.779	5.942	-8.592	0.00	0.00	PROA
ATOM	1886	HG21	VAL	P	118	7.355	5.336	-7.764	0.00	0.00	PROA
ATOM	1887	HG22	VAL	P	118	7.246	5.537	-9.479	0.00	0.00	PROA

```
ATOM   1888 HG23 VAL P 118       7.412   6.973  -8.400  0.00  0.00      PROA
ATOM   1889  C   VAL P 118      10.786   4.025  -9.639  0.00  0.00      PROA
ATOM   1890  O   VAL P 118      10.546   3.440 -10.663  0.00  0.00      PROA
ATOM   1891  N   ASN P 119      12.020   4.382  -9.201  0.00  0.00      PROA
ATOM   1892  HN  ASN P 119      12.229   4.790  -8.316  0.00  0.00      PROA
ATOM   1893  CA  ASN P 119      13.170   3.942  -9.978  0.00  0.00      PROA
ATOM   1894  HA  ASN P 119      13.321   4.457 -10.916  0.00  0.00      PROA
ATOM   1895  CB  ASN P 119      14.364   4.481  -9.165  0.00  0.00      PROA
ATOM   1896  HB1 ASN P 119      14.391   3.970  -8.179  0.00  0.00      PROA
ATOM   1897  HB2 ASN P 119      14.086   5.551  -9.052  0.00  0.00      PROA
ATOM   1898  CG  ASN P 119      15.632   4.094  -9.888  0.00  0.00      PROA
ATOM   1899  OD1 ASN P 119      16.549   3.412  -9.389  0.00  0.00      PROA
ATOM   1900  ND2 ASN P 119      15.727   4.651 -11.138  0.00  0.00      PROA
ATOM   1901 HD21 ASN P 119      16.651   4.534 -11.501  0.00  0.00      PROA
ATOM   1902 HD22 ASN P 119      15.116   5.376 -11.455  0.00  0.00      PROA
ATOM   1903  C   ASN P 119      13.311   2.433 -10.264  0.00  0.00      PROA
ATOM   1904  O   ASN P 119      13.564   2.065 -11.411  0.00  0.00      PROA
ATOM   1905  N   LEU P 120      13.128   1.655  -9.205  0.00  0.00      PROA
ATOM   1906  HN  LEU P 120      12.857   2.105  -8.357  0.00  0.00      PROA
ATOM   1907  CA  LEU P 120      13.099   0.202  -9.148  0.00  0.00      PROA
ATOM   1908  HA  LEU P 120      14.135  -0.102  -9.152  0.00  0.00      PROA
ATOM   1909  CB  LEU P 120      12.467  -0.253  -7.732  0.00  0.00      PROA
ATOM   1910  HB1 LEU P 120      12.054  -1.284  -7.754  0.00  0.00      PROA
ATOM   1911  HB2 LEU P 120      11.666   0.485  -7.511  0.00  0.00      PROA
ATOM   1912  CG  LEU P 120      13.515  -0.358  -6.583  0.00  0.00      PROA
ATOM   1913  HG  LEU P 120      13.909   0.680  -6.541  0.00  0.00      PROA
ATOM   1914  CD1 LEU P 120      12.813  -0.708  -5.340  0.00  0.00      PROA
ATOM   1915 HD11 LEU P 120      12.146   0.153  -5.122  0.00  0.00      PROA
ATOM   1916 HD12 LEU P 120      13.614  -0.704  -4.570  0.00  0.00      PROA
ATOM   1917 HD13 LEU P 120      12.396  -1.732  -5.449  0.00  0.00      PROA
ATOM   1918  CD2 LEU P 120      14.772  -1.317  -6.833  0.00  0.00      PROA
ATOM   1919 HD21 LEU P 120      15.447  -1.243  -5.953  0.00  0.00      PROA
ATOM   1920 HD22 LEU P 120      15.356  -0.944  -7.701  0.00  0.00      PROA
ATOM   1921 HD23 LEU P 120      14.512  -2.396  -6.883  0.00  0.00      PROA
ATOM   1922  C   LEU P 120      12.305  -0.481 -10.284  0.00  0.00      PROA
ATOM   1923  O   LEU P 120      12.857  -0.960 -11.314  0.00  0.00      PROA
ATOM   1924  N   TYR P 121      10.974  -0.357 -10.119  0.00  0.00      PROA
ATOM   1925  HN  TYR P 121      10.582   0.043  -9.294  0.00  0.00      PROA
ATOM   1926  CA  TYR P 121      10.064  -0.864 -11.032  0.00  0.00      PROA
ATOM   1927  HA  TYR P 121      10.480  -1.860 -11.053  0.00  0.00      PROA
ATOM   1928  CB  TYR P 121       8.630  -1.086 -10.508  0.00  0.00      PROA
ATOM   1929  HB1 TYR P 121       7.956  -1.447 -11.314  0.00  0.00      PROA
ATOM   1930  HB2 TYR P 121       8.229  -0.081 -10.257  0.00  0.00      PROA
ATOM   1931  CG  TYR P 121       8.647  -2.038  -9.341  0.00  0.00      PROA
ATOM   1932  CD1 TYR P 121       9.042  -3.369  -9.253  0.00  0.00      PROA
ATOM   1933  HD1 TYR P 121       9.410  -3.835 -10.155  0.00  0.00      PROA
ATOM   1934  CE1 TYR P 121       8.722  -4.196  -8.155  0.00  0.00      PROA
ATOM   1935  HE1 TYR P 121       8.838  -5.270  -8.138  0.00  0.00      PROA
ATOM   1936  CZ  TYR P 121       8.286  -3.605  -6.966  0.00  0.00      PROA
ATOM   1937  OH  TYR P 121       8.019  -4.295  -5.795  0.00  0.00      PROA
ATOM   1938  HH  TYR P 121       7.807  -3.693  -5.078  0.00  0.00      PROA
ATOM   1939  CD2 TYR P 121       8.126  -1.473  -8.182  0.00  0.00      PROA
ATOM   1940  HD2 TYR P 121       7.856  -0.431  -8.100  0.00  0.00      PROA
ATOM   1941  CE2 TYR P 121       7.968  -2.261  -7.063  0.00  0.00      PROA
ATOM   1942  HE2 TYR P 121       7.493  -1.711  -6.265  0.00  0.00      PROA
ATOM   1943  C   TYR P 121      10.034  -0.330 -12.424  0.00  0.00      PROA
ATOM   1944  O   TYR P 121      10.028  -1.140 -13.350  0.00  0.00      PROA
ATOM   1945  N   SER P 122      10.113   1.061 -12.658  0.00  0.00      PROA
ATOM   1946  HN  SER P 122      10.157   1.664 -11.865  0.00  0.00      PROA
ATOM   1947  CA  SER P 122      10.178   1.661 -13.987  0.00  0.00      PROA
ATOM   1948  HA  SER P 122       9.265   1.325 -14.457  0.00  0.00      PROA
```

ATOM	1949	CB	SER	P	122	10.124	3.206	-13.951	0.00	0.00	PROA
ATOM	1950	HB1	SER	P	122	9.193	3.447	-13.394	0.00	0.00	PROA
ATOM	1951	HB2	SER	P	122	10.150	3.624	-14.980	0.00	0.00	PROA
ATOM	1952	OG	SER	P	122	11.225	3.782	-13.236	0.00	0.00	PROA
ATOM	1953	HG1	SER	P	122	11.034	3.855	-12.298	0.00	0.00	PROA
ATOM	1954	C	SER	P	122	11.416	1.110	-14.779	0.00	0.00	PROA
ATOM	1955	O	SER	P	122	11.279	0.789	-15.961	0.00	0.00	PROA
ATOM	1956	N	SER	P	123	12.595	1.002	-14.166	0.00	0.00	PROA
ATOM	1957	HN	SER	P	123	12.688	1.195	-13.192	0.00	0.00	PROA
ATOM	1958	CA	SER	P	123	13.854	0.704	-14.741	0.00	0.00	PROA
ATOM	1959	HA	SER	P	123	14.017	1.399	-15.552	0.00	0.00	PROA
ATOM	1960	CB	SER	P	123	15.051	0.959	-13.831	0.00	0.00	PROA
ATOM	1961	HB1	SER	P	123	15.065	2.026	-13.522	0.00	0.00	PROA
ATOM	1962	HB2	SER	P	123	15.952	0.667	-14.411	0.00	0.00	PROA
ATOM	1963	OG	SER	P	123	15.097	0.165	-12.617	0.00	0.00	PROA
ATOM	1964	HG1	SER	P	123	14.484	0.514	-11.966	0.00	0.00	PROA
ATOM	1965	C	SER	P	123	13.960	-0.652	-15.351	0.00	0.00	PROA
ATOM	1966	O	SER	P	123	14.426	-0.827	-16.457	0.00	0.00	PROA
ATOM	1967	N	VAL	P	124	13.479	-1.727	-14.683	0.00	0.00	PROA
ATOM	1968	HN	VAL	P	124	13.190	-1.619	-13.735	0.00	0.00	PROA
ATOM	1969	CA	VAL	P	124	13.561	-3.018	-15.393	0.00	0.00	PROA
ATOM	1970	HA	VAL	P	124	14.533	-3.073	-15.861	0.00	0.00	PROA
ATOM	1971	CB	VAL	P	124	13.497	-4.154	-14.430	0.00	0.00	PROA
ATOM	1972	HB	VAL	P	124	14.133	-3.941	-13.544	0.00	0.00	PROA
ATOM	1973	CG1	VAL	P	124	12.088	-4.242	-13.783	0.00	0.00	PROA
ATOM	1974	HG11	VAL	P	124	11.935	-3.428	-13.042	0.00	0.00	PROA
ATOM	1975	HG12	VAL	P	124	11.930	-5.218	-13.277	0.00	0.00	PROA
ATOM	1976	HG13	VAL	P	124	11.342	-4.344	-14.600	0.00	0.00	PROA
ATOM	1977	CG2	VAL	P	124	13.977	-5.472	-15.089	0.00	0.00	PROA
ATOM	1978	HG21	VAL	P	124	14.893	-5.191	-15.652	0.00	0.00	PROA
ATOM	1979	HG22	VAL	P	124	13.315	-5.852	-15.897	0.00	0.00	PROA
ATOM	1980	HG23	VAL	P	124	14.218	-6.220	-14.303	0.00	0.00	PROA
ATOM	1981	C	VAL	P	124	12.621	-3.126	-16.632	0.00	0.00	PROA
ATOM	1982	O	VAL	P	124	12.989	-3.740	-17.648	0.00	0.00	PROA
ATOM	1983	N	TRP	P	125	11.410	-2.473	-16.516	0.00	0.00	PROA
ATOM	1984	HN	TRP	P	125	11.303	-1.874	-15.727	0.00	0.00	PROA
ATOM	1985	CA	TRP	P	125	10.320	-2.377	-17.527	0.00	0.00	PROA
ATOM	1986	HA	TRP	P	125	10.222	-3.395	-17.876	0.00	0.00	PROA
ATOM	1987	CB	TRP	P	125	8.937	-1.895	-16.955	0.00	0.00	PROA
ATOM	1988	HB1	TRP	P	125	8.219	-1.569	-17.738	0.00	0.00	PROA
ATOM	1989	HB2	TRP	P	125	9.000	-1.032	-16.258	0.00	0.00	PROA
ATOM	1990	CG	TRP	P	125	8.115	-2.913	-16.194	0.00	0.00	PROA
ATOM	1991	CD1	TRP	P	125	8.489	-3.383	-14.964	0.00	0.00	PROA
ATOM	1992	HD1	TRP	P	125	9.434	-3.083	-14.537	0.00	0.00	PROA
ATOM	1993	NE1	TRP	P	125	7.686	-4.372	-14.612	0.00	0.00	PROA
ATOM	1994	HE1	TRP	P	125	7.893	-4.957	-13.859	0.00	0.00	PROA
ATOM	1995	CE2	TRP	P	125	6.688	-4.487	-15.523	0.00	0.00	PROA
ATOM	1996	CD2	TRP	P	125	6.903	-3.551	-16.547	0.00	0.00	PROA
ATOM	1997	CE3	TRP	P	125	6.025	-3.431	-17.572	0.00	0.00	PROA
ATOM	1998	HE3	TRP	P	125	6.171	-2.738	-18.387	0.00	0.00	PROA
ATOM	1999	CZ3	TRP	P	125	4.941	-4.249	-17.574	0.00	0.00	PROA
ATOM	2000	HZ3	TRP	P	125	4.193	-4.121	-18.342	0.00	0.00	PROA
ATOM	2001	CZ2	TRP	P	125	5.686	-5.414	-15.618	0.00	0.00	PROA
ATOM	2002	HZ2	TRP	P	125	5.484	-6.152	-14.856	0.00	0.00	PROA
ATOM	2003	CH2	TRP	P	125	4.754	-5.218	-16.629	0.00	0.00	PROA
ATOM	2004	HH2	TRP	P	125	3.951	-5.922	-16.787	0.00	0.00	PROA
ATOM	2005	C	TRP	P	125	10.854	-1.638	-18.714	0.00	0.00	PROA
ATOM	2006	O	TRP	P	125	10.780	-2.172	-19.815	0.00	0.00	PROA
ATOM	2007	N	ILE	P	126	11.565	-0.543	-18.563	0.00	0.00	PROA
ATOM	2008	HN	ILE	P	126	11.542	0.061	-17.769	0.00	0.00	PROA
ATOM	2009	CA	ILE	P	126	12.391	0.034	-19.596	0.00	0.00	PROA

ATOM	2010	HA	ILE	P	126	11.757	0.333	-20.418	0.00	0.00	PROA
ATOM	2011	CB	ILE	P	126	12.973	1.331	-19.009	0.00	0.00	PROA
ATOM	2012	HB	ILE	P	126	13.328	1.073	-17.988	0.00	0.00	PROA
ATOM	2013	CG2	ILE	P	126	14.142	1.938	-19.838	0.00	0.00	PROA
ATOM	2014	HG21	ILE	P	126	15.110	1.404	-19.725	0.00	0.00	PROA
ATOM	2015	HG22	ILE	P	126	14.252	3.013	-19.583	0.00	0.00	PROA
ATOM	2016	HG23	ILE	P	126	13.893	1.860	-20.918	0.00	0.00	PROA
ATOM	2017	CG1	ILE	P	126	11.987	2.489	-18.784	0.00	0.00	PROA
ATOM	2018	HG11	ILE	P	126	12.588	3.365	-18.461	0.00	0.00	PROA
ATOM	2019	HG12	ILE	P	126	11.345	2.254	-17.907	0.00	0.00	PROA
ATOM	2020	CD	ILE	P	126	11.111	3.019	-19.985	0.00	0.00	PROA
ATOM	2021	HD1	ILE	P	126	11.705	3.551	-20.758	0.00	0.00	PROA
ATOM	2022	HD2	ILE	P	126	10.449	3.846	-19.649	0.00	0.00	PROA
ATOM	2023	HD3	ILE	P	126	10.397	2.272	-20.393	0.00	0.00	PROA
ATOM	2024	C	ILE	P	126	13.402	-0.890	-20.242	0.00	0.00	PROA
ATOM	2025	O	ILE	P	126	13.513	-0.893	-21.453	0.00	0.00	PROA
ATOM	2026	N	LEU	P	127	14.209	-1.731	-19.510	0.00	0.00	PROA
ATOM	2027	HN	LEU	P	127	14.263	-1.688	-18.515	0.00	0.00	PROA
ATOM	2028	CA	LEU	P	127	15.105	-2.707	-20.133	0.00	0.00	PROA
ATOM	2029	HA	LEU	P	127	15.728	-2.117	-20.789	0.00	0.00	PROA
ATOM	2030	CB	LEU	P	127	15.968	-3.382	-19.046	0.00	0.00	PROA
ATOM	2031	HB1	LEU	P	127	16.679	-4.071	-19.551	0.00	0.00	PROA
ATOM	2032	HB2	LEU	P	127	15.385	-3.822	-18.209	0.00	0.00	PROA
ATOM	2033	CG	LEU	P	127	16.735	-2.323	-18.303	0.00	0.00	PROA
ATOM	2034	HG	LEU	P	127	15.982	-1.639	-17.856	0.00	0.00	PROA
ATOM	2035	CD1	LEU	P	127	17.584	-3.126	-17.310	0.00	0.00	PROA
ATOM	2036	HD11	LEU	P	127	18.228	-2.460	-16.696	0.00	0.00	PROA
ATOM	2037	HD12	LEU	P	127	18.256	-3.793	-17.892	0.00	0.00	PROA
ATOM	2038	HD13	LEU	P	127	17.061	-3.807	-16.605	0.00	0.00	PROA
ATOM	2039	CD2	LEU	P	127	17.625	-1.427	-19.068	0.00	0.00	PROA
ATOM	2040	HD21	LEU	P	127	18.281	-2.151	-19.596	0.00	0.00	PROA
ATOM	2041	HD22	LEU	P	127	18.305	-0.798	-18.454	0.00	0.00	PROA
ATOM	2042	HD23	LEU	P	127	17.124	-0.698	-19.740	0.00	0.00	PROA
ATOM	2043	C	LEU	P	127	14.362	-3.728	-21.014	0.00	0.00	PROA
ATOM	2044	O	LEU	P	127	14.848	-4.189	-22.091	0.00	0.00	PROA
ATOM	2045	N	ALA	P	128	13.162	-4.048	-20.583	0.00	0.00	PROA
ATOM	2046	HN	ALA	P	128	12.799	-3.753	-19.702	0.00	0.00	PROA
ATOM	2047	CA	ALA	P	128	12.233	-4.961	-21.326	0.00	0.00	PROA
ATOM	2048	HA	ALA	P	128	12.798	-5.833	-21.622	0.00	0.00	PROA
ATOM	2049	CB	ALA	P	128	10.996	-5.451	-20.544	0.00	0.00	PROA
ATOM	2050	HB1	ALA	P	128	10.568	-4.569	-20.022	0.00	0.00	PROA
ATOM	2051	HB2	ALA	P	128	11.298	-6.258	-19.843	0.00	0.00	PROA
ATOM	2052	HB3	ALA	P	128	10.205	-5.853	-21.213	0.00	0.00	PROA
ATOM	2053	C	ALA	P	128	11.800	-4.343	-22.690	0.00	0.00	PROA
ATOM	2054	O	ALA	P	128	11.941	-4.948	-23.685	0.00	0.00	PROA
ATOM	2055	N	PHE	P	129	11.381	-3.057	-22.721	0.00	0.00	PROA
ATOM	2056	HN	PHE	P	129	11.138	-2.658	-21.840	0.00	0.00	PROA
ATOM	2057	CA	PHE	P	129	11.046	-2.316	-23.960	0.00	0.00	PROA
ATOM	2058	HA	PHE	P	129	10.483	-3.040	-24.529	0.00	0.00	PROA
ATOM	2059	CB	PHE	P	129	10.209	-1.039	-23.750	0.00	0.00	PROA
ATOM	2060	HB1	PHE	P	129	9.993	-0.550	-24.724	0.00	0.00	PROA
ATOM	2061	HB2	PHE	P	129	10.675	-0.292	-23.072	0.00	0.00	PROA
ATOM	2062	CG	PHE	P	129	9.026	-1.531	-22.996	0.00	0.00	PROA
ATOM	2063	CD1	PHE	P	129	8.833	-1.234	-21.586	0.00	0.00	PROA
ATOM	2064	HD1	PHE	P	129	9.569	-0.695	-21.008	0.00	0.00	PROA
ATOM	2065	CE1	PHE	P	129	7.796	-1.900	-20.936	0.00	0.00	PROA
ATOM	2066	HE1	PHE	P	129	7.724	-1.773	-19.866	0.00	0.00	PROA
ATOM	2067	CZ	PHE	P	129	6.955	-2.795	-21.517	0.00	0.00	PROA
ATOM	2068	HZ	PHE	P	129	6.115	-3.322	-21.090	0.00	0.00	PROA
ATOM	2069	CD2	PHE	P	129	8.160	-2.400	-23.617	0.00	0.00	PROA
ATOM	2070	HD2	PHE	P	129	8.320	-2.600	-24.666	0.00	0.00	PROA

ATOM	2071	CE2	PHE	P	129	7.086	-2.930	-22.941	0.00	0.00	PROA
ATOM	2072	HE2	PHE	P	129	6.486	-3.600	-23.537	0.00	0.00	PROA
ATOM	2073	C	PHE	P	129	12.278	-2.132	-24.826	0.00	0.00	PROA
ATOM	2074	O	PHE	P	129	12.138	-2.089	-26.022	0.00	0.00	PROA
ATOM	2075	N	ILE	P	130	13.512	-1.970	-24.230	0.00	0.00	PROA
ATOM	2076	HN	ILE	P	130	13.691	-1.757	-23.272	0.00	0.00	PROA
ATOM	2077	CA	ILE	P	130	14.721	-1.839	-25.018	0.00	0.00	PROA
ATOM	2078	HA	ILE	P	130	14.677	-1.054	-25.757	0.00	0.00	PROA
ATOM	2079	CB	ILE	P	130	15.878	-1.265	-24.179	0.00	0.00	PROA
ATOM	2080	HB	ILE	P	130	15.768	-1.869	-23.253	0.00	0.00	PROA
ATOM	2081	CG2	ILE	P	130	17.293	-1.468	-24.768	0.00	0.00	PROA
ATOM	2082	HG21	ILE	P	130	17.408	-0.779	-25.632	0.00	0.00	PROA
ATOM	2083	HG22	ILE	P	130	17.374	-2.493	-25.189	0.00	0.00	PROA
ATOM	2084	HG23	ILE	P	130	18.156	-1.332	-24.081	0.00	0.00	PROA
ATOM	2085	CG1	ILE	P	130	15.600	0.191	-23.734	0.00	0.00	PROA
ATOM	2086	HG11	ILE	P	130	14.556	0.239	-23.356	0.00	0.00	PROA
ATOM	2087	HG12	ILE	P	130	15.787	0.856	-24.604	0.00	0.00	PROA
ATOM	2088	CD	ILE	P	130	16.459	0.683	-22.635	0.00	0.00	PROA
ATOM	2089	HD1	ILE	P	130	17.550	0.792	-22.812	0.00	0.00	PROA
ATOM	2090	HD2	ILE	P	130	16.367	0.129	-21.676	0.00	0.00	PROA
ATOM	2091	HD3	ILE	P	130	16.147	1.723	-22.400	0.00	0.00	PROA
ATOM	2092	C	ILE	P	130	15.067	-3.173	-25.694	0.00	0.00	PROA
ATOM	2093	O	ILE	P	130	15.470	-3.217	-26.861	0.00	0.00	PROA
ATOM	2094	N	SER	P	131	14.818	-4.281	-24.918	0.00	0.00	PROA
ATOM	2095	HN	SER	P	131	14.472	-4.198	-23.987	0.00	0.00	PROA
ATOM	2096	CA	SER	P	131	14.977	-5.611	-25.453	0.00	0.00	PROA
ATOM	2097	HA	SER	P	131	15.928	-5.745	-25.946	0.00	0.00	PROA
ATOM	2098	CB	SER	P	131	14.744	-6.637	-24.260	0.00	0.00	PROA
ATOM	2099	HB1	SER	P	131	14.909	-7.672	-24.629	0.00	0.00	PROA
ATOM	2100	HB2	SER	P	131	13.729	-6.479	-23.838	0.00	0.00	PROA
ATOM	2101	OG	SER	P	131	15.663	-6.397	-23.226	0.00	0.00	PROA
ATOM	2102	HG1	SER	P	131	15.422	-5.564	-22.813	0.00	0.00	PROA
ATOM	2103	C	SER	P	131	13.936	-5.904	-26.533	0.00	0.00	PROA
ATOM	2104	O	SER	P	131	14.086	-6.461	-27.583	0.00	0.00	PROA
ATOM	2105	N	LEU	P	132	12.640	-5.448	-26.292	0.00	0.00	PROA
ATOM	2106	HN	LEU	P	132	12.396	-5.025	-25.423	0.00	0.00	PROA
ATOM	2107	CA	LEU	P	132	11.584	-5.658	-27.225	0.00	0.00	PROA
ATOM	2108	HA	LEU	P	132	11.662	-6.693	-27.524	0.00	0.00	PROA
ATOM	2109	CB	LEU	P	132	10.293	-5.372	-26.569	0.00	0.00	PROA
ATOM	2110	HB1	LEU	P	132	10.168	-4.329	-26.207	0.00	0.00	PROA
ATOM	2111	HB2	LEU	P	132	10.368	-5.843	-25.566	0.00	0.00	PROA
ATOM	2112	CG	LEU	P	132	9.037	-6.022	-27.152	0.00	0.00	PROA
ATOM	2113	HG	LEU	P	132	8.898	-5.641	-28.187	0.00	0.00	PROA
ATOM	2114	CD1	LEU	P	132	9.117	-7.528	-27.122	0.00	0.00	PROA
ATOM	2115	HD11	LEU	P	132	8.453	-7.900	-27.931	0.00	0.00	PROA
ATOM	2116	HD12	LEU	P	132	8.705	-7.940	-26.176	0.00	0.00	PROA
ATOM	2117	HD13	LEU	P	132	10.161	-7.898	-27.212	0.00	0.00	PROA
ATOM	2118	CD2	LEU	P	132	7.677	-5.628	-26.419	0.00	0.00	PROA
ATOM	2119	HD21	LEU	P	132	6.927	-6.108	-27.084	0.00	0.00	PROA
ATOM	2120	HD22	LEU	P	132	7.582	-4.521	-26.418	0.00	0.00	PROA
ATOM	2121	HD23	LEU	P	132	7.668	-6.063	-25.397	0.00	0.00	PROA
ATOM	2122	C	LEU	P	132	11.740	-4.799	-28.522	0.00	0.00	PROA
ATOM	2123	O	LEU	P	132	11.524	-5.211	-29.594	0.00	0.00	PROA
ATOM	2124	N	ASP	P	133	12.130	-3.503	-28.364	0.00	0.00	PROA
ATOM	2125	HN	ASP	P	133	12.156	-3.084	-27.459	0.00	0.00	PROA
ATOM	2126	CA	ASP	P	133	12.544	-2.655	-29.466	0.00	0.00	PROA
ATOM	2127	HA	ASP	P	133	11.685	-2.465	-30.092	0.00	0.00	PROA
ATOM	2128	CB	ASP	P	133	12.899	-1.316	-28.874	0.00	0.00	PROA
ATOM	2129	HB1	ASP	P	133	13.863	-1.345	-28.322	0.00	0.00	PROA
ATOM	2130	HB2	ASP	P	133	12.021	-1.022	-28.260	0.00	0.00	PROA
ATOM	2131	CG	ASP	P	133	12.997	-0.267	-29.980	0.00	0.00	PROA

```
ATOM   2132  OD1 ASP P 133      12.115  -0.276 -30.864  0.00  0.00      PROA
ATOM   2133  OD2 ASP P 133      13.999   0.540 -30.081  0.00  0.00      PROA
ATOM   2134  C   ASP P 133      13.666  -3.172 -30.258  0.00  0.00      PROA
ATOM   2135  O   ASP P 133      13.580  -3.323 -31.522  0.00  0.00      PROA
ATOM   2136  N   ARG P 134      14.871  -3.677 -29.660  0.00  0.00      PROA
ATOM   2137  HN  ARG P 134      14.953  -3.727 -28.667  0.00  0.00      PROA
ATOM   2138  CA  ARG P 134      16.034  -4.074 -30.425  0.00  0.00      PROA
ATOM   2139  HA  ARG P 134      16.136  -3.344 -31.214  0.00  0.00      PROA
ATOM   2140  CB  ARG P 134      17.261  -4.083 -29.596  0.00  0.00      PROA
ATOM   2141  HB1 ARG P 134      18.006  -4.514 -30.300  0.00  0.00      PROA
ATOM   2142  HB2 ARG P 134      17.023  -4.664 -28.679  0.00  0.00      PROA
ATOM   2143  CG  ARG P 134      17.702  -2.605 -29.351  0.00  0.00      PROA
ATOM   2144  HG1 ARG P 134      18.687  -2.574 -28.838  0.00  0.00      PROA
ATOM   2145  HG2 ARG P 134      17.020  -2.116 -28.624  0.00  0.00      PROA
ATOM   2146  CD  ARG P 134      18.046  -1.689 -30.647  0.00  0.00      PROA
ATOM   2147  HD1 ARG P 134      18.460  -2.387 -31.405  0.00  0.00      PROA
ATOM   2148  HD2 ARG P 134      18.719  -0.829 -30.443  0.00  0.00      PROA
ATOM   2149  NE  ARG P 134      16.733  -1.095 -31.048  0.00  0.00      PROA
ATOM   2150  HE  ARG P 134      15.985  -1.014 -30.390  0.00  0.00      PROA
ATOM   2151  CZ  ARG P 134      16.665  -0.379 -32.104  0.00  0.00      PROA
ATOM   2152  NH1 ARG P 134      17.589  -0.276 -33.041  0.00  0.00      PROA
ATOM   2153 HH11 ARG P 134      18.416  -0.837 -32.993  0.00  0.00      PROA
ATOM   2154 HH12 ARG P 134      17.372   0.388 -33.756  0.00  0.00      PROA
ATOM   2155  NH2 ARG P 134      15.611   0.372 -32.257  0.00  0.00      PROA
ATOM   2156 HH21 ARG P 134      15.578   0.992 -33.041  0.00  0.00      PROA
ATOM   2157 HH22 ARG P 134      14.966   0.326 -31.494  0.00  0.00      PROA
ATOM   2158  C   ARG P 134      15.776  -5.528 -30.999  0.00  0.00      PROA
ATOM   2159  O   ARG P 134      16.268  -5.806 -32.091  0.00  0.00      PROA
ATOM   2160  N   TYR P 135      14.879  -6.373 -30.427  0.00  0.00      PROA
ATOM   2161  HN  TYR P 135      14.469  -6.302 -29.521  0.00  0.00      PROA
ATOM   2162  CA  TYR P 135      14.448  -7.619 -31.158  0.00  0.00      PROA
ATOM   2163  HA  TYR P 135      15.256  -8.202 -31.574  0.00  0.00      PROA
ATOM   2164  CB  TYR P 135      13.856  -8.496 -30.038  0.00  0.00      PROA
ATOM   2165  HB1 TYR P 135      13.258  -7.762 -29.457  0.00  0.00      PROA
ATOM   2166  HB2 TYR P 135      14.701  -8.914 -29.451  0.00  0.00      PROA
ATOM   2167  CG  TYR P 135      12.955  -9.686 -30.533  0.00  0.00      PROA
ATOM   2168  CD1 TYR P 135      11.527  -9.570 -30.631  0.00  0.00      PROA
ATOM   2169  HD1 TYR P 135      10.956  -8.677 -30.421  0.00  0.00      PROA
ATOM   2170  CE1 TYR P 135      10.790 -10.633 -31.154  0.00  0.00      PROA
ATOM   2171  HE1 TYR P 135       9.739 -10.547 -31.390  0.00  0.00      PROA
ATOM   2172  CZ  TYR P 135      11.451 -11.830 -31.549  0.00  0.00      PROA
ATOM   2173  OH  TYR P 135      10.757 -12.911 -32.164  0.00  0.00      PROA
ATOM   2174  HH  TYR P 135      11.250 -13.714 -31.985  0.00  0.00      PROA
ATOM   2175  CD2 TYR P 135      13.571 -10.785 -31.031  0.00  0.00      PROA
ATOM   2176  HD2 TYR P 135      14.642 -10.912 -31.088  0.00  0.00      PROA
ATOM   2177  CE2 TYR P 135      12.853 -11.878 -31.541  0.00  0.00      PROA
ATOM   2178  HE2 TYR P 135      13.335 -12.758 -31.942  0.00  0.00      PROA
ATOM   2179  C   TYR P 135      13.624  -7.304 -32.350  0.00  0.00      PROA
ATOM   2180  O   TYR P 135      13.864  -7.867 -33.428  0.00  0.00      PROA
ATOM   2181  N   LEU P 136      12.711  -6.327 -32.268  0.00  0.00      PROA
ATOM   2182  HN  LEU P 136      12.478  -5.826 -31.438  0.00  0.00      PROA
ATOM   2183  CA  LEU P 136      11.923  -5.957 -33.406  0.00  0.00      PROA
ATOM   2184  HA  LEU P 136      11.433  -6.851 -33.764  0.00  0.00      PROA
ATOM   2185  CB  LEU P 136      10.663  -5.200 -33.088  0.00  0.00      PROA
ATOM   2186  HB1 LEU P 136      10.141  -4.896 -34.021  0.00  0.00      PROA
ATOM   2187  HB2 LEU P 136      10.977  -4.269 -32.569  0.00  0.00      PROA
ATOM   2188  CG  LEU P 136       9.602  -5.883 -32.209  0.00  0.00      PROA
ATOM   2189  HG  LEU P 136      10.079  -6.069 -31.223  0.00  0.00      PROA
ATOM   2190  CD1 LEU P 136       8.428  -4.883 -31.934  0.00  0.00      PROA
ATOM   2191 HD11 LEU P 136       7.712  -5.273 -31.179  0.00  0.00      PROA
ATOM   2192 HD12 LEU P 136       7.928  -4.556 -32.870  0.00  0.00      PROA
```

```
ATOM   2193 HD13 LEU P 136       8.943  -3.992 -31.516  0.00  0.00      PROA
ATOM   2194  CD2 LEU P 136       8.995  -7.188 -32.672  0.00  0.00      PROA
ATOM   2195 HD21 LEU P 136       9.750  -8.000 -32.591  0.00  0.00      PROA
ATOM   2196 HD22 LEU P 136       8.642  -6.980 -33.704  0.00  0.00      PROA
ATOM   2197 HD23 LEU P 136       8.060  -7.492 -32.155  0.00  0.00      PROA
ATOM   2198  C   LEU P 136      12.784  -5.243 -34.473  0.00  0.00      PROA
ATOM   2199  O   LEU P 136      12.390  -5.094 -35.639  0.00  0.00      PROA
ATOM   2200  N   ALA P 137      14.031  -4.724 -34.118  0.00  0.00      PROA
ATOM   2201  HN  ALA P 137      14.300  -4.793 -33.160  0.00  0.00      PROA
ATOM   2202  CA  ALA P 137      15.009  -4.307 -35.081  0.00  0.00      PROA
ATOM   2203  HA  ALA P 137      14.516  -3.661 -35.793  0.00  0.00      PROA
ATOM   2204  CB  ALA P 137      16.149  -3.503 -34.496  0.00  0.00      PROA
ATOM   2205  HB1 ALA P 137      15.855  -2.636 -33.867  0.00  0.00      PROA
ATOM   2206  HB2 ALA P 137      16.794  -3.213 -35.353  0.00  0.00      PROA
ATOM   2207  HB3 ALA P 137      16.714  -4.195 -33.837  0.00  0.00      PROA
ATOM   2208  C   ALA P 137      15.564  -5.487 -35.966  0.00  0.00      PROA
ATOM   2209  O   ALA P 137      15.729  -5.253 -37.184  0.00  0.00      PROA
ATOM   2210  N   ILE P 138      15.876  -6.627 -35.406  0.00  0.00      PROA
ATOM   2211  HN  ILE P 138      15.757  -6.816 -34.434  0.00  0.00      PROA
ATOM   2212  CA  ILE P 138      16.210  -7.802 -36.118  0.00  0.00      PROA
ATOM   2213  HA  ILE P 138      17.058  -7.536 -36.732  0.00  0.00      PROA
ATOM   2214  CB  ILE P 138      16.668  -8.934 -35.140  0.00  0.00      PROA
ATOM   2215  HB  ILE P 138      15.814  -9.133 -34.459  0.00  0.00      PROA
ATOM   2216  CG2 ILE P 138      17.257 -10.182 -35.829  0.00  0.00      PROA
ATOM   2217 HG21 ILE P 138      16.425 -10.801 -36.230  0.00  0.00      PROA
ATOM   2218 HG22 ILE P 138      17.804 -10.847 -35.127  0.00  0.00      PROA
ATOM   2219 HG23 ILE P 138      17.985  -9.911 -36.623  0.00  0.00      PROA
ATOM   2220  CG1 ILE P 138      17.888  -8.364 -34.247  0.00  0.00      PROA
ATOM   2221 HG11 ILE P 138      17.705  -7.298 -33.995  0.00  0.00      PROA
ATOM   2222 HG12 ILE P 138      18.813  -8.446 -34.856  0.00  0.00      PROA
ATOM   2223  CD  ILE P 138      18.194  -9.129 -33.034  0.00  0.00      PROA
ATOM   2224  HD1 ILE P 138      17.302  -9.150 -32.372  0.00  0.00      PROA
ATOM   2225  HD2 ILE P 138      18.881  -8.688 -32.281  0.00  0.00      PROA
ATOM   2226  HD3 ILE P 138      18.625 -10.146 -33.154  0.00  0.00      PROA
ATOM   2227  C   ILE P 138      15.021  -8.292 -36.990  0.00  0.00      PROA
ATOM   2228  O   ILE P 138      15.144  -8.738 -38.132  0.00  0.00      PROA
ATOM   2229  N   VAL P 139      13.745  -8.172 -36.536  0.00  0.00      PROA
ATOM   2230  HN  VAL P 139      13.553  -7.790 -35.635  0.00  0.00      PROA
ATOM   2231  CA  VAL P 139      12.552  -8.503 -37.252  0.00  0.00      PROA
ATOM   2232  HA  VAL P 139      12.651  -9.471 -37.721  0.00  0.00      PROA
ATOM   2233  CB  VAL P 139      11.216  -8.566 -36.489  0.00  0.00      PROA
ATOM   2234  HB  VAL P 139      11.086  -7.673 -35.841  0.00  0.00      PROA
ATOM   2235  CG1 VAL P 139       9.958  -8.606 -37.378  0.00  0.00      PROA
ATOM   2236 HG11 VAL P 139       9.864  -7.551 -37.712  0.00  0.00      PROA
ATOM   2237 HG12 VAL P 139       9.110  -8.952 -36.749  0.00  0.00      PROA
ATOM   2238 HG13 VAL P 139      10.056  -9.368 -38.181  0.00  0.00      PROA
ATOM   2239  CG2 VAL P 139      11.085  -9.779 -35.603  0.00  0.00      PROA
ATOM   2240 HG21 VAL P 139      10.250  -9.684 -34.876  0.00  0.00      PROA
ATOM   2241 HG22 VAL P 139      11.991  -9.979 -34.992  0.00  0.00      PROA
ATOM   2242 HG23 VAL P 139      10.940 -10.653 -36.274  0.00  0.00      PROA
ATOM   2243  C   VAL P 139      12.431  -7.573 -38.437  0.00  0.00      PROA
ATOM   2244  O   VAL P 139      11.968  -7.995 -39.461  0.00  0.00      PROA
ATOM   2245  N   HSD P 140      12.907  -6.320 -38.315  0.00  0.00      PROA
ATOM   2246  HN  HSD P 140      13.438  -6.261 -37.473  0.00  0.00      PROA
ATOM   2247  CA  HSD P 140      12.758  -5.168 -39.215  0.00  0.00      PROA
ATOM   2248  HA  HSD P 140      13.318  -4.370 -38.752  0.00  0.00      PROA
ATOM   2249  CB  HSD P 140      13.070  -5.486 -40.674  0.00  0.00      PROA
ATOM   2250  HB1 HSD P 140      12.948  -4.536 -41.237  0.00  0.00      PROA
ATOM   2251  HB2 HSD P 140      12.349  -6.224 -41.086  0.00  0.00      PROA
ATOM   2252  ND1 HSD P 140      15.450  -5.123 -41.237  0.00  0.00      PROA
ATOM   2253  HD1 HSD P 140      15.399  -4.124 -41.249  0.00  0.00      PROA
```

ATOM	2254	CG	HSD	P	140	14.450	-6.020	-40.829	0.00	0.00	PROA
ATOM	2255	CE1	HSD	P	140	16.606	-5.878	-41.527	0.00	0.00	PROA
ATOM	2256	HE1	HSD	P	140	17.503	-5.503	-42.020	0.00	0.00	PROA
ATOM	2257	NE2	HSD	P	140	16.371	-7.152	-41.407	0.00	0.00	PROA
ATOM	2258	CD2	HSD	P	140	15.008	-7.288	-41.035	0.00	0.00	PROA
ATOM	2259	HD2	HSD	P	140	14.645	-8.306	-40.963	0.00	0.00	PROA
ATOM	2260	C	HSD	P	140	11.336	-4.602	-39.193	0.00	0.00	PROA
ATOM	2261	O	HSD	P	140	10.308	-5.266	-38.964	0.00	0.00	PROA
ATOM	2262	N	ALA	P	141	11.166	-3.298	-39.554	0.00	0.00	PROA
ATOM	2263	HN	ALA	P	141	11.969	-2.708	-39.570	0.00	0.00	PROA
ATOM	2264	CA	ALA	P	141	9.902	-2.497	-39.583	0.00	0.00	PROA
ATOM	2265	HA	ALA	P	141	9.649	-2.377	-38.540	0.00	0.00	PROA
ATOM	2266	CB	ALA	P	141	10.382	-1.215	-40.111	0.00	0.00	PROA
ATOM	2267	HB1	ALA	P	141	10.796	-1.315	-41.137	0.00	0.00	PROA
ATOM	2268	HB2	ALA	P	141	11.201	-0.737	-39.533	0.00	0.00	PROA
ATOM	2269	HB3	ALA	P	141	9.551	-0.482	-40.190	0.00	0.00	PROA
ATOM	2270	C	ALA	P	141	8.743	-3.122	-40.452	0.00	0.00	PROA
ATOM	2271	O	ALA	P	141	7.602	-3.106	-39.950	0.00	0.00	PROA
ATOM	2272	N	THR	P	142	8.998	-3.706	-41.667	0.00	0.00	PROA
ATOM	2273	HN	THR	P	142	9.887	-3.613	-42.109	0.00	0.00	PROA
ATOM	2274	CA	THR	P	142	7.856	-4.245	-42.454	0.00	0.00	PROA
ATOM	2275	HA	THR	P	142	7.210	-3.393	-42.610	0.00	0.00	PROA
ATOM	2276	CB	THR	P	142	8.295	-4.726	-43.887	0.00	0.00	PROA
ATOM	2277	HB	THR	P	142	9.142	-5.440	-43.806	0.00	0.00	PROA
ATOM	2278	OG1	THR	P	142	8.757	-3.593	-44.616	0.00	0.00	PROA
ATOM	2279	HG1	THR	P	142	7.948	-3.081	-44.686	0.00	0.00	PROA
ATOM	2280	CG2	THR	P	142	7.164	-5.459	-44.706	0.00	0.00	PROA
ATOM	2281	HG21	THR	P	142	7.587	-5.720	-45.699	0.00	0.00	PROA
ATOM	2282	HG22	THR	P	142	6.223	-4.875	-44.799	0.00	0.00	PROA
ATOM	2283	HG23	THR	P	142	6.782	-6.362	-44.184	0.00	0.00	PROA
ATOM	2284	C	THR	P	142	7.152	-5.333	-41.777	0.00	0.00	PROA
ATOM	2285	O	THR	P	142	5.958	-5.476	-41.879	0.00	0.00	PROA
ATOM	2286	N	ASN	P	143	7.877	-6.228	-41.109	0.00	0.00	PROA
ATOM	2287	HN	ASN	P	143	8.872	-6.164	-41.109	0.00	0.00	PROA
ATOM	2288	CA	ASN	P	143	7.258	-7.315	-40.357	0.00	0.00	PROA
ATOM	2289	HA	ASN	P	143	6.278	-7.546	-40.746	0.00	0.00	PROA
ATOM	2290	CB	ASN	P	143	8.098	-8.681	-40.352	0.00	0.00	PROA
ATOM	2291	HB1	ASN	P	143	7.674	-9.528	-39.772	0.00	0.00	PROA
ATOM	2292	HB2	ASN	P	143	9.077	-8.439	-39.884	0.00	0.00	PROA
ATOM	2293	CG	ASN	P	143	8.377	-9.174	-41.696	0.00	0.00	PROA
ATOM	2294	OD1	ASN	P	143	9.503	-9.034	-42.222	0.00	0.00	PROA
ATOM	2295	ND2	ASN	P	143	7.397	-9.844	-42.352	0.00	0.00	PROA
ATOM	2296	HD21	ASN	P	143	7.564	-10.188	-43.276	0.00	0.00	PROA
ATOM	2297	HD22	ASN	P	143	6.572	-10.073	-41.834	0.00	0.00	PROA
ATOM	2298	C	ASN	P	143	6.991	-6.986	-38.877	0.00	0.00	PROA
ATOM	2299	O	ASN	P	143	6.355	-7.739	-38.190	0.00	0.00	PROA
ATOM	2300	N	SER	P	144	7.419	-5.841	-38.303	0.00	0.00	PROA
ATOM	2301	HN	SER	P	144	8.137	-5.390	-38.827	0.00	0.00	PROA
ATOM	2302	CA	SER	P	144	7.121	-5.352	-36.936	0.00	0.00	PROA
ATOM	2303	HA	SER	P	144	6.718	-6.201	-36.404	0.00	0.00	PROA
ATOM	2304	CB	SER	P	144	8.373	-5.055	-36.111	0.00	0.00	PROA
ATOM	2305	HB1	SER	P	144	8.929	-6.013	-36.037	0.00	0.00	PROA
ATOM	2306	HB2	SER	P	144	8.042	-4.714	-35.107	0.00	0.00	PROA
ATOM	2307	OG	SER	P	144	9.221	-4.046	-36.606	0.00	0.00	PROA
ATOM	2308	HG1	SER	P	144	9.605	-4.469	-37.377	0.00	0.00	PROA
ATOM	2309	C	SER	P	144	5.966	-4.379	-36.774	0.00	0.00	PROA
ATOM	2310	O	SER	P	144	5.658	-4.012	-35.618	0.00	0.00	PROA
ATOM	2311	N	GLN	P	145	5.242	-3.957	-37.886	0.00	0.00	PROA
ATOM	2312	HN	GLN	P	145	5.485	-4.344	-38.771	0.00	0.00	PROA
ATOM	2313	CA	GLN	P	145	4.133	-2.998	-37.925	0.00	0.00	PROA
ATOM	2314	HA	GLN	P	145	4.503	-2.025	-37.636	0.00	0.00	PROA

ATOM 2315 CB GLN P 145 3.589 -2.964 -39.434 0.00 0.00 PROA
ATOM 2316 HB1 GLN P 145 2.872 -2.119 -39.510 0.00 0.00 PROA
ATOM 2317 HB2 GLN P 145 3.046 -3.910 -39.641 0.00 0.00 PROA
ATOM 2318 CG GLN P 145 4.666 -2.732 -40.493 0.00 0.00 PROA
ATOM 2319 HG1 GLN P 145 5.336 -3.619 -40.505 0.00 0.00 PROA
ATOM 2320 HG2 GLN P 145 5.322 -1.937 -40.078 0.00 0.00 PROA
ATOM 2321 CD GLN P 145 4.102 -2.423 -41.867 0.00 0.00 PROA
ATOM 2322 OE1 GLN P 145 2.997 -2.863 -42.154 0.00 0.00 PROA
ATOM 2323 NE2 GLN P 145 4.810 -1.722 -42.759 0.00 0.00 PROA
ATOM 2324 HE21 GLN P 145 4.355 -1.514 -43.625 0.00 0.00 PROA
ATOM 2325 HE22 GLN P 145 5.596 -1.212 -42.409 0.00 0.00 PROA
ATOM 2326 C GLN P 145 2.980 -3.273 -37.009 0.00 0.00 PROA
ATOM 2327 O GLN P 145 2.597 -2.446 -36.222 0.00 0.00 PROA
ATOM 2328 N ARG P 146 2.538 -4.529 -37.017 0.00 0.00 PROA
ATOM 2329 HN ARG P 146 2.884 -5.129 -37.734 0.00 0.00 PROA
ATOM 2330 CA ARG P 146 1.449 -5.057 -36.168 0.00 0.00 PROA
ATOM 2331 HA ARG P 146 0.649 -4.357 -36.357 0.00 0.00 PROA
ATOM 2332 CB ARG P 146 0.872 -6.428 -36.532 0.00 0.00 PROA
ATOM 2333 HB1 ARG P 146 1.723 -7.138 -36.600 0.00 0.00 PROA
ATOM 2334 HB2 ARG P 146 0.465 -6.277 -37.554 0.00 0.00 PROA
ATOM 2335 CG ARG P 146 -0.293 -6.932 -35.642 0.00 0.00 PROA
ATOM 2336 HG1 ARG P 146 -1.144 -6.221 -35.572 0.00 0.00 PROA
ATOM 2337 HG2 ARG P 146 0.110 -7.089 -34.619 0.00 0.00 PROA
ATOM 2338 CD ARG P 146 -0.982 -8.287 -35.974 0.00 0.00 PROA
ATOM 2339 HD1 ARG P 146 -0.196 -9.052 -36.152 0.00 0.00 PROA
ATOM 2340 HD2 ARG P 146 -1.710 -8.211 -36.811 0.00 0.00 PROA
ATOM 2341 NE ARG P 146 -1.821 -8.638 -34.812 0.00 0.00 PROA
ATOM 2342 HE ARG P 146 -2.065 -7.892 -34.192 0.00 0.00 PROA
ATOM 2343 CZ ARG P 146 -2.753 -9.558 -34.735 0.00 0.00 PROA
ATOM 2344 NH1 ARG P 146 -2.740 -10.645 -35.478 0.00 0.00 PROA
ATOM 2345 HH11 ARG P 146 -1.993 -10.832 -36.117 0.00 0.00 PROA
ATOM 2346 HH12 ARG P 146 -3.602 -11.076 -35.743 0.00 0.00 PROA
ATOM 2347 NH2 ARG P 146 -3.679 -9.348 -33.801 0.00 0.00 PROA
ATOM 2348 HH21 ARG P 146 -3.856 -8.411 -33.499 0.00 0.00 PROA
ATOM 2349 HH22 ARG P 146 -4.490 -9.925 -33.897 0.00 0.00 PROA
ATOM 2350 C ARG P 146 1.880 -4.955 -34.652 0.00 0.00 PROA
ATOM 2351 O ARG P 146 1.128 -4.352 -33.871 0.00 0.00 PROA
ATOM 2352 N PRO P 147 3.034 -5.396 -34.049 0.00 0.00 PROA
ATOM 2353 CD PRO P 147 3.786 -6.491 -34.689 0.00 0.00 PROA
ATOM 2354 HD1 PRO P 147 4.062 -6.063 -35.677 0.00 0.00 PROA
ATOM 2355 HD2 PRO P 147 3.221 -7.437 -34.832 0.00 0.00 PROA
ATOM 2356 CA PRO P 147 3.409 -5.146 -32.653 0.00 0.00 PROA
ATOM 2357 HA PRO P 147 2.521 -5.330 -32.067 0.00 0.00 PROA
ATOM 2358 CB PRO P 147 4.511 -6.230 -32.410 0.00 0.00 PROA
ATOM 2359 HB1 PRO P 147 4.024 -7.085 -31.893 0.00 0.00 PROA
ATOM 2360 HB2 PRO P 147 5.211 -5.810 -31.657 0.00 0.00 PROA
ATOM 2361 CG PRO P 147 5.014 -6.693 -33.847 0.00 0.00 PROA
ATOM 2362 HG1 PRO P 147 5.933 -6.193 -34.223 0.00 0.00 PROA
ATOM 2363 HG2 PRO P 147 5.332 -7.755 -33.922 0.00 0.00 PROA
ATOM 2364 C PRO P 147 3.797 -3.688 -32.480 0.00 0.00 PROA
ATOM 2365 O PRO P 147 3.488 -3.134 -31.479 0.00 0.00 PROA
ATOM 2366 N ARG P 148 4.432 -3.044 -33.457 0.00 0.00 PROA
ATOM 2367 HN ARG P 148 4.739 -3.526 -34.275 0.00 0.00 PROA
ATOM 2368 CA ARG P 148 4.903 -1.703 -33.243 0.00 0.00 PROA
ATOM 2369 HA ARG P 148 5.604 -1.801 -32.427 0.00 0.00 PROA
ATOM 2370 CB ARG P 148 5.630 -1.195 -34.532 0.00 0.00 PROA
ATOM 2371 HB1 ARG P 148 4.864 -1.260 -35.333 0.00 0.00 PROA
ATOM 2372 HB2 ARG P 148 6.491 -1.861 -34.752 0.00 0.00 PROA
ATOM 2373 CG ARG P 148 6.210 0.226 -34.517 0.00 0.00 PROA
ATOM 2374 HG1 ARG P 148 5.518 1.090 -34.422 0.00 0.00 PROA
ATOM 2375 HG2 ARG P 148 6.725 0.468 -35.471 0.00 0.00 PROA

ATOM 2376 CD ARG P 148 7.055 0.539 -33.232 0.00 0.00 PROA
ATOM 2377 HD1 ARG P 148 6.573 0.336 -32.252 0.00 0.00 PROA
ATOM 2378 HD2 ARG P 148 7.336 1.610 -33.317 0.00 0.00 PROA
ATOM 2379 NE ARG P 148 8.311 -0.276 -33.323 0.00 0.00 PROA
ATOM 2380 HE ARG P 148 8.414 -0.688 -34.229 0.00 0.00 PROA
ATOM 2381 CZ ARG P 148 9.243 -0.366 -32.448 0.00 0.00 PROA
ATOM 2382 NH1 ARG P 148 9.287 0.268 -31.322 0.00 0.00 PROA
ATOM 2383 HH11 ARG P 148 8.439 0.694 -31.007 0.00 0.00 PROA
ATOM 2384 HH12 ARG P 148 10.180 0.281 -30.872 0.00 0.00 PROA
ATOM 2385 NH2 ARG P 148 10.325 -0.998 -32.814 0.00 0.00 PROA
ATOM 2386 HH21 ARG P 148 10.402 -1.174 -33.795 0.00 0.00 PROA
ATOM 2387 HH22 ARG P 148 11.144 -0.788 -32.281 0.00 0.00 PROA
ATOM 2388 C ARG P 148 3.863 -0.674 -32.934 0.00 0.00 PROA
ATOM 2389 O ARG P 148 3.922 0.044 -31.921 0.00 0.00 PROA
ATOM 2390 N LYS P 149 2.750 -0.653 -33.664 0.00 0.00 PROA
ATOM 2391 HN LYS P 149 2.702 -1.171 -34.515 0.00 0.00 PROA
ATOM 2392 CA LYS P 149 1.788 0.345 -33.321 0.00 0.00 PROA
ATOM 2393 HA LYS P 149 2.188 1.253 -32.893 0.00 0.00 PROA
ATOM 2394 CB LYS P 149 1.021 0.761 -34.619 0.00 0.00 PROA
ATOM 2395 HB1 LYS P 149 1.828 0.946 -35.359 0.00 0.00 PROA
ATOM 2396 HB2 LYS P 149 0.400 1.677 -34.518 0.00 0.00 PROA
ATOM 2397 CG LYS P 149 0.079 -0.299 -35.204 0.00 0.00 PROA
ATOM 2398 HG1 LYS P 149 -0.807 -0.389 -34.540 0.00 0.00 PROA
ATOM 2399 HG2 LYS P 149 0.538 -1.310 -35.247 0.00 0.00 PROA
ATOM 2400 CD LYS P 149 -0.509 0.053 -36.558 0.00 0.00 PROA
ATOM 2401 HD1 LYS P 149 -1.149 0.959 -36.495 0.00 0.00 PROA
ATOM 2402 HD2 LYS P 149 -1.240 -0.733 -36.841 0.00 0.00 PROA
ATOM 2403 CE LYS P 149 0.656 0.129 -37.572 0.00 0.00 PROA
ATOM 2404 HE1 LYS P 149 1.441 -0.581 -37.233 0.00 0.00 PROA
ATOM 2405 HE2 LYS P 149 1.018 1.179 -37.556 0.00 0.00 PROA
ATOM 2406 NZ LYS P 149 0.214 -0.145 -38.968 0.00 0.00 PROA
ATOM 2407 HZ1 LYS P 149 -0.191 -1.103 -38.994 0.00 0.00 PROA
ATOM 2408 HZ2 LYS P 149 1.084 -0.136 -39.538 0.00 0.00 PROA
ATOM 2409 HZ3 LYS P 149 -0.542 0.503 -39.268 0.00 0.00 PROA
ATOM 2410 C LYS P 149 0.741 -0.165 -32.388 0.00 0.00 PROA
ATOM 2411 O LYS P 149 -0.198 0.532 -32.037 0.00 0.00 PROA
ATOM 2412 N LEU P 150 0.818 -1.400 -31.926 0.00 0.00 PROA
ATOM 2413 HN LEU P 150 1.512 -2.007 -32.304 0.00 0.00 PROA
ATOM 2414 CA LEU P 150 0.194 -1.983 -30.669 0.00 0.00 PROA
ATOM 2415 HA LEU P 150 -0.732 -1.443 -30.534 0.00 0.00 PROA
ATOM 2416 CB LEU P 150 -0.023 -3.501 -30.823 0.00 0.00 PROA
ATOM 2417 HB1 LEU P 150 0.928 -4.028 -31.052 0.00 0.00 PROA
ATOM 2418 HB2 LEU P 150 -0.636 -3.555 -31.749 0.00 0.00 PROA
ATOM 2419 CG LEU P 150 -0.667 -4.206 -29.610 0.00 0.00 PROA
ATOM 2420 HG LEU P 150 -0.016 -4.049 -28.723 0.00 0.00 PROA
ATOM 2421 CD1 LEU P 150 -1.999 -3.534 -29.148 0.00 0.00 PROA
ATOM 2422 HD11 LEU P 150 -2.322 -3.992 -28.188 0.00 0.00 PROA
ATOM 2423 HD12 LEU P 150 -2.706 -3.862 -29.939 0.00 0.00 PROA
ATOM 2424 HD13 LEU P 150 -1.880 -2.455 -28.911 0.00 0.00 PROA
ATOM 2425 CD2 LEU P 150 -1.002 -5.650 -30.017 0.00 0.00 PROA
ATOM 2426 HD21 LEU P 150 -0.060 -6.238 -30.014 0.00 0.00 PROA
ATOM 2427 HD22 LEU P 150 -1.458 -5.654 -31.031 0.00 0.00 PROA
ATOM 2428 HD23 LEU P 150 -1.534 -6.235 -29.237 0.00 0.00 PROA
ATOM 2429 C LEU P 150 1.037 -1.667 -29.430 0.00 0.00 PROA
ATOM 2430 O LEU P 150 0.472 -1.520 -28.364 0.00 0.00 PROA
ATOM 2431 N LEU P 151 2.387 -1.439 -29.500 0.00 0.00 PROA
ATOM 2432 HN LEU P 151 2.846 -1.509 -30.382 0.00 0.00 PROA
ATOM 2433 CA LEU P 151 3.217 -0.944 -28.439 0.00 0.00 PROA
ATOM 2434 HA LEU P 151 2.873 -1.412 -27.528 0.00 0.00 PROA
ATOM 2435 CB LEU P 151 4.650 -1.559 -28.444 0.00 0.00 PROA
ATOM 2436 HB1 LEU P 151 5.109 -1.433 -29.448 0.00 0.00 PROA

```
ATOM   2437  HB2 LEU P 151       4.503  -2.623 -28.160  0.00  0.00      PROA
ATOM   2438  CG  LEU P 151       5.660  -0.933 -27.432  0.00  0.00      PROA
ATOM   2439  HG  LEU P 151       5.494   0.164 -27.359  0.00  0.00      PROA
ATOM   2440  CD1 LEU P 151       5.462  -1.497 -26.035  0.00  0.00      PROA
ATOM   2441 HD11 LEU P 151       6.423  -1.482 -25.477  0.00  0.00      PROA
ATOM   2442 HD12 LEU P 151       5.254  -2.588 -26.065  0.00  0.00      PROA
ATOM   2443 HD13 LEU P 151       4.702  -1.012 -25.385  0.00  0.00      PROA
ATOM   2444  CD2 LEU P 151       7.121  -1.261 -27.867  0.00  0.00      PROA
ATOM   2445 HD21 LEU P 151       7.838  -0.887 -27.104  0.00  0.00      PROA
ATOM   2446 HD22 LEU P 151       7.299  -0.891 -28.899  0.00  0.00      PROA
ATOM   2447 HD23 LEU P 151       7.320  -2.354 -27.882  0.00  0.00      PROA
ATOM   2448  C   LEU P 151       3.231   0.557 -28.369  0.00  0.00      PROA
ATOM   2449  O   LEU P 151       3.225   1.107 -27.306  0.00  0.00      PROA
ATOM   2450  N   ALA P 152       3.299   1.225 -29.532  0.00  0.00      PROA
ATOM   2451  HN  ALA P 152       3.510   0.798 -30.408  0.00  0.00      PROA
ATOM   2452  CA  ALA P 152       3.123   2.667 -29.717  0.00  0.00      PROA
ATOM   2453  HA  ALA P 152       3.302   2.933 -30.749  0.00  0.00      PROA
ATOM   2454  CB  ALA P 152       1.667   3.003 -29.692  0.00  0.00      PROA
ATOM   2455  HB1 ALA P 152       1.485   4.089 -29.839  0.00  0.00      PROA
ATOM   2456  HB2 ALA P 152       1.148   2.549 -28.821  0.00  0.00      PROA
ATOM   2457  HB3 ALA P 152       1.226   2.507 -30.584  0.00  0.00      PROA
ATOM   2458  C   ALA P 152       4.046   3.547 -28.855  0.00  0.00      PROA
ATOM   2459  O   ALA P 152       5.172   3.073 -28.635  0.00  0.00      PROA
ATOM   2460  N   GLU P 153       3.565   4.773 -28.576  0.00  0.00      PROA
ATOM   2461  HN  GLU P 153       2.695   5.107 -28.931  0.00  0.00      PROA
ATOM   2462  CA  GLU P 153       4.574   5.812 -27.958  0.00  0.00      PROA
ATOM   2463  HA  GLU P 153       5.582   5.445 -27.834  0.00  0.00      PROA
ATOM   2464  CB  GLU P 153       4.684   7.156 -28.795  0.00  0.00      PROA
ATOM   2465  HB1 GLU P 153       5.485   7.720 -28.270  0.00  0.00      PROA
ATOM   2466  HB2 GLU P 153       3.735   7.734 -28.790  0.00  0.00      PROA
ATOM   2467  CG  GLU P 153       5.056   6.829 -30.290  0.00  0.00      PROA
ATOM   2468  HG1 GLU P 153       4.188   6.497 -30.898  0.00  0.00      PROA
ATOM   2469  HG2 GLU P 153       5.731   5.950 -30.377  0.00  0.00      PROA
ATOM   2470  CD  GLU P 153       5.637   8.062 -30.889  0.00  0.00      PROA
ATOM   2471  OE1 GLU P 153       6.125   7.919 -31.973  0.00  0.00      PROA
ATOM   2472  OE2 GLU P 153       5.570   9.172 -30.300  0.00  0.00      PROA
ATOM   2473  C   GLU P 153       4.028   6.267 -26.556  0.00  0.00      PROA
ATOM   2474  O   GLU P 153       4.655   5.969 -25.550  0.00  0.00      PROA
ATOM   2475  N   LYS P 154       2.914   6.901 -26.434  0.00  0.00      PROA
ATOM   2476  HN  LYS P 154       2.475   7.179 -27.285  0.00  0.00      PROA
ATOM   2477  CA  LYS P 154       2.282   7.158 -25.122  0.00  0.00      PROA
ATOM   2478  HA  LYS P 154       2.977   7.699 -24.497  0.00  0.00      PROA
ATOM   2479  CB  LYS P 154       0.915   7.872 -25.163  0.00  0.00      PROA
ATOM   2480  HB1 LYS P 154       0.404   7.635 -24.205  0.00  0.00      PROA
ATOM   2481  HB2 LYS P 154       0.448   7.314 -26.003  0.00  0.00      PROA
ATOM   2482  CG  LYS P 154       0.932   9.398 -25.373  0.00  0.00      PROA
ATOM   2483  HG1 LYS P 154       1.661   9.902 -24.703  0.00  0.00      PROA
ATOM   2484  HG2 LYS P 154      -0.070   9.836 -25.175  0.00  0.00      PROA
ATOM   2485  CD  LYS P 154       1.369   9.763 -26.841  0.00  0.00      PROA
ATOM   2486  HD1 LYS P 154       0.744   9.154 -27.529  0.00  0.00      PROA
ATOM   2487  HD2 LYS P 154       2.399   9.378 -26.997  0.00  0.00      PROA
ATOM   2488  CE  LYS P 154       1.235  11.224 -27.098  0.00  0.00      PROA
ATOM   2489  HE1 LYS P 154       1.823  11.761 -26.323  0.00  0.00      PROA
ATOM   2490  HE2 LYS P 154       0.163  11.490 -26.975  0.00  0.00      PROA
ATOM   2491  NZ  LYS P 154       1.645  11.634 -28.362  0.00  0.00      PROA
ATOM   2492  HZ1 LYS P 154       2.308  10.943 -28.769  0.00  0.00      PROA
ATOM   2493  HZ2 LYS P 154       2.035  12.592 -28.257  0.00  0.00      PROA
ATOM   2494  HZ3 LYS P 154       0.908  11.686 -29.095  0.00  0.00      PROA
ATOM   2495  C   LYS P 154       1.981   5.836 -24.368  0.00  0.00      PROA
ATOM   2496  O   LYS P 154       2.181   5.753 -23.190  0.00  0.00      PROA
ATOM   2497  N   VAL P 155       1.429   4.811 -25.034  0.00  0.00      PROA
```

ATOM	2498	HN	VAL	P	155	1.401	4.760	-26.029	0.00	0.00	PROA
ATOM	2499	CA	VAL	P	155	0.993	3.625	-24.291	0.00	0.00	PROA
ATOM	2500	HA	VAL	P	155	0.188	3.857	-23.609	0.00	0.00	PROA
ATOM	2501	CB	VAL	P	155	0.420	2.732	-25.354	0.00	0.00	PROA
ATOM	2502	HB	VAL	P	155	1.105	2.676	-26.227	0.00	0.00	PROA
ATOM	2503	CG1	VAL	P	155	0.276	1.286	-24.780	0.00	0.00	PROA
ATOM	2504	HG11	VAL	P	155	-0.480	0.792	-25.427	0.00	0.00	PROA
ATOM	2505	HG12	VAL	P	155	-0.223	1.280	-23.787	0.00	0.00	PROA
ATOM	2506	HG13	VAL	P	155	1.112	0.564	-24.894	0.00	0.00	PROA
ATOM	2507	CG2	VAL	P	155	-0.996	3.286	-25.776	0.00	0.00	PROA
ATOM	2508	HG21	VAL	P	155	-1.764	3.199	-24.978	0.00	0.00	PROA
ATOM	2509	HG22	VAL	P	155	-1.348	2.625	-26.596	0.00	0.00	PROA
ATOM	2510	HG23	VAL	P	155	-1.085	4.355	-26.064	0.00	0.00	PROA
ATOM	2511	C	VAL	P	155	2.138	2.821	-23.558	0.00	0.00	PROA
ATOM	2512	O	VAL	P	155	2.055	2.384	-22.428	0.00	0.00	PROA
ATOM	2513	N	VAL	P	156	3.341	2.709	-24.172	0.00	0.00	PROA
ATOM	2514	HN	VAL	P	156	3.452	3.091	-25.086	0.00	0.00	PROA
ATOM	2515	CA	VAL	P	156	4.500	2.114	-23.459	0.00	0.00	PROA
ATOM	2516	HA	VAL	P	156	4.263	1.103	-23.163	0.00	0.00	PROA
ATOM	2517	CB	VAL	P	156	5.698	1.831	-24.469	0.00	0.00	PROA
ATOM	2518	HB	VAL	P	156	5.342	1.253	-25.348	0.00	0.00	PROA
ATOM	2519	CG1	VAL	P	156	6.216	3.157	-25.097	0.00	0.00	PROA
ATOM	2520	HG11	VAL	P	156	5.366	3.653	-25.611	0.00	0.00	PROA
ATOM	2521	HG12	VAL	P	156	7.065	2.949	-25.783	0.00	0.00	PROA
ATOM	2522	HG13	VAL	P	156	6.608	3.802	-24.281	0.00	0.00	PROA
ATOM	2523	CG2	VAL	P	156	6.762	0.932	-23.804	0.00	0.00	PROA
ATOM	2524	HG21	VAL	P	156	7.543	0.766	-24.577	0.00	0.00	PROA
ATOM	2525	HG22	VAL	P	156	6.340	0.008	-23.355	0.00	0.00	PROA
ATOM	2526	HG23	VAL	P	156	7.162	1.480	-22.923	0.00	0.00	PROA
ATOM	2527	C	VAL	P	156	4.861	2.862	-22.158	0.00	0.00	PROA
ATOM	2528	O	VAL	P	156	5.137	2.305	-21.116	0.00	0.00	PROA
ATOM	2529	N	TYR	P	157	4.851	4.179	-22.176	0.00	0.00	PROA
ATOM	2530	HN	TYR	P	157	4.875	4.730	-23.007	0.00	0.00	PROA
ATOM	2531	CA	TYR	P	157	5.173	4.841	-20.908	0.00	0.00	PROA
ATOM	2532	HA	TYR	P	157	6.053	4.305	-20.584	0.00	0.00	PROA
ATOM	2533	CB	TYR	P	157	5.454	6.328	-21.048	0.00	0.00	PROA
ATOM	2534	HB1	TYR	P	157	5.762	6.655	-20.032	0.00	0.00	PROA
ATOM	2535	HB2	TYR	P	157	4.633	6.972	-21.432	0.00	0.00	PROA
ATOM	2536	CG	TYR	P	157	6.661	6.577	-21.934	0.00	0.00	PROA
ATOM	2537	CD1	TYR	P	157	6.381	6.994	-23.268	0.00	0.00	PROA
ATOM	2538	HD1	TYR	P	157	5.376	7.265	-23.556	0.00	0.00	PROA
ATOM	2539	CE1	TYR	P	157	7.470	7.122	-24.141	0.00	0.00	PROA
ATOM	2540	HE1	TYR	P	157	7.233	7.345	-25.171	0.00	0.00	PROA
ATOM	2541	CZ	TYR	P	157	8.795	6.894	-23.673	0.00	0.00	PROA
ATOM	2542	OH	TYR	P	157	9.853	7.223	-24.640	0.00	0.00	PROA
ATOM	2543	HH	TYR	P	157	9.470	7.658	-25.406	0.00	0.00	PROA
ATOM	2544	CD2	TYR	P	157	7.901	6.272	-21.481	0.00	0.00	PROA
ATOM	2545	HD2	TYR	P	157	8.069	5.900	-20.482	0.00	0.00	PROA
ATOM	2546	CE2	TYR	P	157	9.011	6.417	-22.378	0.00	0.00	PROA
ATOM	2547	HE2	TYR	P	157	10.045	6.294	-22.093	0.00	0.00	PROA
ATOM	2548	C	TYR	P	157	4.124	4.615	-19.794	0.00	0.00	PROA
ATOM	2549	O	TYR	P	157	4.378	4.458	-18.619	0.00	0.00	PROA
ATOM	2550	N	VAL	P	158	2.826	4.528	-20.145	0.00	0.00	PROA
ATOM	2551	HN	VAL	P	158	2.529	4.809	-21.054	0.00	0.00	PROA
ATOM	2552	CA	VAL	P	158	1.730	3.972	-19.325	0.00	0.00	PROA
ATOM	2553	HA	VAL	P	158	1.572	4.599	-18.459	0.00	0.00	PROA
ATOM	2554	CB	VAL	P	158	0.404	4.176	-20.067	0.00	0.00	PROA
ATOM	2555	HB	VAL	P	158	0.498	3.629	-21.030	0.00	0.00	PROA
ATOM	2556	CG1	VAL	P	158	-0.735	3.567	-19.287	0.00	0.00	PROA
ATOM	2557	HG11	VAL	P	158	-0.692	2.459	-19.210	0.00	0.00	PROA
ATOM	2558	HG12	VAL	P	158	-1.677	3.763	-19.842	0.00	0.00	PROA

ATOM	2559	HG13	VAL	P	158	-0.850	4.041	-18.289	0.00	0.00	PROA
ATOM	2560	CG2	VAL	P	158	-0.007	5.631	-20.326	0.00	0.00	PROA
ATOM	2561	HG21	VAL	P	158	0.634	6.110	-21.097	0.00	0.00	PROA
ATOM	2562	HG22	VAL	P	158	-0.163	6.227	-19.401	0.00	0.00	PROA
ATOM	2563	HG23	VAL	P	158	-0.964	5.779	-20.869	0.00	0.00	PROA
ATOM	2564	C	VAL	P	158	1.970	2.589	-18.868	0.00	0.00	PROA
ATOM	2565	O	VAL	P	158	1.892	2.349	-17.639	0.00	0.00	PROA
ATOM	2566	N	GLY	P	159	2.389	1.628	-19.760	0.00	0.00	PROA
ATOM	2567	HN	GLY	P	159	2.378	1.838	-20.735	0.00	0.00	PROA
ATOM	2568	CA	GLY	P	159	2.694	0.281	-19.280	0.00	0.00	PROA
ATOM	2569	HA1	GLY	P	159	2.951	-0.393	-20.084	0.00	0.00	PROA
ATOM	2570	HA2	GLY	P	159	1.770	-0.059	-18.838	0.00	0.00	PROA
ATOM	2571	C	GLY	P	159	3.843	0.139	-18.234	0.00	0.00	PROA
ATOM	2572	O	GLY	P	159	3.697	-0.700	-17.285	0.00	0.00	PROA
ATOM	2573	N	VAL	P	160	4.904	0.911	-18.301	0.00	0.00	PROA
ATOM	2574	HN	VAL	P	160	4.853	1.591	-19.029	0.00	0.00	PROA
ATOM	2575	CA	VAL	P	160	5.979	1.077	-17.257	0.00	0.00	PROA
ATOM	2576	HA	VAL	P	160	6.361	0.089	-17.047	0.00	0.00	PROA
ATOM	2577	CB	VAL	P	160	7.017	2.062	-17.821	0.00	0.00	PROA
ATOM	2578	HB	VAL	P	160	6.679	3.067	-18.152	0.00	0.00	PROA
ATOM	2579	CG1	VAL	P	160	8.249	2.334	-16.851	0.00	0.00	PROA
ATOM	2580	HG11	VAL	P	160	9.064	2.897	-17.355	0.00	0.00	PROA
ATOM	2581	HG12	VAL	P	160	8.600	1.324	-16.548	0.00	0.00	PROA
ATOM	2582	HG13	VAL	P	160	7.913	2.828	-15.915	0.00	0.00	PROA
ATOM	2583	CG2	VAL	P	160	7.639	1.446	-19.125	0.00	0.00	PROA
ATOM	2584	HG21	VAL	P	160	7.538	0.351	-18.971	0.00	0.00	PROA
ATOM	2585	HG22	VAL	P	160	8.691	1.799	-19.186	0.00	0.00	PROA
ATOM	2586	HG23	VAL	P	160	6.999	1.589	-20.022	0.00	0.00	PROA
ATOM	2587	C	VAL	P	160	5.481	1.862	-15.955	0.00	0.00	PROA
ATOM	2588	O	VAL	P	160	5.788	1.517	-14.824	0.00	0.00	PROA
ATOM	2589	N	TRP	P	161	4.734	2.935	-16.115	0.00	0.00	PROA
ATOM	2590	HN	TRP	P	161	4.348	3.277	-16.969	0.00	0.00	PROA
ATOM	2591	CA	TRP	P	161	4.296	3.664	-14.966	0.00	0.00	PROA
ATOM	2592	HA	TRP	P	161	5.174	3.601	-14.340	0.00	0.00	PROA
ATOM	2593	CB	TRP	P	161	4.077	5.190	-15.162	0.00	0.00	PROA
ATOM	2594	HB1	TRP	P	161	3.347	5.659	-14.468	0.00	0.00	PROA
ATOM	2595	HB2	TRP	P	161	3.642	5.463	-16.147	0.00	0.00	PROA
ATOM	2596	CG	TRP	P	161	5.386	5.894	-15.113	0.00	0.00	PROA
ATOM	2597	CD1	TRP	P	161	6.251	6.023	-16.209	0.00	0.00	PROA
ATOM	2598	HD1	TRP	P	161	5.878	5.861	-17.209	0.00	0.00	PROA
ATOM	2599	NE1	TRP	P	161	7.382	6.637	-15.750	0.00	0.00	PROA
ATOM	2600	HE1	TRP	P	161	8.067	6.850	-16.413	0.00	0.00	PROA
ATOM	2601	CE2	TRP	P	161	7.252	7.014	-14.490	0.00	0.00	PROA
ATOM	2602	CD2	TRP	P	161	6.060	6.521	-13.973	0.00	0.00	PROA
ATOM	2603	CE3	TRP	P	161	5.787	6.675	-12.600	0.00	0.00	PROA
ATOM	2604	HE3	TRP	P	161	4.835	6.332	-12.223	0.00	0.00	PROA
ATOM	2605	CZ3	TRP	P	161	6.661	7.422	-11.786	0.00	0.00	PROA
ATOM	2606	HZ3	TRP	P	161	6.546	7.644	-10.736	0.00	0.00	PROA
ATOM	2607	CZ2	TRP	P	161	8.164	7.746	-13.635	0.00	0.00	PROA
ATOM	2608	HZ2	TRP	P	161	9.036	8.239	-14.039	0.00	0.00	PROA
ATOM	2609	CH2	TRP	P	161	7.767	8.031	-12.357	0.00	0.00	PROA
ATOM	2610	HH2	TRP	P	161	8.451	8.667	-11.814	0.00	0.00	PROA
ATOM	2611	C	TRP	P	161	3.179	2.993	-14.216	0.00	0.00	PROA
ATOM	2612	O	TRP	P	161	2.851	3.336	-13.042	0.00	0.00	PROA
ATOM	2613	N	ILE	P	162	2.398	2.091	-14.797	0.00	0.00	PROA
ATOM	2614	HN	ILE	P	162	2.454	2.023	-15.790	0.00	0.00	PROA
ATOM	2615	CA	ILE	P	162	1.372	1.261	-14.107	0.00	0.00	PROA
ATOM	2616	HA	ILE	P	162	0.847	2.021	-13.548	0.00	0.00	PROA
ATOM	2617	CB	ILE	P	162	0.359	0.534	-14.931	0.00	0.00	PROA
ATOM	2618	HB	ILE	P	162	0.835	0.153	-15.860	0.00	0.00	PROA
ATOM	2619	CG2	ILE	P	162	-0.352	-0.600	-14.127	0.00	0.00	PROA

ATOM	2620	HG21	ILE	P	162	0.371	-1.441	-14.059	0.00	0.00	PROA
ATOM	2621	HG22	ILE	P	162	-1.334	-0.986	-14.475	0.00	0.00	PROA
ATOM	2622	HG23	ILE	P	162	-0.506	-0.141	-13.127	0.00	0.00	PROA
ATOM	2623	CG1	ILE	P	162	-0.690	1.543	-15.455	0.00	0.00	PROA
ATOM	2624	HG11	ILE	P	162	-0.135	2.403	-15.887	0.00	0.00	PROA
ATOM	2625	HG12	ILE	P	162	-1.330	1.792	-14.582	0.00	0.00	PROA
ATOM	2626	CD	ILE	P	162	-1.416	0.877	-16.640	0.00	0.00	PROA
ATOM	2627	HD1	ILE	P	162	-0.666	0.733	-17.447	0.00	0.00	PROA
ATOM	2628	HD2	ILE	P	162	-2.289	1.439	-17.036	0.00	0.00	PROA
ATOM	2629	HD3	ILE	P	162	-1.892	-0.102	-16.418	0.00	0.00	PROA
ATOM	2630	C	ILE	P	162	1.944	0.496	-12.951	0.00	0.00	PROA
ATOM	2631	O	ILE	P	162	1.425	0.679	-11.780	0.00	0.00	PROA
ATOM	2632	N	PRO	P	163	3.032	-0.245	-13.088	0.00	0.00	PROA
ATOM	2633	CD	PRO	P	163	3.346	-0.911	-14.334	0.00	0.00	PROA
ATOM	2634	HD1	PRO	P	163	4.038	-0.285	-14.937	0.00	0.00	PROA
ATOM	2635	HD2	PRO	P	163	2.462	-1.152	-14.963	0.00	0.00	PROA
ATOM	2636	CA	PRO	P	163	3.452	-1.031	-11.969	0.00	0.00	PROA
ATOM	2637	HA	PRO	P	163	2.647	-1.512	-11.433	0.00	0.00	PROA
ATOM	2638	CB	PRO	P	163	4.441	-1.996	-12.499	0.00	0.00	PROA
ATOM	2639	HB1	PRO	P	163	4.412	-2.926	-11.892	0.00	0.00	PROA
ATOM	2640	HB2	PRO	P	163	5.464	-1.564	-12.469	0.00	0.00	PROA
ATOM	2641	CG	PRO	P	163	4.068	-2.231	-13.918	0.00	0.00	PROA
ATOM	2642	HG1	PRO	P	163	4.997	-2.464	-14.481	0.00	0.00	PROA
ATOM	2643	HG2	PRO	P	163	3.408	-3.115	-14.043	0.00	0.00	PROA
ATOM	2644	C	PRO	P	163	4.040	-0.132	-10.895	0.00	0.00	PROA
ATOM	2645	O	PRO	P	163	4.013	-0.435	-9.679	0.00	0.00	PROA
ATOM	2646	N	ALA	P	164	4.582	0.954	-11.274	0.00	0.00	PROA
ATOM	2647	HN	ALA	P	164	4.526	1.178	-12.244	0.00	0.00	PROA
ATOM	2648	CA	ALA	P	164	5.235	1.925	-10.500	0.00	0.00	PROA
ATOM	2649	HA	ALA	P	164	5.935	1.363	-9.900	0.00	0.00	PROA
ATOM	2650	CB	ALA	P	164	6.014	2.985	-11.300	0.00	0.00	PROA
ATOM	2651	HB1	ALA	P	164	6.554	2.394	-12.070	0.00	0.00	PROA
ATOM	2652	HB2	ALA	P	164	6.708	3.541	-10.635	0.00	0.00	PROA
ATOM	2653	HB3	ALA	P	164	5.388	3.688	-11.891	0.00	0.00	PROA
ATOM	2654	C	ALA	P	164	4.399	2.697	-9.495	0.00	0.00	PROA
ATOM	2655	O	ALA	P	164	4.702	2.772	-8.320	0.00	0.00	PROA
ATOM	2656	N	LEU	P	165	3.190	3.195	-9.913	0.00	0.00	PROA
ATOM	2657	HN	LEU	P	165	3.076	3.142	-10.902	0.00	0.00	PROA
ATOM	2658	CA	LEU	P	165	2.122	3.867	-9.192	0.00	0.00	PROA
ATOM	2659	HA	LEU	P	165	2.552	4.604	-8.530	0.00	0.00	PROA
ATOM	2660	CB	LEU	P	165	1.160	4.518	-10.287	0.00	0.00	PROA
ATOM	2661	HB1	LEU	P	165	0.363	4.963	-9.653	0.00	0.00	PROA
ATOM	2662	HB2	LEU	P	165	0.878	3.753	-11.042	0.00	0.00	PROA
ATOM	2663	CG	LEU	P	165	1.882	5.716	-11.008	0.00	0.00	PROA
ATOM	2664	HG	LEU	P	165	2.686	5.224	-11.595	0.00	0.00	PROA
ATOM	2665	CD1	LEU	P	165	0.858	6.282	-11.957	0.00	0.00	PROA
ATOM	2666	HD11	LEU	P	165	0.281	5.525	-12.529	0.00	0.00	PROA
ATOM	2667	HD12	LEU	P	165	1.407	6.778	-12.786	0.00	0.00	PROA
ATOM	2668	HD13	LEU	P	165	0.123	6.894	-11.391	0.00	0.00	PROA
ATOM	2669	CD2	LEU	P	165	2.457	6.753	-10.046	0.00	0.00	PROA
ATOM	2670	HD21	LEU	P	165	2.772	7.676	-10.578	0.00	0.00	PROA
ATOM	2671	HD22	LEU	P	165	3.329	6.320	-9.511	0.00	0.00	PROA
ATOM	2672	HD23	LEU	P	165	1.704	7.032	-9.279	0.00	0.00	PROA
ATOM	2673	C	LEU	P	165	1.335	2.940	-8.346	0.00	0.00	PROA
ATOM	2674	O	LEU	P	165	0.967	3.217	-7.209	0.00	0.00	PROA
ATOM	2675	N	LEU	P	166	1.113	1.707	-8.841	0.00	0.00	PROA
ATOM	2676	HN	LEU	P	166	1.419	1.447	-9.753	0.00	0.00	PROA
ATOM	2677	CA	LEU	P	166	0.500	0.617	-8.135	0.00	0.00	PROA
ATOM	2678	HA	LEU	P	166	-0.431	1.003	-7.749	0.00	0.00	PROA
ATOM	2679	CB	LEU	P	166	0.332	-0.562	-9.193	0.00	0.00	PROA
ATOM	2680	HB1	LEU	P	166	1.357	-0.822	-9.535	0.00	0.00	PROA

ATOM 2681 HB2 LEU P 166 -0.251 -0.314 -10.106 0.00 0.00 PROA
ATOM 2682 CG LEU P 166 -0.392 -1.810 -8.667 0.00 0.00 PROA
ATOM 2683 HG LEU P 166 0.206 -2.243 -7.837 0.00 0.00 PROA
ATOM 2684 CD1 LEU P 166 -1.785 -1.408 -8.149 0.00 0.00 PROA
ATOM 2685 HD11 LEU P 166 -2.442 -0.992 -8.943 0.00 0.00 PROA
ATOM 2686 HD12 LEU P 166 -1.663 -0.674 -7.325 0.00 0.00 PROA
ATOM 2687 HD13 LEU P 166 -2.213 -2.340 -7.720 0.00 0.00 PROA
ATOM 2688 CD2 LEU P 166 -0.748 -2.868 -9.752 0.00 0.00 PROA
ATOM 2689 HD21 LEU P 166 -1.443 -3.609 -9.302 0.00 0.00 PROA
ATOM 2690 HD22 LEU P 166 0.120 -3.470 -10.096 0.00 0.00 PROA
ATOM 2691 HD23 LEU P 166 -1.179 -2.326 -10.621 0.00 0.00 PROA
ATOM 2692 C LEU P 166 1.252 0.083 -6.916 0.00 0.00 PROA
ATOM 2693 O LEU P 166 0.619 -0.102 -5.859 0.00 0.00 PROA
ATOM 2694 N LEU P 167 2.587 0.009 -6.958 0.00 0.00 PROA
ATOM 2695 HN LEU P 167 3.149 0.163 -7.766 0.00 0.00 PROA
ATOM 2696 CA LEU P 167 3.332 -0.411 -5.797 0.00 0.00 PROA
ATOM 2697 HA LEU P 167 2.704 -0.834 -5.026 0.00 0.00 PROA
ATOM 2698 CB LEU P 167 4.309 -1.590 -6.257 0.00 0.00 PROA
ATOM 2699 HB1 LEU P 167 4.664 -2.081 -5.325 0.00 0.00 PROA
ATOM 2700 HB2 LEU P 167 5.107 -1.113 -6.864 0.00 0.00 PROA
ATOM 2701 CG LEU P 167 3.611 -2.758 -6.995 0.00 0.00 PROA
ATOM 2702 HG LEU P 167 3.199 -2.304 -7.921 0.00 0.00 PROA
ATOM 2703 CD1 LEU P 167 4.588 -3.790 -7.540 0.00 0.00 PROA
ATOM 2704 HD11 LEU P 167 4.233 -4.255 -8.483 0.00 0.00 PROA
ATOM 2705 HD12 LEU P 167 4.736 -4.572 -6.764 0.00 0.00 PROA
ATOM 2706 HD13 LEU P 167 5.596 -3.384 -7.774 0.00 0.00 PROA
ATOM 2707 CD2 LEU P 167 2.468 -3.363 -6.124 0.00 0.00 PROA
ATOM 2708 HD21 LEU P 167 2.795 -3.763 -5.140 0.00 0.00 PROA
ATOM 2709 HD22 LEU P 167 2.104 -4.307 -6.582 0.00 0.00 PROA
ATOM 2710 HD23 LEU P 167 1.651 -2.634 -5.936 0.00 0.00 PROA
ATOM 2711 C LEU P 167 4.060 0.735 -5.110 0.00 0.00 PROA
ATOM 2712 O LEU P 167 4.690 0.484 -4.115 0.00 0.00 PROA
ATOM 2713 N THR P 168 3.898 1.978 -5.558 0.00 0.00 PROA
ATOM 2714 HN THR P 168 3.411 2.204 -6.398 0.00 0.00 PROA
ATOM 2715 CA THR P 168 4.126 3.150 -4.660 0.00 0.00 PROA
ATOM 2716 HA THR P 168 4.946 2.901 -4.004 0.00 0.00 PROA
ATOM 2717 CB THR P 168 4.587 4.477 -5.264 0.00 0.00 PROA
ATOM 2718 HB THR P 168 4.403 5.381 -4.646 0.00 0.00 PROA
ATOM 2719 OG1 THR P 168 3.986 4.686 -6.542 0.00 0.00 PROA
ATOM 2720 HG1 THR P 168 4.442 4.140 -7.187 0.00 0.00 PROA
ATOM 2721 CG2 THR P 168 6.090 4.291 -5.506 0.00 0.00 PROA
ATOM 2722 HG21 THR P 168 6.572 5.221 -5.876 0.00 0.00 PROA
ATOM 2723 HG22 THR P 168 6.310 3.436 -6.181 0.00 0.00 PROA
ATOM 2724 HG23 THR P 168 6.539 4.115 -4.505 0.00 0.00 PROA
ATOM 2725 C THR P 168 2.932 3.514 -3.736 0.00 0.00 PROA
ATOM 2726 O THR P 168 3.102 4.246 -2.803 0.00 0.00 PROA
ATOM 2727 N ILE P 169 1.704 2.910 -3.985 0.00 0.00 PROA
ATOM 2728 HN ILE P 169 1.443 2.562 -4.882 0.00 0.00 PROA
ATOM 2729 CA ILE P 169 0.614 2.994 -3.017 0.00 0.00 PROA
ATOM 2730 HA ILE P 169 0.338 4.037 -2.970 0.00 0.00 PROA
ATOM 2731 CB ILE P 169 -0.721 2.355 -3.439 0.00 0.00 PROA
ATOM 2732 HB ILE P 169 -0.525 1.264 -3.508 0.00 0.00 PROA
ATOM 2733 CG2 ILE P 169 -1.822 2.662 -2.397 0.00 0.00 PROA
ATOM 2734 HG21 ILE P 169 -1.941 3.730 -2.114 0.00 0.00 PROA
ATOM 2735 HG22 ILE P 169 -1.637 2.110 -1.451 0.00 0.00 PROA
ATOM 2736 HG23 ILE P 169 -2.758 2.229 -2.810 0.00 0.00 PROA
ATOM 2737 CG1 ILE P 169 -1.208 2.872 -4.778 0.00 0.00 PROA
ATOM 2738 HG11 ILE P 169 -0.402 2.793 -5.538 0.00 0.00 PROA
ATOM 2739 HG12 ILE P 169 -1.572 3.913 -4.644 0.00 0.00 PROA
ATOM 2740 CD ILE P 169 -2.437 2.206 -5.399 0.00 0.00 PROA
ATOM 2741 HD1 ILE P 169 -2.283 2.216 -6.500 0.00 0.00 PROA

ATOM	2742	HD2	ILE	P	169	-3.323	2.794	-5.077	0.00	0.00	PROA
ATOM	2743	HD3	ILE	P	169	-2.543	1.162	-5.035	0.00	0.00	PROA
ATOM	2744	C	ILE	P	169	0.938	2.514	-1.581	0.00	0.00	PROA
ATOM	2745	O	ILE	P	169	0.567	3.244	-0.684	0.00	0.00	PROA
ATOM	2746	N	PRO	P	170	1.697	1.407	-1.346	0.00	0.00	PROA
ATOM	2747	CD	PRO	P	170	2.059	0.221	-2.163	0.00	0.00	PROA
ATOM	2748	HD1	PRO	P	170	2.514	0.612	-3.098	0.00	0.00	PROA
ATOM	2749	HD2	PRO	P	170	1.145	-0.370	-2.387	0.00	0.00	PROA
ATOM	2750	CA	PRO	P	170	2.199	1.258	0.069	0.00	0.00	PROA
ATOM	2751	HA	PRO	P	170	1.427	1.535	0.772	0.00	0.00	PROA
ATOM	2752	CB	PRO	P	170	2.595	-0.198	0.169	0.00	0.00	PROA
ATOM	2753	HB1	PRO	P	170	1.702	-0.801	0.438	0.00	0.00	PROA
ATOM	2754	HB2	PRO	P	170	3.400	-0.396	0.909	0.00	0.00	PROA
ATOM	2755	CG	PRO	P	170	3.021	-0.558	-1.278	0.00	0.00	PROA
ATOM	2756	HG1	PRO	P	170	4.068	-0.274	-1.515	0.00	0.00	PROA
ATOM	2757	HG2	PRO	P	170	2.981	-1.646	-1.499	0.00	0.00	PROA
ATOM	2758	C	PRO	P	170	3.282	2.210	0.519	0.00	0.00	PROA
ATOM	2759	O	PRO	P	170	3.335	2.738	1.633	0.00	0.00	PROA
ATOM	2760	N	ASP	P	171	4.220	2.622	-0.336	0.00	0.00	PROA
ATOM	2761	HN	ASP	P	171	4.216	2.251	-1.261	0.00	0.00	PROA
ATOM	2762	CA	ASP	P	171	5.271	3.549	0.049	0.00	0.00	PROA
ATOM	2763	HA	ASP	P	171	5.780	3.186	0.930	0.00	0.00	PROA
ATOM	2764	CB	ASP	P	171	6.262	3.674	-1.091	0.00	0.00	PROA
ATOM	2765	HB1	ASP	P	171	7.053	4.415	-0.844	0.00	0.00	PROA
ATOM	2766	HB2	ASP	P	171	5.980	3.868	-2.148	0.00	0.00	PROA
ATOM	2767	CG	ASP	P	171	6.962	2.310	-1.160	0.00	0.00	PROA
ATOM	2768	OD1	ASP	P	171	7.484	1.787	-0.182	0.00	0.00	PROA
ATOM	2769	OD2	ASP	P	171	7.104	1.908	-2.344	0.00	0.00	PROA
ATOM	2770	C	ASP	P	171	4.820	4.941	0.460	0.00	0.00	PROA
ATOM	2771	O	ASP	P	171	5.291	5.519	1.480	0.00	0.00	PROA
ATOM	2772	N	PHE	P	172	3.778	5.476	-0.199	0.00	0.00	PROA
ATOM	2773	HN	PHE	P	172	3.258	4.981	-0.891	0.00	0.00	PROA
ATOM	2774	CA	PHE	P	172	3.175	6.711	0.242	0.00	0.00	PROA
ATOM	2775	HA	PHE	P	172	4.018	7.366	0.402	0.00	0.00	PROA
ATOM	2776	CB	PHE	P	172	2.301	7.328	-0.921	0.00	0.00	PROA
ATOM	2777	HB1	PHE	P	172	1.520	6.615	-1.260	0.00	0.00	PROA
ATOM	2778	HB2	PHE	P	172	2.938	7.440	-1.825	0.00	0.00	PROA
ATOM	2779	CG	PHE	P	172	1.555	8.606	-0.375	0.00	0.00	PROA
ATOM	2780	CD1	PHE	P	172	2.125	9.853	-0.359	0.00	0.00	PROA
ATOM	2781	HD1	PHE	P	172	3.153	9.914	-0.683	0.00	0.00	PROA
ATOM	2782	CE1	PHE	P	172	1.473	10.974	0.119	0.00	0.00	PROA
ATOM	2783	HE1	PHE	P	172	1.979	11.928	0.119	0.00	0.00	PROA
ATOM	2784	CZ	PHE	P	172	0.100	10.803	0.615	0.00	0.00	PROA
ATOM	2785	HZ	PHE	P	172	-0.456	11.663	0.957	0.00	0.00	PROA
ATOM	2786	CD2	PHE	P	172	0.225	8.489	0.028	0.00	0.00	PROA
ATOM	2787	HD2	PHE	P	172	-0.325	7.568	-0.095	0.00	0.00	PROA
ATOM	2788	CE2	PHE	P	172	-0.452	9.536	0.458	0.00	0.00	PROA
ATOM	2789	HE2	PHE	P	172	-1.459	9.260	0.734	0.00	0.00	PROA
ATOM	2790	C	PHE	P	172	2.405	6.534	1.563	0.00	0.00	PROA
ATOM	2791	O	PHE	P	172	2.555	7.316	2.486	0.00	0.00	PROA
ATOM	2792	N	ILE	P	173	1.657	5.456	1.749	0.00	0.00	PROA
ATOM	2793	HN	ILE	P	173	1.539	4.701	1.108	0.00	0.00	PROA
ATOM	2794	CA	ILE	P	173	0.878	5.209	2.932	0.00	0.00	PROA
ATOM	2795	HA	ILE	P	173	0.334	6.124	3.116	0.00	0.00	PROA
ATOM	2796	CB	ILE	P	173	-0.173	4.029	2.691	0.00	0.00	PROA
ATOM	2797	HB	ILE	P	173	0.315	3.255	2.061	0.00	0.00	PROA
ATOM	2798	CG2	ILE	P	173	-0.728	3.457	3.961	0.00	0.00	PROA
ATOM	2799	HG21	ILE	P	173	0.125	3.253	4.643	0.00	0.00	PROA
ATOM	2800	HG22	ILE	P	173	-1.294	2.538	3.700	0.00	0.00	PROA
ATOM	2801	HG23	ILE	P	173	-1.441	4.194	4.388	0.00	0.00	PROA
ATOM	2802	CG1	ILE	P	173	-1.408	4.456	1.743	0.00	0.00	PROA

ATOM	2803	HG11	ILE	P	173	-0.866	4.634	0.790	0.00	0.00	PROA
ATOM	2804	HG12	ILE	P	173	-1.878	5.447	1.925	0.00	0.00	PROA
ATOM	2805	CD	ILE	P	173	-2.425	3.324	1.499	0.00	0.00	PROA
ATOM	2806	HD1	ILE	P	173	-3.198	3.845	0.895	0.00	0.00	PROA
ATOM	2807	HD2	ILE	P	173	-2.770	2.829	2.432	0.00	0.00	PROA
ATOM	2808	HD3	ILE	P	173	-1.923	2.565	0.861	0.00	0.00	PROA
ATOM	2809	C	ILE	P	173	1.637	5.012	4.249	0.00	0.00	PROA
ATOM	2810	O	ILE	P	173	1.334	5.661	5.244	0.00	0.00	PROA
ATOM	2811	N	PHE	P	174	2.588	4.126	4.215	0.00	0.00	PROA
ATOM	2812	HN	PHE	P	174	2.894	3.843	3.310	0.00	0.00	PROA
ATOM	2813	CA	PHE	P	174	3.143	3.548	5.415	0.00	0.00	PROA
ATOM	2814	HA	PHE	P	174	2.444	3.726	6.219	0.00	0.00	PROA
ATOM	2815	CB	PHE	P	174	3.354	1.991	5.340	0.00	0.00	PROA
ATOM	2816	HB1	PHE	P	174	3.825	1.709	6.306	0.00	0.00	PROA
ATOM	2817	HB2	PHE	P	174	4.157	1.747	4.612	0.00	0.00	PROA
ATOM	2818	CG	PHE	P	174	2.125	1.152	5.034	0.00	0.00	PROA
ATOM	2819	CD1	PHE	P	174	1.033	1.111	5.913	0.00	0.00	PROA
ATOM	2820	HD1	PHE	P	174	1.023	1.743	6.788	0.00	0.00	PROA
ATOM	2821	CE1	PHE	P	174	0.076	0.106	5.819	0.00	0.00	PROA
ATOM	2822	HE1	PHE	P	174	-0.753	0.065	6.510	0.00	0.00	PROA
ATOM	2823	CZ	PHE	P	174	0.132	-0.773	4.730	0.00	0.00	PROA
ATOM	2824	HZ	PHE	P	174	-0.697	-1.465	4.698	0.00	0.00	PROA
ATOM	2825	CD2	PHE	P	174	2.105	0.329	3.916	0.00	0.00	PROA
ATOM	2826	HD2	PHE	P	174	2.938	0.303	3.229	0.00	0.00	PROA
ATOM	2827	CE2	PHE	P	174	1.150	-0.661	3.778	0.00	0.00	PROA
ATOM	2828	HE2	PHE	P	174	1.303	-1.374	2.982	0.00	0.00	PROA
ATOM	2829	C	PHE	P	174	4.422	4.220	5.939	0.00	0.00	PROA
ATOM	2830	O	PHE	P	174	4.746	3.981	7.126	0.00	0.00	PROA
ATOM	2831	N	ALA	P	175	5.186	4.968	5.157	0.00	0.00	PROA
ATOM	2832	HN	ALA	P	175	4.863	5.174	4.237	0.00	0.00	PROA
ATOM	2833	CA	ALA	P	175	6.336	5.801	5.605	0.00	0.00	PROA
ATOM	2834	HA	ALA	P	175	7.013	5.087	6.050	0.00	0.00	PROA
ATOM	2835	CB	ALA	P	175	7.090	6.455	4.456	0.00	0.00	PROA
ATOM	2836	HB1	ALA	P	175	7.802	7.226	4.819	0.00	0.00	PROA
ATOM	2837	HB2	ALA	P	175	6.420	6.982	3.743	0.00	0.00	PROA
ATOM	2838	HB3	ALA	P	175	7.459	5.625	3.815	0.00	0.00	PROA
ATOM	2839	C	ALA	P	175	5.959	6.915	6.626	0.00	0.00	PROA
ATOM	2840	O	ALA	P	175	5.023	7.698	6.378	0.00	0.00	PROA
ATOM	2841	N	ASN	P	176	6.711	6.930	7.746	0.00	0.00	PROA
ATOM	2842	HN	ASN	P	176	7.502	6.324	7.758	0.00	0.00	PROA
ATOM	2843	CA	ASN	P	176	6.698	7.825	8.860	0.00	0.00	PROA
ATOM	2844	HA	ASN	P	176	6.444	8.792	8.451	0.00	0.00	PROA
ATOM	2845	CB	ASN	P	176	5.663	7.211	9.826	0.00	0.00	PROA
ATOM	2846	HB1	ASN	P	176	5.747	7.731	10.804	0.00	0.00	PROA
ATOM	2847	HB2	ASN	P	176	5.856	6.123	9.941	0.00	0.00	PROA
ATOM	2848	CG	ASN	P	176	4.188	7.403	9.327	0.00	0.00	PROA
ATOM	2849	OD1	ASN	P	176	3.622	8.522	9.349	0.00	0.00	PROA
ATOM	2850	ND2	ASN	P	176	3.434	6.286	9.018	0.00	0.00	PROA
ATOM	2851	HD21	ASN	P	176	2.500	6.445	8.698	0.00	0.00	PROA
ATOM	2852	HD22	ASN	P	176	3.941	5.432	8.894	0.00	0.00	PROA
ATOM	2853	C	ASN	P	176	8.079	7.932	9.395	0.00	0.00	PROA
ATOM	2854	O	ASN	P	176	9.035	7.195	9.042	0.00	0.00	PROA
ATOM	2855	N	VAL	P	177	8.220	8.814	10.354	0.00	0.00	PROA
ATOM	2856	HN	VAL	P	177	7.567	9.547	10.529	0.00	0.00	PROA
ATOM	2857	CA	VAL	P	177	9.485	8.816	11.012	0.00	0.00	PROA
ATOM	2858	HA	VAL	P	177	10.260	8.241	10.528	0.00	0.00	PROA
ATOM	2859	CB	VAL	P	177	10.002	10.230	11.032	0.00	0.00	PROA
ATOM	2860	HB	VAL	P	177	9.165	10.863	11.397	0.00	0.00	PROA
ATOM	2861	CG1	VAL	P	177	11.171	10.289	12.016	0.00	0.00	PROA
ATOM	2862	HG11	VAL	P	177	11.544	11.334	11.958	0.00	0.00	PROA
ATOM	2863	HG12	VAL	P	177	11.965	9.588	11.679	0.00	0.00	PROA

```
ATOM   2864 HG13 VAL P 177      10.950  10.201  13.101  0.00  0.00      PROA
ATOM   2865  CG2 VAL P 177      10.577  10.633   9.652  0.00  0.00      PROA
ATOM   2866 HG21 VAL P 177       9.855  10.436   8.831  0.00  0.00      PROA
ATOM   2867 HG22 VAL P 177      11.459  10.033   9.346  0.00  0.00      PROA
ATOM   2868 HG23 VAL P 177      10.981  11.664   9.567  0.00  0.00      PROA
ATOM   2869  C   VAL P 177       9.348   8.342  12.383  0.00  0.00      PROA
ATOM   2870  O   VAL P 177       8.642   8.923  13.190  0.00  0.00      PROA
ATOM   2871  N   SER P 178      10.053   7.268  12.763  0.00  0.00      PROA
ATOM   2872  HN  SER P 178      10.580   6.826  12.041  0.00  0.00      PROA
ATOM   2873  CA  SER P 178      10.134   6.811  14.143  0.00  0.00      PROA
ATOM   2874  HA  SER P 178       9.136   6.914  14.542  0.00  0.00      PROA
ATOM   2875  CB  SER P 178      10.733   5.405  14.285  0.00  0.00      PROA
ATOM   2876  HB1 SER P 178      11.744   5.343  13.829  0.00  0.00      PROA
ATOM   2877  HB2 SER P 178      10.109   4.653  13.758  0.00  0.00      PROA
ATOM   2878  OG  SER P 178      10.733   4.945  15.614  0.00  0.00      PROA
ATOM   2879  HG1 SER P 178      11.550   5.289  15.984  0.00  0.00      PROA
ATOM   2880  C   SER P 178      11.055   7.802  14.831  0.00  0.00      PROA
ATOM   2881  O   SER P 178      12.195   8.058  14.392  0.00  0.00      PROA
ATOM   2882  N   GLU P 179      10.463   8.364  15.919  0.00  0.00      PROA
ATOM   2883  HN  GLU P 179       9.628   7.961  16.286  0.00  0.00      PROA
ATOM   2884  CA  GLU P 179      11.056   9.323  16.741  0.00  0.00      PROA
ATOM   2885  HA  GLU P 179      11.927   9.761  16.278  0.00  0.00      PROA
ATOM   2886  CB  GLU P 179       9.967  10.355  17.109  0.00  0.00      PROA
ATOM   2887  HB1 GLU P 179      10.450  11.106  17.770  0.00  0.00      PROA
ATOM   2888  HB2 GLU P 179       9.159   9.916  17.732  0.00  0.00      PROA
ATOM   2889  CG  GLU P 179       9.410  11.049  15.854  0.00  0.00      PROA
ATOM   2890  HG1 GLU P 179       8.858  10.305  15.241  0.00  0.00      PROA
ATOM   2891  HG2 GLU P 179      10.172  11.504  15.186  0.00  0.00      PROA
ATOM   2892  CD  GLU P 179       8.306  12.076  16.201  0.00  0.00      PROA
ATOM   2893  OE1 GLU P 179       7.109  11.982  15.833  0.00  0.00      PROA
ATOM   2894  OE2 GLU P 179       8.663  13.121  16.801  0.00  0.00      PROA
ATOM   2895  C   GLU P 179      11.622   8.727  18.030  0.00  0.00      PROA
ATOM   2896  O   GLU P 179      12.376   9.455  18.715  0.00  0.00      PROA
ATOM   2897  N   ALA P 180      11.194   7.521  18.528  0.00  0.00      PROA
ATOM   2898  HN  ALA P 180      10.366   7.173  18.096  0.00  0.00      PROA
ATOM   2899  CA  ALA P 180      11.714   6.797  19.662  0.00  0.00      PROA
ATOM   2900  HA  ALA P 180      11.308   7.171  20.591  0.00  0.00      PROA
ATOM   2901  CB  ALA P 180      11.252   5.374  19.481  0.00  0.00      PROA
ATOM   2902  HB1 ALA P 180      11.848   4.863  18.695  0.00  0.00      PROA
ATOM   2903  HB2 ALA P 180      10.176   5.314  19.210  0.00  0.00      PROA
ATOM   2904  HB3 ALA P 180      11.460   4.767  20.388  0.00  0.00      PROA
ATOM   2905  C   ALA P 180      13.200   6.823  19.907  0.00  0.00      PROA
ATOM   2906  O   ALA P 180      13.940   6.728  18.901  0.00  0.00      PROA
ATOM   2907  N   ASP P 181      13.707   6.945  21.221  0.00  0.00      PROA
ATOM   2908  HN  ASP P 181      13.047   7.194  21.925  0.00  0.00      PROA
ATOM   2909  CA  ASP P 181      15.079   6.609  21.606  0.00  0.00      PROA
ATOM   2910  HA  ASP P 181      15.143   6.737  22.676  0.00  0.00      PROA
ATOM   2911  CB  ASP P 181      15.247   5.095  21.563  0.00  0.00      PROA
ATOM   2912  HB1 ASP P 181      16.298   4.897  21.863  0.00  0.00      PROA
ATOM   2913  HB2 ASP P 181      15.132   4.712  20.526  0.00  0.00      PROA
ATOM   2914  CG  ASP P 181      14.314   4.310  22.482  0.00  0.00      PROA
ATOM   2915  OD1 ASP P 181      13.554   4.994  23.235  0.00  0.00      PROA
ATOM   2916  OD2 ASP P 181      14.286   3.029  22.430  0.00  0.00      PROA
ATOM   2917  C   ASP P 181      16.282   7.379  21.001  0.00  0.00      PROA
ATOM   2918  O   ASP P 181      16.286   8.581  20.910  0.00  0.00      PROA
ATOM   2919  N   ASP P 182      17.412   6.691  20.585  0.00  0.00      PROA
ATOM   2920  HN  ASP P 182      17.466   5.704  20.716  0.00  0.00      PROA
ATOM   2921  CA  ASP P 182      18.673   7.268  20.159  0.00  0.00      PROA
ATOM   2922  HA  ASP P 182      19.066   7.803  21.011  0.00  0.00      PROA
ATOM   2923  CB  ASP P 182      19.555   6.099  19.715  0.00  0.00      PROA
ATOM   2924  HB1 ASP P 182      20.485   6.404  19.189  0.00  0.00      PROA
```

ATOM	2925	HB2	ASP	P	182	18.949	5.517	18.989	0.00	0.00	PROA
ATOM	2926	CG	ASP	P	182	19.929	5.195	20.861	0.00	0.00	PROA
ATOM	2927	OD1	ASP	P	182	19.612	5.461	22.052	0.00	0.00	PROA
ATOM	2928	OD2	ASP	P	182	20.486	4.101	20.544	0.00	0.00	PROA
ATOM	2929	C	ASP	P	182	18.745	8.302	19.033	0.00	0.00	PROA
ATOM	2930	O	ASP	P	182	19.391	9.372	19.107	0.00	0.00	PROA
ATOM	2931	N	ARG	P	183	18.051	7.989	17.927	0.00	0.00	PROA
ATOM	2932	HN	ARG	P	183	17.695	7.082	17.714	0.00	0.00	PROA
ATOM	2933	CA	ARG	P	183	17.963	8.846	16.780	0.00	0.00	PROA
ATOM	2934	HA	ARG	P	183	18.001	9.878	17.097	0.00	0.00	PROA
ATOM	2935	CB	ARG	P	183	19.164	8.514	15.854	0.00	0.00	PROA
ATOM	2936	HB1	ARG	P	183	19.992	8.930	16.467	0.00	0.00	PROA
ATOM	2937	HB2	ARG	P	183	19.171	9.252	15.024	0.00	0.00	PROA
ATOM	2938	CG	ARG	P	183	19.445	7.082	15.281	0.00	0.00	PROA
ATOM	2939	HG1	ARG	P	183	18.511	6.481	15.245	0.00	0.00	PROA
ATOM	2940	HG2	ARG	P	183	20.023	6.559	16.073	0.00	0.00	PROA
ATOM	2941	CD	ARG	P	183	20.229	7.090	13.989	0.00	0.00	PROA
ATOM	2942	HD1	ARG	P	183	20.518	6.061	13.686	0.00	0.00	PROA
ATOM	2943	HD2	ARG	P	183	21.063	7.823	14.015	0.00	0.00	PROA
ATOM	2944	NE	ARG	P	183	19.261	7.530	13.003	0.00	0.00	PROA
ATOM	2945	HE	ARG	P	183	18.343	7.884	13.183	0.00	0.00	PROA
ATOM	2946	CZ	ARG	P	183	19.364	7.366	11.693	0.00	0.00	PROA
ATOM	2947	NH1	ARG	P	183	20.363	6.826	11.104	0.00	0.00	PROA
ATOM	2948	HH11	ARG	P	183	21.219	6.643	11.589	0.00	0.00	PROA
ATOM	2949	HH12	ARG	P	183	20.336	6.502	10.158	0.00	0.00	PROA
ATOM	2950	NH2	ARG	P	183	18.302	7.646	10.961	0.00	0.00	PROA
ATOM	2951	HH21	ARG	P	183	17.546	8.127	11.405	0.00	0.00	PROA
ATOM	2952	HH22	ARG	P	183	18.308	7.270	10.034	0.00	0.00	PROA
ATOM	2953	C	ARG	P	183	16.548	8.736	16.055	0.00	0.00	PROA
ATOM	2954	O	ARG	P	183	15.749	7.819	16.122	0.00	0.00	PROA
ATOM	2955	N	TYR	P	184	16.182	9.727	15.237	0.00	0.00	PROA
ATOM	2956	HN	TYR	P	184	16.775	10.525	15.155	0.00	0.00	PROA
ATOM	2957	CA	TYR	P	184	15.078	9.591	14.296	0.00	0.00	PROA
ATOM	2958	HA	TYR	P	184	14.234	9.309	14.907	0.00	0.00	PROA
ATOM	2959	CB	TYR	P	184	14.876	10.972	13.634	0.00	0.00	PROA
ATOM	2960	HB1	TYR	P	184	14.144	10.885	12.804	0.00	0.00	PROA
ATOM	2961	HB2	TYR	P	184	15.882	11.236	13.241	0.00	0.00	PROA
ATOM	2962	CG	TYR	P	184	14.476	12.024	14.538	0.00	0.00	PROA
ATOM	2963	CD1	TYR	P	184	13.095	12.077	14.797	0.00	0.00	PROA
ATOM	2964	HD1	TYR	P	184	12.480	11.445	14.173	0.00	0.00	PROA
ATOM	2965	CE1	TYR	P	184	12.537	13.173	15.526	0.00	0.00	PROA
ATOM	2966	HE1	TYR	P	184	11.463	13.238	15.612	0.00	0.00	PROA
ATOM	2967	CZ	TYR	P	184	13.420	14.146	16.113	0.00	0.00	PROA
ATOM	2968	OH	TYR	P	184	12.882	15.280	16.741	0.00	0.00	PROA
ATOM	2969	HH	TYR	P	184	11.938	15.178	16.884	0.00	0.00	PROA
ATOM	2970	CD2	TYR	P	184	15.331	13.132	14.972	0.00	0.00	PROA
ATOM	2971	HD2	TYR	P	184	16.393	13.056	14.790	0.00	0.00	PROA
ATOM	2972	CE2	TYR	P	184	14.768	14.176	15.744	0.00	0.00	PROA
ATOM	2973	HE2	TYR	P	184	15.428	15.008	15.945	0.00	0.00	PROA
ATOM	2974	C	TYR	P	184	15.404	8.562	13.244	0.00	0.00	PROA
ATOM	2975	O	TYR	P	184	16.570	8.417	12.811	0.00	0.00	PROA
ATOM	2976	N	ILE	P	185	14.390	7.698	12.838	0.00	0.00	PROA
ATOM	2977	HN	ILE	P	185	13.560	7.754	13.387	0.00	0.00	PROA
ATOM	2978	CA	ILE	P	185	14.609	6.652	11.839	0.00	0.00	PROA
ATOM	2979	HA	ILE	P	185	15.541	6.802	11.315	0.00	0.00	PROA
ATOM	2980	CB	ILE	P	185	14.742	5.246	12.498	0.00	0.00	PROA
ATOM	2981	HB	ILE	P	185	13.851	4.897	13.063	0.00	0.00	PROA
ATOM	2982	CG2	ILE	P	185	15.030	4.136	11.476	0.00	0.00	PROA
ATOM	2983	HG21	ILE	P	185	14.197	4.085	10.743	0.00	0.00	PROA
ATOM	2984	HG22	ILE	P	185	15.091	3.098	11.867	0.00	0.00	PROA
ATOM	2985	HG23	ILE	P	185	15.961	4.407	10.933	0.00	0.00	PROA

```
ATOM   2986  CG1 ILE P 185      15.907   5.380  13.471  0.00  0.00      PROA
ATOM   2987 HG11 ILE P 185      15.690   6.089  14.299  0.00  0.00      PROA
ATOM   2988 HG12 ILE P 185      16.812   5.637  12.881  0.00  0.00      PROA
ATOM   2989  CD  ILE P 185      16.216   4.064  14.229  0.00  0.00      PROA
ATOM   2990  HD1 ILE P 185      15.262   3.744  14.701  0.00  0.00      PROA
ATOM   2991  HD2 ILE P 185      16.915   4.373  15.035  0.00  0.00      PROA
ATOM   2992  HD3 ILE P 185      16.563   3.254  13.553  0.00  0.00      PROA
ATOM   2993  C   ILE P 185      13.365   6.676  10.892  0.00  0.00      PROA
ATOM   2994  O   ILE P 185      12.197   6.435  11.286  0.00  0.00      PROA
ATOM   2995  N   CYS P 186      13.580   6.940   9.548  0.00  0.00      PROA
ATOM   2996  HN  CYS P 186      14.538   6.865   9.281  0.00  0.00      PROA
ATOM   2997  CA  CYS P 186      12.638   6.860   8.477  0.00  0.00      PROA
ATOM   2998  HA  CYS P 186      11.810   7.498   8.747  0.00  0.00      PROA
ATOM   2999  CB  CYS P 186      13.281   7.552   7.182  0.00  0.00      PROA
ATOM   3000  HB1 CYS P 186      14.219   6.983   7.007  0.00  0.00      PROA
ATOM   3001  HB2 CYS P 186      13.631   8.570   7.458  0.00  0.00      PROA
ATOM   3002  SG  CYS P 186      12.173   7.631   5.708  0.00  0.00      PROA
ATOM   3003  C   CYS P 186      12.229   5.380   8.171  0.00  0.00      PROA
ATOM   3004  O   CYS P 186      13.171   4.676   7.779  0.00  0.00      PROA
ATOM   3005  N   ASP P 187      10.947   5.003   8.255  0.00  0.00      PROA
ATOM   3006  HN  ASP P 187      10.178   5.568   8.543  0.00  0.00      PROA
ATOM   3007  CA  ASP P 187      10.580   3.624   8.145  0.00  0.00      PROA
ATOM   3008  HA  ASP P 187      11.264   3.202   7.424  0.00  0.00      PROA
ATOM   3009  CB  ASP P 187      11.003   2.824   9.392  0.00  0.00      PROA
ATOM   3010  HB1 ASP P 187      10.332   2.999  10.260  0.00  0.00      PROA
ATOM   3011  HB2 ASP P 187      11.976   3.196   9.779  0.00  0.00      PROA
ATOM   3012  CG  ASP P 187      11.021   1.343   9.155  0.00  0.00      PROA
ATOM   3013  OD1 ASP P 187      10.797   0.789   8.030  0.00  0.00      PROA
ATOM   3014  OD2 ASP P 187      11.192   0.592  10.221  0.00  0.00      PROA
ATOM   3015  C   ASP P 187       9.109   3.555   7.722  0.00  0.00      PROA
ATOM   3016  O   ASP P 187       8.325   4.497   7.952  0.00  0.00      PROA
ATOM   3017  N   ARG P 188       8.732   2.476   7.070  0.00  0.00      PROA
ATOM   3018  HN  ARG P 188       9.475   1.829   6.914  0.00  0.00      PROA
ATOM   3019  CA  ARG P 188       7.435   2.147   6.683  0.00  0.00      PROA
ATOM   3020  HA  ARG P 188       6.932   3.092   6.541  0.00  0.00      PROA
ATOM   3021  CB  ARG P 188       7.495   1.610   5.202  0.00  0.00      PROA
ATOM   3022  HB1 ARG P 188       6.461   1.285   4.959  0.00  0.00      PROA
ATOM   3023  HB2 ARG P 188       7.942   0.595   5.145  0.00  0.00      PROA
ATOM   3024  CG  ARG P 188       8.150   2.628   4.272  0.00  0.00      PROA
ATOM   3025  HG1 ARG P 188       9.166   2.961   4.574  0.00  0.00      PROA
ATOM   3026  HG2 ARG P 188       7.426   3.470   4.236  0.00  0.00      PROA
ATOM   3027  CD  ARG P 188       8.220   2.039   2.827  0.00  0.00      PROA
ATOM   3028  HD1 ARG P 188       8.649   2.799   2.140  0.00  0.00      PROA
ATOM   3029  HD2 ARG P 188       7.237   1.624   2.517  0.00  0.00      PROA
ATOM   3030  NE  ARG P 188       9.299   1.024   2.847  0.00  0.00      PROA
ATOM   3031  HE  ARG P 188       9.654   0.816   3.759  0.00  0.00      PROA
ATOM   3032  CZ  ARG P 188       9.943   0.430   1.770  0.00  0.00      PROA
ATOM   3033  NH1 ARG P 188       9.713   0.628   0.523  0.00  0.00      PROA
ATOM   3034 HH11 ARG P 188       8.897   1.131   0.238  0.00  0.00      PROA
ATOM   3035 HH12 ARG P 188      10.437   0.335  -0.101  0.00  0.00      PROA
ATOM   3036  NH2 ARG P 188      11.048  -0.241   2.108  0.00  0.00      PROA
ATOM   3037 HH21 ARG P 188      11.284  -0.437   3.060  0.00  0.00      PROA
ATOM   3038 HH22 ARG P 188      11.654  -0.592   1.394  0.00  0.00      PROA
ATOM   3039  C   ARG P 188       6.727   1.232   7.632  0.00  0.00      PROA
ATOM   3040  O   ARG P 188       7.230   0.236   8.073  0.00  0.00      PROA
ATOM   3041  N   PHE P 189       5.549   1.646   8.174  0.00  0.00      PROA
ATOM   3042  HN  PHE P 189       5.114   2.489   7.868  0.00  0.00      PROA
ATOM   3043  CA  PHE P 189       4.896   0.884   9.254  0.00  0.00      PROA
ATOM   3044  HA  PHE P 189       5.557   0.296   9.873  0.00  0.00      PROA
ATOM   3045  CB  PHE P 189       4.296   1.924  10.298  0.00  0.00      PROA
ATOM   3046  HB1 PHE P 189       3.798   1.413  11.149  0.00  0.00      PROA
```

```
ATOM   3047  HB2 PHE P 189       3.537   2.522   9.749  0.00  0.00      PROA
ATOM   3048  CG  PHE P 189       5.274   2.819  10.867  0.00  0.00      PROA
ATOM   3049  CD1 PHE P 189       6.620   2.346  10.987  0.00  0.00      PROA
ATOM   3050  HD1 PHE P 189       7.065   1.451  10.579  0.00  0.00      PROA
ATOM   3051  CE1 PHE P 189       7.517   3.133  11.727  0.00  0.00      PROA
ATOM   3052  HE1 PHE P 189       8.475   2.640  11.802  0.00  0.00      PROA
ATOM   3053  CZ  PHE P 189       7.162   4.338  12.347  0.00  0.00      PROA
ATOM   3054  HZ  PHE P 189       7.935   4.851  12.898  0.00  0.00      PROA
ATOM   3055  CD2 PHE P 189       4.860   4.005  11.604  0.00  0.00      PROA
ATOM   3056  HD2 PHE P 189       3.814   4.270  11.640  0.00  0.00      PROA
ATOM   3057  CE2 PHE P 189       5.846   4.745  12.286  0.00  0.00      PROA
ATOM   3058  HE2 PHE P 189       5.512   5.628  12.811  0.00  0.00      PROA
ATOM   3059  C   PHE P 189       3.852  -0.060   8.752  0.00  0.00      PROA
ATOM   3060  O   PHE P 189       2.669   0.198   8.651  0.00  0.00      PROA
ATOM   3061  N   TYR P 190       4.297  -1.324   8.428  0.00  0.00      PROA
ATOM   3062  HN  TYR P 190       5.266  -1.558   8.415  0.00  0.00      PROA
ATOM   3063  CA  TYR P 190       3.386  -2.310   7.796  0.00  0.00      PROA
ATOM   3064  HA  TYR P 190       2.594  -1.763   7.307  0.00  0.00      PROA
ATOM   3065  CB  TYR P 190       4.236  -3.242   6.897  0.00  0.00      PROA
ATOM   3066  HB1 TYR P 190       3.695  -3.913   6.196  0.00  0.00      PROA
ATOM   3067  HB2 TYR P 190       4.786  -3.933   7.571  0.00  0.00      PROA
ATOM   3068  CG  TYR P 190       5.133  -2.495   6.016  0.00  0.00      PROA
ATOM   3069  CD1 TYR P 190       4.682  -1.799   4.928  0.00  0.00      PROA
ATOM   3070  HD1 TYR P 190       3.626  -1.614   4.799  0.00  0.00      PROA
ATOM   3071  CE1 TYR P 190       5.482  -1.446   3.798  0.00  0.00      PROA
ATOM   3072  HE1 TYR P 190       5.131  -0.777   3.026  0.00  0.00      PROA
ATOM   3073  CZ  TYR P 190       6.826  -1.821   3.888  0.00  0.00      PROA
ATOM   3074  OH  TYR P 190       7.749  -1.631   2.853  0.00  0.00      PROA
ATOM   3075  HH  TYR P 190       7.196  -1.091   2.283  0.00  0.00      PROA
ATOM   3076  CD2 TYR P 190       6.538  -2.798   6.054  0.00  0.00      PROA
ATOM   3077  HD2 TYR P 190       6.874  -3.485   6.816  0.00  0.00      PROA
ATOM   3078  CE2 TYR P 190       7.357  -2.441   4.984  0.00  0.00      PROA
ATOM   3079  HE2 TYR P 190       8.364  -2.823   4.900  0.00  0.00      PROA
ATOM   3080  C   TYR P 190       2.619  -3.191   8.868  0.00  0.00      PROA
ATOM   3081  O   TYR P 190       3.117  -3.302   9.980  0.00  0.00      PROA
ATOM   3082  N   PRO P 191       1.522  -3.905   8.599  0.00  0.00      PROA
ATOM   3083  CD  PRO P 191       0.829  -3.714   7.423  0.00  0.00      PROA
ATOM   3084  HD1 PRO P 191       1.403  -4.021   6.523  0.00  0.00      PROA
ATOM   3085  HD2 PRO P 191       0.520  -2.679   7.162  0.00  0.00      PROA
ATOM   3086  CA  PRO P 191       0.762  -4.569   9.676  0.00  0.00      PROA
ATOM   3087  HA  PRO P 191       0.550  -3.819  10.424  0.00  0.00      PROA
ATOM   3088  CB  PRO P 191      -0.488  -4.928   9.055  0.00  0.00      PROA
ATOM   3089  HB1 PRO P 191      -1.333  -4.264   9.339  0.00  0.00      PROA
ATOM   3090  HB2 PRO P 191      -0.783  -5.994   9.148  0.00  0.00      PROA
ATOM   3091  CG  PRO P 191      -0.326  -4.717   7.511  0.00  0.00      PROA
ATOM   3092  HG1 PRO P 191      -0.130  -5.730   7.099  0.00  0.00      PROA
ATOM   3093  HG2 PRO P 191      -1.314  -4.323   7.190  0.00  0.00      PROA
ATOM   3094  C   PRO P 191       1.394  -5.770  10.348  0.00  0.00      PROA
ATOM   3095  O   PRO P 191       1.160  -5.945  11.558  0.00  0.00      PROA
ATOM   3096  N   ASN P 192       2.218  -6.601   9.612  0.00  0.00      PROA
ATOM   3097  HN  ASN P 192       2.320  -6.476   8.628  0.00  0.00      PROA
ATOM   3098  CA  ASN P 192       2.993  -7.710  10.168  0.00  0.00      PROA
ATOM   3099  HA  ASN P 192       3.424  -7.359  11.094  0.00  0.00      PROA
ATOM   3100  CB  ASN P 192       1.957  -8.896  10.508  0.00  0.00      PROA
ATOM   3101  HB1 ASN P 192       1.290  -8.488  11.297  0.00  0.00      PROA
ATOM   3102  HB2 ASN P 192       2.508  -9.788  10.875  0.00  0.00      PROA
ATOM   3103  CG  ASN P 192       1.138  -9.342   9.287  0.00  0.00      PROA
ATOM   3104  OD1 ASN P 192       1.147  -8.809   8.202  0.00  0.00      PROA
ATOM   3105  ND2 ASN P 192       0.392 -10.462   9.544  0.00  0.00      PROA
ATOM   3106 HD21 ASN P 192      -0.046 -10.938   8.781  0.00  0.00      PROA
ATOM   3107 HD22 ASN P 192       0.547 -10.932  10.413  0.00  0.00      PROA
```

```
ATOM   3108  C   ASN P 192       4.117  -8.145   9.297  0.00  0.00      PROA
ATOM   3109  O   ASN P 192       4.183  -7.751   8.116  0.00  0.00      PROA
ATOM   3110  N   ASP P 193       5.048  -8.855   9.816  0.00  0.00      PROA
ATOM   3111  HN  ASP P 193       4.954  -9.302  10.702  0.00  0.00      PROA
ATOM   3112  CA  ASP P 193       6.249  -9.225   9.118  0.00  0.00      PROA
ATOM   3113  HA  ASP P 193       6.723  -8.264   8.982  0.00  0.00      PROA
ATOM   3114  CB  ASP P 193       7.121 -10.027  10.066  0.00  0.00      PROA
ATOM   3115  HB1 ASP P 193       8.108 -10.256   9.609  0.00  0.00      PROA
ATOM   3116  HB2 ASP P 193       6.606 -10.993  10.254  0.00  0.00      PROA
ATOM   3117  CG  ASP P 193       7.468  -9.438  11.476  0.00  0.00      PROA
ATOM   3118  OD1 ASP P 193       7.684 -10.259  12.436  0.00  0.00      PROA
ATOM   3119  OD2 ASP P 193       7.395  -8.207  11.578  0.00  0.00      PROA
ATOM   3120  C   ASP P 193       6.013 -10.053   7.844  0.00  0.00      PROA
ATOM   3121  O   ASP P 193       6.844  -9.987   6.984  0.00  0.00      PROA
ATOM   3122  N   LEU P 194       4.882 -10.813   7.784  0.00  0.00      PROA
ATOM   3123  HN  LEU P 194       4.231 -10.826   8.539  0.00  0.00      PROA
ATOM   3124  CA  LEU P 194       4.537 -11.548   6.607  0.00  0.00      PROA
ATOM   3125  HA  LEU P 194       5.293 -12.295   6.416  0.00  0.00      PROA
ATOM   3126  CB  LEU P 194       3.250 -12.504   6.768  0.00  0.00      PROA
ATOM   3127  HB1 LEU P 194       2.299 -11.983   7.013  0.00  0.00      PROA
ATOM   3128  HB2 LEU P 194       3.448 -13.171   7.634  0.00  0.00      PROA
ATOM   3129  CG  LEU P 194       2.895 -13.394   5.603  0.00  0.00      PROA
ATOM   3130  HG  LEU P 194       2.520 -12.799   4.742  0.00  0.00      PROA
ATOM   3131  CD1 LEU P 194       4.050 -14.183   5.146  0.00  0.00      PROA
ATOM   3132 HD11 LEU P 194       4.533 -14.588   6.061  0.00  0.00      PROA
ATOM   3133 HD12 LEU P 194       4.783 -13.599   4.550  0.00  0.00      PROA
ATOM   3134 HD13 LEU P 194       3.704 -15.110   4.642  0.00  0.00      PROA
ATOM   3135  CD2 LEU P 194       1.807 -14.382   5.988  0.00  0.00      PROA
ATOM   3136 HD21 LEU P 194       0.961 -13.982   6.586  0.00  0.00      PROA
ATOM   3137 HD22 LEU P 194       2.135 -15.174   6.696  0.00  0.00      PROA
ATOM   3138 HD23 LEU P 194       1.495 -15.076   5.178  0.00  0.00      PROA
ATOM   3139  C   LEU P 194       4.246 -10.765   5.362  0.00  0.00      PROA
ATOM   3140  O   LEU P 194       4.604 -11.158   4.277  0.00  0.00      PROA
ATOM   3141  N   TRP P 195       3.636  -9.584   5.520  0.00  0.00      PROA
ATOM   3142  HN  TRP P 195       3.316  -9.350   6.434  0.00  0.00      PROA
ATOM   3143  CA  TRP P 195       3.551  -8.515   4.517  0.00  0.00      PROA
ATOM   3144  HA  TRP P 195       2.921  -9.044   3.817  0.00  0.00      PROA
ATOM   3145  CB  TRP P 195       2.917  -7.283   5.204  0.00  0.00      PROA
ATOM   3146  HB1 TRP P 195       3.604  -6.721   5.871  0.00  0.00      PROA
ATOM   3147  HB2 TRP P 195       2.083  -7.681   5.822  0.00  0.00      PROA
ATOM   3148  CG  TRP P 195       2.311  -6.283   4.259  0.00  0.00      PROA
ATOM   3149  CD1 TRP P 195       0.949  -5.928   4.276  0.00  0.00      PROA
ATOM   3150  HD1 TRP P 195       0.159  -6.402   4.838  0.00  0.00      PROA
ATOM   3151  NE1 TRP P 195       0.697  -4.996   3.323  0.00  0.00      PROA
ATOM   3152  HE1 TRP P 195      -0.167  -4.707   2.973  0.00  0.00      PROA
ATOM   3153  CE2 TRP P 195       1.878  -4.591   2.797  0.00  0.00      PROA
ATOM   3154  CD2 TRP P 195       2.949  -5.376   3.364  0.00  0.00      PROA
ATOM   3155  CE3 TRP P 195       4.320  -5.069   3.112  0.00  0.00      PROA
ATOM   3156  HE3 TRP P 195       5.139  -5.488   3.678  0.00  0.00      PROA
ATOM   3157  CZ3 TRP P 195       4.540  -3.924   2.201  0.00  0.00      PROA
ATOM   3158  HZ3 TRP P 195       5.578  -3.634   2.133  0.00  0.00      PROA
ATOM   3159  CZ2 TRP P 195       2.162  -3.699   1.849  0.00  0.00      PROA
ATOM   3160  HZ2 TRP P 195       1.330  -3.186   1.389  0.00  0.00      PROA
ATOM   3161  CH2 TRP P 195       3.475  -3.355   1.515  0.00  0.00      PROA
ATOM   3162  HH2 TRP P 195       3.717  -2.604   0.778  0.00  0.00      PROA
ATOM   3163  C   TRP P 195       4.877  -8.159   3.884  0.00  0.00      PROA
ATOM   3164  O   TRP P 195       4.944  -8.223   2.662  0.00  0.00      PROA
ATOM   3165  N   VAL P 196       5.927  -7.928   4.685  0.00  0.00      PROA
ATOM   3166  HN  VAL P 196       5.788  -7.976   5.671  0.00  0.00      PROA
ATOM   3167  CA  VAL P 196       7.277  -7.663   4.237  0.00  0.00      PROA
ATOM   3168  HA  VAL P 196       7.288  -6.780   3.615  0.00  0.00      PROA
```

ATOM	3169	CB	VAL	P	196	8.234	-7.356	5.453	0.00	0.00	PROA
ATOM	3170	HB	VAL	P	196	8.323	-8.118	6.257	0.00	0.00	PROA
ATOM	3171	CG1	VAL	P	196	9.721	-7.087	5.018	0.00	0.00	PROA
ATOM	3172	HG11	VAL	P	196	10.149	-8.066	4.710	0.00	0.00	PROA
ATOM	3173	HG12	VAL	P	196	10.234	-6.729	5.936	0.00	0.00	PROA
ATOM	3174	HG13	VAL	P	196	9.803	-6.345	4.196	0.00	0.00	PROA
ATOM	3175	CG2	VAL	P	196	7.762	-6.006	5.977	0.00	0.00	PROA
ATOM	3176	HG21	VAL	P	196	6.797	-6.021	6.528	0.00	0.00	PROA
ATOM	3177	HG22	VAL	P	196	7.633	-5.254	5.169	0.00	0.00	PROA
ATOM	3178	HG23	VAL	P	196	8.424	-5.602	6.773	0.00	0.00	PROA
ATOM	3179	C	VAL	P	196	7.837	-8.769	3.437	0.00	0.00	PROA
ATOM	3180	O	VAL	P	196	8.481	-8.597	2.375	0.00	0.00	PROA
ATOM	3181	N	VAL	P	197	7.510	-9.968	3.860	0.00	0.00	PROA
ATOM	3182	HN	VAL	P	197	6.899	-10.083	4.640	0.00	0.00	PROA
ATOM	3183	CA	VAL	P	197	8.066	-11.153	3.138	0.00	0.00	PROA
ATOM	3184	HA	VAL	P	197	9.109	-11.036	2.882	0.00	0.00	PROA
ATOM	3185	CB	VAL	P	197	7.848	-12.494	3.866	0.00	0.00	PROA
ATOM	3186	HB	VAL	P	197	6.782	-12.522	4.179	0.00	0.00	PROA
ATOM	3187	CG1	VAL	P	197	8.303	-13.763	3.094	0.00	0.00	PROA
ATOM	3188	HG11	VAL	P	197	7.641	-14.017	2.239	0.00	0.00	PROA
ATOM	3189	HG12	VAL	P	197	8.402	-14.683	3.710	0.00	0.00	PROA
ATOM	3190	HG13	VAL	P	197	9.282	-13.587	2.599	0.00	0.00	PROA
ATOM	3191	CG2	VAL	P	197	8.589	-12.437	5.222	0.00	0.00	PROA
ATOM	3192	HG21	VAL	P	197	9.681	-12.430	5.017	0.00	0.00	PROA
ATOM	3193	HG22	VAL	P	197	8.445	-13.399	5.758	0.00	0.00	PROA
ATOM	3194	HG23	VAL	P	197	8.377	-11.589	5.907	0.00	0.00	PROA
ATOM	3195	C	VAL	P	197	7.474	-11.394	1.764	0.00	0.00	PROA
ATOM	3196	O	VAL	P	197	8.184	-11.585	0.808	0.00	0.00	PROA
ATOM	3197	N	VAL	P	198	6.132	-11.151	1.695	0.00	0.00	PROA
ATOM	3198	HN	VAL	P	198	5.639	-10.987	2.546	0.00	0.00	PROA
ATOM	3199	CA	VAL	P	198	5.412	-11.034	0.398	0.00	0.00	PROA
ATOM	3200	HA	VAL	P	198	5.535	-11.893	-0.245	0.00	0.00	PROA
ATOM	3201	CB	VAL	P	198	3.910	-10.992	0.509	0.00	0.00	PROA
ATOM	3202	HB	VAL	P	198	3.637	-10.219	1.259	0.00	0.00	PROA
ATOM	3203	CG1	VAL	P	198	3.178	-10.672	-0.791	0.00	0.00	PROA
ATOM	3204	HG11	VAL	P	198	3.570	-9.711	-1.187	0.00	0.00	PROA
ATOM	3205	HG12	VAL	P	198	2.070	-10.629	-0.726	0.00	0.00	PROA
ATOM	3206	HG13	VAL	P	198	3.262	-11.458	-1.571	0.00	0.00	PROA
ATOM	3207	CG2	VAL	P	198	3.407	-12.317	1.059	0.00	0.00	PROA
ATOM	3208	HG21	VAL	P	198	3.513	-12.273	2.164	0.00	0.00	PROA
ATOM	3209	HG22	VAL	P	198	4.008	-13.170	0.676	0.00	0.00	PROA
ATOM	3210	HG23	VAL	P	198	2.311	-12.421	0.912	0.00	0.00	PROA
ATOM	3211	C	VAL	P	198	5.964	-9.858	-0.431	0.00	0.00	PROA
ATOM	3212	O	VAL	P	198	6.146	-9.957	-1.658	0.00	0.00	PROA
ATOM	3213	N	PHE	P	199	6.290	-8.694	0.196	0.00	0.00	PROA
ATOM	3214	HN	PHE	P	199	5.994	-8.614	1.144	0.00	0.00	PROA
ATOM	3215	CA	PHE	P	199	6.878	-7.517	-0.452	0.00	0.00	PROA
ATOM	3216	HA	PHE	P	199	6.168	-7.364	-1.252	0.00	0.00	PROA
ATOM	3217	CB	PHE	P	199	6.708	-6.267	0.511	0.00	0.00	PROA
ATOM	3218	HB1	PHE	P	199	6.910	-6.655	1.532	0.00	0.00	PROA
ATOM	3219	HB2	PHE	P	199	5.615	-6.101	0.621	0.00	0.00	PROA
ATOM	3220	CG	PHE	P	199	7.585	-5.078	0.468	0.00	0.00	PROA
ATOM	3221	CD1	PHE	P	199	7.187	-3.978	-0.284	0.00	0.00	PROA
ATOM	3222	HD1	PHE	P	199	6.230	-3.928	-0.782	0.00	0.00	PROA
ATOM	3223	CE1	PHE	P	199	8.078	-2.969	-0.402	0.00	0.00	PROA
ATOM	3224	HE1	PHE	P	199	7.867	-2.171	-1.098	0.00	0.00	PROA
ATOM	3225	CZ	PHE	P	199	9.385	-2.988	0.107	0.00	0.00	PROA
ATOM	3226	HZ	PHE	P	199	10.144	-2.299	-0.232	0.00	0.00	PROA
ATOM	3227	CD2	PHE	P	199	8.866	-5.166	1.009	0.00	0.00	PROA
ATOM	3228	HD2	PHE	P	199	9.261	-6.057	1.475	0.00	0.00	PROA
ATOM	3229	CE2	PHE	P	199	9.792	-4.113	0.802	0.00	0.00	PROA

ATOM 3230 HE2 PHE P 199 10.854 -4.238 0.952 0.00 0.00 PROA
ATOM 3231 C PHE P 199 8.267 -7.699 -1.056 0.00 0.00 PROA
ATOM 3232 O PHE P 199 8.559 -7.269 -2.167 0.00 0.00 PROA
ATOM 3233 N GLN P 200 9.210 -8.378 -0.359 0.00 0.00 PROA
ATOM 3234 HN GLN P 200 9.046 -8.547 0.609 0.00 0.00 PROA
ATOM 3235 CA GLN P 200 10.517 -8.631 -0.948 0.00 0.00 PROA
ATOM 3236 HA GLN P 200 10.902 -7.744 -1.430 0.00 0.00 PROA
ATOM 3237 CB GLN P 200 11.618 -8.918 0.112 0.00 0.00 PROA
ATOM 3238 HB1 GLN P 200 12.573 -9.128 -0.415 0.00 0.00 PROA
ATOM 3239 HB2 GLN P 200 11.228 -9.714 0.782 0.00 0.00 PROA
ATOM 3240 CG GLN P 200 11.846 -7.746 1.139 0.00 0.00 PROA
ATOM 3241 HG1 GLN P 200 10.936 -7.716 1.777 0.00 0.00 PROA
ATOM 3242 HG2 GLN P 200 12.032 -6.813 0.566 0.00 0.00 PROA
ATOM 3243 CD GLN P 200 13.056 -8.029 2.068 0.00 0.00 PROA
ATOM 3244 OE1 GLN P 200 13.558 -7.109 2.721 0.00 0.00 PROA
ATOM 3245 NE2 GLN P 200 13.536 -9.282 2.195 0.00 0.00 PROA
ATOM 3246 HE21 GLN P 200 14.186 -9.516 2.918 0.00 0.00 PROA
ATOM 3247 HE22 GLN P 200 13.207 -9.933 1.510 0.00 0.00 PROA
ATOM 3248 C GLN P 200 10.503 -9.699 -2.095 0.00 0.00 PROA
ATOM 3249 O GLN P 200 11.215 -9.634 -3.096 0.00 0.00 PROA
ATOM 3250 N PHE P 201 9.538 -10.637 -2.024 0.00 0.00 PROA
ATOM 3251 HN PHE P 201 9.114 -10.849 -1.148 0.00 0.00 PROA
ATOM 3252 CA PHE P 201 9.174 -11.437 -3.096 0.00 0.00 PROA
ATOM 3253 HA PHE P 201 10.099 -11.837 -3.484 0.00 0.00 PROA
ATOM 3254 CB PHE P 201 8.332 -12.680 -2.758 0.00 0.00 PROA
ATOM 3255 HB1 PHE P 201 7.970 -13.237 -3.648 0.00 0.00 PROA
ATOM 3256 HB2 PHE P 201 7.412 -12.283 -2.276 0.00 0.00 PROA
ATOM 3257 CG PHE P 201 9.114 -13.744 -1.909 0.00 0.00 PROA
ATOM 3258 CD1 PHE P 201 8.660 -14.154 -0.696 0.00 0.00 PROA
ATOM 3259 HD1 PHE P 201 7.703 -13.769 -0.375 0.00 0.00 PROA
ATOM 3260 CE1 PHE P 201 9.411 -14.999 0.085 0.00 0.00 PROA
ATOM 3261 HE1 PHE P 201 9.108 -15.347 1.061 0.00 0.00 PROA
ATOM 3262 CZ PHE P 201 10.683 -15.467 -0.337 0.00 0.00 PROA
ATOM 3263 HZ PHE P 201 11.360 -16.141 0.166 0.00 0.00 PROA
ATOM 3264 CD2 PHE P 201 10.284 -14.333 -2.453 0.00 0.00 PROA
ATOM 3265 HD2 PHE P 201 10.697 -14.180 -3.439 0.00 0.00 PROA
ATOM 3266 CE2 PHE P 201 11.068 -15.217 -1.690 0.00 0.00 PROA
ATOM 3267 HE2 PHE P 201 11.941 -15.657 -2.150 0.00 0.00 PROA
ATOM 3268 C PHE P 201 8.589 -10.712 -4.356 0.00 0.00 PROA
ATOM 3269 O PHE P 201 8.808 -11.079 -5.499 0.00 0.00 PROA
ATOM 3270 N GLN P 202 7.957 -9.559 -4.198 0.00 0.00 PROA
ATOM 3271 HN GLN P 202 7.574 -9.343 -3.304 0.00 0.00 PROA
ATOM 3272 CA GLN P 202 7.567 -8.756 -5.351 0.00 0.00 PROA
ATOM 3273 HA GLN P 202 7.185 -9.390 -6.137 0.00 0.00 PROA
ATOM 3274 CB GLN P 202 6.569 -7.578 -5.047 0.00 0.00 PROA
ATOM 3275 HB1 GLN P 202 6.403 -7.048 -6.010 0.00 0.00 PROA
ATOM 3276 HB2 GLN P 202 6.964 -6.826 -4.331 0.00 0.00 PROA
ATOM 3277 CG GLN P 202 5.297 -8.146 -4.431 0.00 0.00 PROA
ATOM 3278 HG1 GLN P 202 4.549 -7.324 -4.420 0.00 0.00 PROA
ATOM 3279 HG2 GLN P 202 5.374 -8.359 -3.344 0.00 0.00 PROA
ATOM 3280 CD GLN P 202 4.743 -9.344 -5.246 0.00 0.00 PROA
ATOM 3281 OE1 GLN P 202 4.260 -9.238 -6.352 0.00 0.00 PROA
ATOM 3282 NE2 GLN P 202 4.840 -10.557 -4.603 0.00 0.00 PROA
ATOM 3283 HE21 GLN P 202 4.491 -11.389 -5.035 0.00 0.00 PROA
ATOM 3284 HE22 GLN P 202 5.538 -10.673 -3.896 0.00 0.00 PROA
ATOM 3285 C GLN P 202 8.792 -8.122 -6.009 0.00 0.00 PROA
ATOM 3286 O GLN P 202 8.939 -8.208 -7.235 0.00 0.00 PROA
ATOM 3287 N HSD P 203 9.798 -7.551 -5.247 0.00 0.00 PROA
ATOM 3288 HN HSD P 203 9.649 -7.638 -4.266 0.00 0.00 PROA
ATOM 3289 CA HSD P 203 11.083 -7.084 -5.712 0.00 0.00 PROA
ATOM 3290 HA HSD P 203 10.792 -6.316 -6.414 0.00 0.00 PROA

```
ATOM   3291  CB  HSD P 203      11.851  -6.609  -4.445  0.00  0.00      PROA
ATOM   3292  HB1 HSD P 203      12.874  -6.428  -4.838  0.00  0.00      PROA
ATOM   3293  HB2 HSD P 203      11.993  -7.303  -3.589  0.00  0.00      PROA
ATOM   3294  ND1 HSD P 203      10.294  -5.056  -3.330  0.00  0.00      PROA
ATOM   3295  HD1 HSD P 203       9.620  -5.758  -3.098  0.00  0.00      PROA
ATOM   3296  CG  HSD P 203      11.479  -5.235  -4.025  0.00  0.00      PROA
ATOM   3297  CE1 HSD P 203      10.268  -3.715  -2.980  0.00  0.00      PROA
ATOM   3298  HE1 HSD P 203       9.384  -3.431  -2.410  0.00  0.00      PROA
ATOM   3299  NE2 HSD P 203      11.384  -3.017  -3.338  0.00  0.00      PROA
ATOM   3300  CD2 HSD P 203      12.100  -4.035  -4.026  0.00  0.00      PROA
ATOM   3301  HD2 HSD P 203      13.059  -3.840  -4.489  0.00  0.00      PROA
ATOM   3302  C   HSD P 203      11.795  -8.099  -6.667  0.00  0.00      PROA
ATOM   3303  O   HSD P 203      12.137  -7.799  -7.770  0.00  0.00      PROA
ATOM   3304  N   ILE P 204      11.818  -9.372  -6.277  0.00  0.00      PROA
ATOM   3305  HN  ILE P 204      11.650  -9.536  -5.308  0.00  0.00      PROA
ATOM   3306  CA  ILE P 204      12.187 -10.529  -7.096  0.00  0.00      PROA
ATOM   3307  HA  ILE P 204      13.180 -10.337  -7.475  0.00  0.00      PROA
ATOM   3308  CB  ILE P 204      12.346 -11.856  -6.245  0.00  0.00      PROA
ATOM   3309  HB  ILE P 204      11.329 -12.222  -5.987  0.00  0.00      PROA
ATOM   3310  CG2 ILE P 204      12.946 -13.004  -7.138  0.00  0.00      PROA
ATOM   3311 HG21 ILE P 204      12.131 -13.357  -7.805  0.00  0.00      PROA
ATOM   3312 HG22 ILE P 204      13.289 -13.845  -6.497  0.00  0.00      PROA
ATOM   3313 HG23 ILE P 204      13.822 -12.687  -7.744  0.00  0.00      PROA
ATOM   3314  CG1 ILE P 204      13.177 -11.700  -4.954  0.00  0.00      PROA
ATOM   3315 HG11 ILE P 204      13.090 -12.640  -4.369  0.00  0.00      PROA
ATOM   3316 HG12 ILE P 204      12.759 -10.855  -4.365  0.00  0.00      PROA
ATOM   3317  CD  ILE P 204      14.679 -11.336  -5.114  0.00  0.00      PROA
ATOM   3318  HD1 ILE P 204      15.185 -12.247  -5.500  0.00  0.00      PROA
ATOM   3319  HD2 ILE P 204      15.220 -10.996  -4.205  0.00  0.00      PROA
ATOM   3320  HD3 ILE P 204      14.705 -10.590  -5.937  0.00  0.00      PROA
ATOM   3321  C   ILE P 204      11.316 -10.772  -8.335  0.00  0.00      PROA
ATOM   3322  O   ILE P 204      11.807 -11.011  -9.424  0.00  0.00      PROA
ATOM   3323  N   MET P 205       9.990 -10.876  -8.236  0.00  0.00      PROA
ATOM   3324  HN  MET P 205       9.546 -10.736  -7.354  0.00  0.00      PROA
ATOM   3325  CA  MET P 205       9.149 -11.360  -9.239  0.00  0.00      PROA
ATOM   3326  HA  MET P 205       9.691 -12.145  -9.746  0.00  0.00      PROA
ATOM   3327  CB  MET P 205       7.904 -11.947  -8.608  0.00  0.00      PROA
ATOM   3328  HB1 MET P 205       7.402 -11.129  -8.048  0.00  0.00      PROA
ATOM   3329  HB2 MET P 205       8.200 -12.743  -7.893  0.00  0.00      PROA
ATOM   3330  CG  MET P 205       6.874 -12.675  -9.501  0.00  0.00      PROA
ATOM   3331  HG1 MET P 205       6.374 -11.988 -10.216  0.00  0.00      PROA
ATOM   3332  HG2 MET P 205       6.166 -13.122  -8.771  0.00  0.00      PROA
ATOM   3333  SD  MET P 205       7.686 -14.018 -10.388  0.00  0.00      PROA
ATOM   3334  CE  MET P 205       6.084 -14.434 -11.187  0.00  0.00      PROA
ATOM   3335  HE1 MET P 205       5.294 -14.470 -10.406  0.00  0.00      PROA
ATOM   3336  HE2 MET P 205       6.135 -15.426 -11.685  0.00  0.00      PROA
ATOM   3337  HE3 MET P 205       5.971 -13.654 -11.970  0.00  0.00      PROA
ATOM   3338  C   MET P 205       8.720 -10.317 -10.289  0.00  0.00      PROA
ATOM   3339  O   MET P 205       8.566 -10.678 -11.439  0.00  0.00      PROA
ATOM   3340  N   VAL P 206       8.499  -9.043  -9.867  0.00  0.00      PROA
ATOM   3341  HN  VAL P 206       8.788  -8.721  -8.968  0.00  0.00      PROA
ATOM   3342  CA  VAL P 206       8.054  -7.960 -10.762  0.00  0.00      PROA
ATOM   3343  HA  VAL P 206       7.725  -8.384 -11.699  0.00  0.00      PROA
ATOM   3344  CB  VAL P 206       6.901  -7.262 -10.015  0.00  0.00      PROA
ATOM   3345  HB  VAL P 206       7.288  -6.675  -9.155  0.00  0.00      PROA
ATOM   3346  CG1 VAL P 206       6.168  -6.370 -11.062  0.00  0.00      PROA
ATOM   3347 HG11 VAL P 206       5.201  -5.971 -10.688  0.00  0.00      PROA
ATOM   3348 HG12 VAL P 206       5.850  -7.024 -11.902  0.00  0.00      PROA
ATOM   3349 HG13 VAL P 206       6.867  -5.590 -11.432  0.00  0.00      PROA
ATOM   3350  CG2 VAL P 206       5.922  -8.254  -9.315  0.00  0.00      PROA
ATOM   3351 HG21 VAL P 206       5.223  -7.606  -8.745  0.00  0.00      PROA
```

ATOM 3352 HG22 VAL P 206 6.454 -8.859 -8.550 0.00 0.00 PROA
ATOM 3353 HG23 VAL P 206 5.420 -8.824 -10.125 0.00 0.00 PROA
ATOM 3354 C VAL P 206 9.215 -7.021 -11.156 0.00 0.00 PROA
ATOM 3355 O VAL P 206 9.163 -6.270 -12.120 0.00 0.00 PROA
ATOM 3356 N GLY P 207 10.286 -7.019 -10.317 0.00 0.00 PROA
ATOM 3357 HN GLY P 207 10.381 -7.353 -9.382 0.00 0.00 PROA
ATOM 3358 CA GLY P 207 11.439 -6.152 -10.611 0.00 0.00 PROA
ATOM 3359 HA1 GLY P 207 11.801 -5.932 -9.617 0.00 0.00 PROA
ATOM 3360 HA2 GLY P 207 11.269 -5.267 -11.205 0.00 0.00 PROA
ATOM 3361 C GLY P 207 12.585 -6.851 -11.306 0.00 0.00 PROA
ATOM 3362 O GLY P 207 13.534 -6.301 -11.780 0.00 0.00 PROA
ATOM 3363 N LEU P 208 12.498 -8.214 -11.456 0.00 0.00 PROA
ATOM 3364 HN LEU P 208 11.629 -8.631 -11.201 0.00 0.00 PROA
ATOM 3365 CA LEU P 208 13.511 -9.068 -12.093 0.00 0.00 PROA
ATOM 3366 HA LEU P 208 14.060 -8.507 -12.835 0.00 0.00 PROA
ATOM 3367 CB LEU P 208 14.457 -9.925 -11.094 0.00 0.00 PROA
ATOM 3368 HB1 LEU P 208 13.948 -10.575 -10.351 0.00 0.00 PROA
ATOM 3369 HB2 LEU P 208 14.922 -9.187 -10.406 0.00 0.00 PROA
ATOM 3370 CG LEU P 208 15.436 -10.860 -11.833 0.00 0.00 PROA
ATOM 3371 HG LEU P 208 14.859 -11.660 -12.344 0.00 0.00 PROA
ATOM 3372 CD1 LEU P 208 16.354 -10.076 -12.847 0.00 0.00 PROA
ATOM 3373 HD11 LEU P 208 16.962 -9.336 -12.283 0.00 0.00 PROA
ATOM 3374 HD12 LEU P 208 15.776 -9.469 -13.577 0.00 0.00 PROA
ATOM 3375 HD13 LEU P 208 17.016 -10.780 -13.396 0.00 0.00 PROA
ATOM 3376 CD2 LEU P 208 16.378 -11.483 -10.855 0.00 0.00 PROA
ATOM 3377 HD21 LEU P 208 17.136 -12.096 -11.388 0.00 0.00 PROA
ATOM 3378 HD22 LEU P 208 15.808 -12.196 -10.222 0.00 0.00 PROA
ATOM 3379 HD23 LEU P 208 17.077 -10.838 -10.281 0.00 0.00 PROA
ATOM 3380 C LEU P 208 12.764 -10.084 -12.956 0.00 0.00 PROA
ATOM 3381 O LEU P 208 12.875 -9.992 -14.131 0.00 0.00 PROA
ATOM 3382 N ILE P 209 12.116 -11.104 -12.356 0.00 0.00 PROA
ATOM 3383 HN ILE P 209 11.967 -11.290 -11.387 0.00 0.00 PROA
ATOM 3384 CA ILE P 209 11.766 -12.250 -13.184 0.00 0.00 PROA
ATOM 3385 HA ILE P 209 12.647 -12.562 -13.725 0.00 0.00 PROA
ATOM 3386 CB ILE P 209 11.399 -13.444 -12.315 0.00 0.00 PROA
ATOM 3387 HB ILE P 209 10.634 -13.149 -11.564 0.00 0.00 PROA
ATOM 3388 CG2 ILE P 209 10.873 -14.656 -13.079 0.00 0.00 PROA
ATOM 3389 HG21 ILE P 209 11.624 -15.474 -13.077 0.00 0.00 PROA
ATOM 3390 HG22 ILE P 209 10.532 -14.431 -14.112 0.00 0.00 PROA
ATOM 3391 HG23 ILE P 209 9.971 -14.907 -12.481 0.00 0.00 PROA
ATOM 3392 CG1 ILE P 209 12.623 -13.957 -11.367 0.00 0.00 PROA
ATOM 3393 HG11 ILE P 209 13.028 -13.026 -10.915 0.00 0.00 PROA
ATOM 3394 HG12 ILE P 209 13.432 -14.265 -12.064 0.00 0.00 PROA
ATOM 3395 CD ILE P 209 12.465 -15.016 -10.283 0.00 0.00 PROA
ATOM 3396 HD1 ILE P 209 11.539 -14.850 -9.692 0.00 0.00 PROA
ATOM 3397 HD2 ILE P 209 13.396 -15.074 -9.679 0.00 0.00 PROA
ATOM 3398 HD3 ILE P 209 12.432 -16.039 -10.713 0.00 0.00 PROA
ATOM 3399 C ILE P 209 10.734 -11.969 -14.375 0.00 0.00 PROA
ATOM 3400 O ILE P 209 11.000 -12.312 -15.529 0.00 0.00 PROA
ATOM 3401 N LEU P 210 9.623 -11.250 -14.151 0.00 0.00 PROA
ATOM 3402 HN LEU P 210 9.426 -11.029 -13.199 0.00 0.00 PROA
ATOM 3403 CA LEU P 210 8.627 -11.029 -15.211 0.00 0.00 PROA
ATOM 3404 HA LEU P 210 8.340 -12.031 -15.492 0.00 0.00 PROA
ATOM 3405 CB LEU P 210 7.311 -10.454 -14.678 0.00 0.00 PROA
ATOM 3406 HB1 LEU P 210 6.559 -10.114 -15.422 0.00 0.00 PROA
ATOM 3407 HB2 LEU P 210 7.534 -9.557 -14.063 0.00 0.00 PROA
ATOM 3408 CG LEU P 210 6.451 -11.517 -13.768 0.00 0.00 PROA
ATOM 3409 HG LEU P 210 6.983 -12.138 -13.016 0.00 0.00 PROA
ATOM 3410 CD1 LEU P 210 5.399 -10.688 -12.910 0.00 0.00 PROA
ATOM 3411 HD11 LEU P 210 4.681 -11.430 -12.499 0.00 0.00 PROA
ATOM 3412 HD12 LEU P 210 4.795 -10.007 -13.547 0.00 0.00 PROA

```
ATOM   3413 HD13 LEU P 210       6.052 -10.134 -12.203  0.00  0.00      PROA
ATOM   3414  CD2 LEU P 210       5.644 -12.479 -14.687  0.00  0.00      PROA
ATOM   3415 HD21 LEU P 210       5.077 -13.286 -14.176  0.00  0.00      PROA
ATOM   3416 HD22 LEU P 210       6.346 -13.004 -15.370  0.00  0.00      PROA
ATOM   3417 HD23 LEU P 210       4.976 -11.872 -15.335  0.00  0.00      PROA
ATOM   3418  C   LEU P 210       9.055 -10.310 -16.440  0.00  0.00      PROA
ATOM   3419  O   LEU P 210       8.940 -10.846 -17.526  0.00  0.00      PROA
ATOM   3420  N   PRO P 211       9.639  -9.124 -16.441  0.00  0.00      PROA
ATOM   3421  CD  PRO P 211       9.740  -8.211 -15.311  0.00  0.00      PROA
ATOM   3422  HD1 PRO P 211      10.087  -8.612 -14.334  0.00  0.00      PROA
ATOM   3423  HD2 PRO P 211       8.721  -7.828 -15.091  0.00  0.00      PROA
ATOM   3424  CA  PRO P 211      10.261  -8.541 -17.622  0.00  0.00      PROA
ATOM   3425  HA  PRO P 211       9.671  -8.681 -18.516  0.00  0.00      PROA
ATOM   3426  CB  PRO P 211      10.361  -7.037 -17.235  0.00  0.00      PROA
ATOM   3427  HB1 PRO P 211       9.395  -6.606 -17.576  0.00  0.00      PROA
ATOM   3428  HB2 PRO P 211      11.132  -6.423 -17.747  0.00  0.00      PROA
ATOM   3429  CG  PRO P 211      10.548  -7.013 -15.685  0.00  0.00      PROA
ATOM   3430  HG1 PRO P 211      11.614  -7.287 -15.530  0.00  0.00      PROA
ATOM   3431  HG2 PRO P 211      10.360  -6.011 -15.245  0.00  0.00      PROA
ATOM   3432  C   PRO P 211      11.615  -9.184 -17.865  0.00  0.00      PROA
ATOM   3433  O   PRO P 211      12.041  -9.254 -19.026  0.00  0.00      PROA
ATOM   3434  N   GLY P 212      12.313  -9.715 -16.887  0.00  0.00      PROA
ATOM   3435  HN  GLY P 212      12.183  -9.739 -15.899  0.00  0.00      PROA
ATOM   3436  CA  GLY P 212      13.514 -10.490 -17.224  0.00  0.00      PROA
ATOM   3437  HA1 GLY P 212      13.899 -10.933 -16.317  0.00  0.00      PROA
ATOM   3438  HA2 GLY P 212      14.201  -9.752 -17.610  0.00  0.00      PROA
ATOM   3439  C   GLY P 212      13.390 -11.739 -18.077  0.00  0.00      PROA
ATOM   3440  O   GLY P 212      14.289 -12.169 -18.772  0.00  0.00      PROA
ATOM   3441  N   ILE P 213      12.259 -12.484 -17.990  0.00  0.00      PROA
ATOM   3442  HN  ILE P 213      11.552 -12.338 -17.302  0.00  0.00      PROA
ATOM   3443  CA  ILE P 213      11.991 -13.573 -18.956  0.00  0.00      PROA
ATOM   3444  HA  ILE P 213      12.840 -14.238 -19.021  0.00  0.00      PROA
ATOM   3445  CB  ILE P 213      10.701 -14.323 -18.529  0.00  0.00      PROA
ATOM   3446  HB  ILE P 213       9.906 -13.699 -18.067  0.00  0.00      PROA
ATOM   3447  CG2 ILE P 213      10.040 -15.085 -19.742  0.00  0.00      PROA
ATOM   3448 HG21 ILE P 213       9.183 -15.726 -19.446  0.00  0.00      PROA
ATOM   3449 HG22 ILE P 213      10.762 -15.723 -20.295  0.00  0.00      PROA
ATOM   3450 HG23 ILE P 213       9.614 -14.460 -20.555  0.00  0.00      PROA
ATOM   3451  CG1 ILE P 213      11.000 -15.388 -17.362  0.00  0.00      PROA
ATOM   3452 HG11 ILE P 213      11.404 -14.837 -16.486  0.00  0.00      PROA
ATOM   3453 HG12 ILE P 213      11.815 -16.072 -17.682  0.00  0.00      PROA
ATOM   3454  CD  ILE P 213       9.729 -16.144 -17.057  0.00  0.00      PROA
ATOM   3455  HD1 ILE P 213       9.686 -16.946 -17.825  0.00  0.00      PROA
ATOM   3456  HD2 ILE P 213       8.813 -15.519 -17.129  0.00  0.00      PROA
ATOM   3457  HD3 ILE P 213       9.709 -16.552 -16.023  0.00  0.00      PROA
ATOM   3458  C   ILE P 213      12.003 -13.139 -20.363  0.00  0.00      PROA
ATOM   3459  O   ILE P 213      12.591 -13.686 -21.321  0.00  0.00      PROA
ATOM   3460  N   VAL P 214      11.396 -11.965 -20.615  0.00  0.00      PROA
ATOM   3461  HN  VAL P 214      11.217 -11.494 -19.755  0.00  0.00      PROA
ATOM   3462  CA  VAL P 214      11.325 -11.144 -21.893  0.00  0.00      PROA
ATOM   3463  HA  VAL P 214      11.147 -11.859 -22.683  0.00  0.00      PROA
ATOM   3464  CB  VAL P 214      10.267 -10.079 -21.762  0.00  0.00      PROA
ATOM   3465  HB  VAL P 214      10.573  -9.434 -20.910  0.00  0.00      PROA
ATOM   3466  CG1 VAL P 214      10.116  -9.365 -23.112  0.00  0.00      PROA
ATOM   3467 HG11 VAL P 214      10.978  -8.711 -23.365  0.00  0.00      PROA
ATOM   3468 HG12 VAL P 214       9.204  -8.731 -23.142  0.00  0.00      PROA
ATOM   3469 HG13 VAL P 214      10.113 -10.079 -23.964  0.00  0.00      PROA
ATOM   3470  CG2 VAL P 214       8.977 -10.769 -21.334  0.00  0.00      PROA
ATOM   3471 HG21 VAL P 214       8.207  -9.968 -21.323  0.00  0.00      PROA
ATOM   3472 HG22 VAL P 214       9.075 -11.065 -20.267  0.00  0.00      PROA
ATOM   3473 HG23 VAL P 214       8.648 -11.590 -22.007  0.00  0.00      PROA
```

ATOM	3474	C	VAL	P	214	12.642	-10.519	-22.306	0.00	0.00	PROA
ATOM	3475	O	VAL	P	214	13.132	-10.607	-23.394	0.00	0.00	PROA
ATOM	3476	N	ILE	P	215	13.335	-9.833	-21.349	0.00	0.00	PROA
ATOM	3477	HN	ILE	P	215	12.846	-9.579	-20.518	0.00	0.00	PROA
ATOM	3478	CA	ILE	P	215	14.736	-9.463	-21.587	0.00	0.00	PROA
ATOM	3479	HA	ILE	P	215	14.696	-8.733	-22.382	0.00	0.00	PROA
ATOM	3480	CB	ILE	P	215	15.244	-8.763	-20.335	0.00	0.00	PROA
ATOM	3481	HB	ILE	P	215	15.158	-9.434	-19.453	0.00	0.00	PROA
ATOM	3482	CG2	ILE	P	215	16.673	-8.353	-20.517	0.00	0.00	PROA
ATOM	3483	HG21	ILE	P	215	17.453	-9.144	-20.534	0.00	0.00	PROA
ATOM	3484	HG22	ILE	P	215	16.922	-7.828	-19.570	0.00	0.00	PROA
ATOM	3485	HG23	ILE	P	215	16.911	-7.819	-21.462	0.00	0.00	PROA
ATOM	3486	CG1	ILE	P	215	14.448	-7.449	-19.996	0.00	0.00	PROA
ATOM	3487	HG11	ILE	P	215	13.367	-7.455	-20.252	0.00	0.00	PROA
ATOM	3488	HG12	ILE	P	215	14.838	-6.667	-20.682	0.00	0.00	PROA
ATOM	3489	CD	ILE	P	215	14.482	-6.934	-18.615	0.00	0.00	PROA
ATOM	3490	HD1	ILE	P	215	15.503	-6.689	-18.252	0.00	0.00	PROA
ATOM	3491	HD2	ILE	P	215	14.291	-7.885	-18.072	0.00	0.00	PROA
ATOM	3492	HD3	ILE	P	215	13.637	-6.254	-18.376	0.00	0.00	PROA
ATOM	3493	C	ILE	P	215	15.670	-10.605	-21.939	0.00	0.00	PROA
ATOM	3494	O	ILE	P	215	16.422	-10.519	-22.953	0.00	0.00	PROA
ATOM	3495	N	LEU	P	216	15.602	-11.719	-21.158	0.00	0.00	PROA
ATOM	3496	HN	LEU	P	216	14.980	-11.829	-20.387	0.00	0.00	PROA
ATOM	3497	CA	LEU	P	216	16.522	-12.815	-21.387	0.00	0.00	PROA
ATOM	3498	HA	LEU	P	216	17.498	-12.352	-21.401	0.00	0.00	PROA
ATOM	3499	CB	LEU	P	216	16.456	-13.830	-20.213	0.00	0.00	PROA
ATOM	3500	HB1	LEU	P	216	15.378	-14.012	-20.017	0.00	0.00	PROA
ATOM	3501	HB2	LEU	P	216	16.817	-13.247	-19.339	0.00	0.00	PROA
ATOM	3502	CG	LEU	P	216	17.234	-15.179	-20.457	0.00	0.00	PROA
ATOM	3503	HG	LEU	P	216	16.918	-15.547	-21.457	0.00	0.00	PROA
ATOM	3504	CD1	LEU	P	216	18.759	-15.035	-20.462	0.00	0.00	PROA
ATOM	3505	HD11	LEU	P	216	19.083	-14.272	-21.201	0.00	0.00	PROA
ATOM	3506	HD12	LEU	P	216	19.237	-15.963	-20.843	0.00	0.00	PROA
ATOM	3507	HD13	LEU	P	216	19.085	-14.703	-19.453	0.00	0.00	PROA
ATOM	3508	CD2	LEU	P	216	16.733	-16.336	-19.565	0.00	0.00	PROA
ATOM	3509	HD21	LEU	P	216	16.744	-15.860	-18.562	0.00	0.00	PROA
ATOM	3510	HD22	LEU	P	216	17.351	-17.259	-19.554	0.00	0.00	PROA
ATOM	3511	HD23	LEU	P	216	15.659	-16.576	-19.719	0.00	0.00	PROA
ATOM	3512	C	LEU	P	216	16.237	-13.516	-22.669	0.00	0.00	PROA
ATOM	3513	O	LEU	P	216	17.124	-13.838	-23.381	0.00	0.00	PROA
ATOM	3514	N	SER	P	217	14.924	-13.670	-23.043	0.00	0.00	PROA
ATOM	3515	HN	SER	P	217	14.115	-13.498	-22.487	0.00	0.00	PROA
ATOM	3516	CA	SER	P	217	14.582	-14.152	-24.353	0.00	0.00	PROA
ATOM	3517	HA	SER	P	217	14.956	-15.165	-24.400	0.00	0.00	PROA
ATOM	3518	CB	SER	P	217	12.971	-14.317	-24.521	0.00	0.00	PROA
ATOM	3519	HB1	SER	P	217	12.875	-14.936	-25.438	0.00	0.00	PROA
ATOM	3520	HB2	SER	P	217	12.341	-13.406	-24.607	0.00	0.00	PROA
ATOM	3521	OG	SER	P	217	12.500	-15.202	-23.518	0.00	0.00	PROA
ATOM	3522	HG1	SER	P	217	12.571	-14.760	-22.669	0.00	0.00	PROA
ATOM	3523	C	SER	P	217	15.112	-13.407	-25.589	0.00	0.00	PROA
ATOM	3524	O	SER	P	217	15.854	-13.950	-26.412	0.00	0.00	PROA
ATOM	3525	N	CYS	P	218	14.956	-12.058	-25.652	0.00	0.00	PROA
ATOM	3526	HN	CYS	P	218	14.658	-11.495	-24.885	0.00	0.00	PROA
ATOM	3527	CA	CYS	P	218	15.532	-11.169	-26.762	0.00	0.00	PROA
ATOM	3528	HA	CYS	P	218	15.294	-11.486	-27.766	0.00	0.00	PROA
ATOM	3529	CB	CYS	P	218	15.023	-9.755	-26.463	0.00	0.00	PROA
ATOM	3530	HB1	CYS	P	218	15.525	-9.102	-27.209	0.00	0.00	PROA
ATOM	3531	HB2	CYS	P	218	15.465	-9.477	-25.482	0.00	0.00	PROA
ATOM	3532	SG	CYS	P	218	13.153	-9.647	-26.413	0.00	0.00	PROA
ATOM	3533	HG1	CYS	P	218	13.001	-10.047	-25.160	0.00	0.00	PROA
ATOM	3534	C	CYS	P	218	17.070	-11.217	-26.781	0.00	0.00	PROA

ATOM	3535	O	CYS	P	218	17.708	-11.109	-27.809	0.00	0.00	PROA
ATOM	3536	N	TYR	P	219	17.704	-11.419	-25.568	0.00	0.00	PROA
ATOM	3537	HN	TYR	P	219	17.092	-11.614	-24.805	0.00	0.00	PROA
ATOM	3538	CA	TYR	P	219	19.079	-11.464	-25.331	0.00	0.00	PROA
ATOM	3539	HA	TYR	P	219	19.565	-10.662	-25.865	0.00	0.00	PROA
ATOM	3540	CB	TYR	P	219	19.484	-11.546	-23.741	0.00	0.00	PROA
ATOM	3541	HB1	TYR	P	219	18.786	-12.293	-23.305	0.00	0.00	PROA
ATOM	3542	HB2	TYR	P	219	19.202	-10.589	-23.252	0.00	0.00	PROA
ATOM	3543	CG	TYR	P	219	20.916	-11.955	-23.424	0.00	0.00	PROA
ATOM	3544	CD1	TYR	P	219	21.323	-13.283	-23.185	0.00	0.00	PROA
ATOM	3545	HD1	TYR	P	219	20.552	-14.038	-23.223	0.00	0.00	PROA
ATOM	3546	CE1	TYR	P	219	22.658	-13.602	-23.048	0.00	0.00	PROA
ATOM	3547	HE1	TYR	P	219	23.014	-14.610	-23.203	0.00	0.00	PROA
ATOM	3548	CZ	TYR	P	219	23.595	-12.559	-23.004	0.00	0.00	PROA
ATOM	3549	OH	TYR	P	219	24.960	-12.836	-22.708	0.00	0.00	PROA
ATOM	3550	HH	TYR	P	219	25.005	-13.787	-22.835	0.00	0.00	PROA
ATOM	3551	CD2	TYR	P	219	21.882	-10.897	-23.447	0.00	0.00	PROA
ATOM	3552	HD2	TYR	P	219	21.578	-9.899	-23.725	0.00	0.00	PROA
ATOM	3553	CE2	TYR	P	219	23.180	-11.221	-23.121	0.00	0.00	PROA
ATOM	3554	HE2	TYR	P	219	23.880	-10.451	-22.833	0.00	0.00	PROA
ATOM	3555	C	TYR	P	219	19.582	-12.716	-26.067	0.00	0.00	PROA
ATOM	3556	O	TYR	P	219	20.637	-12.626	-26.733	0.00	0.00	PROA
ATOM	3557	N	CYS	P	220	18.914	-13.895	-25.993	0.00	0.00	PROA
ATOM	3558	HN	CYS	P	220	18.205	-13.943	-25.294	0.00	0.00	PROA
ATOM	3559	CA	CYS	P	220	19.365	-15.195	-26.647	0.00	0.00	PROA
ATOM	3560	HA	CYS	P	220	20.368	-15.299	-26.261	0.00	0.00	PROA
ATOM	3561	CB	CYS	P	220	18.518	-16.407	-26.145	0.00	0.00	PROA
ATOM	3562	HB1	CYS	P	220	18.806	-17.270	-26.783	0.00	0.00	PROA
ATOM	3563	HB2	CYS	P	220	17.443	-16.214	-26.349	0.00	0.00	PROA
ATOM	3564	SG	CYS	P	220	18.583	-16.833	-24.332	0.00	0.00	PROA
ATOM	3565	HG1	CYS	P	220	18.146	-15.664	-23.888	0.00	0.00	PROA
ATOM	3566	C	CYS	P	220	19.426	-15.024	-28.199	0.00	0.00	PROA
ATOM	3567	O	CYS	P	220	20.327	-15.412	-28.874	0.00	0.00	PROA
ATOM	3568	N	ILE	P	221	18.357	-14.368	-28.764	0.00	0.00	PROA
ATOM	3569	HN	ILE	P	221	17.553	-14.144	-28.217	0.00	0.00	PROA
ATOM	3570	CA	ILE	P	221	18.295	-13.914	-30.176	0.00	0.00	PROA
ATOM	3571	HA	ILE	P	221	18.371	-14.771	-30.829	0.00	0.00	PROA
ATOM	3572	CB	ILE	P	221	16.956	-13.232	-30.525	0.00	0.00	PROA
ATOM	3573	HB	ILE	P	221	16.935	-12.181	-30.167	0.00	0.00	PROA
ATOM	3574	CG2	ILE	P	221	16.889	-13.038	-32.062	0.00	0.00	PROA
ATOM	3575	HG21	ILE	P	221	17.300	-13.909	-32.616	0.00	0.00	PROA
ATOM	3576	HG22	ILE	P	221	17.445	-12.159	-32.452	0.00	0.00	PROA
ATOM	3577	HG23	ILE	P	221	15.867	-12.692	-32.325	0.00	0.00	PROA
ATOM	3578	CG1	ILE	P	221	15.717	-14.063	-30.111	0.00	0.00	PROA
ATOM	3579	HG11	ILE	P	221	14.886	-13.363	-30.342	0.00	0.00	PROA
ATOM	3580	HG12	ILE	P	221	15.711	-14.223	-29.011	0.00	0.00	PROA
ATOM	3581	CD	ILE	P	221	15.556	-15.362	-30.753	0.00	0.00	PROA
ATOM	3582	HD1	ILE	P	221	15.315	-15.088	-31.803	0.00	0.00	PROA
ATOM	3583	HD2	ILE	P	221	14.552	-15.709	-30.427	0.00	0.00	PROA
ATOM	3584	HD3	ILE	P	221	16.341	-16.136	-30.618	0.00	0.00	PROA
ATOM	3585	C	ILE	P	221	19.467	-12.930	-30.504	0.00	0.00	PROA
ATOM	3586	O	ILE	P	221	20.087	-13.135	-31.509	0.00	0.00	PROA
ATOM	3587	N	ILE	P	222	19.732	-11.856	-29.716	0.00	0.00	PROA
ATOM	3588	HN	ILE	P	222	19.198	-11.632	-28.904	0.00	0.00	PROA
ATOM	3589	CA	ILE	P	222	20.817	-10.910	-29.987	0.00	0.00	PROA
ATOM	3590	HA	ILE	P	222	20.700	-10.572	-31.006	0.00	0.00	PROA
ATOM	3591	CB	ILE	P	222	20.771	-9.688	-29.119	0.00	0.00	PROA
ATOM	3592	HB	ILE	P	222	20.753	-9.925	-28.034	0.00	0.00	PROA
ATOM	3593	CG2	ILE	P	222	22.046	-8.931	-29.294	0.00	0.00	PROA
ATOM	3594	HG21	ILE	P	222	23.025	-9.333	-28.957	0.00	0.00	PROA
ATOM	3595	HG22	ILE	P	222	21.950	-7.945	-28.792	0.00	0.00	PROA

```
ATOM   3596 HG23 ILE P 222      22.128  -8.749 -30.386  0.00  0.00      PROA
ATOM   3597  CG1 ILE P 222      19.591  -8.760 -29.545  0.00  0.00      PROA
ATOM   3598 HG11 ILE P 222      18.740  -9.431 -29.788  0.00  0.00      PROA
ATOM   3599 HG12 ILE P 222      19.753  -8.190 -30.485  0.00  0.00      PROA
ATOM   3600  CD  ILE P 222      19.194  -7.807 -28.422  0.00  0.00      PROA
ATOM   3601  HD1 ILE P 222      18.972  -8.449 -27.544  0.00  0.00      PROA
ATOM   3602  HD2 ILE P 222      18.279  -7.228 -28.672  0.00  0.00      PROA
ATOM   3603  HD3 ILE P 222      19.935  -7.006 -28.212  0.00  0.00      PROA
ATOM   3604  C   ILE P 222      22.153 -11.650 -29.956  0.00  0.00      PROA
ATOM   3605  O   ILE P 222      22.947 -11.502 -30.842  0.00  0.00      PROA
ATOM   3606  N   ILE P 223      22.389 -12.641 -29.004  0.00  0.00      PROA
ATOM   3607  HN  ILE P 223      21.883 -12.741 -28.151  0.00  0.00      PROA
ATOM   3608  CA  ILE P 223      23.628 -13.370 -29.042  0.00  0.00      PROA
ATOM   3609  HA  ILE P 223      24.429 -12.649 -29.118  0.00  0.00      PROA
ATOM   3610  CB  ILE P 223      23.786 -14.163 -27.689  0.00  0.00      PROA
ATOM   3611  HB  ILE P 223      22.779 -14.583 -27.483  0.00  0.00      PROA
ATOM   3612  CG2 ILE P 223      24.742 -15.380 -27.782  0.00  0.00      PROA
ATOM   3613 HG21 ILE P 223      25.720 -14.956 -28.096  0.00  0.00      PROA
ATOM   3614 HG22 ILE P 223      24.489 -16.146 -28.546  0.00  0.00      PROA
ATOM   3615 HG23 ILE P 223      24.901 -15.939 -26.836  0.00  0.00      PROA
ATOM   3616  CG1 ILE P 223      24.052 -13.197 -26.543  0.00  0.00      PROA
ATOM   3617 HG11 ILE P 223      23.902 -13.857 -25.661  0.00  0.00      PROA
ATOM   3618 HG12 ILE P 223      23.255 -12.443 -26.368  0.00  0.00      PROA
ATOM   3619  CD  ILE P 223      25.441 -12.618 -26.557  0.00  0.00      PROA
ATOM   3620  HD1 ILE P 223      25.649 -12.044 -27.485  0.00  0.00      PROA
ATOM   3621  HD2 ILE P 223      26.207 -13.416 -26.453  0.00  0.00      PROA
ATOM   3622  HD3 ILE P 223      25.572 -11.919 -25.703  0.00  0.00      PROA
ATOM   3623  C   ILE P 223      23.822 -14.236 -30.226  0.00  0.00      PROA
ATOM   3624  O   ILE P 223      24.903 -14.309 -30.749  0.00  0.00      PROA
ATOM   3625  N   SER P 224      22.757 -14.962 -30.543  0.00  0.00      PROA
ATOM   3626  HN  SER P 224      21.891 -14.900 -30.053  0.00  0.00      PROA
ATOM   3627  CA  SER P 224      22.778 -15.641 -31.824  0.00  0.00      PROA
ATOM   3628  HA  SER P 224      23.634 -16.299 -31.843  0.00  0.00      PROA
ATOM   3629  CB  SER P 224      21.446 -16.504 -32.012  0.00  0.00      PROA
ATOM   3630  HB1 SER P 224      20.665 -15.756 -31.757  0.00  0.00      PROA
ATOM   3631  HB2 SER P 224      21.327 -17.244 -31.192  0.00  0.00      PROA
ATOM   3632  OG  SER P 224      21.271 -17.038 -33.328  0.00  0.00      PROA
ATOM   3633  HG1 SER P 224      21.890 -17.771 -33.378  0.00  0.00      PROA
ATOM   3634  C   SER P 224      22.966 -14.721 -33.093  0.00  0.00      PROA
ATOM   3635  O   SER P 224      23.808 -15.056 -33.840  0.00  0.00      PROA
ATOM   3636  N   LYS P 225      22.256 -13.594 -33.230  0.00  0.00      PROA
ATOM   3637  HN  LYS P 225      21.513 -13.384 -32.600  0.00  0.00      PROA
ATOM   3638  CA  LYS P 225      22.542 -12.631 -34.227  0.00  0.00      PROA
ATOM   3639  HA  LYS P 225      22.517 -13.286 -35.086  0.00  0.00      PROA
ATOM   3640  CB  LYS P 225      21.408 -11.629 -34.306  0.00  0.00      PROA
ATOM   3641  HB1 LYS P 225      21.178 -11.197 -33.308  0.00  0.00      PROA
ATOM   3642  HB2 LYS P 225      20.422 -12.009 -34.649  0.00  0.00      PROA
ATOM   3643  CG  LYS P 225      21.640 -10.401 -35.150  0.00  0.00      PROA
ATOM   3644  HG1 LYS P 225      22.542  -9.884 -34.756  0.00  0.00      PROA
ATOM   3645  HG2 LYS P 225      20.811  -9.700 -34.915  0.00  0.00      PROA
ATOM   3646  CD  LYS P 225      21.727 -10.684 -36.661  0.00  0.00      PROA
ATOM   3647  HD1 LYS P 225      20.787 -10.612 -37.250  0.00  0.00      PROA
ATOM   3648  HD2 LYS P 225      22.069 -11.734 -36.774  0.00  0.00      PROA
ATOM   3649  CE  LYS P 225      22.719  -9.770 -37.440  0.00  0.00      PROA
ATOM   3650  HE1 LYS P 225      23.689  -9.982 -36.942  0.00  0.00      PROA
ATOM   3651  HE2 LYS P 225      22.381  -8.723 -37.290  0.00  0.00      PROA
ATOM   3652  NZ  LYS P 225      22.921 -10.171 -38.858  0.00  0.00      PROA
ATOM   3653  HZ1 LYS P 225      23.684  -9.580 -39.245  0.00  0.00      PROA
ATOM   3654  HZ2 LYS P 225      22.033 -10.053 -39.386  0.00  0.00      PROA
ATOM   3655  HZ3 LYS P 225      23.210 -11.156 -39.026  0.00  0.00      PROA
ATOM   3656  C   LYS P 225      23.921 -11.979 -34.260  0.00  0.00      PROA
```

```
ATOM   3657  O   LYS P 225      24.642 -11.923 -35.264  0.00  0.00      PROA
ATOM   3658  N   LEU P 226      24.531 -11.656 -33.106  0.00  0.00      PROA
ATOM   3659  HN  LEU P 226      24.005 -11.768 -32.266  0.00  0.00      PROA
ATOM   3660  CA  LEU P 226      25.870 -11.292 -32.835  0.00  0.00      PROA
ATOM   3661  HA  LEU P 226      26.194 -10.456 -33.436  0.00  0.00      PROA
ATOM   3662  CB  LEU P 226      26.143 -10.815 -31.332  0.00  0.00      PROA
ATOM   3663  HB1 LEU P 226      25.938 -11.758 -30.782  0.00  0.00      PROA
ATOM   3664  HB2 LEU P 226      25.397 -10.063 -30.996  0.00  0.00      PROA
ATOM   3665  CG  LEU P 226      27.572 -10.471 -31.048  0.00  0.00      PROA
ATOM   3666  HG  LEU P 226      28.413 -11.125 -31.364  0.00  0.00      PROA
ATOM   3667  CD1 LEU P 226      27.961  -9.127 -31.696  0.00  0.00      PROA
ATOM   3668 HD11 LEU P 226      28.953  -8.878 -31.263  0.00  0.00      PROA
ATOM   3669 HD12 LEU P 226      27.335  -8.227 -31.518  0.00  0.00      PROA
ATOM   3670 HD13 LEU P 226      28.043  -9.243 -32.798  0.00  0.00      PROA
ATOM   3671  CD2 LEU P 226      27.732 -10.241 -29.524  0.00  0.00      PROA
ATOM   3672 HD21 LEU P 226      27.679 -11.270 -29.109  0.00  0.00      PROA
ATOM   3673 HD22 LEU P 226      26.918  -9.586 -29.145  0.00  0.00      PROA
ATOM   3674 HD23 LEU P 226      28.735  -9.991 -29.118  0.00  0.00      PROA
ATOM   3675  C   LEU P 226      26.938 -12.293 -33.244  0.00  0.00      PROA
ATOM   3676  O   LEU P 226      27.957 -11.959 -33.915  0.00  0.00      PROA
ATOM   3677  N   SER P 227      26.641 -13.599 -32.931  0.00  0.00      PROA
ATOM   3678  HN  SER P 227      25.818 -13.811 -32.411  0.00  0.00      PROA
ATOM   3679  CA  SER P 227      27.388 -14.800 -33.394  0.00  0.00      PROA
ATOM   3680  HA  SER P 227      28.385 -14.666 -33.003  0.00  0.00      PROA
ATOM   3681  CB  SER P 227      26.891 -16.131 -32.789  0.00  0.00      PROA
ATOM   3682  HB1 SER P 227      27.585 -16.974 -32.994  0.00  0.00      PROA
ATOM   3683  HB2 SER P 227      25.895 -16.306 -33.249  0.00  0.00      PROA
ATOM   3684  OG  SER P 227      26.842 -16.028 -31.335  0.00  0.00      PROA
ATOM   3685  HG1 SER P 227      26.336 -15.262 -31.054  0.00  0.00      PROA
ATOM   3686  C   SER P 227      27.484 -14.898 -34.896  0.00  0.00      PROA
ATOM   3687  O   SER P 227      28.355 -15.467 -35.486  0.00  0.00      PROA
ATOM   3688  N   HSD P 228      26.493 -14.364 -35.657  0.00  0.00      PROA
ATOM   3689  HN  HSD P 228      25.772 -13.920 -35.131  0.00  0.00      PROA
ATOM   3690  CA  HSD P 228      26.439 -14.410 -37.113  0.00  0.00      PROA
ATOM   3691  HA  HSD P 228      27.203 -15.000 -37.598  0.00  0.00      PROA
ATOM   3692  CB  HSD P 228      25.137 -15.053 -37.578  0.00  0.00      PROA
ATOM   3693  HB1 HSD P 228      25.118 -14.984 -38.686  0.00  0.00      PROA
ATOM   3694  HB2 HSD P 228      24.194 -14.513 -37.343  0.00  0.00      PROA
ATOM   3695  ND1 HSD P 228      25.786 -17.546 -37.396  0.00  0.00      PROA
ATOM   3696  HD1 HSD P 228      26.671 -17.398 -37.838  0.00  0.00      PROA
ATOM   3697  CG  HSD P 228      24.964 -16.503 -37.191  0.00  0.00      PROA
ATOM   3698  CE1 HSD P 228      25.139 -18.664 -37.067  0.00  0.00      PROA
ATOM   3699  HE1 HSD P 228      25.552 -19.672 -37.116  0.00  0.00      PROA
ATOM   3700  NE2 HSD P 228      23.929 -18.359 -36.645  0.00  0.00      PROA
ATOM   3701  CD2 HSD P 228      23.806 -17.016 -36.739  0.00  0.00      PROA
ATOM   3702  HD2 HSD P 228      22.951 -16.471 -36.358  0.00  0.00      PROA
ATOM   3703  C   HSD P 228      26.612 -13.045 -37.709  0.00  0.00      PROA
ATOM   3704  O   HSD P 228      26.350 -12.974 -38.921  0.00  0.00      PROA
ATOM   3705  N   SER P 229      27.154 -12.104 -36.940  0.00  0.00      PROA
ATOM   3706  HN  SER P 229      27.314 -12.223 -35.963  0.00  0.00      PROA
ATOM   3707  CA  SER P 229      27.433 -10.748 -37.436  0.00  0.00      PROA
ATOM   3708  HA  SER P 229      26.484 -10.267 -37.622  0.00  0.00      PROA
ATOM   3709  CB  SER P 229      28.038  -9.918 -36.314  0.00  0.00      PROA
ATOM   3710  HB1 SER P 229      28.990 -10.418 -36.037  0.00  0.00      PROA
ATOM   3711  HB2 SER P 229      27.451  -9.827 -35.375  0.00  0.00      PROA
ATOM   3712  OG  SER P 229      28.326  -8.582 -36.721  0.00  0.00      PROA
ATOM   3713  HG1 SER P 229      27.486  -8.120 -36.778  0.00  0.00      PROA
ATOM   3714  C   SER P 229      28.278 -10.743 -38.687  0.00  0.00      PROA
ATOM   3715  O   SER P 229      29.305 -11.404 -38.776  0.00  0.00      PROA
ATOM   3716  N   LYS P 230      27.974  -9.830 -39.617  0.00  0.00      PROA
ATOM   3717  HN  LYS P 230      27.114  -9.327 -39.572  0.00  0.00      PROA
```

```
ATOM   3718  CA  LYS P 230      28.838  -9.534 -40.737  0.00  0.00      PROA
ATOM   3719  HA  LYS P 230      29.591 -10.293 -40.890  0.00  0.00      PROA
ATOM   3720  CB  LYS P 230      28.105  -9.396 -42.073  0.00  0.00      PROA
ATOM   3721  HB1 LYS P 230      28.729  -8.976 -42.890  0.00  0.00      PROA
ATOM   3722  HB2 LYS P 230      27.239  -8.715 -41.926  0.00  0.00      PROA
ATOM   3723  CG  LYS P 230      27.537 -10.736 -42.617  0.00  0.00      PROA
ATOM   3724  HG1 LYS P 230      26.624 -11.081 -42.086  0.00  0.00      PROA
ATOM   3725  HG2 LYS P 230      28.298 -11.507 -42.371  0.00  0.00      PROA
ATOM   3726  CD  LYS P 230      27.152 -10.725 -44.129  0.00  0.00      PROA
ATOM   3727  HD1 LYS P 230      26.434  -9.890 -44.273  0.00  0.00      PROA
ATOM   3728  HD2 LYS P 230      26.660 -11.703 -44.317  0.00  0.00      PROA
ATOM   3729  CE  LYS P 230      28.312 -10.609 -45.109  0.00  0.00      PROA
ATOM   3730  HE1 LYS P 230      29.191 -11.276 -44.977  0.00  0.00      PROA
ATOM   3731  HE2 LYS P 230      28.560  -9.527 -45.070  0.00  0.00      PROA
ATOM   3732  NZ  LYS P 230      27.850 -10.750 -46.553  0.00  0.00      PROA
ATOM   3733  HZ1 LYS P 230      27.083 -10.079 -46.760  0.00  0.00      PROA
ATOM   3734  HZ2 LYS P 230      27.548 -11.740 -46.662  0.00  0.00      PROA
ATOM   3735  HZ3 LYS P 230      28.627 -10.622 -47.233  0.00  0.00      PROA
ATOM   3736  C   LYS P 230      29.690  -8.256 -40.520  0.00  0.00      PROA
ATOM   3737  O   LYS P 230      30.154  -7.594 -41.441  0.00  0.00      PROA
ATOM   3738  N   GLY P 231      29.814  -7.880 -39.213  0.00  0.00      PROA
ATOM   3739  HN  GLY P 231      29.381  -8.341 -38.442  0.00  0.00      PROA
ATOM   3740  CA  GLY P 231      30.552  -6.688 -38.896  0.00  0.00      PROA
ATOM   3741  HA1 GLY P 231      31.600  -6.811 -39.126  0.00  0.00      PROA
ATOM   3742  HA2 GLY P 231      30.313  -6.607 -37.846  0.00  0.00      PROA
ATOM   3743  C   GLY P 231      30.039  -5.351 -39.320  0.00  0.00      PROA
ATOM   3744  O   GLY P 231      28.870  -5.177 -39.534  0.00  0.00      PROA
ATOM   3745  N   HSD P 232      30.908  -4.331 -39.315  0.00  0.00      PROA
ATOM   3746  HN  HSD P 232      31.862  -4.592 -39.187  0.00  0.00      PROA
ATOM   3747  CA  HSD P 232      30.826  -2.901 -39.453  0.00  0.00      PROA
ATOM   3748  HA  HSD P 232      31.827  -2.500 -39.515  0.00  0.00      PROA
ATOM   3749  CB  HSD P 232      30.178  -2.542 -40.723  0.00  0.00      PROA
ATOM   3750  HB1 HSD P 232      30.204  -1.444 -40.892  0.00  0.00      PROA
ATOM   3751  HB2 HSD P 232      29.105  -2.775 -40.551  0.00  0.00      PROA
ATOM   3752  ND1 HSD P 232      31.369  -2.630 -42.930  0.00  0.00      PROA
ATOM   3753  HD1 HSD P 232      32.006  -1.864 -42.841  0.00  0.00      PROA
ATOM   3754  CG  HSD P 232      30.657  -3.298 -41.985  0.00  0.00      PROA
ATOM   3755  CE1 HSD P 232      31.462  -3.413 -44.027  0.00  0.00      PROA
ATOM   3756  HE1 HSD P 232      32.020  -3.128 -44.920  0.00  0.00      PROA
ATOM   3757  NE2 HSD P 232      30.690  -4.484 -43.906  0.00  0.00      PROA
ATOM   3758  CD2 HSD P 232      30.246  -4.446 -42.563  0.00  0.00      PROA
ATOM   3759  HD2 HSD P 232      29.589  -5.201 -42.149  0.00  0.00      PROA
ATOM   3760  C   HSD P 232      30.102  -2.162 -38.361  0.00  0.00      PROA
ATOM   3761  O   HSD P 232      29.745  -2.706 -37.374  0.00  0.00      PROA
ATOM   3762  N   GLN P 233      29.883  -0.882 -38.321  0.00  0.00      PROA
ATOM   3763  HN  GLN P 233      30.342  -0.362 -39.038  0.00  0.00      PROA
ATOM   3764  CA  GLN P 233      29.224  -0.168 -37.345  0.00  0.00      PROA
ATOM   3765  HA  GLN P 233      29.961  -0.225 -36.558  0.00  0.00      PROA
ATOM   3766  CB  GLN P 233      29.045   1.346 -37.754  0.00  0.00      PROA
ATOM   3767  HB1 GLN P 233      28.618   1.821 -36.845  0.00  0.00      PROA
ATOM   3768  HB2 GLN P 233      28.231   1.377 -38.509  0.00  0.00      PROA
ATOM   3769  CG  GLN P 233      30.323   2.240 -38.183  0.00  0.00      PROA
ATOM   3770  HG1 GLN P 233      29.944   3.275 -38.046  0.00  0.00      PROA
ATOM   3771  HG2 GLN P 233      30.662   2.062 -39.225  0.00  0.00      PROA
ATOM   3772  CD  GLN P 233      31.466   2.070 -37.188  0.00  0.00      PROA
ATOM   3773  OE1 GLN P 233      31.418   2.584 -36.069  0.00  0.00      PROA
ATOM   3774  NE2 GLN P 233      32.540   1.390 -37.581  0.00  0.00      PROA
ATOM   3775 HE21 GLN P 233      33.390   1.618 -37.106  0.00  0.00      PROA
ATOM   3776 HE22 GLN P 233      32.745   1.308 -38.556  0.00  0.00      PROA
ATOM   3777  C   GLN P 233      27.980  -0.654 -36.721  0.00  0.00      PROA
ATOM   3778  O   GLN P 233      27.711  -0.438 -35.509  0.00  0.00      PROA
```

ATOM 3779 N LYS P 234 27.114 -1.333 -37.520 0.00 0.00 PROA
ATOM 3780 HN LYS P 234 27.292 -1.179 -38.489 0.00 0.00 PROA
ATOM 3781 CA LYS P 234 26.046 -2.220 -37.136 0.00 0.00 PROA
ATOM 3782 HA LYS P 234 25.349 -1.543 -36.664 0.00 0.00 PROA
ATOM 3783 CB LYS P 234 25.231 -2.836 -38.337 0.00 0.00 PROA
ATOM 3784 HB1 LYS P 234 24.650 -2.044 -38.857 0.00 0.00 PROA
ATOM 3785 HB2 LYS P 234 24.505 -3.611 -38.009 0.00 0.00 PROA
ATOM 3786 CG LYS P 234 26.156 -3.600 -39.282 0.00 0.00 PROA
ATOM 3787 HG1 LYS P 234 26.605 -4.544 -38.905 0.00 0.00 PROA
ATOM 3788 HG2 LYS P 234 27.069 -2.994 -39.469 0.00 0.00 PROA
ATOM 3789 CD LYS P 234 25.521 -3.797 -40.681 0.00 0.00 PROA
ATOM 3790 HD1 LYS P 234 25.108 -2.800 -40.945 0.00 0.00 PROA
ATOM 3791 HD2 LYS P 234 24.600 -4.418 -40.662 0.00 0.00 PROA
ATOM 3792 CE LYS P 234 26.530 -4.305 -41.727 0.00 0.00 PROA
ATOM 3793 HE1 LYS P 234 27.487 -3.782 -41.512 0.00 0.00 PROA
ATOM 3794 HE2 LYS P 234 26.237 -4.114 -42.781 0.00 0.00 PROA
ATOM 3795 NZ LYS P 234 26.763 -5.664 -41.425 0.00 0.00 PROA
ATOM 3796 HZ1 LYS P 234 26.945 -6.053 -42.372 0.00 0.00 PROA
ATOM 3797 HZ2 LYS P 234 26.003 -6.266 -41.048 0.00 0.00 PROA
ATOM 3798 HZ3 LYS P 234 27.585 -5.784 -40.800 0.00 0.00 PROA
ATOM 3799 C LYS P 234 26.303 -3.276 -36.050 0.00 0.00 PROA
ATOM 3800 O LYS P 234 25.414 -3.608 -35.286 0.00 0.00 PROA
ATOM 3801 N ARG P 235 27.527 -3.759 -35.791 0.00 0.00 PROA
ATOM 3802 HN ARG P 235 28.256 -3.478 -36.411 0.00 0.00 PROA
ATOM 3803 CA ARG P 235 27.852 -4.638 -34.659 0.00 0.00 PROA
ATOM 3804 HA ARG P 235 27.020 -5.318 -34.553 0.00 0.00 PROA
ATOM 3805 CB ARG P 235 29.098 -5.419 -34.848 0.00 0.00 PROA
ATOM 3806 HB1 ARG P 235 29.902 -4.658 -34.939 0.00 0.00 PROA
ATOM 3807 HB2 ARG P 235 29.103 -5.977 -35.808 0.00 0.00 PROA
ATOM 3808 CG ARG P 235 29.422 -6.273 -33.595 0.00 0.00 PROA
ATOM 3809 HG1 ARG P 235 28.524 -6.916 -33.476 0.00 0.00 PROA
ATOM 3810 HG2 ARG P 235 29.606 -5.603 -32.728 0.00 0.00 PROA
ATOM 3811 CD ARG P 235 30.599 -7.156 -33.760 0.00 0.00 PROA
ATOM 3812 HD1 ARG P 235 30.403 -7.871 -34.588 0.00 0.00 PROA
ATOM 3813 HD2 ARG P 235 30.748 -7.711 -32.809 0.00 0.00 PROA
ATOM 3814 NE ARG P 235 31.737 -6.282 -33.994 0.00 0.00 PROA
ATOM 3815 HE ARG P 235 31.832 -5.440 -33.462 0.00 0.00 PROA
ATOM 3816 CZ ARG P 235 32.597 -6.418 -34.972 0.00 0.00 PROA
ATOM 3817 NH1 ARG P 235 32.696 -7.550 -35.623 0.00 0.00 PROA
ATOM 3818 HH11 ARG P 235 32.157 -8.299 -35.241 0.00 0.00 PROA
ATOM 3819 HH12 ARG P 235 33.394 -7.644 -36.333 0.00 0.00 PROA
ATOM 3820 NH2 ARG P 235 33.442 -5.459 -35.168 0.00 0.00 PROA
ATOM 3821 HH21 ARG P 235 33.308 -4.548 -34.780 0.00 0.00 PROA
ATOM 3822 HH22 ARG P 235 34.165 -5.642 -35.834 0.00 0.00 PROA
ATOM 3823 C ARG P 235 27.899 -3.915 -33.348 0.00 0.00 PROA
ATOM 3824 O ARG P 235 27.565 -4.505 -32.334 0.00 0.00 PROA
ATOM 3825 N LYS P 236 28.220 -2.621 -33.334 0.00 0.00 PROA
ATOM 3826 HN LYS P 236 28.401 -2.341 -34.273 0.00 0.00 PROA
ATOM 3827 CA LYS P 236 28.349 -1.766 -32.144 0.00 0.00 PROA
ATOM 3828 HA LYS P 236 28.980 -2.349 -31.490 0.00 0.00 PROA
ATOM 3829 CB LYS P 236 28.865 -0.363 -32.373 0.00 0.00 PROA
ATOM 3830 HB1 LYS P 236 29.039 0.184 -31.422 0.00 0.00 PROA
ATOM 3831 HB2 LYS P 236 28.056 0.275 -32.788 0.00 0.00 PROA
ATOM 3832 CG LYS P 236 30.220 -0.302 -33.081 0.00 0.00 PROA
ATOM 3833 HG1 LYS P 236 29.919 -0.477 -34.136 0.00 0.00 PROA
ATOM 3834 HG2 LYS P 236 30.903 -1.083 -32.682 0.00 0.00 PROA
ATOM 3835 CD LYS P 236 30.805 1.126 -33.134 0.00 0.00 PROA
ATOM 3836 HD1 LYS P 236 30.544 1.627 -32.178 0.00 0.00 PROA
ATOM 3837 HD2 LYS P 236 30.294 1.778 -33.874 0.00 0.00 PROA
ATOM 3838 CE LYS P 236 32.319 1.192 -33.536 0.00 0.00 PROA
ATOM 3839 HE1 LYS P 236 32.441 0.829 -34.579 0.00 0.00 PROA

ATOM	3840	HE2	LYS	P	236	32.994	0.632	-32.854	0.00	0.00	PROA
ATOM	3841	NZ	LYS	P	236	32.822	2.551	-33.531	0.00	0.00	PROA
ATOM	3842	HZ1	LYS	P	236	33.748	2.643	-33.996	0.00	0.00	PROA
ATOM	3843	HZ2	LYS	P	236	32.943	3.029	-32.616	0.00	0.00	PROA
ATOM	3844	HZ3	LYS	P	236	32.109	2.986	-34.150	0.00	0.00	PROA
ATOM	3845	C	LYS	P	236	26.929	-1.689	-31.507	0.00	0.00	PROA
ATOM	3846	O	LYS	P	236	26.784	-1.653	-30.298	0.00	0.00	PROA
ATOM	3847	N	ALA	P	237	25.888	-1.665	-32.340	0.00	0.00	PROA
ATOM	3848	HN	ALA	P	237	26.030	-1.695	-33.326	0.00	0.00	PROA
ATOM	3849	CA	ALA	P	237	24.442	-1.664	-31.921	0.00	0.00	PROA
ATOM	3850	HA	ALA	P	237	24.247	-0.789	-31.320	0.00	0.00	PROA
ATOM	3851	CB	ALA	P	237	23.590	-1.657	-33.227	0.00	0.00	PROA
ATOM	3852	HB1	ALA	P	237	23.729	-0.636	-33.643	0.00	0.00	PROA
ATOM	3853	HB2	ALA	P	237	22.498	-1.780	-33.070	0.00	0.00	PROA
ATOM	3854	HB3	ALA	P	237	23.904	-2.356	-34.032	0.00	0.00	PROA
ATOM	3855	C	ALA	P	237	24.141	-2.938	-31.083	0.00	0.00	PROA
ATOM	3856	O	ALA	P	237	23.598	-2.871	-29.961	0.00	0.00	PROA
ATOM	3857	N	LEU	P	238	24.506	-4.103	-31.615	0.00	0.00	PROA
ATOM	3858	HN	LEU	P	238	24.911	-4.154	-32.525	0.00	0.00	PROA
ATOM	3859	CA	LEU	P	238	24.313	-5.358	-30.854	0.00	0.00	PROA
ATOM	3860	HA	LEU	P	238	23.276	-5.465	-30.574	0.00	0.00	PROA
ATOM	3861	CB	LEU	P	238	24.751	-6.620	-31.697	0.00	0.00	PROA
ATOM	3862	HB1	LEU	P	238	24.517	-7.611	-31.252	0.00	0.00	PROA
ATOM	3863	HB2	LEU	P	238	25.856	-6.600	-31.809	0.00	0.00	PROA
ATOM	3864	CG	LEU	P	238	24.019	-6.798	-33.019	0.00	0.00	PROA
ATOM	3865	HG	LEU	P	238	24.205	-5.956	-33.720	0.00	0.00	PROA
ATOM	3866	CD1	LEU	P	238	24.434	-8.058	-33.795	0.00	0.00	PROA
ATOM	3867	HD11	LEU	P	238	23.949	-8.005	-34.793	0.00	0.00	PROA
ATOM	3868	HD12	LEU	P	238	24.014	-8.925	-33.241	0.00	0.00	PROA
ATOM	3869	HD13	LEU	P	238	25.508	-8.060	-34.077	0.00	0.00	PROA
ATOM	3870	CD2	LEU	P	238	22.493	-6.862	-32.848	0.00	0.00	PROA
ATOM	3871	HD21	LEU	P	238	21.965	-7.381	-33.677	0.00	0.00	PROA
ATOM	3872	HD22	LEU	P	238	22.034	-5.854	-32.769	0.00	0.00	PROA
ATOM	3873	HD23	LEU	P	238	22.159	-7.414	-31.943	0.00	0.00	PROA
ATOM	3874	C	LEU	P	238	25.056	-5.418	-29.523	0.00	0.00	PROA
ATOM	3875	O	LEU	P	238	24.485	-5.835	-28.510	0.00	0.00	PROA
ATOM	3876	N	LYS	P	239	26.348	-4.937	-29.534	0.00	0.00	PROA
ATOM	3877	HN	LYS	P	239	26.753	-4.638	-30.394	0.00	0.00	PROA
ATOM	3878	CA	LYS	P	239	27.229	-4.927	-28.362	0.00	0.00	PROA
ATOM	3879	HA	LYS	P	239	27.154	-5.931	-27.971	0.00	0.00	PROA
ATOM	3880	CB	LYS	P	239	28.696	-4.573	-28.792	0.00	0.00	PROA
ATOM	3881	HB1	LYS	P	239	29.270	-4.314	-27.877	0.00	0.00	PROA
ATOM	3882	HB2	LYS	P	239	28.606	-3.732	-29.513	0.00	0.00	PROA
ATOM	3883	CG	LYS	P	239	29.382	-5.803	-29.469	0.00	0.00	PROA
ATOM	3884	HG1	LYS	P	239	30.243	-5.358	-30.012	0.00	0.00	PROA
ATOM	3885	HG2	LYS	P	239	28.677	-6.307	-30.163	0.00	0.00	PROA
ATOM	3886	CD	LYS	P	239	29.847	-6.943	-28.524	0.00	0.00	PROA
ATOM	3887	HD1	LYS	P	239	30.497	-7.629	-29.108	0.00	0.00	PROA
ATOM	3888	HD2	LYS	P	239	28.941	-7.533	-28.270	0.00	0.00	PROA
ATOM	3889	CE	LYS	P	239	30.527	-6.567	-27.156	0.00	0.00	PROA
ATOM	3890	HE1	LYS	P	239	29.734	-6.196	-26.471	0.00	0.00	PROA
ATOM	3891	HE2	LYS	P	239	31.312	-5.797	-27.314	0.00	0.00	PROA
ATOM	3892	NZ	LYS	P	239	31.202	-7.770	-26.615	0.00	0.00	PROA
ATOM	3893	HZ1	LYS	P	239	31.582	-7.574	-25.667	0.00	0.00	PROA
ATOM	3894	HZ2	LYS	P	239	31.967	-8.174	-27.192	0.00	0.00	PROA
ATOM	3895	HZ3	LYS	P	239	30.469	-8.497	-26.492	0.00	0.00	PROA
ATOM	3896	C	LYS	P	239	26.811	-4.087	-27.209	0.00	0.00	PROA
ATOM	3897	O	LYS	P	239	26.881	-4.585	-26.106	0.00	0.00	PROA
ATOM	3898	N	THR	P	240	26.201	-2.931	-27.531	0.00	0.00	PROA
ATOM	3899	HN	THR	P	240	26.191	-2.612	-28.476	0.00	0.00	PROA
ATOM	3900	CA	THR	P	240	25.580	-2.033	-26.612	0.00	0.00	PROA

```
ATOM   3901  HA  THR P 240      26.258  -1.627 -25.875  0.00  0.00      PROA
ATOM   3902  CB  THR P 240      25.074  -0.771 -27.300  0.00  0.00      PROA
ATOM   3903  HB  THR P 240      24.441  -1.091 -28.156  0.00  0.00      PROA
ATOM   3904  OG1 THR P 240      26.189   0.089 -27.711  0.00  0.00      PROA
ATOM   3905  HG1 THR P 240      26.825  -0.427 -28.211  0.00  0.00      PROA
ATOM   3906  CG2 THR P 240      24.177   0.139 -26.479  0.00  0.00      PROA
ATOM   3907 HG21 THR P 240      23.454  -0.476 -25.902  0.00  0.00      PROA
ATOM   3908 HG22 THR P 240      23.630   0.814 -27.171  0.00  0.00      PROA
ATOM   3909 HG23 THR P 240      24.725   0.727 -25.710  0.00  0.00      PROA
ATOM   3910  C   THR P 240      24.432  -2.677 -25.809  0.00  0.00      PROA
ATOM   3911  O   THR P 240      24.389  -2.608 -24.518  0.00  0.00      PROA
ATOM   3912  N   THR P 241      23.563  -3.420 -26.543  0.00  0.00      PROA
ATOM   3913  HN  THR P 241      23.587  -3.404 -27.540  0.00  0.00      PROA
ATOM   3914  CA  THR P 241      22.457  -4.209 -26.012  0.00  0.00      PROA
ATOM   3915  HA  THR P 241      21.858  -3.531 -25.423  0.00  0.00      PROA
ATOM   3916  CB  THR P 241      21.531  -4.647 -27.075  0.00  0.00      PROA
ATOM   3917  HB  THR P 241      21.866  -5.591 -27.556  0.00  0.00      PROA
ATOM   3918  OG1 THR P 241      21.404  -3.718 -28.097  0.00  0.00      PROA
ATOM   3919  HG1 THR P 241      21.073  -4.250 -28.825  0.00  0.00      PROA
ATOM   3920  CG2 THR P 241      20.084  -4.844 -26.534  0.00  0.00      PROA
ATOM   3921 HG21 THR P 241      19.800  -3.864 -26.094  0.00  0.00      PROA
ATOM   3922 HG22 THR P 241      20.026  -5.531 -25.663  0.00  0.00      PROA
ATOM   3923 HG23 THR P 241      19.400  -5.240 -27.315  0.00  0.00      PROA
ATOM   3924  C   THR P 241      22.906  -5.390 -25.091  0.00  0.00      PROA
ATOM   3925  O   THR P 241      22.362  -5.602 -24.005  0.00  0.00      PROA
ATOM   3926  N   VAL P 242      23.872  -6.221 -25.533  0.00  0.00      PROA
ATOM   3927  HN  VAL P 242      24.379  -6.058 -26.376  0.00  0.00      PROA
ATOM   3928  CA  VAL P 242      24.390  -7.362 -24.778  0.00  0.00      PROA
ATOM   3929  HA  VAL P 242      23.506  -7.940 -24.557  0.00  0.00      PROA
ATOM   3930  CB  VAL P 242      25.386  -8.195 -25.569  0.00  0.00      PROA
ATOM   3931  HB  VAL P 242      26.132  -7.487 -25.987  0.00  0.00      PROA
ATOM   3932  CG1 VAL P 242      26.161  -9.190 -24.685  0.00  0.00      PROA
ATOM   3933 HG11 VAL P 242      25.520  -9.732 -23.957  0.00  0.00      PROA
ATOM   3934 HG12 VAL P 242      26.995  -8.686 -24.151  0.00  0.00      PROA
ATOM   3935 HG13 VAL P 242      26.506 -10.053 -25.294  0.00  0.00      PROA
ATOM   3936  CG2 VAL P 242      24.656  -8.848 -26.719  0.00  0.00      PROA
ATOM   3937 HG21 VAL P 242      25.143  -9.647 -27.318  0.00  0.00      PROA
ATOM   3938 HG22 VAL P 242      24.300  -8.132 -27.490  0.00  0.00      PROA
ATOM   3939 HG23 VAL P 242      23.756  -9.301 -26.251  0.00  0.00      PROA
ATOM   3940  C   VAL P 242      24.993  -7.054 -23.388  0.00  0.00      PROA
ATOM   3941  O   VAL P 242      24.676  -7.586 -22.355  0.00  0.00      PROA
ATOM   3942  N   ILE P 243      25.826  -5.968 -23.334  0.00  0.00      PROA
ATOM   3943  HN  ILE P 243      25.991  -5.434 -24.160  0.00  0.00      PROA
ATOM   3944  CA  ILE P 243      26.481  -5.490 -22.152  0.00  0.00      PROA
ATOM   3945  HA  ILE P 243      26.757  -6.343 -21.549  0.00  0.00      PROA
ATOM   3946  CB  ILE P 243      27.812  -4.682 -22.429  0.00  0.00      PROA
ATOM   3947  HB  ILE P 243      28.225  -4.443 -21.425  0.00  0.00      PROA
ATOM   3948  CG2 ILE P 243      28.834  -5.634 -23.298  0.00  0.00      PROA
ATOM   3949 HG21 ILE P 243      29.123  -6.550 -22.740  0.00  0.00      PROA
ATOM   3950 HG22 ILE P 243      29.758  -5.042 -23.477  0.00  0.00      PROA
ATOM   3951 HG23 ILE P 243      28.388  -5.890 -24.283  0.00  0.00      PROA
ATOM   3952  CG1 ILE P 243      27.407  -3.251 -23.059  0.00  0.00      PROA
ATOM   3953 HG11 ILE P 243      27.083  -2.670 -22.169  0.00  0.00      PROA
ATOM   3954 HG12 ILE P 243      26.607  -3.438 -23.806  0.00  0.00      PROA
ATOM   3955  CD  ILE P 243      28.505  -2.603 -23.802  0.00  0.00      PROA
ATOM   3956  HD1 ILE P 243      29.316  -2.286 -23.112  0.00  0.00      PROA
ATOM   3957  HD2 ILE P 243      28.234  -1.689 -24.374  0.00  0.00      PROA
ATOM   3958  HD3 ILE P 243      28.917  -3.356 -24.507  0.00  0.00      PROA
ATOM   3959  C   ILE P 243      25.559  -4.708 -21.209  0.00  0.00      PROA
ATOM   3960  O   ILE P 243      25.731  -4.834 -19.997  0.00  0.00      PROA
ATOM   3961  N   LEU P 244      24.495  -3.998 -21.708  0.00  0.00      PROA
```

ATOM 3962 HN LEU P 244 24.474 -3.870 -22.697 0.00 0.00 PROA
ATOM 3963 CA LEU P 244 23.358 -3.486 -20.988 0.00 0.00 PROA
ATOM 3964 HA LEU P 244 23.766 -2.776 -20.284 0.00 0.00 PROA
ATOM 3965 CB LEU P 244 22.400 -2.715 -21.939 0.00 0.00 PROA
ATOM 3966 HB1 LEU P 244 22.096 -3.341 -22.806 0.00 0.00 PROA
ATOM 3967 HB2 LEU P 244 23.020 -1.872 -22.311 0.00 0.00 PROA
ATOM 3968 CG LEU P 244 21.175 -2.040 -21.202 0.00 0.00 PROA
ATOM 3969 HG LEU P 244 20.573 -2.789 -20.644 0.00 0.00 PROA
ATOM 3970 CD1 LEU P 244 21.502 -0.951 -20.191 0.00 0.00 PROA
ATOM 3971 HD11 LEU P 244 20.650 -0.694 -19.526 0.00 0.00 PROA
ATOM 3972 HD12 LEU P 244 21.832 -0.052 -20.754 0.00 0.00 PROA
ATOM 3973 HD13 LEU P 244 22.420 -1.312 -19.679 0.00 0.00 PROA
ATOM 3974 CD2 LEU P 244 20.328 -1.346 -22.202 0.00 0.00 PROA
ATOM 3975 HD21 LEU P 244 20.189 -2.071 -23.032 0.00 0.00 PROA
ATOM 3976 HD22 LEU P 244 20.686 -0.380 -22.618 0.00 0.00 PROA
ATOM 3977 HD23 LEU P 244 19.317 -1.084 -21.823 0.00 0.00 PROA
ATOM 3978 C LEU P 244 22.724 -4.565 -20.145 0.00 0.00 PROA
ATOM 3979 O LEU P 244 22.505 -4.306 -18.986 0.00 0.00 PROA
ATOM 3980 N ILE P 245 22.439 -5.696 -20.728 0.00 0.00 PROA
ATOM 3981 HN ILE P 245 22.666 -5.946 -21.666 0.00 0.00 PROA
ATOM 3982 CA ILE P 245 21.715 -6.695 -19.980 0.00 0.00 PROA
ATOM 3983 HA ILE P 245 21.083 -6.063 -19.373 0.00 0.00 PROA
ATOM 3984 CB ILE P 245 21.017 -7.596 -21.047 0.00 0.00 PROA
ATOM 3985 HB ILE P 245 21.863 -7.821 -21.730 0.00 0.00 PROA
ATOM 3986 CG2 ILE P 245 20.504 -8.944 -20.413 0.00 0.00 PROA
ATOM 3987 HG21 ILE P 245 19.678 -8.921 -19.670 0.00 0.00 PROA
ATOM 3988 HG22 ILE P 245 21.381 -9.488 -20.002 0.00 0.00 PROA
ATOM 3989 HG23 ILE P 245 19.989 -9.398 -21.287 0.00 0.00 PROA
ATOM 3990 CG1 ILE P 245 19.971 -6.852 -21.862 0.00 0.00 PROA
ATOM 3991 HG11 ILE P 245 20.297 -5.874 -22.277 0.00 0.00 PROA
ATOM 3992 HG12 ILE P 245 19.259 -6.386 -21.148 0.00 0.00 PROA
ATOM 3993 CD ILE P 245 19.429 -7.605 -23.084 0.00 0.00 PROA
ATOM 3994 HD1 ILE P 245 18.696 -7.016 -23.676 0.00 0.00 PROA
ATOM 3995 HD2 ILE P 245 18.768 -8.464 -22.838 0.00 0.00 PROA
ATOM 3996 HD3 ILE P 245 20.248 -7.883 -23.781 0.00 0.00 PROA
ATOM 3997 C ILE P 245 22.547 -7.500 -19.040 0.00 0.00 PROA
ATOM 3998 O ILE P 245 22.066 -7.789 -17.966 0.00 0.00 PROA
ATOM 3999 N LEU P 246 23.800 -7.799 -19.368 0.00 0.00 PROA
ATOM 4000 HN LEU P 246 24.070 -7.649 -20.316 0.00 0.00 PROA
ATOM 4001 CA LEU P 246 24.829 -8.323 -18.534 0.00 0.00 PROA
ATOM 4002 HA LEU P 246 24.454 -9.271 -18.179 0.00 0.00 PROA
ATOM 4003 CB LEU P 246 26.061 -8.828 -19.373 0.00 0.00 PROA
ATOM 4004 HB1 LEU P 246 26.768 -9.112 -18.564 0.00 0.00 PROA
ATOM 4005 HB2 LEU P 246 26.345 -7.917 -19.942 0.00 0.00 PROA
ATOM 4006 CG LEU P 246 25.797 -10.008 -20.309 0.00 0.00 PROA
ATOM 4007 HG LEU P 246 24.941 -9.761 -20.974 0.00 0.00 PROA
ATOM 4008 CD1 LEU P 246 26.933 -10.012 -21.310 0.00 0.00 PROA
ATOM 4009 HD11 LEU P 246 27.898 -10.026 -20.761 0.00 0.00 PROA
ATOM 4010 HD12 LEU P 246 26.992 -9.076 -21.907 0.00 0.00 PROA
ATOM 4011 HD13 LEU P 246 26.858 -10.853 -22.033 0.00 0.00 PROA
ATOM 4012 CD2 LEU P 246 25.701 -11.376 -19.599 0.00 0.00 PROA
ATOM 4013 HD21 LEU P 246 24.919 -11.612 -18.846 0.00 0.00 PROA
ATOM 4014 HD22 LEU P 246 26.586 -11.567 -18.956 0.00 0.00 PROA
ATOM 4015 HD23 LEU P 246 25.711 -12.158 -20.388 0.00 0.00 PROA
ATOM 4016 C LEU P 246 25.140 -7.449 -17.300 0.00 0.00 PROA
ATOM 4017 O LEU P 246 25.243 -8.014 -16.219 0.00 0.00 PROA
ATOM 4018 N ALA P 247 25.214 -6.143 -17.485 0.00 0.00 PROA
ATOM 4019 HN ALA P 247 25.084 -5.610 -18.318 0.00 0.00 PROA
ATOM 4020 CA ALA P 247 25.201 -5.250 -16.303 0.00 0.00 PROA
ATOM 4021 HA ALA P 247 26.060 -5.466 -15.685 0.00 0.00 PROA
ATOM 4022 CB ALA P 247 25.417 -3.755 -16.820 0.00 0.00 PROA

ATOM	4023	HB1	ALA	P	247	26.418	-3.726	-17.302	0.00	0.00	PROA
ATOM	4024	HB2	ALA	P	247	25.240	-2.985	-16.039	0.00	0.00	PROA
ATOM	4025	HB3	ALA	P	247	24.551	-3.535	-17.481	0.00	0.00	PROA
ATOM	4026	C	ALA	P	247	23.976	-5.326	-15.343	0.00	0.00	PROA
ATOM	4027	O	ALA	P	247	24.199	-5.375	-14.151	0.00	0.00	PROA
ATOM	4028	N	PHE	P	248	22.744	-5.342	-15.801	0.00	0.00	PROA
ATOM	4029	HN	PHE	P	248	22.721	-5.102	-16.768	0.00	0.00	PROA
ATOM	4030	CA	PHE	P	248	21.540	-5.592	-14.987	0.00	0.00	PROA
ATOM	4031	HA	PHE	P	248	21.526	-4.868	-14.186	0.00	0.00	PROA
ATOM	4032	CB	PHE	P	248	20.235	-5.464	-15.835	0.00	0.00	PROA
ATOM	4033	HB1	PHE	P	248	20.325	-6.124	-16.724	0.00	0.00	PROA
ATOM	4034	HB2	PHE	P	248	20.161	-4.405	-16.163	0.00	0.00	PROA
ATOM	4035	CG	PHE	P	248	18.980	-5.602	-15.069	0.00	0.00	PROA
ATOM	4036	CD1	PHE	P	248	18.703	-4.695	-14.031	0.00	0.00	PROA
ATOM	4037	HD1	PHE	P	248	19.371	-3.866	-13.848	0.00	0.00	PROA
ATOM	4038	CE1	PHE	P	248	17.545	-4.800	-13.288	0.00	0.00	PROA
ATOM	4039	HE1	PHE	P	248	17.376	-4.143	-12.448	0.00	0.00	PROA
ATOM	4040	CZ	PHE	P	248	16.708	-5.901	-13.467	0.00	0.00	PROA
ATOM	4041	HZ	PHE	P	248	15.789	-5.947	-12.901	0.00	0.00	PROA
ATOM	4042	CD2	PHE	P	248	18.080	-6.671	-15.280	0.00	0.00	PROA
ATOM	4043	HD2	PHE	P	248	18.366	-7.323	-16.092	0.00	0.00	PROA
ATOM	4044	CE2	PHE	P	248	16.983	-6.803	-14.486	0.00	0.00	PROA
ATOM	4045	HE2	PHE	P	248	16.390	-7.702	-14.566	0.00	0.00	PROA
ATOM	4046	C	PHE	P	248	21.655	-7.016	-14.287	0.00	0.00	PROA
ATOM	4047	O	PHE	P	248	21.482	-7.226	-13.092	0.00	0.00	PROA
ATOM	4048	N	PHE	P	249	22.000	-8.122	-15.052	0.00	0.00	PROA
ATOM	4049	HN	PHE	P	249	22.246	-7.989	-16.008	0.00	0.00	PROA
ATOM	4050	CA	PHE	P	249	22.078	-9.416	-14.373	0.00	0.00	PROA
ATOM	4051	HA	PHE	P	249	21.123	-9.487	-13.873	0.00	0.00	PROA
ATOM	4052	CB	PHE	P	249	22.029	-10.653	-15.404	0.00	0.00	PROA
ATOM	4053	HB1	PHE	P	249	22.079	-11.598	-14.822	0.00	0.00	PROA
ATOM	4054	HB2	PHE	P	249	22.911	-10.518	-16.066	0.00	0.00	PROA
ATOM	4055	CG	PHE	P	249	20.764	-10.810	-16.276	0.00	0.00	PROA
ATOM	4056	CD1	PHE	P	249	19.594	-10.138	-15.953	0.00	0.00	PROA
ATOM	4057	HD1	PHE	P	249	19.290	-9.600	-15.067	0.00	0.00	PROA
ATOM	4058	CE1	PHE	P	249	18.513	-10.252	-16.829	0.00	0.00	PROA
ATOM	4059	HE1	PHE	P	249	17.657	-9.616	-16.658	0.00	0.00	PROA
ATOM	4060	CZ	PHE	P	249	18.579	-10.968	-18.063	0.00	0.00	PROA
ATOM	4061	HZ	PHE	P	249	17.789	-11.037	-18.797	0.00	0.00	PROA
ATOM	4062	CD2	PHE	P	249	20.809	-11.571	-17.428	0.00	0.00	PROA
ATOM	4063	HD2	PHE	P	249	21.699	-12.044	-17.816	0.00	0.00	PROA
ATOM	4064	CE2	PHE	P	249	19.658	-11.784	-18.271	0.00	0.00	PROA
ATOM	4065	HE2	PHE	P	249	19.662	-12.249	-19.246	0.00	0.00	PROA
ATOM	4066	C	PHE	P	249	23.223	-9.646	-13.309	0.00	0.00	PROA
ATOM	4067	O	PHE	P	249	22.993	-10.236	-12.206	0.00	0.00	PROA
ATOM	4068	N	ALA	P	250	24.415	-9.144	-13.606	0.00	0.00	PROA
ATOM	4069	HN	ALA	P	250	24.493	-8.579	-14.424	0.00	0.00	PROA
ATOM	4070	CA	ALA	P	250	25.516	-9.064	-12.650	0.00	0.00	PROA
ATOM	4071	HA	ALA	P	250	25.742	-10.033	-12.231	0.00	0.00	PROA
ATOM	4072	CB	ALA	P	250	26.623	-8.448	-13.464	0.00	0.00	PROA
ATOM	4073	HB1	ALA	P	250	27.462	-8.292	-12.753	0.00	0.00	PROA
ATOM	4074	HB2	ALA	P	250	26.305	-7.461	-13.864	0.00	0.00	PROA
ATOM	4075	HB3	ALA	P	250	26.989	-9.092	-14.291	0.00	0.00	PROA
ATOM	4076	C	ALA	P	250	25.248	-8.325	-11.418	0.00	0.00	PROA
ATOM	4077	O	ALA	P	250	25.505	-8.817	-10.345	0.00	0.00	PROA
ATOM	4078	N	CYS	P	251	24.725	-7.113	-11.518	0.00	0.00	PROA
ATOM	4079	HN	CYS	P	251	24.514	-6.697	-12.400	0.00	0.00	PROA
ATOM	4080	CA	CYS	P	251	24.500	-6.253	-10.355	0.00	0.00	PROA
ATOM	4081	HA	CYS	P	251	25.316	-6.356	-9.655	0.00	0.00	PROA
ATOM	4082	CB	CYS	P	251	24.362	-4.752	-10.927	0.00	0.00	PROA
ATOM	4083	HB1	CYS	P	251	23.839	-3.958	-10.351	0.00	0.00	PROA

ATOM	4084	HB2	CYS	P	251	23.683	-4.954	-11.783	0.00	0.00	PROA
ATOM	4085	SG	CYS	P	251	25.953	-3.922	-11.387	0.00	0.00	PROA
ATOM	4086	HG1	CYS	P	251	26.002	-3.153	-10.309	0.00	0.00	PROA
ATOM	4087	C	CYS	P	251	23.327	-6.568	-9.505	0.00	0.00	PROA
ATOM	4088	O	CYS	P	251	23.426	-6.462	-8.260	0.00	0.00	PROA
ATOM	4089	N	TRP	P	252	22.141	-6.935	-10.054	0.00	0.00	PROA
ATOM	4090	HN	TRP	P	252	22.051	-7.028	-11.043	0.00	0.00	PROA
ATOM	4091	CA	TRP	P	252	20.984	-7.176	-9.278	0.00	0.00	PROA
ATOM	4092	HA	TRP	P	252	21.116	-6.642	-8.349	0.00	0.00	PROA
ATOM	4093	CB	TRP	P	252	19.820	-6.462	-10.032	0.00	0.00	PROA
ATOM	4094	HB1	TRP	P	252	18.913	-6.699	-9.437	0.00	0.00	PROA
ATOM	4095	HB2	TRP	P	252	19.841	-7.010	-10.998	0.00	0.00	PROA
ATOM	4096	CG	TRP	P	252	19.942	-4.970	-10.170	0.00	0.00	PROA
ATOM	4097	CD1	TRP	P	252	20.682	-4.337	-11.147	0.00	0.00	PROA
ATOM	4098	HD1	TRP	P	252	21.236	-4.938	-11.853	0.00	0.00	PROA
ATOM	4099	NE1	TRP	P	252	20.504	-3.002	-11.082	0.00	0.00	PROA
ATOM	4100	HE1	TRP	P	252	20.812	-2.337	-11.727	0.00	0.00	PROA
ATOM	4101	CE2	TRP	P	252	19.655	-2.727	-10.110	0.00	0.00	PROA
ATOM	4102	CD2	TRP	P	252	19.241	-3.958	-9.425	0.00	0.00	PROA
ATOM	4103	CE3	TRP	P	252	18.364	-3.890	-8.322	0.00	0.00	PROA
ATOM	4104	HE3	TRP	P	252	17.914	-4.752	-7.854	0.00	0.00	PROA
ATOM	4105	CZ3	TRP	P	252	17.755	-2.694	-7.998	0.00	0.00	PROA
ATOM	4106	HZ3	TRP	P	252	16.954	-2.660	-7.274	0.00	0.00	PROA
ATOM	4107	CZ2	TRP	P	252	19.156	-1.536	-9.649	0.00	0.00	PROA
ATOM	4108	HZ2	TRP	P	252	19.706	-0.667	-9.979	0.00	0.00	PROA
ATOM	4109	CH2	TRP	P	252	18.137	-1.554	-8.639	0.00	0.00	PROA
ATOM	4110	HH2	TRP	P	252	17.777	-0.631	-8.209	0.00	0.00	PROA
ATOM	4111	C	TRP	P	252	20.711	-8.644	-8.889	0.00	0.00	PROA
ATOM	4112	O	TRP	P	252	20.128	-8.862	-7.842	0.00	0.00	PROA
ATOM	4113	N	LEU	P	253	21.213	-9.656	-9.628	0.00	0.00	PROA
ATOM	4114	HN	LEU	P	253	21.652	-9.498	-10.510	0.00	0.00	PROA
ATOM	4115	CA	LEU	P	253	21.014	-11.100	-9.255	0.00	0.00	PROA
ATOM	4116	HA	LEU	P	253	19.997	-11.119	-8.895	0.00	0.00	PROA
ATOM	4117	CB	LEU	P	253	21.250	-12.100	-10.374	0.00	0.00	PROA
ATOM	4118	HB1	LEU	P	253	22.339	-12.083	-10.593	0.00	0.00	PROA
ATOM	4119	HB2	LEU	P	253	20.728	-11.878	-11.329	0.00	0.00	PROA
ATOM	4120	CG	LEU	P	253	20.766	-13.577	-10.186	0.00	0.00	PROA
ATOM	4121	HG	LEU	P	253	20.878	-13.878	-9.122	0.00	0.00	PROA
ATOM	4122	CD1	LEU	P	253	19.359	-13.849	-10.638	0.00	0.00	PROA
ATOM	4123	HD11	LEU	P	253	19.085	-13.368	-11.601	0.00	0.00	PROA
ATOM	4124	HD12	LEU	P	253	18.603	-13.480	-9.912	0.00	0.00	PROA
ATOM	4125	HD13	LEU	P	253	19.064	-14.916	-10.727	0.00	0.00	PROA
ATOM	4126	CD2	LEU	P	253	21.736	-14.484	-10.976	0.00	0.00	PROA
ATOM	4127	HD21	LEU	P	253	21.626	-14.539	-12.080	0.00	0.00	PROA
ATOM	4128	HD22	LEU	P	253	21.633	-15.556	-10.702	0.00	0.00	PROA
ATOM	4129	HD23	LEU	P	253	22.773	-14.266	-10.641	0.00	0.00	PROA
ATOM	4130	C	LEU	P	253	21.655	-11.494	-7.932	0.00	0.00	PROA
ATOM	4131	O	LEU	P	253	20.959	-12.130	-7.151	0.00	0.00	PROA
ATOM	4132	N	PRO	P	254	22.887	-11.168	-7.553	0.00	0.00	PROA
ATOM	4133	CD	PRO	P	254	24.005	-10.912	-8.491	0.00	0.00	PROA
ATOM	4134	HD1	PRO	P	254	23.886	-10.153	-9.293	0.00	0.00	PROA
ATOM	4135	HD2	PRO	P	254	24.311	-11.854	-8.996	0.00	0.00	PROA
ATOM	4136	CA	PRO	P	254	23.400	-11.709	-6.280	0.00	0.00	PROA
ATOM	4137	HA	PRO	P	254	22.999	-12.695	-6.102	0.00	0.00	PROA
ATOM	4138	CB	PRO	P	254	24.943	-11.602	-6.410	0.00	0.00	PROA
ATOM	4139	HB1	PRO	P	254	25.278	-12.647	-6.586	0.00	0.00	PROA
ATOM	4140	HB2	PRO	P	254	25.471	-11.403	-5.453	0.00	0.00	PROA
ATOM	4141	CG	PRO	P	254	25.268	-10.727	-7.595	0.00	0.00	PROA
ATOM	4142	HG1	PRO	P	254	25.268	-9.663	-7.277	0.00	0.00	PROA
ATOM	4143	HG2	PRO	P	254	26.192	-10.993	-8.151	0.00	0.00	PROA
ATOM	4144	C	PRO	P	254	22.834	-10.909	-5.102	0.00	0.00	PROA

ATOM 4145 O PRO P 254 22.677 -11.529 -4.044 0.00 0.00 PROA
ATOM 4146 N TYR P 255 22.536 -9.660 -5.291 0.00 0.00 PROA
ATOM 4147 HN TYR P 255 22.787 -9.214 -6.147 0.00 0.00 PROA
ATOM 4148 CA TYR P 255 21.638 -8.981 -4.466 0.00 0.00 PROA
ATOM 4149 HA TYR P 255 22.058 -8.930 -3.472 0.00 0.00 PROA
ATOM 4150 CB TYR P 255 21.511 -7.519 -5.003 0.00 0.00 PROA
ATOM 4151 HB1 TYR P 255 21.291 -7.599 -6.089 0.00 0.00 PROA
ATOM 4152 HB2 TYR P 255 22.500 -7.044 -4.824 0.00 0.00 PROA
ATOM 4153 CG TYR P 255 20.503 -6.671 -4.256 0.00 0.00 PROA
ATOM 4154 CD1 TYR P 255 20.951 -6.112 -3.094 0.00 0.00 PROA
ATOM 4155 HD1 TYR P 255 21.962 -6.341 -2.791 0.00 0.00 PROA
ATOM 4156 CE1 TYR P 255 20.117 -5.232 -2.363 0.00 0.00 PROA
ATOM 4157 HE1 TYR P 255 20.410 -5.008 -1.348 0.00 0.00 PROA
ATOM 4158 CZ TYR P 255 18.877 -4.820 -2.905 0.00 0.00 PROA
ATOM 4159 OH TYR P 255 18.077 -3.820 -2.256 0.00 0.00 PROA
ATOM 4160 HH TYR P 255 18.601 -3.443 -1.546 0.00 0.00 PROA
ATOM 4161 CD2 TYR P 255 19.286 -6.093 -4.898 0.00 0.00 PROA
ATOM 4162 HD2 TYR P 255 19.046 -6.486 -5.874 0.00 0.00 PROA
ATOM 4163 CE2 TYR P 255 18.513 -5.222 -4.154 0.00 0.00 PROA
ATOM 4164 HE2 TYR P 255 17.563 -4.866 -4.522 0.00 0.00 PROA
ATOM 4165 C TYR P 255 20.274 -9.618 -4.262 0.00 0.00 PROA
ATOM 4166 O TYR P 255 19.900 -9.848 -3.131 0.00 0.00 PROA
ATOM 4167 N TYR P 256 19.537 -9.917 -5.312 0.00 0.00 PROA
ATOM 4168 HN TYR P 256 19.872 -9.692 -6.223 0.00 0.00 PROA
ATOM 4169 CA TYR P 256 18.307 -10.596 -5.357 0.00 0.00 PROA
ATOM 4170 HA TYR P 256 17.678 -10.040 -4.677 0.00 0.00 PROA
ATOM 4171 CB TYR P 256 17.568 -10.454 -6.741 0.00 0.00 PROA
ATOM 4172 HB1 TYR P 256 16.890 -11.323 -6.876 0.00 0.00 PROA
ATOM 4173 HB2 TYR P 256 18.335 -10.518 -7.543 0.00 0.00 PROA
ATOM 4174 CG TYR P 256 16.860 -9.141 -6.867 0.00 0.00 PROA
ATOM 4175 CD1 TYR P 256 16.237 -8.419 -5.825 0.00 0.00 PROA
ATOM 4176 HD1 TYR P 256 16.199 -8.883 -4.851 0.00 0.00 PROA
ATOM 4177 CE1 TYR P 256 15.500 -7.296 -6.040 0.00 0.00 PROA
ATOM 4178 HE1 TYR P 256 14.911 -6.750 -5.318 0.00 0.00 PROA
ATOM 4179 CZ TYR P 256 15.389 -6.794 -7.344 0.00 0.00 PROA
ATOM 4180 OH TYR P 256 14.592 -5.653 -7.575 0.00 0.00 PROA
ATOM 4181 HH TYR P 256 14.580 -5.471 -8.518 0.00 0.00 PROA
ATOM 4182 CD2 TYR P 256 16.768 -8.646 -8.130 0.00 0.00 PROA
ATOM 4183 HD2 TYR P 256 17.315 -9.143 -8.917 0.00 0.00 PROA
ATOM 4184 CE2 TYR P 256 16.059 -7.427 -8.434 0.00 0.00 PROA
ATOM 4185 HE2 TYR P 256 16.058 -7.011 -9.431 0.00 0.00 PROA
ATOM 4186 C TYR P 256 18.411 -12.002 -4.794 0.00 0.00 PROA
ATOM 4187 O TYR P 256 17.503 -12.335 -4.051 0.00 0.00 PROA
ATOM 4188 N ILE P 257 19.548 -12.745 -4.997 0.00 0.00 PROA
ATOM 4189 HN ILE P 257 20.198 -12.479 -5.704 0.00 0.00 PROA
ATOM 4190 CA ILE P 257 19.711 -13.927 -4.265 0.00 0.00 PROA
ATOM 4191 HA ILE P 257 18.882 -14.584 -4.481 0.00 0.00 PROA
ATOM 4192 CB ILE P 257 21.003 -14.732 -4.617 0.00 0.00 PROA
ATOM 4193 HB ILE P 257 21.816 -13.980 -4.525 0.00 0.00 PROA
ATOM 4194 CG2 ILE P 257 21.277 -15.997 -3.790 0.00 0.00 PROA
ATOM 4195 HG21 ILE P 257 21.427 -15.624 -2.754 0.00 0.00 PROA
ATOM 4196 HG22 ILE P 257 22.167 -16.526 -4.195 0.00 0.00 PROA
ATOM 4197 HG23 ILE P 257 20.447 -16.734 -3.823 0.00 0.00 PROA
ATOM 4198 CG1 ILE P 257 20.936 -15.208 -6.030 0.00 0.00 PROA
ATOM 4199 HG11 ILE P 257 20.742 -14.404 -6.772 0.00 0.00 PROA
ATOM 4200 HG12 ILE P 257 20.228 -16.064 -6.041 0.00 0.00 PROA
ATOM 4201 CD ILE P 257 22.347 -15.637 -6.512 0.00 0.00 PROA
ATOM 4202 HD1 ILE P 257 22.729 -16.610 -6.136 0.00 0.00 PROA
ATOM 4203 HD2 ILE P 257 23.075 -14.842 -6.242 0.00 0.00 PROA
ATOM 4204 HD3 ILE P 257 22.285 -15.766 -7.614 0.00 0.00 PROA
ATOM 4205 C ILE P 257 19.724 -13.670 -2.734 0.00 0.00 PROA

ATOM 4206 O ILE P 257 19.021 -14.324 -1.942 0.00 0.00 PROA
ATOM 4207 N GLY P 258 20.512 -12.655 -2.230 0.00 0.00 PROA
ATOM 4208 HN GLY P 258 21.150 -12.188 -2.837 0.00 0.00 PROA
ATOM 4209 CA GLY P 258 20.685 -12.226 -0.893 0.00 0.00 PROA
ATOM 4210 HA1 GLY P 258 21.359 -11.382 -0.887 0.00 0.00 PROA
ATOM 4211 HA2 GLY P 258 21.210 -12.973 -0.317 0.00 0.00 PROA
ATOM 4212 C GLY P 258 19.406 -11.860 -0.157 0.00 0.00 PROA
ATOM 4213 O GLY P 258 19.154 -12.151 0.995 0.00 0.00 PROA
ATOM 4214 N ILE P 259 18.700 -10.969 -0.850 0.00 0.00 PROA
ATOM 4215 HN ILE P 259 19.154 -10.529 -1.621 0.00 0.00 PROA
ATOM 4216 CA ILE P 259 17.345 -10.514 -0.509 0.00 0.00 PROA
ATOM 4217 HA ILE P 259 17.437 -10.095 0.482 0.00 0.00 PROA
ATOM 4218 CB ILE P 259 16.774 -9.446 -1.314 0.00 0.00 PROA
ATOM 4219 HB ILE P 259 16.945 -9.861 -2.331 0.00 0.00 PROA
ATOM 4220 CG2 ILE P 259 15.239 -9.292 -1.269 0.00 0.00 PROA
ATOM 4221 HG21 ILE P 259 14.862 -8.974 -0.274 0.00 0.00 PROA
ATOM 4222 HG22 ILE P 259 14.680 -10.168 -1.662 0.00 0.00 PROA
ATOM 4223 HG23 ILE P 259 14.875 -8.504 -1.963 0.00 0.00 PROA
ATOM 4224 CG1 ILE P 259 17.509 -8.100 -1.281 0.00 0.00 PROA
ATOM 4225 HG11 ILE P 259 17.021 -7.358 -1.950 0.00 0.00 PROA
ATOM 4226 HG12 ILE P 259 18.548 -8.176 -1.664 0.00 0.00 PROA
ATOM 4227 CD ILE P 259 17.494 -7.363 0.038 0.00 0.00 PROA
ATOM 4228 HD1 ILE P 259 18.381 -7.622 0.655 0.00 0.00 PROA
ATOM 4229 HD2 ILE P 259 16.590 -7.549 0.656 0.00 0.00 PROA
ATOM 4230 HD3 ILE P 259 17.607 -6.274 -0.146 0.00 0.00 PROA
ATOM 4231 C ILE P 259 16.354 -11.664 -0.287 0.00 0.00 PROA
ATOM 4232 O ILE P 259 15.520 -11.571 0.647 0.00 0.00 PROA
ATOM 4233 N SER P 260 16.436 -12.650 -1.233 0.00 0.00 PROA
ATOM 4234 HN SER P 260 17.029 -12.573 -2.031 0.00 0.00 PROA
ATOM 4235 CA SER P 260 15.532 -13.828 -1.072 0.00 0.00 PROA
ATOM 4236 HA SER P 260 14.542 -13.445 -0.871 0.00 0.00 PROA
ATOM 4237 CB SER P 260 15.453 -14.580 -2.452 0.00 0.00 PROA
ATOM 4238 HB1 SER P 260 15.504 -13.831 -3.270 0.00 0.00 PROA
ATOM 4239 HB2 SER P 260 14.449 -15.053 -2.513 0.00 0.00 PROA
ATOM 4240 OG SER P 260 16.489 -15.609 -2.591 0.00 0.00 PROA
ATOM 4241 HG1 SER P 260 17.268 -15.063 -2.461 0.00 0.00 PROA
ATOM 4242 C SER P 260 15.892 -14.615 0.198 0.00 0.00 PROA
ATOM 4243 O SER P 260 15.015 -14.980 0.882 0.00 0.00 PROA
ATOM 4244 N ILE P 261 17.198 -14.746 0.458 0.00 0.00 PROA
ATOM 4245 HN ILE P 261 17.877 -14.301 -0.121 0.00 0.00 PROA
ATOM 4246 CA ILE P 261 17.732 -15.391 1.722 0.00 0.00 PROA
ATOM 4247 HA ILE P 261 17.479 -16.439 1.773 0.00 0.00 PROA
ATOM 4248 CB ILE P 261 19.287 -15.511 1.850 0.00 0.00 PROA
ATOM 4249 HB ILE P 261 19.771 -14.516 1.744 0.00 0.00 PROA
ATOM 4250 CG2 ILE P 261 19.699 -15.977 3.262 0.00 0.00 PROA
ATOM 4251 HG21 ILE P 261 20.784 -16.216 3.268 0.00 0.00 PROA
ATOM 4252 HG22 ILE P 261 19.178 -16.931 3.489 0.00 0.00 PROA
ATOM 4253 HG23 ILE P 261 19.477 -15.388 4.178 0.00 0.00 PROA
ATOM 4254 CG1 ILE P 261 19.777 -16.518 0.702 0.00 0.00 PROA
ATOM 4255 HG11 ILE P 261 19.049 -16.405 -0.130 0.00 0.00 PROA
ATOM 4256 HG12 ILE P 261 19.741 -17.500 1.218 0.00 0.00 PROA
ATOM 4257 CD ILE P 261 21.234 -16.235 0.274 0.00 0.00 PROA
ATOM 4258 HD1 ILE P 261 21.382 -15.175 -0.023 0.00 0.00 PROA
ATOM 4259 HD2 ILE P 261 21.644 -16.900 -0.515 0.00 0.00 PROA
ATOM 4260 HD3 ILE P 261 21.894 -16.253 1.167 0.00 0.00 PROA
ATOM 4261 C ILE P 261 17.176 -14.672 2.907 0.00 0.00 PROA
ATOM 4262 O ILE P 261 16.667 -15.309 3.837 0.00 0.00 PROA
ATOM 4263 N ASP P 262 17.285 -13.312 2.933 0.00 0.00 PROA
ATOM 4264 HN ASP P 262 17.800 -12.770 2.273 0.00 0.00 PROA
ATOM 4265 CA ASP P 262 16.752 -12.414 3.956 0.00 0.00 PROA
ATOM 4266 HA ASP P 262 17.290 -12.652 4.863 0.00 0.00 PROA

ATOM 4267 CB ASP P 262 17.293 -10.964 3.642 0.00 0.00 PROA
ATOM 4268 HB1 ASP P 262 16.880 -10.624 2.668 0.00 0.00 PROA
ATOM 4269 HB2 ASP P 262 18.402 -10.900 3.613 0.00 0.00 PROA
ATOM 4270 CG ASP P 262 16.878 -9.871 4.582 0.00 0.00 PROA
ATOM 4271 OD1 ASP P 262 17.639 -9.592 5.539 0.00 0.00 PROA
ATOM 4272 OD2 ASP P 262 15.680 -9.418 4.455 0.00 0.00 PROA
ATOM 4273 C ASP P 262 15.219 -12.579 4.187 0.00 0.00 PROA
ATOM 4274 O ASP P 262 14.814 -12.962 5.309 0.00 0.00 PROA
ATOM 4275 N SER P 263 14.413 -12.568 3.147 0.00 0.00 PROA
ATOM 4276 HN SER P 263 14.828 -12.239 2.303 0.00 0.00 PROA
ATOM 4277 CA SER P 263 12.992 -12.893 3.133 0.00 0.00 PROA
ATOM 4278 HA SER P 263 12.608 -12.109 3.768 0.00 0.00 PROA
ATOM 4279 CB SER P 263 12.460 -12.978 1.649 0.00 0.00 PROA
ATOM 4280 HB1 SER P 263 11.374 -13.211 1.620 0.00 0.00 PROA
ATOM 4281 HB2 SER P 263 12.972 -13.855 1.197 0.00 0.00 PROA
ATOM 4282 OG SER P 263 12.712 -11.771 0.931 0.00 0.00 PROA
ATOM 4283 HG1 SER P 263 13.671 -11.762 0.877 0.00 0.00 PROA
ATOM 4284 C SER P 263 12.645 -14.238 3.789 0.00 0.00 PROA
ATOM 4285 O SER P 263 11.718 -14.333 4.641 0.00 0.00 PROA
ATOM 4286 N PHE P 264 13.346 -15.368 3.532 0.00 0.00 PROA
ATOM 4287 HN PHE P 264 13.903 -15.364 2.705 0.00 0.00 PROA
ATOM 4288 CA PHE P 264 13.180 -16.560 4.291 0.00 0.00 PROA
ATOM 4289 HA PHE P 264 12.117 -16.747 4.248 0.00 0.00 PROA
ATOM 4290 CB PHE P 264 13.922 -17.712 3.498 0.00 0.00 PROA
ATOM 4291 HB1 PHE P 264 13.929 -18.615 4.145 0.00 0.00 PROA
ATOM 4292 HB2 PHE P 264 14.979 -17.406 3.344 0.00 0.00 PROA
ATOM 4293 CG PHE P 264 13.323 -18.082 2.139 0.00 0.00 PROA
ATOM 4294 CD1 PHE P 264 14.029 -18.015 0.940 0.00 0.00 PROA
ATOM 4295 HD1 PHE P 264 14.979 -17.511 1.038 0.00 0.00 PROA
ATOM 4296 CE1 PHE P 264 13.475 -18.336 -0.293 0.00 0.00 PROA
ATOM 4297 HE1 PHE P 264 14.048 -18.098 -1.177 0.00 0.00 PROA
ATOM 4298 CZ PHE P 264 12.199 -18.955 -0.346 0.00 0.00 PROA
ATOM 4299 HZ PHE P 264 11.691 -19.289 -1.238 0.00 0.00 PROA
ATOM 4300 CD2 PHE P 264 12.090 -18.742 2.078 0.00 0.00 PROA
ATOM 4301 HD2 PHE P 264 11.566 -18.833 3.018 0.00 0.00 PROA
ATOM 4302 CE2 PHE P 264 11.541 -19.173 0.852 0.00 0.00 PROA
ATOM 4303 HE2 PHE P 264 10.806 -19.964 0.852 0.00 0.00 PROA
ATOM 4304 C PHE P 264 13.512 -16.562 5.744 0.00 0.00 PROA
ATOM 4305 O PHE P 264 12.834 -17.178 6.564 0.00 0.00 PROA
ATOM 4306 N ILE P 265 14.652 -15.916 6.101 0.00 0.00 PROA
ATOM 4307 HN ILE P 265 15.137 -15.340 5.447 0.00 0.00 PROA
ATOM 4308 CA ILE P 265 15.084 -15.651 7.572 0.00 0.00 PROA
ATOM 4309 HA ILE P 265 15.188 -16.603 8.073 0.00 0.00 PROA
ATOM 4310 CB ILE P 265 16.376 -14.839 7.847 0.00 0.00 PROA
ATOM 4311 HB ILE P 265 16.296 -13.862 7.325 0.00 0.00 PROA
ATOM 4312 CG2 ILE P 265 16.601 -14.558 9.307 0.00 0.00 PROA
ATOM 4313 HG21 ILE P 265 17.556 -14.009 9.454 0.00 0.00 PROA
ATOM 4314 HG22 ILE P 265 16.616 -15.488 9.915 0.00 0.00 PROA
ATOM 4315 HG23 ILE P 265 15.763 -13.996 9.771 0.00 0.00 PROA
ATOM 4316 CG1 ILE P 265 17.622 -15.490 7.259 0.00 0.00 PROA
ATOM 4317 HG11 ILE P 265 17.549 -15.999 6.274 0.00 0.00 PROA
ATOM 4318 HG12 ILE P 265 18.111 -16.250 7.906 0.00 0.00 PROA
ATOM 4319 CD ILE P 265 18.700 -14.491 6.970 0.00 0.00 PROA
ATOM 4320 HD1 ILE P 265 19.190 -14.155 7.909 0.00 0.00 PROA
ATOM 4321 HD2 ILE P 265 18.260 -13.605 6.464 0.00 0.00 PROA
ATOM 4322 HD3 ILE P 265 19.536 -14.847 6.331 0.00 0.00 PROA
ATOM 4323 C ILE P 265 13.928 -15.122 8.415 0.00 0.00 PROA
ATOM 4324 O ILE P 265 13.544 -15.582 9.514 0.00 0.00 PROA
ATOM 4325 N LEU P 266 13.247 -14.085 7.833 0.00 0.00 PROA
ATOM 4326 HN LEU P 266 13.522 -13.592 7.012 0.00 0.00 PROA
ATOM 4327 CA LEU P 266 12.172 -13.374 8.448 0.00 0.00 PROA

```
ATOM   4328  HA  LEU P 266      12.708 -13.085   9.340  0.00  0.00      PROA
ATOM   4329  CB  LEU P 266      11.857 -12.237   7.478  0.00  0.00      PROA
ATOM   4330  HB1 LEU P 266      11.317 -12.623   6.588  0.00  0.00      PROA
ATOM   4331  HB2 LEU P 266      12.800 -11.733   7.176  0.00  0.00      PROA
ATOM   4332  CG  LEU P 266      11.017 -11.023   8.010  0.00  0.00      PROA
ATOM   4333  HG  LEU P 266      10.073 -11.434   8.429  0.00  0.00      PROA
ATOM   4334  CD1 LEU P 266      11.723 -10.426   9.265  0.00  0.00      PROA
ATOM   4335 HD11 LEU P 266      12.643  -9.920   8.901  0.00  0.00      PROA
ATOM   4336 HD12 LEU P 266      11.993 -11.222   9.992  0.00  0.00      PROA
ATOM   4337 HD13 LEU P 266      11.139  -9.745   9.921  0.00  0.00      PROA
ATOM   4338  CD2 LEU P 266      10.759  -9.927   6.995  0.00  0.00      PROA
ATOM   4339 HD21 LEU P 266      10.343  -8.987   7.416  0.00  0.00      PROA
ATOM   4340 HD22 LEU P 266      10.010 -10.288   6.259  0.00  0.00      PROA
ATOM   4341 HD23 LEU P 266      11.668  -9.496   6.522  0.00  0.00      PROA
ATOM   4342  C   LEU P 266      10.927 -14.192   8.777  0.00  0.00      PROA
ATOM   4343  O   LEU P 266      10.141 -13.932   9.702  0.00  0.00      PROA
ATOM   4344  N   LEU P 267      10.654 -15.130   7.908  0.00  0.00      PROA
ATOM   4345  HN  LEU P 267      11.385 -15.258   7.241  0.00  0.00      PROA
ATOM   4346  CA  LEU P 267       9.614 -16.123   7.955  0.00  0.00      PROA
ATOM   4347  HA  LEU P 267       8.674 -15.710   8.289  0.00  0.00      PROA
ATOM   4348  CB  LEU P 267       9.414 -16.534   6.452  0.00  0.00      PROA
ATOM   4349  HB1 LEU P 267      10.149 -17.336   6.229  0.00  0.00      PROA
ATOM   4350  HB2 LEU P 267       9.613 -15.684   5.763  0.00  0.00      PROA
ATOM   4351  CG  LEU P 267       8.055 -17.223   6.128  0.00  0.00      PROA
ATOM   4352  HG  LEU P 267       7.923 -18.148   6.729  0.00  0.00      PROA
ATOM   4353  CD1 LEU P 267       6.899 -16.223   6.224  0.00  0.00      PROA
ATOM   4354 HD11 LEU P 267       6.689 -15.803   7.231  0.00  0.00      PROA
ATOM   4355 HD12 LEU P 267       5.923 -16.717   6.027  0.00  0.00      PROA
ATOM   4356 HD13 LEU P 267       7.137 -15.309   5.640  0.00  0.00      PROA
ATOM   4357  CD2 LEU P 267       8.140 -17.649   4.654  0.00  0.00      PROA
ATOM   4358 HD21 LEU P 267       8.385 -16.837   3.935  0.00  0.00      PROA
ATOM   4359 HD22 LEU P 267       7.197 -18.177   4.400  0.00  0.00      PROA
ATOM   4360 HD23 LEU P 267       8.895 -18.446   4.482  0.00  0.00      PROA
ATOM   4361  C   LEU P 267       9.939 -17.399   8.761  0.00  0.00      PROA
ATOM   4362  O   LEU P 267       9.128 -18.272   8.928  0.00  0.00      PROA
ATOM   4363  N   GLU P 268      11.133 -17.372   9.260  0.00  0.00      PROA
ATOM   4364  HN  GLU P 268      11.686 -16.543   9.221  0.00  0.00      PROA
ATOM   4365  CA  GLU P 268      11.784 -18.417   9.992  0.00  0.00      PROA
ATOM   4366  HA  GLU P 268      12.759 -17.995  10.186  0.00  0.00      PROA
ATOM   4367  CB  GLU P 268      11.263 -18.486  11.453  0.00  0.00      PROA
ATOM   4368  HB1 GLU P 268      11.786 -19.300  11.998  0.00  0.00      PROA
ATOM   4369  HB2 GLU P 268      10.199 -18.804  11.446  0.00  0.00      PROA
ATOM   4370  CG  GLU P 268      11.418 -17.195  12.351  0.00  0.00      PROA
ATOM   4371  HG1 GLU P 268      10.755 -16.350  12.068  0.00  0.00      PROA
ATOM   4372  HG2 GLU P 268      12.363 -16.668  12.099  0.00  0.00      PROA
ATOM   4373  CD  GLU P 268      11.372 -17.530  13.845  0.00  0.00      PROA
ATOM   4374  OE1 GLU P 268      10.326 -18.075  14.314  0.00  0.00      PROA
ATOM   4375  OE2 GLU P 268      12.299 -17.246  14.608  0.00  0.00      PROA
ATOM   4376  C   GLU P 268      11.986 -19.759   9.309  0.00  0.00      PROA
ATOM   4377  O   GLU P 268      11.993 -20.842   9.892  0.00  0.00      PROA
ATOM   4378  N   ILE P 269      12.213 -19.687   7.971  0.00  0.00      PROA
ATOM   4379  HN  ILE P 269      12.189 -18.850   7.429  0.00  0.00      PROA
ATOM   4380  CA  ILE P 269      12.445 -20.945   7.160  0.00  0.00      PROA
ATOM   4381  HA  ILE P 269      12.004 -21.780   7.683  0.00  0.00      PROA
ATOM   4382  CB  ILE P 269      11.801 -20.910   5.753  0.00  0.00      PROA
ATOM   4383  HB  ILE P 269      11.764 -19.818   5.549  0.00  0.00      PROA
ATOM   4384  CG2 ILE P 269      12.664 -21.600   4.657  0.00  0.00      PROA
ATOM   4385 HG21 ILE P 269      13.688 -21.169   4.639  0.00  0.00      PROA
ATOM   4386 HG22 ILE P 269      12.228 -21.383   3.658  0.00  0.00      PROA
ATOM   4387 HG23 ILE P 269      12.662 -22.702   4.799  0.00  0.00      PROA
ATOM   4388  CG1 ILE P 269      10.427 -21.613   5.699  0.00  0.00      PROA
```

ATOM	4389	HG11	ILE	P	269	10.435	-22.717	5.829	0.00	0.00	PROA
ATOM	4390	HG12	ILE	P	269	10.171	-21.462	4.628	0.00	0.00	PROA
ATOM	4391	CD	ILE	P	269	9.478	-20.791	6.613	0.00	0.00	PROA
ATOM	4392	HD1	ILE	P	269	9.701	-19.703	6.646	0.00	0.00	PROA
ATOM	4393	HD2	ILE	P	269	9.660	-21.169	7.642	0.00	0.00	PROA
ATOM	4394	HD3	ILE	P	269	8.424	-21.063	6.393	0.00	0.00	PROA
ATOM	4395	C	ILE	P	269	13.927	-21.262	7.209	0.00	0.00	PROA
ATOM	4396	O	ILE	P	269	14.359	-22.449	7.130	0.00	0.00	PROA
ATOM	4397	N	ILE	P	270	14.795	-20.231	7.423	0.00	0.00	PROA
ATOM	4398	HN	ILE	P	270	14.444	-19.303	7.524	0.00	0.00	PROA
ATOM	4399	CA	ILE	P	270	16.189	-20.437	7.689	0.00	0.00	PROA
ATOM	4400	HA	ILE	P	270	16.426	-21.490	7.729	0.00	0.00	PROA
ATOM	4401	CB	ILE	P	270	17.011	-19.771	6.682	0.00	0.00	PROA
ATOM	4402	HB	ILE	P	270	16.661	-18.771	6.348	0.00	0.00	PROA
ATOM	4403	CG2	ILE	P	270	18.511	-19.831	7.173	0.00	0.00	PROA
ATOM	4404	HG21	ILE	P	270	19.126	-19.570	6.286	0.00	0.00	PROA
ATOM	4405	HG22	ILE	P	270	18.792	-20.807	7.624	0.00	0.00	PROA
ATOM	4406	HG23	ILE	P	270	18.784	-19.135	7.994	0.00	0.00	PROA
ATOM	4407	CG1	ILE	P	270	16.857	-20.579	5.349	0.00	0.00	PROA
ATOM	4408	HG11	ILE	P	270	15.774	-20.688	5.128	0.00	0.00	PROA
ATOM	4409	HG12	ILE	P	270	17.253	-21.603	5.522	0.00	0.00	PROA
ATOM	4410	CD	ILE	P	270	17.466	-19.863	4.132	0.00	0.00	PROA
ATOM	4411	HD1	ILE	P	270	18.564	-19.817	4.297	0.00	0.00	PROA
ATOM	4412	HD2	ILE	P	270	17.133	-18.814	3.979	0.00	0.00	PROA
ATOM	4413	HD3	ILE	P	270	17.309	-20.442	3.197	0.00	0.00	PROA
ATOM	4414	C	ILE	P	270	16.501	-19.903	9.112	0.00	0.00	PROA
ATOM	4415	O	ILE	P	270	16.203	-18.731	9.464	0.00	0.00	PROA
ATOM	4416	N	LYS	P	271	17.021	-20.748	10.056	0.00	0.00	PROA
ATOM	4417	HN	LYS	P	271	17.188	-21.698	9.802	0.00	0.00	PROA
ATOM	4418	CA	LYS	P	271	17.324	-20.351	11.408	0.00	0.00	PROA
ATOM	4419	HA	LYS	P	271	17.325	-19.271	11.390	0.00	0.00	PROA
ATOM	4420	CB	LYS	P	271	16.301	-20.860	12.455	0.00	0.00	PROA
ATOM	4421	HB1	LYS	P	271	16.694	-20.727	13.485	0.00	0.00	PROA
ATOM	4422	HB2	LYS	P	271	16.289	-21.957	12.276	0.00	0.00	PROA
ATOM	4423	CG	LYS	P	271	14.885	-20.386	12.303	0.00	0.00	PROA
ATOM	4424	HG1	LYS	P	271	14.077	-21.001	12.753	0.00	0.00	PROA
ATOM	4425	HG2	LYS	P	271	14.624	-20.662	11.258	0.00	0.00	PROA
ATOM	4426	CD	LYS	P	271	14.766	-18.929	12.739	0.00	0.00	PROA
ATOM	4427	HD1	LYS	P	271	13.695	-18.665	12.611	0.00	0.00	PROA
ATOM	4428	HD2	LYS	P	271	15.217	-18.340	11.911	0.00	0.00	PROA
ATOM	4429	CE	LYS	P	271	15.295	-18.484	14.061	0.00	0.00	PROA
ATOM	4430	HE1	LYS	P	271	16.385	-18.590	14.250	0.00	0.00	PROA
ATOM	4431	HE2	LYS	P	271	14.818	-19.026	14.906	0.00	0.00	PROA
ATOM	4432	NZ	LYS	P	271	14.891	-17.111	14.349	0.00	0.00	PROA
ATOM	4433	HZ1	LYS	P	271	15.060	-16.539	13.497	0.00	0.00	PROA
ATOM	4434	HZ2	LYS	P	271	15.498	-16.780	15.125	0.00	0.00	PROA
ATOM	4435	HZ3	LYS	P	271	13.892	-17.075	14.636	0.00	0.00	PROA
ATOM	4436	C	LYS	P	271	18.725	-20.933	11.807	0.00	0.00	PROA
ATOM	4437	O	LYS	P	271	18.775	-22.028	12.360	0.00	0.00	PROA
ATOM	4438	N	GLN	P	272	19.795	-20.332	11.299	0.00	0.00	PROA
ATOM	4439	HN	GLN	P	272	19.588	-19.420	10.953	0.00	0.00	PROA
ATOM	4440	CA	GLN	P	272	21.189	-20.797	11.431	0.00	0.00	PROA
ATOM	4441	HA	GLN	P	272	21.156	-21.870	11.546	0.00	0.00	PROA
ATOM	4442	CB	GLN	P	272	21.915	-20.486	10.073	0.00	0.00	PROA
ATOM	4443	HB1	GLN	P	272	23.013	-20.623	10.161	0.00	0.00	PROA
ATOM	4444	HB2	GLN	P	272	21.615	-19.439	9.851	0.00	0.00	PROA
ATOM	4445	CG	GLN	P	272	21.349	-21.411	8.952	0.00	0.00	PROA
ATOM	4446	HG1	GLN	P	272	21.598	-20.956	7.969	0.00	0.00	PROA
ATOM	4447	HG2	GLN	P	272	20.242	-21.433	9.049	0.00	0.00	PROA
ATOM	4448	CD	GLN	P	272	21.842	-22.795	9.143	0.00	0.00	PROA
ATOM	4449	OE1	GLN	P	272	21.256	-23.701	9.703	0.00	0.00	PROA

```
ATOM   4450  NE2 GLN P 272      23.103 -23.052   8.686  0.00  0.00      PROA
ATOM   4451 HE21 GLN P 272      23.682 -22.238   8.632  0.00  0.00      PROA
ATOM   4452 HE22 GLN P 272      23.575 -23.895   8.943  0.00  0.00      PROA
ATOM   4453  C   GLN P 272      21.996 -20.272  12.683  0.00  0.00      PROA
ATOM   4454  O   GLN P 272      23.134 -20.622  12.909  0.00  0.00      PROA
ATOM   4455  N   GLY P 273      21.393 -19.350  13.518  0.00  0.00      PROA
ATOM   4456  HN  GLY P 273      20.445 -19.146  13.285  0.00  0.00      PROA
ATOM   4457  CA  GLY P 273      22.083 -18.606  14.626  0.00  0.00      PROA
ATOM   4458  HA1 GLY P 273      22.807 -19.207  15.157  0.00  0.00      PROA
ATOM   4459  HA2 GLY P 273      21.291 -18.407  15.332  0.00  0.00      PROA
ATOM   4460  C   GLY P 273      22.802 -17.411  14.067  0.00  0.00      PROA
ATOM   4461  O   GLY P 273      22.523 -16.980  12.969  0.00  0.00      PROA
ATOM   4462  N   CYS P 274      23.650 -16.769  14.917  0.00  0.00      PROA
ATOM   4463  HN  CYS P 274      23.895 -17.185  15.789  0.00  0.00      PROA
ATOM   4464  CA  CYS P 274      24.192 -15.443  14.865  0.00  0.00      PROA
ATOM   4465  HA  CYS P 274      23.324 -14.829  14.674  0.00  0.00      PROA
ATOM   4466  CB  CYS P 274      24.929 -15.097  16.182  0.00  0.00      PROA
ATOM   4467  HB1 CYS P 274      25.846 -15.715  16.295  0.00  0.00      PROA
ATOM   4468  HB2 CYS P 274      24.214 -15.227  17.022  0.00  0.00      PROA
ATOM   4469  SG  CYS P 274      25.640 -13.432  16.346  0.00  0.00      PROA
ATOM   4470  C   CYS P 274      25.057 -15.201  13.639  0.00  0.00      PROA
ATOM   4471  O   CYS P 274      25.010 -14.177  13.024  0.00  0.00      PROA
ATOM   4472  N   GLU P 275      25.920 -16.114  13.238  0.00  0.00      PROA
ATOM   4473  HN  GLU P 275      26.087 -16.996  13.672  0.00  0.00      PROA
ATOM   4474  CA  GLU P 275      26.941 -15.899  12.193  0.00  0.00      PROA
ATOM   4475  HA  GLU P 275      27.322 -14.911  12.407  0.00  0.00      PROA
ATOM   4476  CB  GLU P 275      28.028 -16.961  12.227  0.00  0.00      PROA
ATOM   4477  HB1 GLU P 275      28.607 -16.538  11.378  0.00  0.00      PROA
ATOM   4478  HB2 GLU P 275      27.573 -17.896  11.836  0.00  0.00      PROA
ATOM   4479  CG  GLU P 275      28.937 -17.012  13.421  0.00  0.00      PROA
ATOM   4480  HG1 GLU P 275      28.427 -16.434  14.221  0.00  0.00      PROA
ATOM   4481  HG2 GLU P 275      29.905 -16.575  13.094  0.00  0.00      PROA
ATOM   4482  CD  GLU P 275      29.160 -18.443  13.904  0.00  0.00      PROA
ATOM   4483  OE1 GLU P 275      29.822 -18.549  14.983  0.00  0.00      PROA
ATOM   4484  OE2 GLU P 275      28.647 -19.391  13.278  0.00  0.00      PROA
ATOM   4485  C   GLU P 275      26.381 -15.674  10.829  0.00  0.00      PROA
ATOM   4486  O   GLU P 275      26.758 -14.791  10.076  0.00  0.00      PROA
ATOM   4487  N   PHE P 276      25.390 -16.603  10.462  0.00  0.00      PROA
ATOM   4488  HN  PHE P 276      25.185 -17.460  10.929  0.00  0.00      PROA
ATOM   4489  CA  PHE P 276      24.670 -16.535   9.209  0.00  0.00      PROA
ATOM   4490  HA  PHE P 276      25.468 -16.555   8.481  0.00  0.00      PROA
ATOM   4491  CB  PHE P 276      23.804 -17.772   9.046  0.00  0.00      PROA
ATOM   4492  HB1 PHE P 276      23.055 -17.689   9.862  0.00  0.00      PROA
ATOM   4493  HB2 PHE P 276      24.378 -18.711   9.201  0.00  0.00      PROA
ATOM   4494  CG  PHE P 276      23.016 -17.831   7.764  0.00  0.00      PROA
ATOM   4495  CD1 PHE P 276      23.623 -18.441   6.618  0.00  0.00      PROA
ATOM   4496  HD1 PHE P 276      24.656 -18.709   6.787  0.00  0.00      PROA
ATOM   4497  CE1 PHE P 276      22.866 -18.797   5.485  0.00  0.00      PROA
ATOM   4498  HE1 PHE P 276      23.354 -19.278   4.651  0.00  0.00      PROA
ATOM   4499  CZ  PHE P 276      21.448 -18.398   5.419  0.00  0.00      PROA
ATOM   4500  HZ  PHE P 276      20.775 -18.719   4.638  0.00  0.00      PROA
ATOM   4501  CD2 PHE P 276      21.671 -17.517   7.734  0.00  0.00      PROA
ATOM   4502  HD2 PHE P 276      21.336 -16.948   8.588  0.00  0.00      PROA
ATOM   4503  CE2 PHE P 276      20.900 -17.796   6.623  0.00  0.00      PROA
ATOM   4504  HE2 PHE P 276      19.845 -17.565   6.589  0.00  0.00      PROA
ATOM   4505  C   PHE P 276      24.027 -15.206   9.045  0.00  0.00      PROA
ATOM   4506  O   PHE P 276      24.264 -14.531   8.023  0.00  0.00      PROA
ATOM   4507  N   GLU P 277      23.182 -14.691   9.986  0.00  0.00      PROA
ATOM   4508  HN  GLU P 277      22.946 -15.225  10.794  0.00  0.00      PROA
ATOM   4509  CA  GLU P 277      22.541 -13.392   9.827  0.00  0.00      PROA
ATOM   4510  HA  GLU P 277      22.079 -13.496   8.857  0.00  0.00      PROA
```

ATOM	4511	CB	GLU	P	277	21.584	-13.083	10.993	0.00	0.00	PROA
ATOM	4512	HB1	GLU	P	277	21.190	-12.050	10.878	0.00	0.00	PROA
ATOM	4513	HB2	GLU	P	277	22.172	-13.072	11.936	0.00	0.00	PROA
ATOM	4514	CG	GLU	P	277	20.315	-13.916	11.139	0.00	0.00	PROA
ATOM	4515	HG1	GLU	P	277	20.649	-14.904	11.523	0.00	0.00	PROA
ATOM	4516	HG2	GLU	P	277	19.947	-14.152	10.118	0.00	0.00	PROA
ATOM	4517	CD	GLU	P	277	19.152	-13.311	11.902	0.00	0.00	PROA
ATOM	4518	OE1	GLU	P	277	18.987	-12.072	11.808	0.00	0.00	PROA
ATOM	4519	OE2	GLU	P	277	18.516	-14.042	12.752	0.00	0.00	PROA
ATOM	4520	C	GLU	P	277	23.594	-12.266	9.610	0.00	0.00	PROA
ATOM	4521	O	GLU	P	277	23.395	-11.477	8.671	0.00	0.00	PROA
ATOM	4522	N	ASN	P	278	24.693	-12.269	10.328	0.00	0.00	PROA
ATOM	4523	HN	ASN	P	278	24.910	-12.920	11.051	0.00	0.00	PROA
ATOM	4524	CA	ASN	P	278	25.739	-11.219	10.125	0.00	0.00	PROA
ATOM	4525	HA	ASN	P	278	25.264	-10.250	10.153	0.00	0.00	PROA
ATOM	4526	CB	ASN	P	278	26.852	-11.369	11.215	0.00	0.00	PROA
ATOM	4527	HB1	ASN	P	278	27.522	-10.484	11.175	0.00	0.00	PROA
ATOM	4528	HB2	ASN	P	278	27.383	-12.334	11.069	0.00	0.00	PROA
ATOM	4529	CG	ASN	P	278	26.309	-11.231	12.670	0.00	0.00	PROA
ATOM	4530	OD1	ASN	P	278	25.109	-11.033	12.832	0.00	0.00	PROA
ATOM	4531	ND2	ASN	P	278	27.202	-11.222	13.673	0.00	0.00	PROA
ATOM	4532	HD21	ASN	P	278	26.915	-10.926	14.585	0.00	0.00	PROA
ATOM	4533	HD22	ASN	P	278	28.188	-11.253	13.508	0.00	0.00	PROA
ATOM	4534	C	ASN	P	278	26.468	-11.391	8.824	0.00	0.00	PROA
ATOM	4535	O	ASN	P	278	26.926	-10.426	8.191	0.00	0.00	PROA
ATOM	4536	N	THR	P	279	26.694	-12.639	8.422	0.00	0.00	PROA
ATOM	4537	HN	THR	P	279	26.288	-13.392	8.934	0.00	0.00	PROA
ATOM	4538	CA	THR	P	279	27.136	-12.882	7.062	0.00	0.00	PROA
ATOM	4539	HA	THR	P	279	27.966	-12.200	6.950	0.00	0.00	PROA
ATOM	4540	CB	THR	P	279	27.626	-14.286	6.840	0.00	0.00	PROA
ATOM	4541	HB	THR	P	279	26.807	-15.021	6.993	0.00	0.00	PROA
ATOM	4542	OG1	THR	P	279	28.505	-14.800	7.868	0.00	0.00	PROA
ATOM	4543	HG1	THR	P	279	28.081	-14.978	8.711	0.00	0.00	PROA
ATOM	4544	CG2	THR	P	279	28.233	-14.608	5.421	0.00	0.00	PROA
ATOM	4545	HG21	THR	P	279	28.610	-15.643	5.277	0.00	0.00	PROA
ATOM	4546	HG22	THR	P	279	29.086	-13.965	5.117	0.00	0.00	PROA
ATOM	4547	HG23	THR	P	279	27.396	-14.457	4.706	0.00	0.00	PROA
ATOM	4548	C	THR	P	279	26.139	-12.373	6.055	0.00	0.00	PROA
ATOM	4549	O	THR	P	279	26.535	-11.637	5.133	0.00	0.00	PROA
ATOM	4550	N	VAL	P	280	24.842	-12.709	6.137	0.00	0.00	PROA
ATOM	4551	HN	VAL	P	280	24.385	-13.259	6.831	0.00	0.00	PROA
ATOM	4552	CA	VAL	P	280	23.775	-12.266	5.244	0.00	0.00	PROA
ATOM	4553	HA	VAL	P	280	24.031	-12.588	4.246	0.00	0.00	PROA
ATOM	4554	CB	VAL	P	280	22.416	-12.935	5.548	0.00	0.00	PROA
ATOM	4555	HB	VAL	P	280	22.205	-12.720	6.617	0.00	0.00	PROA
ATOM	4556	CG1	VAL	P	280	21.273	-12.329	4.711	0.00	0.00	PROA
ATOM	4557	HG11	VAL	P	280	20.350	-12.946	4.726	0.00	0.00	PROA
ATOM	4558	HG12	VAL	P	280	21.555	-12.279	3.638	0.00	0.00	PROA
ATOM	4559	HG13	VAL	P	280	21.029	-11.284	4.998	0.00	0.00	PROA
ATOM	4560	CG2	VAL	P	280	22.326	-14.470	5.217	0.00	0.00	PROA
ATOM	4561	HG21	VAL	P	280	23.119	-14.978	5.806	0.00	0.00	PROA
ATOM	4562	HG22	VAL	P	280	22.279	-14.803	4.158	0.00	0.00	PROA
ATOM	4563	HG23	VAL	P	280	21.440	-14.937	5.698	0.00	0.00	PROA
ATOM	4564	C	VAL	P	280	23.666	-10.778	5.156	0.00	0.00	PROA
ATOM	4565	O	VAL	P	280	23.600	-10.241	4.048	0.00	0.00	PROA
ATOM	4566	N	HSD	P	281	23.728	-10.152	6.326	0.00	0.00	PROA
ATOM	4567	HN	HSD	P	281	23.961	-10.591	7.191	0.00	0.00	PROA
ATOM	4568	CA	HSD	P	281	23.718	-8.629	6.357	0.00	0.00	PROA
ATOM	4569	HA	HSD	P	281	22.779	-8.316	5.925	0.00	0.00	PROA
ATOM	4570	CB	HSD	P	281	23.800	-8.266	7.859	0.00	0.00	PROA
ATOM	4571	HB1	HSD	P	281	24.709	-8.706	8.323	0.00	0.00	PROA

ATOM	4572	HB2	HSD	P	281	22.849	-8.579	8.340	0.00	0.00	PROA
ATOM	4573	ND1	HSD	P	281	24.806	-5.856	7.894	0.00	0.00	PROA
ATOM	4574	HD1	HSD	P	281	25.616	-5.975	7.320	0.00	0.00	PROA
ATOM	4575	CG	HSD	P	281	23.767	-6.813	8.172	0.00	0.00	PROA
ATOM	4576	CE1	HSD	P	281	24.328	-4.666	8.351	0.00	0.00	PROA
ATOM	4577	HE1	HSD	P	281	24.931	-3.759	8.387	0.00	0.00	PROA
ATOM	4578	NE2	HSD	P	281	23.177	-4.805	8.935	0.00	0.00	PROA
ATOM	4579	CD2	HSD	P	281	22.858	-6.136	8.872	0.00	0.00	PROA
ATOM	4580	HD2	HSD	P	281	22.094	-6.597	9.486	0.00	0.00	PROA
ATOM	4581	C	HSD	P	281	24.841	-8.053	5.549	0.00	0.00	PROA
ATOM	4582	O	HSD	P	281	24.647	-7.133	4.718	0.00	0.00	PROA
ATOM	4583	N	LYS	P	282	26.083	-8.560	5.759	0.00	0.00	PROA
ATOM	4584	HN	LYS	P	282	26.164	-9.331	6.387	0.00	0.00	PROA
ATOM	4585	CA	LYS	P	282	27.302	-8.218	4.983	0.00	0.00	PROA
ATOM	4586	HA	LYS	P	282	27.531	-7.169	5.097	0.00	0.00	PROA
ATOM	4587	CB	LYS	P	282	28.516	-9.067	5.539	0.00	0.00	PROA
ATOM	4588	HB1	LYS	P	282	28.317	-10.160	5.508	0.00	0.00	PROA
ATOM	4589	HB2	LYS	P	282	28.637	-8.843	6.620	0.00	0.00	PROA
ATOM	4590	CG	LYS	P	282	29.973	-8.921	4.886	0.00	0.00	PROA
ATOM	4591	HG1	LYS	P	282	29.841	-9.124	3.802	0.00	0.00	PROA
ATOM	4592	HG2	LYS	P	282	30.754	-9.651	5.189	0.00	0.00	PROA
ATOM	4593	CD	LYS	P	282	30.503	-7.502	4.954	0.00	0.00	PROA
ATOM	4594	HD1	LYS	P	282	29.870	-6.697	4.524	0.00	0.00	PROA
ATOM	4595	HD2	LYS	P	282	31.452	-7.521	4.377	0.00	0.00	PROA
ATOM	4596	CE	LYS	P	282	30.795	-6.875	6.399	0.00	0.00	PROA
ATOM	4597	HE1	LYS	P	282	29.833	-6.761	6.943	0.00	0.00	PROA
ATOM	4598	HE2	LYS	P	282	31.234	-5.865	6.260	0.00	0.00	PROA
ATOM	4599	NZ	LYS	P	282	31.672	-7.745	7.236	0.00	0.00	PROA
ATOM	4600	HZ1	LYS	P	282	32.411	-8.139	6.620	0.00	0.00	PROA
ATOM	4601	HZ2	LYS	P	282	31.230	-8.484	7.818	0.00	0.00	PROA
ATOM	4602	HZ3	LYS	P	282	32.180	-7.029	7.793	0.00	0.00	PROA
ATOM	4603	C	LYS	P	282	27.173	-8.512	3.524	0.00	0.00	PROA
ATOM	4604	O	LYS	P	282	27.673	-7.844	2.637	0.00	0.00	PROA
ATOM	4605	N	TRP	P	283	26.550	-9.672	3.127	0.00	0.00	PROA
ATOM	4606	HN	TRP	P	283	26.113	-10.318	3.748	0.00	0.00	PROA
ATOM	4607	CA	TRP	P	283	26.324	-9.921	1.698	0.00	0.00	PROA
ATOM	4608	HA	TRP	P	283	27.201	-9.935	1.069	0.00	0.00	PROA
ATOM	4609	CB	TRP	P	283	25.730	-11.375	1.627	0.00	0.00	PROA
ATOM	4610	HB1	TRP	P	283	24.935	-11.500	2.393	0.00	0.00	PROA
ATOM	4611	HB2	TRP	P	283	26.512	-12.067	2.010	0.00	0.00	PROA
ATOM	4612	CG	TRP	P	283	25.342	-11.790	0.263	0.00	0.00	PROA
ATOM	4613	CD1	TRP	P	283	24.035	-11.960	-0.164	0.00	0.00	PROA
ATOM	4614	HD1	TRP	P	283	23.180	-11.610	0.395	0.00	0.00	PROA
ATOM	4615	NE1	TRP	P	283	24.126	-12.118	-1.529	0.00	0.00	PROA
ATOM	4616	HE1	TRP	P	283	23.489	-11.718	-2.152	0.00	0.00	PROA
ATOM	4617	CE2	TRP	P	283	25.335	-12.457	-1.925	0.00	0.00	PROA
ATOM	4618	CD2	TRP	P	283	26.166	-12.233	-0.827	0.00	0.00	PROA
ATOM	4619	CE3	TRP	P	283	27.561	-12.414	-1.002	0.00	0.00	PROA
ATOM	4620	HE3	TRP	P	283	28.176	-12.280	-0.123	0.00	0.00	PROA
ATOM	4621	CZ3	TRP	P	283	28.043	-12.950	-2.206	0.00	0.00	PROA
ATOM	4622	HZ3	TRP	P	283	29.098	-13.172	-2.266	0.00	0.00	PROA
ATOM	4623	CZ2	TRP	P	283	25.802	-12.930	-3.147	0.00	0.00	PROA
ATOM	4624	HZ2	TRP	P	283	25.075	-13.181	-3.905	0.00	0.00	PROA
ATOM	4625	CH2	TRP	P	283	27.163	-13.211	-3.236	0.00	0.00	PROA
ATOM	4626	HH2	TRP	P	283	27.577	-13.679	-4.117	0.00	0.00	PROA
ATOM	4627	C	TRP	P	283	25.445	-8.914	1.010	0.00	0.00	PROA
ATOM	4628	O	TRP	P	283	25.728	-8.547	-0.104	0.00	0.00	PROA
ATOM	4629	N	ILE	P	284	24.259	-8.522	1.630	0.00	0.00	PROA
ATOM	4630	HN	ILE	P	284	24.047	-9.009	2.474	0.00	0.00	PROA
ATOM	4631	CA	ILE	P	284	23.385	-7.478	1.112	0.00	0.00	PROA
ATOM	4632	HA	ILE	P	284	23.176	-7.689	0.074	0.00	0.00	PROA

ATOM	4633	CB	ILE	P	284	22.115	-7.409	1.969	0.00	0.00	PROA
ATOM	4634	HB	ILE	P	284	22.400	-7.370	3.042	0.00	0.00	PROA
ATOM	4635	CG2	ILE	P	284	21.083	-6.271	1.549	0.00	0.00	PROA
ATOM	4636	HG21	ILE	P	284	20.010	-6.470	1.757	0.00	0.00	PROA
ATOM	4637	HG22	ILE	P	284	21.227	-6.128	0.457	0.00	0.00	PROA
ATOM	4638	HG23	ILE	P	284	21.237	-5.341	2.137	0.00	0.00	PROA
ATOM	4639	CG1	ILE	P	284	21.291	-8.688	1.716	0.00	0.00	PROA
ATOM	4640	HG11	ILE	P	284	21.856	-9.644	1.677	0.00	0.00	PROA
ATOM	4641	HG12	ILE	P	284	20.752	-8.707	0.745	0.00	0.00	PROA
ATOM	4642	CD	ILE	P	284	20.279	-8.873	2.857	0.00	0.00	PROA
ATOM	4643	HD1	ILE	P	284	20.649	-8.533	3.848	0.00	0.00	PROA
ATOM	4644	HD2	ILE	P	284	20.040	-9.944	3.032	0.00	0.00	PROA
ATOM	4645	HD3	ILE	P	284	19.322	-8.355	2.635	0.00	0.00	PROA
ATOM	4646	C	ILE	P	284	24.050	-6.095	1.100	0.00	0.00	PROA
ATOM	4647	O	ILE	P	284	23.824	-5.355	0.186	0.00	0.00	PROA
ATOM	4648	N	SER	P	285	24.957	-5.768	1.998	0.00	0.00	PROA
ATOM	4649	HN	SER	P	285	25.197	-6.335	2.782	0.00	0.00	PROA
ATOM	4650	CA	SER	P	285	25.616	-4.446	2.006	0.00	0.00	PROA
ATOM	4651	HA	SER	P	285	24.844	-3.692	1.959	0.00	0.00	PROA
ATOM	4652	CB	SER	P	285	26.392	-4.106	3.339	0.00	0.00	PROA
ATOM	4653	HB1	SER	P	285	26.867	-3.102	3.366	0.00	0.00	PROA
ATOM	4654	HB2	SER	P	285	27.150	-4.906	3.473	0.00	0.00	PROA
ATOM	4655	OG	SER	P	285	25.505	-4.293	4.430	0.00	0.00	PROA
ATOM	4656	HG1	SER	P	285	24.653	-3.866	4.313	0.00	0.00	PROA
ATOM	4657	C	SER	P	285	26.583	-4.303	0.783	0.00	0.00	PROA
ATOM	4658	O	SER	P	285	26.643	-3.329	0.065	0.00	0.00	PROA
ATOM	4659	N	ILE	P	286	27.425	-5.286	0.584	0.00	0.00	PROA
ATOM	4660	HN	ILE	P	286	27.457	-6.098	1.163	0.00	0.00	PROA
ATOM	4661	CA	ILE	P	286	28.294	-5.325	-0.565	0.00	0.00	PROA
ATOM	4662	HA	ILE	P	286	28.716	-4.331	-0.587	0.00	0.00	PROA
ATOM	4663	CB	ILE	P	286	29.296	-6.491	-0.418	0.00	0.00	PROA
ATOM	4664	HB	ILE	P	286	29.011	-7.361	0.211	0.00	0.00	PROA
ATOM	4665	CG2	ILE	P	286	29.567	-7.025	-1.875	0.00	0.00	PROA
ATOM	4666	HG21	ILE	P	286	28.677	-7.558	-2.271	0.00	0.00	PROA
ATOM	4667	HG22	ILE	P	286	30.403	-7.750	-1.971	0.00	0.00	PROA
ATOM	4668	HG23	ILE	P	286	29.830	-6.155	-2.514	0.00	0.00	PROA
ATOM	4669	CG1	ILE	P	286	30.668	-6.027	0.154	0.00	0.00	PROA
ATOM	4670	HG11	ILE	P	286	31.049	-5.223	-0.511	0.00	0.00	PROA
ATOM	4671	HG12	ILE	P	286	31.302	-6.926	-0.003	0.00	0.00	PROA
ATOM	4672	CD	ILE	P	286	30.609	-5.548	1.636	0.00	0.00	PROA
ATOM	4673	HD1	ILE	P	286	30.205	-4.525	1.791	0.00	0.00	PROA
ATOM	4674	HD2	ILE	P	286	31.655	-5.457	1.999	0.00	0.00	PROA
ATOM	4675	HD3	ILE	P	286	30.091	-6.247	2.328	0.00	0.00	PROA
ATOM	4676	C	ILE	P	286	27.426	-5.384	-1.848	0.00	0.00	PROA
ATOM	4677	O	ILE	P	286	27.657	-4.690	-2.812	0.00	0.00	PROA
ATOM	4678	N	THR	P	287	26.377	-6.307	-1.844	0.00	0.00	PROA
ATOM	4679	HN	THR	P	287	26.268	-6.961	-1.099	0.00	0.00	PROA
ATOM	4680	CA	THR	P	287	25.592	-6.496	-3.040	0.00	0.00	PROA
ATOM	4681	HA	THR	P	287	26.229	-6.467	-3.911	0.00	0.00	PROA
ATOM	4682	CB	THR	P	287	24.934	-7.886	-3.184	0.00	0.00	PROA
ATOM	4683	HB	THR	P	287	24.399	-7.871	-4.157	0.00	0.00	PROA
ATOM	4684	OG1	THR	P	287	24.043	-8.284	-2.086	0.00	0.00	PROA
ATOM	4685	HG1	THR	P	287	24.668	-8.412	-1.369	0.00	0.00	PROA
ATOM	4686	CG2	THR	P	287	25.995	-9.020	-3.338	0.00	0.00	PROA
ATOM	4687	HG21	THR	P	287	26.741	-8.807	-4.133	0.00	0.00	PROA
ATOM	4688	HG22	THR	P	287	25.527	-10.021	-3.458	0.00	0.00	PROA
ATOM	4689	HG23	THR	P	287	26.621	-9.144	-2.428	0.00	0.00	PROA
ATOM	4690	C	THR	P	287	24.706	-5.275	-3.399	0.00	0.00	PROA
ATOM	4691	O	THR	P	287	24.430	-4.951	-4.574	0.00	0.00	PROA
ATOM	4692	N	GLU	P	288	24.159	-4.554	-2.352	0.00	0.00	PROA
ATOM	4693	HN	GLU	P	288	24.113	-4.934	-1.431	0.00	0.00	PROA

ATOM	4694	CA	GLU	P	288	23.415	-3.308	-2.673	0.00	0.00	PROA
ATOM	4695	HA	GLU	P	288	22.712	-3.355	-3.492	0.00	0.00	PROA
ATOM	4696	CB	GLU	P	288	22.566	-2.876	-1.424	0.00	0.00	PROA
ATOM	4697	HB1	GLU	P	288	23.246	-2.465	-0.648	0.00	0.00	PROA
ATOM	4698	HB2	GLU	P	288	22.220	-3.836	-0.986	0.00	0.00	PROA
ATOM	4699	CG	GLU	P	288	21.452	-1.807	-1.675	0.00	0.00	PROA
ATOM	4700	HG1	GLU	P	288	20.850	-2.208	-2.518	0.00	0.00	PROA
ATOM	4701	HG2	GLU	P	288	21.890	-0.828	-1.965	0.00	0.00	PROA
ATOM	4702	CD	GLU	P	288	20.650	-1.684	-0.467	0.00	0.00	PROA
ATOM	4703	OE1	GLU	P	288	21.152	-0.977	0.479	0.00	0.00	PROA
ATOM	4704	OE2	GLU	P	288	19.539	-2.260	-0.426	0.00	0.00	PROA
ATOM	4705	C	GLU	P	288	24.333	-2.182	-3.151	0.00	0.00	PROA
ATOM	4706	O	GLU	P	288	23.908	-1.391	-4.008	0.00	0.00	PROA
ATOM	4707	N	ALA	P	289	25.603	-2.128	-2.716	0.00	0.00	PROA
ATOM	4708	HN	ALA	P	289	25.838	-2.719	-1.948	0.00	0.00	PROA
ATOM	4709	CA	ALA	P	289	26.624	-1.259	-3.189	0.00	0.00	PROA
ATOM	4710	HA	ALA	P	289	26.262	-0.254	-3.032	0.00	0.00	PROA
ATOM	4711	CB	ALA	P	289	27.949	-1.402	-2.389	0.00	0.00	PROA
ATOM	4712	HB1	ALA	P	289	27.817	-1.247	-1.297	0.00	0.00	PROA
ATOM	4713	HB2	ALA	P	289	28.647	-0.652	-2.820	0.00	0.00	PROA
ATOM	4714	HB3	ALA	P	289	28.431	-2.400	-2.463	0.00	0.00	PROA
ATOM	4715	C	ALA	P	289	26.998	-1.413	-4.614	0.00	0.00	PROA
ATOM	4716	O	ALA	P	289	27.174	-0.449	-5.362	0.00	0.00	PROA
ATOM	4717	N	LEU	P	290	27.042	-2.702	-5.037	0.00	0.00	PROA
ATOM	4718	HN	LEU	P	290	26.677	-3.401	-4.427	0.00	0.00	PROA
ATOM	4719	CA	LEU	P	290	27.104	-3.099	-6.455	0.00	0.00	PROA
ATOM	4720	HA	LEU	P	290	28.018	-2.629	-6.787	0.00	0.00	PROA
ATOM	4721	CB	LEU	P	290	27.229	-4.614	-6.444	0.00	0.00	PROA
ATOM	4722	HB1	LEU	P	290	26.350	-5.067	-5.938	0.00	0.00	PROA
ATOM	4723	HB2	LEU	P	290	28.044	-4.847	-5.725	0.00	0.00	PROA
ATOM	4724	CG	LEU	P	290	27.525	-5.371	-7.709	0.00	0.00	PROA
ATOM	4725	HG	LEU	P	290	26.862	-4.942	-8.490	0.00	0.00	PROA
ATOM	4726	CD1	LEU	P	290	28.940	-5.071	-8.192	0.00	0.00	PROA
ATOM	4727	HD11	LEU	P	290	29.713	-5.355	-7.447	0.00	0.00	PROA
ATOM	4728	HD12	LEU	P	290	29.165	-4.043	-8.549	0.00	0.00	PROA
ATOM	4729	HD13	LEU	P	290	29.263	-5.686	-9.060	0.00	0.00	PROA
ATOM	4730	CD2	LEU	P	290	27.376	-6.889	-7.479	0.00	0.00	PROA
ATOM	4731	HD21	LEU	P	290	27.517	-7.533	-8.373	0.00	0.00	PROA
ATOM	4732	HD22	LEU	P	290	26.376	-7.127	-7.057	0.00	0.00	PROA
ATOM	4733	HD23	LEU	P	290	28.123	-7.180	-6.710	0.00	0.00	PROA
ATOM	4734	C	LEU	P	290	25.945	-2.594	-7.358	0.00	0.00	PROA
ATOM	4735	O	LEU	P	290	26.142	-1.960	-8.428	0.00	0.00	PROA
ATOM	4736	N	ALA	P	291	24.656	-2.830	-6.967	0.00	0.00	PROA
ATOM	4737	HN	ALA	P	291	24.468	-3.367	-6.148	0.00	0.00	PROA
ATOM	4738	CA	ALA	P	291	23.454	-2.319	-7.507	0.00	0.00	PROA
ATOM	4739	HA	ALA	P	291	23.416	-2.674	-8.526	0.00	0.00	PROA
ATOM	4740	CB	ALA	P	291	22.254	-2.951	-6.774	0.00	0.00	PROA
ATOM	4741	HB1	ALA	P	291	22.413	-4.050	-6.823	0.00	0.00	PROA
ATOM	4742	HB2	ALA	P	291	21.327	-2.719	-7.341	0.00	0.00	PROA
ATOM	4743	HB3	ALA	P	291	22.124	-2.821	-5.679	0.00	0.00	PROA
ATOM	4744	C	ALA	P	291	23.320	-0.832	-7.604	0.00	0.00	PROA
ATOM	4745	O	ALA	P	291	22.707	-0.318	-8.563	0.00	0.00	PROA
ATOM	4746	N	PHE	P	292	23.878	0.036	-6.648	0.00	0.00	PROA
ATOM	4747	HN	PHE	P	292	24.351	-0.286	-5.832	0.00	0.00	PROA
ATOM	4748	CA	PHE	P	292	23.763	1.494	-6.760	0.00	0.00	PROA
ATOM	4749	HA	PHE	P	292	22.837	1.916	-7.121	0.00	0.00	PROA
ATOM	4750	CB	PHE	P	292	24.014	2.114	-5.343	0.00	0.00	PROA
ATOM	4751	HB1	PHE	P	292	24.247	3.185	-5.521	0.00	0.00	PROA
ATOM	4752	HB2	PHE	P	292	24.881	1.628	-4.847	0.00	0.00	PROA
ATOM	4753	CG	PHE	P	292	22.773	2.041	-4.534	0.00	0.00	PROA
ATOM	4754	CD1	PHE	P	292	21.768	1.093	-4.533	0.00	0.00	PROA

ATOM	4755	HD1	PHE	P	292	21.778	0.275	-5.238	0.00	0.00	PROA
ATOM	4756	CE1	PHE	P	292	20.716	1.139	-3.564	0.00	0.00	PROA
ATOM	4757	HE1	PHE	P	292	20.008	0.326	-3.501	0.00	0.00	PROA
ATOM	4758	CZ	PHE	P	292	20.768	2.049	-2.516	0.00	0.00	PROA
ATOM	4759	HZ	PHE	P	292	20.057	2.028	-1.703	0.00	0.00	PROA
ATOM	4760	CD2	PHE	P	292	22.757	2.931	-3.452	0.00	0.00	PROA
ATOM	4761	HD2	PHE	P	292	23.554	3.644	-3.302	0.00	0.00	PROA
ATOM	4762	CE2	PHE	P	292	21.832	2.957	-2.413	0.00	0.00	PROA
ATOM	4763	HE2	PHE	P	292	21.839	3.673	-1.604	0.00	0.00	PROA
ATOM	4764	C	PHE	P	292	24.909	1.984	-7.687	0.00	0.00	PROA
ATOM	4765	O	PHE	P	292	24.950	3.120	-8.156	0.00	0.00	PROA
ATOM	4766	N	PHE	P	293	25.813	1.005	-8.140	0.00	0.00	PROA
ATOM	4767	HN	PHE	P	293	25.831	0.075	-7.784	0.00	0.00	PROA
ATOM	4768	CA	PHE	P	293	26.843	1.432	-9.175	0.00	0.00	PROA
ATOM	4769	HA	PHE	P	293	27.095	2.482	-9.160	0.00	0.00	PROA
ATOM	4770	CB	PHE	P	293	28.151	0.582	-8.931	0.00	0.00	PROA
ATOM	4771	HB1	PHE	P	293	27.864	-0.470	-9.144	0.00	0.00	PROA
ATOM	4772	HB2	PHE	P	293	28.328	0.514	-7.836	0.00	0.00	PROA
ATOM	4773	CG	PHE	P	293	29.311	1.106	-9.666	0.00	0.00	PROA
ATOM	4774	CD1	PHE	P	293	30.066	0.217	-10.484	0.00	0.00	PROA
ATOM	4775	HD1	PHE	P	293	29.892	-0.834	-10.659	0.00	0.00	PROA
ATOM	4776	CE1	PHE	P	293	31.151	0.701	-11.230	0.00	0.00	PROA
ATOM	4777	HE1	PHE	P	293	31.563	0.008	-11.949	0.00	0.00	PROA
ATOM	4778	CZ	PHE	P	293	31.655	1.994	-11.056	0.00	0.00	PROA
ATOM	4779	HZ	PHE	P	293	32.455	2.425	-11.639	0.00	0.00	PROA
ATOM	4780	CD2	PHE	P	293	29.792	2.417	-9.535	0.00	0.00	PROA
ATOM	4781	HD2	PHE	P	293	29.328	3.164	-8.908	0.00	0.00	PROA
ATOM	4782	CE2	PHE	P	293	30.963	2.848	-10.162	0.00	0.00	PROA
ATOM	4783	HE2	PHE	P	293	31.254	3.887	-10.115	0.00	0.00	PROA
ATOM	4784	C	PHE	P	293	26.226	1.190	-10.540	0.00	0.00	PROA
ATOM	4785	O	PHE	P	293	26.809	1.612	-11.528	0.00	0.00	PROA
ATOM	4786	N	HSD	P	294	25.072	0.570	-10.636	0.00	0.00	PROA
ATOM	4787	HN	HSD	P	294	24.722	0.181	-9.788	0.00	0.00	PROA
ATOM	4788	CA	HSD	P	294	24.382	0.399	-11.864	0.00	0.00	PROA
ATOM	4789	HA	HSD	P	294	24.981	-0.202	-12.533	0.00	0.00	PROA
ATOM	4790	CB	HSD	P	294	23.188	-0.461	-11.615	0.00	0.00	PROA
ATOM	4791	HB1	HSD	P	294	22.475	0.167	-11.039	0.00	0.00	PROA
ATOM	4792	HB2	HSD	P	294	23.517	-1.216	-10.869	0.00	0.00	PROA
ATOM	4793	ND1	HSD	P	294	21.360	-0.504	-13.259	0.00	0.00	PROA
ATOM	4794	HD1	HSD	P	294	21.061	0.392	-12.933	0.00	0.00	PROA
ATOM	4795	CG	HSD	P	294	22.519	-1.052	-12.796	0.00	0.00	PROA
ATOM	4796	CE1	HSD	P	294	20.845	-1.325	-14.199	0.00	0.00	PROA
ATOM	4797	HE1	HSD	P	294	20.106	-1.040	-14.947	0.00	0.00	PROA
ATOM	4798	NE2	HSD	P	294	21.680	-2.381	-14.403	0.00	0.00	PROA
ATOM	4799	CD2	HSD	P	294	22.686	-2.217	-13.504	0.00	0.00	PROA
ATOM	4800	HD2	HSD	P	294	23.516	-2.867	-13.254	0.00	0.00	PROA
ATOM	4801	C	HSD	P	294	24.075	1.699	-12.702	0.00	0.00	PROA
ATOM	4802	O	HSD	P	294	24.462	1.735	-13.875	0.00	0.00	PROA
ATOM	4803	N	CYS	P	295	23.511	2.754	-12.115	0.00	0.00	PROA
ATOM	4804	HN	CYS	P	295	23.283	2.558	-11.164	0.00	0.00	PROA
ATOM	4805	CA	CYS	P	295	23.274	4.129	-12.600	0.00	0.00	PROA
ATOM	4806	HA	CYS	P	295	22.739	4.105	-13.538	0.00	0.00	PROA
ATOM	4807	CB	CYS	P	295	22.444	4.993	-11.594	0.00	0.00	PROA
ATOM	4808	HB1	CYS	P	295	21.484	4.439	-11.506	0.00	0.00	PROA
ATOM	4809	HB2	CYS	P	295	22.197	5.921	-12.154	0.00	0.00	PROA
ATOM	4810	SG	CYS	P	295	23.201	5.122	-9.985	0.00	0.00	PROA
ATOM	4811	HG1	CYS	P	295	22.924	3.909	-9.529	0.00	0.00	PROA
ATOM	4812	C	CYS	P	295	24.659	4.826	-12.885	0.00	0.00	PROA
ATOM	4813	O	CYS	P	295	24.687	5.860	-13.533	0.00	0.00	PROA
ATOM	4814	N	CYS	P	296	25.883	4.246	-12.504	0.00	0.00	PROA
ATOM	4815	HN	CYS	P	296	25.939	3.405	-11.971	0.00	0.00	PROA

ATOM 4816 CA CYS P 296 27.148 4.783 -13.009 0.00 0.00 PROA
ATOM 4817 HA CYS P 296 26.921 5.722 -13.492 0.00 0.00 PROA
ATOM 4818 CB CYS P 296 28.109 4.960 -11.795 0.00 0.00 PROA
ATOM 4819 HB1 CYS P 296 29.077 5.344 -12.183 0.00 0.00 PROA
ATOM 4820 HB2 CYS P 296 28.315 3.976 -11.322 0.00 0.00 PROA
ATOM 4821 SG CYS P 296 27.563 6.186 -10.620 0.00 0.00 PROA
ATOM 4822 HG1 CYS P 296 26.432 5.497 -10.646 0.00 0.00 PROA
ATOM 4823 C CYS P 296 27.811 3.919 -14.120 0.00 0.00 PROA
ATOM 4824 O CYS P 296 28.471 4.436 -15.029 0.00 0.00 PROA
ATOM 4825 N LEU P 297 27.779 2.562 -13.901 0.00 0.00 PROA
ATOM 4826 HN LEU P 297 27.287 2.146 -13.141 0.00 0.00 PROA
ATOM 4827 CA LEU P 297 28.387 1.560 -14.789 0.00 0.00 PROA
ATOM 4828 HA LEU P 297 29.456 1.709 -14.820 0.00 0.00 PROA
ATOM 4829 CB LEU P 297 28.092 0.146 -14.230 0.00 0.00 PROA
ATOM 4830 HB1 LEU P 297 26.996 -0.034 -14.201 0.00 0.00 PROA
ATOM 4831 HB2 LEU P 297 28.365 0.180 -13.154 0.00 0.00 PROA
ATOM 4832 CG LEU P 297 28.708 -1.072 -14.947 0.00 0.00 PROA
ATOM 4833 HG LEU P 297 28.527 -0.913 -16.032 0.00 0.00 PROA
ATOM 4834 CD1 LEU P 297 30.192 -1.179 -14.755 0.00 0.00 PROA
ATOM 4835 HD11 LEU P 297 30.687 -0.335 -15.282 0.00 0.00 PROA
ATOM 4836 HD12 LEU P 297 30.676 -2.138 -15.037 0.00 0.00 PROA
ATOM 4837 HD13 LEU P 297 30.538 -0.986 -13.717 0.00 0.00 PROA
ATOM 4838 CD2 LEU P 297 28.051 -2.382 -14.416 0.00 0.00 PROA
ATOM 4839 HD21 LEU P 297 27.133 -2.505 -15.030 0.00 0.00 PROA
ATOM 4840 HD22 LEU P 297 27.796 -2.393 -13.335 0.00 0.00 PROA
ATOM 4841 HD23 LEU P 297 28.715 -3.259 -14.576 0.00 0.00 PROA
ATOM 4842 C LEU P 297 27.767 1.703 -16.238 0.00 0.00 PROA
ATOM 4843 O LEU P 297 28.430 1.696 -17.263 0.00 0.00 PROA
ATOM 4844 N ASN P 298 26.414 1.824 -16.351 0.00 0.00 PROA
ATOM 4845 HN ASN P 298 25.937 1.629 -15.497 0.00 0.00 PROA
ATOM 4846 CA ASN P 298 25.718 1.928 -17.706 0.00 0.00 PROA
ATOM 4847 HA ASN P 298 26.125 1.149 -18.334 0.00 0.00 PROA
ATOM 4848 CB ASN P 298 24.208 1.670 -17.505 0.00 0.00 PROA
ATOM 4849 HB1 ASN P 298 23.695 1.750 -18.487 0.00 0.00 PROA
ATOM 4850 HB2 ASN P 298 23.782 2.502 -16.904 0.00 0.00 PROA
ATOM 4851 CG ASN P 298 23.894 0.233 -17.039 0.00 0.00 PROA
ATOM 4852 OD1 ASN P 298 24.730 -0.610 -17.292 0.00 0.00 PROA
ATOM 4853 ND2 ASN P 298 22.765 0.029 -16.419 0.00 0.00 PROA
ATOM 4854 HD21 ASN P 298 22.232 0.858 -16.250 0.00 0.00 PROA
ATOM 4855 HD22 ASN P 298 22.449 -0.920 -16.407 0.00 0.00 PROA
ATOM 4856 C ASN P 298 26.096 3.152 -18.508 0.00 0.00 PROA
ATOM 4857 O ASN P 298 26.219 2.955 -19.713 0.00 0.00 PROA
ATOM 4858 N PRO P 299 26.255 4.356 -17.983 0.00 0.00 PROA
ATOM 4859 CD PRO P 299 25.535 4.852 -16.869 0.00 0.00 PROA
ATOM 4860 HD1 PRO P 299 26.033 4.461 -15.956 0.00 0.00 PROA
ATOM 4861 HD2 PRO P 299 24.449 4.621 -16.837 0.00 0.00 PROA
ATOM 4862 CA PRO P 299 27.001 5.432 -18.706 0.00 0.00 PROA
ATOM 4863 HA PRO P 299 26.465 5.624 -19.623 0.00 0.00 PROA
ATOM 4864 CB PRO P 299 27.046 6.554 -17.656 0.00 0.00 PROA
ATOM 4865 HB1 PRO P 299 27.202 7.579 -18.057 0.00 0.00 PROA
ATOM 4866 HB2 PRO P 299 27.866 6.396 -16.925 0.00 0.00 PROA
ATOM 4867 CG PRO P 299 25.693 6.363 -16.913 0.00 0.00 PROA
ATOM 4868 HG1 PRO P 299 25.631 6.842 -15.913 0.00 0.00 PROA
ATOM 4869 HG2 PRO P 299 24.836 6.791 -17.475 0.00 0.00 PROA
ATOM 4870 C PRO P 299 28.370 5.052 -19.332 0.00 0.00 PROA
ATOM 4871 O PRO P 299 28.614 5.277 -20.503 0.00 0.00 PROA
ATOM 4872 N ILE P 300 29.242 4.511 -18.488 0.00 0.00 PROA
ATOM 4873 HN ILE P 300 29.044 4.260 -17.544 0.00 0.00 PROA
ATOM 4874 CA ILE P 300 30.611 4.121 -18.927 0.00 0.00 PROA
ATOM 4875 HA ILE P 300 31.061 5.000 -19.364 0.00 0.00 PROA
ATOM 4876 CB ILE P 300 31.507 3.712 -17.758 0.00 0.00 PROA

ATOM	4877	HB	ILE	P	300	31.028	2.803	-17.336	0.00	0.00	PROA
ATOM	4878	CG2	ILE	P	300	32.919	3.308	-18.250	0.00	0.00	PROA
ATOM	4879	HG21	ILE	P	300	33.421	2.564	-17.595	0.00	0.00	PROA
ATOM	4880	HG22	ILE	P	300	33.516	4.244	-18.229	0.00	0.00	PROA
ATOM	4881	HG23	ILE	P	300	33.018	2.907	-19.281	0.00	0.00	PROA
ATOM	4882	CG1	ILE	P	300	31.529	4.853	-16.715	0.00	0.00	PROA
ATOM	4883	HG11	ILE	P	300	30.537	5.248	-16.407	0.00	0.00	PROA
ATOM	4884	HG12	ILE	P	300	32.075	5.715	-17.155	0.00	0.00	PROA
ATOM	4885	CD	ILE	P	300	32.270	4.408	-15.410	0.00	0.00	PROA
ATOM	4886	HD1	ILE	P	300	31.628	3.675	-14.876	0.00	0.00	PROA
ATOM	4887	HD2	ILE	P	300	32.458	5.177	-14.630	0.00	0.00	PROA
ATOM	4888	HD3	ILE	P	300	33.198	3.802	-15.488	0.00	0.00	PROA
ATOM	4889	C	ILE	P	300	30.556	3.007	-19.958	0.00	0.00	PROA
ATOM	4890	O	ILE	P	300	31.356	3.043	-20.916	0.00	0.00	PROA
ATOM	4891	N	LEU	P	301	29.664	1.983	-19.896	0.00	0.00	PROA
ATOM	4892	HN	LEU	P	301	29.173	1.852	-19.039	0.00	0.00	PROA
ATOM	4893	CA	LEU	P	301	29.453	0.879	-20.802	0.00	0.00	PROA
ATOM	4894	HA	LEU	P	301	30.466	0.597	-21.048	0.00	0.00	PROA
ATOM	4895	CB	LEU	P	301	28.576	-0.328	-20.218	0.00	0.00	PROA
ATOM	4896	HB1	LEU	P	301	28.234	-1.052	-20.989	0.00	0.00	PROA
ATOM	4897	HB2	LEU	P	301	27.681	0.122	-19.738	0.00	0.00	PROA
ATOM	4898	CG	LEU	P	301	29.369	-1.288	-19.210	0.00	0.00	PROA
ATOM	4899	HG	LEU	P	301	29.516	-0.565	-18.379	0.00	0.00	PROA
ATOM	4900	CD1	LEU	P	301	28.578	-2.514	-18.772	0.00	0.00	PROA
ATOM	4901	HD11	LEU	P	301	28.348	-3.218	-19.601	0.00	0.00	PROA
ATOM	4902	HD12	LEU	P	301	27.621	-2.221	-18.290	0.00	0.00	PROA
ATOM	4903	HD13	LEU	P	301	29.073	-3.118	-17.981	0.00	0.00	PROA
ATOM	4904	CD2	LEU	P	301	30.699	-1.671	-19.784	0.00	0.00	PROA
ATOM	4905	HD21	LEU	P	301	31.410	-0.821	-19.854	0.00	0.00	PROA
ATOM	4906	HD22	LEU	P	301	30.627	-2.051	-20.826	0.00	0.00	PROA
ATOM	4907	HD23	LEU	P	301	31.159	-2.490	-19.191	0.00	0.00	PROA
ATOM	4908	C	LEU	P	301	28.923	1.369	-22.145	0.00	0.00	PROA
ATOM	4909	O	LEU	P	301	29.330	0.951	-23.220	0.00	0.00	PROA
ATOM	4910	N	TYR	P	302	27.955	2.351	-22.128	0.00	0.00	PROA
ATOM	4911	HN	TYR	P	302	27.508	2.588	-21.268	0.00	0.00	PROA
ATOM	4912	CA	TYR	P	302	27.404	3.052	-23.254	0.00	0.00	PROA
ATOM	4913	HA	TYR	P	302	27.007	2.273	-23.889	0.00	0.00	PROA
ATOM	4914	CB	TYR	P	302	26.226	3.929	-22.822	0.00	0.00	PROA
ATOM	4915	HB1	TYR	P	302	26.630	4.544	-21.990	0.00	0.00	PROA
ATOM	4916	HB2	TYR	P	302	25.528	3.252	-22.284	0.00	0.00	PROA
ATOM	4917	CG	TYR	P	302	25.487	4.720	-23.897	0.00	0.00	PROA
ATOM	4918	CD1	TYR	P	302	25.566	6.113	-23.914	0.00	0.00	PROA
ATOM	4919	HD1	TYR	P	302	26.080	6.509	-23.051	0.00	0.00	PROA
ATOM	4920	CE1	TYR	P	302	25.101	6.804	-25.011	0.00	0.00	PROA
ATOM	4921	HE1	TYR	P	302	25.278	7.862	-25.134	0.00	0.00	PROA
ATOM	4922	CZ	TYR	P	302	24.568	6.136	-26.127	0.00	0.00	PROA
ATOM	4923	OH	TYR	P	302	24.346	6.905	-27.284	0.00	0.00	PROA
ATOM	4924	HH	TYR	P	302	23.999	6.290	-27.934	0.00	0.00	PROA
ATOM	4925	CD2	TYR	P	302	24.868	4.056	-25.024	0.00	0.00	PROA
ATOM	4926	HD2	TYR	P	302	24.840	2.979	-24.958	0.00	0.00	PROA
ATOM	4927	CE2	TYR	P	302	24.476	4.735	-26.188	0.00	0.00	PROA
ATOM	4928	HE2	TYR	P	302	24.041	4.211	-27.026	0.00	0.00	PROA
ATOM	4929	C	TYR	P	302	28.455	3.814	-24.077	0.00	0.00	PROA
ATOM	4930	O	TYR	P	302	28.400	3.878	-25.282	0.00	0.00	PROA
ATOM	4931	N	ALA	P	303	29.353	4.501	-23.331	0.00	0.00	PROA
ATOM	4932	HN	ALA	P	303	29.337	4.412	-22.338	0.00	0.00	PROA
ATOM	4933	CA	ALA	P	303	30.353	5.347	-23.892	0.00	0.00	PROA
ATOM	4934	HA	ALA	P	303	29.811	5.925	-24.625	0.00	0.00	PROA
ATOM	4935	CB	ALA	P	303	31.047	6.185	-22.843	0.00	0.00	PROA
ATOM	4936	HB1	ALA	P	303	30.262	6.778	-22.326	0.00	0.00	PROA
ATOM	4937	HB2	ALA	P	303	31.686	6.908	-23.394	0.00	0.00	PROA

ATOM	4938	HB3	ALA	P	303	31.568	5.625	-22.036	0.00	0.00	PROA
ATOM	4939	C	ALA	P	303	31.401	4.562	-24.768	0.00	0.00	PROA
ATOM	4940	O	ALA	P	303	32.009	3.577	-24.363	0.00	0.00	PROA
ATOM	4941	N	PHE	P	304	31.523	4.983	-26.043	0.00	0.00	PROA
ATOM	4942	HN	PHE	P	304	31.105	5.854	-26.289	0.00	0.00	PROA
ATOM	4943	CA	PHE	P	304	32.305	4.442	-27.137	0.00	0.00	PROA
ATOM	4944	HA	PHE	P	304	32.284	5.147	-27.955	0.00	0.00	PROA
ATOM	4945	CB	PHE	P	304	33.843	4.202	-26.884	0.00	0.00	PROA
ATOM	4946	HB1	PHE	P	304	34.250	3.568	-27.700	0.00	0.00	PROA
ATOM	4947	HB2	PHE	P	304	33.960	3.588	-25.966	0.00	0.00	PROA
ATOM	4948	CG	PHE	P	304	34.590	5.503	-26.694	0.00	0.00	PROA
ATOM	4949	CD1	PHE	P	304	34.705	6.231	-25.476	0.00	0.00	PROA
ATOM	4950	HD1	PHE	P	304	34.185	5.939	-24.575	0.00	0.00	PROA
ATOM	4951	CE1	PHE	P	304	35.461	7.472	-25.459	0.00	0.00	PROA
ATOM	4952	HE1	PHE	P	304	35.286	8.051	-24.564	0.00	0.00	PROA
ATOM	4953	CZ	PHE	P	304	36.211	7.897	-26.454	0.00	0.00	PROA
ATOM	4954	HZ	PHE	P	304	36.828	8.783	-26.440	0.00	0.00	PROA
ATOM	4955	CD2	PHE	P	304	35.352	6.026	-27.773	0.00	0.00	PROA
ATOM	4956	HD2	PHE	P	304	35.349	5.553	-28.744	0.00	0.00	PROA
ATOM	4957	CE2	PHE	P	304	36.117	7.185	-27.675	0.00	0.00	PROA
ATOM	4958	HE2	PHE	P	304	36.781	7.522	-28.458	0.00	0.00	PROA
ATOM	4959	C	PHE	P	304	31.621	3.144	-27.684	0.00	0.00	PROA
ATOM	4960	O	PHE	P	304	32.210	2.498	-28.529	0.00	0.00	PROA
ATOM	4961	N	LEU	P	305	30.415	2.869	-27.166	0.00	0.00	PROA
ATOM	4962	HN	LEU	P	305	30.036	3.421	-26.428	0.00	0.00	PROA
ATOM	4963	CA	LEU	P	305	29.451	1.925	-27.669	0.00	0.00	PROA
ATOM	4964	HA	LEU	P	305	28.556	1.956	-27.065	0.00	0.00	PROA
ATOM	4965	CB	LEU	P	305	29.109	2.289	-29.167	0.00	0.00	PROA
ATOM	4966	HB1	LEU	P	305	28.264	1.619	-29.435	0.00	0.00	PROA
ATOM	4967	HB2	LEU	P	305	29.930	2.161	-29.905	0.00	0.00	PROA
ATOM	4968	CG	LEU	P	305	28.598	3.668	-29.519	0.00	0.00	PROA
ATOM	4969	HG	LEU	P	305	29.373	4.385	-29.173	0.00	0.00	PROA
ATOM	4970	CD1	LEU	P	305	28.370	3.818	-31.007	0.00	0.00	PROA
ATOM	4971	HD11	LEU	P	305	29.157	3.220	-31.515	0.00	0.00	PROA
ATOM	4972	HD12	LEU	P	305	28.352	4.863	-31.385	0.00	0.00	PROA
ATOM	4973	HD13	LEU	P	305	27.420	3.264	-31.160	0.00	0.00	PROA
ATOM	4974	CD2	LEU	P	305	27.347	4.095	-28.680	0.00	0.00	PROA
ATOM	4975	HD21	LEU	P	305	27.479	4.230	-27.585	0.00	0.00	PROA
ATOM	4976	HD22	LEU	P	305	26.559	3.346	-28.906	0.00	0.00	PROA
ATOM	4977	HD23	LEU	P	305	26.939	5.072	-29.017	0.00	0.00	PROA
ATOM	4978	C	LEU	P	305	29.887	0.486	-27.713	0.00	0.00	PROA
ATOM	4979	O	LEU	P	305	29.744	-0.206	-28.717	0.00	0.00	PROA
ATOM	4980	N	GLY	P	306	30.403	0.001	-26.569	0.00	0.00	PROA
ATOM	4981	HN	GLY	P	306	30.762	0.534	-25.806	0.00	0.00	PROA
ATOM	4982	CA	GLY	P	306	30.432	-1.445	-26.331	0.00	0.00	PROA
ATOM	4983	HA1	GLY	P	306	29.525	-1.924	-26.668	0.00	0.00	PROA
ATOM	4984	HA2	GLY	P	306	30.576	-1.532	-25.264	0.00	0.00	PROA
ATOM	4985	C	GLY	P	306	31.625	-2.133	-26.830	0.00	0.00	PROA
ATOM	4986	O	GLY	P	306	31.584	-3.049	-27.689	0.00	0.00	PROA
ATOM	4987	N	ALA	P	307	32.788	-1.719	-26.278	0.00	0.00	PROA
ATOM	4988	HN	ALA	P	307	32.824	-1.016	-25.573	0.00	0.00	PROA
ATOM	4989	CA	ALA	P	307	34.104	-1.956	-26.868	0.00	0.00	PROA
ATOM	4990	HA	ALA	P	307	34.135	-2.998	-27.152	0.00	0.00	PROA
ATOM	4991	CB	ALA	P	307	34.490	-0.936	-27.978	0.00	0.00	PROA
ATOM	4992	HB1	ALA	P	307	34.309	0.120	-27.683	0.00	0.00	PROA
ATOM	4993	HB2	ALA	P	307	33.816	-1.064	-28.853	0.00	0.00	PROA
ATOM	4994	HB3	ALA	P	307	35.539	-1.108	-28.302	0.00	0.00	PROA
ATOM	4995	C	ALA	P	307	35.113	-1.734	-25.732	0.00	0.00	PROA
ATOM	4996	O	ALA	P	307	34.895	-1.110	-24.708	0.00	0.00	PROA
ATOM	4997	N	LYS	P	308	36.288	-2.284	-25.930	0.00	0.00	PROA
ATOM	4998	HN	LYS	P	308	36.379	-2.878	-26.726	0.00	0.00	PROA

ATOM	4999	CA	LYS	P	308	37.483	-2.040	-25.152	0.00	0.00	PROA
ATOM	5000	HA	LYS	P	308	37.187	-2.287	-24.143	0.00	0.00	PROA
ATOM	5001	CB	LYS	P	308	38.615	-2.966	-25.592	0.00	0.00	PROA
ATOM	5002	HB1	LYS	P	308	38.411	-3.953	-25.124	0.00	0.00	PROA
ATOM	5003	HB2	LYS	P	308	39.540	-2.469	-25.230	0.00	0.00	PROA
ATOM	5004	CG	LYS	P	308	38.729	-3.236	-27.097	0.00	0.00	PROA
ATOM	5005	HG1	LYS	P	308	38.475	-2.341	-27.705	0.00	0.00	PROA
ATOM	5006	HG2	LYS	P	308	38.041	-4.035	-27.448	0.00	0.00	PROA
ATOM	5007	CD	LYS	P	308	40.128	-3.708	-27.505	0.00	0.00	PROA
ATOM	5008	HD1	LYS	P	308	40.897	-3.052	-27.045	0.00	0.00	PROA
ATOM	5009	HD2	LYS	P	308	40.094	-3.640	-28.614	0.00	0.00	PROA
ATOM	5010	CE	LYS	P	308	40.340	-5.177	-26.979	0.00	0.00	PROA
ATOM	5011	HE1	LYS	P	308	39.676	-5.845	-27.567	0.00	0.00	PROA
ATOM	5012	HE2	LYS	P	308	40.076	-5.249	-25.902	0.00	0.00	PROA
ATOM	5013	NZ	LYS	P	308	41.746	-5.599	-27.084	0.00	0.00	PROA
ATOM	5014	HZ1	LYS	P	308	41.881	-6.630	-27.100	0.00	0.00	PROA
ATOM	5015	HZ2	LYS	P	308	42.259	-5.116	-26.319	0.00	0.00	PROA
ATOM	5016	HZ3	LYS	P	308	42.141	-5.192	-27.956	0.00	0.00	PROA
ATOM	5017	C	LYS	P	308	37.845	-0.493	-24.970	0.00	0.00	PROA
ATOM	5018	O	LYS	P	308	37.666	0.281	-25.940	0.00	0.00	PROA
ATOM	5019	N	PHE	P	309	38.351	-0.065	-23.822	0.00	0.00	PROA
ATOM	5020	HN	PHE	P	309	38.496	-0.686	-23.056	0.00	0.00	PROA
ATOM	5021	CA	PHE	P	309	38.475	1.338	-23.380	0.00	0.00	PROA
ATOM	5022	HA	PHE	P	309	37.459	1.658	-23.561	0.00	0.00	PROA
ATOM	5023	CB	PHE	P	309	38.827	1.592	-21.905	0.00	0.00	PROA
ATOM	5024	HB1	PHE	P	309	38.815	2.670	-21.636	0.00	0.00	PROA
ATOM	5025	HB2	PHE	P	309	39.799	1.149	-21.598	0.00	0.00	PROA
ATOM	5026	CG	PHE	P	309	37.735	1.016	-21.014	0.00	0.00	PROA
ATOM	5027	CD1	PHE	P	309	36.482	1.695	-20.894	0.00	0.00	PROA
ATOM	5028	HD1	PHE	P	309	36.174	2.616	-21.367	0.00	0.00	PROA
ATOM	5029	CE1	PHE	P	309	35.506	1.225	-20.048	0.00	0.00	PROA
ATOM	5030	HE1	PHE	P	309	34.602	1.802	-19.921	0.00	0.00	PROA
ATOM	5031	CZ	PHE	P	309	35.654	0.138	-19.277	0.00	0.00	PROA
ATOM	5032	HZ	PHE	P	309	34.896	-0.170	-18.572	0.00	0.00	PROA
ATOM	5033	CD2	PHE	P	309	37.915	-0.168	-20.202	0.00	0.00	PROA
ATOM	5034	HD2	PHE	P	309	38.720	-0.854	-20.419	0.00	0.00	PROA
ATOM	5035	CE2	PHE	P	309	36.875	-0.557	-19.329	0.00	0.00	PROA
ATOM	5036	HE2	PHE	P	309	36.963	-1.526	-18.861	0.00	0.00	PROA
ATOM	5037	C	PHE	P	309	39.338	2.154	-24.306	0.00	0.00	PROA
ATOM	5038	O	PHE	P	309	40.333	1.653	-24.929	0.00	0.00	PROA
ATOM	5039	N	LYS	P	310	39.053	3.491	-24.505	0.00	0.00	PROA
ATOM	5040	HN	LYS	P	310	38.213	3.868	-24.123	0.00	0.00	PROA
ATOM	5041	CA	LYS	P	310	39.792	4.370	-25.480	0.00	0.00	PROA
ATOM	5042	HA	LYS	P	310	40.076	3.806	-26.356	0.00	0.00	PROA
ATOM	5043	CB	LYS	P	310	39.074	5.638	-25.845	0.00	0.00	PROA
ATOM	5044	HB1	LYS	P	310	38.679	6.054	-24.894	0.00	0.00	PROA
ATOM	5045	HB2	LYS	P	310	38.173	5.393	-26.446	0.00	0.00	PROA
ATOM	5046	CG	LYS	P	310	39.824	6.820	-26.508	0.00	0.00	PROA
ATOM	5047	HG1	LYS	P	310	40.810	7.087	-26.070	0.00	0.00	PROA
ATOM	5048	HG2	LYS	P	310	39.258	7.776	-26.538	0.00	0.00	PROA
ATOM	5049	CD	LYS	P	310	40.055	6.508	-28.033	0.00	0.00	PROA
ATOM	5050	HD1	LYS	P	310	39.024	6.339	-28.410	0.00	0.00	PROA
ATOM	5051	HD2	LYS	P	310	40.666	5.580	-28.058	0.00	0.00	PROA
ATOM	5052	CE	LYS	P	310	40.733	7.654	-28.863	0.00	0.00	PROA
ATOM	5053	HE1	LYS	P	310	41.722	7.758	-28.368	0.00	0.00	PROA
ATOM	5054	HE2	LYS	P	310	40.177	8.608	-28.735	0.00	0.00	PROA
ATOM	5055	NZ	LYS	P	310	40.926	7.279	-30.253	0.00	0.00	PROA
ATOM	5056	HZ1	LYS	P	310	39.979	7.179	-30.669	0.00	0.00	PROA
ATOM	5057	HZ2	LYS	P	310	41.399	6.354	-30.298	0.00	0.00	PROA
ATOM	5058	HZ3	LYS	P	310	41.463	7.963	-30.823	0.00	0.00	PROA
ATOM	5059	C	LYS	P	310	41.145	4.835	-24.975	0.00	0.00	PROA

ATOM 5060 O LYS P 310 42.042 5.098 -25.759 0.00 0.00 PROA
ATOM 5061 N THR P 311 41.416 4.932 -23.685 0.00 0.00 PROA
ATOM 5062 HN THR P 311 40.692 4.805 -23.011 0.00 0.00 PROA
ATOM 5063 CA THR P 311 42.724 5.484 -23.224 0.00 0.00 PROA
ATOM 5064 HA THR P 311 42.857 6.337 -23.873 0.00 0.00 PROA
ATOM 5065 CB THR P 311 42.566 5.980 -21.772 0.00 0.00 PROA
ATOM 5066 HB THR P 311 41.575 6.455 -21.608 0.00 0.00 PROA
ATOM 5067 OG1 THR P 311 43.593 6.976 -21.579 0.00 0.00 PROA
ATOM 5068 HG1 THR P 311 43.569 7.691 -22.219 0.00 0.00 PROA
ATOM 5069 CG2 THR P 311 42.753 4.846 -20.781 0.00 0.00 PROA
ATOM 5070 HG21 THR P 311 43.832 4.580 -20.753 0.00 0.00 PROA
ATOM 5071 HG22 THR P 311 42.114 3.959 -20.981 0.00 0.00 PROA
ATOM 5072 HG23 THR P 311 42.689 5.159 -19.717 0.00 0.00 PROA
ATOM 5073 C THR P 311 43.808 4.462 -23.375 0.00 0.00 PROA
ATOM 5074 O THR P 311 43.547 3.323 -23.306 0.00 0.00 PROA
ATOM 5075 N SER P 312 45.076 4.884 -23.698 0.00 0.00 PROA
ATOM 5076 HN SER P 312 45.284 5.855 -23.791 0.00 0.00 PROA
ATOM 5077 CA SER P 312 46.187 4.011 -23.903 0.00 0.00 PROA
ATOM 5078 HA SER P 312 46.124 3.298 -23.094 0.00 0.00 PROA
ATOM 5079 CB SER P 312 46.249 3.318 -25.348 0.00 0.00 PROA
ATOM 5080 HB1 SER P 312 46.190 4.113 -26.122 0.00 0.00 PROA
ATOM 5081 HB2 SER P 312 45.465 2.536 -25.437 0.00 0.00 PROA
ATOM 5082 OG SER P 312 47.470 2.661 -25.557 0.00 0.00 PROA
ATOM 5083 HG1 SER P 312 47.467 2.091 -24.785 0.00 0.00 PROA
ATOM 5084 C SER P 312 47.629 4.619 -23.573 0.00 0.00 PROA
ATOM 5085 O SER P 312 47.993 5.713 -24.062 0.00 0.00 PROA
ATOM 5086 N ALA P 313 48.474 3.853 -22.921 0.00 0.00 PROA
ATOM 5087 HN ALA P 313 48.283 2.897 -22.713 0.00 0.00 PROA
ATOM 5088 CA ALA P 313 49.753 4.324 -22.617 0.00 0.00 PROA
ATOM 5089 HA ALA P 313 49.607 5.233 -22.053 0.00 0.00 PROA
ATOM 5090 CB ALA P 313 50.512 3.346 -21.695 0.00 0.00 PROA
ATOM 5091 HB1 ALA P 313 49.858 2.845 -20.950 0.00 0.00 PROA
ATOM 5092 HB2 ALA P 313 51.394 3.866 -21.263 0.00 0.00 PROA
ATOM 5093 HB3 ALA P 313 50.847 2.440 -22.242 0.00 0.00 PROA
ATOM 5094 C ALA P 313 50.715 4.718 -23.747 0.00 0.00 PROA
ATOM 5095 O ALA P 313 51.357 5.786 -23.766 0.00 0.00 PROA
ATOM 5096 N GLN P 314 50.764 3.856 -24.796 0.00 0.00 PROA
ATOM 5097 HN GLN P 314 50.207 3.037 -24.678 0.00 0.00 PROA
ATOM 5098 CA GLN P 314 51.581 3.905 -25.977 0.00 0.00 PROA
ATOM 5099 HA GLN P 314 52.615 3.905 -25.664 0.00 0.00 PROA
ATOM 5100 CB GLN P 314 51.341 2.619 -26.771 0.00 0.00 PROA
ATOM 5101 HB1 GLN P 314 50.274 2.567 -27.077 0.00 0.00 PROA
ATOM 5102 HB2 GLN P 314 51.667 1.819 -26.072 0.00 0.00 PROA
ATOM 5103 CG GLN P 314 52.143 2.329 -28.005 0.00 0.00 PROA
ATOM 5104 HG1 GLN P 314 51.775 2.951 -28.849 0.00 0.00 PROA
ATOM 5105 HG2 GLN P 314 52.047 1.268 -28.318 0.00 0.00 PROA
ATOM 5106 CD GLN P 314 53.633 2.758 -27.858 0.00 0.00 PROA
ATOM 5107 OE1 GLN P 314 53.963 3.843 -28.351 0.00 0.00 PROA
ATOM 5108 NE2 GLN P 314 54.502 1.884 -27.403 0.00 0.00 PROA
ATOM 5109 HE21 GLN P 314 55.474 2.108 -27.323 0.00 0.00 PROA
ATOM 5110 HE22 GLN P 314 54.230 0.979 -27.075 0.00 0.00 PROA
ATOM 5111 C GLN P 314 51.096 5.183 -26.760 0.00 0.00 PROA
ATOM 5112 O GLN P 314 51.898 6.024 -27.184 0.00 0.00 PROA
ATOM 5113 N HSD P 315 49.824 5.500 -26.706 0.00 0.00 PROA
ATOM 5114 HN HSD P 315 49.184 4.904 -26.226 0.00 0.00 PROA
ATOM 5115 CA HSD P 315 49.323 6.679 -27.400 0.00 0.00 PROA
ATOM 5116 HA HSD P 315 49.852 6.650 -28.341 0.00 0.00 PROA
ATOM 5117 CB HSD P 315 47.899 6.555 -27.863 0.00 0.00 PROA
ATOM 5118 HB1 HSD P 315 47.285 6.546 -26.937 0.00 0.00 PROA
ATOM 5119 HB2 HSD P 315 47.757 5.693 -28.550 0.00 0.00 PROA
ATOM 5120 ND1 HSD P 315 47.942 8.319 -29.769 0.00 0.00 PROA

```
ATOM   5121  HD1 HSD P 315      48.859   8.126 -30.120  0.00  0.00      PROA
ATOM   5122  CG  HSD P 315      47.382   7.751 -28.626  0.00  0.00      PROA
ATOM   5123  CE1 HSD P 315      47.066   9.266 -30.180  0.00  0.00      PROA
ATOM   5124  HE1 HSD P 315      47.150   9.908 -31.057  0.00  0.00      PROA
ATOM   5125  NE2 HSD P 315      46.037   9.400 -29.407  0.00  0.00      PROA
ATOM   5126  CD2 HSD P 315      46.235   8.509 -28.422  0.00  0.00      PROA
ATOM   5127  HD2 HSD P 315      45.585   8.473 -27.556  0.00  0.00      PROA
ATOM   5128  C   HSD P 315      49.683   7.991 -26.707  0.00  0.00      PROA
ATOM   5129  O   HSD P 315      50.179   8.873 -27.338  0.00  0.00      PROA
ATOM   5130  N   ALA P 316      49.528   8.008 -25.404  0.00  0.00      PROA
ATOM   5131  HN  ALA P 316      49.257   7.189 -24.904  0.00  0.00      PROA
ATOM   5132  CA  ALA P 316      49.868   9.166 -24.634  0.00  0.00      PROA
ATOM   5133  HA  ALA P 316      49.341  10.052 -24.955  0.00  0.00      PROA
ATOM   5134  CB  ALA P 316      49.335   8.740 -23.214  0.00  0.00      PROA
ATOM   5135  HB1 ALA P 316      49.996   7.932 -22.835  0.00  0.00      PROA
ATOM   5136  HB2 ALA P 316      48.334   8.265 -23.301  0.00  0.00      PROA
ATOM   5137  HB3 ALA P 316      49.191   9.645 -22.585  0.00  0.00      PROA
ATOM   5138  C   ALA P 316      51.289   9.493 -24.639  0.00  0.00      PROA
ATOM   5139  O   ALA P 316      51.666  10.582 -25.026  0.00  0.00      PROA
ATOM   5140  N   LEU P 317      52.156   8.557 -24.396  0.00  0.00      PROA
ATOM   5141  HN  LEU P 317      51.893   7.595 -24.377  0.00  0.00      PROA
ATOM   5142  CA  LEU P 317      53.591   8.735 -24.137  0.00  0.00      PROA
ATOM   5143  HA  LEU P 317      53.739   9.699 -23.671  0.00  0.00      PROA
ATOM   5144  CB  LEU P 317      53.974   7.720 -23.040  0.00  0.00      PROA
ATOM   5145  HB1 LEU P 317      55.009   7.985 -22.736  0.00  0.00      PROA
ATOM   5146  HB2 LEU P 317      53.962   6.670 -23.405  0.00  0.00      PROA
ATOM   5147  CG  LEU P 317      53.079   7.845 -21.804  0.00  0.00      PROA
ATOM   5148  HG  LEU P 317      52.037   7.492 -21.960  0.00  0.00      PROA
ATOM   5149  CD1 LEU P 317      53.675   6.973 -20.649  0.00  0.00      PROA
ATOM   5150 HD11 LEU P 317      54.766   7.172 -20.578  0.00  0.00      PROA
ATOM   5151 HD12 LEU P 317      53.524   5.899 -20.889  0.00  0.00      PROA
ATOM   5152 HD13 LEU P 317      53.132   7.249 -19.720  0.00  0.00      PROA
ATOM   5153  CD2 LEU P 317      52.900   9.291 -21.210  0.00  0.00      PROA
ATOM   5154 HD21 LEU P 317      52.122   9.741 -21.863  0.00  0.00      PROA
ATOM   5155 HD22 LEU P 317      53.892   9.790 -21.216  0.00  0.00      PROA
ATOM   5156 HD23 LEU P 317      52.505   9.337 -20.172  0.00  0.00      PROA
ATOM   5157  C   LEU P 317      54.539   8.597 -25.393  0.00  0.00      PROA
ATOM   5158  O   LEU P 317      55.747   8.715 -25.232  0.00  0.00      PROA
ATOM   5159  N   THR P 318      54.005   8.383 -26.599  0.00  0.00      PROA
ATOM   5160  HN  THR P 318      53.011   8.417 -26.674  0.00  0.00      PROA
ATOM   5161  CA  THR P 318      54.786   8.339 -27.839  0.00  0.00      PROA
ATOM   5162  HA  THR P 318      55.673   7.781 -27.578  0.00  0.00      PROA
ATOM   5163  CB  THR P 318      54.127   7.612 -28.957  0.00  0.00      PROA
ATOM   5164  HB  THR P 318      53.841   6.572 -28.690  0.00  0.00      PROA
ATOM   5165  OG1 THR P 318      54.920   7.482 -30.131  0.00  0.00      PROA
ATOM   5166  HG1 THR P 318      55.482   6.725 -29.952  0.00  0.00      PROA
ATOM   5167  CG2 THR P 318      52.783   8.223 -29.308  0.00  0.00      PROA
ATOM   5168 HG21 THR P 318      52.154   8.237 -28.392  0.00  0.00      PROA
ATOM   5169 HG22 THR P 318      52.273   7.670 -30.126  0.00  0.00      PROA
ATOM   5170 HG23 THR P 318      52.912   9.283 -29.614  0.00  0.00      PROA
ATOM   5171  C   THR P 318      55.239   9.744 -28.274  0.00  0.00      PROA
ATOM   5172  O   THR P 318      54.640  10.749 -27.796  0.00  0.00      PROA
ATOM   5173  N   SER P 319      56.311   9.852 -29.134  0.00  0.00      PROA
ATOM   5174  HN  SER P 319      56.691   9.015 -29.521  0.00  0.00      PROA
ATOM   5175  CA  SER P 319      56.801  11.090 -29.692  0.00  0.00      PROA
ATOM   5176  HA  SER P 319      56.054  11.835 -29.459  0.00  0.00      PROA
ATOM   5177  CB  SER P 319      58.230  11.536 -29.257  0.00  0.00      PROA
ATOM   5178  HB1 SER P 319      58.524  12.490 -29.744  0.00  0.00      PROA
ATOM   5179  HB2 SER P 319      58.941  10.765 -29.624  0.00  0.00      PROA
ATOM   5180  OG  SER P 319      58.364  11.577 -27.840  0.00  0.00      PROA
ATOM   5181  HG1 SER P 319      58.264  10.694 -27.476  0.00  0.00      PROA
```

```
ATOM   5182  C   SER P 319      56.922  10.992 -31.170  0.00  0.00      PROA
ATOM   5183  OT1 SER P 319      56.957  12.065 -31.880  0.00  0.00      PROA
ATOM   5184  OT2 SER P 319      57.034   9.828 -31.684  0.00  0.00      PROA
ATOM   5185  N   LEU P   1      16.286   4.279   5.592  0.00  0.00      PROB
ATOM   5186  HT1 LEU P   1      16.841   4.970   6.136  0.00  0.00      PROB
ATOM   5187  HT2 LEU P   1      16.151   3.461   6.221  0.00  0.00      PROB
ATOM   5188  HT3 LEU P   1      15.378   4.782   5.525  0.00  0.00      PROB
ATOM   5189  CA  LEU P   1      16.818   4.003   4.254  0.00  0.00      PROB
ATOM   5190  HA  LEU P   1      16.084   3.485   3.655  0.00  0.00      PROB
ATOM   5191  CB  LEU P   1      17.423   5.298   3.646  0.00  0.00      PROB
ATOM   5192  HB1 LEU P   1      17.835   5.109   2.632  0.00  0.00      PROB
ATOM   5193  HB2 LEU P   1      18.176   5.813   4.280  0.00  0.00      PROB
ATOM   5194  CG  LEU P   1      16.316   6.249   3.245  0.00  0.00      PROB
ATOM   5195  HG  LEU P   1      15.803   6.657   4.141  0.00  0.00      PROB
ATOM   5196  CD1 LEU P   1      16.954   7.421   2.567  0.00  0.00      PROB
ATOM   5197 HD11 LEU P   1      16.125   8.083   2.239  0.00  0.00      PROB
ATOM   5198 HD12 LEU P   1      17.697   7.193   1.773  0.00  0.00      PROB
ATOM   5199 HD13 LEU P   1      17.643   7.982   3.234  0.00  0.00      PROB
ATOM   5200  CD2 LEU P   1      15.280   5.608   2.306  0.00  0.00      PROB
ATOM   5201 HD21 LEU P   1      15.775   4.761   1.786  0.00  0.00      PROB
ATOM   5202 HD22 LEU P   1      14.864   6.289   1.534  0.00  0.00      PROB
ATOM   5203 HD23 LEU P   1      14.412   5.238   2.893  0.00  0.00      PROB
ATOM   5204  C   LEU P   1      17.930   3.016   4.391  0.00  0.00      PROB
ATOM   5205  O   LEU P   1      17.702   1.815   4.467  0.00  0.00      PROB
ATOM   5206  N   VAL P   2      19.094   3.541   4.801  0.00  0.00      PROB
ATOM   5207  HN  VAL P   2      19.265   4.517   4.918  0.00  0.00      PROB
ATOM   5208  CA  VAL P   2      20.071   2.640   5.428  0.00  0.00      PROB
ATOM   5209  HA  VAL P   2      20.282   1.778   4.812  0.00  0.00      PROB
ATOM   5210  CB  VAL P   2      21.368   3.415   5.522  0.00  0.00      PROB
ATOM   5211  HB  VAL P   2      21.275   4.388   6.050  0.00  0.00      PROB
ATOM   5212  CG1 VAL P   2      22.553   2.687   6.187  0.00  0.00      PROB
ATOM   5213 HG11 VAL P   2      22.622   1.630   5.852  0.00  0.00      PROB
ATOM   5214 HG12 VAL P   2      22.477   2.767   7.293  0.00  0.00      PROB
ATOM   5215 HG13 VAL P   2      23.474   3.237   5.900  0.00  0.00      PROB
ATOM   5216  CG2 VAL P   2      21.673   3.799   4.109  0.00  0.00      PROB
ATOM   5217 HG21 VAL P   2      21.498   2.918   3.455  0.00  0.00      PROB
ATOM   5218 HG22 VAL P   2      22.739   4.075   3.958  0.00  0.00      PROB
ATOM   5219 HG23 VAL P   2      21.013   4.606   3.726  0.00  0.00      PROB
ATOM   5220  C   VAL P   2      19.603   2.168   6.768  0.00  0.00      PROB
ATOM   5221  O   VAL P   2      19.891   1.039   7.136  0.00  0.00      PROB
ATOM   5222  N   ARG P   3      18.803   2.941   7.512  0.00  0.00      PROB
ATOM   5223  HN  ARG P   3      18.717   3.926   7.380  0.00  0.00      PROB
ATOM   5224  CA  ARG P   3      18.229   2.518   8.750  0.00  0.00      PROB
ATOM   5225  HA  ARG P   3      18.715   1.600   9.044  0.00  0.00      PROB
ATOM   5226  CB  ARG P   3      18.679   3.413   9.967  0.00  0.00      PROB
ATOM   5227  HB1 ARG P   3      18.093   3.087  10.853  0.00  0.00      PROB
ATOM   5228  HB2 ARG P   3      18.426   4.458   9.687  0.00  0.00      PROB
ATOM   5229  CG  ARG P   3      20.211   3.443  10.300  0.00  0.00      PROB
ATOM   5230  HG1 ARG P   3      20.454   4.148  11.124  0.00  0.00      PROB
ATOM   5231  HG2 ARG P   3      20.615   3.958   9.403  0.00  0.00      PROB
ATOM   5232  CD  ARG P   3      20.903   2.163  10.606  0.00  0.00      PROB
ATOM   5233  HD1 ARG P   3      22.007   2.293  10.610  0.00  0.00      PROB
ATOM   5234  HD2 ARG P   3      20.788   1.435   9.774  0.00  0.00      PROB
ATOM   5235  NE  ARG P   3      20.176   1.478  11.661  0.00  0.00      PROB
ATOM   5236  HE  ARG P   3      19.619   0.674  11.454  0.00  0.00      PROB
ATOM   5237  CZ  ARG P   3      20.576   1.584  12.925  0.00  0.00      PROB
ATOM   5238  NH1 ARG P   3      21.681   2.158  13.314  0.00  0.00      PROB
ATOM   5239 HH11 ARG P   3      22.224   2.676  12.653  0.00  0.00      PROB
ATOM   5240 HH12 ARG P   3      21.967   2.235  14.269  0.00  0.00      PROB
ATOM   5241  NH2 ARG P   3      19.817   1.143  13.915  0.00  0.00      PROB
ATOM   5242 HH21 ARG P   3      19.083   0.498  13.698  0.00  0.00      PROB
```

```
ATOM   5243 HH22 ARG P   3      19.921   1.404  14.874  0.00  0.00      PROB
ATOM   5244  C   ARG P   3      16.741   2.177   8.638  0.00  0.00      PROB
ATOM   5245  O   ARG P   3      15.996   3.059   8.201  0.00  0.00      PROB
ATOM   5246  N   TYR P   4      16.319   0.960   9.119  0.00  0.00      PROB
ATOM   5247  HN  TYR P   4      17.032   0.335   9.428  0.00  0.00      PROB
ATOM   5248  CA  TYR P   4      15.022   0.497   9.355  0.00  0.00      PROB
ATOM   5249  HA  TYR P   4      14.351   1.332   9.219  0.00  0.00      PROB
ATOM   5250  CB  TYR P   4      14.584  -0.574   8.340  0.00  0.00      PROB
ATOM   5251  HB1 TYR P   4      13.603  -1.010   8.627  0.00  0.00      PROB
ATOM   5252  HB2 TYR P   4      15.322  -1.397   8.224  0.00  0.00      PROB
ATOM   5253  CG  TYR P   4      14.430  -0.043   6.932  0.00  0.00      PROB
ATOM   5254  CD1 TYR P   4      15.092  -0.794   5.877  0.00  0.00      PROB
ATOM   5255  HD1 TYR P   4      15.756  -1.577   6.213  0.00  0.00      PROB
ATOM   5256  CE1 TYR P   4      15.033  -0.331   4.554  0.00  0.00      PROB
ATOM   5257  HE1 TYR P   4      15.631  -0.869   3.833  0.00  0.00      PROB
ATOM   5258  CZ  TYR P   4      14.199   0.760   4.221  0.00  0.00      PROB
ATOM   5259  OH  TYR P   4      14.191   1.333   2.936  0.00  0.00      PROB
ATOM   5260  HH  TYR P   4      14.826   0.799   2.454  0.00  0.00      PROB
ATOM   5261  CD2 TYR P   4      13.696   1.079   6.564  0.00  0.00      PROB
ATOM   5262  HD2 TYR P   4      13.141   1.601   7.330  0.00  0.00      PROB
ATOM   5263  CE2 TYR P   4      13.457   1.390   5.248  0.00  0.00      PROB
ATOM   5264  HE2 TYR P   4      12.950   2.301   4.964  0.00  0.00      PROB
ATOM   5265  C   TYR P   4      14.875   0.024  10.719  0.00  0.00      PROB
ATOM   5266  O   TYR P   4      15.761  -0.612  11.361  0.00  0.00      PROB
ATOM   5267  N   THR P   5      13.725   0.269  11.354  0.00  0.00      PROB
ATOM   5268  HN  THR P   5      12.925   0.644  10.891  0.00  0.00      PROB
ATOM   5269  CA  THR P   5      13.558   0.021  12.840  0.00  0.00      PROB
ATOM   5270  HA  THR P   5      14.263   0.644  13.369  0.00  0.00      PROB
ATOM   5271  CB  THR P   5      12.170   0.478  13.412  0.00  0.00      PROB
ATOM   5272  HB  THR P   5      12.211   0.381  14.518  0.00  0.00      PROB
ATOM   5273  OG1 THR P   5      11.065  -0.116  12.759  0.00  0.00      PROB
ATOM   5274  HG1 THR P   5      11.244   0.058  11.832  0.00  0.00      PROB
ATOM   5275  CG2 THR P   5      12.056   1.953  13.077  0.00  0.00      PROB
ATOM   5276 HG21 THR P   5      12.903   2.652  13.247  0.00  0.00      PROB
ATOM   5277 HG22 THR P   5      11.155   2.296  13.629  0.00  0.00      PROB
ATOM   5278 HG23 THR P   5      11.781   1.982  12.001  0.00  0.00      PROB
ATOM   5279  C   THR P   5      13.662  -1.434  13.212  0.00  0.00      PROB
ATOM   5280  O   THR P   5      14.188  -1.710  14.243  0.00  0.00      PROB
ATOM   5281  N   LYS P   6      13.137  -2.330  12.403  0.00  0.00      PROB
ATOM   5282  HN  LYS P   6      12.720  -2.186  11.509  0.00  0.00      PROB
ATOM   5283  CA  LYS P   6      13.063  -3.669  12.783  0.00  0.00      PROB
ATOM   5284  HA  LYS P   6      13.202  -3.744  13.852  0.00  0.00      PROB
ATOM   5285  CB  LYS P   6      11.747  -4.339  12.360  0.00  0.00      PROB
ATOM   5286  HB1 LYS P   6      11.773  -5.370  12.774  0.00  0.00      PROB
ATOM   5287  HB2 LYS P   6      11.644  -4.341  11.254  0.00  0.00      PROB
ATOM   5288  CG  LYS P   6      10.471  -3.798  12.963  0.00  0.00      PROB
ATOM   5289  HG1 LYS P   6      10.215  -2.779  12.599  0.00  0.00      PROB
ATOM   5290  HG2 LYS P   6      10.655  -3.910  14.053  0.00  0.00      PROB
ATOM   5291  CD  LYS P   6       9.286  -4.777  12.840  0.00  0.00      PROB
ATOM   5292  HD1 LYS P   6       9.418  -5.860  13.050  0.00  0.00      PROB
ATOM   5293  HD2 LYS P   6       8.778  -4.886  11.859  0.00  0.00      PROB
ATOM   5294  CE  LYS P   6       8.153  -4.343  13.810  0.00  0.00      PROB
ATOM   5295  HE1 LYS P   6       7.788  -3.317  13.589  0.00  0.00      PROB
ATOM   5296  HE2 LYS P   6       8.542  -4.301  14.850  0.00  0.00      PROB
ATOM   5297  NZ  LYS P   6       7.008  -5.338  13.985  0.00  0.00      PROB
ATOM   5298  HZ1 LYS P   6       7.349  -6.320  14.010  0.00  0.00      PROB
ATOM   5299  HZ2 LYS P   6       6.397  -5.336  13.143  0.00  0.00      PROB
ATOM   5300  HZ3 LYS P   6       6.441  -5.135  14.833  0.00  0.00      PROB
ATOM   5301  C   LYS P   6      14.212  -4.505  12.197  0.00  0.00      PROB
ATOM   5302  O   LYS P   6      14.328  -5.671  12.546  0.00  0.00      PROB
ATOM   5303  N   LYS P   7      15.114  -3.956  11.377  0.00  0.00      PROB
```

ATOM	5304	HN	LYS	P	7	14.882	-3.139	10.854	0.00	0.00	PROB
ATOM	5305	CA	LYS	P	7	16.397	-4.608	11.052	0.00	0.00	PROB
ATOM	5306	HA	LYS	P	7	16.265	-5.670	11.200	0.00	0.00	PROB
ATOM	5307	CB	LYS	P	7	16.636	-4.403	9.620	0.00	0.00	PROB
ATOM	5308	HB1	LYS	P	7	16.583	-3.308	9.443	0.00	0.00	PROB
ATOM	5309	HB2	LYS	P	7	15.844	-4.818	8.960	0.00	0.00	PROB
ATOM	5310	CG	LYS	P	7	18.015	-4.937	9.180	0.00	0.00	PROB
ATOM	5311	HG1	LYS	P	7	18.842	-4.378	9.667	0.00	0.00	PROB
ATOM	5312	HG2	LYS	P	7	18.123	-4.640	8.115	0.00	0.00	PROB
ATOM	5313	CD	LYS	P	7	18.376	-6.462	9.211	0.00	0.00	PROB
ATOM	5314	HD1	LYS	P	7	18.264	-6.756	10.276	0.00	0.00	PROB
ATOM	5315	HD2	LYS	P	7	19.404	-6.633	8.827	0.00	0.00	PROB
ATOM	5316	CE	LYS	P	7	17.443	-7.317	8.392	0.00	0.00	PROB
ATOM	5317	HE1	LYS	P	7	16.399	-7.056	8.669	0.00	0.00	PROB
ATOM	5318	HE2	LYS	P	7	17.611	-8.365	8.719	0.00	0.00	PROB
ATOM	5319	NZ	LYS	P	7	17.591	-7.231	6.905	0.00	0.00	PROB
ATOM	5320	HZ1	LYS	P	7	17.454	-8.156	6.449	0.00	0.00	PROB
ATOM	5321	HZ2	LYS	P	7	18.525	-6.907	6.584	0.00	0.00	PROB
ATOM	5322	HZ3	LYS	P	7	16.816	-6.618	6.579	0.00	0.00	PROB
ATOM	5323	C	LYS	P	7	17.516	-4.083	11.824	0.00	0.00	PROB
ATOM	5324	O	LYS	P	7	17.831	-2.873	11.753	0.00	0.00	PROB
ATOM	5325	N	VAL	P	8	18.102	-4.934	12.635	0.00	0.00	PROB
ATOM	5326	HN	VAL	P	8	17.812	-5.886	12.692	0.00	0.00	PROB
ATOM	5327	CA	VAL	P	8	19.100	-4.670	13.654	0.00	0.00	PROB
ATOM	5328	HA	VAL	P	8	19.061	-5.523	14.316	0.00	0.00	PROB
ATOM	5329	CB	VAL	P	8	20.543	-4.493	13.202	0.00	0.00	PROB
ATOM	5330	HB	VAL	P	8	20.689	-3.565	12.609	0.00	0.00	PROB
ATOM	5331	CG1	VAL	P	8	21.524	-4.405	14.414	0.00	0.00	PROB
ATOM	5332	HG11	VAL	P	8	21.247	-3.555	15.074	0.00	0.00	PROB
ATOM	5333	HG12	VAL	P	8	22.517	-4.212	13.955	0.00	0.00	PROB
ATOM	5334	HG13	VAL	P	8	21.520	-5.358	14.986	0.00	0.00	PROB
ATOM	5335	CG2	VAL	P	8	20.976	-5.624	12.266	0.00	0.00	PROB
ATOM	5336	HG21	VAL	P	8	20.484	-5.523	11.275	0.00	0.00	PROB
ATOM	5337	HG22	VAL	P	8	20.807	-6.640	12.682	0.00	0.00	PROB
ATOM	5338	HG23	VAL	P	8	22.072	-5.578	12.091	0.00	0.00	PROB
ATOM	5339	C	VAL	P	8	18.644	-3.541	14.643	0.00	0.00	PROB
ATOM	5340	O	VAL	P	8	19.289	-2.509	14.610	0.00	0.00	PROB
ATOM	5341	N	PRO	P	9	17.698	-3.688	15.542	0.00	0.00	PROB
ATOM	5342	CD	PRO	P	9	16.614	-4.700	15.455	0.00	0.00	PROB
ATOM	5343	HD1	PRO	P	9	17.017	-5.546	16.053	0.00	0.00	PROB
ATOM	5344	HD2	PRO	P	9	16.356	-5.003	14.418	0.00	0.00	PROB
ATOM	5345	CA	PRO	P	9	17.397	-2.700	16.624	0.00	0.00	PROB
ATOM	5346	HA	PRO	P	9	17.219	-1.712	16.226	0.00	0.00	PROB
ATOM	5347	CB	PRO	P	9	16.160	-3.133	17.372	0.00	0.00	PROB
ATOM	5348	HB1	PRO	P	9	15.465	-2.310	17.645	0.00	0.00	PROB
ATOM	5349	HB2	PRO	P	9	16.251	-3.881	18.187	0.00	0.00	PROB
ATOM	5350	CG	PRO	P	9	15.491	-4.066	16.249	0.00	0.00	PROB
ATOM	5351	HG1	PRO	P	9	14.967	-4.879	16.796	0.00	0.00	PROB
ATOM	5352	HG2	PRO	P	9	14.868	-3.395	15.620	0.00	0.00	PROB
ATOM	5353	C	PRO	P	9	18.587	-2.542	17.485	0.00	0.00	PROB
ATOM	5354	O	PRO	P	9	18.906	-1.367	17.741	0.00	0.00	PROB
ATOM	5355	N	GLN	P	10	19.165	-3.678	17.903	0.00	0.00	PROB
ATOM	5356	HN	GLN	P	10	18.833	-4.577	17.628	0.00	0.00	PROB
ATOM	5357	CA	GLN	P	10	20.276	-3.656	18.815	0.00	0.00	PROB
ATOM	5358	HA	GLN	P	10	20.598	-2.666	19.102	0.00	0.00	PROB
ATOM	5359	CB	GLN	P	10	19.970	-4.420	20.168	0.00	0.00	PROB
ATOM	5360	HB1	GLN	P	10	20.966	-4.502	20.653	0.00	0.00	PROB
ATOM	5361	HB2	GLN	P	10	19.591	-5.436	19.922	0.00	0.00	PROB
ATOM	5362	CG	GLN	P	10	18.938	-3.651	21.036	0.00	0.00	PROB
ATOM	5363	HG1	GLN	P	10	18.493	-4.129	21.935	0.00	0.00	PROB
ATOM	5364	HG2	GLN	P	10	18.024	-3.423	20.447	0.00	0.00	PROB

ATOM	5365	CD	GLN	P	10	19.455	-2.338	21.684	0.00	0.00 PROB
ATOM	5366	OE1	GLN	P	10	20.186	-2.340	22.705	0.00	0.00 PROB
ATOM	5367	NE2	GLN	P	10	19.134	-1.170	21.098	0.00	0.00 PROB
ATOM	5368	HE21	GLN	P	10	18.395	-1.143	20.425	0.00	0.00 PROB
ATOM	5369	HE22	GLN	P	10	19.214	-0.302	21.589	0.00	0.00 PROB
ATOM	5370	C	GLN	P	10	21.567	-4.366	18.265	0.00	0.00 PROB
ATOM	5371	O	GLN	P	10	21.589	-5.414	17.628	0.00	0.00 PROB
ATOM	5372	N	VAL	P	11	22.732	-3.650	18.472	0.00	0.00 PROB
ATOM	5373	HN	VAL	P	11	22.745	-2.820	19.023	0.00	0.00 PROB
ATOM	5374	CA	VAL	P	11	23.982	-4.191	17.943	0.00	0.00 PROB
ATOM	5375	HA	VAL	P	11	23.845	-4.474	16.910	0.00	0.00 PROB
ATOM	5376	CB	VAL	P	11	25.031	-3.112	17.952	0.00	0.00 PROB
ATOM	5377	HB	VAL	P	11	25.939	-3.494	17.438	0.00	0.00 PROB
ATOM	5378	CG1	VAL	P	11	24.687	-1.914	17.026	0.00	0.00 PROB
ATOM	5379	HG11	VAL	P	11	25.158	-0.956	17.336	0.00	0.00 PROB
ATOM	5380	HG12	VAL	P	11	23.583	-1.816	16.952	0.00	0.00 PROB
ATOM	5381	HG13	VAL	P	11	24.968	-2.037	15.959	0.00	0.00 PROB
ATOM	5382	CG2	VAL	P	11	25.416	-2.577	19.371	0.00	0.00 PROB
ATOM	5383	HG21	VAL	P	11	24.552	-2.497	20.066	0.00	0.00 PROB
ATOM	5384	HG22	VAL	P	11	25.812	-1.557	19.183	0.00	0.00 PROB
ATOM	5385	HG23	VAL	P	11	26.251	-3.142	19.839	0.00	0.00 PROB
ATOM	5386	C	VAL	P	11	24.447	-5.437	18.799	0.00	0.00 PROB
ATOM	5387	O	VAL	P	11	25.217	-6.252	18.242	0.00	0.00 PROB
ATOM	5388	N	SER	P	12	23.829	-5.606	19.994	0.00	0.00 PROB
ATOM	5389	HN	SER	P	12	23.296	-4.876	20.415	0.00	0.00 PROB
ATOM	5390	CA	SER	P	12	24.179	-6.783	20.907	0.00	0.00 PROB
ATOM	5391	HA	SER	P	12	24.523	-7.614	20.309	0.00	0.00 PROB
ATOM	5392	CB	SER	P	12	25.290	-6.410	21.995	0.00	0.00 PROB
ATOM	5393	HB1	SER	P	12	24.715	-5.661	22.581	0.00	0.00 PROB
ATOM	5394	HB2	SER	P	12	26.166	-5.983	21.462	0.00	0.00 PROB
ATOM	5395	OG	SER	P	12	25.717	-7.494	22.842	0.00	0.00 PROB
ATOM	5396	HG1	SER	P	12	26.075	-8.213	22.316	0.00	0.00 PROB
ATOM	5397	C	SER	P	12	22.879	-7.147	21.549	0.00	0.00 PROB
ATOM	5398	O	SER	P	12	22.134	-6.366	22.187	0.00	0.00 PROB
ATOM	5399	N	THR	P	13	22.475	-8.406	21.378	0.00	0.00 PROB
ATOM	5400	HN	THR	P	13	23.148	-8.993	20.934	0.00	0.00 PROB
ATOM	5401	CA	THR	P	13	21.180	-8.946	21.822	0.00	0.00 PROB
ATOM	5402	HA	THR	P	13	20.885	-8.405	22.709	0.00	0.00 PROB
ATOM	5403	CB	THR	P	13	20.051	-8.598	20.835	0.00	0.00 PROB
ATOM	5404	HB	THR	P	13	19.984	-7.513	20.606	0.00	0.00 PROB
ATOM	5405	OG1	THR	P	13	18.784	-9.057	21.289	0.00	0.00 PROB
ATOM	5406	HG1	THR	P	13	18.315	-8.317	21.682	0.00	0.00 PROB
ATOM	5407	CG2	THR	P	13	20.166	-9.273	19.531	0.00	0.00 PROB
ATOM	5408	HG21	THR	P	13	21.177	-9.087	19.110	0.00	0.00 PROB
ATOM	5409	HG22	THR	P	13	19.405	-9.150	18.731	0.00	0.00 PROB
ATOM	5410	HG23	THR	P	13	20.236	-10.380	19.590	0.00	0.00 PROB
ATOM	5411	C	THR	P	13	21.249	-10.481	21.910	0.00	0.00 PROB
ATOM	5412	O	THR	P	13	21.995	-11.136	21.178	0.00	0.00 PROB
ATOM	5413	N	PRO	P	14	20.579	-11.260	22.768	0.00	0.00 PROB
ATOM	5414	CD	PRO	P	14	19.850	-10.735	23.965	0.00	0.00 PROB
ATOM	5415	HD1	PRO	P	14	18.971	-10.191	23.558	0.00	0.00 PROB
ATOM	5416	HD2	PRO	P	14	20.409	-9.998	24.580	0.00	0.00 PROB
ATOM	5417	CA	PRO	P	14	20.664	-12.765	22.737	0.00	0.00 PROB
ATOM	5418	HA	PRO	P	14	21.716	-12.999	22.806	0.00	0.00 PROB
ATOM	5419	CB	PRO	P	14	19.705	-13.178	23.859	0.00	0.00 PROB
ATOM	5420	HB1	PRO	P	14	20.072	-14.002	24.506	0.00	0.00 PROB
ATOM	5421	HB2	PRO	P	14	18.679	-13.320	23.455	0.00	0.00 PROB
ATOM	5422	CG	PRO	P	14	19.647	-11.982	24.848	0.00	0.00 PROB
ATOM	5423	HG1	PRO	P	14	18.694	-12.002	25.418	0.00	0.00 PROB
ATOM	5424	HG2	PRO	P	14	20.515	-12.229	25.496	0.00	0.00 PROB
ATOM	5425	C	PRO	P	14	20.226	-13.296	21.394	0.00	0.00 PROB

```
ATOM   5426  O   PRO P  14      19.290 -12.736  20.831  0.00  0.00      PROB
ATOM   5427  N   THR P  15      20.886 -14.377  20.865  0.00  0.00      PROB
ATOM   5428  HN  THR P  15      21.717 -14.719  21.298  0.00  0.00      PROB
ATOM   5429  CA  THR P  15      20.455 -15.114  19.636  0.00  0.00      PROB
ATOM   5430  HA  THR P  15      20.579 -14.380  18.853  0.00  0.00      PROB
ATOM   5431  CB  THR P  15      21.344 -16.232  19.222  0.00  0.00      PROB
ATOM   5432  HB  THR P  15      22.395 -15.874  19.183  0.00  0.00      PROB
ATOM   5433  OG1 THR P  15      21.091 -16.675  17.884  0.00  0.00      PROB
ATOM   5434  HG1 THR P  15      21.030 -15.904  17.315  0.00  0.00      PROB
ATOM   5435  CG2 THR P  15      21.354 -17.538  20.057  0.00  0.00      PROB
ATOM   5436 HG21 THR P  15      20.373 -18.048  19.955  0.00  0.00      PROB
ATOM   5437 HG22 THR P  15      21.519 -17.341  21.138  0.00  0.00      PROB
ATOM   5438 HG23 THR P  15      22.175 -18.205  19.717  0.00  0.00      PROB
ATOM   5439  C   THR P  15      18.997 -15.507  19.593  0.00  0.00      PROB
ATOM   5440  O   THR P  15      18.441 -15.977  20.628  0.00  0.00      PROB
ATOM   5441  N   LEU P  16      18.361 -15.308  18.416  0.00  0.00      PROB
ATOM   5442  HN  LEU P  16      18.884 -14.973  17.635  0.00  0.00      PROB
ATOM   5443  CA  LEU P  16      16.882 -15.500  18.328  0.00  0.00      PROB
ATOM   5444  HA  LEU P  16      16.605 -16.271  19.031  0.00  0.00      PROB
ATOM   5445  CB  LEU P  16      16.051 -14.288  18.636  0.00  0.00      PROB
ATOM   5446  HB1 LEU P  16      16.304 -13.475  17.923  0.00  0.00      PROB
ATOM   5447  HB2 LEU P  16      16.364 -13.974  19.654  0.00  0.00      PROB
ATOM   5448  CG  LEU P  16      14.522 -14.436  18.510  0.00  0.00      PROB
ATOM   5449  HG  LEU P  16      14.291 -14.871  17.515  0.00  0.00      PROB
ATOM   5450  CD1 LEU P  16      13.926 -15.350  19.594  0.00  0.00      PROB
ATOM   5451 HD11 LEU P  16      12.821 -15.241  19.624  0.00  0.00      PROB
ATOM   5452 HD12 LEU P  16      14.390 -15.193  20.591  0.00  0.00      PROB
ATOM   5453 HD13 LEU P  16      14.142 -16.414  19.359  0.00  0.00      PROB
ATOM   5454  CD2 LEU P  16      13.857 -13.000  18.583  0.00  0.00      PROB
ATOM   5455 HD21 LEU P  16      14.105 -12.537  19.562  0.00  0.00      PROB
ATOM   5456 HD22 LEU P  16      12.768 -13.140  18.412  0.00  0.00      PROB
ATOM   5457 HD23 LEU P  16      14.419 -12.416  17.824  0.00  0.00      PROB
ATOM   5458  C   LEU P  16      16.545 -16.074  16.947  0.00  0.00      PROB
ATOM   5459  OT1 LEU P  16      16.141 -17.256  16.872  0.00  0.00      PROB
ATOM   5460  OT2 LEU P  16      16.555 -15.337  15.970  0.00  0.00      PROB
END
```

5. A representative structure from the highly populated cluster of CXCR4/JM#21

```
CRYST1   93.956   93.956  178.124  90.00  90.00  90.00 P 1           1
ATOM      1  N   MET A   1      18.890  67.460  44.510  0.00  0.00
ATOM      2  HT1 MET A   1      18.440  66.520  44.470  0.00  0.00
ATOM      3  HT2 MET A   1      19.850  67.190  44.200  0.00  0.00
ATOM      4  HT3 MET A   1      18.560  68.090  43.750  0.00  0.00
ATOM      5  CA  MET A   1      18.990  68.240  45.740  0.00  0.00
ATOM      6  HA  MET A   1      19.840  68.910  45.740  0.00  0.00
ATOM      7  CB  MET A   1      17.630  68.930  46.040  0.00  0.00
ATOM      8  HB1 MET A   1      17.210  69.500  45.180  0.00  0.00
ATOM      9  HB2 MET A   1      17.860  69.780  46.730  0.00  0.00
ATOM     10  CG  MET A   1      16.410  68.100  46.600  0.00  0.00
ATOM     11  HG1 MET A   1      15.560  68.760  46.890  0.00  0.00
ATOM     12  HG2 MET A   1      16.730  67.520  47.490  0.00  0.00
ATOM     13  SD  MET A   1      15.710  67.030  45.330  0.00  0.00
ATOM     14  CE  MET A   1      14.460  66.060  46.150  0.00  0.00
ATOM     15  HE1 MET A   1      13.620  66.730  46.430  0.00  0.00
ATOM     16  HE2 MET A   1      14.840  65.470  47.010  0.00  0.00
ATOM     17  HE3 MET A   1      14.040  65.310  45.440  0.00  0.00
ATOM     18  C   MET A   1      19.320  67.210  46.810  0.00  0.00
ATOM     19  O   MET A   1      19.090  65.990  46.660  0.00  0.00
ATOM     20  N   GLU A   2      19.930  67.640  47.970  0.00  0.00
ATOM     21  HN  GLU A   2      20.320  68.550  48.080  0.00  0.00
ATOM     22  CA  GLU A   2      20.190  66.780  49.130  0.00  0.00
ATOM     23  HA  GLU A   2      20.710  65.940  48.720  0.00  0.00
ATOM     24  CB  GLU A   2      21.080  67.540  50.200  0.00  0.00
ATOM     25  HB1 GLU A   2      21.220  66.830  51.040  0.00  0.00
ATOM     26  HB2 GLU A   2      20.460  68.380  50.590  0.00  0.00
ATOM     27  CG  GLU A   2      22.330  68.120  49.580  0.00  0.00
ATOM     28  HG1 GLU A   2      22.230  69.060  48.990  0.00  0.00
ATOM     29  HG2 GLU A   2      22.730  67.340  48.900  0.00  0.00
ATOM     30  CD  GLU A   2      23.260  68.330  50.700  0.00  0.00
ATOM     31  OE1 GLU A   2      23.800  67.400  51.340  0.00  0.00
ATOM     32  OE2 GLU A   2      23.500  69.600  50.940  0.00  0.00
ATOM     33  C   GLU A   2      19.020  66.100  49.870  0.00  0.00
ATOM     34  O   GLU A   2      17.900  66.560  49.870  0.00  0.00
ATOM     35  N   GLY A   3      19.290  64.950  50.560  0.00  0.00
ATOM     36  HN  GLY A   3      20.190  64.540  50.410  0.00  0.00
ATOM     37  CA  GLY A   3      18.330  64.210  51.360  0.00  0.00
ATOM     38  HA1 GLY A   3      18.770  63.300  51.750  0.00  0.00
ATOM     39  HA2 GLY A   3      17.460  64.010  50.750  0.00  0.00
ATOM     40  C   GLY A   3      18.000  64.890  52.700  0.00  0.00
ATOM     41  O   GLY A   3      17.750  66.070  52.750  0.00  0.00
ATOM     42  N   ILE A   4      18.030  64.190  53.840  0.00  0.00
ATOM     43  HN  ILE A   4      18.210  63.210  53.800  0.00  0.00
ATOM     44  CA  ILE A   4      17.720  64.710  55.160  0.00  0.00
ATOM     45  HA  ILE A   4      17.410  65.740  55.070  0.00  0.00
ATOM     46  CB  ILE A   4      16.640  63.770  55.740  0.00  0.00
ATOM     47  HB  ILE A   4      16.060  63.440  54.860  0.00  0.00
ATOM     48  CG2 ILE A   4      17.080  62.340  56.400  0.00  0.00
ATOM     49 HG21 ILE A   4      17.800  62.670  57.190  0.00  0.00
ATOM     50 HG22 ILE A   4      17.550  61.570  55.760  0.00  0.00
ATOM     51 HG23 ILE A   4      16.220  61.850  56.890  0.00  0.00
ATOM     52  CG1 ILE A   4      15.800  64.640  56.650  0.00  0.00
ATOM     53 HG11 ILE A   4      15.460  65.530  56.080  0.00  0.00
ATOM     54 HG12 ILE A   4      16.480  64.970  57.460  0.00  0.00
ATOM     55  CD  ILE A   4      14.550  63.820  57.260  0.00  0.00
ATOM     56  HD1 ILE A   4      13.720  64.440  57.670  0.00  0.00
```

ATOM	57	HD2	ILE	A	4	14.990	63.090	57.970	0.00	0.00
ATOM	58	HD3	ILE	A	4	14.090	63.220	56.440	0.00	0.00
ATOM	59	C	ILE	A	4	18.990	64.800	55.940	0.00	0.00
ATOM	60	O	ILE	A	4	19.700	63.840	56.170	0.00	0.00
ATOM	61	N	SER	A	5	19.410	66.080	56.310	0.00	0.00
ATOM	62	HN	SER	A	5	18.800	66.870	56.230	0.00	0.00
ATOM	63	CA	SER	A	5	20.780	66.370	56.850	0.00	0.00
ATOM	64	HA	SER	A	5	21.510	65.950	56.170	0.00	0.00
ATOM	65	CB	SER	A	5	21.060	67.860	57.080	0.00	0.00
ATOM	66	HB1	SER	A	5	20.500	68.430	57.850	0.00	0.00
ATOM	67	HB2	SER	A	5	20.750	68.400	56.160	0.00	0.00
ATOM	68	OG	SER	A	5	22.460	68.210	57.260	0.00	0.00
ATOM	69	HG1	SER	A	5	22.900	68.150	56.410	0.00	0.00
ATOM	70	C	SER	A	5	21.000	65.770	58.240	0.00	0.00
ATOM	71	O	SER	A	5	20.040	65.690	59.020	0.00	0.00
ATOM	72	N	ILE	A	6	22.270	65.290	58.500	0.00	0.00
ATOM	73	HN	ILE	A	6	23.040	65.450	57.890	0.00	0.00
ATOM	74	CA	ILE	A	6	22.720	64.440	59.640	0.00	0.00
ATOM	75	HA	ILE	A	6	21.900	63.800	59.950	0.00	0.00
ATOM	76	CB	ILE	A	6	23.800	63.440	59.170	0.00	0.00
ATOM	77	HB	ILE	A	6	24.520	64.100	58.650	0.00	0.00
ATOM	78	CG2	ILE	A	6	24.330	62.570	60.280	0.00	0.00
ATOM	79	HG21	ILE	A	6	23.560	61.920	60.740	0.00	0.00
ATOM	80	HG22	ILE	A	6	24.810	63.160	61.090	0.00	0.00
ATOM	81	HG23	ILE	A	6	25.130	61.930	59.850	0.00	0.00
ATOM	82	CG1	ILE	A	6	23.320	62.490	58.050	0.00	0.00
ATOM	83	HG11	ILE	A	6	22.390	62.910	57.620	0.00	0.00
ATOM	84	HG12	ILE	A	6	23.020	61.460	58.310	0.00	0.00
ATOM	85	CD	ILE	A	6	24.310	62.400	56.890	0.00	0.00
ATOM	86	HD1	ILE	A	6	24.870	63.360	56.830	0.00	0.00
ATOM	87	HD2	ILE	A	6	23.710	62.160	55.980	0.00	0.00
ATOM	88	HD3	ILE	A	6	25.080	61.610	56.990	0.00	0.00
ATOM	89	C	ILE	A	6	22.940	65.270	60.900	0.00	0.00
ATOM	90	O	ILE	A	6	23.770	66.220	60.970	0.00	0.00
ATOM	91	N	TYR	A	7	22.180	64.900	61.930	0.00	0.00
ATOM	92	HN	TYR	A	7	21.460	64.220	61.820	0.00	0.00
ATOM	93	CA	TYR	A	7	22.220	65.670	63.200	0.00	0.00
ATOM	94	HA	TYR	A	7	22.990	66.440	63.150	0.00	0.00
ATOM	95	CB	TYR	A	7	20.870	66.170	63.580	0.00	0.00
ATOM	96	HB1	TYR	A	7	20.860	66.830	64.460	0.00	0.00
ATOM	97	HB2	TYR	A	7	20.190	65.290	63.650	0.00	0.00
ATOM	98	CG	TYR	A	7	20.240	67.060	62.460	0.00	0.00
ATOM	99	CD1	TYR	A	7	18.860	67.100	62.340	0.00	0.00
ATOM	100	HD1	TYR	A	7	18.330	66.390	62.960	0.00	0.00
ATOM	101	CE1	TYR	A	7	18.240	67.700	61.290	0.00	0.00
ATOM	102	HE1	TYR	A	7	17.180	67.540	61.140	0.00	0.00
ATOM	103	CZ	TYR	A	7	19.020	68.540	60.520	0.00	0.00
ATOM	104	OH	TYR	A	7	18.330	69.350	59.540	0.00	0.00
ATOM	105	HH	TYR	A	7	17.600	68.800	59.240	0.00	0.00
ATOM	106	CD2	TYR	A	7	21.060	67.900	61.700	0.00	0.00
ATOM	107	HD2	TYR	A	7	22.090	68.010	61.980	0.00	0.00
ATOM	108	CE2	TYR	A	7	20.420	68.650	60.720	0.00	0.00
ATOM	109	HE2	TYR	A	7	20.830	69.430	60.100	0.00	0.00
ATOM	110	C	TYR	A	7	22.690	64.840	64.350	0.00	0.00
ATOM	111	O	TYR	A	7	23.000	65.310	65.460	0.00	0.00
ATOM	112	N	THR	A	8	22.890	63.520	64.120	0.00	0.00
ATOM	113	HN	THR	A	8	22.530	63.150	63.270	0.00	0.00
ATOM	114	CA	THR	A	8	23.360	62.580	65.160	0.00	0.00
ATOM	115	HA	THR	A	8	23.940	63.130	65.890	0.00	0.00
ATOM	116	CB	THR	A	8	22.070	62.140	65.940	0.00	0.00
ATOM	117	HB	THR	A	8	21.480	63.020	66.270	0.00	0.00

ATOM	118	OG1	THR	A	8	22.510	61.380	67.050	0.00	0.00
ATOM	119	HG1	THR	A	8	21.660	61.150	67.420	0.00	0.00
ATOM	120	CG2	THR	A	8	21.240	61.250	65.090	0.00	0.00
ATOM	121	HG21	THR	A	8	20.880	61.710	64.150	0.00	0.00
ATOM	122	HG22	THR	A	8	20.280	61.040	65.610	0.00	0.00
ATOM	123	HG23	THR	A	8	21.800	60.340	64.800	0.00	0.00
ATOM	124	C	THR	A	8	24.230	61.510	64.630	0.00	0.00
ATOM	125	O	THR	A	8	24.330	61.280	63.420	0.00	0.00
ATOM	126	N	SER	A	9	24.920	60.870	65.510	0.00	0.00
ATOM	127	HN	SER	A	9	24.930	61.160	66.470	0.00	0.00
ATOM	128	CA	SER	A	9	25.650	59.650	65.160	0.00	0.00
ATOM	129	HA	SER	A	9	25.390	59.340	64.160	0.00	0.00
ATOM	130	CB	SER	A	9	27.190	60.050	65.110	0.00	0.00
ATOM	131	HB1	SER	A	9	27.500	60.400	66.120	0.00	0.00
ATOM	132	HB2	SER	A	9	27.280	60.800	64.290	0.00	0.00
ATOM	133	OG	SER	A	9	28.030	58.950	64.730	0.00	0.00
ATOM	134	HG1	SER	A	9	28.580	58.940	65.520	0.00	0.00
ATOM	135	C	SER	A	9	25.370	58.490	66.060	0.00	0.00
ATOM	136	O	SER	A	9	26.320	57.800	66.380	0.00	0.00
ATOM	137	N	ASP	A	10	24.120	58.240	66.540	0.00	0.00
ATOM	138	HN	ASP	A	10	23.450	58.970	66.400	0.00	0.00
ATOM	139	CA	ASP	A	10	23.750	57.120	67.320	0.00	0.00
ATOM	140	HA	ASP	A	10	24.520	56.830	68.020	0.00	0.00
ATOM	141	CB	ASP	A	10	22.380	57.270	68.040	0.00	0.00
ATOM	142	HB1	ASP	A	10	22.050	56.340	68.560	0.00	0.00
ATOM	143	HB2	ASP	A	10	21.540	57.490	67.350	0.00	0.00
ATOM	144	CG	ASP	A	10	22.370	58.360	69.100	0.00	0.00
ATOM	145	OD1	ASP	A	10	21.260	58.570	69.650	0.00	0.00
ATOM	146	OD2	ASP	A	10	23.410	58.960	69.440	0.00	0.00
ATOM	147	C	ASP	A	10	23.730	55.920	66.440	0.00	0.00
ATOM	148	O	ASP	A	10	23.440	55.980	65.250	0.00	0.00
ATOM	149	N	ASN	A	11	24.020	54.800	67.060	0.00	0.00
ATOM	150	HN	ASN	A	11	24.200	54.920	68.040	0.00	0.00
ATOM	151	CA	ASN	A	11	23.970	53.480	66.500	0.00	0.00
ATOM	152	HA	ASN	A	11	24.340	53.670	65.500	0.00	0.00
ATOM	153	CB	ASN	A	11	24.990	52.630	67.380	0.00	0.00
ATOM	154	HB1	ASN	A	11	24.510	52.520	68.370	0.00	0.00
ATOM	155	HB2	ASN	A	11	25.970	53.130	67.360	0.00	0.00
ATOM	156	CG	ASN	A	11	25.210	51.270	66.740	0.00	0.00
ATOM	157	OD1	ASN	A	11	24.660	51.000	65.720	0.00	0.00
ATOM	158	ND2	ASN	A	11	26.130	50.460	67.220	0.00	0.00
ATOM	159	HD21	ASN	A	11	26.290	49.620	66.690	0.00	0.00
ATOM	160	HD22	ASN	A	11	26.740	50.820	67.920	0.00	0.00
ATOM	161	C	ASN	A	11	22.570	52.880	66.300	0.00	0.00
ATOM	162	O	ASN	A	11	21.770	52.760	67.210	0.00	0.00
ATOM	163	N	TYR	A	12	22.150	52.700	64.990	0.00	0.00
ATOM	164	HN	TYR	A	12	22.690	53.190	64.310	0.00	0.00
ATOM	165	CA	TYR	A	12	20.810	52.220	64.510	0.00	0.00
ATOM	166	HA	TYR	A	12	20.180	52.160	65.380	0.00	0.00
ATOM	167	CB	TYR	A	12	20.240	53.190	63.430	0.00	0.00
ATOM	168	HB1	TYR	A	12	19.200	52.960	63.120	0.00	0.00
ATOM	169	HB2	TYR	A	12	20.940	53.220	62.570	0.00	0.00
ATOM	170	CG	TYR	A	12	20.100	54.570	63.900	0.00	0.00
ATOM	171	CD1	TYR	A	12	19.570	54.880	65.160	0.00	0.00
ATOM	172	HD1	TYR	A	12	19.270	54.080	65.820	0.00	0.00
ATOM	173	CE1	TYR	A	12	19.450	56.170	65.600	0.00	0.00
ATOM	174	HE1	TYR	A	12	19.140	56.300	66.620	0.00	0.00
ATOM	175	CZ	TYR	A	12	19.800	57.220	64.710	0.00	0.00
ATOM	176	OH	TYR	A	12	19.850	58.550	65.290	0.00	0.00
ATOM	177	HH	TYR	A	12	19.720	59.160	64.560	0.00	0.00
ATOM	178	CD2	TYR	A	12	20.440	55.640	63.020	0.00	0.00

ATOM	179	HD2	TYR	A	12	20.720	55.380	62.010	0.00	0.00
ATOM	180	CE2	TYR	A	12	20.260	56.930	63.470	0.00	0.00
ATOM	181	HE2	TYR	A	12	20.620	57.680	62.780	0.00	0.00
ATOM	182	C	TYR	A	12	20.930	50.780	64.040	0.00	0.00
ATOM	183	O	TYR	A	12	20.090	50.300	63.280	0.00	0.00
ATOM	184	N	THR	A	13	21.930	49.990	64.520	0.00	0.00
ATOM	185	HN	THR	A	13	22.610	50.320	65.170	0.00	0.00
ATOM	186	CA	THR	A	13	22.040	48.560	64.280	0.00	0.00
ATOM	187	HA	THR	A	13	21.980	48.470	63.210	0.00	0.00
ATOM	188	CB	THR	A	13	23.350	47.900	64.790	0.00	0.00
ATOM	189	HB	THR	A	13	23.170	46.810	64.890	0.00	0.00
ATOM	190	OG1	THR	A	13	23.930	48.360	65.950	0.00	0.00
ATOM	191	HG1	THR	A	13	24.310	49.160	65.580	0.00	0.00
ATOM	192	CG2	THR	A	13	24.390	48.070	63.650	0.00	0.00
ATOM	193	HG21	THR	A	13	24.600	49.140	63.450	0.00	0.00
ATOM	194	HG22	THR	A	13	24.150	47.510	62.720	0.00	0.00
ATOM	195	HG23	THR	A	13	25.330	47.690	64.100	0.00	0.00
ATOM	196	C	THR	A	13	20.790	47.850	64.850	0.00	0.00
ATOM	197	O	THR	A	13	20.320	48.180	65.950	0.00	0.00
ATOM	198	N	GLU	A	14	20.220	46.850	64.170	0.00	0.00
ATOM	199	HN	GLU	A	14	20.530	46.700	63.240	0.00	0.00
ATOM	200	CA	GLU	A	14	19.140	45.950	64.550	0.00	0.00
ATOM	201	HA	GLU	A	14	18.800	46.200	65.550	0.00	0.00
ATOM	202	CB	GLU	A	14	17.970	45.980	63.480	0.00	0.00
ATOM	203	HB1	GLU	A	14	17.800	47.080	63.430	0.00	0.00
ATOM	204	HB2	GLU	A	14	17.060	45.540	63.940	0.00	0.00
ATOM	205	CG	GLU	A	14	18.370	45.410	62.130	0.00	0.00
ATOM	206	HG1	GLU	A	14	18.430	44.310	62.260	0.00	0.00
ATOM	207	HG2	GLU	A	14	19.390	45.700	61.800	0.00	0.00
ATOM	208	CD	GLU	A	14	17.430	45.840	61.030	0.00	0.00
ATOM	209	OE1	GLU	A	14	16.620	45.040	60.560	0.00	0.00
ATOM	210	OE2	GLU	A	14	17.560	47.040	60.640	0.00	0.00
ATOM	211	C	GLU	A	14	19.620	44.440	64.710	0.00	0.00
ATOM	212	O	GLU	A	14	18.830	43.530	64.960	0.00	0.00
ATOM	213	N	GLU	A	15	20.930	44.160	64.630	0.00	0.00
ATOM	214	HN	GLU	A	15	21.600	44.800	64.270	0.00	0.00
ATOM	215	CA	GLU	A	15	21.550	42.900	65.010	0.00	0.00
ATOM	216	HA	GLU	A	15	20.790	42.240	65.410	0.00	0.00
ATOM	217	CB	GLU	A	15	22.210	42.140	63.810	0.00	0.00
ATOM	218	HB1	GLU	A	15	22.960	41.410	64.200	0.00	0.00
ATOM	219	HB2	GLU	A	15	22.740	42.890	63.190	0.00	0.00
ATOM	220	CG	GLU	A	15	21.200	41.420	62.930	0.00	0.00
ATOM	221	HG1	GLU	A	15	20.650	42.170	62.320	0.00	0.00
ATOM	222	HG2	GLU	A	15	20.480	40.840	63.560	0.00	0.00
ATOM	223	CD	GLU	A	15	21.920	40.530	61.890	0.00	0.00
ATOM	224	OE1	GLU	A	15	22.050	40.890	60.680	0.00	0.00
ATOM	225	OE2	GLU	A	15	22.380	39.430	62.360	0.00	0.00
ATOM	226	C	GLU	A	15	22.510	43.210	66.070	0.00	0.00
ATOM	227	O	GLU	A	15	23.350	44.090	65.780	0.00	0.00
ATOM	228	N	MET	A	16	22.470	42.520	67.220	0.00	0.00
ATOM	229	HN	MET	A	16	21.720	41.870	67.360	0.00	0.00
ATOM	230	CA	MET	A	16	23.440	42.570	68.200	0.00	0.00
ATOM	231	HA	MET	A	16	23.580	43.610	68.460	0.00	0.00
ATOM	232	CB	MET	A	16	22.880	41.860	69.600	0.00	0.00
ATOM	233	HB1	MET	A	16	23.810	41.790	70.210	0.00	0.00
ATOM	234	HB2	MET	A	16	22.550	40.850	69.290	0.00	0.00
ATOM	235	CG	MET	A	16	21.640	42.570	70.350	0.00	0.00
ATOM	236	HG1	MET	A	16	21.350	41.850	71.140	0.00	0.00
ATOM	237	HG2	MET	A	16	20.800	42.610	69.620	0.00	0.00
ATOM	238	SD	MET	A	16	22.010	44.200	71.010	0.00	0.00
ATOM	239	CE	MET	A	16	22.960	43.410	72.450	0.00	0.00

ATOM	240	HE1	MET	A	16	22.560	42.460	72.870	0.00	0.00
ATOM	241	HE2	MET	A	16	22.980	44.080	73.330	0.00	0.00
ATOM	242	HE3	MET	A	16	24.000	43.280	72.090	0.00	0.00
ATOM	243	C	MET	A	16	24.810	41.980	67.790	0.00	0.00
ATOM	244	O	MET	A	16	24.960	40.890	67.300	0.00	0.00
ATOM	245	N	GLY	A	17	25.870	42.780	68.190	0.00	0.00
ATOM	246	HN	GLY	A	17	25.720	43.650	68.650	0.00	0.00
ATOM	247	CA	GLY	A	17	27.270	42.490	67.990	0.00	0.00
ATOM	248	HA1	GLY	A	17	27.350	41.490	68.390	0.00	0.00
ATOM	249	HA2	GLY	A	17	27.840	43.270	68.460	0.00	0.00
ATOM	250	C	GLY	A	17	27.750	42.370	66.520	0.00	0.00
ATOM	251	O	GLY	A	17	27.130	42.840	65.560	0.00	0.00
ATOM	252	N	SER	A	18	28.940	41.740	66.350	0.00	0.00
ATOM	253	HN	SER	A	18	29.390	41.190	67.040	0.00	0.00
ATOM	254	CA	SER	A	18	29.510	41.800	65.050	0.00	0.00
ATOM	255	HA	SER	A	18	29.010	42.420	64.320	0.00	0.00
ATOM	256	CB	SER	A	18	31.000	42.100	65.250	0.00	0.00
ATOM	257	HB1	SER	A	18	31.550	42.130	64.280	0.00	0.00
ATOM	258	HB2	SER	A	18	31.520	41.290	65.810	0.00	0.00
ATOM	259	OG	SER	A	18	31.260	43.290	65.930	0.00	0.00
ATOM	260	HG1	SER	A	18	30.930	43.320	66.830	0.00	0.00
ATOM	261	C	SER	A	18	29.380	40.400	64.540	0.00	0.00
ATOM	262	O	SER	A	18	28.710	39.580	65.220	0.00	0.00
ATOM	263	N	GLY	A	19	29.920	40.070	63.320	0.00	0.00
ATOM	264	HN	GLY	A	19	30.290	40.860	62.840	0.00	0.00
ATOM	265	CA	GLY	A	19	29.990	38.810	62.560	0.00	0.00
ATOM	266	HA1	GLY	A	19	30.430	38.840	61.580	0.00	0.00
ATOM	267	HA2	GLY	A	19	29.000	38.380	62.640	0.00	0.00
ATOM	268	C	GLY	A	19	30.940	37.860	63.280	0.00	0.00
ATOM	269	O	GLY	A	19	30.460	36.750	63.650	0.00	0.00
ATOM	270	N	ASP	A	20	32.090	38.350	63.720	0.00	0.00
ATOM	271	HN	ASP	A	20	32.340	39.270	63.440	0.00	0.00
ATOM	272	CA	ASP	A	20	33.090	37.480	64.380	0.00	0.00
ATOM	273	HA	ASP	A	20	32.640	36.830	65.120	0.00	0.00
ATOM	274	CB	ASP	A	20	33.840	36.610	63.340	0.00	0.00
ATOM	275	HB1	ASP	A	20	33.260	35.720	63.040	0.00	0.00
ATOM	276	HB2	ASP	A	20	34.850	36.230	63.650	0.00	0.00
ATOM	277	CG	ASP	A	20	34.190	37.270	62.020	0.00	0.00
ATOM	278	OD1	ASP	A	20	34.520	36.420	61.090	0.00	0.00
ATOM	279	OD2	ASP	A	20	34.370	38.480	61.920	0.00	0.00
ATOM	280	C	ASP	A	20	33.940	38.420	65.350	0.00	0.00
ATOM	281	O	ASP	A	20	33.550	38.530	66.540	0.00	0.00
ATOM	282	N	TYR	A	21	35.010	39.000	64.860	0.00	0.00
ATOM	283	HN	TYR	A	21	35.270	38.780	63.930	0.00	0.00
ATOM	284	CA	TYR	A	21	35.820	40.010	65.460	0.00	0.00
ATOM	285	HA	TYR	A	21	35.970	39.720	66.490	0.00	0.00
ATOM	286	CB	TYR	A	21	37.170	39.960	64.710	0.00	0.00
ATOM	287	HB1	TYR	A	21	37.940	40.720	64.970	0.00	0.00
ATOM	288	HB2	TYR	A	21	36.950	40.060	63.620	0.00	0.00
ATOM	289	CG	TYR	A	21	37.800	38.550	65.010	0.00	0.00
ATOM	290	CD1	TYR	A	21	37.940	37.590	64.000	0.00	0.00
ATOM	291	HD1	TYR	A	21	37.520	37.680	63.010	0.00	0.00
ATOM	292	CE1	TYR	A	21	38.530	36.380	64.330	0.00	0.00
ATOM	293	HE1	TYR	A	21	38.620	35.680	63.510	0.00	0.00
ATOM	294	CZ	TYR	A	21	38.970	36.050	65.610	0.00	0.00
ATOM	295	OH	TYR	A	21	39.650	34.800	65.840	0.00	0.00
ATOM	296	HH	TYR	A	21	39.560	34.760	66.800	0.00	0.00
ATOM	297	CD2	TYR	A	21	38.200	38.240	66.320	0.00	0.00
ATOM	298	HD2	TYR	A	21	37.910	38.930	67.100	0.00	0.00
ATOM	299	CE2	TYR	A	21	38.850	37.010	66.630	0.00	0.00
ATOM	300	HE2	TYR	A	21	39.120	36.740	67.640	0.00	0.00

ATOM	301	C	TYR	A	21	35.180	41.430	65.360	0.00	0.00
ATOM	302	O	TYR	A	21	34.320	41.670	64.470	0.00	0.00
ATOM	303	N	ASP	A	22	35.620	42.360	66.290	0.00	0.00
ATOM	304	HN	ASP	A	22	36.350	42.210	66.960	0.00	0.00
ATOM	305	CA	ASP	A	22	35.100	43.680	66.600	0.00	0.00
ATOM	306	HA	ASP	A	22	34.170	43.590	67.140	0.00	0.00
ATOM	307	CB	ASP	A	22	35.930	44.520	67.570	0.00	0.00
ATOM	308	HB1	ASP	A	22	35.370	45.410	67.930	0.00	0.00
ATOM	309	HB2	ASP	A	22	36.940	44.940	67.350	0.00	0.00
ATOM	310	CG	ASP	A	22	36.190	43.760	68.890	0.00	0.00
ATOM	311	OD1	ASP	A	22	35.440	43.900	69.860	0.00	0.00
ATOM	312	OD2	ASP	A	22	37.130	42.870	68.830	0.00	0.00
ATOM	313	C	ASP	A	22	34.910	44.640	65.390	0.00	0.00
ATOM	314	O	ASP	A	22	35.600	44.660	64.370	0.00	0.00
ATOM	315	N	SER	A	23	33.950	45.590	65.600	0.00	0.00
ATOM	316	HN	SER	A	23	33.480	45.610	66.480	0.00	0.00
ATOM	317	CA	SER	A	23	33.370	46.490	64.540	0.00	0.00
ATOM	318	HA	SER	A	23	34.160	46.990	64.000	0.00	0.00
ATOM	319	CB	SER	A	23	32.420	45.830	63.520	0.00	0.00
ATOM	320	HB1	SER	A	23	32.970	44.990	63.040	0.00	0.00
ATOM	321	HB2	SER	A	23	32.050	46.580	62.800	0.00	0.00
ATOM	322	OG	SER	A	23	31.240	45.240	64.120	0.00	0.00
ATOM	323	HG1	SER	A	23	31.520	44.470	64.610	0.00	0.00
ATOM	324	C	SER	A	23	32.630	47.560	65.310	0.00	0.00
ATOM	325	O	SER	A	23	32.370	47.430	66.460	0.00	0.00
ATOM	326	N	MET	A	24	32.530	48.700	64.730	0.00	0.00
ATOM	327	HN	MET	A	24	33.020	48.870	63.880	0.00	0.00
ATOM	328	CA	MET	A	24	31.690	49.810	65.190	0.00	0.00
ATOM	329	HA	MET	A	24	31.700	50.590	64.450	0.00	0.00
ATOM	330	CB	MET	A	24	30.220	49.370	65.460	0.00	0.00
ATOM	331	HB1	MET	A	24	29.610	50.170	65.940	0.00	0.00
ATOM	332	HB2	MET	A	24	30.110	48.570	66.210	0.00	0.00
ATOM	333	CG	MET	A	24	29.580	48.990	64.120	0.00	0.00
ATOM	334	HG1	MET	A	24	28.630	48.480	64.380	0.00	0.00
ATOM	335	HG2	MET	A	24	30.280	48.240	63.680	0.00	0.00
ATOM	336	SD	MET	A	24	29.370	50.280	62.860	0.00	0.00
ATOM	337	CE	MET	A	24	27.640	50.660	63.280	0.00	0.00
ATOM	338	HE1	MET	A	24	27.290	51.510	62.660	0.00	0.00
ATOM	339	HE2	MET	A	24	27.450	50.820	64.360	0.00	0.00
ATOM	340	HE3	MET	A	24	27.020	49.810	62.920	0.00	0.00
ATOM	341	C	MET	A	24	32.280	50.440	66.380	0.00	0.00
ATOM	342	O	MET	A	24	31.580	50.720	67.370	0.00	0.00
ATOM	343	N	LYS	A	25	33.600	50.620	66.380	0.00	0.00
ATOM	344	HN	LYS	A	25	34.140	50.400	65.570	0.00	0.00
ATOM	345	CA	LYS	A	25	34.270	51.260	67.450	0.00	0.00
ATOM	346	HA	LYS	A	25	33.590	51.680	68.170	0.00	0.00
ATOM	347	CB	LYS	A	25	35.170	50.230	68.260	0.00	0.00
ATOM	348	HB1	LYS	A	25	35.830	50.710	69.020	0.00	0.00
ATOM	349	HB2	LYS	A	25	35.830	49.750	67.500	0.00	0.00
ATOM	350	CG	LYS	A	25	34.370	49.200	69.070	0.00	0.00
ATOM	351	HG1	LYS	A	25	33.650	48.680	68.410	0.00	0.00
ATOM	352	HG2	LYS	A	25	33.750	49.670	69.870	0.00	0.00
ATOM	353	CD	LYS	A	25	35.210	48.190	69.810	0.00	0.00
ATOM	354	HD1	LYS	A	25	35.920	48.700	70.490	0.00	0.00
ATOM	355	HD2	LYS	A	25	35.840	47.670	69.050	0.00	0.00
ATOM	356	CE	LYS	A	25	34.350	47.110	70.460	0.00	0.00
ATOM	357	HE1	LYS	A	25	33.670	46.650	69.710	0.00	0.00
ATOM	358	HE2	LYS	A	25	33.770	47.650	71.230	0.00	0.00
ATOM	359	NZ	LYS	A	25	35.150	46.100	71.120	0.00	0.00
ATOM	360	HZ1	LYS	A	25	34.790	46.000	72.090	0.00	0.00
ATOM	361	HZ2	LYS	A	25	36.170	46.240	71.280	0.00	0.00

ATOM	362	HZ3	LYS	A	25	35.020	45.190	70.650	0.00	0.00
ATOM	363	C	LYS	A	25	35.190	52.410	67.000	0.00	0.00
ATOM	364	O	LYS	A	25	36.150	52.220	66.220	0.00	0.00
ATOM	365	N	GLU	A	26	34.860	53.570	67.510	0.00	0.00
ATOM	366	HN	GLU	A	26	34.020	53.620	68.040	0.00	0.00
ATOM	367	CA	GLU	A	26	35.490	54.850	67.330	0.00	0.00
ATOM	368	HA	GLU	A	26	35.530	55.120	66.280	0.00	0.00
ATOM	369	CB	GLU	A	26	34.710	55.950	68.060	0.00	0.00
ATOM	370	HB1	GLU	A	26	35.190	56.950	68.100	0.00	0.00
ATOM	371	HB2	GLU	A	26	34.490	55.630	69.100	0.00	0.00
ATOM	372	CG	GLU	A	26	33.440	56.290	67.190	0.00	0.00
ATOM	373	HG1	GLU	A	26	32.760	55.460	66.890	0.00	0.00
ATOM	374	HG2	GLU	A	26	33.750	56.840	66.280	0.00	0.00
ATOM	375	CD	GLU	A	26	32.540	57.180	68.000	0.00	0.00
ATOM	376	OE1	GLU	A	26	32.180	58.300	67.510	0.00	0.00
ATOM	377	OE2	GLU	A	26	31.970	56.840	69.140	0.00	0.00
ATOM	378	C	GLU	A	26	36.900	54.830	67.940	0.00	0.00
ATOM	379	O	GLU	A	26	37.050	54.330	69.020	0.00	0.00
ATOM	380	N	PRO	A	27	38.000	55.270	67.270	0.00	0.00
ATOM	381	CD	PRO	A	27	38.070	55.450	65.810	0.00	0.00
ATOM	382	HD1	PRO	A	27	37.660	56.460	65.610	0.00	0.00
ATOM	383	HD2	PRO	A	27	37.600	54.670	65.190	0.00	0.00
ATOM	384	CA	PRO	A	27	39.320	55.290	67.820	0.00	0.00
ATOM	385	HA	PRO	A	27	39.640	54.270	67.990	0.00	0.00
ATOM	386	CB	PRO	A	27	40.180	55.960	66.740	0.00	0.00
ATOM	387	HB1	PRO	A	27	41.240	55.660	66.800	0.00	0.00
ATOM	388	HB2	PRO	A	27	40.190	57.060	66.820	0.00	0.00
ATOM	389	CG	PRO	A	27	39.560	55.420	65.430	0.00	0.00
ATOM	390	HG1	PRO	A	27	39.830	56.000	64.520	0.00	0.00
ATOM	391	HG2	PRO	A	27	39.870	54.360	65.300	0.00	0.00
ATOM	392	C	PRO	A	27	39.520	55.940	69.160	0.00	0.00
ATOM	393	O	PRO	A	27	38.900	56.930	69.510	0.00	0.00
ATOM	394	N	CYS	A	28	40.420	55.300	69.920	0.00	0.00
ATOM	395	HN	CYS	A	28	40.690	54.370	69.670	0.00	0.00
ATOM	396	CA	CYS	A	28	40.860	55.790	71.210	0.00	0.00
ATOM	397	HA	CYS	A	28	40.430	56.730	71.520	0.00	0.00
ATOM	398	CB	CYS	A	28	40.500	54.740	72.330	0.00	0.00
ATOM	399	HB1	CYS	A	28	41.090	53.800	72.280	0.00	0.00
ATOM	400	HB2	CYS	A	28	39.400	54.580	72.280	0.00	0.00
ATOM	401	SG	CYS	A	28	40.820	55.350	74.020	0.00	0.00
ATOM	402	C	CYS	A	28	42.350	56.090	71.210	0.00	0.00
ATOM	403	O	CYS	A	28	43.180	55.300	70.760	0.00	0.00
ATOM	404	N	PHE	A	29	42.720	57.330	71.790	0.00	0.00
ATOM	405	HN	PHE	A	29	42.040	57.960	72.150	0.00	0.00
ATOM	406	CA	PHE	A	29	44.060	57.770	72.070	0.00	0.00
ATOM	407	HA	PHE	A	29	44.830	57.210	71.570	0.00	0.00
ATOM	408	CB	PHE	A	29	44.310	59.230	71.600	0.00	0.00
ATOM	409	HB1	PHE	A	29	45.200	59.650	72.100	0.00	0.00
ATOM	410	HB2	PHE	A	29	43.410	59.810	71.890	0.00	0.00
ATOM	411	CG	PHE	A	29	44.420	59.250	70.120	0.00	0.00
ATOM	412	CD1	PHE	A	29	45.680	59.290	69.580	0.00	0.00
ATOM	413	HD1	PHE	A	29	46.520	59.410	70.240	0.00	0.00
ATOM	414	CE1	PHE	A	29	45.790	59.330	68.210	0.00	0.00
ATOM	415	HE1	PHE	A	29	46.780	59.360	67.760	0.00	0.00
ATOM	416	CZ	PHE	A	29	44.660	59.220	67.360	0.00	0.00
ATOM	417	HZ	PHE	A	29	44.800	59.440	66.320	0.00	0.00
ATOM	418	CD2	PHE	A	29	43.270	59.160	69.280	0.00	0.00
ATOM	419	HD2	PHE	A	29	42.300	59.270	69.730	0.00	0.00
ATOM	420	CE2	PHE	A	29	43.420	59.170	67.890	0.00	0.00
ATOM	421	HE2	PHE	A	29	42.540	59.210	67.250	0.00	0.00
ATOM	422	C	PHE	A	29	44.200	57.800	73.660	0.00	0.00

ATOM	423	O	PHE	A	29	43.370	58.370	74.350	0.00	0.00
ATOM	424	N	ARG	A	30	45.160	57.110	74.220	0.00	0.00
ATOM	425	HN	ARG	A	30	45.710	56.460	73.700	0.00	0.00
ATOM	426	CA	ARG	A	30	45.340	56.910	75.640	0.00	0.00
ATOM	427	HA	ARG	A	30	44.430	57.080	76.190	0.00	0.00
ATOM	428	CB	ARG	A	30	45.500	55.410	75.980	0.00	0.00
ATOM	429	HB1	ARG	A	30	45.740	55.260	77.050	0.00	0.00
ATOM	430	HB2	ARG	A	30	46.340	54.980	75.390	0.00	0.00
ATOM	431	CG	ARG	A	30	44.220	54.630	75.640	0.00	0.00
ATOM	432	HG1	ARG	A	30	44.240	54.450	74.550	0.00	0.00
ATOM	433	HG2	ARG	A	30	43.250	55.130	75.870	0.00	0.00
ATOM	434	CD	ARG	A	30	44.260	53.230	76.220	0.00	0.00
ATOM	435	HD1	ARG	A	30	44.450	53.230	77.310	0.00	0.00
ATOM	436	HD2	ARG	A	30	44.980	52.560	75.710	0.00	0.00
ATOM	437	NE	ARG	A	30	42.930	52.530	76.110	0.00	0.00
ATOM	438	HE	ARG	A	30	42.140	53.030	76.460	0.00	0.00
ATOM	439	CZ	ARG	A	30	42.700	51.480	75.390	0.00	0.00
ATOM	440	NH1	ARG	A	30	43.570	50.710	74.760	0.00	0.00
ATOM	441	HH11	ARG	A	30	44.560	50.820	74.860	0.00	0.00
ATOM	442	HH12	ARG	A	30	43.160	49.890	74.370	0.00	0.00
ATOM	443	NH2	ARG	A	30	41.400	51.140	75.240	0.00	0.00
ATOM	444	HH21	ARG	A	30	40.770	51.830	75.590	0.00	0.00
ATOM	445	HH22	ARG	A	30	41.150	50.370	74.640	0.00	0.00
ATOM	446	C	ARG	A	30	46.450	57.820	76.160	0.00	0.00
ATOM	447	O	ARG	A	30	46.810	57.740	77.320	0.00	0.00
ATOM	448	N	GLU	A	31	46.950	58.720	75.250	0.00	0.00
ATOM	449	HN	GLU	A	31	46.720	58.720	74.280	0.00	0.00
ATOM	450	CA	GLU	A	31	48.090	59.540	75.510	0.00	0.00
ATOM	451	HA	GLU	A	31	48.900	58.870	75.770	0.00	0.00
ATOM	452	CB	GLU	A	31	48.540	60.420	74.410	0.00	0.00
ATOM	453	HB1	GLU	A	31	47.710	60.950	73.900	0.00	0.00
ATOM	454	HB2	GLU	A	31	49.110	59.800	73.690	0.00	0.00
ATOM	455	CG	GLU	A	31	49.620	61.530	74.650	0.00	0.00
ATOM	456	HG1	GLU	A	31	50.490	61.190	75.250	0.00	0.00
ATOM	457	HG2	GLU	A	31	49.220	62.510	74.990	0.00	0.00
ATOM	458	CD	GLU	A	31	50.200	61.950	73.260	0.00	0.00
ATOM	459	OE1	GLU	A	31	51.300	61.470	72.890	0.00	0.00
ATOM	460	OE2	GLU	A	31	49.460	62.630	72.580	0.00	0.00
ATOM	461	C	GLU	A	31	47.900	60.500	76.680	0.00	0.00
ATOM	462	O	GLU	A	31	48.680	60.590	77.580	0.00	0.00
ATOM	463	N	GLU	A	32	46.720	61.180	76.780	0.00	0.00
ATOM	464	HN	GLU	A	32	45.950	61.150	76.150	0.00	0.00
ATOM	465	CA	GLU	A	32	46.420	62.050	77.860	0.00	0.00
ATOM	466	HA	GLU	A	32	47.050	62.930	77.850	0.00	0.00
ATOM	467	CB	GLU	A	32	44.990	62.670	77.700	0.00	0.00
ATOM	468	HB1	GLU	A	32	44.970	63.060	76.660	0.00	0.00
ATOM	469	HB2	GLU	A	32	44.850	63.450	78.470	0.00	0.00
ATOM	470	CG	GLU	A	32	43.780	61.700	77.850	0.00	0.00
ATOM	471	HG1	GLU	A	32	43.780	61.140	78.810	0.00	0.00
ATOM	472	HG2	GLU	A	32	43.860	60.940	77.040	0.00	0.00
ATOM	473	CD	GLU	A	32	42.460	62.480	77.800	0.00	0.00
ATOM	474	OE1	GLU	A	32	41.990	62.900	78.890	0.00	0.00
ATOM	475	OE2	GLU	A	32	41.820	62.670	76.780	0.00	0.00
ATOM	476	C	GLU	A	32	46.590	61.390	79.210	0.00	0.00
ATOM	477	O	GLU	A	32	46.950	62.090	80.180	0.00	0.00
ATOM	478	N	ASN	A	33	46.310	60.090	79.330	0.00	0.00
ATOM	479	HN	ASN	A	33	45.970	59.630	78.520	0.00	0.00
ATOM	480	CA	ASN	A	33	46.430	59.300	80.580	0.00	0.00
ATOM	481	HA	ASN	A	33	45.850	59.770	81.360	0.00	0.00
ATOM	482	CB	ASN	A	33	45.960	57.830	80.410	0.00	0.00
ATOM	483	HB1	ASN	A	33	46.090	57.190	81.310	0.00	0.00

ATOM	484	HB2	ASN	A	33	46.460	57.230	79.610	0.00	0.00
ATOM	485	CG	ASN	A	33	44.500	57.860	80.060	0.00	0.00
ATOM	486	OD1	ASN	A	33	43.750	58.730	80.470	0.00	0.00
ATOM	487	ND2	ASN	A	33	43.980	56.910	79.270	0.00	0.00
ATOM	488	HD21	ASN	A	33	43.070	57.180	78.930	0.00	0.00
ATOM	489	HD22	ASN	A	33	44.500	56.100	79.020	0.00	0.00
ATOM	490	C	ASN	A	33	47.820	59.110	80.920	0.00	0.00
ATOM	491	O	ASN	A	33	48.250	59.180	82.040	0.00	0.00
ATOM	492	N	ALA	A	34	48.720	58.690	79.960	0.00	0.00
ATOM	493	HN	ALA	A	34	48.440	58.500	79.030	0.00	0.00
ATOM	494	CA	ALA	A	34	50.100	58.590	80.190	0.00	0.00
ATOM	495	HA	ALA	A	34	50.170	57.960	81.070	0.00	0.00
ATOM	496	CB	ALA	A	34	50.940	57.800	79.110	0.00	0.00
ATOM	497	HB1	ALA	A	34	50.530	56.770	79.160	0.00	0.00
ATOM	498	HB2	ALA	A	34	52.040	57.770	79.290	0.00	0.00
ATOM	499	HB3	ALA	A	34	50.840	58.300	78.120	0.00	0.00
ATOM	500	C	ALA	A	34	50.790	59.960	80.470	0.00	0.00
ATOM	501	O	ALA	A	34	51.620	60.120	81.400	0.00	0.00
ATOM	502	N	ASN	A	35	50.380	60.970	79.740	0.00	0.00
ATOM	503	HN	ASN	A	35	49.630	60.930	79.090	0.00	0.00
ATOM	504	CA	ASN	A	35	50.850	62.340	79.980	0.00	0.00
ATOM	505	HA	ASN	A	35	51.930	62.350	80.040	0.00	0.00
ATOM	506	CB	ASN	A	35	50.340	63.340	78.930	0.00	0.00
ATOM	507	HB1	ASN	A	35	50.720	64.330	79.260	0.00	0.00
ATOM	508	HB2	ASN	A	35	49.230	63.310	78.890	0.00	0.00
ATOM	509	CG	ASN	A	35	50.950	63.060	77.600	0.00	0.00
ATOM	510	OD1	ASN	A	35	51.630	62.080	77.340	0.00	0.00
ATOM	511	ND2	ASN	A	35	50.950	64.160	76.830	0.00	0.00
ATOM	512	HD21	ASN	A	35	51.450	64.140	75.970	0.00	0.00
ATOM	513	HD22	ASN	A	35	50.160	64.770	76.930	0.00	0.00
ATOM	514	C	ASN	A	35	50.530	62.870	81.380	0.00	0.00
ATOM	515	O	ASN	A	35	51.390	63.370	82.070	0.00	0.00
ATOM	516	N	PHE	A	36	49.240	62.710	81.830	0.00	0.00
ATOM	517	HN	PHE	A	36	48.540	62.330	81.220	0.00	0.00
ATOM	518	CA	PHE	A	36	48.700	62.850	83.170	0.00	0.00
ATOM	519	HA	PHE	A	36	48.870	63.850	83.530	0.00	0.00
ATOM	520	CB	PHE	A	36	47.230	62.280	83.360	0.00	0.00
ATOM	521	HB1	PHE	A	36	47.140	61.210	83.100	0.00	0.00
ATOM	522	HB2	PHE	A	36	46.530	62.790	82.670	0.00	0.00
ATOM	523	CG	PHE	A	36	46.710	62.600	84.750	0.00	0.00
ATOM	524	CD1	PHE	A	36	45.820	63.720	84.910	0.00	0.00
ATOM	525	HD1	PHE	A	36	45.710	64.540	84.210	0.00	0.00
ATOM	526	CE1	PHE	A	36	45.130	63.890	86.080	0.00	0.00
ATOM	527	HE1	PHE	A	36	44.360	64.630	86.160	0.00	0.00
ATOM	528	CZ	PHE	A	36	45.420	63.040	87.140	0.00	0.00
ATOM	529	HZ	PHE	A	36	44.940	63.170	88.100	0.00	0.00
ATOM	530	CD2	PHE	A	36	46.830	61.680	85.790	0.00	0.00
ATOM	531	HD2	PHE	A	36	47.480	60.830	85.770	0.00	0.00
ATOM	532	CE2	PHE	A	36	46.240	61.970	87.020	0.00	0.00
ATOM	533	HE2	PHE	A	36	46.550	61.310	87.820	0.00	0.00
ATOM	534	C	PHE	A	36	49.730	62.180	84.150	0.00	0.00
ATOM	535	O	PHE	A	36	50.320	62.890	84.940	0.00	0.00
ATOM	536	N	ASN	A	37	49.950	60.940	84.060	0.00	0.00
ATOM	537	HN	ASN	A	37	49.440	60.390	83.400	0.00	0.00
ATOM	538	CA	ASN	A	37	50.720	60.150	85.020	0.00	0.00
ATOM	539	HA	ASN	A	37	50.120	60.230	85.910	0.00	0.00
ATOM	540	CB	ASN	A	37	50.840	58.660	84.750	0.00	0.00
ATOM	541	HB1	ASN	A	37	51.570	58.140	85.400	0.00	0.00
ATOM	542	HB2	ASN	A	37	51.180	58.480	83.710	0.00	0.00
ATOM	543	CG	ASN	A	37	49.480	57.920	84.990	0.00	0.00
ATOM	544	OD1	ASN	A	37	48.860	58.040	86.040	0.00	0.00

```
ATOM    545  ND2 ASN A  37      49.120  57.120  83.970  0.00  0.00
ATOM    546 HD21 ASN A  37      48.370  56.470  84.090  0.00  0.00
ATOM    547 HD22 ASN A  37      49.630  56.900  83.140  0.00  0.00
ATOM    548  C   ASN A  37      52.160  60.660  85.200  0.00  0.00
ATOM    549  O   ASN A  37      52.570  60.870  86.320  0.00  0.00
ATOM    550  N   LYS A  38      52.860  60.910  84.090  0.00  0.00
ATOM    551  HN  LYS A  38      52.430  60.740  83.210  0.00  0.00
ATOM    552  CA  LYS A  38      54.210  61.410  84.190  0.00  0.00
ATOM    553  HA  LYS A  38      54.750  60.830  84.920  0.00  0.00
ATOM    554  CB  LYS A  38      55.010  61.370  82.860  0.00  0.00
ATOM    555  HB1 LYS A  38      55.150  60.330  82.500  0.00  0.00
ATOM    556  HB2 LYS A  38      56.030  61.740  83.090  0.00  0.00
ATOM    557  CG  LYS A  38      54.450  62.240  81.730  0.00  0.00
ATOM    558  HG1 LYS A  38      54.520  63.300  82.030  0.00  0.00
ATOM    559  HG2 LYS A  38      53.420  61.880  81.530  0.00  0.00
ATOM    560  CD  LYS A  38      55.330  62.190  80.430  0.00  0.00
ATOM    561  HD1 LYS A  38      56.250  62.760  80.710  0.00  0.00
ATOM    562  HD2 LYS A  38      54.820  62.840  79.690  0.00  0.00
ATOM    563  CE  LYS A  38      55.560  60.780  79.860  0.00  0.00
ATOM    564  HE1 LYS A  38      54.670  60.120  79.880  0.00  0.00
ATOM    565  HE2 LYS A  38      56.360  60.150  80.300  0.00  0.00
ATOM    566  NZ  LYS A  38      56.010  60.800  78.450  0.00  0.00
ATOM    567  HZ1 LYS A  38      55.340  61.230  77.780  0.00  0.00
ATOM    568  HZ2 LYS A  38      56.050  59.840  78.050  0.00  0.00
ATOM    569  HZ3 LYS A  38      56.890  61.350  78.340  0.00  0.00
ATOM    570  C   LYS A  38      54.310  62.830  84.850  0.00  0.00
ATOM    571  O   LYS A  38      55.280  63.000  85.530  0.00  0.00
ATOM    572  N   ILE A  39      53.410  63.760  84.650  0.00  0.00
ATOM    573  HN  ILE A  39      52.630  63.730  84.030  0.00  0.00
ATOM    574  CA  ILE A  39      53.420  65.090  85.170  0.00  0.00
ATOM    575  HA  ILE A  39      54.460  65.380  85.200  0.00  0.00
ATOM    576  CB  ILE A  39      52.670  66.020  84.270  0.00  0.00
ATOM    577  HB  ILE A  39      51.610  65.750  84.080  0.00  0.00
ATOM    578  CG2 ILE A  39      52.710  67.440  84.880  0.00  0.00
ATOM    579 HG21 ILE A  39      53.730  67.870  84.910  0.00  0.00
ATOM    580 HG22 ILE A  39      52.480  67.390  85.970  0.00  0.00
ATOM    581 HG23 ILE A  39      51.940  68.140  84.510  0.00  0.00
ATOM    582  CG1 ILE A  39      53.310  65.950  82.870  0.00  0.00
ATOM    583 HG11 ILE A  39      53.130  64.910  82.510  0.00  0.00
ATOM    584 HG12 ILE A  39      54.380  66.170  83.020  0.00  0.00
ATOM    585  CD  ILE A  39      52.790  66.950  81.850  0.00  0.00
ATOM    586  HD1 ILE A  39      51.680  67.030  81.900  0.00  0.00
ATOM    587  HD2 ILE A  39      53.070  66.660  80.810  0.00  0.00
ATOM    588  HD3 ILE A  39      53.060  68.000  82.110  0.00  0.00
ATOM    589  C   ILE A  39      52.860  65.100  86.610  0.00  0.00
ATOM    590  O   ILE A  39      53.280  65.840  87.530  0.00  0.00
ATOM    591  N   PHE A  40      51.880  64.240  86.940  0.00  0.00
ATOM    592  HN  PHE A  40      51.690  63.540  86.250  0.00  0.00
ATOM    593  CA  PHE A  40      51.120  64.040  88.110  0.00  0.00
ATOM    594  HA  PHE A  40      50.800  65.030  88.390  0.00  0.00
ATOM    595  CB  PHE A  40      49.900  63.130  87.740  0.00  0.00
ATOM    596  HB1 PHE A  40      50.200  62.140  87.350  0.00  0.00
ATOM    597  HB2 PHE A  40      49.220  63.690  87.070  0.00  0.00
ATOM    598  CG  PHE A  40      49.010  62.910  88.920  0.00  0.00
ATOM    599  CD1 PHE A  40      48.770  61.650  89.470  0.00  0.00
ATOM    600  HD1 PHE A  40      49.190  60.750  89.050  0.00  0.00
ATOM    601  CE1 PHE A  40      47.770  61.440  90.450  0.00  0.00
ATOM    602  HE1 PHE A  40      47.480  60.430  90.690  0.00  0.00
ATOM    603  CZ  PHE A  40      46.970  62.510  90.790  0.00  0.00
ATOM    604  HZ  PHE A  40      46.090  62.370  91.400  0.00  0.00
ATOM    605  CD2 PHE A  40      48.300  63.960  89.470  0.00  0.00
```

ATOM 606 HD2 PHE A 40 48.450 64.960 89.100 0.00 0.00
ATOM 607 CE2 PHE A 40 47.270 63.770 90.380 0.00 0.00
ATOM 608 HE2 PHE A 40 46.660 64.610 90.670 0.00 0.00
ATOM 609 C PHE A 40 51.920 63.450 89.220 0.00 0.00
ATOM 610 O PHE A 40 51.910 63.960 90.330 0.00 0.00
ATOM 611 N LEU A 41 52.560 62.360 88.920 0.00 0.00
ATOM 612 HN LEU A 41 52.700 61.990 88.010 0.00 0.00
ATOM 613 CA LEU A 41 53.130 61.510 89.920 0.00 0.00
ATOM 614 HA LEU A 41 52.400 61.490 90.720 0.00 0.00
ATOM 615 CB LEU A 41 53.310 60.080 89.440 0.00 0.00
ATOM 616 HB1 LEU A 41 53.680 59.440 90.270 0.00 0.00
ATOM 617 HB2 LEU A 41 54.180 60.020 88.740 0.00 0.00
ATOM 618 CG LEU A 41 51.990 59.370 89.070 0.00 0.00
ATOM 619 HG LEU A 41 51.450 59.940 88.290 0.00 0.00
ATOM 620 CD1 LEU A 41 52.240 58.050 88.410 0.00 0.00
ATOM 621 HD11 LEU A 41 51.320 57.450 88.220 0.00 0.00
ATOM 622 HD12 LEU A 41 52.880 57.410 89.060 0.00 0.00
ATOM 623 HD13 LEU A 41 52.760 58.210 87.440 0.00 0.00
ATOM 624 CD2 LEU A 41 51.000 59.290 90.220 0.00 0.00
ATOM 625 HD21 LEU A 41 49.980 59.020 89.890 0.00 0.00
ATOM 626 HD22 LEU A 41 50.690 60.230 90.730 0.00 0.00
ATOM 627 HD23 LEU A 41 51.310 58.580 91.020 0.00 0.00
ATOM 628 C LEU A 41 54.340 62.010 90.780 0.00 0.00
ATOM 629 O LEU A 41 54.450 61.530 91.900 0.00 0.00
ATOM 630 N PRO A 42 55.220 62.970 90.460 0.00 0.00
ATOM 631 CD PRO A 42 55.460 63.270 89.030 0.00 0.00
ATOM 632 HD1 PRO A 42 54.910 64.190 88.720 0.00 0.00
ATOM 633 HD2 PRO A 42 55.210 62.420 88.360 0.00 0.00
ATOM 634 CA PRO A 42 56.450 63.090 91.190 0.00 0.00
ATOM 635 HA PRO A 42 56.910 62.110 91.240 0.00 0.00
ATOM 636 CB PRO A 42 57.300 63.990 90.290 0.00 0.00
ATOM 637 HB1 PRO A 42 58.380 63.980 90.530 0.00 0.00
ATOM 638 HB2 PRO A 42 56.980 65.060 90.280 0.00 0.00
ATOM 639 CG PRO A 42 56.950 63.480 88.900 0.00 0.00
ATOM 640 HG1 PRO A 42 57.300 64.140 88.080 0.00 0.00
ATOM 641 HG2 PRO A 42 57.460 62.500 88.770 0.00 0.00
ATOM 642 C PRO A 42 56.250 63.720 92.580 0.00 0.00
ATOM 643 O PRO A 42 56.870 63.420 93.580 0.00 0.00
ATOM 644 N THR A 43 55.360 64.740 92.690 0.00 0.00
ATOM 645 HN THR A 43 54.910 65.130 91.890 0.00 0.00
ATOM 646 CA THR A 43 55.120 65.520 93.920 0.00 0.00
ATOM 647 HA THR A 43 56.040 65.850 94.380 0.00 0.00
ATOM 648 CB THR A 43 54.360 66.790 93.650 0.00 0.00
ATOM 649 HB THR A 43 53.280 66.640 93.430 0.00 0.00
ATOM 650 OG1 THR A 43 54.850 67.380 92.440 0.00 0.00
ATOM 651 HG1 THR A 43 54.520 66.770 91.780 0.00 0.00
ATOM 652 CG2 THR A 43 54.520 67.780 94.850 0.00 0.00
ATOM 653 HG21 THR A 43 53.950 67.390 95.730 0.00 0.00
ATOM 654 HG22 THR A 43 54.190 68.780 94.520 0.00 0.00
ATOM 655 HG23 THR A 43 55.600 67.930 95.070 0.00 0.00
ATOM 656 C THR A 43 54.400 64.680 94.900 0.00 0.00
ATOM 657 O THR A 43 54.610 64.710 96.100 0.00 0.00
ATOM 658 N ILE A 44 53.470 63.830 94.460 0.00 0.00
ATOM 659 HN ILE A 44 53.360 63.810 93.470 0.00 0.00
ATOM 660 CA ILE A 44 52.690 62.930 95.220 0.00 0.00
ATOM 661 HA ILE A 44 52.370 63.500 96.080 0.00 0.00
ATOM 662 CB ILE A 44 51.550 62.340 94.450 0.00 0.00
ATOM 663 HB ILE A 44 51.790 61.630 93.630 0.00 0.00
ATOM 664 CG2 ILE A 44 50.730 61.520 95.520 0.00 0.00
ATOM 665 HG21 ILE A 44 49.680 61.410 95.170 0.00 0.00
ATOM 666 HG22 ILE A 44 50.800 62.060 96.490 0.00 0.00

ATOM 667 HG23 ILE A 44 51.210 60.520 95.580 0.00 0.00
ATOM 668 CG1 ILE A 44 50.670 63.410 93.740 0.00 0.00
ATOM 669 HG11 ILE A 44 51.230 64.180 93.160 0.00 0.00
ATOM 670 HG12 ILE A 44 50.250 63.910 94.630 0.00 0.00
ATOM 671 CD ILE A 44 49.720 62.760 92.810 0.00 0.00
ATOM 672 HD1 ILE A 44 50.250 62.380 91.910 0.00 0.00
ATOM 673 HD2 ILE A 44 49.000 63.470 92.340 0.00 0.00
ATOM 674 HD3 ILE A 44 49.100 61.940 93.230 0.00 0.00
ATOM 675 C ILE A 44 53.570 61.930 95.860 0.00 0.00
ATOM 676 O ILE A 44 53.580 61.710 97.090 0.00 0.00
ATOM 677 N TYR A 45 54.490 61.240 95.050 0.00 0.00
ATOM 678 HN TYR A 45 54.480 61.340 94.060 0.00 0.00
ATOM 679 CA TYR A 45 55.400 60.230 95.510 0.00 0.00
ATOM 680 HA TYR A 45 54.910 59.470 96.090 0.00 0.00
ATOM 681 CB TYR A 45 56.050 59.600 94.250 0.00 0.00
ATOM 682 HB1 TYR A 45 57.060 59.200 94.440 0.00 0.00
ATOM 683 HB2 TYR A 45 56.140 60.430 93.510 0.00 0.00
ATOM 684 CG TYR A 45 55.090 58.520 93.560 0.00 0.00
ATOM 685 CD1 TYR A 45 55.500 57.880 92.380 0.00 0.00
ATOM 686 HD1 TYR A 45 56.500 58.070 92.010 0.00 0.00
ATOM 687 CE1 TYR A 45 54.600 56.980 91.660 0.00 0.00
ATOM 688 HE1 TYR A 45 54.930 56.640 90.690 0.00 0.00
ATOM 689 CZ TYR A 45 53.370 56.750 92.170 0.00 0.00
ATOM 690 OH TYR A 45 52.440 55.930 91.550 0.00 0.00
ATOM 691 HH TYR A 45 51.630 56.060 92.050 0.00 0.00
ATOM 692 CD2 TYR A 45 53.790 58.390 94.000 0.00 0.00
ATOM 693 HD2 TYR A 45 53.400 58.900 94.860 0.00 0.00
ATOM 694 CE2 TYR A 45 52.940 57.420 93.360 0.00 0.00
ATOM 695 HE2 TYR A 45 51.940 57.330 93.740 0.00 0.00
ATOM 696 C TYR A 45 56.470 60.880 96.440 0.00 0.00
ATOM 697 O TYR A 45 56.950 60.160 97.330 0.00 0.00
ATOM 698 N SER A 46 56.910 62.140 96.170 0.00 0.00
ATOM 699 HN SER A 46 56.630 62.720 95.410 0.00 0.00
ATOM 700 CA SER A 46 57.800 62.800 97.110 0.00 0.00
ATOM 701 HA SER A 46 58.560 62.080 97.380 0.00 0.00
ATOM 702 CB SER A 46 58.450 64.050 96.530 0.00 0.00
ATOM 703 HB1 SER A 46 59.050 64.670 97.230 0.00 0.00
ATOM 704 HB2 SER A 46 57.660 64.760 96.200 0.00 0.00
ATOM 705 OG SER A 46 59.350 63.780 95.420 0.00 0.00
ATOM 706 HG1 SER A 46 58.930 63.550 94.580 0.00 0.00
ATOM 707 C SER A 46 57.110 63.080 98.490 0.00 0.00
ATOM 708 O SER A 46 57.850 62.850 99.430 0.00 0.00
ATOM 709 N ILE A 47 55.800 63.490 98.570 0.00 0.00
ATOM 710 HN ILE A 47 55.360 63.940 97.790 0.00 0.00
ATOM 711 CA ILE A 47 55.040 63.580 99.910 0.00 0.00
ATOM 712 HA ILE A 47 55.740 63.990 100.630 0.00 0.00
ATOM 713 CB ILE A 47 53.810 64.450 99.810 0.00 0.00
ATOM 714 HB ILE A 47 53.150 64.090 99.000 0.00 0.00
ATOM 715 CG2 ILE A 47 52.940 64.310 101.140 0.00 0.00
ATOM 716 HG21 ILE A 47 52.040 64.940 101.010 0.00 0.00
ATOM 717 HG22 ILE A 47 53.440 64.730 102.050 0.00 0.00
ATOM 718 HG23 ILE A 47 52.470 63.320 101.320 0.00 0.00
ATOM 719 CG1 ILE A 47 54.150 65.960 99.450 0.00 0.00
ATOM 720 HG11 ILE A 47 53.130 66.410 99.410 0.00 0.00
ATOM 721 HG12 ILE A 47 54.580 65.830 98.440 0.00 0.00
ATOM 722 CD ILE A 47 55.020 66.690 100.480 0.00 0.00
ATOM 723 HD1 ILE A 47 55.370 67.630 100.000 0.00 0.00
ATOM 724 HD2 ILE A 47 55.860 66.150 100.960 0.00 0.00
ATOM 725 HD3 ILE A 47 54.500 67.110 101.370 0.00 0.00
ATOM 726 C ILE A 47 54.900 62.230 100.560 0.00 0.00
ATOM 727 O ILE A 47 55.050 62.050 101.730 0.00 0.00

ATOM	728	N	ILE	A	48	54.530	61.190	99.800	0.00	0.00
ATOM	729	HN	ILE	A	48	54.250	61.240	98.850	0.00	0.00
ATOM	730	CA	ILE	A	48	54.390	59.880	100.330	0.00	0.00
ATOM	731	HA	ILE	A	48	53.790	59.940	101.220	0.00	0.00
ATOM	732	CB	ILE	A	48	53.620	59.050	99.340	0.00	0.00
ATOM	733	HB	ILE	A	48	54.170	59.010	98.370	0.00	0.00
ATOM	734	CG2	ILE	A	48	53.670	57.570	99.730	0.00	0.00
ATOM	735	HG21	ILE	A	48	52.940	57.020	99.100	0.00	0.00
ATOM	736	HG22	ILE	A	48	53.420	57.520	100.820	0.00	0.00
ATOM	737	HG23	ILE	A	48	54.630	57.070	99.510	0.00	0.00
ATOM	738	CG1	ILE	A	48	52.200	59.670	99.120	0.00	0.00
ATOM	739	HG11	ILE	A	48	52.260	60.780	99.110	0.00	0.00
ATOM	740	HG12	ILE	A	48	51.560	59.520	100.010	0.00	0.00
ATOM	741	CD	ILE	A	48	51.540	59.110	97.910	0.00	0.00
ATOM	742	HD1	ILE	A	48	50.600	59.490	97.460	0.00	0.00
ATOM	743	HD2	ILE	A	48	51.460	58.020	98.110	0.00	0.00
ATOM	744	HD3	ILE	A	48	52.240	59.130	97.040	0.00	0.00
ATOM	745	C	ILE	A	48	55.750	59.320	100.830	0.00	0.00
ATOM	746	O	ILE	A	48	55.780	58.720	101.900	0.00	0.00
ATOM	747	N	PHE	A	49	56.830	59.440	100.110	0.00	0.00
ATOM	748	HN	PHE	A	49	56.750	59.780	99.170	0.00	0.00
ATOM	749	CA	PHE	A	49	58.200	59.150	100.570	0.00	0.00
ATOM	750	HA	PHE	A	49	58.170	58.090	100.790	0.00	0.00
ATOM	751	CB	PHE	A	49	59.240	59.310	99.370	0.00	0.00
ATOM	752	HB1	PHE	A	49	59.150	60.330	98.930	0.00	0.00
ATOM	753	HB2	PHE	A	49	59.000	58.700	98.470	0.00	0.00
ATOM	754	CG	PHE	A	49	60.730	59.190	99.700	0.00	0.00
ATOM	755	CD1	PHE	A	49	61.610	60.290	99.870	0.00	0.00
ATOM	756	HD1	PHE	A	49	61.190	61.270	99.740	0.00	0.00
ATOM	757	CE1	PHE	A	49	62.990	60.030	99.910	0.00	0.00
ATOM	758	HE1	PHE	A	49	63.660	60.880	99.970	0.00	0.00
ATOM	759	CZ	PHE	A	49	63.530	58.730	99.920	0.00	0.00
ATOM	760	HZ	PHE	A	49	64.610	58.650	99.980	0.00	0.00
ATOM	761	CD2	PHE	A	49	61.290	57.850	99.640	0.00	0.00
ATOM	762	HD2	PHE	A	49	60.550	57.080	99.450	0.00	0.00
ATOM	763	CE2	PHE	A	49	62.670	57.660	99.660	0.00	0.00
ATOM	764	HE2	PHE	A	49	63.050	56.640	99.660	0.00	0.00
ATOM	765	C	PHE	A	49	58.690	59.780	101.900	0.00	0.00
ATOM	766	O	PHE	A	49	59.140	59.090	102.830	0.00	0.00
ATOM	767	N	LEU	A	50	58.600	61.130	102.010	0.00	0.00
ATOM	768	HN	LEU	A	50	58.010	61.570	101.340	0.00	0.00
ATOM	769	CA	LEU	A	50	58.780	61.870	103.250	0.00	0.00
ATOM	770	HA	LEU	A	50	59.760	61.680	103.660	0.00	0.00
ATOM	771	CB	LEU	A	50	58.770	63.370	102.950	0.00	0.00
ATOM	772	HB1	LEU	A	50	58.870	63.940	103.900	0.00	0.00
ATOM	773	HB2	LEU	A	50	57.860	63.750	102.450	0.00	0.00
ATOM	774	CG	LEU	A	50	59.940	63.770	101.970	0.00	0.00
ATOM	775	HG	LEU	A	50	59.750	63.200	101.030	0.00	0.00
ATOM	776	CD1	LEU	A	50	60.070	65.260	101.590	0.00	0.00
ATOM	777	HD11	LEU	A	50	60.950	65.300	100.910	0.00	0.00
ATOM	778	HD12	LEU	A	50	60.300	65.900	102.460	0.00	0.00
ATOM	779	HD13	LEU	A	50	59.160	65.780	101.220	0.00	0.00
ATOM	780	CD2	LEU	A	50	61.320	63.260	102.450	0.00	0.00
ATOM	781	HD21	LEU	A	50	61.750	63.750	103.350	0.00	0.00
ATOM	782	HD22	LEU	A	50	62.040	63.570	101.670	0.00	0.00
ATOM	783	HD23	LEU	A	50	61.320	62.150	102.450	0.00	0.00
ATOM	784	C	LEU	A	50	57.880	61.480	104.440	0.00	0.00
ATOM	785	O	LEU	A	50	58.450	61.450	105.510	0.00	0.00
ATOM	786	N	THR	A	51	56.570	61.170	104.290	0.00	0.00
ATOM	787	HN	THR	A	51	56.270	61.210	103.350	0.00	0.00
ATOM	788	CA	THR	A	51	55.710	60.550	105.240	0.00	0.00

ATOM	789	HA	THR	A	51	55.800	60.960	106.230	0.00	0.00
ATOM	790	CB	THR	A	51	54.190	60.550	104.730	0.00	0.00
ATOM	791	HB	THR	A	51	53.980	59.790	103.950	0.00	0.00
ATOM	792	OG1	THR	A	51	53.850	61.840	104.290	0.00	0.00
ATOM	793	HG1	THR	A	51	54.270	62.040	103.450	0.00	0.00
ATOM	794	CG2	THR	A	51	53.310	60.350	105.920	0.00	0.00
ATOM	795	HG21	THR	A	51	53.540	61.060	106.750	0.00	0.00
ATOM	796	HG22	THR	A	51	53.430	59.370	106.440	0.00	0.00
ATOM	797	HG23	THR	A	51	52.240	60.380	105.650	0.00	0.00
ATOM	798	C	THR	A	51	56.090	59.190	105.530	0.00	0.00
ATOM	799	O	THR	A	51	56.230	58.870	106.720	0.00	0.00
ATOM	800	N	GLY	A	52	56.350	58.280	104.550	0.00	0.00
ATOM	801	HN	GLY	A	52	56.050	58.380	103.610	0.00	0.00
ATOM	802	CA	GLY	A	52	56.920	56.960	104.790	0.00	0.00
ATOM	803	HA1	GLY	A	52	57.060	56.450	103.850	0.00	0.00
ATOM	804	HA2	GLY	A	52	56.200	56.390	105.350	0.00	0.00
ATOM	805	C	GLY	A	52	58.130	56.730	105.690	0.00	0.00
ATOM	806	O	GLY	A	52	58.030	55.960	106.680	0.00	0.00
ATOM	807	N	ILE	A	53	59.220	57.480	105.390	0.00	0.00
ATOM	808	HN	ILE	A	53	59.180	58.080	104.600	0.00	0.00
ATOM	809	CA	ILE	A	53	60.470	57.440	106.090	0.00	0.00
ATOM	810	HA	ILE	A	53	60.800	56.420	106.090	0.00	0.00
ATOM	811	CB	ILE	A	53	61.640	58.150	105.330	0.00	0.00
ATOM	812	HB	ILE	A	53	61.070	59.000	104.900	0.00	0.00
ATOM	813	CG2	ILE	A	53	62.830	58.540	106.230	0.00	0.00
ATOM	814	HG21	ILE	A	53	63.610	59.150	105.730	0.00	0.00
ATOM	815	HG22	ILE	A	53	63.180	57.550	106.600	0.00	0.00
ATOM	816	HG23	ILE	A	53	62.560	59.190	107.080	0.00	0.00
ATOM	817	CG1	ILE	A	53	62.110	57.270	104.250	0.00	0.00
ATOM	818	HG11	ILE	A	53	61.200	56.970	103.680	0.00	0.00
ATOM	819	HG12	ILE	A	53	62.490	56.320	104.680	0.00	0.00
ATOM	820	CD	ILE	A	53	63.160	57.930	103.340	0.00	0.00
ATOM	821	HD1	ILE	A	53	63.490	57.160	102.600	0.00	0.00
ATOM	822	HD2	ILE	A	53	64.120	58.170	103.850	0.00	0.00
ATOM	823	HD3	ILE	A	53	62.850	58.870	102.850	0.00	0.00
ATOM	824	C	ILE	A	53	60.310	57.770	107.590	0.00	0.00
ATOM	825	O	ILE	A	53	60.710	56.970	108.400	0.00	0.00
ATOM	826	N	VAL	A	54	59.680	58.940	107.880	0.00	0.00
ATOM	827	HN	VAL	A	54	59.220	59.400	107.120	0.00	0.00
ATOM	828	CA	VAL	A	54	59.360	59.370	109.220	0.00	0.00
ATOM	829	HA	VAL	A	54	60.270	59.520	109.790	0.00	0.00
ATOM	830	CB	VAL	A	54	58.650	60.750	109.180	0.00	0.00
ATOM	831	HB	VAL	A	54	57.610	60.690	108.800	0.00	0.00
ATOM	832	CG1	VAL	A	54	58.640	61.280	110.630	0.00	0.00
ATOM	833	HG11	VAL	A	54	57.830	60.790	111.210	0.00	0.00
ATOM	834	HG12	VAL	A	54	58.460	62.370	110.610	0.00	0.00
ATOM	835	HG13	VAL	A	54	59.560	61.030	111.210	0.00	0.00
ATOM	836	CG2	VAL	A	54	59.600	61.810	108.440	0.00	0.00
ATOM	837	HG21	VAL	A	54	59.890	61.400	107.450	0.00	0.00
ATOM	838	HG22	VAL	A	54	60.560	61.930	108.990	0.00	0.00
ATOM	839	HG23	VAL	A	54	59.170	62.830	108.300	0.00	0.00
ATOM	840	C	VAL	A	54	58.490	58.460	110.060	0.00	0.00
ATOM	841	O	VAL	A	54	58.850	58.130	111.220	0.00	0.00
ATOM	842	N	GLY	A	55	57.390	58.000	109.450	0.00	0.00
ATOM	843	HN	GLY	A	55	57.180	58.330	108.540	0.00	0.00
ATOM	844	CA	GLY	A	55	56.440	57.140	110.090	0.00	0.00
ATOM	845	HA1	GLY	A	55	55.570	57.070	109.460	0.00	0.00
ATOM	846	HA2	GLY	A	55	56.240	57.460	111.100	0.00	0.00
ATOM	847	C	GLY	A	55	56.880	55.750	110.330	0.00	0.00
ATOM	848	O	GLY	A	55	56.920	55.360	111.500	0.00	0.00
ATOM	849	N	ASN	A	56	57.320	55.050	109.230	0.00	0.00

ATOM 850 HN ASN A 56 57.360 55.410 108.310 0.00 0.00
ATOM 851 CA ASN A 56 57.690 53.680 109.450 0.00 0.00
ATOM 852 HA ASN A 56 57.020 53.240 110.170 0.00 0.00
ATOM 853 CB ASN A 56 57.540 52.910 108.080 0.00 0.00
ATOM 854 HB1 ASN A 56 57.900 51.870 108.220 0.00 0.00
ATOM 855 HB2 ASN A 56 58.160 53.390 107.300 0.00 0.00
ATOM 856 CG ASN A 56 56.020 52.840 107.740 0.00 0.00
ATOM 857 OD1 ASN A 56 55.180 52.240 108.420 0.00 0.00
ATOM 858 ND2 ASN A 56 55.660 53.400 106.590 0.00 0.00
ATOM 859 HD21 ASN A 56 54.720 53.290 106.260 0.00 0.00
ATOM 860 HD22 ASN A 56 56.480 53.570 106.030 0.00 0.00
ATOM 861 C ASN A 56 59.120 53.540 110.060 0.00 0.00
ATOM 862 O ASN A 56 59.430 52.530 110.660 0.00 0.00
ATOM 863 N GLY A 57 59.920 54.560 109.840 0.00 0.00
ATOM 864 HN GLY A 57 59.580 55.310 109.270 0.00 0.00
ATOM 865 CA GLY A 57 61.120 54.750 110.620 0.00 0.00
ATOM 866 HA1 GLY A 57 61.650 55.620 110.280 0.00 0.00
ATOM 867 HA2 GLY A 57 61.700 53.860 110.400 0.00 0.00
ATOM 868 C GLY A 57 60.940 54.710 112.080 0.00 0.00
ATOM 869 O GLY A 57 61.550 53.920 112.760 0.00 0.00
ATOM 870 N LEU A 58 60.070 55.620 112.580 0.00 0.00
ATOM 871 HN LEU A 58 59.710 56.250 111.910 0.00 0.00
ATOM 872 CA LEU A 58 59.710 55.800 113.950 0.00 0.00
ATOM 873 HA LEU A 58 60.680 56.040 114.360 0.00 0.00
ATOM 874 CB LEU A 58 58.800 57.080 114.160 0.00 0.00
ATOM 875 HB1 LEU A 58 57.850 56.970 113.590 0.00 0.00
ATOM 876 HB2 LEU A 58 59.390 57.930 113.780 0.00 0.00
ATOM 877 CG LEU A 58 58.530 57.510 115.630 0.00 0.00
ATOM 878 HG LEU A 58 57.830 56.820 116.150 0.00 0.00
ATOM 879 CD1 LEU A 58 59.750 57.520 116.540 0.00 0.00
ATOM 880 HD11 LEU A 58 60.310 56.560 116.520 0.00 0.00
ATOM 881 HD12 LEU A 58 59.530 57.730 117.610 0.00 0.00
ATOM 882 HD13 LEU A 58 60.380 58.340 116.140 0.00 0.00
ATOM 883 CD2 LEU A 58 57.990 58.920 115.620 0.00 0.00
ATOM 884 HD21 LEU A 58 58.090 59.380 116.620 0.00 0.00
ATOM 885 HD22 LEU A 58 56.930 58.980 115.320 0.00 0.00
ATOM 886 HD23 LEU A 58 58.500 59.610 114.910 0.00 0.00
ATOM 887 C LEU A 58 59.040 54.570 114.580 0.00 0.00
ATOM 888 O LEU A 58 59.290 54.340 115.800 0.00 0.00
ATOM 889 N VAL A 59 58.200 53.720 113.950 0.00 0.00
ATOM 890 HN VAL A 59 58.040 53.780 112.970 0.00 0.00
ATOM 891 CA VAL A 59 57.680 52.550 114.640 0.00 0.00
ATOM 892 HA VAL A 59 57.240 52.910 115.560 0.00 0.00
ATOM 893 CB VAL A 59 56.500 51.850 114.010 0.00 0.00
ATOM 894 HB VAL A 59 56.800 51.600 112.970 0.00 0.00
ATOM 895 CG1 VAL A 59 56.100 50.470 114.570 0.00 0.00
ATOM 896 HG11 VAL A 59 55.200 50.090 114.020 0.00 0.00
ATOM 897 HG12 VAL A 59 55.750 50.610 115.620 0.00 0.00
ATOM 898 HG13 VAL A 59 56.900 49.700 114.530 0.00 0.00
ATOM 899 CG2 VAL A 59 55.310 52.780 113.750 0.00 0.00
ATOM 900 HG21 VAL A 59 54.990 53.250 114.700 0.00 0.00
ATOM 901 HG22 VAL A 59 54.420 52.160 113.500 0.00 0.00
ATOM 902 HG23 VAL A 59 55.500 53.590 113.000 0.00 0.00
ATOM 903 C VAL A 59 58.700 51.540 115.110 0.00 0.00
ATOM 904 O VAL A 59 58.650 51.080 116.250 0.00 0.00
ATOM 905 N ILE A 60 59.670 51.300 114.210 0.00 0.00
ATOM 906 HN ILE A 60 59.550 51.720 113.320 0.00 0.00
ATOM 907 CA ILE A 60 60.920 50.550 114.510 0.00 0.00
ATOM 908 HA ILE A 60 60.750 49.540 114.850 0.00 0.00
ATOM 909 CB ILE A 60 61.650 50.380 113.110 0.00 0.00
ATOM 910 HB ILE A 60 61.610 51.430 112.730 0.00 0.00

ATOM	911	CG2	ILE	A	60	63.090	49.890	113.410	0.00	0.00
ATOM	912	HG21	ILE	A	60	63.610	49.740	112.440	0.00	0.00
ATOM	913	HG22	ILE	A	60	63.200	48.980	114.030	0.00	0.00
ATOM	914	HG23	ILE	A	60	63.650	50.690	113.960	0.00	0.00
ATOM	915	CG1	ILE	A	60	60.810	49.450	112.210	0.00	0.00
ATOM	916	HG11	ILE	A	60	59.740	49.710	112.270	0.00	0.00
ATOM	917	HG12	ILE	A	60	61.020	48.390	112.480	0.00	0.00
ATOM	918	CD	ILE	A	60	61.260	49.580	110.700	0.00	0.00
ATOM	919	HD1	ILE	A	60	60.960	50.500	110.150	0.00	0.00
ATOM	920	HD2	ILE	A	60	61.140	48.610	110.170	0.00	0.00
ATOM	921	HD3	ILE	A	60	62.360	49.760	110.720	0.00	0.00
ATOM	922	C	ILE	A	60	61.770	51.100	115.600	0.00	0.00
ATOM	923	O	ILE	A	60	62.180	50.380	116.570	0.00	0.00
ATOM	924	N	LEU	A	61	62.030	52.460	115.530	0.00	0.00
ATOM	925	HN	LEU	A	61	61.840	52.850	114.630	0.00	0.00
ATOM	926	CA	LEU	A	61	62.600	53.120	116.670	0.00	0.00
ATOM	927	HA	LEU	A	61	63.540	52.620	116.840	0.00	0.00
ATOM	928	CB	LEU	A	61	62.670	54.700	116.420	0.00	0.00
ATOM	929	HB1	LEU	A	61	62.980	55.220	117.350	0.00	0.00
ATOM	930	HB2	LEU	A	61	61.710	55.120	116.060	0.00	0.00
ATOM	931	CG	LEU	A	61	63.730	55.180	115.350	0.00	0.00
ATOM	932	HG	LEU	A	61	63.420	54.680	114.400	0.00	0.00
ATOM	933	CD1	LEU	A	61	63.790	56.690	115.210	0.00	0.00
ATOM	934	HD11	LEU	A	61	63.980	57.220	116.170	0.00	0.00
ATOM	935	HD12	LEU	A	61	62.800	57.020	114.830	0.00	0.00
ATOM	936	HD13	LEU	A	61	64.550	57.060	114.490	0.00	0.00
ATOM	937	CD2	LEU	A	61	65.130	54.570	115.580	0.00	0.00
ATOM	938	HD21	LEU	A	61	65.300	53.490	115.370	0.00	0.00
ATOM	939	HD22	LEU	A	61	65.450	54.560	116.640	0.00	0.00
ATOM	940	HD23	LEU	A	61	65.900	55.140	115.020	0.00	0.00
ATOM	941	C	LEU	A	61	61.810	52.930	118.020	0.00	0.00
ATOM	942	O	LEU	A	61	62.430	52.750	119.050	0.00	0.00
ATOM	943	N	VAL	A	62	60.460	52.910	118.110	0.00	0.00
ATOM	944	HN	VAL	A	62	59.990	53.220	117.290	0.00	0.00
ATOM	945	CA	VAL	A	62	59.610	52.710	119.210	0.00	0.00
ATOM	946	HA	VAL	A	62	59.950	53.370	119.990	0.00	0.00
ATOM	947	CB	VAL	A	62	58.190	53.130	119.020	0.00	0.00
ATOM	948	HB	VAL	A	62	58.010	52.650	118.030	0.00	0.00
ATOM	949	CG1	VAL	A	62	57.150	52.600	120.120	0.00	0.00
ATOM	950	HG11	VAL	A	62	57.310	53.040	121.120	0.00	0.00
ATOM	951	HG12	VAL	A	62	56.980	51.500	120.150	0.00	0.00
ATOM	952	HG13	VAL	A	62	56.160	52.940	119.760	0.00	0.00
ATOM	953	CG2	VAL	A	62	58.010	54.690	118.940	0.00	0.00
ATOM	954	HG21	VAL	A	62	58.390	55.030	119.920	0.00	0.00
ATOM	955	HG22	VAL	A	62	56.960	54.980	118.710	0.00	0.00
ATOM	956	HG23	VAL	A	62	58.660	54.940	118.070	0.00	0.00
ATOM	957	C	VAL	A	62	59.700	51.240	119.830	0.00	0.00
ATOM	958	O	VAL	A	62	59.670	51.010	121.030	0.00	0.00
ATOM	959	N	MET	A	63	59.940	50.130	118.980	0.00	0.00
ATOM	960	HN	MET	A	63	60.110	50.170	118.000	0.00	0.00
ATOM	961	CA	MET	A	63	60.290	48.930	119.500	0.00	0.00
ATOM	962	HA	MET	A	63	59.610	48.750	120.320	0.00	0.00
ATOM	963	CB	MET	A	63	60.330	47.790	118.370	0.00	0.00
ATOM	964	HB1	MET	A	63	60.810	48.070	117.410	0.00	0.00
ATOM	965	HB2	MET	A	63	59.240	47.620	118.240	0.00	0.00
ATOM	966	CG	MET	A	63	61.040	46.510	118.820	0.00	0.00
ATOM	967	HG1	MET	A	63	62.140	46.640	118.980	0.00	0.00
ATOM	968	HG2	MET	A	63	61.070	45.740	118.030	0.00	0.00
ATOM	969	SD	MET	A	63	60.160	45.700	120.270	0.00	0.00
ATOM	970	CE	MET	A	63	61.420	44.420	120.520	0.00	0.00
ATOM	971	HE1	MET	A	63	61.160	43.750	121.370	0.00	0.00

ATOM	972	HE2	MET	A	63	62.410	44.880	120.750	0.00	0.00
ATOM	973	HE3	MET	A	63	61.470	43.740	119.640	0.00	0.00
ATOM	974	C	MET	A	63	61.610	48.980	120.210	0.00	0.00
ATOM	975	O	MET	A	63	61.850	48.390	121.260	0.00	0.00
ATOM	976	N	GLY	A	64	62.590	49.800	119.710	0.00	0.00
ATOM	977	HN	GLY	A	64	62.390	50.340	118.900	0.00	0.00
ATOM	978	CA	GLY	A	64	63.790	50.080	120.500	0.00	0.00
ATOM	979	HA1	GLY	A	64	64.390	50.690	119.840	0.00	0.00
ATOM	980	HA2	GLY	A	64	64.290	49.130	120.630	0.00	0.00
ATOM	981	C	GLY	A	64	63.660	50.710	121.850	0.00	0.00
ATOM	982	O	GLY	A	64	64.530	50.520	122.680	0.00	0.00
ATOM	983	N	TYR	A	65	62.530	51.460	122.100	0.00	0.00
ATOM	984	HN	TYR	A	65	61.850	51.610	121.390	0.00	0.00
ATOM	985	CA	TYR	A	65	62.310	52.200	123.360	0.00	0.00
ATOM	986	HA	TYR	A	65	63.270	52.470	123.780	0.00	0.00
ATOM	987	CB	TYR	A	65	61.370	53.420	123.160	0.00	0.00
ATOM	988	HB1	TYR	A	65	61.180	54.000	124.090	0.00	0.00
ATOM	989	HB2	TYR	A	65	60.410	53.090	122.710	0.00	0.00
ATOM	990	CG	TYR	A	65	61.940	54.480	122.260	0.00	0.00
ATOM	991	CD1	TYR	A	65	63.330	54.660	122.050	0.00	0.00
ATOM	992	HD1	TYR	A	65	64.090	54.070	122.550	0.00	0.00
ATOM	993	CE1	TYR	A	65	63.850	55.560	121.130	0.00	0.00
ATOM	994	HE1	TYR	A	65	64.920	55.680	121.120	0.00	0.00
ATOM	995	CZ	TYR	A	65	63.040	56.310	120.370	0.00	0.00
ATOM	996	OH	TYR	A	65	63.530	57.290	119.510	0.00	0.00
ATOM	997	HH	TYR	A	65	62.790	57.610	118.990	0.00	0.00
ATOM	998	CD2	TYR	A	65	61.090	55.250	121.430	0.00	0.00
ATOM	999	HD2	TYR	A	65	60.010	55.190	121.520	0.00	0.00
ATOM	1000	CE2	TYR	A	65	61.620	56.150	120.490	0.00	0.00
ATOM	1001	HE2	TYR	A	65	61.020	56.660	119.750	0.00	0.00
ATOM	1002	C	TYR	A	65	61.690	51.270	124.380	0.00	0.00
ATOM	1003	O	TYR	A	65	61.690	51.650	125.570	0.00	0.00
ATOM	1004	N	GLN	A	66	61.310	50.040	124.010	0.00	0.00
ATOM	1005	HN	GLN	A	66	61.530	49.900	123.050	0.00	0.00
ATOM	1006	CA	GLN	A	66	61.020	48.930	124.900	0.00	0.00
ATOM	1007	HA	GLN	A	66	60.700	48.120	124.260	0.00	0.00
ATOM	1008	CB	GLN	A	66	62.330	48.480	125.780	0.00	0.00
ATOM	1009	HB1	GLN	A	66	62.000	47.890	126.660	0.00	0.00
ATOM	1010	HB2	GLN	A	66	62.790	49.360	126.260	0.00	0.00
ATOM	1011	CG	GLN	A	66	63.400	47.620	125.040	0.00	0.00
ATOM	1012	HG1	GLN	A	66	64.270	47.330	125.660	0.00	0.00
ATOM	1013	HG2	GLN	A	66	63.820	48.090	124.120	0.00	0.00
ATOM	1014	CD	GLN	A	66	62.770	46.320	124.490	0.00	0.00
ATOM	1015	OE1	GLN	A	66	62.560	45.370	125.280	0.00	0.00
ATOM	1016	NE2	GLN	A	66	62.360	46.180	123.270	0.00	0.00
ATOM	1017	HE21	GLN	A	66	62.400	46.910	122.590	0.00	0.00
ATOM	1018	HE22	GLN	A	66	61.680	45.470	123.120	0.00	0.00
ATOM	1019	C	GLN	A	66	59.890	49.230	125.880	0.00	0.00
ATOM	1020	O	GLN	A	66	59.970	49.020	127.020	0.00	0.00
ATOM	1021	N	LYS	A	67	58.750	49.740	125.360	0.00	0.00
ATOM	1022	HN	LYS	A	67	58.660	49.700	124.360	0.00	0.00
ATOM	1023	CA	LYS	A	67	57.450	50.030	125.970	0.00	0.00
ATOM	1024	HA	LYS	A	67	57.610	50.620	126.860	0.00	0.00
ATOM	1025	CB	LYS	A	67	56.470	50.620	124.920	0.00	0.00
ATOM	1026	HB1	LYS	A	67	55.500	50.610	125.460	0.00	0.00
ATOM	1027	HB2	LYS	A	67	56.330	49.850	124.120	0.00	0.00
ATOM	1028	CG	LYS	A	67	56.800	51.980	124.330	0.00	0.00
ATOM	1029	HG1	LYS	A	67	56.050	52.330	123.590	0.00	0.00
ATOM	1030	HG2	LYS	A	67	57.760	51.820	123.780	0.00	0.00
ATOM	1031	CD	LYS	A	67	56.990	53.030	125.400	0.00	0.00
ATOM	1032	HD1	LYS	A	67	57.770	52.680	126.110	0.00	0.00

```
ATOM   1033  HD2 LYS A  67      55.980  53.130 125.850  0.00  0.00
ATOM   1034  CE  LYS A  67      57.650  54.260 124.730  0.00  0.00
ATOM   1035  HE1 LYS A  67      56.830  54.630 124.090  0.00  0.00
ATOM   1036  HE2 LYS A  67      58.580  53.960 124.200  0.00  0.00
ATOM   1037  NZ  LYS A  67      57.950  55.280 125.700  0.00  0.00
ATOM   1038  HZ1 LYS A  67      57.110  55.480 126.280  0.00  0.00
ATOM   1039  HZ2 LYS A  67      58.200  56.150 125.180  0.00  0.00
ATOM   1040  HZ3 LYS A  67      58.790  55.010 126.230  0.00  0.00
ATOM   1041  C   LYS A  67      56.760  48.750 126.560  0.00  0.00
ATOM   1042  O   LYS A  67      56.860  47.700 126.040  0.00  0.00
ATOM   1043  N   LYS A  68      56.110  48.910 127.700  0.00  0.00
ATOM   1044  HN  LYS A  68      55.820  49.770 128.120  0.00  0.00
ATOM   1045  CA  LYS A  68      55.700  47.750 128.450  0.00  0.00
ATOM   1046  HA  LYS A  68      56.260  46.850 128.260  0.00  0.00
ATOM   1047  CB  LYS A  68      55.970  47.860 129.970  0.00  0.00
ATOM   1048  HB1 LYS A  68      55.610  47.010 130.590  0.00  0.00
ATOM   1049  HB2 LYS A  68      55.550  48.820 130.330  0.00  0.00
ATOM   1050  CG  LYS A  68      57.520  48.020 130.200  0.00  0.00
ATOM   1051  HG1 LYS A  68      57.660  48.460 131.210  0.00  0.00
ATOM   1052  HG2 LYS A  68      58.030  48.680 129.470  0.00  0.00
ATOM   1053  CD  LYS A  68      58.170  46.670 130.160  0.00  0.00
ATOM   1054  HD1 LYS A  68      58.130  46.160 129.170  0.00  0.00
ATOM   1055  HD2 LYS A  68      57.730  45.950 130.880  0.00  0.00
ATOM   1056  CE  LYS A  68      59.620  46.910 130.690  0.00  0.00
ATOM   1057  HE1 LYS A  68      59.540  47.300 131.730  0.00  0.00
ATOM   1058  HE2 LYS A  68      60.210  47.570 130.030  0.00  0.00
ATOM   1059  NZ  LYS A  68      60.320  45.590 130.620  0.00  0.00
ATOM   1060  HZ1 LYS A  68      61.290  45.780 130.930  0.00  0.00
ATOM   1061  HZ2 LYS A  68      60.400  45.260 129.630  0.00  0.00
ATOM   1062  HZ3 LYS A  68      59.830  44.930 131.250  0.00  0.00
ATOM   1063  C   LYS A  68      54.210  47.500 128.310  0.00  0.00
ATOM   1064  O   LYS A  68      53.320  48.330 128.140  0.00  0.00
ATOM   1065  N   LEU A  69      53.730  46.250 128.400  0.00  0.00
ATOM   1066  HN  LEU A  69      54.430  45.540 128.410  0.00  0.00
ATOM   1067  CA  LEU A  69      52.390  45.730 128.110  0.00  0.00
ATOM   1068  HA  LEU A  69      52.500  44.670 128.260  0.00  0.00
ATOM   1069  CB  LEU A  69      51.260  46.260 129.030  0.00  0.00
ATOM   1070  HB1 LEU A  69      50.310  45.820 128.690  0.00  0.00
ATOM   1071  HB2 LEU A  69      51.210  47.310 128.680  0.00  0.00
ATOM   1072  CG  LEU A  69      51.520  46.060 130.530  0.00  0.00
ATOM   1073  HG  LEU A  69      52.460  46.570 130.810  0.00  0.00
ATOM   1074  CD1 LEU A  69      50.360  46.690 131.290  0.00  0.00
ATOM   1075 HD11 LEU A  69      50.520  46.530 132.380  0.00  0.00
ATOM   1076 HD12 LEU A  69      49.360  46.310 131.000  0.00  0.00
ATOM   1077 HD13 LEU A  69      50.350  47.790 131.170  0.00  0.00
ATOM   1078  CD2 LEU A  69      51.750  44.650 130.990  0.00  0.00
ATOM   1079 HD21 LEU A  69      51.660  44.630 132.100  0.00  0.00
ATOM   1080 HD22 LEU A  69      52.710  44.220 130.620  0.00  0.00
ATOM   1081 HD23 LEU A  69      50.960  43.970 130.610  0.00  0.00
ATOM   1082  C   LEU A  69      52.050  45.960 126.600  0.00  0.00
ATOM   1083  O   LEU A  69      50.970  46.500 126.250  0.00  0.00
ATOM   1084  N   ARG A  70      52.900  45.520 125.720  0.00  0.00
ATOM   1085  HN  ARG A  70      53.720  45.030 125.990  0.00  0.00
ATOM   1086  CA  ARG A  70      52.550  45.610 124.310  0.00  0.00
ATOM   1087  HA  ARG A  70      52.150  46.600 124.160  0.00  0.00
ATOM   1088  CB  ARG A  70      53.790  45.320 123.410  0.00  0.00
ATOM   1089  HB1 ARG A  70      53.550  45.120 122.350  0.00  0.00
ATOM   1090  HB2 ARG A  70      54.070  44.300 123.740  0.00  0.00
ATOM   1091  CG  ARG A  70      54.870  46.400 123.430  0.00  0.00
ATOM   1092  HG1 ARG A  70      55.410  46.340 124.390  0.00  0.00
ATOM   1093  HG2 ARG A  70      54.440  47.430 123.410  0.00  0.00
```

```
ATOM   1094  CD  ARG A  70      55.790  46.070 122.160  0.00  0.00
ATOM   1095  HD1 ARG A  70      56.680  46.730 122.080  0.00  0.00
ATOM   1096  HD2 ARG A  70      55.200  46.240 121.230  0.00  0.00
ATOM   1097  NE  ARG A  70      56.210  44.620 122.100  0.00  0.00
ATOM   1098  HE  ARG A  70      56.100  44.080 122.930  0.00  0.00
ATOM   1099  CZ  ARG A  70      56.860  43.950 121.130  0.00  0.00
ATOM   1100  NH1 ARG A  70      57.010  44.320 119.900  0.00  0.00
ATOM   1101 HH11 ARG A  70      56.600  45.070 119.370  0.00  0.00
ATOM   1102 HH12 ARG A  70      57.780  43.900 119.420  0.00  0.00
ATOM   1103  NH2 ARG A  70      57.440  42.800 121.450  0.00  0.00
ATOM   1104 HH21 ARG A  70      58.050  42.300 120.830  0.00  0.00
ATOM   1105 HH22 ARG A  70      57.190  42.480 122.370  0.00  0.00
ATOM   1106  C   ARG A  70      51.500  44.670 123.860  0.00  0.00
ATOM   1107  O   ARG A  70      51.450  43.500 124.200  0.00  0.00
ATOM   1108  N   SER A  71      50.610  45.130 122.980  0.00  0.00
ATOM   1109  HN  SER A  71      50.750  46.050 122.630  0.00  0.00
ATOM   1110  CA  SER A  71      49.540  44.310 122.530  0.00  0.00
ATOM   1111  HA  SER A  71      49.310  43.610 123.320  0.00  0.00
ATOM   1112  CB  SER A  71      48.220  45.220 122.230  0.00  0.00
ATOM   1113  HB1 SER A  71      48.130  45.750 123.210  0.00  0.00
ATOM   1114  HB2 SER A  71      47.320  44.610 122.000  0.00  0.00
ATOM   1115  OG  SER A  71      48.290  46.210 121.170  0.00  0.00
ATOM   1116  HG1 SER A  71      48.960  46.820 121.470  0.00  0.00
ATOM   1117  C   SER A  71      49.930  43.540 121.270  0.00  0.00
ATOM   1118  O   SER A  71      51.010  43.720 120.690  0.00  0.00
ATOM   1119  N   MET A  72      49.030  42.560 120.900  0.00  0.00
ATOM   1120  HN  MET A  72      48.240  42.430 121.490  0.00  0.00
ATOM   1121  CA  MET A  72      49.150  41.800 119.680  0.00  0.00
ATOM   1122  HA  MET A  72      50.080  41.250 119.710  0.00  0.00
ATOM   1123  CB  MET A  72      48.040  40.720 119.610  0.00  0.00
ATOM   1124  HB1 MET A  72      47.930  40.120 118.690  0.00  0.00
ATOM   1125  HB2 MET A  72      47.030  41.160 119.740  0.00  0.00
ATOM   1126  CG  MET A  72      48.290  39.700 120.690  0.00  0.00
ATOM   1127  HG1 MET A  72      47.340  39.140 120.760  0.00  0.00
ATOM   1128  HG2 MET A  72      48.460  40.230 121.650  0.00  0.00
ATOM   1129  SD  MET A  72      49.800  38.590 120.420  0.00  0.00
ATOM   1130  CE  MET A  72      49.490  37.400 121.790  0.00  0.00
ATOM   1131  HE1 MET A  72      50.040  36.460 121.570  0.00  0.00
ATOM   1132  HE2 MET A  72      48.410  37.220 121.980  0.00  0.00
ATOM   1133  HE3 MET A  72      50.070  37.870 122.620  0.00  0.00
ATOM   1134  C   MET A  72      49.110  42.700 118.400  0.00  0.00
ATOM   1135  O   MET A  72      49.740  42.450 117.370  0.00  0.00
ATOM   1136  N   THR A  73      48.210  43.720 118.410  0.00  0.00
ATOM   1137  HN  THR A  73      47.560  43.660 119.160  0.00  0.00
ATOM   1138  CA  THR A  73      48.160  44.800 117.400  0.00  0.00
ATOM   1139  HA  THR A  73      48.110  44.610 116.340  0.00  0.00
ATOM   1140  CB  THR A  73      47.000  45.710 117.660  0.00  0.00
ATOM   1141  HB  THR A  73      46.940  46.030 118.720  0.00  0.00
ATOM   1142  OG1 THR A  73      45.740  45.050 117.370  0.00  0.00
ATOM   1143  HG1 THR A  73      45.660  44.290 117.950  0.00  0.00
ATOM   1144  CG2 THR A  73      46.920  46.900 116.680  0.00  0.00
ATOM   1145 HG21 THR A  73      46.520  46.550 115.700  0.00  0.00
ATOM   1146 HG22 THR A  73      47.870  47.460 116.650  0.00  0.00
ATOM   1147 HG23 THR A  73      46.090  47.570 116.980  0.00  0.00
ATOM   1148  C   THR A  73      49.410  45.640 117.380  0.00  0.00
ATOM   1149  O   THR A  73      49.870  46.030 116.320  0.00  0.00
ATOM   1150  N   ASP A  74      50.050  45.960 118.530  0.00  0.00
ATOM   1151  HN  ASP A  74      49.630  45.630 119.370  0.00  0.00
ATOM   1152  CA  ASP A  74      51.390  46.610 118.580  0.00  0.00
ATOM   1153  HA  ASP A  74      51.340  47.560 118.080  0.00  0.00
ATOM   1154  CB  ASP A  74      51.850  46.860 120.020  0.00  0.00
```

ATOM	1155	HB1	ASP	A	74	52.880	47.230	120.180	0.00	0.00
ATOM	1156	HB2	ASP	A	74	51.750	45.960	120.670	0.00	0.00
ATOM	1157	CG	ASP	A	74	50.940	47.790	120.760	0.00	0.00
ATOM	1158	OD1	ASP	A	74	50.870	47.610	122.030	0.00	0.00
ATOM	1159	OD2	ASP	A	74	50.440	48.790	120.210	0.00	0.00
ATOM	1160	C	ASP	A	74	52.360	45.760	117.810	0.00	0.00
ATOM	1161	O	ASP	A	74	53.030	46.190	116.920	0.00	0.00
ATOM	1162	N	LYS	A	75	52.360	44.490	118.010	0.00	0.00
ATOM	1163	HN	LYS	A	75	51.660	44.110	118.620	0.00	0.00
ATOM	1164	CA	LYS	A	75	53.070	43.510	117.260	0.00	0.00
ATOM	1165	HA	LYS	A	75	54.080	43.860	117.370	0.00	0.00
ATOM	1166	CB	LYS	A	75	52.910	42.080	117.910	0.00	0.00
ATOM	1167	HB1	LYS	A	75	53.250	41.200	117.320	0.00	0.00
ATOM	1168	HB2	LYS	A	75	51.840	41.870	118.090	0.00	0.00
ATOM	1169	CG	LYS	A	75	53.670	42.090	119.250	0.00	0.00
ATOM	1170	HG1	LYS	A	75	53.520	43.040	119.810	0.00	0.00
ATOM	1171	HG2	LYS	A	75	54.740	42.110	118.960	0.00	0.00
ATOM	1172	CD	LYS	A	75	53.290	40.930	120.150	0.00	0.00
ATOM	1173	HD1	LYS	A	75	53.400	40.050	119.480	0.00	0.00
ATOM	1174	HD2	LYS	A	75	52.260	41.100	120.530	0.00	0.00
ATOM	1175	CE	LYS	A	75	54.360	40.760	121.300	0.00	0.00
ATOM	1176	HE1	LYS	A	75	54.600	41.740	121.750	0.00	0.00
ATOM	1177	HE2	LYS	A	75	55.290	40.250	120.960	0.00	0.00
ATOM	1178	NZ	LYS	A	75	53.800	39.930	122.450	0.00	0.00
ATOM	1179	HZ1	LYS	A	75	54.520	39.770	123.180	0.00	0.00
ATOM	1180	HZ2	LYS	A	75	53.570	39.020	121.990	0.00	0.00
ATOM	1181	HZ3	LYS	A	75	52.940	40.430	122.750	0.00	0.00
ATOM	1182	C	LYS	A	75	52.770	43.490	115.710	0.00	0.00
ATOM	1183	O	LYS	A	75	53.690	43.630	114.860	0.00	0.00
ATOM	1184	N	TYR	A	76	51.500	43.420	115.240	0.00	0.00
ATOM	1185	HN	TYR	A	76	50.830	43.280	115.960	0.00	0.00
ATOM	1186	CA	TYR	A	76	51.080	43.410	113.850	0.00	0.00
ATOM	1187	HA	TYR	A	76	51.610	42.600	113.380	0.00	0.00
ATOM	1188	CB	TYR	A	76	49.590	43.220	113.670	0.00	0.00
ATOM	1189	HB1	TYR	A	76	49.360	43.100	112.590	0.00	0.00
ATOM	1190	HB2	TYR	A	76	49.010	44.020	114.180	0.00	0.00
ATOM	1191	CG	TYR	A	76	49.180	41.900	114.330	0.00	0.00
ATOM	1192	CD1	TYR	A	76	50.070	40.810	114.440	0.00	0.00
ATOM	1193	HD1	TYR	A	76	51.090	40.900	114.100	0.00	0.00
ATOM	1194	CE1	TYR	A	76	49.620	39.610	115.070	0.00	0.00
ATOM	1195	HE1	TYR	A	76	50.370	38.840	115.130	0.00	0.00
ATOM	1196	CZ	TYR	A	76	48.380	39.500	115.750	0.00	0.00
ATOM	1197	OH	TYR	A	76	48.260	38.340	116.520	0.00	0.00
ATOM	1198	HH	TYR	A	76	47.490	38.410	117.080	0.00	0.00
ATOM	1199	CD2	TYR	A	76	47.870	41.750	114.940	0.00	0.00
ATOM	1200	HD2	TYR	A	76	47.100	42.490	114.790	0.00	0.00
ATOM	1201	CE2	TYR	A	76	47.580	40.530	115.680	0.00	0.00
ATOM	1202	HE2	TYR	A	76	46.610	40.430	116.140	0.00	0.00
ATOM	1203	C	TYR	A	76	51.420	44.630	113.140	0.00	0.00
ATOM	1204	O	TYR	A	76	51.820	44.520	112.000	0.00	0.00
ATOM	1205	N	ARG	A	77	51.310	45.780	113.770	0.00	0.00
ATOM	1206	HN	ARG	A	77	50.840	45.900	114.640	0.00	0.00
ATOM	1207	CA	ARG	A	77	51.770	47.010	113.200	0.00	0.00
ATOM	1208	HA	ARG	A	77	51.260	47.200	112.270	0.00	0.00
ATOM	1209	CB	ARG	A	77	51.400	48.210	114.110	0.00	0.00
ATOM	1210	HB1	ARG	A	77	51.880	49.140	113.720	0.00	0.00
ATOM	1211	HB2	ARG	A	77	51.790	48.160	115.150	0.00	0.00
ATOM	1212	CG	ARG	A	77	49.880	48.540	113.950	0.00	0.00
ATOM	1213	HG1	ARG	A	77	49.360	47.680	114.420	0.00	0.00
ATOM	1214	HG2	ARG	A	77	49.650	48.490	112.870	0.00	0.00
ATOM	1215	CD	ARG	A	77	49.550	49.900	114.570	0.00	0.00

```
ATOM   1216  HD1 ARG A  77      50.150  50.730 114.140  0.00  0.00
ATOM   1217  HD2 ARG A  77      49.780  49.810 115.650  0.00  0.00
ATOM   1218  NE  ARG A  77      48.050  50.020 114.370  0.00  0.00
ATOM   1219  HE  ARG A  77      47.740  49.930 113.430  0.00  0.00
ATOM   1220  CZ  ARG A  77      47.260  50.600 115.260  0.00  0.00
ATOM   1221  NH1 ARG A  77      47.540  50.980 116.550  0.00  0.00
ATOM   1222 HH11 ARG A  77      48.240  50.530 117.100  0.00  0.00
ATOM   1223 HH12 ARG A  77      46.900  51.590 117.030  0.00  0.00
ATOM   1224  NH2 ARG A  77      46.010  50.840 114.850  0.00  0.00
ATOM   1225 HH21 ARG A  77      45.740  50.390 114.000  0.00  0.00
ATOM   1226 HH22 ARG A  77      45.400  51.410 115.390  0.00  0.00
ATOM   1227  C   ARG A  77      53.270  47.050 112.930  0.00  0.00
ATOM   1228  O   ARG A  77      53.730  47.600 111.930  0.00  0.00
ATOM   1229  N   LEU A  78      54.130  46.610 113.850  0.00  0.00
ATOM   1230  HN  LEU A  78      53.880  46.220 114.730  0.00  0.00
ATOM   1231  CA  LEU A  78      55.600  46.550 113.580  0.00  0.00
ATOM   1232  HA  LEU A  78      55.930  47.560 113.350  0.00  0.00
ATOM   1233  CB  LEU A  78      56.430  46.160 114.830  0.00  0.00
ATOM   1234  HB1 LEU A  78      56.090  45.180 115.210  0.00  0.00
ATOM   1235  HB2 LEU A  78      56.210  46.950 115.570  0.00  0.00
ATOM   1236  CG  LEU A  78      57.940  46.050 114.710  0.00  0.00
ATOM   1237  HG  LEU A  78      58.260  45.590 113.760  0.00  0.00
ATOM   1238  CD1 LEU A  78      58.660  47.410 114.670  0.00  0.00
ATOM   1239 HD11 LEU A  78      59.740  47.200 114.490  0.00  0.00
ATOM   1240 HD12 LEU A  78      58.570  47.900 115.660  0.00  0.00
ATOM   1241 HD13 LEU A  78      58.200  47.970 113.830  0.00  0.00
ATOM   1242  CD2 LEU A  78      58.590  45.180 115.790  0.00  0.00
ATOM   1243 HD21 LEU A  78      57.930  44.350 116.130  0.00  0.00
ATOM   1244 HD22 LEU A  78      58.730  45.880 116.640  0.00  0.00
ATOM   1245 HD23 LEU A  78      59.600  44.860 115.470  0.00  0.00
ATOM   1246  C   LEU A  78      55.990  45.610 112.430  0.00  0.00
ATOM   1247  O   LEU A  78      56.860  45.860 111.610  0.00  0.00
ATOM   1248  N   HSD A  79      55.310  44.430 112.390  0.00  0.00
ATOM   1249  HN  HSD A  79      54.530  44.140 112.940  0.00  0.00
ATOM   1250  CA  HSD A  79      55.320  43.570 111.180  0.00  0.00
ATOM   1251  HA  HSD A  79      56.340  43.230 111.100  0.00  0.00
ATOM   1252  CB  HSD A  79      54.400  42.270 111.300  0.00  0.00
ATOM   1253  HB1 HSD A  79      54.440  41.680 110.360  0.00  0.00
ATOM   1254  HB2 HSD A  79      53.360  42.660 111.320  0.00  0.00
ATOM   1255  ND1 HSD A  79      54.270  41.320 113.630  0.00  0.00
ATOM   1256  HD1 HSD A  79      53.780  42.130 113.950  0.00  0.00
ATOM   1257  CG  HSD A  79      54.680  41.210 112.310  0.00  0.00
ATOM   1258  CE1 HSD A  79      54.710  40.230 114.260  0.00  0.00
ATOM   1259  HE1 HSD A  79      54.430  39.930 115.270  0.00  0.00
ATOM   1260  NE2 HSD A  79      55.320  39.380 113.460  0.00  0.00
ATOM   1261  CD2 HSD A  79      55.390  40.020 112.260  0.00  0.00
ATOM   1262  HD2 HSD A  79      55.950  39.640 111.410  0.00  0.00
ATOM   1263  C   HSD A  79      55.000  44.190 109.810  0.00  0.00
ATOM   1264  O   HSD A  79      55.820  44.080 108.910  0.00  0.00
ATOM   1265  N   LEU A  80      53.870  44.920 109.700  0.00  0.00
ATOM   1266  HN  LEU A  80      53.140  45.000 110.380  0.00  0.00
ATOM   1267  CA  LEU A  80      53.620  45.740 108.600  0.00  0.00
ATOM   1268  HA  LEU A  80      53.640  45.030 107.790  0.00  0.00
ATOM   1269  CB  LEU A  80      52.140  46.200 108.790  0.00  0.00
ATOM   1270  HB1 LEU A  80      52.000  46.770 109.730  0.00  0.00
ATOM   1271  HB2 LEU A  80      51.450  45.330 108.890  0.00  0.00
ATOM   1272  CG  LEU A  80      51.560  47.110 107.670  0.00  0.00
ATOM   1273  HG  LEU A  80      52.250  47.970 107.530  0.00  0.00
ATOM   1274  CD1 LEU A  80      51.420  46.380 106.270  0.00  0.00
ATOM   1275 HD11 LEU A  80      50.850  47.010 105.560  0.00  0.00
ATOM   1276 HD12 LEU A  80      50.810  45.470 106.410  0.00  0.00
```

ATOM 1277 HD13 LEU A 80 52.420 46.040 105.910 0.00 0.00
ATOM 1278 CD2 LEU A 80 50.250 47.700 108.200 0.00 0.00
ATOM 1279 HD21 LEU A 80 49.840 48.300 107.360 0.00 0.00
ATOM 1280 HD22 LEU A 80 50.530 48.360 109.050 0.00 0.00
ATOM 1281 HD23 LEU A 80 49.630 46.830 108.510 0.00 0.00
ATOM 1282 C LEU A 80 54.580 46.880 108.340 0.00 0.00
ATOM 1283 O LEU A 80 54.770 47.200 107.160 0.00 0.00
ATOM 1284 N SER A 81 55.130 47.530 109.360 0.00 0.00
ATOM 1285 HN SER A 81 54.760 47.220 110.240 0.00 0.00
ATOM 1286 CA SER A 81 55.960 48.740 109.290 0.00 0.00
ATOM 1287 HA SER A 81 55.460 49.530 108.750 0.00 0.00
ATOM 1288 CB SER A 81 56.400 49.210 110.690 0.00 0.00
ATOM 1289 HB1 SER A 81 57.040 50.120 110.640 0.00 0.00
ATOM 1290 HB2 SER A 81 56.950 48.410 111.230 0.00 0.00
ATOM 1291 OG SER A 81 55.190 49.630 111.370 0.00 0.00
ATOM 1292 HG1 SER A 81 54.620 48.860 111.440 0.00 0.00
ATOM 1293 C SER A 81 57.260 48.580 108.540 0.00 0.00
ATOM 1294 O SER A 81 57.600 49.410 107.720 0.00 0.00
ATOM 1295 N VAL A 82 57.920 47.410 108.770 0.00 0.00
ATOM 1296 HN VAL A 82 57.630 46.830 109.530 0.00 0.00
ATOM 1297 CA VAL A 82 59.050 47.010 107.900 0.00 0.00
ATOM 1298 HA VAL A 82 59.780 47.800 107.950 0.00 0.00
ATOM 1299 CB VAL A 82 59.660 45.710 108.350 0.00 0.00
ATOM 1300 HB VAL A 82 58.780 45.040 108.180 0.00 0.00
ATOM 1301 CG1 VAL A 82 60.820 45.120 107.470 0.00 0.00
ATOM 1302 HG11 VAL A 82 61.270 44.280 108.040 0.00 0.00
ATOM 1303 HG12 VAL A 82 61.620 45.850 107.240 0.00 0.00
ATOM 1304 HG13 VAL A 82 60.380 44.670 106.550 0.00 0.00
ATOM 1305 CG2 VAL A 82 60.170 45.850 109.870 0.00 0.00
ATOM 1306 HG21 VAL A 82 60.980 46.610 109.890 0.00 0.00
ATOM 1307 HG22 VAL A 82 60.570 44.850 110.140 0.00 0.00
ATOM 1308 HG23 VAL A 82 59.380 46.190 110.560 0.00 0.00
ATOM 1309 C VAL A 82 58.720 46.830 106.360 0.00 0.00
ATOM 1310 O VAL A 82 59.500 47.300 105.540 0.00 0.00
ATOM 1311 N ALA A 83 57.600 46.200 106.020 0.00 0.00
ATOM 1312 HN ALA A 83 57.040 45.880 106.780 0.00 0.00
ATOM 1313 CA ALA A 83 57.260 45.960 104.630 0.00 0.00
ATOM 1314 HA ALA A 83 58.160 45.610 104.150 0.00 0.00
ATOM 1315 CB ALA A 83 56.160 44.910 104.380 0.00 0.00
ATOM 1316 HB1 ALA A 83 56.130 44.810 103.270 0.00 0.00
ATOM 1317 HB2 ALA A 83 55.140 45.170 104.720 0.00 0.00
ATOM 1318 HB3 ALA A 83 56.530 43.970 104.840 0.00 0.00
ATOM 1319 C ALA A 83 56.910 47.200 103.850 0.00 0.00
ATOM 1320 O ALA A 83 57.470 47.470 102.760 0.00 0.00
ATOM 1321 N ASP A 84 56.080 48.080 104.450 0.00 0.00
ATOM 1322 HN ASP A 84 55.560 47.830 105.260 0.00 0.00
ATOM 1323 CA ASP A 84 55.780 49.450 103.920 0.00 0.00
ATOM 1324 HA ASP A 84 55.310 49.350 102.950 0.00 0.00
ATOM 1325 CB ASP A 84 54.920 50.150 104.950 0.00 0.00
ATOM 1326 HB1 ASP A 84 54.930 51.240 104.760 0.00 0.00
ATOM 1327 HB2 ASP A 84 55.320 50.080 105.980 0.00 0.00
ATOM 1328 CG ASP A 84 53.530 49.830 104.850 0.00 0.00
ATOM 1329 OD1 ASP A 84 53.080 49.170 103.820 0.00 0.00
ATOM 1330 OD2 ASP A 84 52.760 50.230 105.770 0.00 0.00
ATOM 1331 C ASP A 84 57.050 50.290 103.730 0.00 0.00
ATOM 1332 O ASP A 84 57.140 50.980 102.720 0.00 0.00
ATOM 1333 N LEU A 85 58.030 50.190 104.740 0.00 0.00
ATOM 1334 HN LEU A 85 57.860 49.640 105.560 0.00 0.00
ATOM 1335 CA LEU A 85 59.250 50.860 104.560 0.00 0.00
ATOM 1336 HA LEU A 85 59.050 51.910 104.420 0.00 0.00
ATOM 1337 CB LEU A 85 60.050 50.710 105.820 0.00 0.00

ATOM	1338	HB1	LEU	A	85	60.220	49.640	106.090	0.00	0.00
ATOM	1339	HB2	LEU	A	85	59.330	51.010	106.620	0.00	0.00
ATOM	1340	CG	LEU	A	85	61.260	51.620	105.840	0.00	0.00
ATOM	1341	HG	LEU	A	85	62.050	51.310	105.130	0.00	0.00
ATOM	1342	CD1	LEU	A	85	60.900	53.130	105.570	0.00	0.00
ATOM	1343	HD11	LEU	A	85	59.830	53.300	105.820	0.00	0.00
ATOM	1344	HD12	LEU	A	85	60.870	53.410	104.490	0.00	0.00
ATOM	1345	HD13	LEU	A	85	61.510	53.830	106.180	0.00	0.00
ATOM	1346	CD2	LEU	A	85	61.830	51.590	107.280	0.00	0.00
ATOM	1347	HD21	LEU	A	85	62.010	50.550	107.640	0.00	0.00
ATOM	1348	HD22	LEU	A	85	60.980	51.930	107.910	0.00	0.00
ATOM	1349	HD23	LEU	A	85	62.730	52.220	107.450	0.00	0.00
ATOM	1350	C	LEU	A	85	60.100	50.420	103.330	0.00	0.00
ATOM	1351	O	LEU	A	85	60.600	51.270	102.620	0.00	0.00
ATOM	1352	N	LEU	A	86	60.160	49.080	103.070	0.00	0.00
ATOM	1353	HN	LEU	A	86	59.810	48.360	103.660	0.00	0.00
ATOM	1354	CA	LEU	A	86	60.770	48.480	101.910	0.00	0.00
ATOM	1355	HA	LEU	A	86	61.810	48.760	101.980	0.00	0.00
ATOM	1356	CB	LEU	A	86	60.660	46.940	102.050	0.00	0.00
ATOM	1357	HB1	LEU	A	86	59.600	46.640	101.940	0.00	0.00
ATOM	1358	HB2	LEU	A	86	60.990	46.710	103.090	0.00	0.00
ATOM	1359	CG	LEU	A	86	61.540	46.130	101.020	0.00	0.00
ATOM	1360	HG	LEU	A	86	61.760	46.730	100.110	0.00	0.00
ATOM	1361	CD1	LEU	A	86	62.870	45.860	101.760	0.00	0.00
ATOM	1362	HD11	LEU	A	86	63.560	45.420	101.010	0.00	0.00
ATOM	1363	HD12	LEU	A	86	62.780	45.130	102.590	0.00	0.00
ATOM	1364	HD13	LEU	A	86	63.370	46.790	102.100	0.00	0.00
ATOM	1365	CD2	LEU	A	86	60.880	44.810	100.540	0.00	0.00
ATOM	1366	HD21	LEU	A	86	60.390	44.400	101.440	0.00	0.00
ATOM	1367	HD22	LEU	A	86	61.600	44.060	100.130	0.00	0.00
ATOM	1368	HD23	LEU	A	86	60.110	45.130	99.800	0.00	0.00
ATOM	1369	C	LEU	A	86	60.280	48.960	100.570	0.00	0.00
ATOM	1370	O	LEU	A	86	61.050	49.420	99.710	0.00	0.00
ATOM	1371	N	PHE	A	87	58.900	49.000	100.400	0.00	0.00
ATOM	1372	HN	PHE	A	87	58.350	48.670	101.150	0.00	0.00
ATOM	1373	CA	PHE	A	87	58.110	49.630	99.400	0.00	0.00
ATOM	1374	HA	PHE	A	87	58.470	49.310	98.430	0.00	0.00
ATOM	1375	CB	PHE	A	87	56.590	49.260	99.690	0.00	0.00
ATOM	1376	HB1	PHE	A	87	56.300	49.630	100.690	0.00	0.00
ATOM	1377	HB2	PHE	A	87	56.530	48.150	99.660	0.00	0.00
ATOM	1378	CG	PHE	A	87	55.550	49.870	98.760	0.00	0.00
ATOM	1379	CD1	PHE	A	87	55.190	51.240	98.770	0.00	0.00
ATOM	1380	HD1	PHE	A	87	55.600	51.830	99.580	0.00	0.00
ATOM	1381	CE1	PHE	A	87	54.400	51.760	97.790	0.00	0.00
ATOM	1382	HE1	PHE	A	87	54.190	52.810	97.950	0.00	0.00
ATOM	1383	CZ	PHE	A	87	53.870	50.930	96.790	0.00	0.00
ATOM	1384	HZ	PHE	A	87	53.150	51.380	96.130	0.00	0.00
ATOM	1385	CD2	PHE	A	87	54.990	49.000	97.810	0.00	0.00
ATOM	1386	HD2	PHE	A	87	55.020	47.940	98.010	0.00	0.00
ATOM	1387	CE2	PHE	A	87	54.170	49.590	96.820	0.00	0.00
ATOM	1388	HE2	PHE	A	87	53.770	48.900	96.090	0.00	0.00
ATOM	1389	C	PHE	A	87	58.350	51.150	99.270	0.00	0.00
ATOM	1390	O	PHE	A	87	58.460	51.700	98.130	0.00	0.00
ATOM	1391	N	VAL	A	88	58.460	51.850	100.360	0.00	0.00
ATOM	1392	HN	VAL	A	88	58.370	51.430	101.260	0.00	0.00
ATOM	1393	CA	VAL	A	88	58.740	53.280	100.340	0.00	0.00
ATOM	1394	HA	VAL	A	88	58.050	53.850	99.730	0.00	0.00
ATOM	1395	CB	VAL	A	88	58.500	53.850	101.750	0.00	0.00
ATOM	1396	HB	VAL	A	88	58.960	53.260	102.560	0.00	0.00
ATOM	1397	CG1	VAL	A	88	59.120	55.280	101.810	0.00	0.00
ATOM	1398	HG11	VAL	A	88	58.850	55.860	100.900	0.00	0.00

ATOM 1399 HG12 VAL A 88 60.220 55.170 101.900 0.00 0.00
ATOM 1400 HG13 VAL A 88 58.940 55.870 102.740 0.00 0.00
ATOM 1401 CG2 VAL A 88 57.020 53.930 102.080 0.00 0.00
ATOM 1402 HG21 VAL A 88 56.800 54.220 103.130 0.00 0.00
ATOM 1403 HG22 VAL A 88 56.440 53.030 101.760 0.00 0.00
ATOM 1404 HG23 VAL A 88 56.460 54.670 101.470 0.00 0.00
ATOM 1405 C VAL A 88 60.120 53.630 99.810 0.00 0.00
ATOM 1406 O VAL A 88 60.330 54.550 98.990 0.00 0.00
ATOM 1407 N ILE A 89 61.140 52.890 100.180 0.00 0.00
ATOM 1408 HN ILE A 89 61.020 52.160 100.850 0.00 0.00
ATOM 1409 CA ILE A 89 62.390 52.960 99.480 0.00 0.00
ATOM 1410 HA ILE A 89 62.510 54.010 99.290 0.00 0.00
ATOM 1411 CB ILE A 89 63.610 52.570 100.270 0.00 0.00
ATOM 1412 HB ILE A 89 64.520 52.960 99.760 0.00 0.00
ATOM 1413 CG2 ILE A 89 63.560 53.380 101.580 0.00 0.00
ATOM 1414 HG21 ILE A 89 64.470 53.340 102.220 0.00 0.00
ATOM 1415 HG22 ILE A 89 62.770 53.020 102.280 0.00 0.00
ATOM 1416 HG23 ILE A 89 63.310 54.430 101.350 0.00 0.00
ATOM 1417 CG1 ILE A 89 63.800 51.050 100.380 0.00 0.00
ATOM 1418 HG11 ILE A 89 63.840 50.740 99.310 0.00 0.00
ATOM 1419 HG12 ILE A 89 62.980 50.580 100.960 0.00 0.00
ATOM 1420 CD ILE A 89 65.090 50.710 100.910 0.00 0.00
ATOM 1421 HD1 ILE A 89 65.180 49.610 100.990 0.00 0.00
ATOM 1422 HD2 ILE A 89 65.230 51.150 101.920 0.00 0.00
ATOM 1423 HD3 ILE A 89 65.870 51.100 100.220 0.00 0.00
ATOM 1424 C ILE A 89 62.350 52.340 98.020 0.00 0.00
ATOM 1425 O ILE A 89 63.240 52.620 97.220 0.00 0.00
ATOM 1426 N THR A 90 61.250 51.710 97.630 0.00 0.00
ATOM 1427 HN THR A 90 60.530 51.630 98.320 0.00 0.00
ATOM 1428 CA THR A 90 61.050 51.170 96.330 0.00 0.00
ATOM 1429 HA THR A 90 62.050 50.910 95.990 0.00 0.00
ATOM 1430 CB THR A 90 60.270 49.850 96.340 0.00 0.00
ATOM 1431 HB THR A 90 59.180 49.990 96.520 0.00 0.00
ATOM 1432 OG1 THR A 90 60.880 48.860 97.190 0.00 0.00
ATOM 1433 HG1 THR A 90 60.950 49.200 98.090 0.00 0.00
ATOM 1434 CG2 THR A 90 60.350 49.170 94.950 0.00 0.00
ATOM 1435 HG21 THR A 90 61.430 49.070 94.700 0.00 0.00
ATOM 1436 HG22 THR A 90 59.800 49.720 94.150 0.00 0.00
ATOM 1437 HG23 THR A 90 60.030 48.110 95.040 0.00 0.00
ATOM 1438 C THR A 90 60.500 52.160 95.300 0.00 0.00
ATOM 1439 O THR A 90 61.060 52.170 94.160 0.00 0.00
ATOM 1440 N LEU A 91 59.590 53.010 95.760 0.00 0.00
ATOM 1441 HN LEU A 91 59.150 52.950 96.660 0.00 0.00
ATOM 1442 CA LEU A 91 59.030 54.120 94.940 0.00 0.00
ATOM 1443 HA LEU A 91 58.280 53.790 94.240 0.00 0.00
ATOM 1444 CB LEU A 91 58.230 55.240 95.720 0.00 0.00
ATOM 1445 HB1 LEU A 91 57.790 56.070 95.130 0.00 0.00
ATOM 1446 HB2 LEU A 91 58.930 55.720 96.450 0.00 0.00
ATOM 1447 CG LEU A 91 57.050 54.710 96.520 0.00 0.00
ATOM 1448 HG LEU A 91 57.190 53.620 96.720 0.00 0.00
ATOM 1449 CD1 LEU A 91 56.870 55.410 97.830 0.00 0.00
ATOM 1450 HD11 LEU A 91 56.520 56.450 97.660 0.00 0.00
ATOM 1451 HD12 LEU A 91 57.850 55.410 98.360 0.00 0.00
ATOM 1452 HD13 LEU A 91 56.180 54.820 98.470 0.00 0.00
ATOM 1453 CD2 LEU A 91 55.680 54.890 95.730 0.00 0.00
ATOM 1454 HD21 LEU A 91 54.850 54.490 96.330 0.00 0.00
ATOM 1455 HD22 LEU A 91 55.660 54.360 94.750 0.00 0.00
ATOM 1456 HD23 LEU A 91 55.640 55.990 95.560 0.00 0.00
ATOM 1457 C LEU A 91 59.930 54.920 94.080 0.00 0.00
ATOM 1458 O LEU A 91 59.460 55.180 92.970 0.00 0.00
ATOM 1459 N PRO A 92 61.260 55.280 94.350 0.00 0.00

ATOM	1460	CD	PRO	A	92	61.690	55.500	95.720	0.00	0.00
ATOM	1461	HD1	PRO	A	92	61.820	54.480	96.130	0.00	0.00
ATOM	1462	HD2	PRO	A	92	61.030	56.270	96.170	0.00	0.00
ATOM	1463	CA	PRO	A	92	62.090	56.120	93.430	0.00	0.00
ATOM	1464	HA	PRO	A	92	61.680	57.120	93.370	0.00	0.00
ATOM	1465	CB	PRO	A	92	63.470	56.140	94.090	0.00	0.00
ATOM	1466	HB1	PRO	A	92	64.270	56.830	93.730	0.00	0.00
ATOM	1467	HB2	PRO	A	92	64.030	55.200	93.910	0.00	0.00
ATOM	1468	CG	PRO	A	92	63.150	56.100	95.620	0.00	0.00
ATOM	1469	HG1	PRO	A	92	63.890	55.520	96.210	0.00	0.00
ATOM	1470	HG2	PRO	A	92	63.230	57.120	96.060	0.00	0.00
ATOM	1471	C	PRO	A	92	62.120	55.540	91.970	0.00	0.00
ATOM	1472	O	PRO	A	92	61.880	56.210	90.980	0.00	0.00
ATOM	1473	N	PHE	A	93	62.220	54.210	91.890	0.00	0.00
ATOM	1474	HN	PHE	A	93	62.170	53.720	92.760	0.00	0.00
ATOM	1475	CA	PHE	A	93	62.250	53.340	90.720	0.00	0.00
ATOM	1476	HA	PHE	A	93	63.110	53.750	90.210	0.00	0.00
ATOM	1477	CB	PHE	A	93	62.530	51.870	91.150	0.00	0.00
ATOM	1478	HB1	PHE	A	93	62.520	51.380	90.150	0.00	0.00
ATOM	1479	HB2	PHE	A	93	61.800	51.380	91.820	0.00	0.00
ATOM	1480	CG	PHE	A	93	63.950	51.810	91.740	0.00	0.00
ATOM	1481	CD1	PHE	A	93	64.170	51.540	93.140	0.00	0.00
ATOM	1482	HD1	PHE	A	93	63.320	51.210	93.710	0.00	0.00
ATOM	1483	CE1	PHE	A	93	65.500	51.420	93.530	0.00	0.00
ATOM	1484	HE1	PHE	A	93	65.780	51.160	94.540	0.00	0.00
ATOM	1485	CZ	PHE	A	93	66.610	51.670	92.690	0.00	0.00
ATOM	1486	HZ	PHE	A	93	67.620	51.440	92.990	0.00	0.00
ATOM	1487	CD2	PHE	A	93	65.030	52.000	90.920	0.00	0.00
ATOM	1488	HD2	PHE	A	93	64.880	52.150	89.860	0.00	0.00
ATOM	1489	CE2	PHE	A	93	66.360	51.860	91.360	0.00	0.00
ATOM	1490	HE2	PHE	A	93	67.210	51.920	90.690	0.00	0.00
ATOM	1491	C	PHE	A	93	60.980	53.450	89.960	0.00	0.00
ATOM	1492	O	PHE	A	93	60.950	53.520	88.730	0.00	0.00
ATOM	1493	N	TRP	A	94	59.850	53.410	90.700	0.00	0.00
ATOM	1494	HN	TRP	A	94	59.990	53.420	91.690	0.00	0.00
ATOM	1495	CA	TRP	A	94	58.530	53.510	90.110	0.00	0.00
ATOM	1496	HA	TRP	A	94	58.520	52.970	89.170	0.00	0.00
ATOM	1497	CB	TRP	A	94	57.390	52.880	91.040	0.00	0.00
ATOM	1498	HB1	TRP	A	94	56.400	52.830	90.550	0.00	0.00
ATOM	1499	HB2	TRP	A	94	57.260	53.460	91.970	0.00	0.00
ATOM	1500	CG	TRP	A	94	57.690	51.460	91.320	0.00	0.00
ATOM	1501	CD1	TRP	A	94	58.000	50.470	90.490	0.00	0.00
ATOM	1502	HD1	TRP	A	94	58.090	50.630	89.430	0.00	0.00
ATOM	1503	NE1	TRP	A	94	57.800	49.260	91.160	0.00	0.00
ATOM	1504	HE1	TRP	A	94	57.790	48.310	90.900	0.00	0.00
ATOM	1505	CE2	TRP	A	94	57.400	49.480	92.430	0.00	0.00
ATOM	1506	CD2	TRP	A	94	57.370	50.870	92.610	0.00	0.00
ATOM	1507	CE3	TRP	A	94	56.950	51.490	93.840	0.00	0.00
ATOM	1508	HE3	TRP	A	94	56.850	52.560	93.950	0.00	0.00
ATOM	1509	CZ3	TRP	A	94	56.770	50.600	94.920	0.00	0.00
ATOM	1510	HZ3	TRP	A	94	56.640	51.010	95.910	0.00	0.00
ATOM	1511	CZ2	TRP	A	94	57.090	48.670	93.490	0.00	0.00
ATOM	1512	HZ2	TRP	A	94	57.170	47.590	93.500	0.00	0.00
ATOM	1513	CH2	TRP	A	94	56.820	49.260	94.750	0.00	0.00
ATOM	1514	HH2	TRP	A	94	56.680	48.700	95.660	0.00	0.00
ATOM	1515	C	TRP	A	94	58.180	54.960	89.730	0.00	0.00
ATOM	1516	O	TRP	A	94	57.410	55.240	88.830	0.00	0.00
ATOM	1517	N	ALA	A	95	58.740	56.000	90.420	0.00	0.00
ATOM	1518	HN	ALA	A	95	59.210	55.780	91.270	0.00	0.00
ATOM	1519	CA	ALA	A	95	58.680	57.430	90.000	0.00	0.00
ATOM	1520	HA	ALA	A	95	57.640	57.530	89.710	0.00	0.00

ATOM	1521	CB	ALA	A	95	59.180	58.440	91.130	0.00	0.00
ATOM	1522	HB1	ALA	A	95	58.980	59.500	90.880	0.00	0.00
ATOM	1523	HB2	ALA	A	95	60.260	58.190	91.260	0.00	0.00
ATOM	1524	HB3	ALA	A	95	58.640	58.290	92.090	0.00	0.00
ATOM	1525	C	ALA	A	95	59.500	57.620	88.720	0.00	0.00
ATOM	1526	O	ALA	A	95	59.080	58.370	87.830	0.00	0.00
ATOM	1527	N	VAL	A	96	60.750	57.070	88.560	0.00	0.00
ATOM	1528	HN	VAL	A	96	61.170	56.540	89.290	0.00	0.00
ATOM	1529	CA	VAL	A	96	61.600	57.070	87.380	0.00	0.00
ATOM	1530	HA	VAL	A	96	61.920	58.090	87.220	0.00	0.00
ATOM	1531	CB	VAL	A	96	62.930	56.460	87.710	0.00	0.00
ATOM	1532	HB	VAL	A	96	62.810	55.480	88.220	0.00	0.00
ATOM	1533	CG1	VAL	A	96	63.770	56.200	86.440	0.00	0.00
ATOM	1534	HG11	VAL	A	96	63.720	57.080	85.770	0.00	0.00
ATOM	1535	HG12	VAL	A	96	63.370	55.300	85.920	0.00	0.00
ATOM	1536	HG13	VAL	A	96	64.840	55.960	86.640	0.00	0.00
ATOM	1537	CG2	VAL	A	96	63.850	57.290	88.660	0.00	0.00
ATOM	1538	HG21	VAL	A	96	64.730	56.690	88.970	0.00	0.00
ATOM	1539	HG22	VAL	A	96	63.230	57.690	89.490	0.00	0.00
ATOM	1540	HG23	VAL	A	96	64.300	58.120	88.080	0.00	0.00
ATOM	1541	C	VAL	A	96	60.940	56.390	86.190	0.00	0.00
ATOM	1542	O	VAL	A	96	60.900	56.880	85.080	0.00	0.00
ATOM	1543	N	ASP	A	97	60.380	55.240	86.340	0.00	0.00
ATOM	1544	HN	ASP	A	97	60.470	54.840	87.250	0.00	0.00
ATOM	1545	CA	ASP	A	97	59.530	54.510	85.370	0.00	0.00
ATOM	1546	HA	ASP	A	97	60.090	54.310	84.470	0.00	0.00
ATOM	1547	CB	ASP	A	97	59.140	53.170	86.060	0.00	0.00
ATOM	1548	HB1	ASP	A	97	58.990	53.340	87.150	0.00	0.00
ATOM	1549	HB2	ASP	A	97	60.090	52.600	86.010	0.00	0.00
ATOM	1550	CG	ASP	A	97	58.070	52.310	85.440	0.00	0.00
ATOM	1551	OD1	ASP	A	97	57.700	52.530	84.270	0.00	0.00
ATOM	1552	OD2	ASP	A	97	57.560	51.440	86.110	0.00	0.00
ATOM	1553	C	ASP	A	97	58.310	55.360	84.970	0.00	0.00
ATOM	1554	O	ASP	A	97	57.910	55.370	83.800	0.00	0.00
ATOM	1555	N	ALA	A	98	57.630	56.090	85.810	0.00	0.00
ATOM	1556	HN	ALA	A	98	57.960	56.090	86.750	0.00	0.00
ATOM	1557	CA	ALA	A	98	56.560	57.030	85.440	0.00	0.00
ATOM	1558	HA	ALA	A	98	55.750	56.510	84.950	0.00	0.00
ATOM	1559	CB	ALA	A	98	55.960	57.630	86.700	0.00	0.00
ATOM	1560	HB1	ALA	A	98	55.630	56.780	87.330	0.00	0.00
ATOM	1561	HB2	ALA	A	98	55.010	58.150	86.460	0.00	0.00
ATOM	1562	HB3	ALA	A	98	56.590	58.280	87.340	0.00	0.00
ATOM	1563	C	ALA	A	98	56.980	58.200	84.550	0.00	0.00
ATOM	1564	O	ALA	A	98	56.390	58.470	83.510	0.00	0.00
ATOM	1565	N	VAL	A	99	58.030	58.940	85.010	0.00	0.00
ATOM	1566	HN	VAL	A	99	58.440	58.710	85.890	0.00	0.00
ATOM	1567	CA	VAL	A	99	58.510	60.030	84.260	0.00	0.00
ATOM	1568	HA	VAL	A	99	57.700	60.670	83.950	0.00	0.00
ATOM	1569	CB	VAL	A	99	59.260	60.990	85.100	0.00	0.00
ATOM	1570	HB	VAL	A	99	60.030	60.420	85.670	0.00	0.00
ATOM	1571	CG1	VAL	A	99	59.870	62.090	84.270	0.00	0.00
ATOM	1572	HG11	VAL	A	99	59.180	62.740	83.680	0.00	0.00
ATOM	1573	HG12	VAL	A	99	60.690	61.760	83.590	0.00	0.00
ATOM	1574	HG13	VAL	A	99	60.280	62.860	84.950	0.00	0.00
ATOM	1575	CG2	VAL	A	99	58.430	61.630	86.250	0.00	0.00
ATOM	1576	HG21	VAL	A	99	57.510	62.100	85.840	0.00	0.00
ATOM	1577	HG22	VAL	A	99	58.980	62.440	86.770	0.00	0.00
ATOM	1578	HG23	VAL	A	99	58.100	60.790	86.900	0.00	0.00
ATOM	1579	C	VAL	A	99	59.260	59.650	83.000	0.00	0.00
ATOM	1580	O	VAL	A	99	59.000	60.230	81.980	0.00	0.00
ATOM	1581	N	ALA	A	100	60.140	58.580	83.040	0.00	0.00

ATOM	1582	HN	ALA	A	100	60.240	58.090	83.900	0.00	0.00
ATOM	1583	CA	ALA	A	100	61.020	58.230	81.950	0.00	0.00
ATOM	1584	HA	ALA	A	100	60.920	58.870	81.090	0.00	0.00
ATOM	1585	CB	ALA	A	100	62.530	58.500	82.360	0.00	0.00
ATOM	1586	HB1	ALA	A	100	63.110	58.410	81.420	0.00	0.00
ATOM	1587	HB2	ALA	A	100	62.880	57.680	83.010	0.00	0.00
ATOM	1588	HB3	ALA	A	100	62.680	59.500	82.820	0.00	0.00
ATOM	1589	C	ALA	A	100	60.800	56.790	81.380	0.00	0.00
ATOM	1590	O	ALA	A	100	59.950	56.570	80.440	0.00	0.00
ATOM	1591	N	ASN	A	101	61.460	55.740	81.930	0.00	0.00
ATOM	1592	HN	ASN	A	101	61.950	55.780	82.790	0.00	0.00
ATOM	1593	CA	ASN	A	101	61.470	54.490	81.260	0.00	0.00
ATOM	1594	HA	ASN	A	101	60.440	54.420	80.950	0.00	0.00
ATOM	1595	CB	ASN	A	101	62.490	54.470	80.060	0.00	0.00
ATOM	1596	HB1	ASN	A	101	63.520	54.670	80.440	0.00	0.00
ATOM	1597	HB2	ASN	A	101	62.190	55.330	79.420	0.00	0.00
ATOM	1598	CG	ASN	A	101	62.470	53.260	79.200	0.00	0.00
ATOM	1599	OD1	ASN	A	101	61.680	52.360	79.240	0.00	0.00
ATOM	1600	ND2	ASN	A	101	63.430	53.200	78.250	0.00	0.00
ATOM	1601	HD21	ASN	A	101	63.600	52.360	77.740	0.00	0.00
ATOM	1602	HD22	ASN	A	101	63.900	54.060	78.050	0.00	0.00
ATOM	1603	C	ASN	A	101	61.920	53.530	82.300	0.00	0.00
ATOM	1604	O	ASN	A	101	62.540	53.960	83.290	0.00	0.00
ATOM	1605	N	TRP	A	102	61.530	52.210	82.120	0.00	0.00
ATOM	1606	HN	TRP	A	102	61.060	51.900	81.290	0.00	0.00
ATOM	1607	CA	TRP	A	102	62.130	51.160	82.940	0.00	0.00
ATOM	1608	HA	TRP	A	102	62.150	51.540	83.950	0.00	0.00
ATOM	1609	CB	TRP	A	102	61.360	49.840	82.780	0.00	0.00
ATOM	1610	HB1	TRP	A	102	61.350	49.330	81.790	0.00	0.00
ATOM	1611	HB2	TRP	A	102	60.320	50.140	83.030	0.00	0.00
ATOM	1612	CG	TRP	A	102	61.780	48.860	83.860	0.00	0.00
ATOM	1613	CD1	TRP	A	102	62.640	47.850	83.730	0.00	0.00
ATOM	1614	HD1	TRP	A	102	63.190	47.700	82.820	0.00	0.00
ATOM	1615	NE1	TRP	A	102	62.970	47.330	84.960	0.00	0.00
ATOM	1616	HE1	TRP	A	102	63.640	46.650	85.150	0.00	0.00
ATOM	1617	CE2	TRP	A	102	62.220	47.940	85.890	0.00	0.00
ATOM	1618	CD2	TRP	A	102	61.360	48.930	85.240	0.00	0.00
ATOM	1619	CE3	TRP	A	102	60.510	49.710	86.070	0.00	0.00
ATOM	1620	HE3	TRP	A	102	59.900	50.460	85.620	0.00	0.00
ATOM	1621	CZ3	TRP	A	102	60.550	49.620	87.450	0.00	0.00
ATOM	1622	HZ3	TRP	A	102	59.910	50.250	88.040	0.00	0.00
ATOM	1623	CZ2	TRP	A	102	62.300	47.900	87.260	0.00	0.00
ATOM	1624	HZ2	TRP	A	102	63.020	47.290	87.770	0.00	0.00
ATOM	1625	CH2	TRP	A	102	61.400	48.690	88.050	0.00	0.00
ATOM	1626	HH2	TRP	A	102	61.390	48.660	89.130	0.00	0.00
ATOM	1627	C	TRP	A	102	63.500	50.960	82.480	0.00	0.00
ATOM	1628	O	TRP	A	102	63.860	50.410	81.440	0.00	0.00
ATOM	1629	N	TYR	A	103	64.460	51.360	83.360	0.00	0.00
ATOM	1630	HN	TYR	A	103	64.130	52.080	83.980	0.00	0.00
ATOM	1631	CA	TYR	A	103	65.880	51.170	83.150	0.00	0.00
ATOM	1632	HA	TYR	A	103	66.140	51.090	82.100	0.00	0.00
ATOM	1633	CB	TYR	A	103	66.550	52.490	83.660	0.00	0.00
ATOM	1634	HB1	TYR	A	103	67.650	52.630	83.650	0.00	0.00
ATOM	1635	HB2	TYR	A	103	66.220	52.670	84.710	0.00	0.00
ATOM	1636	CG	TYR	A	103	66.250	53.730	82.830	0.00	0.00
ATOM	1637	CD1	TYR	A	103	66.530	53.830	81.470	0.00	0.00
ATOM	1638	HD1	TYR	A	103	66.910	52.960	80.950	0.00	0.00
ATOM	1639	CE1	TYR	A	103	66.250	55.000	80.780	0.00	0.00
ATOM	1640	HE1	TYR	A	103	66.460	55.080	79.720	0.00	0.00
ATOM	1641	CZ	TYR	A	103	65.820	56.140	81.360	0.00	0.00
ATOM	1642	OH	TYR	A	103	65.470	57.370	80.750	0.00	0.00

ATOM	1643	HH	TYR	A	103	65.550	57.230	79.810	0.00	0.00
ATOM	1644	CD2	TYR	A	103	65.730	54.850	83.460	0.00	0.00
ATOM	1645	HD2	TYR	A	103	65.350	54.830	84.470	0.00	0.00
ATOM	1646	CE2	TYR	A	103	65.440	56.040	82.740	0.00	0.00
ATOM	1647	HE2	TYR	A	103	65.120	56.890	83.320	0.00	0.00
ATOM	1648	C	TYR	A	103	66.570	50.020	83.800	0.00	0.00
ATOM	1649	O	TYR	A	103	67.750	49.760	83.510	0.00	0.00
ATOM	1650	N	PHE	A	104	65.810	49.270	84.610	0.00	0.00
ATOM	1651	HN	PHE	A	104	64.880	49.520	84.850	0.00	0.00
ATOM	1652	CA	PHE	A	104	66.470	48.420	85.690	0.00	0.00
ATOM	1653	HA	PHE	A	104	67.540	48.570	85.770	0.00	0.00
ATOM	1654	CB	PHE	A	104	65.970	48.820	87.120	0.00	0.00
ATOM	1655	HB1	PHE	A	104	66.700	48.300	87.770	0.00	0.00
ATOM	1656	HB2	PHE	A	104	64.930	48.440	87.250	0.00	0.00
ATOM	1657	CG	PHE	A	104	65.990	50.320	87.270	0.00	0.00
ATOM	1658	CD1	PHE	A	104	67.170	50.980	87.630	0.00	0.00
ATOM	1659	HD1	PHE	A	104	68.050	50.390	87.840	0.00	0.00
ATOM	1660	CE1	PHE	A	104	67.330	52.420	87.650	0.00	0.00
ATOM	1661	HE1	PHE	A	104	68.220	52.830	88.100	0.00	0.00
ATOM	1662	CZ	PHE	A	104	66.160	53.190	87.400	0.00	0.00
ATOM	1663	HZ	PHE	A	104	66.230	54.260	87.420	0.00	0.00
ATOM	1664	CD2	PHE	A	104	64.900	51.160	87.180	0.00	0.00
ATOM	1665	HD2	PHE	A	104	63.920	50.740	87.010	0.00	0.00
ATOM	1666	CE2	PHE	A	104	64.930	52.590	87.190	0.00	0.00
ATOM	1667	HE2	PHE	A	104	64.030	53.160	87.000	0.00	0.00
ATOM	1668	C	PHE	A	104	66.430	46.960	85.400	0.00	0.00
ATOM	1669	O	PHE	A	104	66.650	46.070	86.250	0.00	0.00
ATOM	1670	N	GLY	A	105	66.140	46.590	84.150	0.00	0.00
ATOM	1671	HN	GLY	A	105	65.850	47.350	83.570	0.00	0.00
ATOM	1672	CA	GLY	A	105	66.170	45.250	83.580	0.00	0.00
ATOM	1673	HA1	GLY	A	105	67.190	44.900	83.650	0.00	0.00
ATOM	1674	HA2	GLY	A	105	65.920	45.360	82.540	0.00	0.00
ATOM	1675	C	GLY	A	105	65.230	44.220	84.180	0.00	0.00
ATOM	1676	O	GLY	A	105	64.310	44.570	84.890	0.00	0.00
ATOM	1677	N	ASN	A	106	65.370	42.890	83.830	0.00	0.00
ATOM	1678	HN	ASN	A	106	66.200	42.480	83.460	0.00	0.00
ATOM	1679	CA	ASN	A	106	64.340	41.890	84.230	0.00	0.00
ATOM	1680	HA	ASN	A	106	63.300	42.130	84.030	0.00	0.00
ATOM	1681	CB	ASN	A	106	64.550	40.480	83.490	0.00	0.00
ATOM	1682	HB1	ASN	A	106	63.990	39.640	83.960	0.00	0.00
ATOM	1683	HB2	ASN	A	106	65.650	40.300	83.450	0.00	0.00
ATOM	1684	CG	ASN	A	106	63.980	40.490	82.050	0.00	0.00
ATOM	1685	OD1	ASN	A	106	62.820	40.750	81.740	0.00	0.00
ATOM	1686	ND2	ASN	A	106	64.870	40.150	81.080	0.00	0.00
ATOM	1687	HD21	ASN	A	106	64.460	40.090	80.170	0.00	0.00
ATOM	1688	HD22	ASN	A	106	65.850	40.100	81.300	0.00	0.00
ATOM	1689	C	ASN	A	106	64.400	41.680	85.740	0.00	0.00
ATOM	1690	O	ASN	A	106	63.370	41.740	86.440	0.00	0.00
ATOM	1691	N	PHE	A	107	65.670	41.560	86.290	0.00	0.00
ATOM	1692	HN	PHE	A	107	66.480	41.430	85.720	0.00	0.00
ATOM	1693	CA	PHE	A	107	66.050	41.500	87.680	0.00	0.00
ATOM	1694	HA	PHE	A	107	65.700	40.540	88.020	0.00	0.00
ATOM	1695	CB	PHE	A	107	67.570	41.640	87.890	0.00	0.00
ATOM	1696	HB1	PHE	A	107	67.770	42.630	87.430	0.00	0.00
ATOM	1697	HB2	PHE	A	107	68.090	40.920	87.220	0.00	0.00
ATOM	1698	CG	PHE	A	107	68.160	41.490	89.300	0.00	0.00
ATOM	1699	CD1	PHE	A	107	69.190	42.240	89.860	0.00	0.00
ATOM	1700	HD1	PHE	A	107	69.600	43.070	89.300	0.00	0.00
ATOM	1701	CE1	PHE	A	107	69.630	42.000	91.140	0.00	0.00
ATOM	1702	HE1	PHE	A	107	70.330	42.650	91.640	0.00	0.00
ATOM	1703	CZ	PHE	A	107	69.090	40.860	91.890	0.00	0.00

ATOM	1704	HZ	PHE	A	107	69.430	40.690	92.900	0.00	0.00
ATOM	1705	CD2	PHE	A	107	67.760	40.330	89.900	0.00	0.00
ATOM	1706	HD2	PHE	A	107	66.950	39.720	89.530	0.00	0.00
ATOM	1707	CE2	PHE	A	107	68.230	40.020	91.180	0.00	0.00
ATOM	1708	HE2	PHE	A	107	67.960	39.090	91.660	0.00	0.00
ATOM	1709	C	PHE	A	107	65.250	42.410	88.670	0.00	0.00
ATOM	1710	O	PHE	A	107	64.370	41.940	89.450	0.00	0.00
ATOM	1711	N	LEU	A	108	65.390	43.790	88.600	0.00	0.00
ATOM	1712	HN	LEU	A	108	66.060	44.170	87.960	0.00	0.00
ATOM	1713	CA	LEU	A	108	64.640	44.670	89.390	0.00	0.00
ATOM	1714	HA	LEU	A	108	64.760	44.290	90.400	0.00	0.00
ATOM	1715	CB	LEU	A	108	65.230	46.130	89.500	0.00	0.00
ATOM	1716	HB1	LEU	A	108	65.050	46.600	88.500	0.00	0.00
ATOM	1717	HB2	LEU	A	108	66.300	46.110	89.770	0.00	0.00
ATOM	1718	CG	LEU	A	108	64.550	46.950	90.620	0.00	0.00
ATOM	1719	HG	LEU	A	108	63.440	46.870	90.580	0.00	0.00
ATOM	1720	CD1	LEU	A	108	64.980	46.400	92.010	0.00	0.00
ATOM	1721	HD11	LEU	A	108	64.740	45.310	92.000	0.00	0.00
ATOM	1722	HD12	LEU	A	108	64.370	46.940	92.760	0.00	0.00
ATOM	1723	HD13	LEU	A	108	66.040	46.460	92.310	0.00	0.00
ATOM	1724	CD2	LEU	A	108	64.950	48.440	90.550	0.00	0.00
ATOM	1725	HD21	LEU	A	108	64.640	48.920	89.600	0.00	0.00
ATOM	1726	HD22	LEU	A	108	66.060	48.420	90.620	0.00	0.00
ATOM	1727	HD23	LEU	A	108	64.470	48.960	91.400	0.00	0.00
ATOM	1728	C	LEU	A	108	63.110	44.510	89.150	0.00	0.00
ATOM	1729	O	LEU	A	108	62.350	44.500	90.130	0.00	0.00
ATOM	1730	N	CYS	A	109	62.610	44.420	87.890	0.00	0.00
ATOM	1731	HN	CYS	A	109	63.220	44.300	87.120	0.00	0.00
ATOM	1732	CA	CYS	A	109	61.250	44.270	87.510	0.00	0.00
ATOM	1733	HA	CYS	A	109	60.680	45.140	87.830	0.00	0.00
ATOM	1734	CB	CYS	A	109	61.110	44.100	85.890	0.00	0.00
ATOM	1735	HB1	CYS	A	109	61.670	43.150	85.780	0.00	0.00
ATOM	1736	HB2	CYS	A	109	61.720	44.860	85.360	0.00	0.00
ATOM	1737	SG	CYS	A	109	59.410	44.020	85.300	0.00	0.00
ATOM	1738	C	CYS	A	109	60.520	43.080	88.180	0.00	0.00
ATOM	1739	O	CYS	A	109	59.480	43.240	88.690	0.00	0.00
ATOM	1740	N	LYS	A	110	61.180	41.900	88.340	0.00	0.00
ATOM	1741	HN	LYS	A	110	62.080	41.790	87.930	0.00	0.00
ATOM	1742	CA	LYS	A	110	60.660	40.770	89.060	0.00	0.00
ATOM	1743	HA	LYS	A	110	59.620	40.640	88.770	0.00	0.00
ATOM	1744	CB	LYS	A	110	61.470	39.540	88.740	0.00	0.00
ATOM	1745	HB1	LYS	A	110	61.450	38.690	89.460	0.00	0.00
ATOM	1746	HB2	LYS	A	110	62.520	39.890	88.830	0.00	0.00
ATOM	1747	CG	LYS	A	110	61.310	39.030	87.290	0.00	0.00
ATOM	1748	HG1	LYS	A	110	61.640	39.740	86.510	0.00	0.00
ATOM	1749	HG2	LYS	A	110	60.210	38.930	87.310	0.00	0.00
ATOM	1750	CD	LYS	A	110	62.150	37.720	87.070	0.00	0.00
ATOM	1751	HD1	LYS	A	110	61.790	37.040	87.870	0.00	0.00
ATOM	1752	HD2	LYS	A	110	63.220	37.960	87.240	0.00	0.00
ATOM	1753	CE	LYS	A	110	61.890	37.070	85.700	0.00	0.00
ATOM	1754	HE1	LYS	A	110	62.440	36.110	85.770	0.00	0.00
ATOM	1755	HE2	LYS	A	110	62.150	37.770	84.880	0.00	0.00
ATOM	1756	NZ	LYS	A	110	60.500	36.650	85.590	0.00	0.00
ATOM	1757	HZ1	LYS	A	110	59.910	37.500	85.690	0.00	0.00
ATOM	1758	HZ2	LYS	A	110	60.310	35.910	86.290	0.00	0.00
ATOM	1759	HZ3	LYS	A	110	60.410	36.330	84.610	0.00	0.00
ATOM	1760	C	LYS	A	110	60.660	41.100	90.600	0.00	0.00
ATOM	1761	O	LYS	A	110	59.680	40.790	91.340	0.00	0.00
ATOM	1762	N	ALA	A	111	61.690	41.880	91.070	0.00	0.00
ATOM	1763	HN	ALA	A	111	62.390	42.230	90.450	0.00	0.00
ATOM	1764	CA	ALA	A	111	61.880	42.210	92.470	0.00	0.00

ATOM	1765	HA	ALA	A	111	61.770	41.280	93.000	0.00	0.00
ATOM	1766	CB	ALA	A	111	63.300	42.660	92.660	0.00	0.00
ATOM	1767	HB1	ALA	A	111	63.500	43.570	92.060	0.00	0.00
ATOM	1768	HB2	ALA	A	111	63.950	41.820	92.330	0.00	0.00
ATOM	1769	HB3	ALA	A	111	63.470	42.790	93.760	0.00	0.00
ATOM	1770	C	ALA	A	111	60.880	43.080	93.050	0.00	0.00
ATOM	1771	O	ALA	A	111	60.240	42.840	94.070	0.00	0.00
ATOM	1772	N	VAL	A	112	60.490	44.110	92.260	0.00	0.00
ATOM	1773	HN	VAL	A	112	61.050	44.210	91.440	0.00	0.00
ATOM	1774	CA	VAL	A	112	59.380	45.130	92.480	0.00	0.00
ATOM	1775	HA	VAL	A	112	59.590	45.430	93.490	0.00	0.00
ATOM	1776	CB	VAL	A	112	59.510	46.390	91.680	0.00	0.00
ATOM	1777	HB	VAL	A	112	58.690	47.100	91.930	0.00	0.00
ATOM	1778	CG1	VAL	A	112	60.860	47.070	92.030	0.00	0.00
ATOM	1779	HG11	VAL	A	112	60.970	48.020	91.470	0.00	0.00
ATOM	1780	HG12	VAL	A	112	61.730	46.470	91.720	0.00	0.00
ATOM	1781	HG13	VAL	A	112	61.010	47.110	93.130	0.00	0.00
ATOM	1782	CG2	VAL	A	112	59.360	45.990	90.210	0.00	0.00
ATOM	1783	HG21	VAL	A	112	60.300	45.460	89.940	0.00	0.00
ATOM	1784	HG22	VAL	A	112	59.300	46.940	89.630	0.00	0.00
ATOM	1785	HG23	VAL	A	112	58.520	45.350	89.870	0.00	0.00
ATOM	1786	C	VAL	A	112	57.940	44.540	92.390	0.00	0.00
ATOM	1787	O	VAL	A	112	57.170	44.990	93.180	0.00	0.00
ATOM	1788	N	HSD	A	113	57.590	43.590	91.510	0.00	0.00
ATOM	1789	HN	HSD	A	113	58.160	43.370	90.720	0.00	0.00
ATOM	1790	CA	HSD	A	113	56.340	42.860	91.600	0.00	0.00
ATOM	1791	HA	HSD	A	113	55.540	43.570	91.690	0.00	0.00
ATOM	1792	CB	HSD	A	113	56.080	41.870	90.360	0.00	0.00
ATOM	1793	HB1	HSD	A	113	55.170	41.310	90.650	0.00	0.00
ATOM	1794	HB2	HSD	A	113	56.850	41.120	90.090	0.00	0.00
ATOM	1795	ND1	HSD	A	113	56.730	43.220	88.290	0.00	0.00
ATOM	1796	HD1	HSD	A	113	57.730	43.170	88.370	0.00	0.00
ATOM	1797	CG	HSD	A	113	55.800	42.640	89.170	0.00	0.00
ATOM	1798	CE1	HSD	A	113	55.990	43.680	87.270	0.00	0.00
ATOM	1799	HE1	HSD	A	113	56.490	44.190	86.440	0.00	0.00
ATOM	1800	NE2	HSD	A	113	54.730	43.470	87.340	0.00	0.00
ATOM	1801	CD2	HSD	A	113	54.610	42.760	88.540	0.00	0.00
ATOM	1802	HD2	HSD	A	113	53.680	42.320	88.880	0.00	0.00
ATOM	1803	C	HSD	A	113	56.360	42.000	92.860	0.00	0.00
ATOM	1804	O	HSD	A	113	55.350	41.890	93.560	0.00	0.00
ATOM	1805	N	VAL	A	114	57.480	41.350	93.200	0.00	0.00
ATOM	1806	HN	VAL	A	114	58.310	41.150	92.680	0.00	0.00
ATOM	1807	CA	VAL	A	114	57.450	40.620	94.530	0.00	0.00
ATOM	1808	HA	VAL	A	114	56.630	39.920	94.560	0.00	0.00
ATOM	1809	CB	VAL	A	114	58.700	39.650	94.680	0.00	0.00
ATOM	1810	HB	VAL	A	114	59.560	40.280	94.390	0.00	0.00
ATOM	1811	CG1	VAL	A	114	58.930	39.270	96.140	0.00	0.00
ATOM	1812	HG11	VAL	A	114	59.590	40.020	96.610	0.00	0.00
ATOM	1813	HG12	VAL	A	114	59.390	38.270	96.260	0.00	0.00
ATOM	1814	HG13	VAL	A	114	58.000	39.160	96.740	0.00	0.00
ATOM	1815	CG2	VAL	A	114	58.640	38.410	93.760	0.00	0.00
ATOM	1816	HG21	VAL	A	114	57.920	37.700	94.220	0.00	0.00
ATOM	1817	HG22	VAL	A	114	59.670	38.000	93.730	0.00	0.00
ATOM	1818	HG23	VAL	A	114	58.270	38.610	92.730	0.00	0.00
ATOM	1819	C	VAL	A	114	57.220	41.560	95.720	0.00	0.00
ATOM	1820	O	VAL	A	114	56.450	41.230	96.610	0.00	0.00
ATOM	1821	N	ILE	A	115	57.860	42.720	95.730	0.00	0.00
ATOM	1822	HN	ILE	A	115	58.360	42.900	94.890	0.00	0.00
ATOM	1823	CA	ILE	A	115	57.680	43.680	96.820	0.00	0.00
ATOM	1824	HA	ILE	A	115	57.960	43.130	97.710	0.00	0.00
ATOM	1825	CB	ILE	A	115	58.710	44.890	96.740	0.00	0.00

ATOM	1826	HB	ILE	A	115	58.430	45.400	95.790	0.00	0.00
ATOM	1827	CG2	ILE	A	115	58.390	46.050	97.800	0.00	0.00
ATOM	1828	HG21	ILE	A	115	58.080	45.470	98.700	0.00	0.00
ATOM	1829	HG22	ILE	A	115	57.520	46.680	97.500	0.00	0.00
ATOM	1830	HG23	ILE	A	115	59.320	46.650	97.920	0.00	0.00
ATOM	1831	CG1	ILE	A	115	60.150	44.440	96.810	0.00	0.00
ATOM	1832	HG11	ILE	A	115	60.280	43.430	96.360	0.00	0.00
ATOM	1833	HG12	ILE	A	115	60.400	44.210	97.870	0.00	0.00
ATOM	1834	CD	ILE	A	115	61.100	45.560	96.260	0.00	0.00
ATOM	1835	HD1	ILE	A	115	61.180	46.400	96.970	0.00	0.00
ATOM	1836	HD2	ILE	A	115	60.690	45.890	95.280	0.00	0.00
ATOM	1837	HD3	ILE	A	115	62.100	45.180	95.950	0.00	0.00
ATOM	1838	C	ILE	A	115	56.290	44.140	97.100	0.00	0.00
ATOM	1839	O	ILE	A	115	55.820	44.160	98.240	0.00	0.00
ATOM	1840	N	TYR	A	116	55.670	44.610	96.010	0.00	0.00
ATOM	1841	HN	TYR	A	116	56.050	44.450	95.100	0.00	0.00
ATOM	1842	CA	TYR	A	116	54.230	45.110	95.820	0.00	0.00
ATOM	1843	HA	TYR	A	116	54.060	45.750	96.670	0.00	0.00
ATOM	1844	CB	TYR	A	116	54.050	45.940	94.530	0.00	0.00
ATOM	1845	HB1	TYR	A	116	54.610	45.460	93.700	0.00	0.00
ATOM	1846	HB2	TYR	A	116	54.570	46.920	94.660	0.00	0.00
ATOM	1847	CG	TYR	A	116	52.600	46.170	94.040	0.00	0.00
ATOM	1848	CD1	TYR	A	116	52.130	45.910	92.710	0.00	0.00
ATOM	1849	HD1	TYR	A	116	52.920	45.550	92.060	0.00	0.00
ATOM	1850	CE1	TYR	A	116	50.810	46.130	92.310	0.00	0.00
ATOM	1851	HE1	TYR	A	116	50.470	45.960	91.300	0.00	0.00
ATOM	1852	CZ	TYR	A	116	49.900	46.630	93.230	0.00	0.00
ATOM	1853	OH	TYR	A	116	48.610	46.780	92.880	0.00	0.00
ATOM	1854	HH	TYR	A	116	48.160	47.160	93.640	0.00	0.00
ATOM	1855	CD2	TYR	A	116	51.640	46.680	94.910	0.00	0.00
ATOM	1856	HD2	TYR	A	116	51.860	46.890	95.940	0.00	0.00
ATOM	1857	CE2	TYR	A	116	50.280	46.880	94.570	0.00	0.00
ATOM	1858	HE2	TYR	A	116	49.490	47.140	95.250	0.00	0.00
ATOM	1859	C	TYR	A	116	53.280	43.950	96.080	0.00	0.00
ATOM	1860	O	TYR	A	116	52.330	44.170	96.750	0.00	0.00
ATOM	1861	N	THR	A	117	53.520	42.790	95.520	0.00	0.00
ATOM	1862	HN	THR	A	117	54.190	42.640	94.800	0.00	0.00
ATOM	1863	CA	THR	A	117	52.640	41.640	95.740	0.00	0.00
ATOM	1864	HA	THR	A	117	51.660	42.060	95.550	0.00	0.00
ATOM	1865	CB	THR	A	117	52.960	40.570	94.800	0.00	0.00
ATOM	1866	HB	THR	A	117	54.030	40.310	94.680	0.00	0.00
ATOM	1867	OG1	THR	A	117	52.280	40.960	93.600	0.00	0.00
ATOM	1868	HG1	THR	A	117	51.960	40.160	93.200	0.00	0.00
ATOM	1869	CG2	THR	A	117	52.360	39.300	95.150	0.00	0.00
ATOM	1870	HG21	THR	A	117	52.860	38.680	95.920	0.00	0.00
ATOM	1871	HG22	THR	A	117	52.520	38.560	94.340	0.00	0.00
ATOM	1872	HG23	THR	A	117	51.280	39.420	95.360	0.00	0.00
ATOM	1873	C	THR	A	117	52.640	41.220	97.170	0.00	0.00
ATOM	1874	O	THR	A	117	51.550	41.050	97.790	0.00	0.00
ATOM	1875	N	VAL	A	118	53.850	41.060	97.860	0.00	0.00
ATOM	1876	HN	VAL	A	118	54.730	41.210	97.420	0.00	0.00
ATOM	1877	CA	VAL	A	118	53.820	40.800	99.320	0.00	0.00
ATOM	1878	HA	VAL	A	118	53.180	39.940	99.450	0.00	0.00
ATOM	1879	CB	VAL	A	118	55.230	40.310	99.890	0.00	0.00
ATOM	1880	HB	VAL	A	118	55.940	41.130	99.660	0.00	0.00
ATOM	1881	CG1	VAL	A	118	55.170	40.070	101.450	0.00	0.00
ATOM	1882	HG11	VAL	A	118	56.000	39.390	101.740	0.00	0.00
ATOM	1883	HG12	VAL	A	118	54.240	39.570	101.780	0.00	0.00
ATOM	1884	HG13	VAL	A	118	55.320	41.020	102.000	0.00	0.00
ATOM	1885	CG2	VAL	A	118	55.650	39.070	99.090	0.00	0.00
ATOM	1886	HG21	VAL	A	118	54.850	38.290	99.120	0.00	0.00

ATOM 1887 HG22 VAL A 118 56.540 38.600 99.580 0.00 0.00
ATOM 1888 HG23 VAL A 118 55.810 39.340 98.020 0.00 0.00
ATOM 1889 C VAL A 118 53.380 41.890 100.140 0.00 0.00
ATOM 1890 O VAL A 118 52.610 41.650 101.050 0.00 0.00
ATOM 1891 N ASN A 119 53.740 43.140 99.880 0.00 0.00
ATOM 1892 HN ASN A 119 54.350 43.450 99.160 0.00 0.00
ATOM 1893 CA ASN A 119 53.060 44.330 100.490 0.00 0.00
ATOM 1894 HA ASN A 119 53.190 44.210 101.560 0.00 0.00
ATOM 1895 CB ASN A 119 53.910 45.550 100.020 0.00 0.00
ATOM 1896 HB1 ASN A 119 53.790 45.680 98.920 0.00 0.00
ATOM 1897 HB2 ASN A 119 54.950 45.400 100.370 0.00 0.00
ATOM 1898 CG ASN A 119 53.400 46.860 100.510 0.00 0.00
ATOM 1899 OD1 ASN A 119 52.810 47.670 99.770 0.00 0.00
ATOM 1900 ND2 ASN A 119 53.570 47.080 101.850 0.00 0.00
ATOM 1901 HD21 ASN A 119 53.080 47.830 102.290 0.00 0.00
ATOM 1902 HD22 ASN A 119 54.100 46.480 102.450 0.00 0.00
ATOM 1903 C ASN A 119 51.500 44.590 100.330 0.00 0.00
ATOM 1904 O ASN A 119 50.900 45.100 101.310 0.00 0.00
ATOM 1905 N LEU A 120 50.940 44.440 99.080 0.00 0.00
ATOM 1906 HN LEU A 120 51.510 44.370 98.270 0.00 0.00
ATOM 1907 CA LEU A 120 49.520 44.460 98.850 0.00 0.00
ATOM 1908 HA LEU A 120 49.180 45.490 98.890 0.00 0.00
ATOM 1909 CB LEU A 120 49.260 44.010 97.360 0.00 0.00
ATOM 1910 HB1 LEU A 120 49.770 43.030 97.250 0.00 0.00
ATOM 1911 HB2 LEU A 120 49.730 44.740 96.670 0.00 0.00
ATOM 1912 CG LEU A 120 47.790 43.810 96.960 0.00 0.00
ATOM 1913 HG LEU A 120 47.450 42.840 97.370 0.00 0.00
ATOM 1914 CD1 LEU A 120 46.930 45.110 97.270 0.00 0.00
ATOM 1915 HD11 LEU A 120 47.400 46.030 96.860 0.00 0.00
ATOM 1916 HD12 LEU A 120 46.920 45.210 98.380 0.00 0.00
ATOM 1917 HD13 LEU A 120 45.930 45.070 96.790 0.00 0.00
ATOM 1918 CD2 LEU A 120 47.720 43.680 95.500 0.00 0.00
ATOM 1919 HD21 LEU A 120 48.010 44.600 94.950 0.00 0.00
ATOM 1920 HD22 LEU A 120 46.680 43.400 95.220 0.00 0.00
ATOM 1921 HD23 LEU A 120 48.270 42.730 95.330 0.00 0.00
ATOM 1922 C LEU A 120 48.750 43.520 99.820 0.00 0.00
ATOM 1923 O LEU A 120 47.950 43.910 100.640 0.00 0.00
ATOM 1924 N TYR A 121 49.130 42.220 99.760 0.00 0.00
ATOM 1925 HN TYR A 121 49.720 41.870 99.040 0.00 0.00
ATOM 1926 CA TYR A 121 48.350 41.200 100.510 0.00 0.00
ATOM 1927 HA TYR A 121 47.320 41.450 100.310 0.00 0.00
ATOM 1928 CB TYR A 121 48.650 39.790 99.810 0.00 0.00
ATOM 1929 HB1 TYR A 121 48.020 38.990 100.270 0.00 0.00
ATOM 1930 HB2 TYR A 121 49.720 39.560 100.000 0.00 0.00
ATOM 1931 CG TYR A 121 48.300 39.730 98.360 0.00 0.00
ATOM 1932 CD1 TYR A 121 47.030 40.110 98.030 0.00 0.00
ATOM 1933 HD1 TYR A 121 46.410 40.560 98.790 0.00 0.00
ATOM 1934 CE1 TYR A 121 46.580 40.150 96.660 0.00 0.00
ATOM 1935 HE1 TYR A 121 45.680 40.590 96.270 0.00 0.00
ATOM 1936 CZ TYR A 121 47.390 39.650 95.730 0.00 0.00
ATOM 1937 OH TYR A 121 46.880 39.660 94.420 0.00 0.00
ATOM 1938 HH TYR A 121 47.660 39.670 93.860 0.00 0.00
ATOM 1939 CD2 TYR A 121 49.170 39.280 97.380 0.00 0.00
ATOM 1940 HD2 TYR A 121 50.200 39.000 97.550 0.00 0.00
ATOM 1941 CE2 TYR A 121 48.680 39.100 96.060 0.00 0.00
ATOM 1942 HE2 TYR A 121 49.310 38.660 95.290 0.00 0.00
ATOM 1943 C TYR A 121 48.670 41.240 101.940 0.00 0.00
ATOM 1944 O TYR A 121 47.760 41.040 102.720 0.00 0.00
ATOM 1945 N SER A 122 49.880 41.710 102.340 0.00 0.00
ATOM 1946 HN SER A 122 50.590 41.940 101.680 0.00 0.00
ATOM 1947 CA SER A 122 50.190 41.990 103.700 0.00 0.00

ATOM	1948	HA	SER	A	122	49.920	41.140	104.300	0.00	0.00
ATOM	1949	CB	SER	A	122	51.710	42.310	104.050	0.00	0.00
ATOM	1950	HB1	SER	A	122	51.760	42.330	105.160	0.00	0.00
ATOM	1951	HB2	SER	A	122	52.010	43.280	103.610	0.00	0.00
ATOM	1952	OG	SER	A	122	52.500	41.210	103.660	0.00	0.00
ATOM	1953	HG1	SER	A	122	52.710	41.280	102.720	0.00	0.00
ATOM	1954	C	SER	A	122	49.330	43.090	104.340	0.00	0.00
ATOM	1955	O	SER	A	122	48.850	42.880	105.400	0.00	0.00
ATOM	1956	N	SER	A	123	49.140	44.250	103.730	0.00	0.00
ATOM	1957	HN	SER	A	123	49.370	44.300	102.760	0.00	0.00
ATOM	1958	CA	SER	A	123	48.410	45.360	104.320	0.00	0.00
ATOM	1959	HA	SER	A	123	48.860	45.530	105.290	0.00	0.00
ATOM	1960	CB	SER	A	123	48.390	46.590	103.420	0.00	0.00
ATOM	1961	HB1	SER	A	123	47.740	46.420	102.540	0.00	0.00
ATOM	1962	HB2	SER	A	123	49.480	46.650	103.220	0.00	0.00
ATOM	1963	OG	SER	A	123	48.070	47.760	104.090	0.00	0.00
ATOM	1964	HG1	SER	A	123	48.850	48.210	104.440	0.00	0.00
ATOM	1965	C	SER	A	123	46.990	45.080	104.580	0.00	0.00
ATOM	1966	O	SER	A	123	46.350	45.520	105.590	0.00	0.00
ATOM	1967	N	VAL	A	124	46.340	44.490	103.580	0.00	0.00
ATOM	1968	HN	VAL	A	124	46.680	44.230	102.680	0.00	0.00
ATOM	1969	CA	VAL	A	124	44.890	44.310	103.720	0.00	0.00
ATOM	1970	HA	VAL	A	124	44.500	45.230	104.130	0.00	0.00
ATOM	1971	CB	VAL	A	124	44.080	44.140	102.410	0.00	0.00
ATOM	1972	HB	VAL	A	124	43.020	43.890	102.630	0.00	0.00
ATOM	1973	CG1	VAL	A	124	43.870	45.580	101.770	0.00	0.00
ATOM	1974	HG11	VAL	A	124	43.620	46.330	102.550	0.00	0.00
ATOM	1975	HG12	VAL	A	124	42.980	45.510	101.110	0.00	0.00
ATOM	1976	HG13	VAL	A	124	44.700	45.950	101.140	0.00	0.00
ATOM	1977	CG2	VAL	A	124	44.650	43.160	101.520	0.00	0.00
ATOM	1978	HG21	VAL	A	124	44.820	42.150	101.960	0.00	0.00
ATOM	1979	HG22	VAL	A	124	45.570	43.510	101.000	0.00	0.00
ATOM	1980	HG23	VAL	A	124	43.870	42.870	100.780	0.00	0.00
ATOM	1981	C	VAL	A	124	44.510	43.230	104.720	0.00	0.00
ATOM	1982	O	VAL	A	124	43.730	43.400	105.640	0.00	0.00
ATOM	1983	N	LEU	A	125	45.240	42.050	104.630	0.00	0.00
ATOM	1984	HN	LEU	A	125	45.930	42.060	103.910	0.00	0.00
ATOM	1985	CA	LEU	A	125	44.930	40.860	105.410	0.00	0.00
ATOM	1986	HA	LEU	A	125	43.860	40.740	105.450	0.00	0.00
ATOM	1987	CB	LEU	A	125	45.590	39.600	104.770	0.00	0.00
ATOM	1988	HB1	LEU	A	125	45.370	38.760	105.470	0.00	0.00
ATOM	1989	HB2	LEU	A	125	46.700	39.720	104.800	0.00	0.00
ATOM	1990	CG	LEU	A	125	45.230	39.160	103.360	0.00	0.00
ATOM	1991	HG	LEU	A	125	45.470	39.980	102.650	0.00	0.00
ATOM	1992	CD1	LEU	A	125	45.950	37.910	102.920	0.00	0.00
ATOM	1993	HD11	LEU	A	125	45.670	37.570	101.900	0.00	0.00
ATOM	1994	HD12	LEU	A	125	45.660	37.140	103.660	0.00	0.00
ATOM	1995	HD13	LEU	A	125	47.060	38.010	102.970	0.00	0.00
ATOM	1996	CD2	LEU	A	125	43.700	39.070	103.230	0.00	0.00
ATOM	1997	HD21	LEU	A	125	43.260	40.090	103.330	0.00	0.00
ATOM	1998	HD22	LEU	A	125	43.310	38.470	104.080	0.00	0.00
ATOM	1999	HD23	LEU	A	125	43.430	38.550	102.290	0.00	0.00
ATOM	2000	C	LEU	A	125	45.320	41.080	106.830	0.00	0.00
ATOM	2001	O	LEU	A	125	44.560	40.620	107.760	0.00	0.00
ATOM	2002	N	ILE	A	126	46.470	41.780	107.160	0.00	0.00
ATOM	2003	HN	ILE	A	126	47.080	42.040	106.420	0.00	0.00
ATOM	2004	CA	ILE	A	126	46.910	42.140	108.500	0.00	0.00
ATOM	2005	HA	ILE	A	126	46.760	41.230	109.060	0.00	0.00
ATOM	2006	CB	ILE	A	126	48.320	42.620	108.650	0.00	0.00
ATOM	2007	HB	ILE	A	126	49.020	42.020	108.030	0.00	0.00
ATOM	2008	CG2	ILE	A	126	48.490	44.130	108.110	0.00	0.00

ATOM 2009 HG21 ILE A 126 48.080 44.940 108.750 0.00 0.00
ATOM 2010 HG22 ILE A 126 48.100 44.200 107.070 0.00 0.00
ATOM 2011 HG23 ILE A 126 49.570 44.360 107.990 0.00 0.00
ATOM 2012 CG1 ILE A 126 48.940 42.410 110.050 0.00 0.00
ATOM 2013 HG11 ILE A 126 48.280 43.030 110.690 0.00 0.00
ATOM 2014 HG12 ILE A 126 49.970 42.810 110.010 0.00 0.00
ATOM 2015 CD ILE A 126 48.960 40.900 110.390 0.00 0.00
ATOM 2016 HD1 ILE A 126 49.460 40.410 109.530 0.00 0.00
ATOM 2017 HD2 ILE A 126 47.940 40.480 110.480 0.00 0.00
ATOM 2018 HD3 ILE A 126 49.580 40.770 111.300 0.00 0.00
ATOM 2019 C ILE A 126 45.890 43.100 109.200 0.00 0.00
ATOM 2020 O ILE A 126 45.700 43.000 110.360 0.00 0.00
ATOM 2021 N LEU A 127 45.230 44.030 108.490 0.00 0.00
ATOM 2022 HN LEU A 127 45.390 44.040 107.510 0.00 0.00
ATOM 2023 CA LEU A 127 44.250 45.020 108.940 0.00 0.00
ATOM 2024 HA LEU A 127 44.720 45.590 109.730 0.00 0.00
ATOM 2025 CB LEU A 127 43.770 46.010 107.860 0.00 0.00
ATOM 2026 HB1 LEU A 127 43.270 45.440 107.050 0.00 0.00
ATOM 2027 HB2 LEU A 127 44.690 46.550 107.550 0.00 0.00
ATOM 2028 CG LEU A 127 42.610 47.100 108.170 0.00 0.00
ATOM 2029 HG LEU A 127 41.710 46.510 108.470 0.00 0.00
ATOM 2030 CD1 LEU A 127 43.020 48.070 109.260 0.00 0.00
ATOM 2031 HD11 LEU A 127 42.290 48.870 109.510 0.00 0.00
ATOM 2032 HD12 LEU A 127 43.960 48.520 108.870 0.00 0.00
ATOM 2033 HD13 LEU A 127 43.300 47.510 110.180 0.00 0.00
ATOM 2034 CD2 LEU A 127 42.360 47.860 106.900 0.00 0.00
ATOM 2035 HD21 LEU A 127 43.210 48.540 106.680 0.00 0.00
ATOM 2036 HD22 LEU A 127 41.470 48.520 107.010 0.00 0.00
ATOM 2037 HD23 LEU A 127 42.150 47.150 106.070 0.00 0.00
ATOM 2038 C LEU A 127 43.120 44.280 109.580 0.00 0.00
ATOM 2039 O LEU A 127 42.610 44.700 110.670 0.00 0.00
ATOM 2040 N ALA A 128 42.650 43.180 109.000 0.00 0.00
ATOM 2041 HN ALA A 128 43.060 42.920 108.140 0.00 0.00
ATOM 2042 CA ALA A 128 41.590 42.360 109.590 0.00 0.00
ATOM 2043 HA ALA A 128 40.780 42.970 109.960 0.00 0.00
ATOM 2044 CB ALA A 128 41.090 41.400 108.520 0.00 0.00
ATOM 2045 HB1 ALA A 128 40.240 40.760 108.830 0.00 0.00
ATOM 2046 HB2 ALA A 128 41.990 40.780 108.340 0.00 0.00
ATOM 2047 HB3 ALA A 128 40.910 41.930 107.560 0.00 0.00
ATOM 2048 C ALA A 128 42.090 41.670 110.870 0.00 0.00
ATOM 2049 O ALA A 128 41.360 41.430 111.850 0.00 0.00
ATOM 2050 N PHE A 129 43.370 41.200 110.930 0.00 0.00
ATOM 2051 HN PHE A 129 43.850 41.110 110.070 0.00 0.00
ATOM 2052 CA PHE A 129 43.920 40.650 112.130 0.00 0.00
ATOM 2053 HA PHE A 129 43.260 39.920 112.570 0.00 0.00
ATOM 2054 CB PHE A 129 45.210 39.860 112.010 0.00 0.00
ATOM 2055 HB1 PHE A 129 45.650 39.570 112.980 0.00 0.00
ATOM 2056 HB2 PHE A 129 46.010 40.370 111.430 0.00 0.00
ATOM 2057 CG PHE A 129 44.830 38.540 111.260 0.00 0.00
ATOM 2058 CD1 PHE A 129 45.010 38.330 109.930 0.00 0.00
ATOM 2059 HD1 PHE A 129 45.400 39.080 109.250 0.00 0.00
ATOM 2060 CE1 PHE A 129 44.770 37.100 109.310 0.00 0.00
ATOM 2061 HE1 PHE A 129 45.060 37.020 108.270 0.00 0.00
ATOM 2062 CZ PHE A 129 44.330 36.000 110.010 0.00 0.00
ATOM 2063 HZ PHE A 129 44.130 35.080 109.480 0.00 0.00
ATOM 2064 CD2 PHE A 129 44.160 37.470 111.900 0.00 0.00
ATOM 2065 HD2 PHE A 129 43.870 37.570 112.940 0.00 0.00
ATOM 2066 CE2 PHE A 129 43.990 36.180 111.340 0.00 0.00
ATOM 2067 HE2 PHE A 129 43.480 35.430 111.920 0.00 0.00
ATOM 2068 C PHE A 129 44.010 41.590 113.300 0.00 0.00
ATOM 2069 O PHE A 129 43.600 41.270 114.460 0.00 0.00

```
ATOM   2070  N   ILE A 130      44.480  42.850 112.960  0.00  0.00
ATOM   2071  HN  ILE A 130      44.740  43.060 112.030  0.00  0.00
ATOM   2072  CA  ILE A 130      44.370  43.990 113.870  0.00  0.00
ATOM   2073  HA  ILE A 130      44.940  43.830 114.770  0.00  0.00
ATOM   2074  CB  ILE A 130      44.890  45.260 113.210  0.00  0.00
ATOM   2075  HB  ILE A 130      44.500  45.330 112.170  0.00  0.00
ATOM   2076  CG2 ILE A 130      44.480  46.520 113.940  0.00  0.00
ATOM   2077 HG21 ILE A 130      45.080  47.390 113.580  0.00  0.00
ATOM   2078 HG22 ILE A 130      44.560  46.410 115.050  0.00  0.00
ATOM   2079 HG23 ILE A 130      43.420  46.720 113.690  0.00  0.00
ATOM   2080  CG1 ILE A 130      46.450  45.190 113.060  0.00  0.00
ATOM   2081 HG11 ILE A 130      46.660  44.160 112.710  0.00  0.00
ATOM   2082 HG12 ILE A 130      46.790  45.290 114.120  0.00  0.00
ATOM   2083  CD  ILE A 130      47.070  46.290 112.150  0.00  0.00
ATOM   2084  HD1 ILE A 130      48.180  46.320 112.100  0.00  0.00
ATOM   2085  HD2 ILE A 130      46.760  47.250 112.620  0.00  0.00
ATOM   2086  HD3 ILE A 130      46.590  46.290 111.150  0.00  0.00
ATOM   2087  C   ILE A 130      42.950  44.210 114.330  0.00  0.00
ATOM   2088  O   ILE A 130      42.640  44.200 115.500  0.00  0.00
ATOM   2089  N   SER A 131      42.020  44.310 113.390  0.00  0.00
ATOM   2090  HN  SER A 131      42.400  44.330 112.460  0.00  0.00
ATOM   2091  CA  SER A 131      40.660  44.470 113.710  0.00  0.00
ATOM   2092  HA  SER A 131      40.490  45.370 114.280  0.00  0.00
ATOM   2093  CB  SER A 131      39.690  44.560 112.490  0.00  0.00
ATOM   2094  HB1 SER A 131      38.670  44.600 112.930  0.00  0.00
ATOM   2095  HB2 SER A 131      39.770  43.640 111.880  0.00  0.00
ATOM   2096  OG  SER A 131      39.860  45.690 111.610  0.00  0.00
ATOM   2097  HG1 SER A 131      40.690  45.600 111.150  0.00  0.00
ATOM   2098  C   SER A 131      40.040  43.390 114.580  0.00  0.00
ATOM   2099  O   SER A 131      39.280  43.680 115.470  0.00  0.00
ATOM   2100  N   LEU A 132      40.390  42.110 114.330  0.00  0.00
ATOM   2101  HN  LEU A 132      40.940  41.830 113.550  0.00  0.00
ATOM   2102  CA  LEU A 132      40.030  41.000 115.160  0.00  0.00
ATOM   2103  HA  LEU A 132      38.960  41.120 115.150  0.00  0.00
ATOM   2104  CB  LEU A 132      40.500  39.640 114.390  0.00  0.00
ATOM   2105  HB1 LEU A 132      40.460  38.820 115.140  0.00  0.00
ATOM   2106  HB2 LEU A 132      41.570  39.660 114.090  0.00  0.00
ATOM   2107  CG  LEU A 132      39.660  39.200 113.210  0.00  0.00
ATOM   2108  HG  LEU A 132      39.450  40.060 112.530  0.00  0.00
ATOM   2109  CD1 LEU A 132      40.400  38.140 112.320  0.00  0.00
ATOM   2110 HD11 LEU A 132      41.050  37.480 112.940  0.00  0.00
ATOM   2111 HD12 LEU A 132      41.030  38.670 111.580  0.00  0.00
ATOM   2112 HD13 LEU A 132      39.580  37.510 111.900  0.00  0.00
ATOM   2113  CD2 LEU A 132      38.300  38.670 113.660  0.00  0.00
ATOM   2114 HD21 LEU A 132      37.840  39.230 114.510  0.00  0.00
ATOM   2115 HD22 LEU A 132      38.450  37.710 114.190  0.00  0.00
ATOM   2116 HD23 LEU A 132      37.690  38.490 112.750  0.00  0.00
ATOM   2117  C   LEU A 132      40.590  41.060 116.570  0.00  0.00
ATOM   2118  O   LEU A 132      39.860  40.770 117.570  0.00  0.00
ATOM   2119  N   ASP A 133      41.850  41.480 116.730  0.00  0.00
ATOM   2120  HN  ASP A 133      42.390  41.700 115.920  0.00  0.00
ATOM   2121  CA  ASP A 133      42.520  41.800 117.960  0.00  0.00
ATOM   2122  HA  ASP A 133      42.420  40.960 118.630  0.00  0.00
ATOM   2123  CB  ASP A 133      44.000  41.850 117.820  0.00  0.00
ATOM   2124  HB1 ASP A 133      44.270  42.630 117.070  0.00  0.00
ATOM   2125  HB2 ASP A 133      44.380  40.890 117.410  0.00  0.00
ATOM   2126  CG  ASP A 133      44.690  42.180 119.090  0.00  0.00
ATOM   2127  OD1 ASP A 133      44.520  41.500 120.140  0.00  0.00
ATOM   2128  OD2 ASP A 133      45.520  43.150 119.120  0.00  0.00
ATOM   2129  C   ASP A 133      41.810  42.980 118.780  0.00  0.00
ATOM   2130  O   ASP A 133      41.470  42.870 119.940  0.00  0.00
```

```
ATOM   2131  N   ARG A 134      41.440  44.020 118.140  0.00  0.00
ATOM   2132  HN  ARG A 134      41.780  44.050 117.200  0.00  0.00
ATOM   2133  CA  ARG A 134      40.680  45.100 118.570  0.00  0.00
ATOM   2134  HA  ARG A 134      41.150  45.350 119.500  0.00  0.00
ATOM   2135  CB  ARG A 134      40.680  46.240 117.510  0.00  0.00
ATOM   2136  HB1 ARG A 134      39.890  46.880 117.940  0.00  0.00
ATOM   2137  HB2 ARG A 134      40.240  46.050 116.510  0.00  0.00
ATOM   2138  CG  ARG A 134      42.040  47.000 117.270  0.00  0.00
ATOM   2139  HG1 ARG A 134      41.770  47.950 116.760  0.00  0.00
ATOM   2140  HG2 ARG A 134      42.690  46.320 116.670  0.00  0.00
ATOM   2141  CD  ARG A 134      42.820  47.360 118.590  0.00  0.00
ATOM   2142  HD1 ARG A 134      42.070  47.690 119.340  0.00  0.00
ATOM   2143  HD2 ARG A 134      43.490  48.220 118.380  0.00  0.00
ATOM   2144  NE  ARG A 134      43.680  46.270 119.140  0.00  0.00
ATOM   2145  HE  ARG A 134      44.160  45.640 118.530  0.00  0.00
ATOM   2146  CZ  ARG A 134      43.950  46.040 120.480  0.00  0.00
ATOM   2147  NH1 ARG A 134      43.670  46.910 121.460  0.00  0.00
ATOM   2148 HH11 ARG A 134      43.300  47.770 121.100  0.00  0.00
ATOM   2149 HH12 ARG A 134      44.000  46.870 122.410  0.00  0.00
ATOM   2150  NH2 ARG A 134      44.710  45.060 120.860  0.00  0.00
ATOM   2151 HH21 ARG A 134      44.860  44.300 120.230  0.00  0.00
ATOM   2152 HH22 ARG A 134      44.670  44.830 121.830  0.00  0.00
ATOM   2153  C   ARG A 134      39.280  44.780 118.950  0.00  0.00
ATOM   2154  O   ARG A 134      38.770  45.130 120.060  0.00  0.00
ATOM   2155  N   TYR A 135      38.660  43.890 118.250  0.00  0.00
ATOM   2156  HN  TYR A 135      39.060  43.600 117.380  0.00  0.00
ATOM   2157  CA  TYR A 135      37.420  43.270 118.750  0.00  0.00
ATOM   2158  HA  TYR A 135      36.720  44.050 119.000  0.00  0.00
ATOM   2159  CB  TYR A 135      36.890  42.370 117.640  0.00  0.00
ATOM   2160  HB1 TYR A 135      37.660  41.870 117.010  0.00  0.00
ATOM   2161  HB2 TYR A 135      36.300  42.980 116.920  0.00  0.00
ATOM   2162  CG  TYR A 135      35.870  41.310 118.020  0.00  0.00
ATOM   2163  CD1 TYR A 135      34.610  41.730 118.530  0.00  0.00
ATOM   2164  HD1 TYR A 135      34.250  42.750 118.540  0.00  0.00
ATOM   2165  CE1 TYR A 135      33.650  40.680 118.720  0.00  0.00
ATOM   2166  HE1 TYR A 135      32.700  40.930 119.180  0.00  0.00
ATOM   2167  CZ  TYR A 135      33.950  39.320 118.620  0.00  0.00
ATOM   2168  OH  TYR A 135      32.880  38.400 118.920  0.00  0.00
ATOM   2169  HH  TYR A 135      32.020  38.730 118.660  0.00  0.00
ATOM   2170  CD2 TYR A 135      36.150  39.900 117.850  0.00  0.00
ATOM   2171  HD2 TYR A 135      37.080  39.580 117.420  0.00  0.00
ATOM   2172  CE2 TYR A 135      35.200  38.930 118.140  0.00  0.00
ATOM   2173  HE2 TYR A 135      35.490  37.890 118.010  0.00  0.00
ATOM   2174  C   TYR A 135      37.550  42.540 120.050  0.00  0.00
ATOM   2175  O   TYR A 135      36.860  42.800 120.990  0.00  0.00
ATOM   2176  N   LEU A 136      38.570  41.670 120.160  0.00  0.00
ATOM   2177  HN  LEU A 136      39.070  41.360 119.360  0.00  0.00
ATOM   2178  CA  LEU A 136      38.820  40.880 121.350  0.00  0.00
ATOM   2179  HA  LEU A 136      37.850  40.510 121.630  0.00  0.00
ATOM   2180  CB  LEU A 136      39.890  39.800 121.030  0.00  0.00
ATOM   2181  HB1 LEU A 136      40.280  39.430 122.000  0.00  0.00
ATOM   2182  HB2 LEU A 136      40.770  40.190 120.490  0.00  0.00
ATOM   2183  CG  LEU A 136      39.220  38.580 120.350  0.00  0.00
ATOM   2184  HG  LEU A 136      38.750  38.950 119.410  0.00  0.00
ATOM   2185  CD1 LEU A 136      40.350  37.570 119.940  0.00  0.00
ATOM   2186 HD11 LEU A 136      39.930  36.810 119.250  0.00  0.00
ATOM   2187 HD12 LEU A 136      40.610  37.000 120.850  0.00  0.00
ATOM   2188 HD13 LEU A 136      41.180  38.130 119.450  0.00  0.00
ATOM   2189  CD2 LEU A 136      38.230  37.860 121.240  0.00  0.00
ATOM   2190 HD21 LEU A 136      38.810  37.270 121.990  0.00  0.00
ATOM   2191 HD22 LEU A 136      37.780  37.130 120.530  0.00  0.00
```

```
ATOM   2192 HD23 LEU A 136      37.420  38.400 121.760  0.00  0.00
ATOM   2193  C   LEU A 136      39.280  41.850 122.500  0.00  0.00
ATOM   2194  O   LEU A 136      38.980  41.670 123.670  0.00  0.00
ATOM   2195  N   ALA A 137      39.920  42.980 122.130  0.00  0.00
ATOM   2196  HN  ALA A 137      40.040  43.180 121.160  0.00  0.00
ATOM   2197  CA  ALA A 137      40.320  43.910 123.180  0.00  0.00
ATOM   2198  HA  ALA A 137      40.870  43.320 123.900  0.00  0.00
ATOM   2199  CB  ALA A 137      41.330  44.880 122.500  0.00  0.00
ATOM   2200  HB1 ALA A 137      42.120  44.380 121.890  0.00  0.00
ATOM   2201  HB2 ALA A 137      41.730  45.530 123.310  0.00  0.00
ATOM   2202  HB3 ALA A 137      40.710  45.510 121.820  0.00  0.00
ATOM   2203  C   ALA A 137      39.280  44.690 123.970  0.00  0.00
ATOM   2204  O   ALA A 137      39.590  45.390 124.900  0.00  0.00
ATOM   2205  N   ILE A 138      38.000  44.640 123.520  0.00  0.00
ATOM   2206  HN  ILE A 138      37.840  44.110 122.690  0.00  0.00
ATOM   2207  CA  ILE A 138      36.790  45.170 124.230  0.00  0.00
ATOM   2208  HA  ILE A 138      37.120  45.750 125.070  0.00  0.00
ATOM   2209  CB  ILE A 138      36.030  46.190 123.450  0.00  0.00
ATOM   2210  HB  ILE A 138      35.200  46.610 124.060  0.00  0.00
ATOM   2211  CG2 ILE A 138      36.980  47.280 123.000  0.00  0.00
ATOM   2212 HG21 ILE A 138      37.840  46.940 122.380  0.00  0.00
ATOM   2213 HG22 ILE A 138      37.360  47.840 123.890  0.00  0.00
ATOM   2214 HG23 ILE A 138      36.590  48.150 122.440  0.00  0.00
ATOM   2215  CG1 ILE A 138      35.270  45.540 122.230  0.00  0.00
ATOM   2216 HG11 ILE A 138      34.450  44.910 122.630  0.00  0.00
ATOM   2217 HG12 ILE A 138      35.980  44.840 121.730  0.00  0.00
ATOM   2218  CD  ILE A 138      34.770  46.590 121.190  0.00  0.00
ATOM   2219  HD1 ILE A 138      34.120  47.290 121.760  0.00  0.00
ATOM   2220  HD2 ILE A 138      34.130  46.130 120.400  0.00  0.00
ATOM   2221  HD3 ILE A 138      35.570  47.120 120.630  0.00  0.00
ATOM   2222  C   ILE A 138      35.980  44.030 124.780  0.00  0.00
ATOM   2223  O   ILE A 138      34.900  44.240 125.220  0.00  0.00
ATOM   2224  N   VAL A 139      36.420  42.730 124.740  0.00  0.00
ATOM   2225  HN  VAL A 139      37.300  42.390 124.410  0.00  0.00
ATOM   2226  CA  VAL A 139      35.710  41.630 125.330  0.00  0.00
ATOM   2227  HA  VAL A 139      34.770  41.930 125.750  0.00  0.00
ATOM   2228  CB  VAL A 139      35.520  40.510 124.270  0.00  0.00
ATOM   2229  HB  VAL A 139      36.450  40.380 123.680  0.00  0.00
ATOM   2230  CG1 VAL A 139      35.190  39.070 124.830  0.00  0.00
ATOM   2231 HG11 VAL A 139      35.930  38.620 125.530  0.00  0.00
ATOM   2232 HG12 VAL A 139      35.030  38.370 123.990  0.00  0.00
ATOM   2233 HG13 VAL A 139      34.190  39.090 125.310  0.00  0.00
ATOM   2234  CG2 VAL A 139      34.480  40.990 123.290  0.00  0.00
ATOM   2235 HG21 VAL A 139      34.240  40.220 122.530  0.00  0.00
ATOM   2236 HG22 VAL A 139      34.880  41.820 122.660  0.00  0.00
ATOM   2237 HG23 VAL A 139      33.520  41.310 123.770  0.00  0.00
ATOM   2238  C   VAL A 139      36.490  41.120 126.510  0.00  0.00
ATOM   2239  O   VAL A 139      37.680  41.290 126.590  0.00  0.00
ATOM   2240  N   HSD A 140      35.680  40.600 127.400  0.00  0.00
ATOM   2241  HN  HSD A 140      34.700  40.540 127.200  0.00  0.00
ATOM   2242  CA  HSD A 140      36.020  40.140 128.790  0.00  0.00
ATOM   2243  HA  HSD A 140      36.220  41.040 129.360  0.00  0.00
ATOM   2244  CB  HSD A 140      34.890  39.380 129.480  0.00  0.00
ATOM   2245  HB1 HSD A 140      35.250  38.960 130.440  0.00  0.00
ATOM   2246  HB2 HSD A 140      34.550  38.560 128.810  0.00  0.00
ATOM   2247  ND1 HSD A 140      33.730  41.520 130.320  0.00  0.00
ATOM   2248  HD1 HSD A 140      34.570  41.980 130.610  0.00  0.00
ATOM   2249  CG  HSD A 140      33.660  40.220 129.800  0.00  0.00
ATOM   2250  CE1 HSD A 140      32.450  41.880 130.560  0.00  0.00
ATOM   2251  HE1 HSD A 140      32.150  42.900 130.810  0.00  0.00
ATOM   2252  NE2 HSD A 140      31.590  40.940 130.240  0.00  0.00
```

ATOM 2253 CD2 HSD A 140 32.370 39.900 129.810 0.00 0.00
ATOM 2254 HD2 HSD A 140 31.950 38.930 129.580 0.00 0.00
ATOM 2255 C HSD A 140 37.330 39.260 128.860 0.00 0.00
ATOM 2256 O HSD A 140 37.560 38.350 128.050 0.00 0.00
ATOM 2257 N ALA A 141 38.210 39.460 129.920 0.00 0.00
ATOM 2258 HN ALA A 141 37.960 40.050 130.680 0.00 0.00
ATOM 2259 CA ALA A 141 39.480 38.760 130.060 0.00 0.00
ATOM 2260 HA ALA A 141 40.160 38.990 129.260 0.00 0.00
ATOM 2261 CB ALA A 141 40.210 39.140 131.370 0.00 0.00
ATOM 2262 HB1 ALA A 141 39.670 38.910 132.310 0.00 0.00
ATOM 2263 HB2 ALA A 141 40.450 40.230 131.380 0.00 0.00
ATOM 2264 HB3 ALA A 141 41.220 38.690 131.410 0.00 0.00
ATOM 2265 C ALA A 141 39.410 37.240 130.130 0.00 0.00
ATOM 2266 O ALA A 141 40.250 36.590 129.460 0.00 0.00
ATOM 2267 N THR A 142 38.330 36.700 130.760 0.00 0.00
ATOM 2268 HN THR A 142 37.730 37.260 131.330 0.00 0.00
ATOM 2269 CA THR A 142 38.000 35.310 130.920 0.00 0.00
ATOM 2270 HA THR A 142 38.920 34.820 131.220 0.00 0.00
ATOM 2271 CB THR A 142 36.830 35.050 131.880 0.00 0.00
ATOM 2272 HB THR A 142 36.440 34.010 131.780 0.00 0.00
ATOM 2273 OG1 THR A 142 35.870 36.110 131.720 0.00 0.00
ATOM 2274 HG1 THR A 142 36.300 36.850 132.160 0.00 0.00
ATOM 2275 CG2 THR A 142 37.370 35.120 133.340 0.00 0.00
ATOM 2276 HG21 THR A 142 38.110 34.300 133.480 0.00 0.00
ATOM 2277 HG22 THR A 142 36.540 34.940 134.060 0.00 0.00
ATOM 2278 HG23 THR A 142 37.880 36.060 133.630 0.00 0.00
ATOM 2279 C THR A 142 37.700 34.690 129.530 0.00 0.00
ATOM 2280 O THR A 142 38.000 33.550 129.240 0.00 0.00
ATOM 2281 N ASN A 143 37.000 35.350 128.700 0.00 0.00
ATOM 2282 HN ASN A 143 36.760 36.240 129.090 0.00 0.00
ATOM 2283 CA ASN A 143 36.490 34.910 127.430 0.00 0.00
ATOM 2284 HA ASN A 143 36.340 33.840 127.450 0.00 0.00
ATOM 2285 CB ASN A 143 35.230 35.710 127.000 0.00 0.00
ATOM 2286 HB1 ASN A 143 34.800 35.440 126.010 0.00 0.00
ATOM 2287 HB2 ASN A 143 35.490 36.790 127.070 0.00 0.00
ATOM 2288 CG ASN A 143 34.210 35.470 128.140 0.00 0.00
ATOM 2289 OD1 ASN A 143 34.250 34.510 128.940 0.00 0.00
ATOM 2290 ND2 ASN A 143 33.260 36.390 128.360 0.00 0.00
ATOM 2291 HD21 ASN A 143 32.470 36.150 128.910 0.00 0.00
ATOM 2292 HD22 ASN A 143 33.280 37.080 127.640 0.00 0.00
ATOM 2293 C ASN A 143 37.490 35.050 126.330 0.00 0.00
ATOM 2294 O ASN A 143 37.600 34.210 125.440 0.00 0.00
ATOM 2295 N SER A 144 38.120 36.210 126.170 0.00 0.00
ATOM 2296 HN SER A 144 38.110 36.850 126.940 0.00 0.00
ATOM 2297 CA SER A 144 38.900 36.620 125.010 0.00 0.00
ATOM 2298 HA SER A 144 38.440 36.240 124.110 0.00 0.00
ATOM 2299 CB SER A 144 38.850 38.100 124.750 0.00 0.00
ATOM 2300 HB1 SER A 144 37.800 38.370 124.520 0.00 0.00
ATOM 2301 HB2 SER A 144 39.480 38.450 123.910 0.00 0.00
ATOM 2302 OG SER A 144 39.320 38.820 125.940 0.00 0.00
ATOM 2303 HG1 SER A 144 38.550 38.870 126.510 0.00 0.00
ATOM 2304 C SER A 144 40.350 36.130 124.850 0.00 0.00
ATOM 2305 O SER A 144 40.800 36.030 123.700 0.00 0.00
ATOM 2306 N GLN A 145 41.030 35.720 126.000 0.00 0.00
ATOM 2307 HN GLN A 145 40.560 35.710 126.890 0.00 0.00
ATOM 2308 CA GLN A 145 42.430 35.370 125.900 0.00 0.00
ATOM 2309 HA GLN A 145 42.930 36.160 125.360 0.00 0.00
ATOM 2310 CB GLN A 145 43.000 35.130 127.360 0.00 0.00
ATOM 2311 HB1 GLN A 145 42.420 34.210 127.620 0.00 0.00
ATOM 2312 HB2 GLN A 145 42.490 35.980 127.870 0.00 0.00
ATOM 2313 CG GLN A 145 44.570 34.970 127.590 0.00 0.00

ATOM 2314 HG1 GLN A 145 44.870 33.980 127.160 0.00 0.00
ATOM 2315 HG2 GLN A 145 44.660 34.970 128.700 0.00 0.00
ATOM 2316 CD GLN A 145 45.450 36.020 126.860 0.00 0.00
ATOM 2317 OE1 GLN A 145 45.060 37.050 126.430 0.00 0.00
ATOM 2318 NE2 GLN A 145 46.780 35.700 126.800 0.00 0.00
ATOM 2319 HE21 GLN A 145 47.090 34.850 127.230 0.00 0.00
ATOM 2320 HE22 GLN A 145 47.470 36.420 126.750 0.00 0.00
ATOM 2321 C GLN A 145 42.680 34.120 124.990 0.00 0.00
ATOM 2322 O GLN A 145 43.620 34.180 124.190 0.00 0.00
ATOM 2323 N ARG A 146 41.930 32.980 125.180 0.00 0.00
ATOM 2324 HN ARG A 146 41.270 32.970 125.930 0.00 0.00
ATOM 2325 CA ARG A 146 42.030 31.780 124.370 0.00 0.00
ATOM 2326 HA ARG A 146 43.030 31.430 124.550 0.00 0.00
ATOM 2327 CB ARG A 146 41.100 30.730 124.980 0.00 0.00
ATOM 2328 HB1 ARG A 146 40.100 31.190 125.100 0.00 0.00
ATOM 2329 HB2 ARG A 146 41.560 30.430 125.950 0.00 0.00
ATOM 2330 CG ARG A 146 41.000 29.370 124.190 0.00 0.00
ATOM 2331 HG1 ARG A 146 40.490 29.580 123.230 0.00 0.00
ATOM 2332 HG2 ARG A 146 40.340 28.680 124.750 0.00 0.00
ATOM 2333 CD ARG A 146 42.280 28.660 123.730 0.00 0.00
ATOM 2334 HD1 ARG A 146 42.940 28.220 124.510 0.00 0.00
ATOM 2335 HD2 ARG A 146 42.910 29.390 123.180 0.00 0.00
ATOM 2336 NE ARG A 146 41.790 27.640 122.750 0.00 0.00
ATOM 2337 HE ARG A 146 40.870 27.250 122.830 0.00 0.00
ATOM 2338 CZ ARG A 146 42.410 27.040 121.750 0.00 0.00
ATOM 2339 NH1 ARG A 146 43.610 27.380 121.310 0.00 0.00
ATOM 2340 HH11 ARG A 146 44.190 27.870 121.960 0.00 0.00
ATOM 2341 HH12 ARG A 146 44.120 26.820 120.650 0.00 0.00
ATOM 2342 NH2 ARG A 146 41.640 26.200 121.020 0.00 0.00
ATOM 2343 HH21 ARG A 146 40.760 25.890 121.350 0.00 0.00
ATOM 2344 HH22 ARG A 146 42.040 25.830 120.170 0.00 0.00
ATOM 2345 C ARG A 146 41.810 32.070 122.920 0.00 0.00
ATOM 2346 O ARG A 146 42.680 31.640 122.140 0.00 0.00
ATOM 2347 N PRO A 147 40.810 32.800 122.430 0.00 0.00
ATOM 2348 CD PRO A 147 39.420 32.800 122.960 0.00 0.00
ATOM 2349 HD1 PRO A 147 39.260 33.600 123.720 0.00 0.00
ATOM 2350 HD2 PRO A 147 39.070 31.780 123.240 0.00 0.00
ATOM 2351 CA PRO A 147 40.760 33.430 121.120 0.00 0.00
ATOM 2352 HA PRO A 147 40.680 32.550 120.490 0.00 0.00
ATOM 2353 CB PRO A 147 39.420 34.110 120.960 0.00 0.00
ATOM 2354 HB1 PRO A 147 39.060 34.190 119.910 0.00 0.00
ATOM 2355 HB2 PRO A 147 39.440 35.130 121.400 0.00 0.00
ATOM 2356 CG PRO A 147 38.480 33.180 121.780 0.00 0.00
ATOM 2357 HG1 PRO A 147 37.560 33.720 122.110 0.00 0.00
ATOM 2358 HG2 PRO A 147 38.270 32.250 121.220 0.00 0.00
ATOM 2359 C PRO A 147 41.910 34.230 120.590 0.00 0.00
ATOM 2360 O PRO A 147 42.430 33.940 119.540 0.00 0.00
ATOM 2361 N ARG A 148 42.400 35.240 121.410 0.00 0.00
ATOM 2362 HN ARG A 148 41.970 35.550 122.250 0.00 0.00
ATOM 2363 CA ARG A 148 43.600 36.000 121.090 0.00 0.00
ATOM 2364 HA ARG A 148 43.490 36.410 120.100 0.00 0.00
ATOM 2365 CB ARG A 148 43.740 37.160 122.200 0.00 0.00
ATOM 2366 HB1 ARG A 148 43.250 36.760 123.110 0.00 0.00
ATOM 2367 HB2 ARG A 148 43.080 37.990 121.860 0.00 0.00
ATOM 2368 CG ARG A 148 45.160 37.720 122.560 0.00 0.00
ATOM 2369 HG1 ARG A 148 45.800 37.820 121.660 0.00 0.00
ATOM 2370 HG2 ARG A 148 45.650 37.000 123.260 0.00 0.00
ATOM 2371 CD ARG A 148 45.030 39.110 123.270 0.00 0.00
ATOM 2372 HD1 ARG A 148 44.490 39.910 122.720 0.00 0.00
ATOM 2373 HD2 ARG A 148 46.040 39.360 123.660 0.00 0.00
ATOM 2374 NE ARG A 148 44.300 38.860 124.550 0.00 0.00

ATOM	2375	HE	ARG	A	148	44.640	38.190	125.210	0.00	0.00
ATOM	2376	CZ	ARG	A	148	43.040	39.260	124.830	0.00	0.00
ATOM	2377	NH1	ARG	A	148	42.220	39.940	124.080	0.00	0.00
ATOM	2378	HH11	ARG	A	148	41.560	40.500	124.570	0.00	0.00
ATOM	2379	HH12	ARG	A	148	42.600	40.280	123.210	0.00	0.00
ATOM	2380	NH2	ARG	A	148	42.490	38.910	125.980	0.00	0.00
ATOM	2381	HH21	ARG	A	148	41.570	39.260	126.150	0.00	0.00
ATOM	2382	HH22	ARG	A	148	43.050	38.450	126.670	0.00	0.00
ATOM	2383	C	ARG	A	148	44.920	35.170	121.060	0.00	0.00
ATOM	2384	O	ARG	A	148	45.660	35.270	120.090	0.00	0.00
ATOM	2385	N	LYS	A	149	45.140	34.200	121.980	0.00	0.00
ATOM	2386	HN	LYS	A	149	44.540	34.000	122.750	0.00	0.00
ATOM	2387	CA	LYS	A	149	46.070	33.160	121.930	0.00	0.00
ATOM	2388	HA	LYS	A	149	46.970	33.700	121.700	0.00	0.00
ATOM	2389	CB	LYS	A	149	46.130	32.250	123.160	0.00	0.00
ATOM	2390	HB1	LYS	A	149	46.880	31.440	123.030	0.00	0.00
ATOM	2391	HB2	LYS	A	149	45.170	31.770	123.430	0.00	0.00
ATOM	2392	CG	LYS	A	149	46.520	33.030	124.400	0.00	0.00
ATOM	2393	HG1	LYS	A	149	46.220	32.400	125.260	0.00	0.00
ATOM	2394	HG2	LYS	A	149	45.990	34.010	124.490	0.00	0.00
ATOM	2395	CD	LYS	A	149	48.050	33.340	124.500	0.00	0.00
ATOM	2396	HD1	LYS	A	149	48.220	33.870	125.460	0.00	0.00
ATOM	2397	HD2	LYS	A	149	48.360	34.140	123.800	0.00	0.00
ATOM	2398	CE	LYS	A	149	49.010	32.150	124.320	0.00	0.00
ATOM	2399	HE1	LYS	A	149	49.160	31.930	123.240	0.00	0.00
ATOM	2400	HE2	LYS	A	149	48.530	31.230	124.710	0.00	0.00
ATOM	2401	NZ	LYS	A	149	50.250	32.420	124.980	0.00	0.00
ATOM	2402	HZ1	LYS	A	149	50.700	33.280	124.610	0.00	0.00
ATOM	2403	HZ2	LYS	A	149	50.910	31.620	124.840	0.00	0.00
ATOM	2404	HZ3	LYS	A	149	50.150	32.650	125.990	0.00	0.00
ATOM	2405	C	LYS	A	149	46.090	32.260	120.700	0.00	0.00
ATOM	2406	O	LYS	A	149	47.140	32.020	120.070	0.00	0.00
ATOM	2407	N	LEU	A	150	44.950	31.710	120.320	0.00	0.00
ATOM	2408	HN	LEU	A	150	44.090	31.820	120.800	0.00	0.00
ATOM	2409	CA	LEU	A	150	44.730	31.000	119.090	0.00	0.00
ATOM	2410	HA	LEU	A	150	45.360	30.130	119.110	0.00	0.00
ATOM	2411	CB	LEU	A	150	43.310	30.480	119.000	0.00	0.00
ATOM	2412	HB1	LEU	A	150	42.570	31.310	118.940	0.00	0.00
ATOM	2413	HB2	LEU	A	150	43.210	29.880	119.940	0.00	0.00
ATOM	2414	CG	LEU	A	150	43.030	29.550	117.810	0.00	0.00
ATOM	2415	HG	LEU	A	150	43.330	30.070	116.870	0.00	0.00
ATOM	2416	CD1	LEU	A	150	43.990	28.320	117.840	0.00	0.00
ATOM	2417	HD11	LEU	A	150	44.060	27.930	118.880	0.00	0.00
ATOM	2418	HD12	LEU	A	150	45.030	28.620	117.580	0.00	0.00
ATOM	2419	HD13	LEU	A	150	43.650	27.630	117.040	0.00	0.00
ATOM	2420	CD2	LEU	A	150	41.570	29.100	117.850	0.00	0.00
ATOM	2421	HD21	LEU	A	150	41.280	28.870	118.890	0.00	0.00
ATOM	2422	HD22	LEU	A	150	41.490	28.200	117.190	0.00	0.00
ATOM	2423	HD23	LEU	A	150	40.900	29.860	117.380	0.00	0.00
ATOM	2424	C	LEU	A	150	45.080	31.830	117.880	0.00	0.00
ATOM	2425	O	LEU	A	150	45.610	31.420	116.890	0.00	0.00
ATOM	2426	N	LEU	A	151	44.600	33.100	117.880	0.00	0.00
ATOM	2427	HN	LEU	A	151	44.130	33.490	118.660	0.00	0.00
ATOM	2428	CA	LEU	A	151	44.790	34.020	116.730	0.00	0.00
ATOM	2429	HA	LEU	A	151	44.390	33.560	115.840	0.00	0.00
ATOM	2430	CB	LEU	A	151	44.040	35.360	117.100	0.00	0.00
ATOM	2431	HB1	LEU	A	151	44.510	36.000	117.870	0.00	0.00
ATOM	2432	HB2	LEU	A	151	43.020	35.190	117.510	0.00	0.00
ATOM	2433	CG	LEU	A	151	43.830	36.390	115.900	0.00	0.00
ATOM	2434	HG	LEU	A	151	44.800	36.580	115.410	0.00	0.00
ATOM	2435	CD1	LEU	A	151	42.980	35.620	114.850	0.00	0.00

ATOM	2436	HD11	LEU	A	151	42.410	36.220	114.100	0.00	0.00
ATOM	2437	HD12	LEU	A	151	42.210	34.990	115.340	0.00	0.00
ATOM	2438	HD13	LEU	A	151	43.650	34.960	114.250	0.00	0.00
ATOM	2439	CD2	LEU	A	151	43.210	37.710	116.380	0.00	0.00
ATOM	2440	HD21	LEU	A	151	43.800	38.070	117.240	0.00	0.00
ATOM	2441	HD22	LEU	A	151	42.190	37.640	116.820	0.00	0.00
ATOM	2442	HD23	LEU	A	151	43.270	38.410	115.510	0.00	0.00
ATOM	2443	C	LEU	A	151	46.260	34.230	116.510	0.00	0.00
ATOM	2444	O	LEU	A	151	46.840	34.090	115.450	0.00	0.00
ATOM	2445	N	ALA	A	152	47.020	34.520	117.620	0.00	0.00
ATOM	2446	HN	ALA	A	152	46.540	34.720	118.470	0.00	0.00
ATOM	2447	CA	ALA	A	152	48.380	34.970	117.590	0.00	0.00
ATOM	2448	HA	ALA	A	152	48.480	35.860	116.980	0.00	0.00
ATOM	2449	CB	ALA	A	152	48.920	35.470	118.960	0.00	0.00
ATOM	2450	HB1	ALA	A	152	48.270	36.280	119.370	0.00	0.00
ATOM	2451	HB2	ALA	A	152	49.900	35.960	118.790	0.00	0.00
ATOM	2452	HB3	ALA	A	152	48.970	34.600	119.640	0.00	0.00
ATOM	2453	C	ALA	A	152	49.410	33.830	117.190	0.00	0.00
ATOM	2454	O	ALA	A	152	50.250	34.040	116.370	0.00	0.00
ATOM	2455	N	GLU	A	153	49.250	32.590	117.780	0.00	0.00
ATOM	2456	HN	GLU	A	153	48.530	32.480	118.460	0.00	0.00
ATOM	2457	CA	GLU	A	153	50.030	31.400	117.500	0.00	0.00
ATOM	2458	HA	GLU	A	153	51.070	31.670	117.370	0.00	0.00
ATOM	2459	CB	GLU	A	153	49.870	30.360	118.690	0.00	0.00
ATOM	2460	HB1	GLU	A	153	50.500	29.470	118.490	0.00	0.00
ATOM	2461	HB2	GLU	A	153	48.800	30.090	118.790	0.00	0.00
ATOM	2462	CG	GLU	A	153	50.410	31.020	120.050	0.00	0.00
ATOM	2463	HG1	GLU	A	153	49.750	31.770	120.530	0.00	0.00
ATOM	2464	HG2	GLU	A	153	51.420	31.430	119.840	0.00	0.00
ATOM	2465	CD	GLU	A	153	50.620	29.960	121.130	0.00	0.00
ATOM	2466	OE1	GLU	A	153	49.690	29.350	121.700	0.00	0.00
ATOM	2467	OE2	GLU	A	153	51.840	29.800	121.470	0.00	0.00
ATOM	2468	C	GLU	A	153	49.710	30.550	116.340	0.00	0.00
ATOM	2469	O	GLU	A	153	50.550	29.780	115.860	0.00	0.00
ATOM	2470	N	LYS	A	154	48.540	30.620	115.780	0.00	0.00
ATOM	2471	HN	LYS	A	154	47.930	31.320	116.150	0.00	0.00
ATOM	2472	CA	LYS	A	154	48.190	29.800	114.620	0.00	0.00
ATOM	2473	HA	LYS	A	154	49.030	29.440	114.050	0.00	0.00
ATOM	2474	CB	LYS	A	154	47.270	28.610	114.940	0.00	0.00
ATOM	2475	HB1	LYS	A	154	47.060	28.020	114.030	0.00	0.00
ATOM	2476	HB2	LYS	A	154	46.330	28.910	115.470	0.00	0.00
ATOM	2477	CG	LYS	A	154	47.930	27.570	115.900	0.00	0.00
ATOM	2478	HG1	LYS	A	154	48.020	28.030	116.900	0.00	0.00
ATOM	2479	HG2	LYS	A	154	48.990	27.500	115.570	0.00	0.00
ATOM	2480	CD	LYS	A	154	47.070	26.310	116.000	0.00	0.00
ATOM	2481	HD1	LYS	A	154	46.620	26.100	115.000	0.00	0.00
ATOM	2482	HD2	LYS	A	154	46.210	26.410	116.690	0.00	0.00
ATOM	2483	CE	LYS	A	154	47.870	25.100	116.500	0.00	0.00
ATOM	2484	HE1	LYS	A	154	48.600	24.750	115.740	0.00	0.00
ATOM	2485	HE2	LYS	A	154	47.260	24.180	116.650	0.00	0.00
ATOM	2486	NZ	LYS	A	154	48.570	25.340	117.760	0.00	0.00
ATOM	2487	HZ1	LYS	A	154	49.410	25.950	117.640	0.00	0.00
ATOM	2488	HZ2	LYS	A	154	48.860	24.440	118.200	0.00	0.00
ATOM	2489	HZ3	LYS	A	154	47.970	25.820	118.460	0.00	0.00
ATOM	2490	C	LYS	A	154	47.580	30.760	113.560	0.00	0.00
ATOM	2491	O	LYS	A	154	48.180	30.970	112.550	0.00	0.00
ATOM	2492	N	VAL	A	155	46.320	31.280	113.780	0.00	0.00
ATOM	2493	HN	VAL	A	155	45.810	31.200	114.630	0.00	0.00
ATOM	2494	CA	VAL	A	155	45.490	31.840	112.680	0.00	0.00
ATOM	2495	HA	VAL	A	155	45.400	31.000	112.000	0.00	0.00
ATOM	2496	CB	VAL	A	155	44.070	32.020	113.060	0.00	0.00

ATOM	2497	HB	VAL	A	155	44.030	32.780	113.870	0.00	0.00
ATOM	2498	CG1	VAL	A	155	43.300	32.350	111.860	0.00	0.00
ATOM	2499	HG11	VAL	A	155	43.630	33.280	111.340	0.00	0.00
ATOM	2500	HG12	VAL	A	155	42.260	32.560	112.180	0.00	0.00
ATOM	2501	HG13	VAL	A	155	43.310	31.590	111.050	0.00	0.00
ATOM	2502	CG2	VAL	A	155	43.540	30.810	113.750	0.00	0.00
ATOM	2503	HG21	VAL	A	155	42.440	30.850	113.860	0.00	0.00
ATOM	2504	HG22	VAL	A	155	43.930	30.490	114.730	0.00	0.00
ATOM	2505	HG23	VAL	A	155	43.680	29.880	113.160	0.00	0.00
ATOM	2506	C	VAL	A	155	46.130	33.000	111.910	0.00	0.00
ATOM	2507	O	VAL	A	155	45.960	33.030	110.690	0.00	0.00
ATOM	2508	N	VAL	A	156	46.880	33.980	112.580	0.00	0.00
ATOM	2509	HN	VAL	A	156	47.080	33.890	113.550	0.00	0.00
ATOM	2510	CA	VAL	A	156	47.540	35.110	111.940	0.00	0.00
ATOM	2511	HA	VAL	A	156	46.700	35.500	111.390	0.00	0.00
ATOM	2512	CB	VAL	A	156	48.020	36.270	112.900	0.00	0.00
ATOM	2513	HB	VAL	A	156	47.230	36.420	113.670	0.00	0.00
ATOM	2514	CG1	VAL	A	156	49.240	35.930	113.770	0.00	0.00
ATOM	2515	HG11	VAL	A	156	48.950	35.120	114.470	0.00	0.00
ATOM	2516	HG12	VAL	A	156	49.410	36.890	114.310	0.00	0.00
ATOM	2517	HG13	VAL	A	156	50.120	35.520	113.240	0.00	0.00
ATOM	2518	CG2	VAL	A	156	48.220	37.520	112.070	0.00	0.00
ATOM	2519	HG21	VAL	A	156	49.200	37.510	111.540	0.00	0.00
ATOM	2520	HG22	VAL	A	156	48.320	38.370	112.780	0.00	0.00
ATOM	2521	HG23	VAL	A	156	47.380	37.660	111.350	0.00	0.00
ATOM	2522	C	VAL	A	156	48.590	34.640	110.990	0.00	0.00
ATOM	2523	O	VAL	A	156	48.790	35.130	109.870	0.00	0.00
ATOM	2524	N	TYR	A	157	49.360	33.620	111.410	0.00	0.00
ATOM	2525	HN	TYR	A	157	49.150	33.270	112.310	0.00	0.00
ATOM	2526	CA	TYR	A	157	50.480	33.120	110.620	0.00	0.00
ATOM	2527	HA	TYR	A	157	51.010	33.980	110.240	0.00	0.00
ATOM	2528	CB	TYR	A	157	51.320	31.960	111.250	0.00	0.00
ATOM	2529	HB1	TYR	A	157	52.100	31.610	110.540	0.00	0.00
ATOM	2530	HB2	TYR	A	157	50.650	31.070	111.350	0.00	0.00
ATOM	2531	CG	TYR	A	157	52.020	32.230	112.530	0.00	0.00
ATOM	2532	CD1	TYR	A	157	52.560	33.490	112.940	0.00	0.00
ATOM	2533	HD1	TYR	A	157	52.470	34.350	112.290	0.00	0.00
ATOM	2534	CE1	TYR	A	157	53.260	33.680	114.180	0.00	0.00
ATOM	2535	HE1	TYR	A	157	53.570	34.660	114.510	0.00	0.00
ATOM	2536	CZ	TYR	A	157	53.400	32.550	114.970	0.00	0.00
ATOM	2537	OH	TYR	A	157	53.980	32.690	116.330	0.00	0.00
ATOM	2538	HH	TYR	A	157	54.460	31.880	116.550	0.00	0.00
ATOM	2539	CD2	TYR	A	157	52.340	31.110	113.260	0.00	0.00
ATOM	2540	HD2	TYR	A	157	52.040	30.120	112.960	0.00	0.00
ATOM	2541	CE2	TYR	A	157	53.040	31.260	114.510	0.00	0.00
ATOM	2542	HE2	TYR	A	157	53.300	30.410	115.120	0.00	0.00
ATOM	2543	C	TYR	A	157	49.980	32.580	109.300	0.00	0.00
ATOM	2544	O	TYR	A	157	50.410	32.910	108.190	0.00	0.00
ATOM	2545	N	VAL	A	158	48.930	31.720	109.350	0.00	0.00
ATOM	2546	HN	VAL	A	158	48.480	31.340	110.160	0.00	0.00
ATOM	2547	CA	VAL	A	158	48.490	31.160	108.120	0.00	0.00
ATOM	2548	HA	VAL	A	158	49.330	31.110	107.450	0.00	0.00
ATOM	2549	CB	VAL	A	158	47.860	29.800	108.230	0.00	0.00
ATOM	2550	HB	VAL	A	158	47.440	29.490	107.250	0.00	0.00
ATOM	2551	CG1	VAL	A	158	48.830	28.810	108.810	0.00	0.00
ATOM	2552	HG11	VAL	A	158	48.390	27.790	108.820	0.00	0.00
ATOM	2553	HG12	VAL	A	158	49.050	29.120	109.850	0.00	0.00
ATOM	2554	HG13	VAL	A	158	49.790	28.690	108.260	0.00	0.00
ATOM	2555	CG2	VAL	A	158	46.740	29.850	109.210	0.00	0.00
ATOM	2556	HG21	VAL	A	158	46.490	28.830	109.600	0.00	0.00
ATOM	2557	HG22	VAL	A	158	45.830	30.180	108.660	0.00	0.00

ATOM	2558	HG23	VAL	A	158	46.870	30.560	110.050	0.00	0.00
ATOM	2559	C	VAL	A	158	47.440	31.980	107.340	0.00	0.00
ATOM	2560	O	VAL	A	158	47.070	31.580	106.260	0.00	0.00
ATOM	2561	N	GLY	A	159	47.030	33.210	107.950	0.00	0.00
ATOM	2562	HN	GLY	A	159	47.340	33.450	108.860	0.00	0.00
ATOM	2563	CA	GLY	A	159	46.060	34.060	107.290	0.00	0.00
ATOM	2564	HA1	GLY	A	159	45.600	34.610	108.100	0.00	0.00
ATOM	2565	HA2	GLY	A	159	45.410	33.590	106.580	0.00	0.00
ATOM	2566	C	GLY	A	159	46.760	35.070	106.450	0.00	0.00
ATOM	2567	O	GLY	A	159	46.120	35.520	105.500	0.00	0.00
ATOM	2568	N	VAL	A	160	48.010	35.430	106.710	0.00	0.00
ATOM	2569	HN	VAL	A	160	48.570	35.160	107.490	0.00	0.00
ATOM	2570	CA	VAL	A	160	48.720	36.560	106.030	0.00	0.00
ATOM	2571	HA	VAL	A	160	48.080	36.930	105.240	0.00	0.00
ATOM	2572	CB	VAL	A	160	49.040	37.770	106.840	0.00	0.00
ATOM	2573	HB	VAL	A	160	49.790	37.590	107.640	0.00	0.00
ATOM	2574	CG1	VAL	A	160	49.600	38.900	105.920	0.00	0.00
ATOM	2575	HG11	VAL	A	160	50.650	38.640	105.670	0.00	0.00
ATOM	2576	HG12	VAL	A	160	49.580	39.900	106.420	0.00	0.00
ATOM	2577	HG13	VAL	A	160	48.960	38.980	105.010	0.00	0.00
ATOM	2578	CG2	VAL	A	160	47.780	38.310	107.580	0.00	0.00
ATOM	2579	HG21	VAL	A	160	47.950	39.040	108.400	0.00	0.00
ATOM	2580	HG22	VAL	A	160	47.190	37.540	108.130	0.00	0.00
ATOM	2581	HG23	VAL	A	160	47.070	38.740	106.850	0.00	0.00
ATOM	2582	C	VAL	A	160	49.960	35.960	105.390	0.00	0.00
ATOM	2583	O	VAL	A	160	50.020	35.710	104.220	0.00	0.00
ATOM	2584	N	TRP	A	161	50.990	35.590	106.210	0.00	0.00
ATOM	2585	HN	TRP	A	161	50.910	35.610	107.200	0.00	0.00
ATOM	2586	CA	TRP	A	161	52.230	35.120	105.660	0.00	0.00
ATOM	2587	HA	TRP	A	161	52.790	35.860	105.100	0.00	0.00
ATOM	2588	CB	TRP	A	161	53.280	34.670	106.810	0.00	0.00
ATOM	2589	HB1	TRP	A	161	54.350	34.600	106.520	0.00	0.00
ATOM	2590	HB2	TRP	A	161	53.040	33.650	107.170	0.00	0.00
ATOM	2591	CG	TRP	A	161	53.260	35.640	108.000	0.00	0.00
ATOM	2592	CD1	TRP	A	161	53.140	35.330	109.340	0.00	0.00
ATOM	2593	HD1	TRP	A	161	53.160	34.290	109.620	0.00	0.00
ATOM	2594	NE1	TRP	A	161	52.920	36.500	110.070	0.00	0.00
ATOM	2595	HE1	TRP	A	161	52.940	36.740	111.020	0.00	0.00
ATOM	2596	CE2	TRP	A	161	52.930	37.550	109.260	0.00	0.00
ATOM	2597	CD2	TRP	A	161	53.180	37.070	107.950	0.00	0.00
ATOM	2598	CE3	TRP	A	161	53.120	37.990	106.880	0.00	0.00
ATOM	2599	HE3	TRP	A	161	53.270	37.630	105.880	0.00	0.00
ATOM	2600	CZ3	TRP	A	161	52.870	39.370	107.120	0.00	0.00
ATOM	2601	HZ3	TRP	A	161	52.710	40.120	106.360	0.00	0.00
ATOM	2602	CZ2	TRP	A	161	52.760	38.930	109.510	0.00	0.00
ATOM	2603	HZ2	TRP	A	161	52.500	39.330	110.480	0.00	0.00
ATOM	2604	CH2	TRP	A	161	52.670	39.830	108.400	0.00	0.00
ATOM	2605	HH2	TRP	A	161	52.510	40.870	108.600	0.00	0.00
ATOM	2606	C	TRP	A	161	52.130	33.930	104.610	0.00	0.00
ATOM	2607	O	TRP	A	161	52.810	33.930	103.570	0.00	0.00
ATOM	2608	N	ILE	A	162	51.220	32.880	104.880	0.00	0.00
ATOM	2609	HN	ILE	A	162	50.750	33.010	105.750	0.00	0.00
ATOM	2610	CA	ILE	A	162	51.080	31.770	103.970	0.00	0.00
ATOM	2611	HA	ILE	A	162	52.080	31.550	103.650	0.00	0.00
ATOM	2612	CB	ILE	A	162	50.600	30.530	104.700	0.00	0.00
ATOM	2613	HB	ILE	A	162	49.630	30.790	105.180	0.00	0.00
ATOM	2614	CG2	ILE	A	162	50.390	29.290	103.680	0.00	0.00
ATOM	2615	HG21	ILE	A	162	51.340	28.850	103.320	0.00	0.00
ATOM	2616	HG22	ILE	A	162	49.780	29.590	102.790	0.00	0.00
ATOM	2617	HG23	ILE	A	162	49.850	28.500	104.250	0.00	0.00
ATOM	2618	CG1	ILE	A	162	51.490	30.030	105.890	0.00	0.00

ATOM 2619 HG11 ILE A 162 51.040 29.090 106.280 0.00 0.00
ATOM 2620 HG12 ILE A 162 51.370 30.810 106.680 0.00 0.00
ATOM 2621 CD ILE A 162 52.940 29.760 105.570 0.00 0.00
ATOM 2622 HD1 ILE A 162 53.550 29.740 106.500 0.00 0.00
ATOM 2623 HD2 ILE A 162 53.370 30.640 105.050 0.00 0.00
ATOM 2624 HD3 ILE A 162 53.110 28.830 104.990 0.00 0.00
ATOM 2625 C ILE A 162 50.280 32.000 102.660 0.00 0.00
ATOM 2626 O ILE A 162 50.760 31.670 101.600 0.00 0.00
ATOM 2627 N PRO A 163 49.080 32.650 102.570 0.00 0.00
ATOM 2628 CD PRO A 163 48.120 32.800 103.640 0.00 0.00
ATOM 2629 HD1 PRO A 163 48.570 33.250 104.560 0.00 0.00
ATOM 2630 HD2 PRO A 163 47.800 31.770 103.910 0.00 0.00
ATOM 2631 CA PRO A 163 48.420 32.860 101.300 0.00 0.00
ATOM 2632 HA PRO A 163 48.420 31.940 100.740 0.00 0.00
ATOM 2633 CB PRO A 163 47.000 33.300 101.640 0.00 0.00
ATOM 2634 HB1 PRO A 163 46.310 32.460 101.450 0.00 0.00
ATOM 2635 HB2 PRO A 163 46.610 34.070 100.940 0.00 0.00
ATOM 2636 CG PRO A 163 47.120 33.790 103.060 0.00 0.00
ATOM 2637 HG1 PRO A 163 47.560 34.810 103.020 0.00 0.00
ATOM 2638 HG2 PRO A 163 46.170 33.750 103.640 0.00 0.00
ATOM 2639 C PRO A 163 49.230 33.950 100.540 0.00 0.00
ATOM 2640 O PRO A 163 49.260 33.900 99.330 0.00 0.00
ATOM 2641 N ALA A 164 49.820 34.990 101.180 0.00 0.00
ATOM 2642 HN ALA A 164 49.790 34.960 102.180 0.00 0.00
ATOM 2643 CA ALA A 164 50.620 35.980 100.520 0.00 0.00
ATOM 2644 HA ALA A 164 49.920 36.410 99.820 0.00 0.00
ATOM 2645 CB ALA A 164 51.180 37.010 101.600 0.00 0.00
ATOM 2646 HB1 ALA A 164 51.920 36.440 102.190 0.00 0.00
ATOM 2647 HB2 ALA A 164 50.510 37.350 102.420 0.00 0.00
ATOM 2648 HB3 ALA A 164 51.710 37.870 101.130 0.00 0.00
ATOM 2649 C ALA A 164 51.840 35.410 99.730 0.00 0.00
ATOM 2650 O ALA A 164 52.030 35.800 98.580 0.00 0.00
ATOM 2651 N LEU A 165 52.630 34.470 100.300 0.00 0.00
ATOM 2652 HN LEU A 165 52.470 34.190 101.240 0.00 0.00
ATOM 2653 CA LEU A 165 53.650 33.580 99.700 0.00 0.00
ATOM 2654 HA LEU A 165 54.460 34.200 99.330 0.00 0.00
ATOM 2655 CB LEU A 165 54.220 32.690 100.740 0.00 0.00
ATOM 2656 HB1 LEU A 165 53.340 32.080 101.040 0.00 0.00
ATOM 2657 HB2 LEU A 165 54.430 33.360 101.600 0.00 0.00
ATOM 2658 CG LEU A 165 55.350 31.810 100.340 0.00 0.00
ATOM 2659 HG LEU A 165 55.100 31.380 99.350 0.00 0.00
ATOM 2660 CD1 LEU A 165 56.690 32.510 100.060 0.00 0.00
ATOM 2661 HD11 LEU A 165 56.640 33.280 99.270 0.00 0.00
ATOM 2662 HD12 LEU A 165 57.410 31.780 99.630 0.00 0.00
ATOM 2663 HD13 LEU A 165 57.140 32.960 100.970 0.00 0.00
ATOM 2664 CD2 LEU A 165 55.500 30.710 101.460 0.00 0.00
ATOM 2665 HD21 LEU A 165 56.280 30.010 101.100 0.00 0.00
ATOM 2666 HD22 LEU A 165 54.560 30.130 101.580 0.00 0.00
ATOM 2667 HD23 LEU A 165 55.770 31.230 102.400 0.00 0.00
ATOM 2668 C LEU A 165 53.100 32.780 98.470 0.00 0.00
ATOM 2669 O LEU A 165 53.680 32.750 97.400 0.00 0.00
ATOM 2670 N LEU A 166 51.870 32.290 98.640 0.00 0.00
ATOM 2671 HN LEU A 166 51.360 32.400 99.500 0.00 0.00
ATOM 2672 CA LEU A 166 51.270 31.560 97.480 0.00 0.00
ATOM 2673 HA LEU A 166 51.870 30.700 97.260 0.00 0.00
ATOM 2674 CB LEU A 166 49.950 30.990 97.970 0.00 0.00
ATOM 2675 HB1 LEU A 166 49.410 31.900 98.330 0.00 0.00
ATOM 2676 HB2 LEU A 166 50.130 30.350 98.860 0.00 0.00
ATOM 2677 CG LEU A 166 49.010 30.350 96.880 0.00 0.00
ATOM 2678 HG LEU A 166 48.780 31.090 96.090 0.00 0.00
ATOM 2679 CD1 LEU A 166 49.550 29.010 96.340 0.00 0.00

ATOM	2680	HD11	LEU	A	166	48.810	28.440	95.740	0.00	0.00
ATOM	2681	HD12	LEU	A	166	49.870	28.230	97.060	0.00	0.00
ATOM	2682	HD13	LEU	A	166	50.420	29.360	95.740	0.00	0.00
ATOM	2683	CD2	LEU	A	166	47.690	30.010	97.630	0.00	0.00
ATOM	2684	HD21	LEU	A	166	47.360	30.990	98.030	0.00	0.00
ATOM	2685	HD22	LEU	A	166	47.840	29.310	98.480	0.00	0.00
ATOM	2686	HD23	LEU	A	166	46.960	29.670	96.870	0.00	0.00
ATOM	2687	C	LEU	A	166	51.060	32.340	96.160	0.00	0.00
ATOM	2688	O	LEU	A	166	51.360	31.880	95.070	0.00	0.00
ATOM	2689	N	LEU	A	167	50.620	33.610	96.340	0.00	0.00
ATOM	2690	HN	LEU	A	167	50.460	33.850	97.290	0.00	0.00
ATOM	2691	CA	LEU	A	167	50.330	34.580	95.230	0.00	0.00
ATOM	2692	HA	LEU	A	167	49.950	34.120	94.340	0.00	0.00
ATOM	2693	CB	LEU	A	167	49.110	35.530	95.690	0.00	0.00
ATOM	2694	HB1	LEU	A	167	48.830	36.270	94.910	0.00	0.00
ATOM	2695	HB2	LEU	A	167	49.460	36.130	96.560	0.00	0.00
ATOM	2696	CG	LEU	A	167	47.810	34.800	96.100	0.00	0.00
ATOM	2697	HG	LEU	A	167	48.040	34.020	96.850	0.00	0.00
ATOM	2698	CD1	LEU	A	167	46.860	35.810	96.810	0.00	0.00
ATOM	2699	HD11	LEU	A	167	47.460	36.090	97.710	0.00	0.00
ATOM	2700	HD12	LEU	A	167	45.850	35.410	97.020	0.00	0.00
ATOM	2701	HD13	LEU	A	167	46.760	36.660	96.100	0.00	0.00
ATOM	2702	CD2	LEU	A	167	47.130	34.180	94.920	0.00	0.00
ATOM	2703	HD21	LEU	A	167	47.680	33.260	94.610	0.00	0.00
ATOM	2704	HD22	LEU	A	167	47.160	34.800	94.000	0.00	0.00
ATOM	2705	HD23	LEU	A	167	46.070	33.980	95.190	0.00	0.00
ATOM	2706	C	LEU	A	167	51.590	35.360	94.830	0.00	0.00
ATOM	2707	O	LEU	A	167	51.660	35.950	93.760	0.00	0.00
ATOM	2708	N	THR	A	168	52.630	35.220	95.610	0.00	0.00
ATOM	2709	HN	THR	A	168	52.470	34.720	96.460	0.00	0.00
ATOM	2710	CA	THR	A	168	54.020	35.500	95.170	0.00	0.00
ATOM	2711	HA	THR	A	168	53.950	36.500	94.780	0.00	0.00
ATOM	2712	CB	THR	A	168	55.080	35.550	96.290	0.00	0.00
ATOM	2713	HB	THR	A	168	55.280	34.610	96.850	0.00	0.00
ATOM	2714	OG1	THR	A	168	54.610	36.510	97.270	0.00	0.00
ATOM	2715	HG1	THR	A	168	53.790	36.150	97.630	0.00	0.00
ATOM	2716	CG2	THR	A	168	56.380	36.180	95.700	0.00	0.00
ATOM	2717	HG21	THR	A	168	56.860	35.520	94.950	0.00	0.00
ATOM	2718	HG22	THR	A	168	57.000	36.550	96.550	0.00	0.00
ATOM	2719	HG23	THR	A	168	56.030	37.110	95.200	0.00	0.00
ATOM	2720	C	THR	A	168	54.580	34.590	93.980	0.00	0.00
ATOM	2721	O	THR	A	168	55.270	35.100	93.070	0.00	0.00
ATOM	2722	N	ILE	A	169	54.290	33.290	94.060	0.00	0.00
ATOM	2723	HN	ILE	A	169	53.700	32.920	94.770	0.00	0.00
ATOM	2724	CA	ILE	A	169	54.730	32.290	93.120	0.00	0.00
ATOM	2725	HA	ILE	A	169	55.810	32.290	93.120	0.00	0.00
ATOM	2726	CB	ILE	A	169	54.350	30.880	93.550	0.00	0.00
ATOM	2727	HB	ILE	A	169	53.270	30.790	93.780	0.00	0.00
ATOM	2728	CG2	ILE	A	169	54.560	29.800	92.420	0.00	0.00
ATOM	2729	HG21	ILE	A	169	54.200	30.210	91.450	0.00	0.00
ATOM	2730	HG22	ILE	A	169	54.090	28.800	92.540	0.00	0.00
ATOM	2731	HG23	ILE	A	169	55.640	29.690	92.190	0.00	0.00
ATOM	2732	CG1	ILE	A	169	55.030	30.440	94.910	0.00	0.00
ATOM	2733	HG11	ILE	A	169	55.100	31.310	95.600	0.00	0.00
ATOM	2734	HG12	ILE	A	169	56.090	30.140	94.780	0.00	0.00
ATOM	2735	CD	ILE	A	169	54.230	29.260	95.610	0.00	0.00
ATOM	2736	HD1	ILE	A	169	54.210	28.290	95.070	0.00	0.00
ATOM	2737	HD2	ILE	A	169	53.200	29.560	95.890	0.00	0.00
ATOM	2738	HD3	ILE	A	169	54.710	28.990	96.570	0.00	0.00
ATOM	2739	C	ILE	A	169	54.340	32.690	91.600	0.00	0.00
ATOM	2740	O	ILE	A	169	55.240	32.570	90.760	0.00	0.00

ATOM	2741	N	PRO	A	170	53.160	33.180	91.270	0.00	0.00
ATOM	2742	CD	PRO	A	170	51.980	32.890	92.020	0.00	0.00
ATOM	2743	HD1	PRO	A	170	52.160	33.080	93.100	0.00	0.00
ATOM	2744	HD2	PRO	A	170	51.780	31.810	91.830	0.00	0.00
ATOM	2745	CA	PRO	A	170	52.900	33.470	89.890	0.00	0.00
ATOM	2746	HA	PRO	A	170	53.360	32.720	89.270	0.00	0.00
ATOM	2747	CB	PRO	A	170	51.340	33.470	89.810	0.00	0.00
ATOM	2748	HB1	PRO	A	170	50.980	32.490	89.440	0.00	0.00
ATOM	2749	HB2	PRO	A	170	50.970	34.330	89.210	0.00	0.00
ATOM	2750	CG	PRO	A	170	50.950	33.690	91.250	0.00	0.00
ATOM	2751	HG1	PRO	A	170	51.020	34.790	91.340	0.00	0.00
ATOM	2752	HG2	PRO	A	170	49.870	33.470	91.410	0.00	0.00
ATOM	2753	C	PRO	A	170	53.530	34.730	89.500	0.00	0.00
ATOM	2754	O	PRO	A	170	54.170	34.750	88.450	0.00	0.00
ATOM	2755	N	ASP	A	171	53.400	35.860	90.210	0.00	0.00
ATOM	2756	HN	ASP	A	171	52.810	35.850	91.000	0.00	0.00
ATOM	2757	CA	ASP	A	171	53.960	37.130	89.820	0.00	0.00
ATOM	2758	HA	ASP	A	171	53.640	37.350	88.810	0.00	0.00
ATOM	2759	CB	ASP	A	171	53.550	38.330	90.710	0.00	0.00
ATOM	2760	HB1	ASP	A	171	54.120	39.220	90.360	0.00	0.00
ATOM	2761	HB2	ASP	A	171	53.730	38.130	91.790	0.00	0.00
ATOM	2762	CG	ASP	A	171	52.090	38.640	90.680	0.00	0.00
ATOM	2763	OD1	ASP	A	171	51.350	38.280	89.680	0.00	0.00
ATOM	2764	OD2	ASP	A	171	51.600	39.320	91.610	0.00	0.00
ATOM	2765	C	ASP	A	171	55.440	37.070	89.640	0.00	0.00
ATOM	2766	O	ASP	A	171	55.940	37.600	88.660	0.00	0.00
ATOM	2767	N	PHE	A	172	56.200	36.330	90.490	0.00	0.00
ATOM	2768	HN	PHE	A	172	55.760	35.970	91.310	0.00	0.00
ATOM	2769	CA	PHE	A	172	57.640	36.140	90.320	0.00	0.00
ATOM	2770	HA	PHE	A	172	57.990	37.150	90.220	0.00	0.00
ATOM	2771	CB	PHE	A	172	58.320	35.410	91.600	0.00	0.00
ATOM	2772	HB1	PHE	A	172	57.600	34.570	91.690	0.00	0.00
ATOM	2773	HB2	PHE	A	172	58.240	36.020	92.520	0.00	0.00
ATOM	2774	CG	PHE	A	172	59.600	34.710	91.440	0.00	0.00
ATOM	2775	CD1	PHE	A	172	60.600	35.350	90.790	0.00	0.00
ATOM	2776	HD1	PHE	A	172	60.490	36.400	90.550	0.00	0.00
ATOM	2777	CE1	PHE	A	172	61.780	34.700	90.420	0.00	0.00
ATOM	2778	HE1	PHE	A	172	62.560	35.310	89.970	0.00	0.00
ATOM	2779	CZ	PHE	A	172	61.980	33.390	90.720	0.00	0.00
ATOM	2780	HZ	PHE	A	172	62.810	32.820	90.320	0.00	0.00
ATOM	2781	CD2	PHE	A	172	59.740	33.370	91.770	0.00	0.00
ATOM	2782	HD2	PHE	A	172	59.020	32.730	92.240	0.00	0.00
ATOM	2783	CE2	PHE	A	172	60.940	32.730	91.350	0.00	0.00
ATOM	2784	HE2	PHE	A	172	61.090	31.690	91.590	0.00	0.00
ATOM	2785	C	PHE	A	172	57.970	35.430	88.980	0.00	0.00
ATOM	2786	O	PHE	A	172	58.800	35.900	88.230	0.00	0.00
ATOM	2787	N	ILE	A	173	57.320	34.360	88.660	0.00	0.00
ATOM	2788	HN	ILE	A	173	56.740	33.910	89.340	0.00	0.00
ATOM	2789	CA	ILE	A	173	57.410	33.670	87.390	0.00	0.00
ATOM	2790	HA	ILE	A	173	58.440	33.370	87.240	0.00	0.00
ATOM	2791	CB	ILE	A	173	56.660	32.300	87.560	0.00	0.00
ATOM	2792	HB	ILE	A	173	55.570	32.460	87.660	0.00	0.00
ATOM	2793	CG2	ILE	A	173	56.810	31.440	86.280	0.00	0.00
ATOM	2794	HG21	ILE	A	173	57.820	31.600	85.850	0.00	0.00
ATOM	2795	HG22	ILE	A	173	56.020	31.730	85.550	0.00	0.00
ATOM	2796	HG23	ILE	A	173	56.680	30.340	86.390	0.00	0.00
ATOM	2797	CG1	ILE	A	173	57.350	31.460	88.610	0.00	0.00
ATOM	2798	HG11	ILE	A	173	56.630	30.620	88.690	0.00	0.00
ATOM	2799	HG12	ILE	A	173	57.200	31.860	89.640	0.00	0.00
ATOM	2800	CD	ILE	A	173	58.810	31.020	88.460	0.00	0.00
ATOM	2801	HD1	ILE	A	173	59.080	30.380	89.320	0.00	0.00

ATOM	2802	HD2	ILE	A	173	59.500	31.890	88.400	0.00	0.00
ATOM	2803	HD3	ILE	A	173	59.030	30.380	87.580	0.00	0.00
ATOM	2804	C	ILE	A	173	57.070	34.380	86.030	0.00	0.00
ATOM	2805	O	ILE	A	173	57.760	34.440	85.030	0.00	0.00
ATOM	2806	N	PHE	A	174	55.920	35.050	86.100	0.00	0.00
ATOM	2807	HN	PHE	A	174	55.420	35.120	86.960	0.00	0.00
ATOM	2808	CA	PHE	A	174	55.360	35.810	84.980	0.00	0.00
ATOM	2809	HA	PHE	A	174	55.450	35.240	84.060	0.00	0.00
ATOM	2810	CB	PHE	A	174	53.780	36.010	85.120	0.00	0.00
ATOM	2811	HB1	PHE	A	174	53.370	36.570	84.250	0.00	0.00
ATOM	2812	HB2	PHE	A	174	53.610	36.700	85.970	0.00	0.00
ATOM	2813	CG	PHE	A	174	52.850	34.790	85.250	0.00	0.00
ATOM	2814	CD1	PHE	A	174	51.770	34.780	86.170	0.00	0.00
ATOM	2815	HD1	PHE	A	174	51.520	35.680	86.710	0.00	0.00
ATOM	2816	CE1	PHE	A	174	50.920	33.700	86.280	0.00	0.00
ATOM	2817	HE1	PHE	A	174	50.190	33.670	87.080	0.00	0.00
ATOM	2818	CZ	PHE	A	174	51.010	32.670	85.280	0.00	0.00
ATOM	2819	HZ	PHE	A	174	50.280	31.870	85.300	0.00	0.00
ATOM	2820	CD2	PHE	A	174	52.940	33.730	84.340	0.00	0.00
ATOM	2821	HD2	PHE	A	174	53.780	33.800	83.670	0.00	0.00
ATOM	2822	CE2	PHE	A	174	52.050	32.630	84.310	0.00	0.00
ATOM	2823	HE2	PHE	A	174	52.080	31.770	83.650	0.00	0.00
ATOM	2824	C	PHE	A	174	56.010	37.200	84.670	0.00	0.00
ATOM	2825	O	PHE	A	174	55.980	37.590	83.500	0.00	0.00
ATOM	2826	N	ALA	A	175	56.520	38.010	85.620	0.00	0.00
ATOM	2827	HN	ALA	A	175	56.400	37.790	86.590	0.00	0.00
ATOM	2828	CA	ALA	A	175	57.000	39.430	85.360	0.00	0.00
ATOM	2829	HA	ALA	A	175	56.220	40.010	84.890	0.00	0.00
ATOM	2830	CB	ALA	A	175	57.410	40.080	86.680	0.00	0.00
ATOM	2831	HB1	ALA	A	175	56.610	39.990	87.450	0.00	0.00
ATOM	2832	HB2	ALA	A	175	57.640	41.130	86.380	0.00	0.00
ATOM	2833	HB3	ALA	A	175	58.360	39.700	87.110	0.00	0.00
ATOM	2834	C	ALA	A	175	58.230	39.560	84.490	0.00	0.00
ATOM	2835	O	ALA	A	175	59.250	38.950	84.830	0.00	0.00
ATOM	2836	N	ASN	A	176	58.190	40.320	83.390	0.00	0.00
ATOM	2837	HN	ASN	A	176	57.300	40.740	83.260	0.00	0.00
ATOM	2838	CA	ASN	A	176	59.220	40.390	82.440	0.00	0.00
ATOM	2839	HA	ASN	A	176	60.170	40.200	82.920	0.00	0.00
ATOM	2840	CB	ASN	A	176	59.070	39.350	81.310	0.00	0.00
ATOM	2841	HB1	ASN	A	176	58.910	38.290	81.610	0.00	0.00
ATOM	2842	HB2	ASN	A	176	60.070	39.350	80.810	0.00	0.00
ATOM	2843	CG	ASN	A	176	57.870	39.600	80.250	0.00	0.00
ATOM	2844	OD1	ASN	A	176	58.040	39.100	79.130	0.00	0.00
ATOM	2845	ND2	ASN	A	176	56.780	40.380	80.480	0.00	0.00
ATOM	2846	HD21	ASN	A	176	56.230	40.690	79.710	0.00	0.00
ATOM	2847	HD22	ASN	A	176	56.600	40.940	81.290	0.00	0.00
ATOM	2848	C	ASN	A	176	59.230	41.790	81.770	0.00	0.00
ATOM	2849	O	ASN	A	176	58.260	42.580	81.920	0.00	0.00
ATOM	2850	N	VAL	A	177	60.350	42.260	81.230	0.00	0.00
ATOM	2851	HN	VAL	A	177	61.150	41.670	81.190	0.00	0.00
ATOM	2852	CA	VAL	A	177	60.430	43.590	80.610	0.00	0.00
ATOM	2853	HA	VAL	A	177	59.620	44.130	81.080	0.00	0.00
ATOM	2854	CB	VAL	A	177	61.680	44.430	80.840	0.00	0.00
ATOM	2855	HB	VAL	A	177	62.500	43.870	80.330	0.00	0.00
ATOM	2856	CG1	VAL	A	177	61.640	45.810	80.200	0.00	0.00
ATOM	2857	HG11	VAL	A	177	60.800	46.490	80.430	0.00	0.00
ATOM	2858	HG12	VAL	A	177	61.580	45.900	79.100	0.00	0.00
ATOM	2859	HG13	VAL	A	177	62.620	46.210	80.540	0.00	0.00
ATOM	2860	CG2	VAL	A	177	61.940	44.490	82.330	0.00	0.00
ATOM	2861	HG21	VAL	A	177	61.150	44.980	82.940	0.00	0.00
ATOM	2862	HG22	VAL	A	177	62.880	45.000	82.630	0.00	0.00

```
ATOM   2863 HG23 VAL A 177      62.020  43.450  82.700  0.00  0.00
ATOM   2864  C   VAL A 177     60.100  43.460  79.170  0.00  0.00
ATOM   2865  O   VAL A 177     60.670  42.640  78.460  0.00  0.00
ATOM   2866  N   SER A 178     59.170  44.260  78.660  0.00  0.00
ATOM   2867  HN  SER A 178     58.780  45.030  79.180  0.00  0.00
ATOM   2868  CA  SER A 178     58.580  44.030  77.350  0.00  0.00
ATOM   2869  HA  SER A 178     59.140  43.360  76.730  0.00  0.00
ATOM   2870  CB  SER A 178     57.140  43.490  77.570  0.00  0.00
ATOM   2871  HB1 SER A 178     56.510  44.350  77.890  0.00  0.00
ATOM   2872  HB2 SER A 178     57.160  42.700  78.350  0.00  0.00
ATOM   2873  OG  SER A 178     56.580  42.830  76.490  0.00  0.00
ATOM   2874  HG1 SER A 178     57.360  42.340  76.210  0.00  0.00
ATOM   2875  C   SER A 178     58.480  45.420  76.700  0.00  0.00
ATOM   2876  O   SER A 178     58.310  46.470  77.370  0.00  0.00
ATOM   2877  N   GLU A 179     58.420  45.430  75.360  0.00  0.00
ATOM   2878  HN  GLU A 179     58.400  44.570  74.860  0.00  0.00
ATOM   2879  CA  GLU A 179     58.330  46.620  74.570  0.00  0.00
ATOM   2880  HA  GLU A 179     59.090  47.300  74.910  0.00  0.00
ATOM   2881  CB  GLU A 179     58.530  46.340  73.050  0.00  0.00
ATOM   2882  HB1 GLU A 179     58.350  47.210  72.380  0.00  0.00
ATOM   2883  HB2 GLU A 179     57.850  45.490  72.810  0.00  0.00
ATOM   2884  CG  GLU A 179     60.050  46.080  72.770  0.00  0.00
ATOM   2885  HG1 GLU A 179     60.530  45.360  73.470  0.00  0.00
ATOM   2886  HG2 GLU A 179     60.550  47.070  72.850  0.00  0.00
ATOM   2887  CD  GLU A 179     60.430  45.510  71.400  0.00  0.00
ATOM   2888  OE1 GLU A 179     61.250  44.550  71.360  0.00  0.00
ATOM   2889  OE2 GLU A 179     59.980  46.010  70.340  0.00  0.00
ATOM   2890  C   GLU A 179     57.030  47.370  74.690  0.00  0.00
ATOM   2891  O   GLU A 179     55.930  46.890  74.590  0.00  0.00
ATOM   2892  N   ALA A 180     57.230  48.710  74.490  0.00  0.00
ATOM   2893  HN  ALA A 180     58.130  49.130  74.470  0.00  0.00
ATOM   2894  CA  ALA A 180     56.270  49.670  73.900  0.00  0.00
ATOM   2895  HA  ALA A 180     55.410  49.100  73.560  0.00  0.00
ATOM   2896  CB  ALA A 180     55.740  50.700  74.980  0.00  0.00
ATOM   2897  HB1 ALA A 180     55.180  49.950  75.570  0.00  0.00
ATOM   2898  HB2 ALA A 180     55.000  51.380  74.520  0.00  0.00
ATOM   2899  HB3 ALA A 180     56.480  51.370  75.480  0.00  0.00
ATOM   2900  C   ALA A 180     56.990  50.310  72.750  0.00  0.00
ATOM   2901  O   ALA A 180     58.160  49.920  72.480  0.00  0.00
ATOM   2902  N   ASP A 181     56.390  51.090  71.840  0.00  0.00
ATOM   2903  HN  ASP A 181     55.450  51.410  71.930  0.00  0.00
ATOM   2904  CA  ASP A 181     57.050  51.590  70.700  0.00  0.00
ATOM   2905  HA  ASP A 181     57.410  50.740  70.140  0.00  0.00
ATOM   2906  CB  ASP A 181     56.080  52.500  70.000  0.00  0.00
ATOM   2907  HB1 ASP A 181     56.140  53.560  70.320  0.00  0.00
ATOM   2908  HB2 ASP A 181     55.020  52.330  70.300  0.00  0.00
ATOM   2909  CG  ASP A 181     56.110  52.410  68.530  0.00  0.00
ATOM   2910  OD1 ASP A 181     56.190  51.300  67.980  0.00  0.00
ATOM   2911  OD2 ASP A 181     55.910  53.550  67.970  0.00  0.00
ATOM   2912  C   ASP A 181     58.270  52.470  71.060  0.00  0.00
ATOM   2913  O   ASP A 181     59.330  52.270  70.470  0.00  0.00
ATOM   2914  N   ASP A 182     58.150  53.340  72.070  0.00  0.00
ATOM   2915  HN  ASP A 182     57.290  53.430  72.560  0.00  0.00
ATOM   2916  CA  ASP A 182     59.220  54.300  72.320  0.00  0.00
ATOM   2917  HA  ASP A 182     60.060  54.120  71.650  0.00  0.00
ATOM   2918  CB  ASP A 182     58.720  55.740  72.010  0.00  0.00
ATOM   2919  HB1 ASP A 182     58.390  55.820  70.960  0.00  0.00
ATOM   2920  HB2 ASP A 182     59.580  56.430  72.130  0.00  0.00
ATOM   2921  CG  ASP A 182     57.480  56.200  72.780  0.00  0.00
ATOM   2922  OD1 ASP A 182     57.150  55.580  73.790  0.00  0.00
ATOM   2923  OD2 ASP A 182     56.870  57.200  72.360  0.00  0.00
```

ATOM	2924	C	ASP	A	182	59.860	54.120	73.690	0.00	0.00
ATOM	2925	O	ASP	A	182	60.840	54.830	73.970	0.00	0.00
ATOM	2926	N	ARG	A	183	59.440	53.160	74.470	0.00	0.00
ATOM	2927	HN	ARG	A	183	58.860	52.430	74.120	0.00	0.00
ATOM	2928	CA	ARG	A	183	60.000	52.930	75.820	0.00	0.00
ATOM	2929	HA	ARG	A	183	61.070	53.110	75.840	0.00	0.00
ATOM	2930	CB	ARG	A	183	59.380	53.780	76.850	0.00	0.00
ATOM	2931	HB1	ARG	A	183	59.530	54.850	76.610	0.00	0.00
ATOM	2932	HB2	ARG	A	183	59.910	53.550	77.800	0.00	0.00
ATOM	2933	CG	ARG	A	183	57.840	53.570	77.150	0.00	0.00
ATOM	2934	HG1	ARG	A	183	57.450	52.530	77.000	0.00	0.00
ATOM	2935	HG2	ARG	A	183	57.300	54.120	76.350	0.00	0.00
ATOM	2936	CD	ARG	A	183	57.260	54.150	78.450	0.00	0.00
ATOM	2937	HD1	ARG	A	183	56.160	54.110	78.590	0.00	0.00
ATOM	2938	HD2	ARG	A	183	57.570	55.210	78.560	0.00	0.00
ATOM	2939	NE	ARG	A	183	57.920	53.400	79.560	0.00	0.00
ATOM	2940	HE	ARG	A	183	58.470	52.610	79.310	0.00	0.00
ATOM	2941	CZ	ARG	A	183	57.740	53.420	80.870	0.00	0.00
ATOM	2942	NH1	ARG	A	183	57.080	54.440	81.430	0.00	0.00
ATOM	2943	HH11	ARG	A	183	57.320	54.700	82.360	0.00	0.00
ATOM	2944	HH12	ARG	A	183	56.840	55.180	80.800	0.00	0.00
ATOM	2945	NH2	ARG	A	183	58.320	52.490	81.590	0.00	0.00
ATOM	2946	HH21	ARG	A	183	58.530	51.610	81.170	0.00	0.00
ATOM	2947	HH22	ARG	A	183	58.120	52.580	82.570	0.00	0.00
ATOM	2948	C	ARG	A	183	59.890	51.420	76.170	0.00	0.00
ATOM	2949	O	ARG	A	183	59.550	50.630	75.300	0.00	0.00
ATOM	2950	N	TYR	A	184	60.280	51.020	77.400	0.00	0.00
ATOM	2951	HN	TYR	A	184	60.520	51.740	78.040	0.00	0.00
ATOM	2952	CA	TYR	A	184	60.270	49.680	77.900	0.00	0.00
ATOM	2953	HA	TYR	A	184	59.830	48.960	77.230	0.00	0.00
ATOM	2954	CB	TYR	A	184	61.670	49.190	78.350	0.00	0.00
ATOM	2955	HB1	TYR	A	184	61.690	48.250	78.950	0.00	0.00
ATOM	2956	HB2	TYR	A	184	62.240	49.940	78.940	0.00	0.00
ATOM	2957	CG	TYR	A	184	62.410	48.950	77.080	0.00	0.00
ATOM	2958	CD1	TYR	A	184	62.370	47.590	76.600	0.00	0.00
ATOM	2959	HD1	TYR	A	184	61.730	46.830	77.020	0.00	0.00
ATOM	2960	CE1	TYR	A	184	62.960	47.300	75.370	0.00	0.00
ATOM	2961	HE1	TYR	A	184	62.870	46.350	74.860	0.00	0.00
ATOM	2962	CZ	TYR	A	184	63.620	48.270	74.610	0.00	0.00
ATOM	2963	OH	TYR	A	184	64.250	47.920	73.430	0.00	0.00
ATOM	2964	HH	TYR	A	184	64.830	48.650	73.190	0.00	0.00
ATOM	2965	CD2	TYR	A	184	63.210	49.840	76.440	0.00	0.00
ATOM	2966	HD2	TYR	A	184	63.420	50.800	76.880	0.00	0.00
ATOM	2967	CE2	TYR	A	184	63.720	49.540	75.180	0.00	0.00
ATOM	2968	HE2	TYR	A	184	64.360	50.310	74.770	0.00	0.00
ATOM	2969	C	TYR	A	184	59.330	49.700	79.070	0.00	0.00
ATOM	2970	O	TYR	A	184	59.210	50.730	79.770	0.00	0.00
ATOM	2971	N	ILE	A	185	58.560	48.630	79.240	0.00	0.00
ATOM	2972	HN	ILE	A	185	58.690	47.900	78.570	0.00	0.00
ATOM	2973	CA	ILE	A	185	57.510	48.510	80.270	0.00	0.00
ATOM	2974	HA	ILE	A	185	57.400	49.450	80.810	0.00	0.00
ATOM	2975	CB	ILE	A	185	56.080	48.260	79.620	0.00	0.00
ATOM	2976	HB	ILE	A	185	56.100	47.270	79.130	0.00	0.00
ATOM	2977	CG2	ILE	A	185	54.960	48.280	80.740	0.00	0.00
ATOM	2978	HG21	ILE	A	185	53.980	48.010	80.300	0.00	0.00
ATOM	2979	HG22	ILE	A	185	54.810	49.240	81.280	0.00	0.00
ATOM	2980	HG23	ILE	A	185	55.090	47.530	81.550	0.00	0.00
ATOM	2981	CG1	ILE	A	185	55.720	49.190	78.400	0.00	0.00
ATOM	2982	HG11	ILE	A	185	54.730	48.800	78.110	0.00	0.00
ATOM	2983	HG12	ILE	A	185	56.550	49.000	77.680	0.00	0.00
ATOM	2984	CD	ILE	A	185	55.570	50.670	78.610	0.00	0.00

ATOM 2985 HD1 ILE A 185 54.980 51.070 77.760 0.00 0.00
ATOM 2986 HD2 ILE A 185 56.580 51.140 78.590 0.00 0.00
ATOM 2987 HD3 ILE A 185 55.070 50.850 79.590 0.00 0.00
ATOM 2988 C ILE A 185 57.770 47.210 81.130 0.00 0.00
ATOM 2989 O ILE A 185 58.320 46.220 80.580 0.00 0.00
ATOM 2990 N CYS A 186 57.680 47.280 82.490 0.00 0.00
ATOM 2991 HN CYS A 186 57.520 48.150 82.960 0.00 0.00
ATOM 2992 CA CYS A 186 57.690 46.200 83.470 0.00 0.00
ATOM 2993 HA CYS A 186 58.350 45.400 83.160 0.00 0.00
ATOM 2994 CB CYS A 186 58.230 46.870 84.760 0.00 0.00
ATOM 2995 HB1 CYS A 186 57.500 47.640 85.080 0.00 0.00
ATOM 2996 HB2 CYS A 186 59.200 47.340 84.510 0.00 0.00
ATOM 2997 SG CYS A 186 58.510 45.650 86.180 0.00 0.00
ATOM 2998 C CYS A 186 56.350 45.570 83.690 0.00 0.00
ATOM 2999 O CYS A 186 55.450 46.190 84.300 0.00 0.00
ATOM 3000 N ASP A 187 56.070 44.360 83.140 0.00 0.00
ATOM 3001 HN ASP A 187 56.800 43.810 82.750 0.00 0.00
ATOM 3002 CA ASP A 187 54.740 43.800 82.970 0.00 0.00
ATOM 3003 HA ASP A 187 54.010 44.140 83.700 0.00 0.00
ATOM 3004 CB ASP A 187 54.180 44.350 81.540 0.00 0.00
ATOM 3005 HB1 ASP A 187 54.680 43.880 80.660 0.00 0.00
ATOM 3006 HB2 ASP A 187 54.400 45.440 81.510 0.00 0.00
ATOM 3007 CG ASP A 187 52.700 44.150 81.520 0.00 0.00
ATOM 3008 OD1 ASP A 187 51.990 43.730 82.470 0.00 0.00
ATOM 3009 OD2 ASP A 187 52.130 44.290 80.360 0.00 0.00
ATOM 3010 C ASP A 187 54.810 42.210 83.090 0.00 0.00
ATOM 3011 O ASP A 187 55.880 41.610 83.060 0.00 0.00
ATOM 3012 N ARG A 188 53.650 41.580 83.340 0.00 0.00
ATOM 3013 HN ARG A 188 52.780 42.020 83.540 0.00 0.00
ATOM 3014 CA ARG A 188 53.570 40.090 83.410 0.00 0.00
ATOM 3015 HA ARG A 188 54.560 39.670 83.470 0.00 0.00
ATOM 3016 CB ARG A 188 52.670 39.700 84.590 0.00 0.00
ATOM 3017 HB1 ARG A 188 52.270 38.660 84.540 0.00 0.00
ATOM 3018 HB2 ARG A 188 51.830 40.420 84.580 0.00 0.00
ATOM 3019 CG ARG A 188 53.420 39.760 85.920 0.00 0.00
ATOM 3020 HG1 ARG A 188 54.070 40.670 85.920 0.00 0.00
ATOM 3021 HG2 ARG A 188 54.120 38.900 85.900 0.00 0.00
ATOM 3022 CD ARG A 188 52.460 39.730 87.190 0.00 0.00
ATOM 3023 HD1 ARG A 188 52.970 39.480 88.150 0.00 0.00
ATOM 3024 HD2 ARG A 188 51.670 38.960 87.070 0.00 0.00
ATOM 3025 NE ARG A 188 51.930 41.090 87.320 0.00 0.00
ATOM 3026 HE ARG A 188 52.250 41.820 86.720 0.00 0.00
ATOM 3027 CZ ARG A 188 50.960 41.480 88.170 0.00 0.00
ATOM 3028 NH1 ARG A 188 50.370 40.690 89.040 0.00 0.00
ATOM 3029 HH11 ARG A 188 50.790 39.790 89.190 0.00 0.00
ATOM 3030 HH12 ARG A 188 49.990 41.100 89.870 0.00 0.00
ATOM 3031 NH2 ARG A 188 50.690 42.790 88.180 0.00 0.00
ATOM 3032 HH21 ARG A 188 51.230 43.280 87.490 0.00 0.00
ATOM 3033 HH22 ARG A 188 50.110 43.150 88.910 0.00 0.00
ATOM 3034 C ARG A 188 53.050 39.540 82.070 0.00 0.00
ATOM 3035 O ARG A 188 52.130 40.110 81.550 0.00 0.00
ATOM 3036 N PHE A 189 53.680 38.500 81.600 0.00 0.00
ATOM 3037 HN PHE A 189 54.400 38.120 82.180 0.00 0.00
ATOM 3038 CA PHE A 189 53.220 37.650 80.500 0.00 0.00
ATOM 3039 HA PHE A 189 52.560 38.260 79.900 0.00 0.00
ATOM 3040 CB PHE A 189 54.430 37.170 79.650 0.00 0.00
ATOM 3041 HB1 PHE A 189 55.030 36.370 80.150 0.00 0.00
ATOM 3042 HB2 PHE A 189 55.220 37.950 79.610 0.00 0.00
ATOM 3043 CG PHE A 189 54.070 36.830 78.240 0.00 0.00
ATOM 3044 CD1 PHE A 189 54.180 37.720 77.150 0.00 0.00
ATOM 3045 HD1 PHE A 189 54.790 38.610 77.270 0.00 0.00

ATOM	3046	CE1	PHE	A	189	53.770	37.440	75.840	0.00	0.00
ATOM	3047	HE1	PHE	A	189	53.990	38.060	74.990	0.00	0.00
ATOM	3048	CZ	PHE	A	189	53.110	36.220	75.670	0.00	0.00
ATOM	3049	HZ	PHE	A	189	52.780	36.000	74.660	0.00	0.00
ATOM	3050	CD2	PHE	A	189	53.490	35.580	77.990	0.00	0.00
ATOM	3051	HD2	PHE	A	189	53.500	34.780	78.720	0.00	0.00
ATOM	3052	CE2	PHE	A	189	53.050	35.240	76.710	0.00	0.00
ATOM	3053	HE2	PHE	A	189	52.510	34.320	76.540	0.00	0.00
ATOM	3054	C	PHE	A	189	52.560	36.470	81.070	0.00	0.00
ATOM	3055	O	PHE	A	189	53.070	35.890	82.000	0.00	0.00
ATOM	3056	N	TYR	A	190	51.390	36.130	80.520	0.00	0.00
ATOM	3057	HN	TYR	A	190	51.090	36.600	79.690	0.00	0.00
ATOM	3058	CA	TYR	A	190	50.530	35.110	81.010	0.00	0.00
ATOM	3059	HA	TYR	A	190	50.890	34.610	81.890	0.00	0.00
ATOM	3060	CB	TYR	A	190	49.180	35.740	81.460	0.00	0.00
ATOM	3061	HB1	TYR	A	190	48.410	34.960	81.620	0.00	0.00
ATOM	3062	HB2	TYR	A	190	48.880	36.430	80.640	0.00	0.00
ATOM	3063	CG	TYR	A	190	49.290	36.610	82.700	0.00	0.00
ATOM	3064	CD1	TYR	A	190	48.890	36.060	83.880	0.00	0.00
ATOM	3065	HD1	TYR	A	190	48.530	35.040	83.890	0.00	0.00
ATOM	3066	CE1	TYR	A	190	48.960	36.820	85.100	0.00	0.00
ATOM	3067	HE1	TYR	A	190	48.840	36.360	86.070	0.00	0.00
ATOM	3068	CZ	TYR	A	190	49.230	38.220	85.000	0.00	0.00
ATOM	3069	OH	TYR	A	190	49.540	38.870	86.210	0.00	0.00
ATOM	3070	HH	TYR	A	190	49.480	38.280	86.960	0.00	0.00
ATOM	3071	CD2	TYR	A	190	49.600	38.020	82.600	0.00	0.00
ATOM	3072	HD2	TYR	A	190	49.880	38.450	81.650	0.00	0.00
ATOM	3073	CE2	TYR	A	190	49.420	38.810	83.740	0.00	0.00
ATOM	3074	HE2	TYR	A	190	49.630	39.870	83.700	0.00	0.00
ATOM	3075	C	TYR	A	190	50.330	34.040	80.000	0.00	0.00
ATOM	3076	O	TYR	A	190	50.260	34.320	78.840	0.00	0.00
ATOM	3077	N	PRO	A	191	50.070	32.740	80.330	0.00	0.00
ATOM	3078	CD	PRO	A	191	50.280	32.210	81.650	0.00	0.00
ATOM	3079	HD1	PRO	A	191	49.760	32.810	82.430	0.00	0.00
ATOM	3080	HD2	PRO	A	191	51.370	32.340	81.830	0.00	0.00
ATOM	3081	CA	PRO	A	191	49.940	31.640	79.380	0.00	0.00
ATOM	3082	HA	PRO	A	191	50.640	31.700	78.570	0.00	0.00
ATOM	3083	CB	PRO	A	191	50.110	30.380	80.170	0.00	0.00
ATOM	3084	HB1	PRO	A	191	51.180	30.090	80.200	0.00	0.00
ATOM	3085	HB2	PRO	A	191	49.620	29.490	79.740	0.00	0.00
ATOM	3086	CG	PRO	A	191	49.810	30.760	81.600	0.00	0.00
ATOM	3087	HG1	PRO	A	191	48.710	30.810	81.730	0.00	0.00
ATOM	3088	HG2	PRO	A	191	50.250	30.070	82.350	0.00	0.00
ATOM	3089	C	PRO	A	191	48.650	31.740	78.560	0.00	0.00
ATOM	3090	O	PRO	A	191	48.560	31.360	77.410	0.00	0.00
ATOM	3091	N	ASN	A	192	47.560	32.260	79.140	0.00	0.00
ATOM	3092	HN	ASN	A	192	47.510	32.460	80.120	0.00	0.00
ATOM	3093	CA	ASN	A	192	46.500	32.630	78.270	0.00	0.00
ATOM	3094	HA	ASN	A	192	46.850	32.920	77.290	0.00	0.00
ATOM	3095	CB	ASN	A	192	45.590	31.380	78.030	0.00	0.00
ATOM	3096	HB1	ASN	A	192	46.140	30.640	77.420	0.00	0.00
ATOM	3097	HB2	ASN	A	192	44.750	31.640	77.350	0.00	0.00
ATOM	3098	CG	ASN	A	192	45.010	30.740	79.350	0.00	0.00
ATOM	3099	OD1	ASN	A	192	44.980	31.330	80.430	0.00	0.00
ATOM	3100	ND2	ASN	A	192	44.480	29.520	79.250	0.00	0.00
ATOM	3101	HD21	ASN	A	192	44.280	29.010	80.080	0.00	0.00
ATOM	3102	HD22	ASN	A	192	44.400	29.070	78.360	0.00	0.00
ATOM	3103	C	ASN	A	192	45.820	33.830	78.930	0.00	0.00
ATOM	3104	O	ASN	A	192	45.940	34.260	80.070	0.00	0.00
ATOM	3105	N	ASP	A	193	44.880	34.420	78.140	0.00	0.00
ATOM	3106	HN	ASP	A	193	44.690	34.150	77.200	0.00	0.00

ATOM	3107	CA	ASP	A	193	44.080	35.570	78.470	0.00	0.00
ATOM	3108	HA	ASP	A	193	44.870	36.230	78.780	0.00	0.00
ATOM	3109	CB	ASP	A	193	43.270	36.310	77.330	0.00	0.00
ATOM	3110	HB1	ASP	A	193	42.720	37.140	77.820	0.00	0.00
ATOM	3111	HB2	ASP	A	193	42.500	35.650	76.880	0.00	0.00
ATOM	3112	CG	ASP	A	193	44.150	36.940	76.320	0.00	0.00
ATOM	3113	OD1	ASP	A	193	45.350	36.710	76.330	0.00	0.00
ATOM	3114	OD2	ASP	A	193	43.630	37.770	75.560	0.00	0.00
ATOM	3115	C	ASP	A	193	43.140	35.280	79.690	0.00	0.00
ATOM	3116	O	ASP	A	193	42.930	36.140	80.570	0.00	0.00
ATOM	3117	N	LEU	A	194	42.630	34.040	79.680	0.00	0.00
ATOM	3118	HN	LEU	A	194	42.800	33.460	78.890	0.00	0.00
ATOM	3119	CA	LEU	A	194	41.800	33.420	80.710	0.00	0.00
ATOM	3120	HA	LEU	A	194	40.930	34.050	80.690	0.00	0.00
ATOM	3121	CB	LEU	A	194	41.500	31.990	80.300	0.00	0.00
ATOM	3122	HB1	LEU	A	194	42.460	31.490	80.040	0.00	0.00
ATOM	3123	HB2	LEU	A	194	40.920	31.890	79.360	0.00	0.00
ATOM	3124	CG	LEU	A	194	40.770	31.060	81.280	0.00	0.00
ATOM	3125	HG	LEU	A	194	41.400	31.020	82.200	0.00	0.00
ATOM	3126	CD1	LEU	A	194	39.400	31.480	81.740	0.00	0.00
ATOM	3127	HD11	LEU	A	194	38.870	30.660	82.280	0.00	0.00
ATOM	3128	HD12	LEU	A	194	38.820	31.600	80.810	0.00	0.00
ATOM	3129	HD13	LEU	A	194	39.420	32.400	82.360	0.00	0.00
ATOM	3130	CD2	LEU	A	194	40.690	29.670	80.790	0.00	0.00
ATOM	3131	HD21	LEU	A	194	39.730	29.470	80.260	0.00	0.00
ATOM	3132	HD22	LEU	A	194	41.000	28.980	81.610	0.00	0.00
ATOM	3133	HD23	LEU	A	194	41.500	29.580	80.030	0.00	0.00
ATOM	3134	C	LEU	A	194	42.450	33.570	82.160	0.00	0.00
ATOM	3135	O	LEU	A	194	41.720	33.940	83.080	0.00	0.00
ATOM	3136	N	TRP	A	195	43.770	33.320	82.210	0.00	0.00
ATOM	3137	HN	TRP	A	195	44.300	33.110	81.390	0.00	0.00
ATOM	3138	CA	TRP	A	195	44.560	33.390	83.480	0.00	0.00
ATOM	3139	HA	TRP	A	195	44.030	32.800	84.210	0.00	0.00
ATOM	3140	CB	TRP	A	195	46.030	33.010	83.390	0.00	0.00
ATOM	3141	HB1	TRP	A	195	46.620	33.820	82.920	0.00	0.00
ATOM	3142	HB2	TRP	A	195	46.020	32.200	82.630	0.00	0.00
ATOM	3143	CG	TRP	A	195	46.680	32.510	84.610	0.00	0.00
ATOM	3144	CD1	TRP	A	195	46.930	33.270	85.680	0.00	0.00
ATOM	3145	HD1	TRP	A	195	46.640	34.300	85.760	0.00	0.00
ATOM	3146	NE1	TRP	A	195	47.450	32.600	86.720	0.00	0.00
ATOM	3147	HE1	TRP	A	195	47.270	32.980	87.590	0.00	0.00
ATOM	3148	CE2	TRP	A	195	47.510	31.290	86.310	0.00	0.00
ATOM	3149	CD2	TRP	A	195	47.030	31.170	84.960	0.00	0.00
ATOM	3150	CE3	TRP	A	195	46.950	29.880	84.390	0.00	0.00
ATOM	3151	HE3	TRP	A	195	46.490	29.670	83.430	0.00	0.00
ATOM	3152	CZ3	TRP	A	195	47.260	28.780	85.190	0.00	0.00
ATOM	3153	HZ3	TRP	A	195	47.190	27.750	84.880	0.00	0.00
ATOM	3154	CZ2	TRP	A	195	47.970	30.250	87.020	0.00	0.00
ATOM	3155	HZ2	TRP	A	195	48.430	30.420	87.980	0.00	0.00
ATOM	3156	CH2	TRP	A	195	47.800	29.010	86.450	0.00	0.00
ATOM	3157	HH2	TRP	A	195	48.140	28.220	87.100	0.00	0.00
ATOM	3158	C	TRP	A	195	44.490	34.830	83.940	0.00	0.00
ATOM	3159	O	TRP	A	195	44.220	35.120	85.130	0.00	0.00
ATOM	3160	N	VAL	A	196	44.570	35.780	83.000	0.00	0.00
ATOM	3161	HN	VAL	A	196	44.720	35.550	82.040	0.00	0.00
ATOM	3162	CA	VAL	A	196	44.420	37.240	83.300	0.00	0.00
ATOM	3163	HA	VAL	A	196	45.020	37.380	84.190	0.00	0.00
ATOM	3164	CB	VAL	A	196	44.840	38.260	82.210	0.00	0.00
ATOM	3165	HB	VAL	A	196	44.220	38.220	81.290	0.00	0.00
ATOM	3166	CG1	VAL	A	196	44.720	39.710	82.610	0.00	0.00
ATOM	3167	HG11	VAL	A	196	45.140	40.310	81.770	0.00	0.00

ATOM	3168	HG12	VAL	A	196	45.470	39.790	83.430	0.00	0.00
ATOM	3169	HG13	VAL	A	196	43.750	40.060	83.020	0.00	0.00
ATOM	3170	CG2	VAL	A	196	46.290	37.970	81.780	0.00	0.00
ATOM	3171	HG21	VAL	A	196	46.970	38.230	82.620	0.00	0.00
ATOM	3172	HG22	VAL	A	196	46.630	38.650	80.970	0.00	0.00
ATOM	3173	HG23	VAL	A	196	46.480	36.900	81.530	0.00	0.00
ATOM	3174	C	VAL	A	196	43.080	37.650	83.900	0.00	0.00
ATOM	3175	O	VAL	A	196	43.000	38.410	84.850	0.00	0.00
ATOM	3176	N	VAL	A	197	41.960	37.070	83.380	0.00	0.00
ATOM	3177	HN	VAL	A	197	42.010	36.440	82.600	0.00	0.00
ATOM	3178	CA	VAL	A	197	40.580	37.180	83.810	0.00	0.00
ATOM	3179	HA	VAL	A	197	40.370	38.230	83.920	0.00	0.00
ATOM	3180	CB	VAL	A	197	39.670	36.610	82.710	0.00	0.00
ATOM	3181	HB	VAL	A	197	40.030	35.620	82.360	0.00	0.00
ATOM	3182	CG1	VAL	A	197	38.250	36.430	83.380	0.00	0.00
ATOM	3183	HG11	VAL	A	197	37.900	37.350	83.900	0.00	0.00
ATOM	3184	HG12	VAL	A	197	38.260	35.500	83.990	0.00	0.00
ATOM	3185	HG13	VAL	A	197	37.570	36.180	82.540	0.00	0.00
ATOM	3186	CG2	VAL	A	197	39.630	37.680	81.640	0.00	0.00
ATOM	3187	HG21	VAL	A	197	39.000	37.420	80.760	0.00	0.00
ATOM	3188	HG22	VAL	A	197	40.630	37.740	81.160	0.00	0.00
ATOM	3189	HG23	VAL	A	197	39.390	38.680	82.050	0.00	0.00
ATOM	3190	C	VAL	A	197	40.350	36.670	85.220	0.00	0.00
ATOM	3191	O	VAL	A	197	39.780	37.330	86.100	0.00	0.00
ATOM	3192	N	VAL	A	198	40.930	35.540	85.560	0.00	0.00
ATOM	3193	HN	VAL	A	198	41.410	35.020	84.860	0.00	0.00
ATOM	3194	CA	VAL	A	198	40.650	34.870	86.790	0.00	0.00
ATOM	3195	HA	VAL	A	198	39.660	35.130	87.130	0.00	0.00
ATOM	3196	CB	VAL	A	198	40.630	33.370	86.610	0.00	0.00
ATOM	3197	HB	VAL	A	198	41.670	33.120	86.310	0.00	0.00
ATOM	3198	CG1	VAL	A	198	40.300	32.610	87.930	0.00	0.00
ATOM	3199	HG11	VAL	A	198	39.240	32.670	88.270	0.00	0.00
ATOM	3200	HG12	VAL	A	198	40.880	32.980	88.800	0.00	0.00
ATOM	3201	HG13	VAL	A	198	40.510	31.530	87.750	0.00	0.00
ATOM	3202	CG2	VAL	A	198	39.480	33.100	85.580	0.00	0.00
ATOM	3203	HG21	VAL	A	198	38.470	33.520	85.800	0.00	0.00
ATOM	3204	HG22	VAL	A	198	39.390	32.000	85.390	0.00	0.00
ATOM	3205	HG23	VAL	A	198	39.760	33.430	84.560	0.00	0.00
ATOM	3206	C	VAL	A	198	41.620	35.400	87.870	0.00	0.00
ATOM	3207	O	VAL	A	198	41.400	35.330	89.080	0.00	0.00
ATOM	3208	N	PHE	A	199	42.760	36.030	87.420	0.00	0.00
ATOM	3209	HN	PHE	A	199	42.950	35.950	86.440	0.00	0.00
ATOM	3210	CA	PHE	A	199	43.600	36.890	88.160	0.00	0.00
ATOM	3211	HA	PHE	A	199	43.860	36.460	89.110	0.00	0.00
ATOM	3212	CB	PHE	A	199	45.020	37.160	87.460	0.00	0.00
ATOM	3213	HB1	PHE	A	199	44.840	37.620	86.470	0.00	0.00
ATOM	3214	HB2	PHE	A	199	45.630	36.240	87.390	0.00	0.00
ATOM	3215	CG	PHE	A	199	45.930	38.130	88.240	0.00	0.00
ATOM	3216	CD1	PHE	A	199	46.410	39.350	87.620	0.00	0.00
ATOM	3217	HD1	PHE	A	199	46.210	39.550	86.580	0.00	0.00
ATOM	3218	CE1	PHE	A	199	47.050	40.240	88.450	0.00	0.00
ATOM	3219	HE1	PHE	A	199	47.530	41.130	88.090	0.00	0.00
ATOM	3220	CZ	PHE	A	199	47.220	39.980	89.870	0.00	0.00
ATOM	3221	HZ	PHE	A	199	47.710	40.740	90.470	0.00	0.00
ATOM	3222	CD2	PHE	A	199	46.210	37.860	89.590	0.00	0.00
ATOM	3223	HD2	PHE	A	199	45.850	36.940	90.020	0.00	0.00
ATOM	3224	CE2	PHE	A	199	46.820	38.820	90.450	0.00	0.00
ATOM	3225	HE2	PHE	A	199	47.060	38.580	91.480	0.00	0.00
ATOM	3226	C	PHE	A	199	42.880	38.170	88.600	0.00	0.00
ATOM	3227	O	PHE	A	199	42.900	38.580	89.780	0.00	0.00
ATOM	3228	N	GLN	A	200	42.170	38.880	87.680	0.00	0.00

ATOM	3229	HN	GLN	A	200	42.240	38.610	86.720	0.00	0.00
ATOM	3230	CA	GLN	A	200	41.510	40.060	88.120	0.00	0.00
ATOM	3231	HA	GLN	A	200	42.170	40.710	88.680	0.00	0.00
ATOM	3232	CB	GLN	A	200	40.780	40.830	86.960	0.00	0.00
ATOM	3233	HB1	GLN	A	200	40.270	41.770	87.280	0.00	0.00
ATOM	3234	HB2	GLN	A	200	40.030	40.180	86.470	0.00	0.00
ATOM	3235	CG	GLN	A	200	41.710	41.430	85.860	0.00	0.00
ATOM	3236	HG1	GLN	A	200	41.170	42.170	85.230	0.00	0.00
ATOM	3237	HG2	GLN	A	200	42.070	40.610	85.200	0.00	0.00
ATOM	3238	CD	GLN	A	200	42.930	42.200	86.350	0.00	0.00
ATOM	3239	OE1	GLN	A	200	42.800	43.260	86.940	0.00	0.00
ATOM	3240	NE2	GLN	A	200	44.150	41.810	85.920	0.00	0.00
ATOM	3241	HE21	GLN	A	200	44.910	42.400	86.180	0.00	0.00
ATOM	3242	HE22	GLN	A	200	44.260	41.080	85.250	0.00	0.00
ATOM	3243	C	GLN	A	200	40.470	39.810	89.200	0.00	0.00
ATOM	3244	O	GLN	A	200	40.420	40.580	90.160	0.00	0.00
ATOM	3245	N	PHE	A	201	39.730	38.690	89.070	0.00	0.00
ATOM	3246	HN	PHE	A	201	39.820	38.030	88.320	0.00	0.00
ATOM	3247	CA	PHE	A	201	38.840	38.240	90.160	0.00	0.00
ATOM	3248	HA	PHE	A	201	38.250	39.130	90.250	0.00	0.00
ATOM	3249	CB	PHE	A	201	38.000	36.950	89.670	0.00	0.00
ATOM	3250	HB1	PHE	A	201	38.820	36.200	89.570	0.00	0.00
ATOM	3251	HB2	PHE	A	201	37.490	37.060	88.680	0.00	0.00
ATOM	3252	CG	PHE	A	201	37.040	36.450	90.670	0.00	0.00
ATOM	3253	CD1	PHE	A	201	36.000	37.330	91.070	0.00	0.00
ATOM	3254	HD1	PHE	A	201	35.820	38.280	90.590	0.00	0.00
ATOM	3255	CE1	PHE	A	201	35.090	36.980	92.090	0.00	0.00
ATOM	3256	HE1	PHE	A	201	34.380	37.720	92.440	0.00	0.00
ATOM	3257	CZ	PHE	A	201	35.170	35.740	92.650	0.00	0.00
ATOM	3258	HZ	PHE	A	201	34.500	35.480	93.460	0.00	0.00
ATOM	3259	CD2	PHE	A	201	37.070	35.200	91.300	0.00	0.00
ATOM	3260	HD2	PHE	A	201	37.780	34.460	90.970	0.00	0.00
ATOM	3261	CE2	PHE	A	201	36.190	34.820	92.320	0.00	0.00
ATOM	3262	HE2	PHE	A	201	36.030	33.840	92.750	0.00	0.00
ATOM	3263	C	PHE	A	201	39.470	37.970	91.500	0.00	0.00
ATOM	3264	O	PHE	A	201	39.190	38.570	92.600	0.00	0.00
ATOM	3265	N	GLN	A	202	40.530	37.160	91.490	0.00	0.00
ATOM	3266	HN	GLN	A	202	40.870	36.780	90.630	0.00	0.00
ATOM	3267	CA	GLN	A	202	41.400	36.840	92.640	0.00	0.00
ATOM	3268	HA	GLN	A	202	40.760	36.380	93.390	0.00	0.00
ATOM	3269	CB	GLN	A	202	42.460	35.790	92.260	0.00	0.00
ATOM	3270	HB1	GLN	A	202	42.970	36.040	91.300	0.00	0.00
ATOM	3271	HB2	GLN	A	202	41.970	34.830	92.000	0.00	0.00
ATOM	3272	CG	GLN	A	202	43.570	35.500	93.370	0.00	0.00
ATOM	3273	HG1	GLN	A	202	43.850	34.440	93.170	0.00	0.00
ATOM	3274	HG2	GLN	A	202	43.110	35.400	94.380	0.00	0.00
ATOM	3275	CD	GLN	A	202	44.810	36.400	93.340	0.00	0.00
ATOM	3276	OE1	GLN	A	202	45.490	36.230	92.320	0.00	0.00
ATOM	3277	NE2	GLN	A	202	45.130	37.300	94.290	0.00	0.00
ATOM	3278	HE21	GLN	A	202	45.970	37.820	94.160	0.00	0.00
ATOM	3279	HE22	GLN	A	202	44.480	37.550	95.010	0.00	0.00
ATOM	3280	C	GLN	A	202	42.060	38.070	93.230	0.00	0.00
ATOM	3281	O	GLN	A	202	42.130	38.260	94.440	0.00	0.00
ATOM	3282	N	HSD	A	203	42.560	38.990	92.380	0.00	0.00
ATOM	3283	HN	HSD	A	203	42.460	38.810	91.410	0.00	0.00
ATOM	3284	CA	HSD	A	203	43.200	40.250	92.830	0.00	0.00
ATOM	3285	HA	HSD	A	203	44.030	39.990	93.480	0.00	0.00
ATOM	3286	CB	HSD	A	203	43.940	40.970	91.720	0.00	0.00
ATOM	3287	HB1	HSD	A	203	43.130	41.130	90.970	0.00	0.00
ATOM	3288	HB2	HSD	A	203	44.780	40.320	91.390	0.00	0.00
ATOM	3289	ND1	HSD	A	203	45.860	42.560	92.670	0.00	0.00

ATOM	3290	HD1	HSD	A	203	46.480	41.960	93.170	0.00	0.00
ATOM	3291	CG	HSD	A	203	44.600	42.340	92.110	0.00	0.00
ATOM	3292	CE1	HSD	A	203	46.160	43.880	92.600	0.00	0.00
ATOM	3293	HE1	HSD	A	203	46.910	44.420	93.170	0.00	0.00
ATOM	3294	NE2	HSD	A	203	45.220	44.530	91.990	0.00	0.00
ATOM	3295	CD2	HSD	A	203	44.200	43.540	91.630	0.00	0.00
ATOM	3296	HD2	HSD	A	203	43.420	43.870	90.970	0.00	0.00
ATOM	3297	C	HSD	A	203	42.250	41.160	93.580	0.00	0.00
ATOM	3298	O	HSD	A	203	42.620	41.650	94.670	0.00	0.00
ATOM	3299	N	ILE	A	204	40.950	41.260	93.230	0.00	0.00
ATOM	3300	HN	ILE	A	204	40.490	40.910	92.410	0.00	0.00
ATOM	3301	CA	ILE	A	204	40.020	42.210	93.850	0.00	0.00
ATOM	3302	HA	ILE	A	204	40.490	43.100	94.270	0.00	0.00
ATOM	3303	CB	ILE	A	204	38.960	42.700	92.880	0.00	0.00
ATOM	3304	HB	ILE	A	204	38.450	41.820	92.420	0.00	0.00
ATOM	3305	CG2	ILE	A	204	37.790	43.460	93.560	0.00	0.00
ATOM	3306	HG21	ILE	A	204	37.170	42.720	94.100	0.00	0.00
ATOM	3307	HG22	ILE	A	204	37.150	43.900	92.760	0.00	0.00
ATOM	3308	HG23	ILE	A	204	38.240	44.210	94.250	0.00	0.00
ATOM	3309	CG1	ILE	A	204	39.600	43.540	91.700	0.00	0.00
ATOM	3310	HG11	ILE	A	204	40.420	42.960	91.230	0.00	0.00
ATOM	3311	HG12	ILE	A	204	39.900	44.510	92.140	0.00	0.00
ATOM	3312	CD	ILE	A	204	38.720	43.760	90.440	0.00	0.00
ATOM	3313	HD1	ILE	A	204	38.500	42.810	89.910	0.00	0.00
ATOM	3314	HD2	ILE	A	204	39.270	44.270	89.620	0.00	0.00
ATOM	3315	HD3	ILE	A	204	37.790	44.340	90.630	0.00	0.00
ATOM	3316	C	ILE	A	204	39.240	41.510	95.010	0.00	0.00
ATOM	3317	O	ILE	A	204	38.950	42.100	96.010	0.00	0.00
ATOM	3318	N	MET	A	205	39.070	40.150	94.910	0.00	0.00
ATOM	3319	HN	MET	A	205	39.300	39.670	94.070	0.00	0.00
ATOM	3320	CA	MET	A	205	38.680	39.390	96.030	0.00	0.00
ATOM	3321	HA	MET	A	205	37.770	39.830	96.410	0.00	0.00
ATOM	3322	CB	MET	A	205	38.370	37.900	95.740	0.00	0.00
ATOM	3323	HB1	MET	A	205	38.400	37.320	96.690	0.00	0.00
ATOM	3324	HB2	MET	A	205	39.140	37.420	95.090	0.00	0.00
ATOM	3325	CG	MET	A	205	37.010	37.620	95.070	0.00	0.00
ATOM	3326	HG1	MET	A	205	36.980	36.630	94.560	0.00	0.00
ATOM	3327	HG2	MET	A	205	36.990	38.450	94.330	0.00	0.00
ATOM	3328	SD	MET	A	205	35.550	37.740	96.100	0.00	0.00
ATOM	3329	CE	MET	A	205	35.660	35.980	96.600	0.00	0.00
ATOM	3330	HE1	MET	A	205	36.640	35.690	97.040	0.00	0.00
ATOM	3331	HE2	MET	A	205	35.690	35.350	95.680	0.00	0.00
ATOM	3332	HE3	MET	A	205	34.770	35.680	97.190	0.00	0.00
ATOM	3333	C	MET	A	205	39.610	39.490	97.270	0.00	0.00
ATOM	3334	O	MET	A	205	39.160	39.810	98.390	0.00	0.00
ATOM	3335	N	VAL	A	206	40.900	39.330	97.050	0.00	0.00
ATOM	3336	HN	VAL	A	206	41.200	39.170	96.120	0.00	0.00
ATOM	3337	CA	VAL	A	206	41.930	39.350	98.050	0.00	0.00
ATOM	3338	HA	VAL	A	206	41.520	38.970	98.970	0.00	0.00
ATOM	3339	CB	VAL	A	206	43.190	38.550	97.590	0.00	0.00
ATOM	3340	HB	VAL	A	206	43.730	38.910	96.680	0.00	0.00
ATOM	3341	CG1	VAL	A	206	44.170	38.460	98.740	0.00	0.00
ATOM	3342	HG11	VAL	A	206	44.990	37.760	98.480	0.00	0.00
ATOM	3343	HG12	VAL	A	206	43.690	38.080	99.660	0.00	0.00
ATOM	3344	HG13	VAL	A	206	44.630	39.480	98.820	0.00	0.00
ATOM	3345	CG2	VAL	A	206	42.740	37.160	97.300	0.00	0.00
ATOM	3346	HG21	VAL	A	206	41.990	37.150	96.480	0.00	0.00
ATOM	3347	HG22	VAL	A	206	42.260	36.580	98.120	0.00	0.00
ATOM	3348	HG23	VAL	A	206	43.580	36.580	96.860	0.00	0.00
ATOM	3349	C	VAL	A	206	42.360	40.860	98.400	0.00	0.00
ATOM	3350	O	VAL	A	206	42.690	41.180	99.550	0.00	0.00

ATOM 3351 N GLY A 207 42.300 41.760 97.380 0.00 0.00
ATOM 3352 HN GLY A 207 42.210 41.420 96.450 0.00 0.00
ATOM 3353 CA GLY A 207 42.750 43.150 97.440 0.00 0.00
ATOM 3354 HA1 GLY A 207 42.720 43.480 96.410 0.00 0.00
ATOM 3355 HA2 GLY A 207 43.670 43.280 98.000 0.00 0.00
ATOM 3356 C GLY A 207 41.750 43.920 98.230 0.00 0.00
ATOM 3357 O GLY A 207 42.080 44.890 98.910 0.00 0.00
ATOM 3358 N LEU A 208 40.530 43.510 98.120 0.00 0.00
ATOM 3359 HN LEU A 208 40.290 42.650 97.660 0.00 0.00
ATOM 3360 CA LEU A 208 39.430 44.160 98.780 0.00 0.00
ATOM 3361 HA LEU A 208 39.830 44.880 99.480 0.00 0.00
ATOM 3362 CB LEU A 208 38.570 45.020 97.880 0.00 0.00
ATOM 3363 HB1 LEU A 208 38.220 44.430 97.010 0.00 0.00
ATOM 3364 HB2 LEU A 208 39.110 45.840 97.360 0.00 0.00
ATOM 3365 CG LEU A 208 37.330 45.740 98.510 0.00 0.00
ATOM 3366 HG LEU A 208 36.920 45.120 99.350 0.00 0.00
ATOM 3367 CD1 LEU A 208 37.750 47.090 99.240 0.00 0.00
ATOM 3368 HD11 LEU A 208 38.060 47.810 98.460 0.00 0.00
ATOM 3369 HD12 LEU A 208 38.520 47.050 100.040 0.00 0.00
ATOM 3370 HD13 LEU A 208 36.860 47.590 99.670 0.00 0.00
ATOM 3371 CD2 LEU A 208 36.320 46.070 97.410 0.00 0.00
ATOM 3372 HD21 LEU A 208 35.360 46.280 97.930 0.00 0.00
ATOM 3373 HD22 LEU A 208 36.170 45.160 96.780 0.00 0.00
ATOM 3374 HD23 LEU A 208 36.630 46.980 96.860 0.00 0.00
ATOM 3375 C LEU A 208 38.480 43.230 99.490 0.00 0.00
ATOM 3376 O LEU A 208 38.470 43.160 100.730 0.00 0.00
ATOM 3377 N ILE A 209 37.500 42.540 98.790 0.00 0.00
ATOM 3378 HN ILE A 209 37.480 42.600 97.790 0.00 0.00
ATOM 3379 CA ILE A 209 36.300 41.870 99.360 0.00 0.00
ATOM 3380 HA ILE A 209 35.670 42.660 99.730 0.00 0.00
ATOM 3381 CB ILE A 209 35.430 41.190 98.300 0.00 0.00
ATOM 3382 HB ILE A 209 35.950 40.280 97.930 0.00 0.00
ATOM 3383 CG2 ILE A 209 34.160 40.650 98.910 0.00 0.00
ATOM 3384 HG21 ILE A 209 34.140 39.860 99.690 0.00 0.00
ATOM 3385 HG22 ILE A 209 33.560 40.250 98.070 0.00 0.00
ATOM 3386 HG23 ILE A 209 33.620 41.510 99.350 0.00 0.00
ATOM 3387 CG1 ILE A 209 35.000 42.170 97.150 0.00 0.00
ATOM 3388 HG11 ILE A 209 35.680 43.050 97.110 0.00 0.00
ATOM 3389 HG12 ILE A 209 33.950 42.490 97.340 0.00 0.00
ATOM 3390 CD ILE A 209 35.000 41.520 95.770 0.00 0.00
ATOM 3391 HD1 ILE A 209 34.300 40.660 95.830 0.00 0.00
ATOM 3392 HD2 ILE A 209 36.030 41.310 95.430 0.00 0.00
ATOM 3393 HD3 ILE A 209 34.470 42.230 95.080 0.00 0.00
ATOM 3394 C ILE A 209 36.580 41.070 100.590 0.00 0.00
ATOM 3395 O ILE A 209 36.080 41.350 101.680 0.00 0.00
ATOM 3396 N LEU A 210 37.420 40.030 100.470 0.00 0.00
ATOM 3397 HN LEU A 210 38.000 39.790 99.700 0.00 0.00
ATOM 3398 CA LEU A 210 37.780 39.030 101.590 0.00 0.00
ATOM 3399 HA LEU A 210 36.840 38.600 101.910 0.00 0.00
ATOM 3400 CB LEU A 210 38.610 37.780 101.160 0.00 0.00
ATOM 3401 HB1 LEU A 210 38.710 37.100 102.030 0.00 0.00
ATOM 3402 HB2 LEU A 210 39.600 38.170 100.840 0.00 0.00
ATOM 3403 CG LEU A 210 38.090 36.960 99.960 0.00 0.00
ATOM 3404 HG LEU A 210 38.050 37.690 99.130 0.00 0.00
ATOM 3405 CD1 LEU A 210 39.200 35.980 99.530 0.00 0.00
ATOM 3406 HD11 LEU A 210 40.160 36.460 99.250 0.00 0.00
ATOM 3407 HD12 LEU A 210 38.860 35.520 98.580 0.00 0.00
ATOM 3408 HD13 LEU A 210 39.290 35.210 100.320 0.00 0.00
ATOM 3409 CD2 LEU A 210 36.750 36.210 100.240 0.00 0.00
ATOM 3410 HD21 LEU A 210 35.800 36.790 100.300 0.00 0.00
ATOM 3411 HD22 LEU A 210 36.920 35.660 101.190 0.00 0.00

ATOM 3412 HD23 LEU A 210 36.700 35.520 99.370 0.00 0.00
ATOM 3413 C LEU A 210 38.360 39.570 102.920 0.00 0.00
ATOM 3414 O LEU A 210 37.870 39.220 104.010 0.00 0.00
ATOM 3415 N PRO A 211 39.320 40.520 103.080 0.00 0.00
ATOM 3416 CD PRO A 211 40.440 40.820 102.100 0.00 0.00
ATOM 3417 HD1 PRO A 211 40.050 41.060 101.090 0.00 0.00
ATOM 3418 HD2 PRO A 211 41.150 39.980 101.950 0.00 0.00
ATOM 3419 CA PRO A 211 39.570 41.120 104.400 0.00 0.00
ATOM 3420 HA PRO A 211 39.610 40.390 105.200 0.00 0.00
ATOM 3421 CB PRO A 211 40.890 41.850 104.310 0.00 0.00
ATOM 3422 HB1 PRO A 211 41.720 41.400 104.900 0.00 0.00
ATOM 3423 HB2 PRO A 211 40.990 42.880 104.690 0.00 0.00
ATOM 3424 CG PRO A 211 41.220 41.980 102.750 0.00 0.00
ATOM 3425 HG1 PRO A 211 40.760 42.900 102.340 0.00 0.00
ATOM 3426 HG2 PRO A 211 42.320 41.890 102.590 0.00 0.00
ATOM 3427 C PRO A 211 38.390 41.960 104.820 0.00 0.00
ATOM 3428 O PRO A 211 37.980 42.130 105.970 0.00 0.00
ATOM 3429 N GLY A 212 38.010 42.750 103.780 0.00 0.00
ATOM 3430 HN GLY A 212 38.630 42.650 103.000 0.00 0.00
ATOM 3431 CA GLY A 212 37.040 43.890 103.740 0.00 0.00
ATOM 3432 HA1 GLY A 212 36.810 43.930 102.690 0.00 0.00
ATOM 3433 HA2 GLY A 212 37.540 44.800 104.050 0.00 0.00
ATOM 3434 C GLY A 212 35.810 43.690 104.570 0.00 0.00
ATOM 3435 O GLY A 212 35.390 44.620 105.280 0.00 0.00
ATOM 3436 N ILE A 213 35.220 42.470 104.460 0.00 0.00
ATOM 3437 HN ILE A 213 35.520 41.840 103.740 0.00 0.00
ATOM 3438 CA ILE A 213 33.990 42.080 105.200 0.00 0.00
ATOM 3439 HA ILE A 213 33.350 42.950 105.140 0.00 0.00
ATOM 3440 CB ILE A 213 33.180 40.970 104.500 0.00 0.00
ATOM 3441 HB ILE A 213 32.320 40.750 105.160 0.00 0.00
ATOM 3442 CG2 ILE A 213 32.540 41.580 103.190 0.00 0.00
ATOM 3443 HG21 ILE A 213 31.880 40.840 102.700 0.00 0.00
ATOM 3444 HG22 ILE A 213 33.250 42.010 102.440 0.00 0.00
ATOM 3445 HG23 ILE A 213 31.960 42.450 103.550 0.00 0.00
ATOM 3446 CG1 ILE A 213 33.890 39.610 104.360 0.00 0.00
ATOM 3447 HG11 ILE A 213 33.810 39.110 105.350 0.00 0.00
ATOM 3448 HG12 ILE A 213 34.920 39.830 104.000 0.00 0.00
ATOM 3449 CD ILE A 213 33.350 38.600 103.330 0.00 0.00
ATOM 3450 HD1 ILE A 213 33.670 38.750 102.270 0.00 0.00
ATOM 3451 HD2 ILE A 213 32.250 38.520 103.440 0.00 0.00
ATOM 3452 HD3 ILE A 213 33.660 37.580 103.650 0.00 0.00
ATOM 3453 C ILE A 213 34.290 41.680 106.610 0.00 0.00
ATOM 3454 O ILE A 213 33.390 41.770 107.420 0.00 0.00
ATOM 3455 N VAL A 214 35.490 41.240 106.950 0.00 0.00
ATOM 3456 HN VAL A 214 36.200 41.260 106.240 0.00 0.00
ATOM 3457 CA VAL A 214 35.850 41.000 108.300 0.00 0.00
ATOM 3458 HA VAL A 214 35.000 40.560 108.810 0.00 0.00
ATOM 3459 CB VAL A 214 37.010 40.050 108.320 0.00 0.00
ATOM 3460 HB VAL A 214 37.870 40.530 107.810 0.00 0.00
ATOM 3461 CG1 VAL A 214 37.390 39.610 109.770 0.00 0.00
ATOM 3462 HG11 VAL A 214 38.170 38.820 109.790 0.00 0.00
ATOM 3463 HG12 VAL A 214 36.470 39.240 110.270 0.00 0.00
ATOM 3464 HG13 VAL A 214 37.810 40.520 110.240 0.00 0.00
ATOM 3465 CG2 VAL A 214 36.850 38.740 107.490 0.00 0.00
ATOM 3466 HG21 VAL A 214 37.780 38.120 107.510 0.00 0.00
ATOM 3467 HG22 VAL A 214 36.600 38.830 106.410 0.00 0.00
ATOM 3468 HG23 VAL A 214 36.130 38.050 107.980 0.00 0.00
ATOM 3469 C VAL A 214 36.010 42.310 108.940 0.00 0.00
ATOM 3470 O VAL A 214 35.480 42.430 110.040 0.00 0.00
ATOM 3471 N ILE A 215 36.780 43.280 108.390 0.00 0.00
ATOM 3472 HN ILE A 215 37.320 43.130 107.570 0.00 0.00

ATOM	3473	CA	ILE	A	215	37.040	44.580	109.030	0.00	0.00
ATOM	3474	HA	ILE	A	215	37.520	44.360	109.970	0.00	0.00
ATOM	3475	CB	ILE	A	215	37.910	45.380	108.080	0.00	0.00
ATOM	3476	HB	ILE	A	215	37.400	45.490	107.100	0.00	0.00
ATOM	3477	CG2	ILE	A	215	38.120	46.860	108.670	0.00	0.00
ATOM	3478	HG21	ILE	A	215	38.670	47.510	107.960	0.00	0.00
ATOM	3479	HG22	ILE	A	215	38.640	46.960	109.650	0.00	0.00
ATOM	3480	HG23	ILE	A	215	37.110	47.310	108.600	0.00	0.00
ATOM	3481	CG1	ILE	A	215	39.250	44.730	107.800	0.00	0.00
ATOM	3482	HG11	ILE	A	215	39.230	43.620	107.820	0.00	0.00
ATOM	3483	HG12	ILE	A	215	40.040	44.870	108.570	0.00	0.00
ATOM	3484	CD	ILE	A	215	39.770	45.090	106.360	0.00	0.00
ATOM	3485	HD1	ILE	A	215	40.130	46.140	106.300	0.00	0.00
ATOM	3486	HD2	ILE	A	215	38.960	44.980	105.610	0.00	0.00
ATOM	3487	HD3	ILE	A	215	40.690	44.480	106.240	0.00	0.00
ATOM	3488	C	ILE	A	215	35.720	45.220	109.270	0.00	0.00
ATOM	3489	O	ILE	A	215	35.430	45.640	110.360	0.00	0.00
ATOM	3490	N	LEU	A	216	34.750	45.230	108.210	0.00	0.00
ATOM	3491	HN	LEU	A	216	34.970	44.710	107.390	0.00	0.00
ATOM	3492	CA	LEU	A	216	33.420	45.700	108.320	0.00	0.00
ATOM	3493	HA	LEU	A	216	33.450	46.710	108.690	0.00	0.00
ATOM	3494	CB	LEU	A	216	32.680	45.750	106.950	0.00	0.00
ATOM	3495	HB1	LEU	A	216	32.450	44.700	106.660	0.00	0.00
ATOM	3496	HB2	LEU	A	216	33.350	46.030	106.110	0.00	0.00
ATOM	3497	CG	LEU	A	216	31.370	46.580	106.880	0.00	0.00
ATOM	3498	HG	LEU	A	216	30.860	46.450	107.850	0.00	0.00
ATOM	3499	CD1	LEU	A	216	31.650	48.100	106.550	0.00	0.00
ATOM	3500	HD11	LEU	A	216	32.300	48.130	105.650	0.00	0.00
ATOM	3501	HD12	LEU	A	216	32.130	48.520	107.450	0.00	0.00
ATOM	3502	HD13	LEU	A	216	30.710	48.690	106.430	0.00	0.00
ATOM	3503	CD2	LEU	A	216	30.470	45.990	105.750	0.00	0.00
ATOM	3504	HD21	LEU	A	216	31.080	45.990	104.820	0.00	0.00
ATOM	3505	HD22	LEU	A	216	29.590	46.640	105.540	0.00	0.00
ATOM	3506	HD23	LEU	A	216	30.120	44.970	106.010	0.00	0.00
ATOM	3507	C	LEU	A	216	32.580	44.920	109.390	0.00	0.00
ATOM	3508	O	LEU	A	216	31.870	45.500	110.200	0.00	0.00
ATOM	3509	N	SER	A	217	32.690	43.600	109.470	0.00	0.00
ATOM	3510	HN	SER	A	217	33.120	43.140	108.700	0.00	0.00
ATOM	3511	CA	SER	A	217	31.980	42.860	110.520	0.00	0.00
ATOM	3512	HA	SER	A	217	30.950	43.130	110.390	0.00	0.00
ATOM	3513	CB	SER	A	217	32.240	41.260	110.510	0.00	0.00
ATOM	3514	HB1	SER	A	217	31.920	40.780	111.460	0.00	0.00
ATOM	3515	HB2	SER	A	217	33.310	41.020	110.340	0.00	0.00
ATOM	3516	OG	SER	A	217	31.720	40.620	109.350	0.00	0.00
ATOM	3517	HG1	SER	A	217	32.330	40.770	108.620	0.00	0.00
ATOM	3518	C	SER	A	217	32.450	43.200	111.970	0.00	0.00
ATOM	3519	O	SER	A	217	31.630	43.370	112.880	0.00	0.00
ATOM	3520	N	CYS	A	218	33.790	43.380	112.160	0.00	0.00
ATOM	3521	HN	CYS	A	218	34.540	43.180	111.530	0.00	0.00
ATOM	3522	CA	CYS	A	218	34.310	43.930	113.350	0.00	0.00
ATOM	3523	HA	CYS	A	218	34.020	43.320	114.190	0.00	0.00
ATOM	3524	CB	CYS	A	218	35.850	44.090	113.170	0.00	0.00
ATOM	3525	HB1	CYS	A	218	36.270	44.410	114.150	0.00	0.00
ATOM	3526	HB2	CYS	A	218	36.090	44.790	112.350	0.00	0.00
ATOM	3527	SG	CYS	A	218	36.670	42.530	112.830	0.00	0.00
ATOM	3528	HG1	CYS	A	218	36.420	42.330	111.550	0.00	0.00
ATOM	3529	C	CYS	A	218	33.890	45.340	113.660	0.00	0.00
ATOM	3530	O	CYS	A	218	33.450	45.670	114.760	0.00	0.00
ATOM	3531	N	TYR	A	219	34.010	46.260	112.660	0.00	0.00
ATOM	3532	HN	TYR	A	219	34.250	45.930	111.750	0.00	0.00
ATOM	3533	CA	TYR	A	219	33.610	47.650	112.630	0.00	0.00

ATOM	3534	HA	TYR	A	219	34.220	48.210	113.330	0.00	0.00
ATOM	3535	CB	TYR	A	219	33.970	48.360	111.250	0.00	0.00
ATOM	3536	HB1	TYR	A	219	33.450	47.720	110.500	0.00	0.00
ATOM	3537	HB2	TYR	A	219	35.030	48.320	110.930	0.00	0.00
ATOM	3538	CG	TYR	A	219	33.550	49.750	110.990	0.00	0.00
ATOM	3539	CD1	TYR	A	219	34.250	50.740	111.550	0.00	0.00
ATOM	3540	HD1	TYR	A	219	35.100	50.440	112.150	0.00	0.00
ATOM	3541	CE1	TYR	A	219	33.870	52.110	111.330	0.00	0.00
ATOM	3542	HE1	TYR	A	219	34.510	52.860	111.780	0.00	0.00
ATOM	3543	CZ	TYR	A	219	32.880	52.350	110.370	0.00	0.00
ATOM	3544	OH	TYR	A	219	32.480	53.670	110.090	0.00	0.00
ATOM	3545	HH	TYR	A	219	31.550	53.560	109.920	0.00	0.00
ATOM	3546	CD2	TYR	A	219	32.470	50.060	110.120	0.00	0.00
ATOM	3547	HD2	TYR	A	219	31.920	49.200	109.760	0.00	0.00
ATOM	3548	CE2	TYR	A	219	32.170	51.410	109.770	0.00	0.00
ATOM	3549	HE2	TYR	A	219	31.450	51.610	108.990	0.00	0.00
ATOM	3550	C	TYR	A	219	32.200	47.900	113.130	0.00	0.00
ATOM	3551	O	TYR	A	219	31.910	48.810	113.930	0.00	0.00
ATOM	3552	N	CYS	A	220	31.290	47.000	112.710	0.00	0.00
ATOM	3553	HN	CYS	A	220	31.390	46.290	112.020	0.00	0.00
ATOM	3554	CA	CYS	A	220	29.950	47.020	113.210	0.00	0.00
ATOM	3555	HA	CYS	A	220	29.510	47.980	112.990	0.00	0.00
ATOM	3556	CB	CYS	A	220	29.240	45.800	112.530	0.00	0.00
ATOM	3557	HB1	CYS	A	220	28.310	45.560	113.070	0.00	0.00
ATOM	3558	HB2	CYS	A	220	29.950	44.950	112.580	0.00	0.00
ATOM	3559	SG	CYS	A	220	28.970	46.190	110.750	0.00	0.00
ATOM	3560	HG1	CYS	A	220	30.240	46.260	110.410	0.00	0.00
ATOM	3561	C	CYS	A	220	29.820	46.880	114.720	0.00	0.00
ATOM	3562	O	CYS	A	220	29.150	47.710	115.340	0.00	0.00
ATOM	3563	N	ILE	A	221	30.460	45.860	115.310	0.00	0.00
ATOM	3564	HN	ILE	A	221	30.950	45.200	114.740	0.00	0.00
ATOM	3565	CA	ILE	A	221	30.640	45.700	116.730	0.00	0.00
ATOM	3566	HA	ILE	A	221	29.630	45.660	117.140	0.00	0.00
ATOM	3567	CB	ILE	A	221	31.320	44.370	116.980	0.00	0.00
ATOM	3568	HB	ILE	A	221	32.320	44.290	116.510	0.00	0.00
ATOM	3569	CG2	ILE	A	221	31.570	44.190	118.550	0.00	0.00
ATOM	3570	HG21	ILE	A	221	30.590	44.350	119.060	0.00	0.00
ATOM	3571	HG22	ILE	A	221	32.380	44.880	118.860	0.00	0.00
ATOM	3572	HG23	ILE	A	221	32.050	43.200	118.670	0.00	0.00
ATOM	3573	CG1	ILE	A	221	30.470	43.130	116.490	0.00	0.00
ATOM	3574	HG11	ILE	A	221	31.180	42.280	116.600	0.00	0.00
ATOM	3575	HG12	ILE	A	221	30.290	43.250	115.400	0.00	0.00
ATOM	3576	CD	ILE	A	221	29.110	42.930	117.290	0.00	0.00
ATOM	3577	HD1	ILE	A	221	29.210	42.410	118.270	0.00	0.00
ATOM	3578	HD2	ILE	A	221	28.480	42.440	116.520	0.00	0.00
ATOM	3579	HD3	ILE	A	221	28.550	43.880	117.420	0.00	0.00
ATOM	3580	C	ILE	A	221	31.380	46.890	117.420	0.00	0.00
ATOM	3581	O	ILE	A	221	30.960	47.460	118.490	0.00	0.00
ATOM	3582	N	ILE	A	222	32.410	47.460	116.820	0.00	0.00
ATOM	3583	HN	ILE	A	222	32.870	46.990	116.080	0.00	0.00
ATOM	3584	CA	ILE	A	222	33.180	48.470	117.480	0.00	0.00
ATOM	3585	HA	ILE	A	222	33.240	48.270	118.540	0.00	0.00
ATOM	3586	CB	ILE	A	222	34.670	48.440	117.070	0.00	0.00
ATOM	3587	HB	ILE	A	222	34.650	48.710	115.990	0.00	0.00
ATOM	3588	CG2	ILE	A	222	35.490	49.500	117.920	0.00	0.00
ATOM	3589	HG21	ILE	A	222	36.520	49.600	117.500	0.00	0.00
ATOM	3590	HG22	ILE	A	222	35.490	49.080	118.950	0.00	0.00
ATOM	3591	HG23	ILE	A	222	34.990	50.480	117.790	0.00	0.00
ATOM	3592	CG1	ILE	A	222	35.310	47.020	117.160	0.00	0.00
ATOM	3593	HG11	ILE	A	222	34.600	46.300	116.720	0.00	0.00
ATOM	3594	HG12	ILE	A	222	35.390	46.660	118.200	0.00	0.00

ATOM 3595 CD ILE A 222 36.600 46.800 116.450 0.00 0.00
ATOM 3596 HD1 ILE A 222 36.710 47.420 115.530 0.00 0.00
ATOM 3597 HD2 ILE A 222 36.750 45.750 116.100 0.00 0.00
ATOM 3598 HD3 ILE A 222 37.500 46.960 117.090 0.00 0.00
ATOM 3599 C ILE A 222 32.480 49.790 117.430 0.00 0.00
ATOM 3600 O ILE A 222 32.610 50.550 118.390 0.00 0.00
ATOM 3601 N ILE A 223 31.620 50.100 116.450 0.00 0.00
ATOM 3602 HN ILE A 223 31.570 49.560 115.610 0.00 0.00
ATOM 3603 CA ILE A 223 30.750 51.270 116.600 0.00 0.00
ATOM 3604 HA ILE A 223 31.270 52.070 117.100 0.00 0.00
ATOM 3605 CB ILE A 223 30.200 51.630 115.240 0.00 0.00
ATOM 3606 HB ILE A 223 29.760 50.760 114.700 0.00 0.00
ATOM 3607 CG2 ILE A 223 29.060 52.720 115.470 0.00 0.00
ATOM 3608 HG21 ILE A 223 28.070 52.370 115.830 0.00 0.00
ATOM 3609 HG22 ILE A 223 28.950 53.270 114.520 0.00 0.00
ATOM 3610 HG23 ILE A 223 29.450 53.520 116.140 0.00 0.00
ATOM 3611 CG1 ILE A 223 31.290 52.330 114.360 0.00 0.00
ATOM 3612 HG11 ILE A 223 30.720 52.700 113.480 0.00 0.00
ATOM 3613 HG12 ILE A 223 32.070 51.600 114.050 0.00 0.00
ATOM 3614 CD ILE A 223 31.850 53.580 114.980 0.00 0.00
ATOM 3615 HD1 ILE A 223 32.280 53.430 116.000 0.00 0.00
ATOM 3616 HD2 ILE A 223 31.120 54.410 115.040 0.00 0.00
ATOM 3617 HD3 ILE A 223 32.670 54.020 114.370 0.00 0.00
ATOM 3618 C ILE A 223 29.610 51.010 117.580 0.00 0.00
ATOM 3619 O ILE A 223 29.200 51.880 118.340 0.00 0.00
ATOM 3620 N SER A 224 29.130 49.760 117.650 0.00 0.00
ATOM 3621 HN SER A 224 29.530 49.100 117.010 0.00 0.00
ATOM 3622 CA SER A 224 28.070 49.360 118.590 0.00 0.00
ATOM 3623 HA SER A 224 27.180 49.930 118.360 0.00 0.00
ATOM 3624 CB SER A 224 27.700 47.890 118.410 0.00 0.00
ATOM 3625 HB1 SER A 224 28.540 47.280 118.800 0.00 0.00
ATOM 3626 HB2 SER A 224 27.490 47.660 117.350 0.00 0.00
ATOM 3627 OG SER A 224 26.630 47.510 119.180 0.00 0.00
ATOM 3628 HG1 SER A 224 26.720 46.570 119.360 0.00 0.00
ATOM 3629 C SER A 224 28.450 49.580 120.040 0.00 0.00
ATOM 3630 O SER A 224 27.580 49.910 120.850 0.00 0.00
ATOM 3631 N LYS A 225 29.730 49.390 120.340 0.00 0.00
ATOM 3632 HN LYS A 225 30.380 49.030 119.680 0.00 0.00
ATOM 3633 CA LYS A 225 30.230 49.450 121.720 0.00 0.00
ATOM 3634 HA LYS A 225 29.450 49.740 122.410 0.00 0.00
ATOM 3635 CB LYS A 225 30.760 48.120 122.290 0.00 0.00
ATOM 3636 HB1 LYS A 225 31.030 48.240 123.360 0.00 0.00
ATOM 3637 HB2 LYS A 225 31.620 47.830 121.650 0.00 0.00
ATOM 3638 CG LYS A 225 29.680 47.030 122.250 0.00 0.00
ATOM 3639 HG1 LYS A 225 30.160 46.050 122.480 0.00 0.00
ATOM 3640 HG2 LYS A 225 29.380 46.960 121.180 0.00 0.00
ATOM 3641 CD LYS A 225 28.460 47.320 123.150 0.00 0.00
ATOM 3642 HD1 LYS A 225 28.050 48.300 122.850 0.00 0.00
ATOM 3643 HD2 LYS A 225 28.810 47.350 124.210 0.00 0.00
ATOM 3644 CE LYS A 225 27.300 46.240 123.120 0.00 0.00
ATOM 3645 HE1 LYS A 225 27.640 45.390 123.750 0.00 0.00
ATOM 3646 HE2 LYS A 225 27.110 45.850 122.100 0.00 0.00
ATOM 3647 NZ LYS A 225 26.090 46.840 123.640 0.00 0.00
ATOM 3648 HZ1 LYS A 225 26.410 47.370 124.470 0.00 0.00
ATOM 3649 HZ2 LYS A 225 25.320 46.200 123.930 0.00 0.00
ATOM 3650 HZ3 LYS A 225 25.760 47.530 122.940 0.00 0.00
ATOM 3651 C LYS A 225 31.230 50.600 121.860 0.00 0.00
ATOM 3652 O LYS A 225 32.080 50.590 122.720 0.00 0.00
ATOM 3653 N LEU A 226 31.200 51.550 120.890 0.00 0.00
ATOM 3654 HN LEU A 226 30.480 51.420 120.210 0.00 0.00
ATOM 3655 CA LEU A 226 32.050 52.720 120.850 0.00 0.00

ATOM	3656	HA	LEU	A	226	33.030	52.330	121.080	0.00	0.00
ATOM	3657	CB	LEU	A	226	31.940	53.510	119.510	0.00	0.00
ATOM	3658	HB1	LEU	A	226	30.850	53.620	119.340	0.00	0.00
ATOM	3659	HB2	LEU	A	226	32.430	52.830	118.780	0.00	0.00
ATOM	3660	CG	LEU	A	226	32.650	54.910	119.570	0.00	0.00
ATOM	3661	HG	LEU	A	226	32.160	55.520	120.360	0.00	0.00
ATOM	3662	CD1	LEU	A	226	34.150	54.860	120.020	0.00	0.00
ATOM	3663	HD11	LEU	A	226	34.620	55.870	119.970	0.00	0.00
ATOM	3664	HD12	LEU	A	226	34.740	54.230	119.320	0.00	0.00
ATOM	3665	HD13	LEU	A	226	34.270	54.460	121.050	0.00	0.00
ATOM	3666	CD2	LEU	A	226	32.580	55.660	118.210	0.00	0.00
ATOM	3667	HD21	LEU	A	226	31.560	55.840	117.810	0.00	0.00
ATOM	3668	HD22	LEU	A	226	33.290	55.270	117.450	0.00	0.00
ATOM	3669	HD23	LEU	A	226	32.940	56.710	118.200	0.00	0.00
ATOM	3670	C	LEU	A	226	31.640	53.680	122.080	0.00	0.00
ATOM	3671	O	LEU	A	226	32.480	54.140	122.770	0.00	0.00
ATOM	3672	N	SER	A	227	30.300	53.990	122.230	0.00	0.00
ATOM	3673	HN	SER	A	227	29.590	53.550	121.690	0.00	0.00
ATOM	3674	CA	SER	A	227	29.710	54.820	123.270	0.00	0.00
ATOM	3675	HA	SER	A	227	28.660	54.590	123.150	0.00	0.00
ATOM	3676	CB	SER	A	227	30.160	54.480	124.700	0.00	0.00
ATOM	3677	HB1	SER	A	227	30.190	53.380	124.850	0.00	0.00
ATOM	3678	HB2	SER	A	227	29.450	54.850	125.470	0.00	0.00
ATOM	3679	OG	SER	A	227	31.390	55.120	125.010	0.00	0.00
ATOM	3680	HG1	SER	A	227	32.120	54.640	124.600	0.00	0.00
ATOM	3681	C	SER	A	227	29.560	56.340	122.920	0.00	0.00
ATOM	3682	O	SER	A	227	30.240	56.820	122.010	0.00	0.00
ATOM	3683	N	HSD	A	228	28.730	56.990	123.720	0.00	0.00
ATOM	3684	HN	HSD	A	228	28.160	56.580	124.430	0.00	0.00
ATOM	3685	CA	HSD	A	228	28.700	58.460	123.660	0.00	0.00
ATOM	3686	HA	HSD	A	228	28.670	58.740	122.620	0.00	0.00
ATOM	3687	CB	HSD	A	228	27.420	59.100	124.260	0.00	0.00
ATOM	3688	HB1	HSD	A	228	27.440	60.180	124.020	0.00	0.00
ATOM	3689	HB2	HSD	A	228	27.220	58.870	125.330	0.00	0.00
ATOM	3690	ND1	HSD	A	228	25.690	57.330	123.660	0.00	0.00
ATOM	3691	HD1	HSD	A	228	26.030	56.700	124.350	0.00	0.00
ATOM	3692	CG	HSD	A	228	26.210	58.600	123.440	0.00	0.00
ATOM	3693	CE1	HSD	A	228	24.520	57.200	123.010	0.00	0.00
ATOM	3694	HE1	HSD	A	228	23.830	56.350	122.980	0.00	0.00
ATOM	3695	NE2	HSD	A	228	24.190	58.350	122.430	0.00	0.00
ATOM	3696	CD2	HSD	A	228	25.240	59.260	122.730	0.00	0.00
ATOM	3697	HD2	HSD	A	228	25.210	60.330	122.590	0.00	0.00
ATOM	3698	C	HSD	A	228	29.870	59.180	124.340	0.00	0.00
ATOM	3699	O	HSD	A	228	30.110	58.930	125.520	0.00	0.00
ATOM	3700	N	SER	A	229	30.490	60.170	123.680	0.00	0.00
ATOM	3701	HN	SER	A	229	30.080	60.480	122.830	0.00	0.00
ATOM	3702	CA	SER	A	229	31.500	61.040	124.310	0.00	0.00
ATOM	3703	HA	SER	A	229	32.250	60.320	124.570	0.00	0.00
ATOM	3704	CB	SER	A	229	32.120	62.100	123.250	0.00	0.00
ATOM	3705	HB1	SER	A	229	31.340	62.800	122.890	0.00	0.00
ATOM	3706	HB2	SER	A	229	32.440	61.460	122.400	0.00	0.00
ATOM	3707	OG	SER	A	229	33.100	62.870	123.890	0.00	0.00
ATOM	3708	HG1	SER	A	229	32.650	63.690	124.120	0.00	0.00
ATOM	3709	C	SER	A	229	31.070	61.840	125.590	0.00	0.00
ATOM	3710	O	SER	A	229	30.220	62.720	125.570	0.00	0.00
ATOM	3711	N	LYS	A	230	31.750	61.620	126.710	0.00	0.00
ATOM	3712	HN	LYS	A	230	32.390	60.870	126.760	0.00	0.00
ATOM	3713	CA	LYS	A	230	31.810	62.510	127.890	0.00	0.00
ATOM	3714	HA	LYS	A	230	31.180	63.360	127.670	0.00	0.00
ATOM	3715	CB	LYS	A	230	31.250	61.930	129.220	0.00	0.00
ATOM	3716	HB1	LYS	A	230	31.340	62.710	130.000	0.00	0.00

ATOM 3717 HB2 LYS A 230 31.830 61.010 129.460 0.00 0.00
ATOM 3718 CG LYS A 230 29.750 61.480 129.180 0.00 0.00
ATOM 3719 HG1 LYS A 230 29.360 61.240 130.190 0.00 0.00
ATOM 3720 HG2 LYS A 230 29.610 60.640 128.460 0.00 0.00
ATOM 3721 CD LYS A 230 28.790 62.590 128.580 0.00 0.00
ATOM 3722 HD1 LYS A 230 27.830 62.070 128.360 0.00 0.00
ATOM 3723 HD2 LYS A 230 29.140 62.920 127.580 0.00 0.00
ATOM 3724 CE LYS A 230 28.500 63.900 129.480 0.00 0.00
ATOM 3725 HE1 LYS A 230 27.790 64.590 128.970 0.00 0.00
ATOM 3726 HE2 LYS A 230 29.470 64.410 129.640 0.00 0.00
ATOM 3727 NZ LYS A 230 27.900 63.520 130.780 0.00 0.00
ATOM 3728 HZ1 LYS A 230 27.670 64.310 131.420 0.00 0.00
ATOM 3729 HZ2 LYS A 230 28.500 62.940 131.410 0.00 0.00
ATOM 3730 HZ3 LYS A 230 27.070 62.900 130.660 0.00 0.00
ATOM 3731 C LYS A 230 33.150 63.190 128.180 0.00 0.00
ATOM 3732 O LYS A 230 33.360 63.850 129.190 0.00 0.00
ATOM 3733 N GLY A 231 34.060 63.200 127.130 0.00 0.00
ATOM 3734 HN GLY A 231 33.890 62.590 126.370 0.00 0.00
ATOM 3735 CA GLY A 231 35.380 63.860 127.150 0.00 0.00
ATOM 3736 HA1 GLY A 231 35.220 64.780 127.700 0.00 0.00
ATOM 3737 HA2 GLY A 231 35.700 64.040 126.140 0.00 0.00
ATOM 3738 C GLY A 231 36.430 63.070 127.930 0.00 0.00
ATOM 3739 O GLY A 231 37.120 63.610 128.800 0.00 0.00
ATOM 3740 N HSD A 232 36.430 61.790 127.680 0.00 0.00
ATOM 3741 HN HSD A 232 35.930 61.550 126.860 0.00 0.00
ATOM 3742 CA HSD A 232 37.130 60.780 128.430 0.00 0.00
ATOM 3743 HA HSD A 232 37.950 61.210 128.990 0.00 0.00
ATOM 3744 CB HSD A 232 36.210 60.000 129.400 0.00 0.00
ATOM 3745 HB1 HSD A 232 36.670 59.000 129.550 0.00 0.00
ATOM 3746 HB2 HSD A 232 35.200 59.990 128.940 0.00 0.00
ATOM 3747 ND1 HSD A 232 36.810 60.570 131.790 0.00 0.00
ATOM 3748 HD1 HSD A 232 37.550 59.920 131.980 0.00 0.00
ATOM 3749 CG HSD A 232 36.020 60.710 130.740 0.00 0.00
ATOM 3750 CE1 HSD A 232 36.190 61.160 132.860 0.00 0.00
ATOM 3751 HE1 HSD A 232 36.520 61.190 133.900 0.00 0.00
ATOM 3752 NE2 HSD A 232 34.980 61.600 132.590 0.00 0.00
ATOM 3753 CD2 HSD A 232 34.870 61.310 131.230 0.00 0.00
ATOM 3754 HD2 HSD A 232 33.920 61.560 130.780 0.00 0.00
ATOM 3755 C HSD A 232 37.890 59.770 127.500 0.00 0.00
ATOM 3756 O HSD A 232 37.620 59.480 126.340 0.00 0.00
ATOM 3757 N GLN A 233 39.030 59.210 127.990 0.00 0.00
ATOM 3758 HN GLN A 233 39.130 59.350 128.970 0.00 0.00
ATOM 3759 CA GLN A 233 40.120 58.600 127.260 0.00 0.00
ATOM 3760 HA GLN A 233 40.330 59.330 126.480 0.00 0.00
ATOM 3761 CB GLN A 233 41.410 58.420 128.100 0.00 0.00
ATOM 3762 HB1 GLN A 233 42.130 57.980 127.370 0.00 0.00
ATOM 3763 HB2 GLN A 233 41.300 57.600 128.840 0.00 0.00
ATOM 3764 CG GLN A 233 42.080 59.630 128.690 0.00 0.00
ATOM 3765 HG1 GLN A 233 43.160 59.430 128.870 0.00 0.00
ATOM 3766 HG2 GLN A 233 41.600 59.750 129.680 0.00 0.00
ATOM 3767 CD GLN A 233 42.060 61.000 128.030 0.00 0.00
ATOM 3768 OE1 GLN A 233 41.860 62.030 128.680 0.00 0.00
ATOM 3769 NE2 GLN A 233 42.430 61.010 126.770 0.00 0.00
ATOM 3770 HE21 GLN A 233 42.580 61.920 126.390 0.00 0.00
ATOM 3771 HE22 GLN A 233 42.860 60.220 126.340 0.00 0.00
ATOM 3772 C GLN A 233 39.850 57.300 126.530 0.00 0.00
ATOM 3773 O GLN A 233 40.470 57.010 125.490 0.00 0.00
ATOM 3774 N LYS A 234 39.010 56.490 127.150 0.00 0.00
ATOM 3775 HN LYS A 234 38.490 56.860 127.920 0.00 0.00
ATOM 3776 CA LYS A 234 38.710 55.160 126.660 0.00 0.00
ATOM 3777 HA LYS A 234 39.650 54.640 126.490 0.00 0.00

ATOM	3778	CB	LYS	A	234	37.810	54.350	127.570	0.00	0.00
ATOM	3779	HB1	LYS	A	234	36.760	54.700	127.720	0.00	0.00
ATOM	3780	HB2	LYS	A	234	38.190	54.450	128.610	0.00	0.00
ATOM	3781	CG	LYS	A	234	37.620	52.810	127.250	0.00	0.00
ATOM	3782	HG1	LYS	A	234	38.630	52.340	127.260	0.00	0.00
ATOM	3783	HG2	LYS	A	234	37.260	52.730	126.200	0.00	0.00
ATOM	3784	CD	LYS	A	234	36.740	52.210	128.290	0.00	0.00
ATOM	3785	HD1	LYS	A	234	35.880	52.900	128.420	0.00	0.00
ATOM	3786	HD2	LYS	A	234	37.410	52.270	129.180	0.00	0.00
ATOM	3787	CE	LYS	A	234	36.280	50.840	127.830	0.00	0.00
ATOM	3788	HE1	LYS	A	234	37.130	50.250	127.440	0.00	0.00
ATOM	3789	HE2	LYS	A	234	35.570	50.940	126.970	0.00	0.00
ATOM	3790	NZ	LYS	A	234	35.720	50.100	128.940	0.00	0.00
ATOM	3791	HZ1	LYS	A	234	35.280	50.670	129.690	0.00	0.00
ATOM	3792	HZ2	LYS	A	234	36.430	49.420	129.300	0.00	0.00
ATOM	3793	HZ3	LYS	A	234	34.910	49.560	128.580	0.00	0.00
ATOM	3794	C	LYS	A	234	37.980	55.330	125.300	0.00	0.00
ATOM	3795	O	LYS	A	234	38.270	54.690	124.270	0.00	0.00
ATOM	3796	N	ARG	A	235	36.960	56.280	125.230	0.00	0.00
ATOM	3797	HN	ARG	A	235	36.690	56.710	126.090	0.00	0.00
ATOM	3798	CA	ARG	A	235	36.330	56.710	123.970	0.00	0.00
ATOM	3799	HA	ARG	A	235	35.810	55.900	123.480	0.00	0.00
ATOM	3800	CB	ARG	A	235	35.210	57.660	124.320	0.00	0.00
ATOM	3801	HB1	ARG	A	235	35.620	58.360	125.080	0.00	0.00
ATOM	3802	HB2	ARG	A	235	34.400	57.090	124.830	0.00	0.00
ATOM	3803	CG	ARG	A	235	34.650	58.530	123.160	0.00	0.00
ATOM	3804	HG1	ARG	A	235	35.510	59.090	122.730	0.00	0.00
ATOM	3805	HG2	ARG	A	235	34.050	59.340	123.630	0.00	0.00
ATOM	3806	CD	ARG	A	235	33.700	57.860	122.120	0.00	0.00
ATOM	3807	HD1	ARG	A	235	32.680	57.820	122.550	0.00	0.00
ATOM	3808	HD2	ARG	A	235	34.140	56.880	121.840	0.00	0.00
ATOM	3809	NE	ARG	A	235	33.730	58.720	120.860	0.00	0.00
ATOM	3810	HE	ARG	A	235	34.460	59.400	120.820	0.00	0.00
ATOM	3811	CZ	ARG	A	235	32.680	59.180	120.220	0.00	0.00
ATOM	3812	NH1	ARG	A	235	31.420	58.750	120.290	0.00	0.00
ATOM	3813	HH11	ARG	A	235	31.160	57.880	120.710	0.00	0.00
ATOM	3814	HH12	ARG	A	235	30.650	59.210	119.850	0.00	0.00
ATOM	3815	NH2	ARG	A	235	32.770	60.220	119.440	0.00	0.00
ATOM	3816	HH21	ARG	A	235	33.660	60.660	119.390	0.00	0.00
ATOM	3817	HH22	ARG	A	235	32.080	60.400	118.740	0.00	0.00
ATOM	3818	C	ARG	A	235	37.200	57.340	122.800	0.00	0.00
ATOM	3819	O	ARG	A	235	37.130	56.910	121.660	0.00	0.00
ATOM	3820	N	LYS	A	236	38.120	58.240	123.150	0.00	0.00
ATOM	3821	HN	LYS	A	236	38.200	58.370	124.140	0.00	0.00
ATOM	3822	CA	LYS	A	236	39.170	58.800	122.260	0.00	0.00
ATOM	3823	HA	LYS	A	236	38.640	59.280	121.450	0.00	0.00
ATOM	3824	CB	LYS	A	236	39.880	59.970	123.080	0.00	0.00
ATOM	3825	HB1	LYS	A	236	40.660	60.460	122.460	0.00	0.00
ATOM	3826	HB2	LYS	A	236	40.390	59.570	123.990	0.00	0.00
ATOM	3827	CG	LYS	A	236	38.900	60.990	123.630	0.00	0.00
ATOM	3828	HG1	LYS	A	236	38.060	60.460	124.140	0.00	0.00
ATOM	3829	HG2	LYS	A	236	38.500	61.410	122.680	0.00	0.00
ATOM	3830	CD	LYS	A	236	39.550	62.070	124.510	0.00	0.00
ATOM	3831	HD1	LYS	A	236	40.500	62.460	124.100	0.00	0.00
ATOM	3832	HD2	LYS	A	236	39.810	61.650	125.500	0.00	0.00
ATOM	3833	CE	LYS	A	236	38.670	63.310	124.710	0.00	0.00
ATOM	3834	HE1	LYS	A	236	39.170	64.080	125.340	0.00	0.00
ATOM	3835	HE2	LYS	A	236	37.800	62.940	125.280	0.00	0.00
ATOM	3836	NZ	LYS	A	236	38.300	63.830	123.380	0.00	0.00
ATOM	3837	HZ1	LYS	A	236	37.640	63.270	122.800	0.00	0.00
ATOM	3838	HZ2	LYS	A	236	39.090	64.150	122.780	0.00	0.00

ATOM 3839 HZ3 LYS A 236 37.800 64.710 123.610 0.00 0.00
ATOM 3840 C LYS A 236 40.160 57.760 121.720 0.00 0.00
ATOM 3841 O LYS A 236 40.340 57.680 120.480 0.00 0.00
ATOM 3842 N ALA A 237 40.650 56.850 122.570 0.00 0.00
ATOM 3843 HN ALA A 237 40.420 56.920 123.540 0.00 0.00
ATOM 3844 CA ALA A 237 41.520 55.790 122.120 0.00 0.00
ATOM 3845 HA ALA A 237 42.440 56.130 121.670 0.00 0.00
ATOM 3846 CB ALA A 237 41.850 54.910 123.430 0.00 0.00
ATOM 3847 HB1 ALA A 237 42.420 54.000 123.180 0.00 0.00
ATOM 3848 HB2 ALA A 237 40.930 54.760 124.030 0.00 0.00
ATOM 3849 HB3 ALA A 237 42.550 55.570 123.990 0.00 0.00
ATOM 3850 C ALA A 237 40.820 54.850 121.110 0.00 0.00
ATOM 3851 O ALA A 237 41.300 54.620 119.970 0.00 0.00
ATOM 3852 N LEU A 238 39.540 54.430 121.370 0.00 0.00
ATOM 3853 HN LEU A 238 39.050 54.620 122.220 0.00 0.00
ATOM 3854 CA LEU A 238 38.810 53.590 120.490 0.00 0.00
ATOM 3855 HA LEU A 238 39.460 52.750 120.260 0.00 0.00
ATOM 3856 CB LEU A 238 37.630 53.030 121.290 0.00 0.00
ATOM 3857 HB1 LEU A 238 36.910 53.850 121.470 0.00 0.00
ATOM 3858 HB2 LEU A 238 38.050 52.740 122.280 0.00 0.00
ATOM 3859 CG LEU A 238 36.780 51.880 120.660 0.00 0.00
ATOM 3860 HG LEU A 238 36.270 52.160 119.720 0.00 0.00
ATOM 3861 CD1 LEU A 238 37.650 50.700 120.420 0.00 0.00
ATOM 3862 HD11 LEU A 238 38.320 50.490 121.280 0.00 0.00
ATOM 3863 HD12 LEU A 238 38.380 50.800 119.580 0.00 0.00
ATOM 3864 HD13 LEU A 238 37.150 49.730 120.220 0.00 0.00
ATOM 3865 CD2 LEU A 238 35.690 51.380 121.710 0.00 0.00
ATOM 3866 HD21 LEU A 238 34.960 50.660 121.310 0.00 0.00
ATOM 3867 HD22 LEU A 238 35.090 52.280 121.980 0.00 0.00
ATOM 3868 HD23 LEU A 238 36.140 50.940 122.630 0.00 0.00
ATOM 3869 C LEU A 238 38.400 54.240 119.160 0.00 0.00
ATOM 3870 O LEU A 238 38.360 53.690 118.050 0.00 0.00
ATOM 3871 N LYS A 239 38.060 55.530 119.240 0.00 0.00
ATOM 3872 HN LYS A 239 37.940 55.900 120.150 0.00 0.00
ATOM 3873 CA LYS A 239 37.750 56.430 118.130 0.00 0.00
ATOM 3874 HA LYS A 239 36.910 56.050 117.560 0.00 0.00
ATOM 3875 CB LYS A 239 37.380 57.810 118.640 0.00 0.00
ATOM 3876 HB1 LYS A 239 38.250 58.230 119.210 0.00 0.00
ATOM 3877 HB2 LYS A 239 36.520 57.810 119.340 0.00 0.00
ATOM 3878 CG LYS A 239 36.940 58.880 117.600 0.00 0.00
ATOM 3879 HG1 LYS A 239 37.710 58.920 116.800 0.00 0.00
ATOM 3880 HG2 LYS A 239 36.890 59.860 118.120 0.00 0.00
ATOM 3881 CD LYS A 239 35.580 58.520 117.000 0.00 0.00
ATOM 3882 HD1 LYS A 239 34.830 58.280 117.780 0.00 0.00
ATOM 3883 HD2 LYS A 239 35.770 57.650 116.330 0.00 0.00
ATOM 3884 CE LYS A 239 35.150 59.690 116.080 0.00 0.00
ATOM 3885 HE1 LYS A 239 35.780 59.890 115.190 0.00 0.00
ATOM 3886 HE2 LYS A 239 35.180 60.630 116.670 0.00 0.00
ATOM 3887 NZ LYS A 239 33.760 59.540 115.610 0.00 0.00
ATOM 3888 HZ1 LYS A 239 33.660 58.620 115.140 0.00 0.00
ATOM 3889 HZ2 LYS A 239 33.550 60.320 114.970 0.00 0.00
ATOM 3890 HZ3 LYS A 239 33.000 59.570 116.320 0.00 0.00
ATOM 3891 C LYS A 239 38.860 56.550 117.000 0.00 0.00
ATOM 3892 O LYS A 239 38.620 56.710 115.810 0.00 0.00
ATOM 3893 N THR A 240 40.090 56.580 117.470 0.00 0.00
ATOM 3894 HN THR A 240 40.310 56.490 118.440 0.00 0.00
ATOM 3895 CA THR A 240 41.200 56.560 116.620 0.00 0.00
ATOM 3896 HA THR A 240 41.220 57.350 115.890 0.00 0.00
ATOM 3897 CB THR A 240 42.530 56.630 117.370 0.00 0.00
ATOM 3898 HB THR A 240 42.530 55.780 118.080 0.00 0.00
ATOM 3899 OG1 THR A 240 42.700 57.830 118.090 0.00 0.00

ATOM	3900	HG1	THR	A	240	41.930	57.840	118.650	0.00	0.00
ATOM	3901	CG2	THR	A	240	43.710	56.560	116.440	0.00	0.00
ATOM	3902	HG21	THR	A	240	43.680	57.420	115.750	0.00	0.00
ATOM	3903	HG22	THR	A	240	43.680	55.600	115.890	0.00	0.00
ATOM	3904	HG23	THR	A	240	44.710	56.680	116.910	0.00	0.00
ATOM	3905	C	THR	A	240	41.170	55.300	115.770	0.00	0.00
ATOM	3906	O	THR	A	240	41.330	55.400	114.550	0.00	0.00
ATOM	3907	N	THR	A	241	40.860	54.130	116.370	0.00	0.00
ATOM	3908	HN	THR	A	241	40.480	54.130	117.290	0.00	0.00
ATOM	3909	CA	THR	A	241	40.870	52.760	115.760	0.00	0.00
ATOM	3910	HA	THR	A	241	41.760	52.560	115.180	0.00	0.00
ATOM	3911	CB	THR	A	241	40.720	51.610	116.860	0.00	0.00
ATOM	3912	HB	THR	A	241	39.990	51.900	117.650	0.00	0.00
ATOM	3913	OG1	THR	A	241	41.980	51.280	117.490	0.00	0.00
ATOM	3914	HG1	THR	A	241	41.670	50.820	118.270	0.00	0.00
ATOM	3915	CG2	THR	A	241	40.230	50.290	116.250	0.00	0.00
ATOM	3916	HG21	THR	A	241	40.960	49.850	115.540	0.00	0.00
ATOM	3917	HG22	THR	A	241	39.270	50.450	115.710	0.00	0.00
ATOM	3918	HG23	THR	A	241	40.000	49.530	117.030	0.00	0.00
ATOM	3919	C	THR	A	241	39.800	52.720	114.620	0.00	0.00
ATOM	3920	O	THR	A	241	40.060	52.190	113.530	0.00	0.00
ATOM	3921	N	VAL	A	242	38.570	53.280	114.840	0.00	0.00
ATOM	3922	HN	VAL	A	242	38.340	53.680	115.720	0.00	0.00
ATOM	3923	CA	VAL	A	242	37.540	53.330	113.820	0.00	0.00
ATOM	3924	HA	VAL	A	242	37.530	52.390	113.280	0.00	0.00
ATOM	3925	CB	VAL	A	242	36.110	53.480	114.370	0.00	0.00
ATOM	3926	HB	VAL	A	242	35.380	53.730	113.570	0.00	0.00
ATOM	3927	CG1	VAL	A	242	35.750	52.100	115.010	0.00	0.00
ATOM	3928	HG11	VAL	A	242	35.940	51.370	114.190	0.00	0.00
ATOM	3929	HG12	VAL	A	242	34.680	51.960	115.300	0.00	0.00
ATOM	3930	HG13	VAL	A	242	36.460	51.980	115.850	0.00	0.00
ATOM	3931	CG2	VAL	A	242	35.930	54.500	115.440	0.00	0.00
ATOM	3932	HG21	VAL	A	242	35.990	55.540	115.040	0.00	0.00
ATOM	3933	HG22	VAL	A	242	36.600	54.370	116.310	0.00	0.00
ATOM	3934	HG23	VAL	A	242	34.930	54.270	115.860	0.00	0.00
ATOM	3935	C	VAL	A	242	37.790	54.320	112.760	0.00	0.00
ATOM	3936	O	VAL	A	242	37.410	54.070	111.610	0.00	0.00
ATOM	3937	N	ILE	A	243	38.500	55.430	113.100	0.00	0.00
ATOM	3938	HN	ILE	A	243	38.690	55.700	114.040	0.00	0.00
ATOM	3939	CA	ILE	A	243	38.920	56.450	112.110	0.00	0.00
ATOM	3940	HA	ILE	A	243	38.040	56.700	111.530	0.00	0.00
ATOM	3941	CB	ILE	A	243	39.400	57.750	112.810	0.00	0.00
ATOM	3942	HB	ILE	A	243	40.160	57.510	113.580	0.00	0.00
ATOM	3943	CG2	ILE	A	243	40.050	58.720	111.810	0.00	0.00
ATOM	3944	HG21	ILE	A	243	40.200	59.750	112.190	0.00	0.00
ATOM	3945	HG22	ILE	A	243	39.380	58.740	110.920	0.00	0.00
ATOM	3946	HG23	ILE	A	243	40.950	58.280	111.320	0.00	0.00
ATOM	3947	CG1	ILE	A	243	38.230	58.460	113.530	0.00	0.00
ATOM	3948	HG11	ILE	A	243	38.650	59.110	114.330	0.00	0.00
ATOM	3949	HG12	ILE	A	243	37.600	57.710	114.040	0.00	0.00
ATOM	3950	CD	ILE	A	243	37.280	59.250	112.570	0.00	0.00
ATOM	3951	HD1	ILE	A	243	37.660	60.030	111.890	0.00	0.00
ATOM	3952	HD2	ILE	A	243	36.600	59.790	113.270	0.00	0.00
ATOM	3953	HD3	ILE	A	243	36.560	58.570	112.060	0.00	0.00
ATOM	3954	C	ILE	A	243	39.830	55.940	111.010	0.00	0.00
ATOM	3955	O	ILE	A	243	39.710	56.150	109.820	0.00	0.00
ATOM	3956	N	LEU	A	244	40.810	55.100	111.410	0.00	0.00
ATOM	3957	HN	LEU	A	244	41.000	55.050	112.390	0.00	0.00
ATOM	3958	CA	LEU	A	244	41.670	54.360	110.520	0.00	0.00
ATOM	3959	HA	LEU	A	244	42.160	55.130	109.930	0.00	0.00
ATOM	3960	CB	LEU	A	244	42.750	53.540	111.300	0.00	0.00

```
ATOM   3961  HB1 LEU A 244      43.280  52.820 110.630  0.00  0.00
ATOM   3962  HB2 LEU A 244      42.200  52.930 112.050  0.00  0.00
ATOM   3963  CG  LEU A 244      43.760  54.410 112.090  0.00  0.00
ATOM   3964  HG  LEU A 244      43.200  55.290 112.460  0.00  0.00
ATOM   3965  CD1 LEU A 244      44.290  53.630 113.310  0.00  0.00
ATOM   3966 HD11 LEU A 244      43.630  53.440 114.180  0.00  0.00
ATOM   3967 HD12 LEU A 244      44.870  54.400 113.860  0.00  0.00
ATOM   3968 HD13 LEU A 244      44.910  52.750 113.040  0.00  0.00
ATOM   3969  CD2 LEU A 244      44.880  54.750 111.140  0.00  0.00
ATOM   3970 HD21 LEU A 244      45.450  53.820 110.920  0.00  0.00
ATOM   3971 HD22 LEU A 244      45.540  55.420 111.720  0.00  0.00
ATOM   3972 HD23 LEU A 244      44.500  55.220 110.200  0.00  0.00
ATOM   3973  C   LEU A 244      40.900  53.310 109.570  0.00  0.00
ATOM   3974  O   LEU A 244      41.000  53.280 108.320  0.00  0.00
ATOM   3975  N   ILE A 245      39.920  52.620 110.180  0.00  0.00
ATOM   3976  HN  ILE A 245      39.790  52.470 111.160  0.00  0.00
ATOM   3977  CA  ILE A 245      38.920  51.890 109.400  0.00  0.00
ATOM   3978  HA  ILE A 245      39.460  51.140 108.850  0.00  0.00
ATOM   3979  CB  ILE A 245      37.960  51.080 110.340  0.00  0.00
ATOM   3980  HB  ILE A 245      37.350  51.740 110.980  0.00  0.00
ATOM   3981  CG2 ILE A 245      36.890  50.360 109.460  0.00  0.00
ATOM   3982 HG21 ILE A 245      36.180  50.990 108.900  0.00  0.00
ATOM   3983 HG22 ILE A 245      36.310  49.720 110.160  0.00  0.00
ATOM   3984 HG23 ILE A 245      37.500  49.880 108.660  0.00  0.00
ATOM   3985  CG1 ILE A 245      38.790  50.040 111.140  0.00  0.00
ATOM   3986 HG11 ILE A 245      39.600  50.620 111.630  0.00  0.00
ATOM   3987 HG12 ILE A 245      39.230  49.240 110.520  0.00  0.00
ATOM   3988  CD  ILE A 245      37.900  49.550 112.330  0.00  0.00
ATOM   3989  HD1 ILE A 245      37.200  48.770 111.960  0.00  0.00
ATOM   3990  HD2 ILE A 245      37.280  50.360 112.760  0.00  0.00
ATOM   3991  HD3 ILE A 245      38.470  49.100 113.170  0.00  0.00
ATOM   3992  C   ILE A 245      38.000  52.740 108.480  0.00  0.00
ATOM   3993  O   ILE A 245      37.800  52.460 107.300  0.00  0.00
ATOM   3994  N   LEU A 246      37.440  53.900 108.920  0.00  0.00
ATOM   3995  HN  LEU A 246      37.180  53.940 109.880  0.00  0.00
ATOM   3996  CA  LEU A 246      36.760  54.840 107.990  0.00  0.00
ATOM   3997  HA  LEU A 246      35.960  54.300 107.510  0.00  0.00
ATOM   3998  CB  LEU A 246      36.260  56.080 108.700  0.00  0.00
ATOM   3999  HB1 LEU A 246      37.170  56.610 109.050  0.00  0.00
ATOM   4000  HB2 LEU A 246      35.780  55.780 109.660  0.00  0.00
ATOM   4001  CG  LEU A 246      35.320  57.050 107.950  0.00  0.00
ATOM   4002  HG  LEU A 246      35.930  57.430 107.110  0.00  0.00
ATOM   4003  CD1 LEU A 246      34.110  56.220 107.450  0.00  0.00
ATOM   4004 HD11 LEU A 246      33.180  56.820 107.450  0.00  0.00
ATOM   4005 HD12 LEU A 246      33.870  55.350 108.100  0.00  0.00
ATOM   4006 HD13 LEU A 246      34.200  55.760 106.440  0.00  0.00
ATOM   4007  CD2 LEU A 246      34.930  58.250 108.830  0.00  0.00
ATOM   4008 HD21 LEU A 246      35.810  58.700 109.320  0.00  0.00
ATOM   4009 HD22 LEU A 246      34.210  57.910 109.610  0.00  0.00
ATOM   4010 HD23 LEU A 246      34.430  59.040 108.220  0.00  0.00
ATOM   4011  C   LEU A 246      37.630  55.280 106.800  0.00  0.00
ATOM   4012  O   LEU A 246      37.120  55.330 105.680  0.00  0.00
ATOM   4013  N   ALA A 247      38.920  55.550 107.100  0.00  0.00
ATOM   4014  HN  ALA A 247      39.310  55.450 108.010  0.00  0.00
ATOM   4015  CA  ALA A 247      39.950  55.890 106.160  0.00  0.00
ATOM   4016  HA  ALA A 247      39.540  56.720 105.620  0.00  0.00
ATOM   4017  CB  ALA A 247      41.220  56.380 106.980  0.00  0.00
ATOM   4018  HB1 ALA A 247      42.130  56.340 106.350  0.00  0.00
ATOM   4019  HB2 ALA A 247      41.310  55.780 107.910  0.00  0.00
ATOM   4020  HB3 ALA A 247      41.120  57.400 107.410  0.00  0.00
ATOM   4021  C   ALA A 247      40.150  54.800 105.100  0.00  0.00
```

ATOM	4022	O	ALA	A	247	40.070	55.020	103.860	0.00	0.00
ATOM	4023	N	PHE	A	248	40.200	53.530	105.560	0.00	0.00
ATOM	4024	HN	PHE	A	248	40.170	53.320	106.540	0.00	0.00
ATOM	4025	CA	PHE	A	248	40.190	52.370	104.580	0.00	0.00
ATOM	4026	HA	PHE	A	248	41.090	52.460	103.990	0.00	0.00
ATOM	4027	CB	PHE	A	248	40.320	51.030	105.350	0.00	0.00
ATOM	4028	HB1	PHE	A	248	39.530	50.980	106.130	0.00	0.00
ATOM	4029	HB2	PHE	A	248	41.320	50.920	105.820	0.00	0.00
ATOM	4030	CG	PHE	A	248	40.120	49.760	104.430	0.00	0.00
ATOM	4031	CD1	PHE	A	248	39.010	48.890	104.630	0.00	0.00
ATOM	4032	HD1	PHE	A	248	38.260	49.180	105.350	0.00	0.00
ATOM	4033	CE1	PHE	A	248	38.970	47.670	103.900	0.00	0.00
ATOM	4034	HE1	PHE	A	248	38.250	46.860	103.920	0.00	0.00
ATOM	4035	CZ	PHE	A	248	39.900	47.290	102.990	0.00	0.00
ATOM	4036	HZ	PHE	A	248	39.830	46.350	102.460	0.00	0.00
ATOM	4037	CD2	PHE	A	248	41.140	49.310	103.570	0.00	0.00
ATOM	4038	HD2	PHE	A	248	42.100	49.800	103.560	0.00	0.00
ATOM	4039	CE2	PHE	A	248	41.010	48.120	102.830	0.00	0.00
ATOM	4040	HE2	PHE	A	248	41.790	47.820	102.150	0.00	0.00
ATOM	4041	C	PHE	A	248	39.050	52.390	103.520	0.00	0.00
ATOM	4042	O	PHE	A	248	39.250	52.270	102.280	0.00	0.00
ATOM	4043	N	PHE	A	249	37.800	52.610	104.010	0.00	0.00
[truncated: 544,063 more chars]
