# Supplementary material for: Computational modeling and experimental validation of the EPI-X4/CXCR4 complex allows rational design of small peptide antagonists
Source: Commun Biol. 2021 Sep 22;4:1113. doi: 10.1038/s42003-021-02638-5 (PMC8458281; doi:10.1038/s42003-021-02638-5)
Supplement: Supplementary file 6 — Reporting Summary [file 42003_2021_2638_MOESM6_ESM.pdf]

nature research

Corresponding author(s): Jan Münch
Elsa Sanchez-Garcia

Last updated by author(s): Aug 4, 2021

Reporting Summary

Nature Research wishes to improve the reproducibility of the work that we publish. This form provides structure for consistency and transparency in reporting. For further information on Nature Research policies, see our Editorial Policies and the Editorial Policy Checklist.

Statistics

For all statistical analyses, confirm that the following items are present in the figure legend, table legend, main text, or Methods section.

n/a	Confirmed	
☐	☒	The exact sample size (n) for each experimental group/condition, given as a discrete number and unit of measurement
☐	☒	A statement on whether measurements were taken from distinct samples or whether the same sample was measured repeatedly
☐	☒	The statistical test(s) used AND whether they are one- or two-sided<br>*Only common tests should be described solely by name; describe more complex techniques in the Methods section.*
☐	☐	A description of all covariates tested
☐	☒	A description of any assumptions or corrections, such as tests of normality and adjustment for multiple comparisons
☐	☒	A full description of the statistical parameters including central tendency (e.g. means) or other basic estimates (e.g. regression coefficient) AND variation (e.g. standard deviation) or associated estimates of uncertainty (e.g. confidence intervals)
☐	☐	For null hypothesis testing, the test statistic (e.g. F, t, r) with confidence intervals, effect sizes, degrees of freedom and P value noted<br>*Give P values as exact values whenever suitable.*
☐	☐	For Bayesian analysis, information on the choice of priors and Markov chain Monte Carlo settings
☐	☐	For hierarchical and complex designs, identification of the appropriate level for tests and full reporting of outcomes
☐	☐	Estimates of effect sizes (e.g. Cohen's d, Pearson's r), indicating how they were calculated

Our web collection on statistics for biologists contains articles on many of the points above.

Software and code

Policy information about availability of computer code

Data collection	Molecular Dynamics simulation trajectories were obtained by using a standard version of publicly available software, NAMD version 2.11 and GROMACS version 2016.3. Custom modifications were not employed. Homology modeling was carried out using the standard Modeller program version 9.18.
Data analysis	The molecular dynamics simulation trajectories were analysed using the tools bundled with VMD program version 1.9.2. GROMACS/MMPBSA Tool was used to calculate the interaction energy between CXCR4 and antagonist peptides reported in this study.

For manuscripts utilizing custom algorithms or software that are central to the research but not yet described in published literature, software must be made available to editors and reviewers. We strongly encourage code deposition in a community repository (e.g. GitHub). See the Nature Research guidelines for submitting code & software for further information.

Data

Policy information about availability of data

All manuscripts must include a data availability statement. This statement should provide the following information, where applicable:

- Accession codes, unique identifiers, or web links for publicly available datasets
- A list of figures that have associated raw data
- A description of any restrictions on data availability

Done

Field-specific reporting

Please select the one below that is the best fit for your research. If you are not sure, read the appropriate sections before making your selection.

☒ Life sciences ☐ Behavioural & social sciences ☐ Ecological, evolutionary & environmental sciences

For a reference copy of the document with all sections, see nature.com/documents/nr-reporting-summary-flat.pdf

Life sciences study design

All studies must disclose on these points even when the disclosure is negative.

Sample size	All biological assays were performed at least in three individual biological replicates. Where cell signaling or migration was involved, each replicates were additionally performed in triplicates. For zebrafish experiments each peptide concentration was tested in two independent assays, each of which was performed on 10 × 3 embryos, to allow meaningful statistical analysis.
Data exclusions	No data were excluded
Replication	All data were reproducible and all experiments and data points shown
Randomization	Randomization was not necessary, since the focus is on molecular modeling and designed optimized derivatives were tested in cell lines only
Blinding	Blinding was not relevant, since all data were obtained in cell lines using multichannel pipettes.

Reporting for specific materials, systems and methods

We require information from authors about some types of materials, experimental systems and methods used in many studies. Here, indicate whether each material, system or method listed is relevant to your study. If you are not sure if a list item applies to your research, read the appropriate section before selecting a response.

Materials & experimental systems

n/a	Involved in the study	
☐	☒	Antibodies
☐	☒	Eukaryotic cell lines
☒	☐	Palaeontology and archaeology
☐	☒	Animals and other organisms
☒	☐	Human research participants
☒	☐	Clinical data
☒	☐	Dual use research of concern

Methods

n/a	Involved in the study	
☒	☐	ChIP-seq
☐	☒	Flow cytometry
☒	☐	MRI-based neuroimaging

Antibodies

Antibodies used	phospho-p44/42 MAPK (Erk1) (Tyr204)/ (Erk2) (Tyr187) (D1H6G) mouse mAb #5726 cell signaling; phospho-Akt (Ser473) (193H12) rabbit mAb #4058 Cell Signaling; conjugated CXCR4 antibody clone 12G5; #555976, PD PharmingenTM; conjugated CXCR4 antibody clone 1D9 #551510, BD PharmingenTM<br><br>Secondary antibodies: Goat anti-Mouse IgG (H+L) Cross-Adsorbed Secondary Antibody, Alexa Fluor 488, Invitrogen; Anti-rabbit IgG (H +L), F(ab')2 fragment (Alexa Fluor 647 Conjugate), Cell Signaling<br><br>Isotype controls: Mouse mAb (E5Y6Q) IgG2a Isotype control, Cell Signaling; Normal Rabbit IgG, Cell Signaling; PE Rat IgG2a, κ, BD Pharmingen; APC Mouse IgG2a, κ, BD Pharmingen
Validation	Phospho-p44/42 MAPK (Erk1) (Tyr204)/(Erk2) (Tyr187) (D1H6G) Mouse mAb recognizes endogenous levels of p44/42 MAPK/Erk protein when phosphorylated at Tyr204 of p44 MAPK/Erk1 (Tyr187 of p42 MAPK/Erk2). This antibody detects dual-phosphorylated p44 MAPK/Erk1 (Thr202/Tyr204)/p42 MAPK/Erk2 (Thr185/Tyr187), but does not detect threonine mono-phosphorylated p44/42 MAPK/Erk. This antibody does not cross-react with any other MAP kinases. Species Reactivity:Human, Mouse, Rat, Monkey. Species predicted to react based on 100% sequence homology: Chicken, D. melanogaster, Xenopus, Zebrafish, Bovine, C. elegans. Source / Purification: Monoclonal antibody is produced by immunizing animals with a synthetic phosphopeptide corresponding to residues surrounding Tyr187 of human Erk2 protein.<br><br>Phospho-Akt (Ser473) (193H12) Rabbit mAb detects endogenous levels of Akt only when phosphorylated at Ser473. Species Reactivity: Human, Mouse, Rat. Source / Purification. Monoclonal antibody is produced by immunizing animals with a synthetic phosphopeptide corresponding to residues around Ser473 of mouse Akt.

CXCR4 12G5 antibody, BD pharmingenTM; IIsotype: Mouse BALB/c IgG2a, κ. Reactivity: Human (QC Testing); Application: Flow cytometry (Routinely Tested), Immunogen: SIVmac variant CP-MAC-infected Sup-T1 cells; Workshop No. VII 70204, 70305

CXCR4 1D9 antibody, BD pharmingenTM; Isotype: Rat IgG2a, κ; Reactivity: Human (QC Testing); Application: Flow cytometry (Routinely Tested); Immunogen: Human CXCR4 fusion protein

Eukaryotic cell lines

Policy information about cell lines

Cell line source(s)	HEK293T: ATCC® CRL-3216™<br>SUP-T1 [VB] (ATCC® CRL-1942™)
Authentication	ATCC uses morphology, karyotyping, and PCR based approaches to confirm the identity of human cell lines and to rule out both intra- and interspecies contamination. These include an assay to detect species specific variants of the cytochrome C oxidase I gene (COI analysis) to rule out inter-species contamination and short tandem repeat (STR) profiling to distinguish between individual human cell lines and rule out intra-species contamination.
Mycoplasma contamination	All cell lines were tested negative for mycoplasma contamination
Commonly misidentified lines<br>(See ICLAC register)	*Name any commonly misidentified cell lines used in the study and provide a rationale for their use.*

Animals and other organisms

Policy information about studies involving animals; ARRIVE guidelines recommended for reporting animal research

Laboratory animals	wild type zebrafish embryos
Wild animals	the study does not involve wild animals
Field-collected samples	the study does not involve field collected samples
Ethics oversight	Ethical approval is not required for zebrafish

Note that full information on the approval of the study protocol must also be provided in the manuscript.

Flow Cytometry

Plots

Confirm that:

☒ The axis labels state the marker and fluorochrome used (e.g. CD4-FITC).

☒ The axis scales are clearly visible. Include numbers along axes only for bottom left plot of group (a 'group' is an analysis of identical markers).

☒ All plots are contour plots with outliers or pseudocolor plots.

☒ A numerical value for number of cells or percentage (with statistics) is provided.

Methodology

Sample preparation	Antibody competition: the antibody was applied at low concentration in the presence of inhibitors to SupT1 cells for 2 hours. Unbound antibody was removed and cells fixed using 2% PFA before 5,000 cells were used for quantification (cell number has been confirmed in the publication where the assay was established, Harms et al. 2020, Sci Rep)<br><br>For signaling assays, SupT1 cells were first fixed using 2% PFA (to stop the signaling reaction) and then permeabilized using methanol. Cells were then stained with the respective antibodies according to the manufacturers instructions.
Instrument	Cytoflex flow cytometer, Beckman coulter
Software	CytExpert, Beckman Coulter, version 2.3.0.84
Cell population abundance	A cell line (SupT1) was used with no impurities
Gating strategy	FSC/SSC gates were set to identify all cells. For competition experiments MFI of the single stains were then directly determined for this population. Data were normalized to isotype controls. For Erk/Akt signaling experiments this cell population was also chosen, MFI determined and signal normalized to respective isotype controls, unstimulated controls and controls without inhibitor. Not further gating strategies were applied.

☒ Tick this box to confirm that a figure exemplifying the gating strategy is provided in the Supplementary Information.
